# Supplementary material for: Instability of estimation results based on caliper matching with propensity scores
Source: PLoS One. 2025 Jun 6;20(6):e0325317. doi: 10.1371/journal.pone.0325317 (PMC12143538; doi:10.1371/journal.pone.0325317)
Supplement: S1 File — (PDF) [file pone.0325317.s001.pdf]

Supplementary Material for "Instability of Estimation Results Based on Caliper Matching with Propensity Scores"

All simulation results (caliper: 25%)

Kazushi Maruo, Yusuke Yamaguchi, Ryota Ishii, Masahiko Gosho

Contents

S1. Median width of OR for random order matching (caliper: 25%) 1
S2. Median bias for OR (caliper: 25%) 8
S3. IQR for OR (precision, caliper: 25%) 44
S4. Median absolute difference of OR for 10% data addition (caliper: 25%) 80
S5. Coverage probability of confidence interval for OR (caliper: 25%) 116
S6. Mean percentage bias of standard error for log odds ratio (caliper: 25%) 188

S1. Median width of OR for random order matching (caliper: 25%)

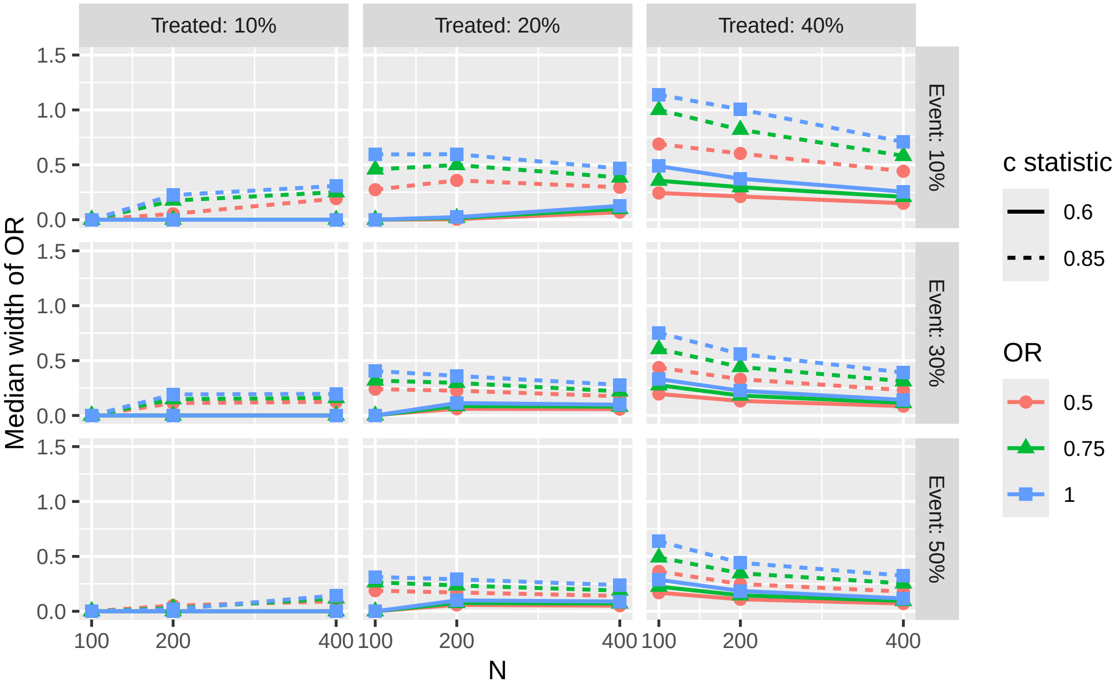

Figure S1. Median width of OR for random order matching (unimodal continuous covariate, with caliper, matching ratio 1:1)

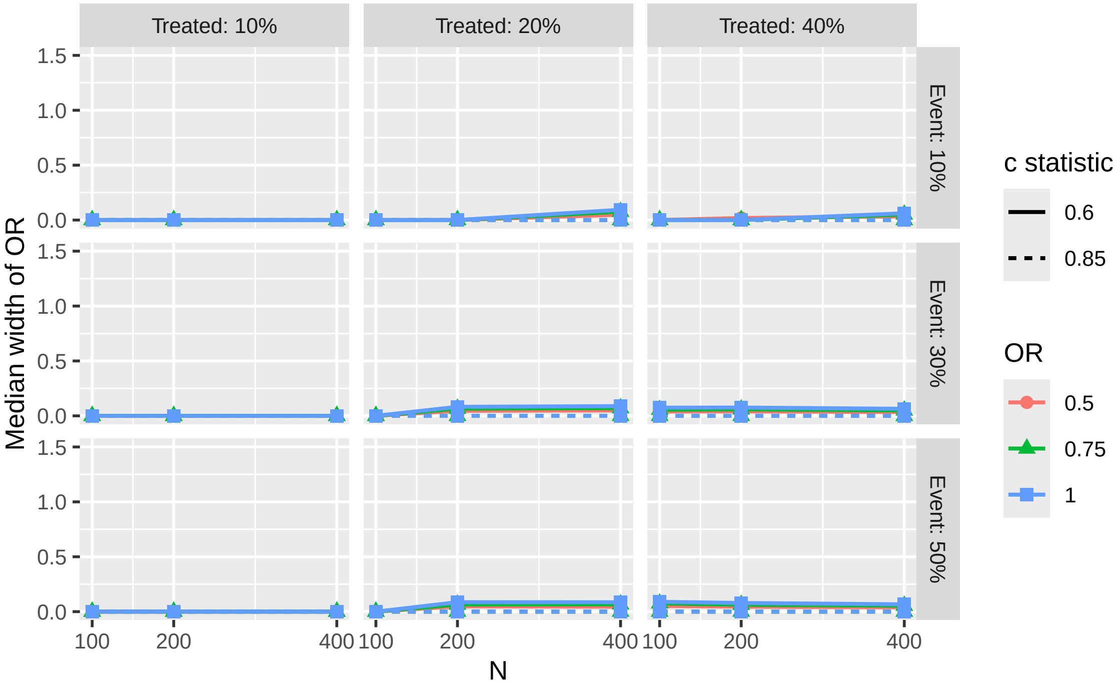

Figure S2. Median width of OR for random order matching (unimodal continuous covariate, without caliper, matching ratio 1:1)

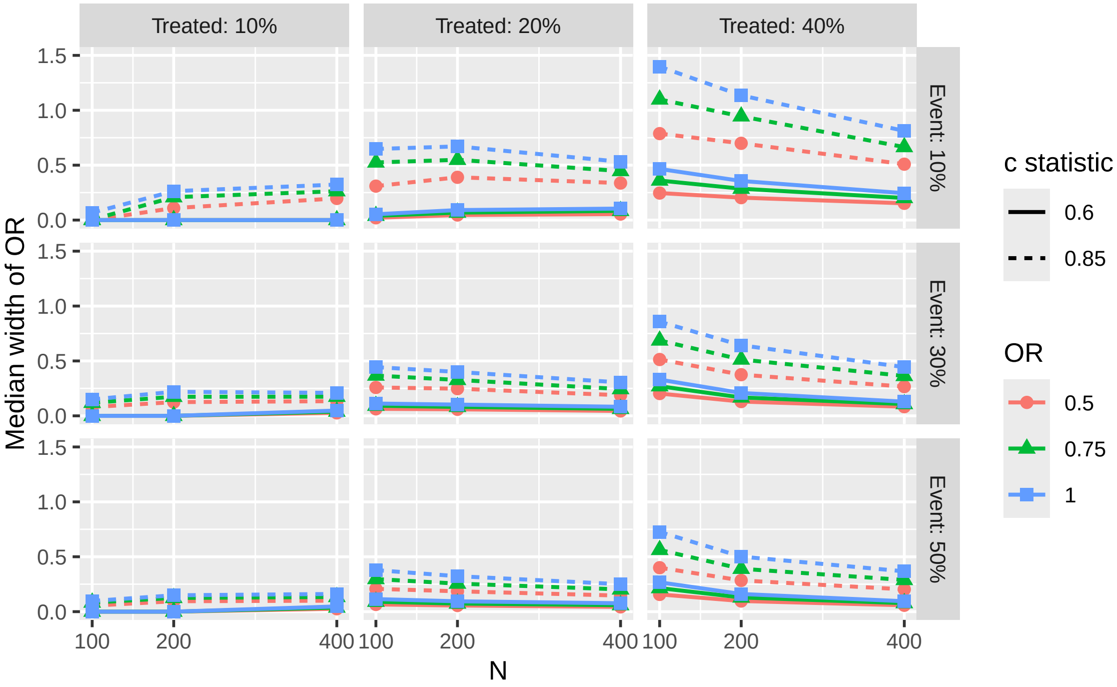

Figure S3. Median width of OR for random order matching (unimodal continuous covariate, with caliper, matching ratio 1:2)

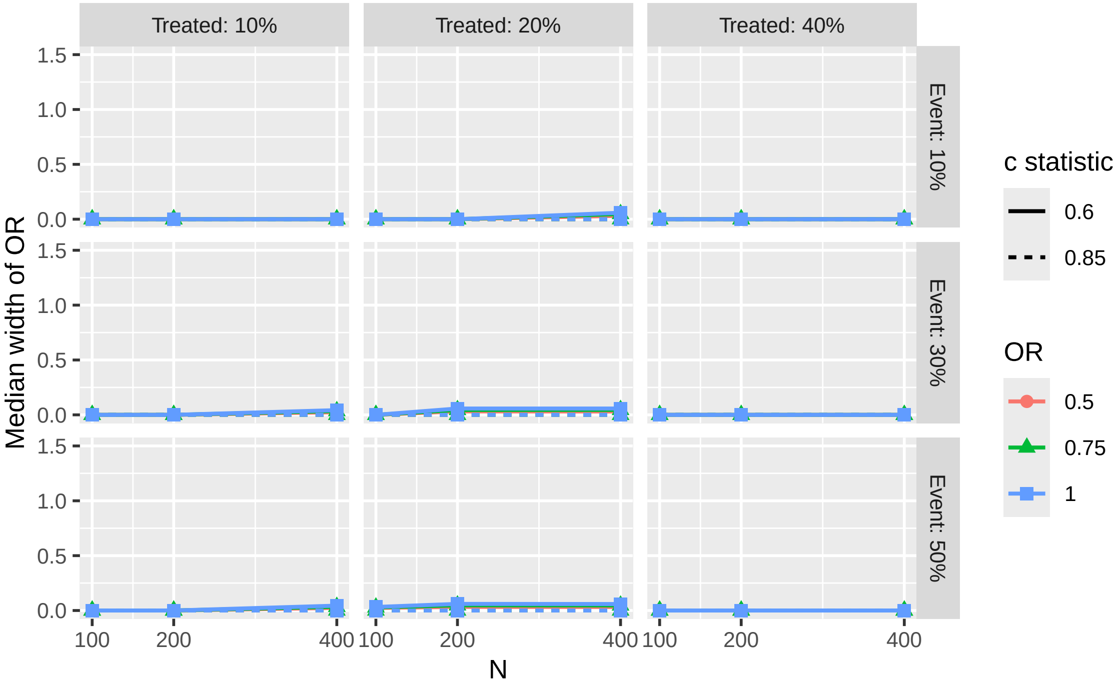

Figure S4. Median width of OR for random order matching (unimodal continuous covariate, without caliper, matching ratio 1:2)

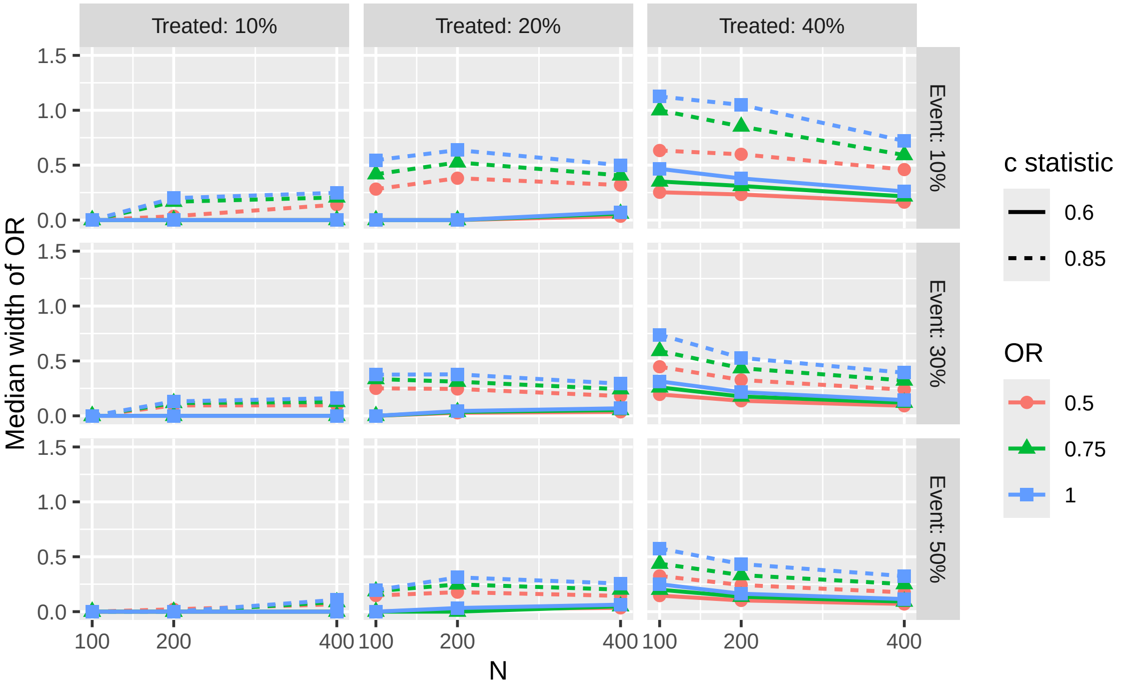

Figure S5. Median width of OR for random order matching (categorical covariate, with caliper, matching ratio 1:1)

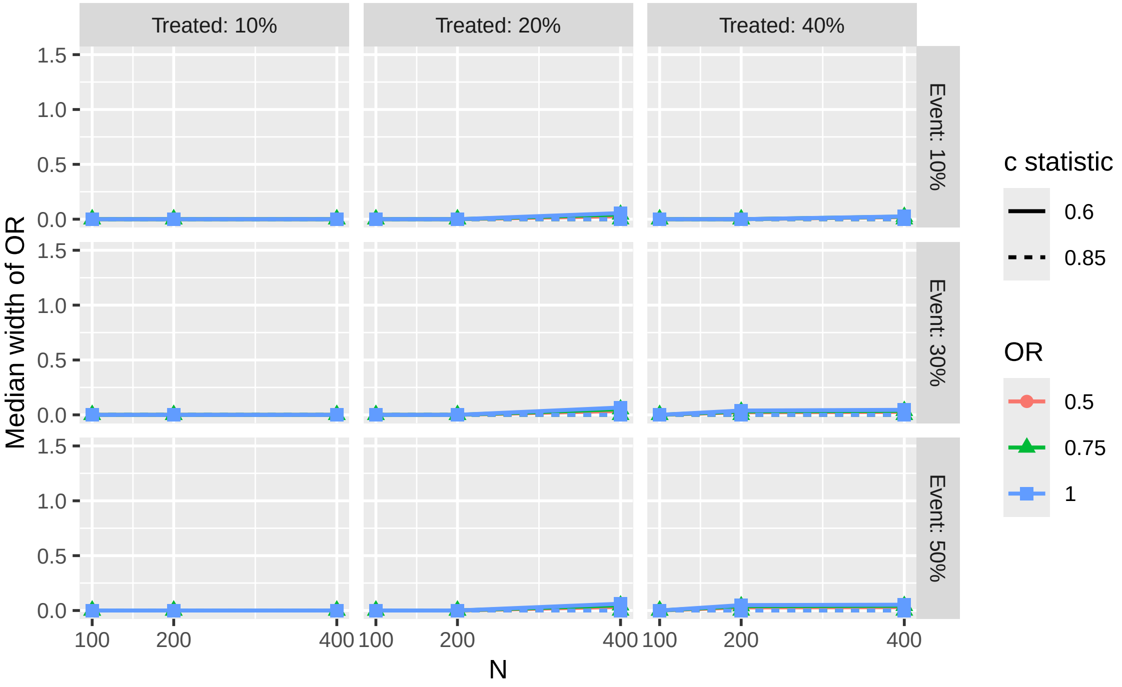

Figure S6. Median width of OR for random order matching (categorical covariate, without caliper, matching ratio 1:1)

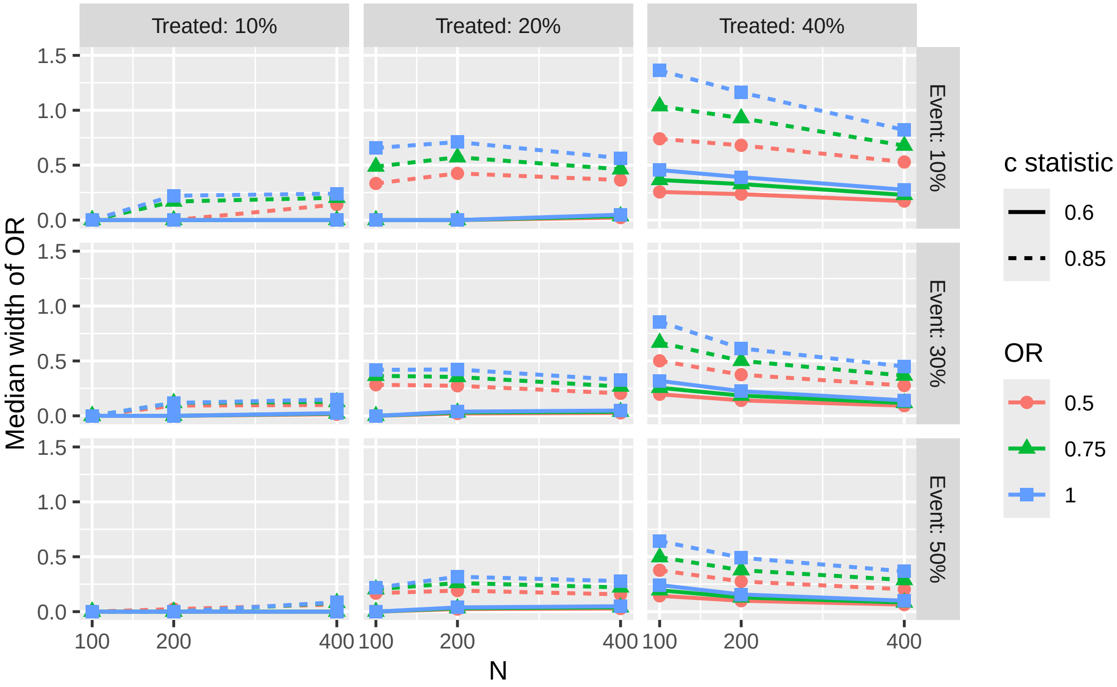

Figure S7. Median width of OR for random order matching (categorical covariate, with caliper, matching ratio 1:2)

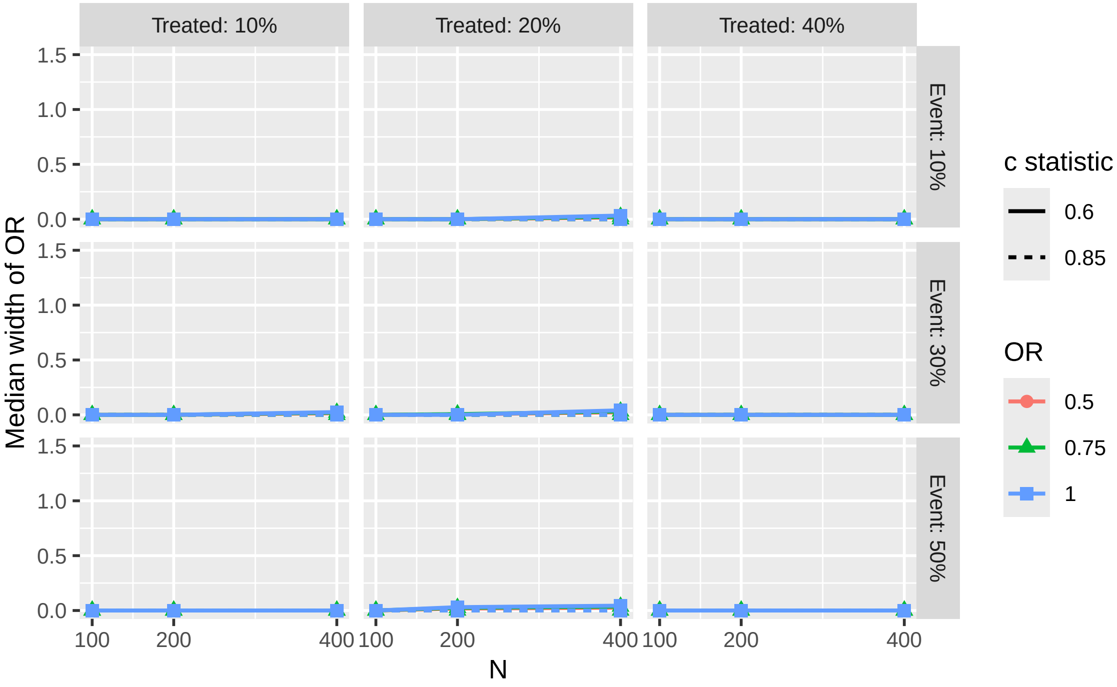

Figure S8. Median width of OR for random order matching (categorical covariate, without caliper, matching ratio 1:2)

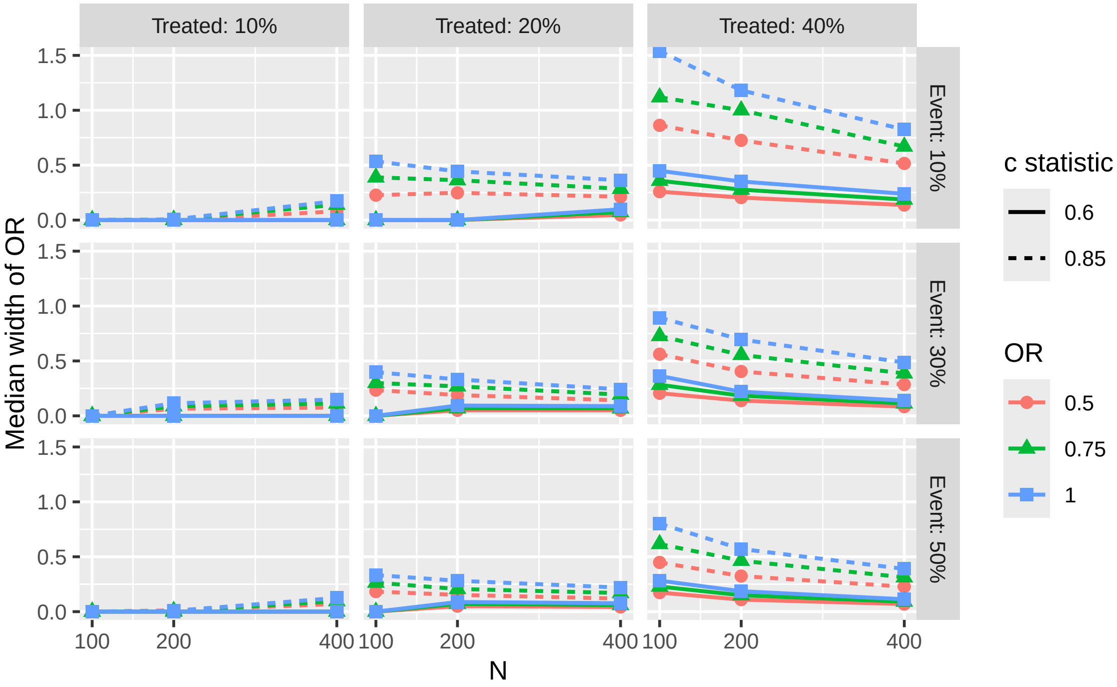

Figure S9. Median width of OR for random order matching (multimodal continuous covariate, with caliper, matching ratio 1:1)

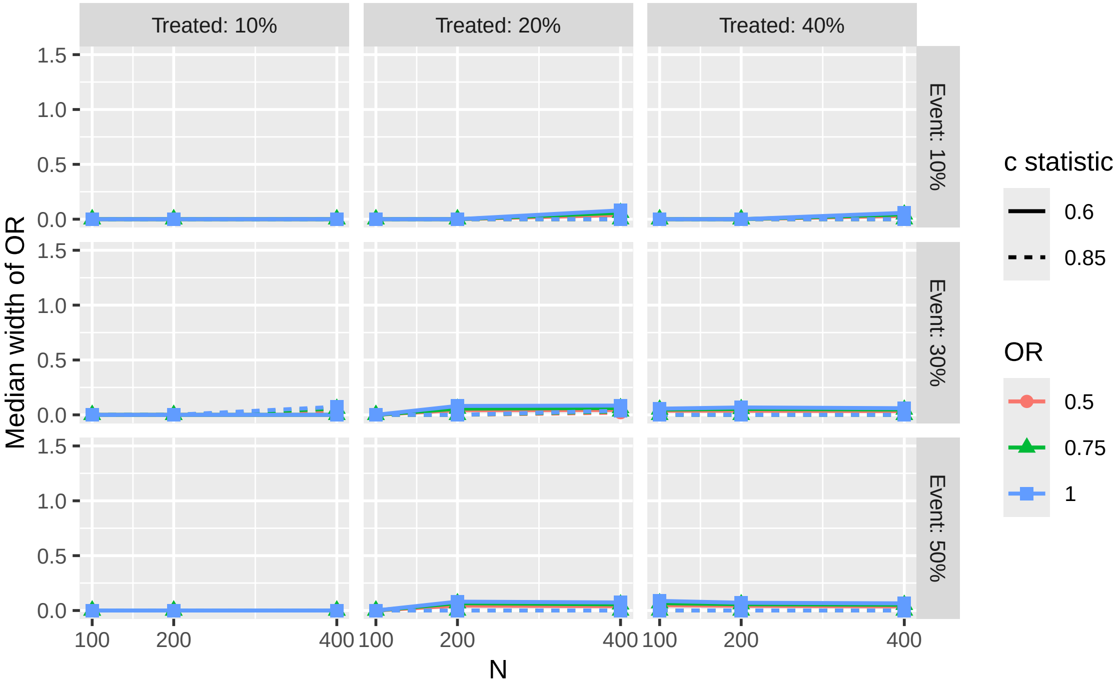

Figure S10. Median width of OR for random order matching (multimodal continuous covariate, without caliper, matching ratio 1:1)

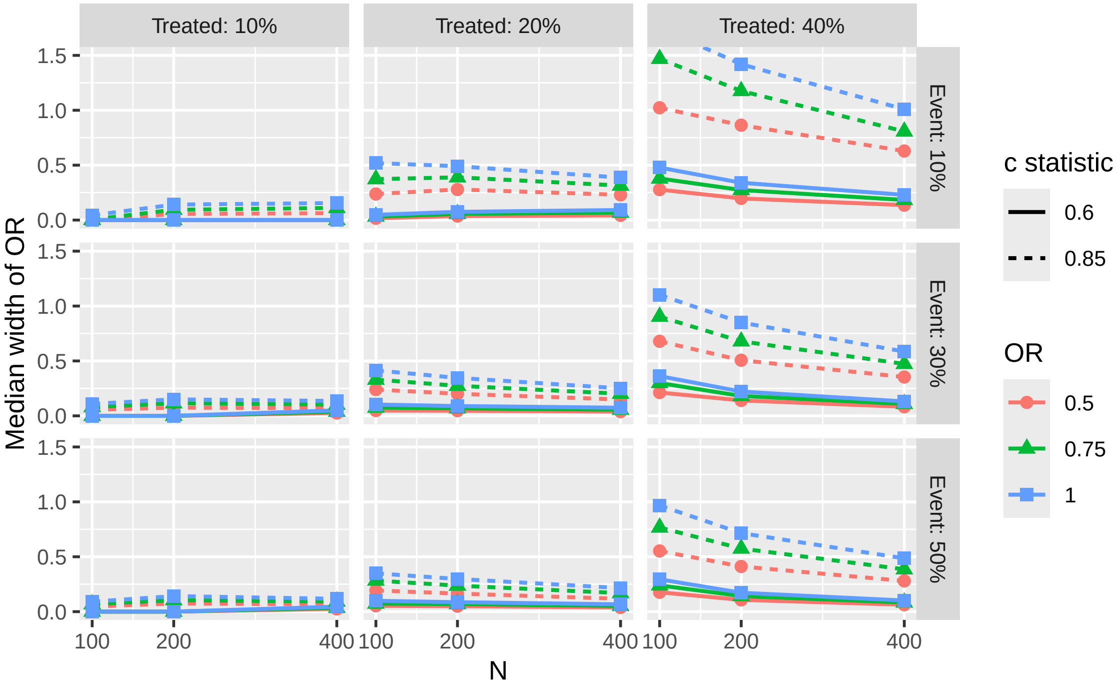

Figure S11. Median width of OR for random order matching (multimodal continuous covariate, with caliper, matching ratio 1:2)

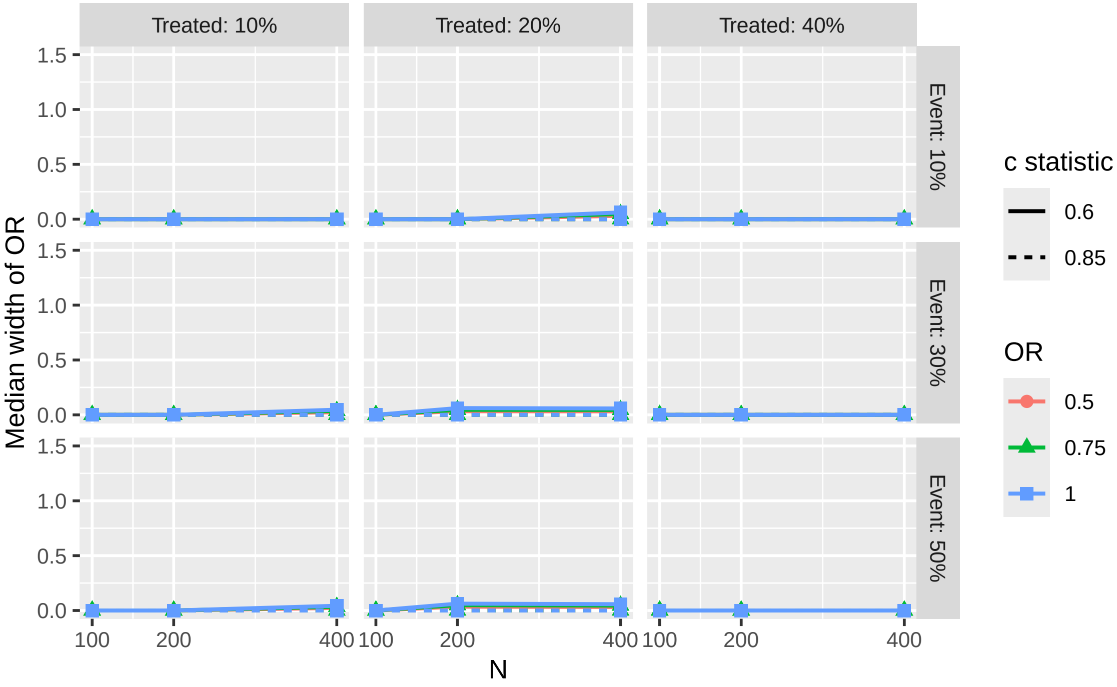

Figure S12. Median width of OR for random order matching (multimodal continuous covariate, without caliper, matching ratio 1:2)

S2. Median bias for OR (caliper: 25%)

Weighting methods is not related to the matching ratio, but shown for reference. For M_NoCal method, bias may be too large to be within the range.

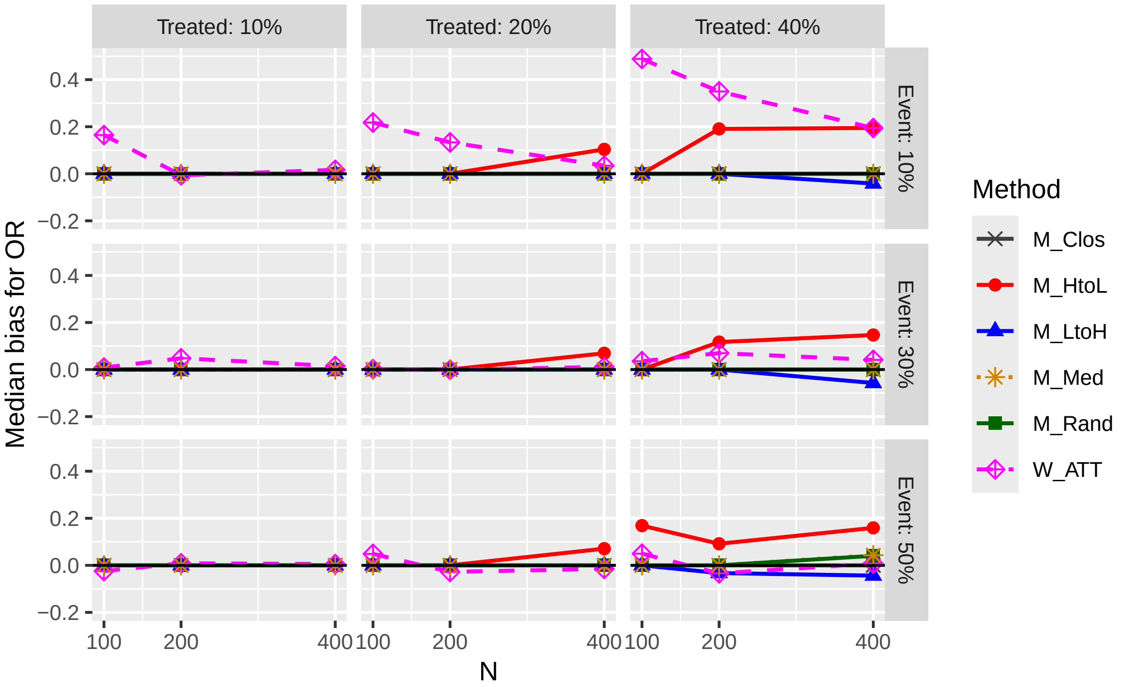

Figure S13. Median bias for OR (unimodal continuous covariate, matching ratio 1:1, true OR: 1, c statistic: 0.85).

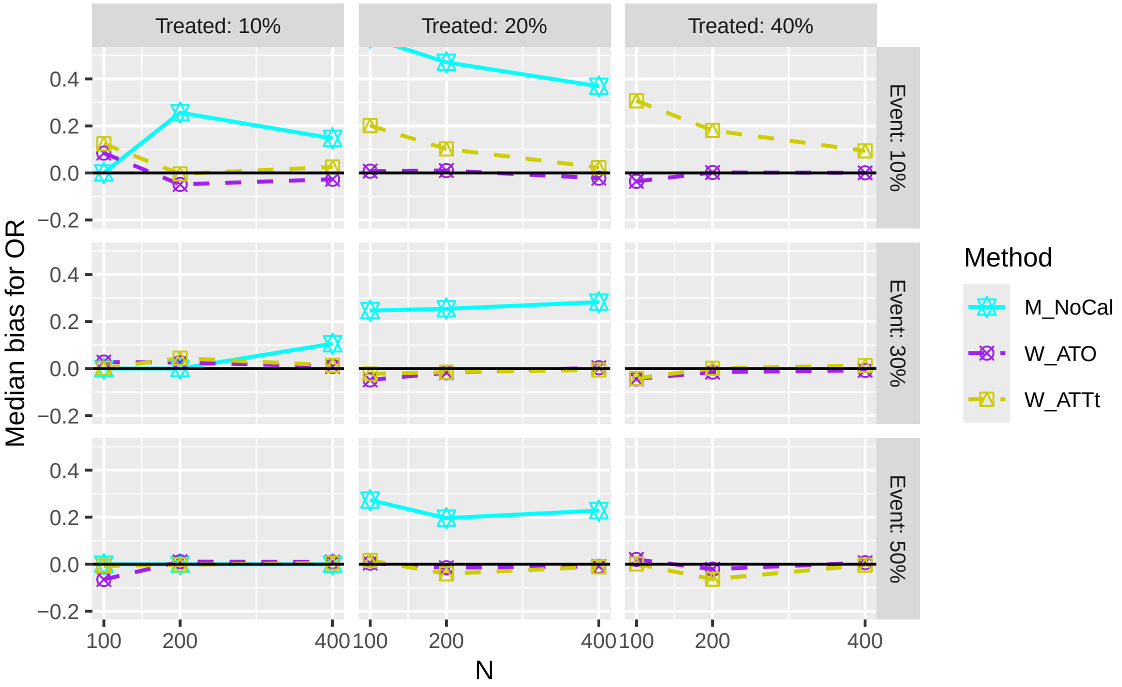

Figure S14. Median bias for OR (unimodal continuous covariate, matching ratio 1:1, true OR: 1, c statistic: 0.85); other methods.

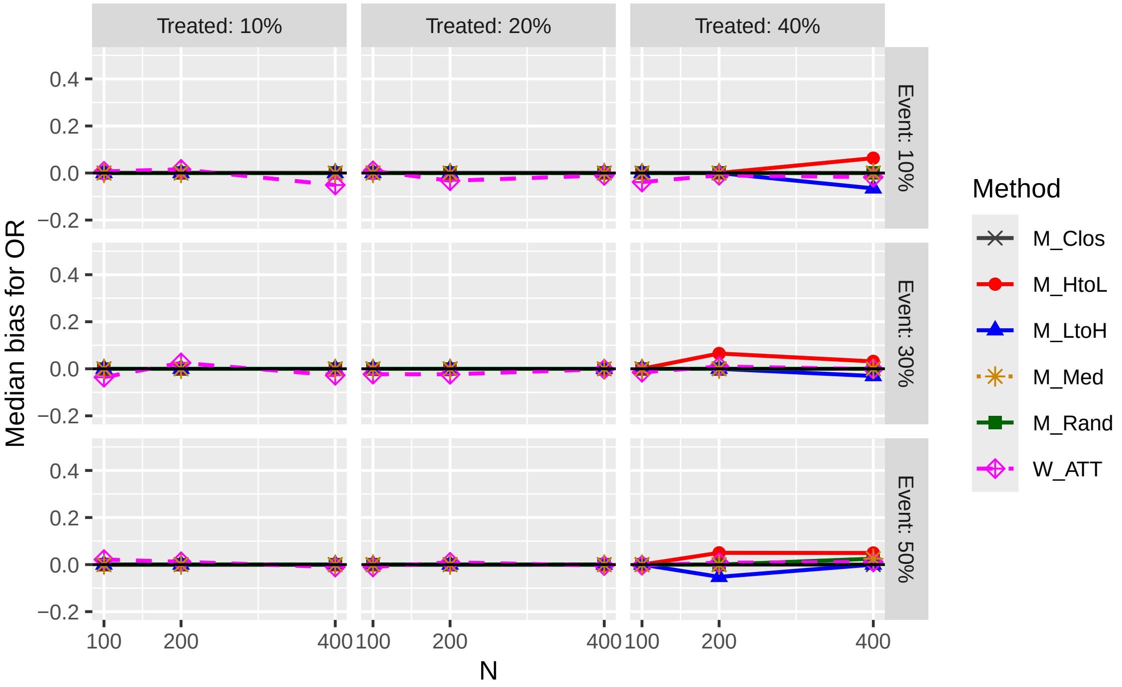

Figure S15. Median bias for OR (unimodal continuous covariate, matching ratio 1:1, true OR: 1, c statistic: 0.6).

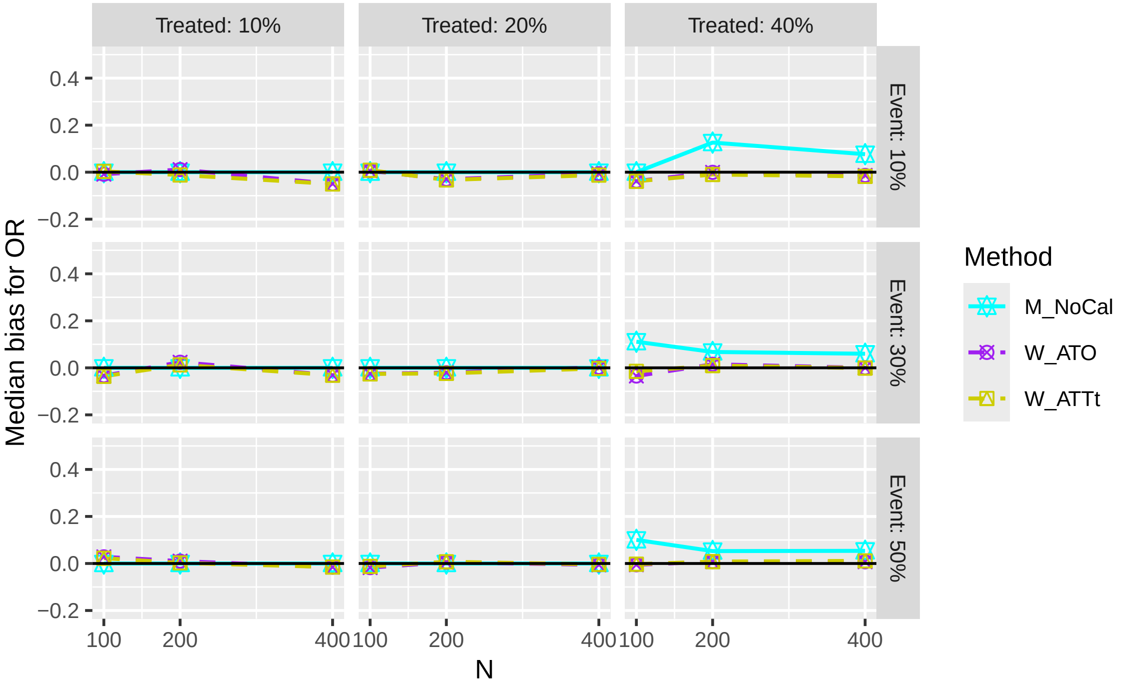

Figure S16. Median bias for OR (unimodal continuous covariate, matching ratio 1:1, true OR: 1, c statistic: 0.6); other methods.

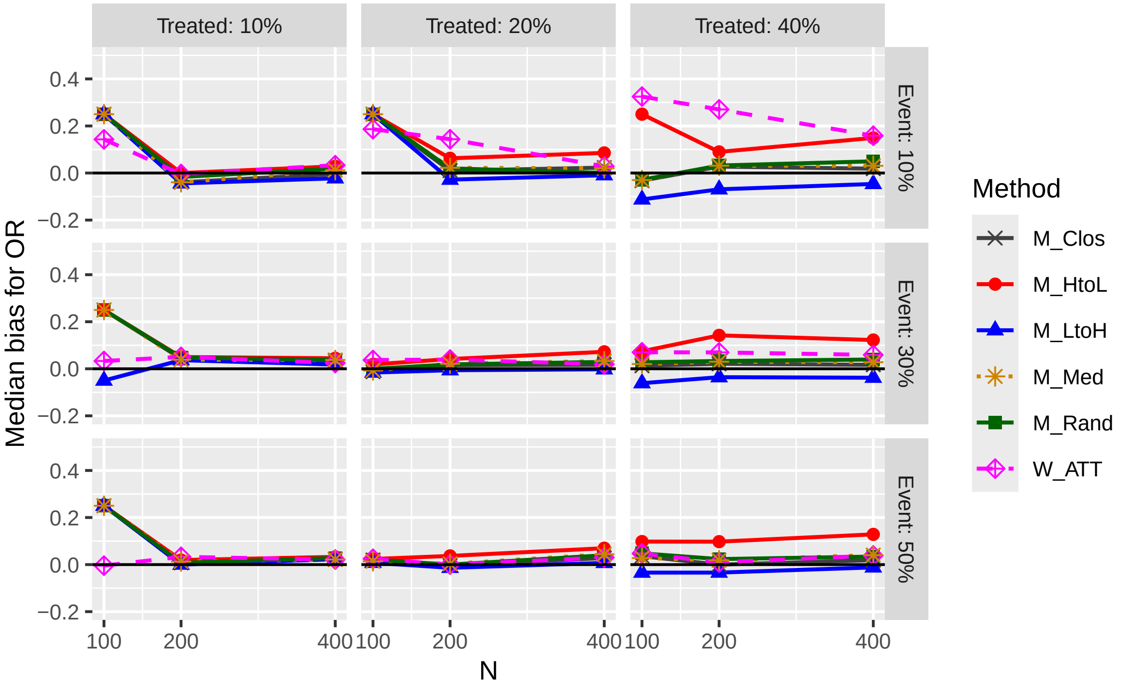

Figure S17. Median bias for OR (unimodal continuous covariate, matching ratio 1:1, true OR: 0.75, c statistic: 0.85).

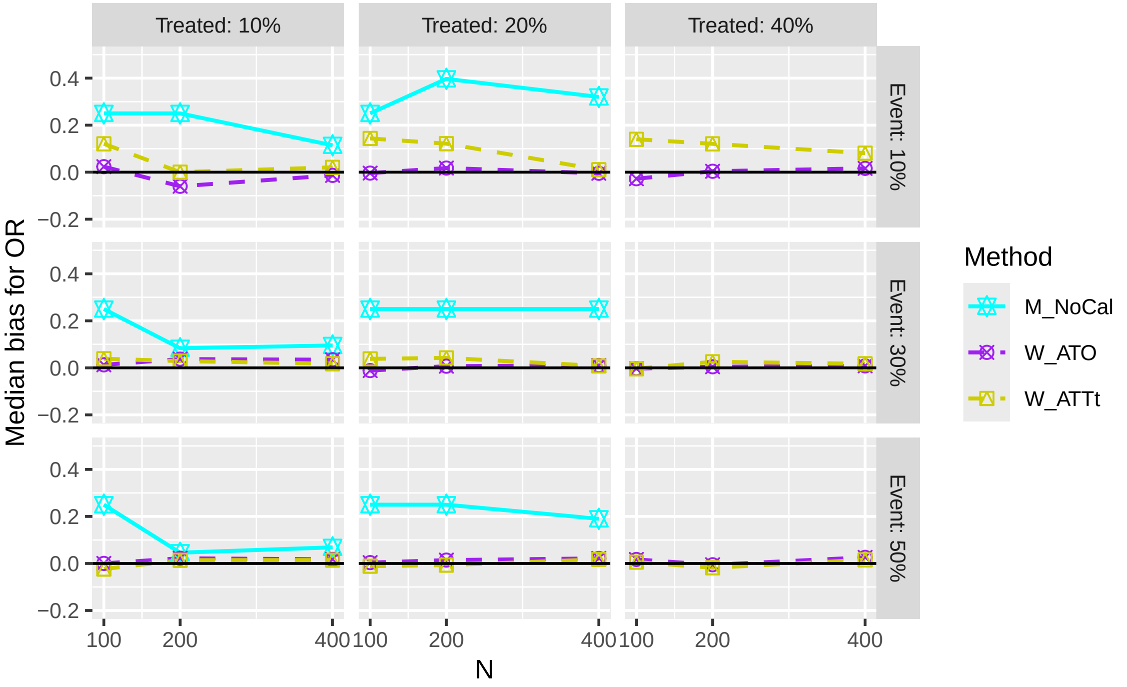

Figure S18. Median bias for OR (unimodal continuous covariate, matching ratio 1:1, true OR: 0.75, c statistic: 0.85); other methods.

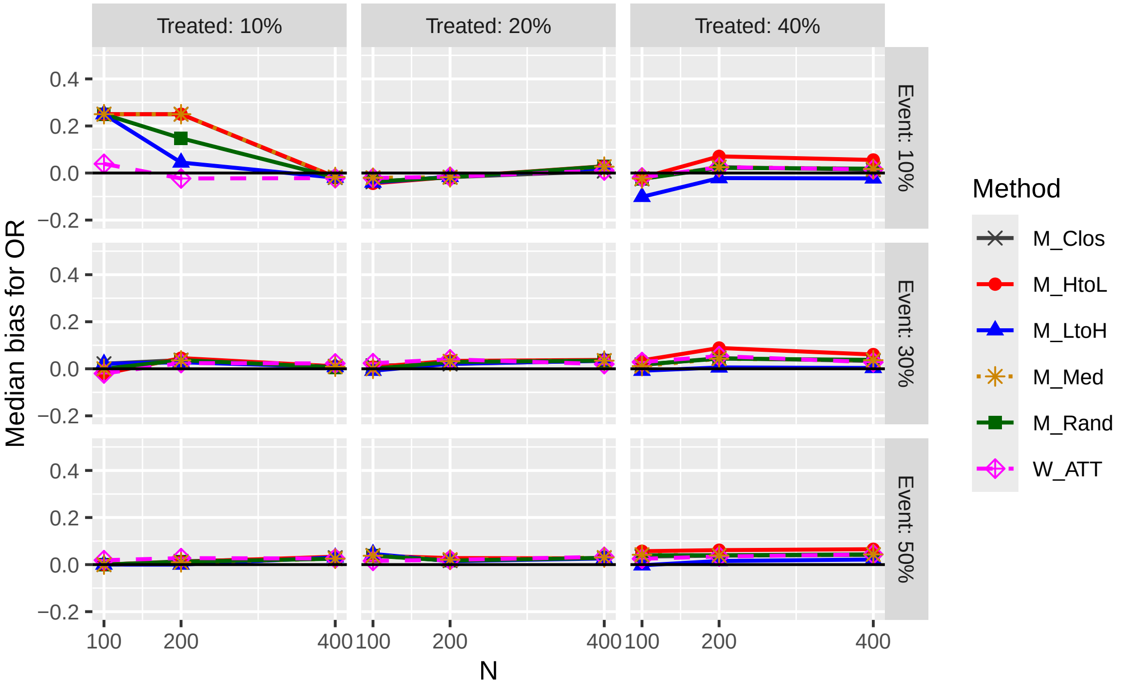

Figure S19. Median bias for OR (unimodal continuous covariate, matching ratio 1:1, true OR: 0.75, c statistic: 0.6).

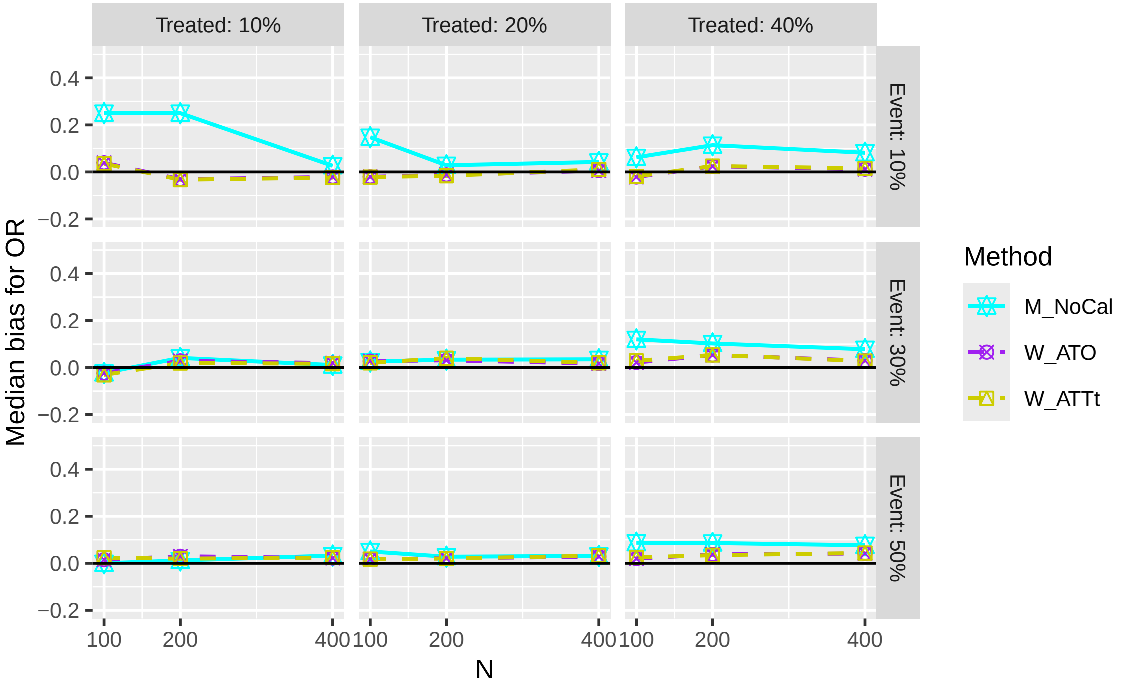

Figure S20. Median bias for OR (unimodal continuous covariate, matching ratio 1:1, true OR: 0.75, c statistic: 0.6); other methods.

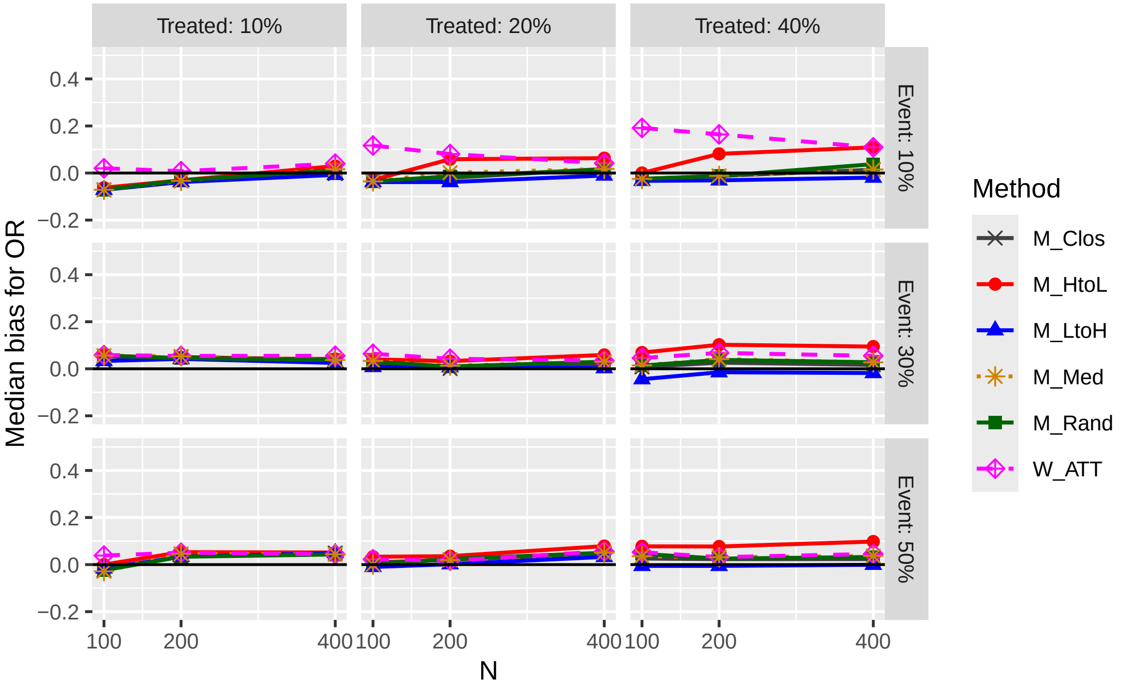

Figure S21. Median bias for OR (unimodal continuous covariate, matching ratio 1:1, true OR: 0.5, c statistic: 0.85).

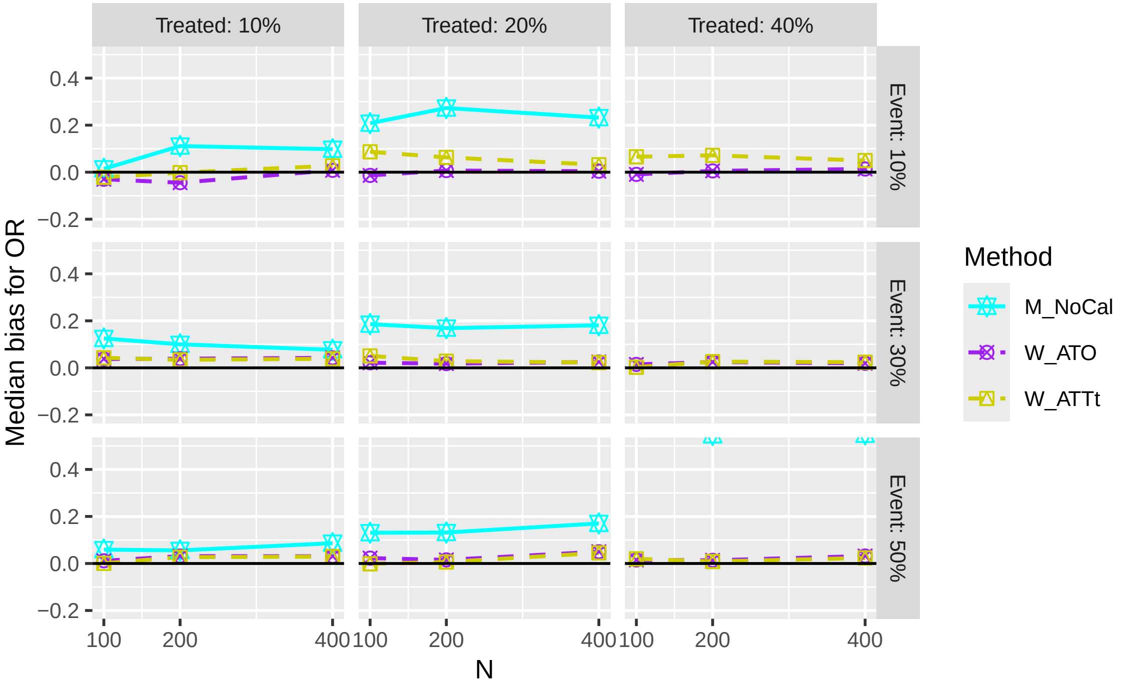

Figure S22. Median bias for OR (unimodal continuous covariate, matching ratio 1:1, true OR: 0.5, c statistic: 0.85); other methods.

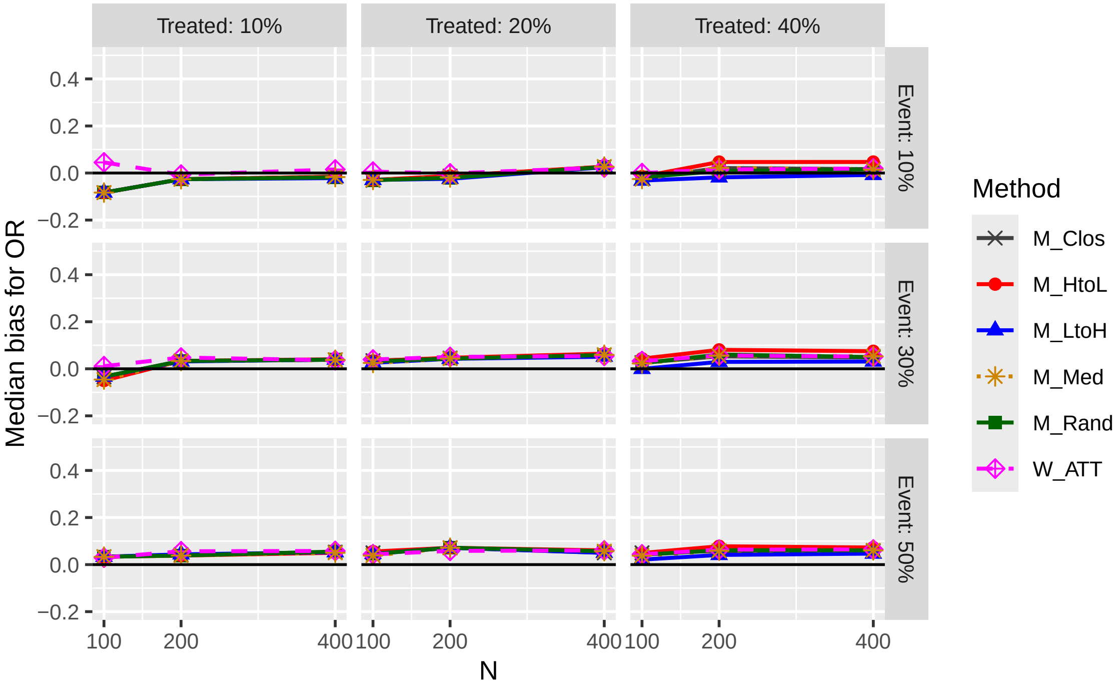

Figure S23. Median bias for OR (unimodal continuous covariate, matching ratio 1:1, true OR: 0.5, c statistic: 0.6).

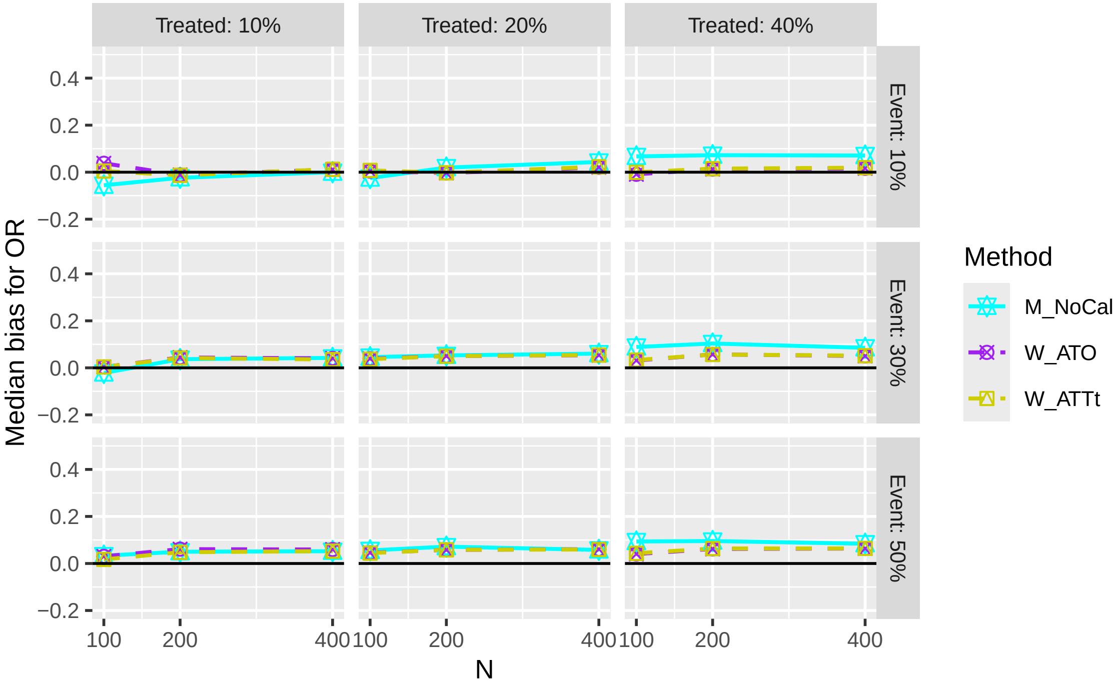

Figure S24. Median bias for OR (unimodal continuous covariate, matching ratio 1:1, true OR: 0.5, c statistic: 0.6); other methods.

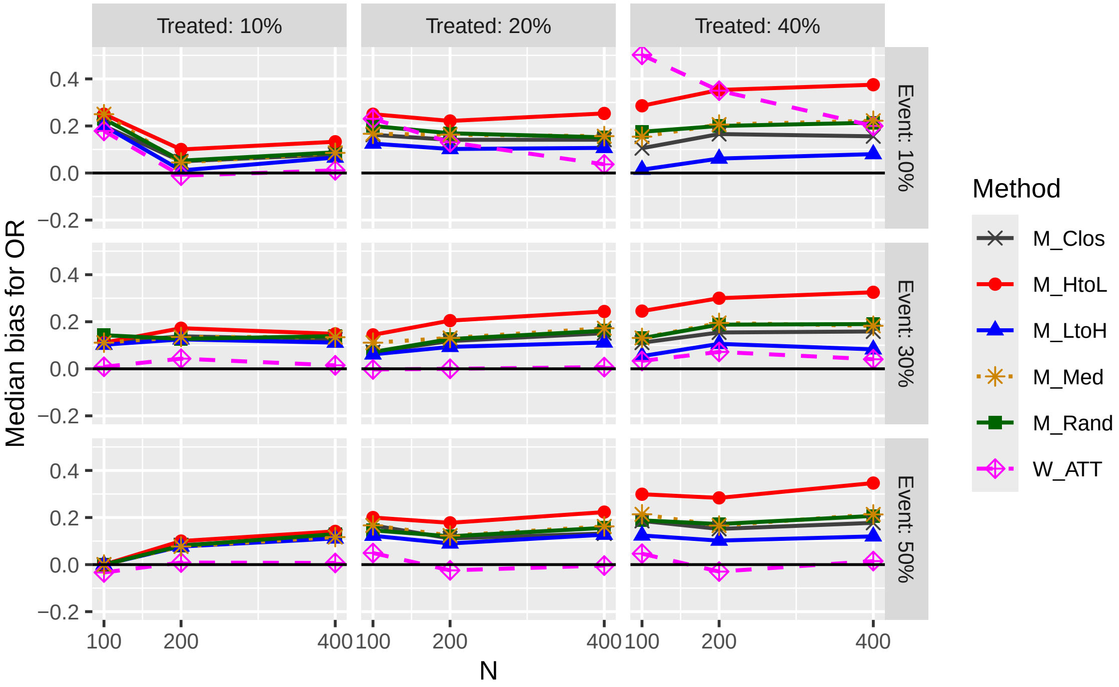

Figure S25. Median bias for OR (unimodal continuous covariate, matching ratio 1:2, true OR: 1, c statistic: 0.85).

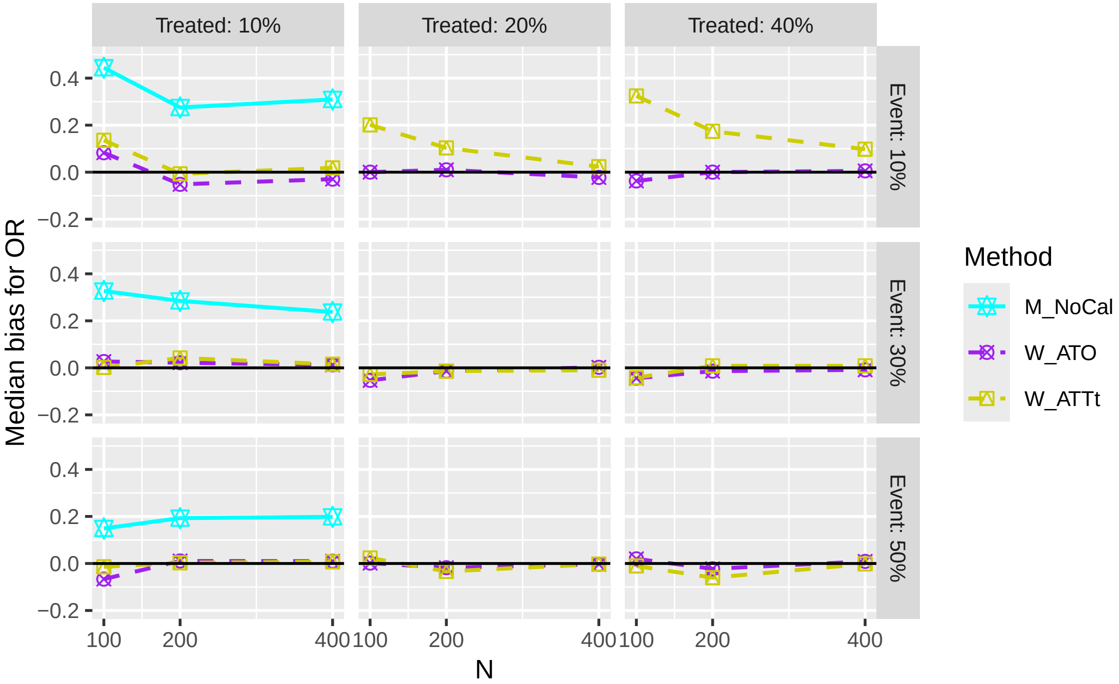

Figure S26. Median bias for OR (unimodal continuous covariate, matching ratio 1:2, true OR: 1, c statistic: 0.85); other methods.

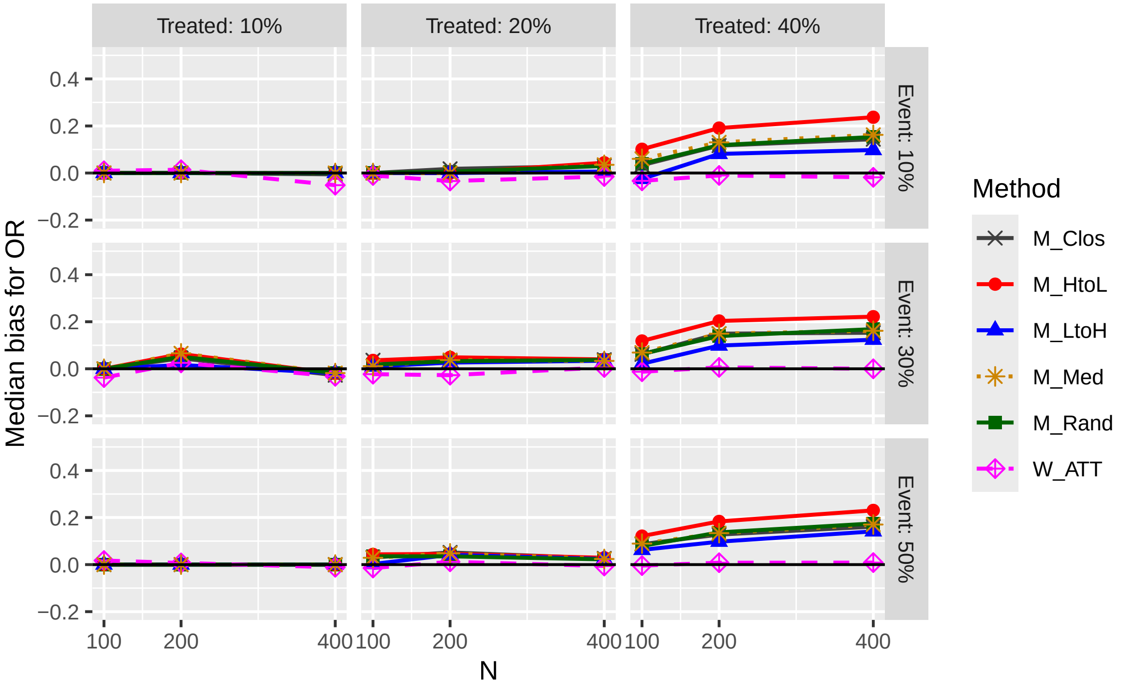

Figure S27. Median bias for OR (unimodal continuous covariate, matching ratio 1:2, true OR: 1, c statistic: 0.6).

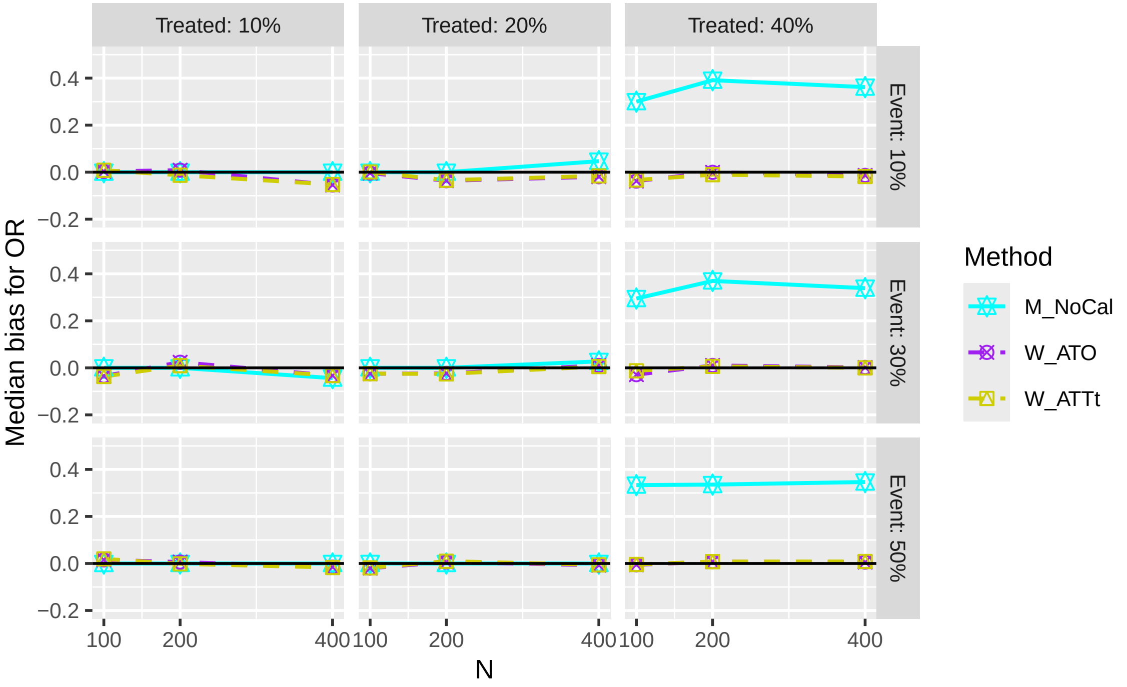

Figure S28. Median bias for OR (unimodal continuous covariate, matching ratio 1:2, true OR: 1, c statistic: 0.6); other methods.

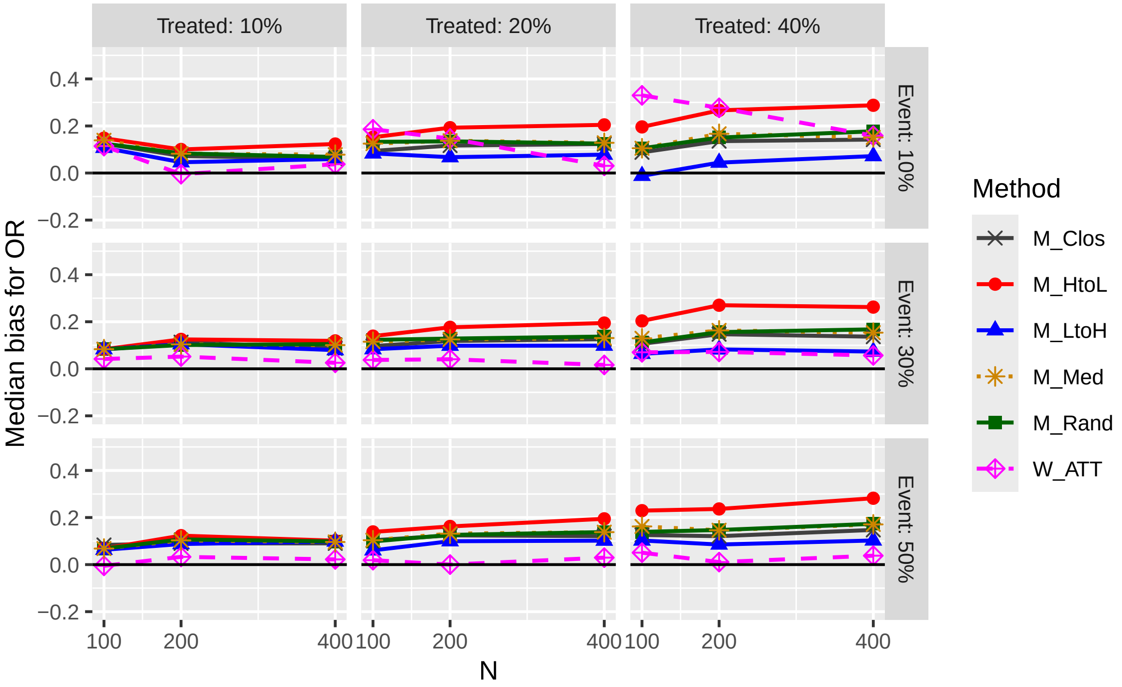

Figure S29. Median bias for OR (unimodal continuous covariate, matching ratio 1:2, true OR: 0.75, c statistic: 0.85).

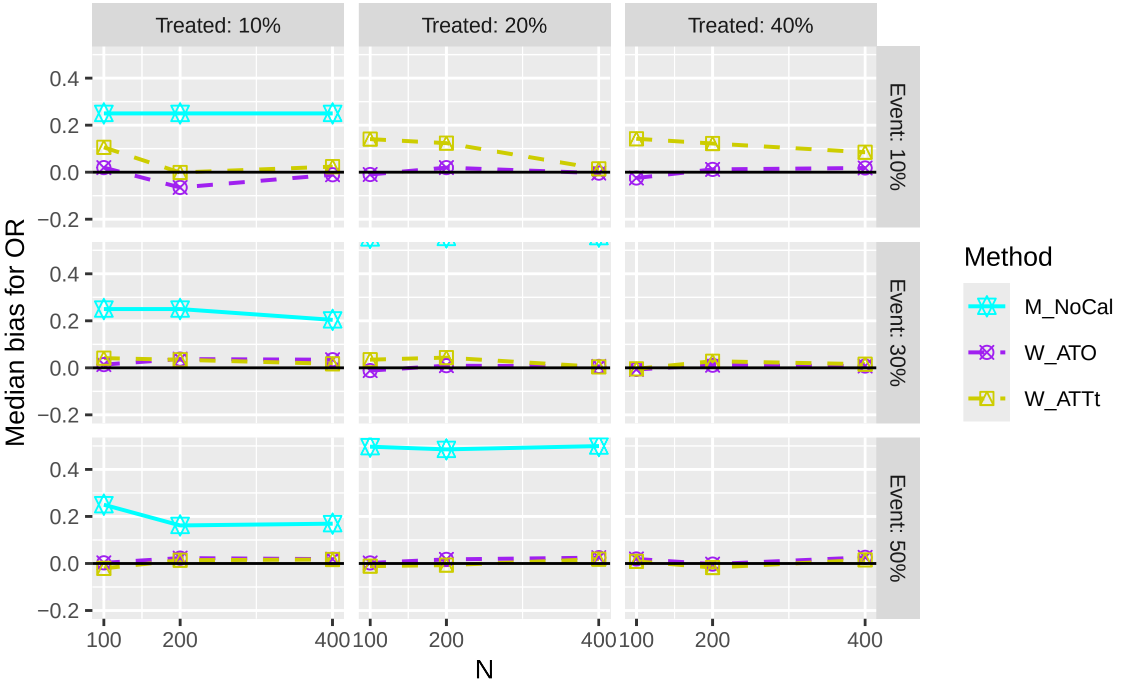

Figure S30. Median bias for OR (unimodal continuous covariate, matching ratio 1:2, true OR: 0.75, c statistic: 0.85); other methods.

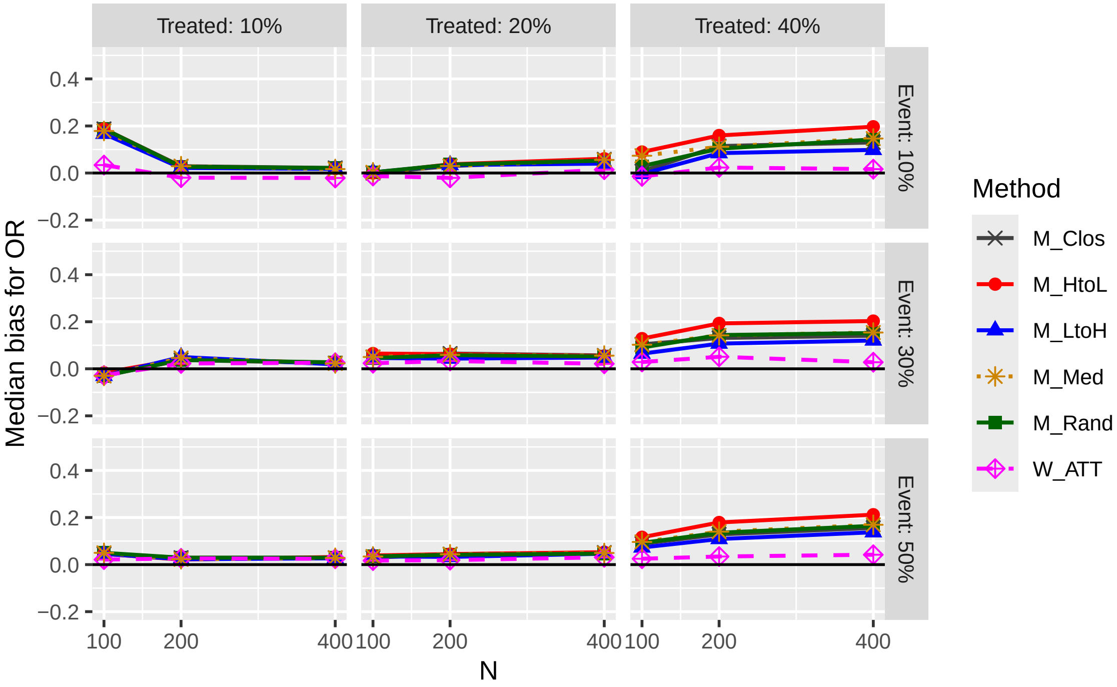

Figure S31. Median bias for OR (unimodal continuous covariate, matching ratio 1:2, true OR: 0.75, c statistic: 0.6).

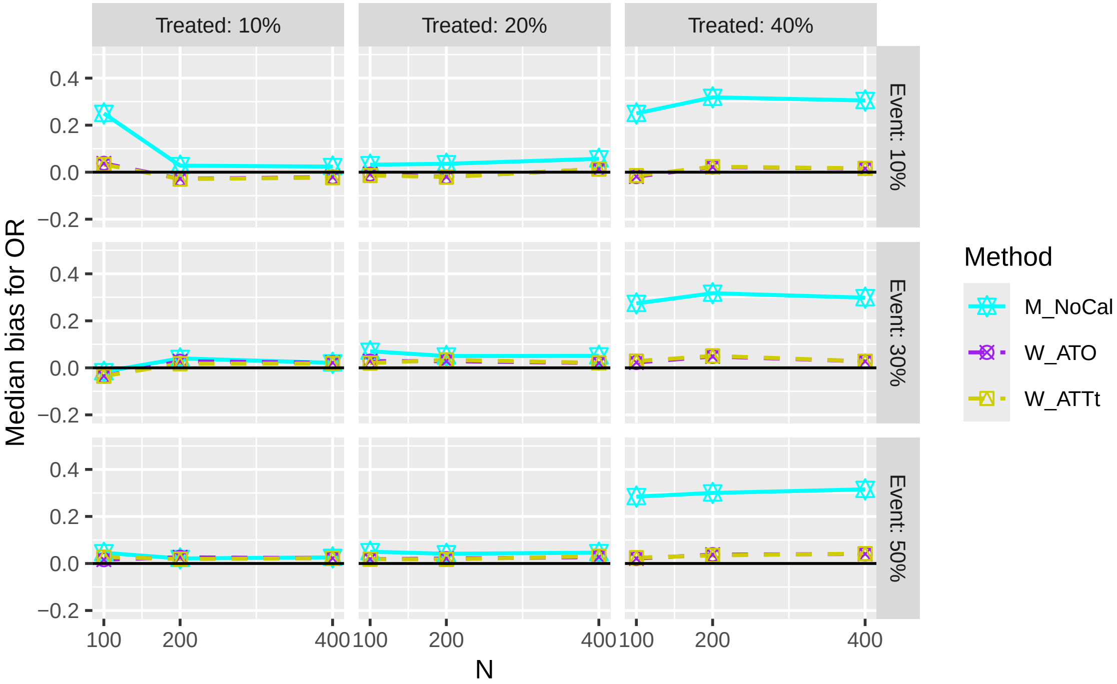

Figure S32. Median bias for OR (unimodal continuous covariate, matching ratio 1:2, true OR: 0.75, c statistic: 0.6); other methods.

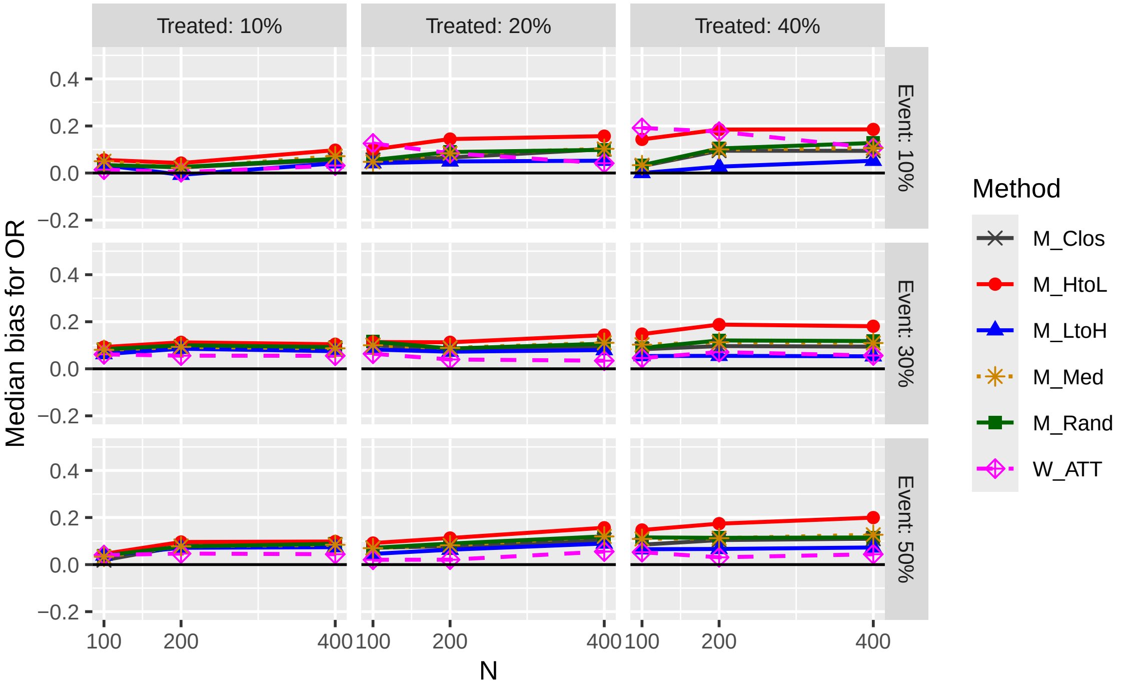

Figure S33. Median bias for OR (unimodal continuous covariate, matching ratio 1:2, true OR: 0.5, c statistic: 0.85).

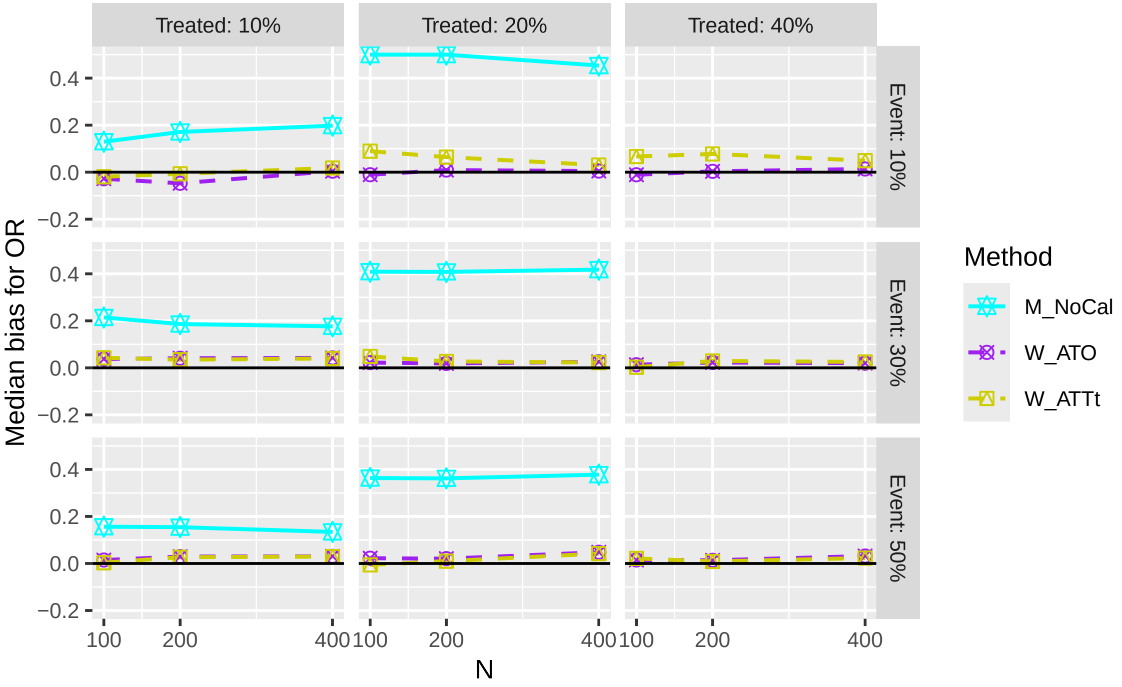

Figure S34. Median bias for OR (unimodal continuous covariate, matching ratio 1:2, true OR: 0.5, c statistic: 0.85); other methods.

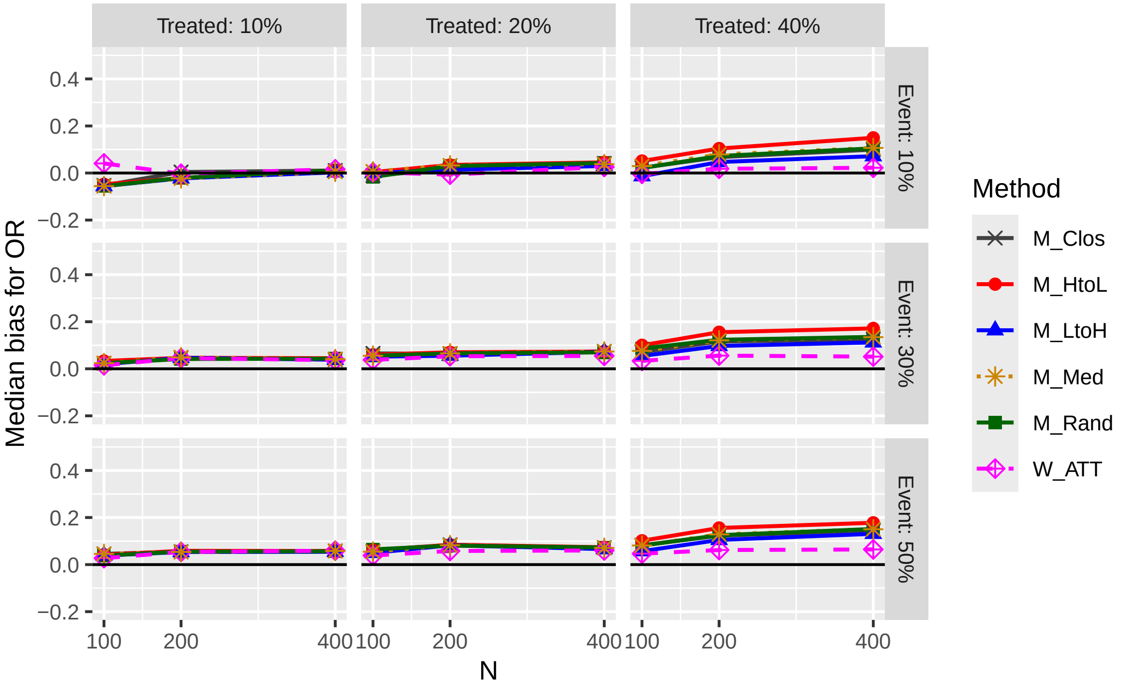

Figure S35. Median bias for OR (unimodal continuous covariate, matching ratio 1:2, true OR: 0.5, c statistic: 0.6).

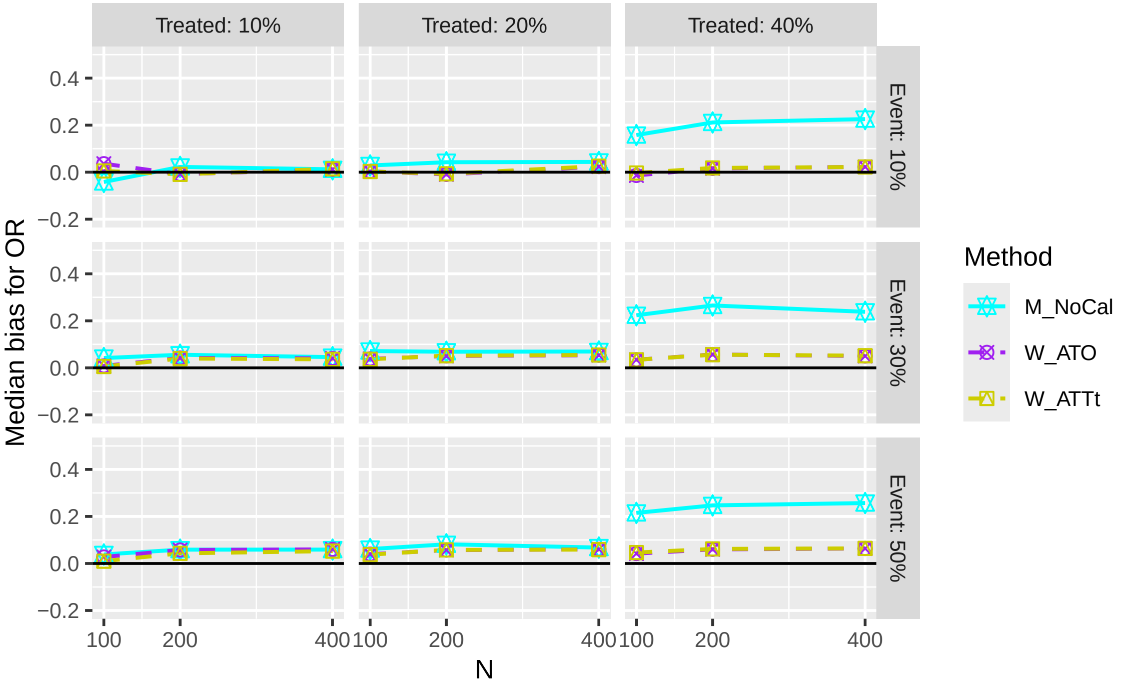

Figure S36. Median bias for OR (unimodal continuous covariate, matching ratio 1:2, true OR: 0.5, c statistic: 0.6); other methods.

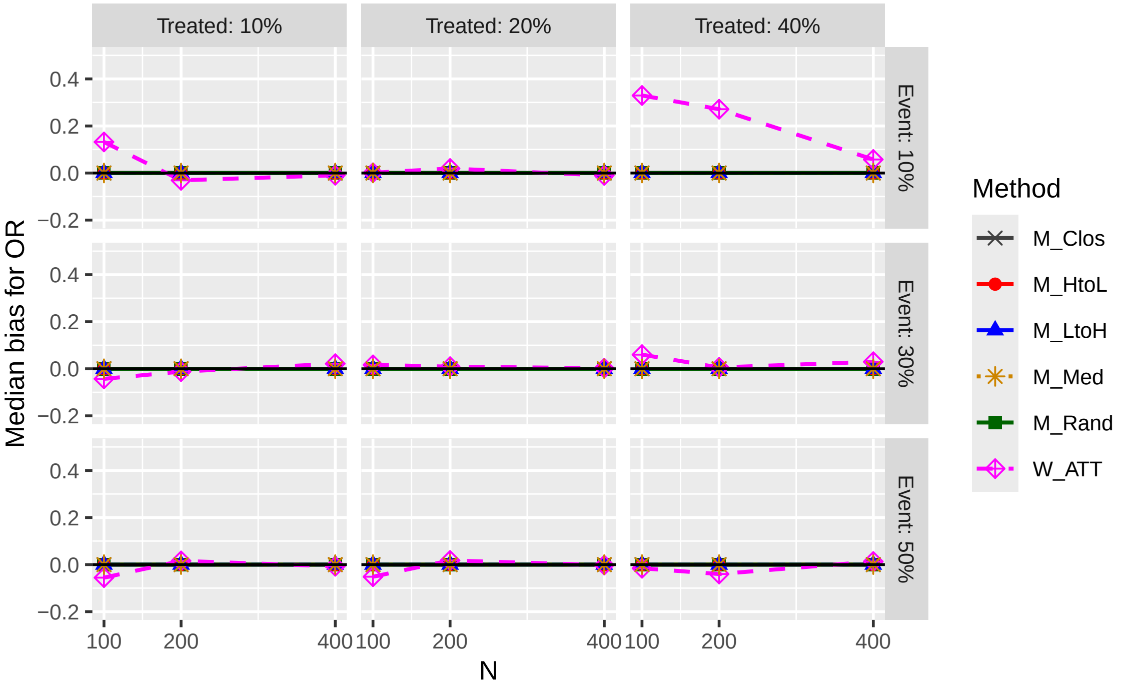

Figure S37. Median bias for OR (categorical covariate, matching ratio 1:1, true OR: 1, c statistic: 0.85).

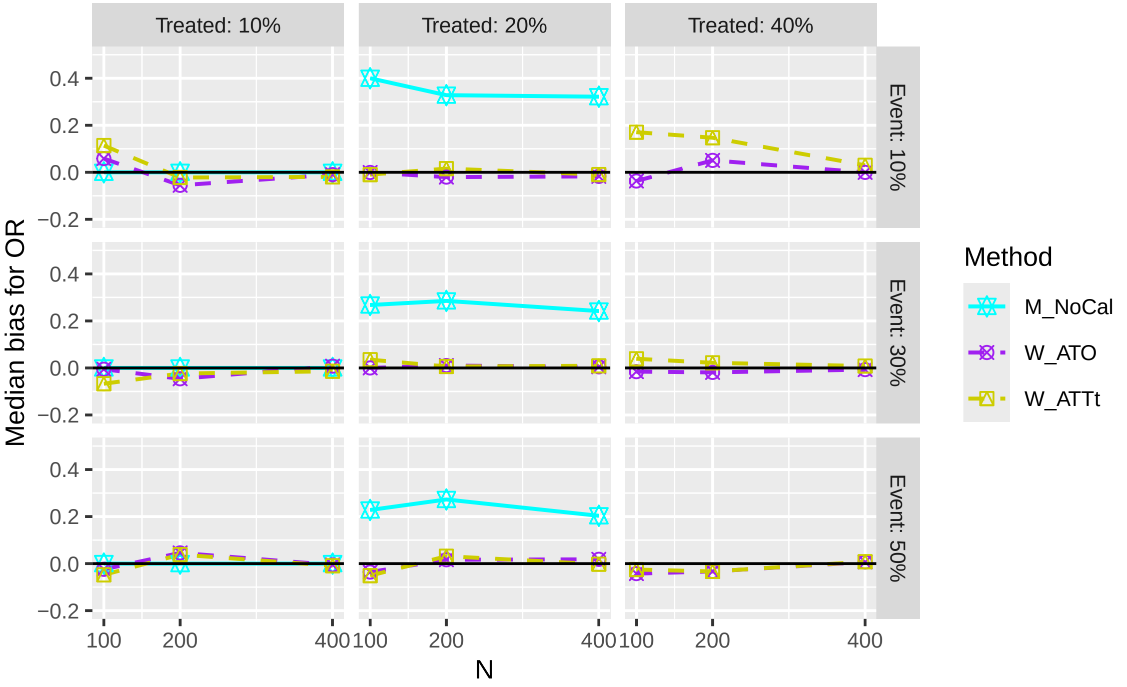

Figure S38. Median bias for OR (categorical covariate, matching ratio 1:1, true OR: 1, c statistic: 0.85); other methods.

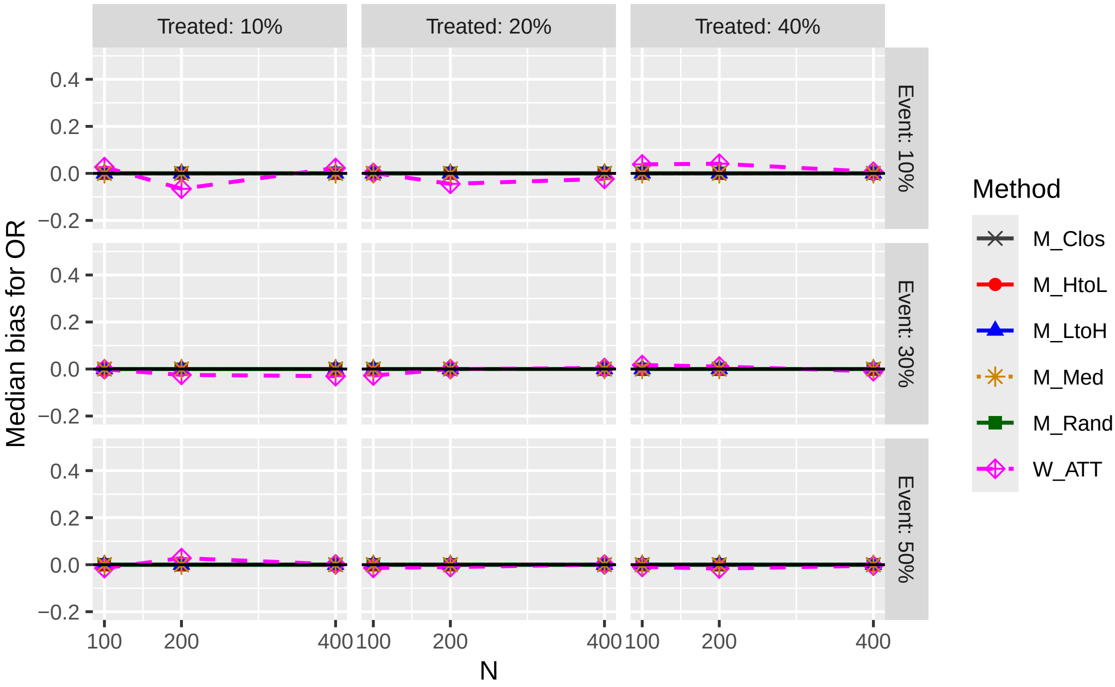

Figure S39. Median bias for OR (categorical covariate, matching ratio 1:1, true OR: 1, c statistic: 0.6).

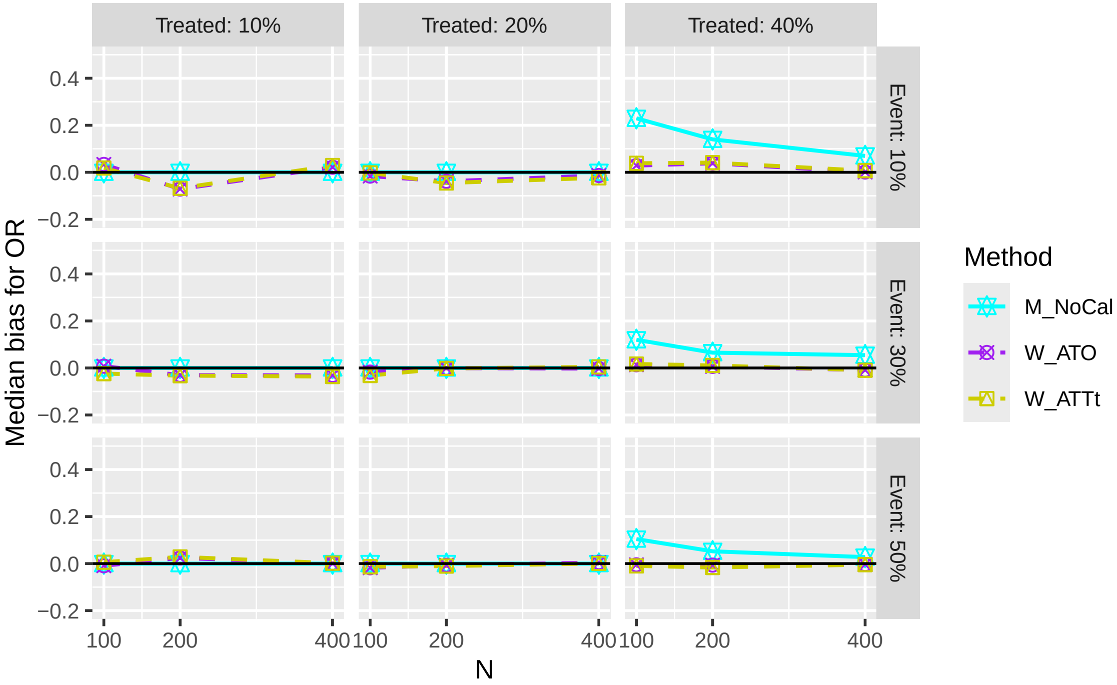

Figure S40. Median bias for OR (categorical covariate, matching ratio 1:1, true OR: 1, c statistic: 0.6); other methods.

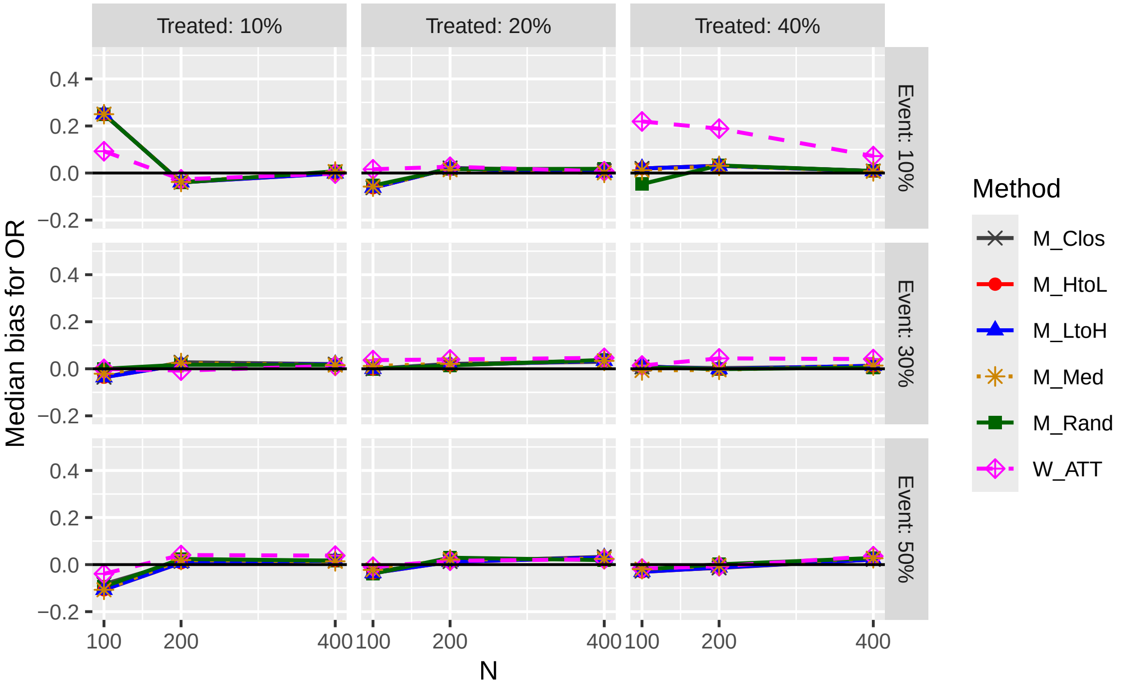

Figure S41. Median bias for OR (categorical covariate, matching ratio 1:1, true OR: 0.75, c statistic: 0.85).

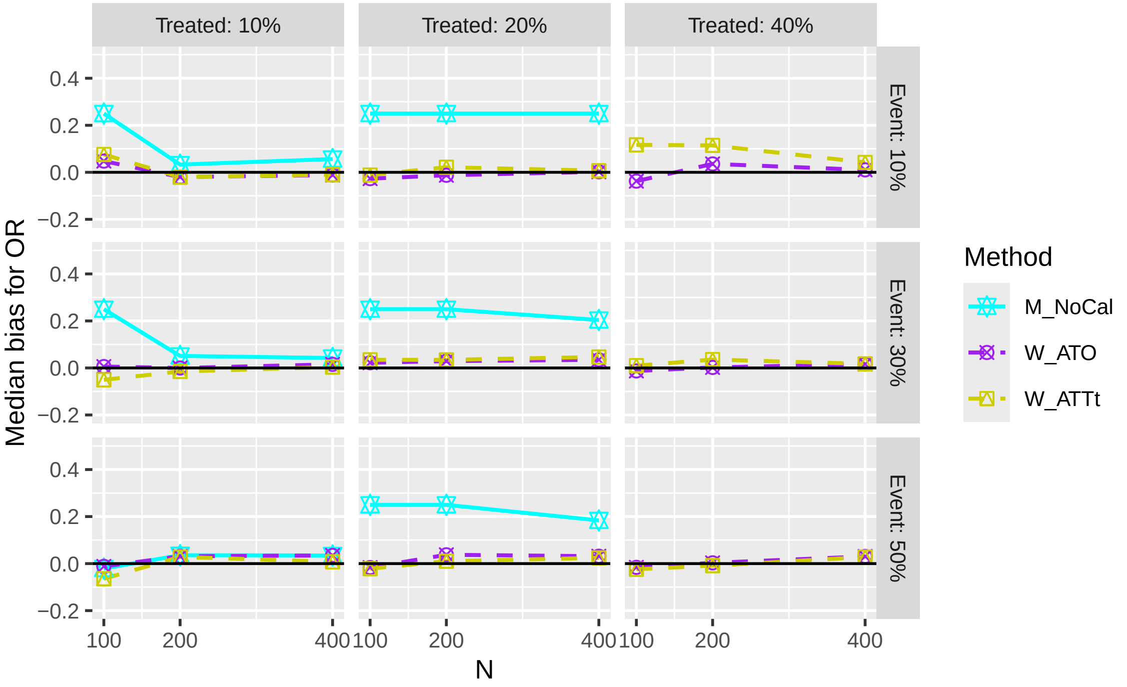

Figure S42. Median bias for OR (categorical covariate, matching ratio 1:1, true OR: 0.75, c statistic: 0.85); other methods.

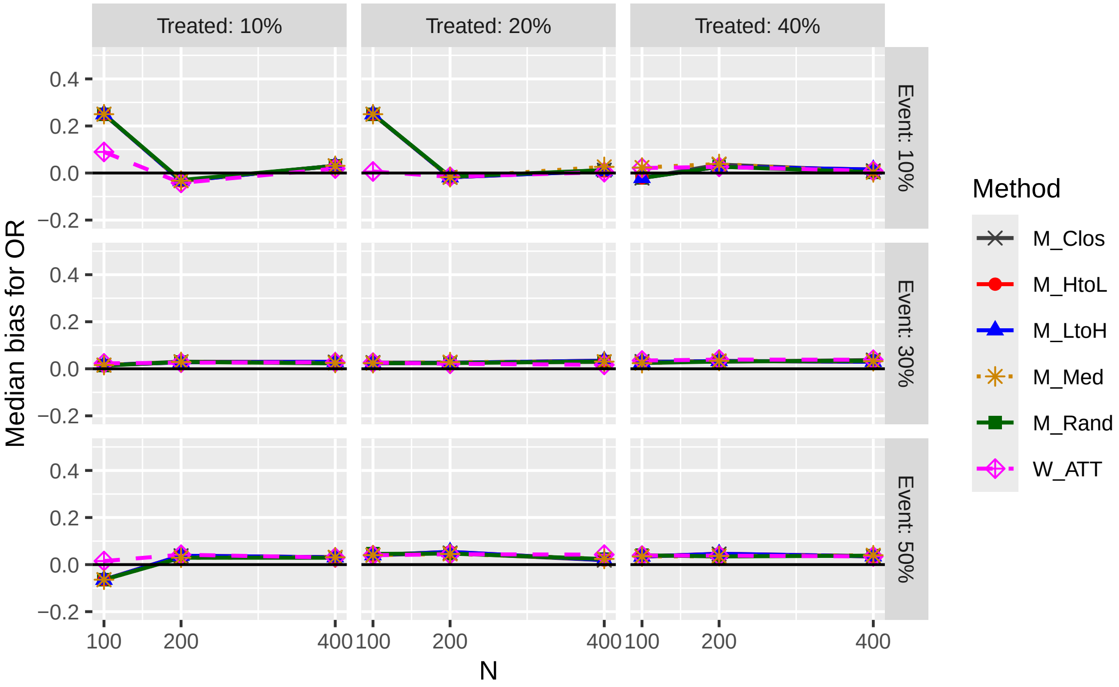

Figure S43. Median bias for OR (categorical covariate, matching ratio 1:1, true OR: 0.75, c statistic: 0.6).

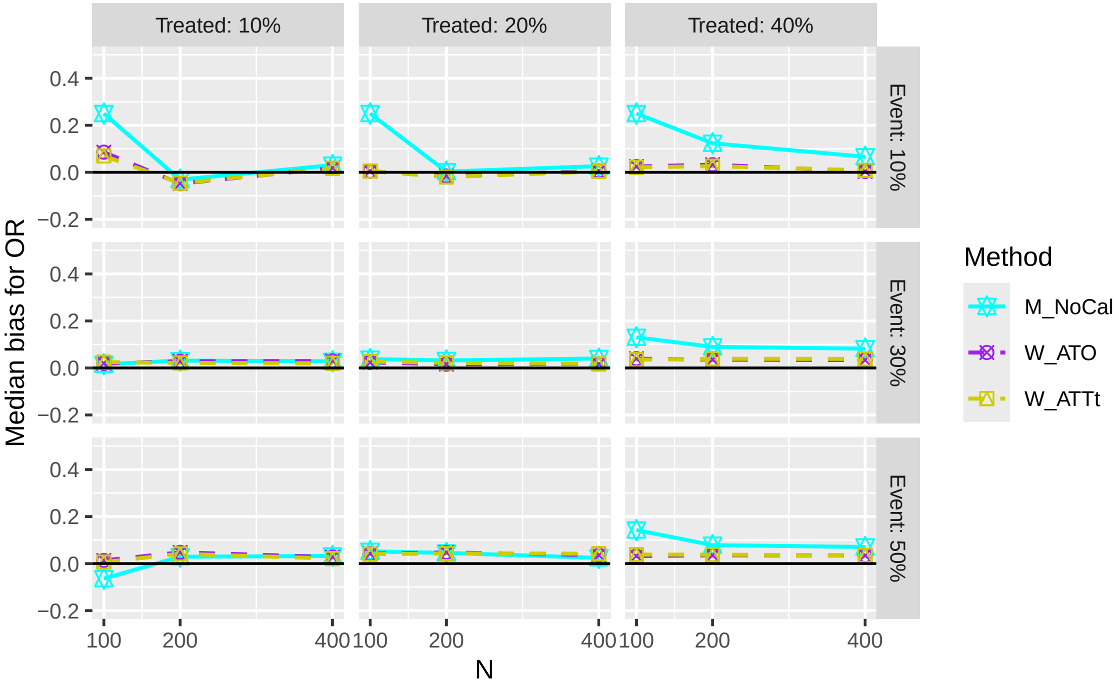

Figure S44. Median bias for OR (categorical covariate, matching ratio 1:1, true OR: 0.75, c statistic: 0.6); other methods.

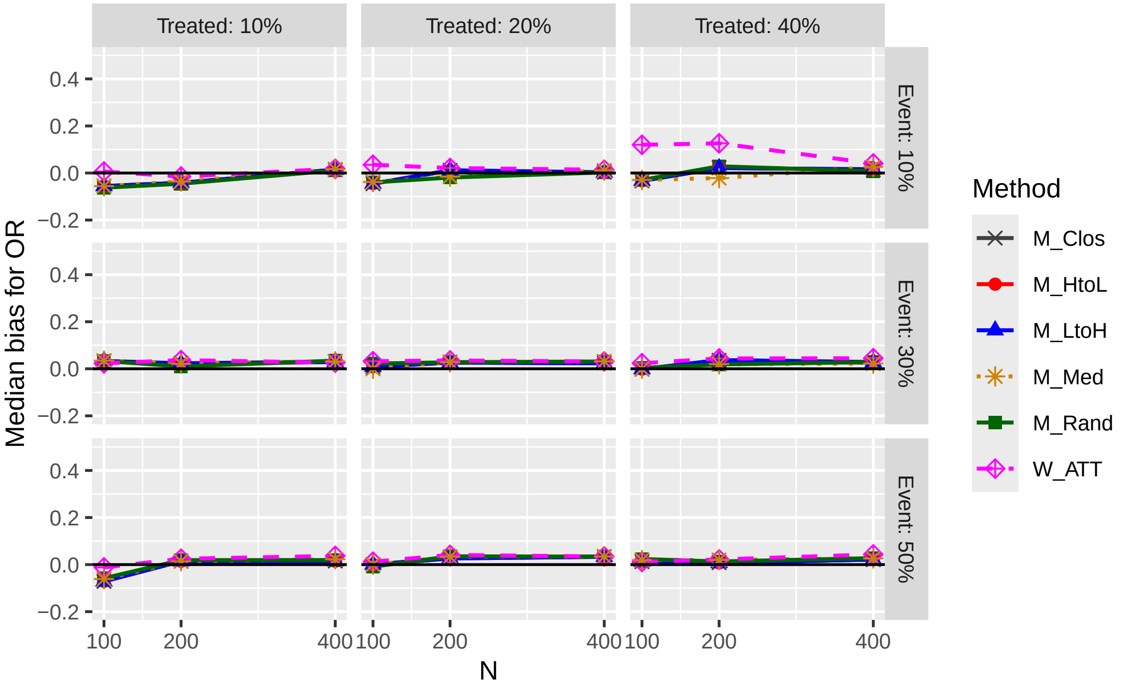

Figure S45. Median bias for OR (categorical covariate, matching ratio 1:1, true OR: 0.5, c statistic: 0.85).

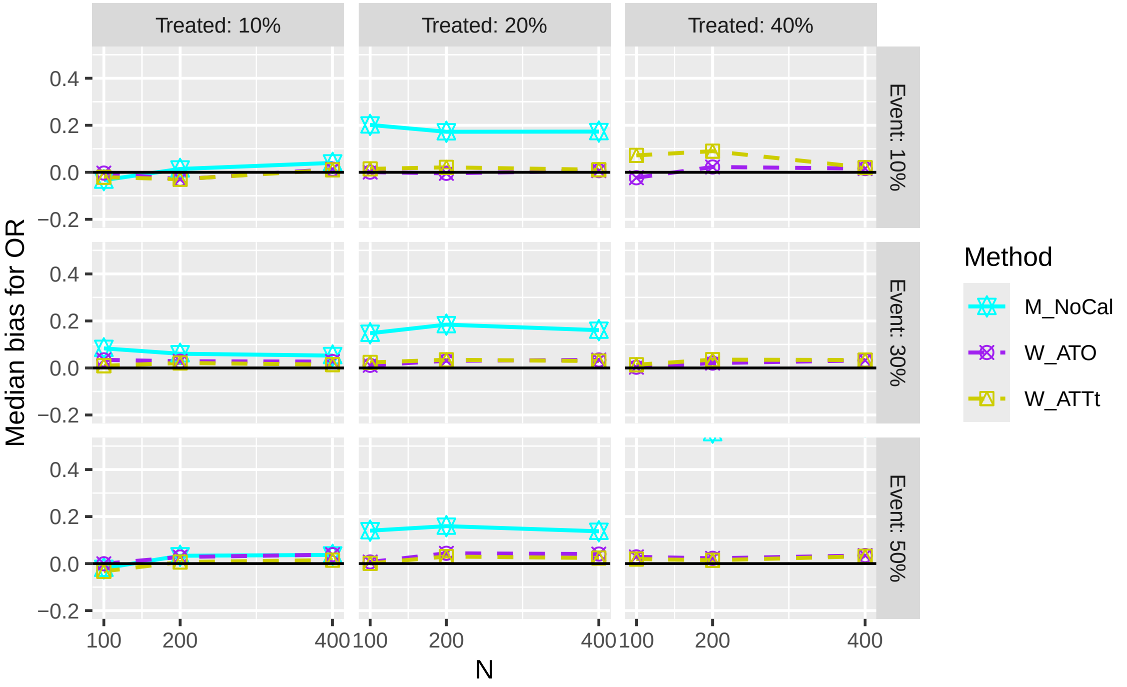

Figure S46. Median bias for OR (categorical covariate, matching ratio 1:1, true OR: 0.5, c statistic: 0.85); other methods.

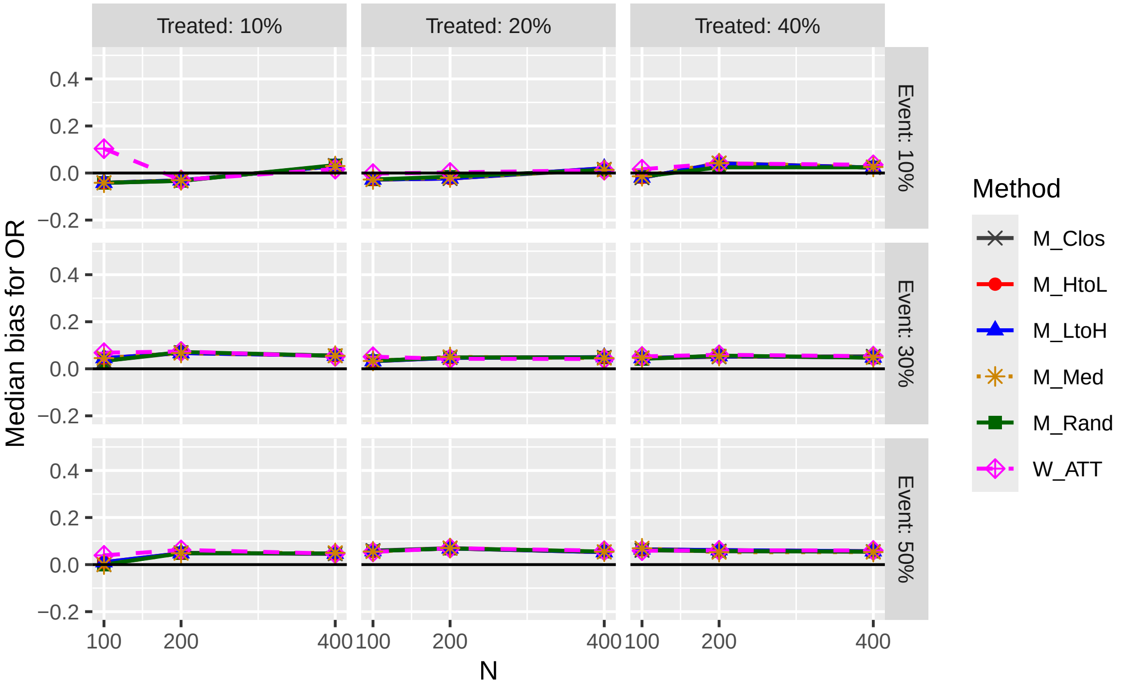

Figure S47. Median bias for OR (categorical covariate, matching ratio 1:1, true OR: 0.5, c statistic: 0.6).

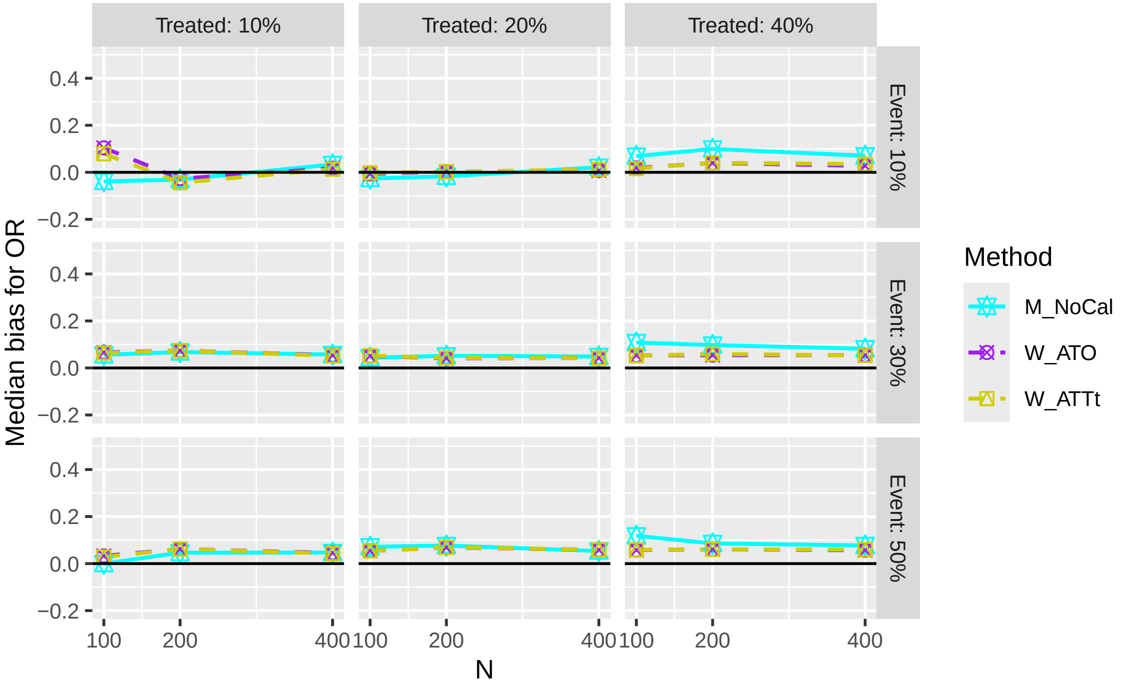

Figure S48. Median bias for OR (categorical covariate, matching ratio 1:1, true OR: 0.5, c statistic: 0.6); other methods.

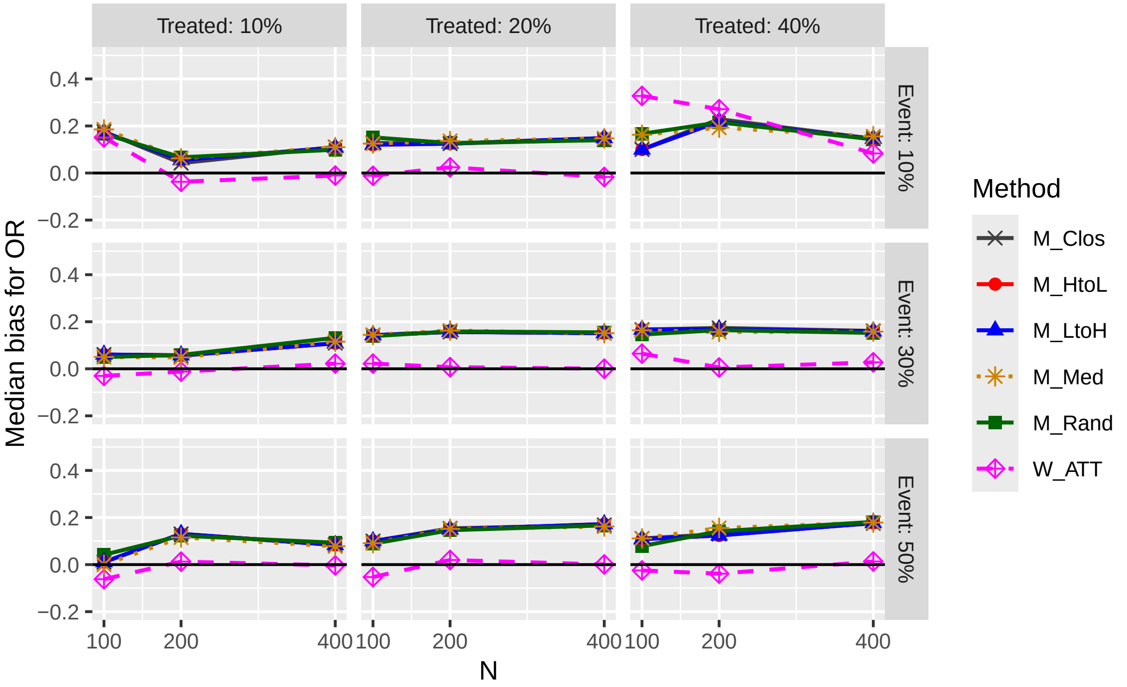

Figure S49. Median bias for OR (categorical covariate, matching ratio 1:2, true OR: 1, c statistic: 0.85).

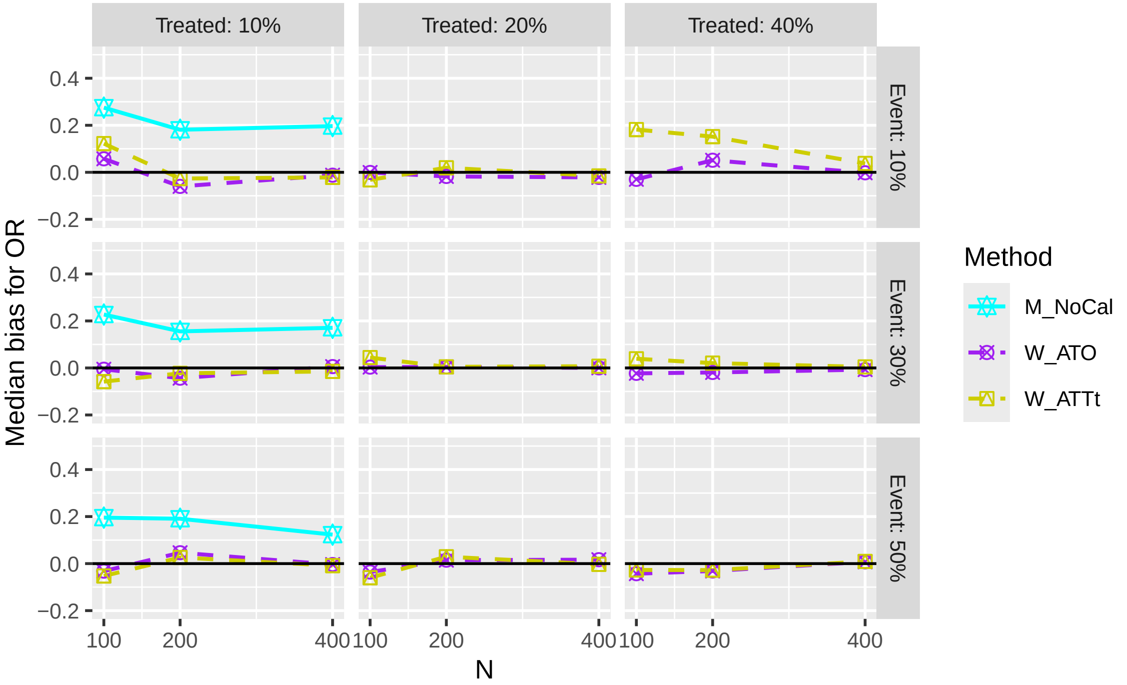

Figure S50. Median bias for OR (categorical covariate, matching ratio 1:2, true OR: 1, c statistic: 0.85); other methods.

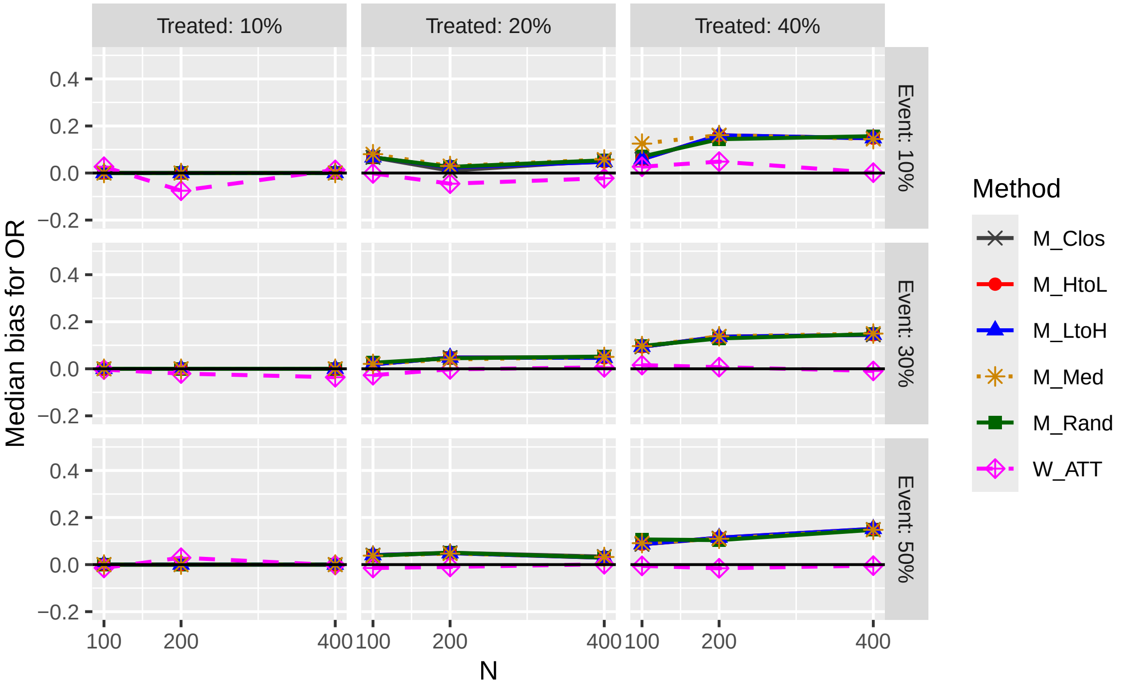

Figure S51. Median bias for OR (categorical covariate, matching ratio 1:2, true OR: 1, c statistic: 0.6).

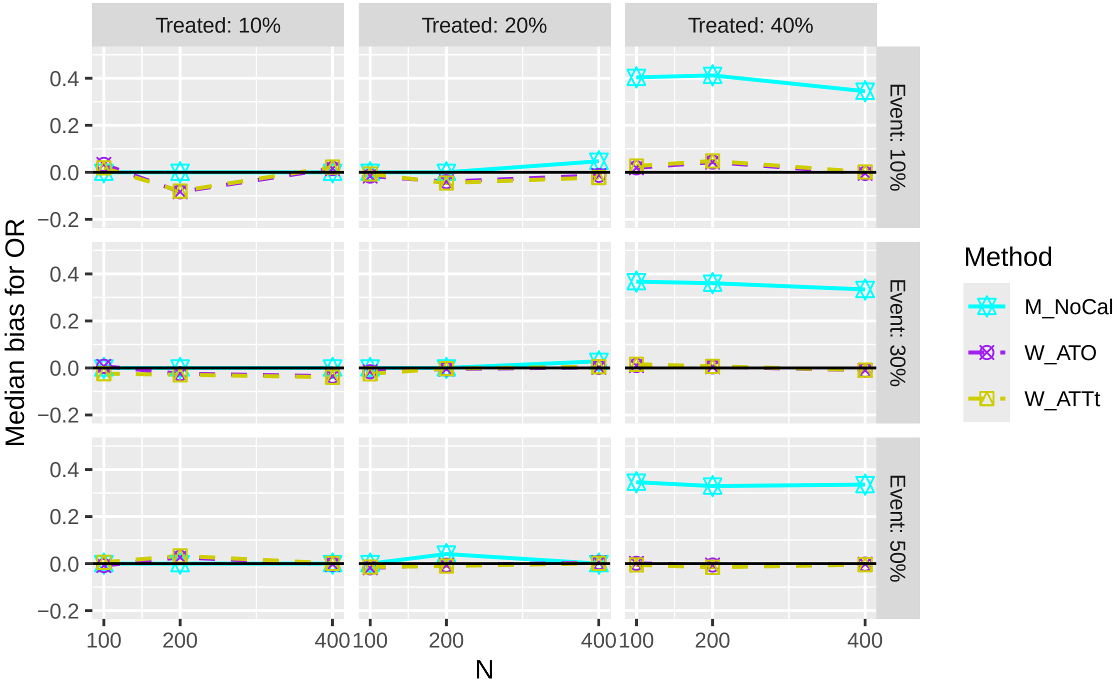

Figure S52. Median bias for OR (categorical covariate, matching ratio 1:2, true OR: 1, c statistic: 0.6); other methods.

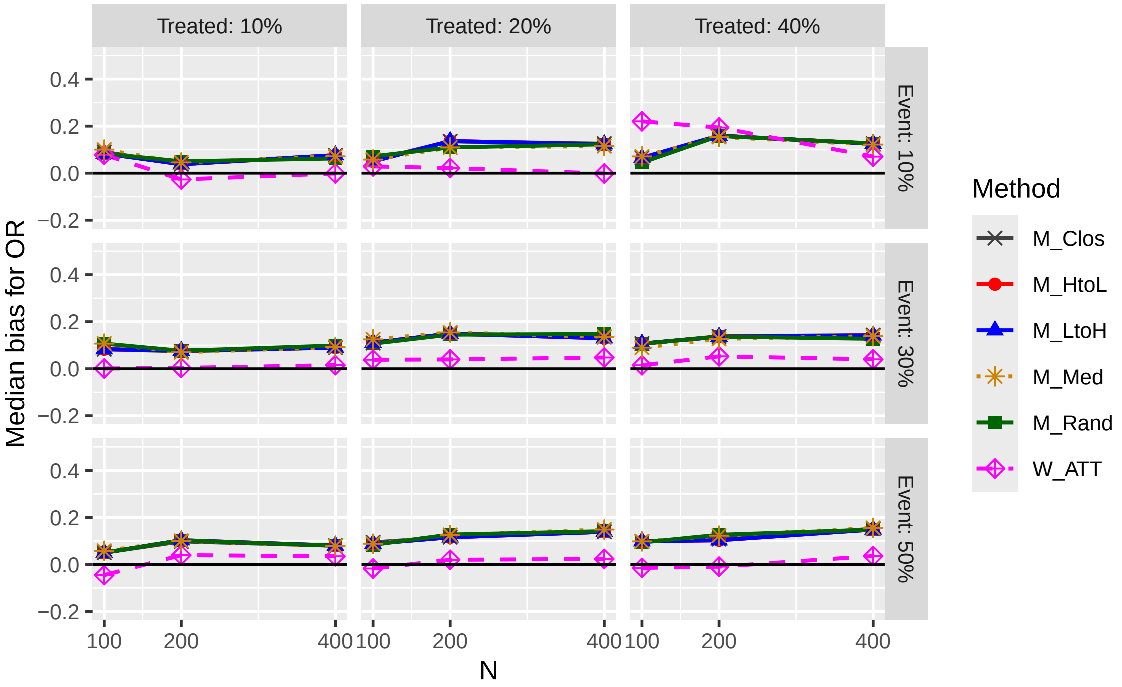

Figure S53. Median bias for OR (categorical covariate, matching ratio 1:2, true OR: 0.75, c statistic: 0.85).

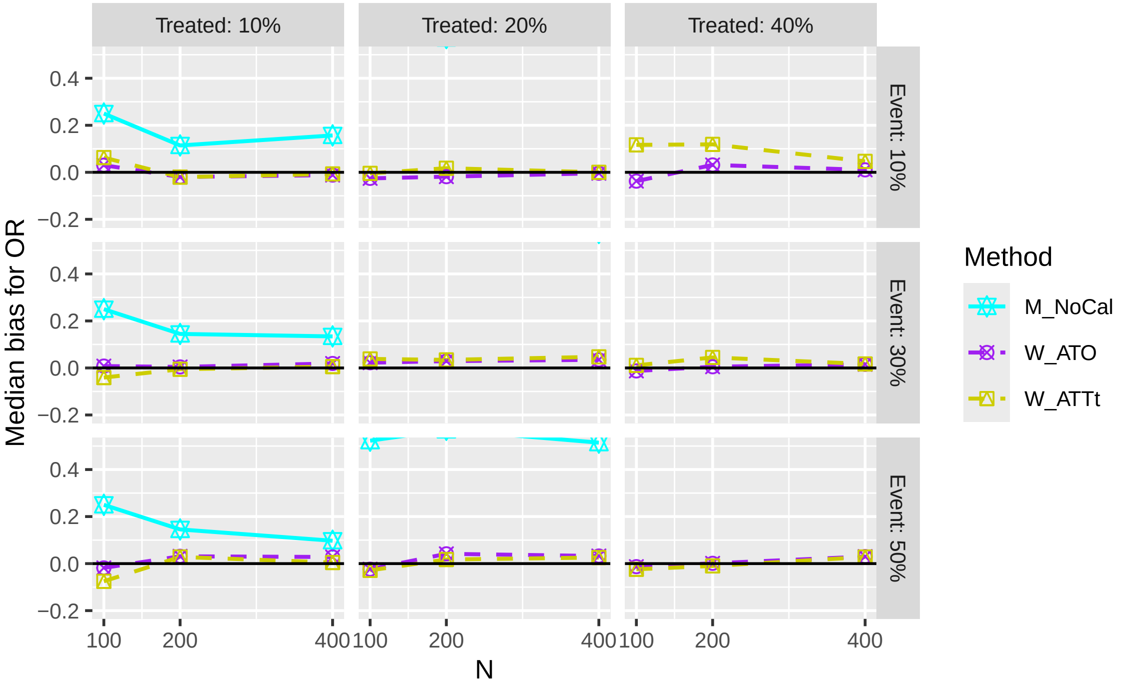

Figure S54. Median bias for OR (categorical covariate, matching ratio 1:2, true OR: 0.75, c statistic: 0.85); other methods.

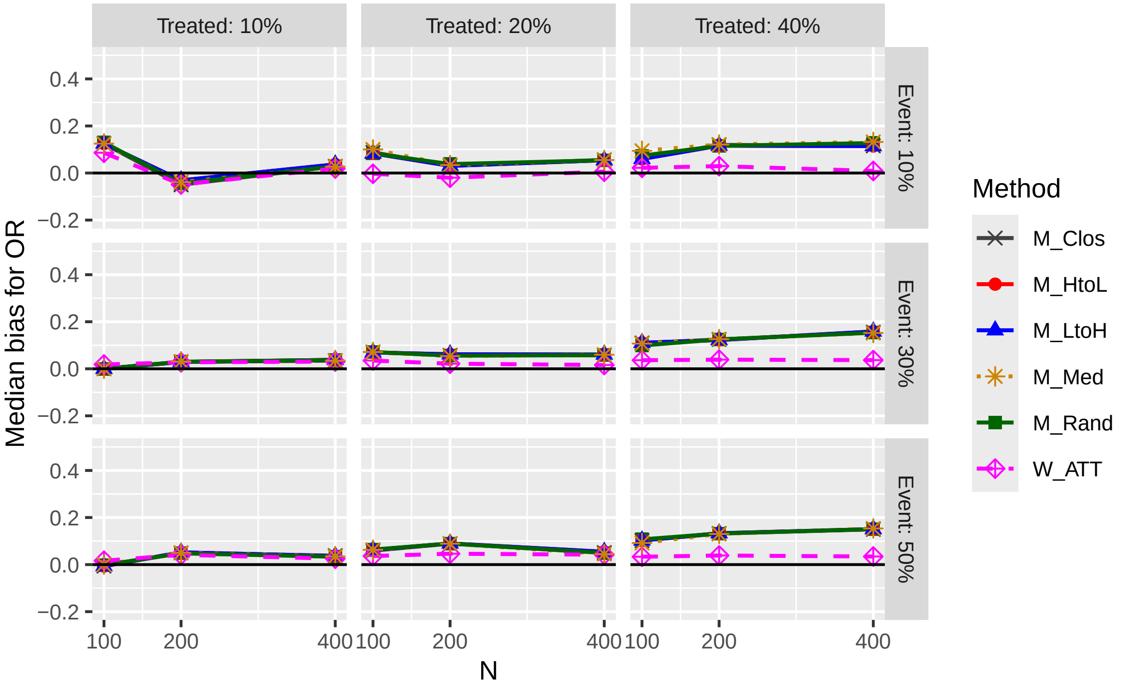

Figure S55. Median bias for OR (categorical covariate, matching ratio 1:2, true OR: 0.75, c statistic: 0.6).

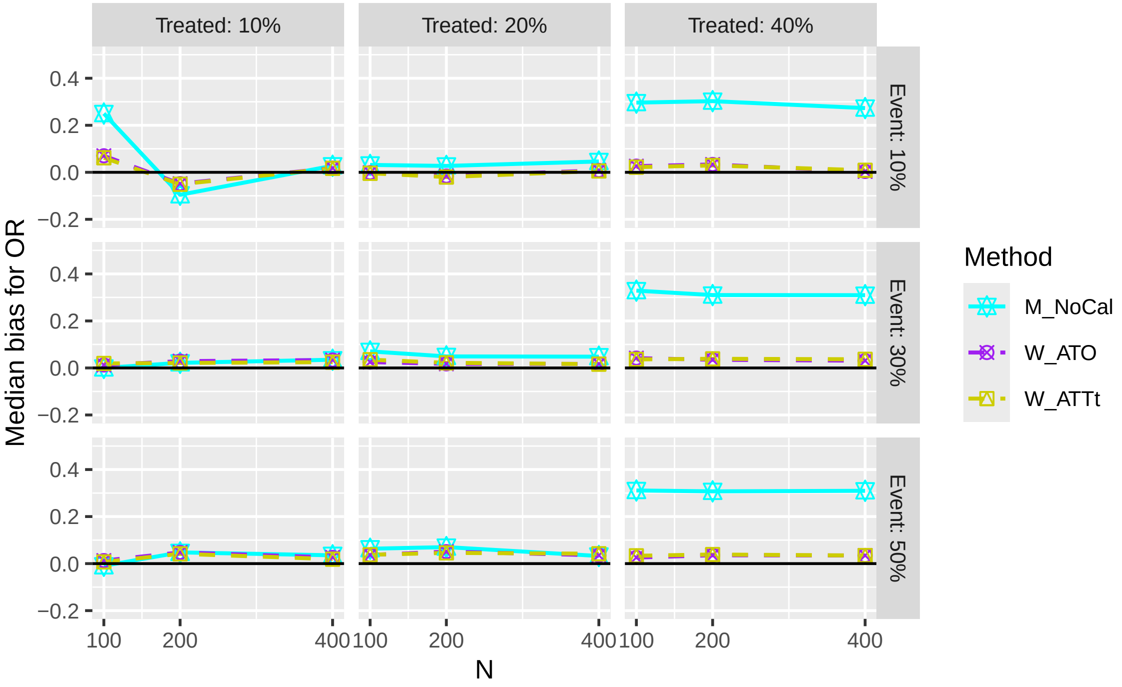

Figure S56. Median bias for OR (categorical covariate, matching ratio 1:2, true OR: 0.75, c statistic: 0.6); other methods.

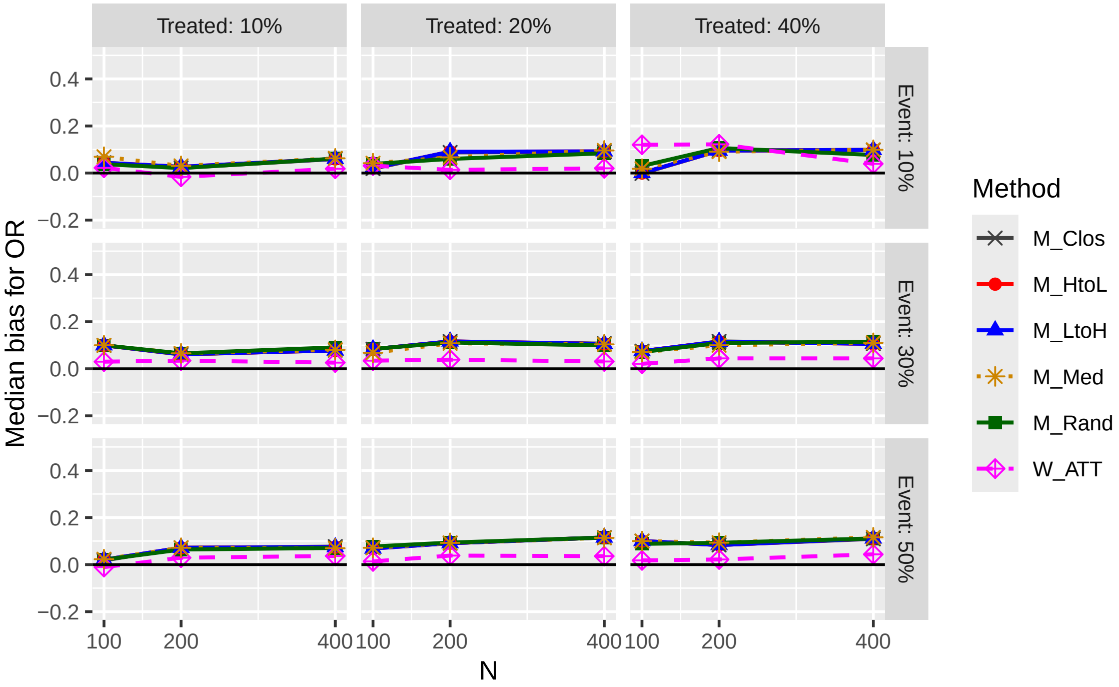

Figure S57. Median bias for OR (categorical covariate, matching ratio 1:2, true OR: 0.5, c statistic: 0.85).

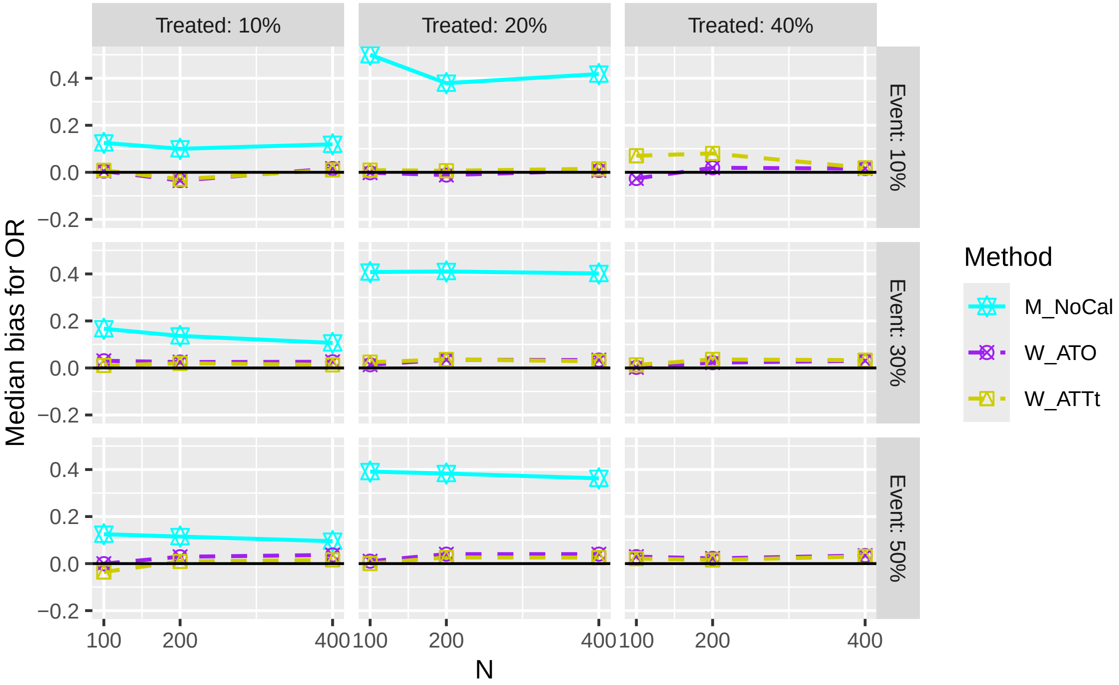

Figure S58. Median bias for OR (categorical covariate, matching ratio 1:2, true OR: 0.5, c statistic: 0.85); other methods.

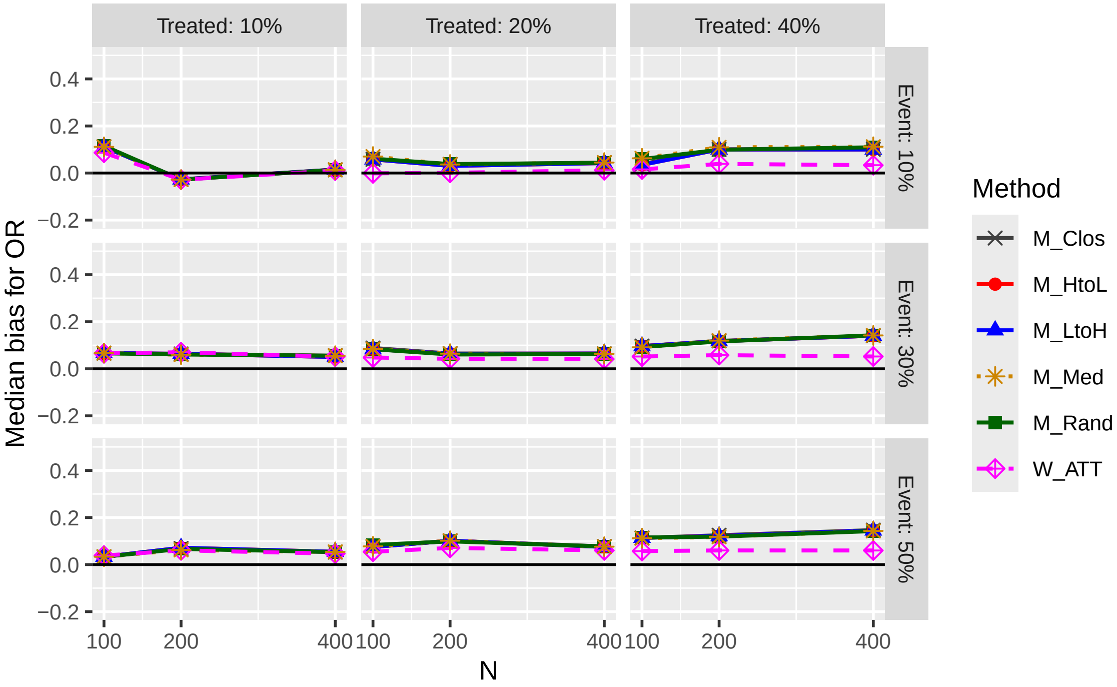

Figure S59. Median bias for OR (categorical covariate, matching ratio 1:2, true OR: 0.5, c statistic: 0.6).

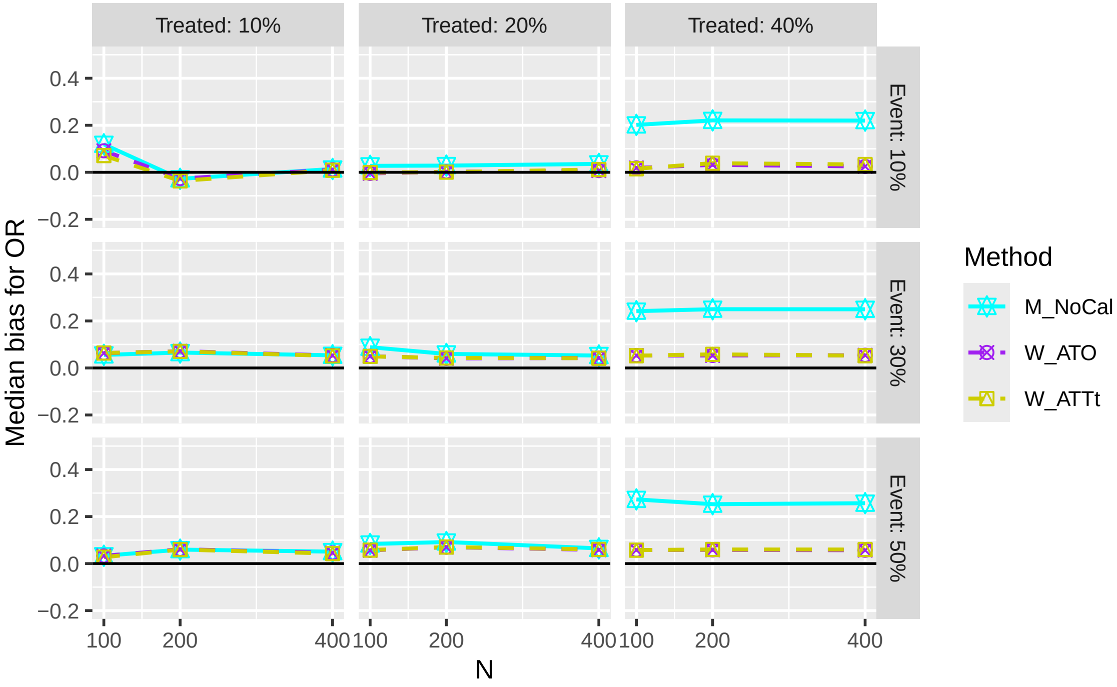

Figure S60. Median bias for OR (categorical covariate, matching ratio 1:2, true OR: 0.5, c statistic: 0.6); other methods.

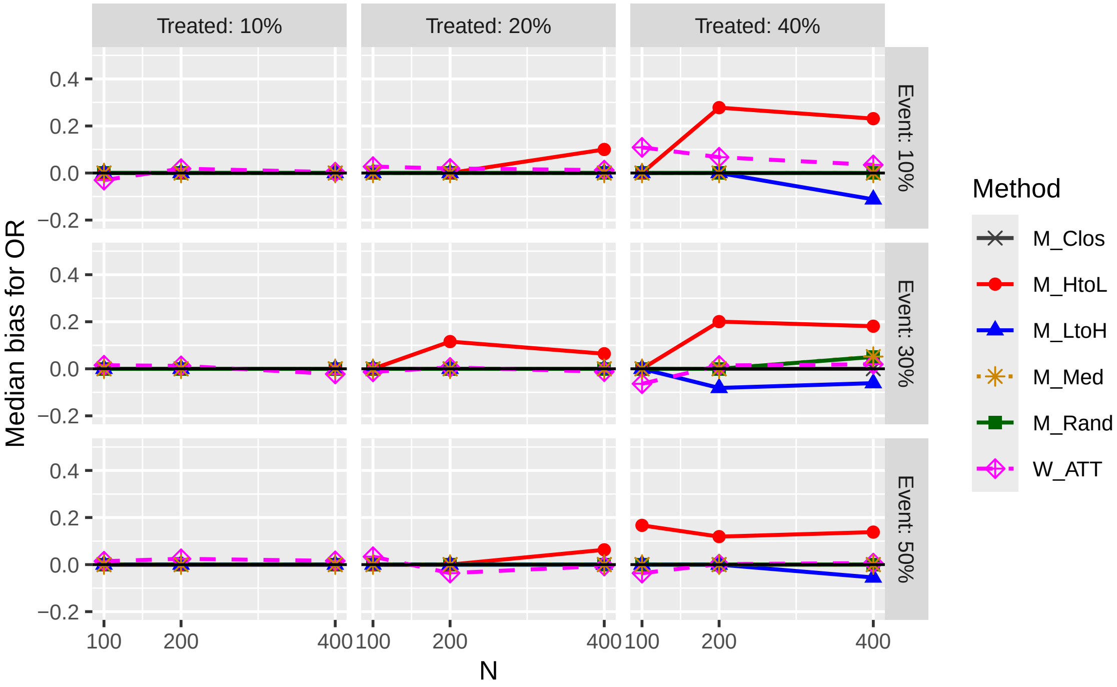

Figure S61. Median bias for OR (multimodal continuous covariate, matching ratio 1:1, true OR: 1, c statistic: 0.85).

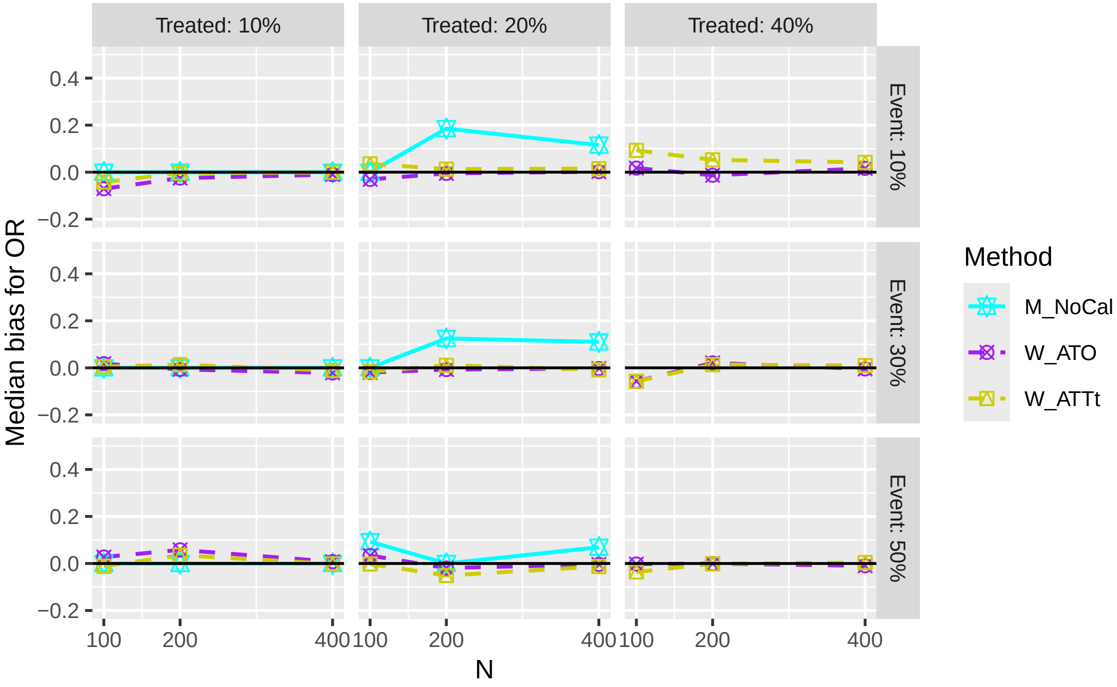

Figure S62. Median bias for OR (multimodal continuous covariate, matching ratio 1:1, true OR: 1, c statistic: 0.85); other methods.

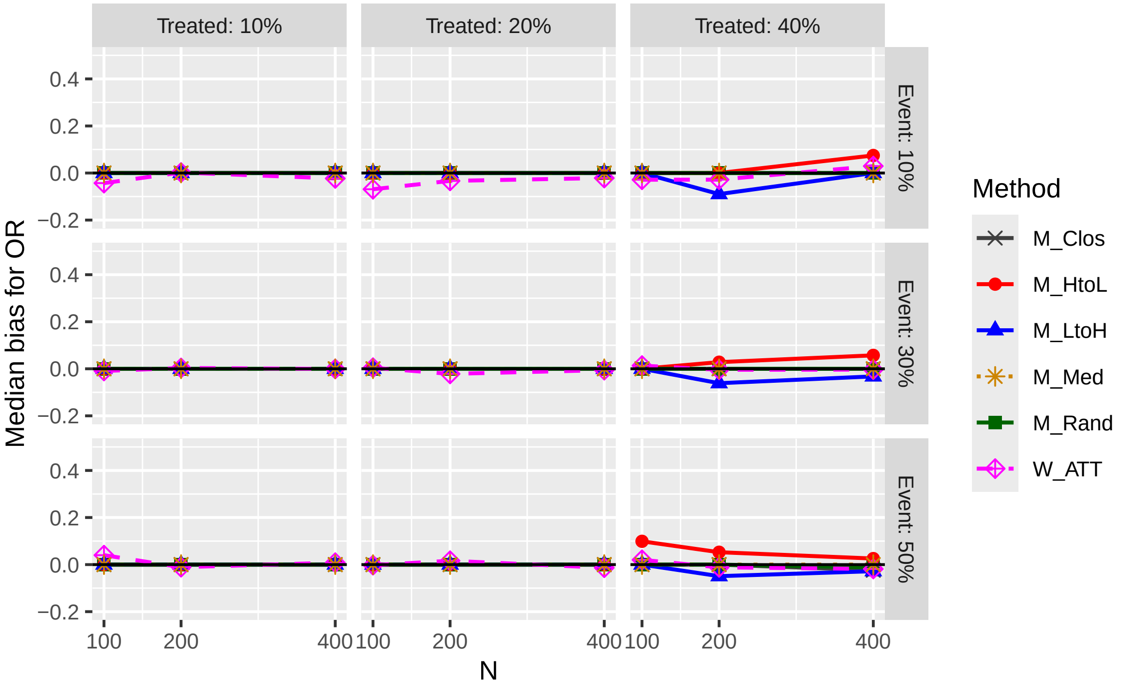

Figure S63. Median bias for OR (multimodal continuous covariate, matching ratio 1:1, true OR: 1, c statistic: 0.6).

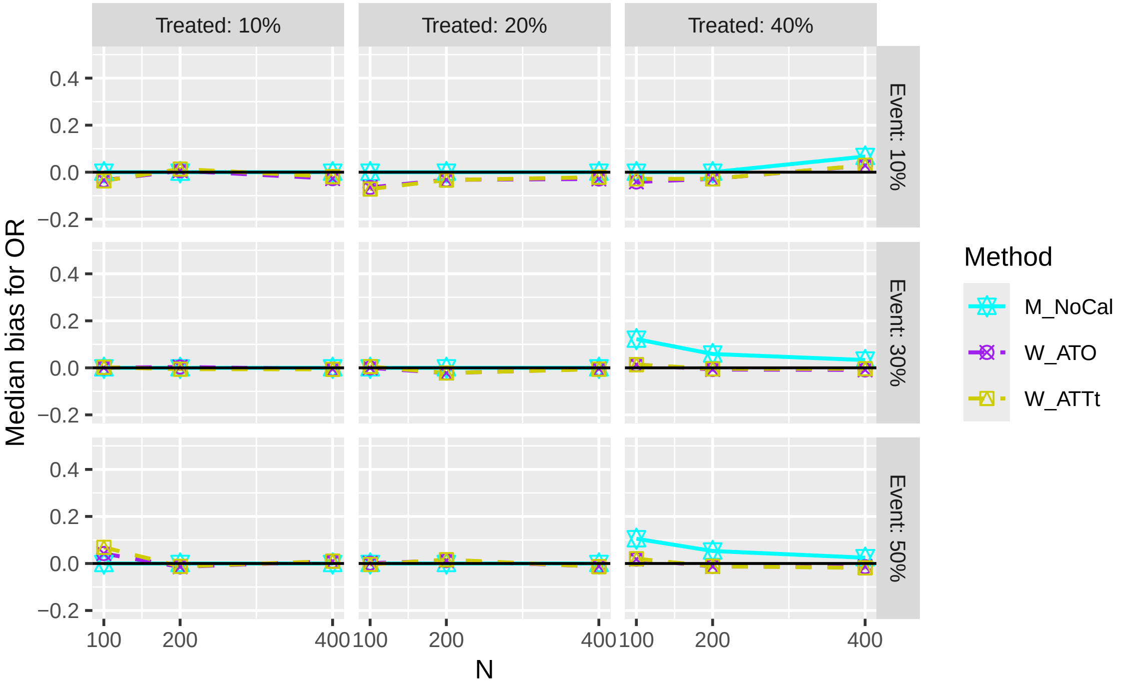

Figure S64. Median bias for OR (multimodal continuous covariate, matching ratio 1:1, true OR: 1, c statistic: 0.6); other methods.

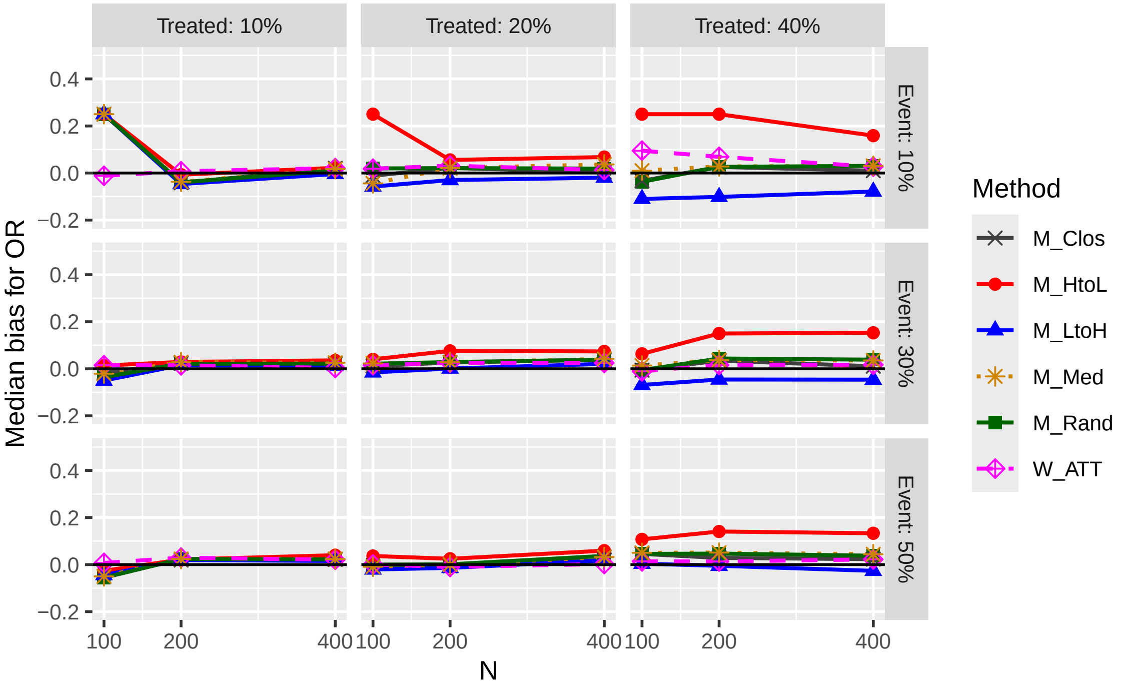

Figure S65. Median bias for OR (multimodal continuous covariate, matching ratio 1:1, true OR: 0.75, c statistic: 0.85).

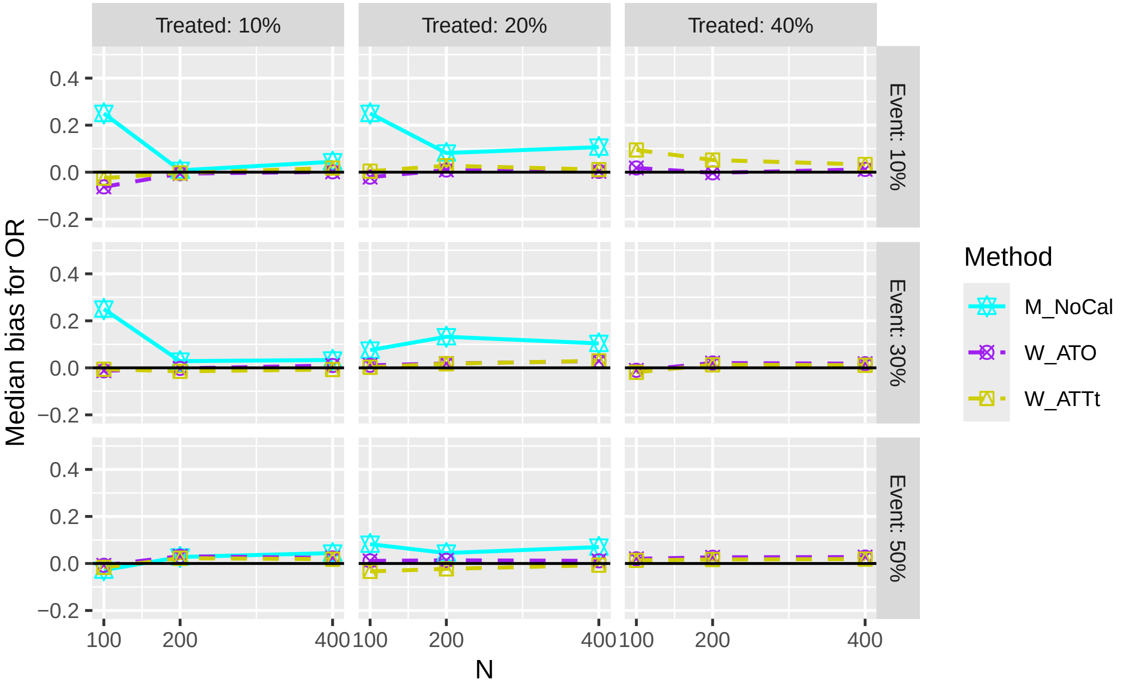

Figure S66. Median bias for OR (multimodal continuous covariate, matching ratio 1:1, true OR: 0.75, c statistic: 0.85); other methods.

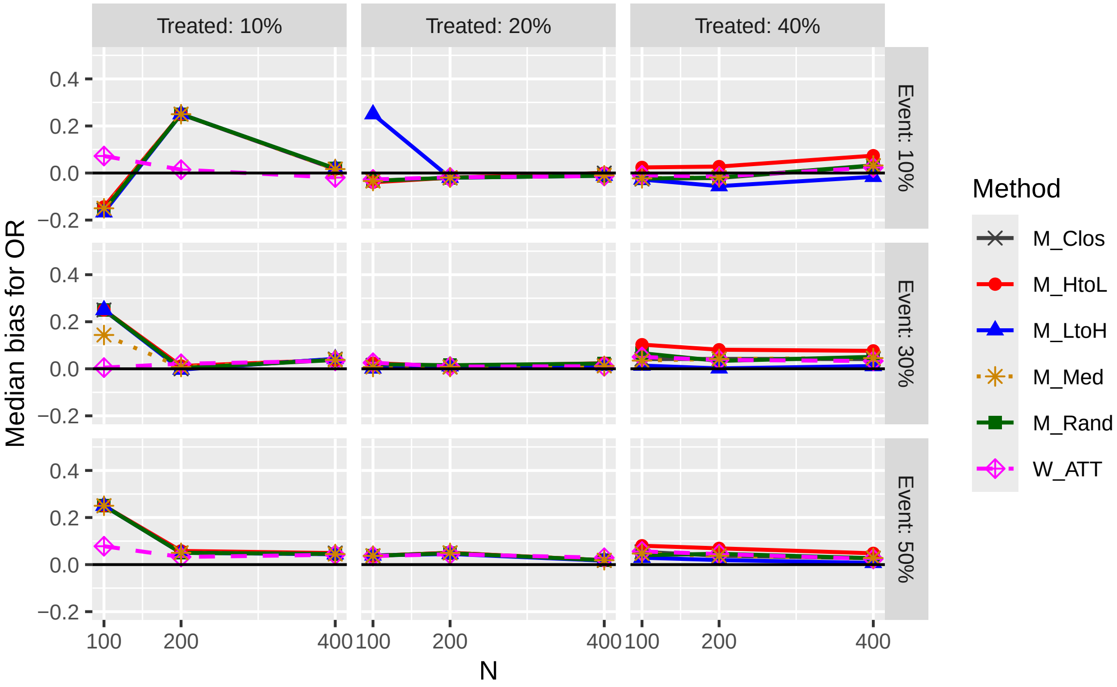

Figure S67. Median bias for OR (multimodal continuous covariate, matching ratio 1:1, true OR: 0.75, c statistic: 0.6).

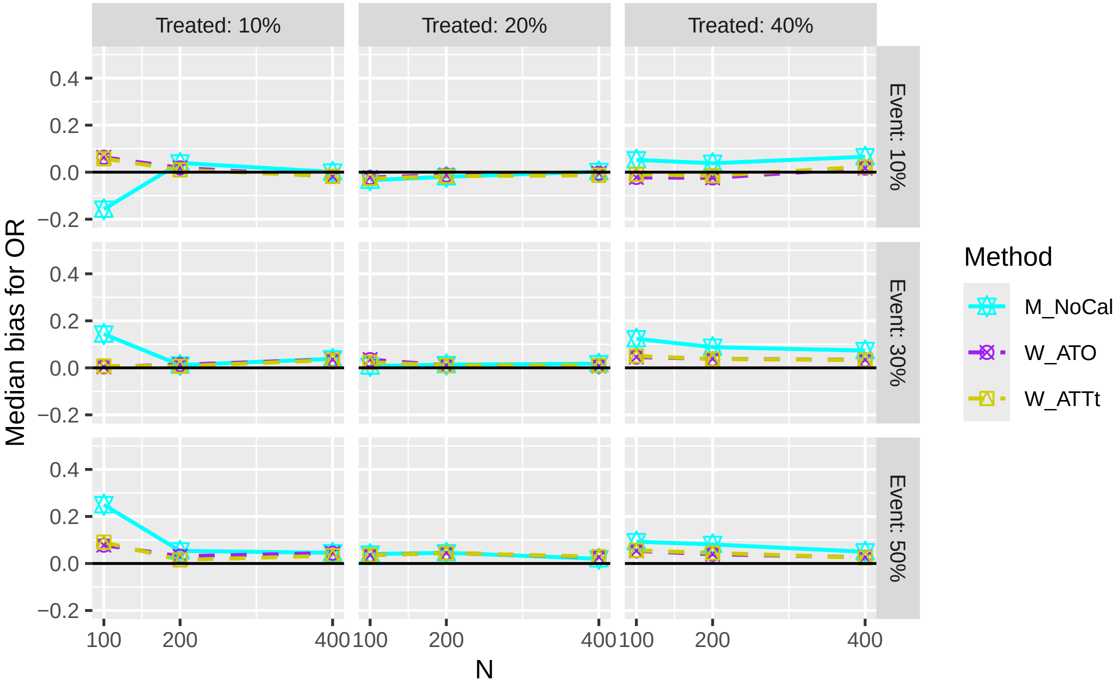

Figure S68. Median bias for OR (multimodal continuous covariate, matching ratio 1:1, true OR: 0.75, c statistic: 0.6); other methods.

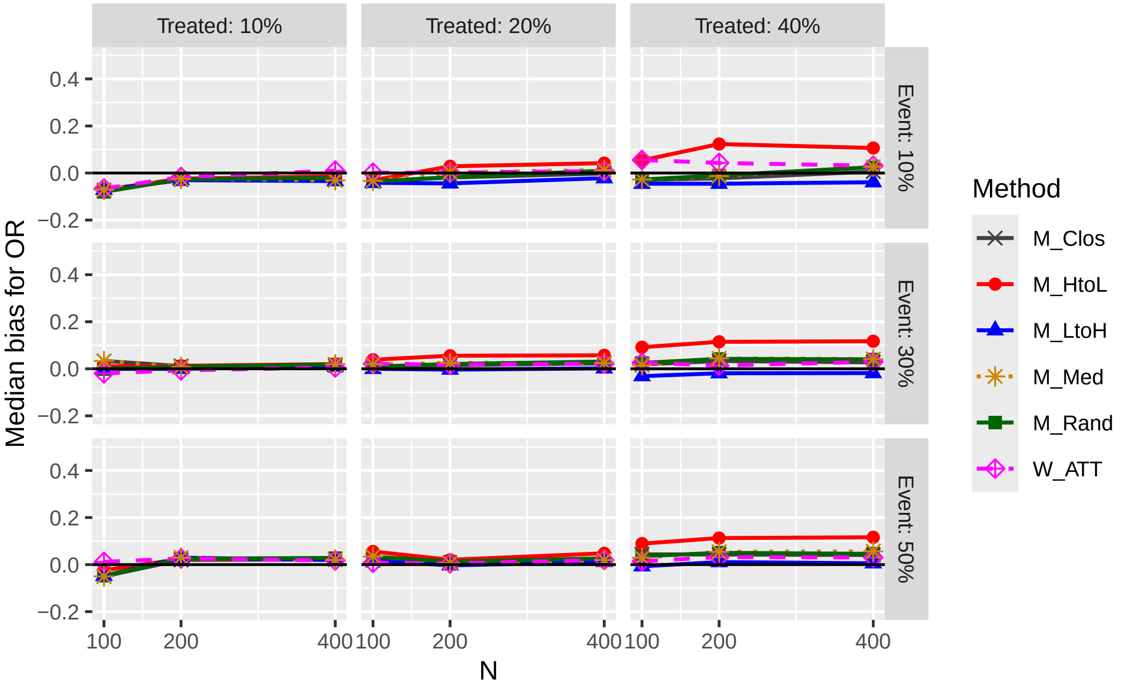

Figure S69. Median bias for OR (multimodal continuous covariate, matching ratio 1:1, true OR: 0.5, c statistic: 0.85).

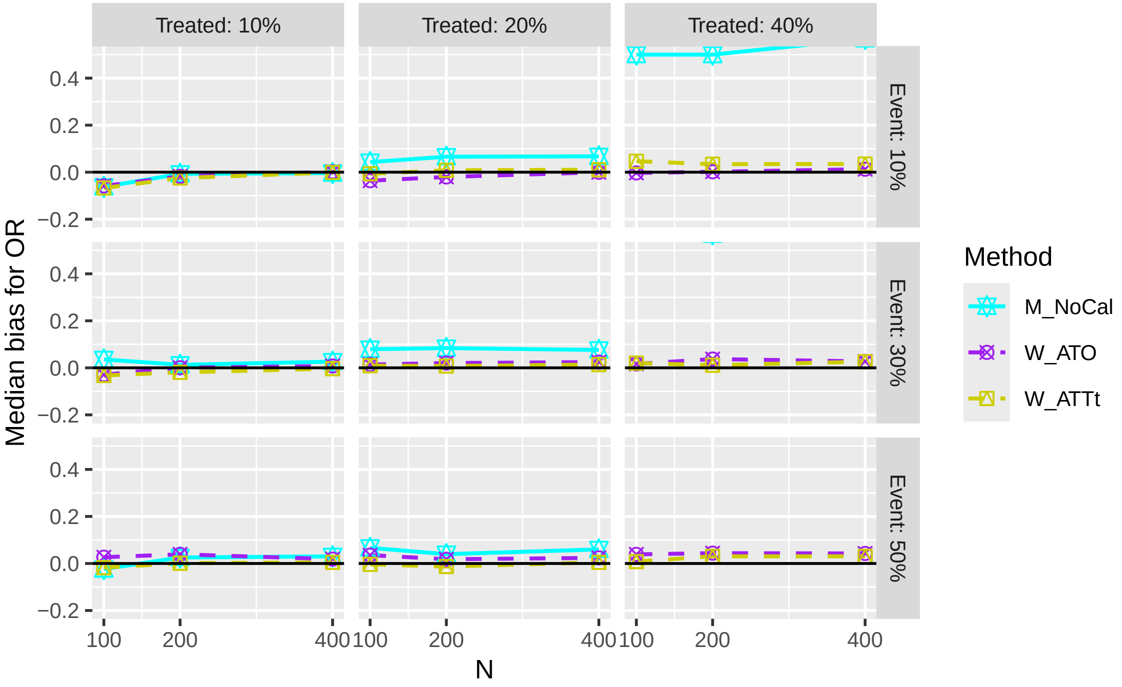

Figure S70. Median bias for OR (multimodal continuous covariate, matching ratio 1:1, true OR: 0.5, c statistic: 0.85); other methods.

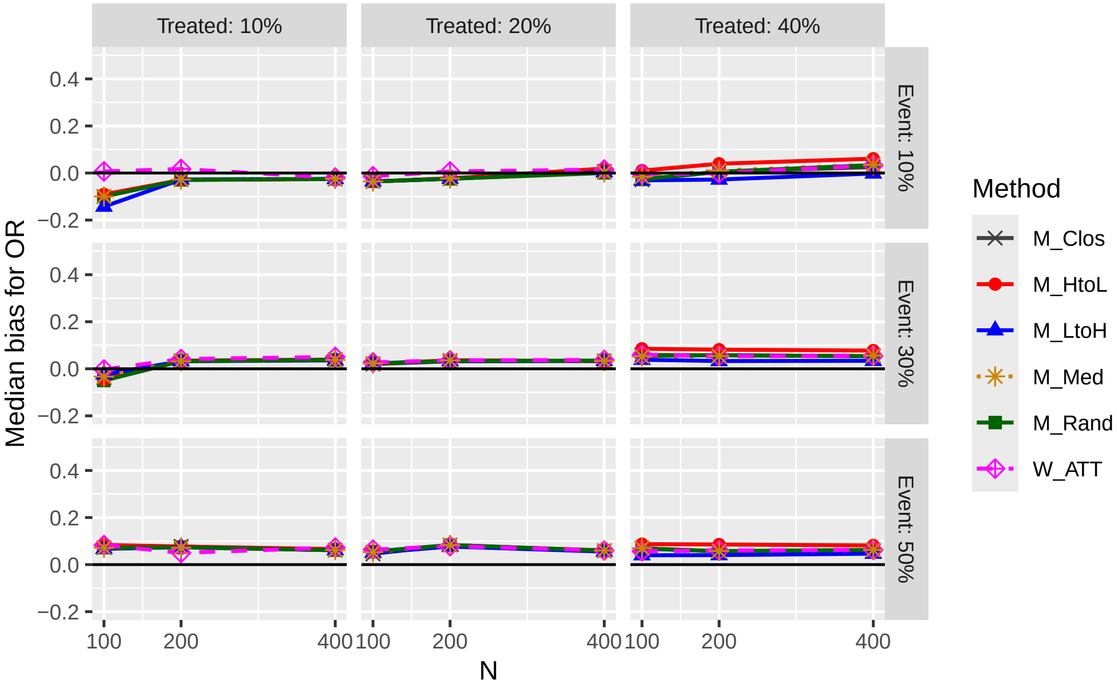

Figure S71. Median bias for OR (multimodal continuous covariate, matching ratio 1:1, true OR: 0.5, c statistic: 0.6).

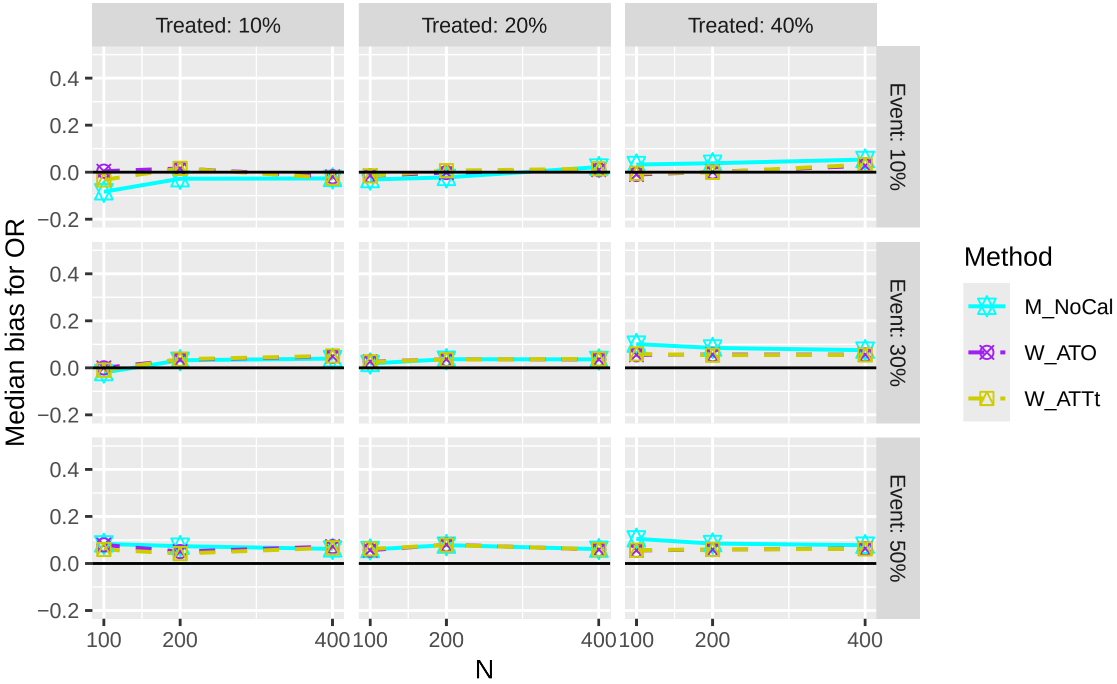

Figure S72. Median bias for OR (multimodal continuous covariate, matching ratio 1:1, true OR: 0.5, c statistic: 0.6); other methods.

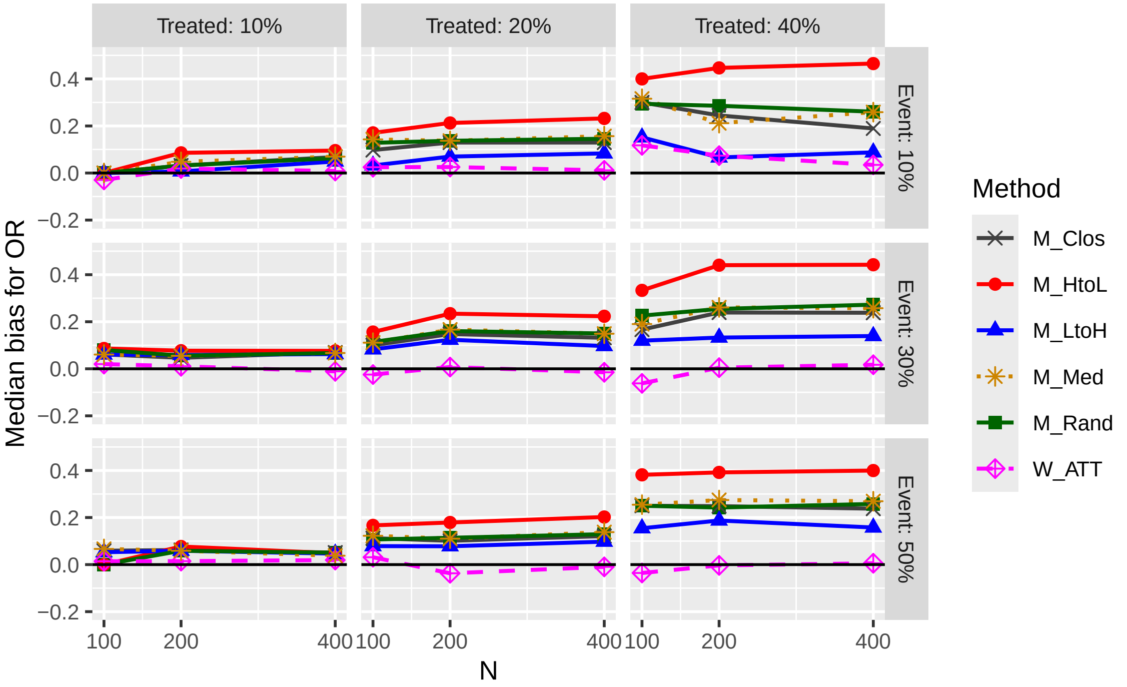

Figure S73. Median bias for OR (multimodal continuous covariate, matching ratio 1:2, true OR: 1, c statistic: 0.85).

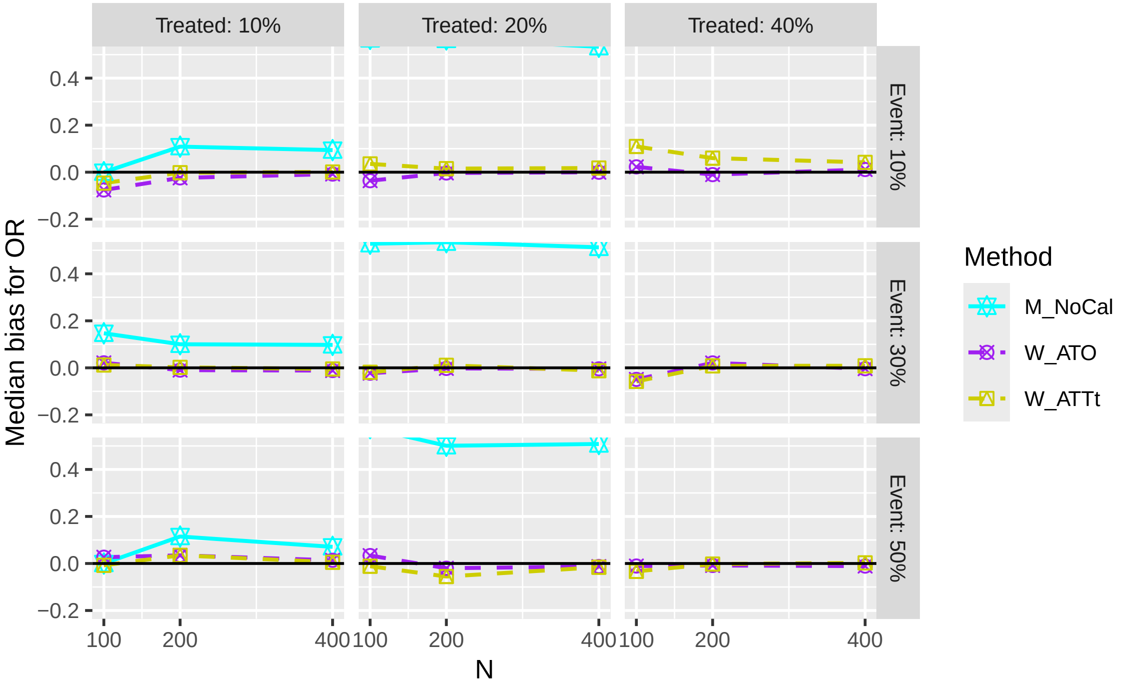

Figure S74. Median bias for OR (multimodal continuous covariate, matching ratio 1:2, true OR: 1, c statistic: 0.85); other methods.

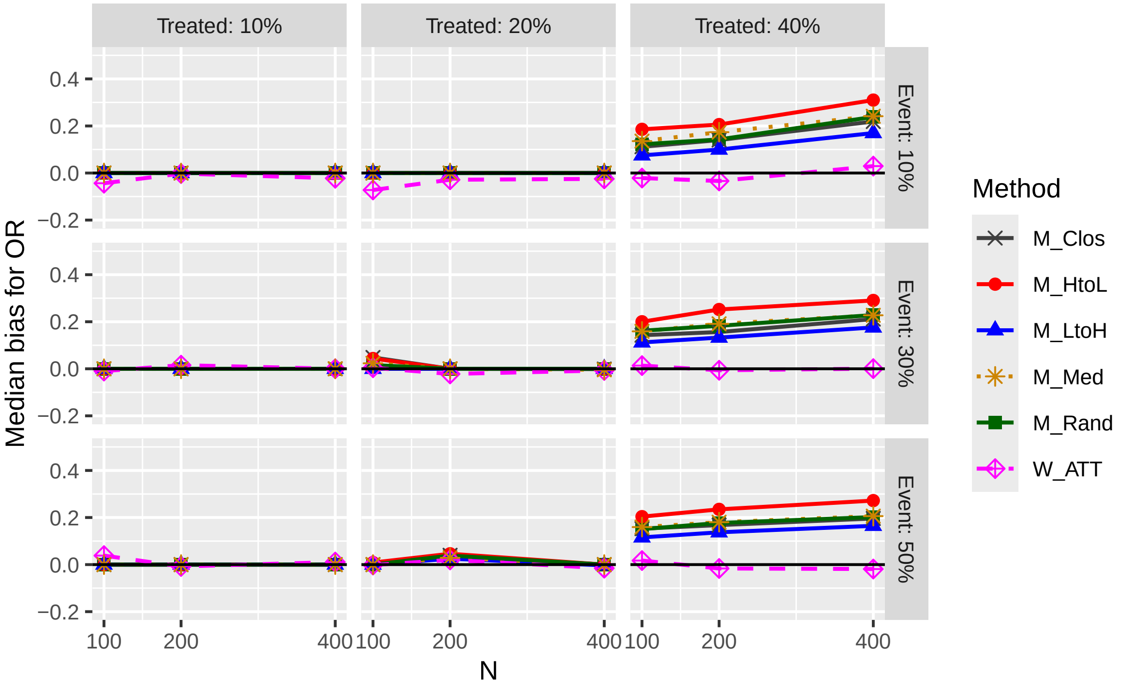

Figure S75. Median bias for OR (multimodal continuous covariate, matching ratio 1:2, true OR: 1, c statistic: 0.6).

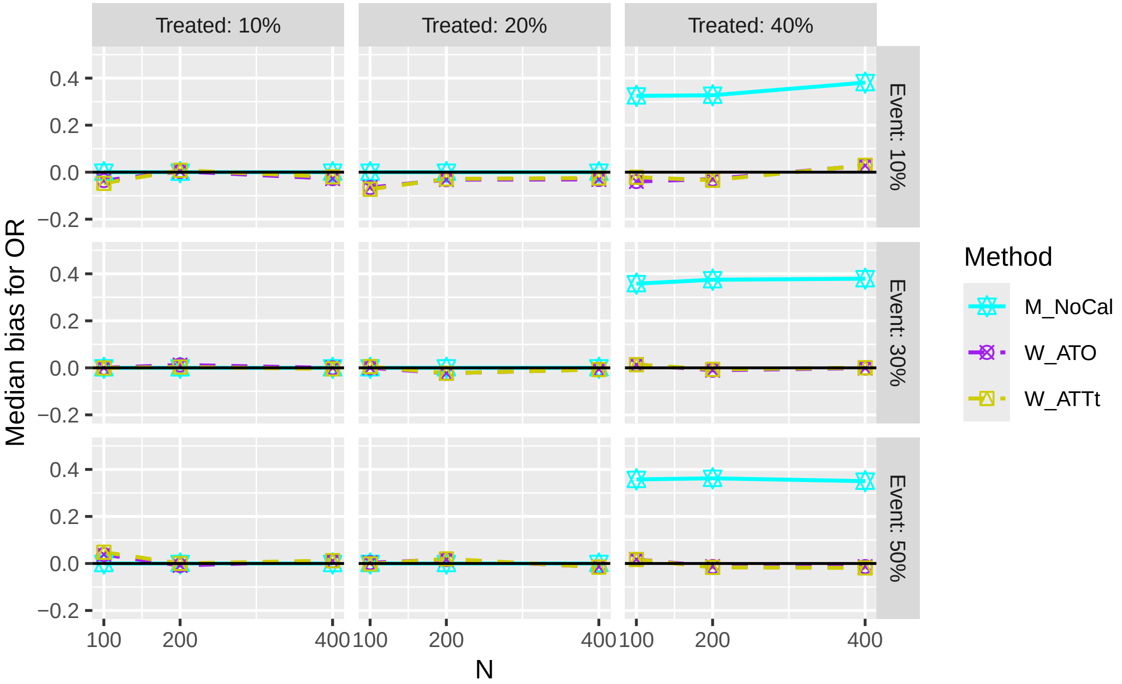

Figure S76. Median bias for OR (multimodal continuous covariate, matching ratio 1:2, true OR: 1, c statistic: 0.6); other methods.

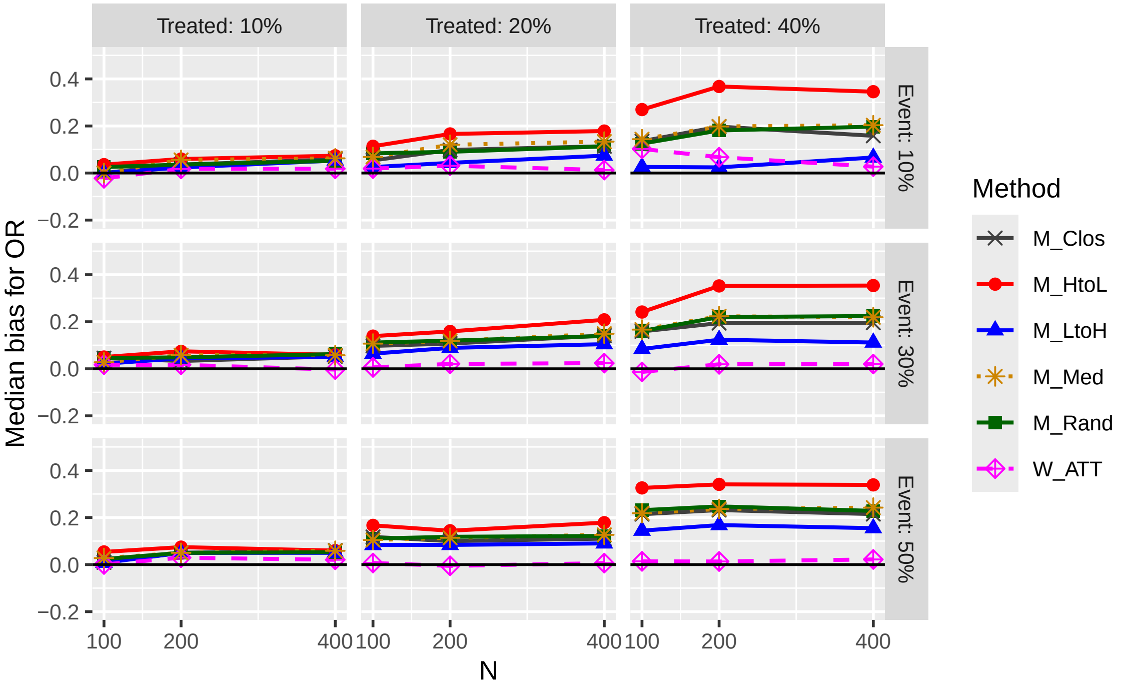

Figure S77. Median bias for OR (multimodal continuous covariate, matching ratio 1:2, true OR: 0.75, c statistic: 0.85).

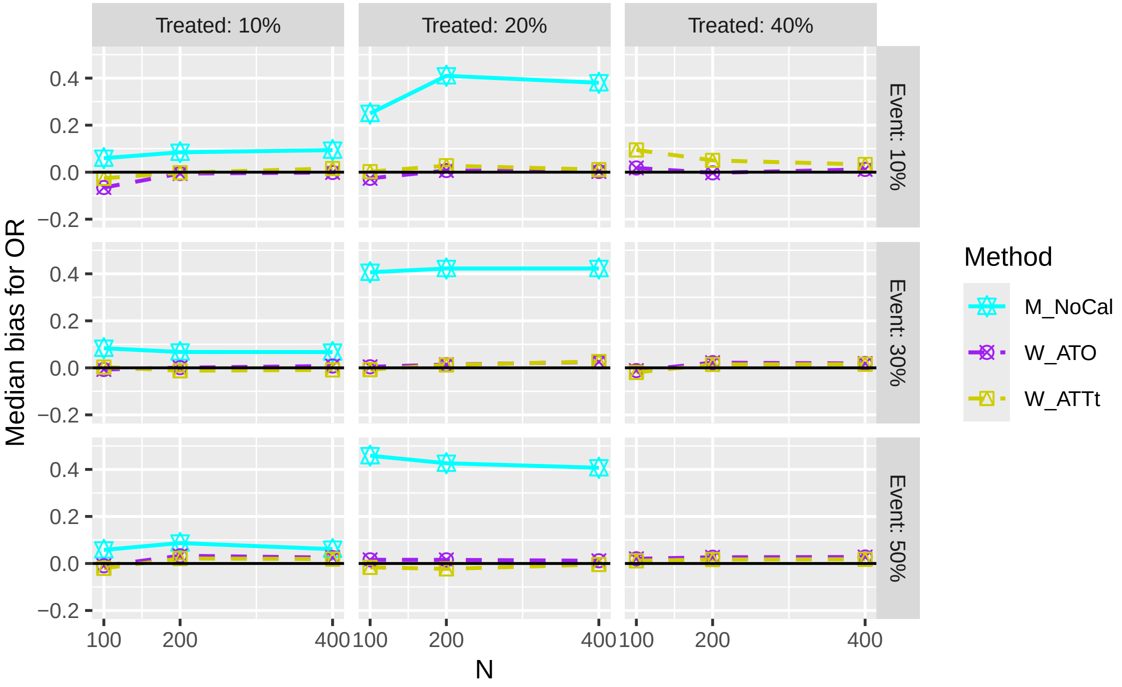

Figure S78. Median bias for OR (multimodal continuous covariate, matching ratio 1:2, true OR: 0.75, c statistic: 0.85); other methods.

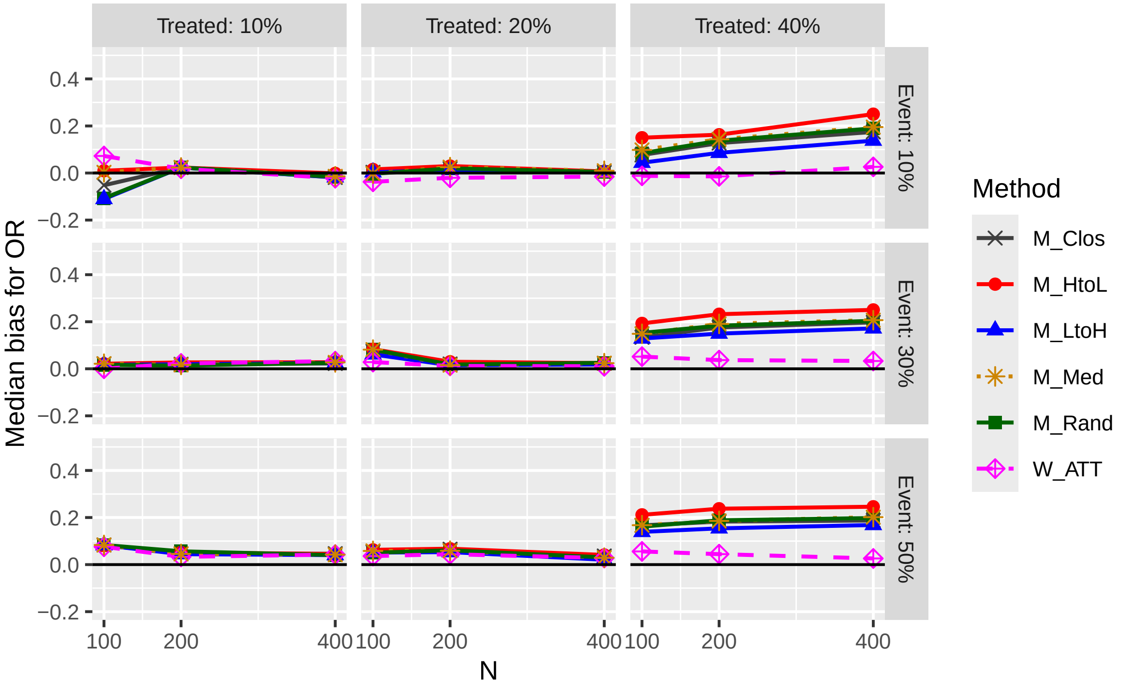

Figure S79. Median bias for OR (multimodal continuous covariate, matching ratio 1:2, true OR: 0.75, c statistic: 0.6).

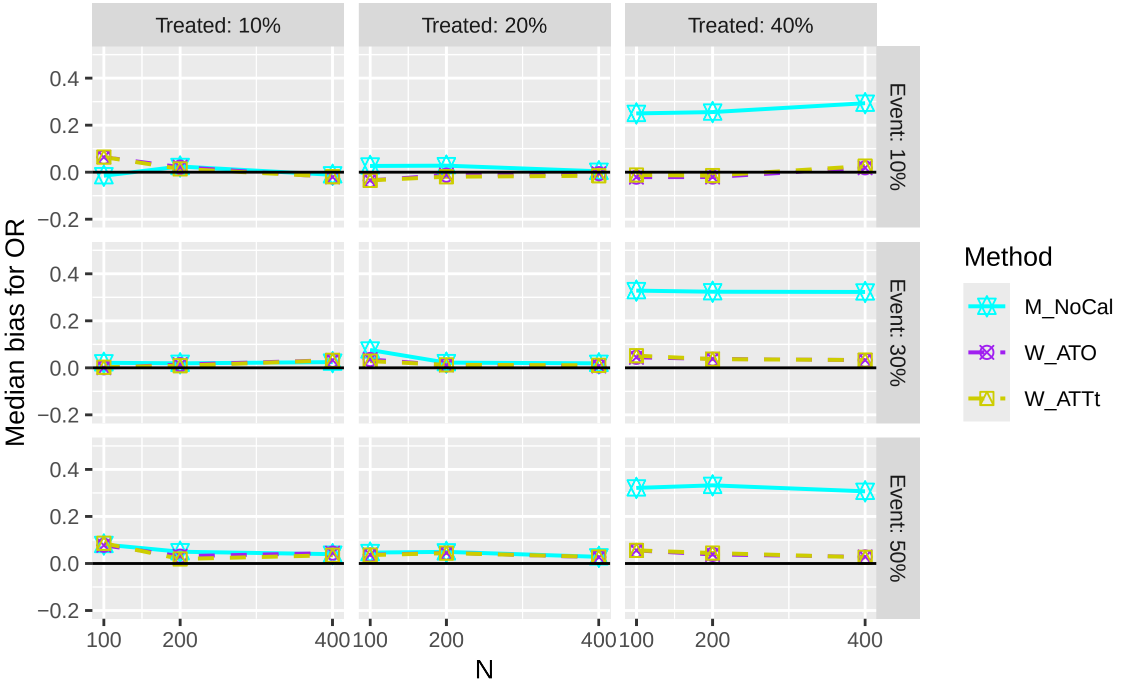

Figure S80. Median bias for OR (multimodal continuous covariate, matching ratio 1:2, true OR: 0.75, c statistic: 0.6); other methods.

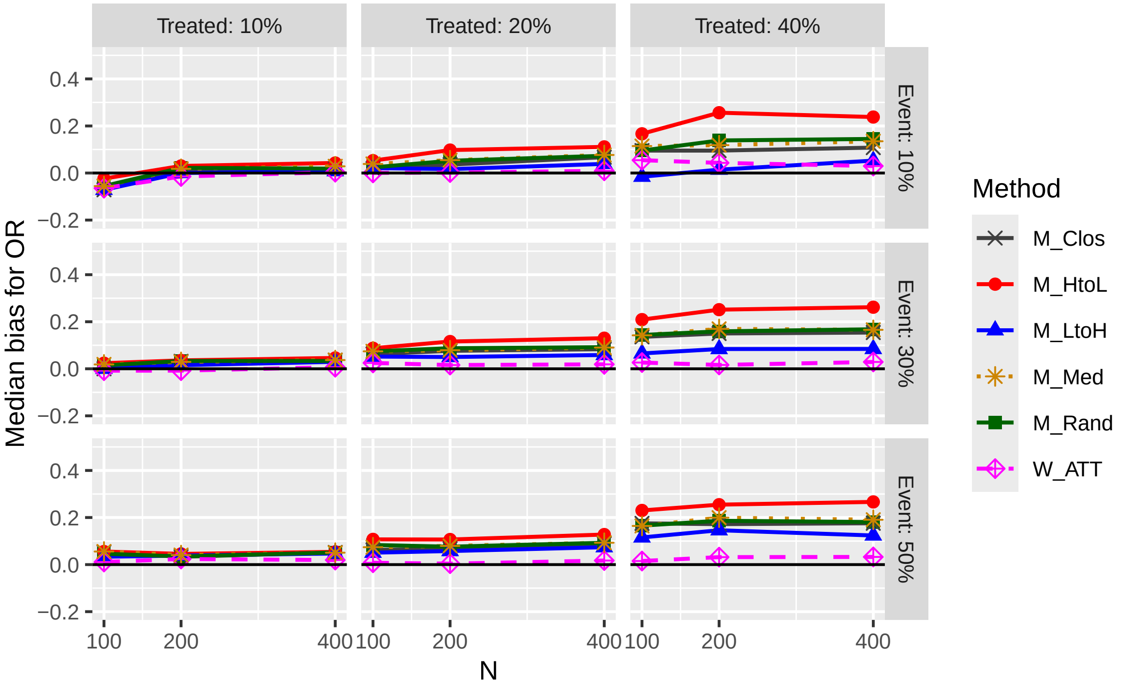

Figure S81. Median bias for OR (multimodal continuous covariate, matching ratio 1:2, true OR: 0.5, c statistic: 0.85).

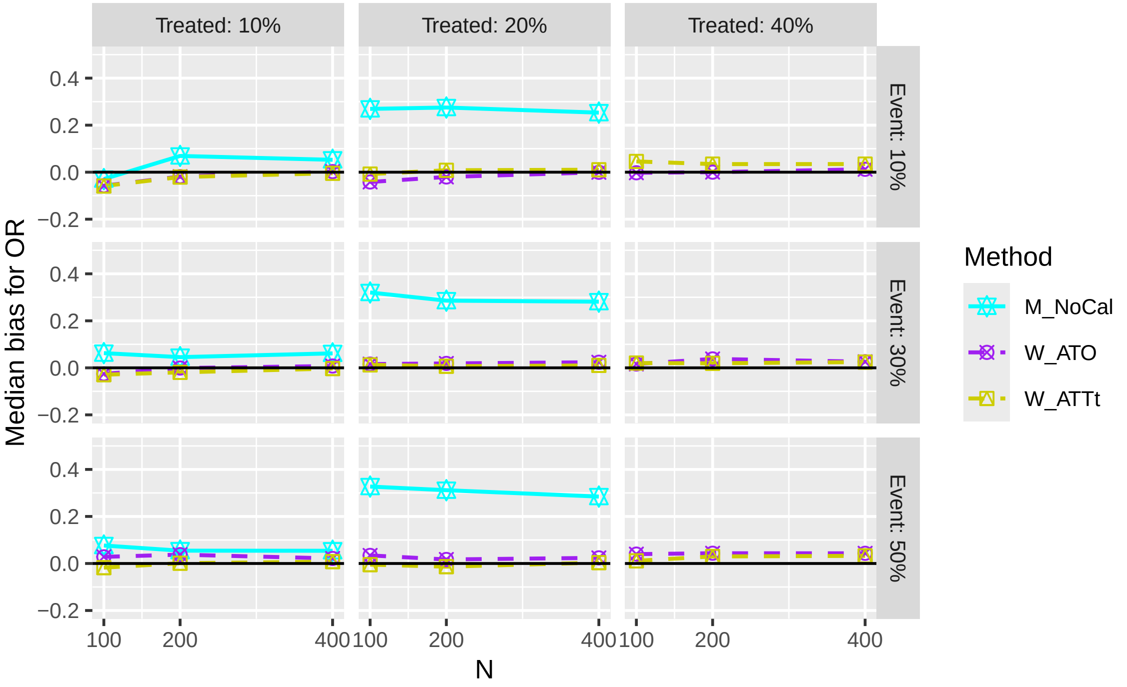

Figure S82. Median bias for OR (multimodal continuous covariate, matching ratio 1:2, true OR: 0.5, c statistic: 0.85); other methods.

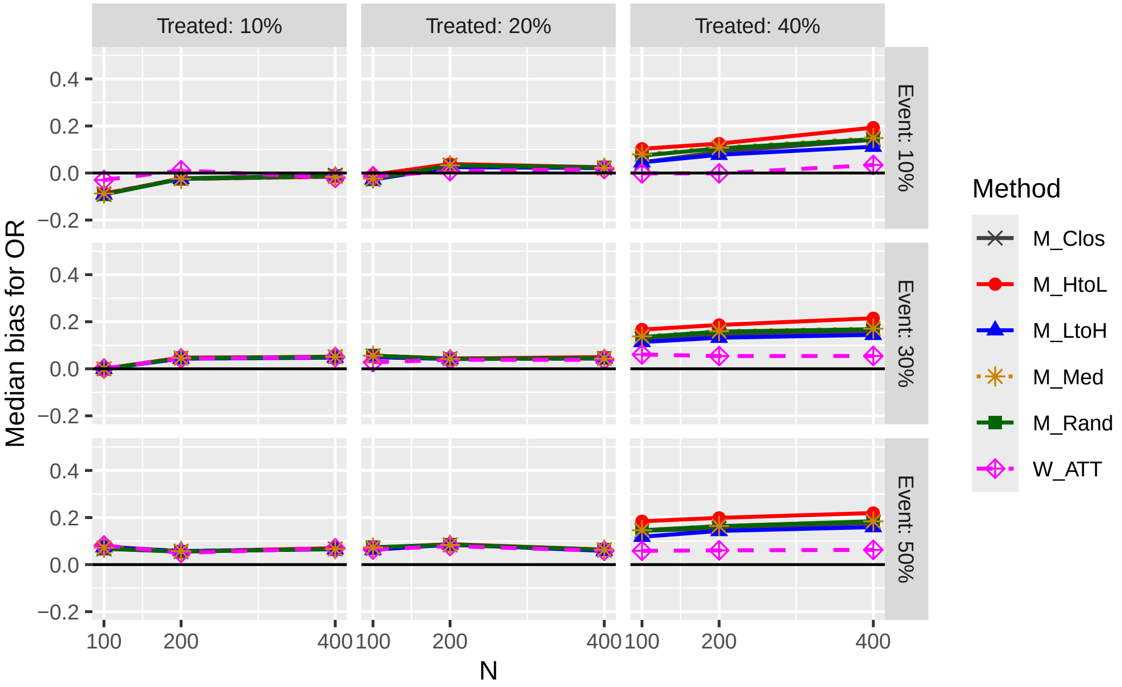

Figure S83. Median bias for OR (multimodal continuous covariate, matching ratio 1:2, true OR: 0.5, c statistic: 0.6).

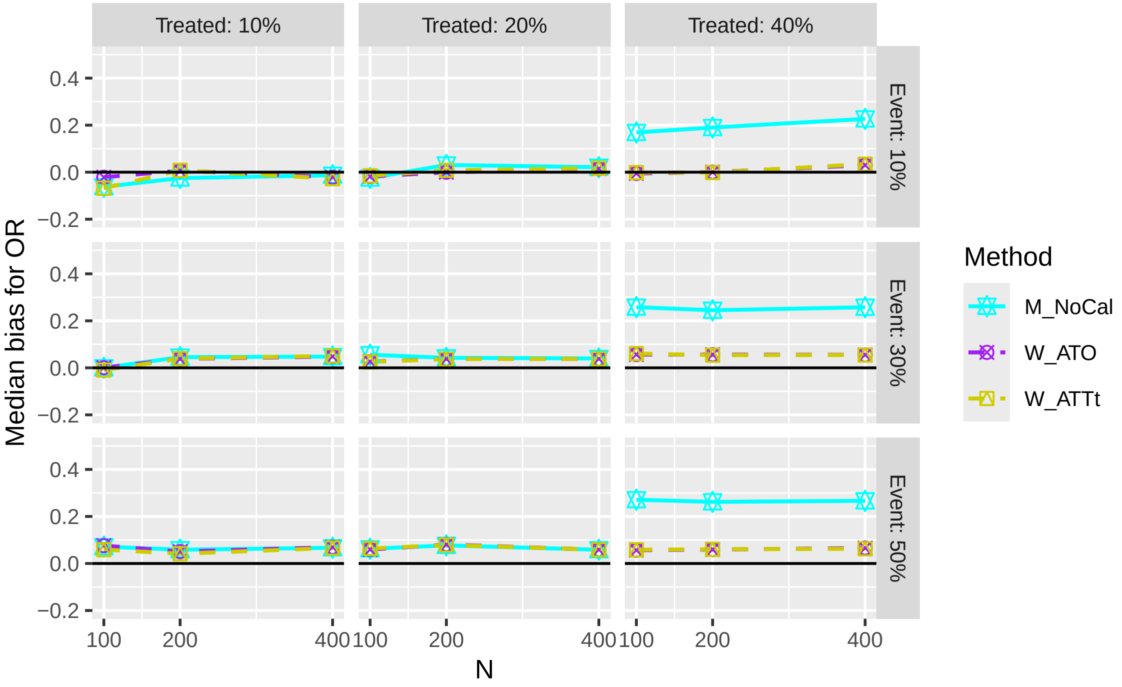

Figure S84. Median bias for OR (multimodal continuous covariate, matching ratio 1:2, true OR: 0.5, c statistic: 0.6); other methods.

S3. IQR for OR (precision, caliper: 25%)

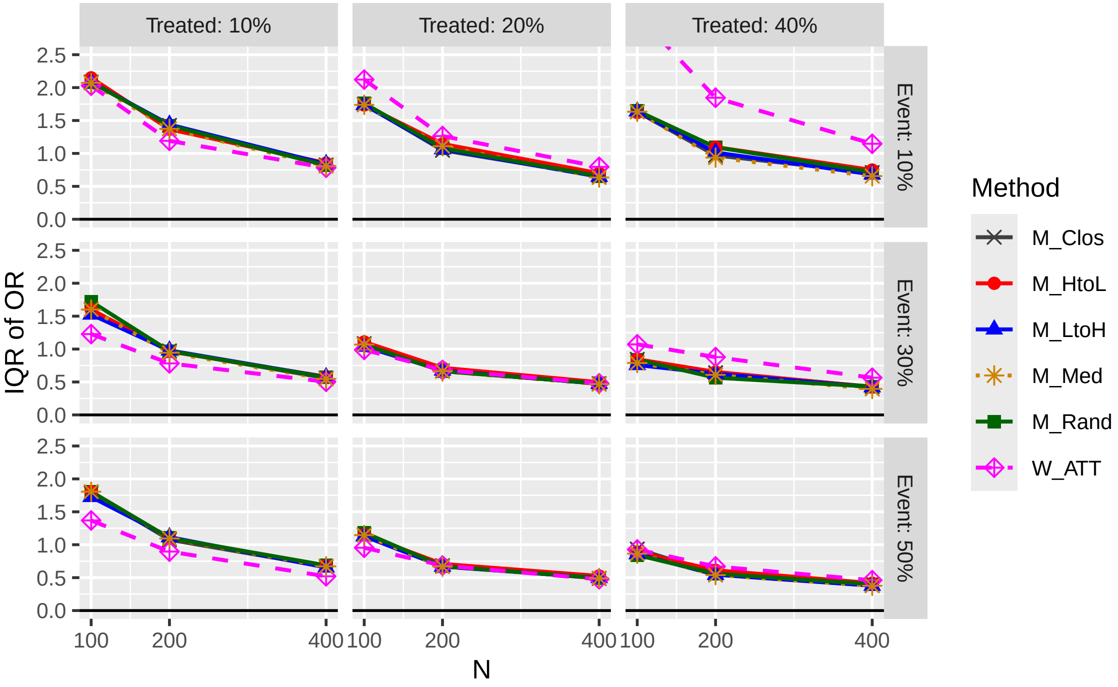

Figure S85. IQR for OR (unimodal continuous covariate, matching ratio 1:1, true OR: 1, c statistic: 0.85).

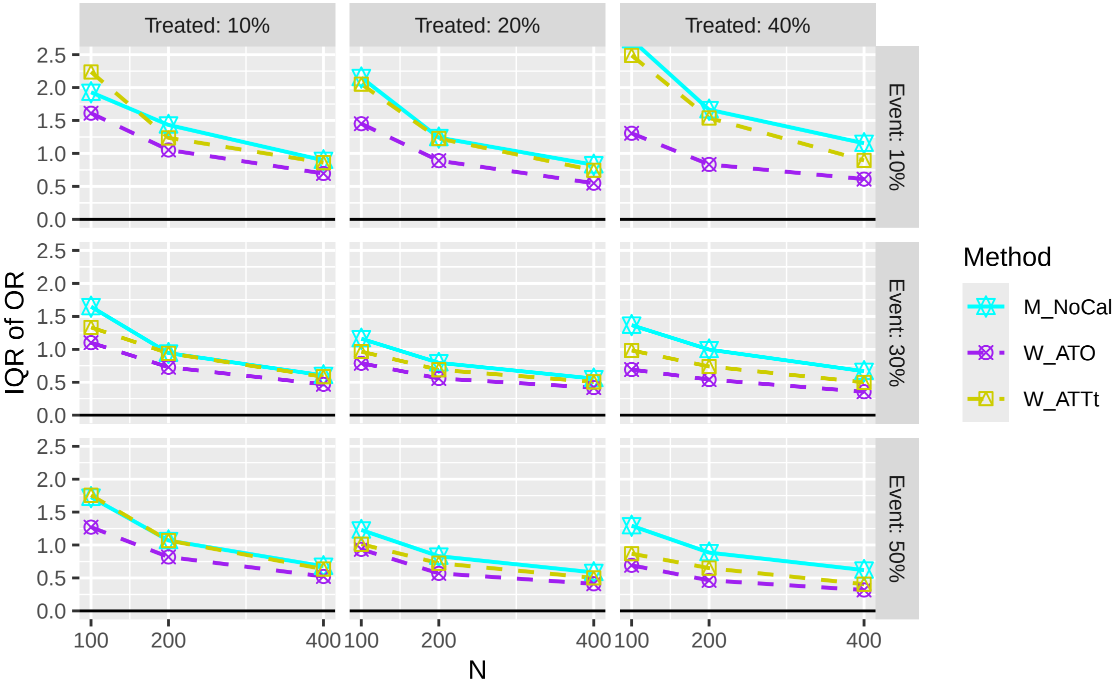

Figure S86. IQR for OR (unimodal continuous covariate, matching ratio 1:1, true OR: 1, c statistic: 0.85); other methods.

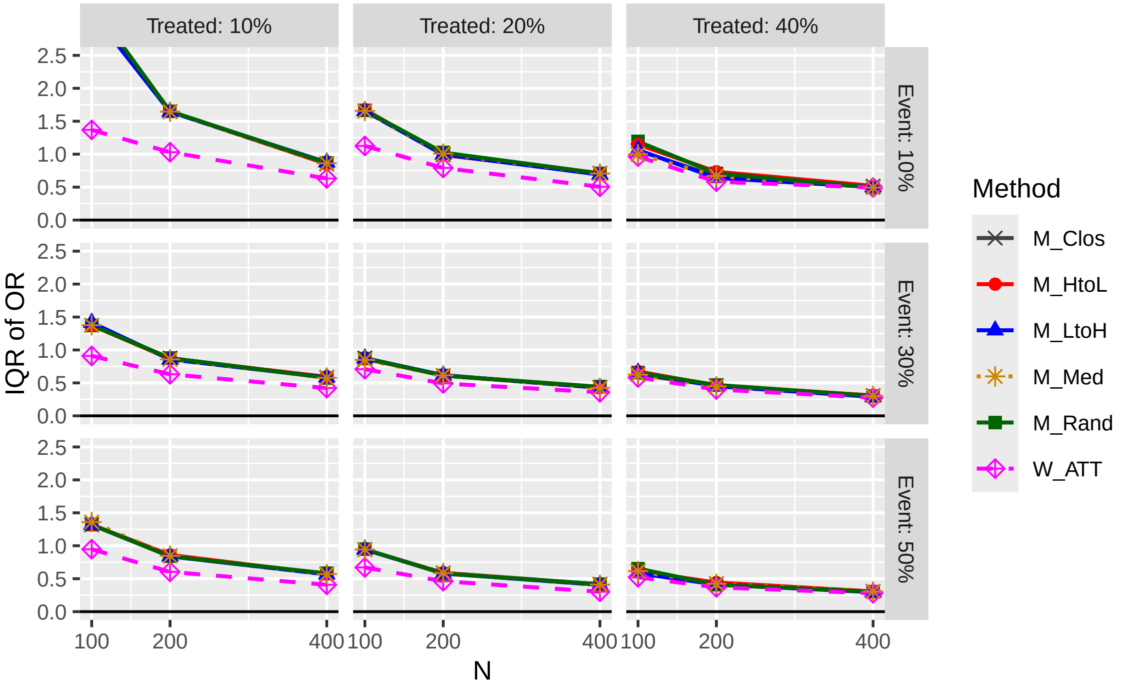

Figure S87. IQR for OR (unimodal continuous covariate, matching ratio 1:1, true OR: 1, c statistic: 0.6).

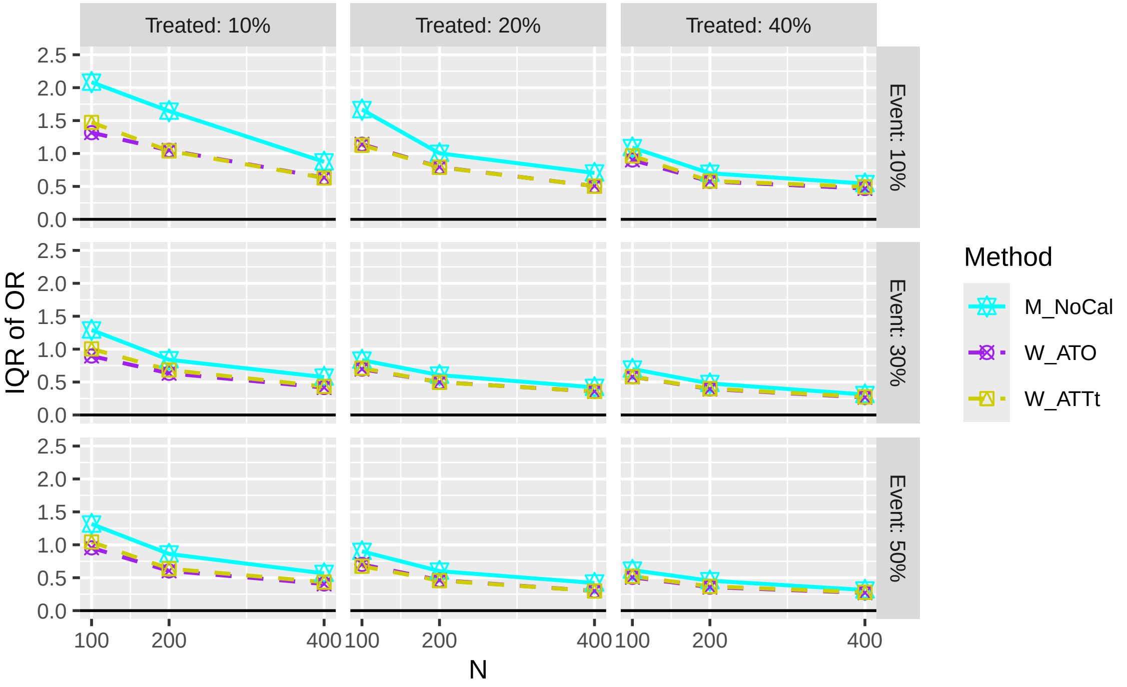

Figure S88. IQR for OR (unimodal continuous covariate, matching ratio 1:1, true OR: 1, c statistic: 0.6); other methods.

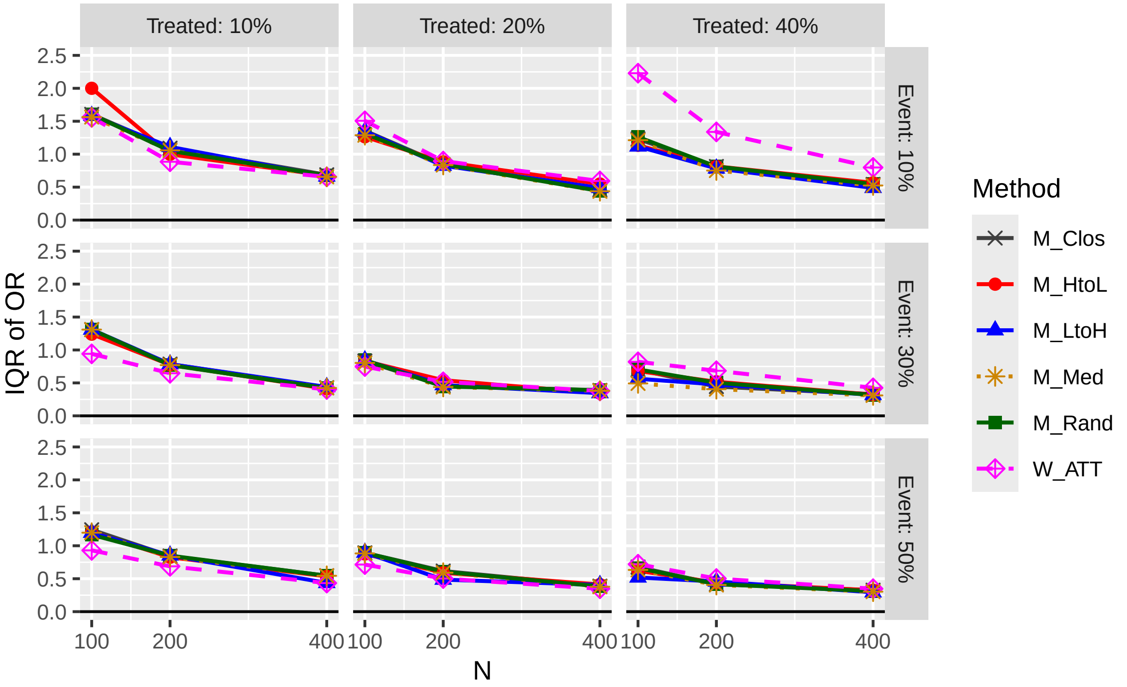

Figure S89. IQR for OR (unimodal continuous covariate, matching ratio 1:1, true OR: 0.75, c statistic: 0.85).

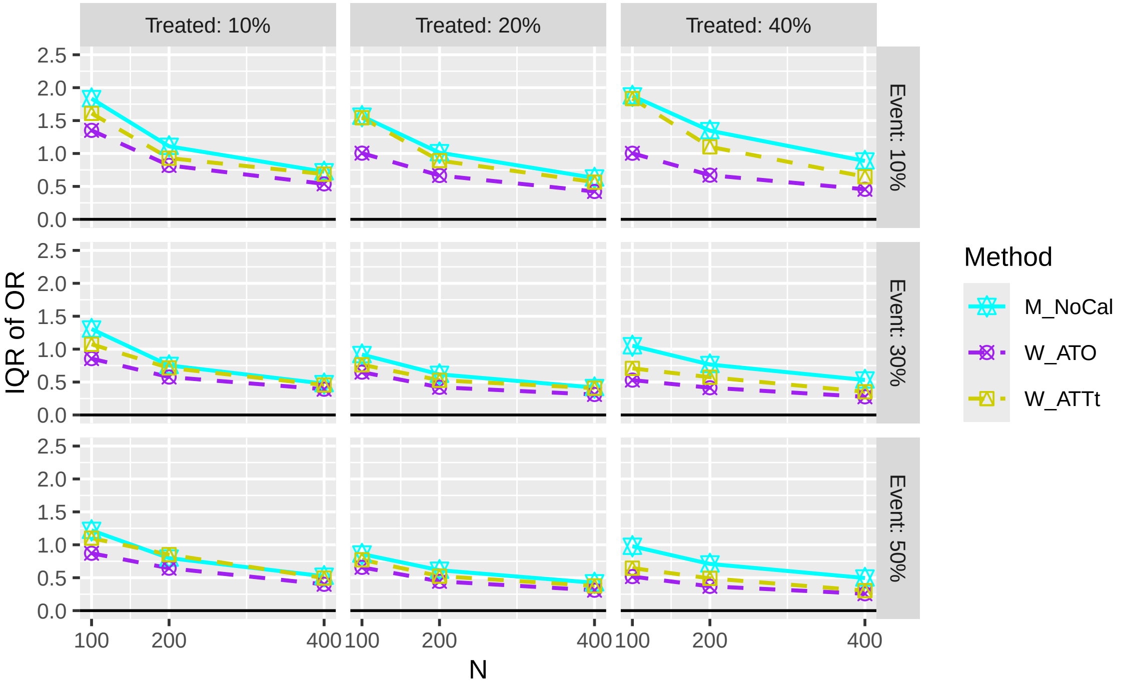

Figure S90. IQR for OR (unimodal continuous covariate, matching ratio 1:1, true OR: 0.75, c statistic: 0.85); other methods.

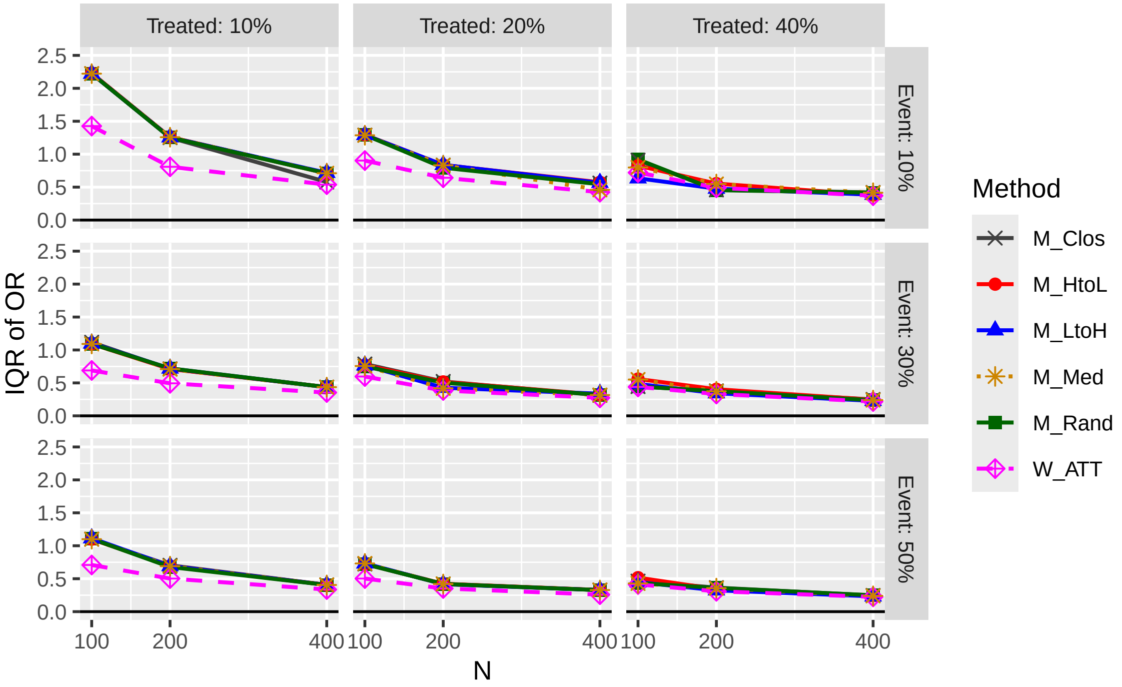

Figure S91. IQR for OR (unimodal continuous covariate, matching ratio 1:1, true OR: 0.75, c statistic: 0.6).

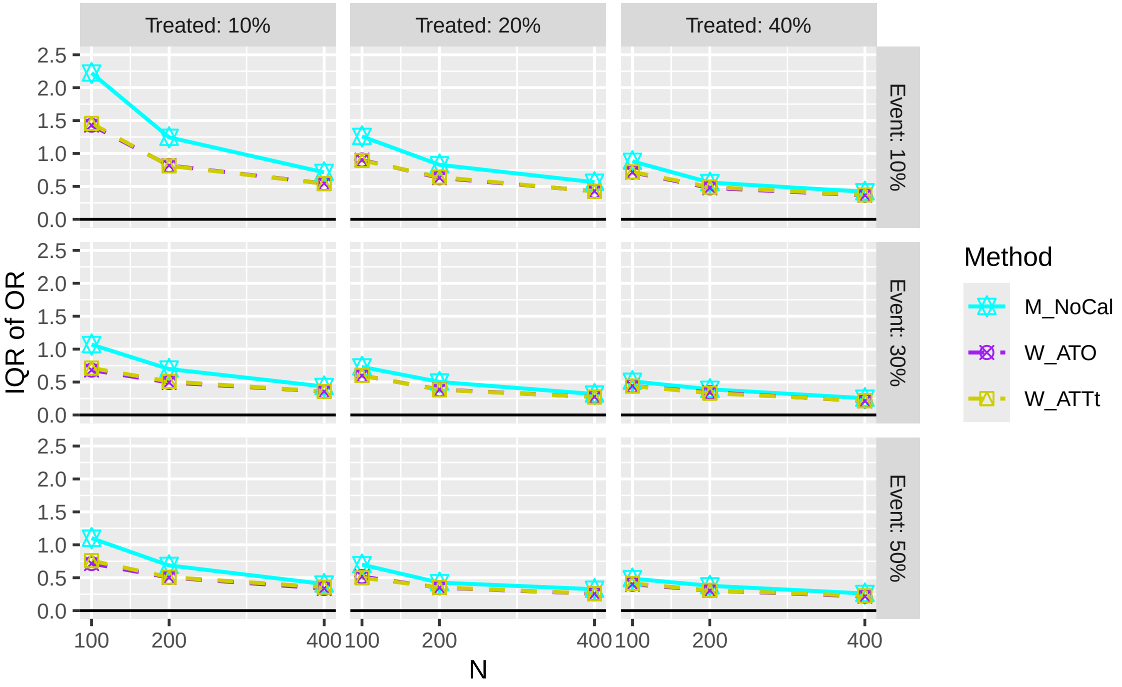

Figure S92. IQR for OR (unimodal continuous covariate, matching ratio 1:1, true OR: 0.75, c statistic: 0.6); other methods.

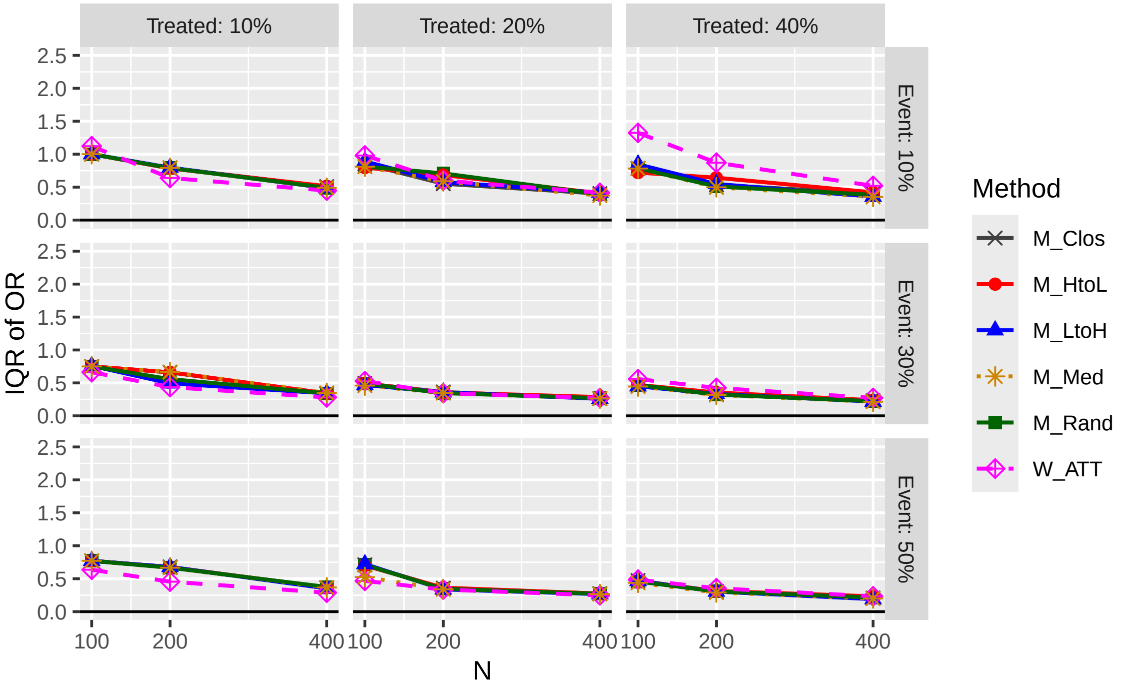

Figure S93. IQR for OR (unimodal continuous covariate, matching ratio 1:1, true OR: 0.5, c statistic: 0.85).

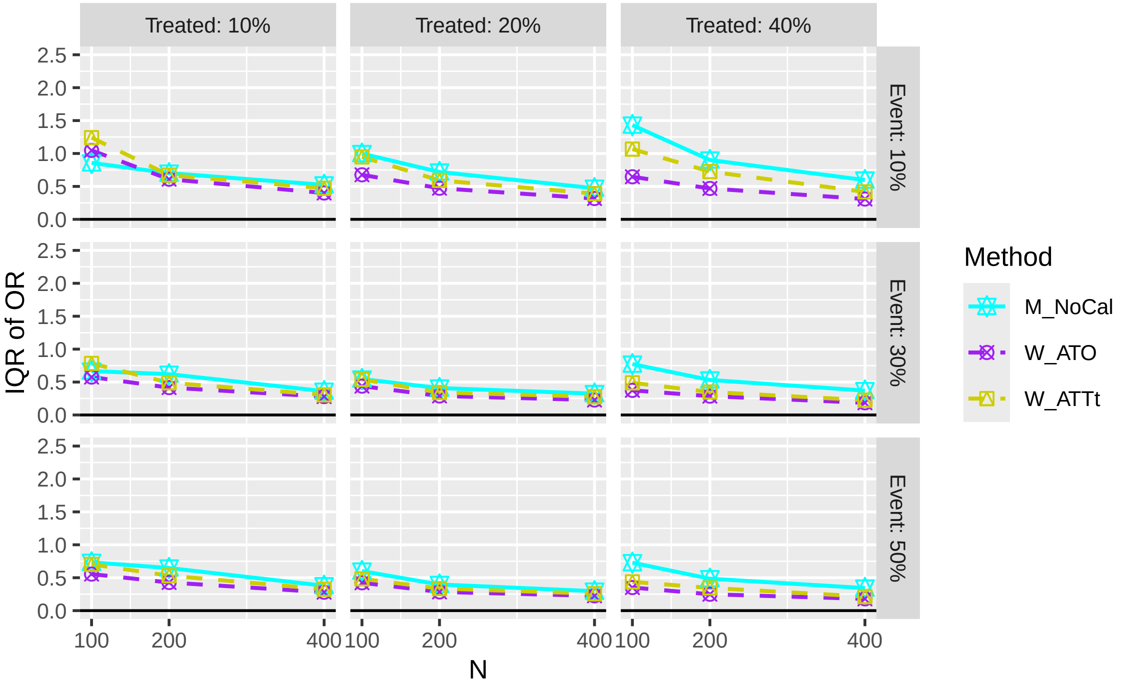

Figure S94. IQR for OR (unimodal continuous covariate, matching ratio 1:1, true OR: 0.5, c statistic: 0.85); other methods.

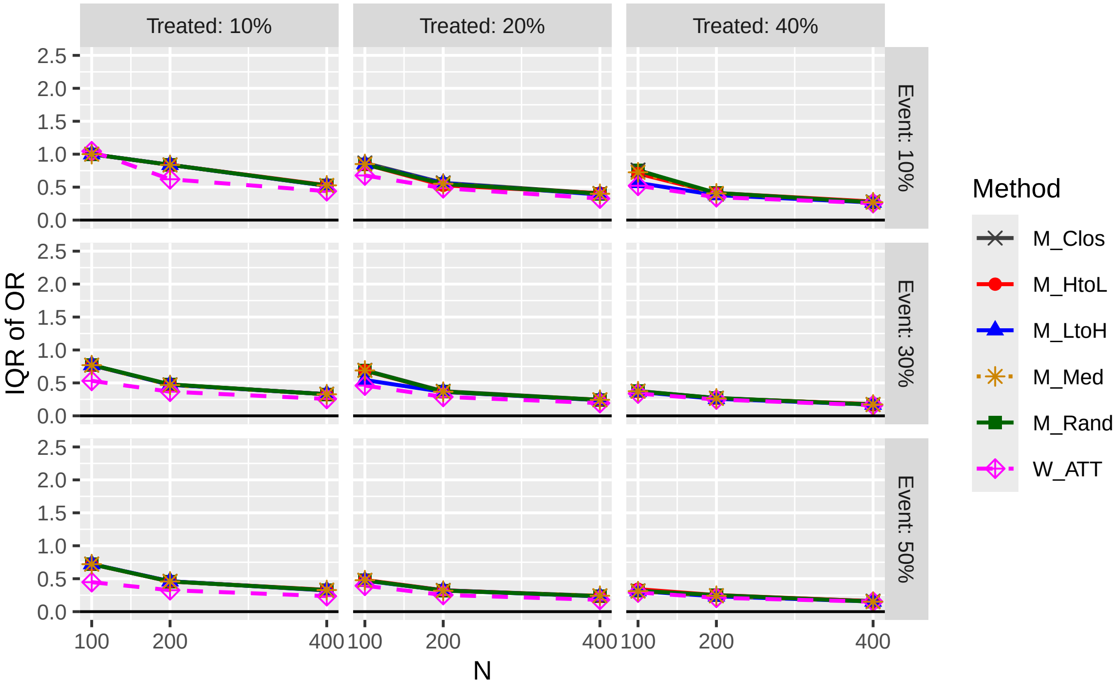

Figure S95. IQR for OR (unimodal continuous covariate, matching ratio 1:1, true OR: 0.5, c statistic: 0.6).

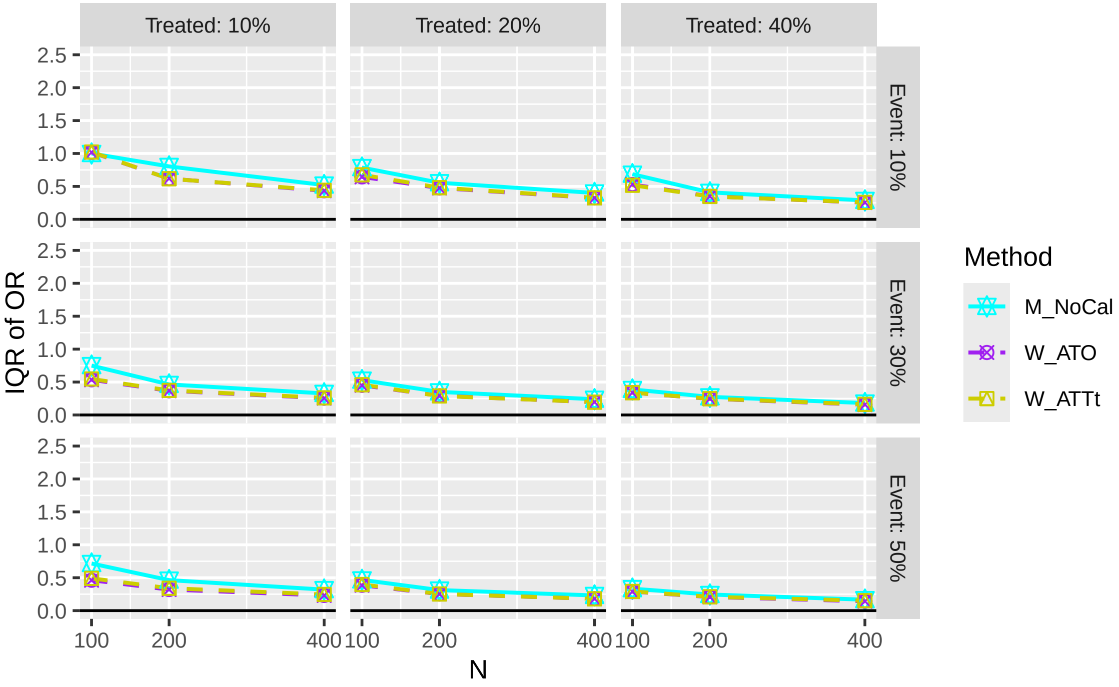

Figure S96. IQR for OR (unimodal continuous covariate, matching ratio 1:1, true OR: 0.5, c statistic: 0.6); other methods.

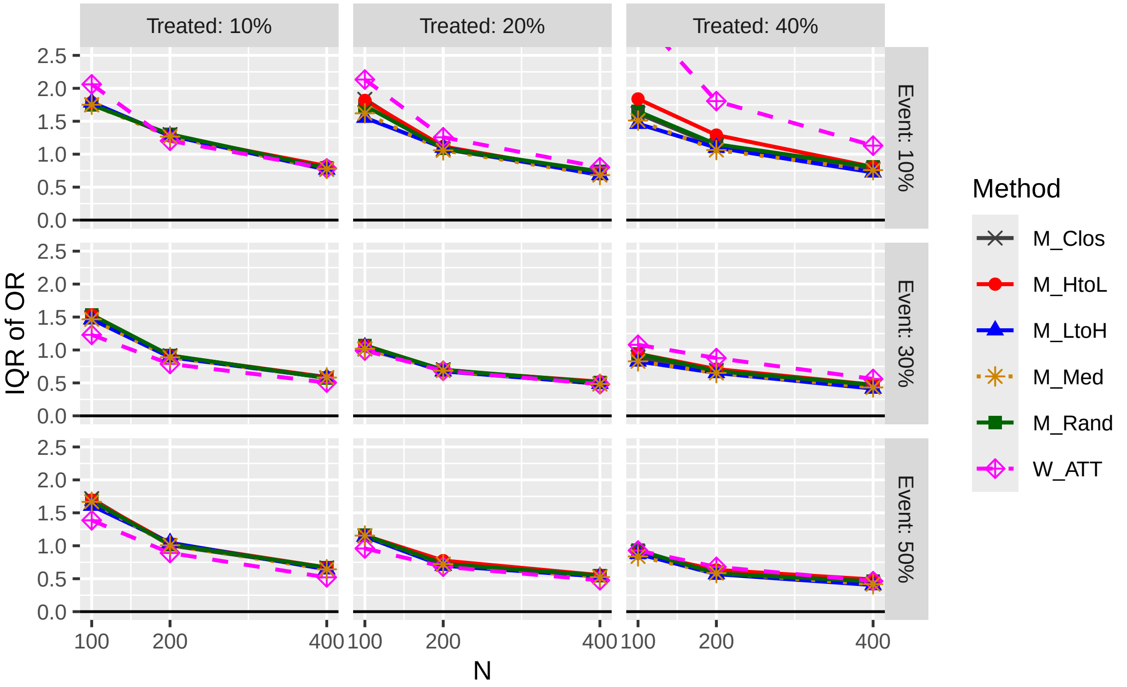

Figure S97. IQR for OR (unimodal continuous covariate, matching ratio 1:2, true OR: 1, c statistic: 0.85).

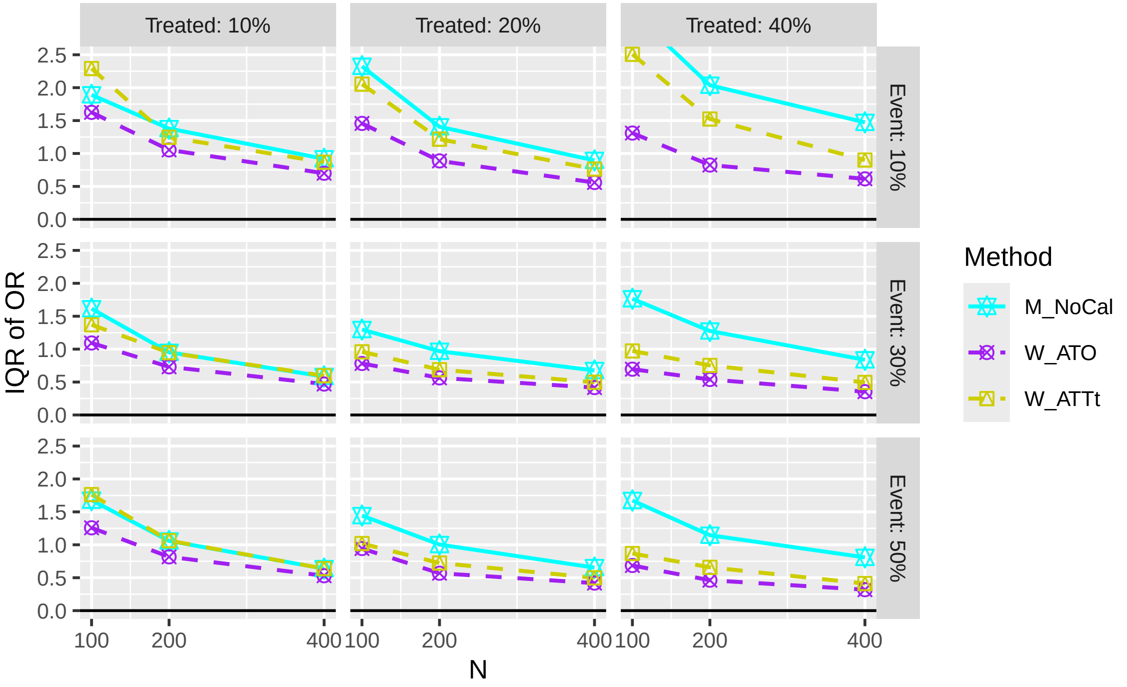

Figure S98. IQR for OR (unimodal continuous covariate, matching ratio 1:2, true OR: 1, c statistic: 0.85); other methods.

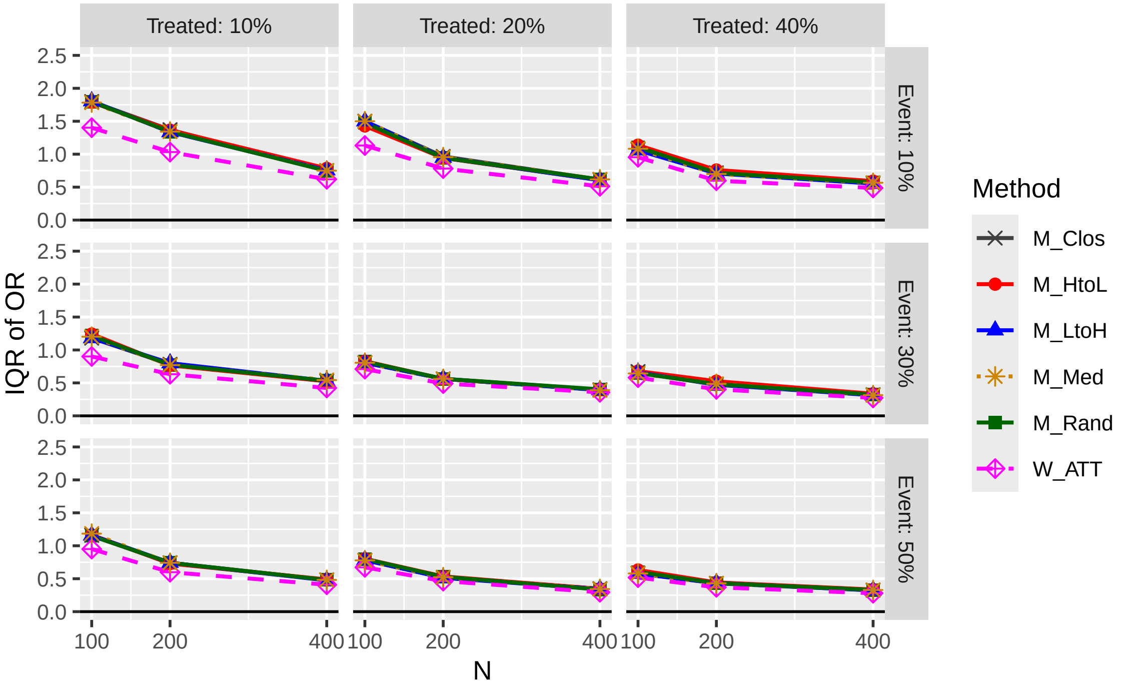

Figure S99. IQR for OR (unimodal continuous covariate, matching ratio 1:2, true OR: 1, c statistic: 0.6).

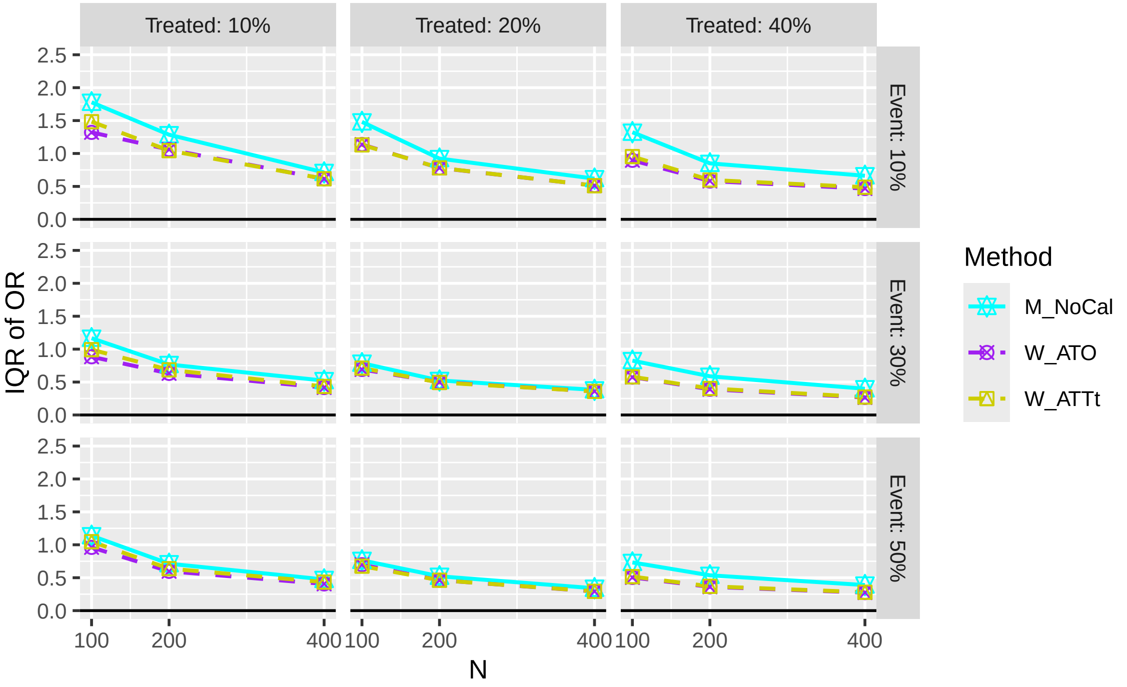

Figure S100. IQR for OR (unimodal continuous covariate, matching ratio 1:2, true OR: 1, c statistic: 0.6); other methods.

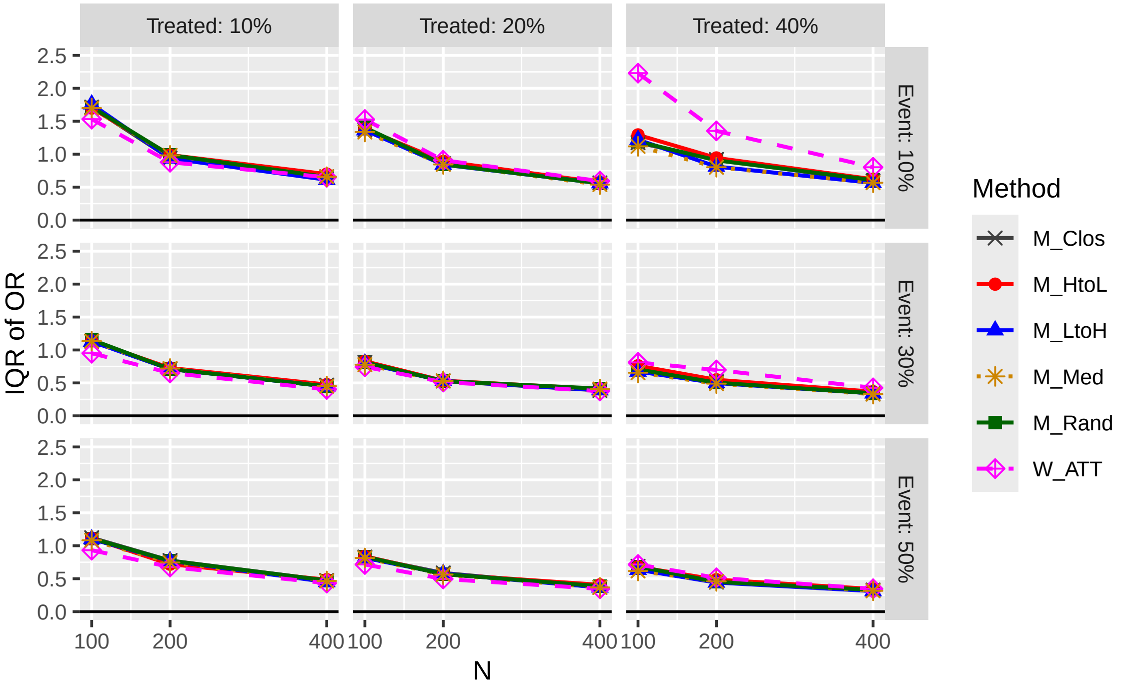

Figure S101. IQR for OR (unimodal continuous covariate, matching ratio 1:2, true OR: 0.75, c statistic: 0.85).

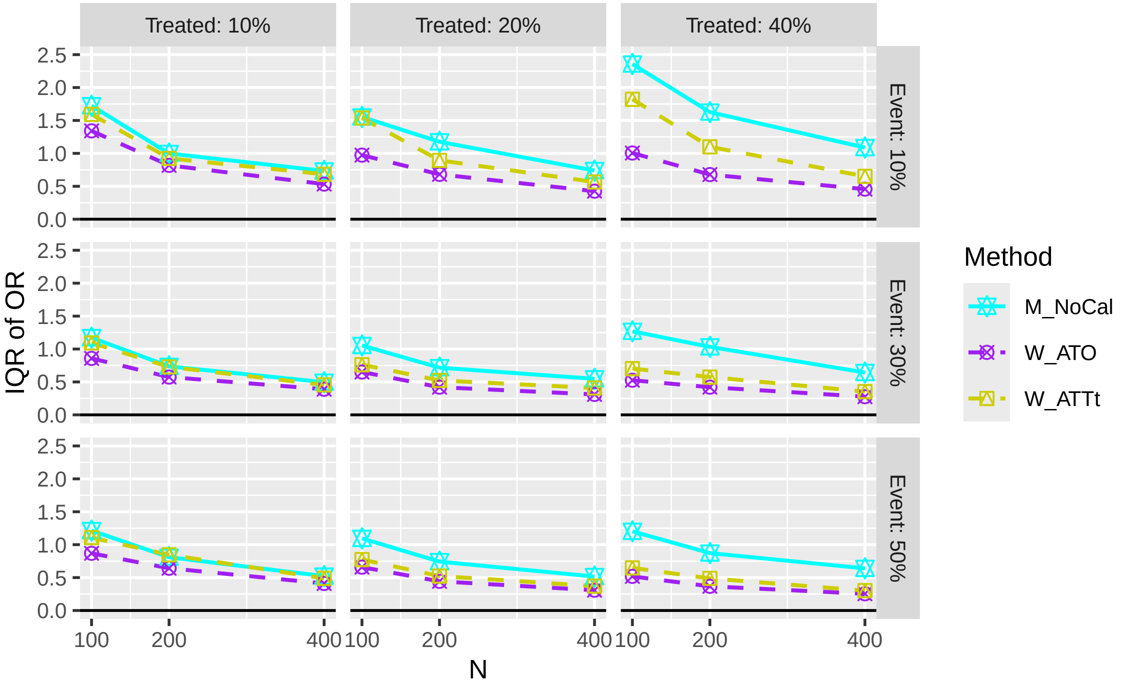

Figure S102. IQR for OR (unimodal continuous covariate, matching ratio 1:2, true OR: 0.75, c statistic: 0.85); other methods.

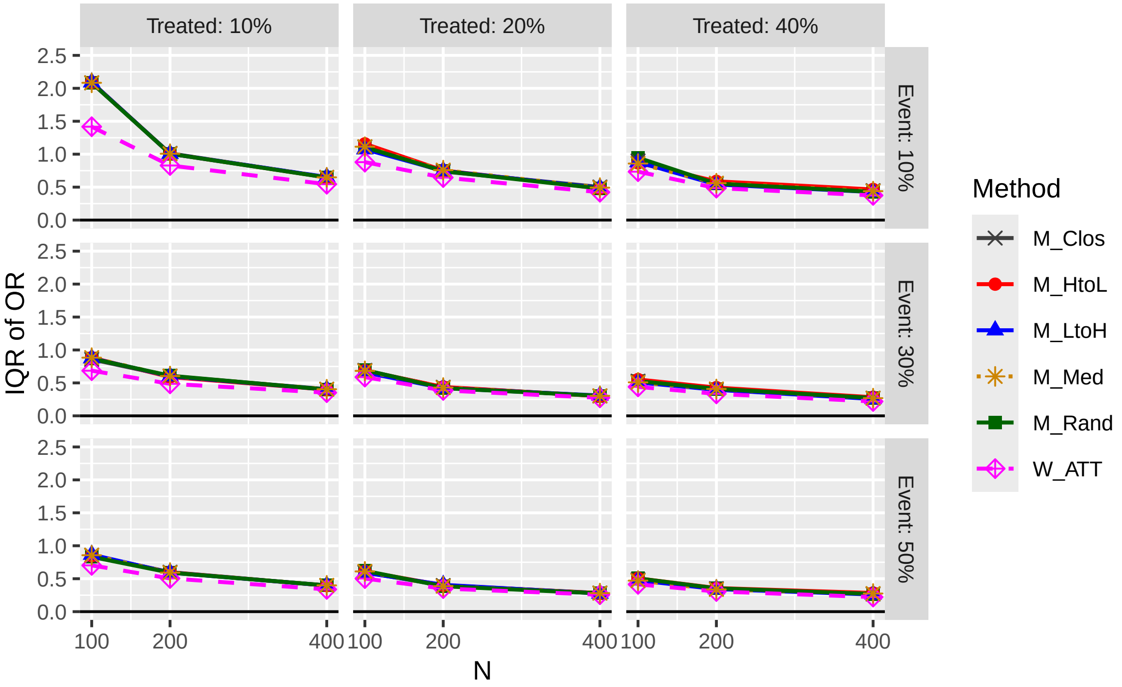

Figure S103. IQR for OR (unimodal continuous covariate, matching ratio 1:2, true OR: 0.75, c statistic: 0.6).

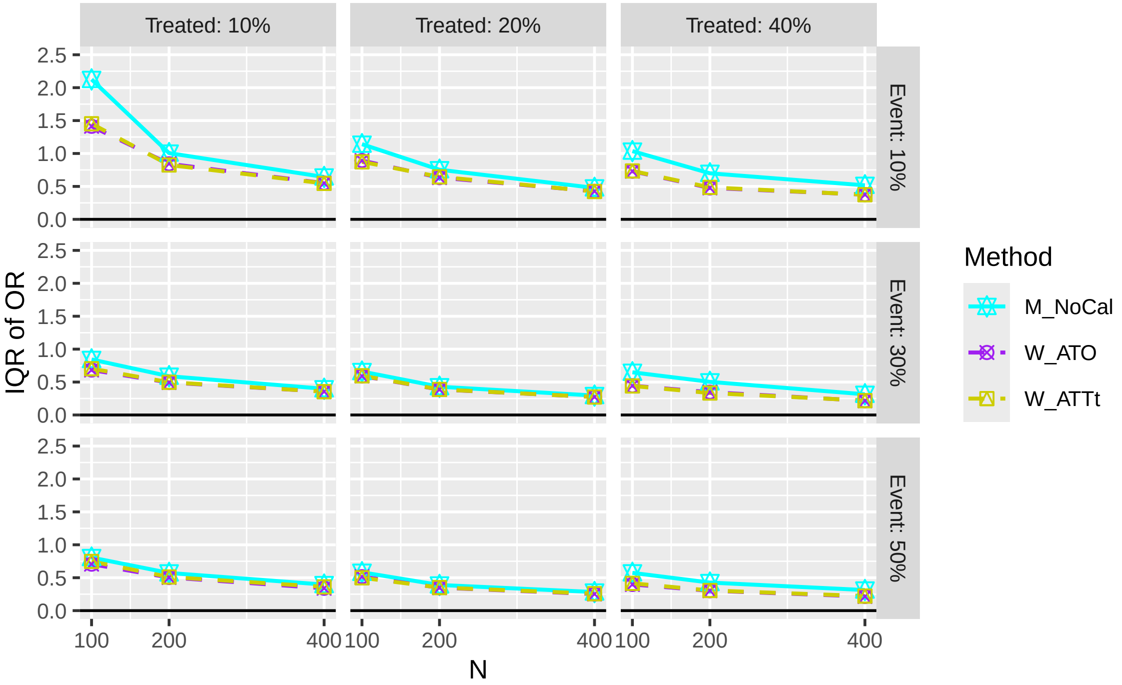

Figure S104. IQR for OR (unimodal continuous covariate, matching ratio 1:2, true OR: 0.75, c statistic: 0.6); other methods.

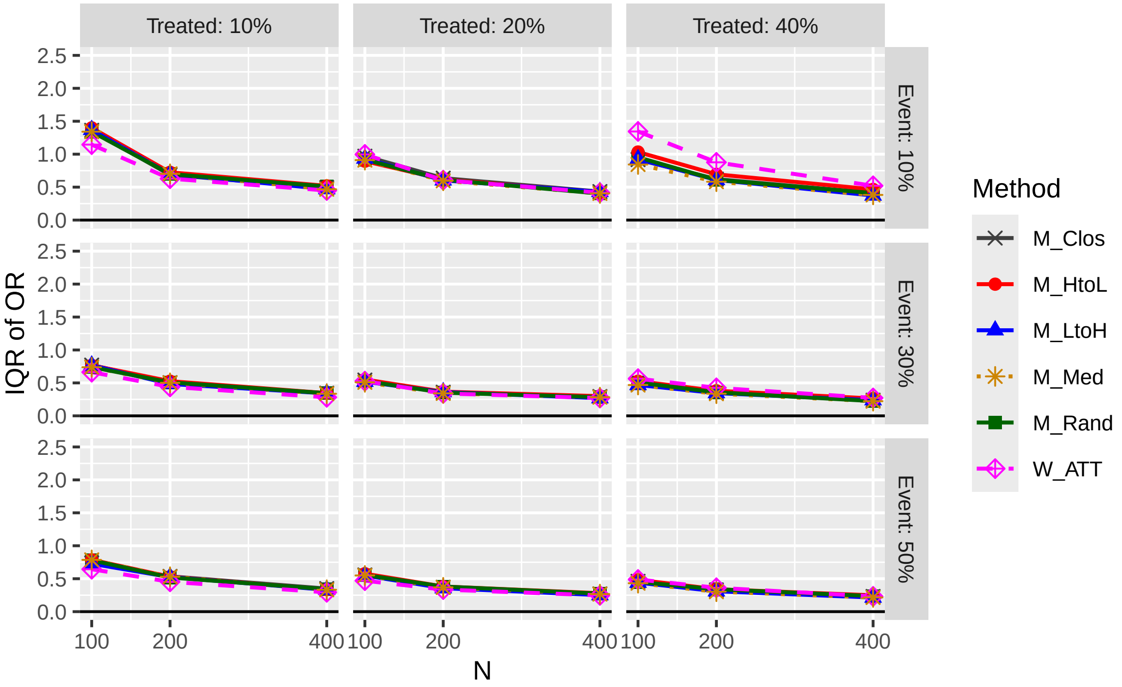

Figure S105. IQR for OR (unimodal continuous covariate, matching ratio 1:2, true OR: 0.5, c statistic: 0.85).

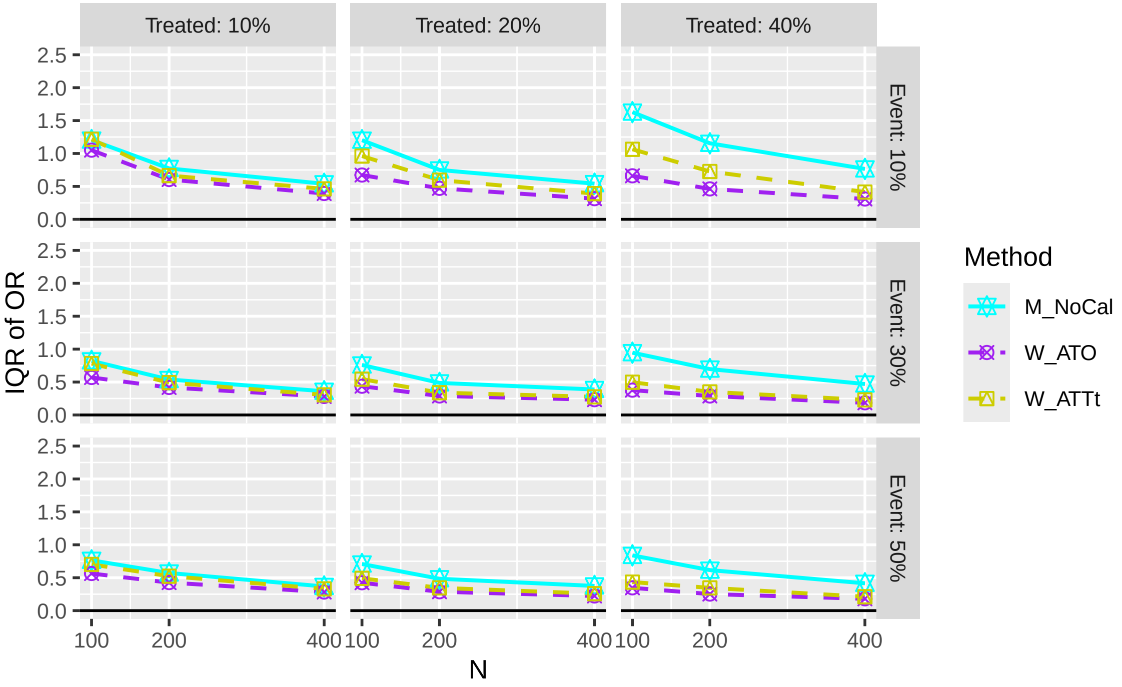

Figure S106. IQR for OR (unimodal continuous covariate, matching ratio 1:2, true OR: 0.5, c statistic: 0.85); other methods.

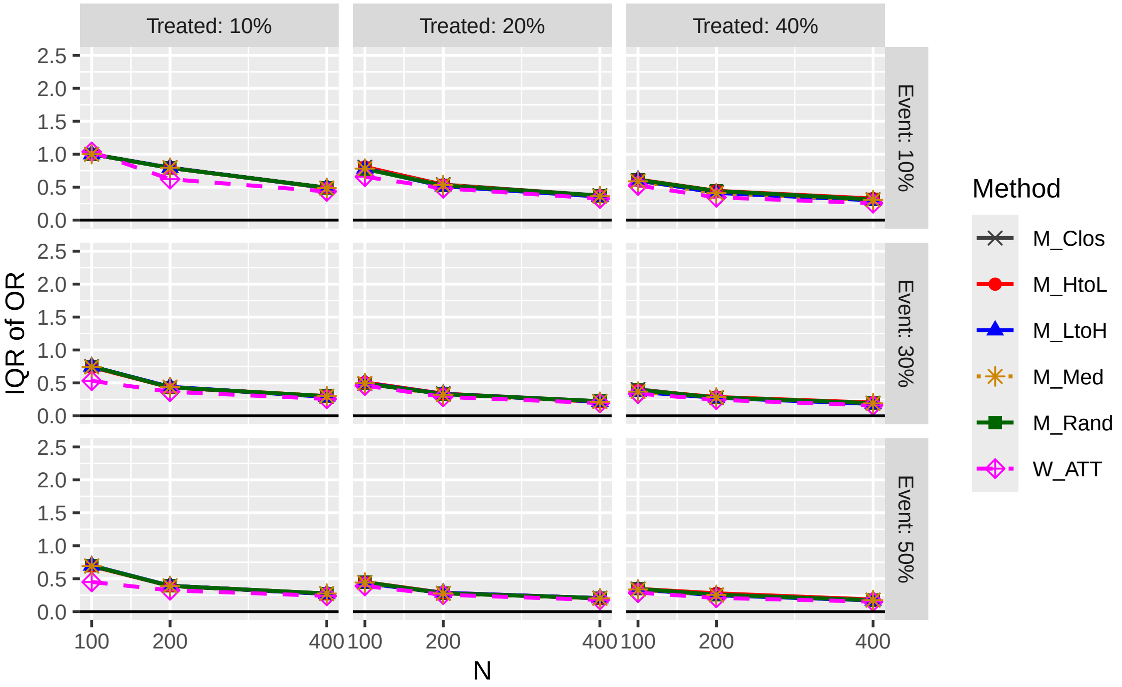

Figure S107. IQR for OR (unimodal continuous covariate, matching ratio 1:2, true OR: 0.5, c statistic: 0.6).

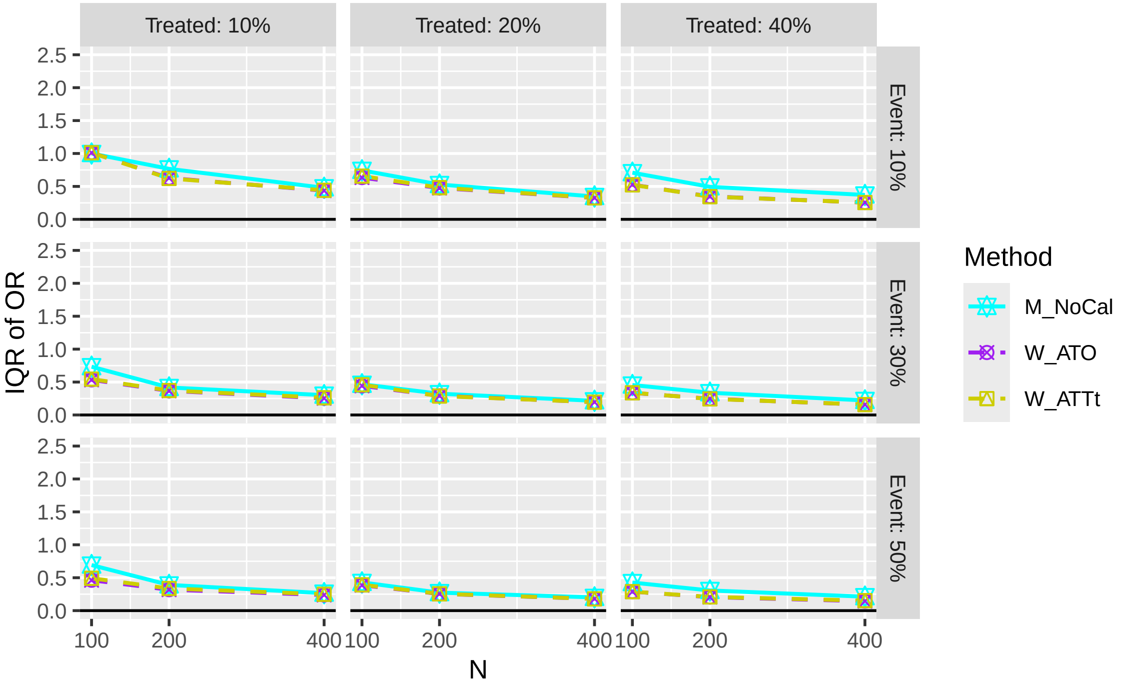

Figure S108. IQR for OR (unimodal continuous covariate, matching ratio 1:2, true OR: 0.5, c statistic: 0.6); other methods.

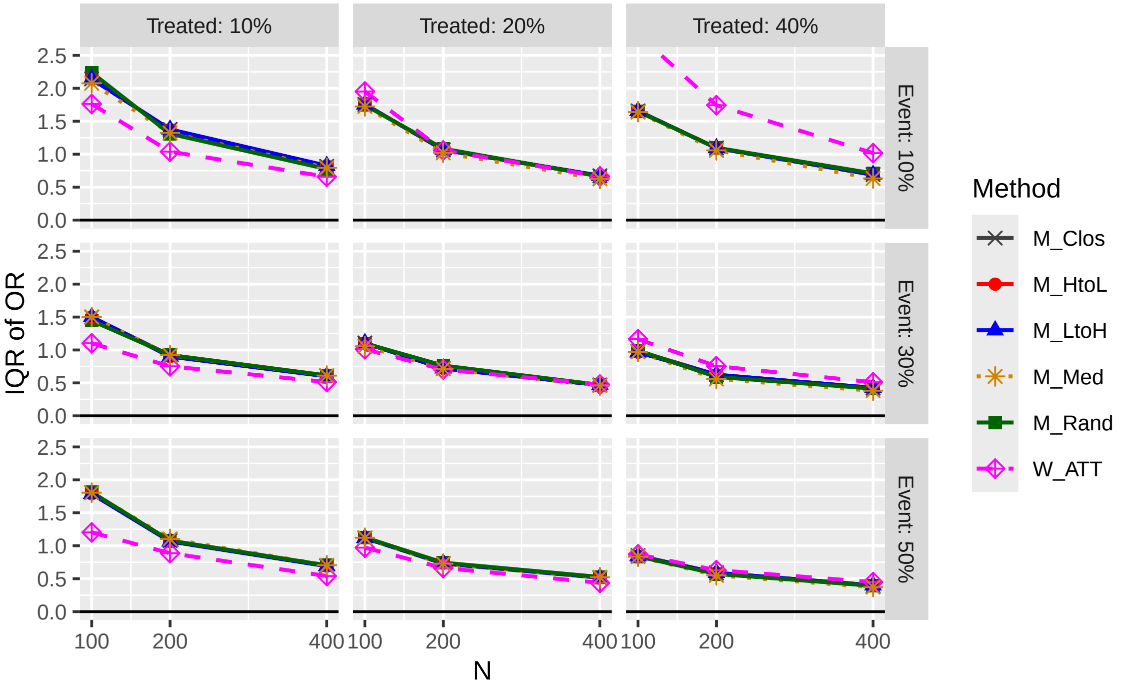

Figure S109. IQR for OR (categorical covariate, matching ratio 1:1, true OR: 1, c statistic: 0.85).

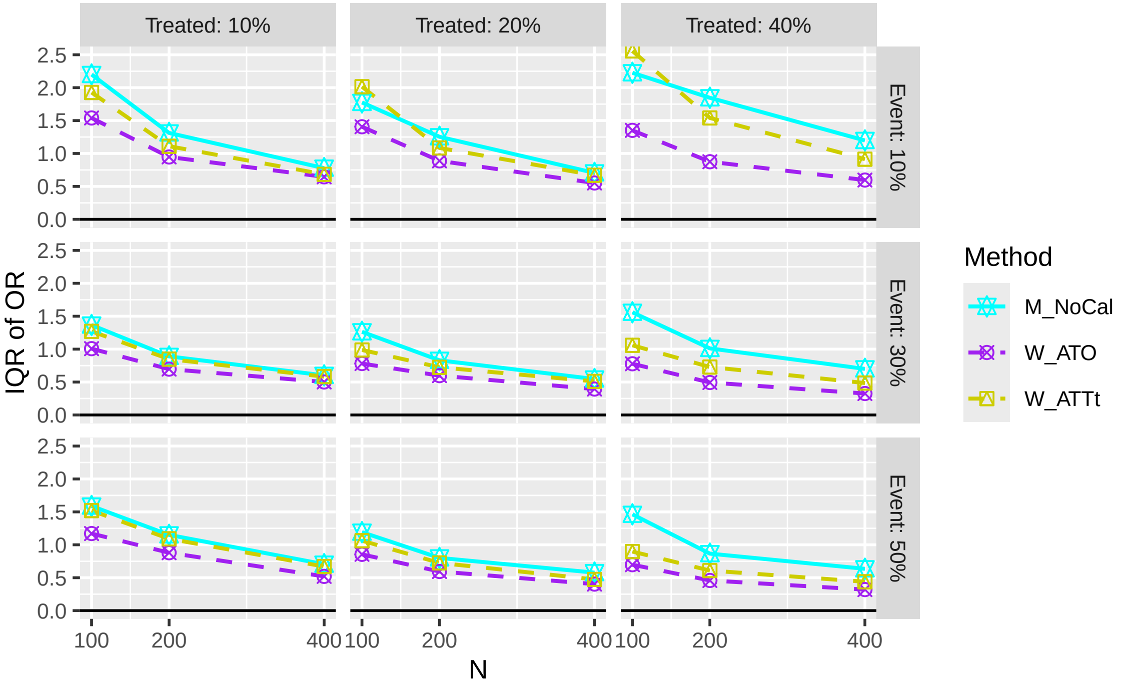

Figure S110. IQR for OR (categorical covariate, matching ratio 1:1, true OR: 1, c statistic: 0.85); other methods.

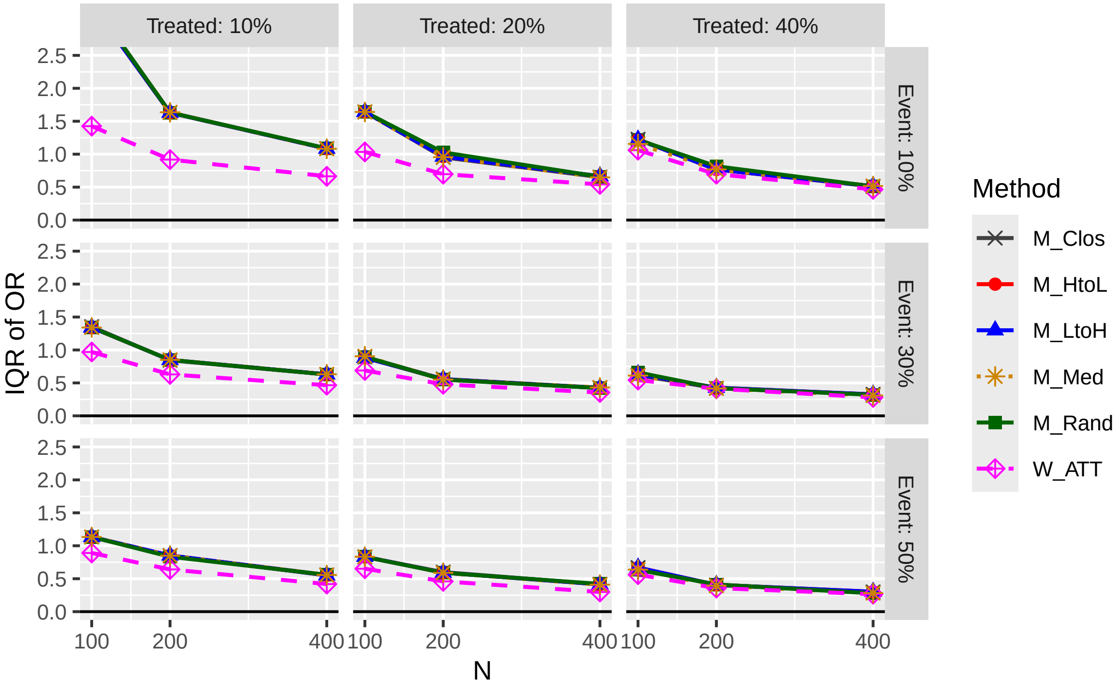

Figure S111. IQR for OR (categorical covariate, matching ratio 1:1, true OR: 1, c statistic: 0.6).

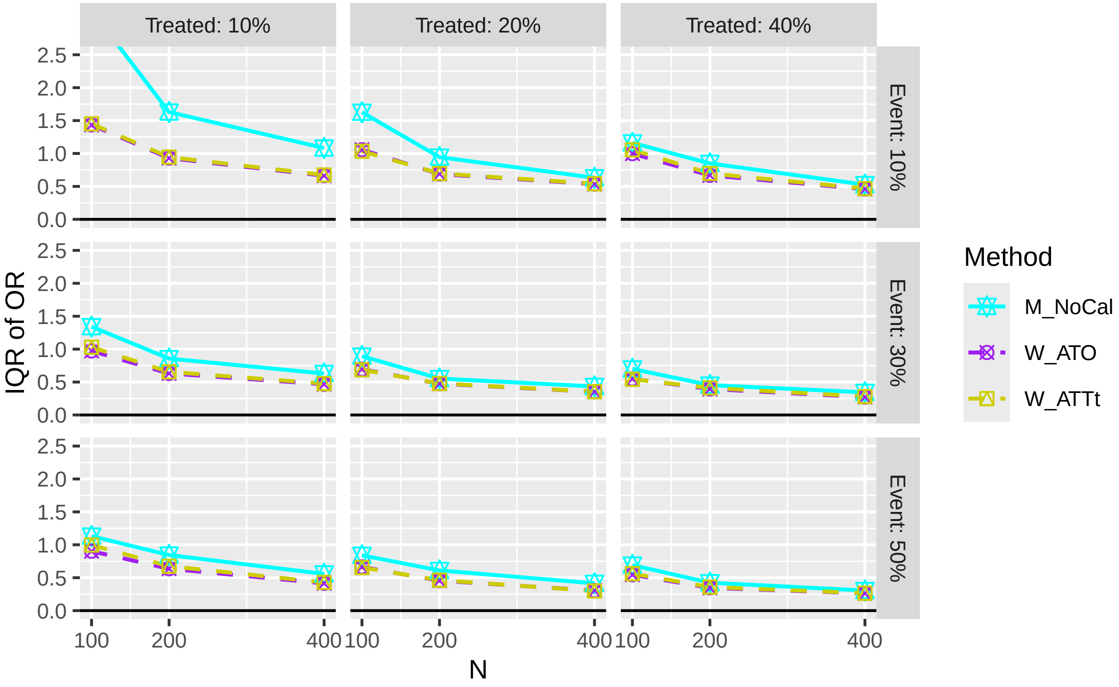

Figure S112. IQR for OR (categorical covariate, matching ratio 1:1, true OR: 1, c statistic: 0.6); other methods.

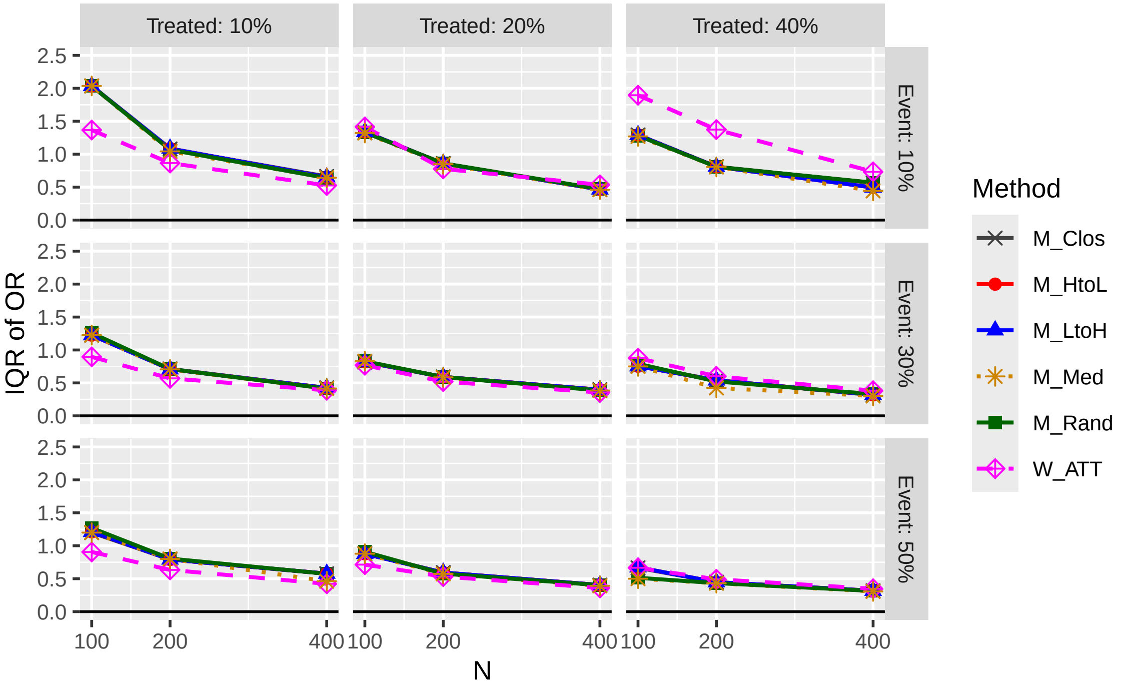

Figure S113. IQR for OR (categorical covariate, matching ratio 1:1, true OR: 0.75, c statistic: 0.85).

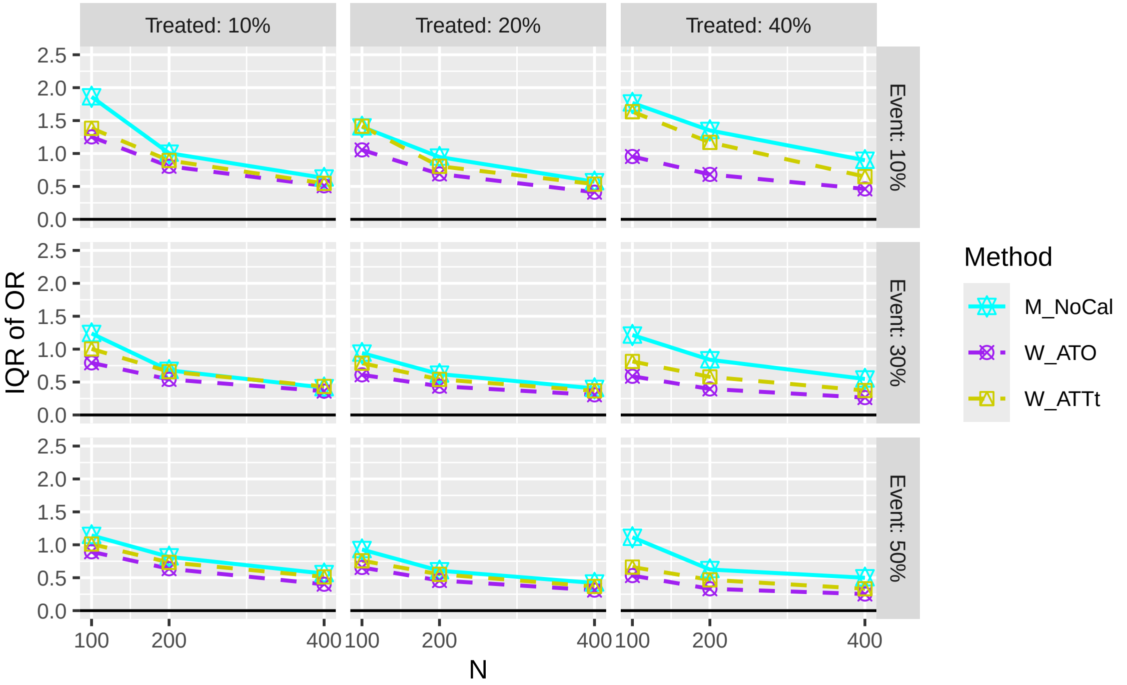

Figure S114. IQR for OR (categorical covariate, matching ratio 1:1, true OR: 0.75, c statistic: 0.85); other methods.

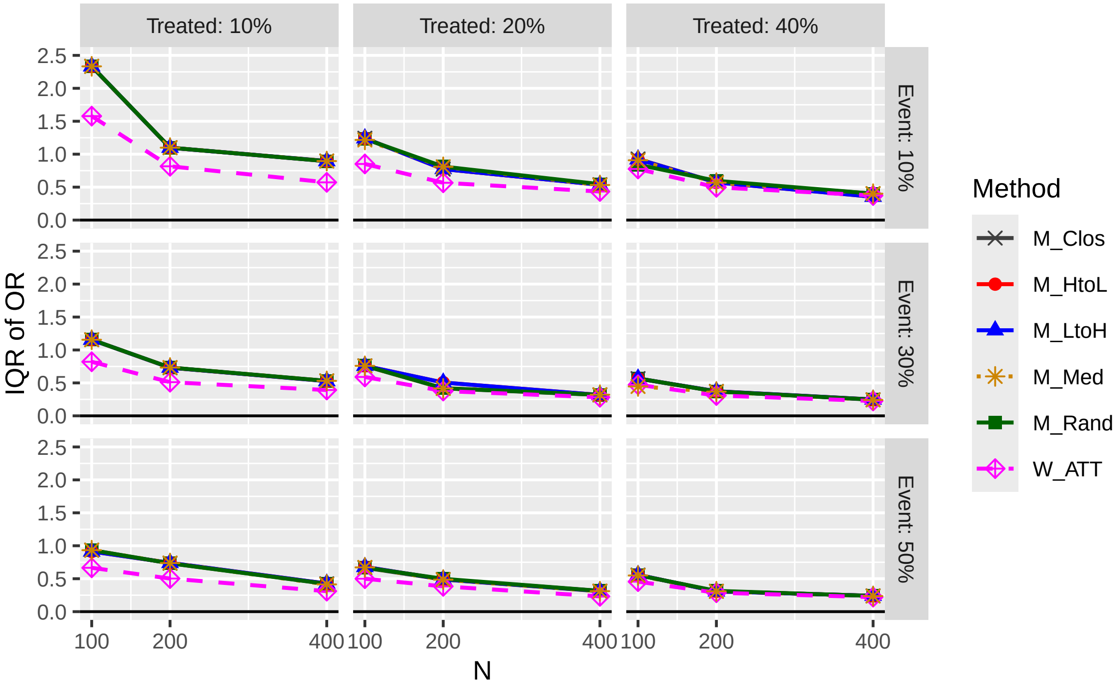

Figure S115. IQR for OR (categorical covariate, matching ratio 1:1, true OR: 0.75, c statistic: 0.6).

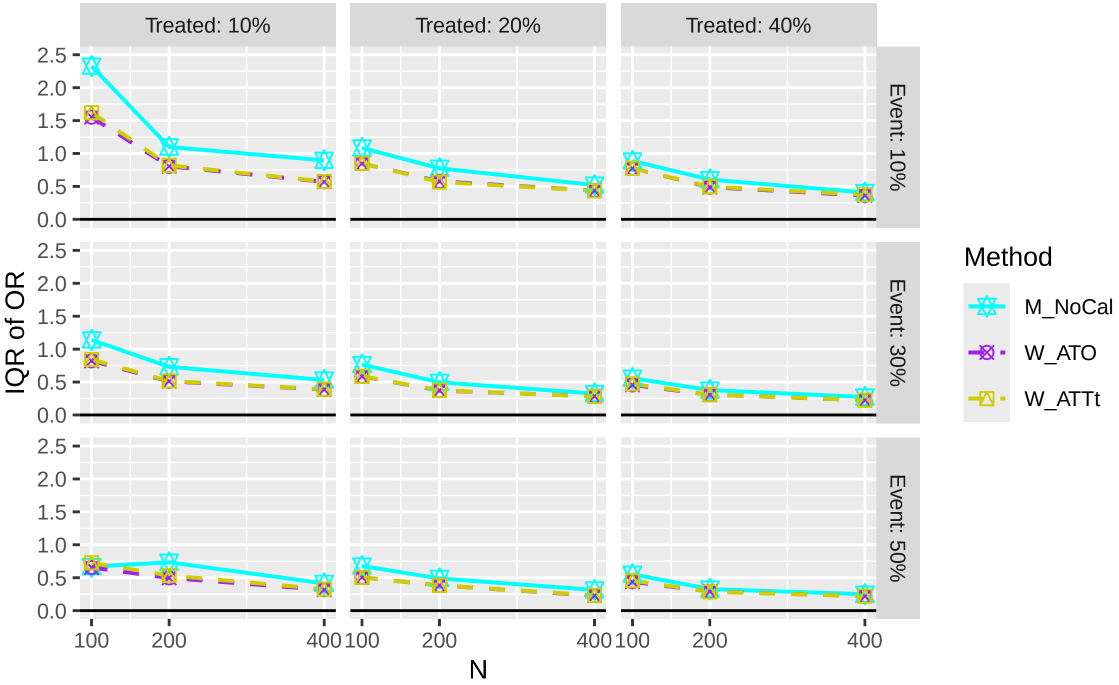

Figure S116. IQR for OR (categorical covariate, matching ratio 1:1, true OR: 0.75, c statistic: 0.6); other methods.

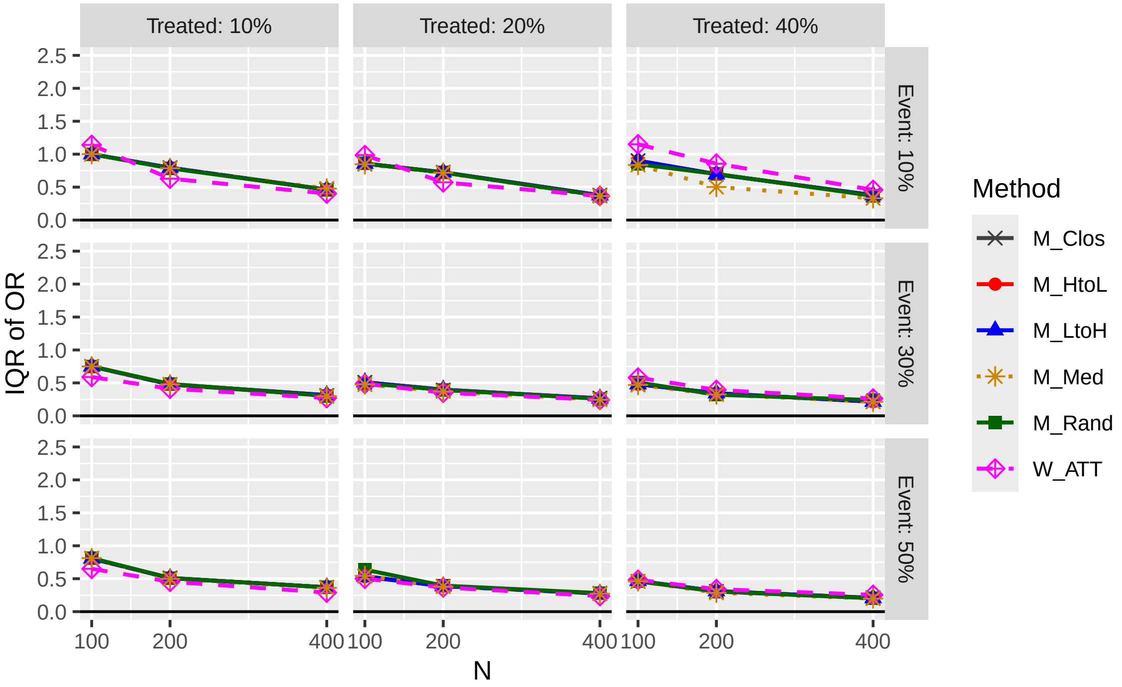

Figure S117. IQR for OR (categorical covariate, matching ratio 1:1, true OR: 0.5, c statistic: 0.85).

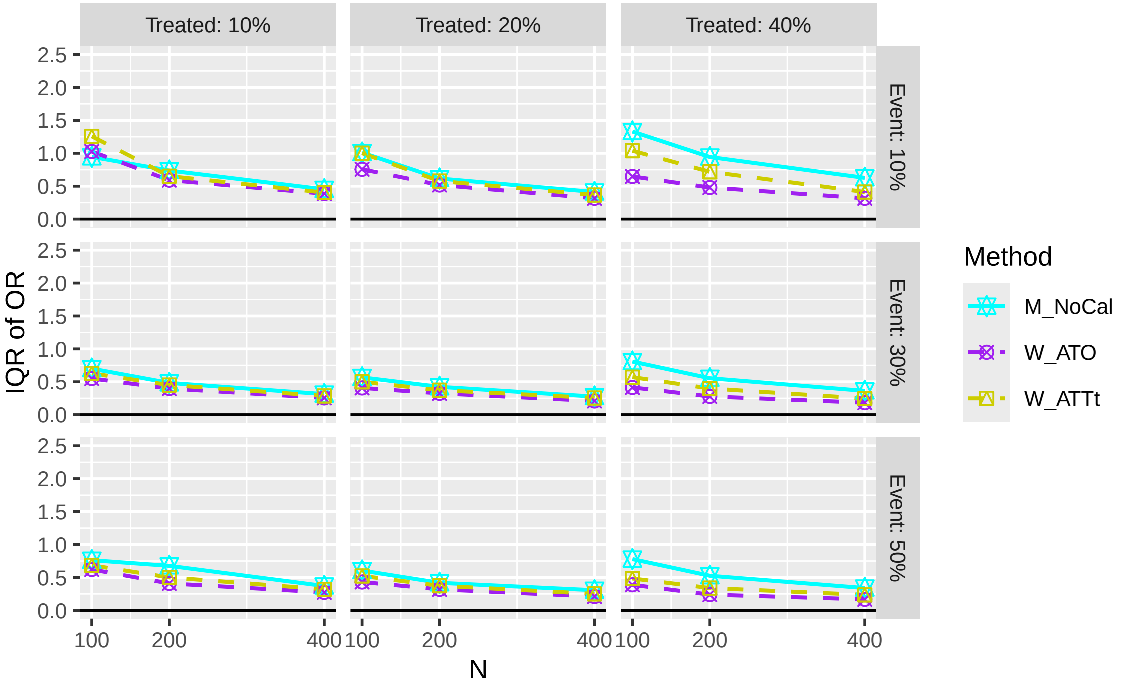

Figure S118. IQR for OR (categorical covariate, matching ratio 1:1, true OR: 0.5, c statistic: 0.85); other methods.

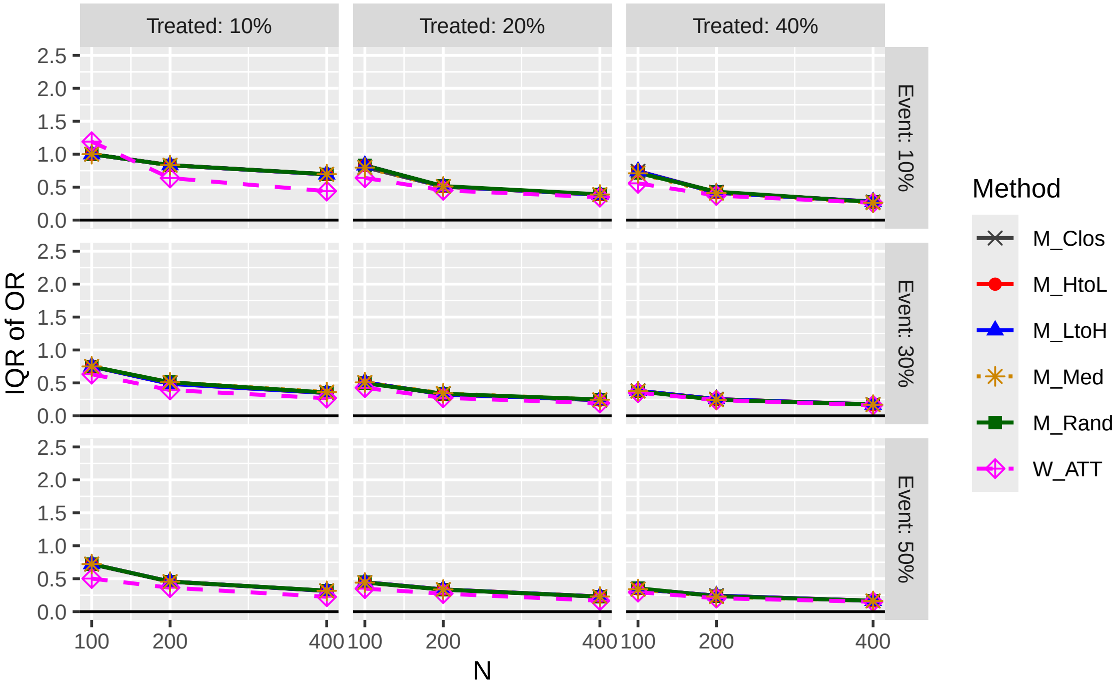

Figure S119. IQR for OR (categorical covariate, matching ratio 1:1, true OR: 0.5, c statistic: 0.6).

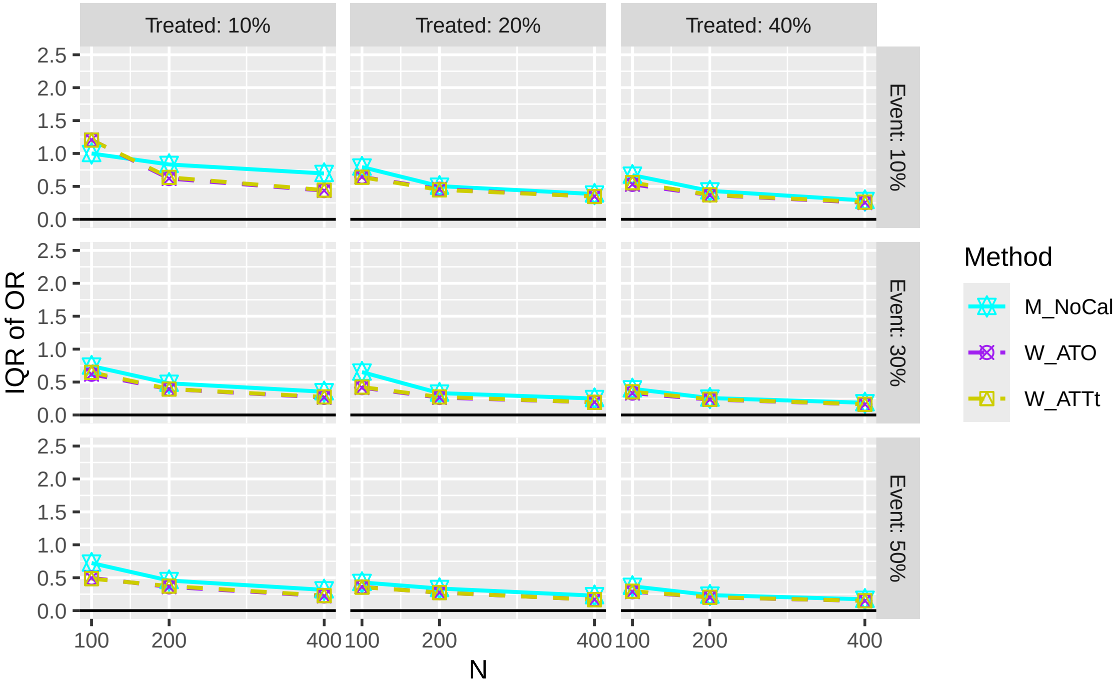

Figure S120. IQR for OR (categorical covariate, matching ratio 1:1, true OR: 0.5, c statistic: 0.6); other methods.

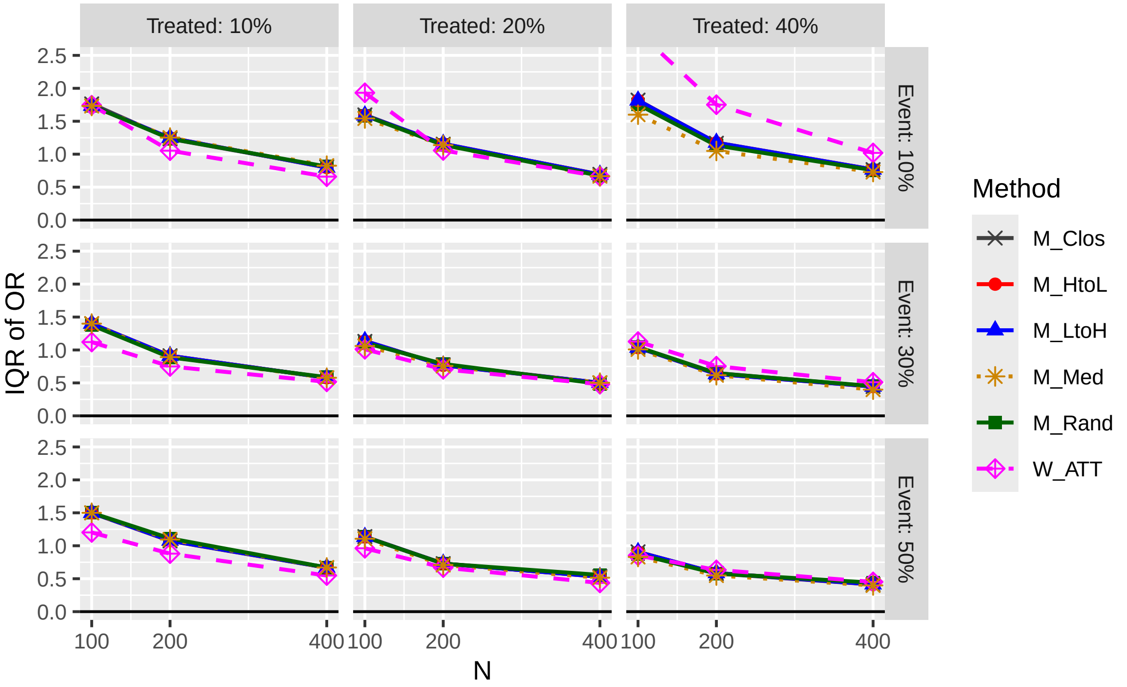

Figure S121. IQR for OR (categorical covariate, matching ratio 1:2, true OR: 1, c statistic: 0.85).

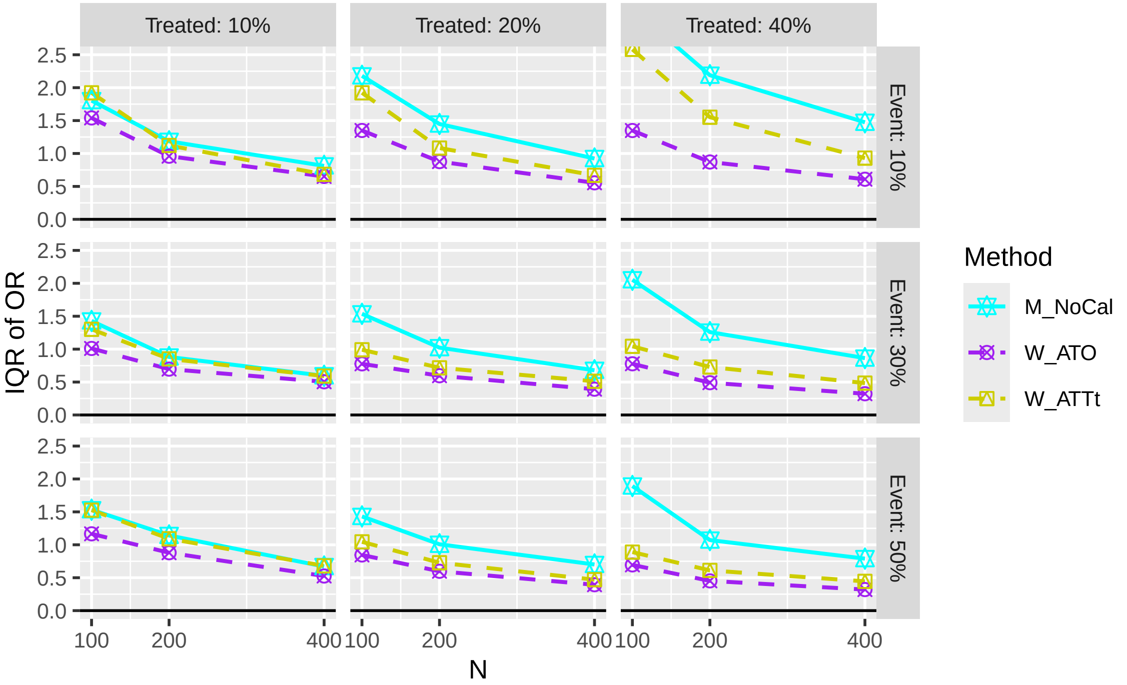

Figure S122. IQR for OR (categorical covariate, matching ratio 1:2, true OR: 1, c statistic: 0.85); other methods.

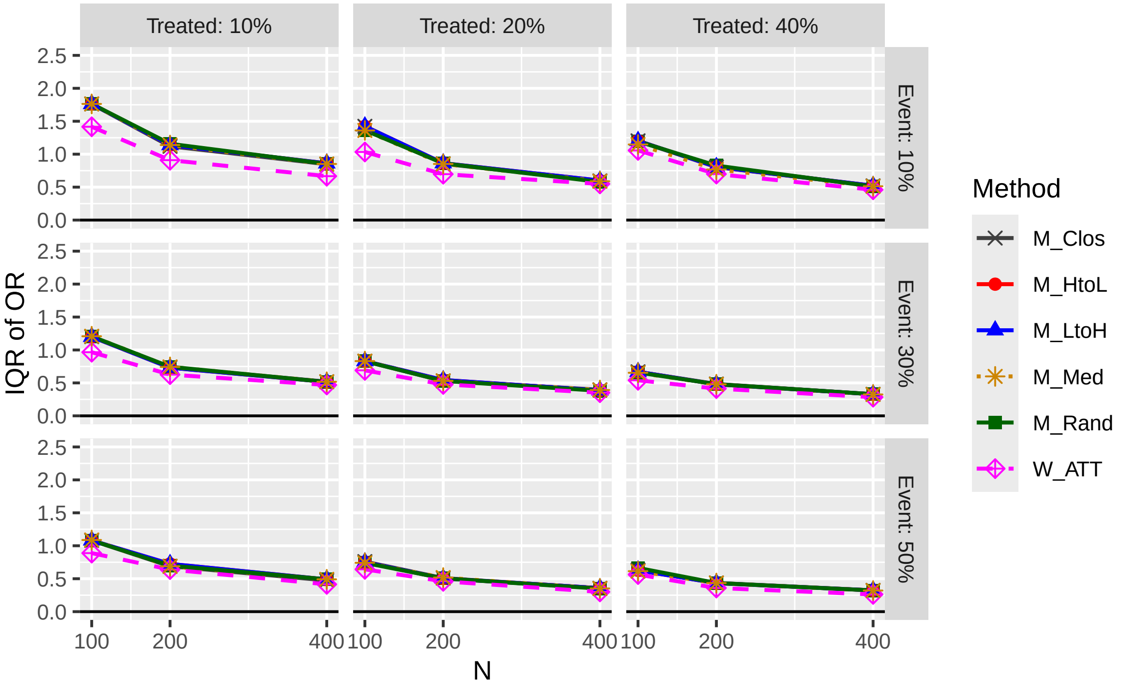

Figure S123. IQR for OR (categorical covariate, matching ratio 1:2, true OR: 1, c statistic: 0.6).

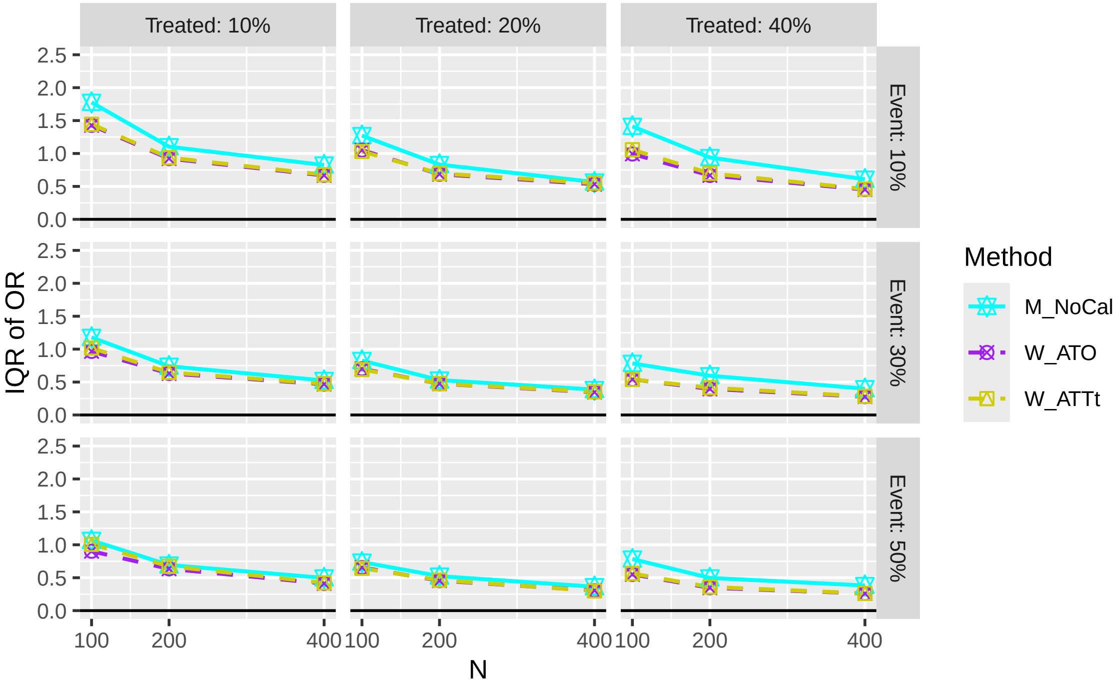

Figure S124. IQR for OR (categorical covariate, matching ratio 1:2, true OR: 1, c statistic: 0.6); other methods.

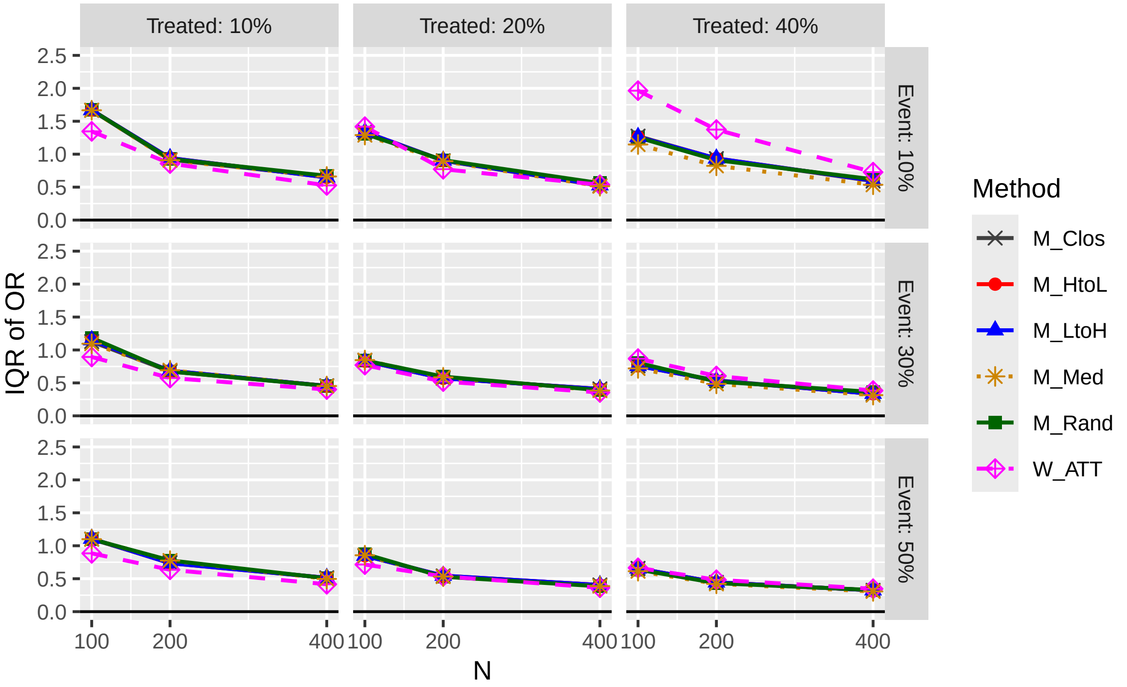

Figure S125. IQR for OR (categorical covariate, matching ratio 1:2, true OR: 0.75, c statistic: 0.85).

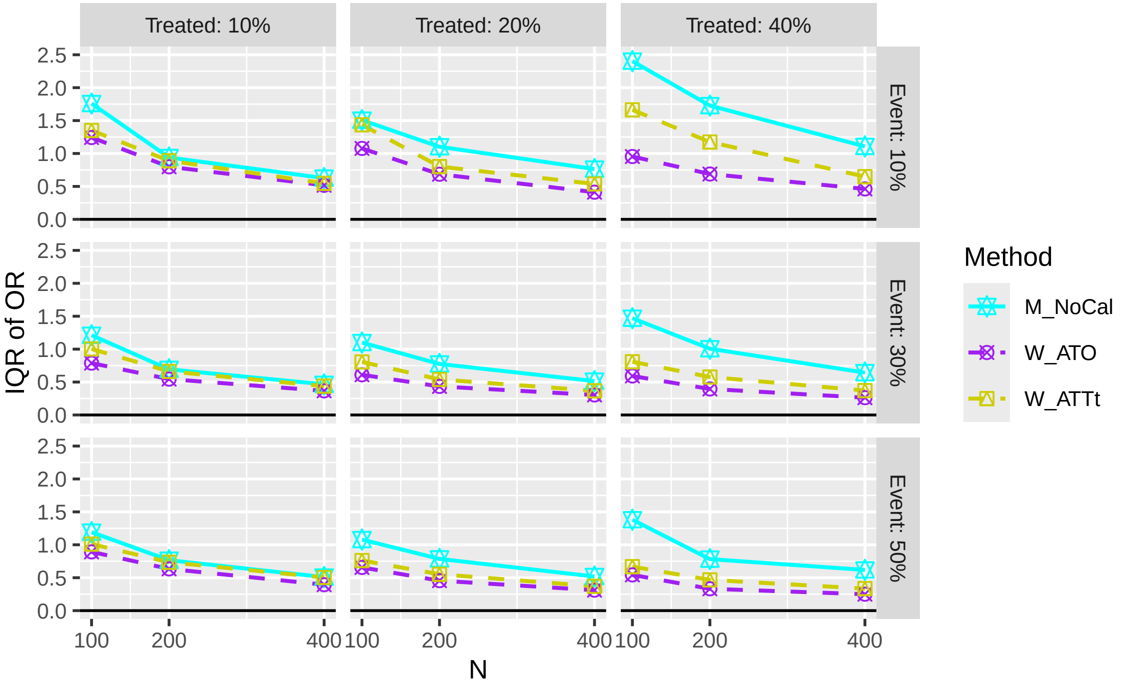

Figure S126. IQR for OR (categorical covariate, matching ratio 1:2, true OR: 0.75, c statistic: 0.85); other methods.

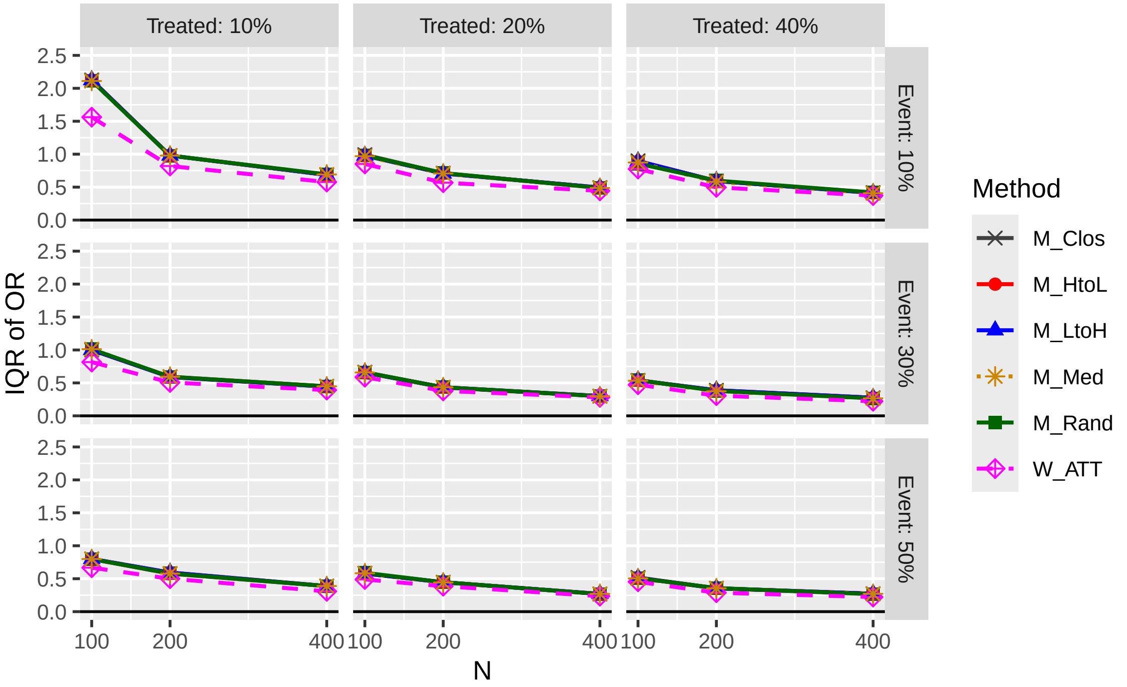

Figure S127. IQR for OR (categorical covariate, matching ratio 1:2, true OR: 0.75, c statistic: 0.6).

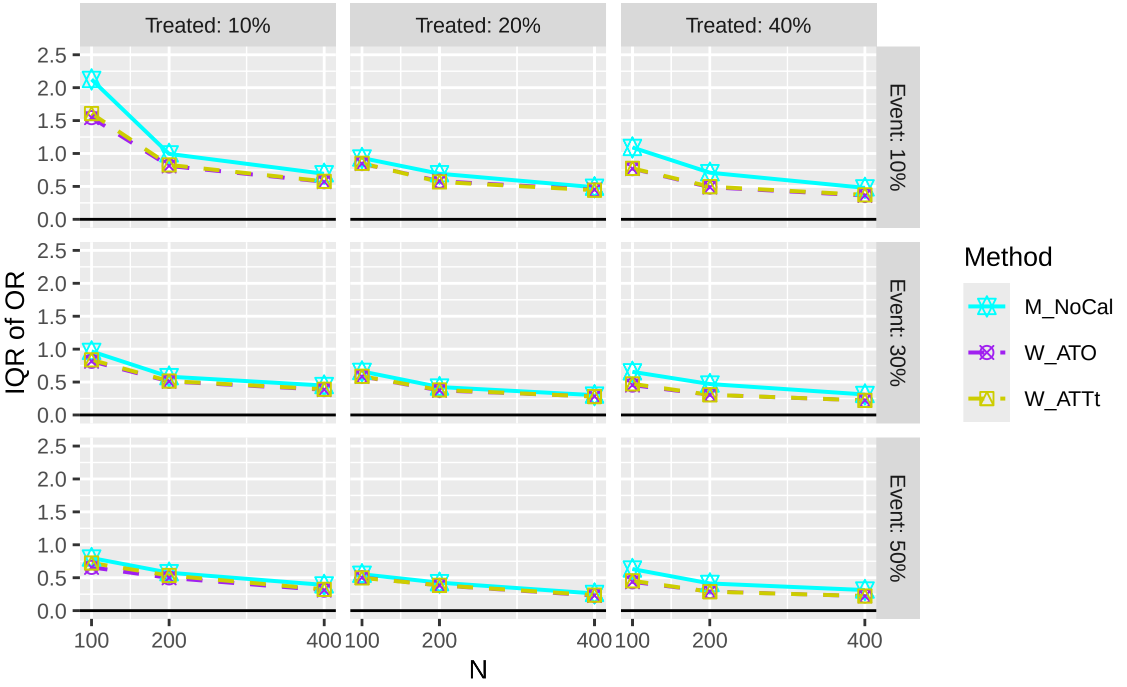

Figure S128. IQR for OR (categorical covariate, matching ratio 1:2, true OR: 0.75, c statistic: 0.6); other methods.

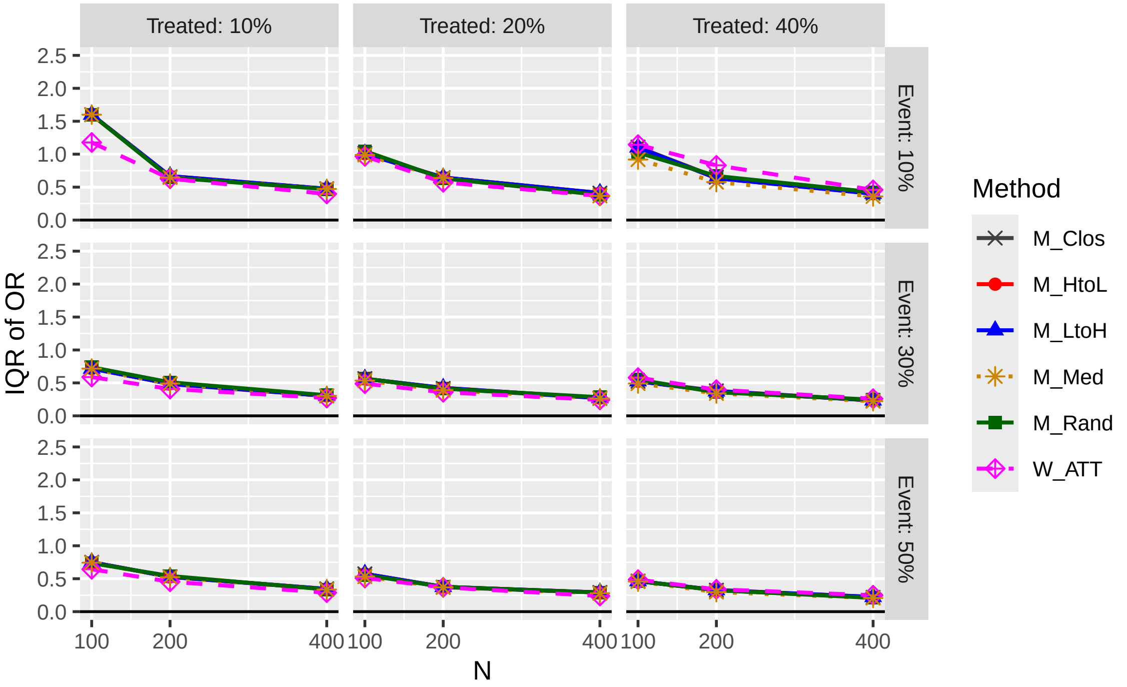

Figure S129. IQR for OR (categorical covariate, matching ratio 1:2, true OR: 0.5, c statistic: 0.85).

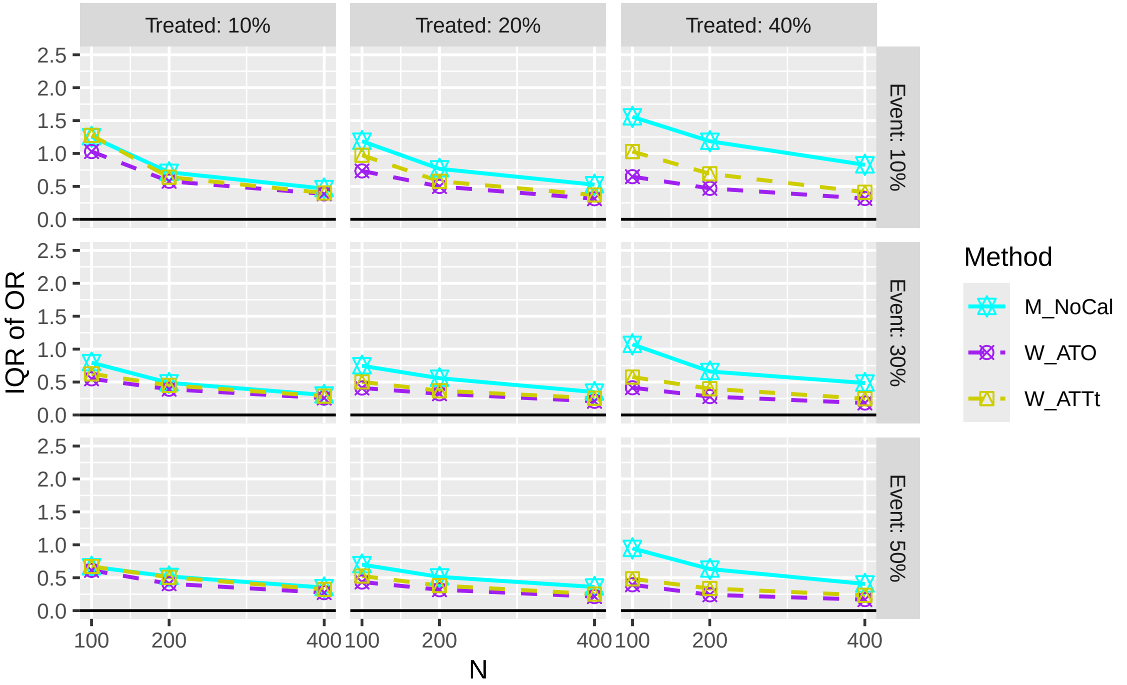

Figure S130. IQR for OR (categorical covariate, matching ratio 1:2, true OR: 0.5, c statistic: 0.85); other methods.

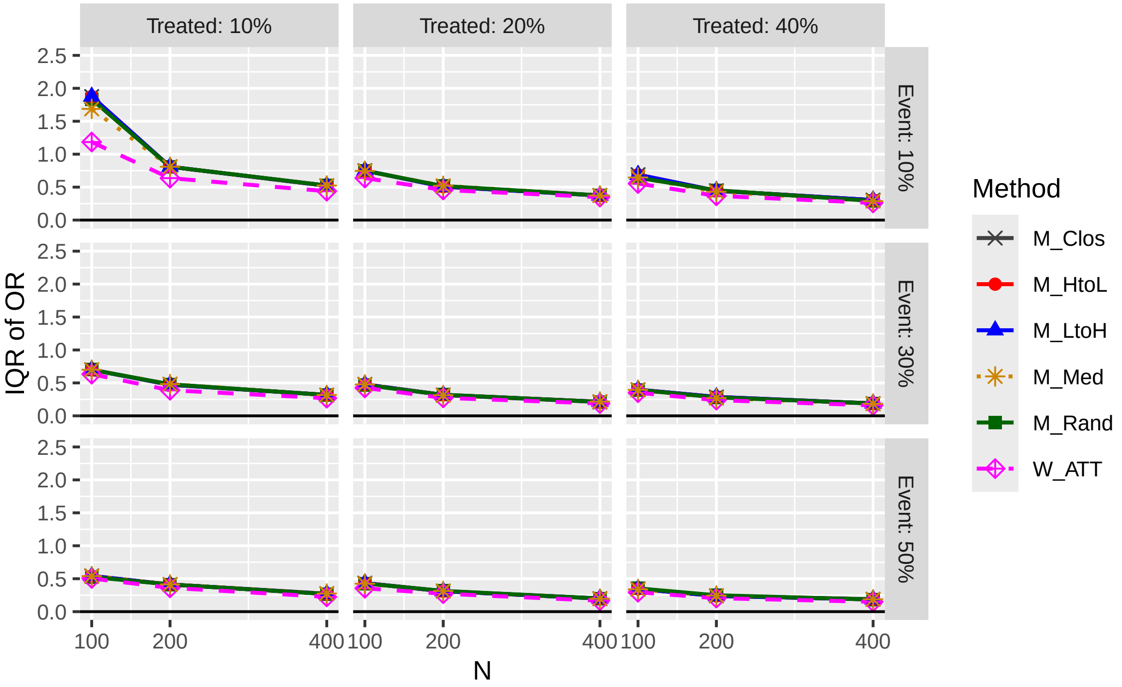

Figure S131. IQR for OR (categorical covariate, matching ratio 1:2, true OR: 0.5, c statistic: 0.6).

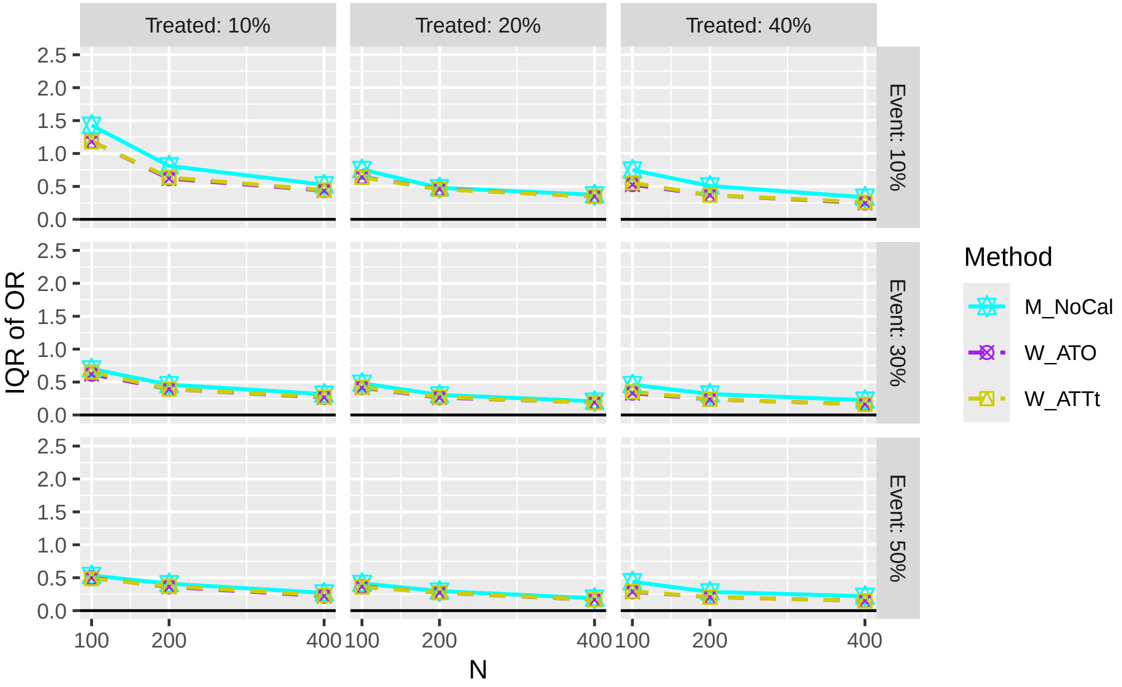

Figure S132. IQR for OR (categorical covariate, matching ratio 1:2, true OR: 0.5, c statistic: 0.6); other methods.

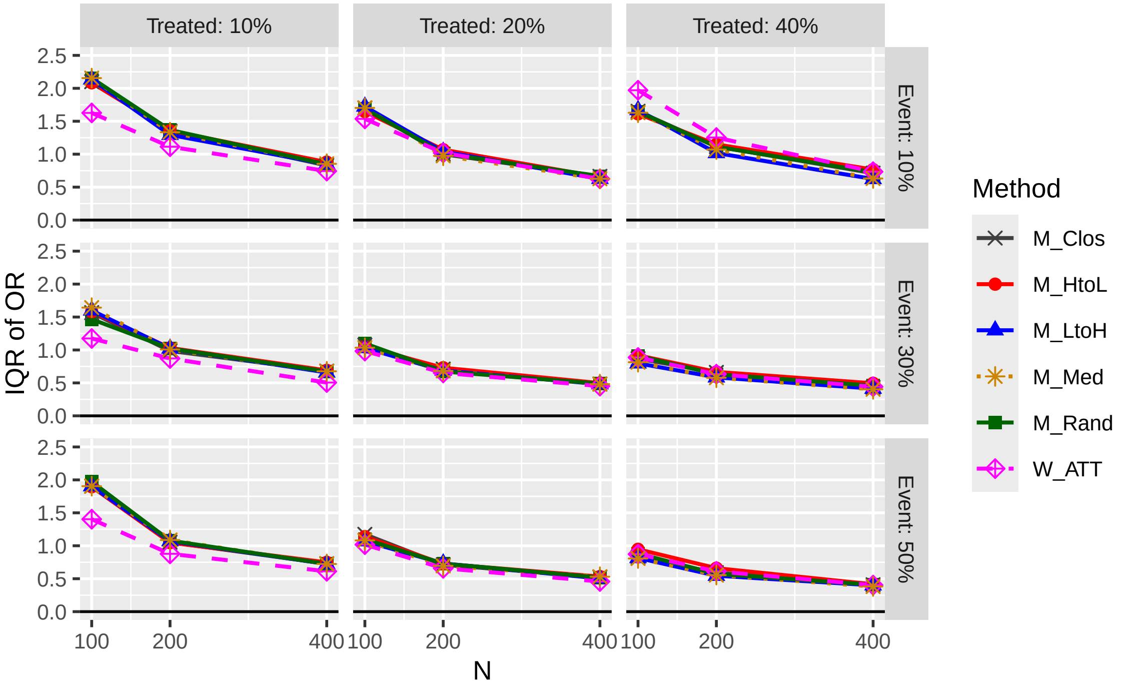

Figure S133. IQR for OR (multimodal continuous covariate, matching ratio 1:1, true OR: 1, c statistic: 0.85).

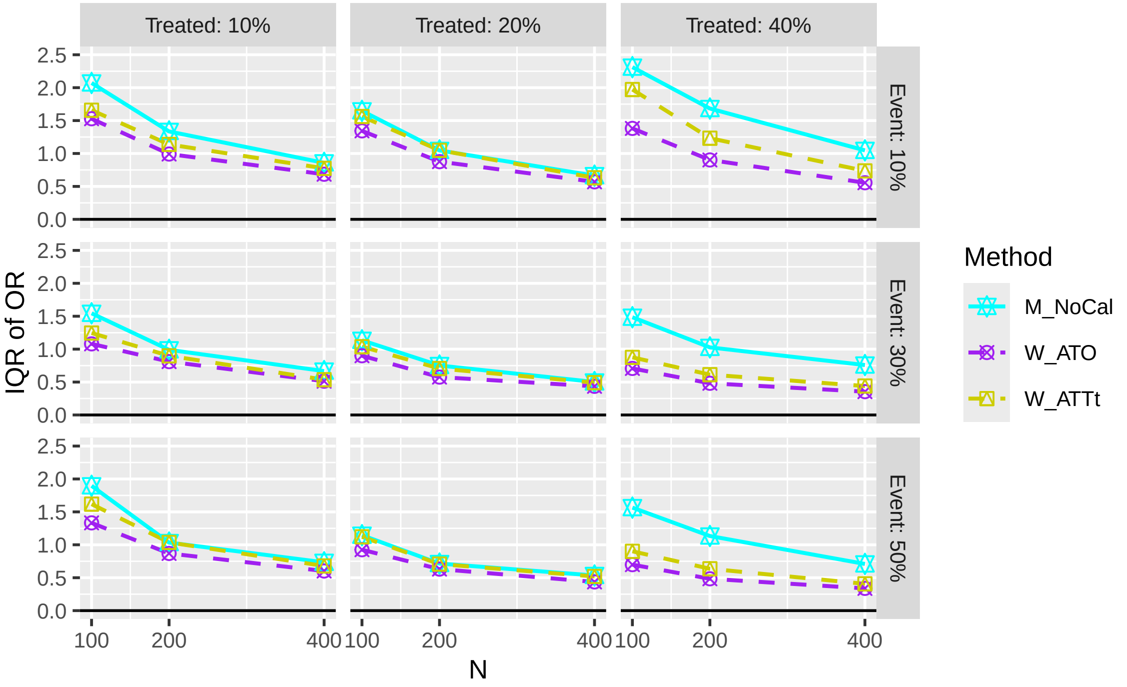

Figure S134. IQR for OR (multimodal continuous covariate, matching ratio 1:1, true OR: 1, c statistic: 0.85); other methods.

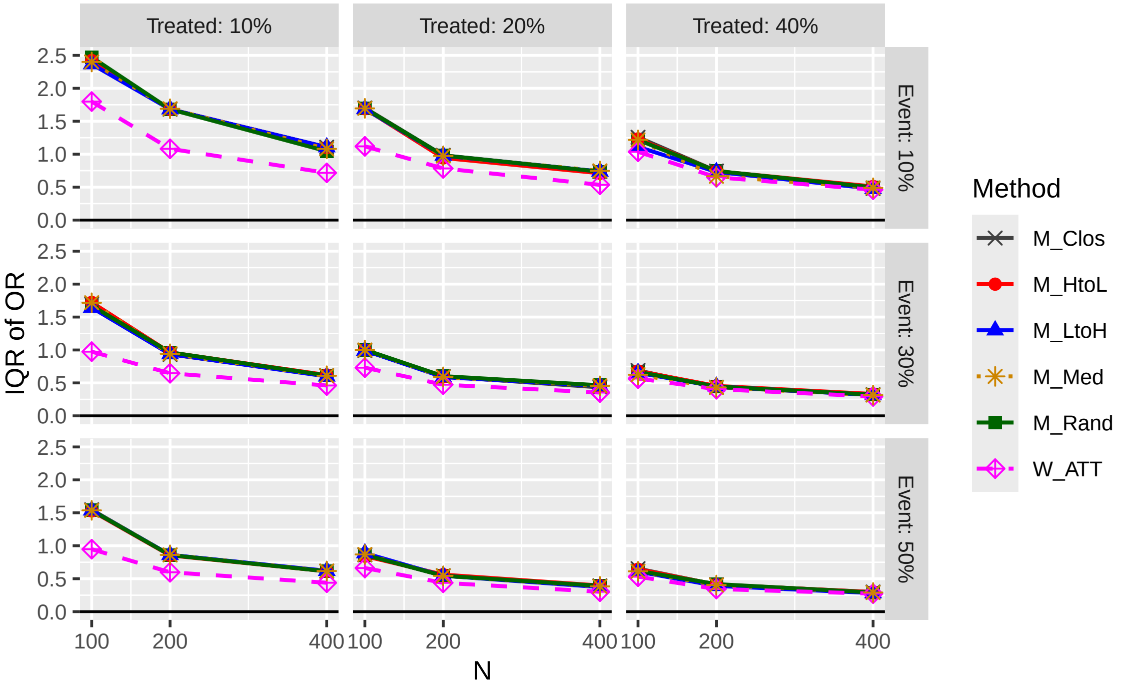

Figure S135. IQR for OR (multimodal continuous covariate, matching ratio 1:1, true OR: 1, c statistic: 0.6).

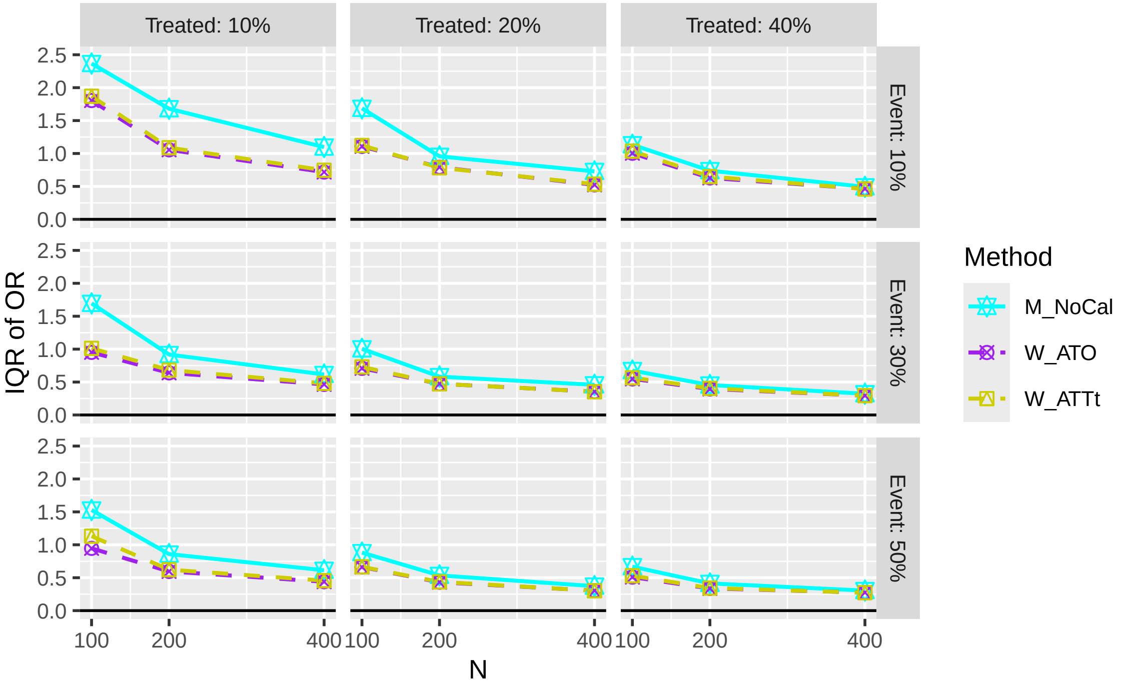

Figure S136. IQR for OR (multimodal continuous covariate, matching ratio 1:1, true OR: 1, c statistic: 0.6); other methods.

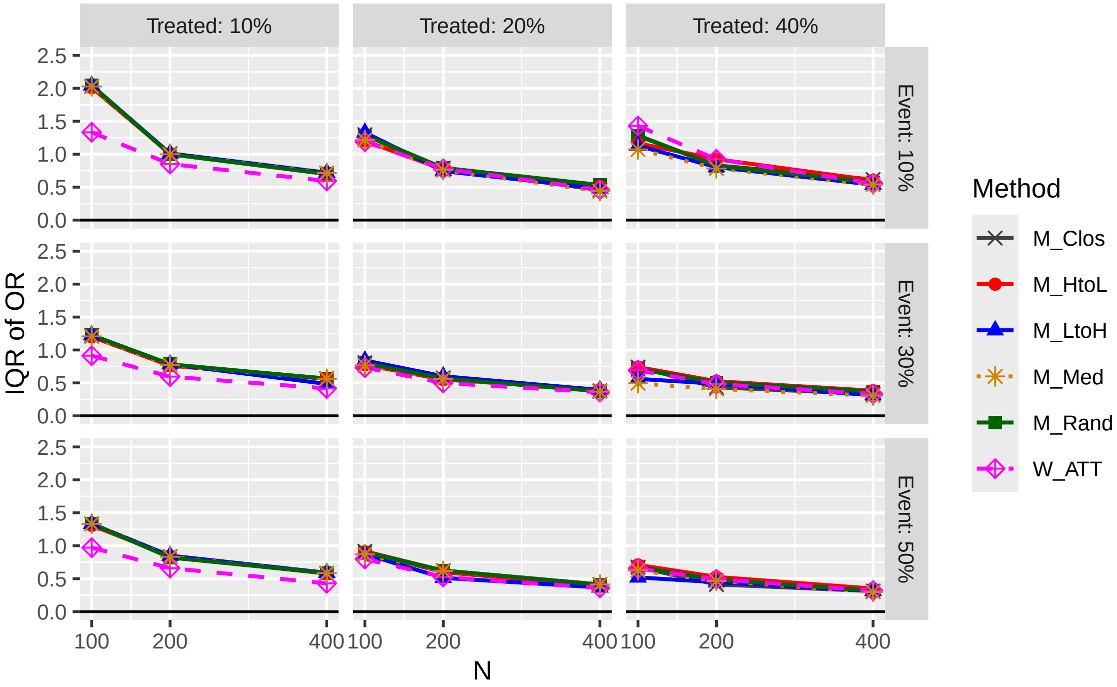

Figure S137. IQR for OR (multimodal continuous covariate, matching ratio 1:1, true OR: 0.75, c statistic: 0.85).

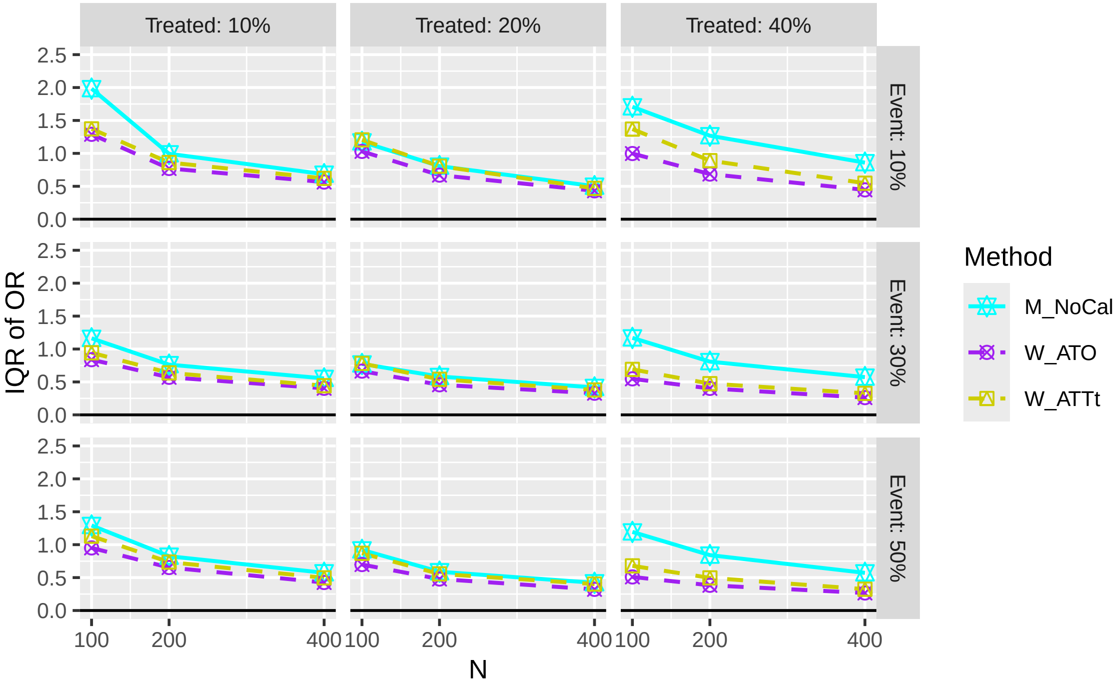

Figure S138. IQR for OR (multimodal continuous covariate, matching ratio 1:1, true OR: 0.75, c statistic: 0.85); other methods.

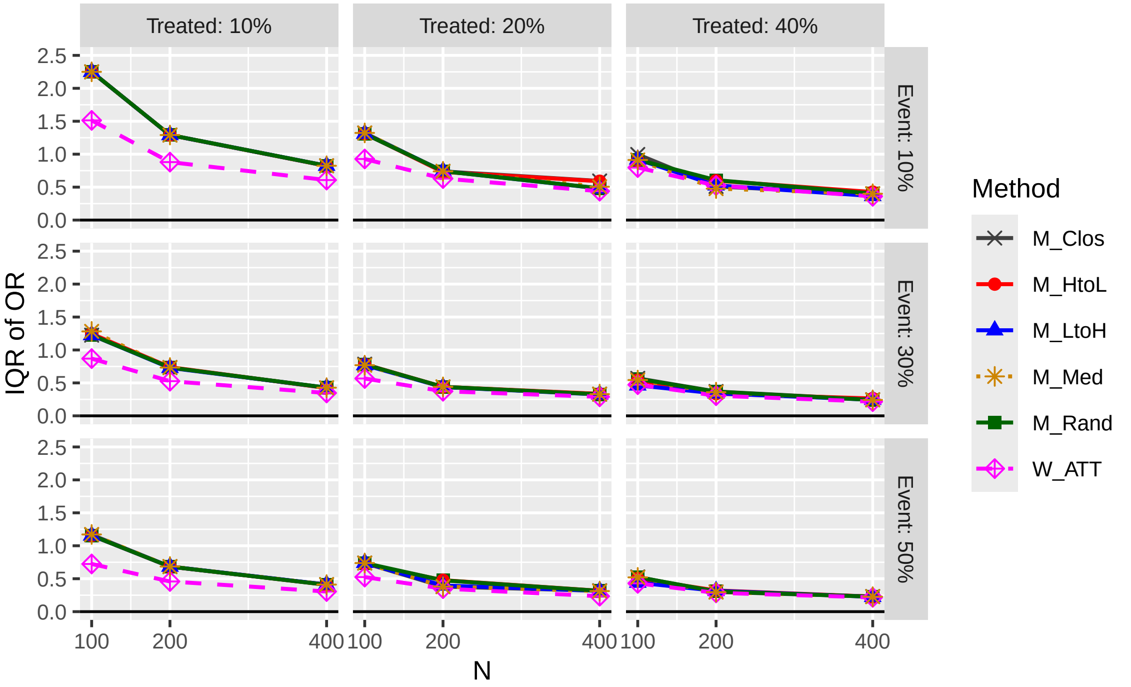

Figure S139. IQR for OR (multimodal continuous covariate, matching ratio 1:1, true OR: 0.75, c statistic: 0.6).

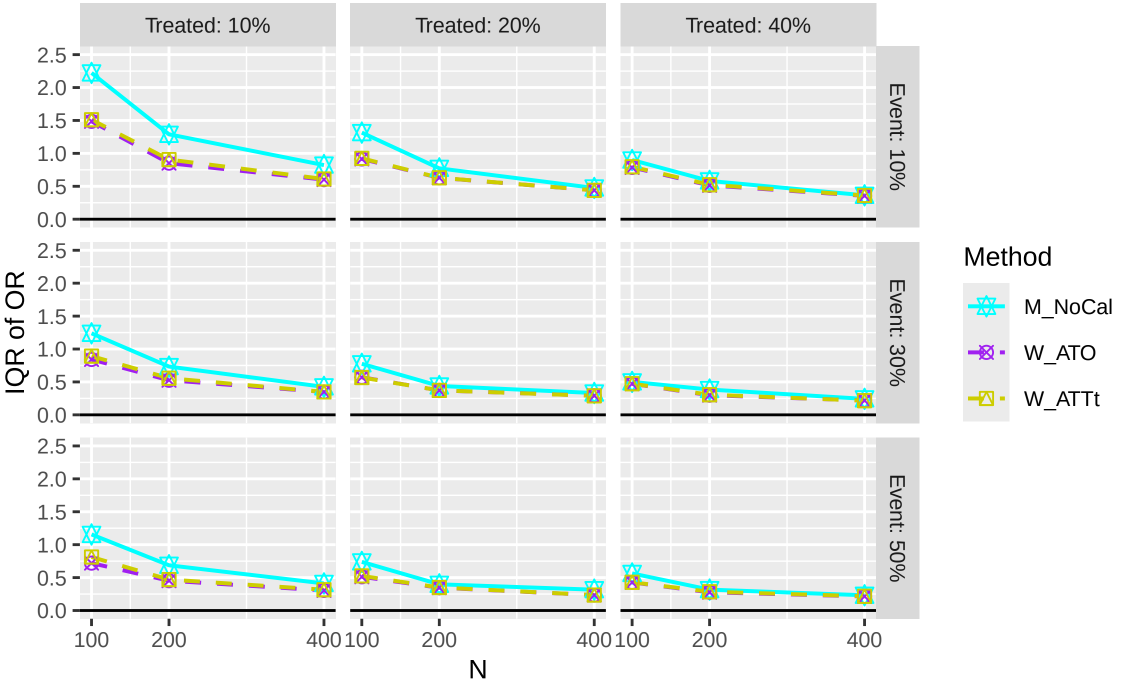

Figure S140. IQR for OR (multimodal continuous covariate, matching ratio 1:1, true OR: 0.75, c statistic: 0.6); other methods.

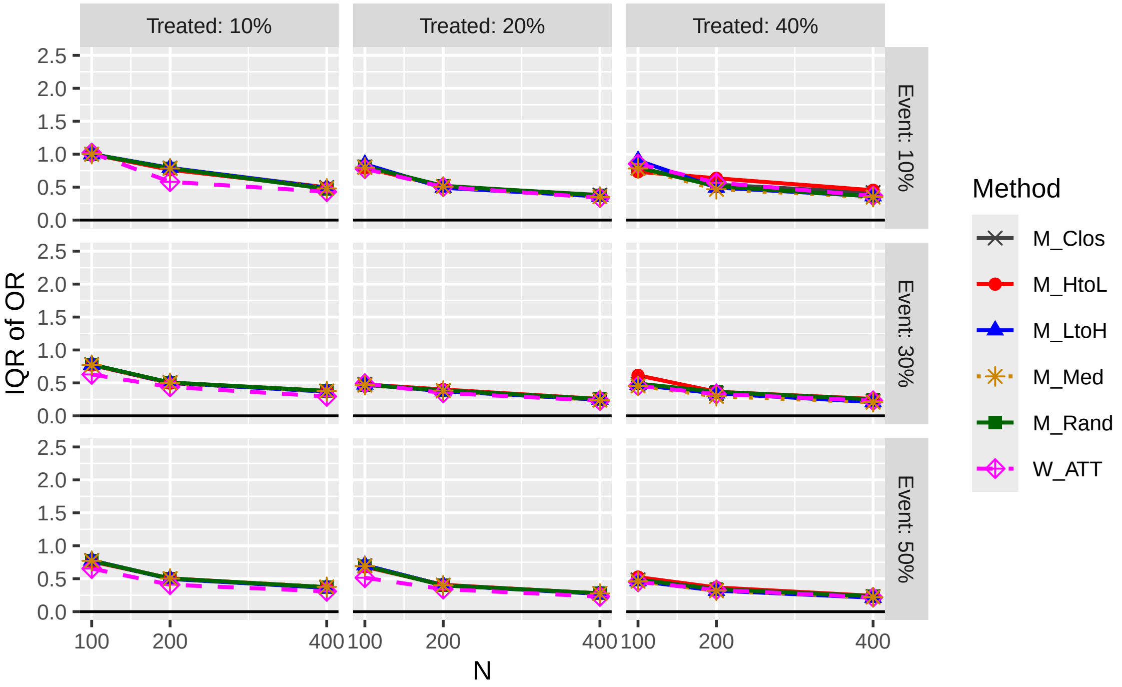

Figure S141. IQR for OR (multimodal continuous covariate, matching ratio 1:1, true OR: 0.5, c statistic: 0.85).

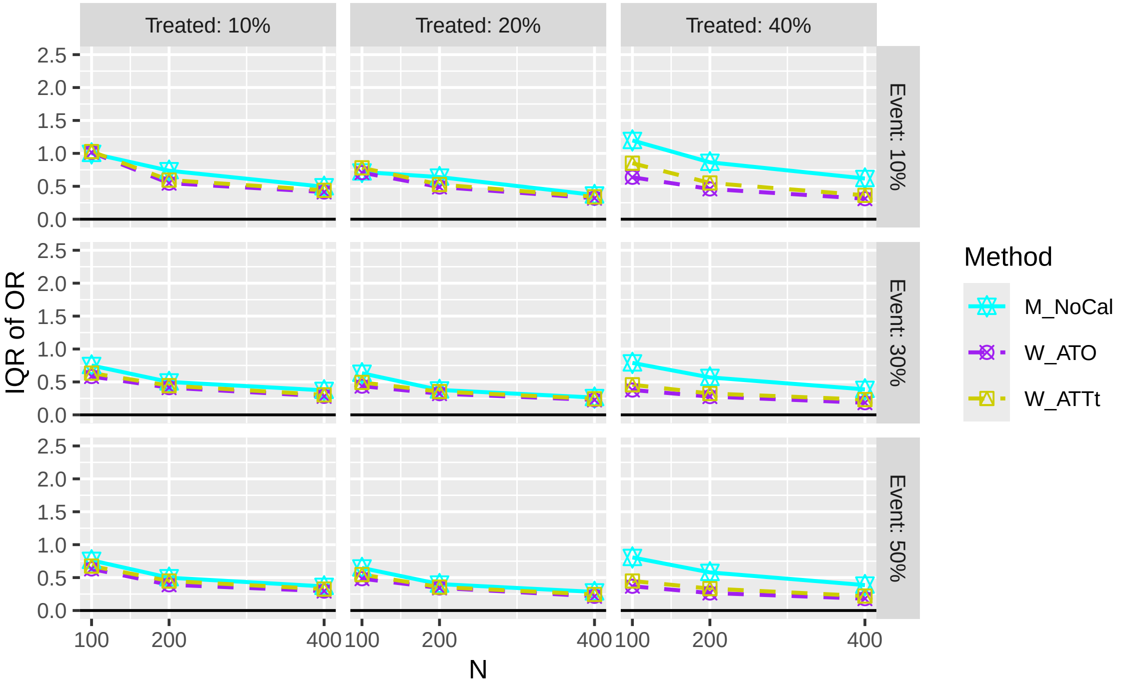

Figure S142. IQR for OR (multimodal continuous covariate, matching ratio 1:1, true OR: 0.5, c statistic: 0.85); other methods.

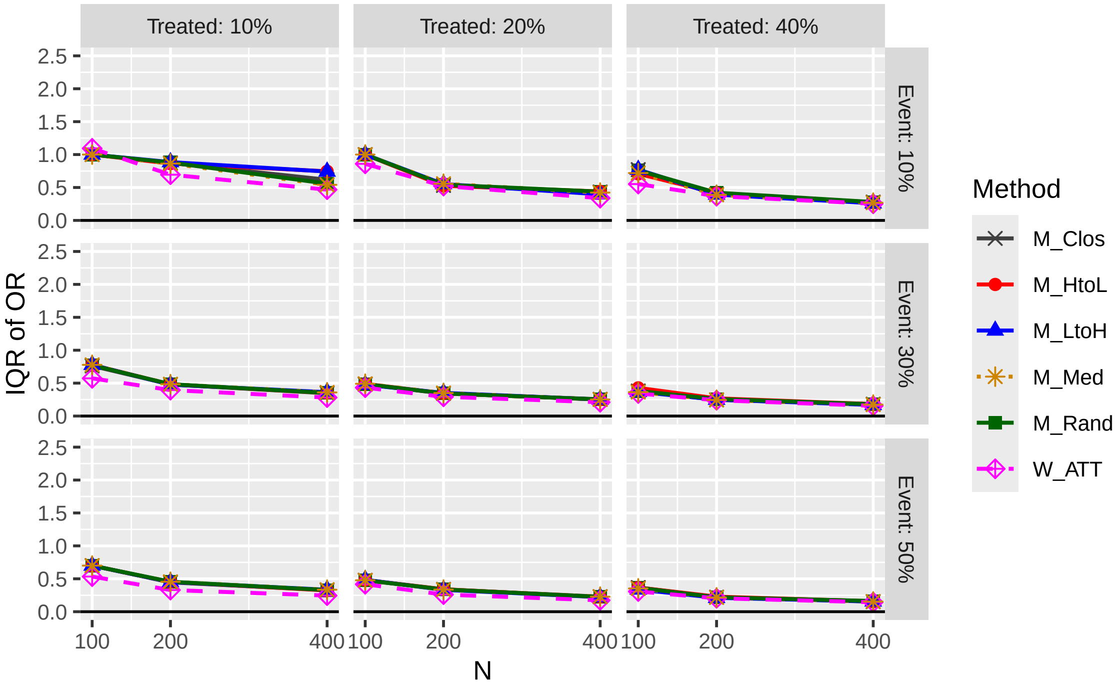

Figure S143. IQR for OR (multimodal continuous covariate, matching ratio 1:1, true OR: 0.5, c statistic: 0.6).

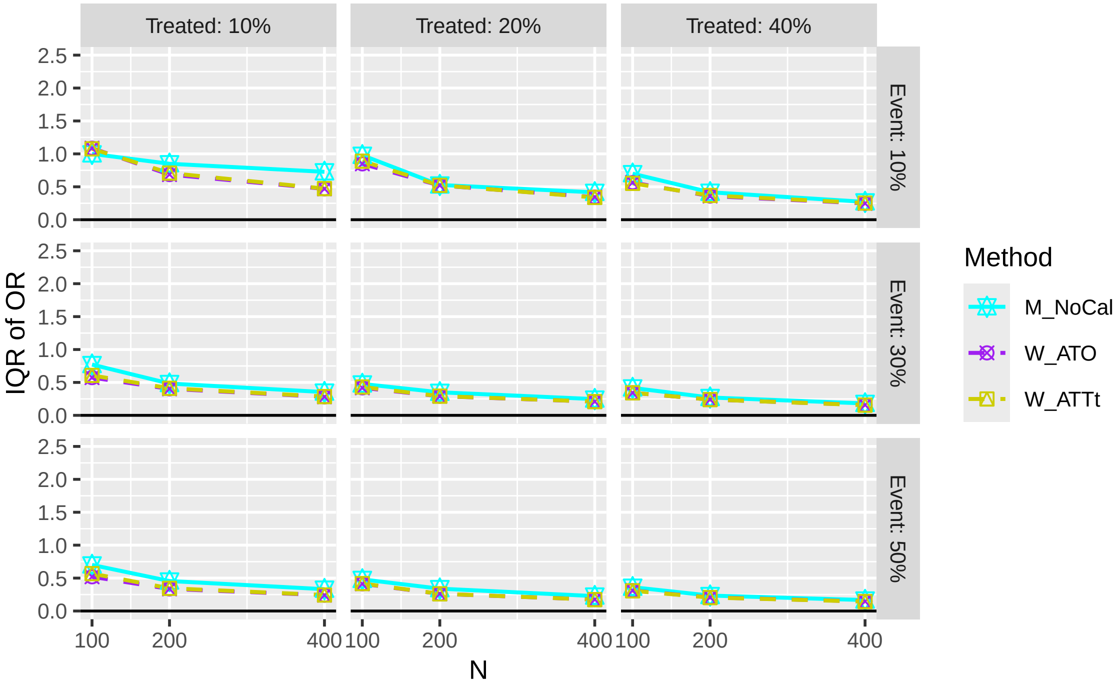

Figure S144. IQR for OR (multimodal continuous covariate, matching ratio 1:1, true OR: 0.5, c statistic: 0.6); other methods.

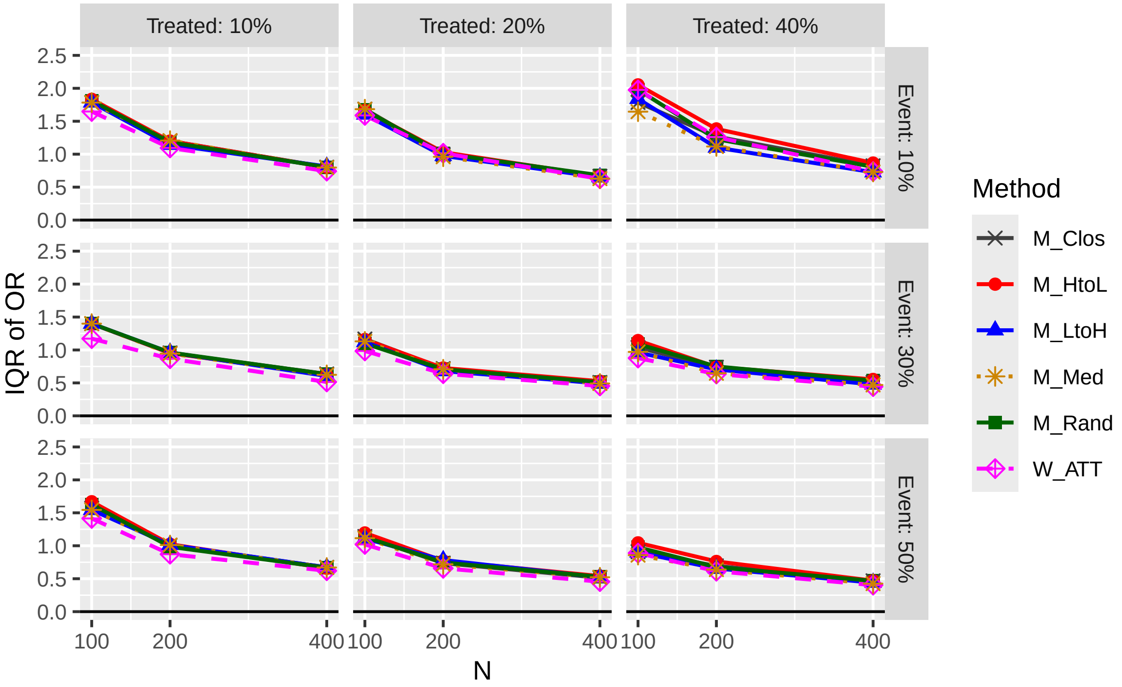

Figure S145. IQR for OR (multimodal continuous covariate, matching ratio 1:2, true OR: 1, c statistic: 0.85).

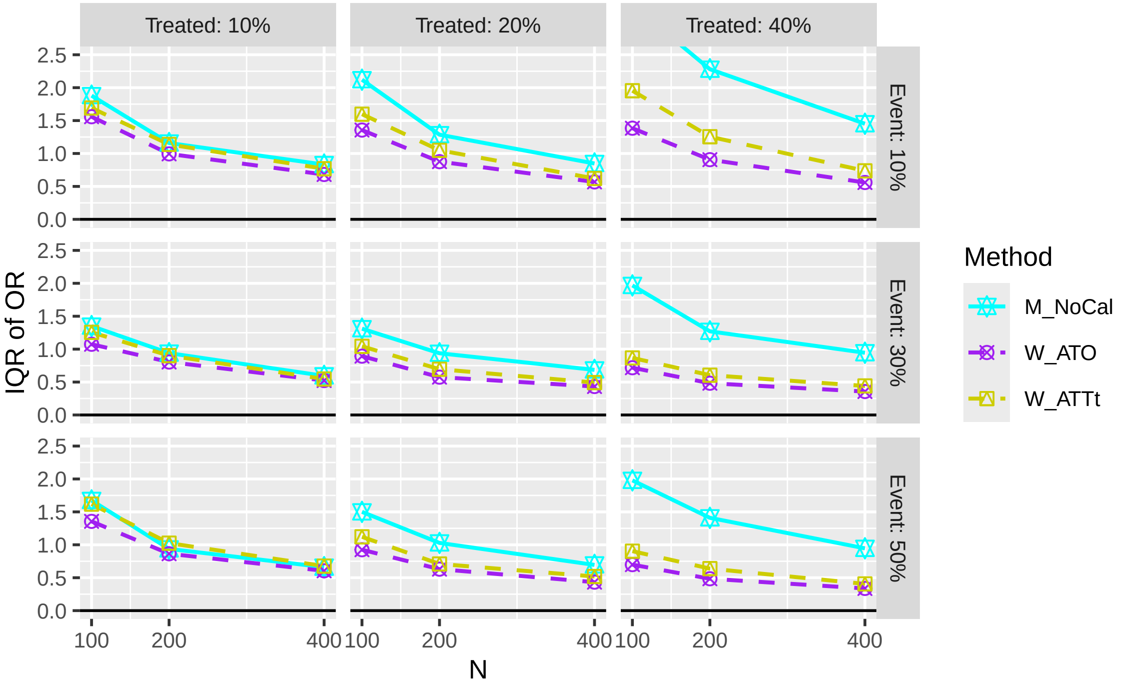

Figure S146. IQR for OR (multimodal continuous covariate, matching ratio 1:2, true OR: 1, c statistic: 0.85); other methods.

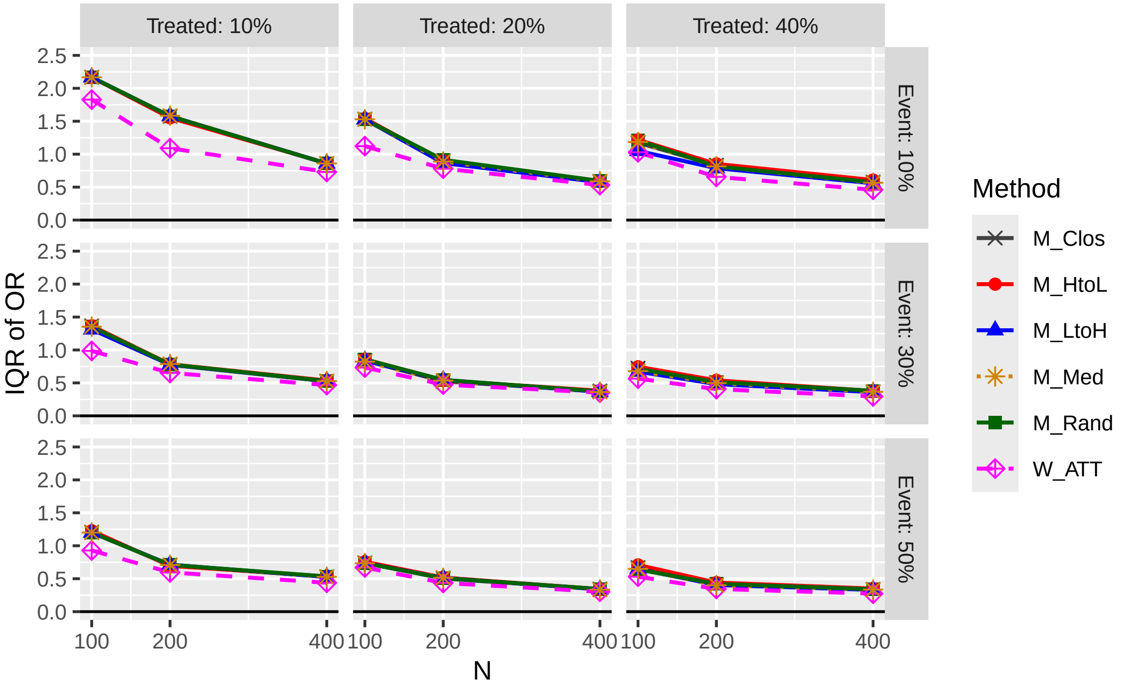

Figure S147. IQR for OR (multimodal continuous covariate, matching ratio 1:2, true OR: 1, c statistic: 0.6).

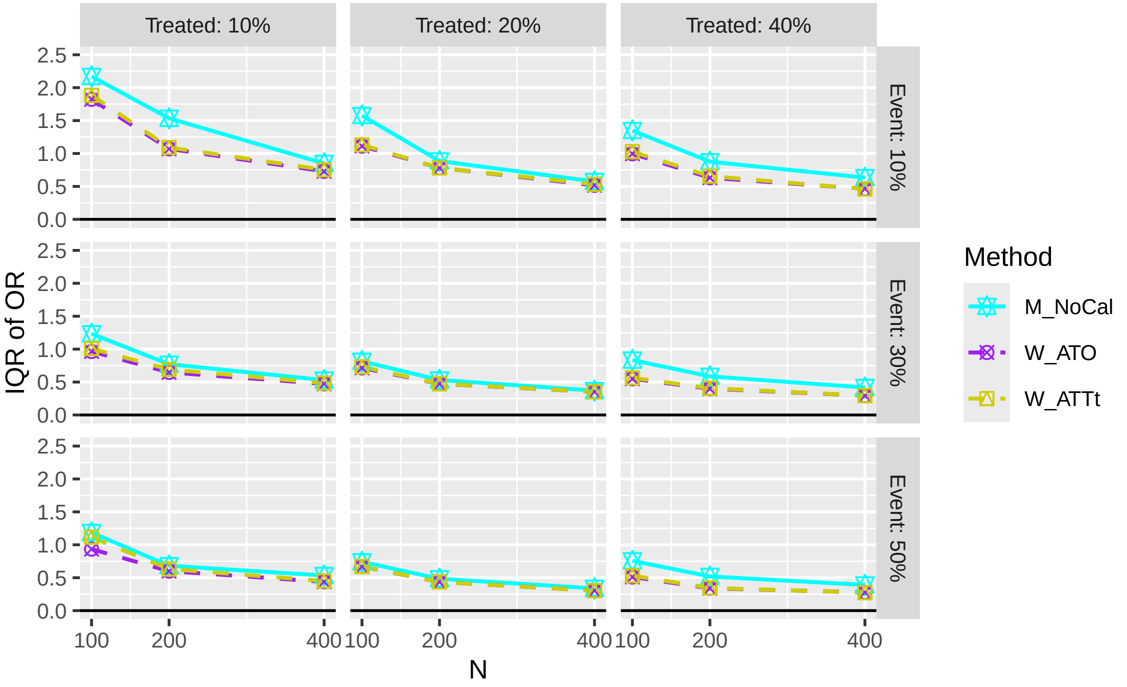

Figure S148. IQR for OR (multimodal continuous covariate, matching ratio 1:2, true OR: 1, c statistic: 0.6); other methods.

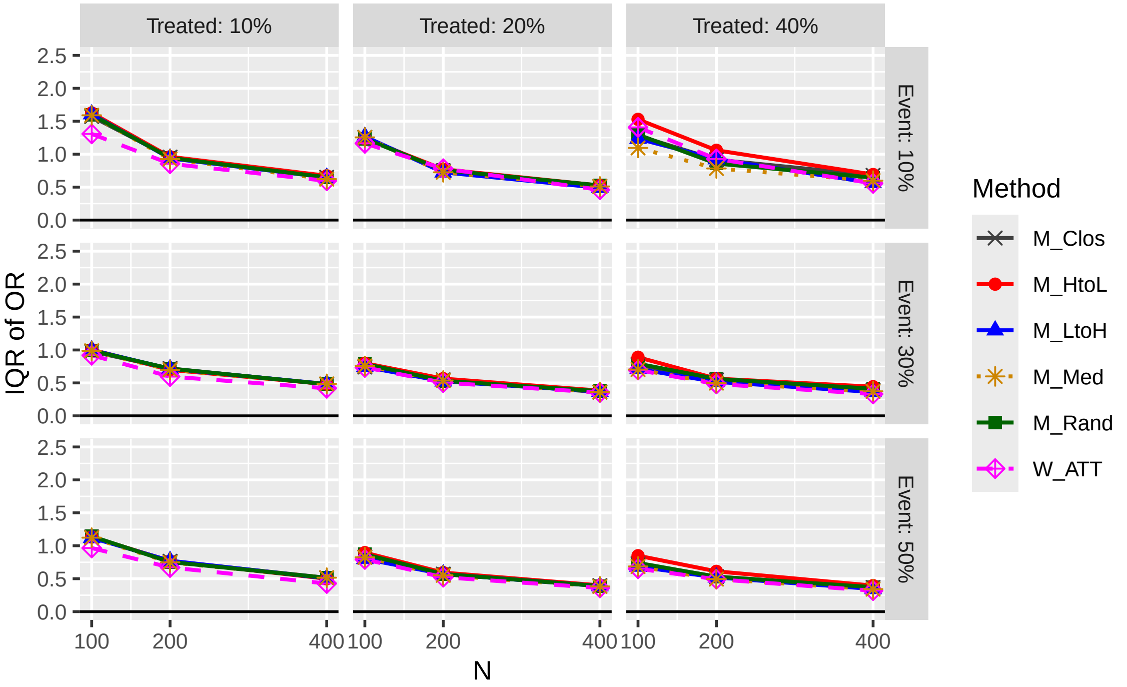

Figure S149. IQR for OR (multimodal continuous covariate, matching ratio 1:2, true OR: 0.75, c statistic: 0.85).

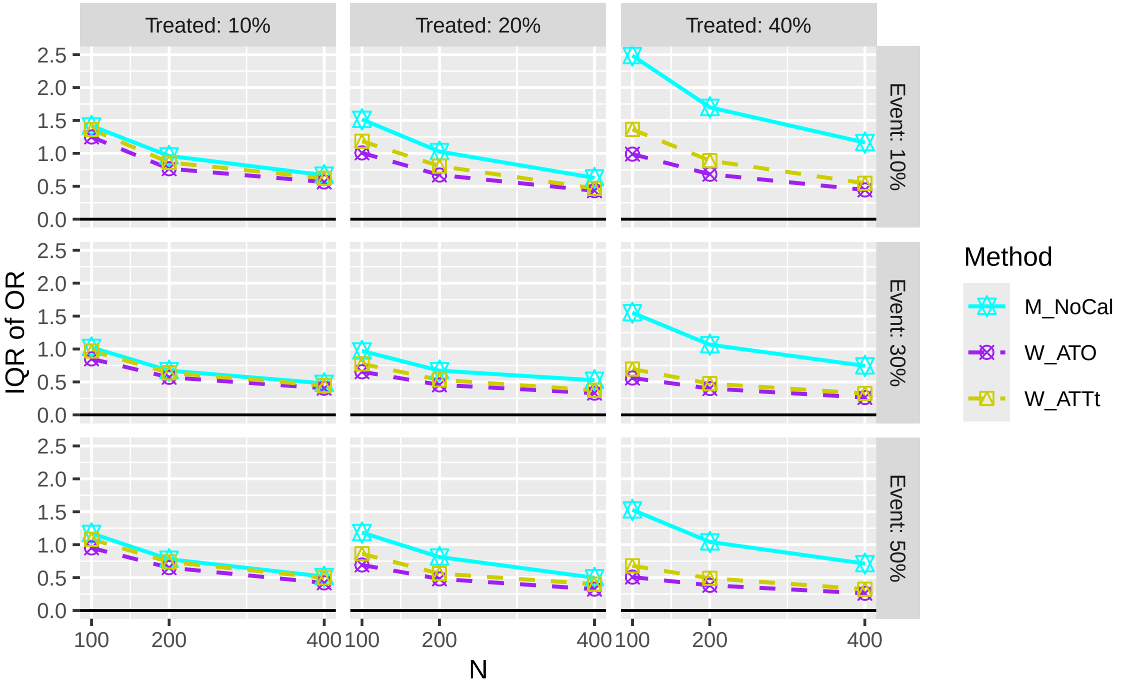

Figure S150. IQR for OR (multimodal continuous covariate, matching ratio 1:2, true OR: 0.75, c statistic: 0.85); other methods.

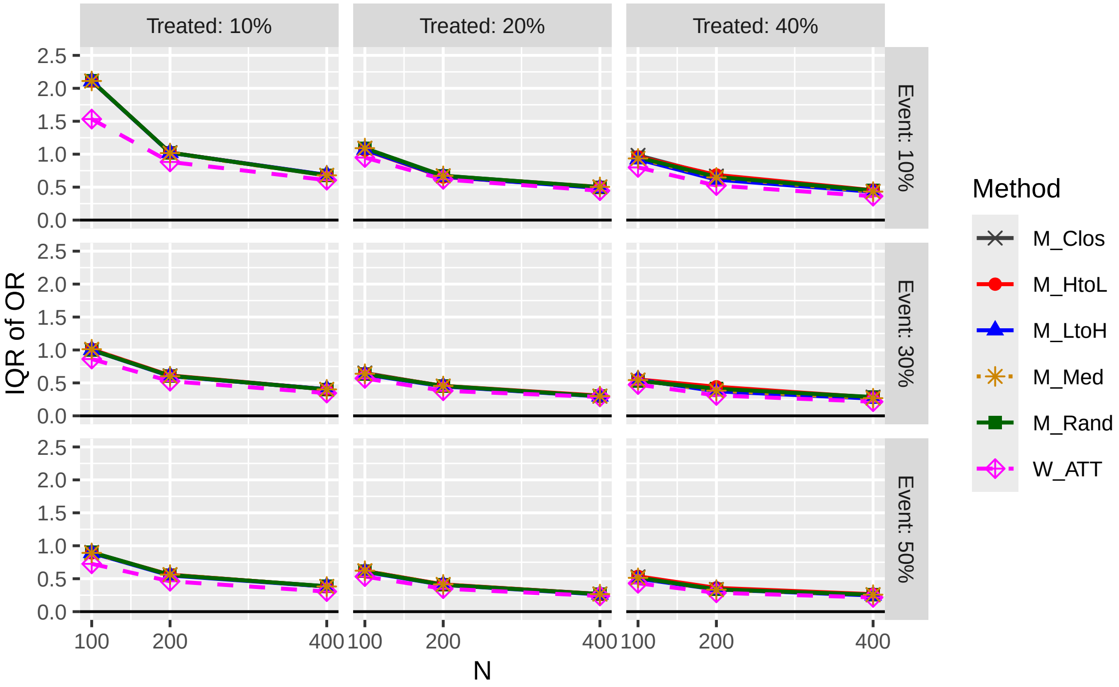

Figure S151. IQR for OR (multimodal continuous covariate, matching ratio 1:2, true OR: 0.75, c statistic: 0.6).

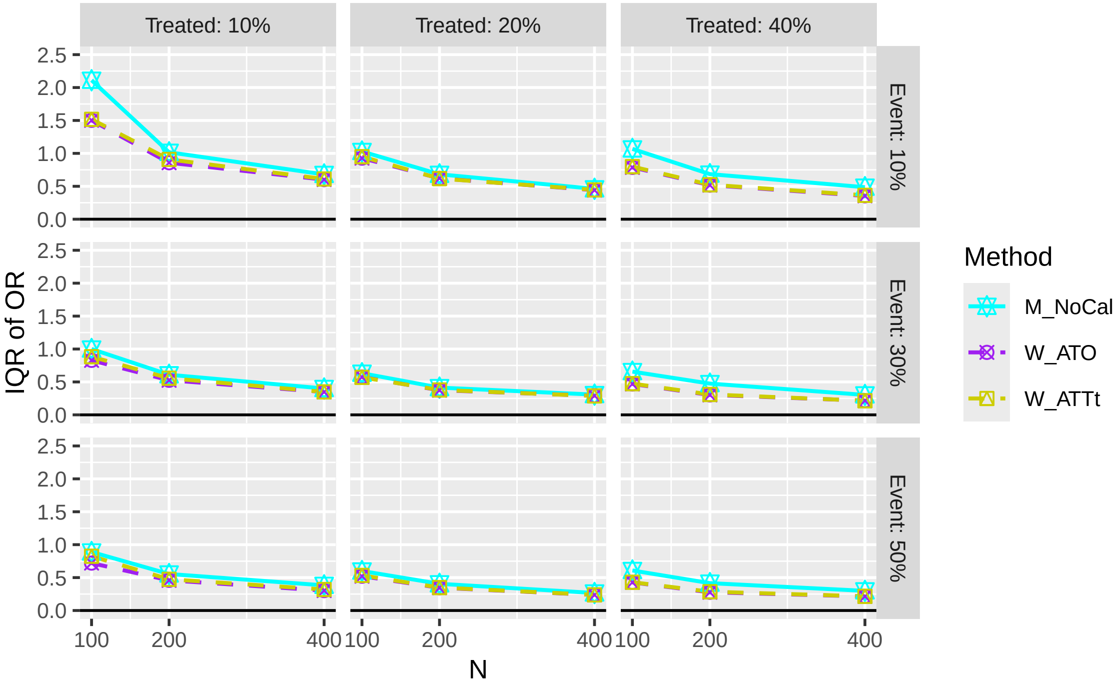

Figure S152. IQR for OR (multimodal continuous covariate, matching ratio 1:2, true OR: 0.75, c statistic: 0.6); other methods.

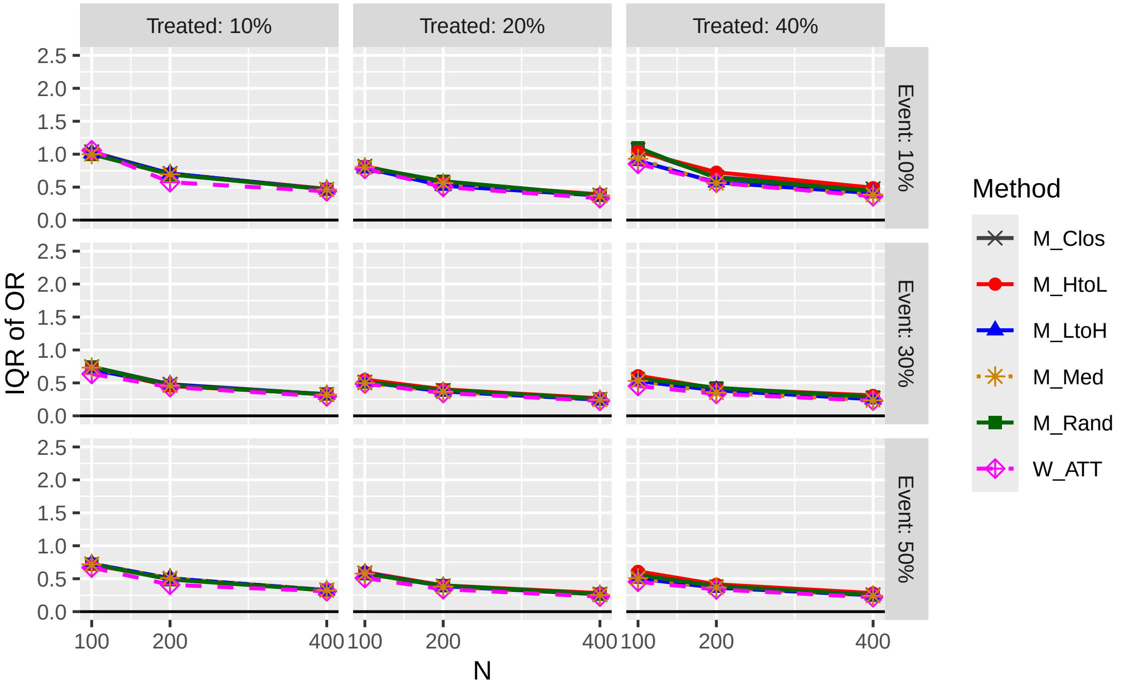

Figure S153. IQR for OR (multimodal continuous covariate, matching ratio 1:2, true OR: 0.5, c statistic: 0.85).

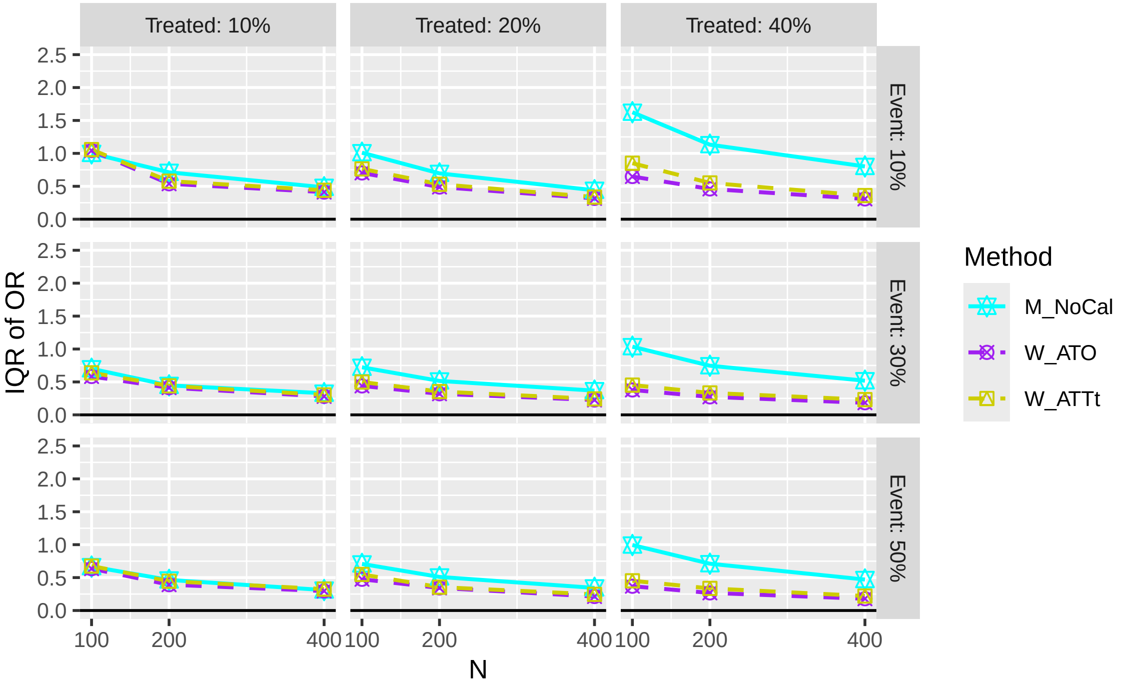

Figure S154. IQR for OR (multimodal continuous covariate, matching ratio 1:2, true OR: 0.5, c statistic: 0.85); other methods.

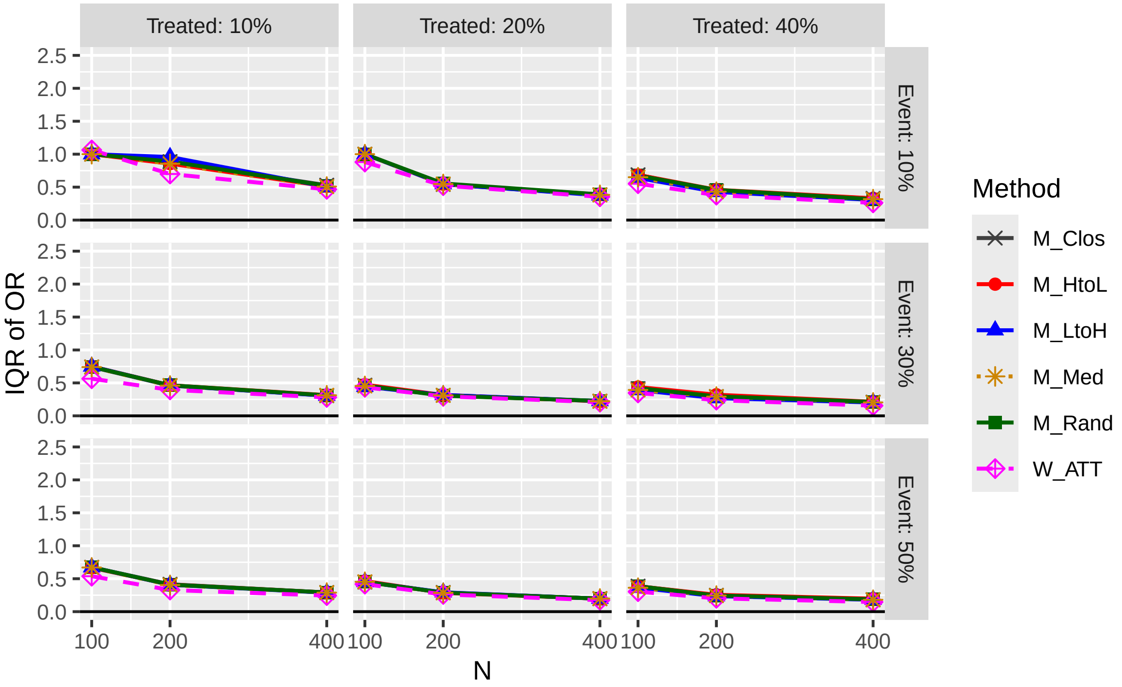

Figure S155. IQR for OR (multimodal continuous covariate, matching ratio 1:2, true OR: 0.5, c statistic: 0.6).

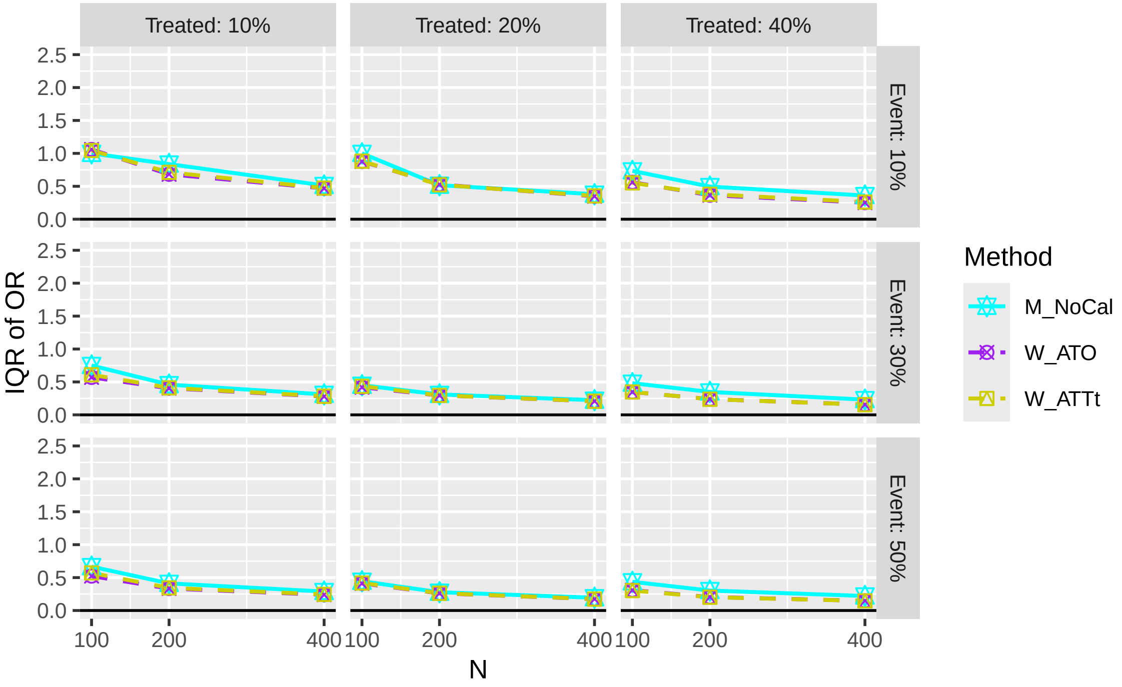

Figure S156. IQR for OR (multimodal continuous covariate, matching ratio 1:2, true OR: 0.5, c statistic: 0.6); other methods.

S4. Median absolute difference of OR for 10% data addition (caliper: 25%)

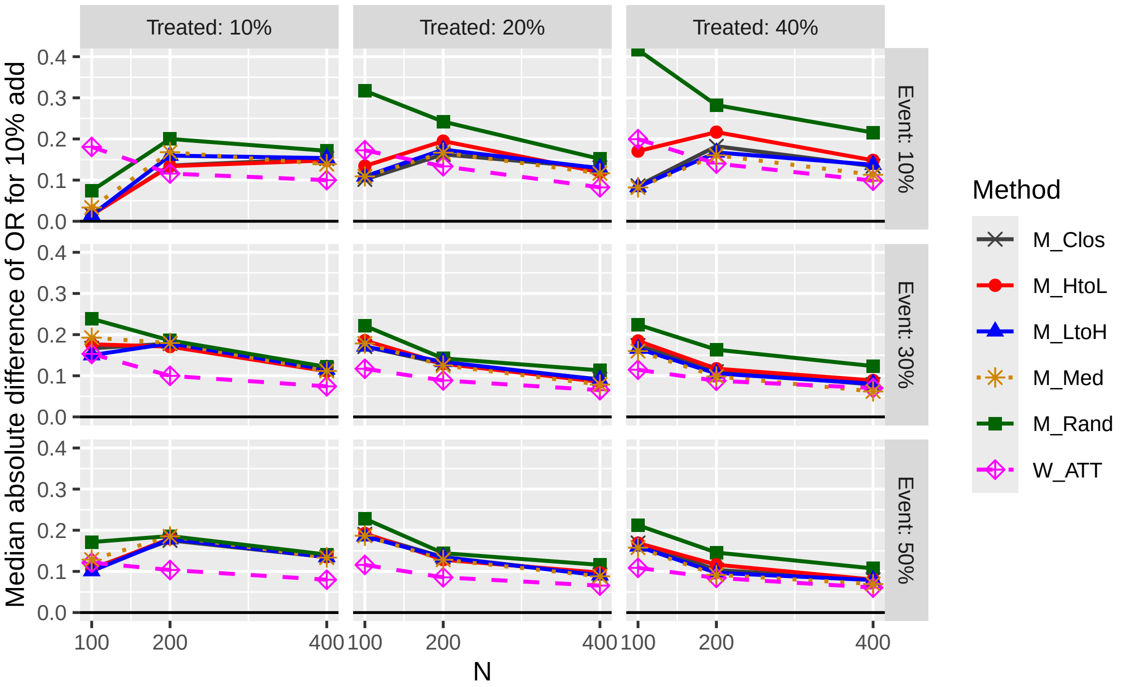

Figure S157. Median absolute difference of OR for 10% data addition (unimodal continuous covariate, matching ratio 1:1, true OR: 1, c statistic: 0.85).

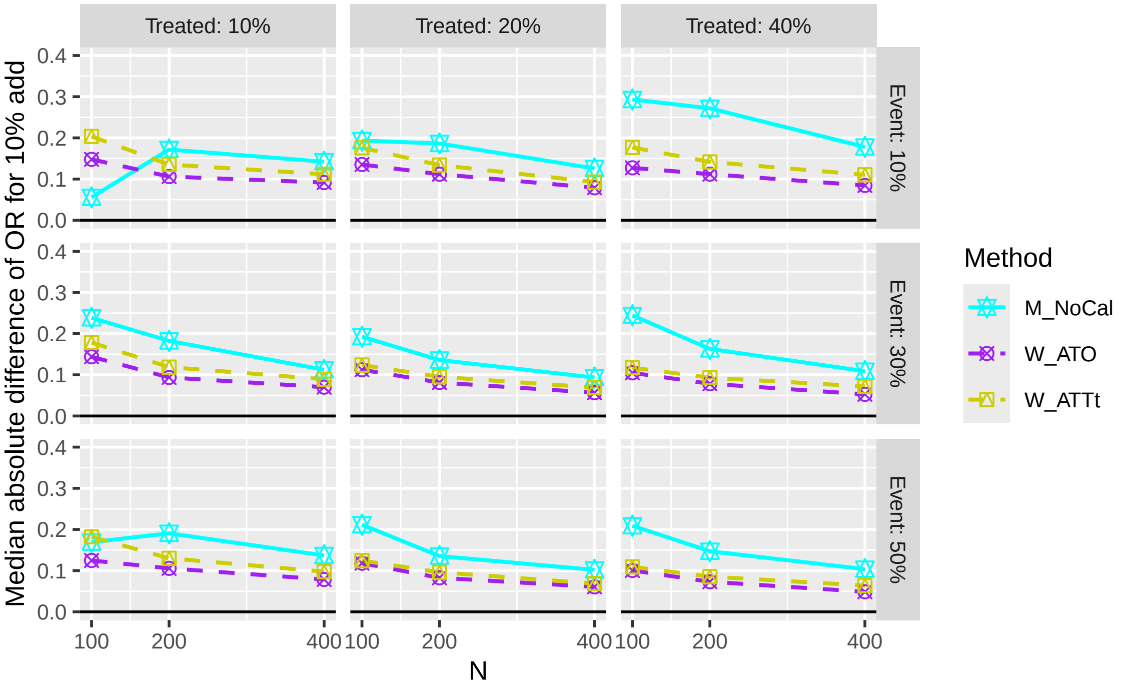

Figure S158. Median absolute difference of OR for 10% data addition (unimodal continuous covariate, matching ratio 1:1, true OR: 1, c statistic: 0.85); other methods.

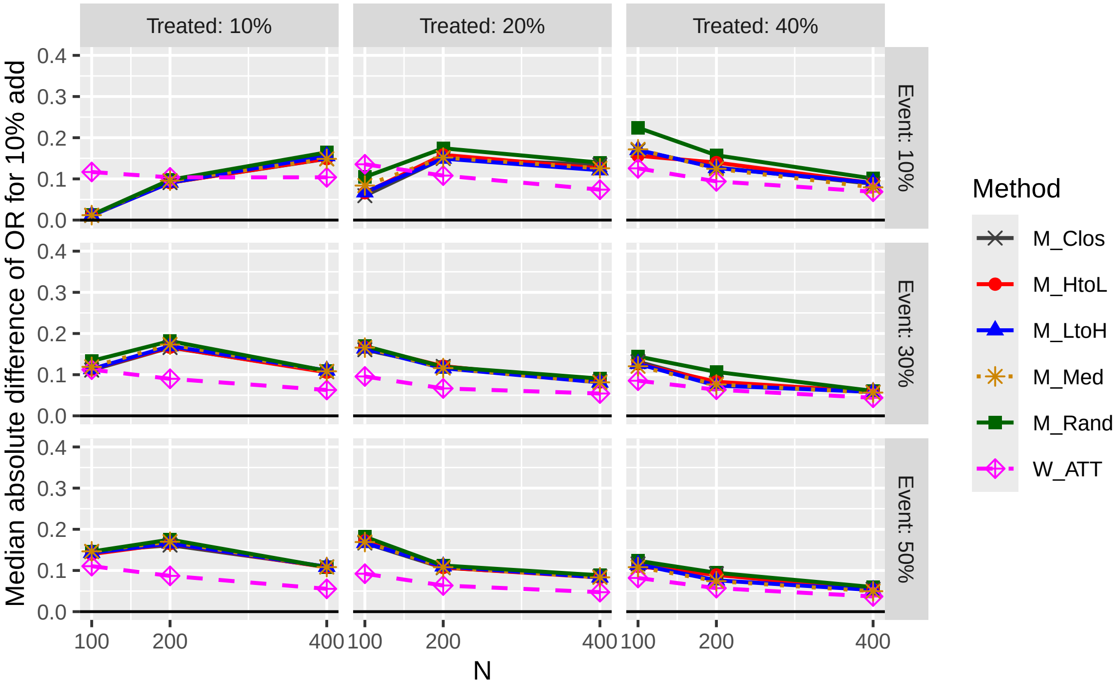

Figure S159. Median absolute difference of OR for 10% data addition (unimodal continuous covariate, matching ratio 1:1, true OR: 1, c statistic: 0.6).

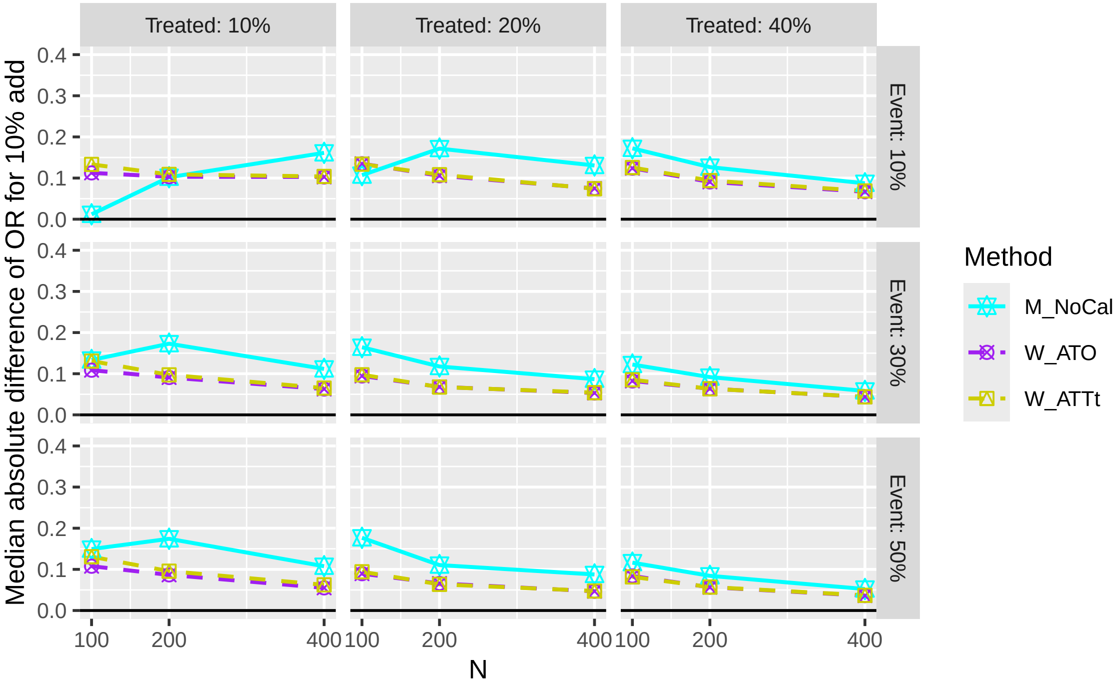

Figure S160. Median absolute difference of OR for 10% data addition (unimodal continuous covariate, matching ratio 1:1, true OR: 1, c statistic: 0.6); other methods.

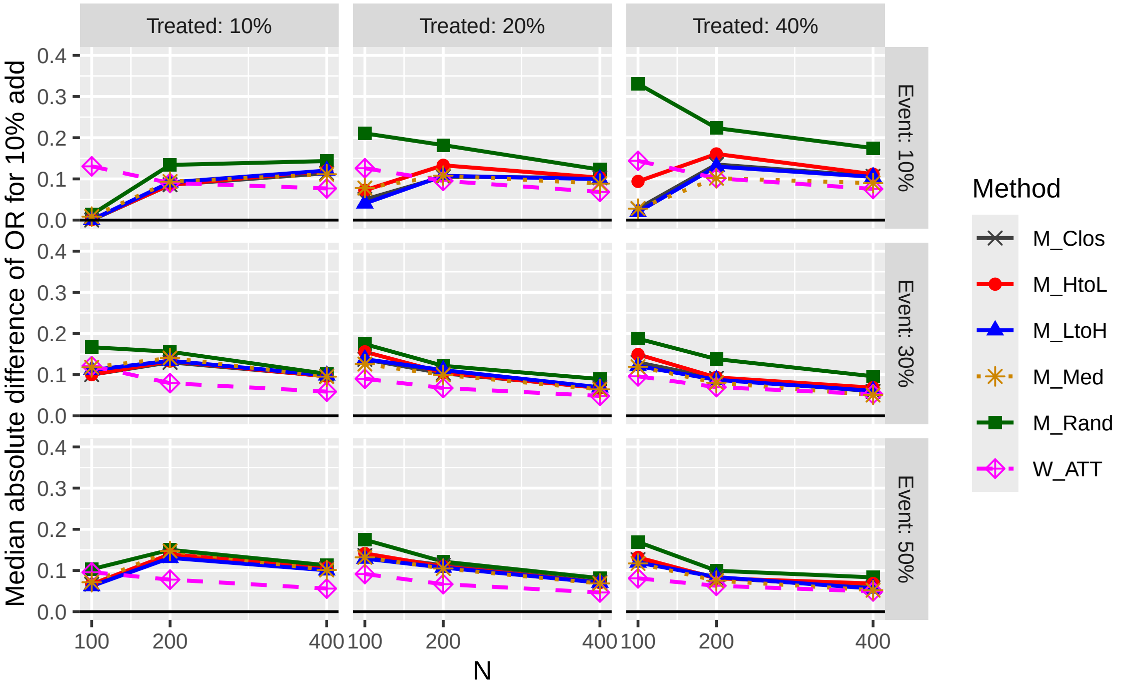

Figure S161. Median absolute difference of OR for 10% data addition (unimodal continuous covariate, matching ratio 1:1, true OR: 0.75, c statistic: 0.85).

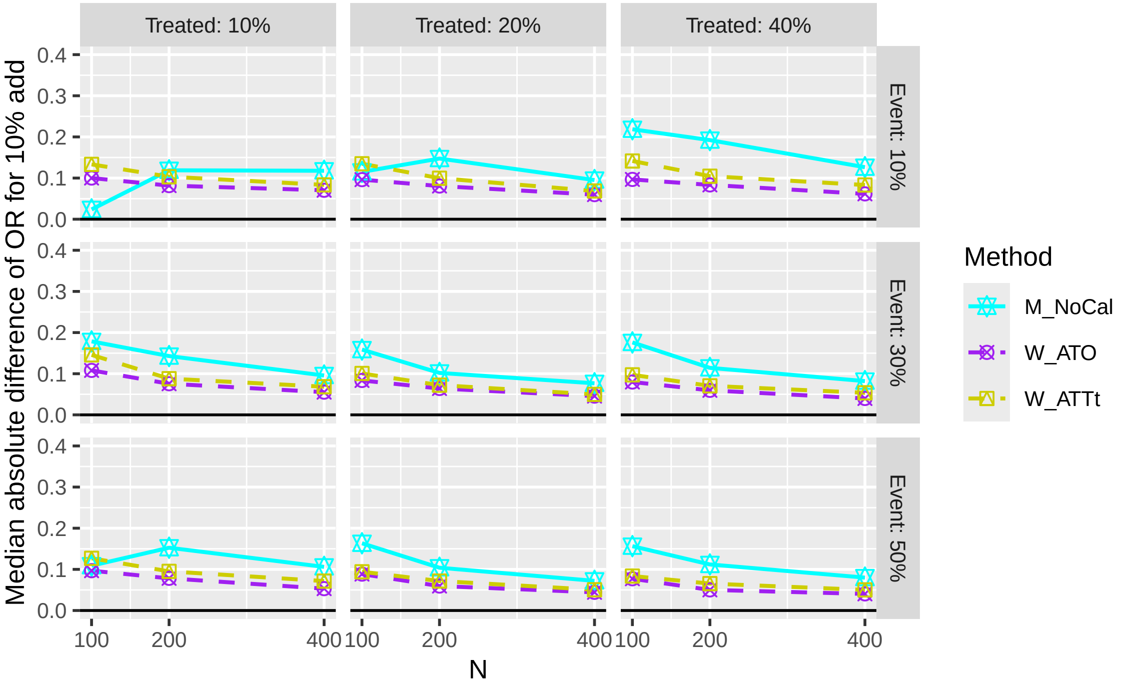

Figure S162. Median absolute difference of OR for 10% data addition (unimodal continuous covariate, matching ratio 1:1, true OR: 0.75, c statistic: 0.85); other methods.

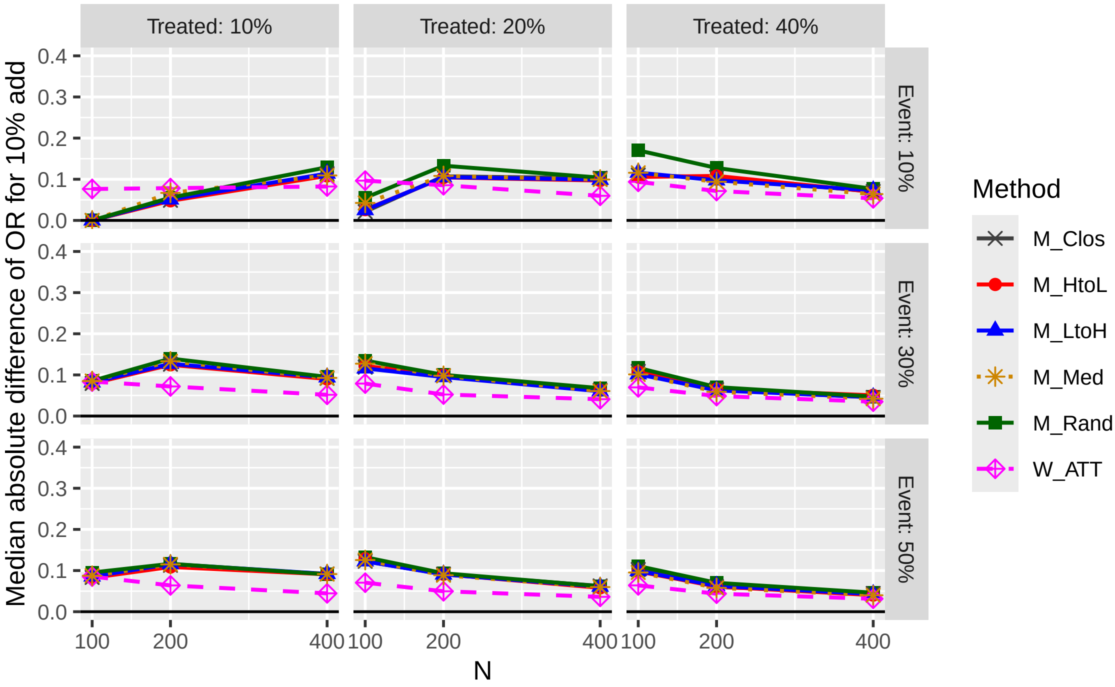

Figure S163. Median absolute difference of OR for 10% data addition (unimodal continuous covariate, matching ratio 1:1, true OR: 0.75, c statistic: 0.6).

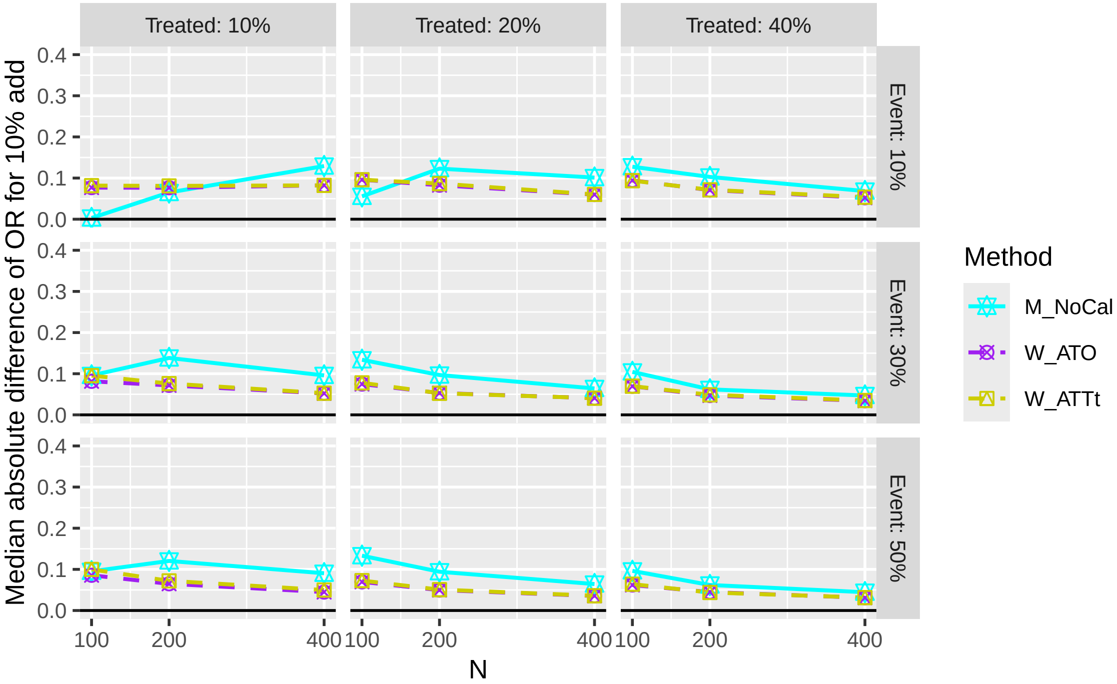

Figure S164. Median absolute difference of OR for 10% data addition (unimodal continuous covariate, matching ratio 1:1, true OR: 0.75, c statistic: 0.6); other methods.

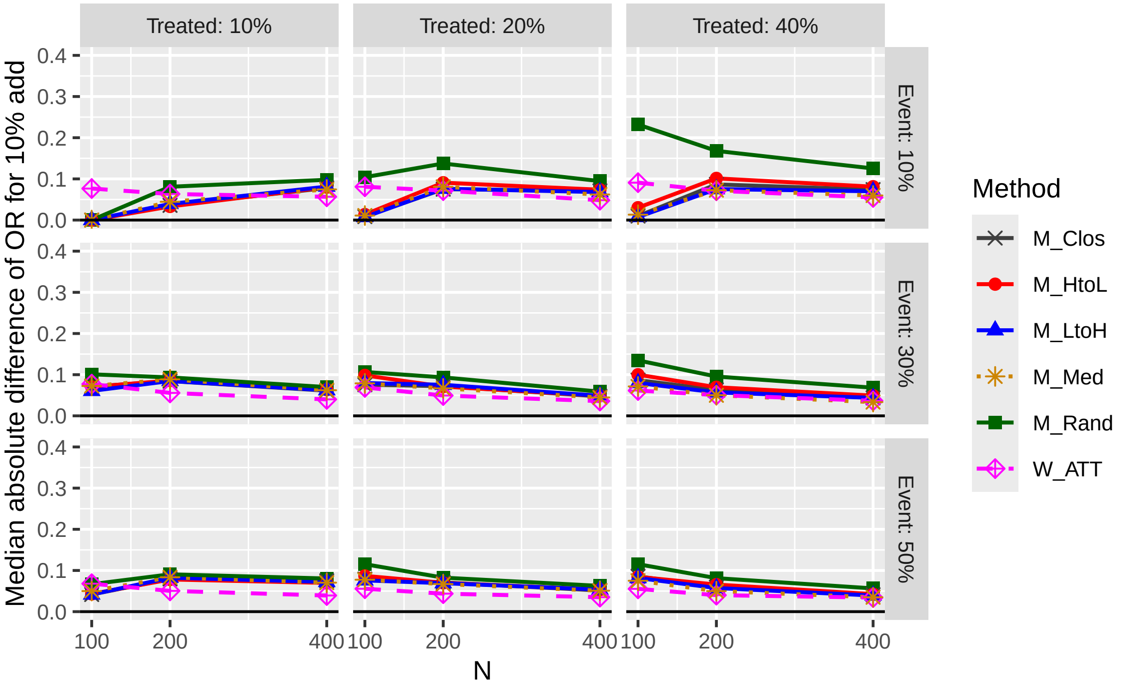

Figure S165. Median absolute difference of OR for 10% data addition (unimodal continuous covariate, matching ratio 1:1, true OR: 0.5, c statistic: 0.85).

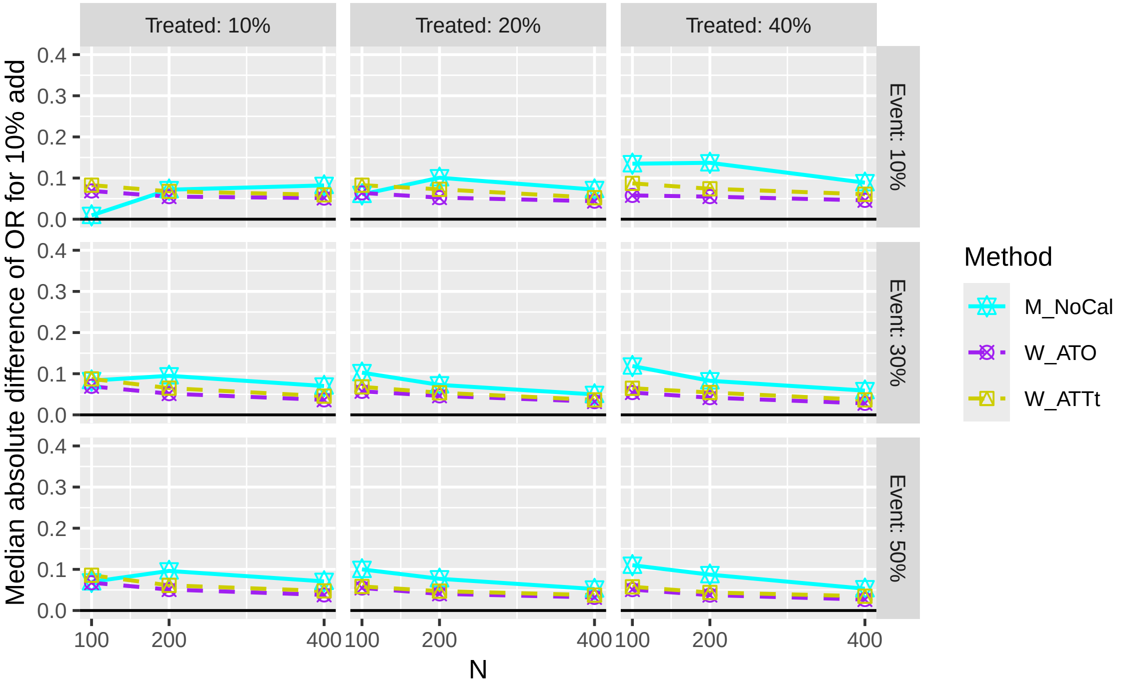

Figure S166. Median absolute difference of OR for 10% data addition (unimodal continuous covariate, matching ratio 1:1, true OR: 0.5, c statistic: 0.85); other methods.

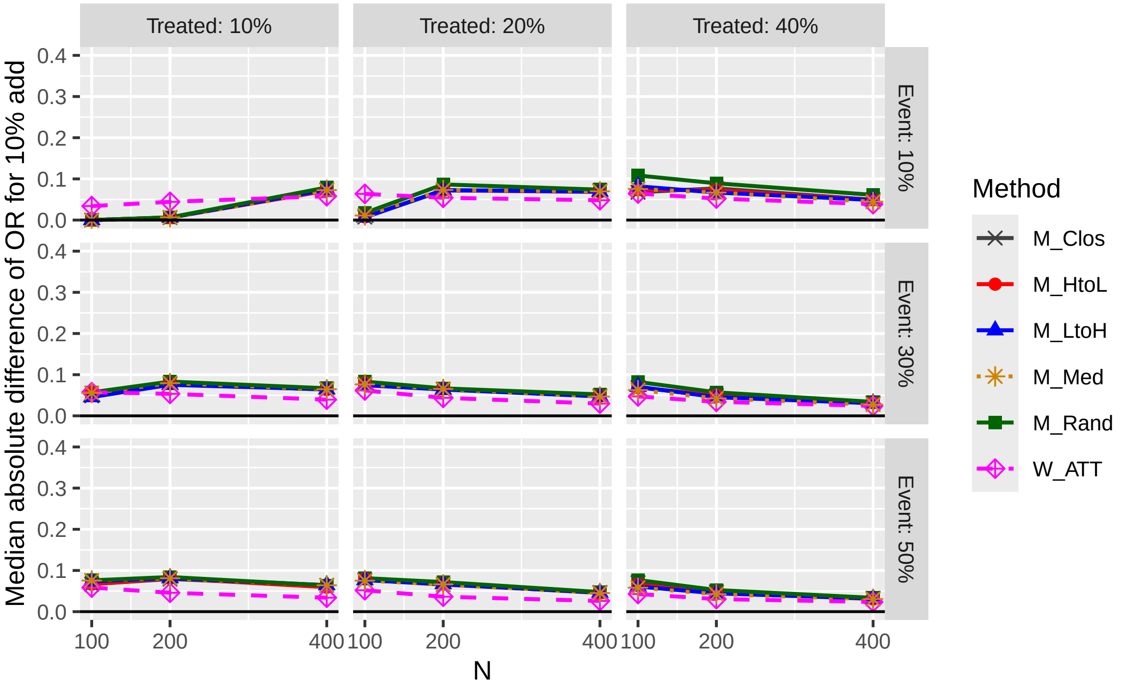

Figure S167. Median absolute difference of OR for 10% data addition (unimodal continuous covariate, matching ratio 1:1, true OR: 0.5, c statistic: 0.6).

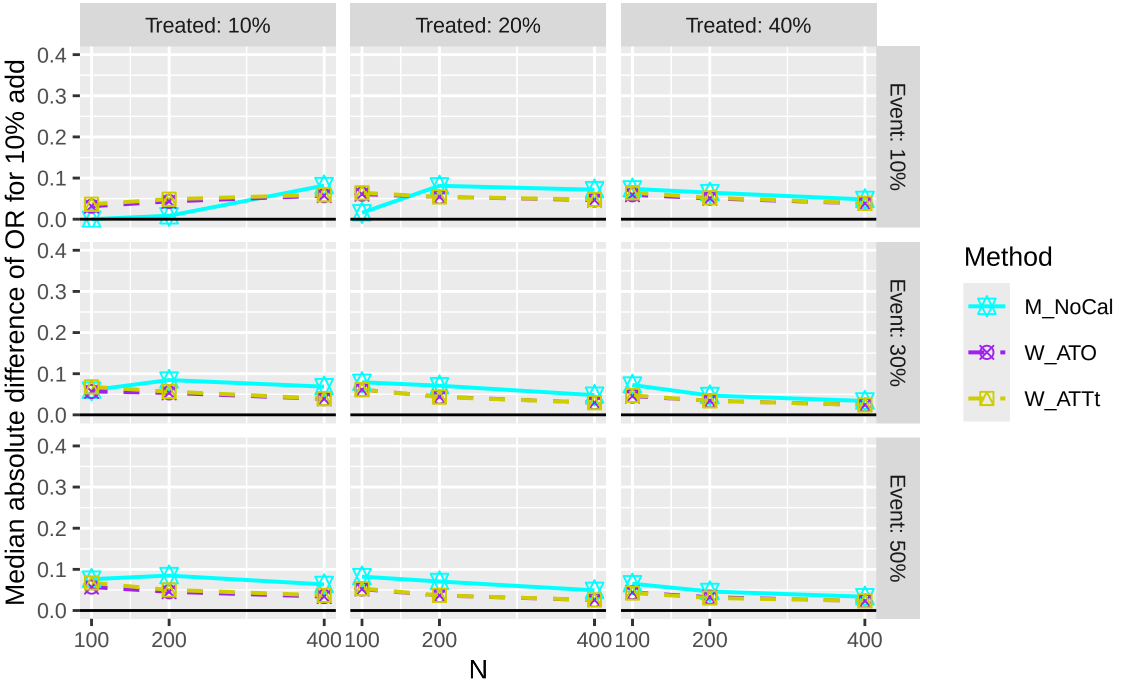

Figure S168. Median absolute difference of OR for 10% data addition (unimodal continuous covariate, matching ratio 1:1, true OR: 0.5, c statistic: 0.6); other methods.

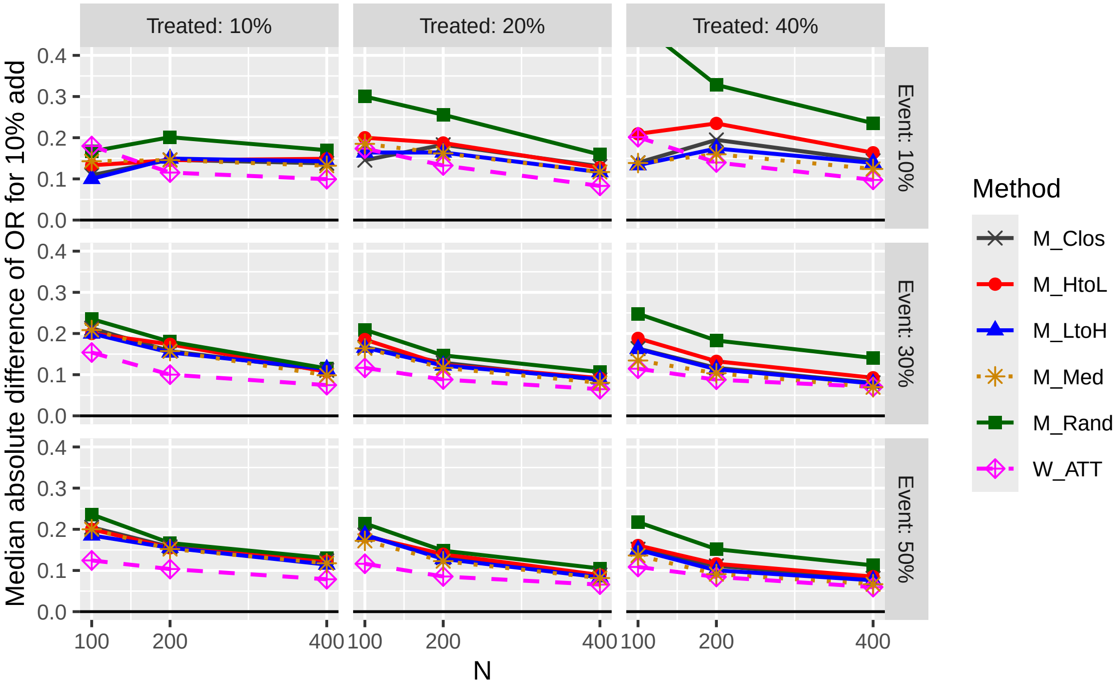

Figure S169. Median absolute difference of OR for 10% data addition (unimodal continuous covariate, matching ratio 1:2, true OR: 1, c statistic: 0.85).

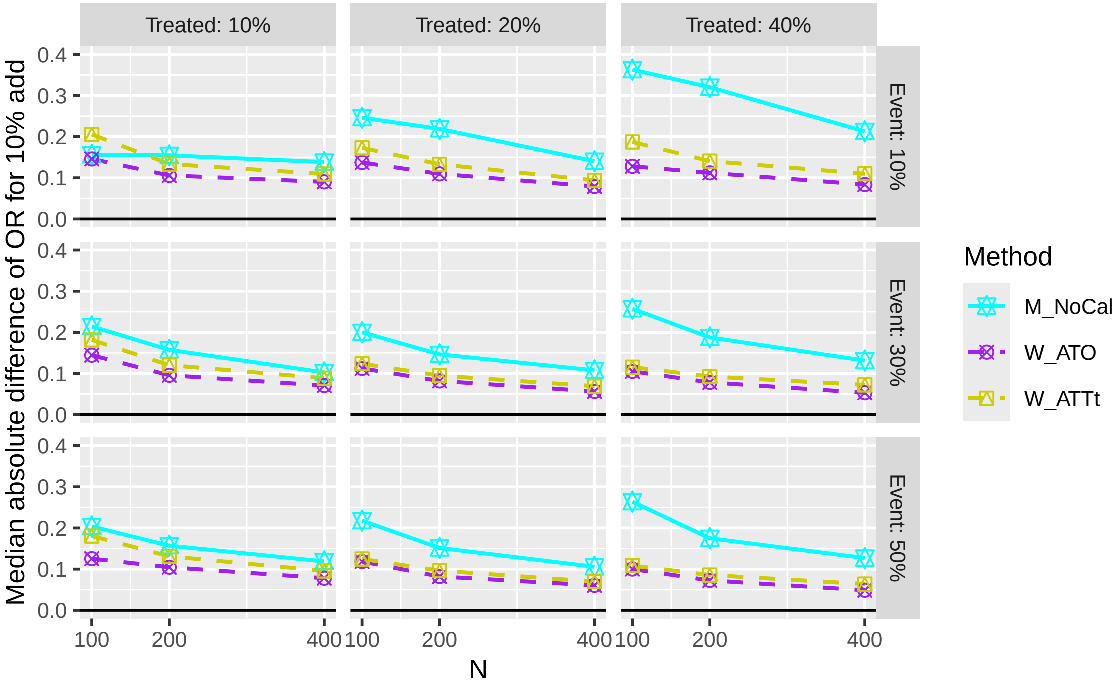

Figure S170. Median absolute difference of OR for 10% data addition (unimodal continuous covariate, matching ratio 1:2, true OR: 1, c statistic: 0.85); other methods.

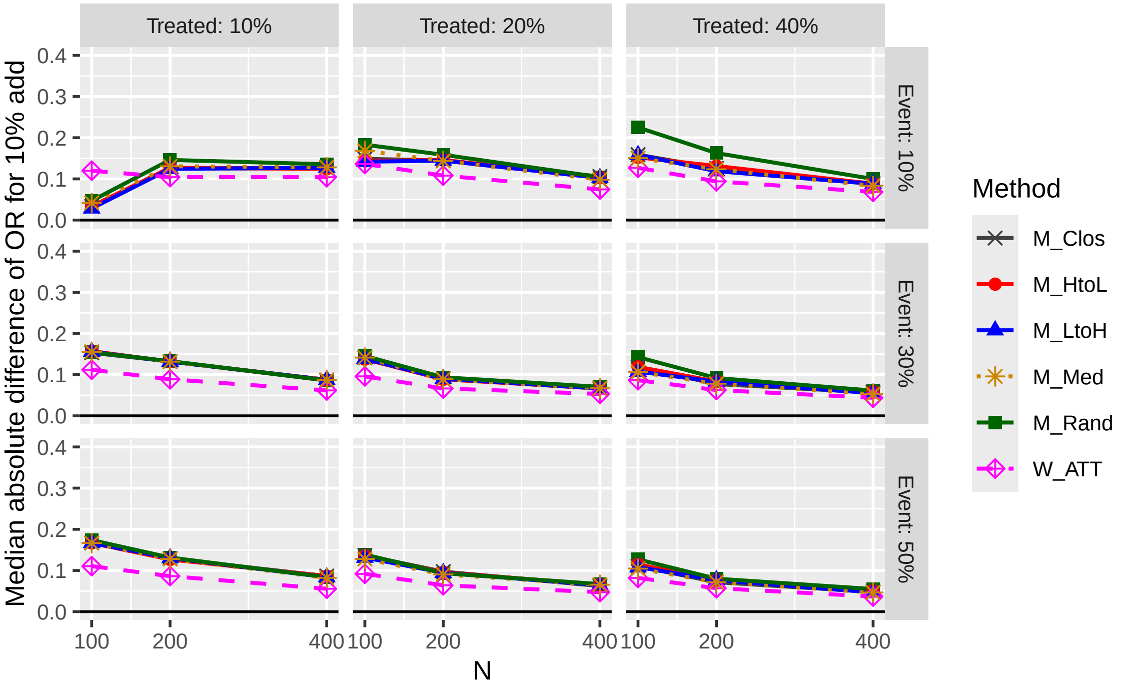

Figure S171. Median absolute difference of OR for 10% data addition (unimodal continuous covariate, matching ratio 1:2, true OR: 1, c statistic: 0.6).

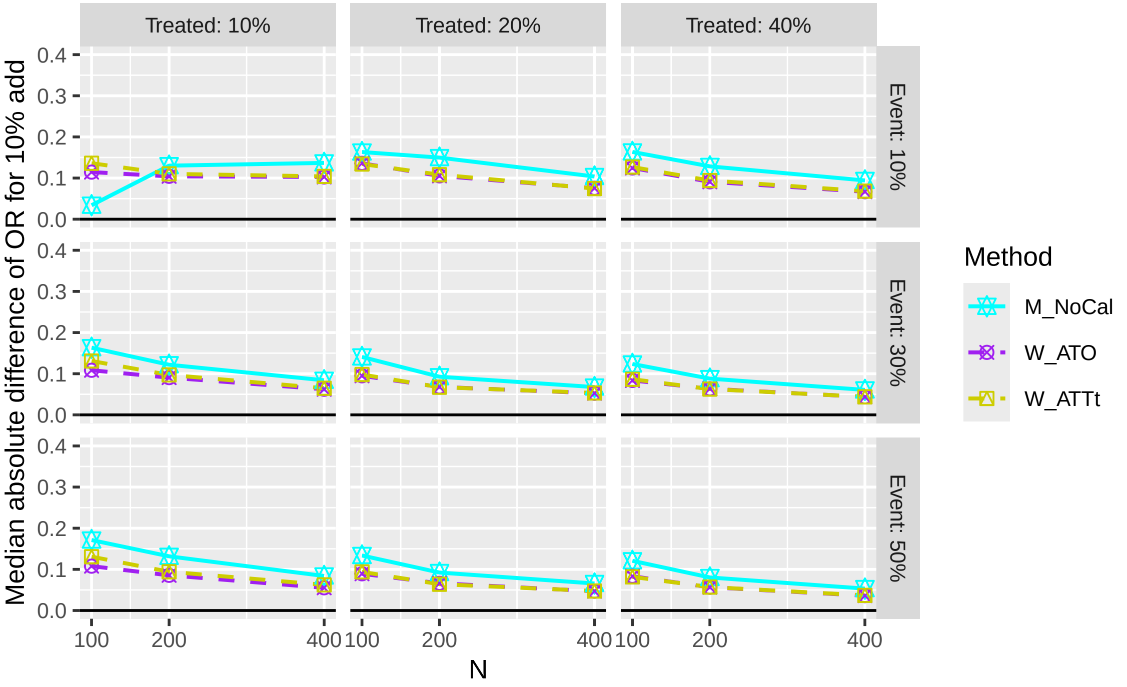

Figure S172. Median absolute difference of OR for 10% data addition (unimodal continuous covariate, matching ratio 1:2, true OR: 1, c statistic: 0.6); other methods.

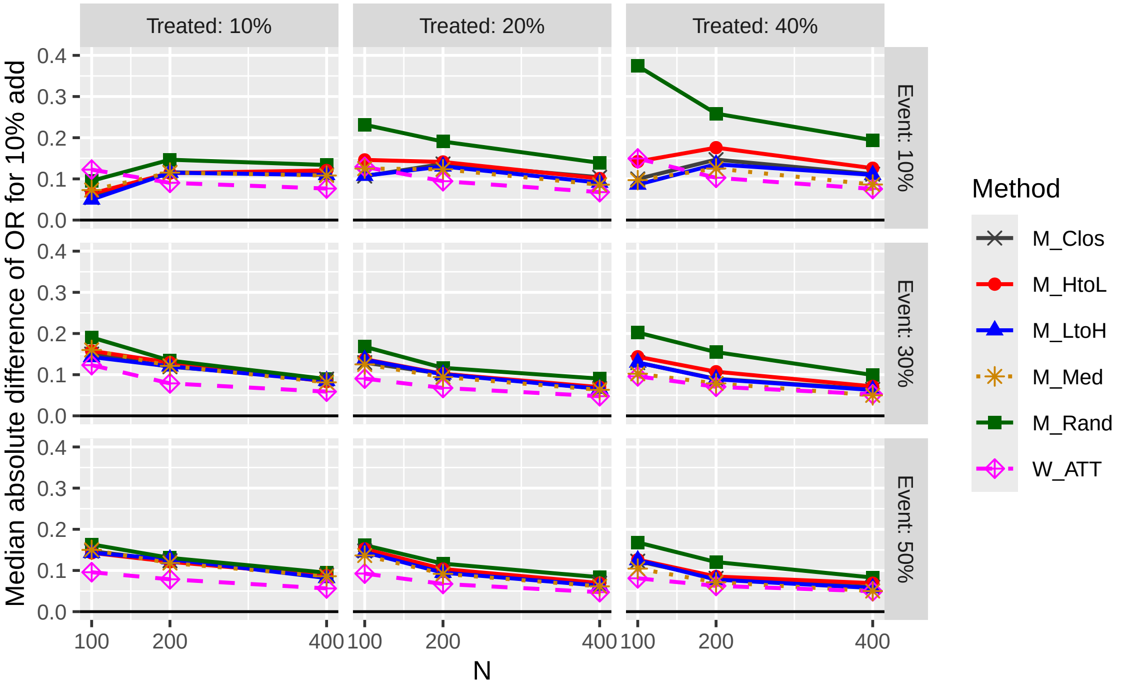

Figure S173. Median absolute difference of OR for 10% data addition (unimodal continuous covariate, matching ratio 1:2, true OR: 0.75, c statistic: 0.85).

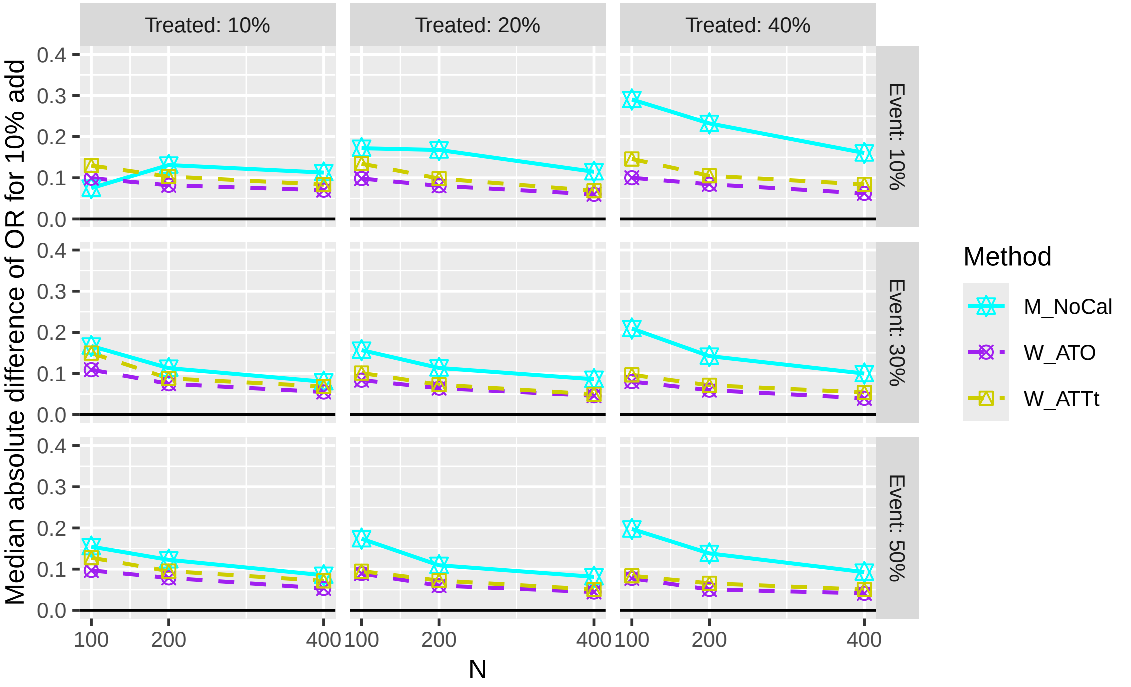

Figure S174. Median absolute difference of OR for 10% data addition (unimodal continuous covariate, matching ratio 1:2, true OR: 0.75, c statistic: 0.85); other methods.

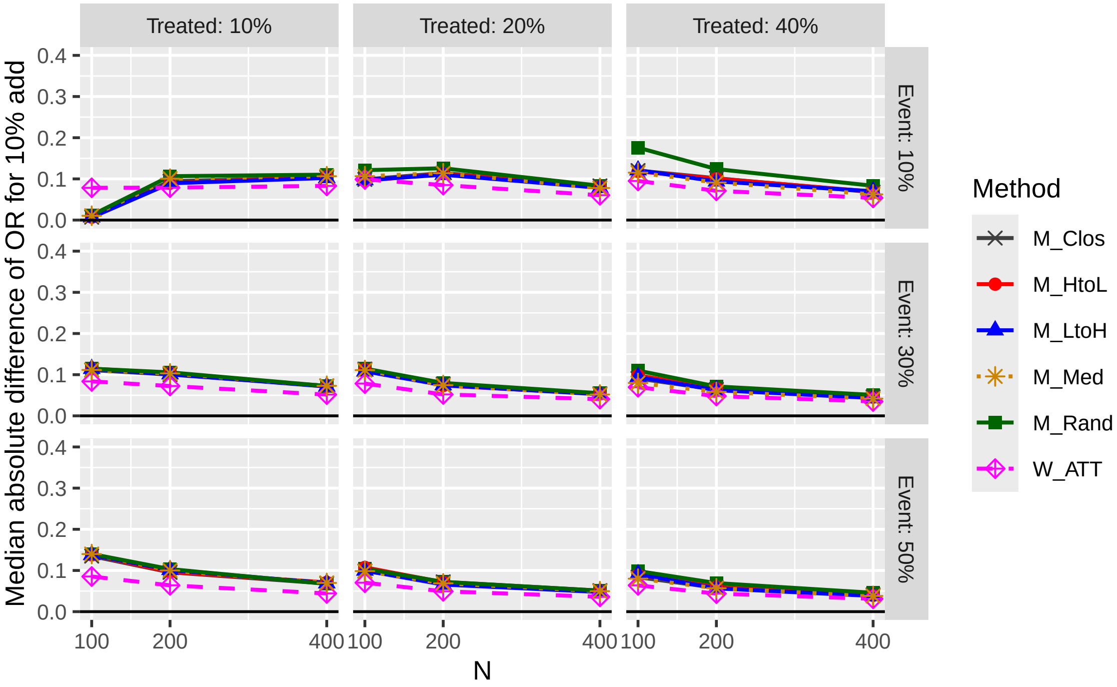

Figure S175. Median absolute difference of OR for 10% data addition (unimodal continuous covariate, matching ratio 1:2, true OR: 0.75, c statistic: 0.6).

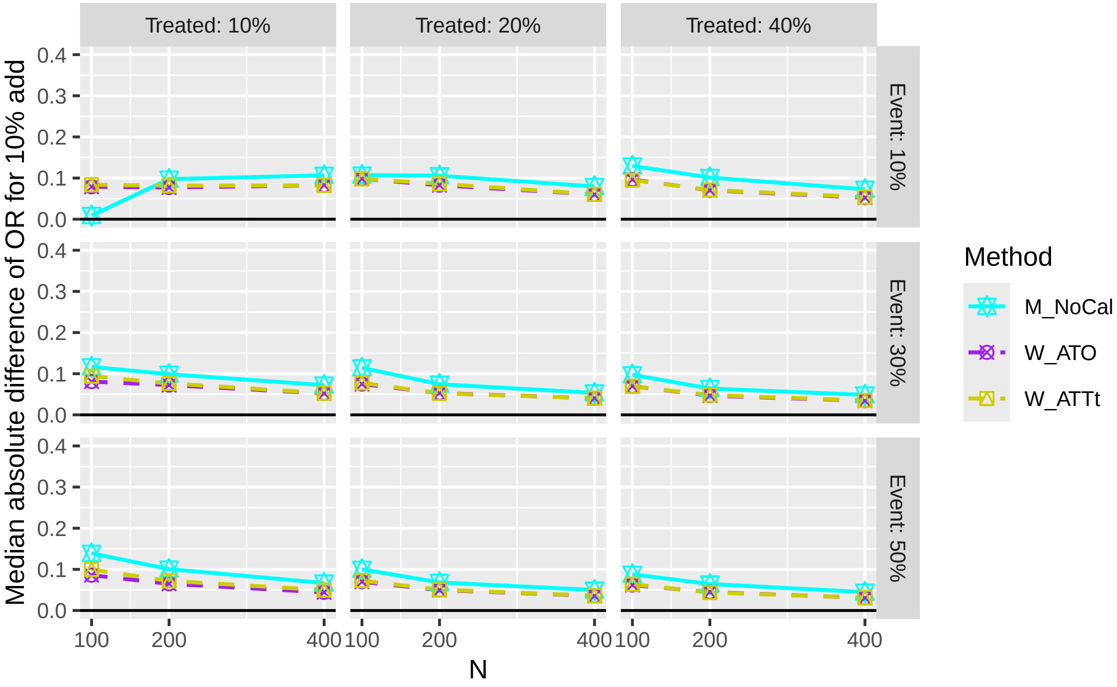

Figure S176. Median absolute difference of OR for 10% data addition (unimodal continuous covariate, matching ratio 1:2, true OR: 0.75, c statistic: 0.6); other methods.

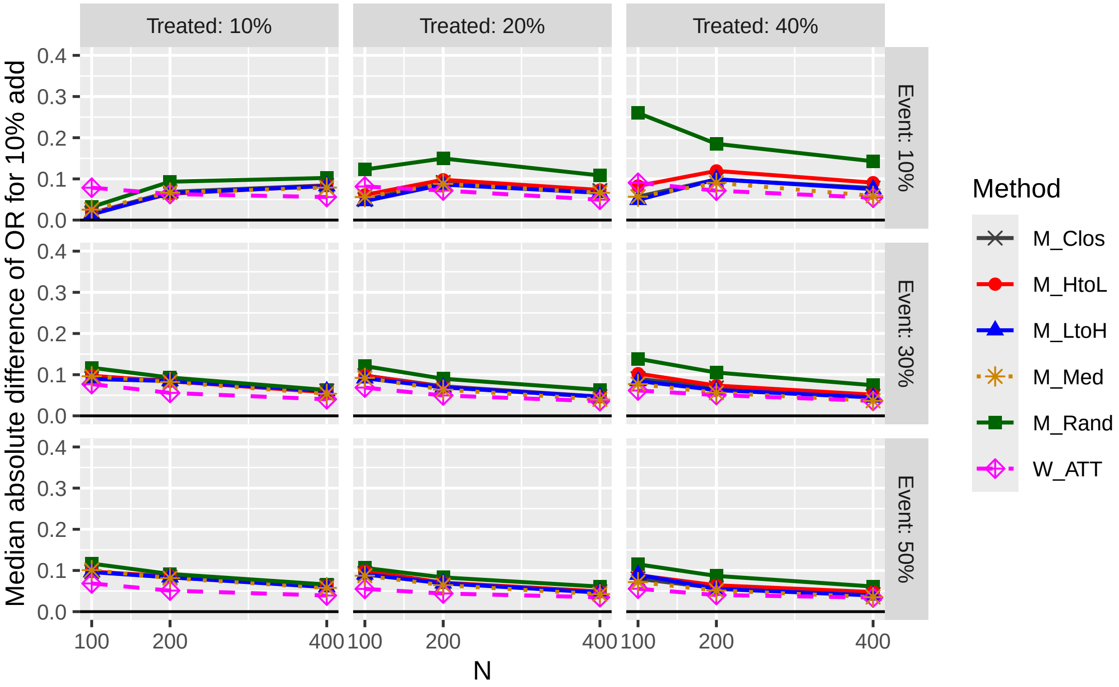

Figure S177. Median absolute difference of OR for 10% data addition (unimodal continuous covariate, matching ratio 1:2, true OR: 0.5, c statistic: 0.85).

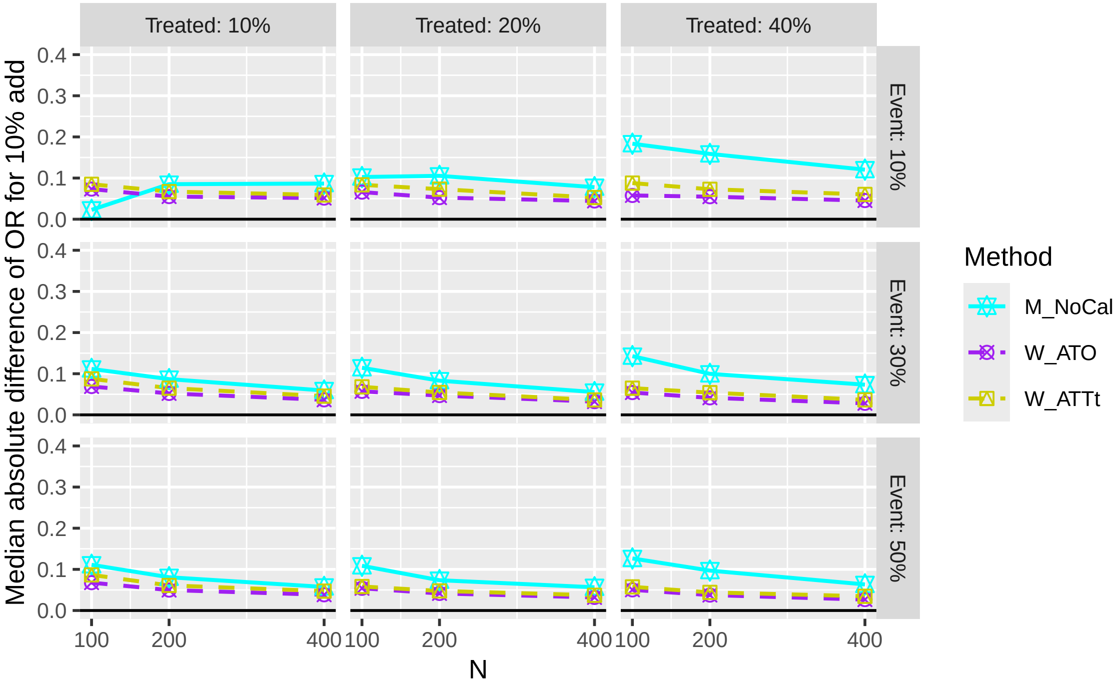

Figure S178. Median absolute difference of OR for 10% data addition (unimodal continuous covariate, matching ratio 1:2, true OR: 0.5, c statistic: 0.85); other methods.

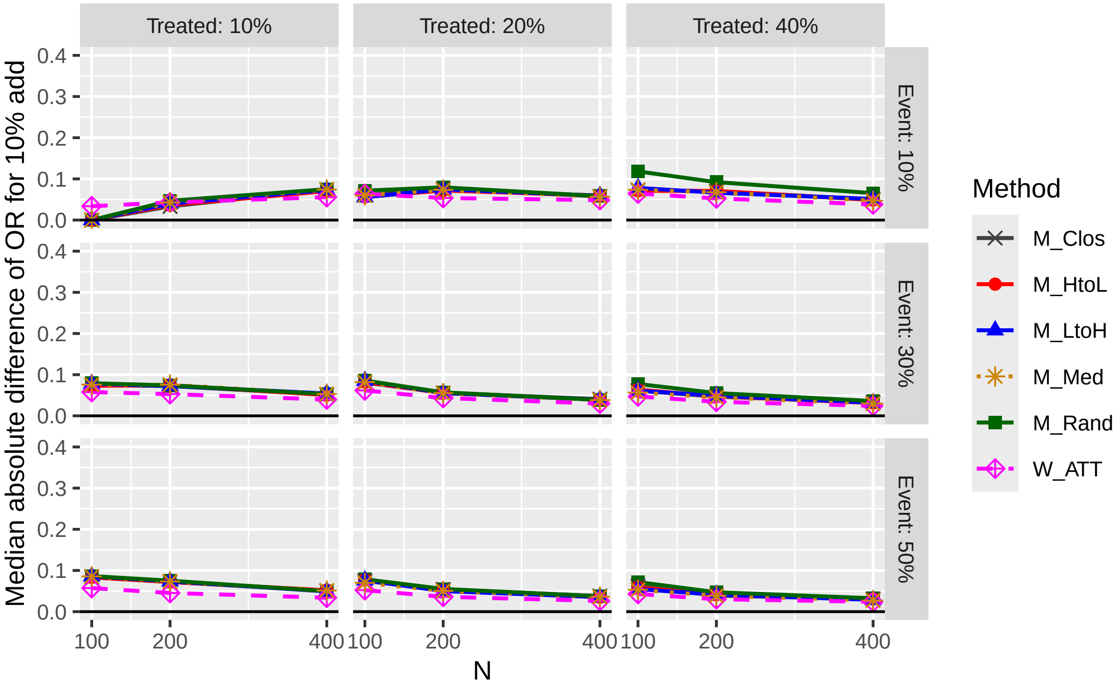

Figure S179. Median absolute difference of OR for 10% data addition (unimodal continuous covariate, matching ratio 1:2, true OR: 0.5, c statistic: 0.6).

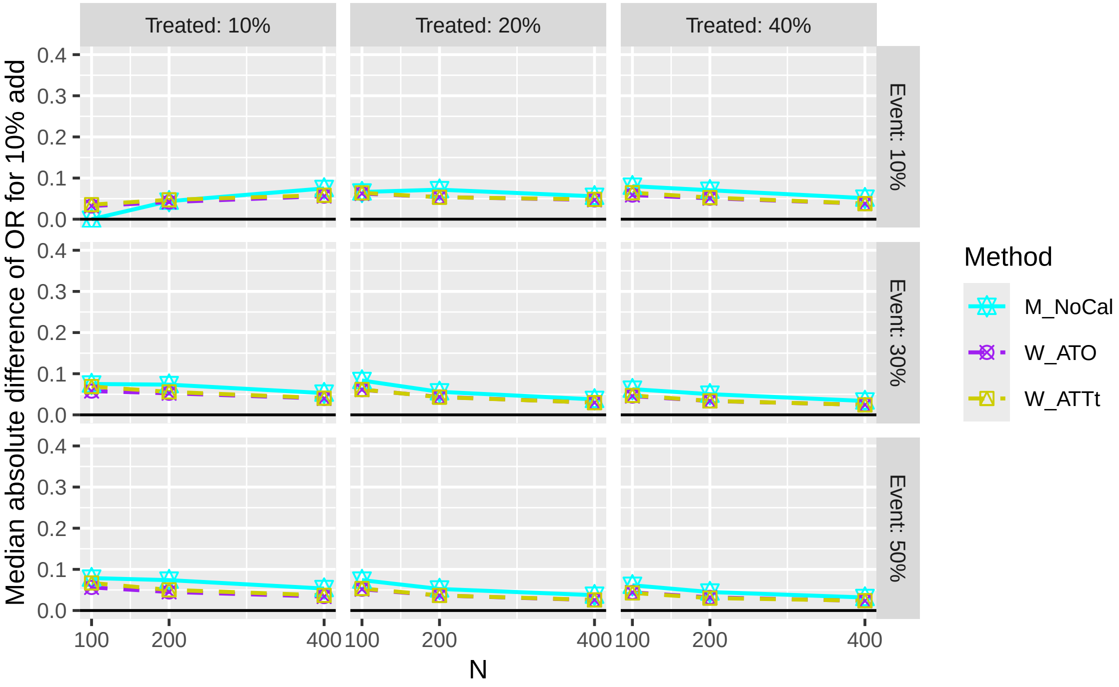

Figure S180. Median absolute difference of OR for 10% data addition (unimodal continuous covariate, matching ratio 1:2, true OR: 0.5, c statistic: 0.6); other methods.

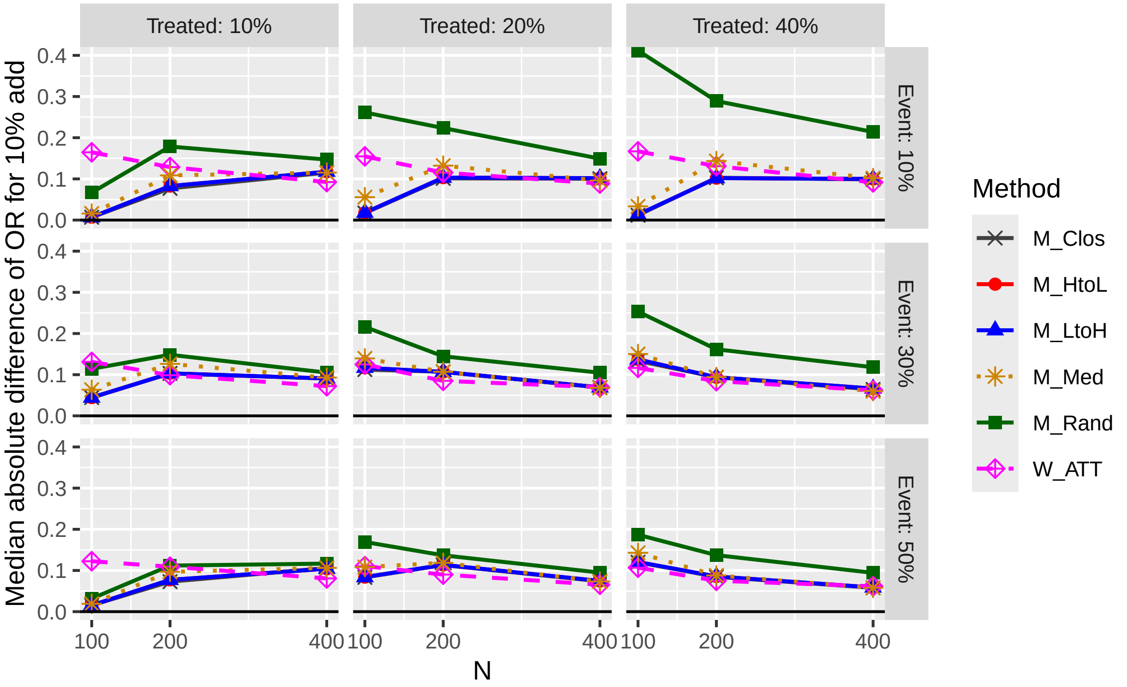

Figure S181. Median absolute difference of OR for 10% data addition (categorical covariate, matching ratio 1:1, true OR: 1, c statistic: 0.85).

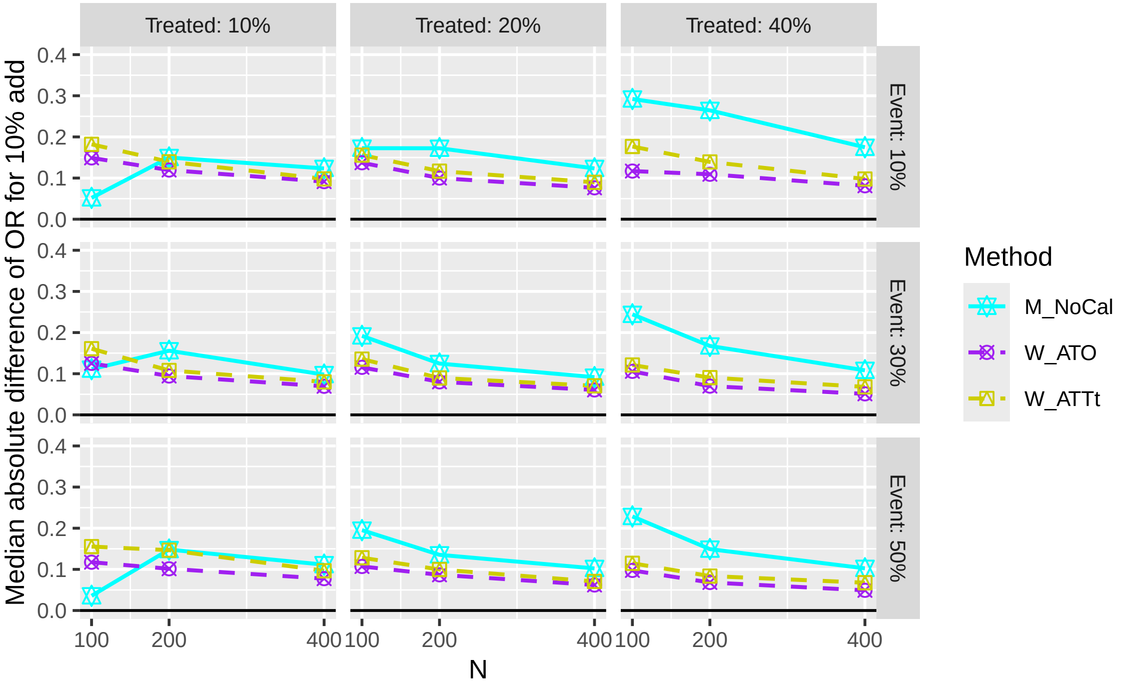

Figure S182. Median absolute difference of OR for 10% data addition (categorical covariate, matching ratio 1:1, true OR: 1, c statistic: 0.85); other methods.

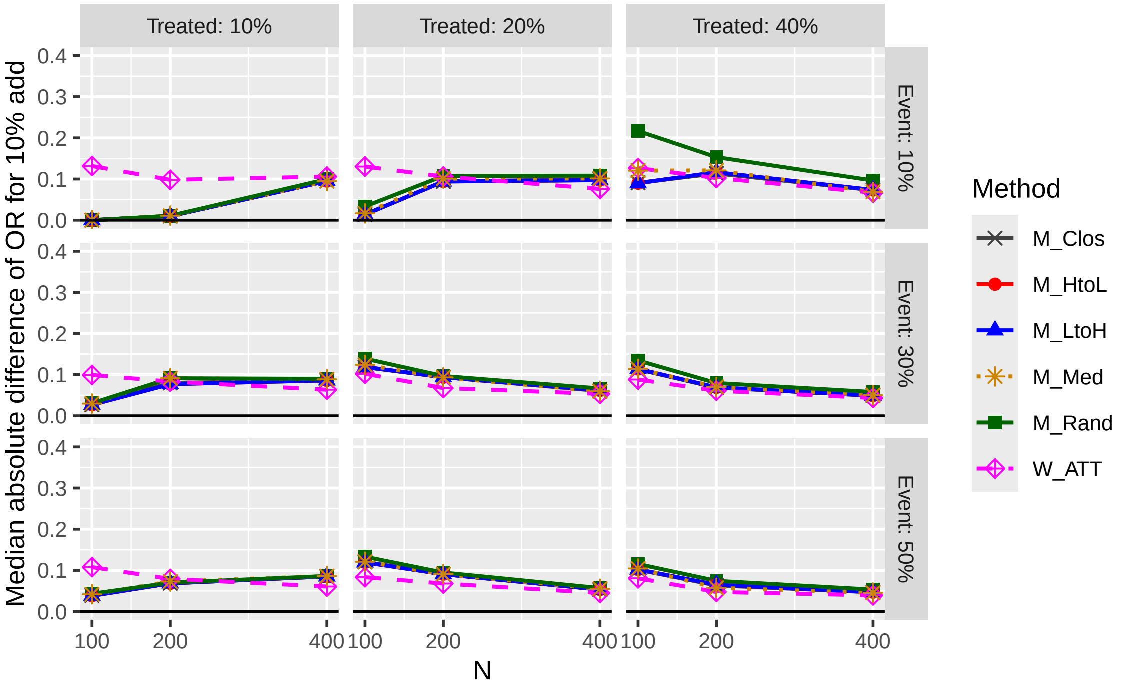

Figure S183. Median absolute difference of OR for 10% data addition (categorical covariate, matching ratio 1:1, true OR: 1, c statistic: 0.6).

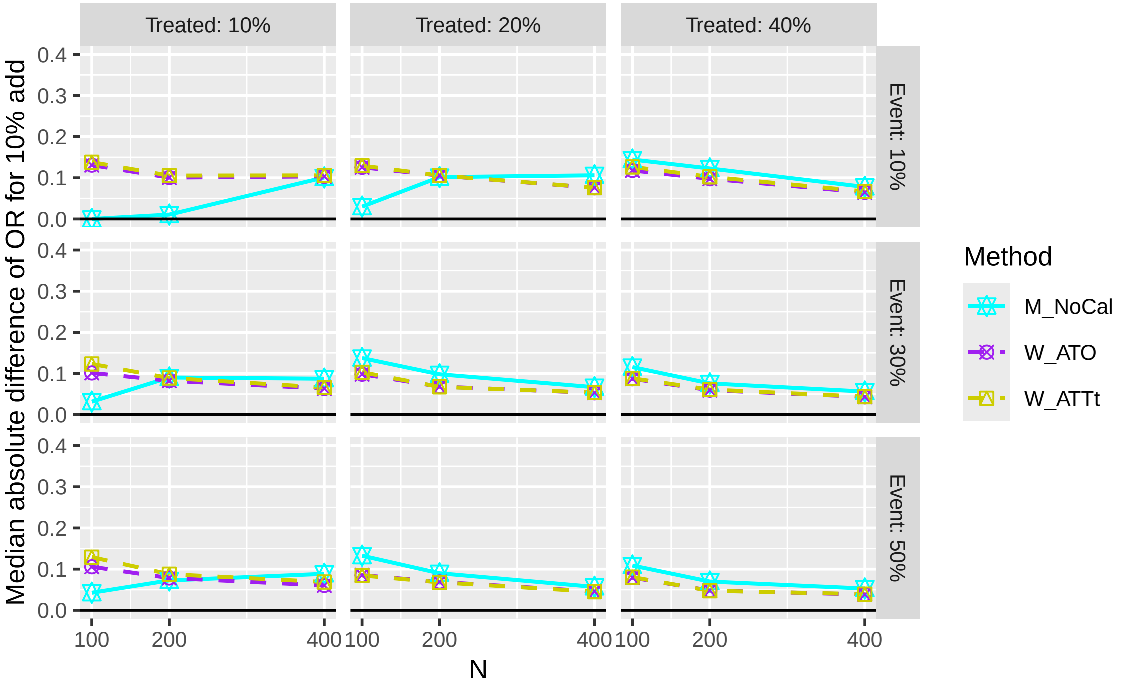

Figure S184. Median absolute difference of OR for 10% data addition (categorical covariate, matching ratio 1:1, true OR: 1, c statistic: 0.6); other methods.

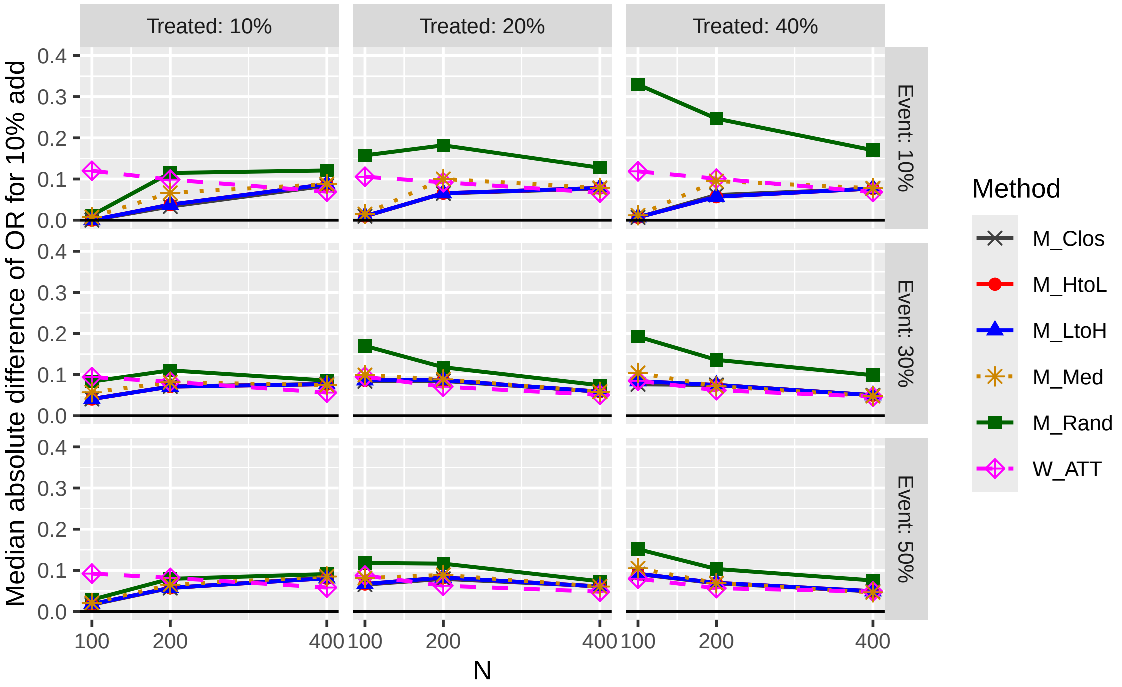

Figure S185. Median absolute difference of OR for 10% data addition (categorical covariate, matching ratio 1:1, true OR: 0.75, c statistic: 0.85).

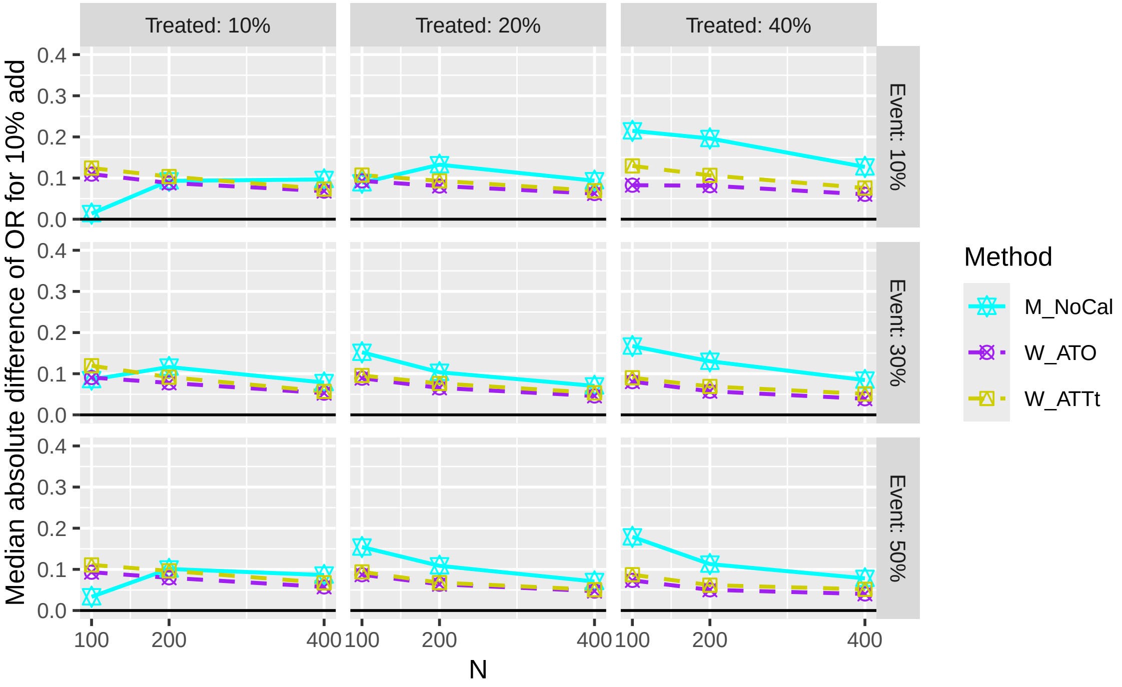

Figure S186. Median absolute difference of OR for 10% data addition (categorical covariate, matching ratio 1:1, true OR: 0.75, c statistic: 0.85); other methods.

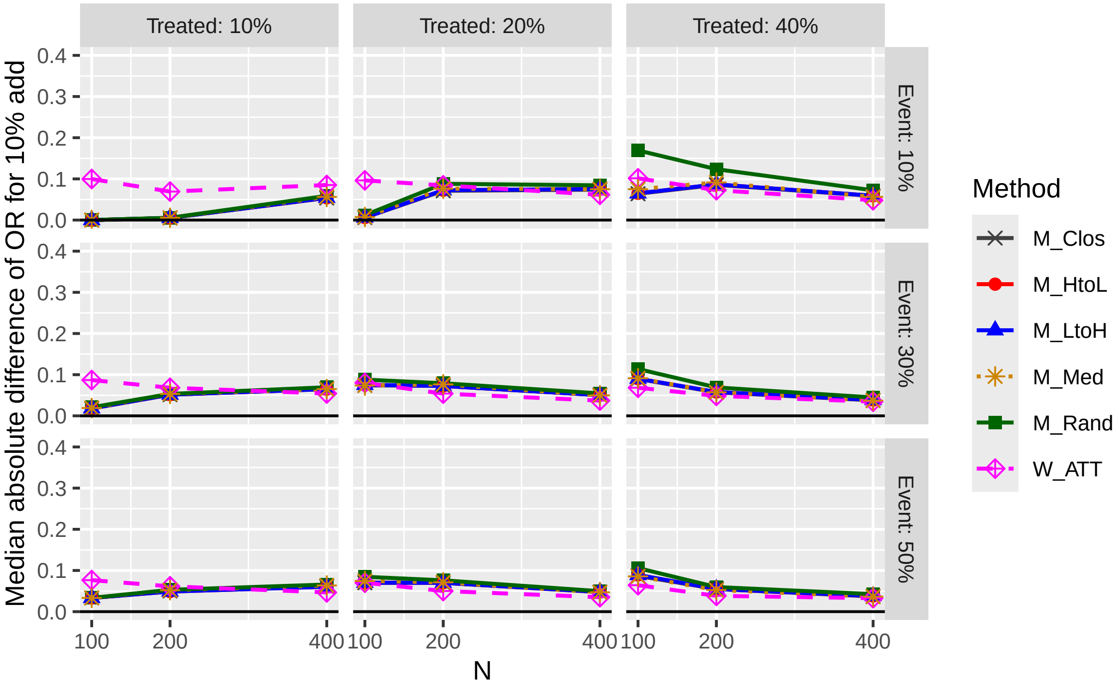

Figure S187. Median absolute difference of OR for 10% data addition (categorical covariate, matching ratio 1:1, true OR: 0.75, c statistic: 0.6).

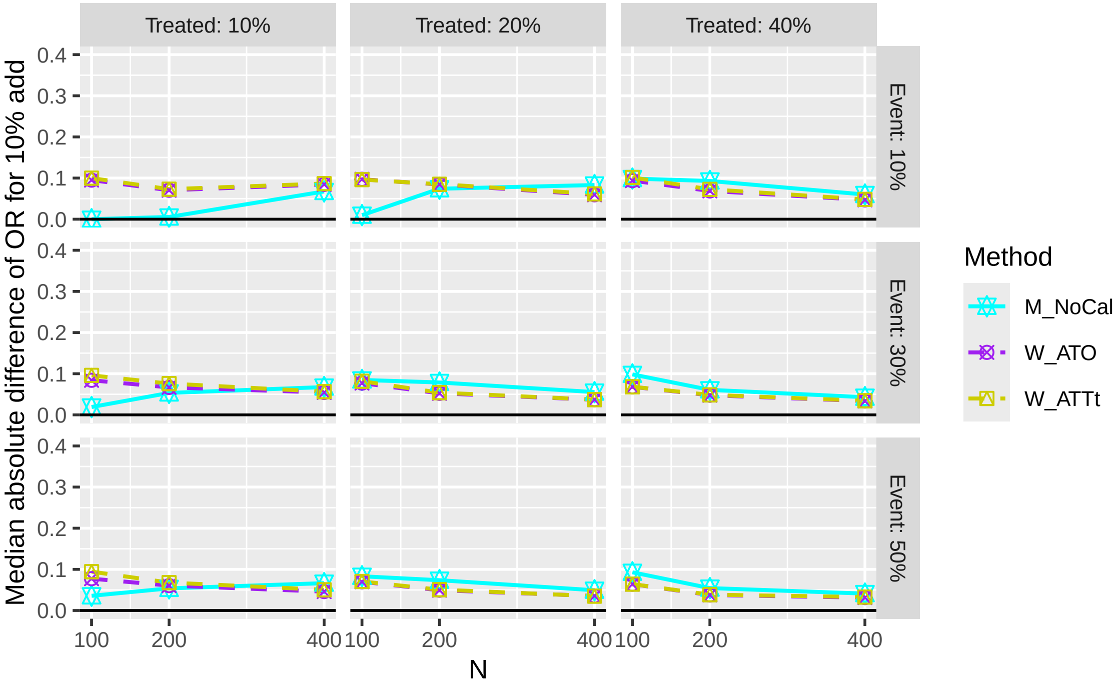

Figure S188. Median absolute difference of OR for 10% data addition (categorical covariate, matching ratio 1:1, true OR: 0.75, c statistic: 0.6); other methods.

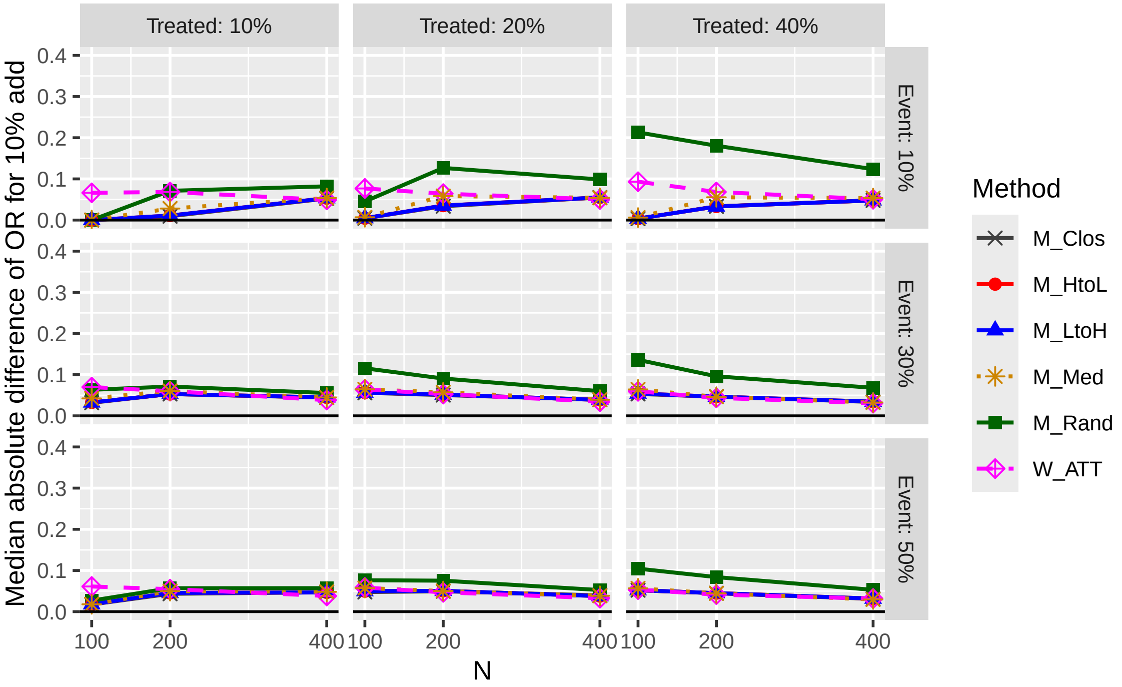

Figure S189. Median absolute difference of OR for 10% data addition (categorical covariate, matching ratio 1:1, true OR: 0.5, c statistic: 0.85).

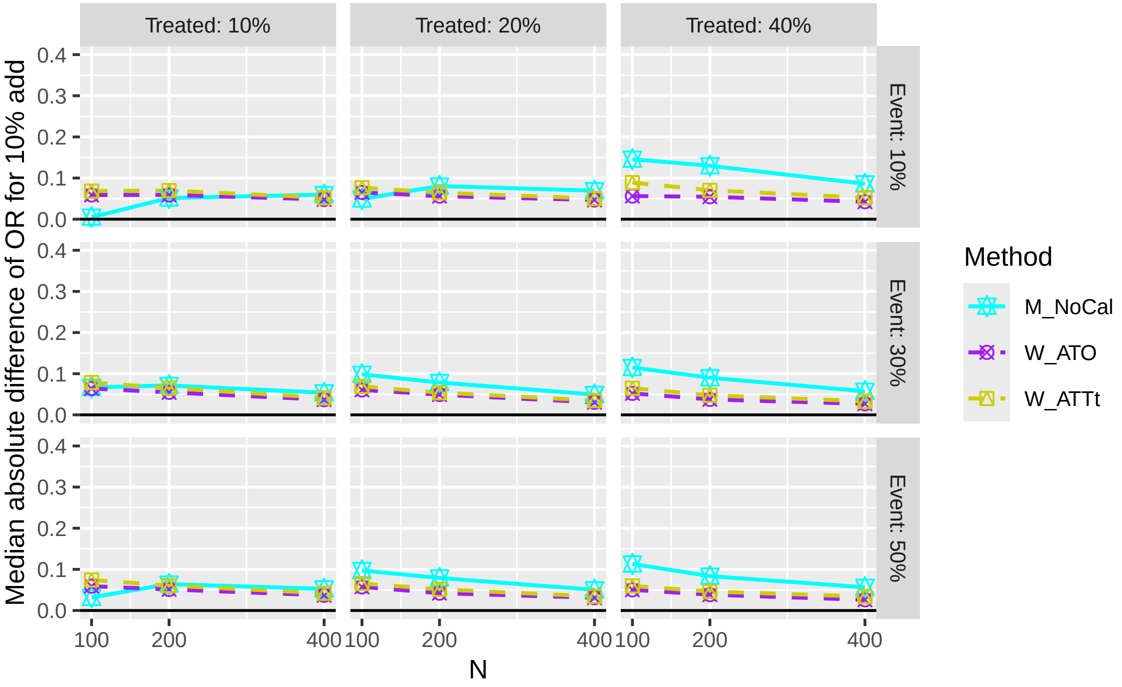

Figure S190. Median absolute difference of OR for 10% data addition (categorical covariate, matching ratio 1:1, true OR: 0.5, c statistic: 0.85); other methods.

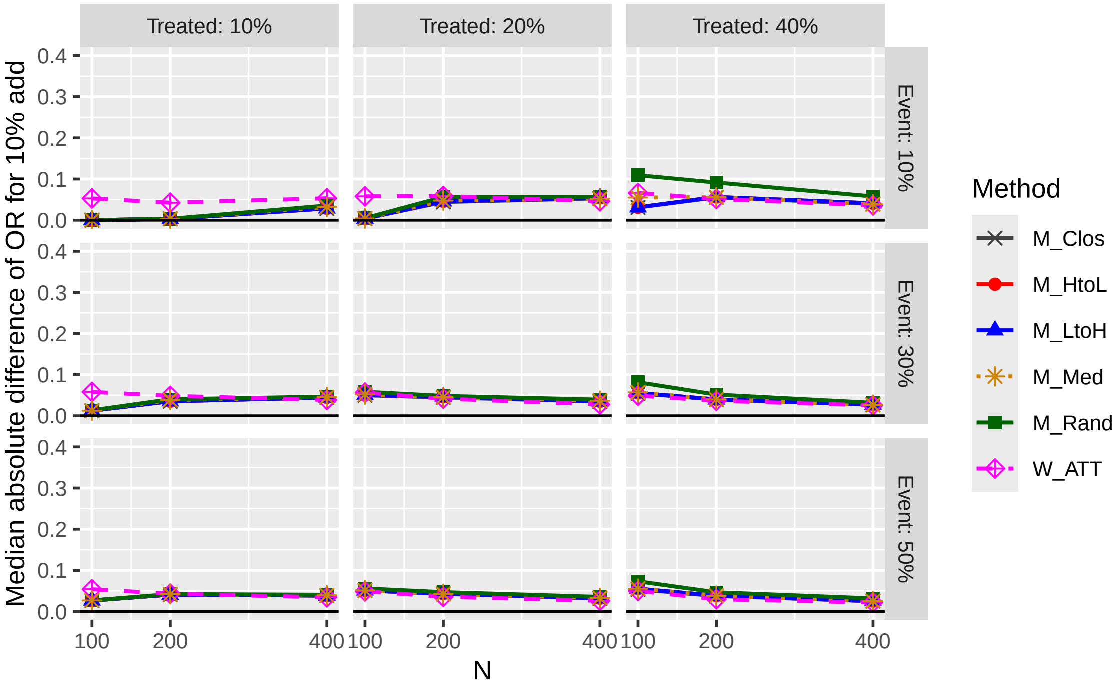

Figure S191. Median absolute difference of OR for 10% data addition (categorical covariate, matching ratio 1:1, true OR: 0.5, c statistic: 0.6).

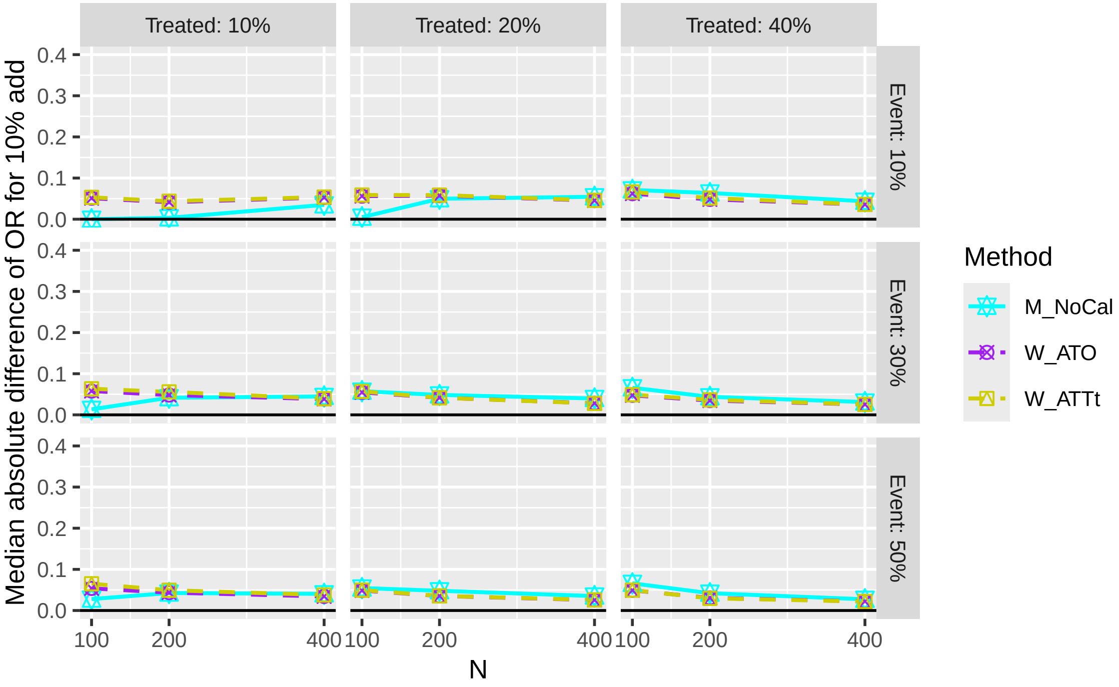

Figure S192. Median absolute difference of OR for 10% data addition (categorical covariate, matching ratio 1:1, true OR: 0.5, c statistic: 0.6); other methods.

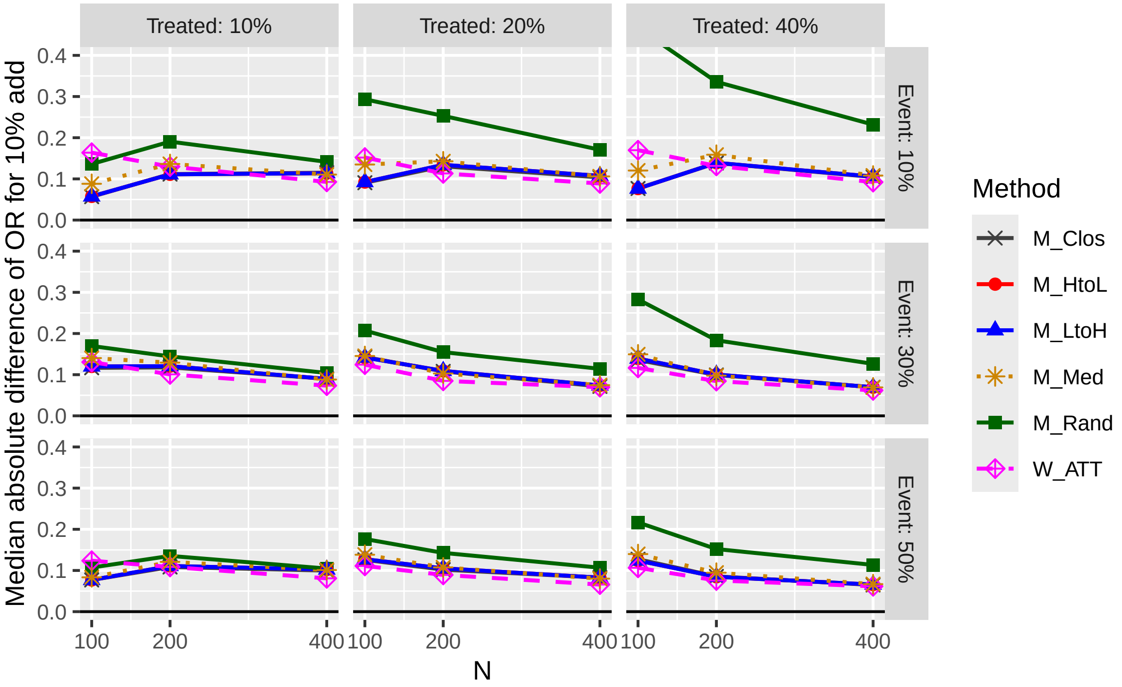

Figure S193. Median absolute difference of OR for 10% data addition (categorical covariate, matching ratio 1:2, true OR: 1, c statistic: 0.85).

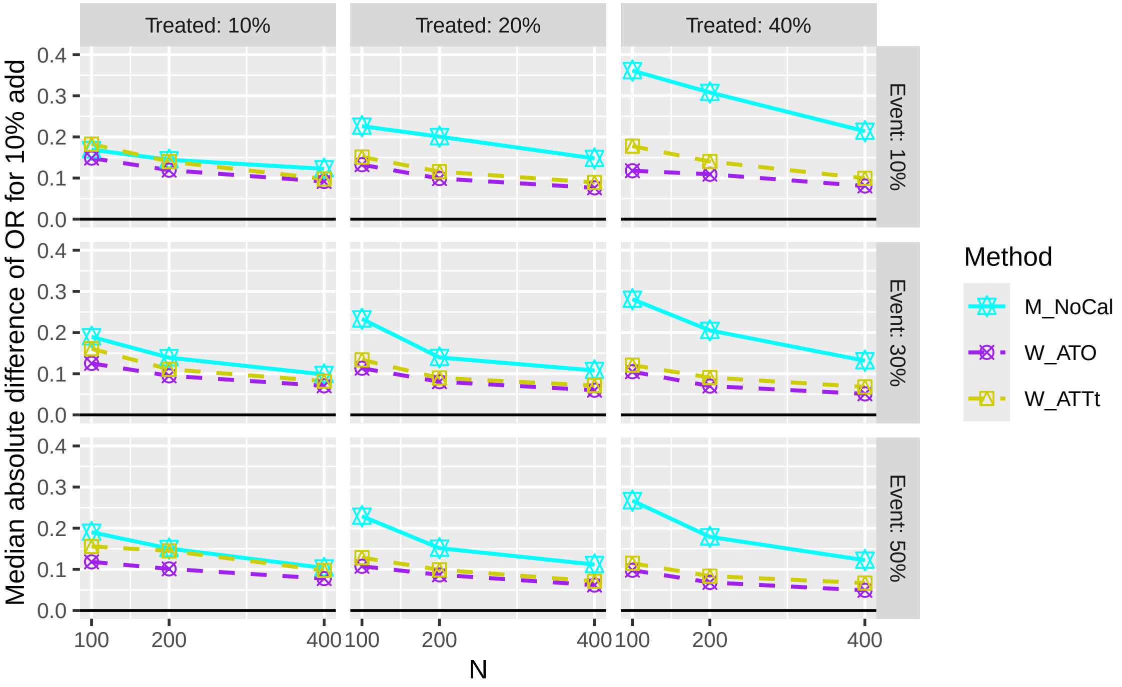

Figure S194. Median absolute difference of OR for 10% data addition (categorical covariate, matching ratio 1:2, true OR: 1, c statistic: 0.85); other methods.

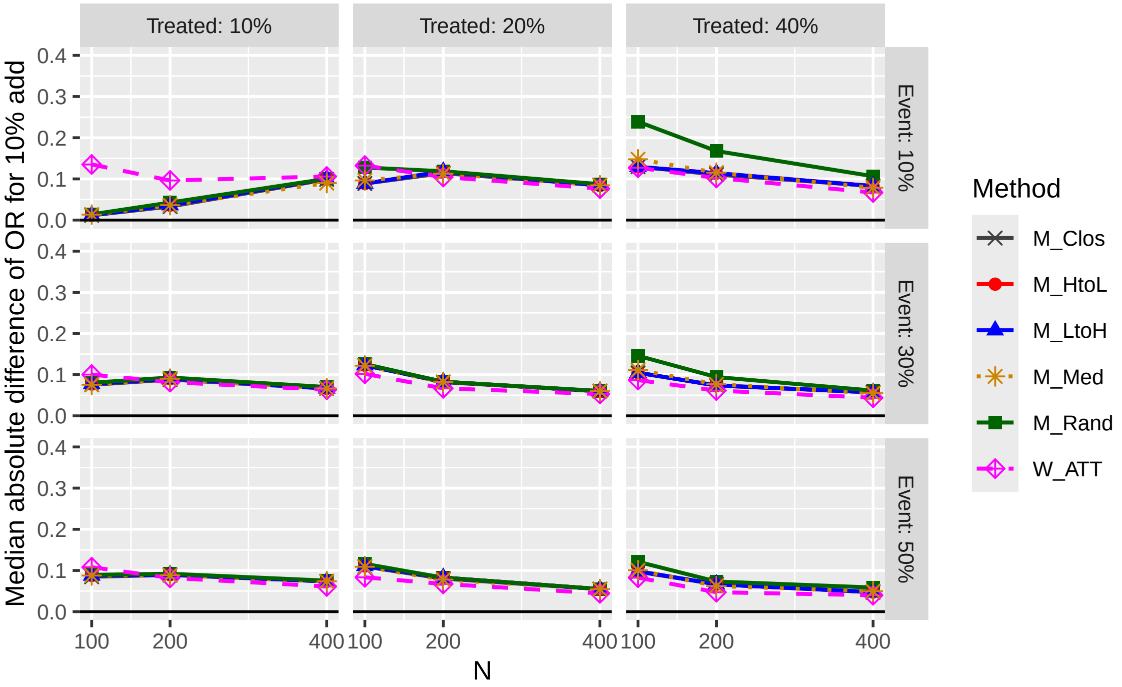

Figure S195. Median absolute difference of OR for 10% data addition (categorical covariate, matching ratio 1:2, true OR: 1, c statistic: 0.6).

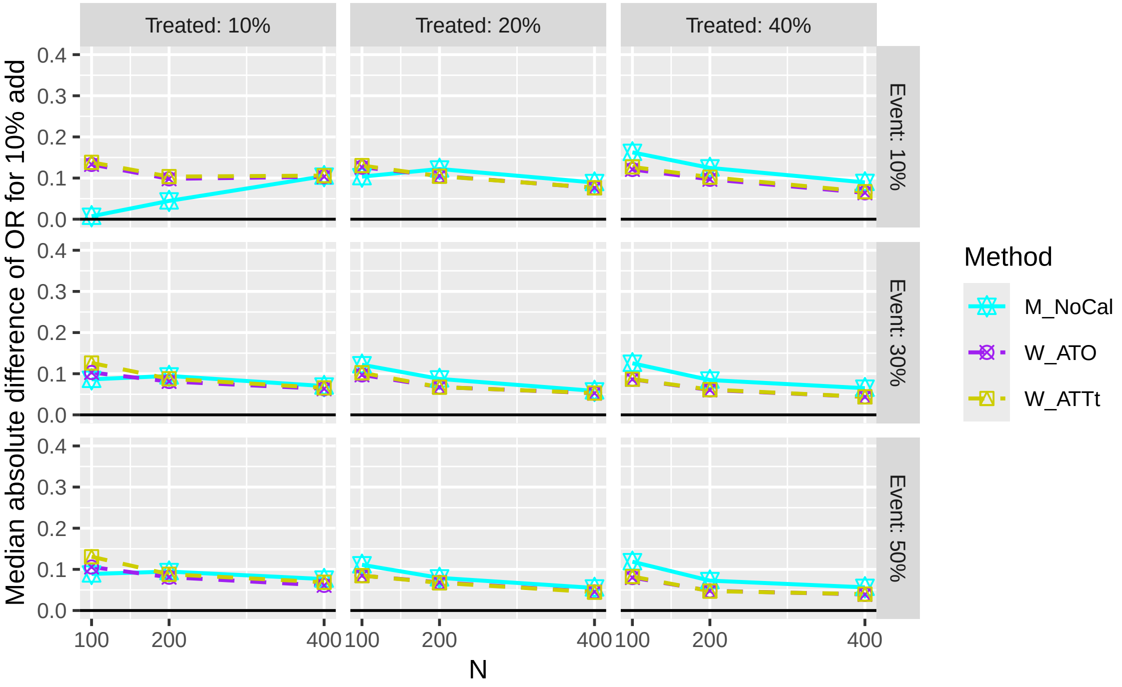

Figure S196. Median absolute difference of OR for 10% data addition (categorical covariate, matching ratio 1:2, true OR: 1, c statistic: 0.6); other methods.

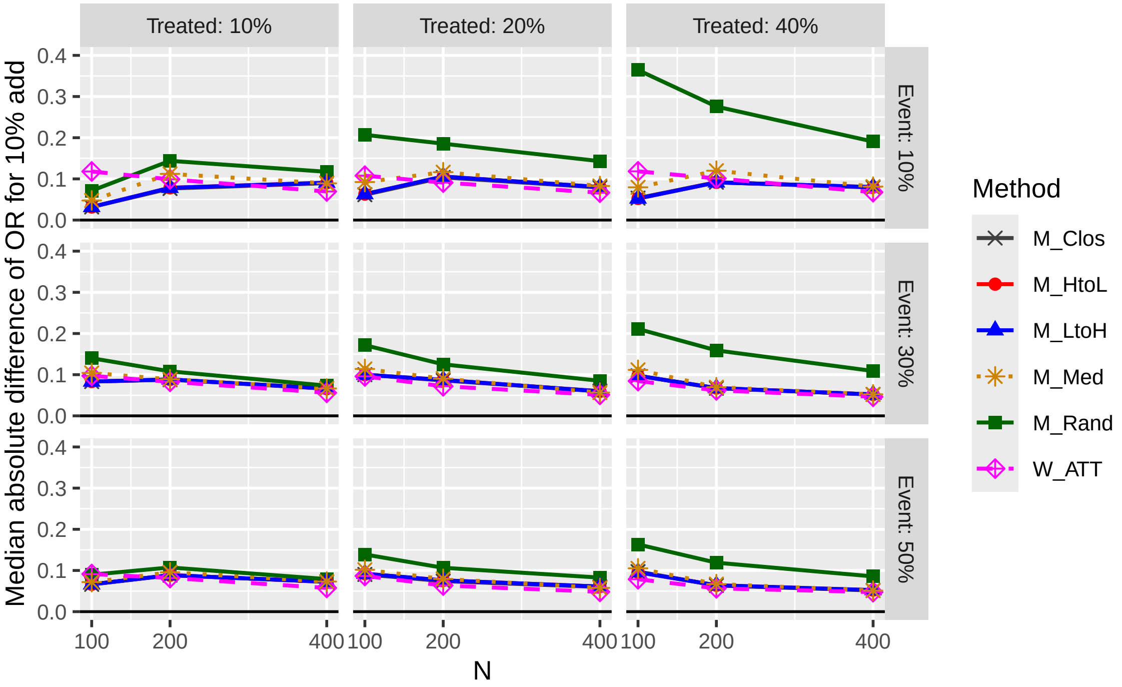

Figure S197. Median absolute difference of OR for 10% data addition (categorical covariate, matching ratio 1:2, true OR: 0.75, c statistic: 0.85).

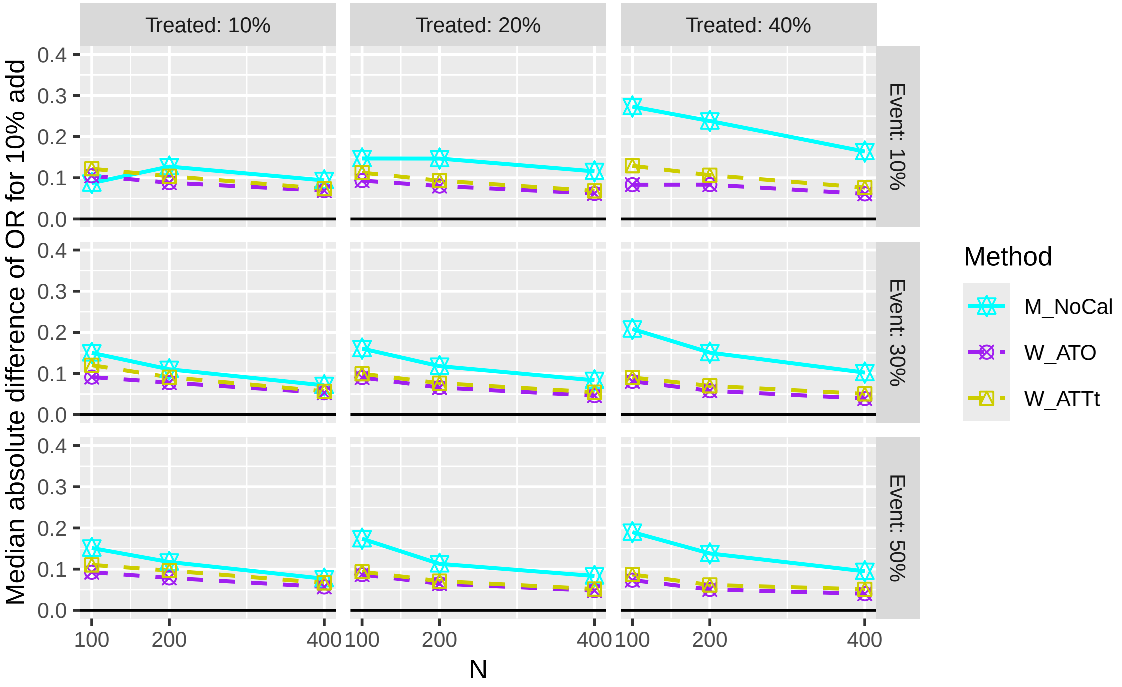

Figure S198. Median absolute difference of OR for 10% data addition (categorical covariate, matching ratio 1:2, true OR: 0.75, c statistic: 0.85); other methods.

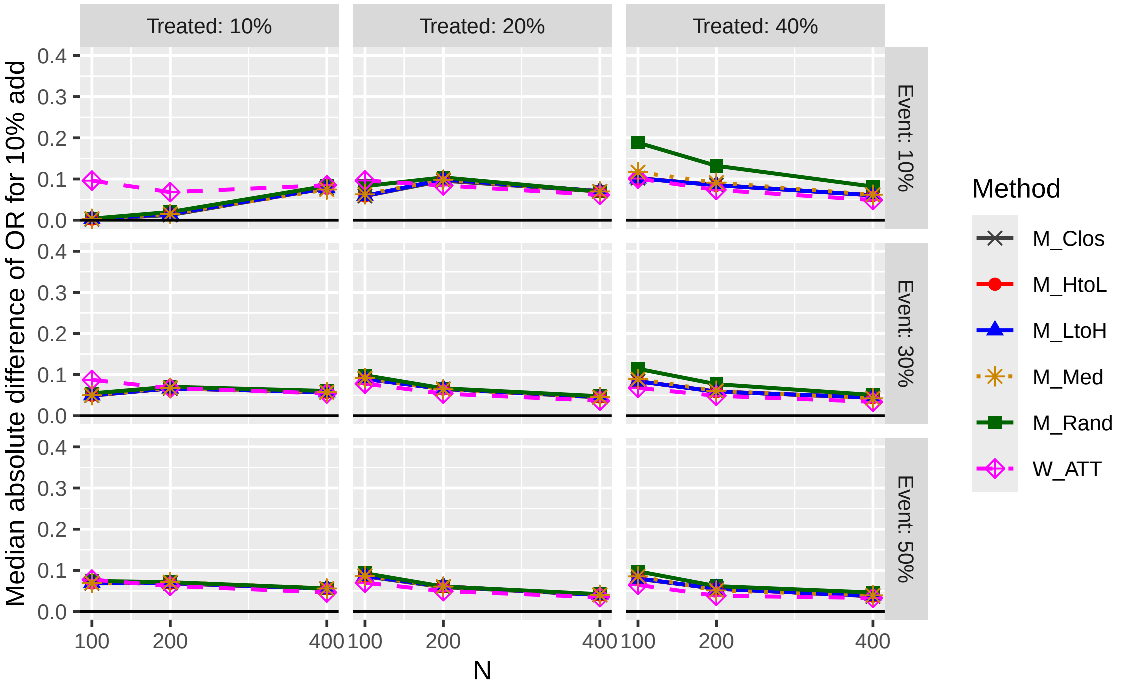

Figure S199. Median absolute difference of OR for 10% data addition (categorical covariate, matching ratio 1:2, true OR: 0.75, c statistic: 0.6).

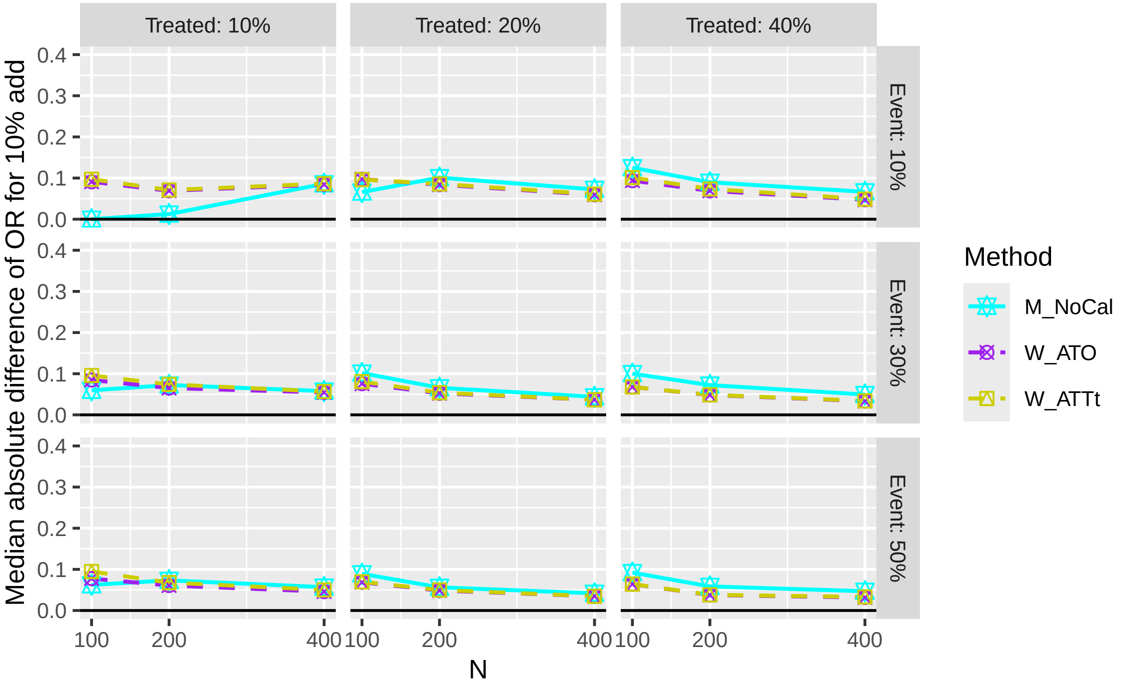

Figure S200. Median absolute difference of OR for 10% data addition (categorical covariate, matching ratio 1:2, true OR: 0.75, c statistic: 0.6); other methods.

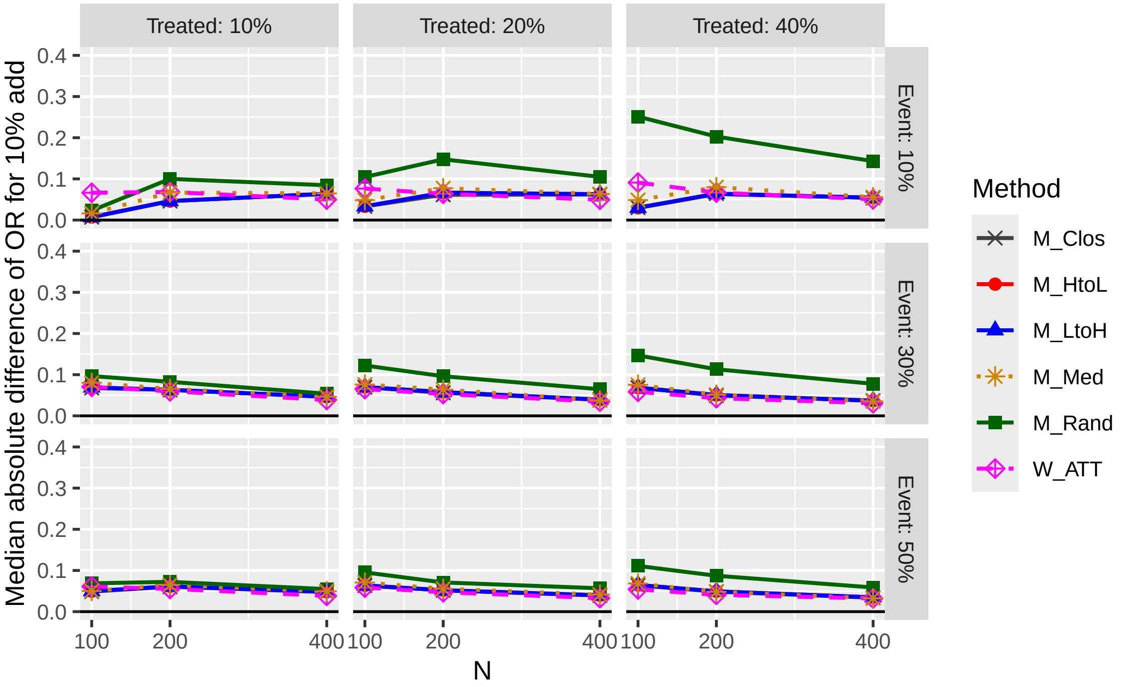

Figure S201. Median absolute difference of OR for 10% data addition (categorical covariate, matching ratio 1:2, true OR: 0.5, c statistic: 0.85).

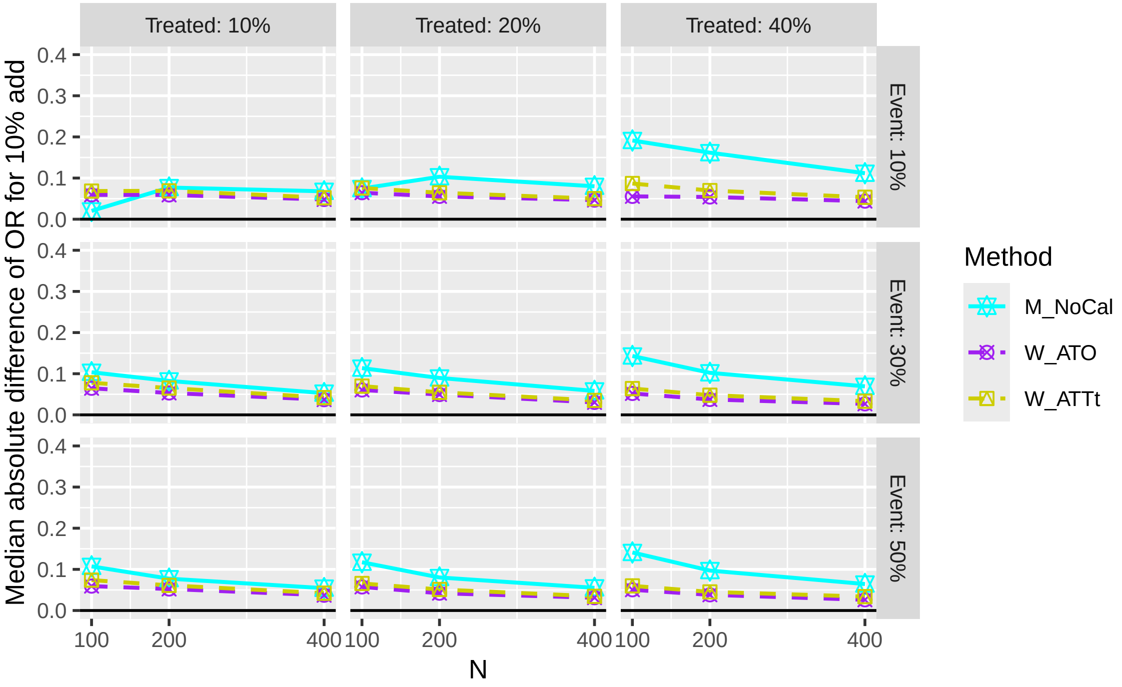

Figure S202. Median absolute difference of OR for 10% data addition (categorical covariate, matching ratio 1:2, true OR: 0.5, c statistic: 0.85); other methods.

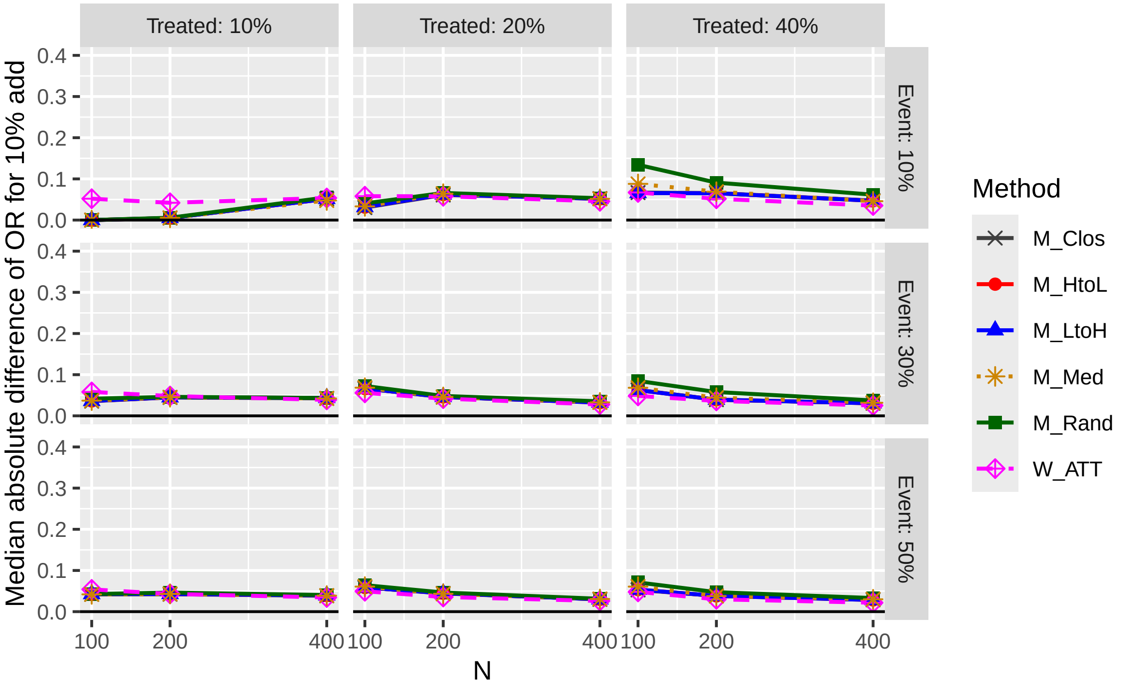

Figure S203. Median absolute difference of OR for 10% data addition (categorical covariate, matching ratio 1:2, true OR: 0.5, c statistic: 0.6).

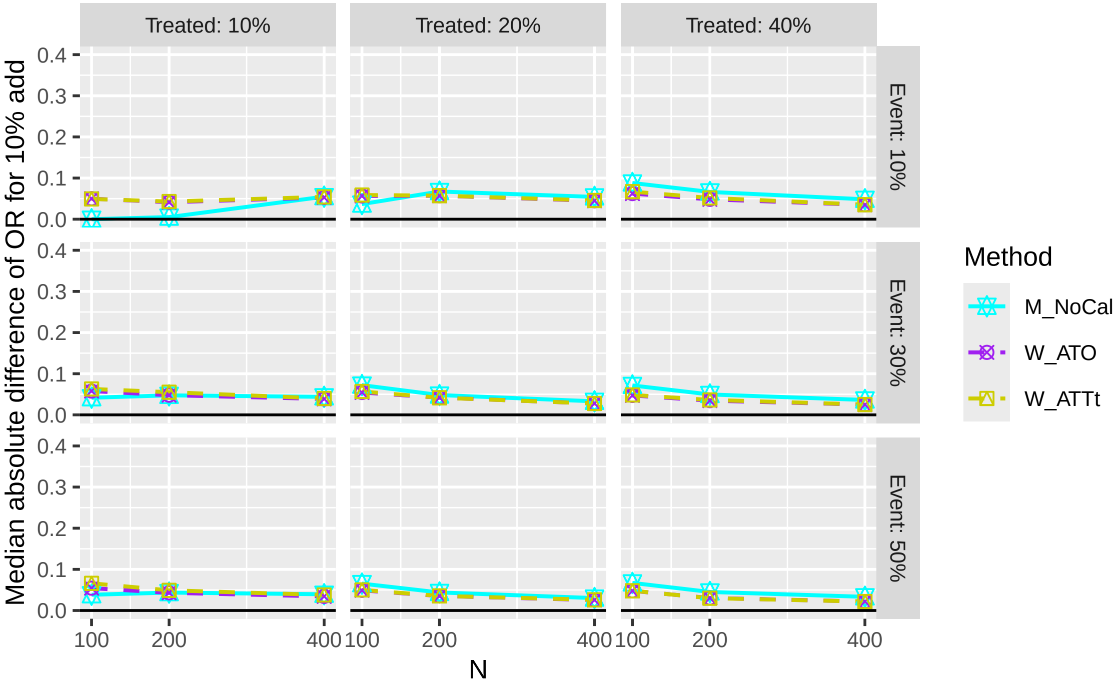

Figure S204. Median absolute difference of OR for 10% data addition (categorical covariate, matching ratio 1:2, true OR: 0.5, c statistic: 0.6); other methods.

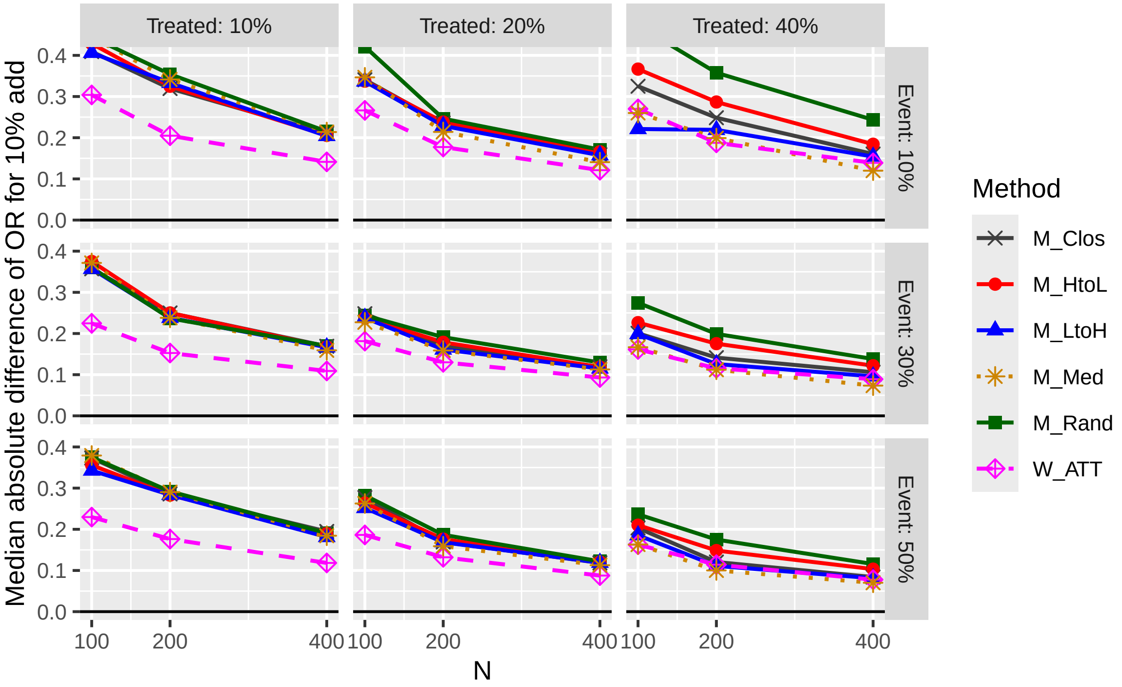

Figure S205. Median absolute difference of OR for 10% data addition (multimodal continuous covariate, matching ratio 1:1, true OR: 1, c statistic: 0.85).

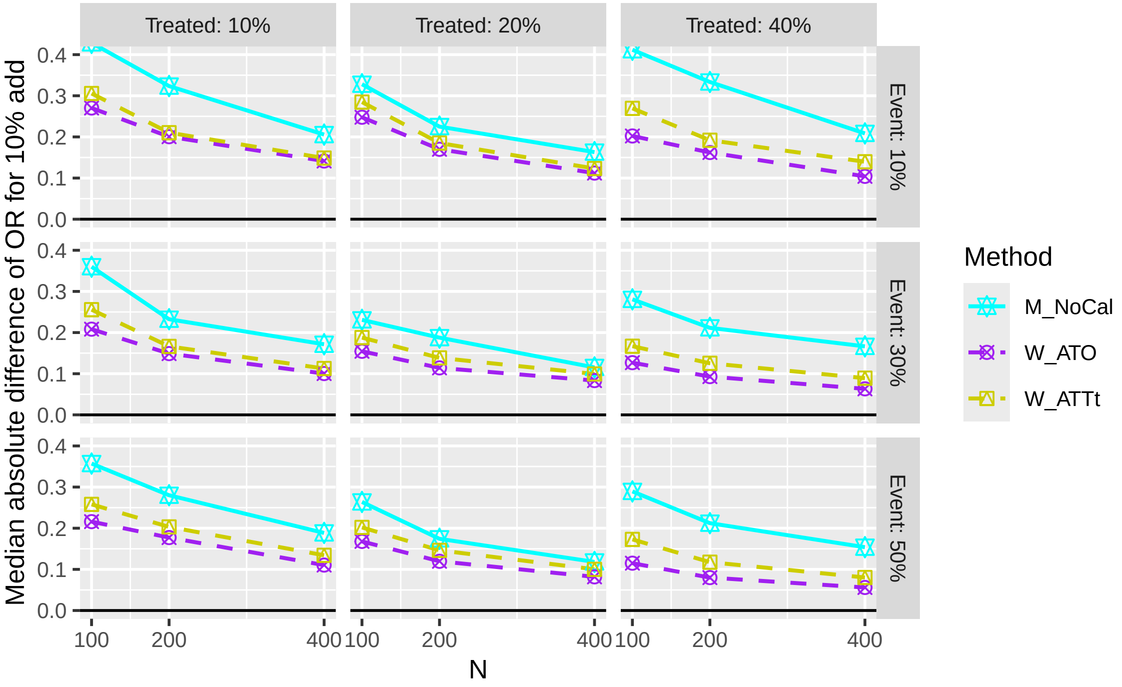

Figure S206. Median absolute difference of OR for 10% data addition (multimodal continuous covariate, matching ratio 1:1, true OR: 1, c statistic: 0.85); other methods.

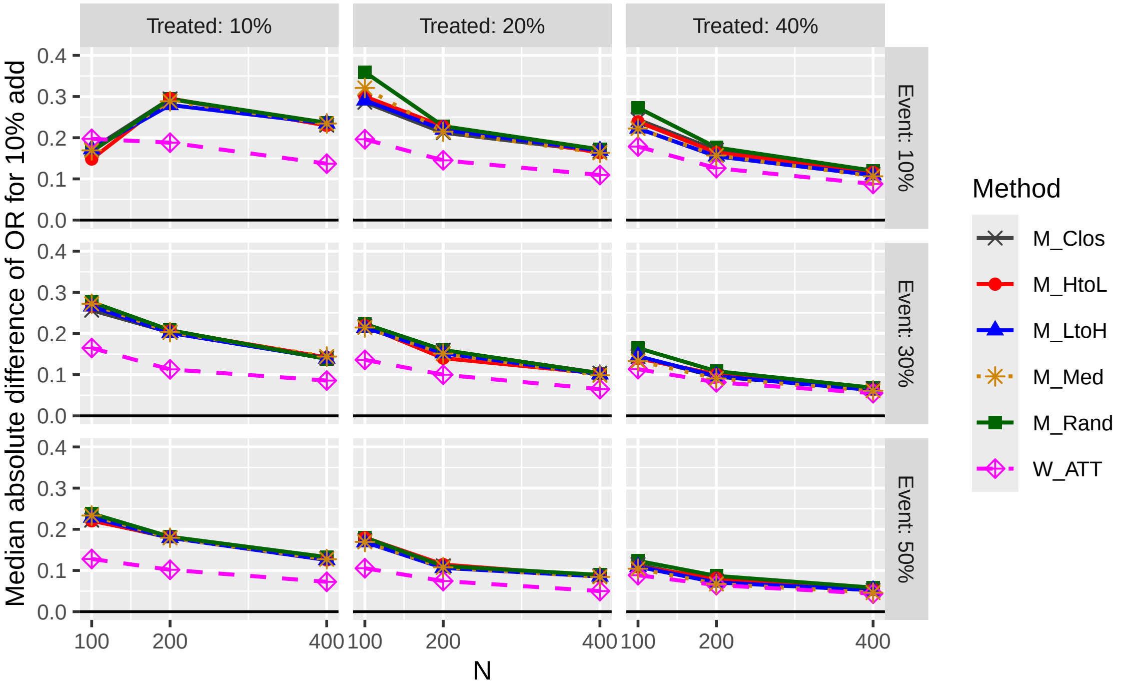

Figure S207. Median absolute difference of OR for 10% data addition (multimodal continuous covariate, matching ratio 1:1, true OR: 1, c statistic: 0.6).

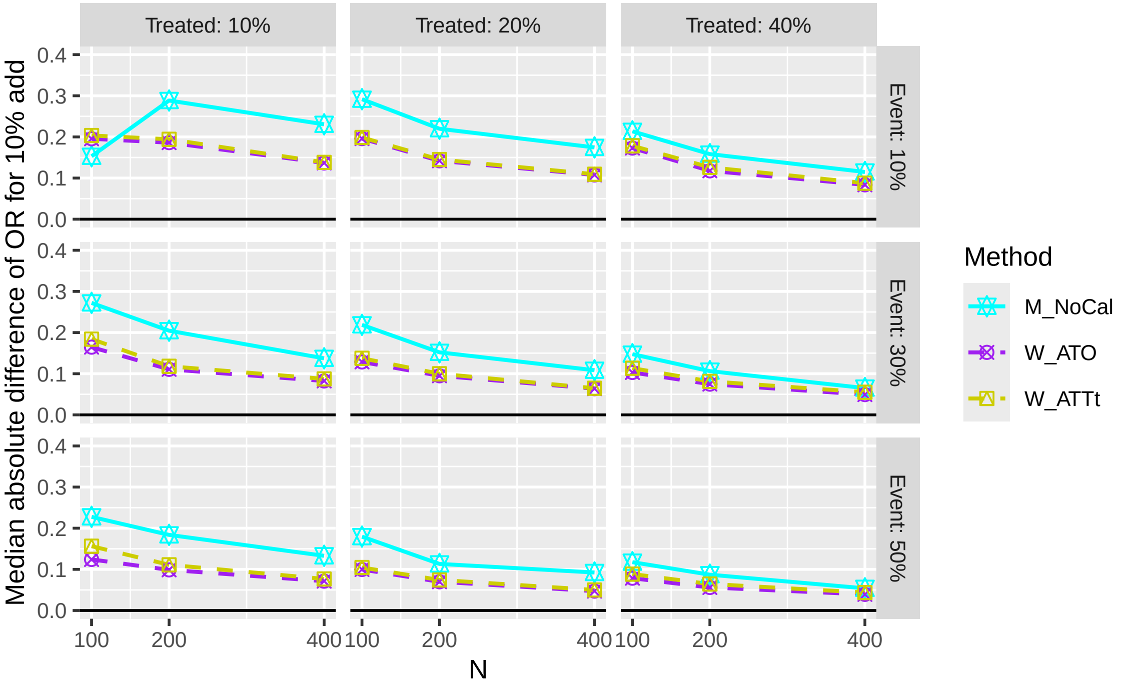

Figure S208. Median absolute difference of OR for 10% data addition (multimodal continuous covariate, matching ratio 1:1, true OR: 1, c statistic: 0.6); other methods.

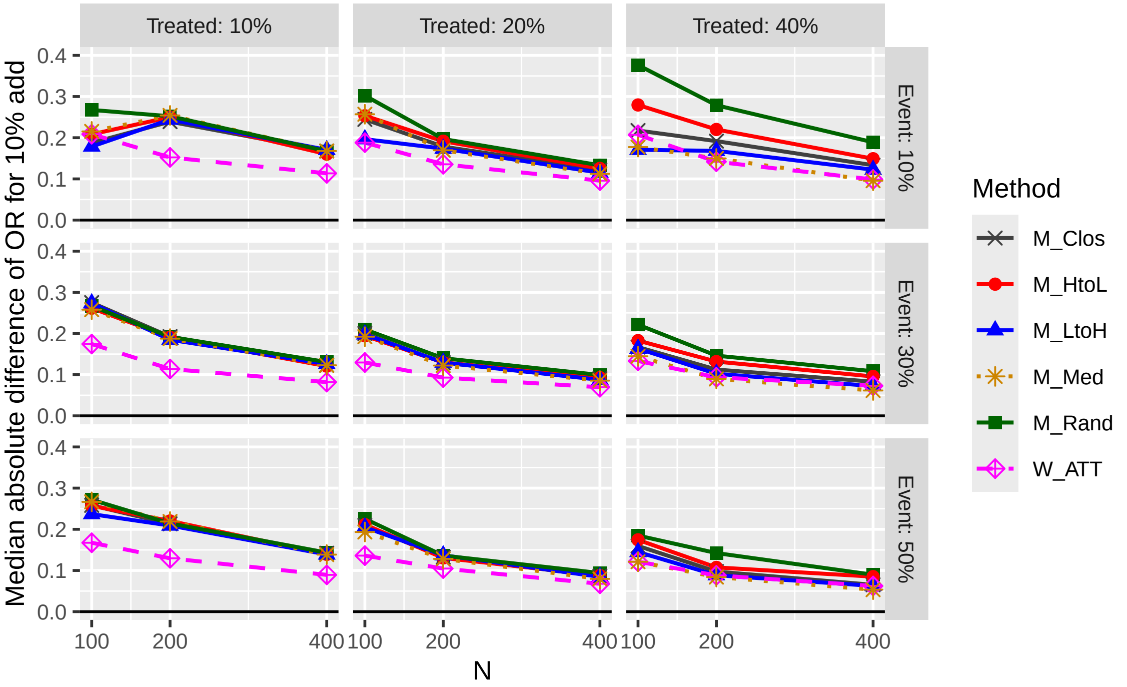

Figure S209. Median absolute difference of OR for 10% data addition (multimodal continuous covariate, matching ratio 1:1, true OR: 0.75, c statistic: 0.85).

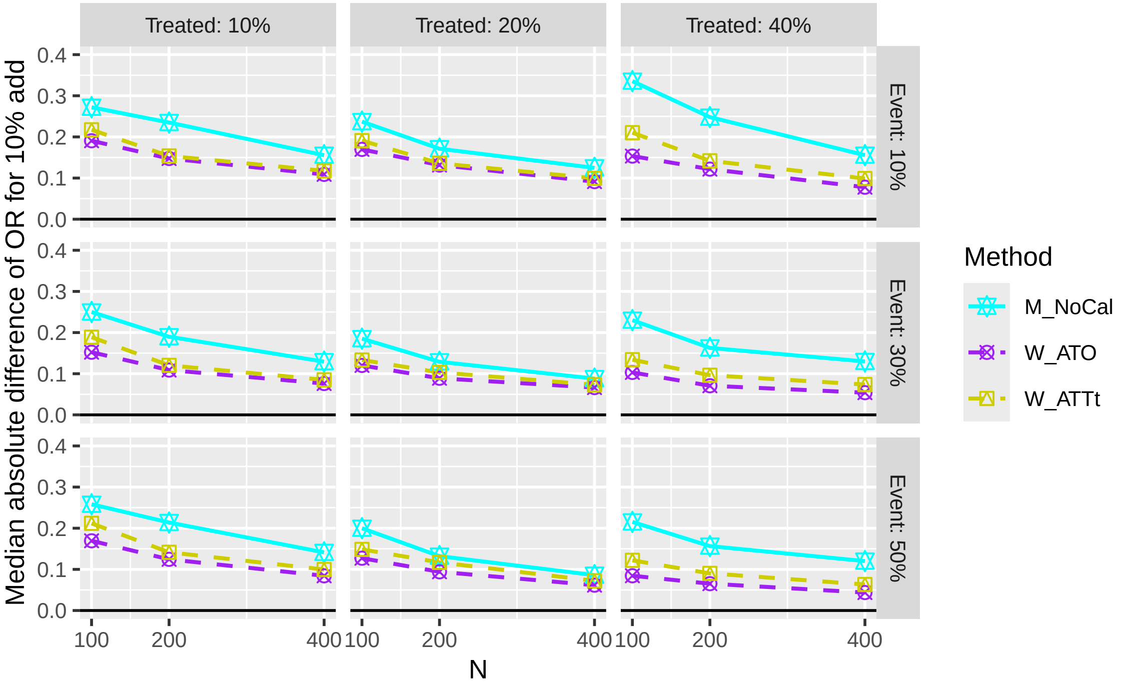

Figure S210. Median absolute difference of OR for 10% data addition (multimodal continuous covariate, matching ratio 1:1, true OR: 0.75, c statistic: 0.85); other methods.

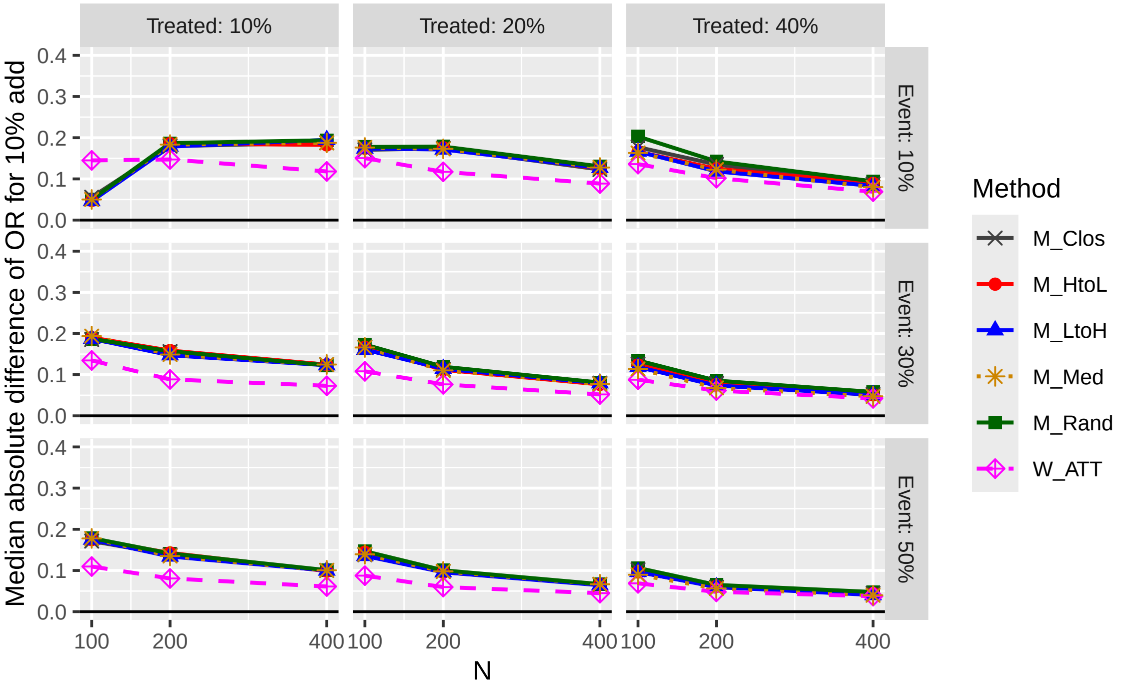

Figure S211. Median absolute difference of OR for 10% data addition (multimodal continuous covariate, matching ratio 1:1, true OR: 0.75, c statistic: 0.6).

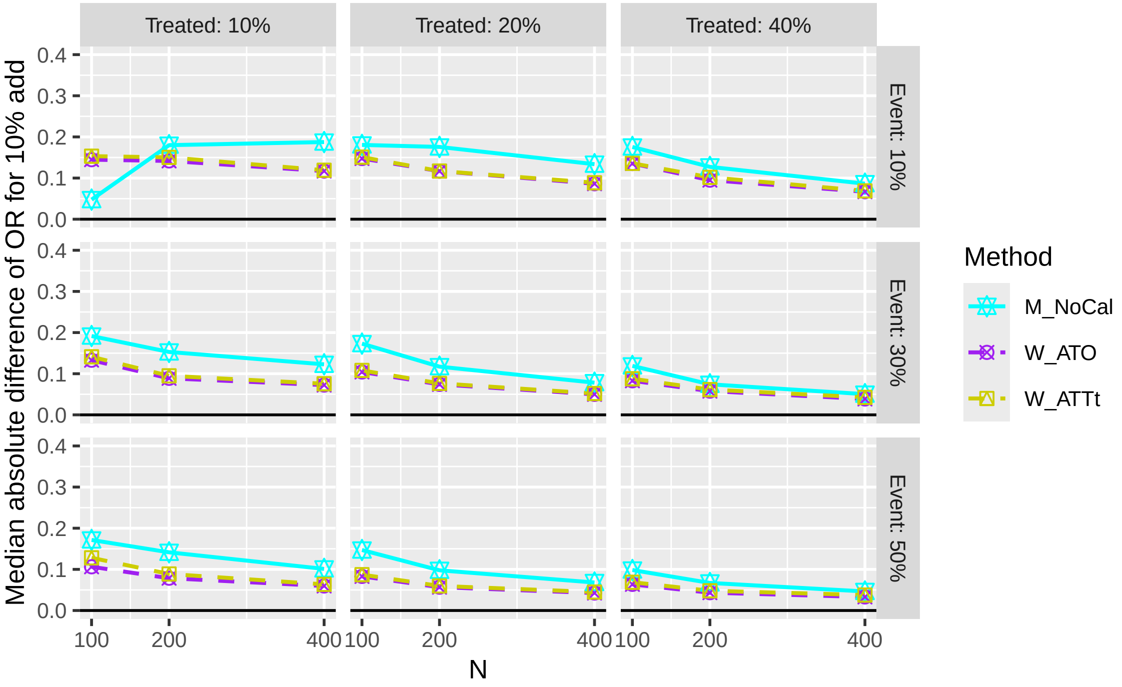

Figure S212. Median absolute difference of OR for 10% data addition (multimodal continuous covariate, matching ratio 1:1, true OR: 0.75, c statistic: 0.6); other methods.

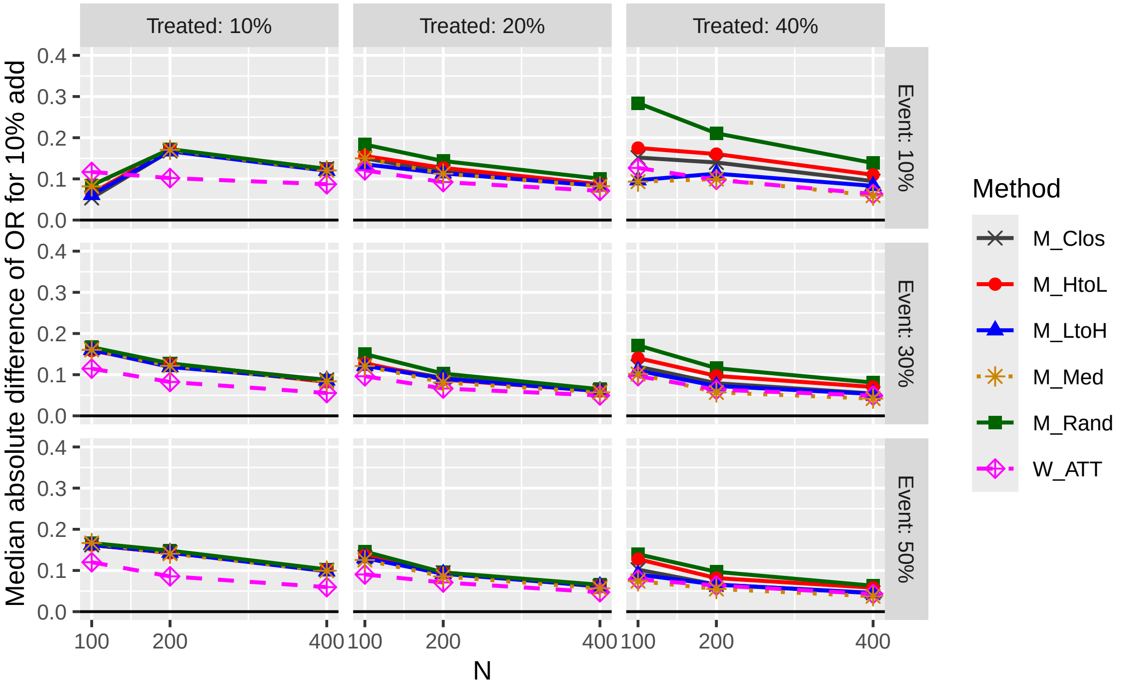

Figure S213. Median absolute difference of OR for 10% data addition (multimodal continuous covariate, matching ratio 1:1, true OR: 0.5, c statistic: 0.85).

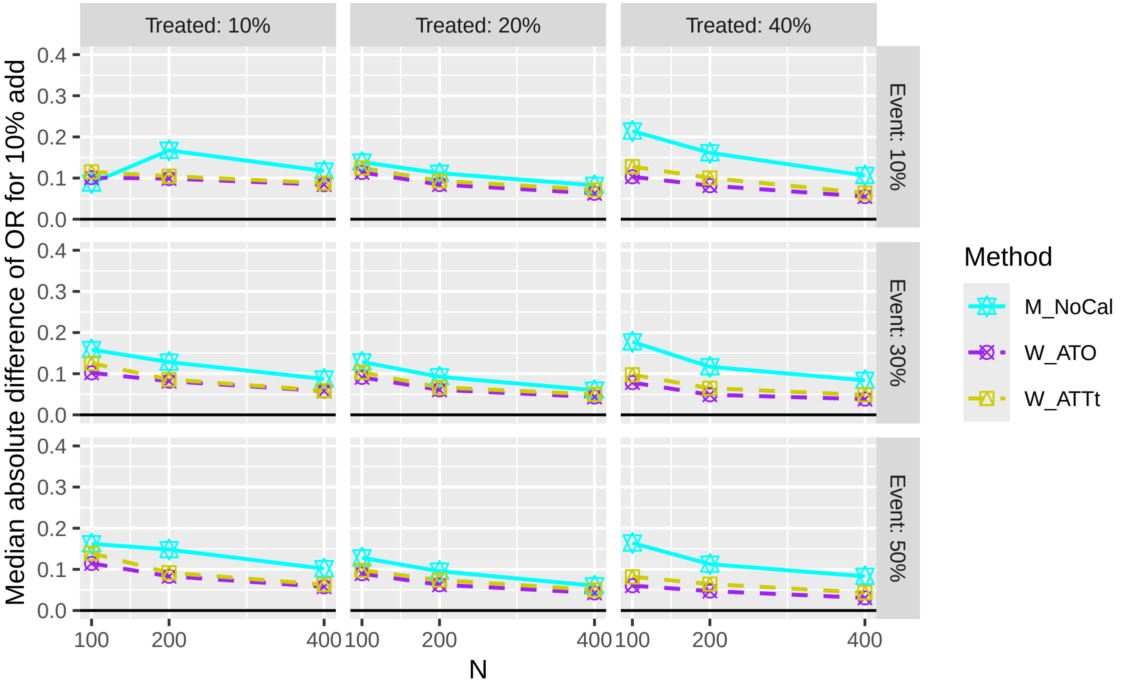

Figure S214. Median absolute difference of OR for 10% data addition (multimodal continuous covariate, matching ratio 1:1, true OR: 0.5, c statistic: 0.85); other methods.

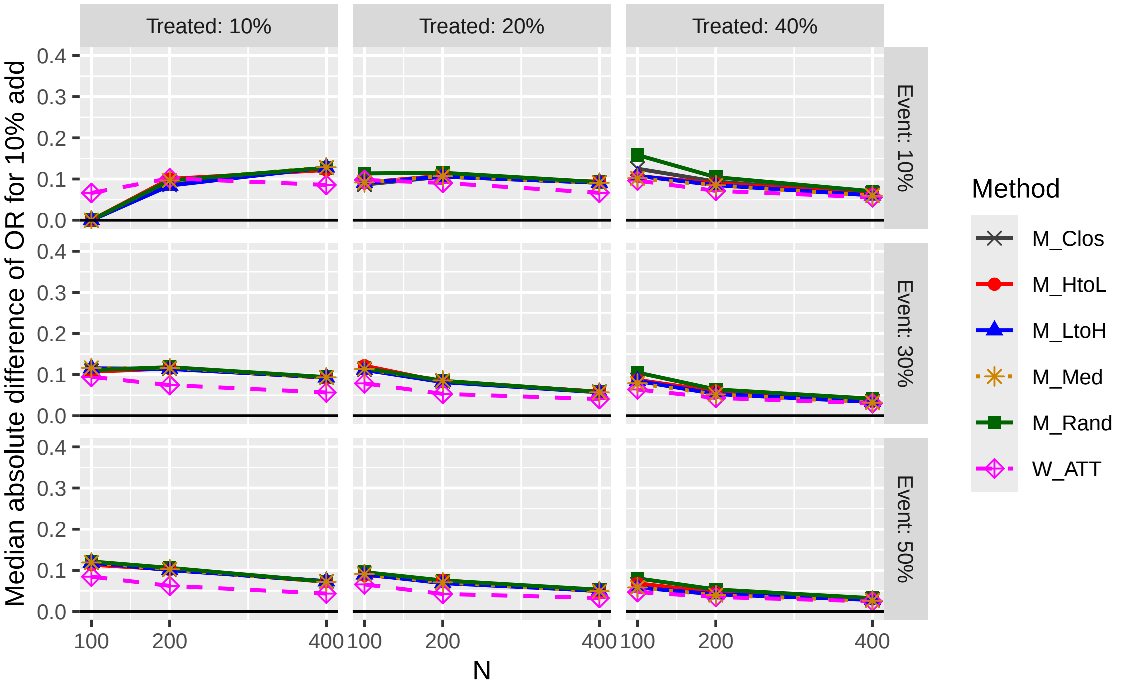

Figure S215. Median absolute difference of OR for 10% data addition (multimodal continuous covariate, matching ratio 1:1, true OR: 0.5, c statistic: 0.6).

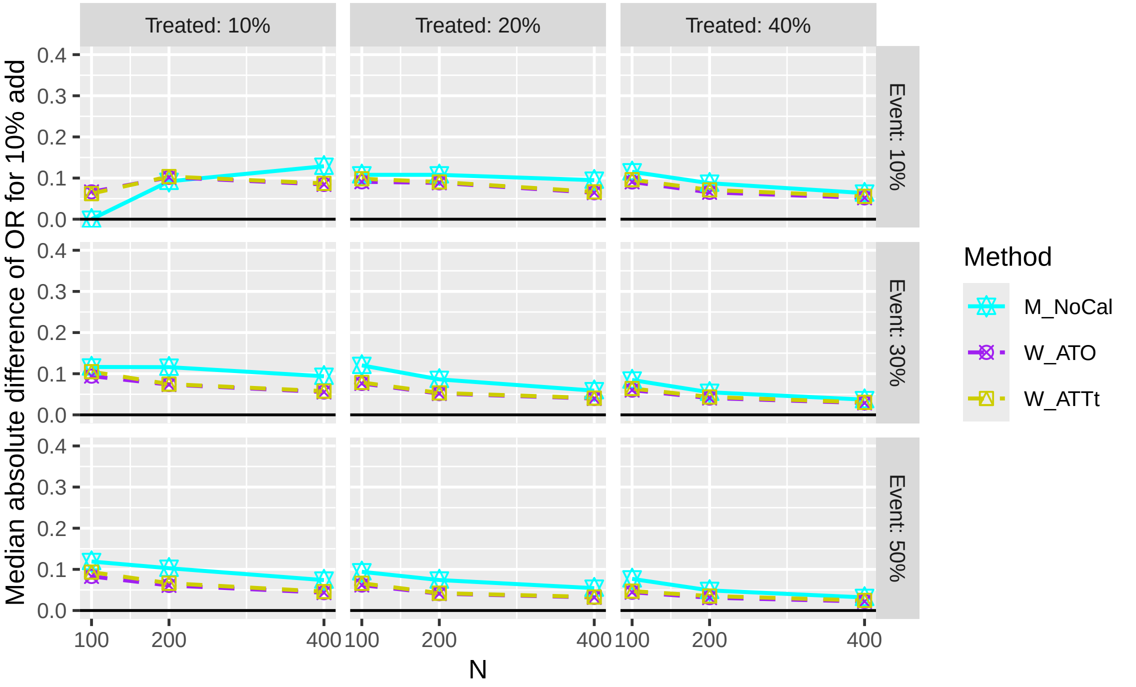

Figure S216. Median absolute difference of OR for 10% data addition (multimodal continuous covariate, matching ratio 1:1, true OR: 0.5, c statistic: 0.6); other methods.

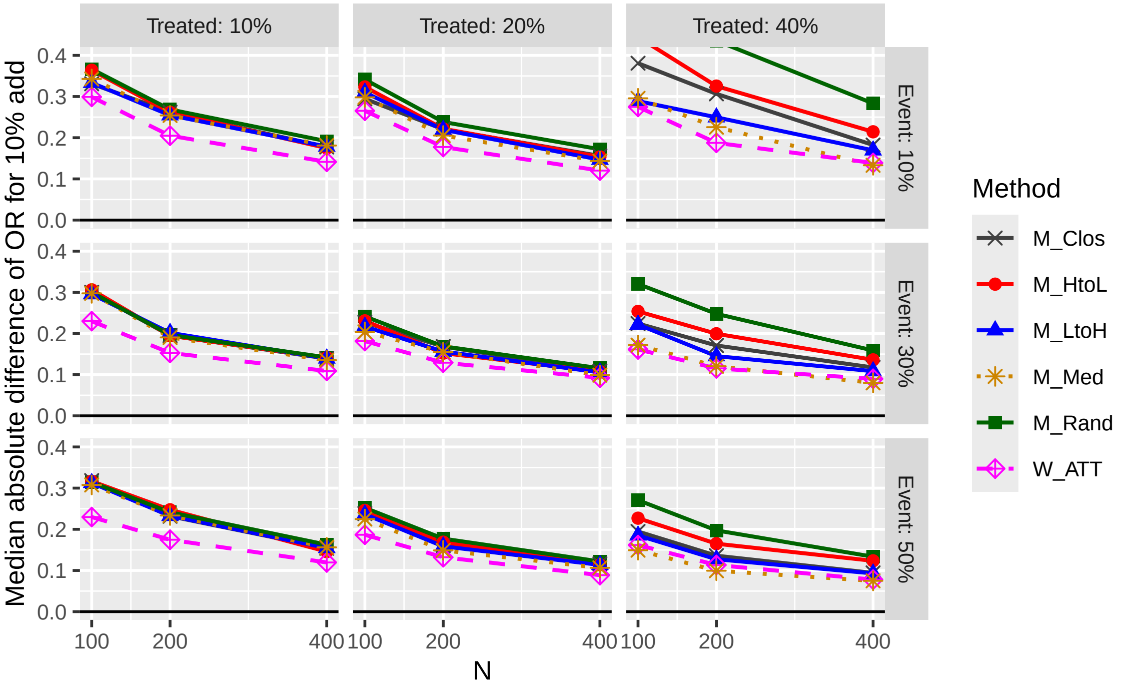

Figure S217. Median absolute difference of OR for 10% data addition (multimodal continuous covariate, matching ratio 1:2, true OR: 1, c statistic: 0.85).

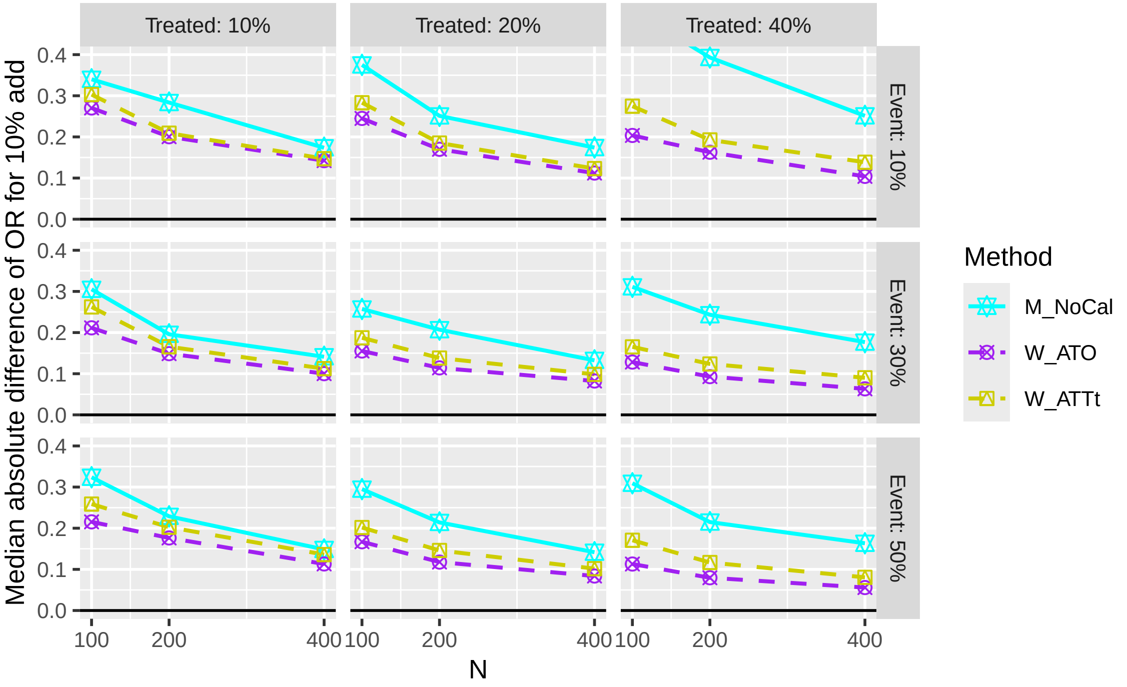

Figure S218. Median absolute difference of OR for 10% data addition (multimodal continuous covariate, matching ratio 1:2, true OR: 1, c statistic: 0.85); other methods.

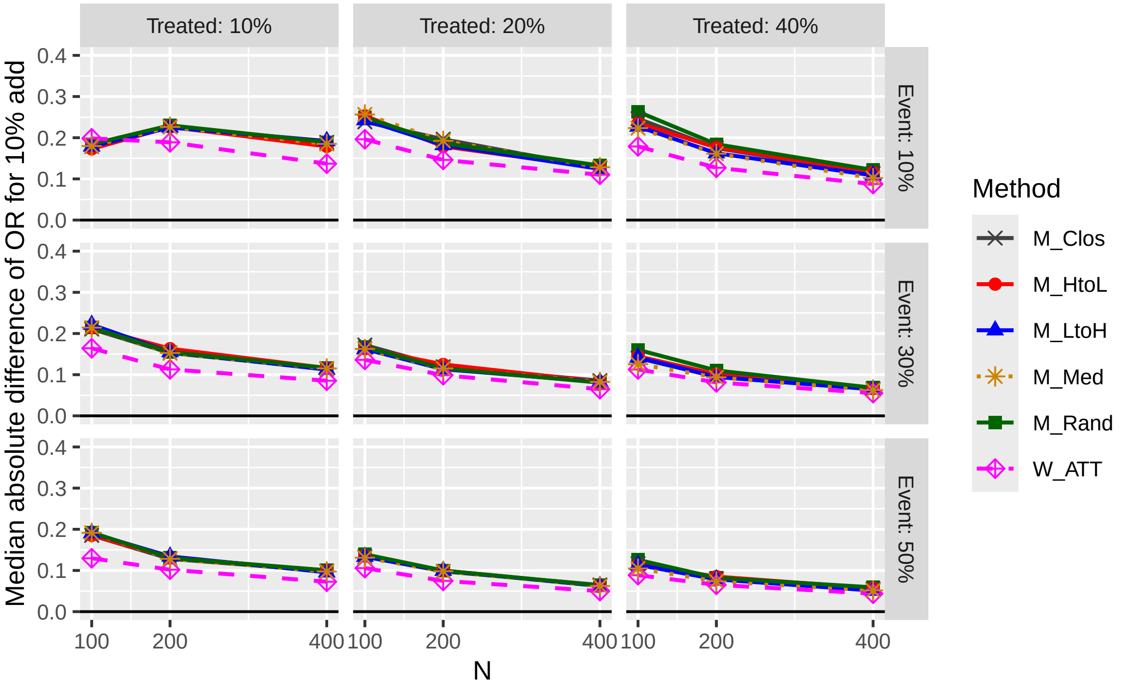

Figure S219. Median absolute difference of OR for 10% data addition (multimodal continuous covariate, matching ratio 1:2, true OR: 1, c statistic: 0.6).

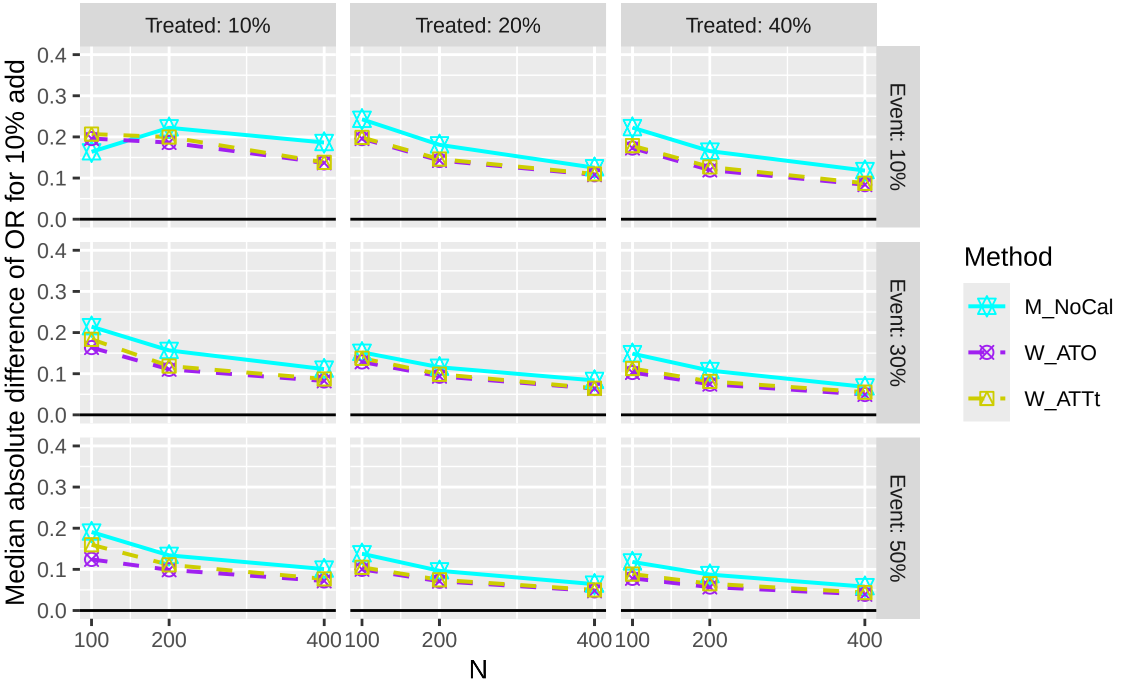

Figure S220. Median absolute difference of OR for 10% data addition (multimodal continuous covariate, matching ratio 1:2, true OR: 1, c statistic: 0.6); other methods.

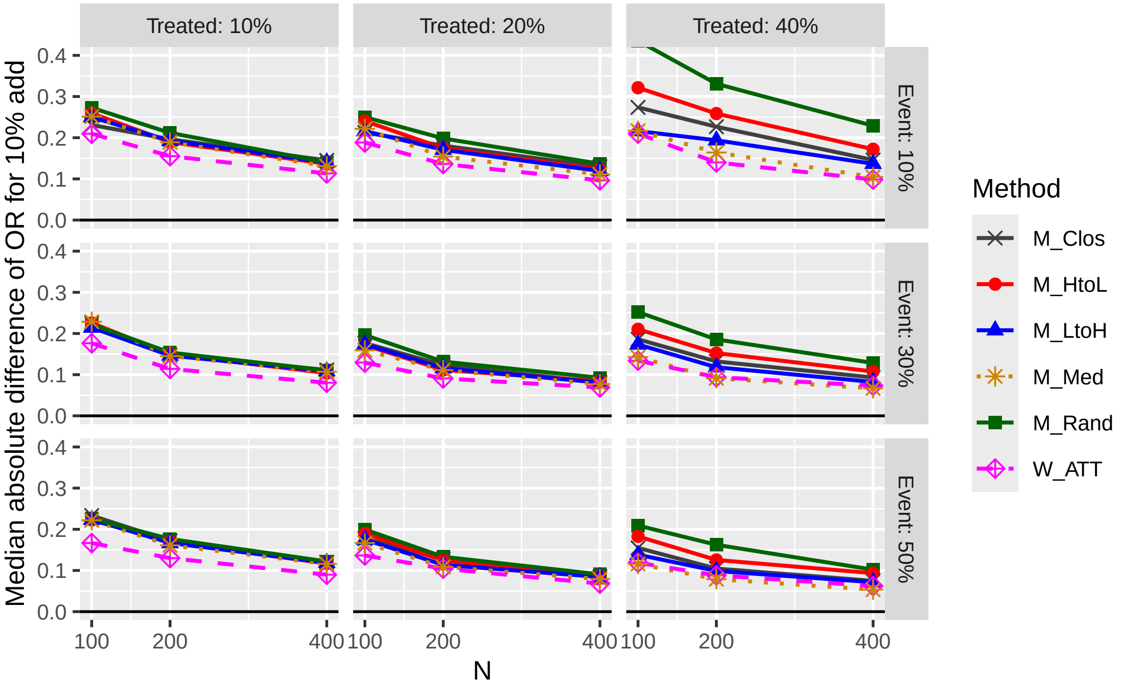

Figure S221. Median absolute difference of OR for 10% data addition (multimodal continuous covariate, matching ratio 1:2, true OR: 0.75, c statistic: 0.85).

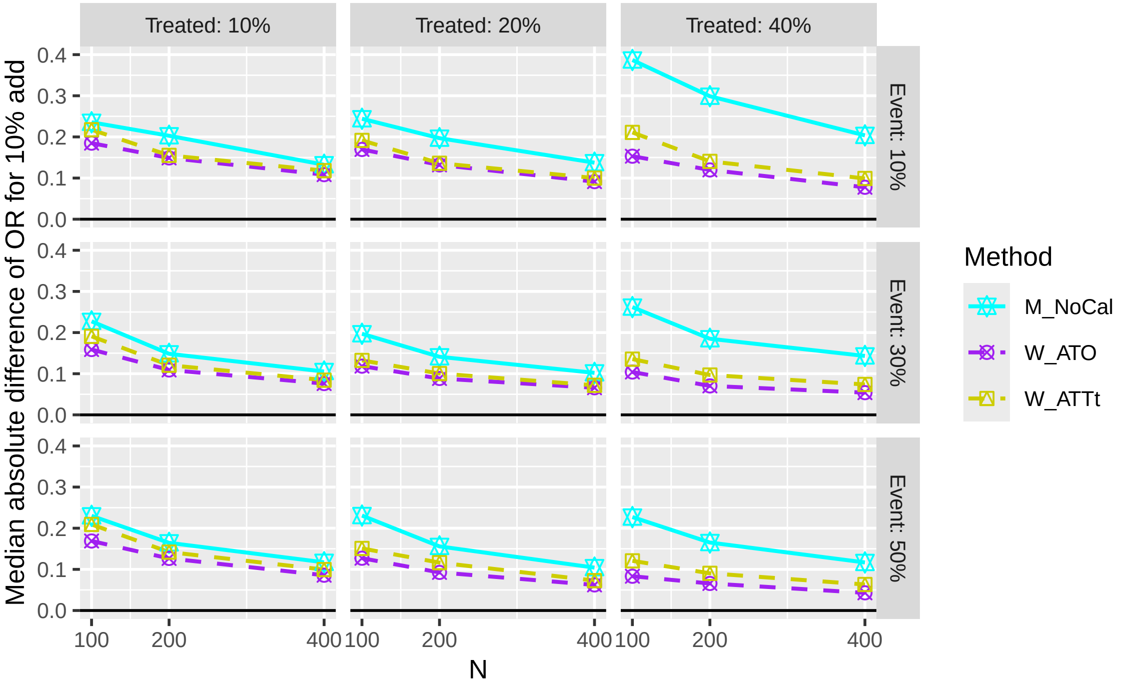

Figure S222. Median absolute difference of OR for 10% data addition (multimodal continuous covariate, matching ratio 1:2, true OR: 0.75, c statistic: 0.85); other methods.

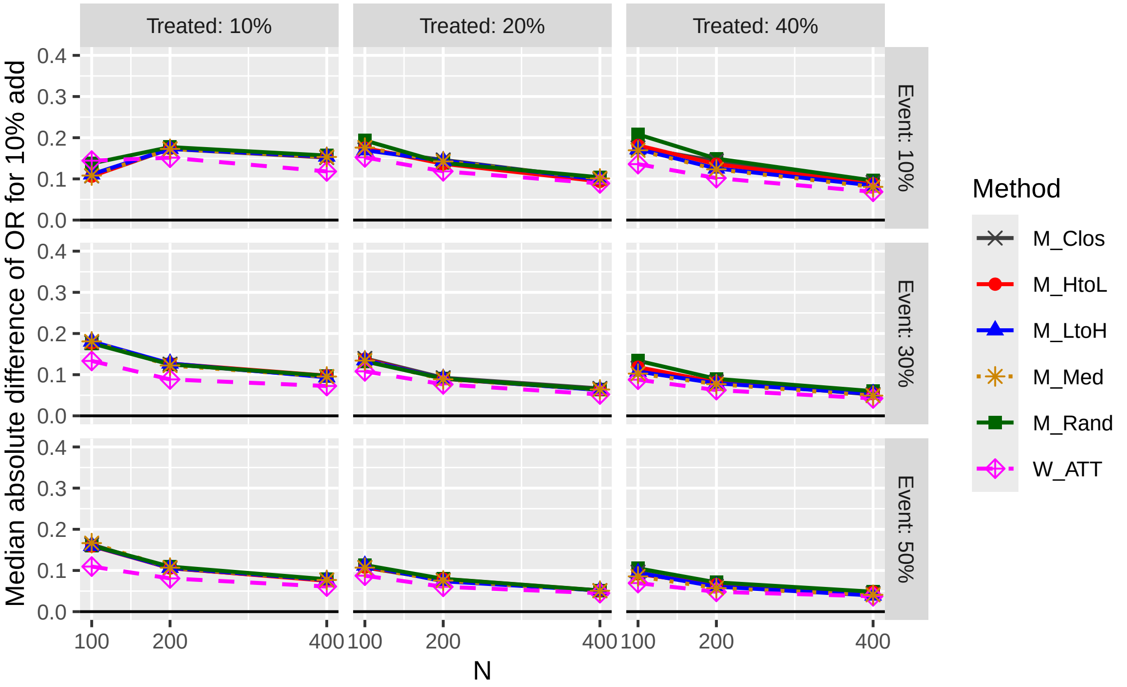

Figure S223. Median absolute difference of OR for 10% data addition (multimodal continuous covariate, matching ratio 1:2, true OR: 0.75, c statistic: 0.6).

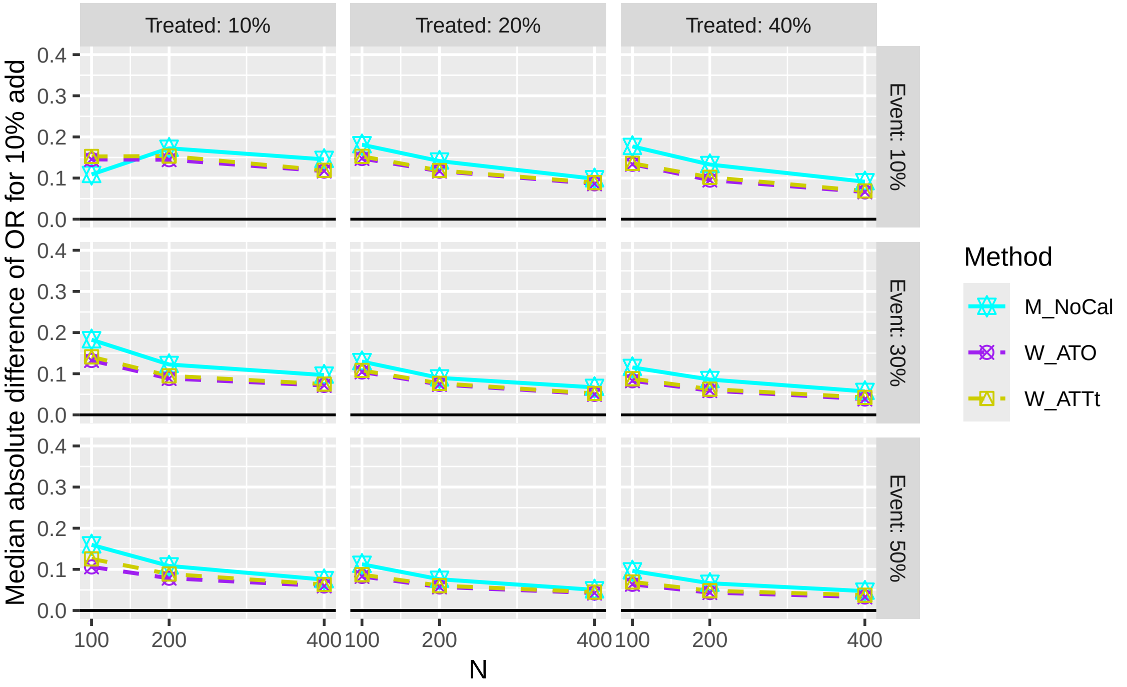

Figure S224. Median absolute difference of OR for 10% data addition (multimodal continuous covariate, matching ratio 1:2, true OR: 0.75, c statistic: 0.6); other methods.

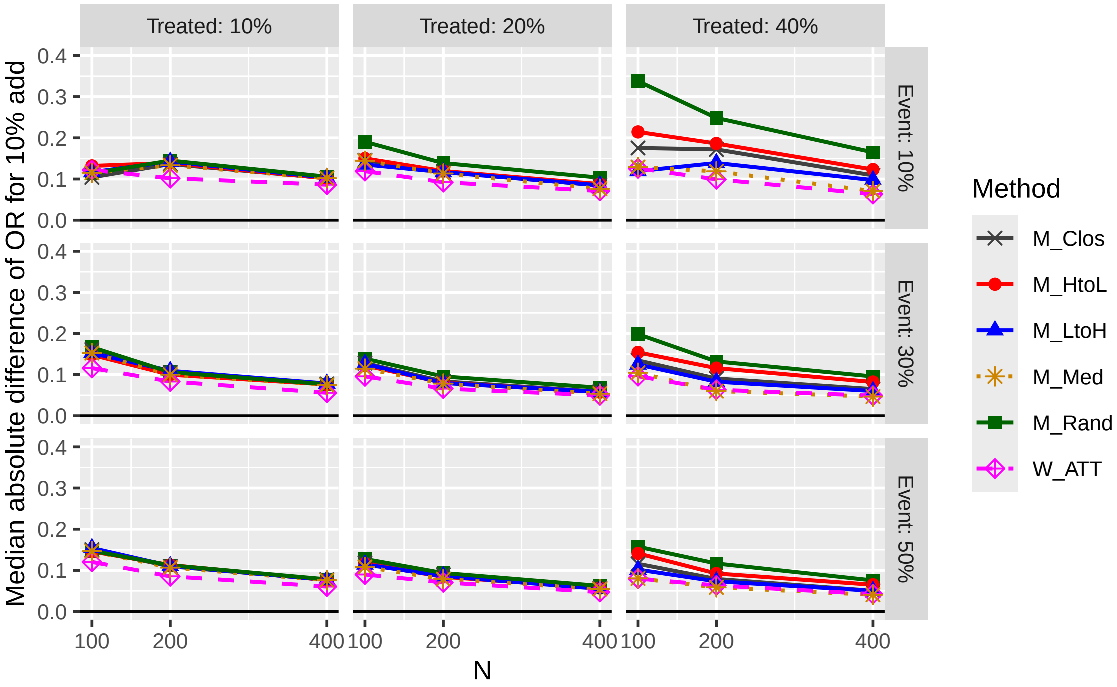

Figure S225. Median absolute difference of OR for 10% data addition (multimodal continuous covariate, matching ratio 1:2, true OR: 0.5, c statistic: 0.85).

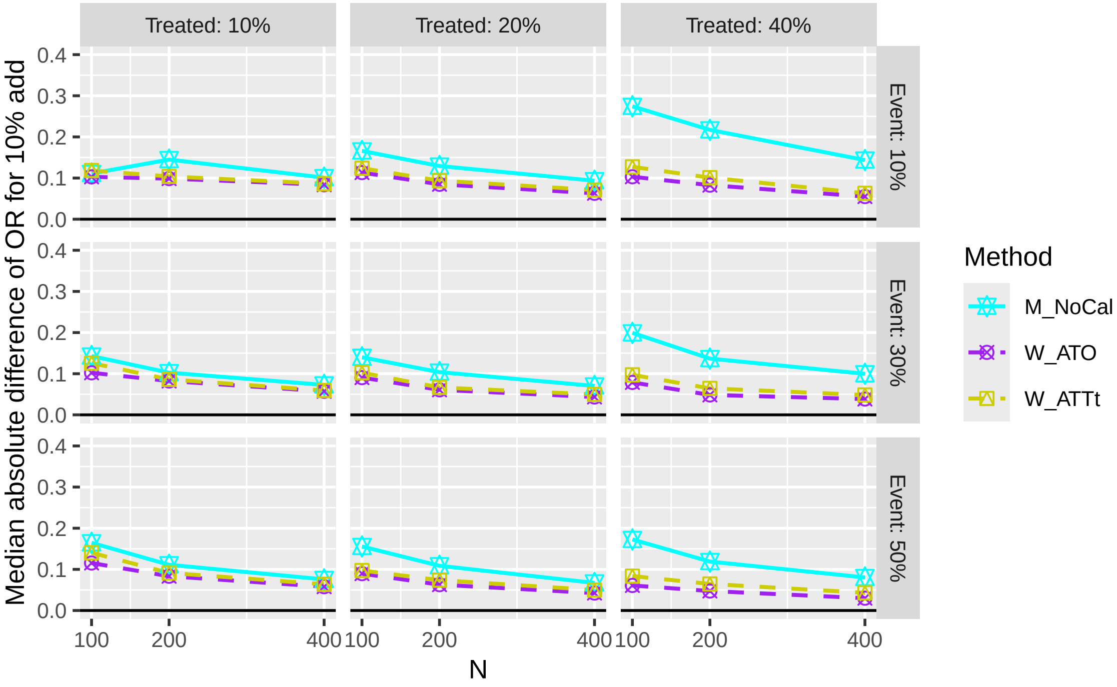

Figure S226. Median absolute difference of OR for 10% data addition (multimodal continuous covariate, matching ratio 1:2, true OR: 0.5, c statistic: 0.85); other methods.

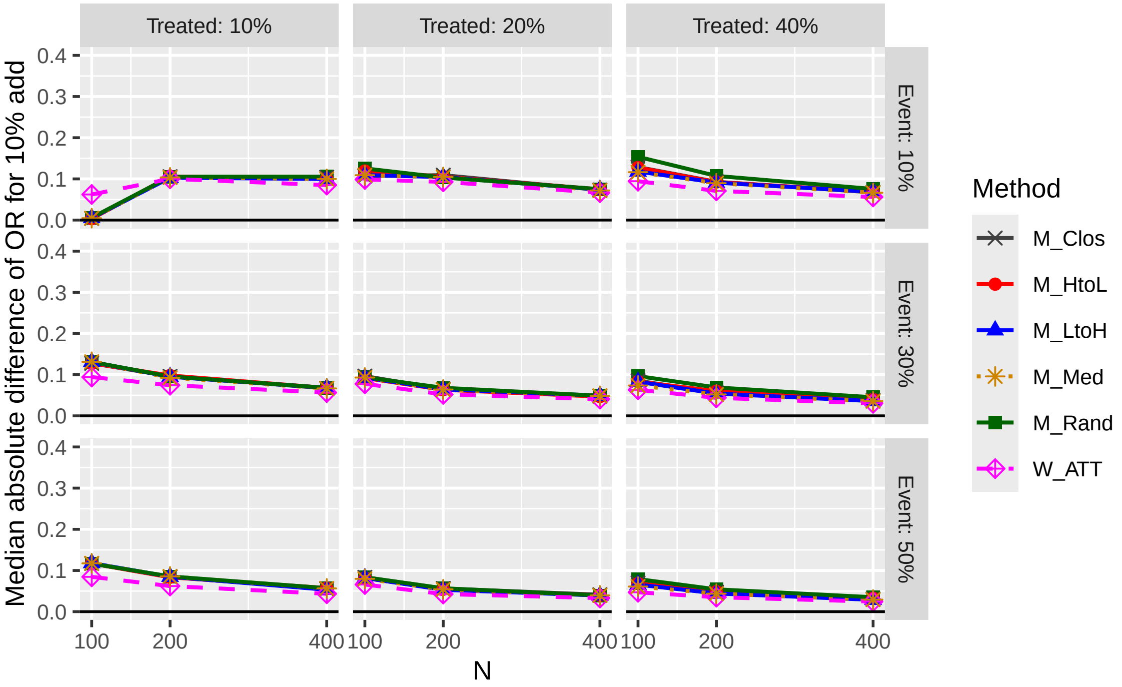

Figure S227. Median absolute difference of OR for 10% data addition (multimodal continuous covariate, matching ratio 1:2, true OR: 0.5, c statistic: 0.6).

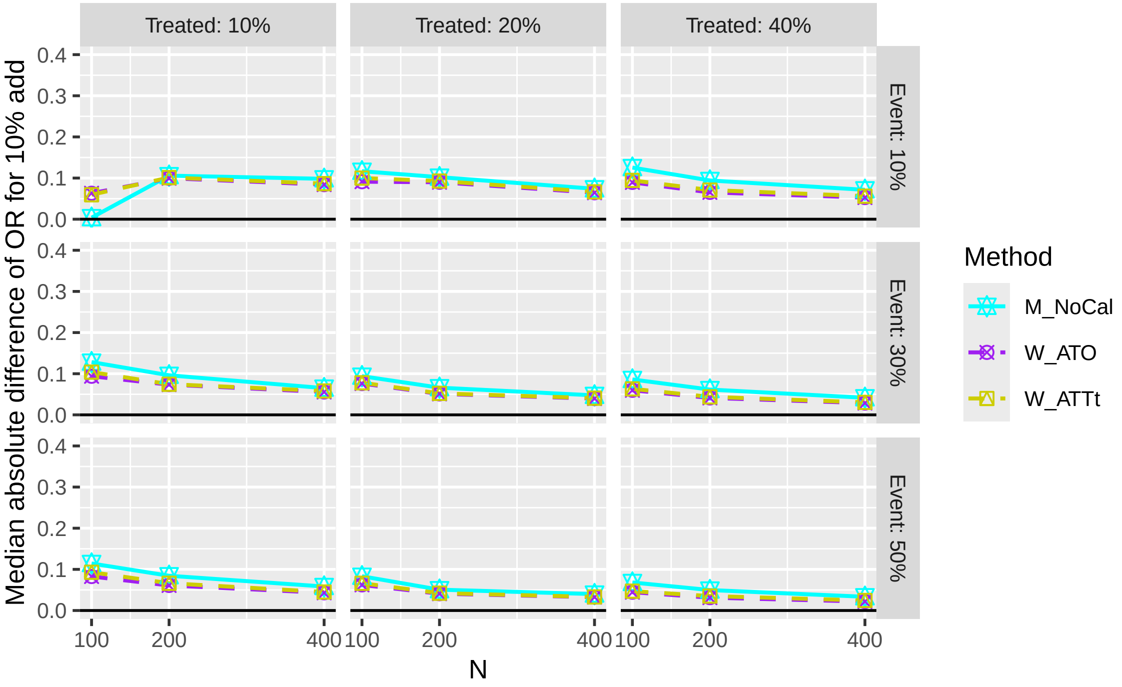

Figure S228. Median absolute difference of OR for 10% data addition (multimodal continuous covariate, matching ratio 1:2, true OR: 0.5, c statistic: 0.6); other methods.

S5. Coverage probability of confidence interval for OR (caliper: 25%)

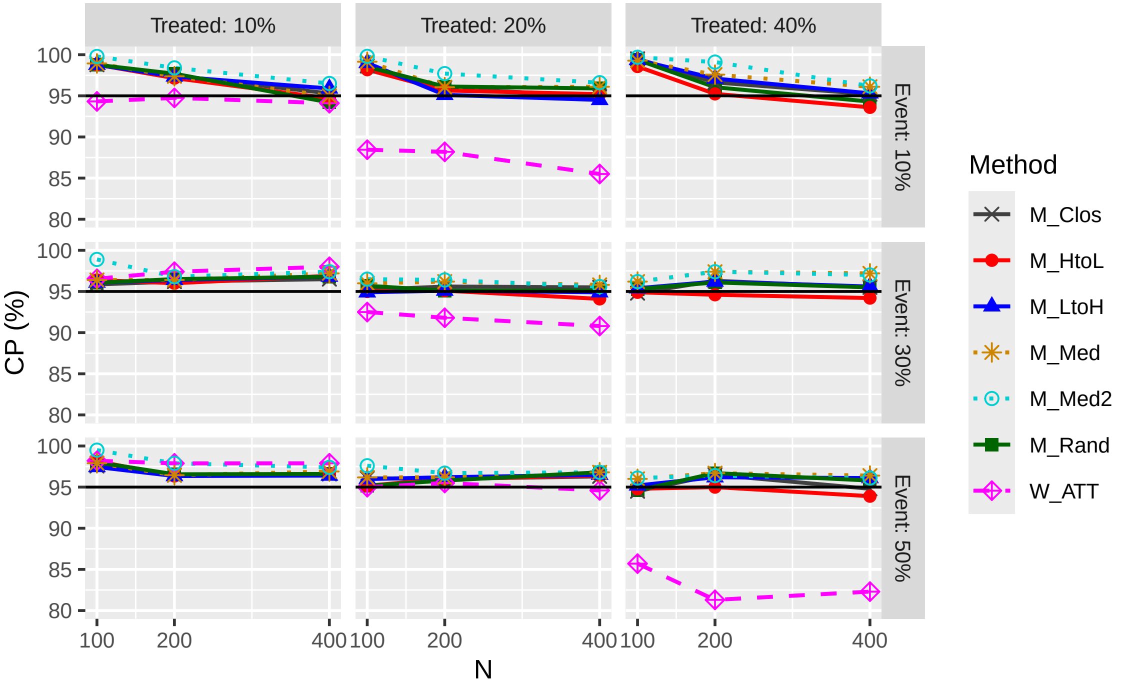

Figure S229. Coverage probability of confidence interval for OR (unimodal continuous covariate, matching ratio 1:1, true OR: 1, c statistic: 0.85, naive inference).

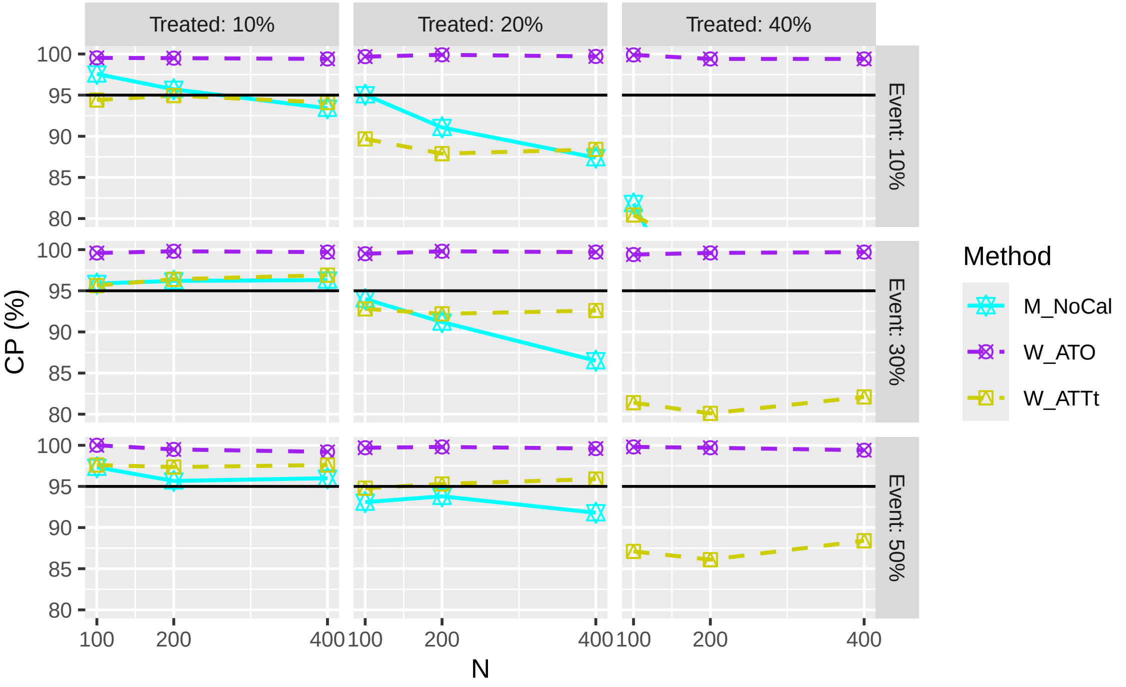

Figure S230. Coverage probability of confidence interval for OR (unimodal continuous covariate, matching ratio 1:1, true OR: 1, c statistic: 0.85, naive inference); other methods.

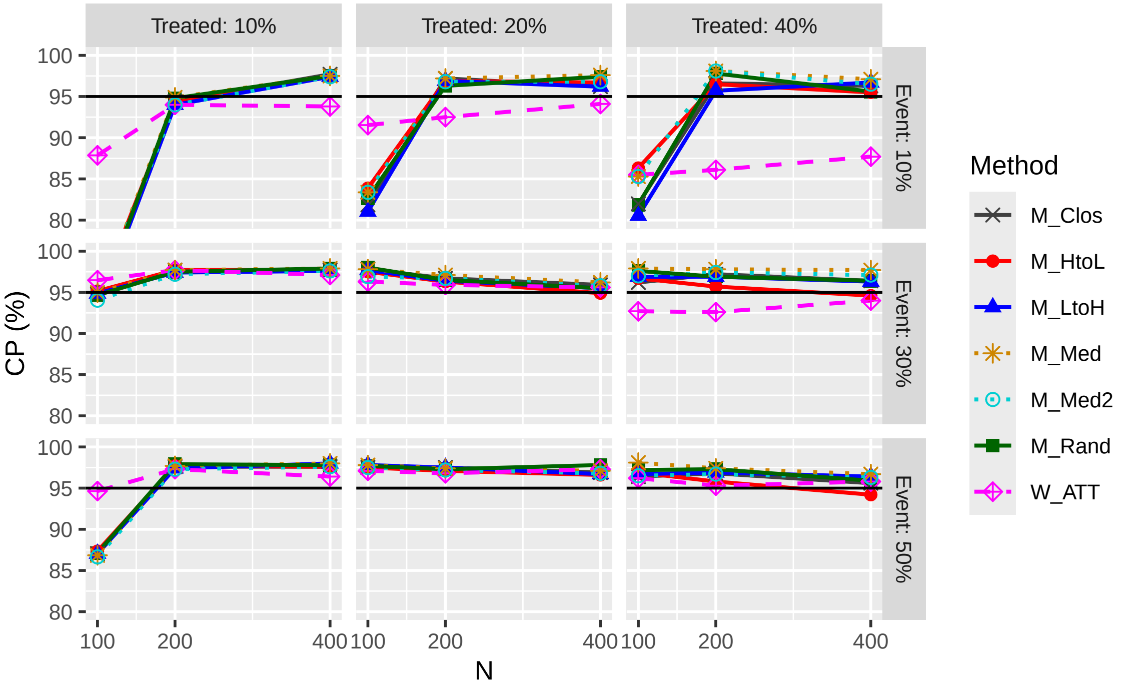

Figure S231. Coverage probability of confidence interval for OR (unimodal continuous covariate, matching ratio 1:1, true OR: 1, c statistic: 0.85, robust inference).

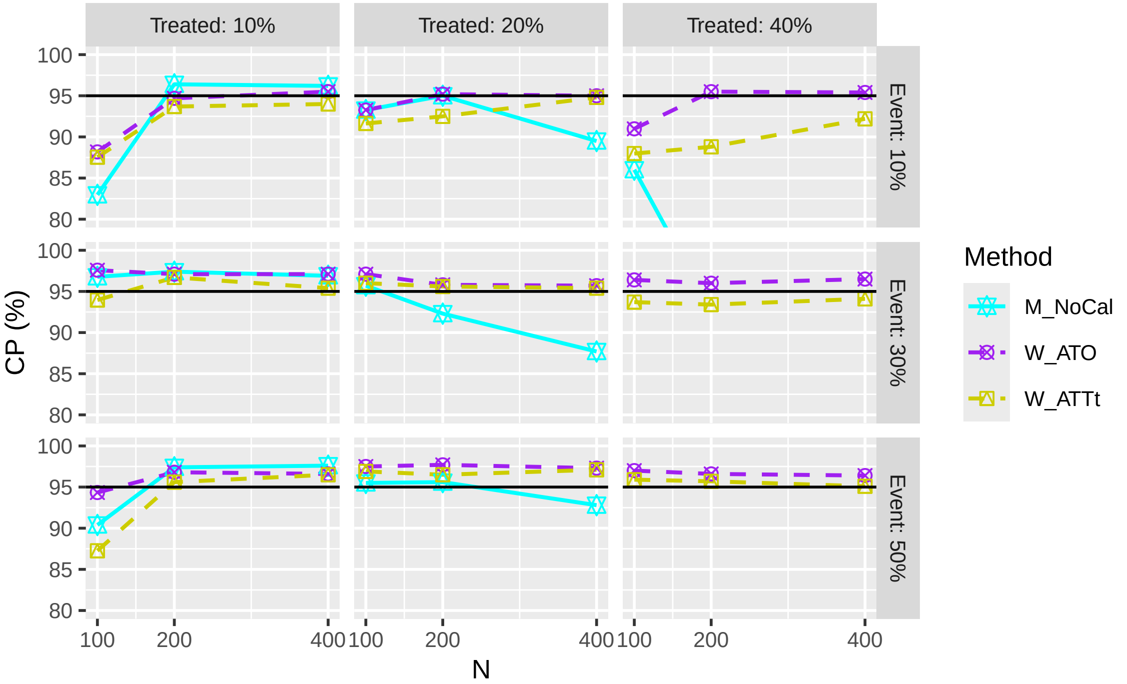

Figure S232. Coverage probability of confidence interval for OR (unimodal continuous covariate, matching ratio 1:1, true OR: 1, c statistic: 0.85, robust inference); other methods.

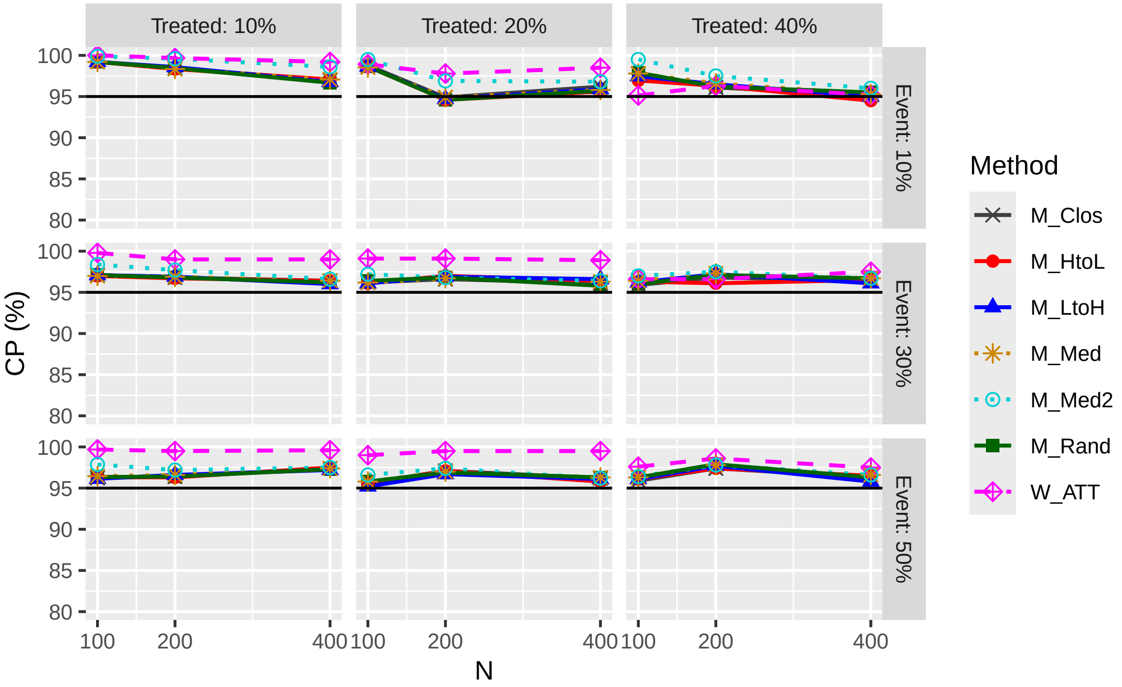

Figure S233. Coverage probability of confidence interval for OR (unimodal continuous covariate, matching ratio 1:1, true OR: 1, c statistic: 0.6, naive inference).

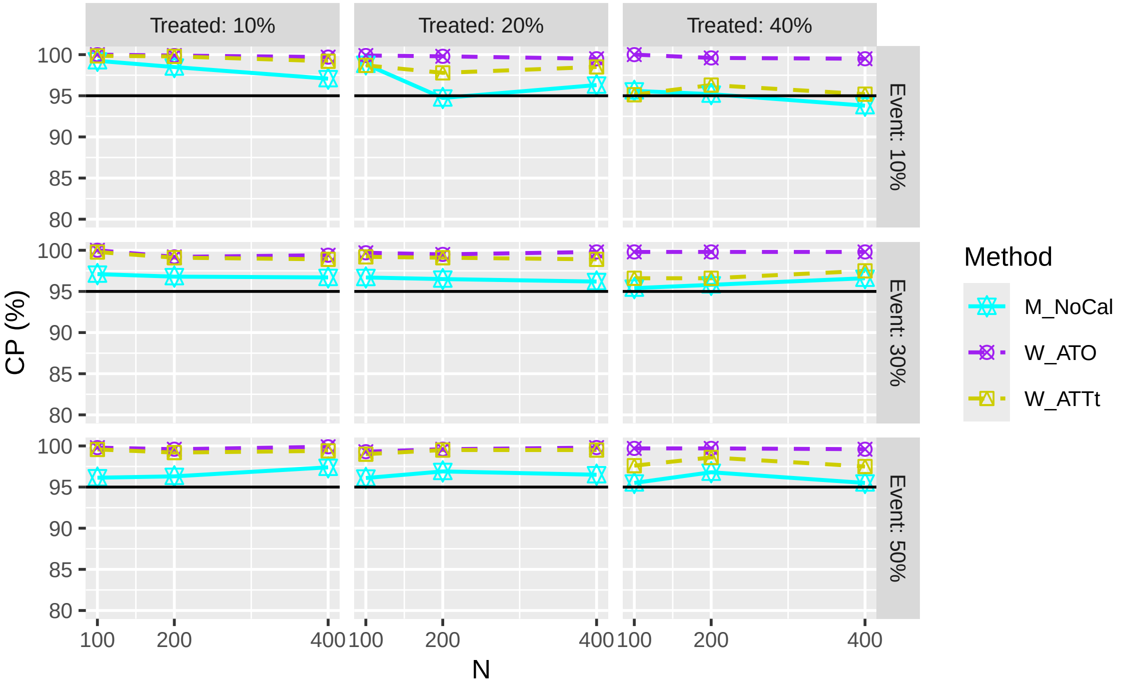

Figure S234. Coverage probability of confidence interval for OR (unimodal continuous covariate, matching ratio 1:1, true OR: 1, c statistic: 0.6, naive inference); other methods.

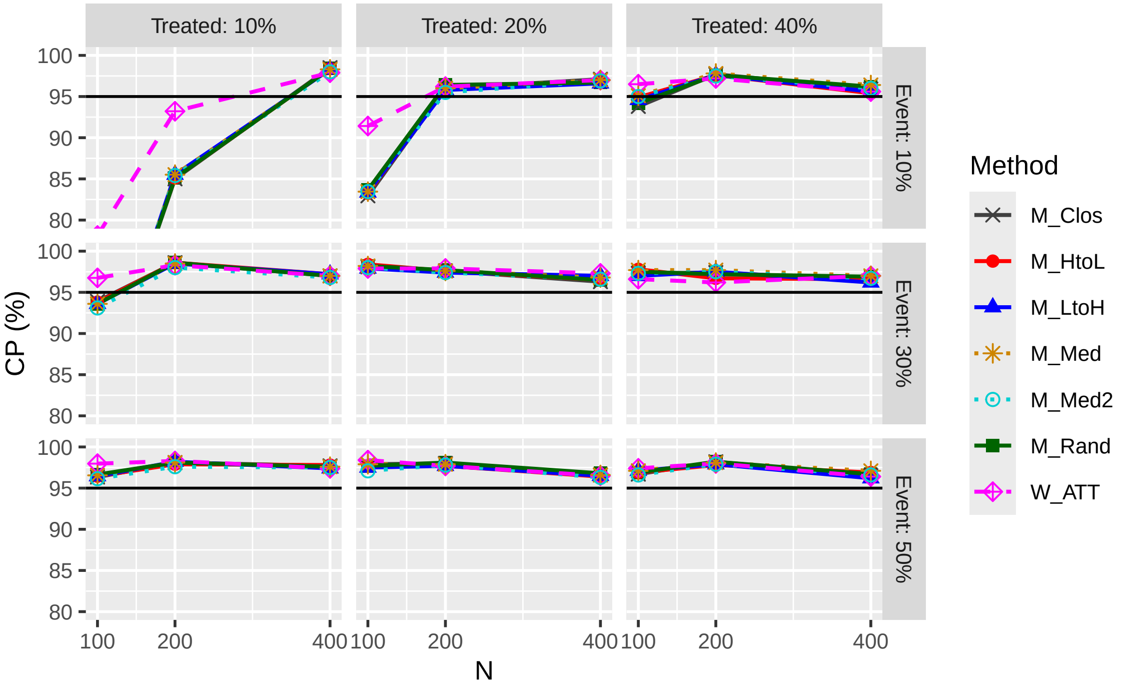

Figure S235. Coverage probability of confidence interval for OR (unimodal continuous covariate, matching ratio 1:1, true OR: 1, c statistic: 0.6, robust inference).

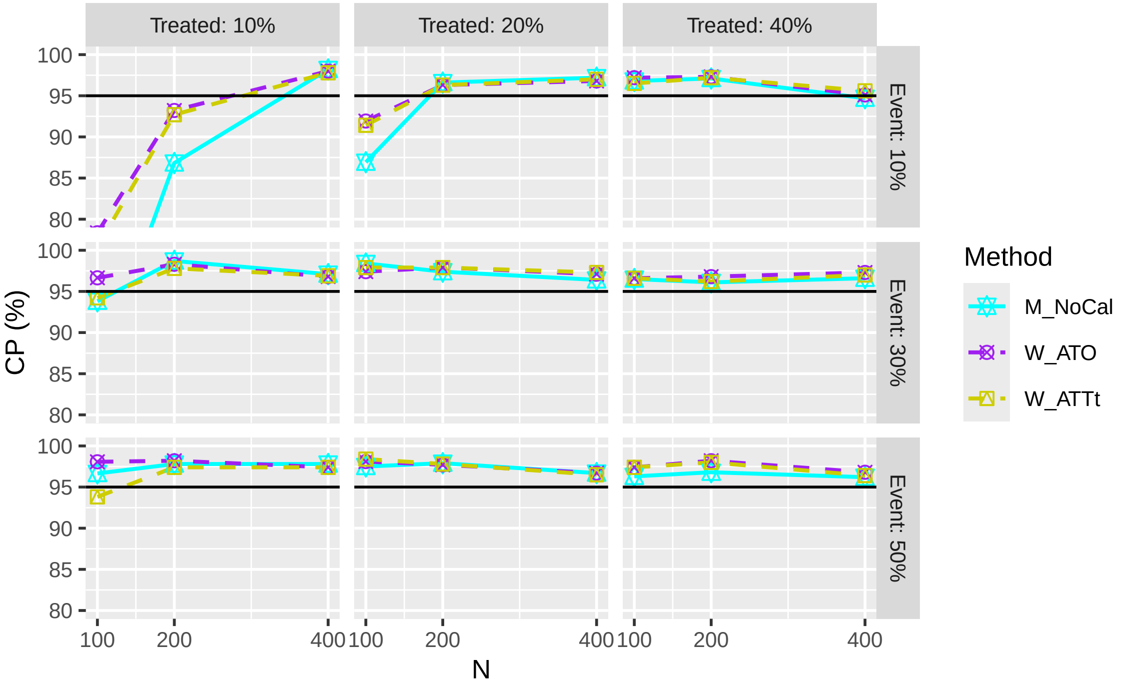

Figure S236. Coverage probability of confidence interval for OR (unimodal continuous covariate, matching ratio 1:1, true OR: 1, c statistic: 0.6, robust inference); other methods.

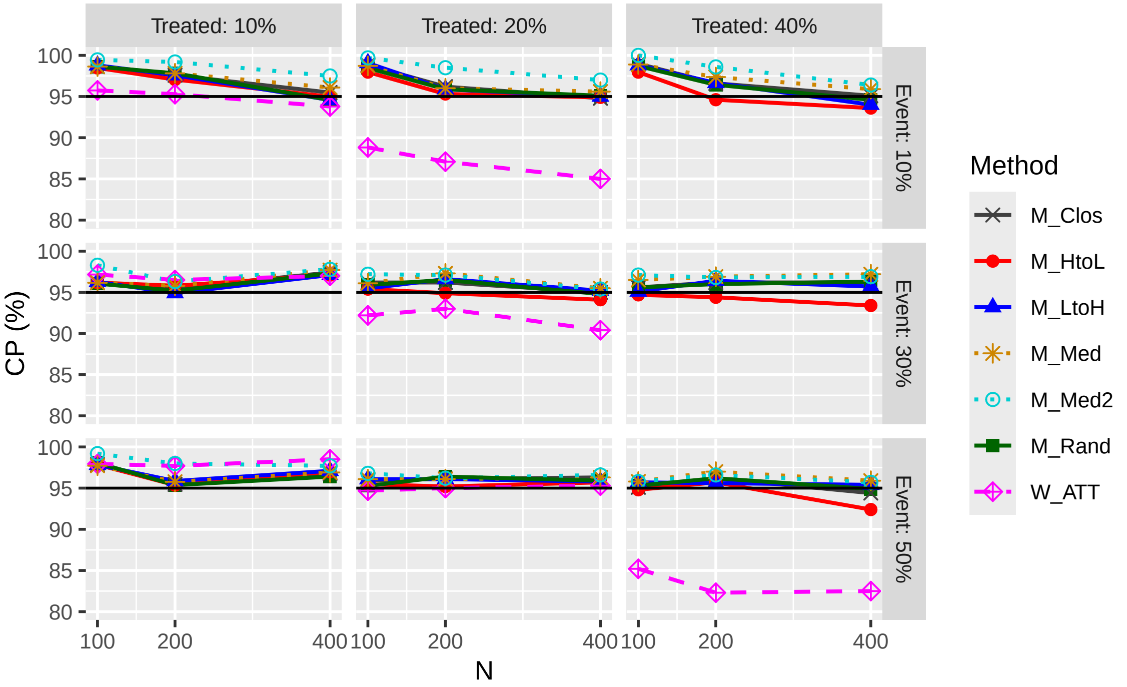

Figure S237. Coverage probability of confidence interval for OR (unimodal continuous covariate, matching ratio 1:1, true OR: 0.75, c statistic: 0.85, naive inference).

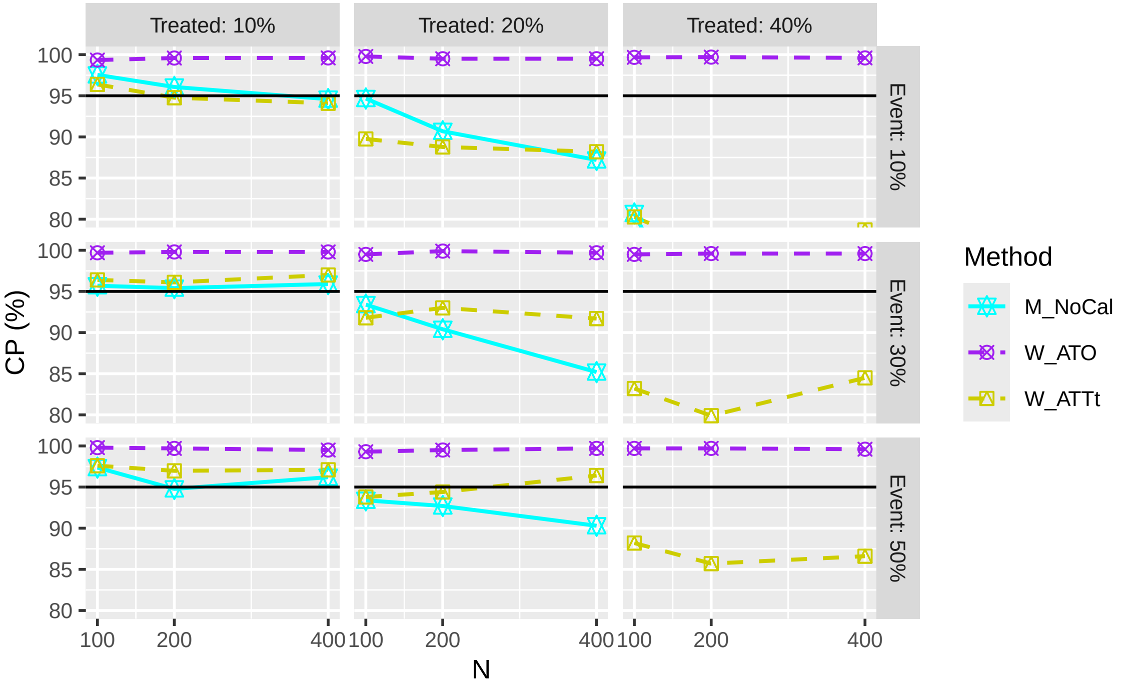

Figure S238. Coverage probability of confidence interval for OR (unimodal continuous covariate, matching ratio 1:1, true OR: 0.75, c statistic: 0.85, naive inference); other methods.

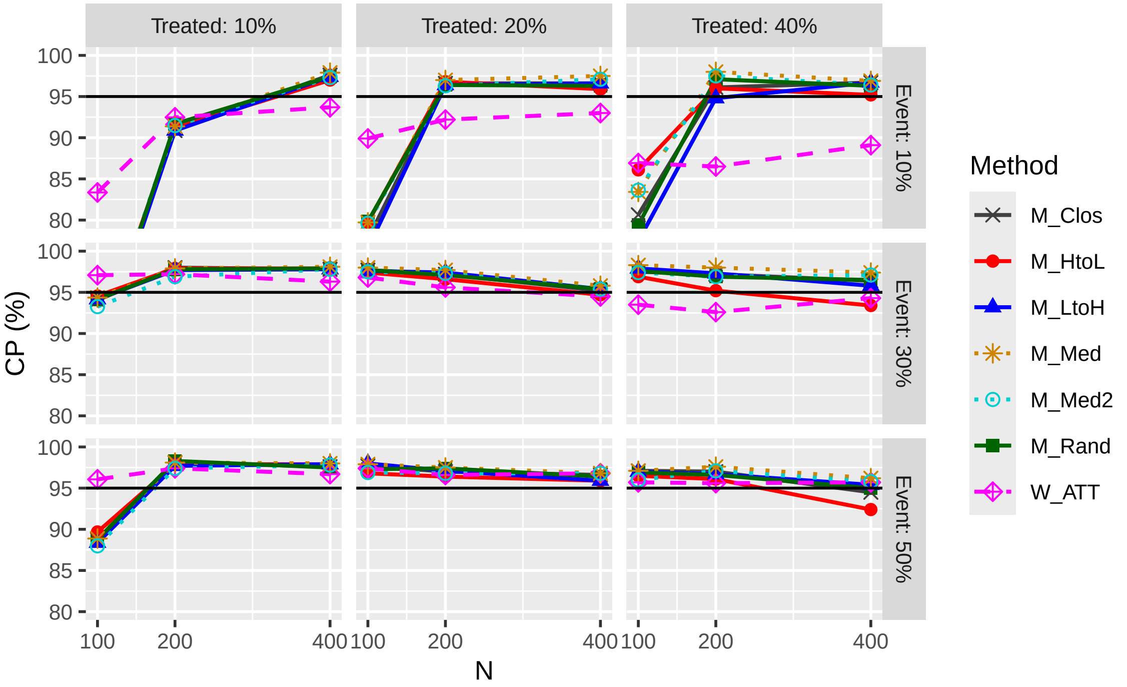

Figure S239. Coverage probability of confidence interval for OR (unimodal continuous covariate, matching ratio 1:1, true OR: 0.75, c statistic: 0.85, robust inference).

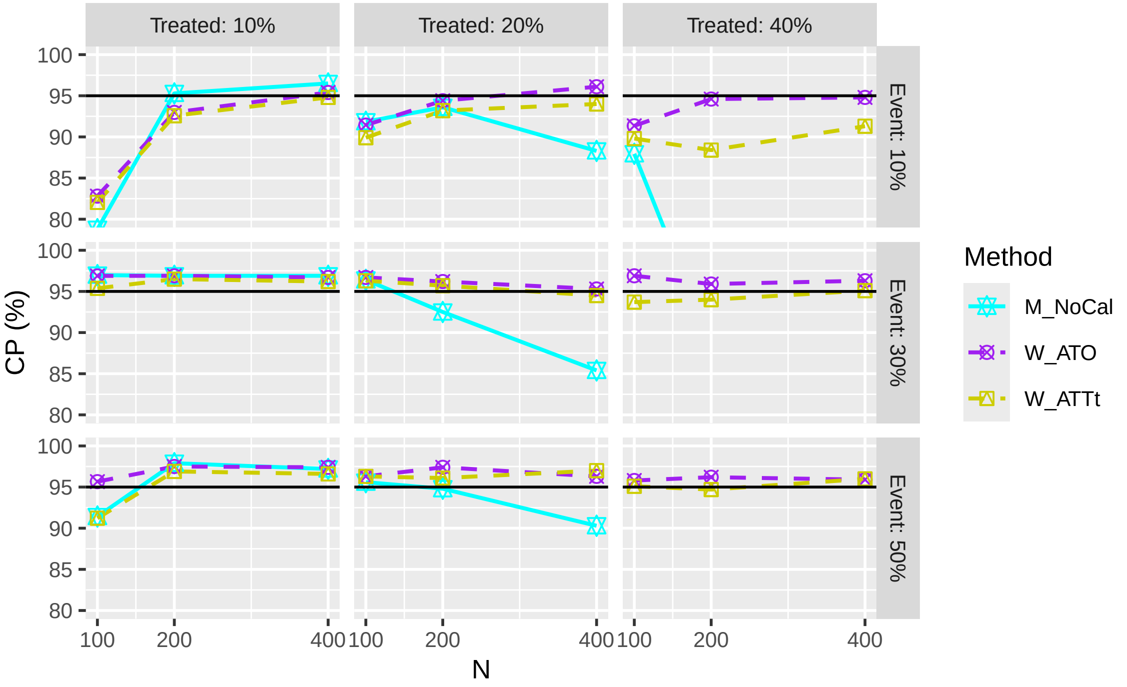

Figure S240. Coverage probability of confidence interval for OR (unimodal continuous covariate, matching ratio 1:1, true OR: 0.75, c statistic: 0.85, robust inference); other methods.

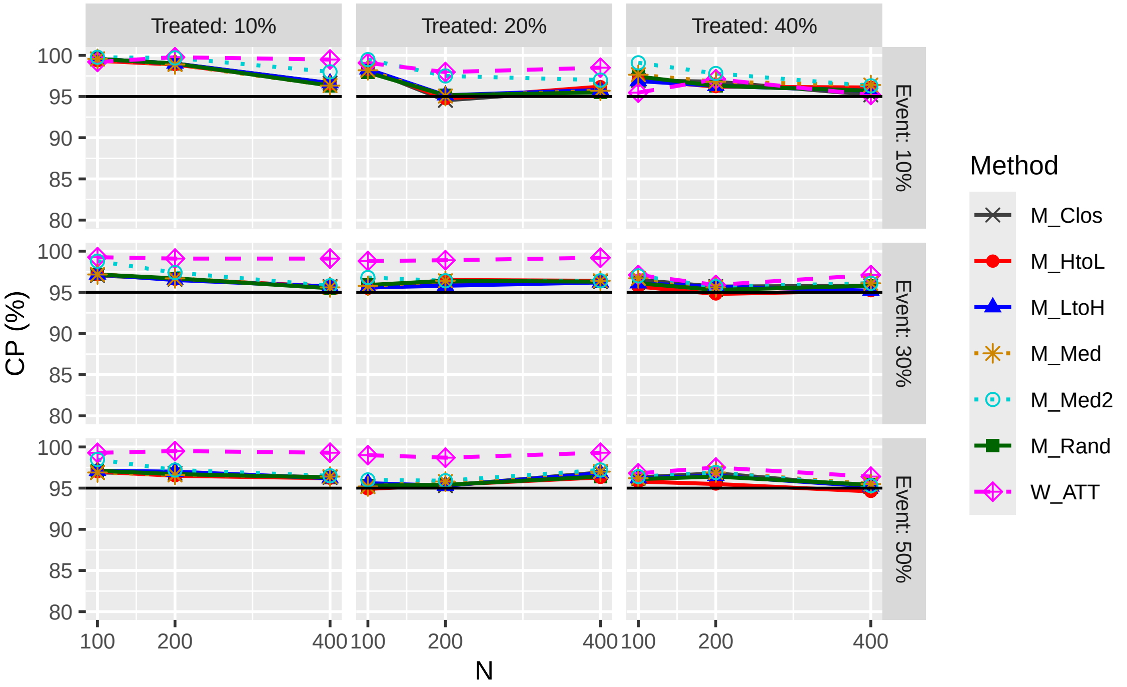

Figure S241. Coverage probability of confidence interval for OR (unimodal continuous covariate, matching ratio 1:1, true OR: 0.75, c statistic: 0.6, naive inference).

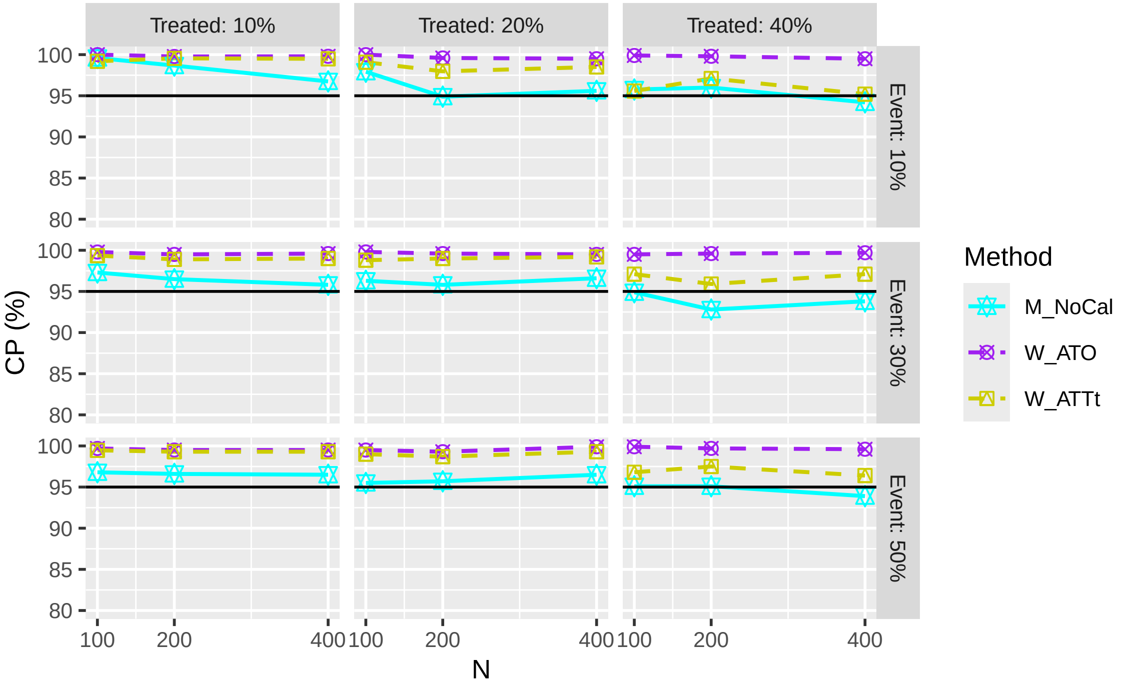

Figure S242. Coverage probability of confidence interval for OR (unimodal continuous covariate, matching ratio 1:1, true OR: 0.75, c statistic: 0.6, naive inference); other methods.

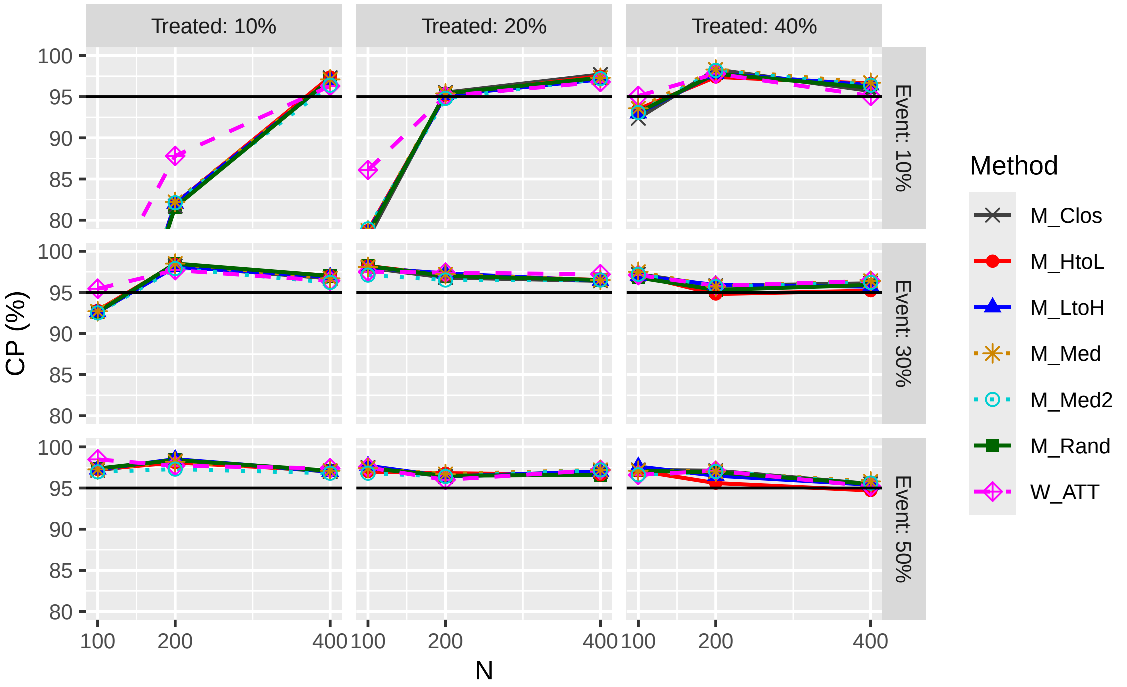

Figure S243. Coverage probability of confidence interval for OR (unimodal continuous covariate, matching ratio 1:1, true OR: 0.75, c statistic: 0.6, robust inference).

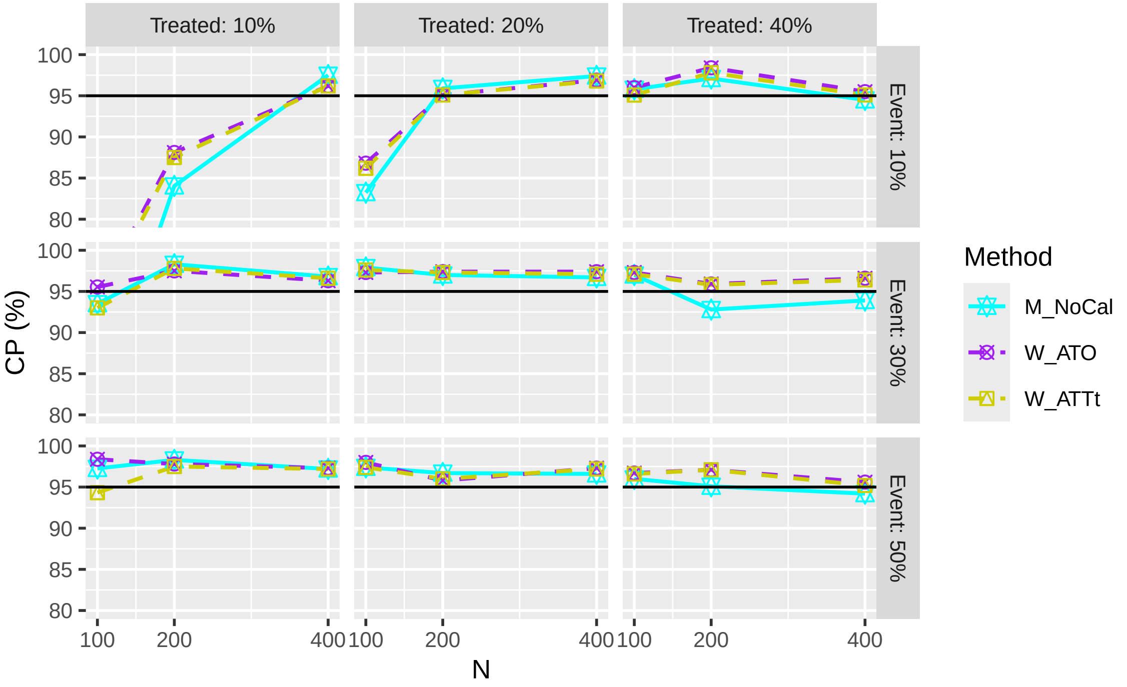

Figure S244. Coverage probability of confidence interval for OR (unimodal continuous covariate, matching ratio 1:1, true OR: 0.75, c statistic: 0.6, robust inference); other methods.

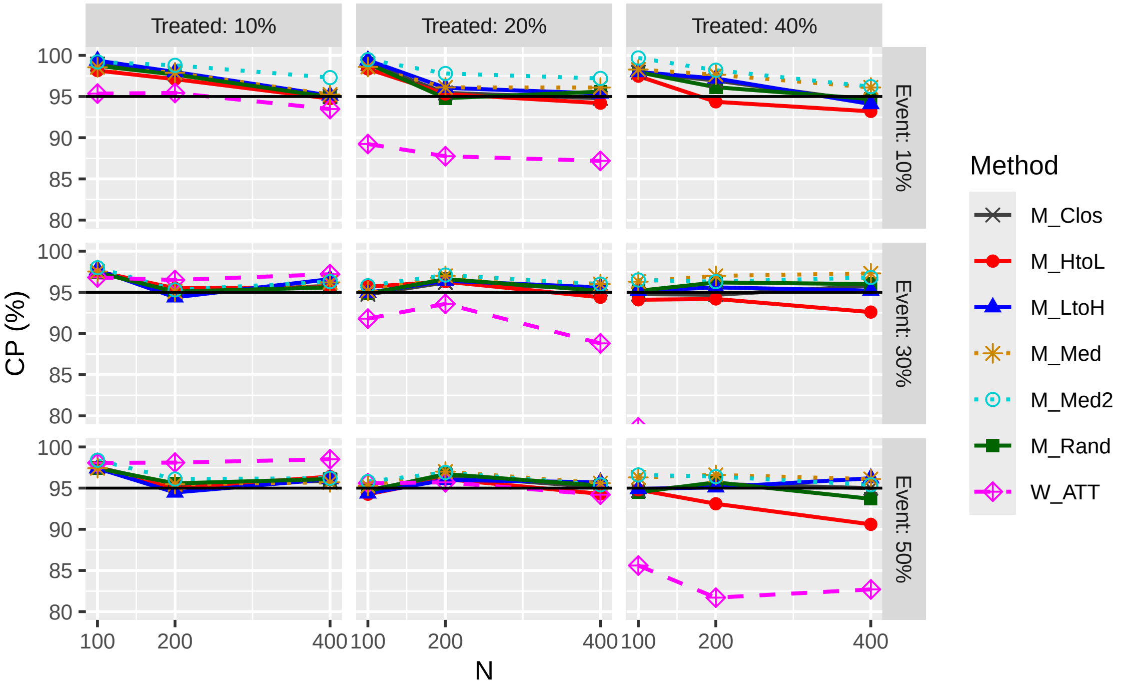

Figure S245. Coverage probability of confidence interval for OR (unimodal continuous covariate, matching ratio 1:1, true OR: 0.5, c statistic: 0.85, naive inference).

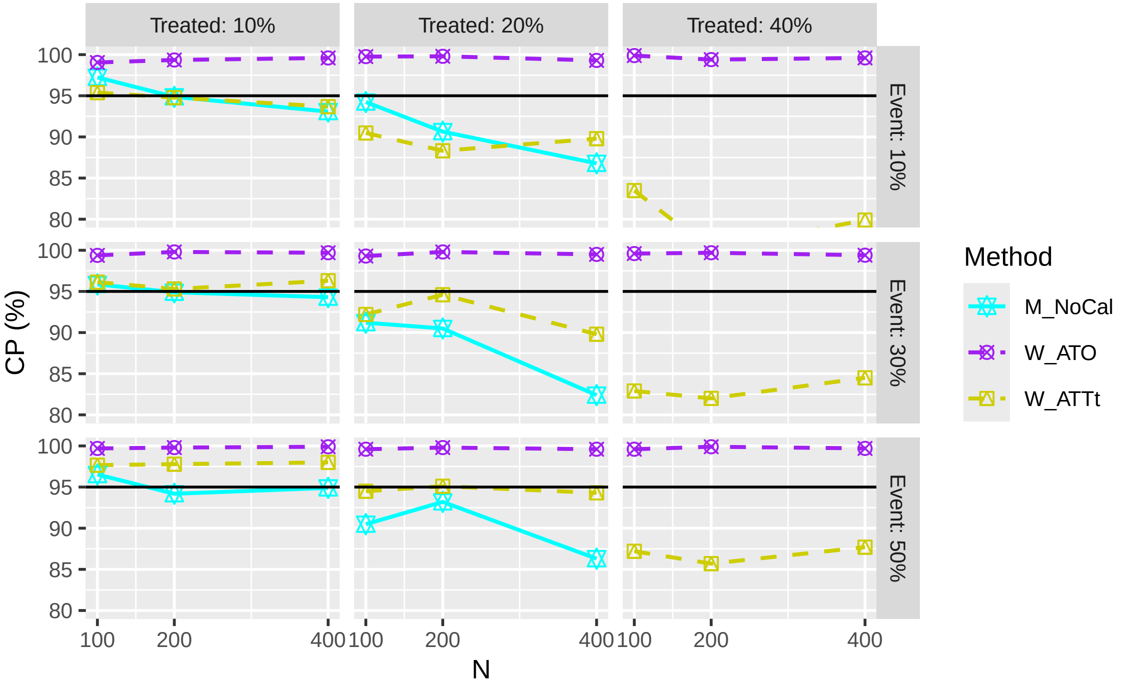

Figure S246. Coverage probability of confidence interval for OR (unimodal continuous covariate, matching ratio 1:1, true OR: 0.5, c statistic: 0.85, naive inference); other methods.

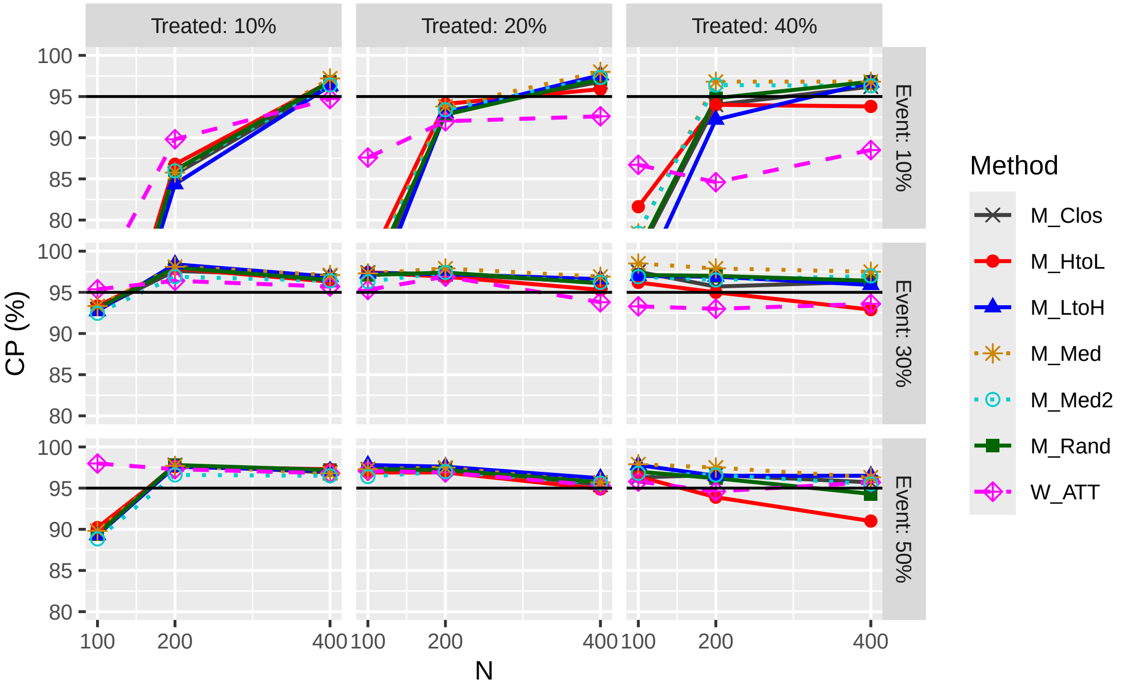

Figure S247. Coverage probability of confidence interval for OR (unimodal continuous covariate, matching ratio 1:1, true OR: 0.5, c statistic: 0.85, robust inference).

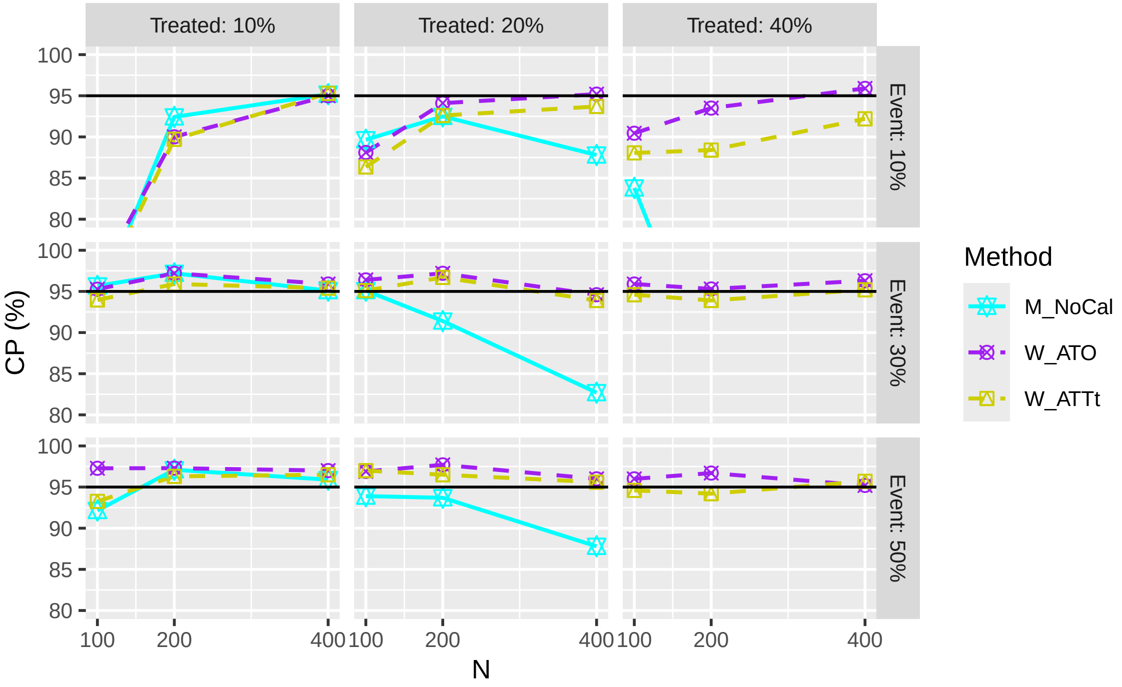

Figure S248. Coverage probability of confidence interval for OR (unimodal continuous covariate, matching ratio 1:1, true OR: 0.5, c statistic: 0.85, robust inference); other methods.

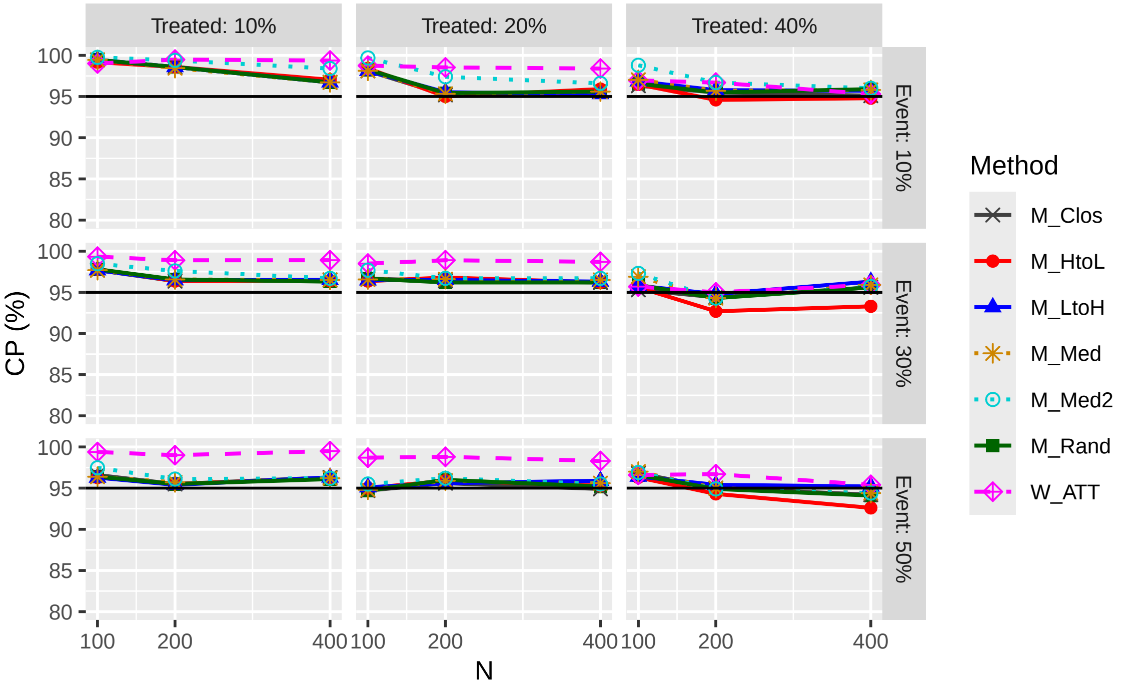

Figure S249. Coverage probability of confidence interval for OR (unimodal continuous covariate, matching ratio 1:1, true OR: 0.5, c statistic: 0.6, naive inference).

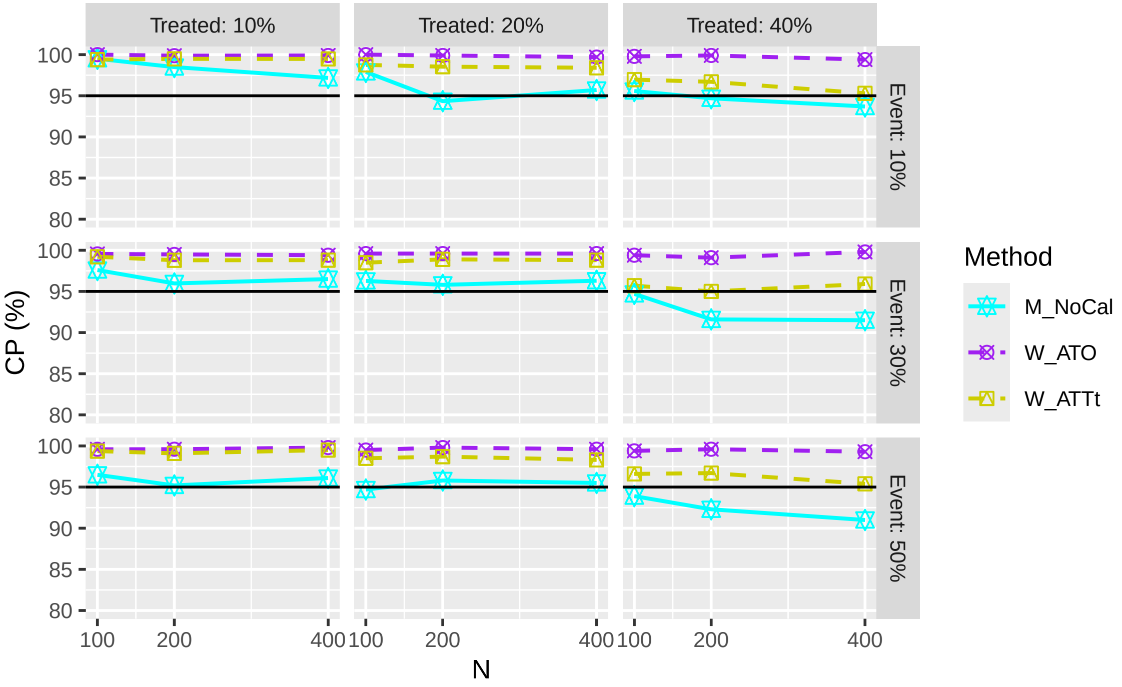

Figure S250. Coverage probability of confidence interval for OR (unimodal continuous covariate, matching ratio 1:1, true OR: 0.5, c statistic: 0.6, naive inference); other methods.

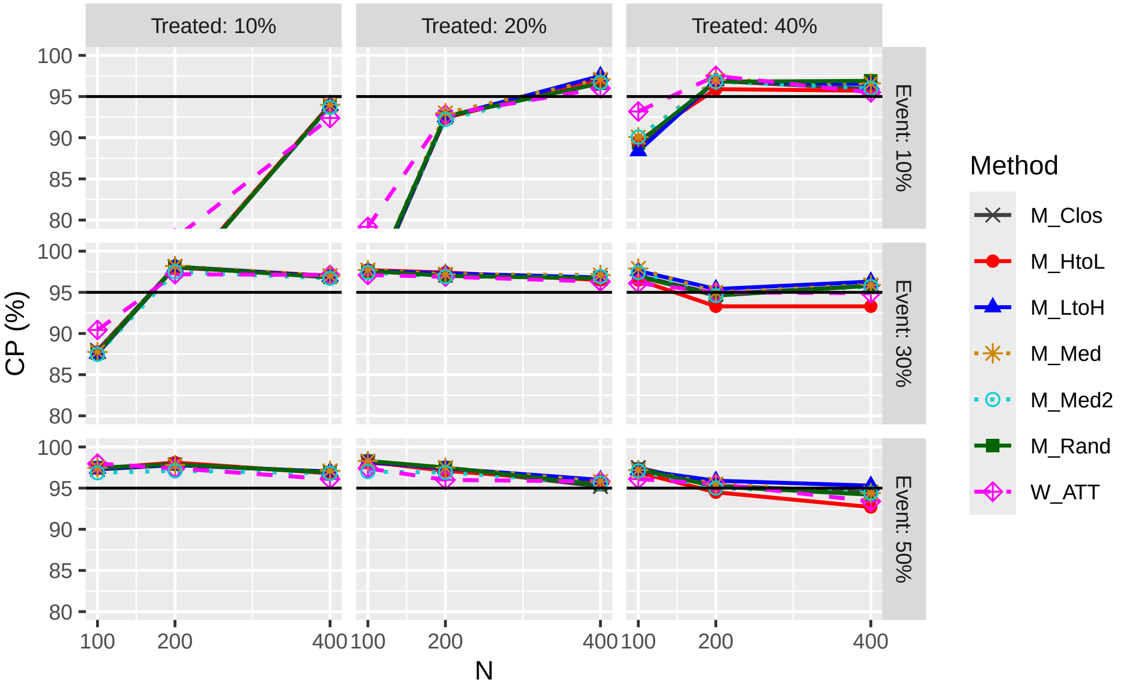

Figure S251. Coverage probability of confidence interval for OR (unimodal continuous covariate, matching ratio 1:1, true OR: 0.5, c statistic: 0.6, robust inference).

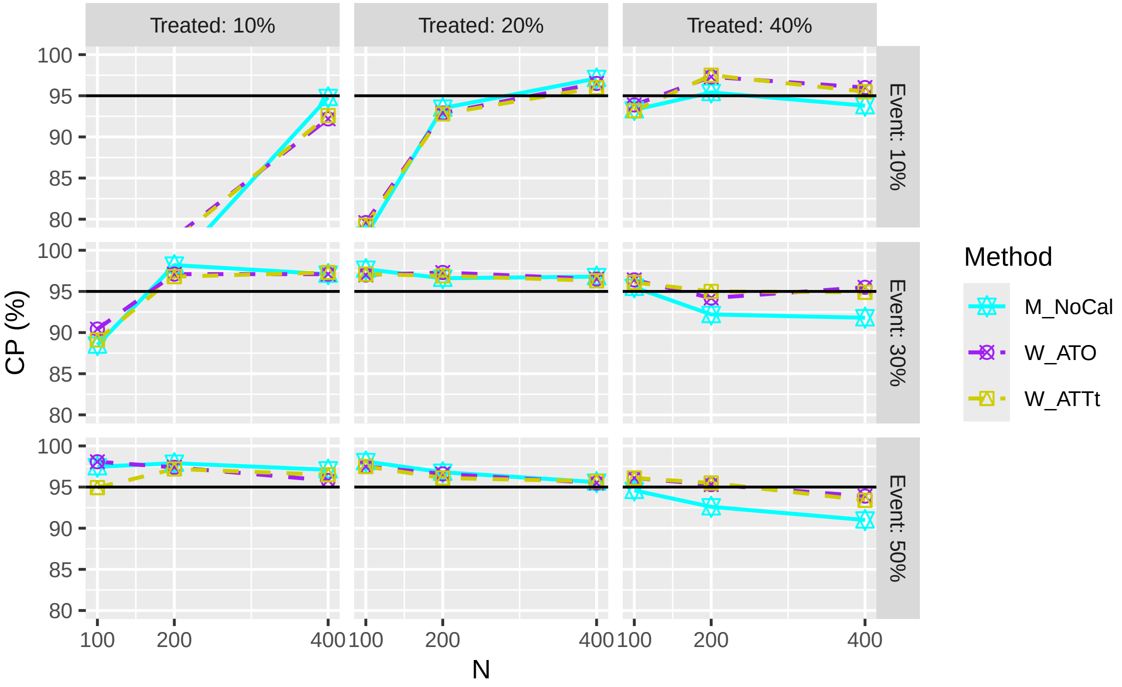

Figure S252. Coverage probability of confidence interval for OR (unimodal continuous covariate, matching ratio 1:1, true OR: 0.5, c statistic: 0.6, robust inference); other methods.

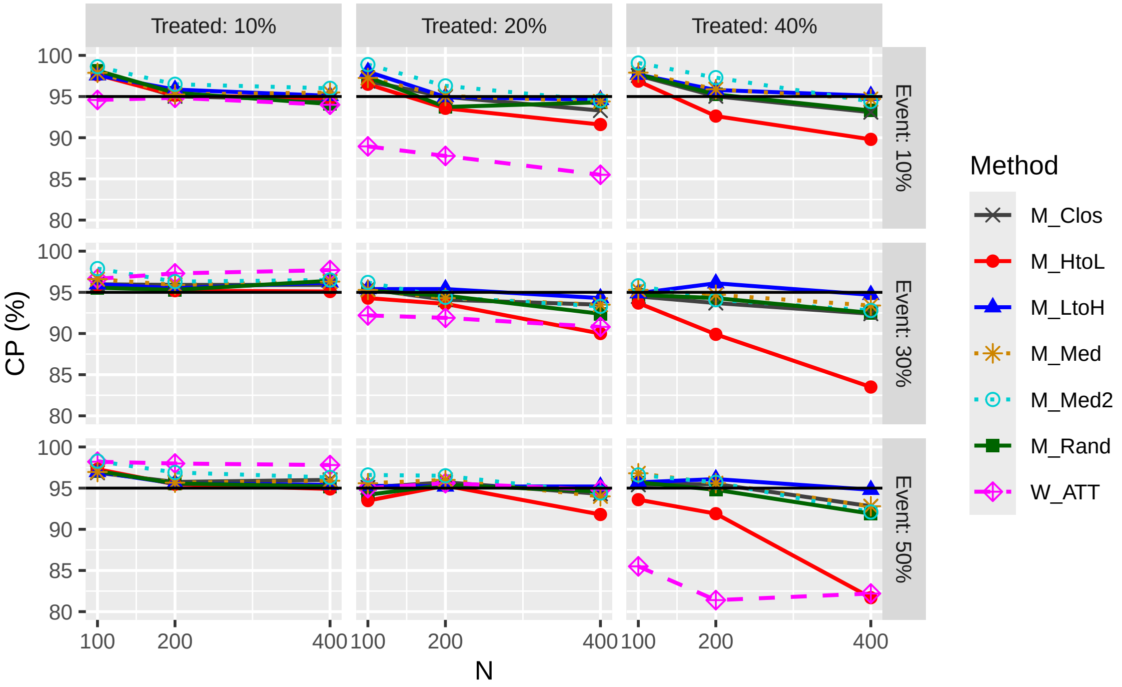

Figure S253. Coverage probability of confidence interval for OR (unimodal continuous covariate, matching ratio 1:2, true OR: 1, c statistic: 0.85, naive inference).

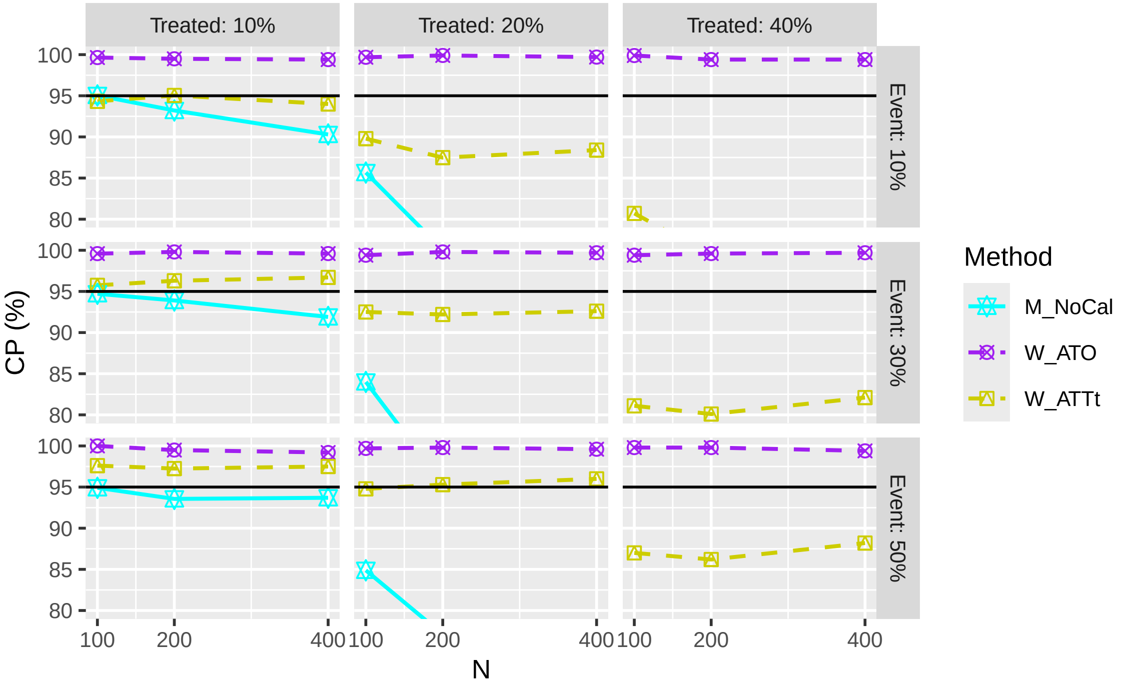

Figure S254. Coverage probability of confidence interval for OR (unimodal continuous covariate, matching ratio 1:2, true OR: 1, c statistic: 0.85, naive inference); other methods.

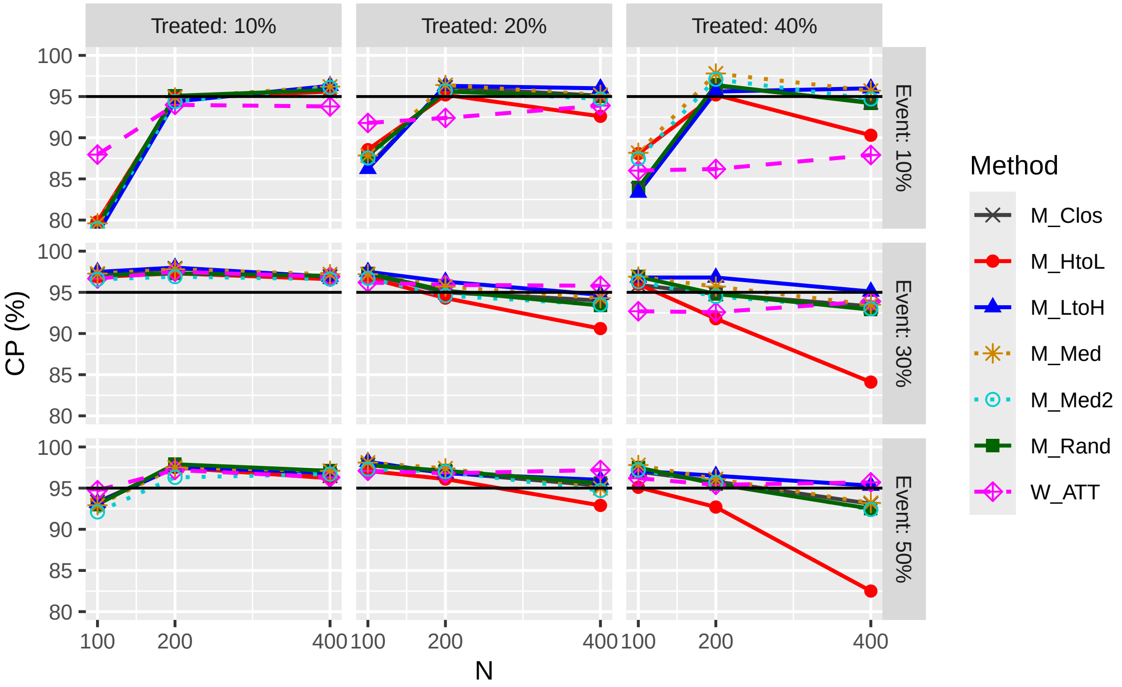

Figure S255. Coverage probability of confidence interval for OR (unimodal continuous covariate, matching ratio 1:2, true OR: 1, c statistic: 0.85, robust inference).

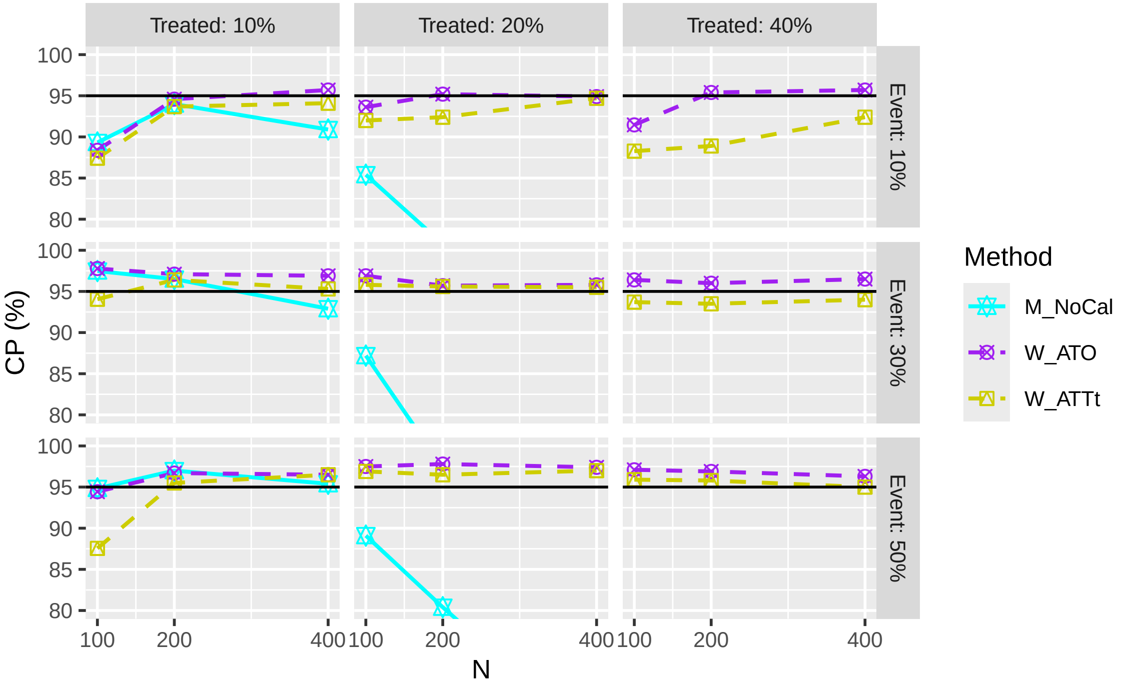

Figure S256. Coverage probability of confidence interval for OR (unimodal continuous covariate, matching ratio 1:2, true OR: 1, c statistic: 0.85, robust inference); other methods.

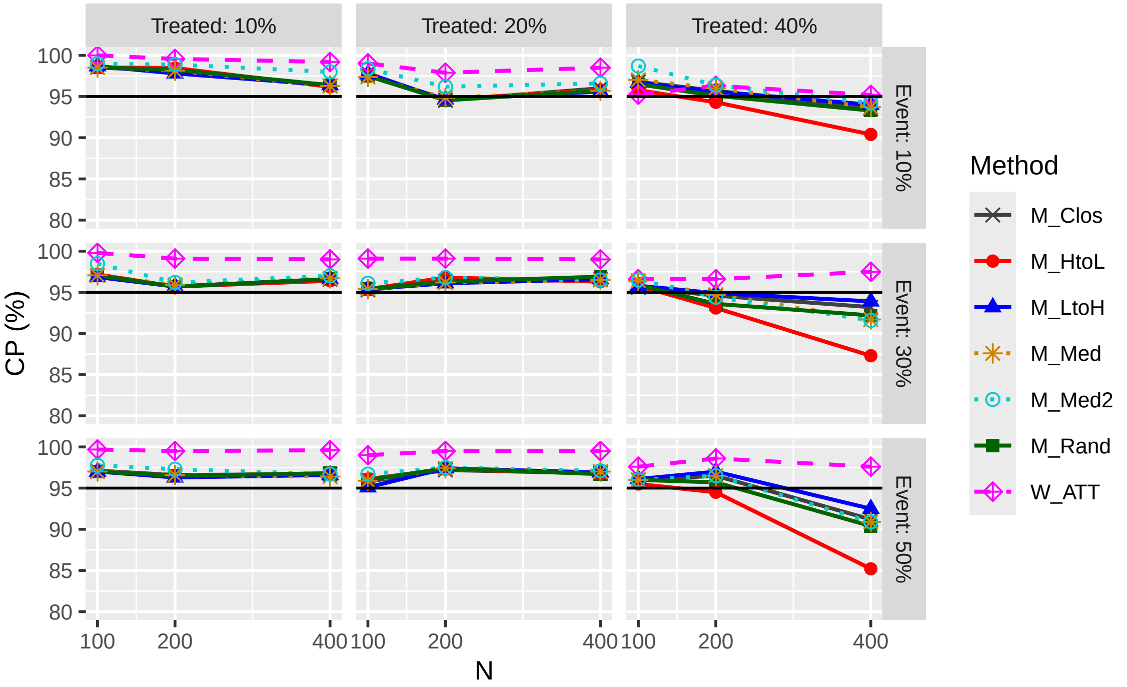

Figure S257. Coverage probability of confidence interval for OR (unimodal continuous covariate, matching ratio 1:2, true OR: 1, c statistic: 0.6, naive inference).

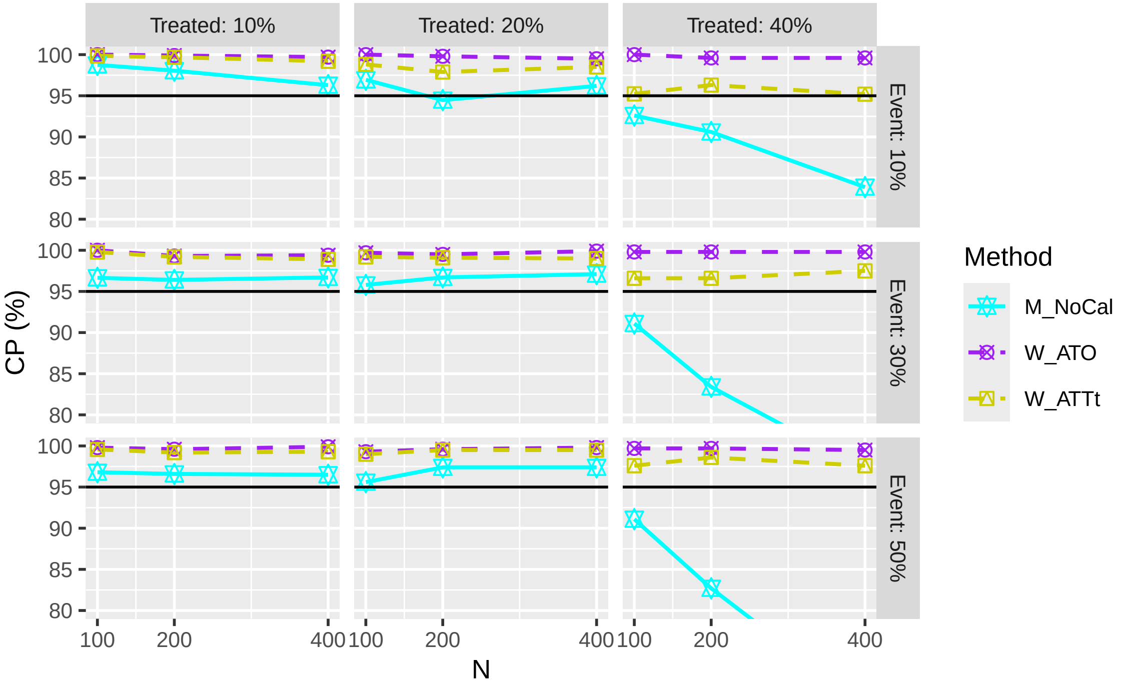

Figure S258. Coverage probability of confidence interval for OR (unimodal continuous covariate, matching ratio 1:2, true OR: 1, c statistic: 0.6, naive inference); other methods.

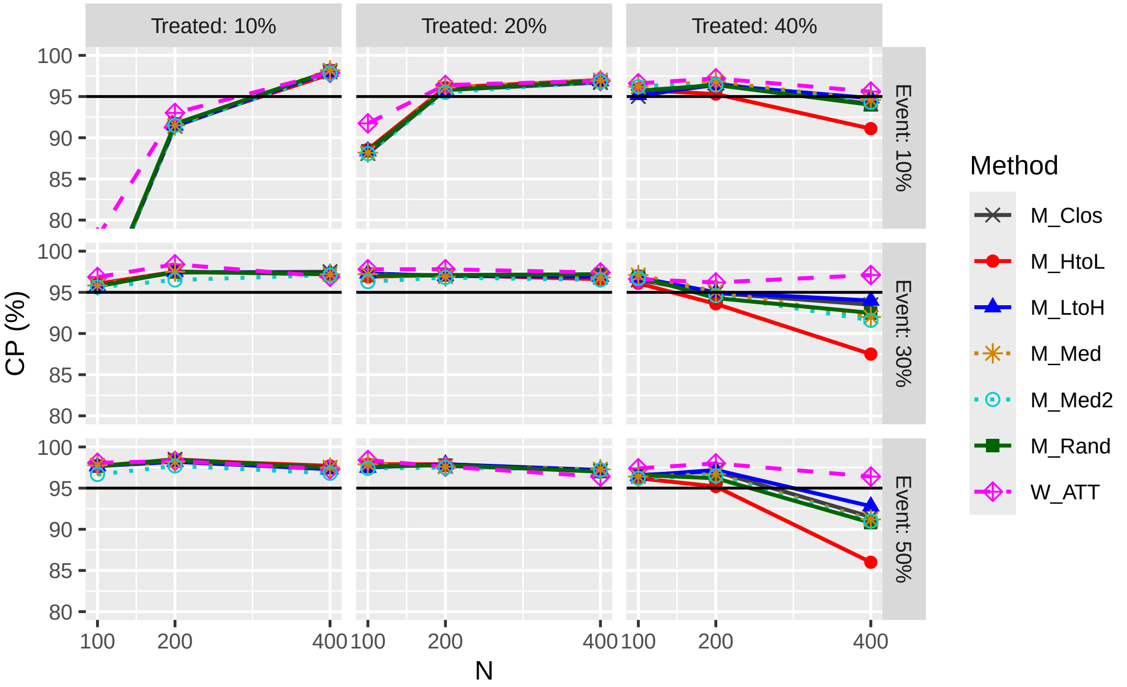

Figure S259. Coverage probability of confidence interval for OR (unimodal continuous covariate, matching ratio 1:2, true OR: 1, c statistic: 0.6, robust inference).

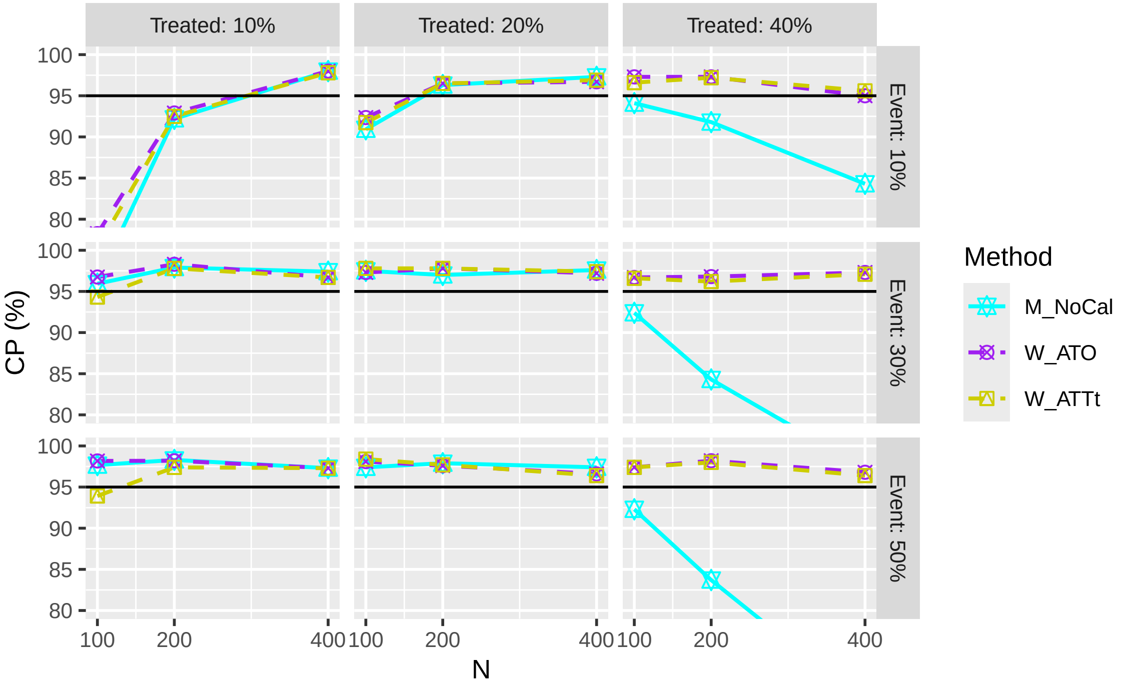

Figure S260. Coverage probability of confidence interval for OR (unimodal continuous covariate, matching ratio 1:2, true OR: 1, c statistic: 0.6, robust inference); other methods.

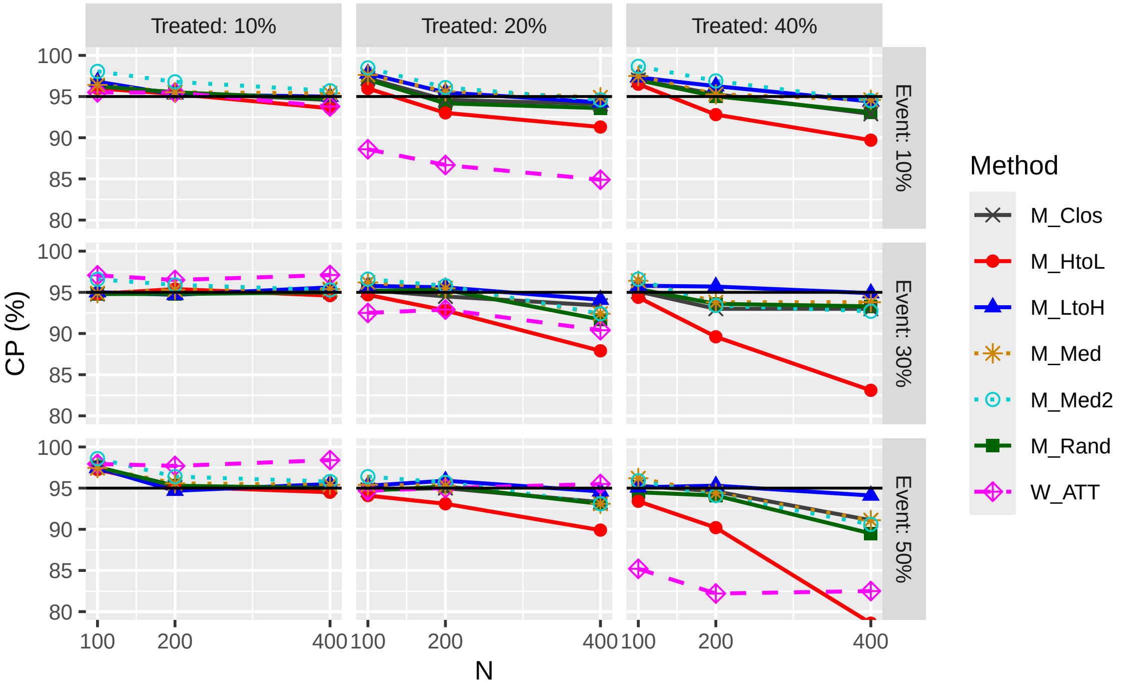

Figure S261. Coverage probability of confidence interval for OR (unimodal continuous covariate, matching ratio 1:2, true OR: 0.75, c statistic: 0.85, naive inference).

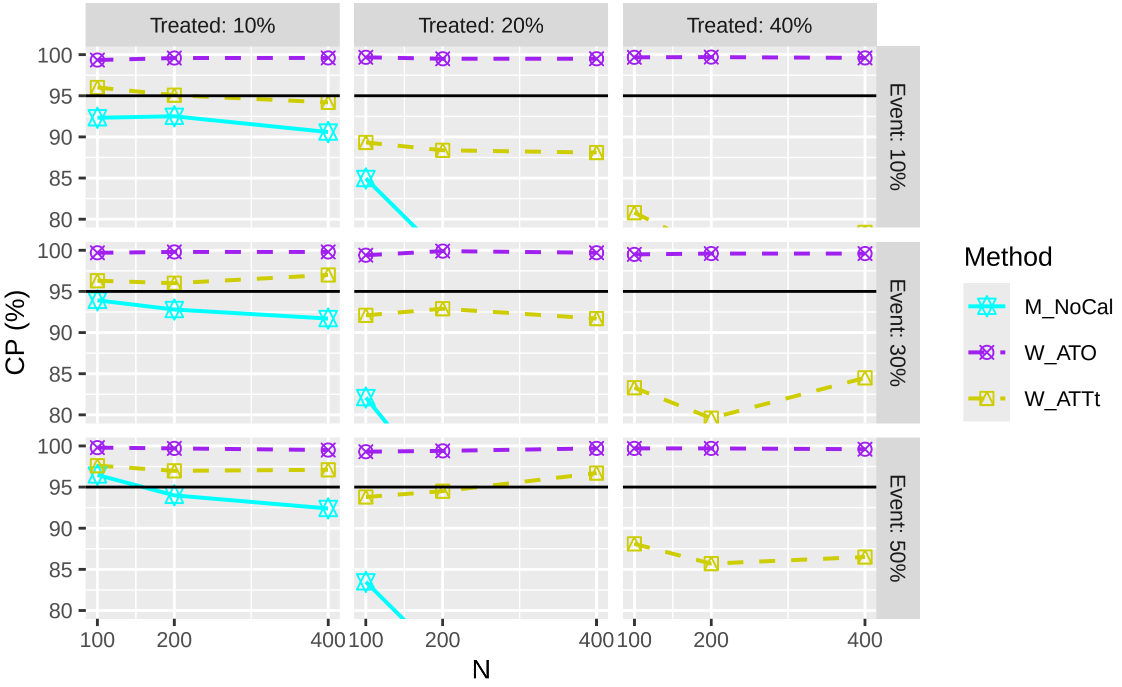

Figure S262. Coverage probability of confidence interval for OR (unimodal continuous covariate, matching ratio 1:2, true OR: 0.75, c statistic: 0.85, naive inference); other methods.

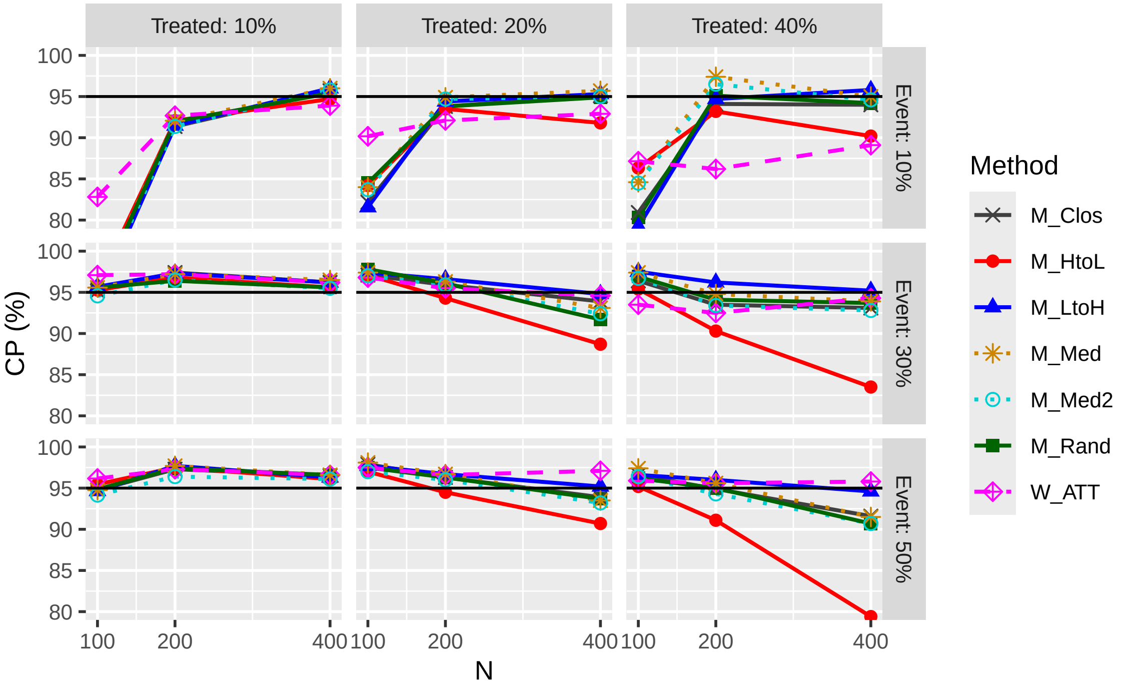

Figure S263. Coverage probability of confidence interval for OR (unimodal continuous covariate, matching ratio 1:2, true OR: 0.75, c statistic: 0.85, robust inference).

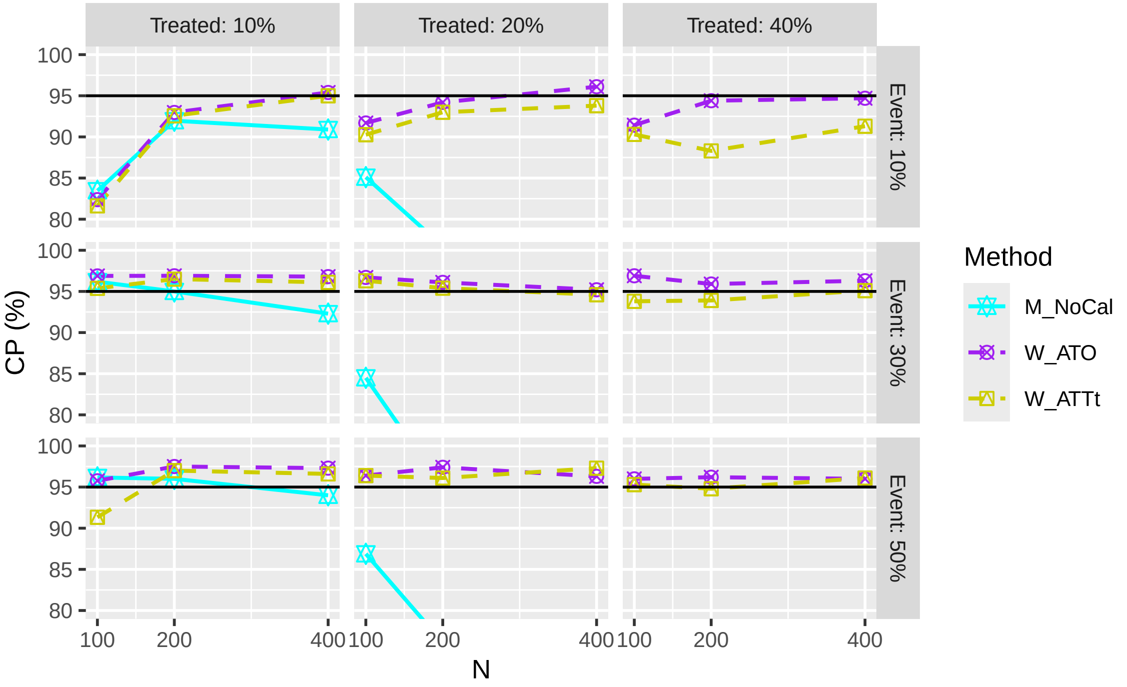

Figure S264. Coverage probability of confidence interval for OR (unimodal continuous covariate, matching ratio 1:2, true OR: 0.75, c statistic: 0.85, robust inference); other methods.

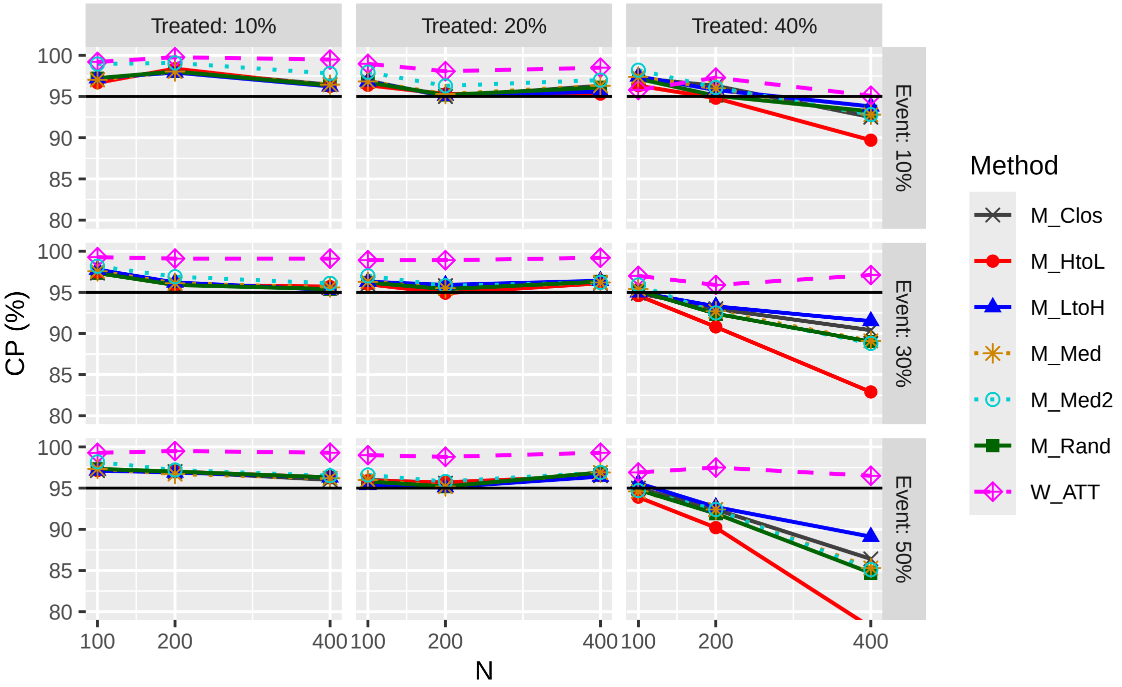

Figure S265. Coverage probability of confidence interval for OR (unimodal continuous covariate, matching ratio 1:2, true OR: 0.75, c statistic: 0.6, naive inference).

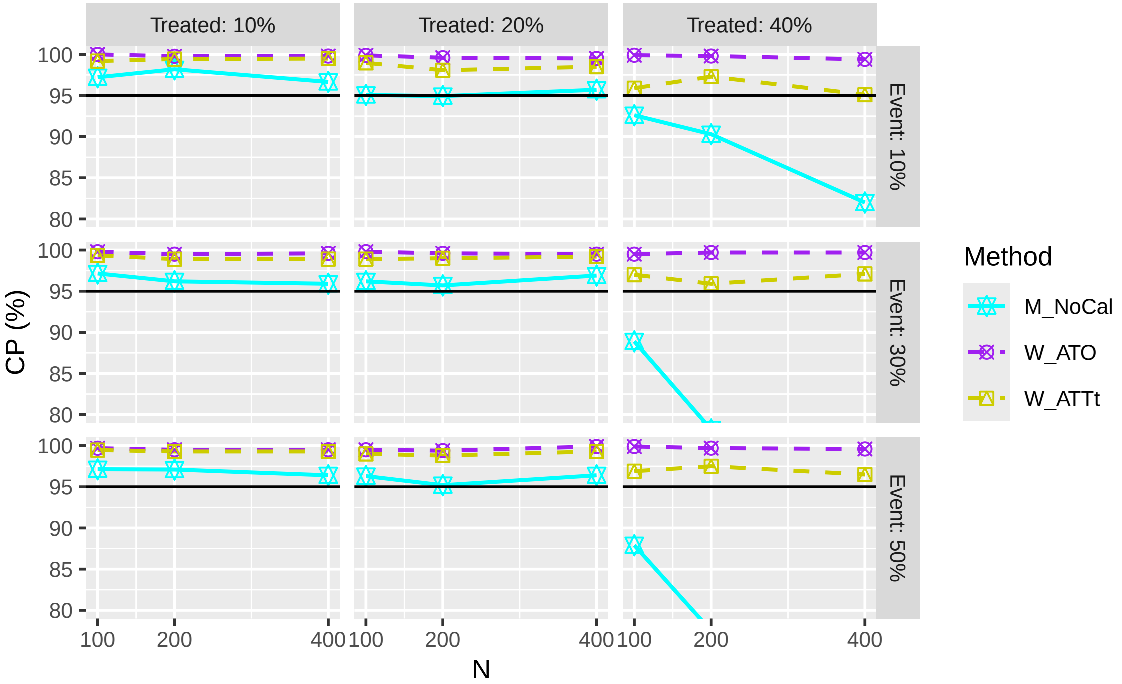

Figure S266. Coverage probability of confidence interval for OR (unimodal continuous covariate, matching ratio 1:2, true OR: 0.75, c statistic: 0.6, naive inference); other methods.

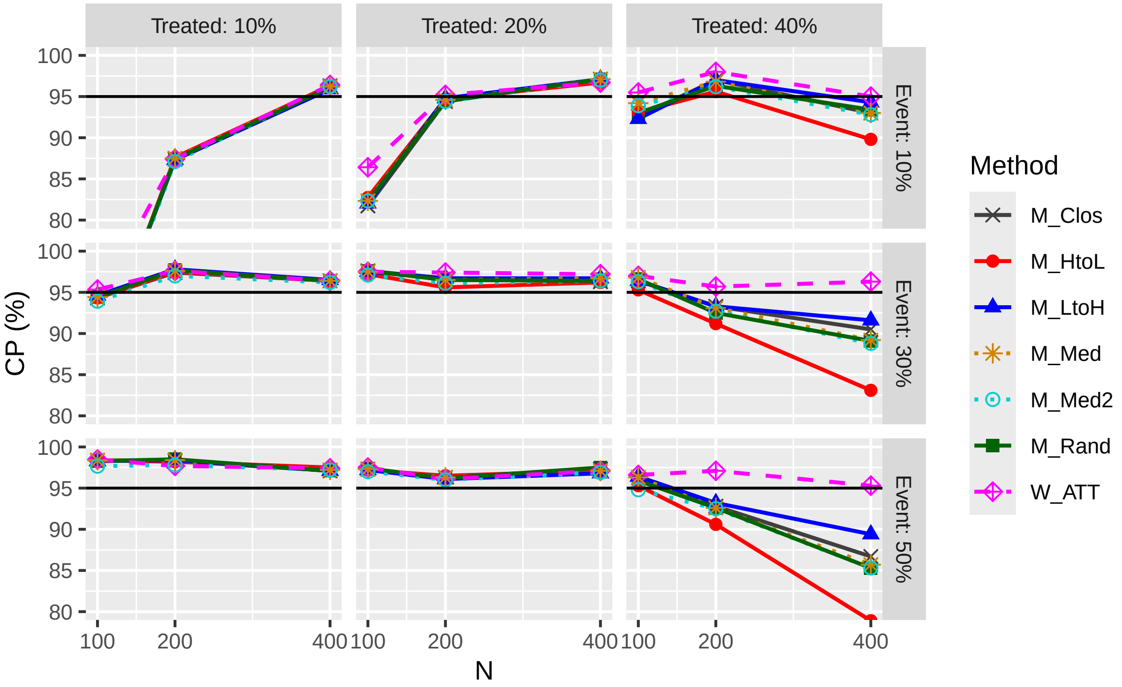

Figure S267. Coverage probability of confidence interval for OR (unimodal continuous covariate, matching ratio 1:2, true OR: 0.75, c statistic: 0.6, robust inference).

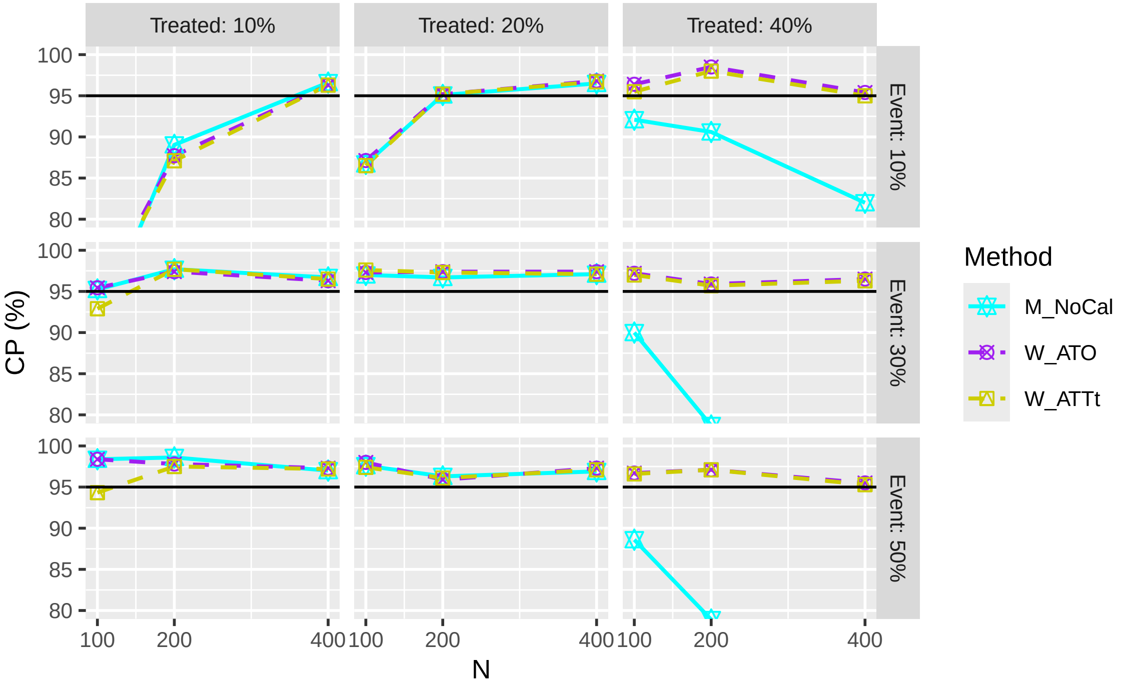

Figure S268. Coverage probability of confidence interval for OR (unimodal continuous covariate, matching ratio 1:2, true OR: 0.75, c statistic: 0.6, robust inference); other methods.

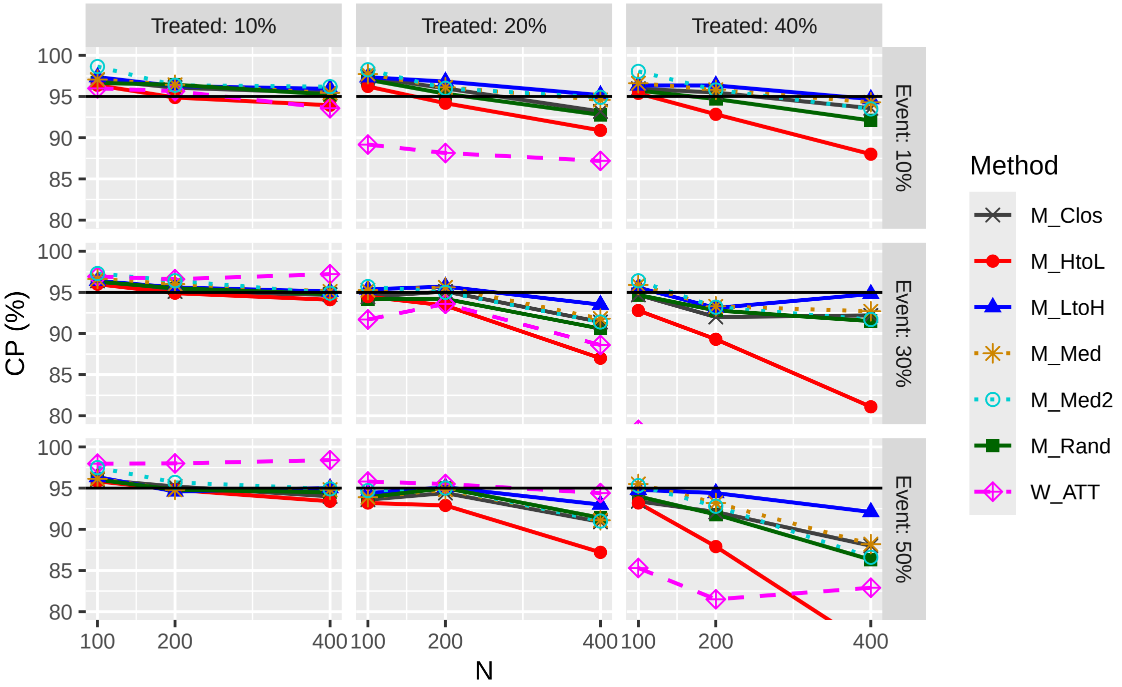

Figure S269. Coverage probability of confidence interval for OR (unimodal continuous covariate, matching ratio 1:2, true OR: 0.5, c statistic: 0.85, naive inference).

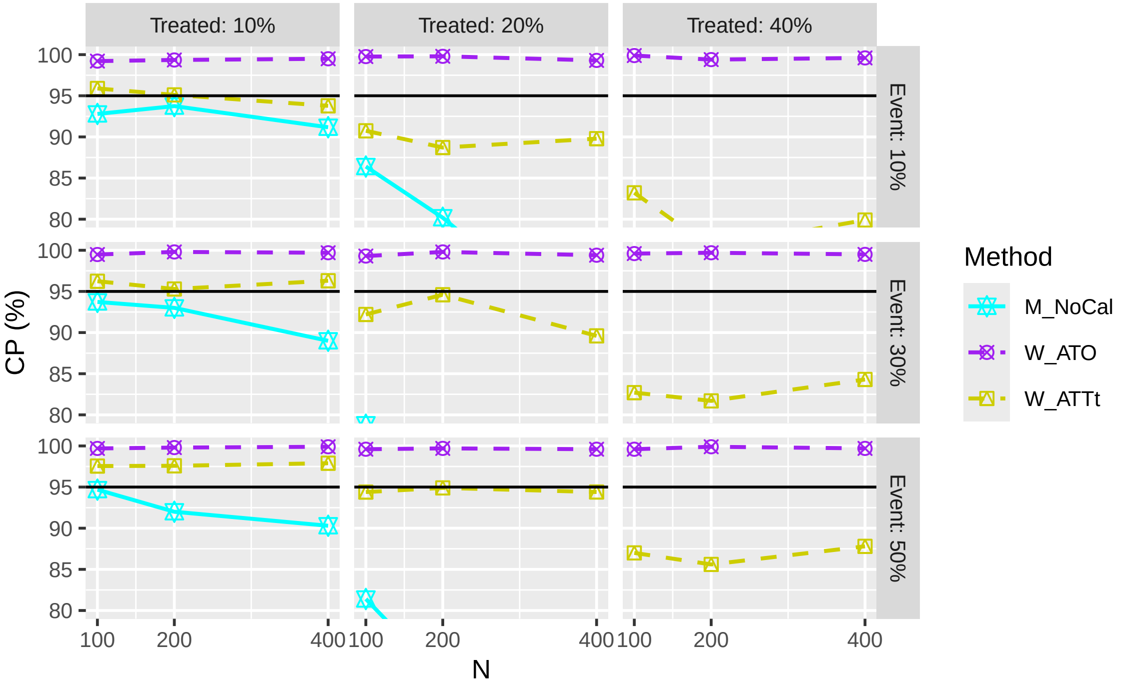

Figure S270. Coverage probability of confidence interval for OR (unimodal continuous covariate, matching ratio 1:2, true OR: 0.5, c statistic: 0.85, naive inference); other methods.

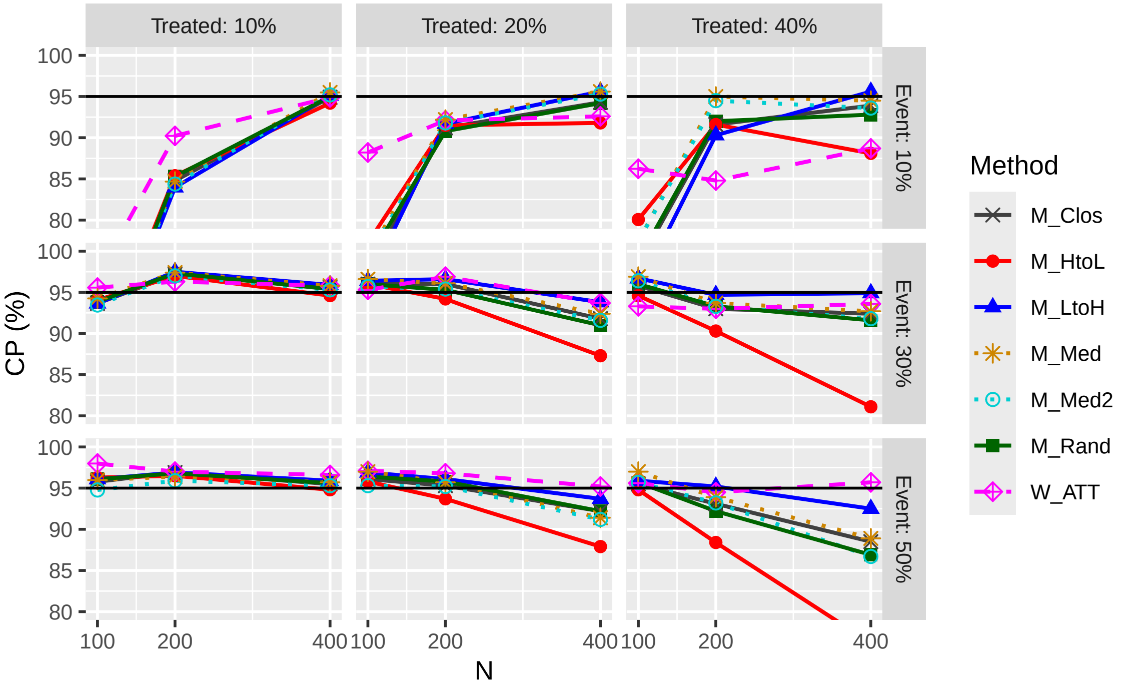

Figure S271. Coverage probability of confidence interval for OR (unimodal continuous covariate, matching ratio 1:2, true OR: 0.5, c statistic: 0.85, robust inference).

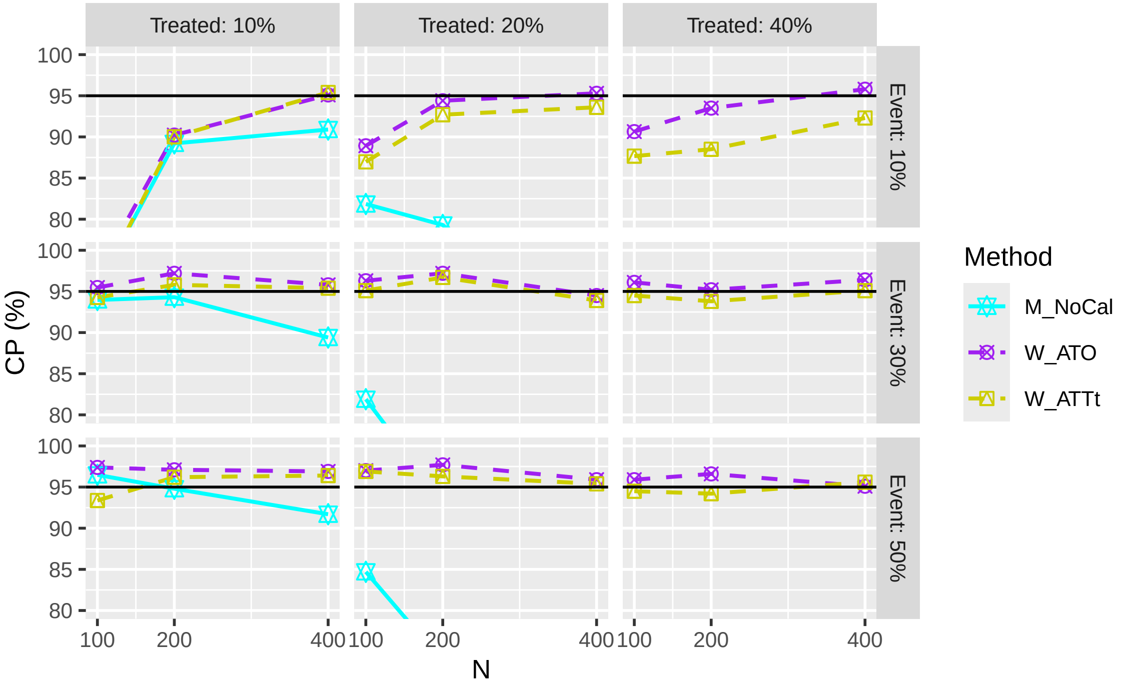

Figure S272. Coverage probability of confidence interval for OR (unimodal continuous covariate, matching ratio 1:2, true OR: 0.5, c statistic: 0.85, robust inference); other methods.

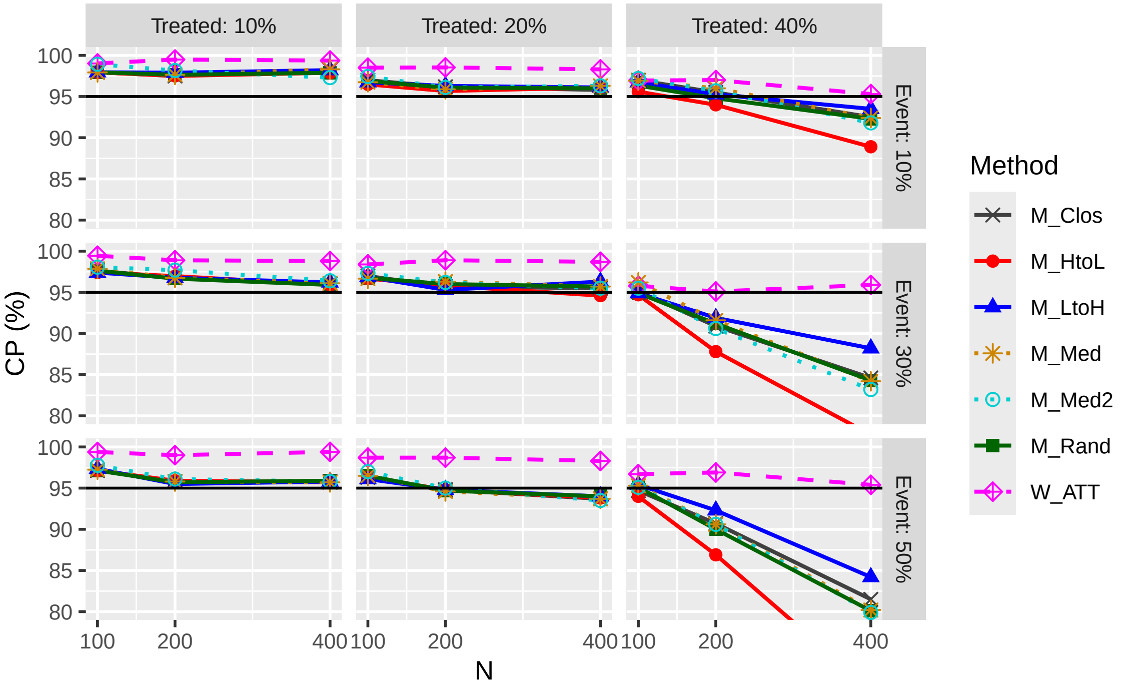

Figure S273. Coverage probability of confidence interval for OR (unimodal continuous covariate, matching ratio 1:2, true OR: 0.5, c statistic: 0.6, naive inference).

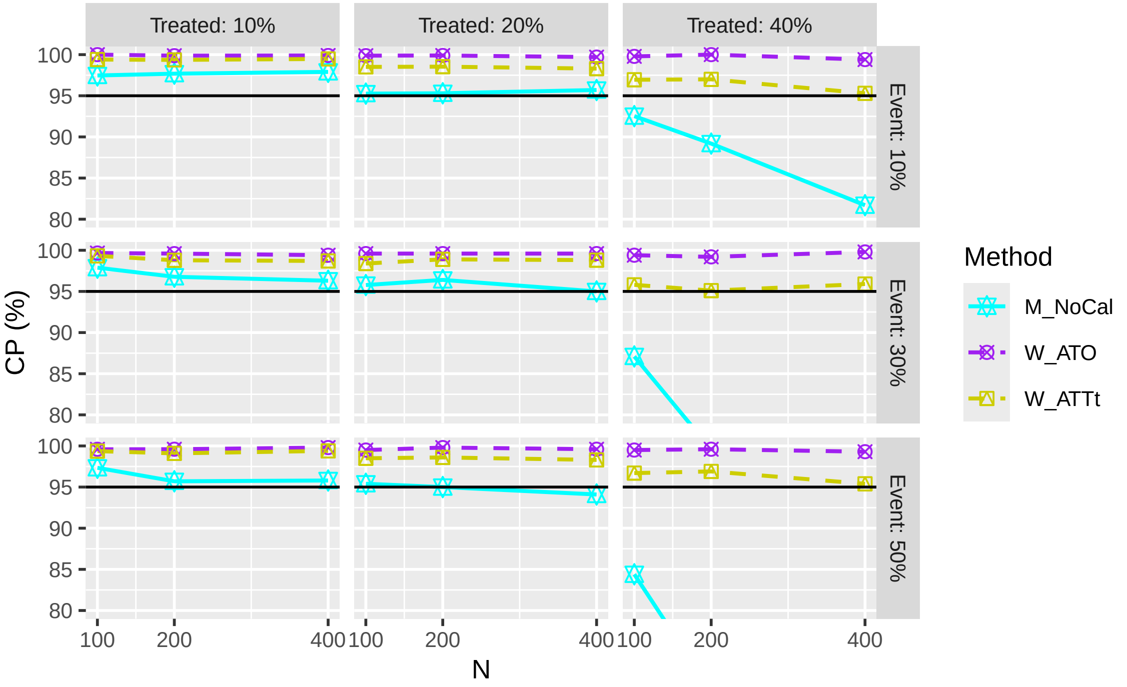

Figure S274. Coverage probability of confidence interval for OR (unimodal continuous covariate, matching ratio 1:2, true OR: 0.5, c statistic: 0.6, naive inference); other methods.

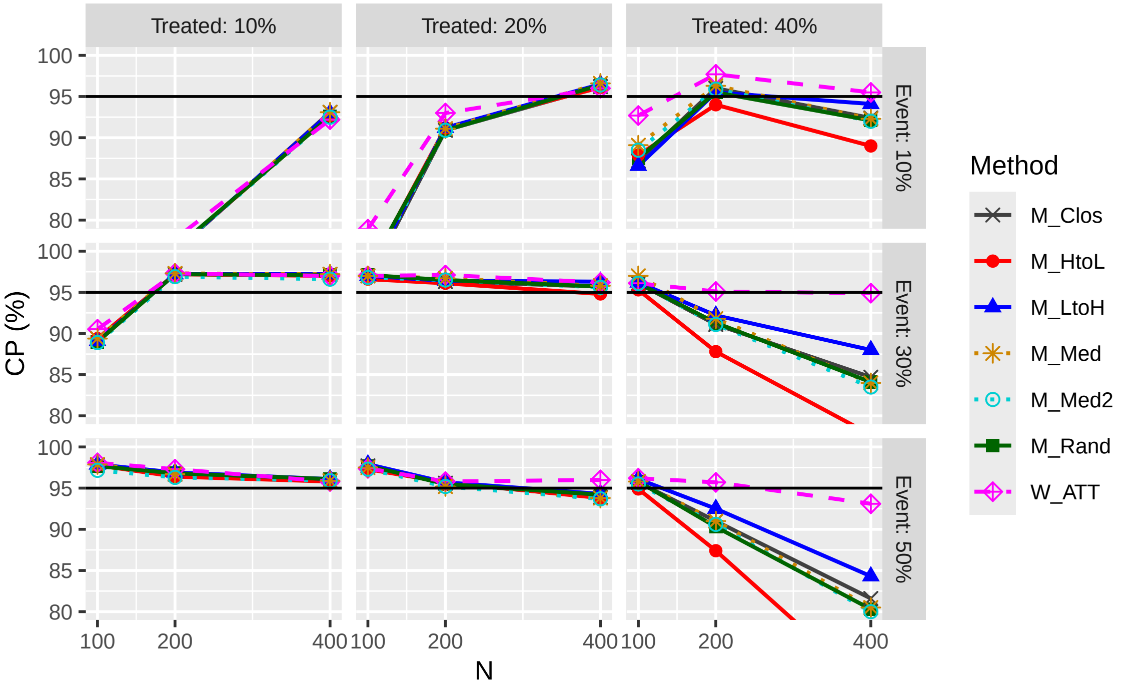

Figure S275. Coverage probability of confidence interval for OR (unimodal continuous covariate, matching ratio 1:2, true OR: 0.5, c statistic: 0.6, robust inference).

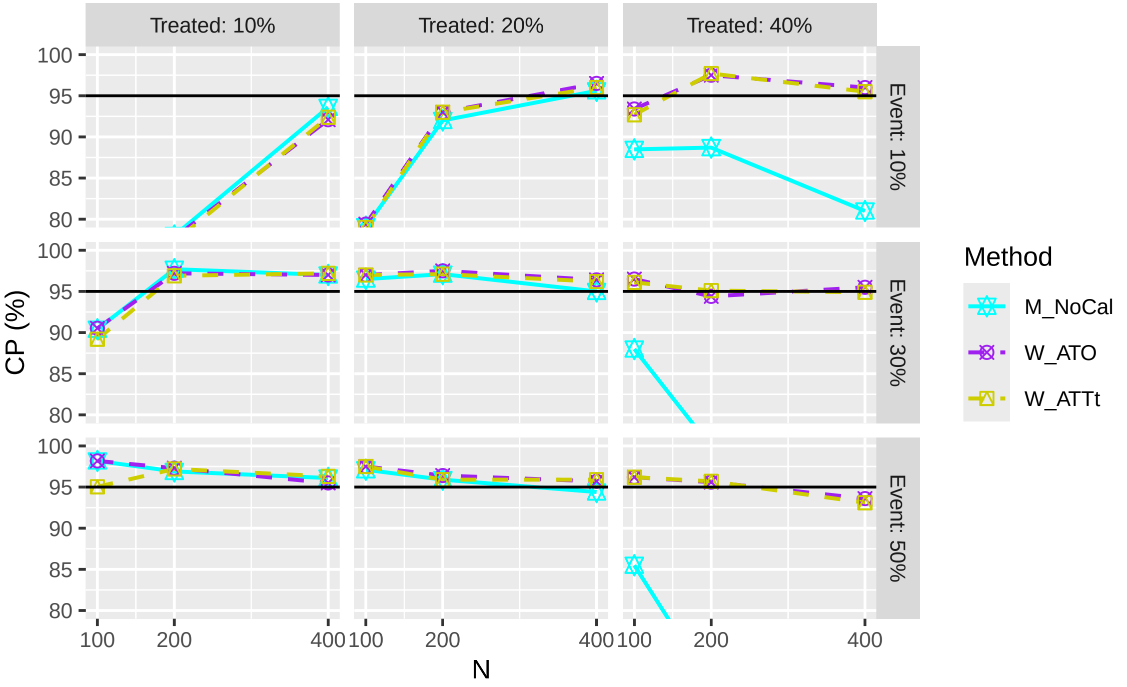

Figure S276. Coverage probability of confidence interval for OR (unimodal continuous covariate, matching ratio 1:2, true OR: 0.5, c statistic: 0.6, robust inference); other methods.

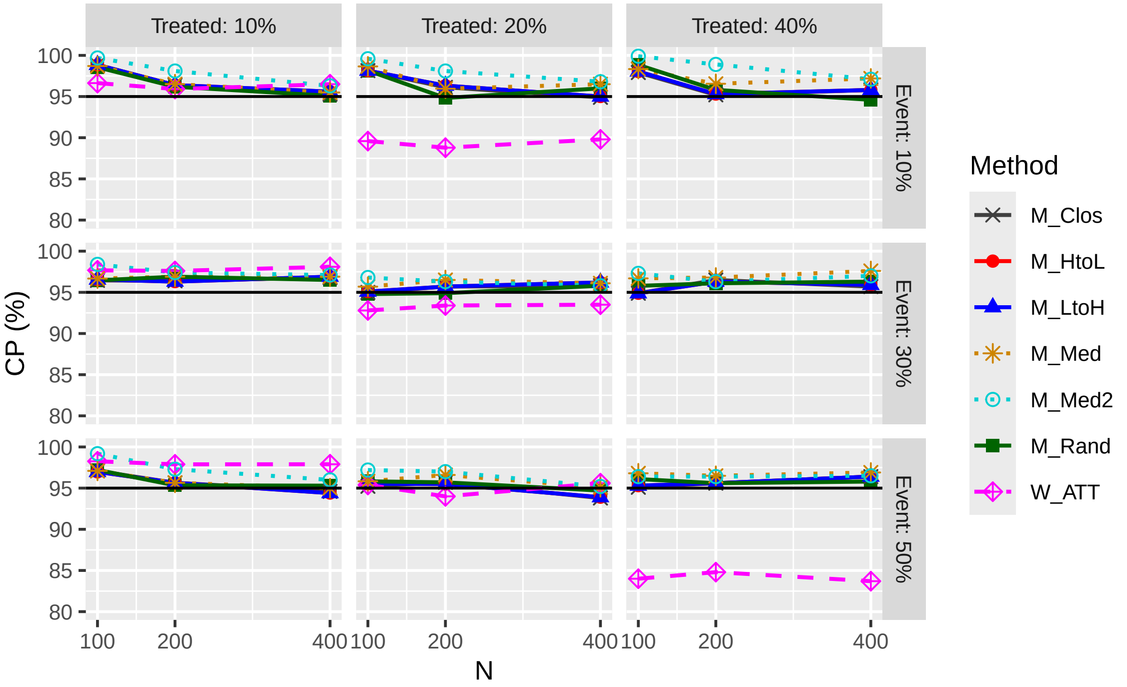

Figure S277. Coverage probability of confidence interval for OR (categorical covariate, matching ratio 1:1, true OR: 1, c statistic: 0.85, naive inference).

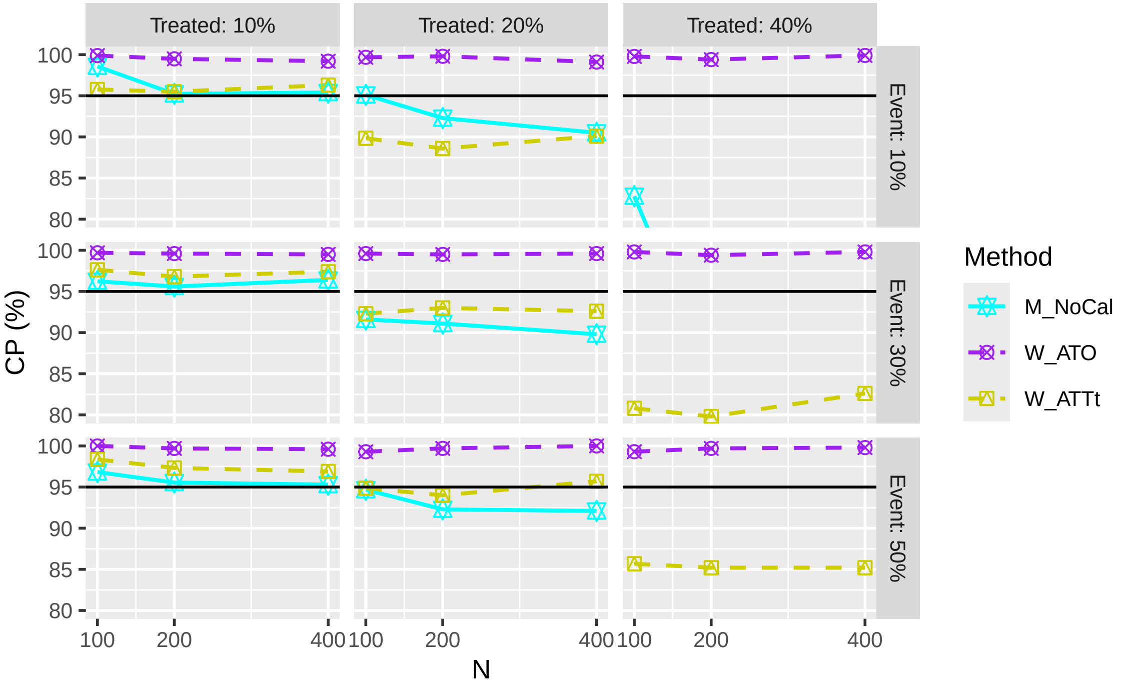

Figure S278. Coverage probability of confidence interval for OR (categorical covariate, matching ratio 1:1, true OR: 1, c statistic: 0.85, naive inference); other methods.

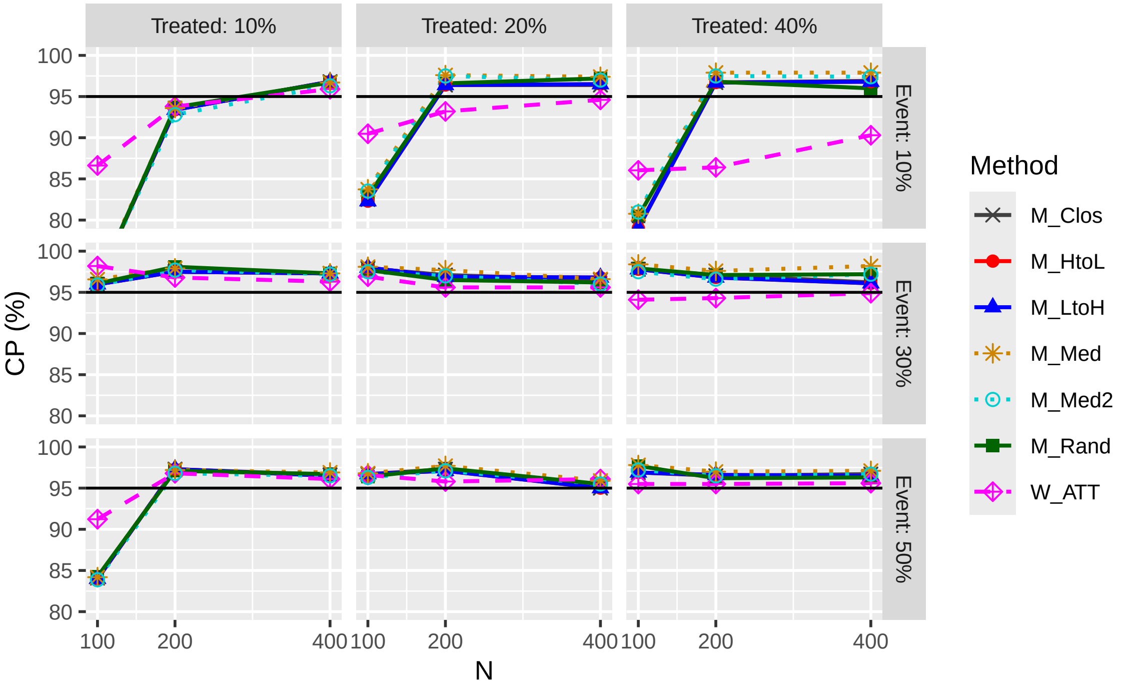

Figure S279. Coverage probability of confidence interval for OR (categorical covariate, matching ratio 1:1, true OR: 1, c statistic: 0.85, robust inference).

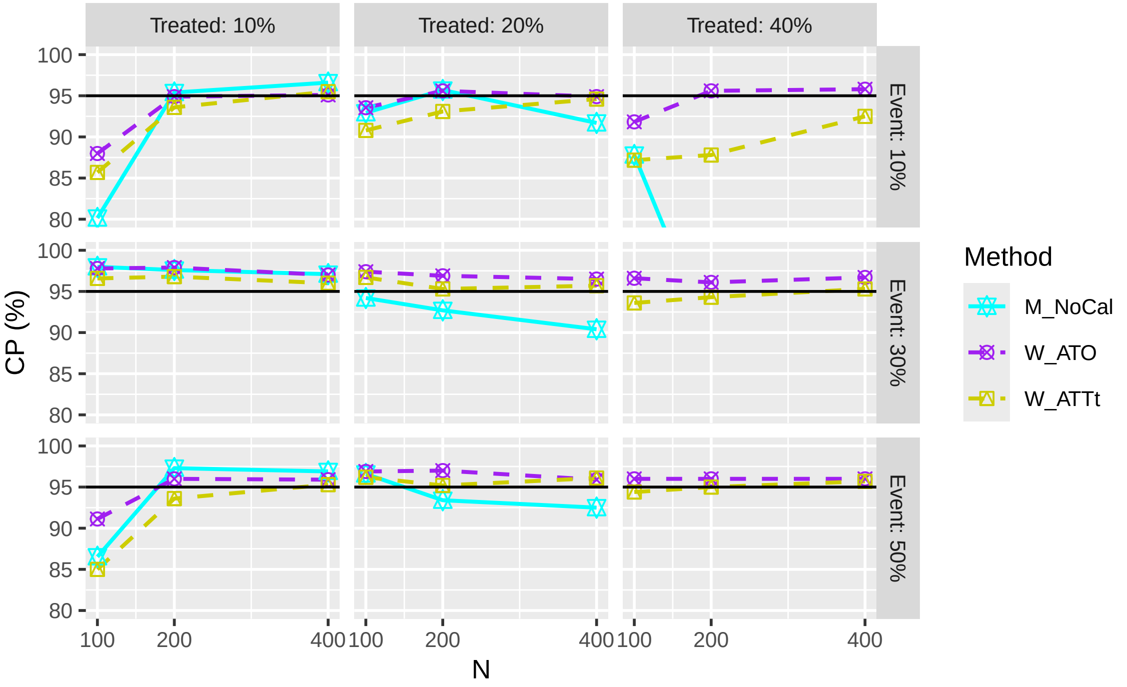

Figure S280. Coverage probability of confidence interval for OR (categorical covariate, matching ratio 1:1, true OR: 1, c statistic: 0.85, robust inference); other methods.

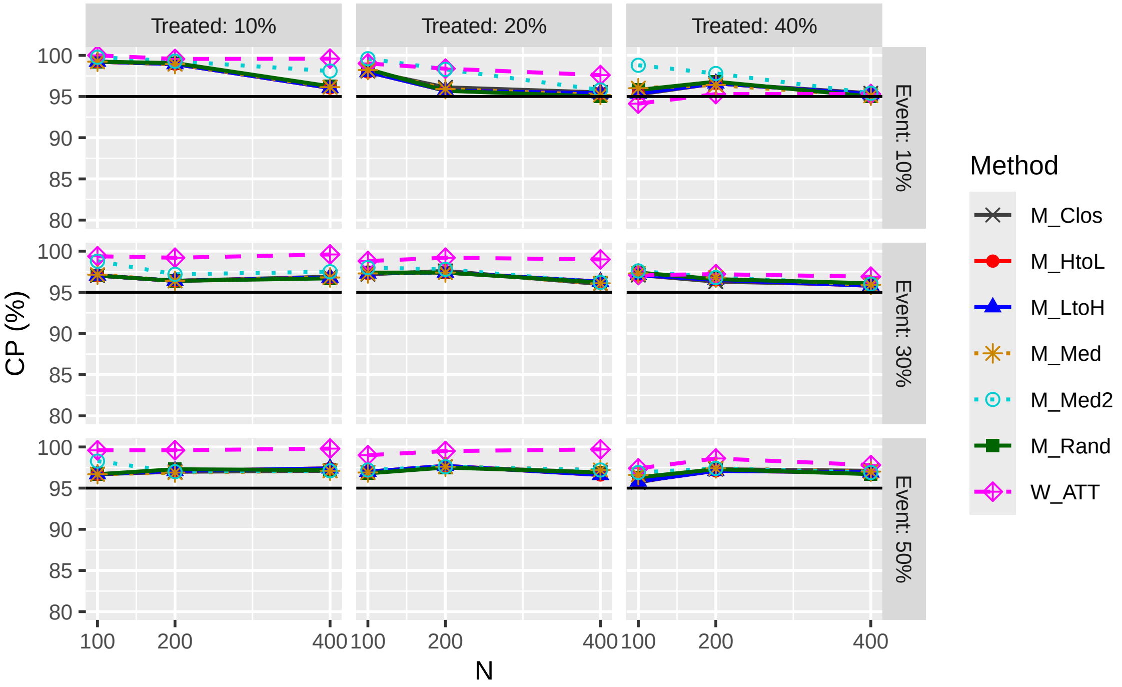

Figure S281. Coverage probability of confidence interval for OR (categorical covariate, matching ratio 1:1, true OR: 1, c statistic: 0.6, naive inference).

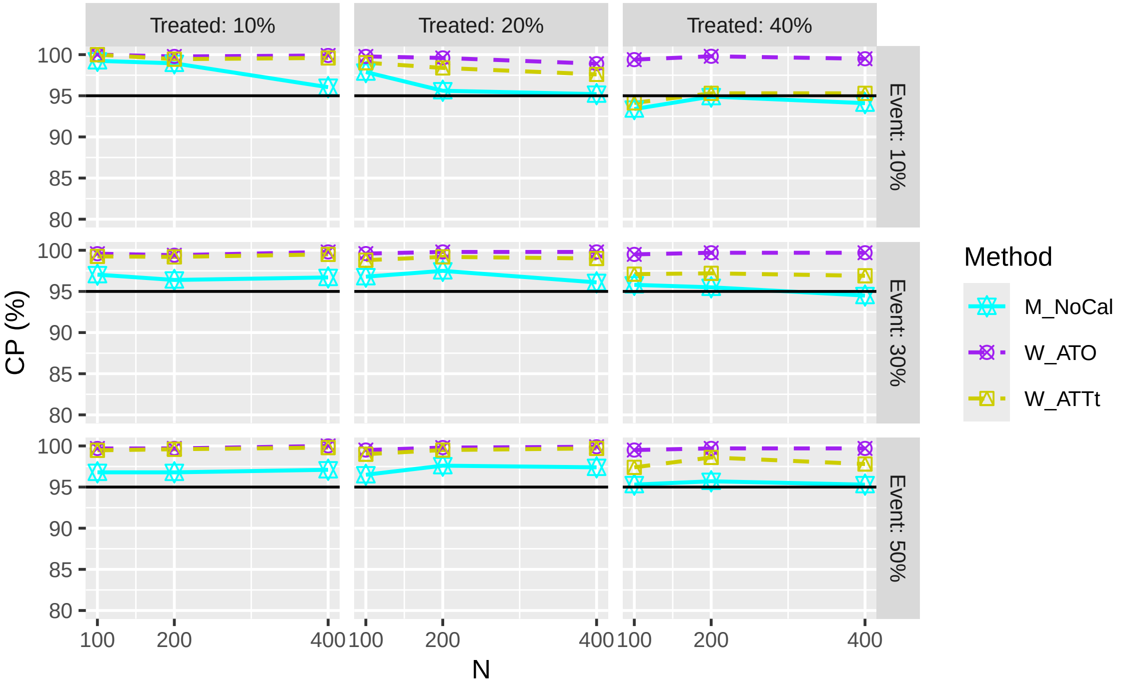

Figure S282. Coverage probability of confidence interval for OR (categorical covariate, matching ratio 1:1, true OR: 1, c statistic: 0.6, naive inference); other methods.

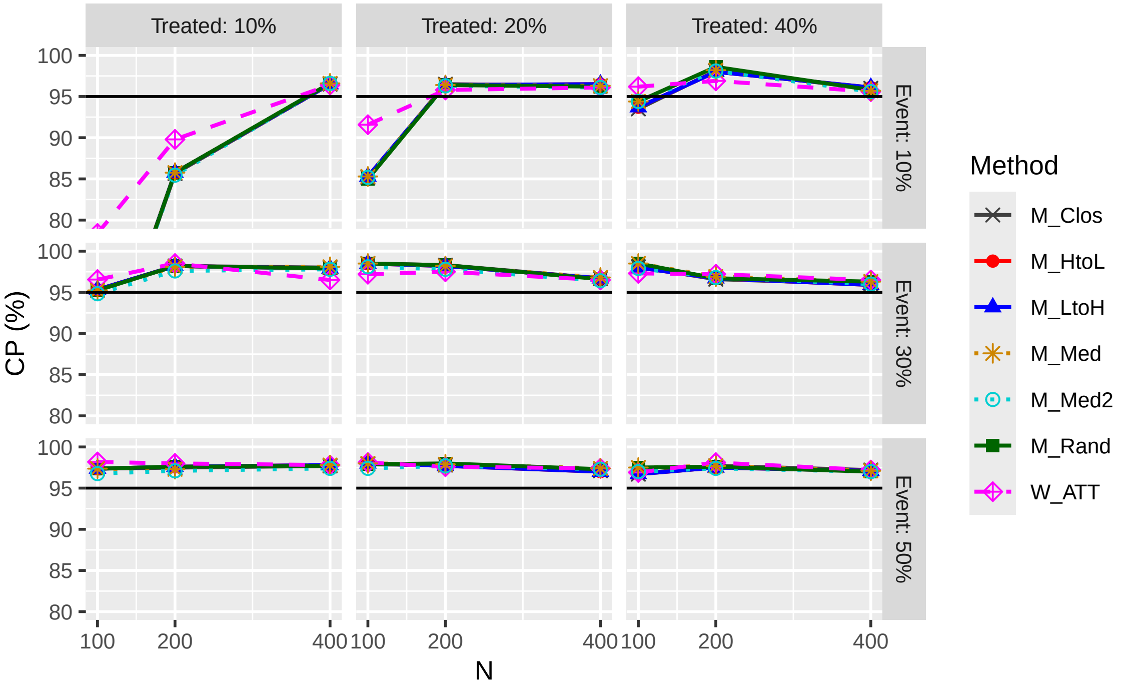

Figure S283. Coverage probability of confidence interval for OR (categorical covariate, matching ratio 1:1, true OR: 1, c statistic: 0.6, robust inference).

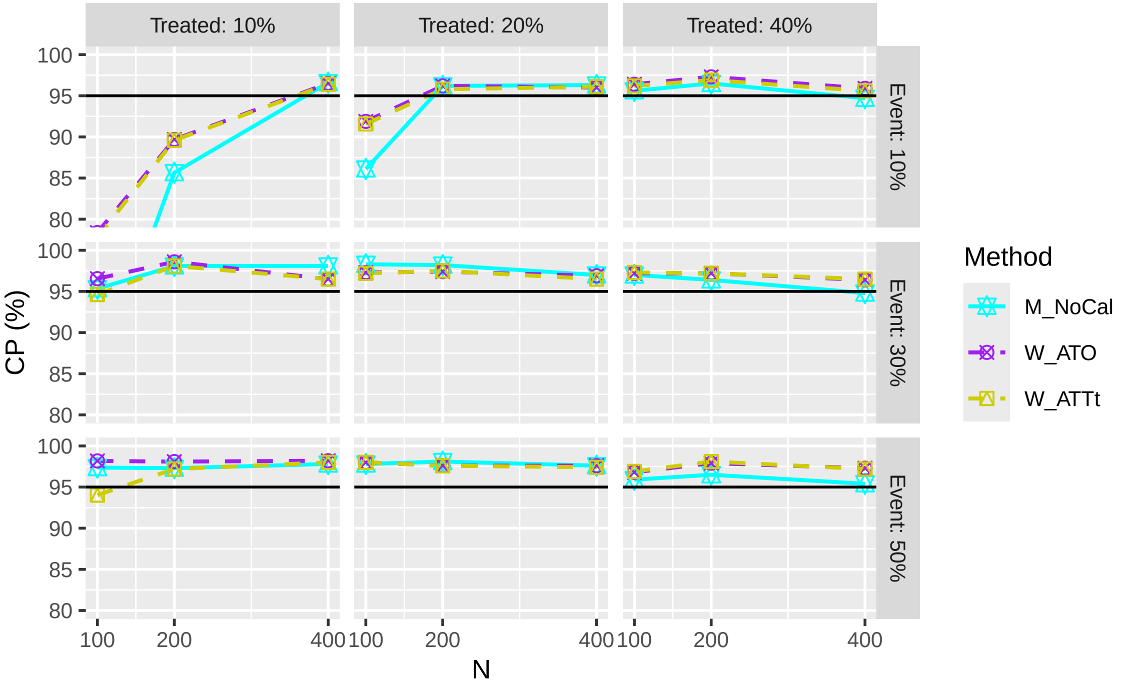

Figure S284. Coverage probability of confidence interval for OR (categorical covariate, matching ratio 1:1, true OR: 1, c statistic: 0.6, robust inference); other methods.

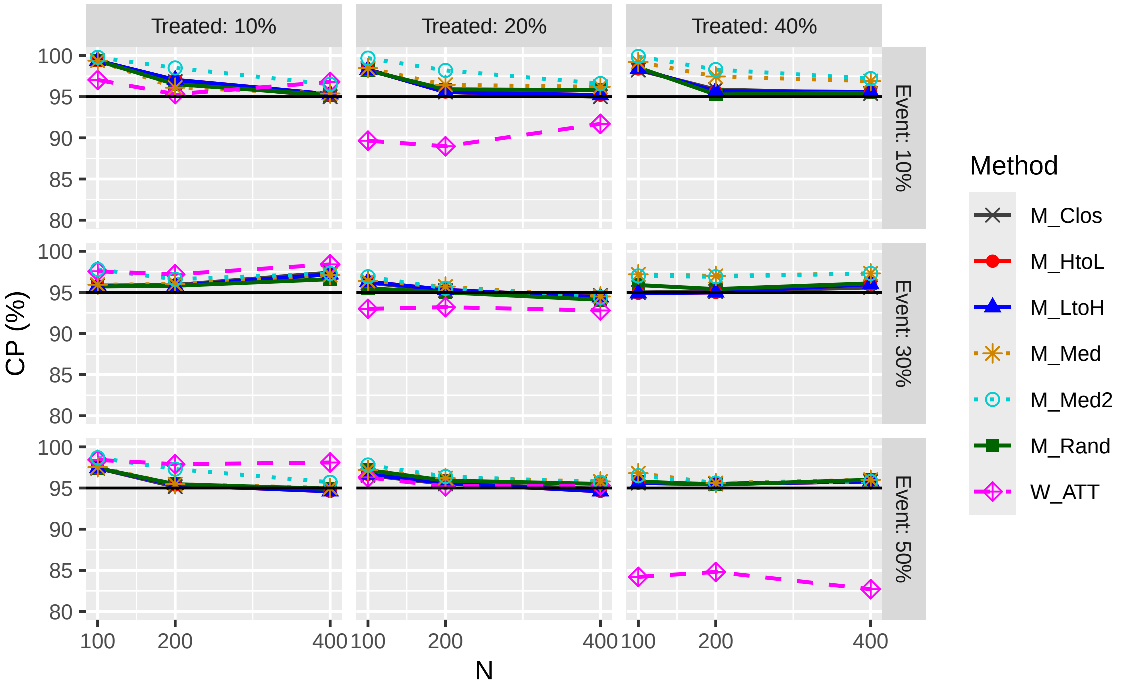

Figure S285. Coverage probability of confidence interval for OR (categorical covariate, matching ratio 1:1, true OR: 0.75, c statistic: 0.85, naive inference).

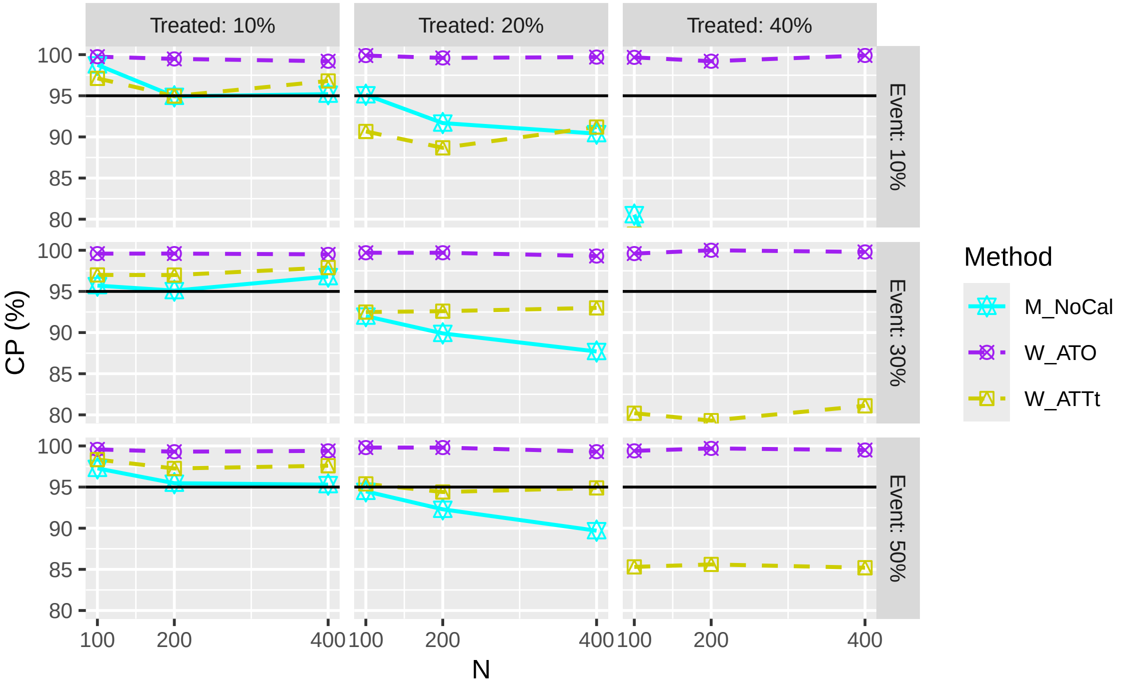

Figure S286. Coverage probability of confidence interval for OR (categorical covariate, matching ratio 1:1, true OR: 0.75, c statistic: 0.85, naive inference); other methods.

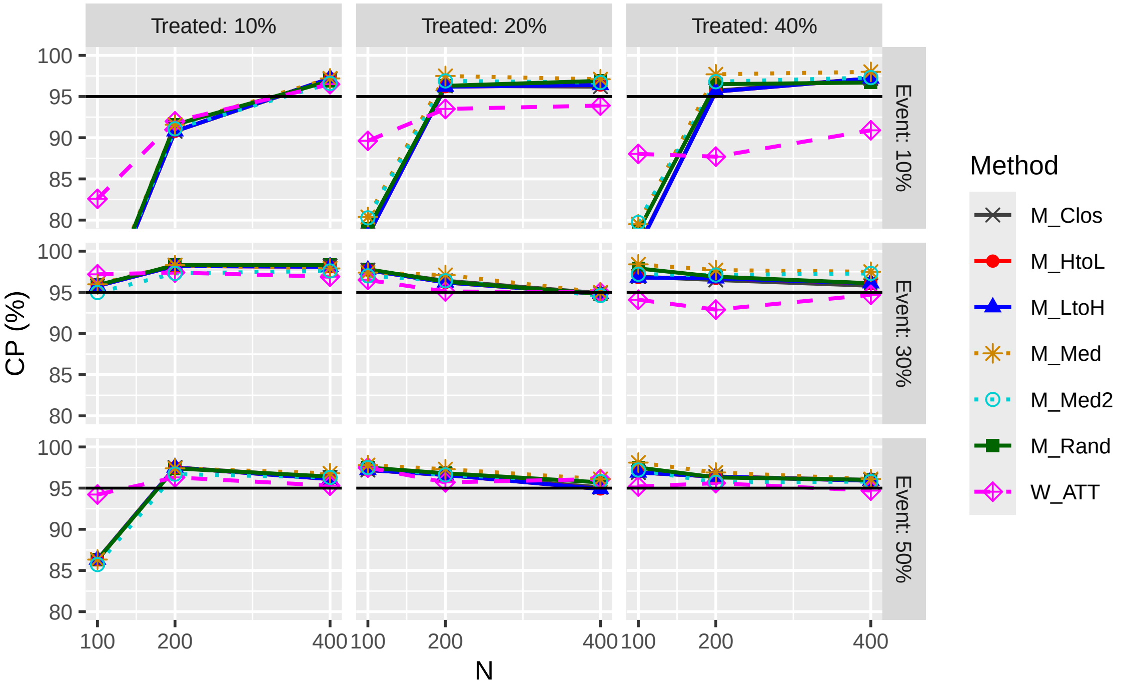

Figure S287. Coverage probability of confidence interval for OR (categorical covariate, matching ratio 1:1, true OR: 0.75, c statistic: 0.85, robust inference).

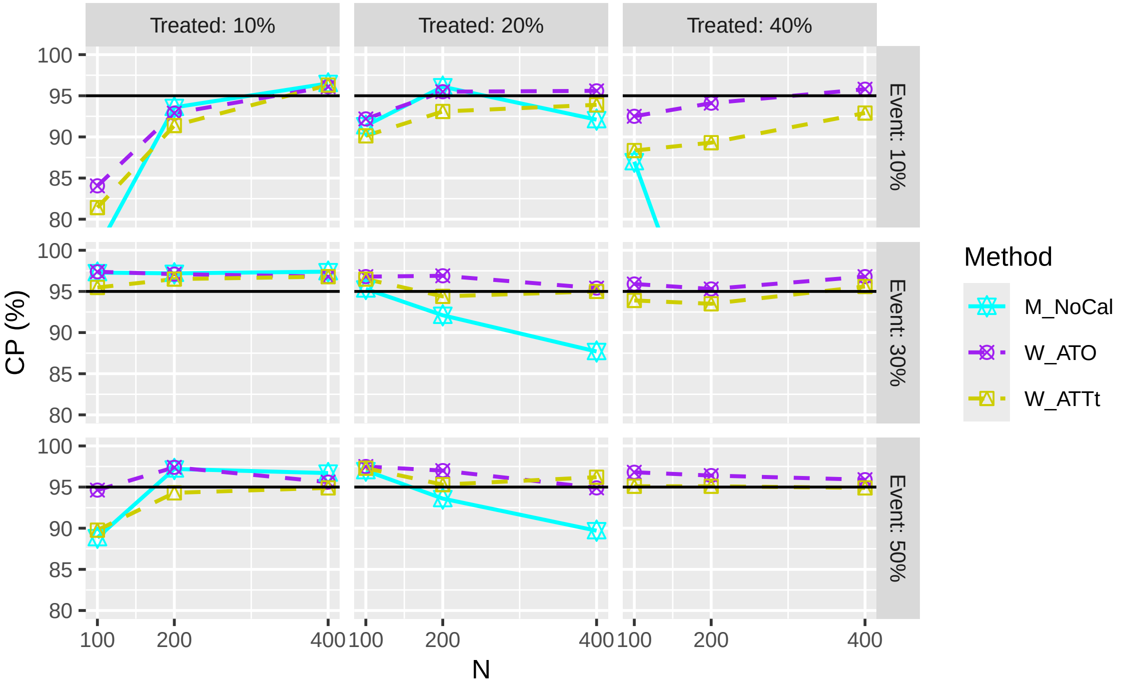

Figure S288. Coverage probability of confidence interval for OR (categorical covariate, matching ratio 1:1, true OR: 0.75, c statistic: 0.85, robust inference); other methods.

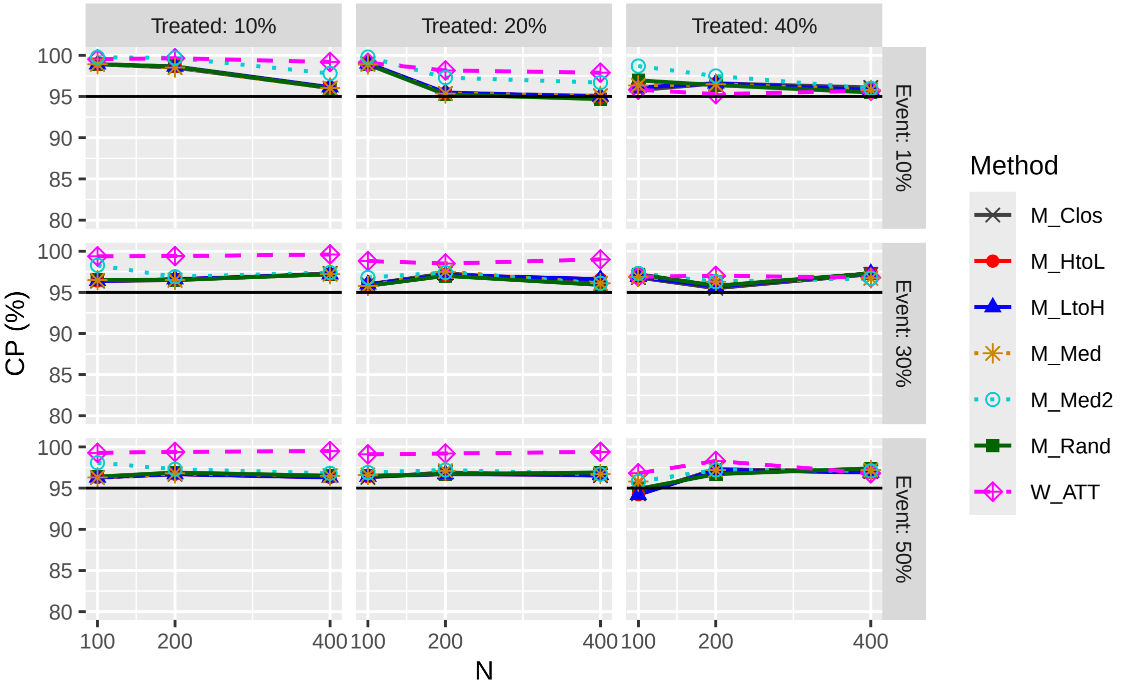

Figure S289. Coverage probability of confidence interval for OR (categorical covariate, matching ratio 1:1, true OR: 0.75, c statistic: 0.6, naive inference).

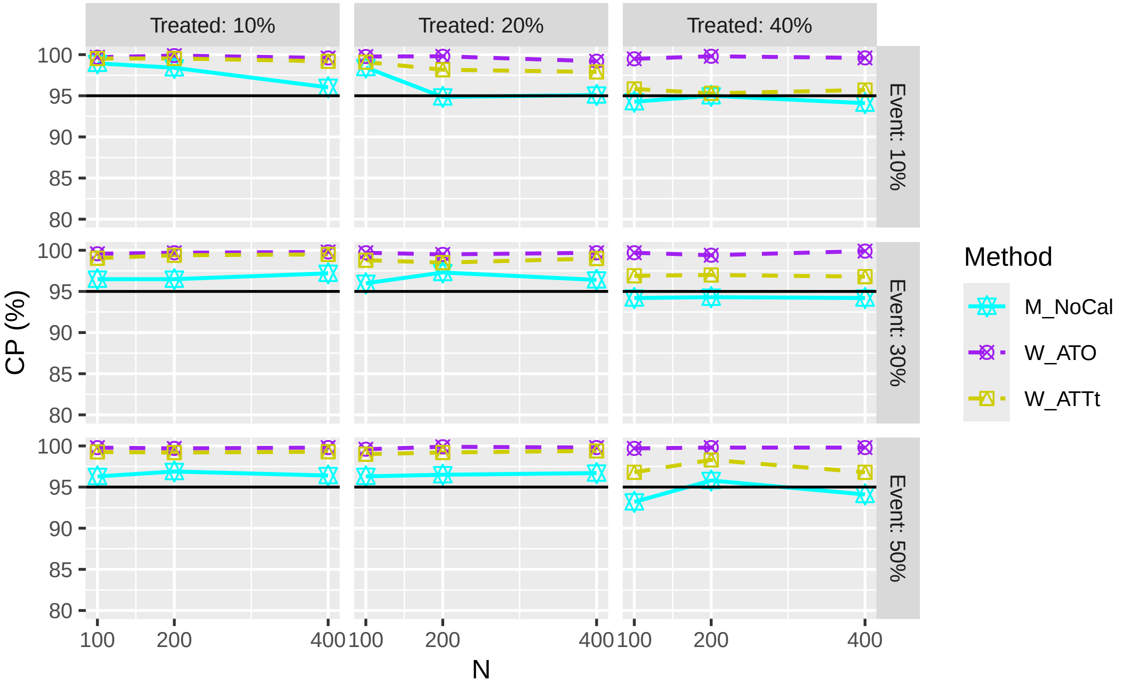

Figure S290. Coverage probability of confidence interval for OR (categorical covariate, matching ratio 1:1, true OR: 0.75, c statistic: 0.6, naive inference); other methods.

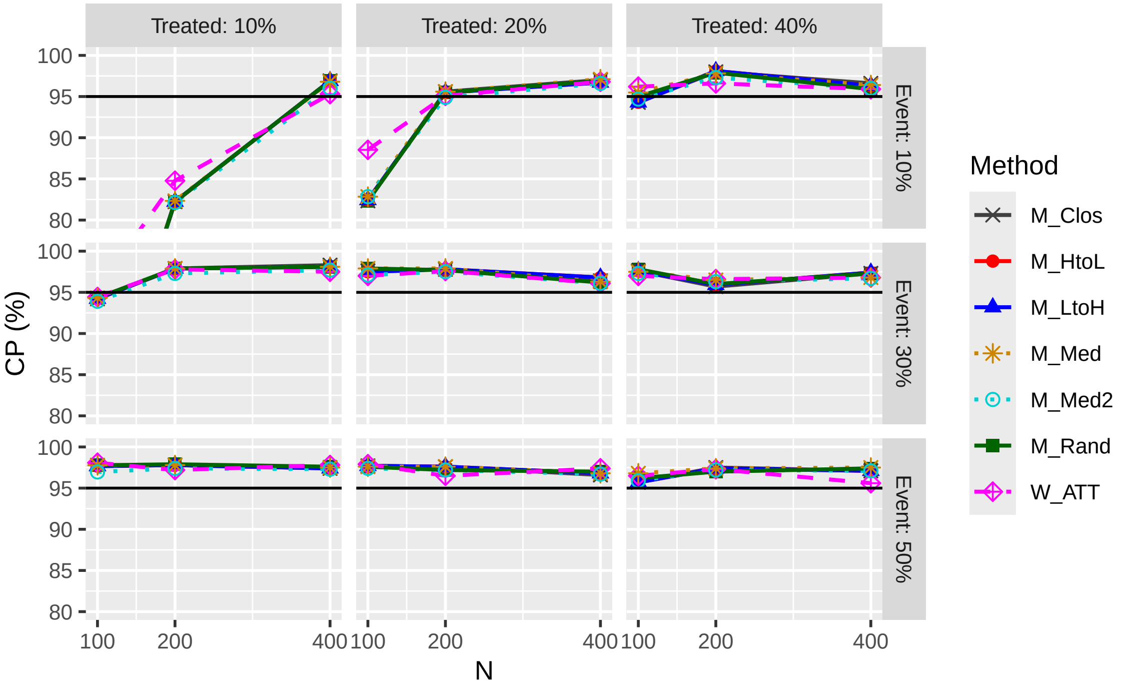

Figure S291. Coverage probability of confidence interval for OR (categorical covariate, matching ratio 1:1, true OR: 0.75, c statistic: 0.6, robust inference).

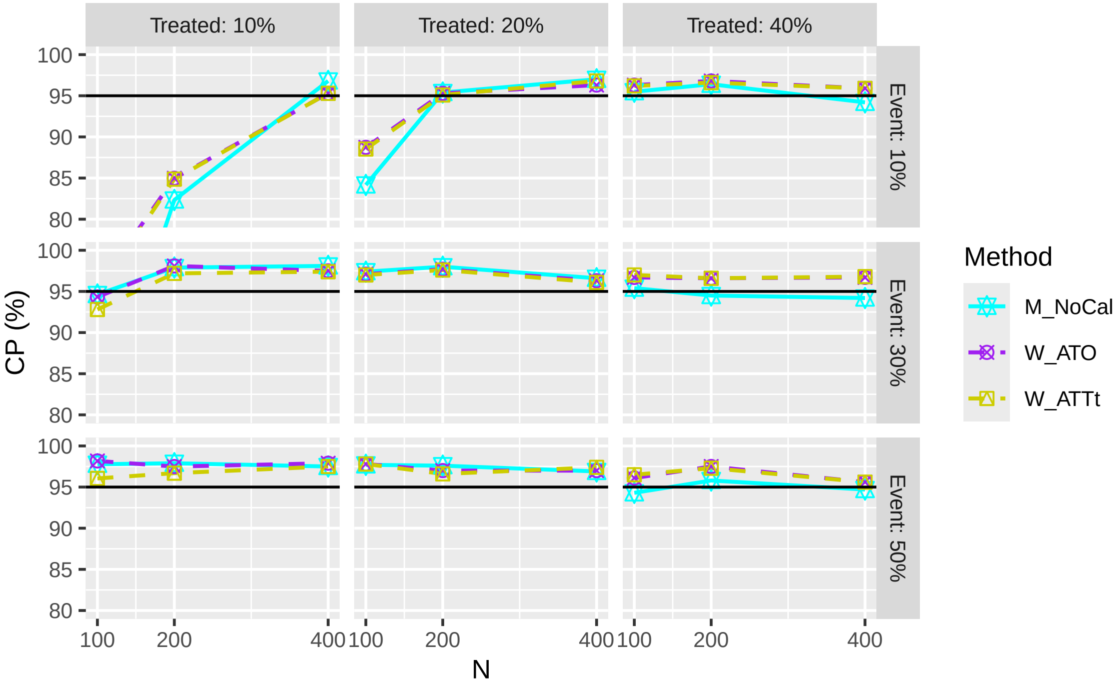

Figure S292. Coverage probability of confidence interval for OR (categorical covariate, matching ratio 1:1, true OR: 0.75, c statistic: 0.6, robust inference); other methods.

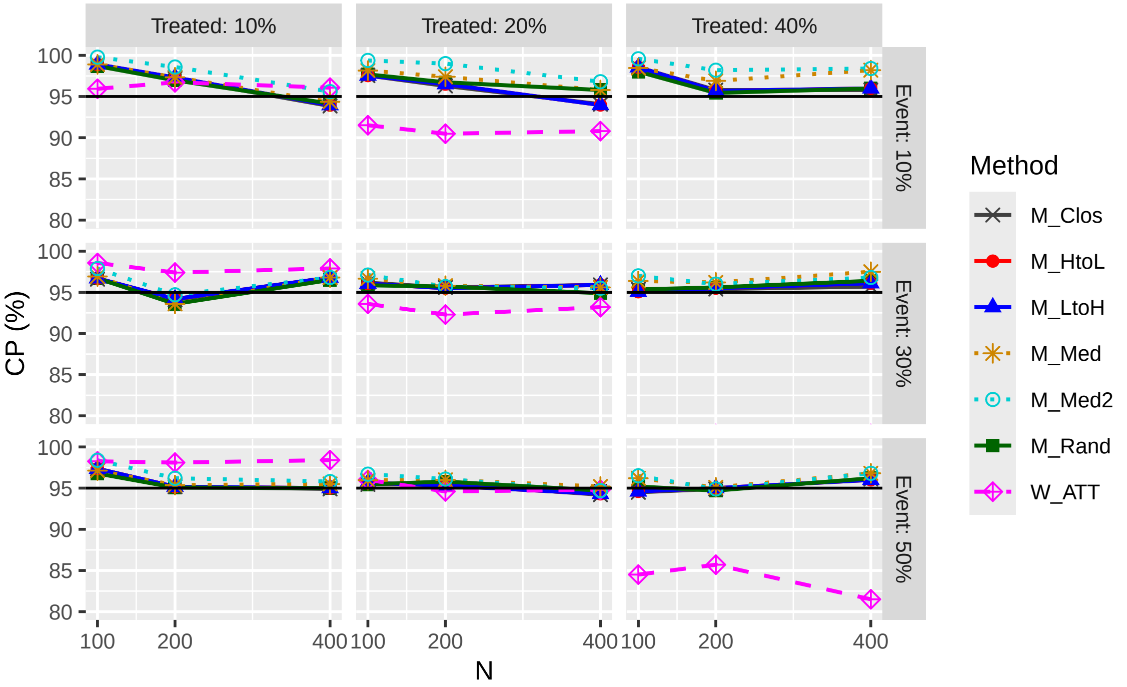

Figure S293. Coverage probability of confidence interval for OR (categorical covariate, matching ratio 1:1, true OR: 0.5, c statistic: 0.85, naive inference).

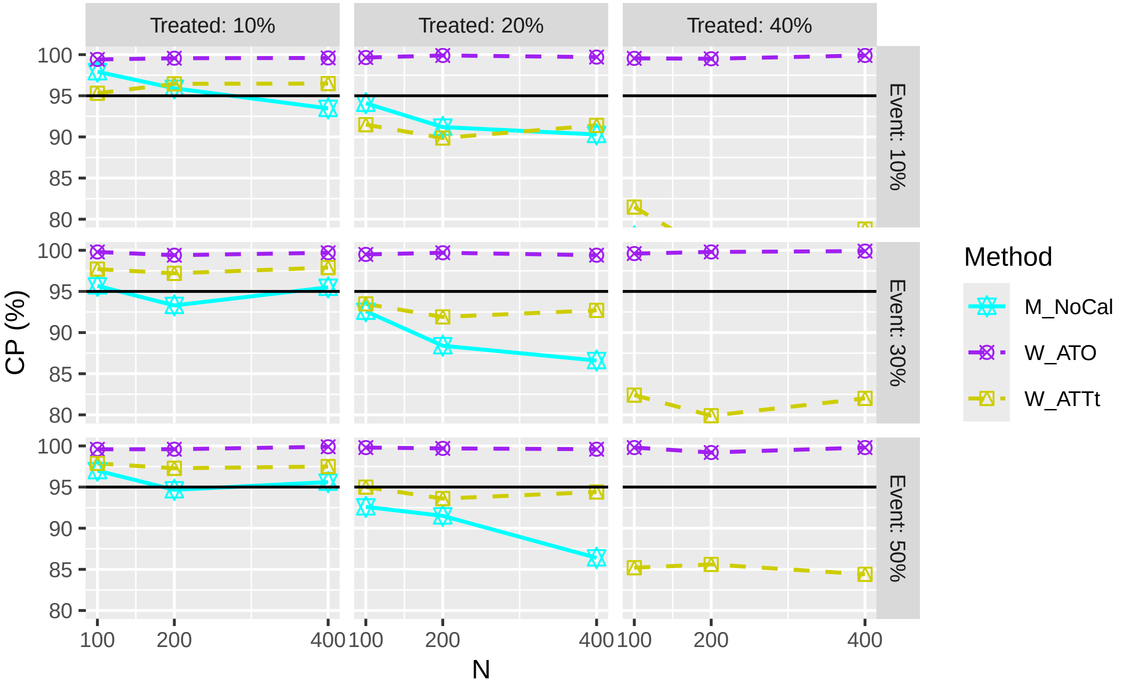

Figure S294. Coverage probability of confidence interval for OR (categorical covariate, matching ratio 1:1, true OR: 0.5, c statistic: 0.85, naive inference); other methods.

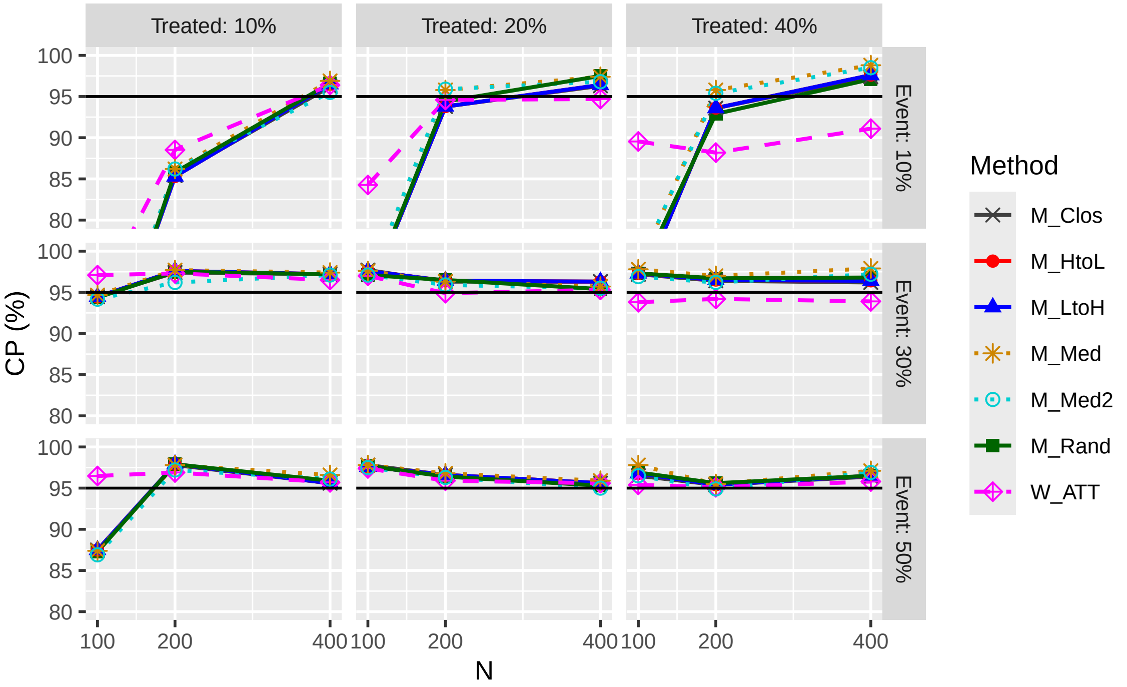

Figure S295. Coverage probability of confidence interval for OR (categorical covariate, matching ratio 1:1, true OR: 0.5, c statistic: 0.85, robust inference).

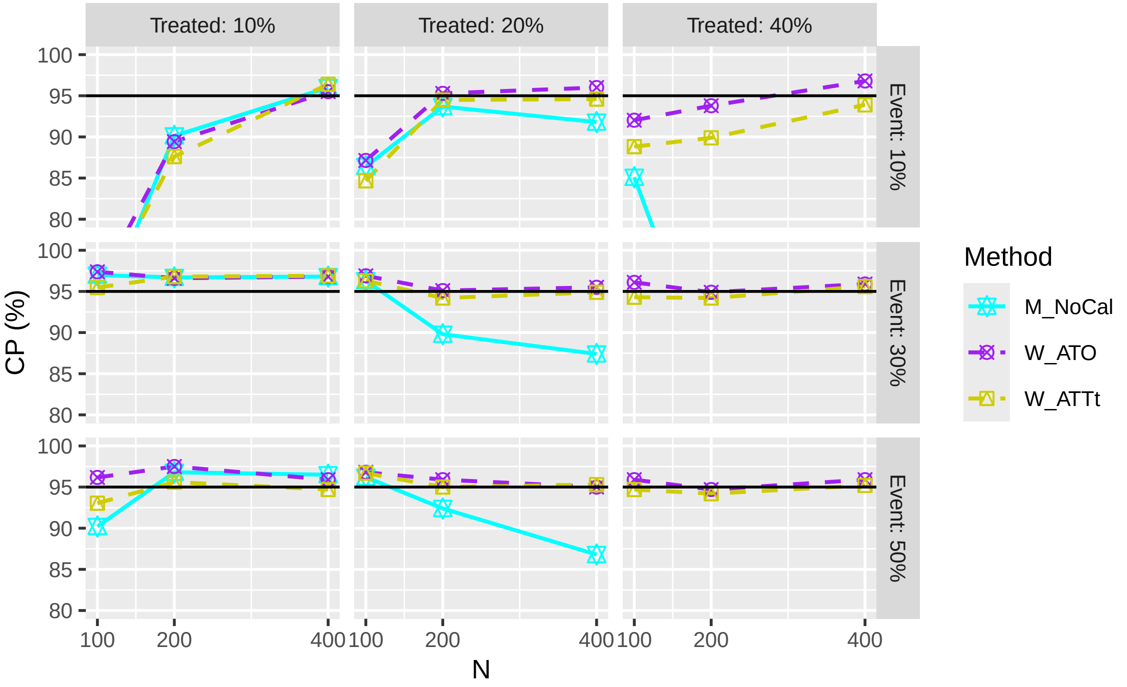

Figure S296. Coverage probability of confidence interval for OR (categorical covariate, matching ratio 1:1, true OR: 0.5, c statistic: 0.85, robust inference); other methods.

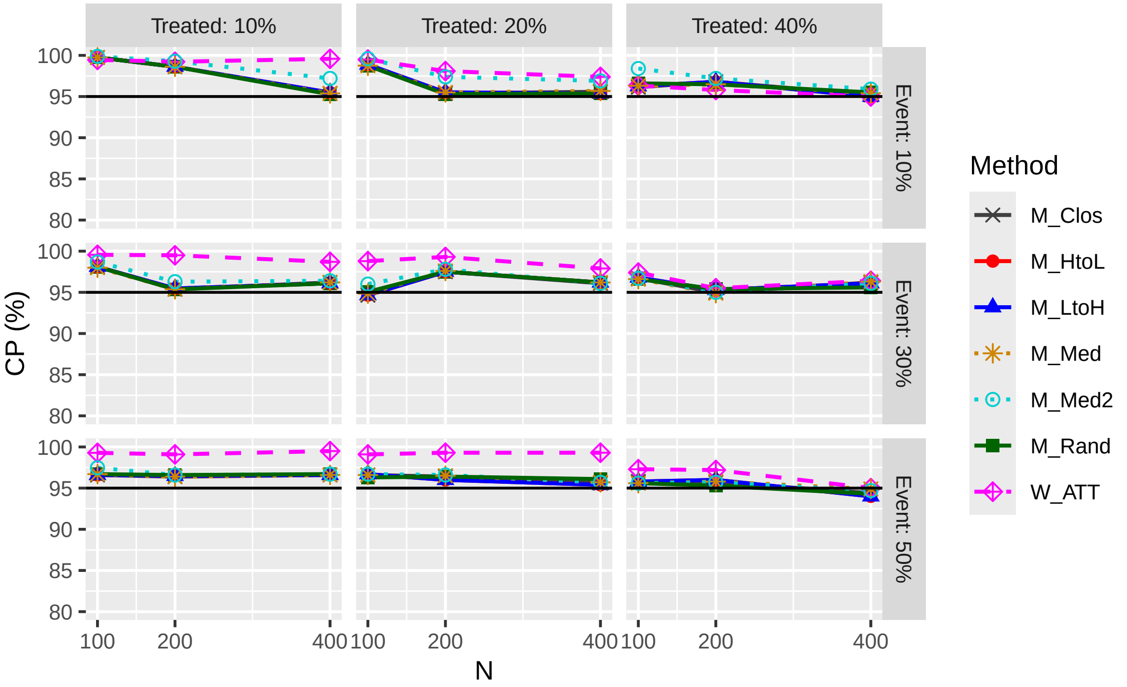

Figure S297. Coverage probability of confidence interval for OR (categorical covariate, matching ratio 1:1, true OR: 0.5, c statistic: 0.6, naive inference).

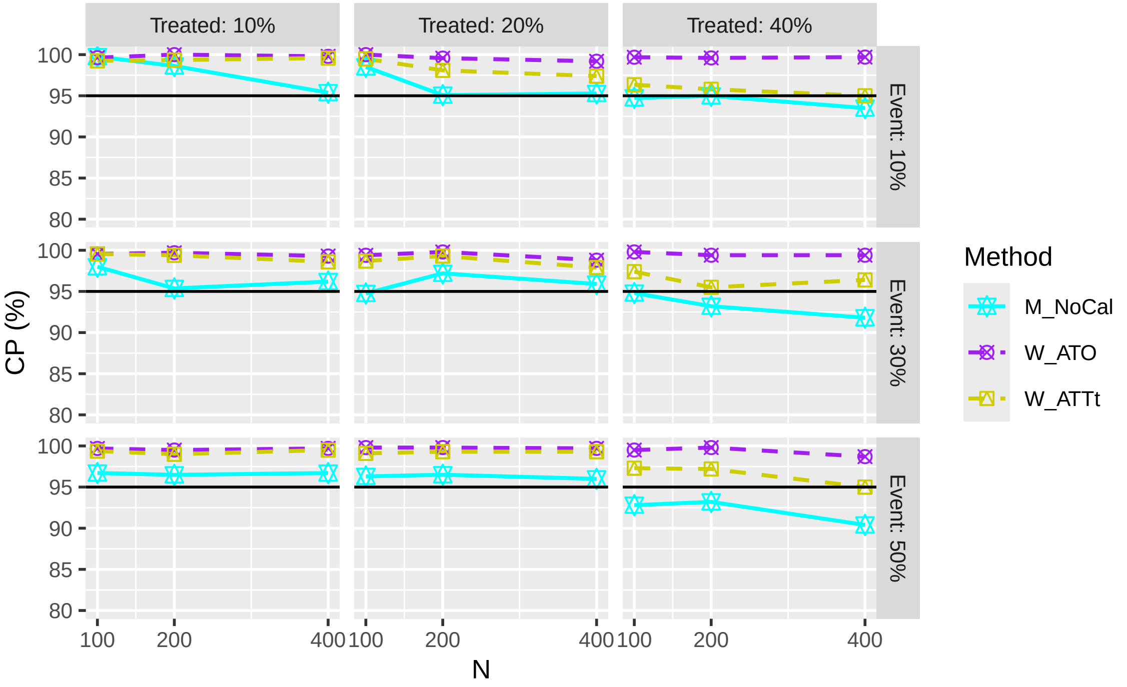

Figure S298. Coverage probability of confidence interval for OR (categorical covariate, matching ratio 1:1, true OR: 0.5, c statistic: 0.6, naive inference); other methods.

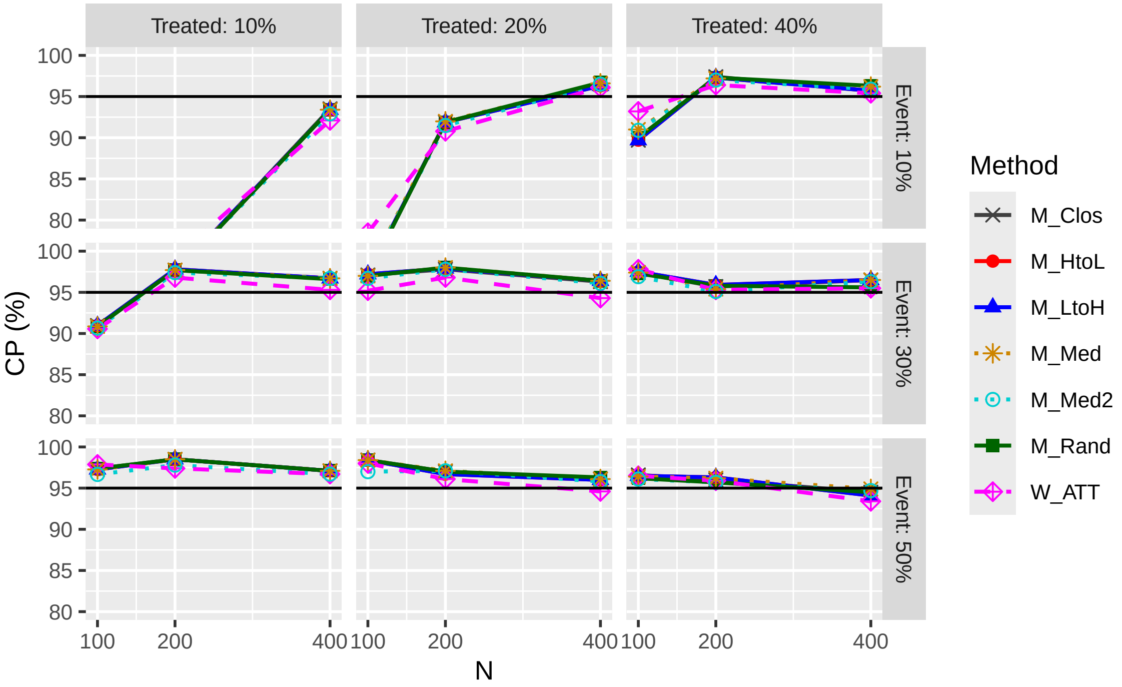

Figure S299. Coverage probability of confidence interval for OR (categorical covariate, matching ratio 1:1, true OR: 0.5, c statistic: 0.6, robust inference).

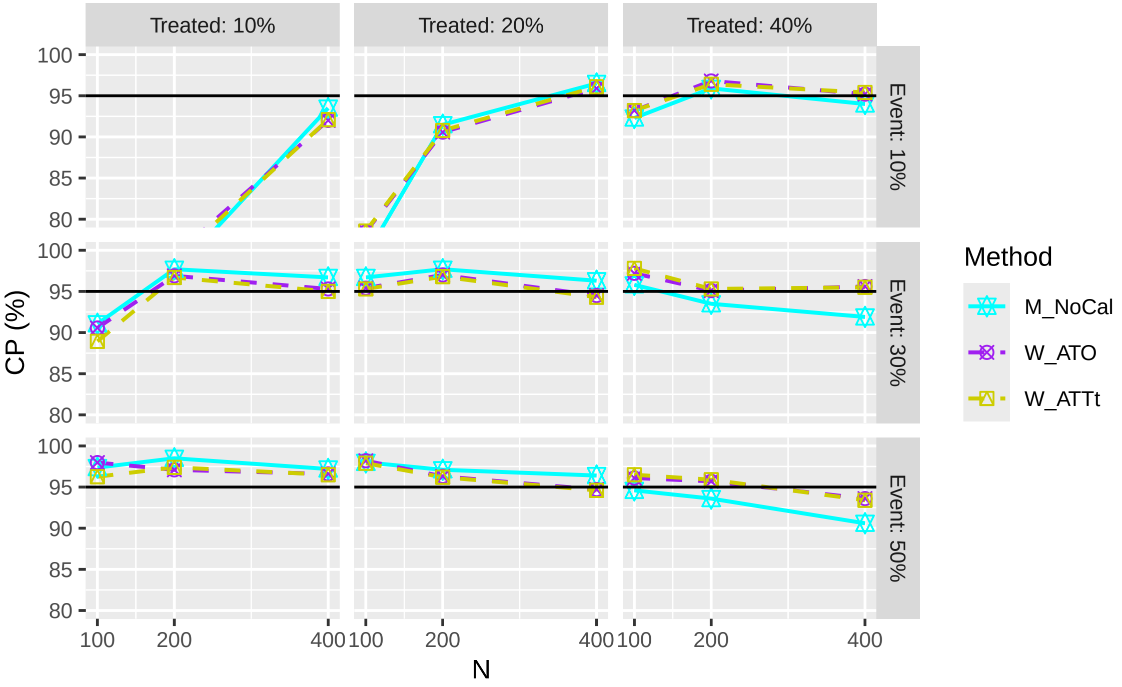

Figure S300. Coverage probability of confidence interval for OR (categorical covariate, matching ratio 1:1, true OR: 0.5, c statistic: 0.6, robust inference); other methods.

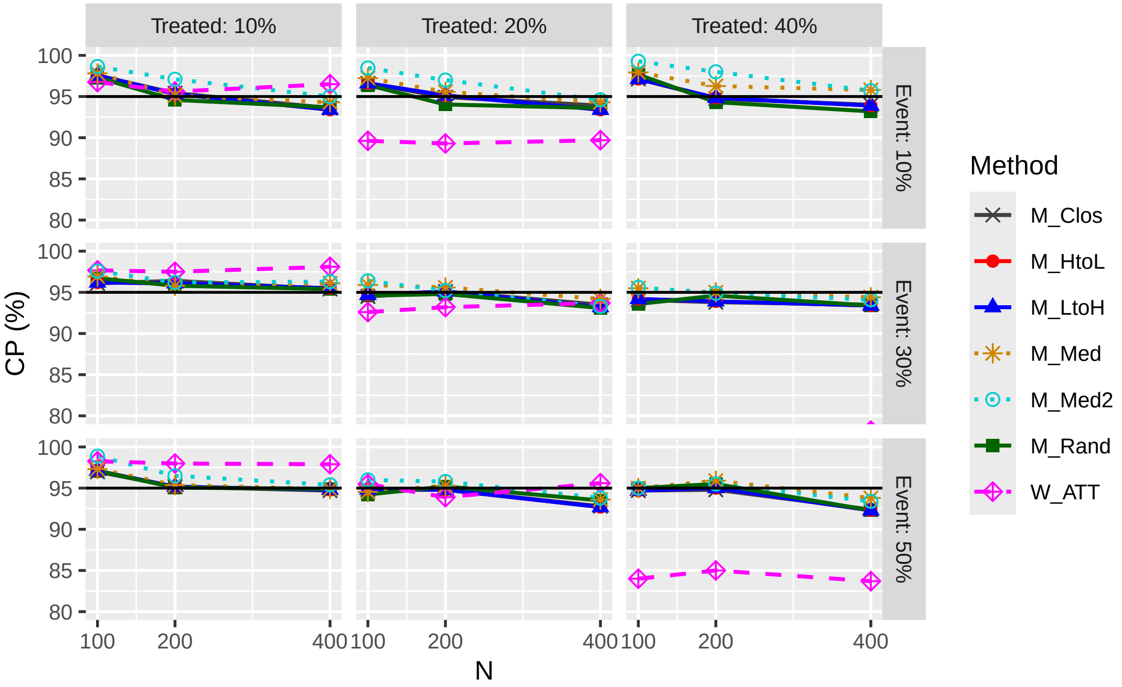

Figure S301. Coverage probability of confidence interval for OR (categorical covariate, matching ratio 1:2, true OR: 1, c statistic: 0.85, naive inference).

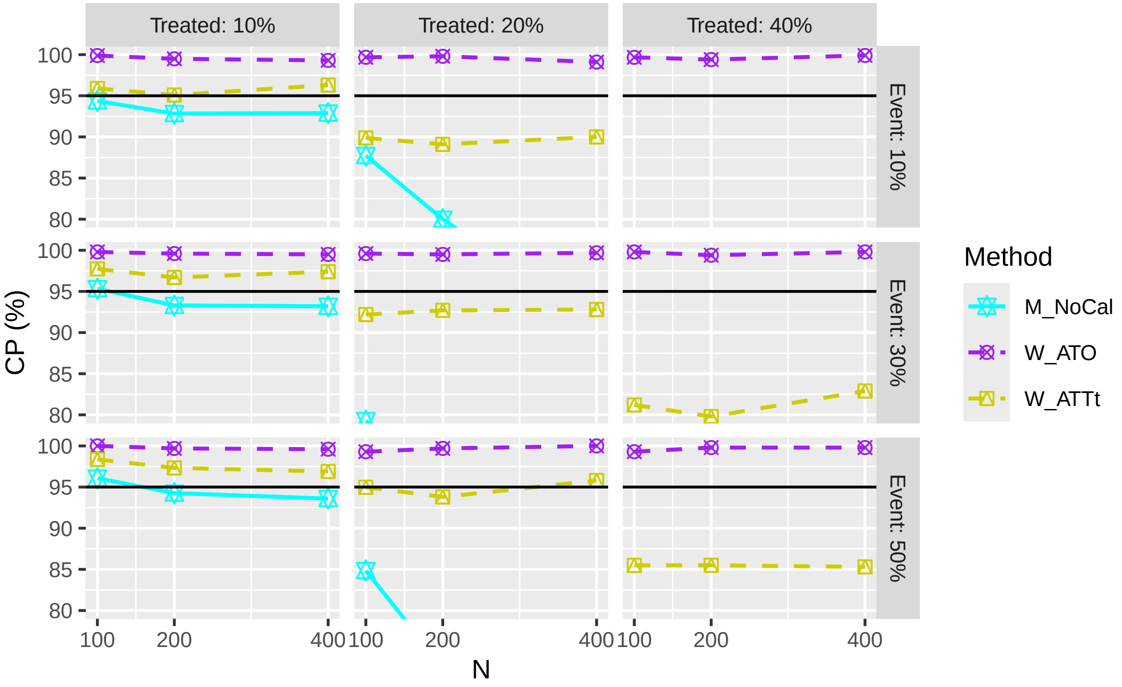

Figure S302. Coverage probability of confidence interval for OR (categorical covariate, matching ratio 1:2, true OR: 1, c statistic: 0.85, naive inference); other methods.

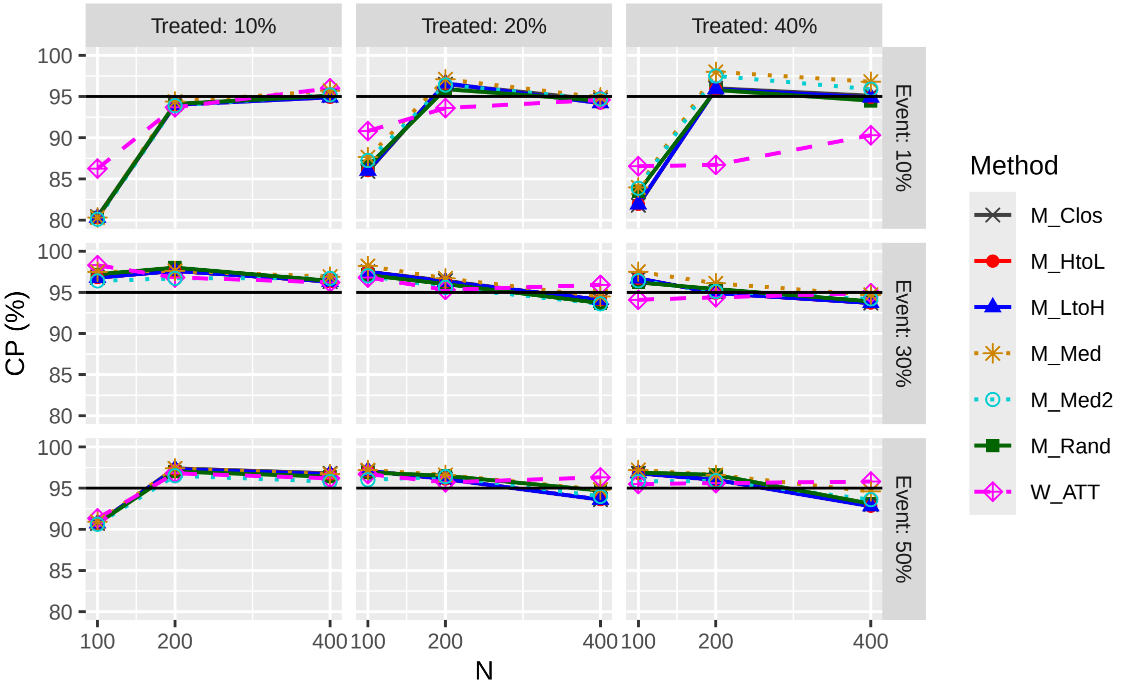

Figure S303. Coverage probability of confidence interval for OR (categorical covariate, matching ratio 1:2, true OR: 1, c statistic: 0.85, robust inference).

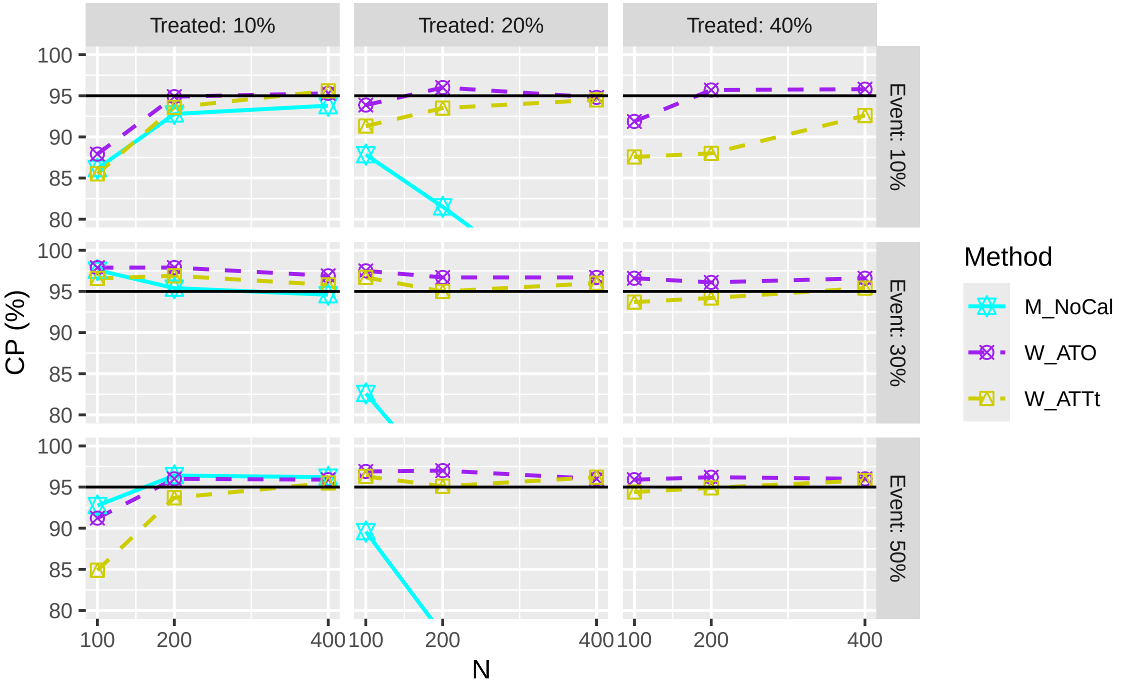

Figure S304. Coverage probability of confidence interval for OR (categorical covariate, matching ratio 1:2, true OR: 1, c statistic: 0.85, robust inference); other methods.

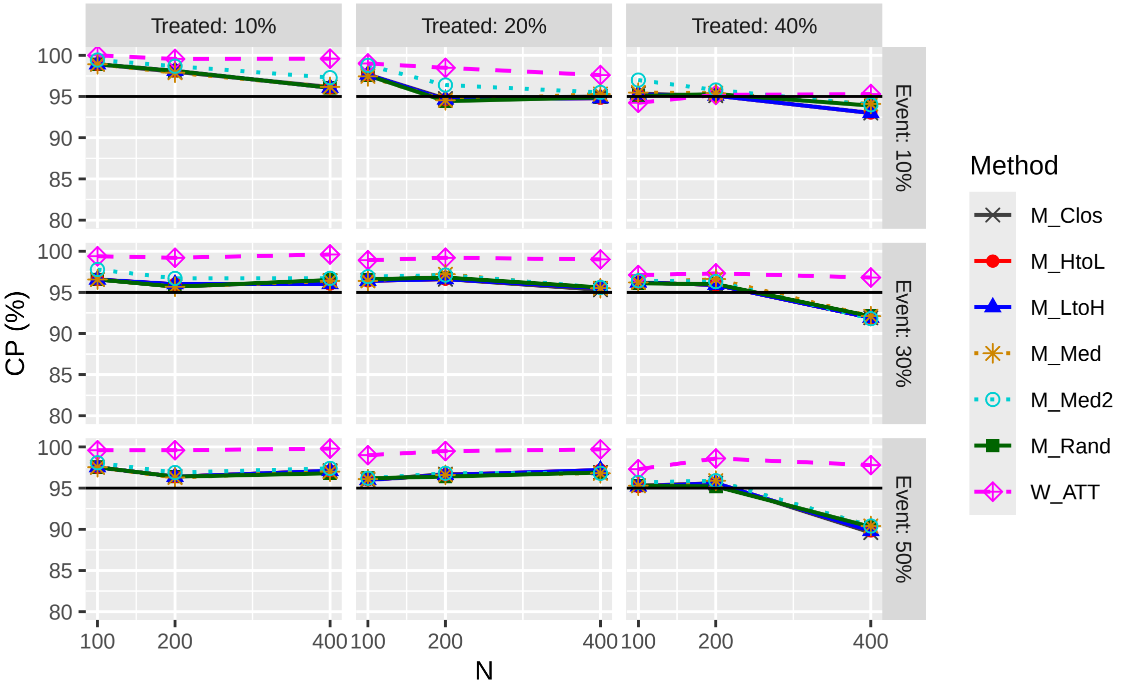

Figure S305. Coverage probability of confidence interval for OR (categorical covariate, matching ratio 1:2, true OR: 1, c statistic: 0.6, naive inference).

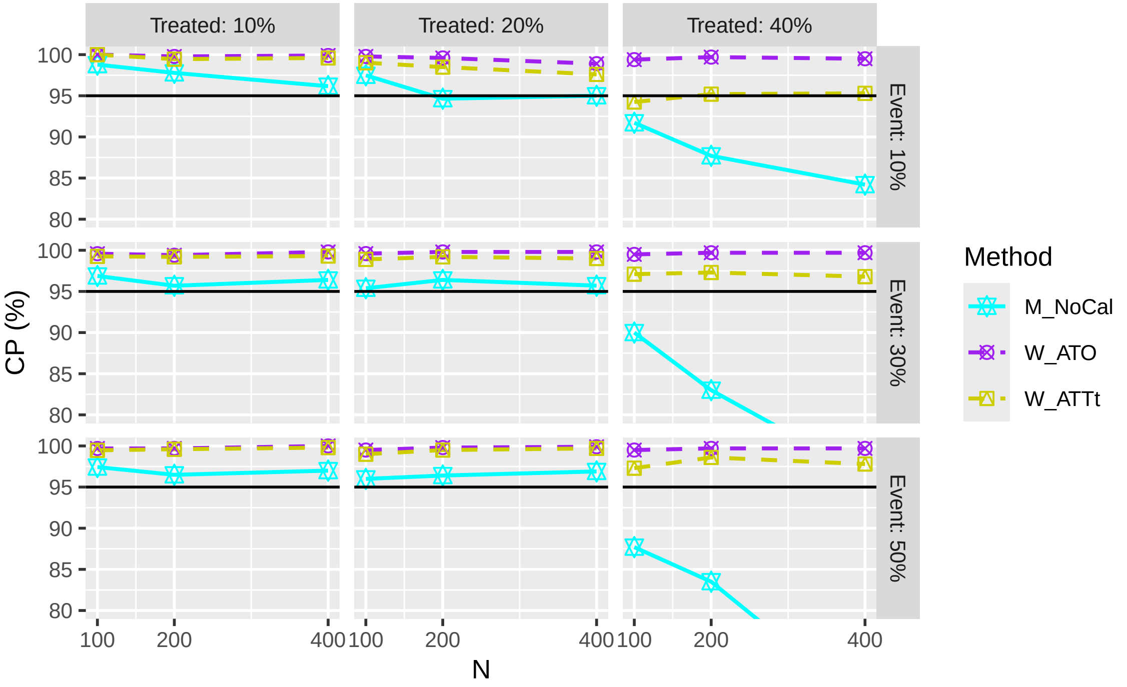

Figure S306. Coverage probability of confidence interval for OR (categorical covariate, matching ratio 1:2, true OR: 1, c statistic: 0.6, naive inference); other methods.

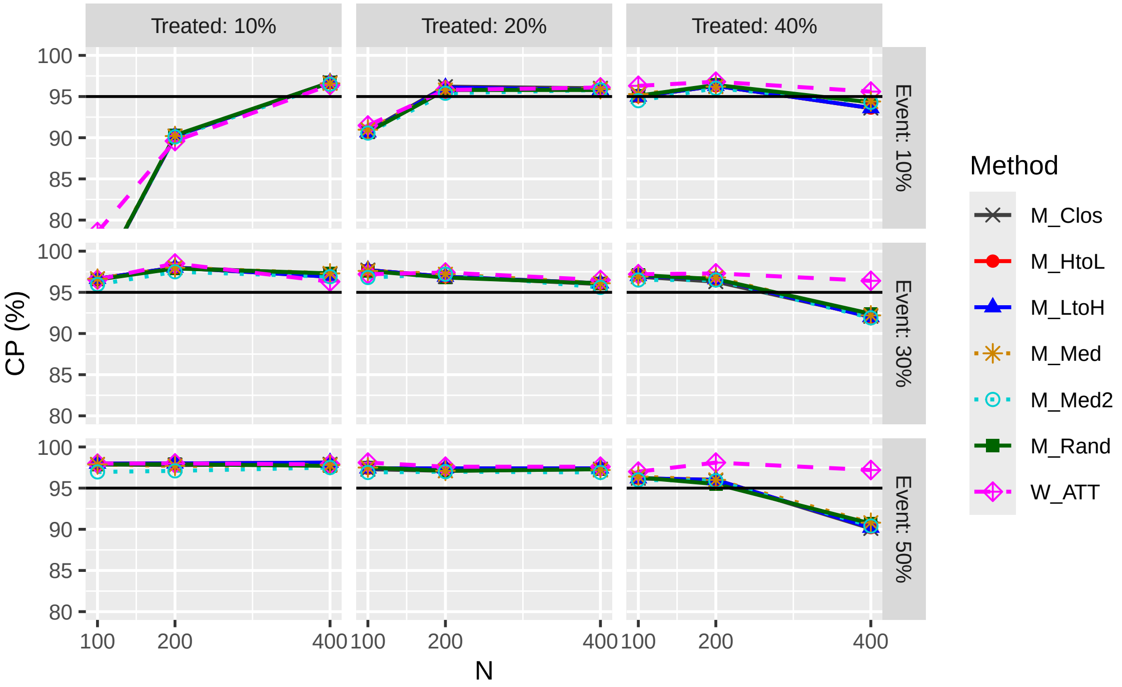

Figure S307. Coverage probability of confidence interval for OR (categorical covariate, matching ratio 1:2, true OR: 1, c statistic: 0.6, robust inference).

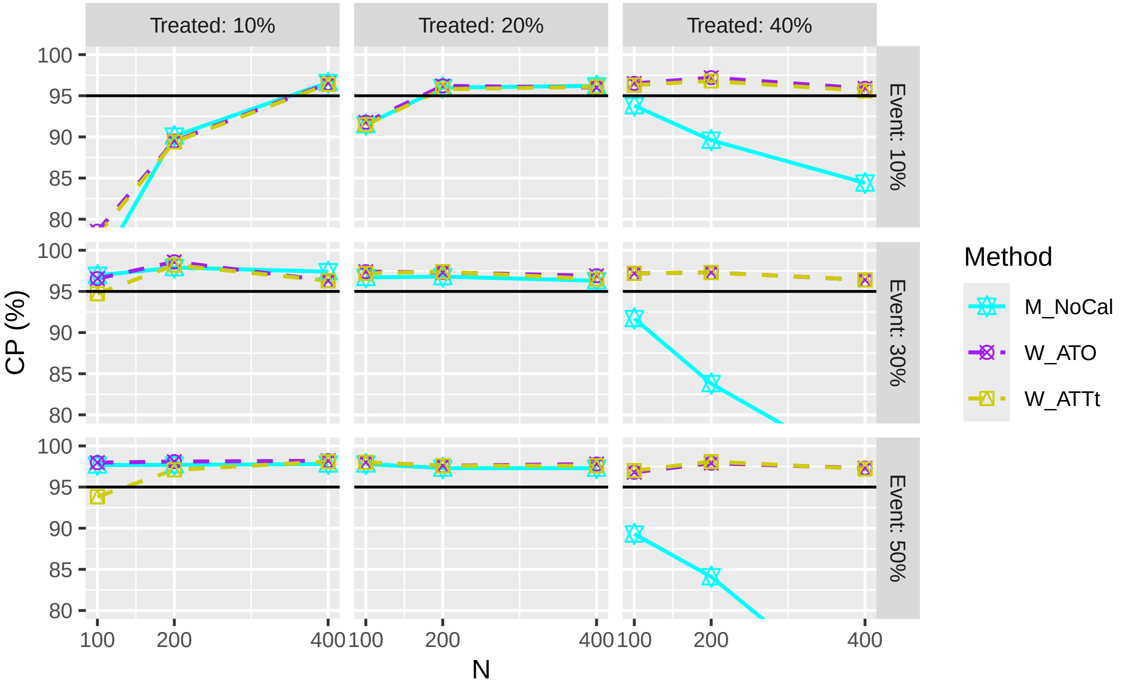

Figure S308. Coverage probability of confidence interval for OR (categorical covariate, matching ratio 1:2, true OR: 1, c statistic: 0.6, robust inference); other methods.

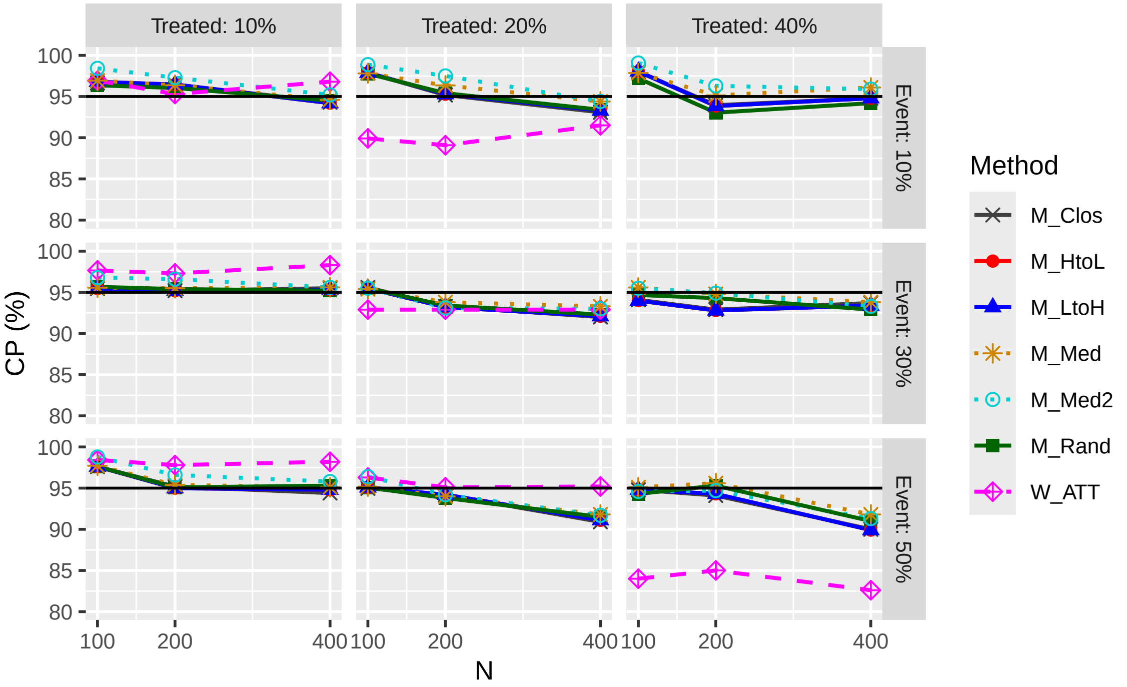

Figure S309. Coverage probability of confidence interval for OR (categorical covariate, matching ratio 1:2, true OR: 0.75, c statistic: 0.85, naive inference).

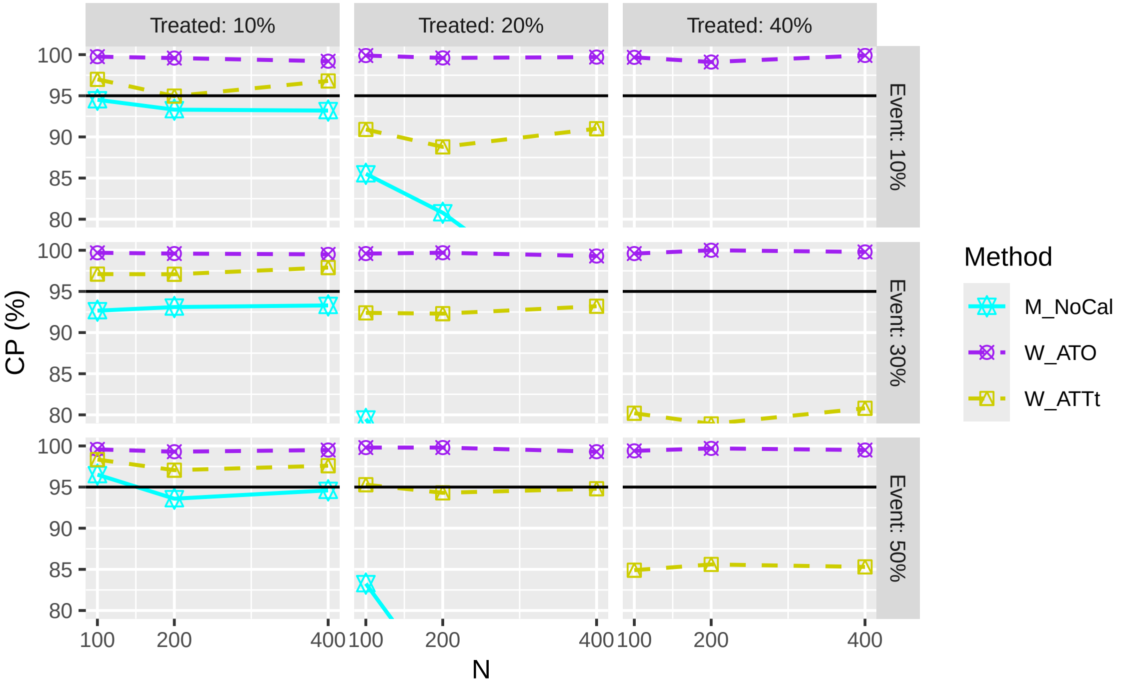

Figure S310. Coverage probability of confidence interval for OR (categorical covariate, matching ratio 1:2, true OR: 0.75, c statistic: 0.85, naive inference); other methods.

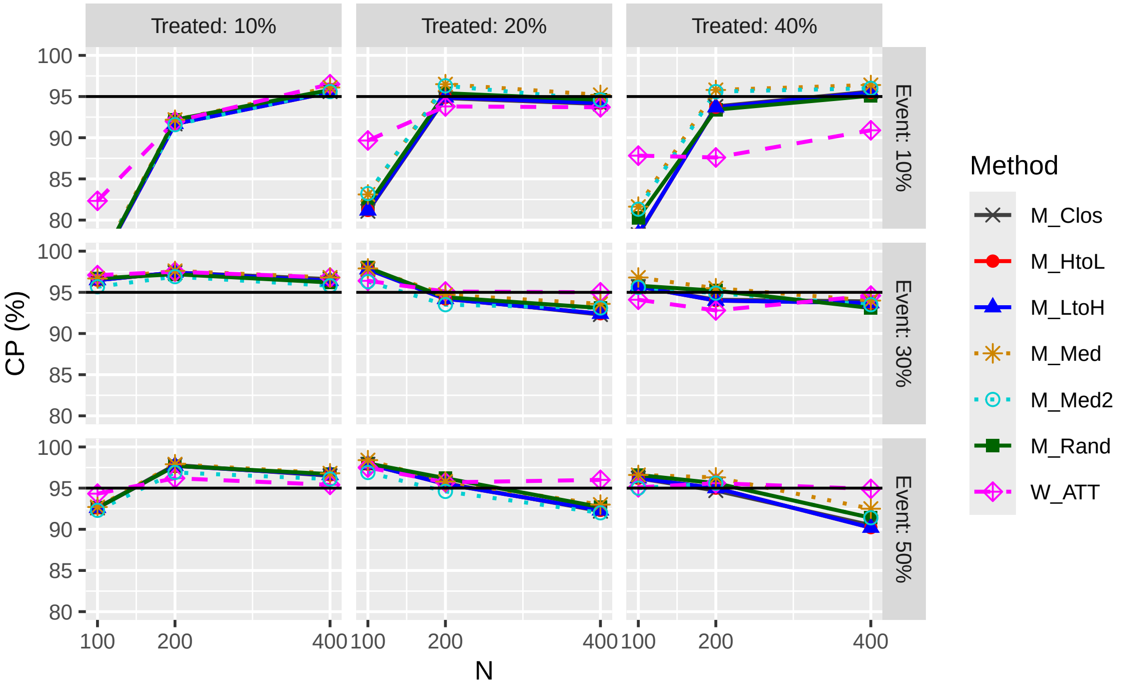

Figure S311. Coverage probability of confidence interval for OR (categorical covariate, matching ratio 1:2, true OR: 0.75, c statistic: 0.85, robust inference).

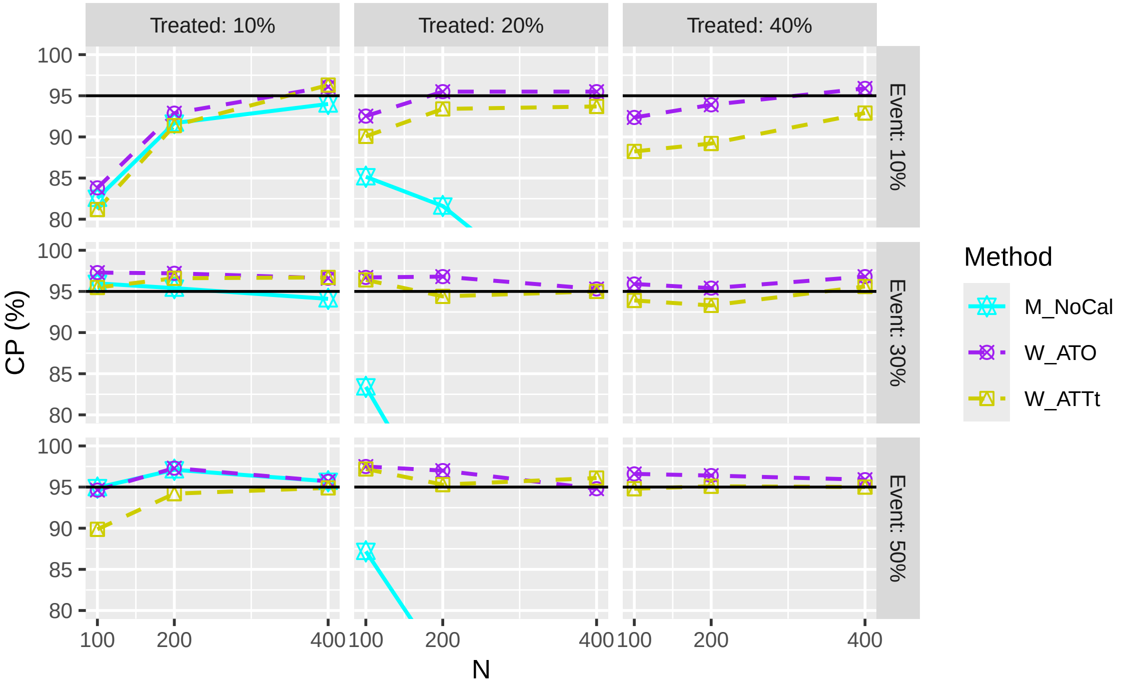

Figure S312. Coverage probability of confidence interval for OR (categorical covariate, matching ratio 1:2, true OR: 0.75, c statistic: 0.85, robust inference); other methods.

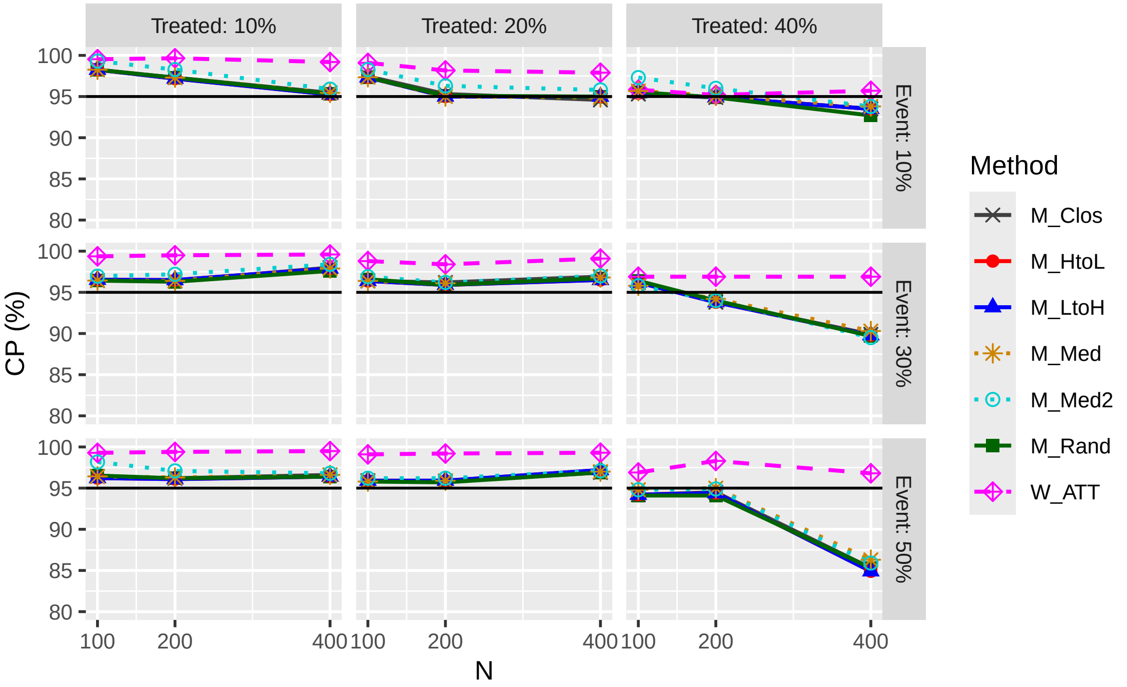

Figure S313. Coverage probability of confidence interval for OR (categorical covariate, matching ratio 1:2, true OR: 0.75, c statistic: 0.6, naive inference).

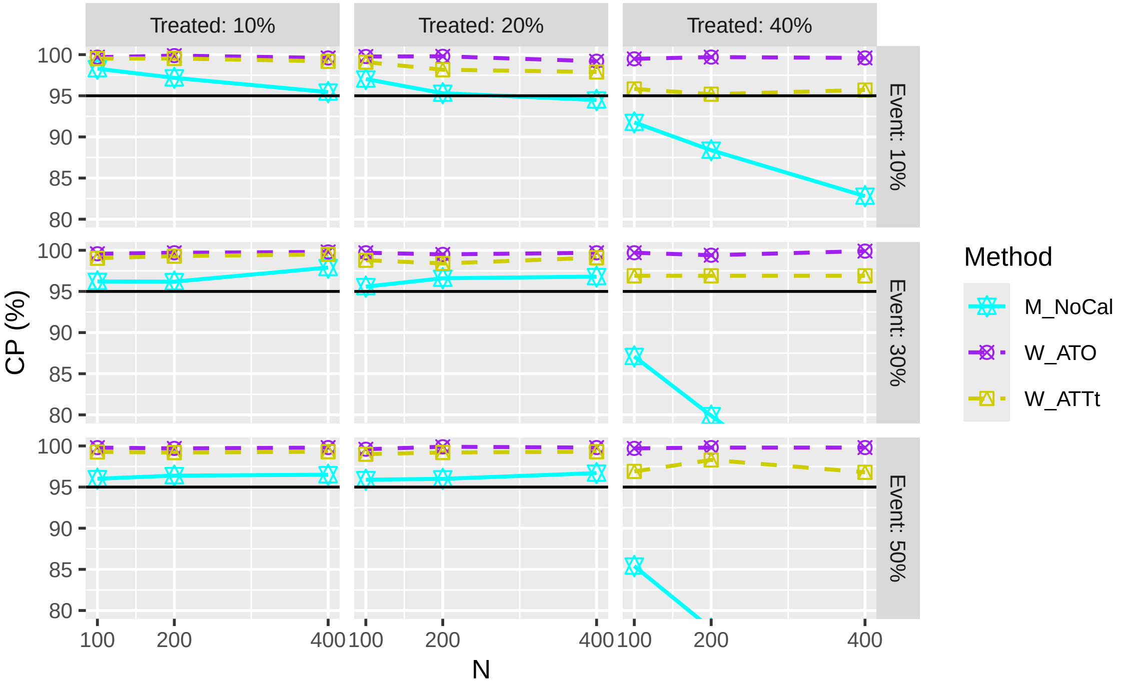

Figure S314. Coverage probability of confidence interval for OR (categorical covariate, matching ratio 1:2, true OR: 0.75, c statistic: 0.6, naive inference); other methods.

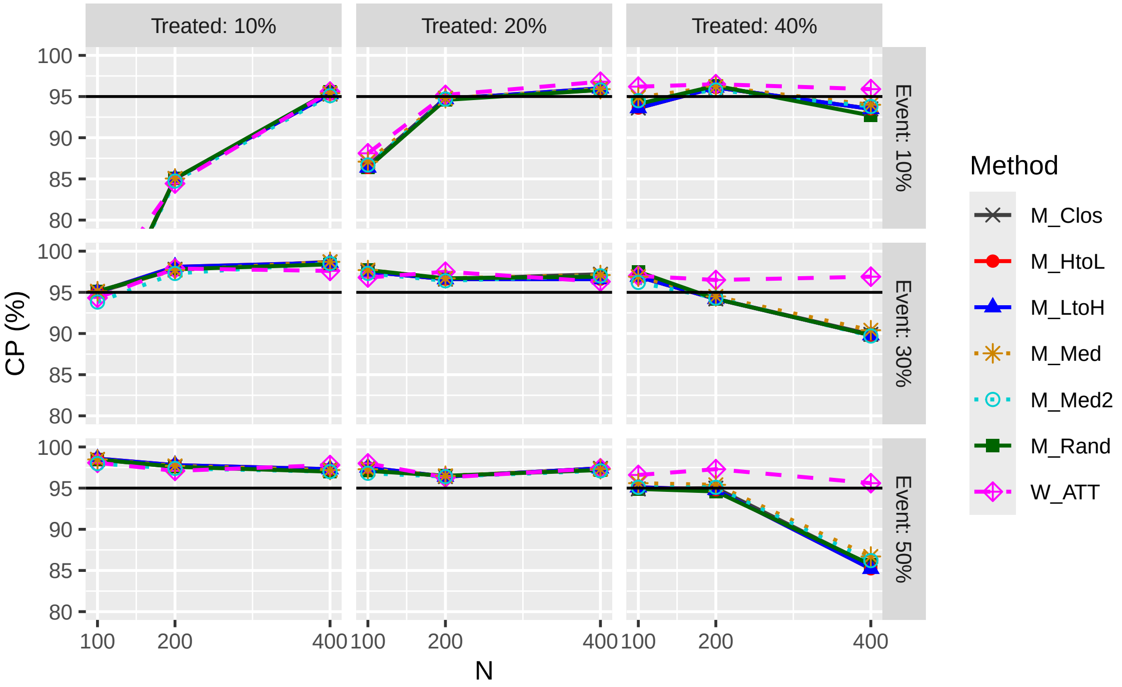

Figure S315. Coverage probability of confidence interval for OR (categorical covariate, matching ratio 1:2, true OR: 0.75, c statistic: 0.6, robust inference).

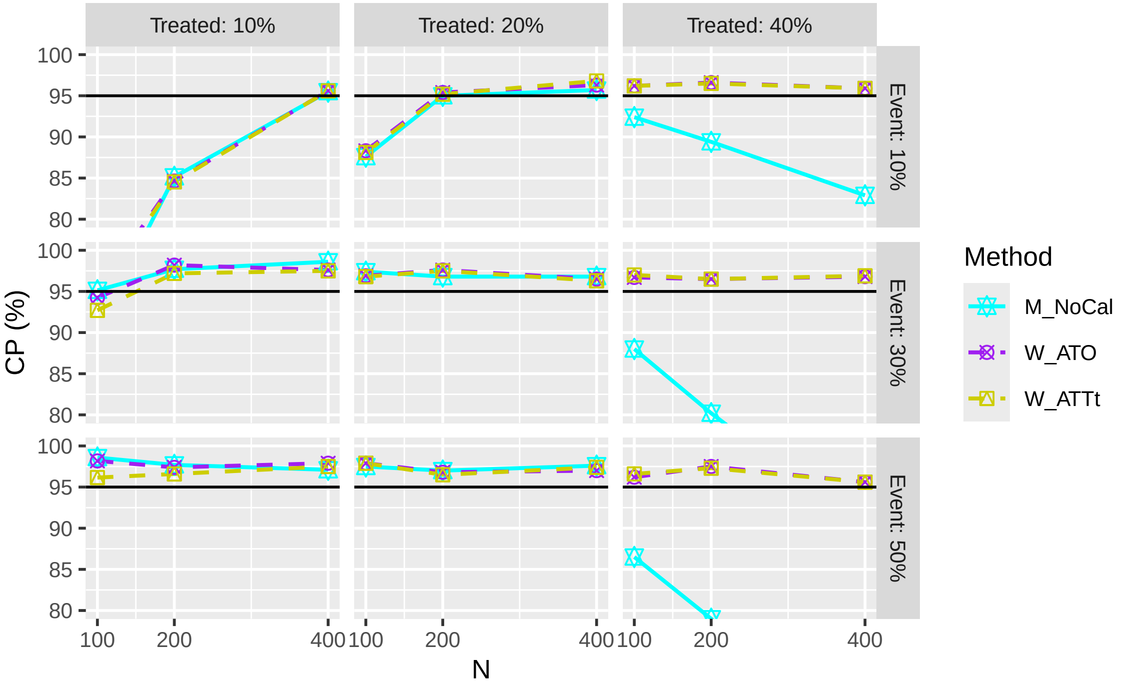

Figure S316. Coverage probability of confidence interval for OR (categorical covariate, matching ratio 1:2, true OR: 0.75, c statistic: 0.6, robust inference); other methods.

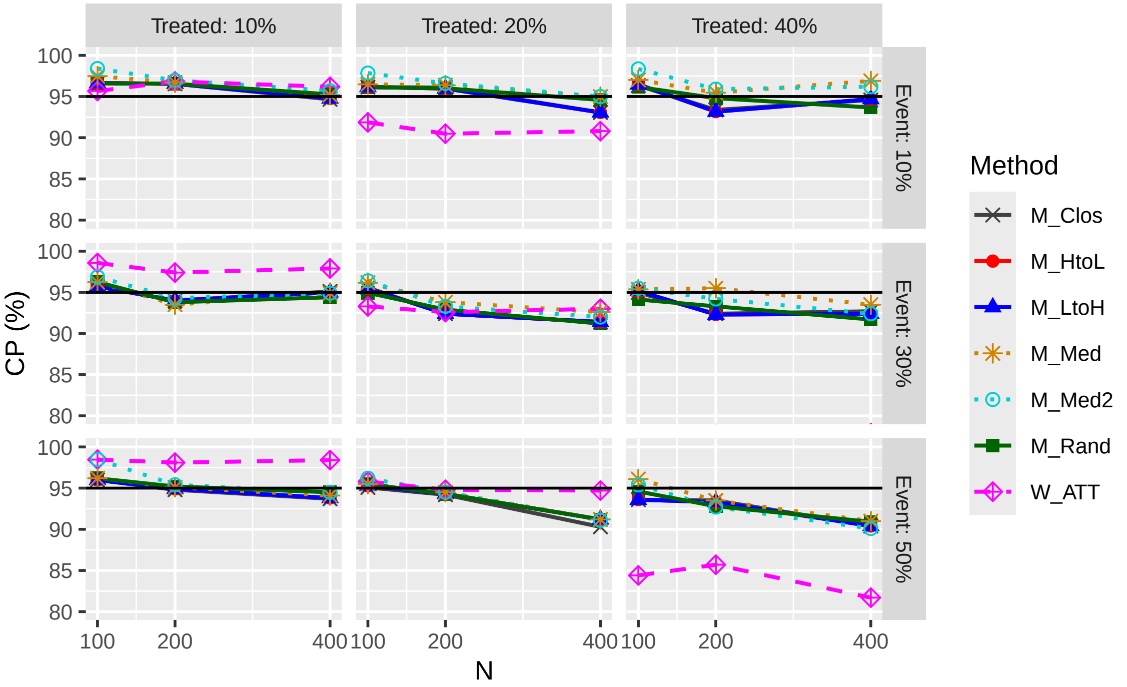

Figure S317. Coverage probability of confidence interval for OR (categorical covariate, matching ratio 1:2, true OR: 0.5, c statistic: 0.85, naive inference).

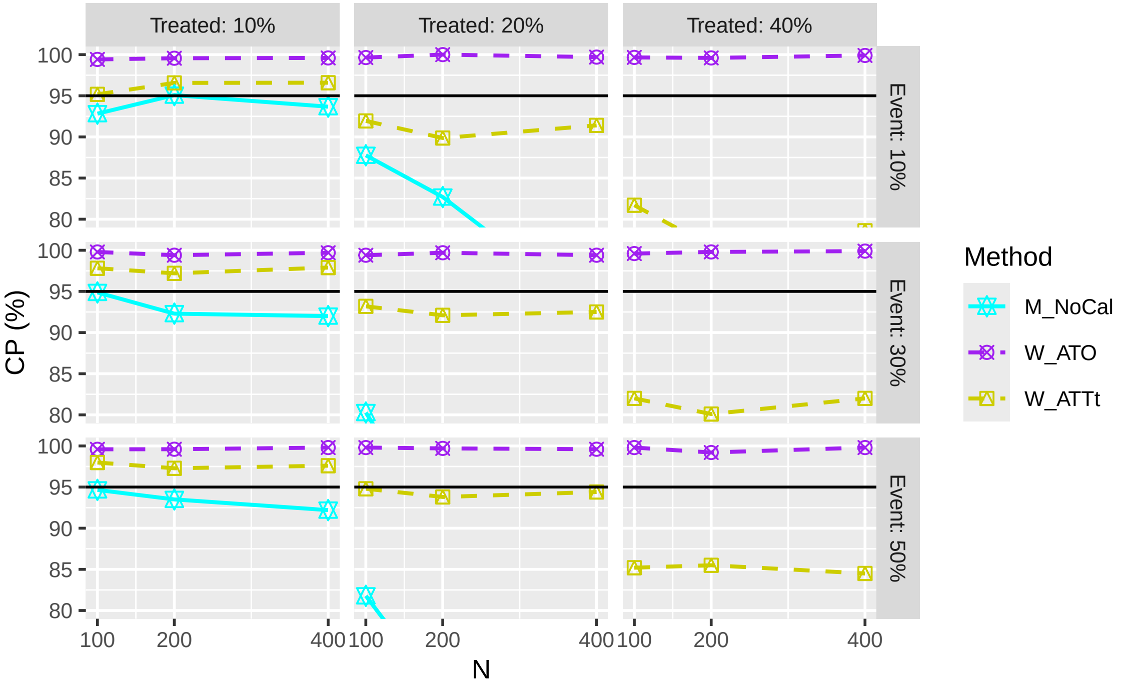

Figure S318. Coverage probability of confidence interval for OR (categorical covariate, matching ratio 1:2, true OR: 0.5, c statistic: 0.85, naive inference); other methods.

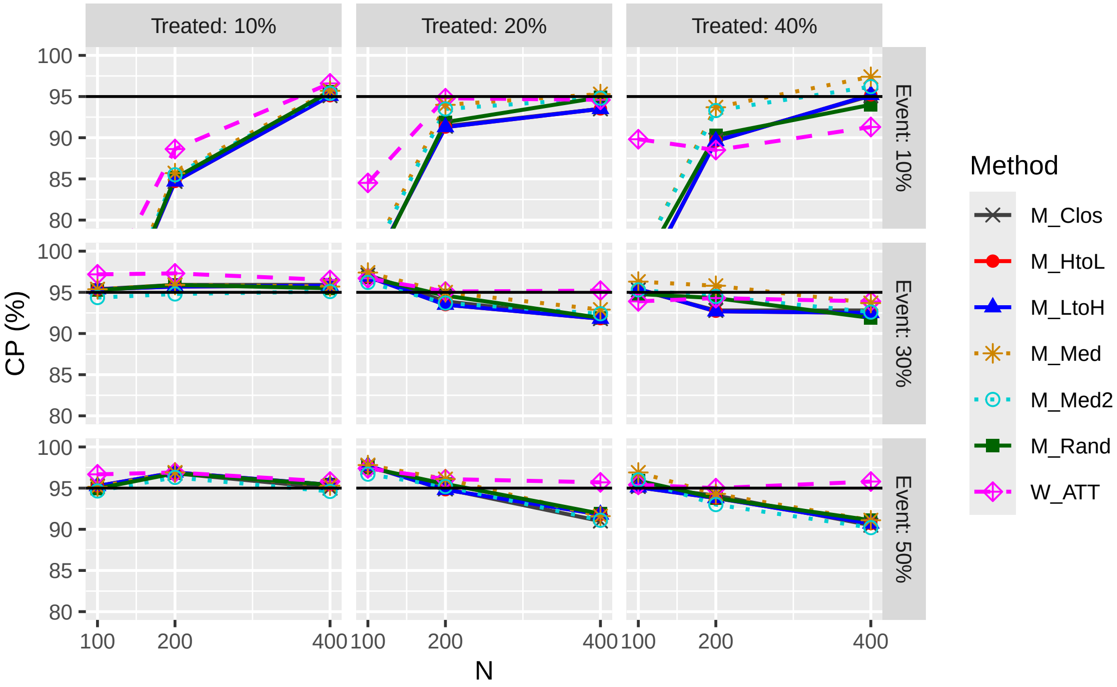

Figure S319. Coverage probability of confidence interval for OR (categorical covariate, matching ratio 1:2, true OR: 0.5, c statistic: 0.85, robust inference).

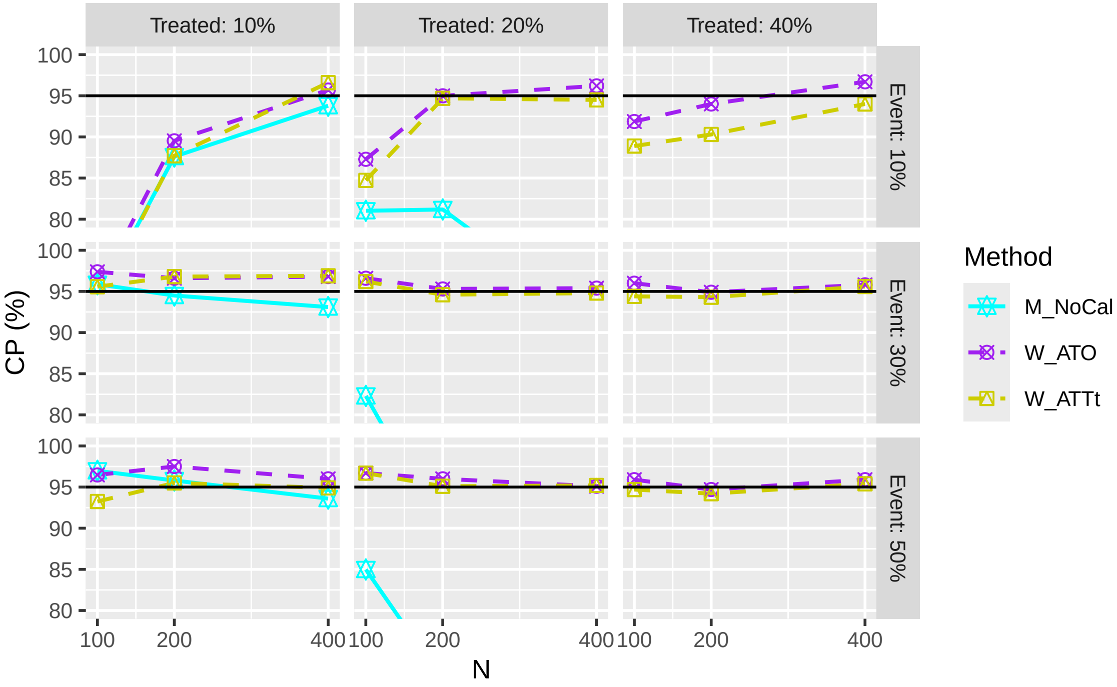

Figure S320. Coverage probability of confidence interval for OR (categorical covariate, matching ratio 1:2, true OR: 0.5, c statistic: 0.85, robust inference); other methods.

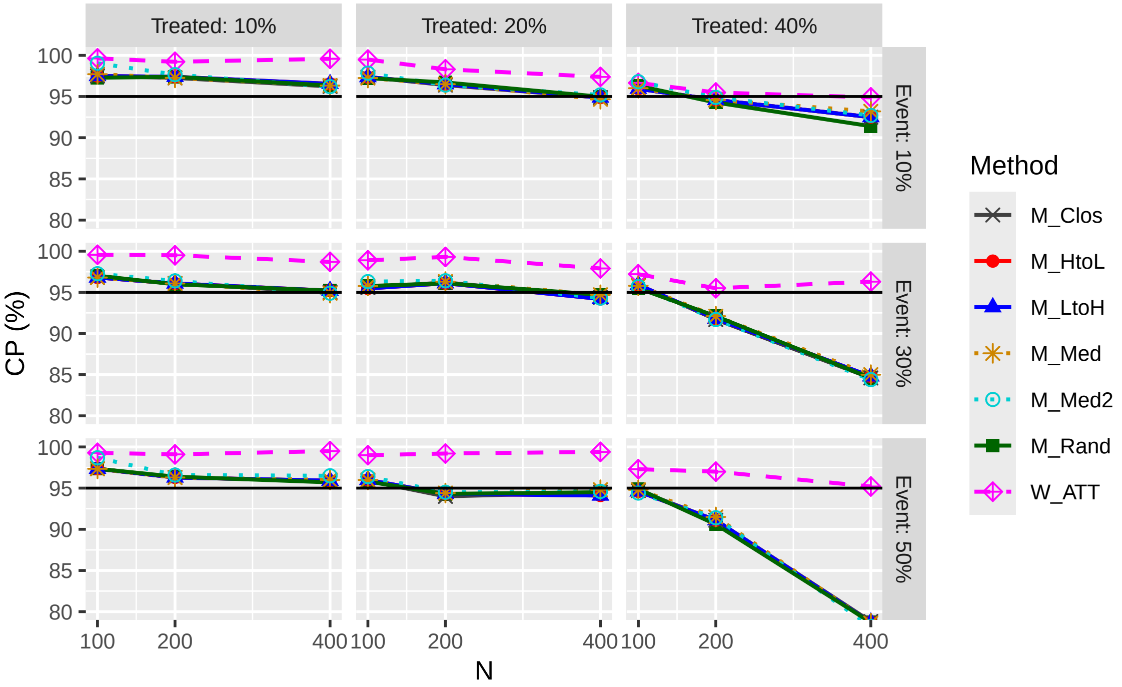

Figure S321. Coverage probability of confidence interval for OR (categorical covariate, matching ratio 1:2, true OR: 0.5, c statistic: 0.6, naive inference).

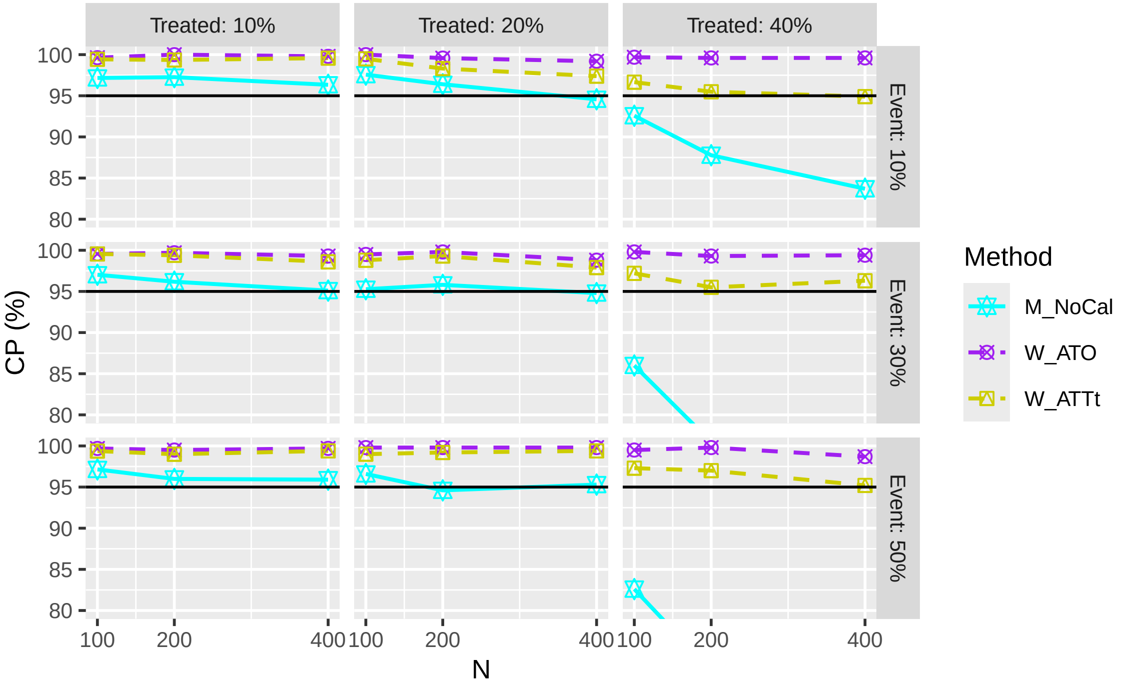

Figure S322. Coverage probability of confidence interval for OR (categorical covariate, matching ratio 1:2, true OR: 0.5, c statistic: 0.6, naive inference); other methods.

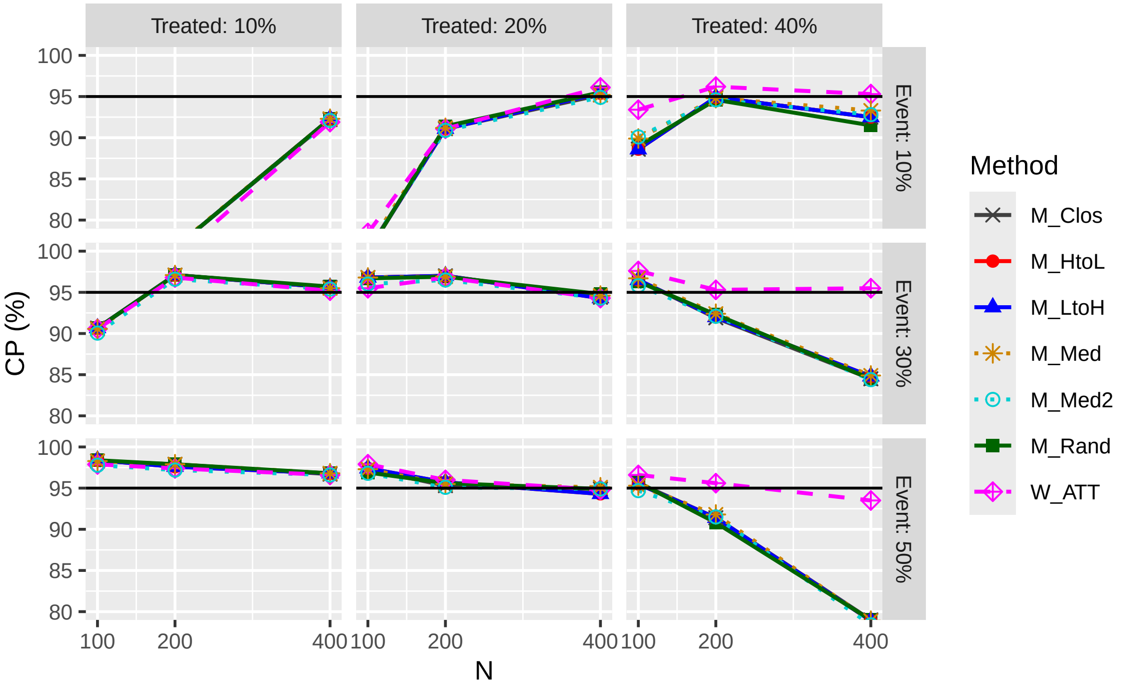

Figure S323. Coverage probability of confidence interval for OR (categorical covariate, matching ratio 1:2, true OR: 0.5, c statistic: 0.6, robust inference).

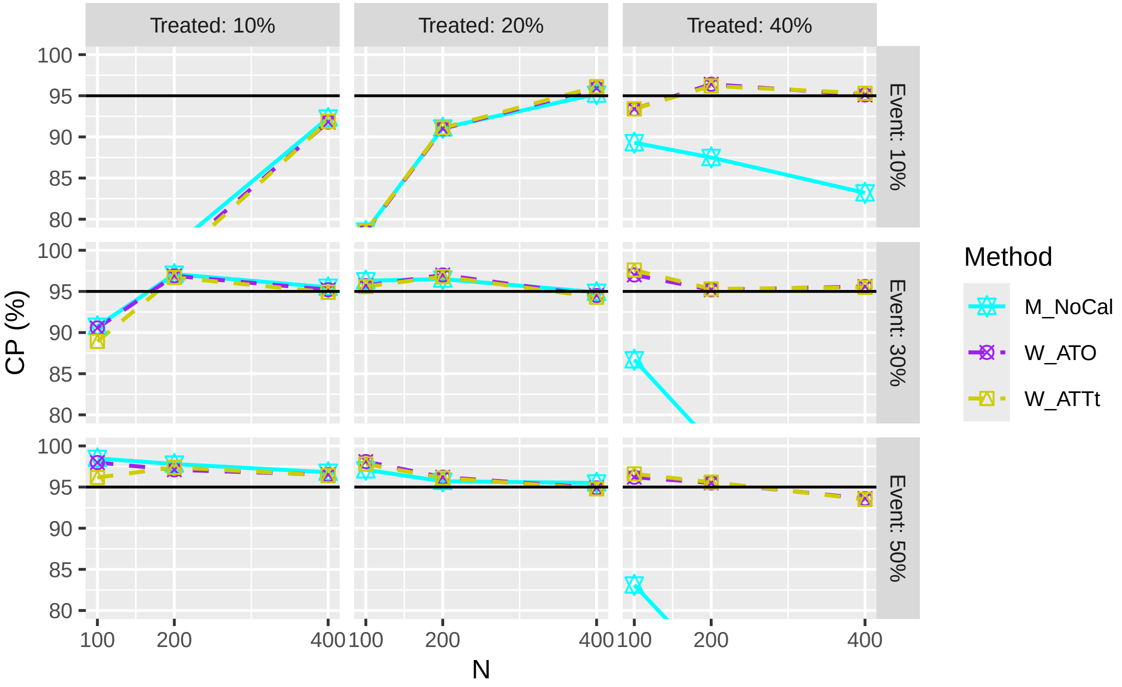

Figure S324. Coverage probability of confidence interval for OR (categorical covariate, matching ratio 1:2, true OR: 0.5, c statistic: 0.6, robust inference); other methods.

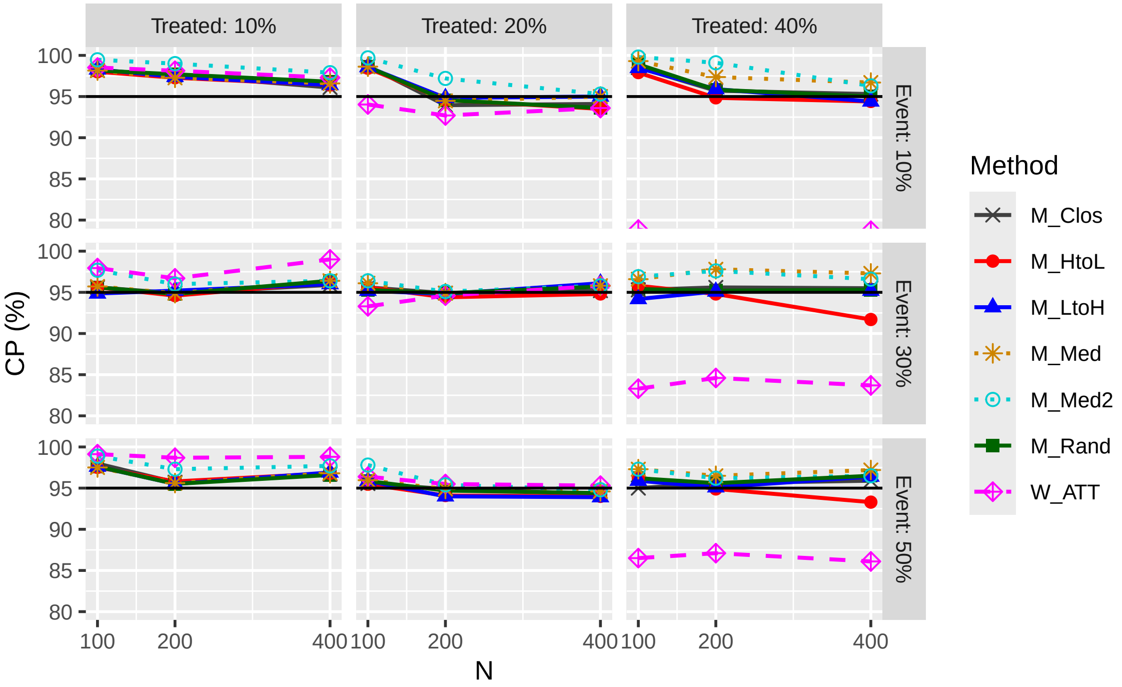

Figure S325. Coverage probability of confidence interval for OR (multimodal continuous covariate, matching ratio 1:1, true OR: 1, c statistic: 0.85, naive inference).

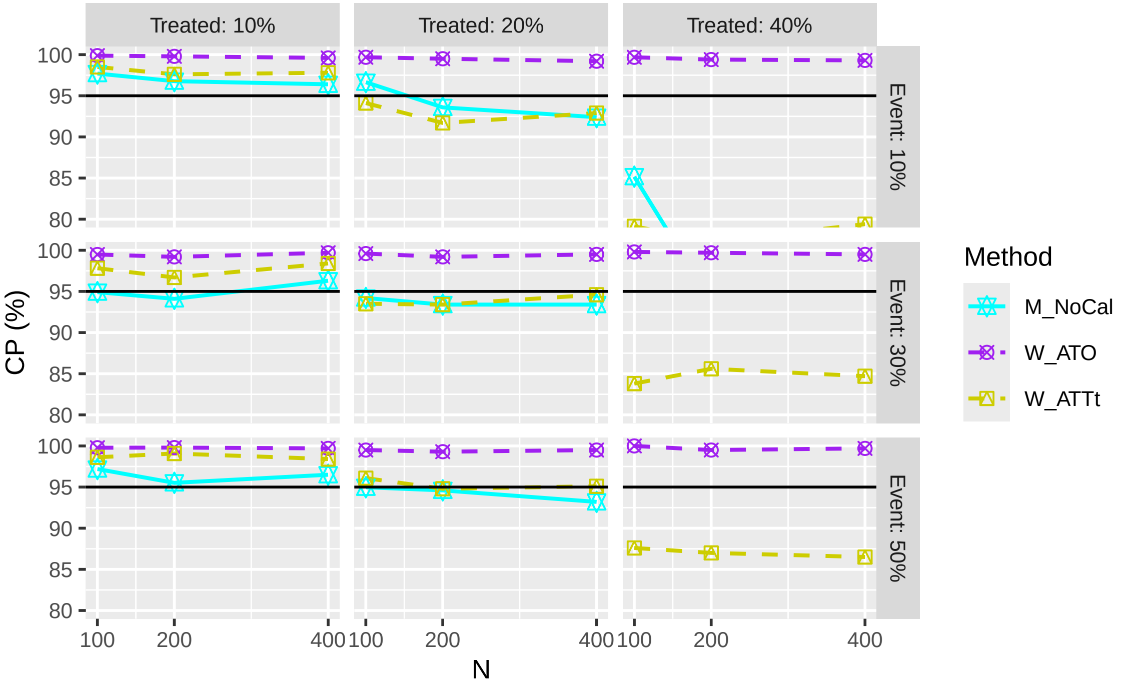

Figure S326. Coverage probability of confidence interval for OR (multimodal continuous covariate, matching ratio 1:1, true OR: 1, c statistic: 0.85, naive inference); other methods.

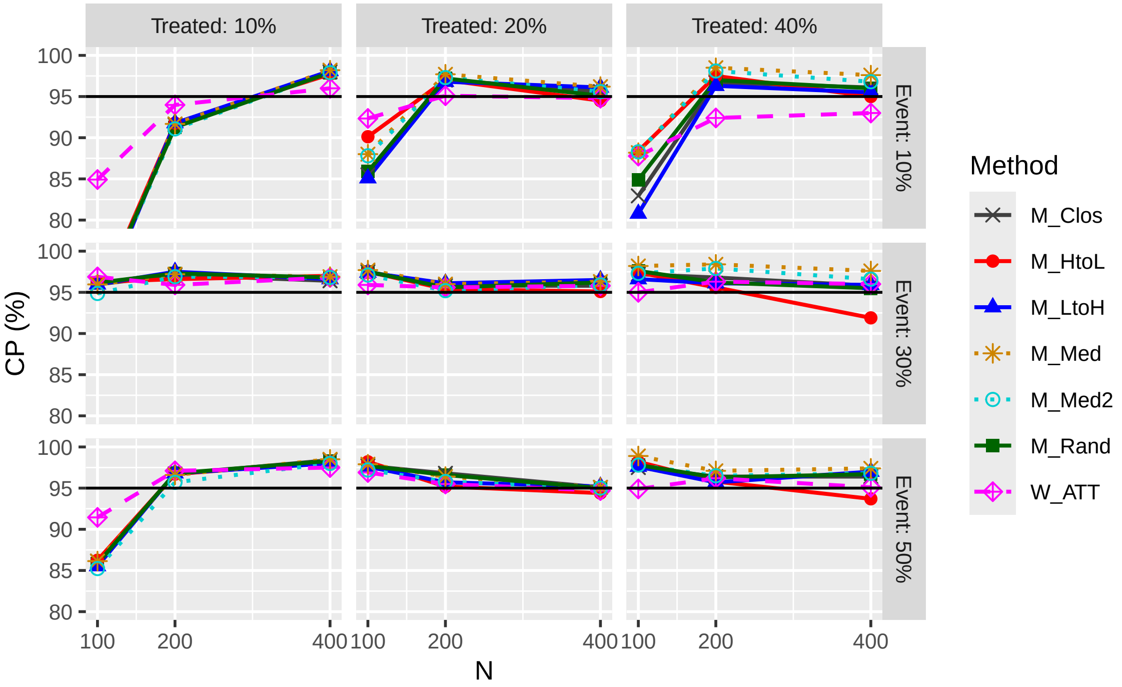

Figure S327. Coverage probability of confidence interval for OR (multimodal continuous covariate, matching ratio 1:1, true OR: 1, c statistic: 0.85, robust inference).

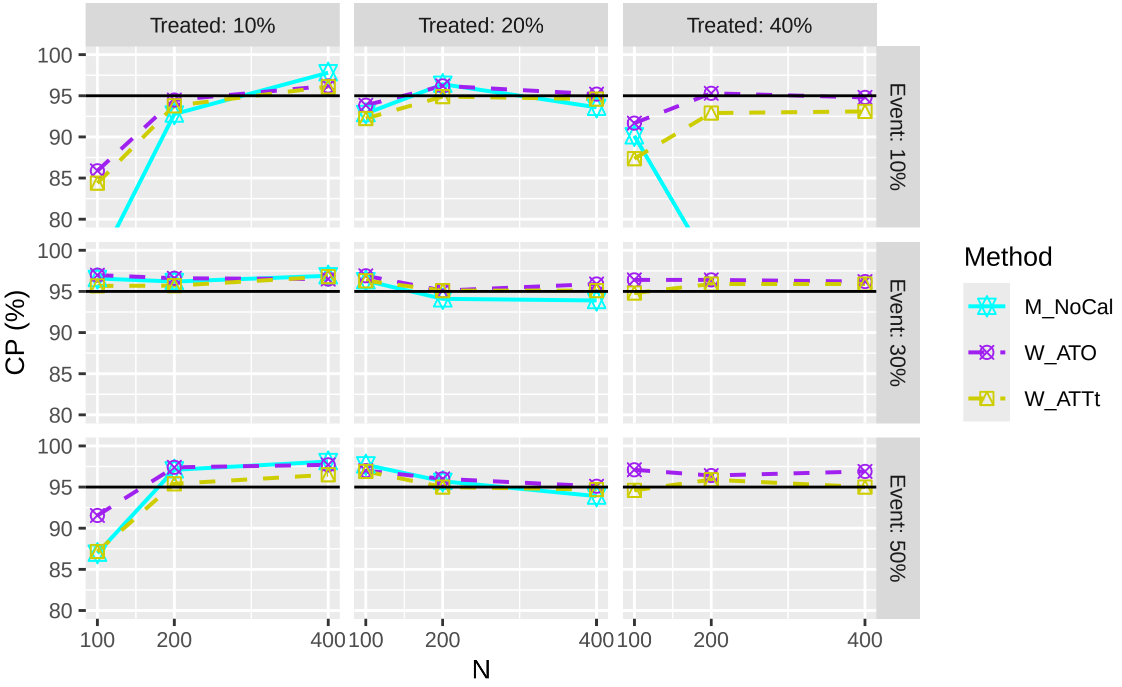

Figure S328. Coverage probability of confidence interval for OR (multimodal continuous covariate, matching ratio 1:1, true OR: 1, c statistic: 0.85, robust inference); other methods.

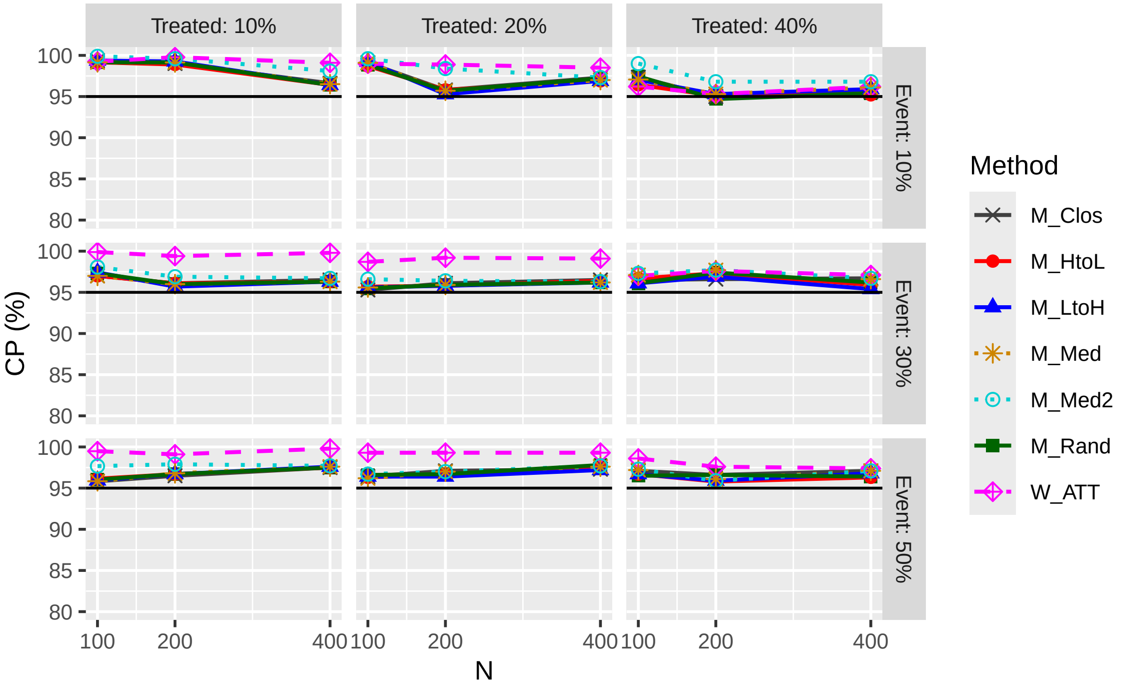

Figure S329. Coverage probability of confidence interval for OR (multimodal continuous covariate, matching ratio 1:1, true OR: 1, c statistic: 0.6, naive inference).

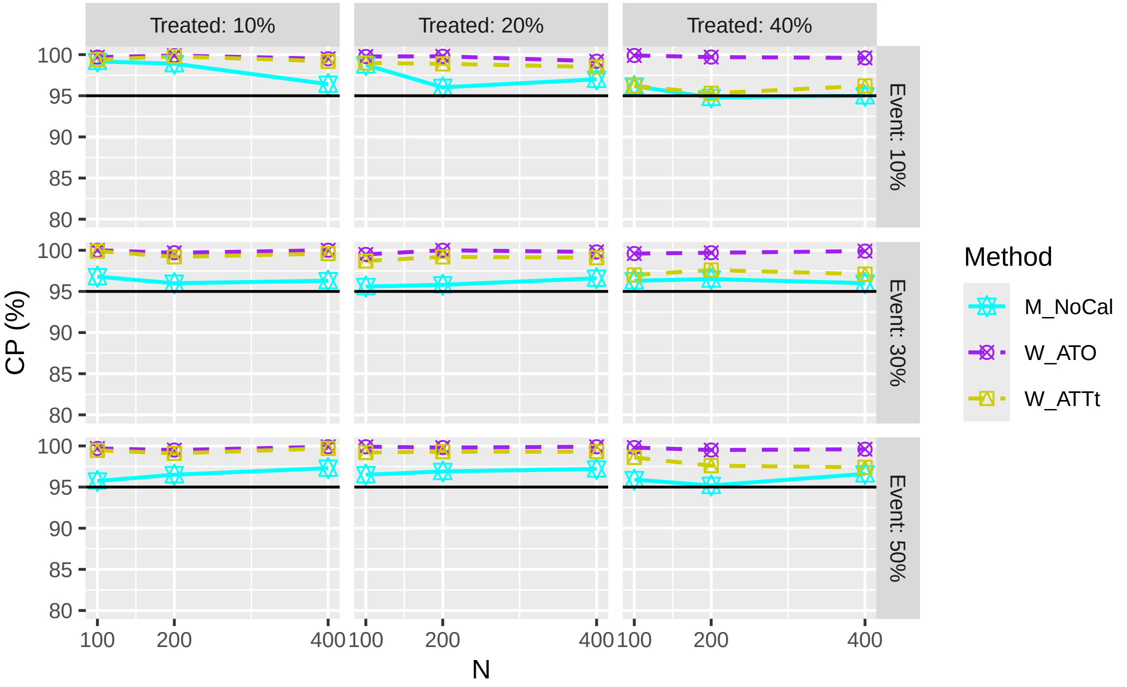

Figure S330. Coverage probability of confidence interval for OR (multimodal continuous covariate, matching ratio 1:1, true OR: 1, c statistic: 0.6, naive inference); other methods.

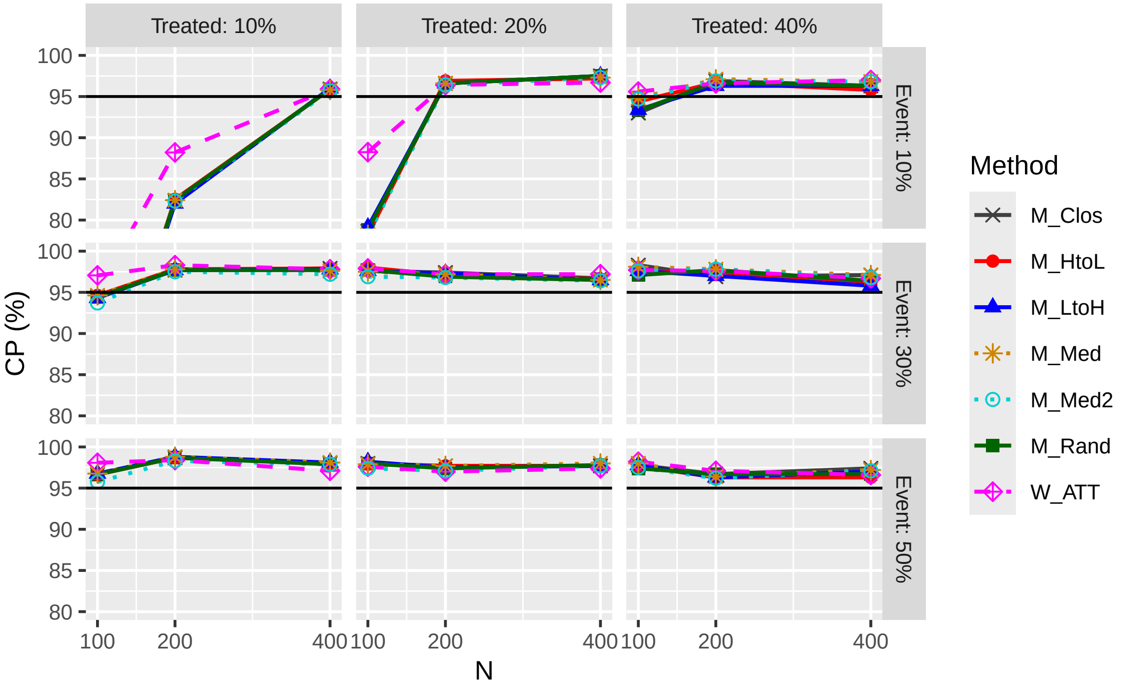

Figure S331. Coverage probability of confidence interval for OR (multimodal continuous covariate, matching ratio 1:1, true OR: 1, c statistic: 0.6, robust inference).

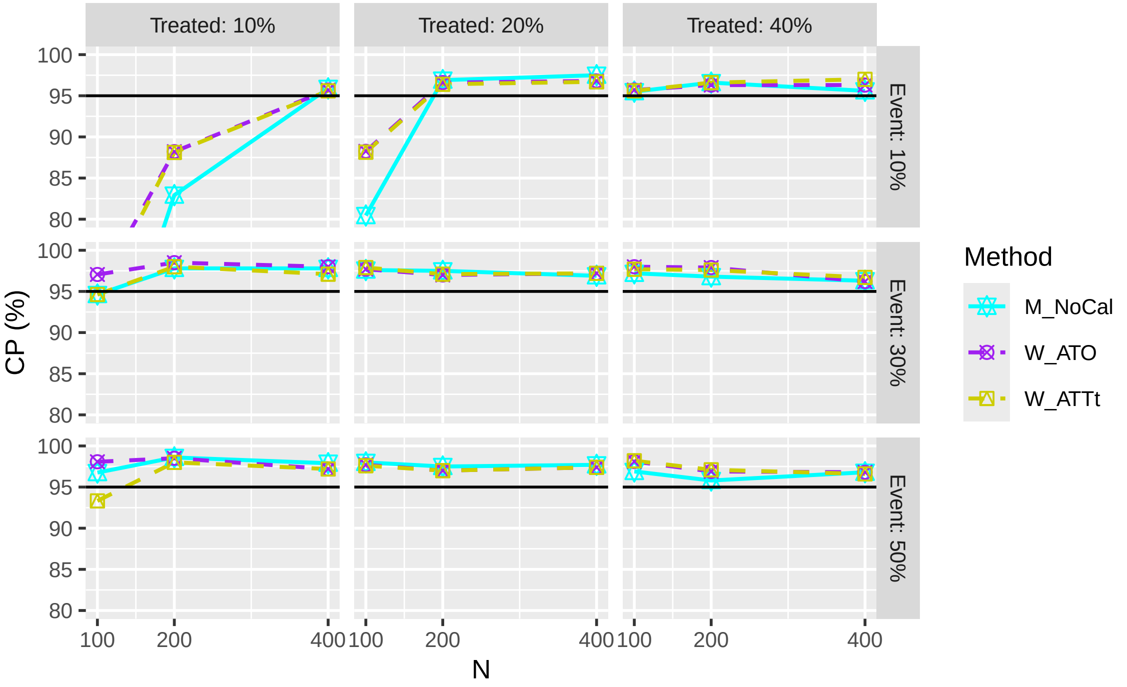

Figure S332. Coverage probability of confidence interval for OR (multimodal continuous covariate, matching ratio 1:1, true OR: 1, c statistic: 0.6, robust inference); other methods.

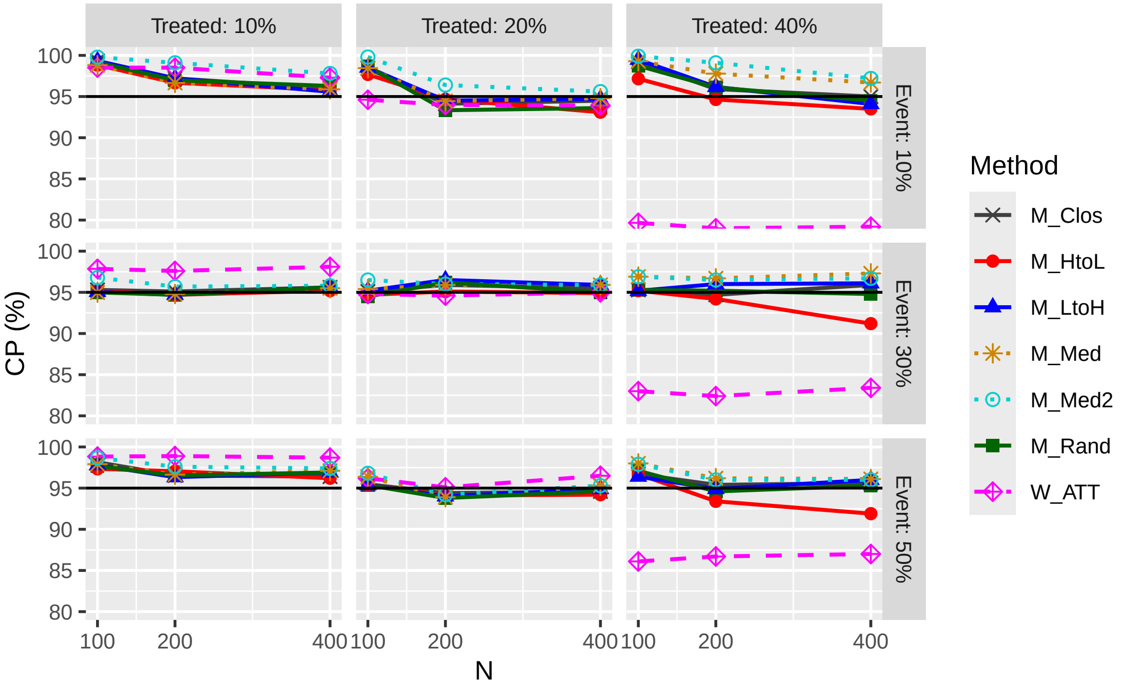

Figure S333. Coverage probability of confidence interval for OR (multimodal continuous covariate, matching ratio 1:1, true OR: 0.75, c statistic: 0.85, naive inference).

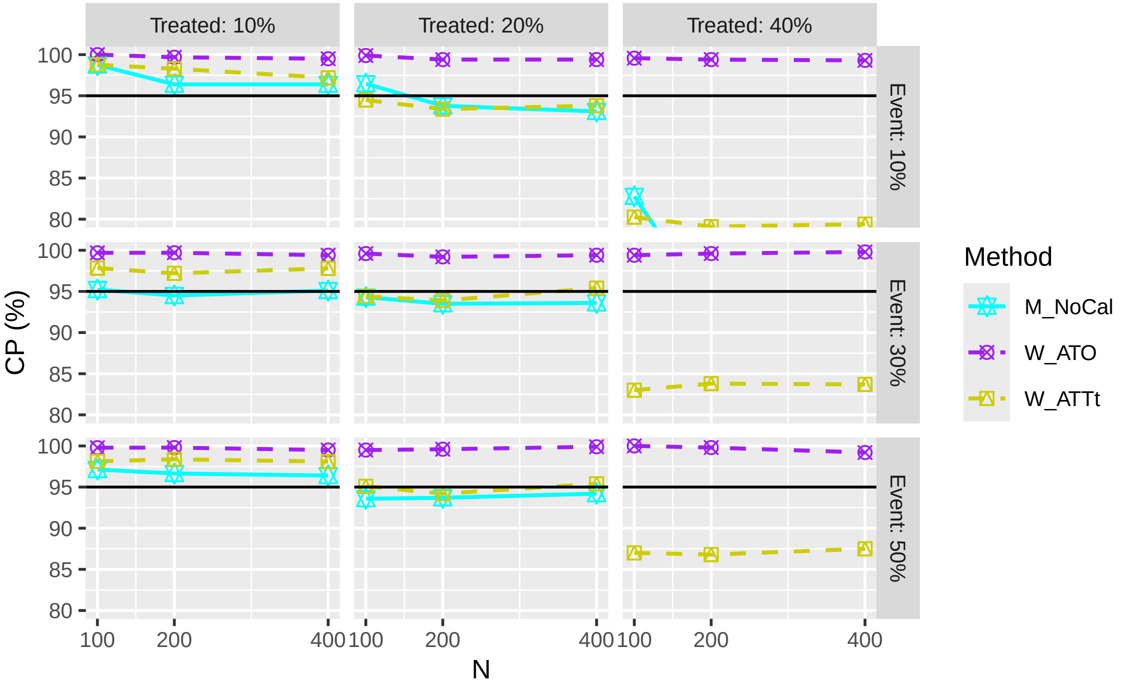

Figure S334. Coverage probability of confidence interval for OR (multimodal continuous covariate, matching ratio 1:1, true OR: 0.75, c statistic: 0.85, naive inference); other methods.

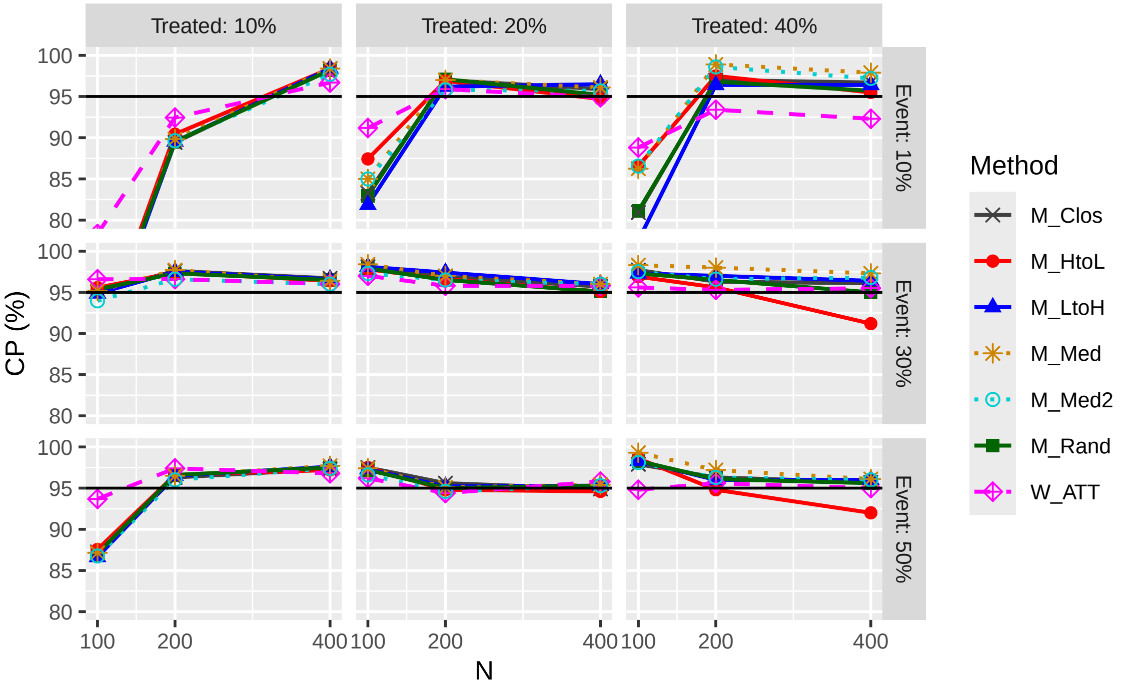

Figure S335. Coverage probability of confidence interval for OR (multimodal continuous covariate, matching ratio 1:1, true OR: 0.75, c statistic: 0.85, robust inference).

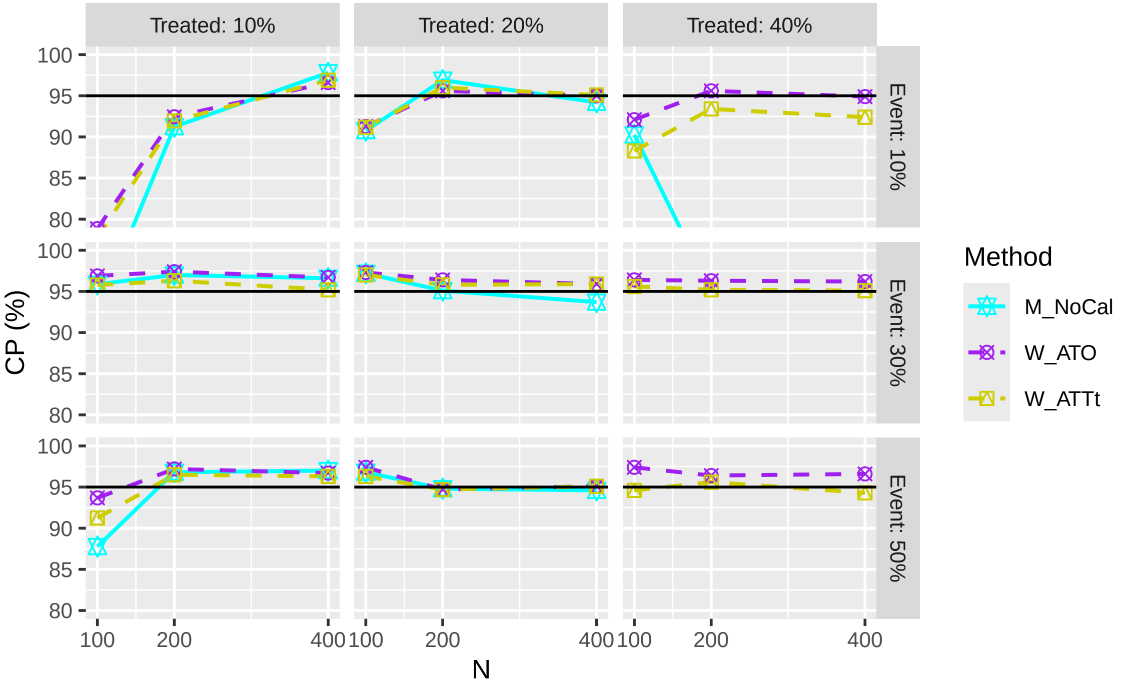

Figure S336. Coverage probability of confidence interval for OR (multimodal continuous covariate, matching ratio 1:1, true OR: 0.75, c statistic: 0.85, robust inference); other methods.

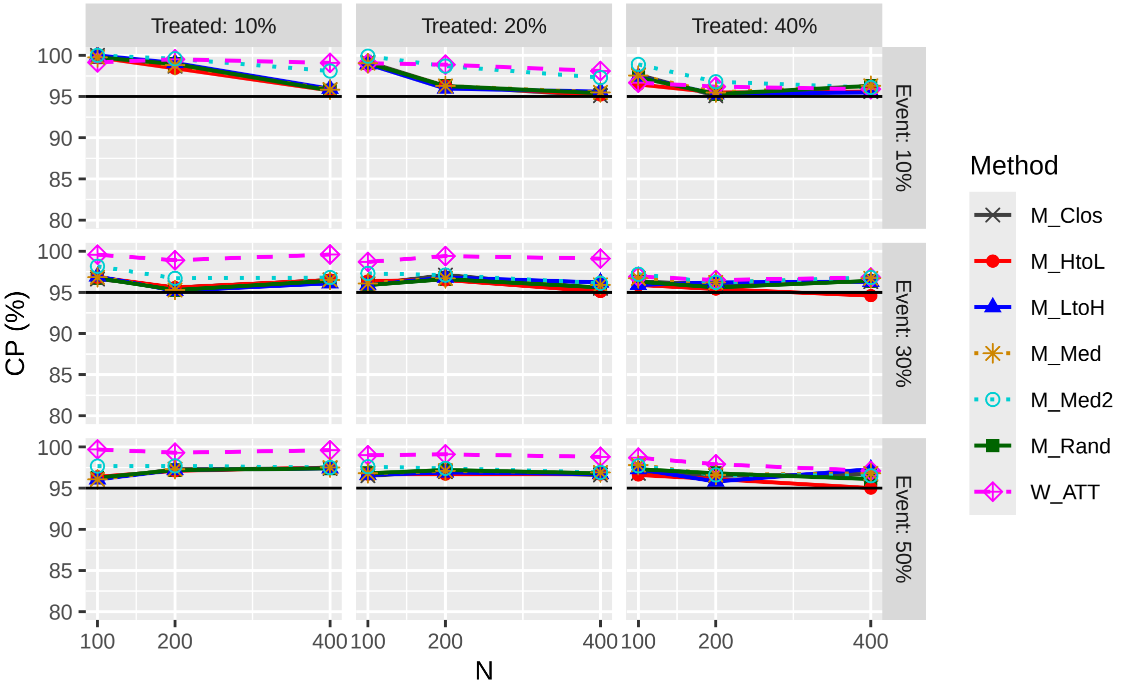

Figure S337. Coverage probability of confidence interval for OR (multimodal continuous covariate, matching ratio 1:1, true OR: 0.75, c statistic: 0.6, naive inference).

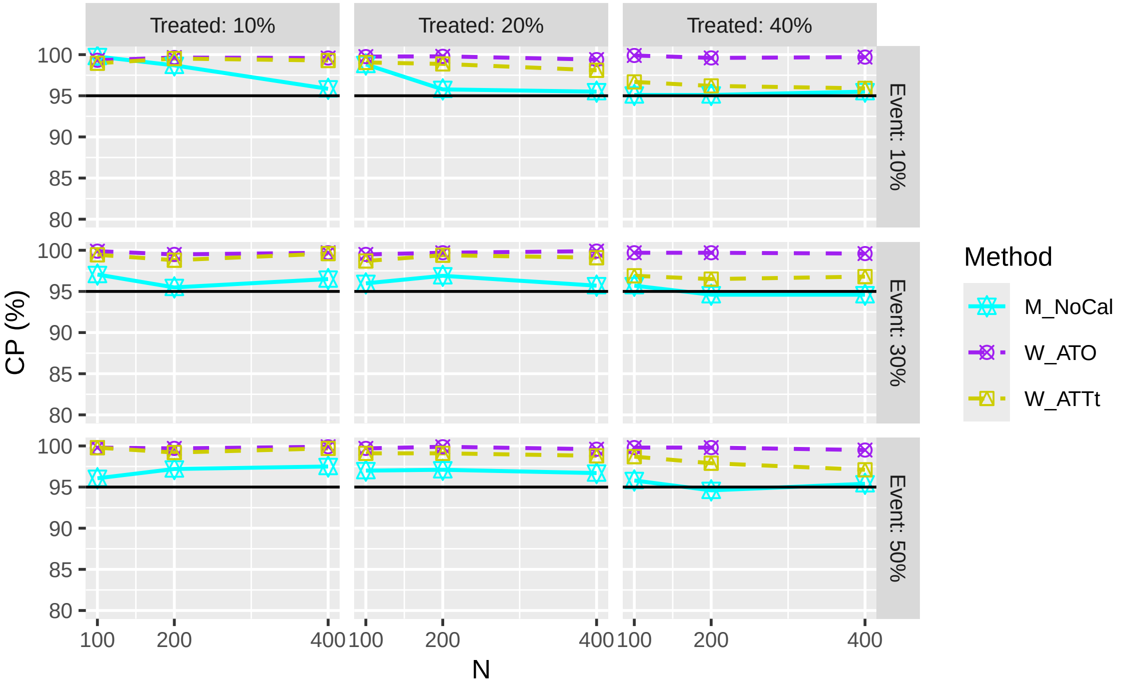

Figure S338. Coverage probability of confidence interval for OR (multimodal continuous covariate, matching ratio 1:1, true OR: 0.75, c statistic: 0.6, naive inference); other methods.

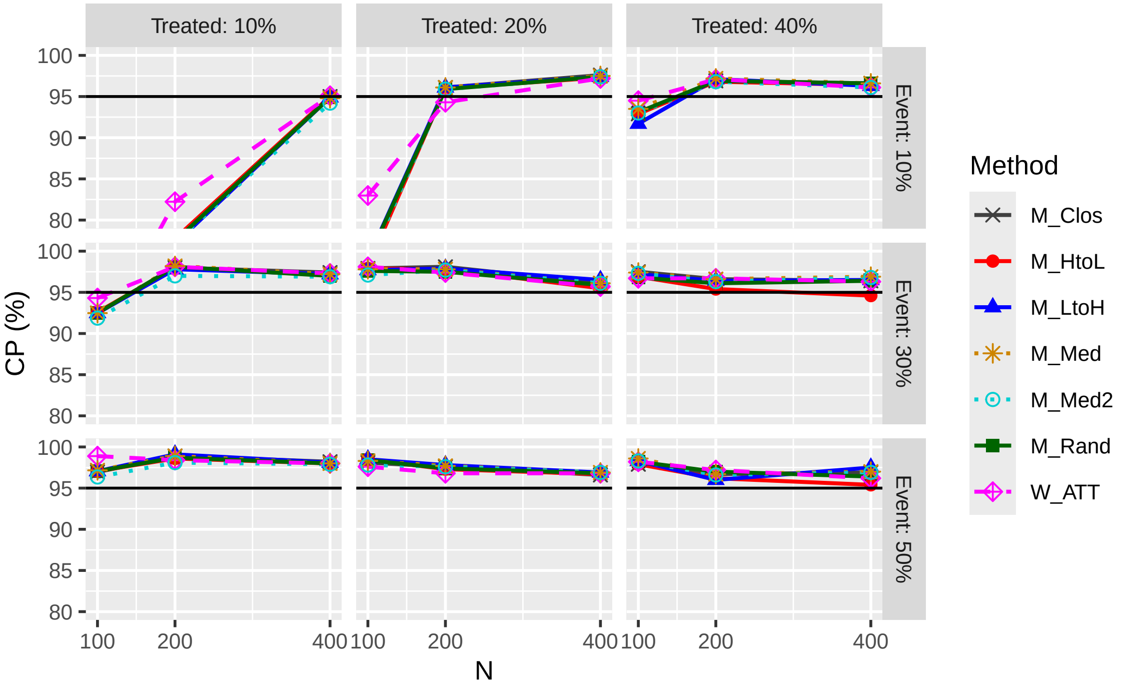

Figure S339. Coverage probability of confidence interval for OR (multimodal continuous covariate, matching ratio 1:1, true OR: 0.75, c statistic: 0.6, robust inference).

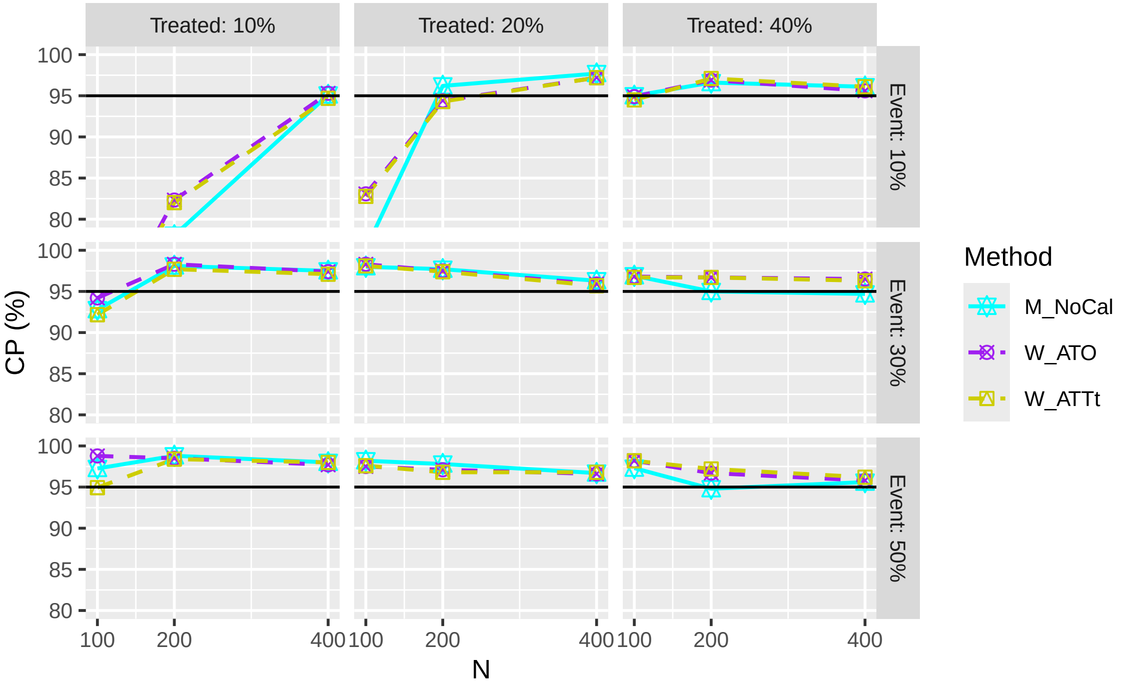

Figure S340. Coverage probability of confidence interval for OR (multimodal continuous covariate, matching ratio 1:1, true OR: 0.75, c statistic: 0.6, robust inference); other methods.

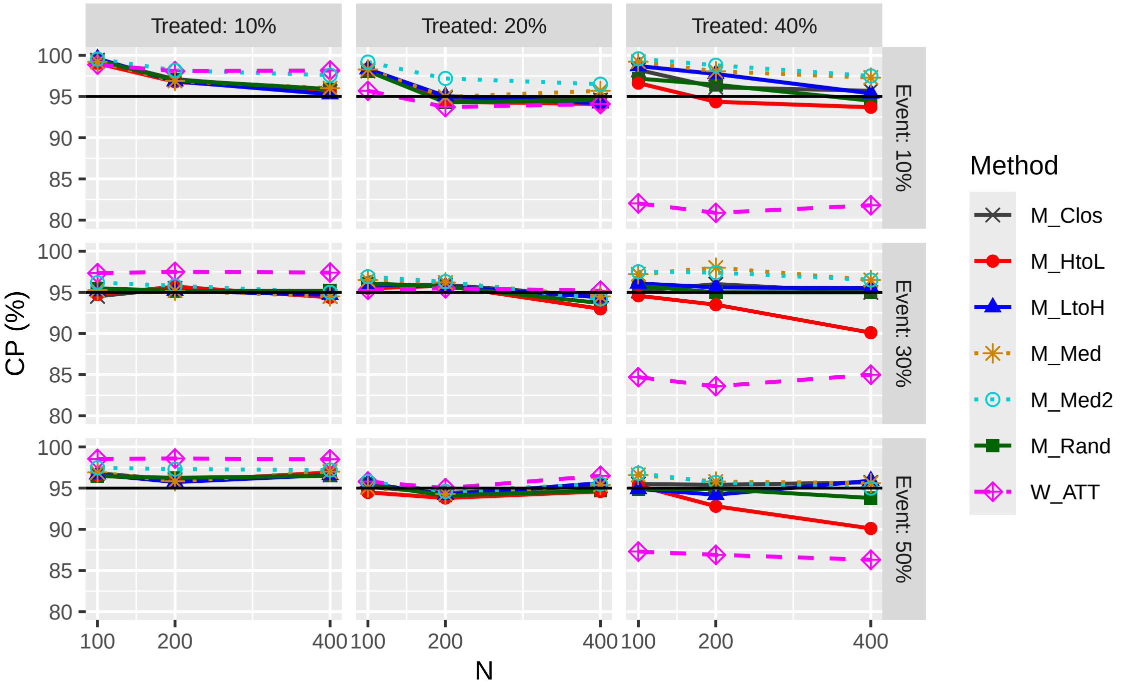

Figure S341. Coverage probability of confidence interval for OR (multimodal continuous covariate, matching ratio 1:1, true OR: 0.5, c statistic: 0.85, naive inference).

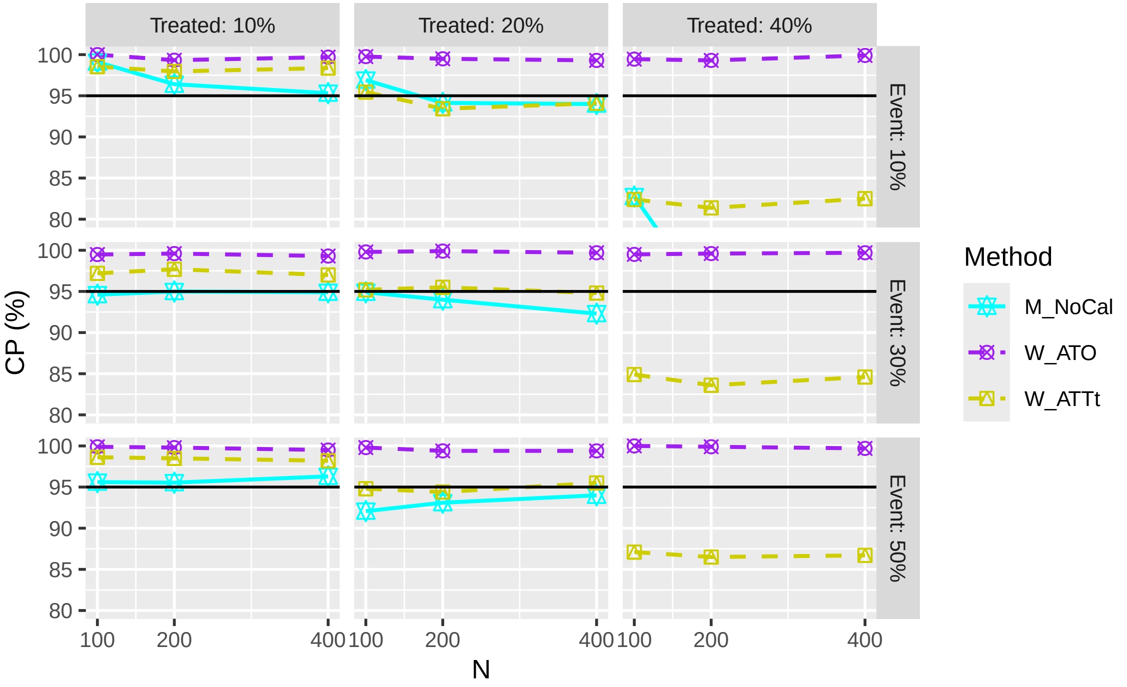

Figure S342. Coverage probability of confidence interval for OR (multimodal continuous covariate, matching ratio 1:1, true OR: 0.5, c statistic: 0.85, naive inference); other methods.

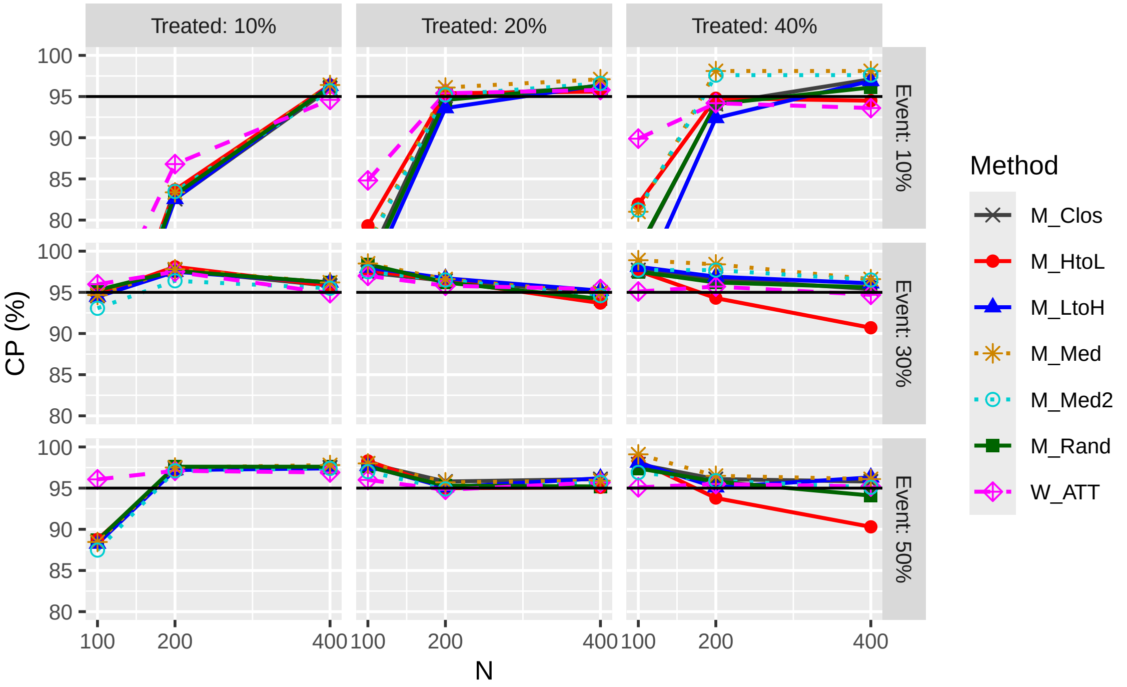

Figure S343. Coverage probability of confidence interval for OR (multimodal continuous covariate, matching ratio 1:1, true OR: 0.5, c statistic: 0.85, robust inference).

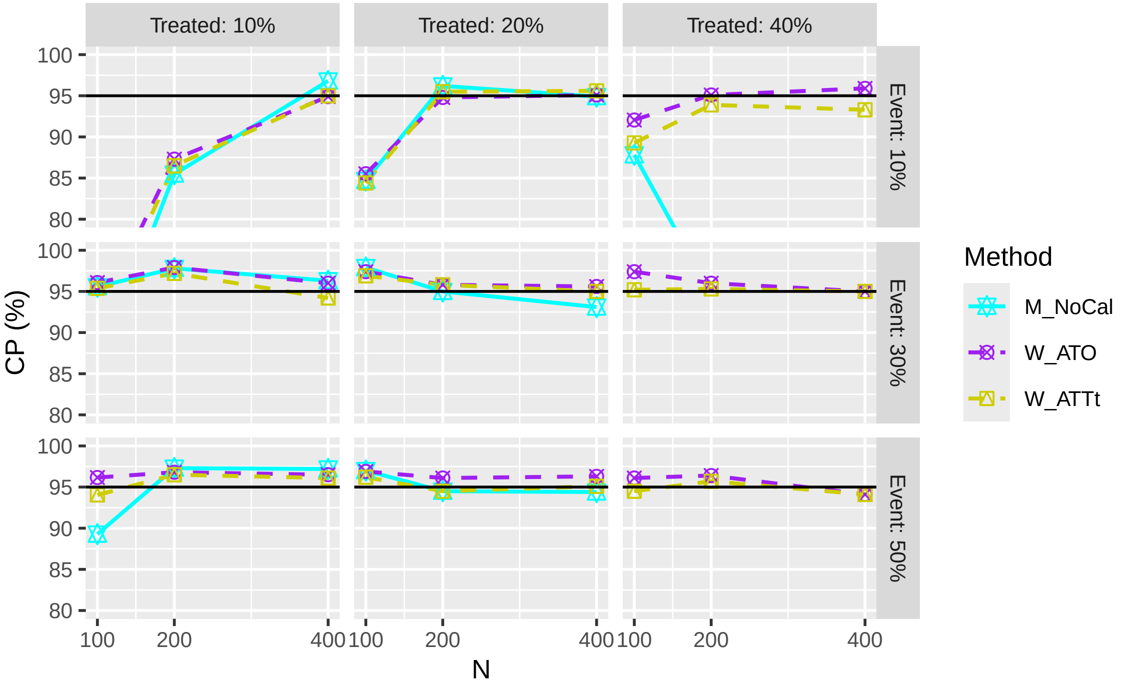

Figure S344. Coverage probability of confidence interval for OR (multimodal continuous covariate, matching ratio 1:1, true OR: 0.5, c statistic: 0.85, robust inference); other methods.

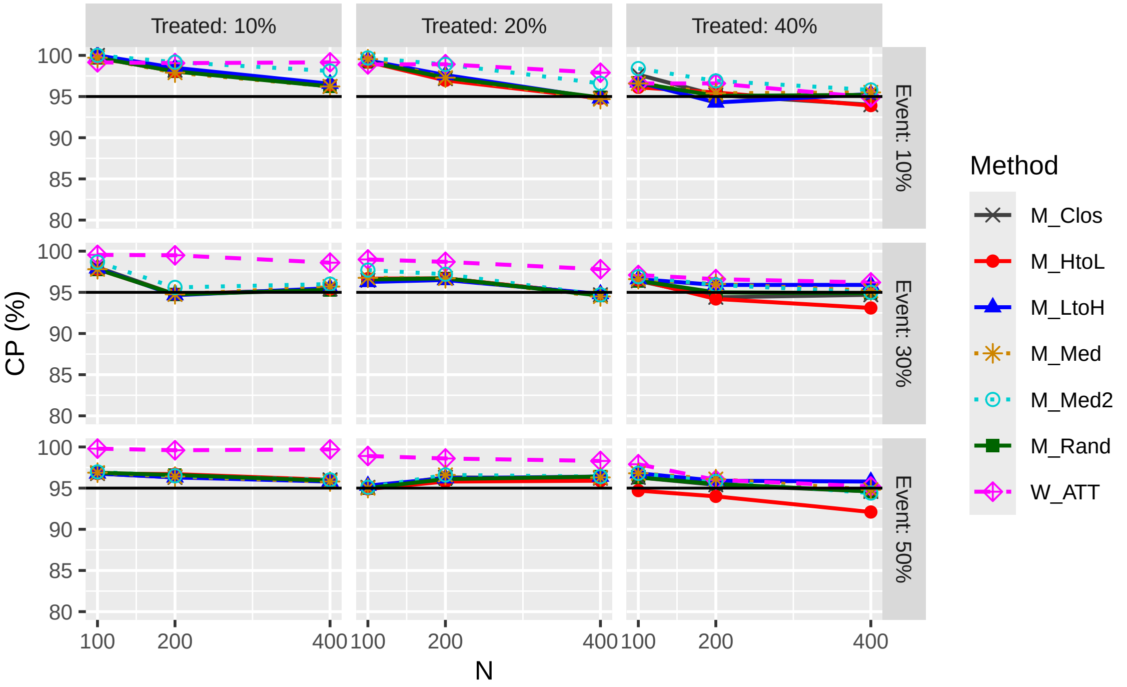

Figure S345. Coverage probability of confidence interval for OR (multimodal continuous covariate, matching ratio 1:1, true OR: 0.5, c statistic: 0.6, naive inference).

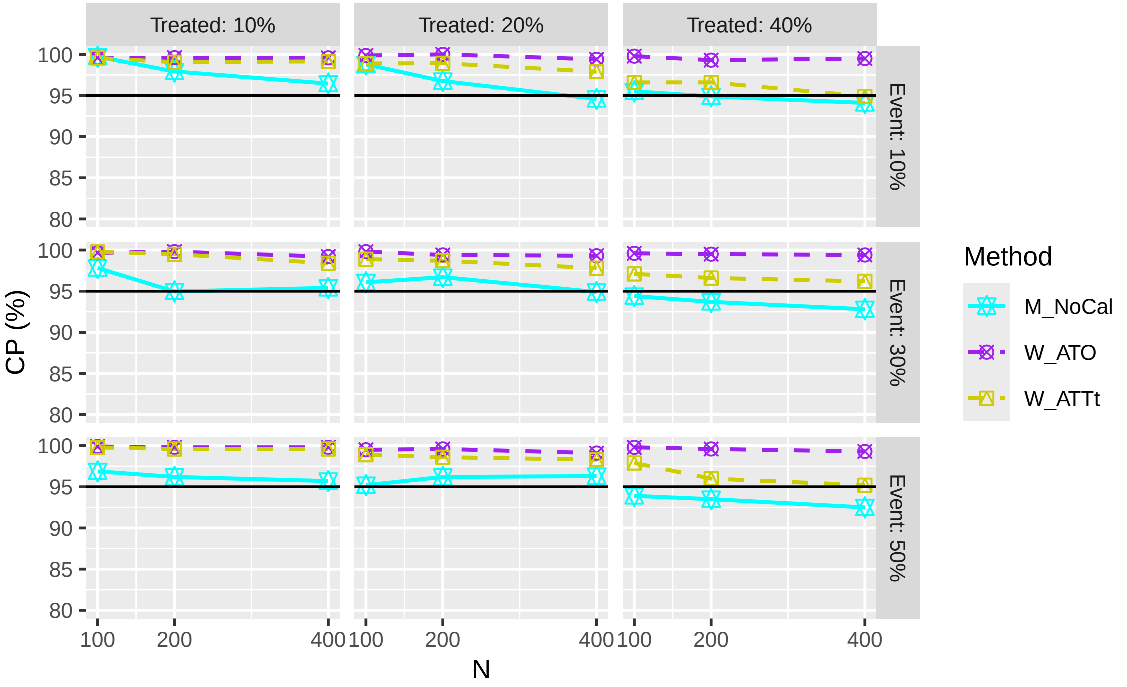

Figure S346. Coverage probability of confidence interval for OR (multimodal continuous covariate, matching ratio 1:1, true OR: 0.5, c statistic: 0.6, naive inference); other methods.

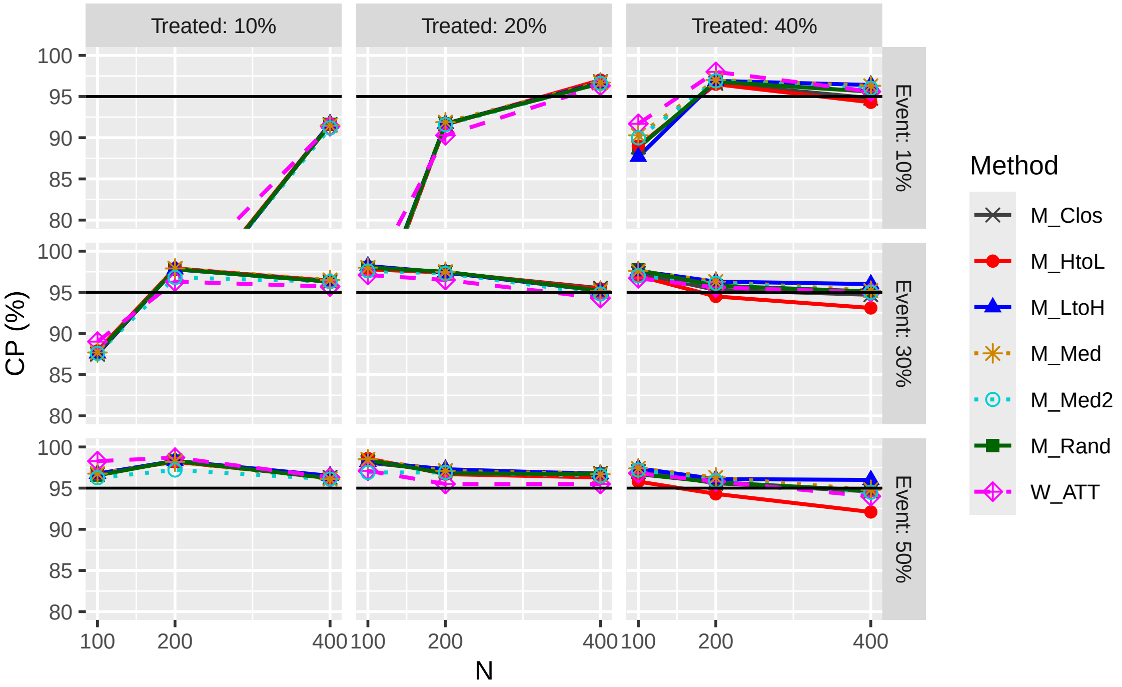

Figure S347. Coverage probability of confidence interval for OR (multimodal continuous covariate, matching ratio 1:1, true OR: 0.5, c statistic: 0.6, robust inference).

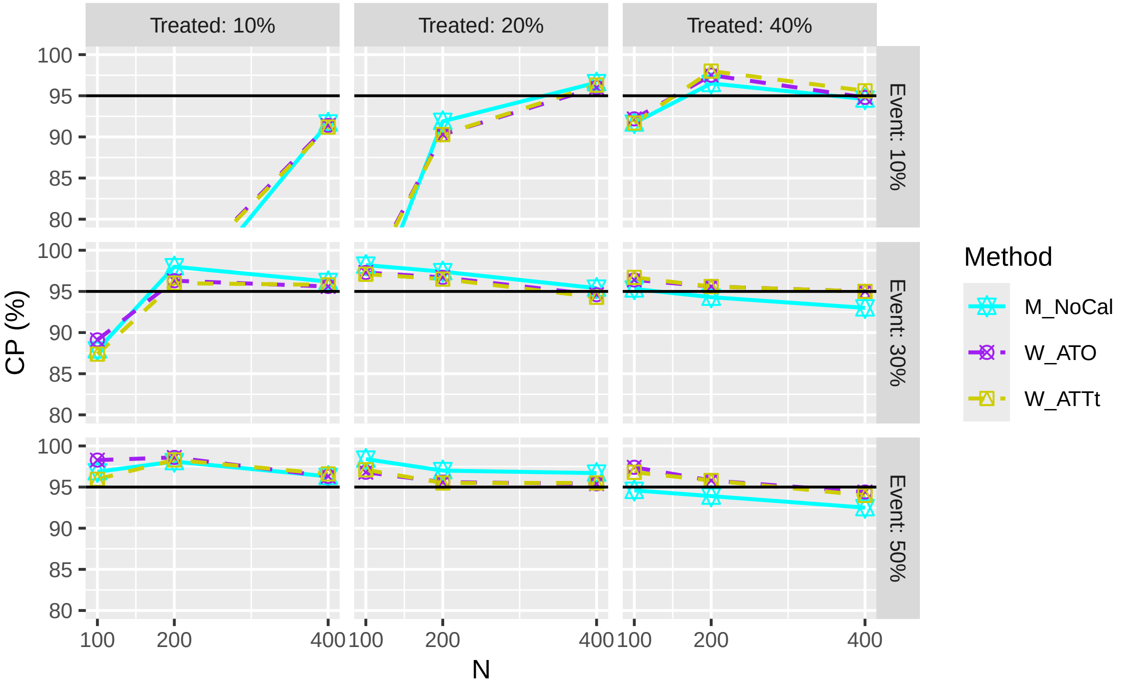

Figure S348. Coverage probability of confidence interval for OR (multimodal continuous covariate, matching ratio 1:1, true OR: 0.5, c statistic: 0.6, robust inference); other methods.

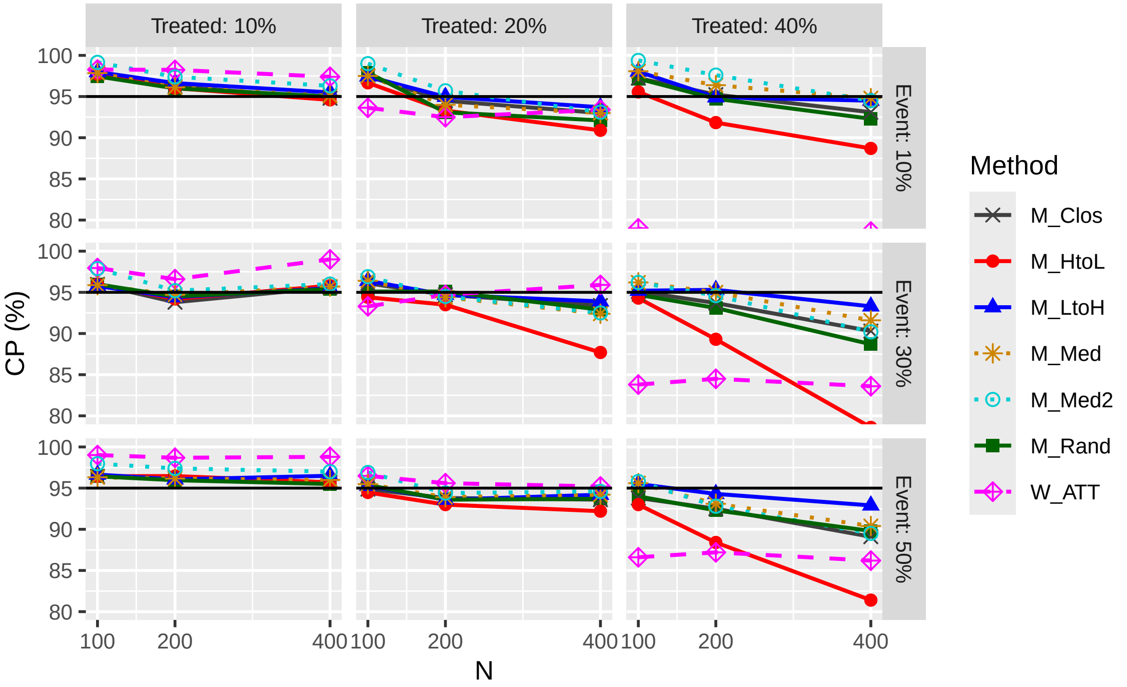

Figure S349. Coverage probability of confidence interval for OR (multimodal continuous covariate, matching ratio 1:2, true OR: 1, c statistic: 0.85, naive inference).

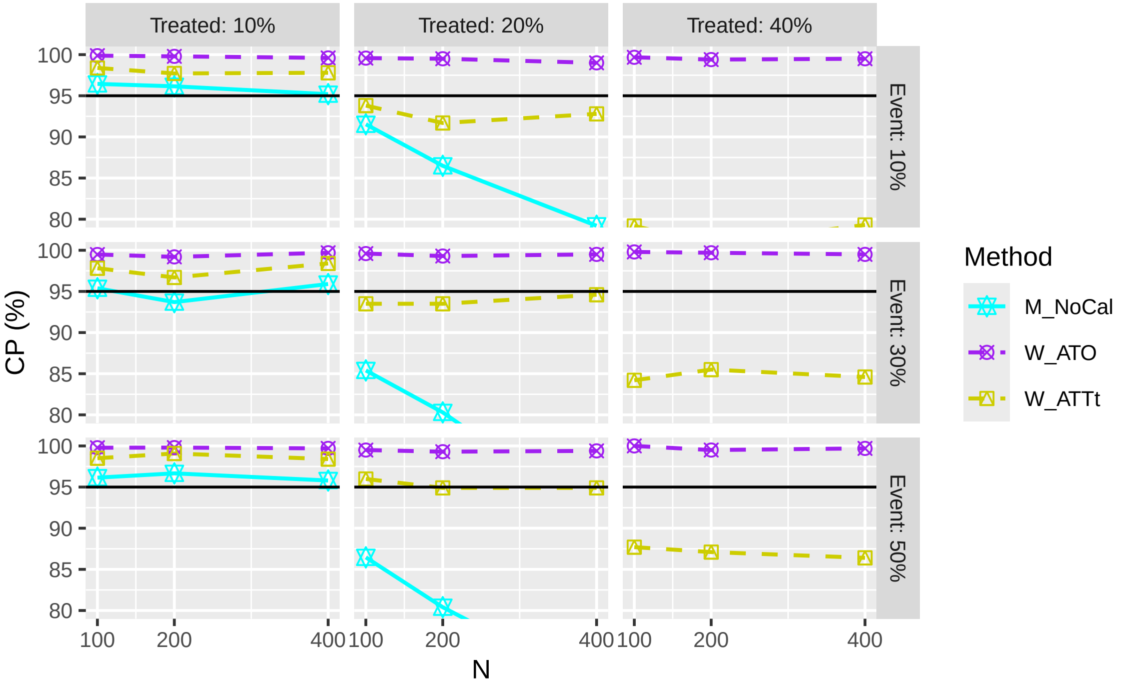

Figure S350. Coverage probability of confidence interval for OR (multimodal continuous covariate, matching ratio 1:2, true OR: 1, c statistic: 0.85, naive inference); other methods.

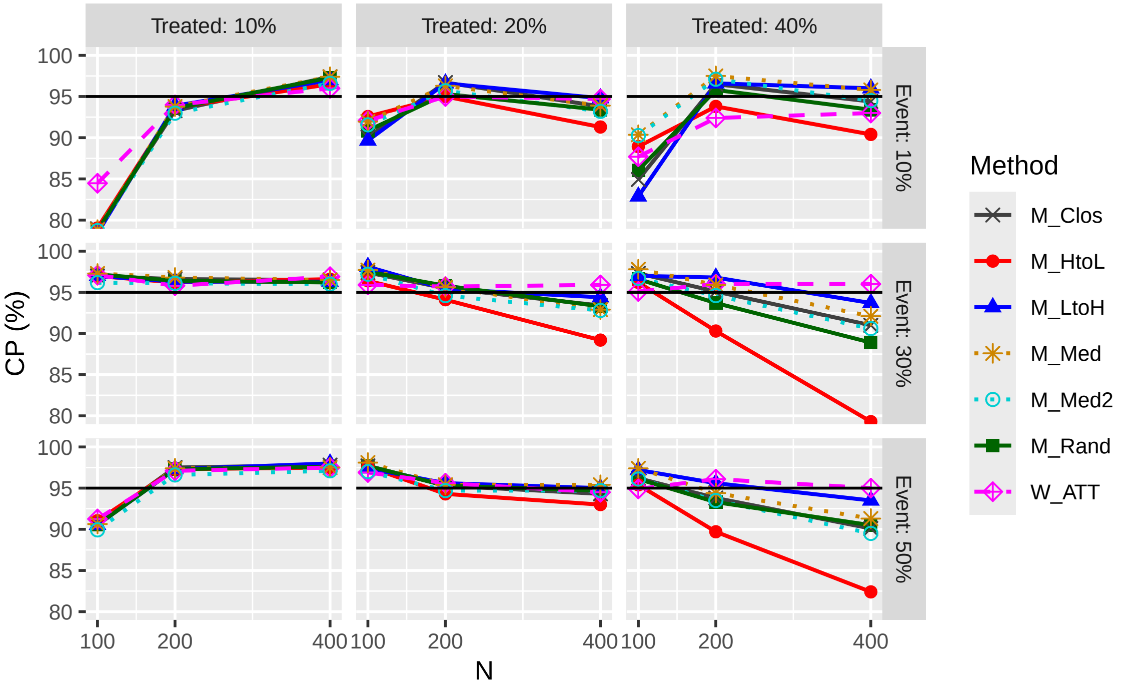

Figure S351. Coverage probability of confidence interval for OR (multimodal continuous covariate, matching ratio 1:2, true OR: 1, c statistic: 0.85, robust inference).

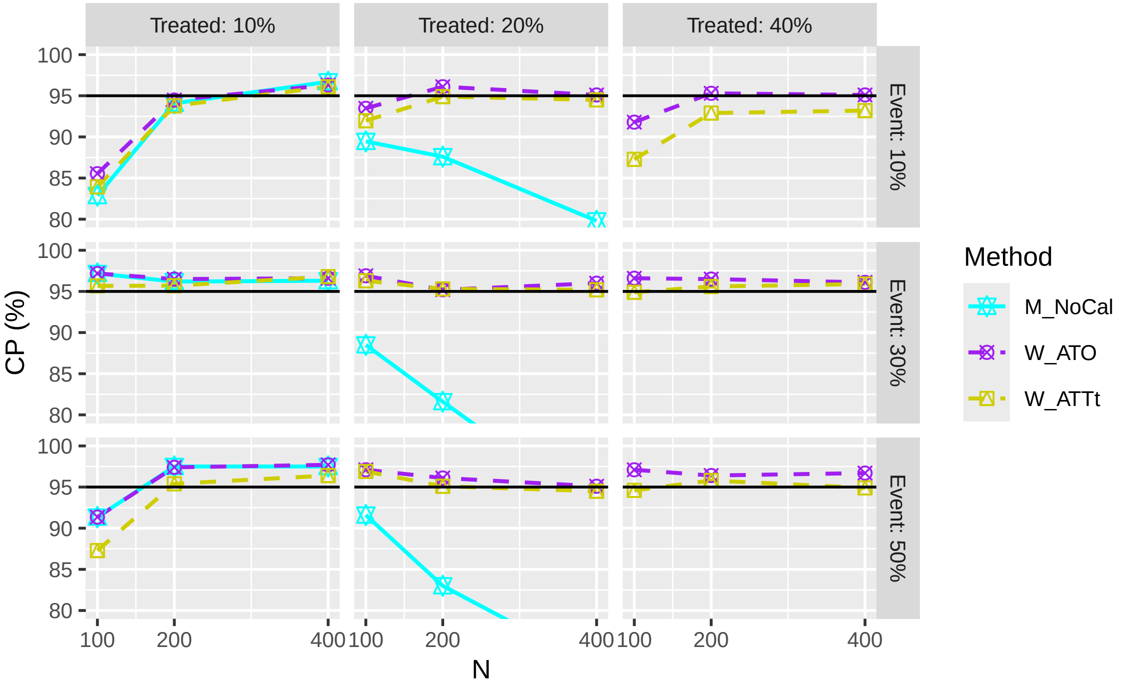

Figure S352. Coverage probability of confidence interval for OR (multimodal continuous covariate, matching ratio 1:2, true OR: 1, c statistic: 0.85, robust inference); other methods.

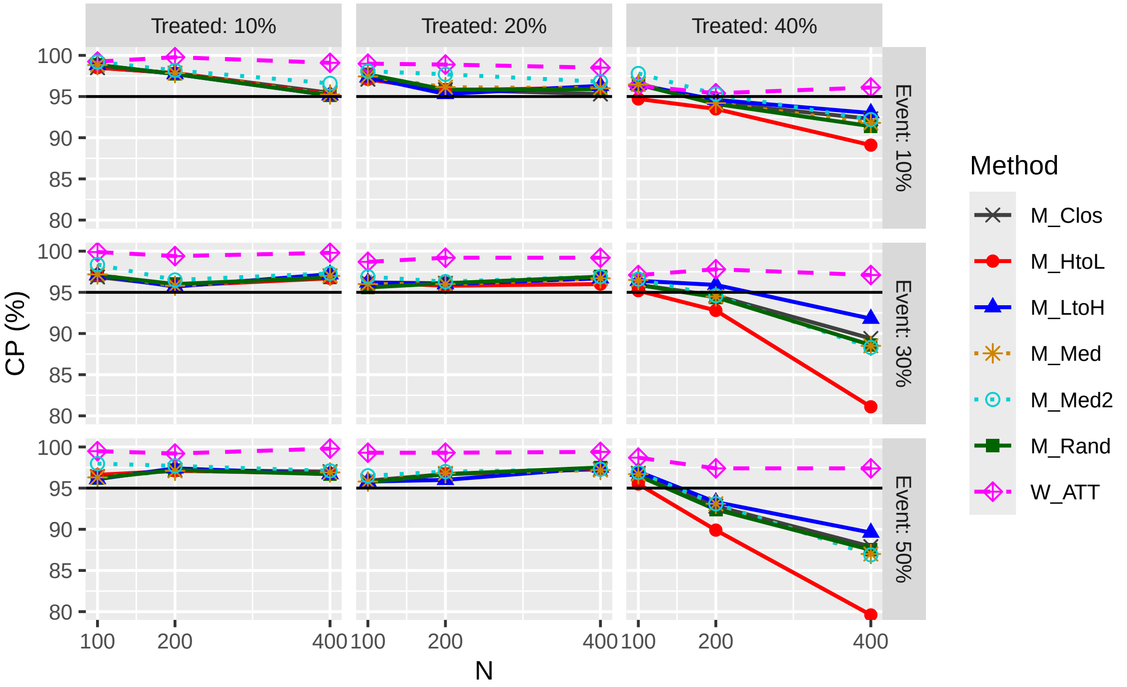

Figure S353. Coverage probability of confidence interval for OR (multimodal continuous covariate, matching ratio 1:2, true OR: 1, c statistic: 0.6, naive inference).

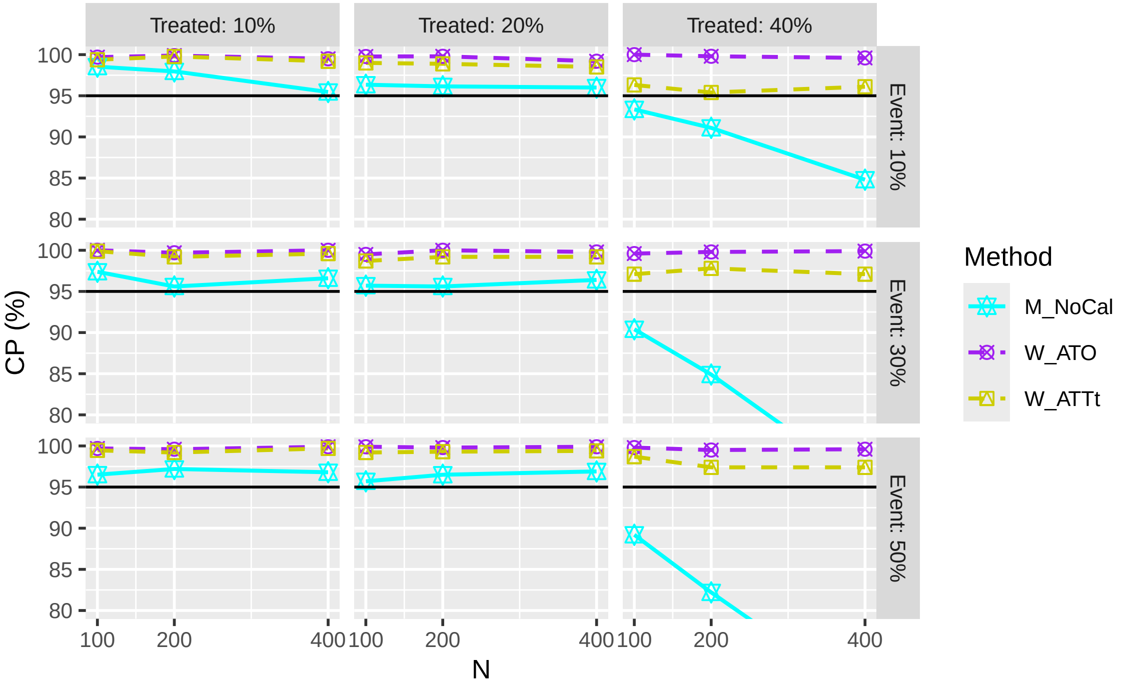

Figure S354. Coverage probability of confidence interval for OR (multimodal continuous covariate, matching ratio 1:2, true OR: 1, c statistic: 0.6, naive inference); other methods.

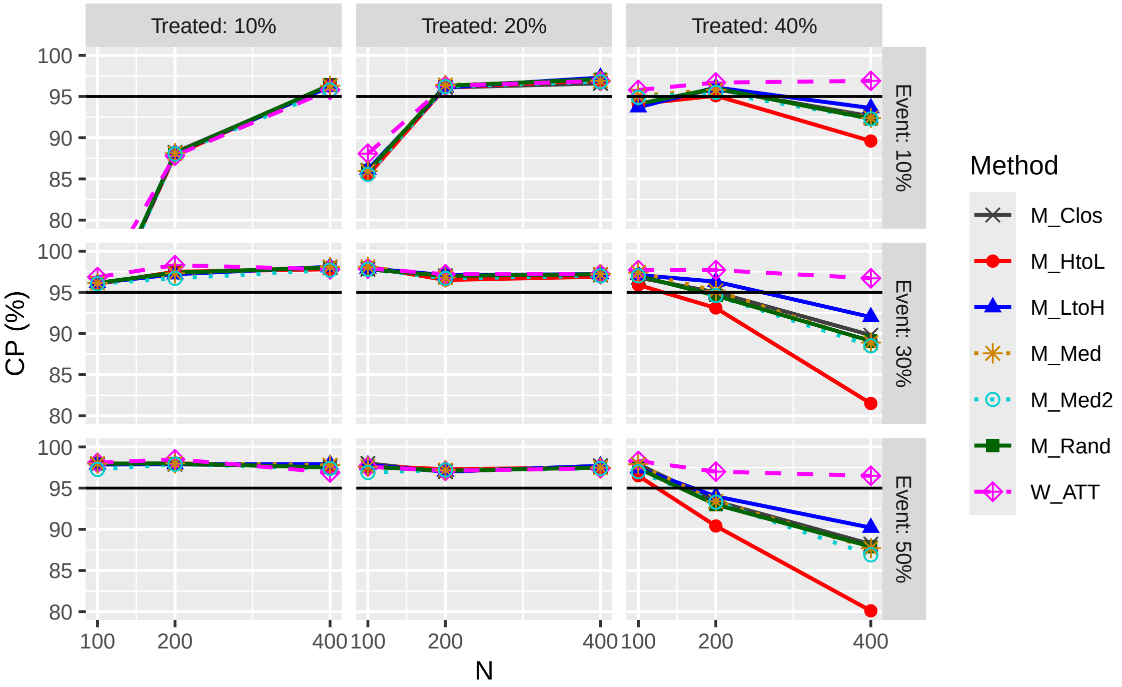

Figure S355. Coverage probability of confidence interval for OR (multimodal continuous covariate, matching ratio 1:2, true OR: 1, c statistic: 0.6, robust inference).

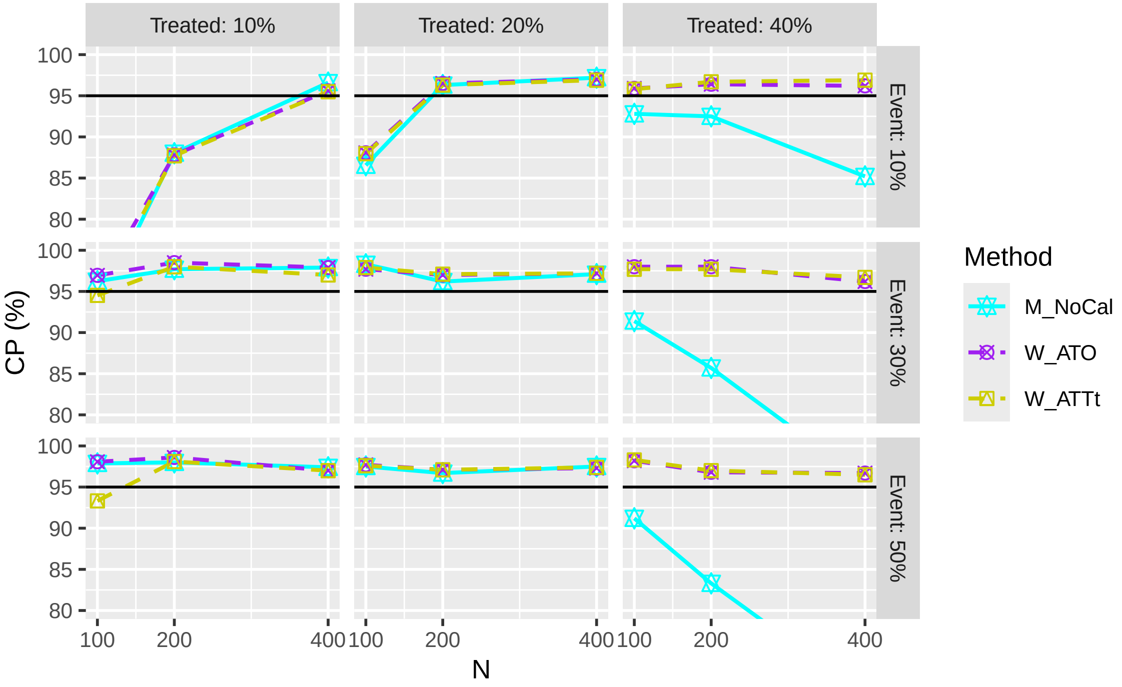

Figure S356. Coverage probability of confidence interval for OR (multimodal continuous covariate, matching ratio 1:2, true OR: 1, c statistic: 0.6, robust inference); other methods.

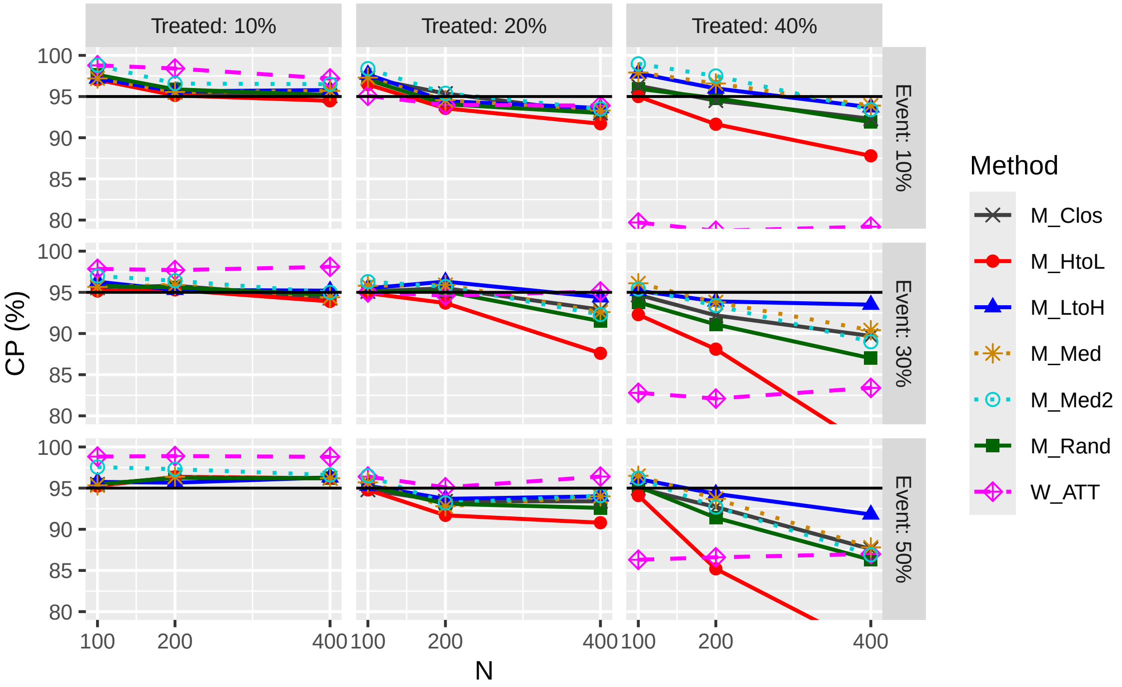

Figure S357. Coverage probability of confidence interval for OR (multimodal continuous covariate, matching ratio 1:2, true OR: 0.75, c statistic: 0.85, naive inference).

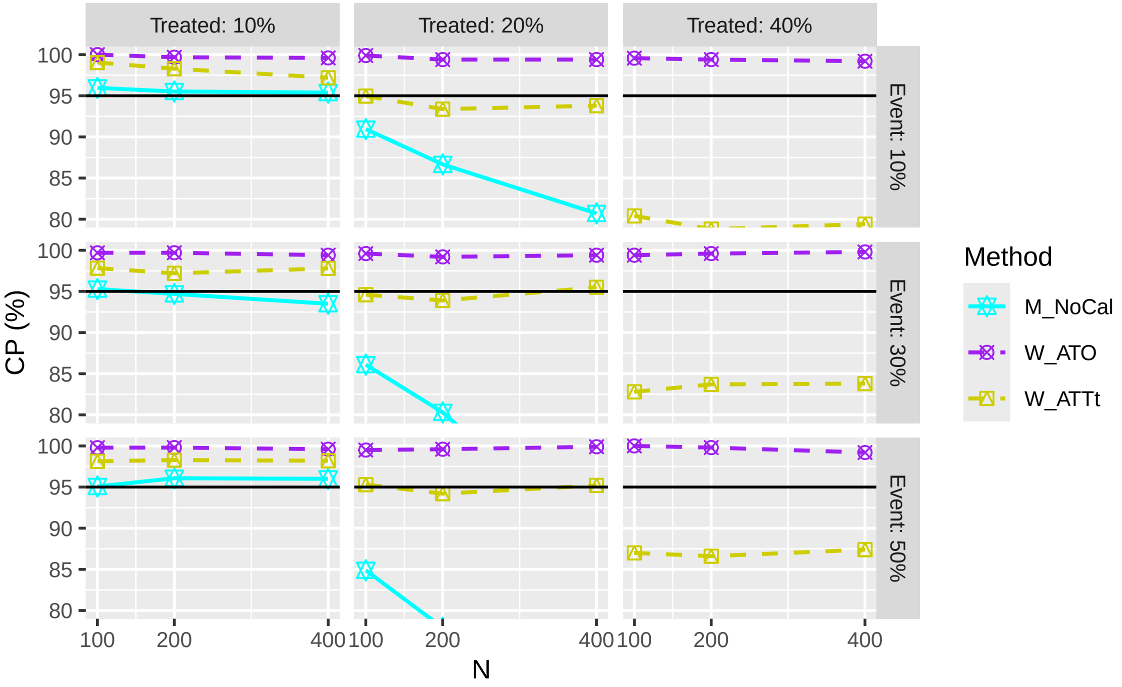

Figure S358. Coverage probability of confidence interval for OR (multimodal continuous covariate, matching ratio 1:2, true OR: 0.75, c statistic: 0.85, naive inference); other methods.

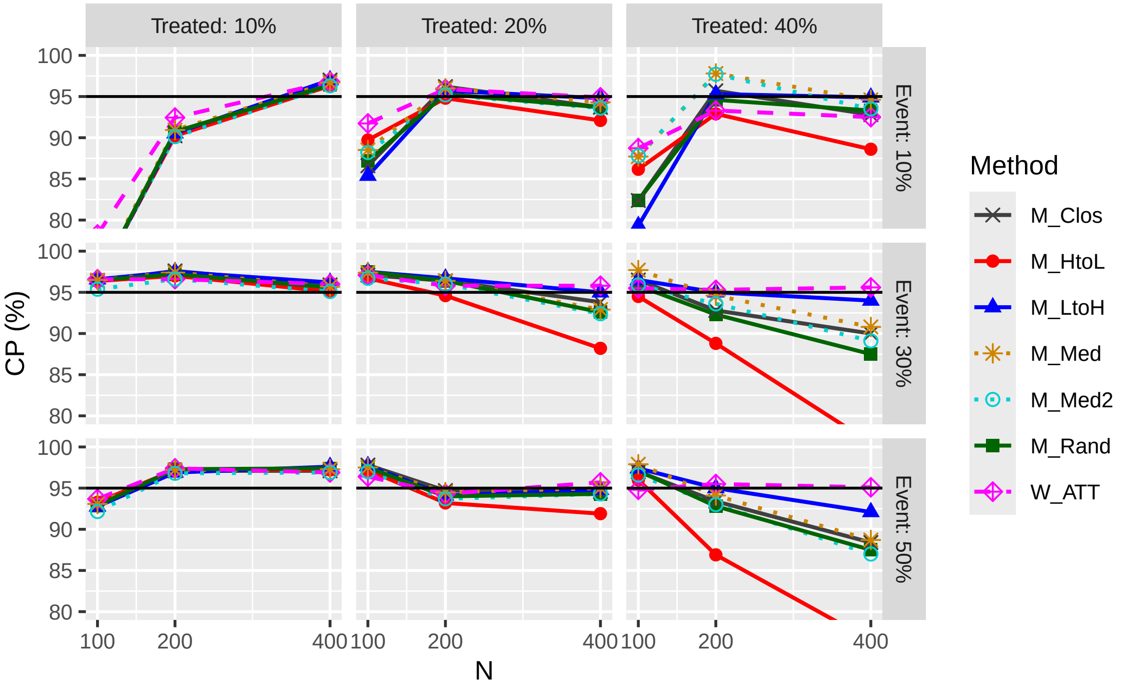

Figure S359. Coverage probability of confidence interval for OR (multimodal continuous covariate, matching ratio 1:2, true OR: 0.75, c statistic: 0.85, robust inference).

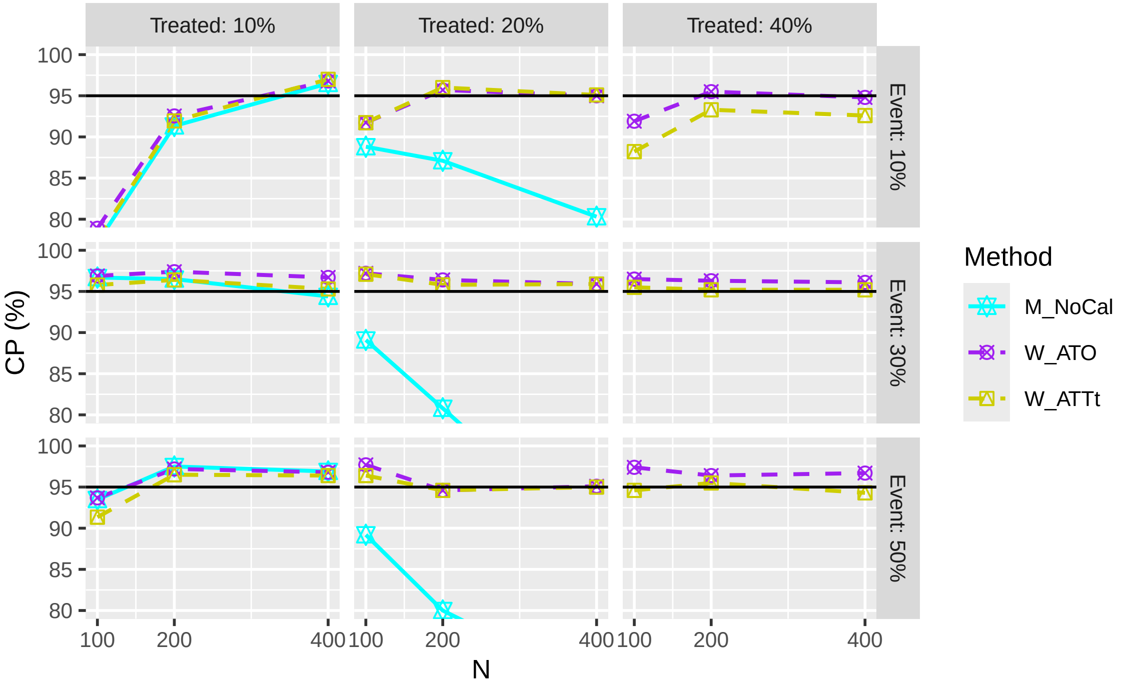

Figure S360. Coverage probability of confidence interval for OR (multimodal continuous covariate, matching ratio 1:2, true OR: 0.75, c statistic: 0.85, robust inference); other methods.

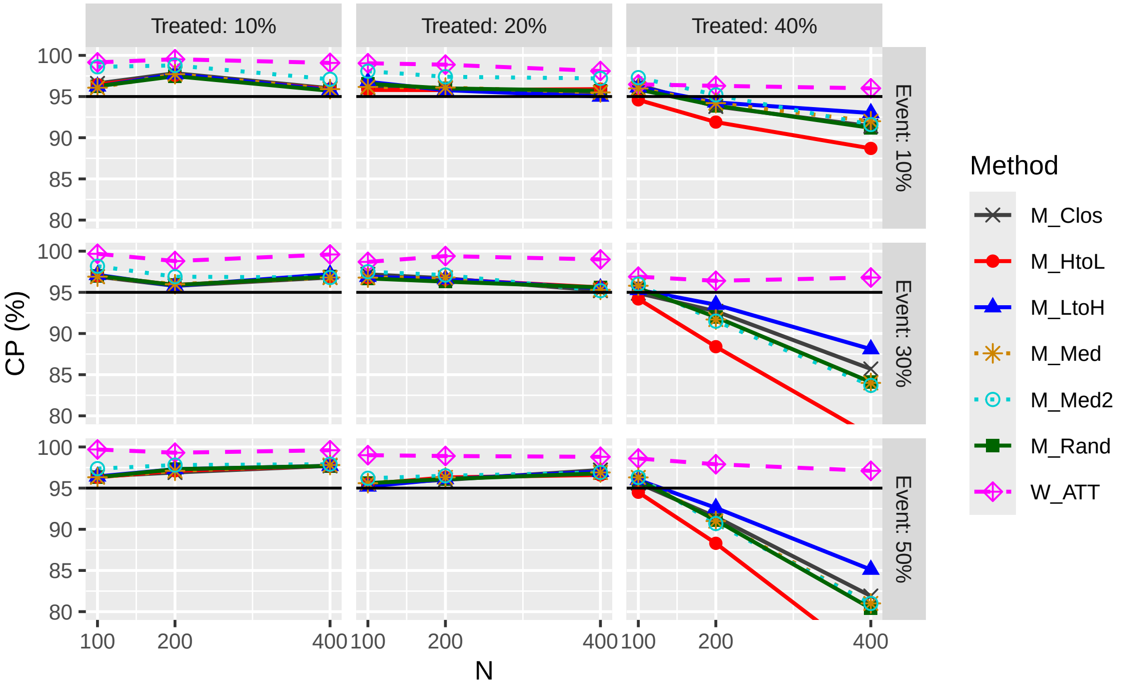

Figure S361. Coverage probability of confidence interval for OR (multimodal continuous covariate, matching ratio 1:2, true OR: 0.75, c statistic: 0.6, naive inference).

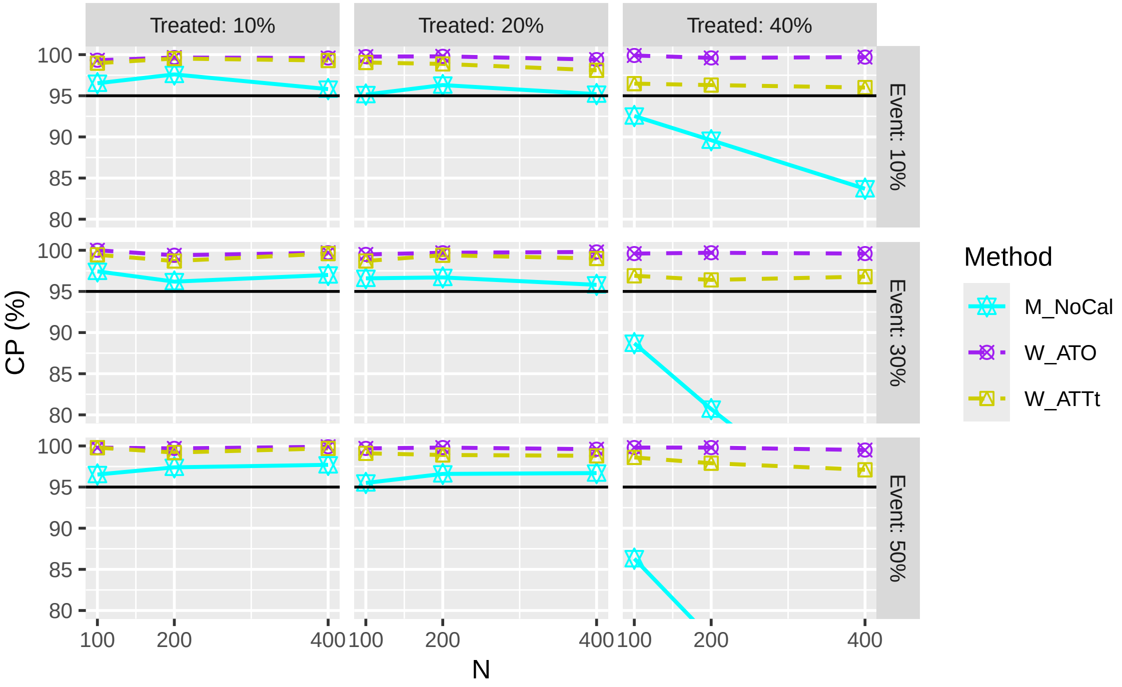

Figure S362. Coverage probability of confidence interval for OR (multimodal continuous covariate, matching ratio 1:2, true OR: 0.75, c statistic: 0.6, naive inference); other methods.

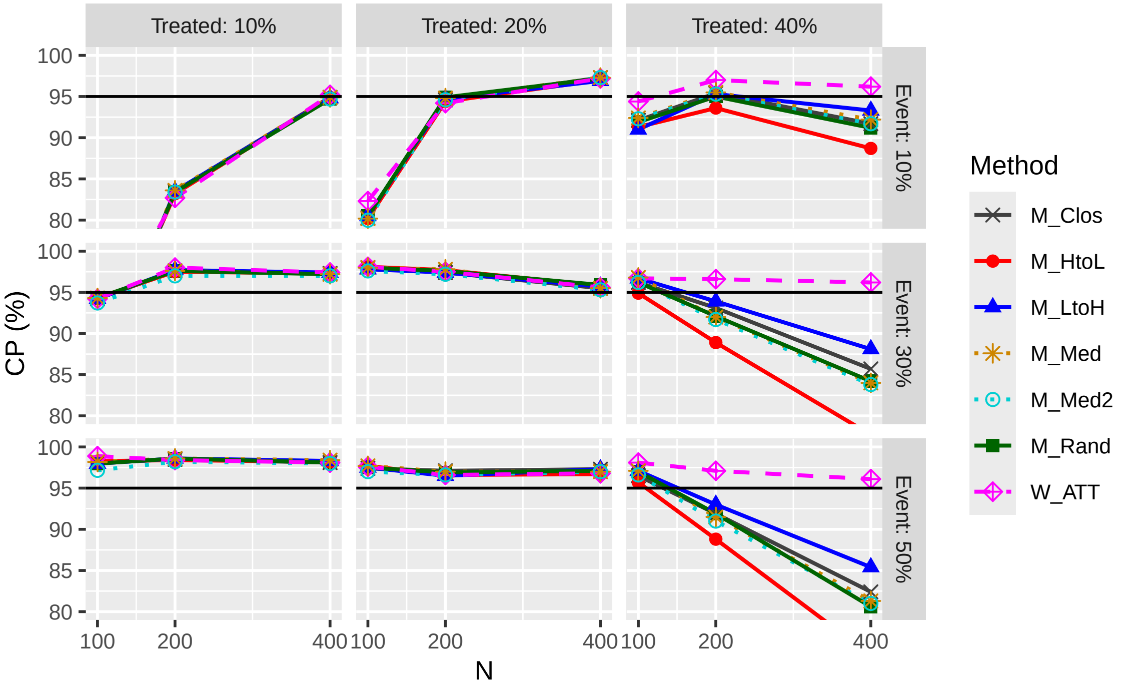

Figure S363. Coverage probability of confidence interval for OR (multimodal continuous covariate, matching ratio 1:2, true OR: 0.75, c statistic: 0.6, robust inference).

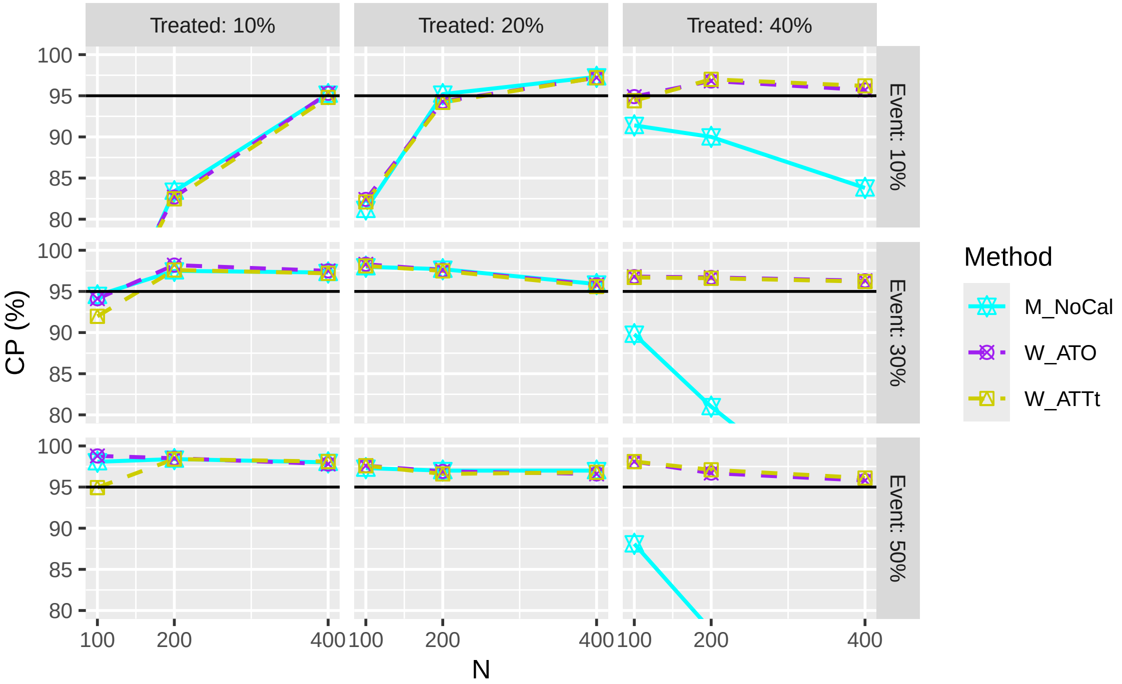

Figure S364. Coverage probability of confidence interval for OR (multimodal continuous covariate, matching ratio 1:2, true OR: 0.75, c statistic: 0.6, robust inference); other methods.

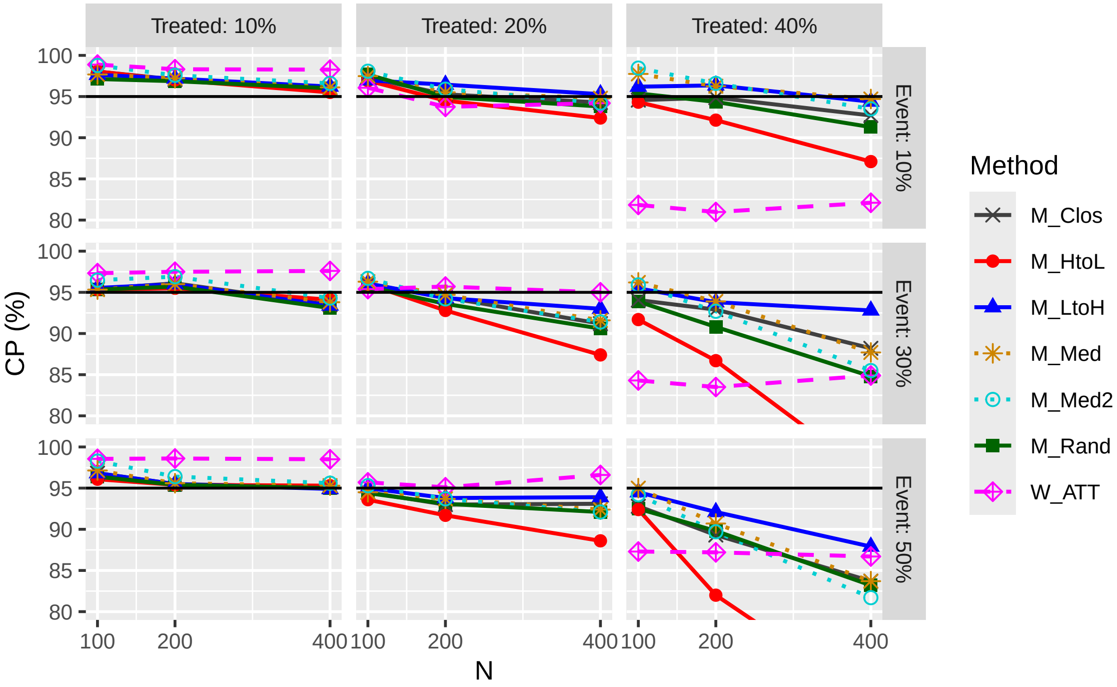

Figure S365. Coverage probability of confidence interval for OR (multimodal continuous covariate, matching ratio 1:2, true OR: 0.5, c statistic: 0.85, naive inference).

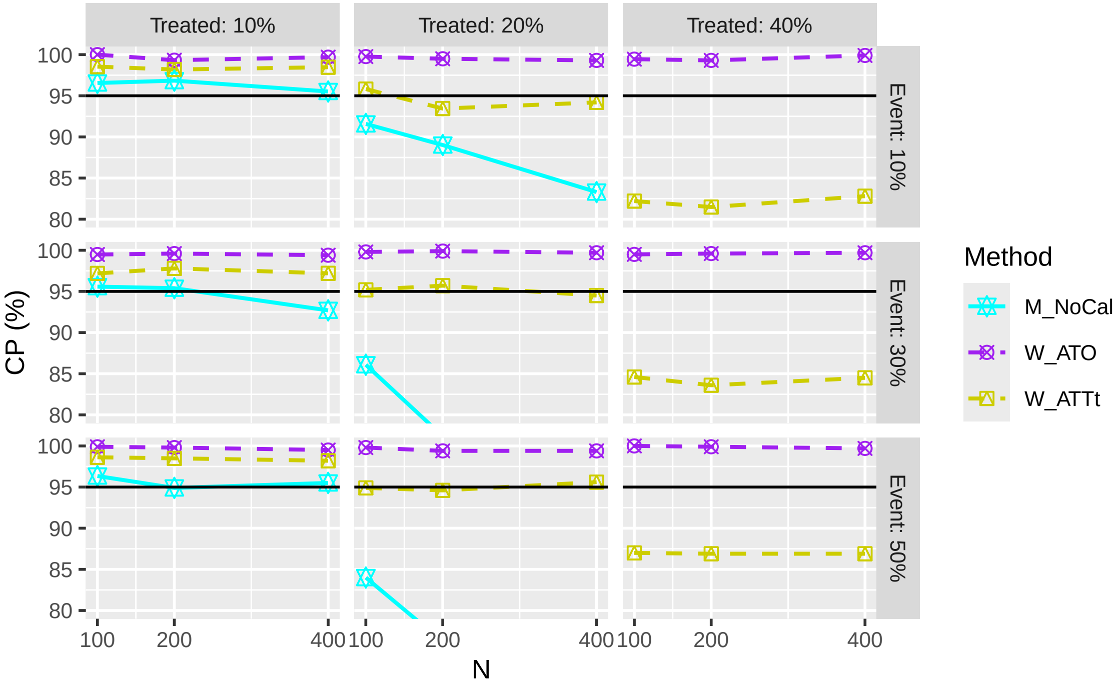

Figure S366. Coverage probability of confidence interval for OR (multimodal continuous covariate, matching ratio 1:2, true OR: 0.5, c statistic: 0.85, naive inference); other methods.

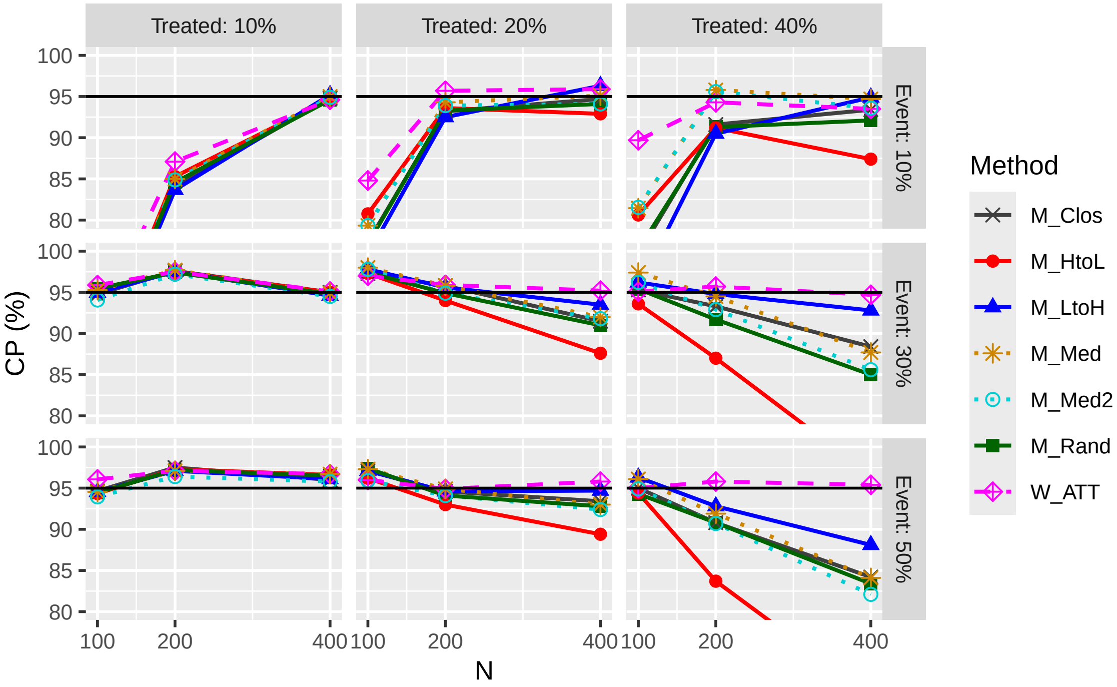

Figure S367. Coverage probability of confidence interval for OR (multimodal continuous covariate, matching ratio 1:2, true OR: 0.5, c statistic: 0.85, robust inference).

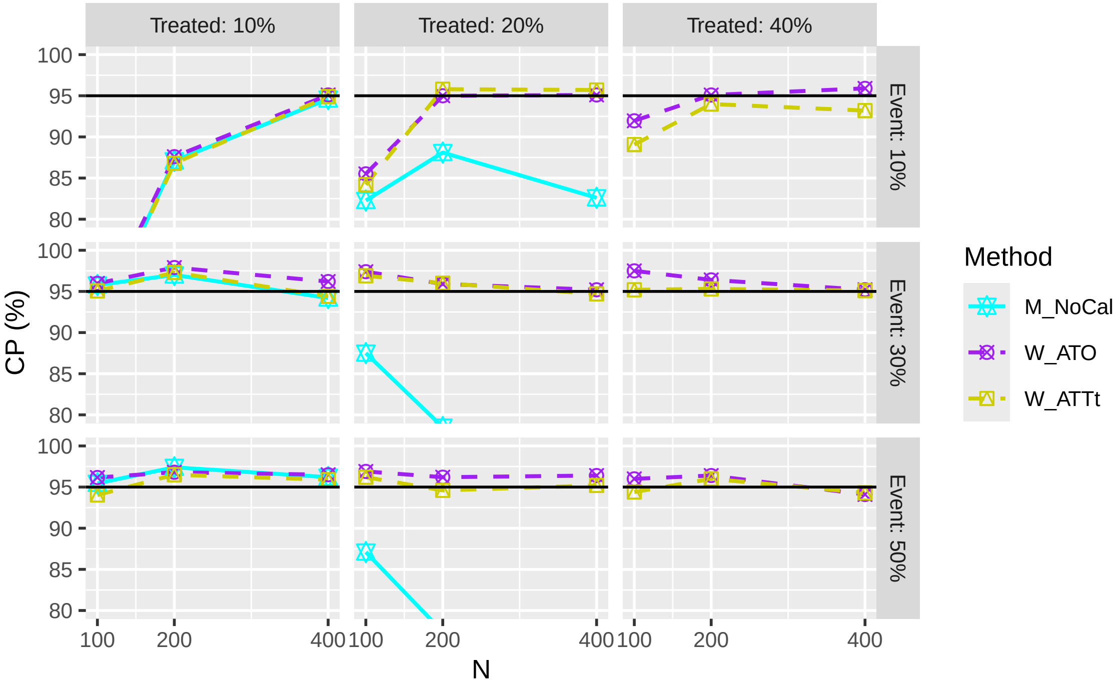

Figure S368. Coverage probability of confidence interval for OR (multimodal continuous covariate, matching ratio 1:2, true OR: 0.5, c statistic: 0.85, robust inference); other methods.

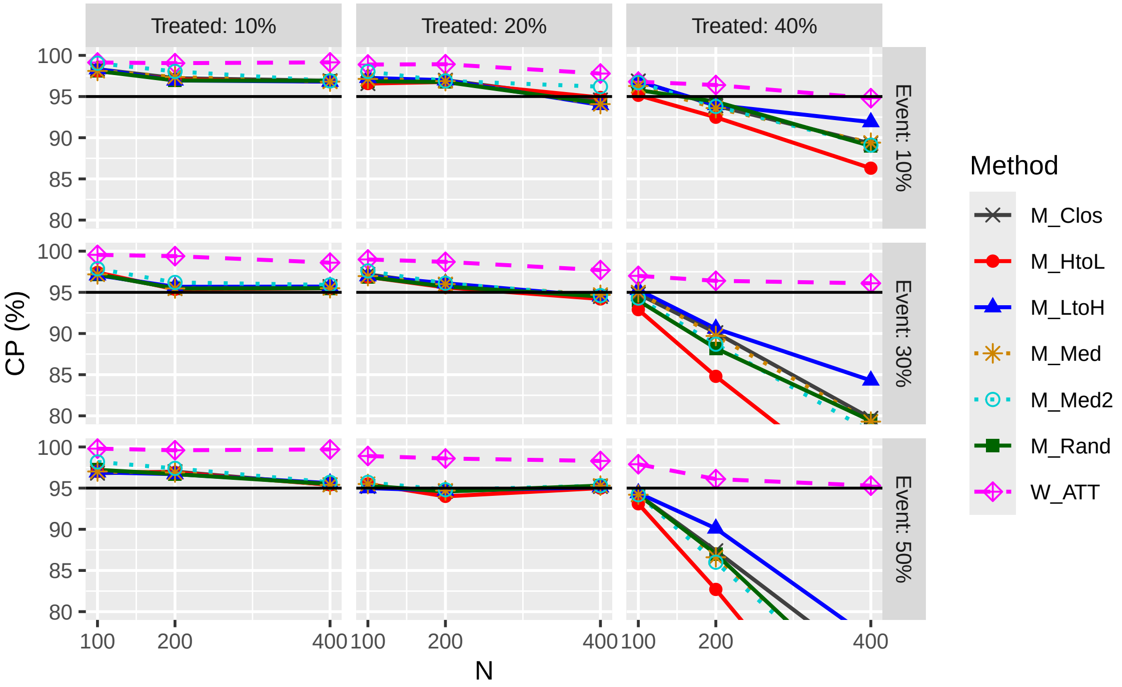

Figure S369. Coverage probability of confidence interval for OR (multimodal continuous covariate, matching ratio 1:2, true OR: 0.5, c statistic: 0.6, naive inference).

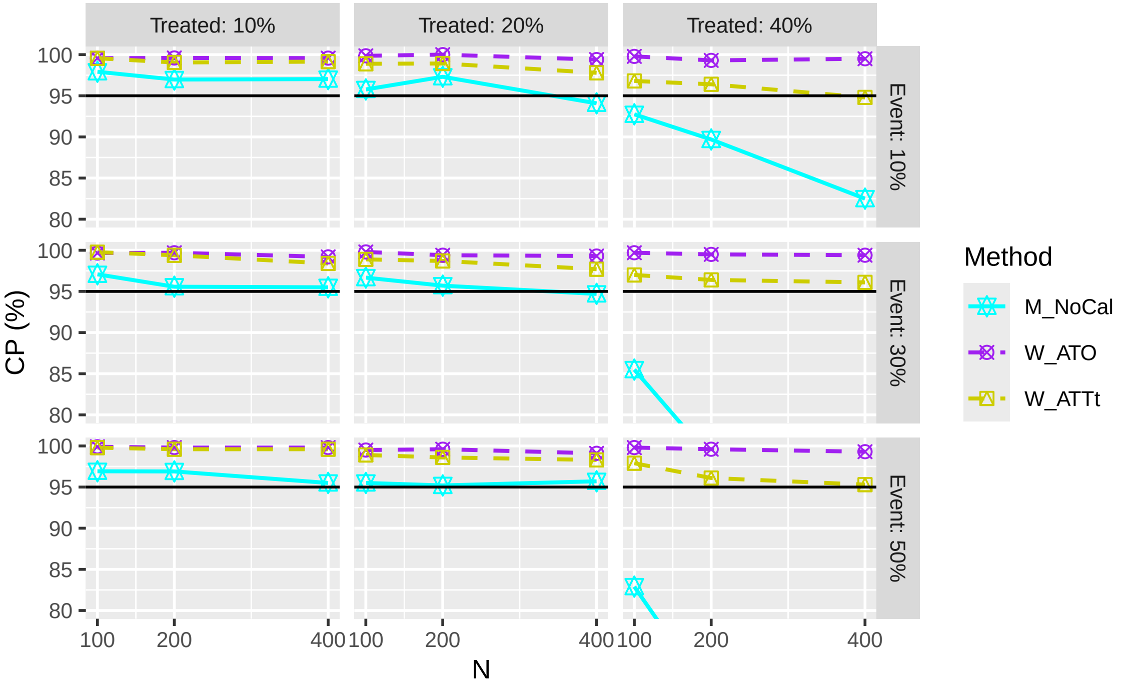

Figure S370. Coverage probability of confidence interval for OR (multimodal continuous covariate, matching ratio 1:2, true OR: 0.5, c statistic: 0.6, naive inference); other methods.

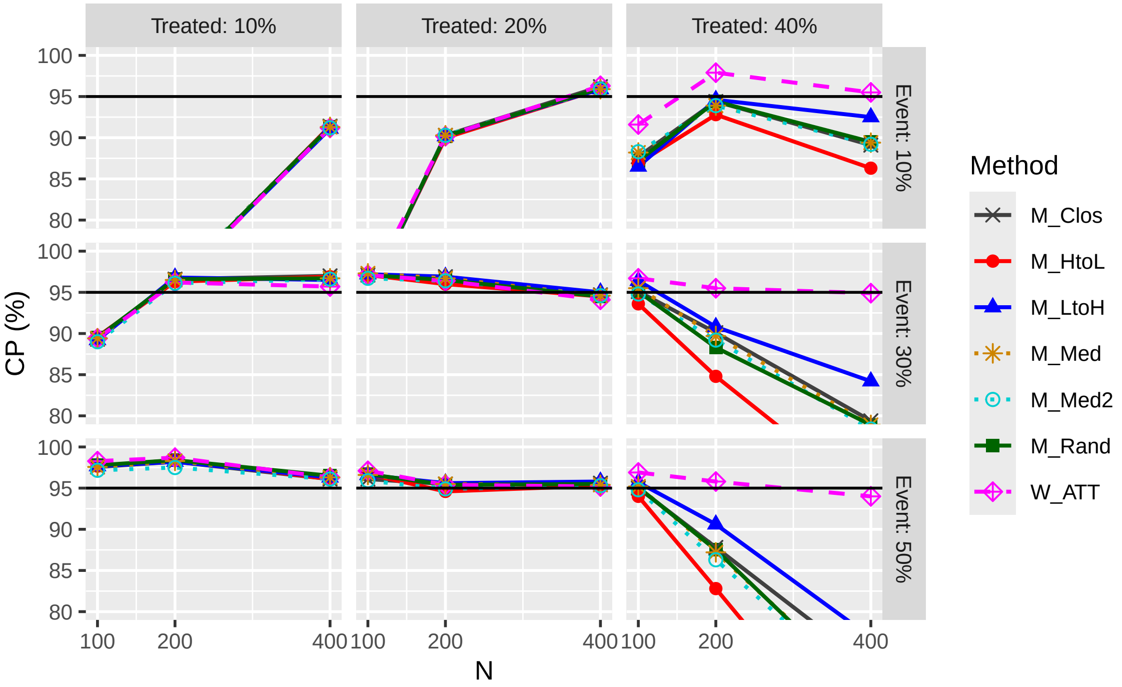

Figure S371. Coverage probability of confidence interval for OR (multimodal continuous covariate, matching ratio 1:2, true OR: 0.5, c statistic: 0.6, robust inference).

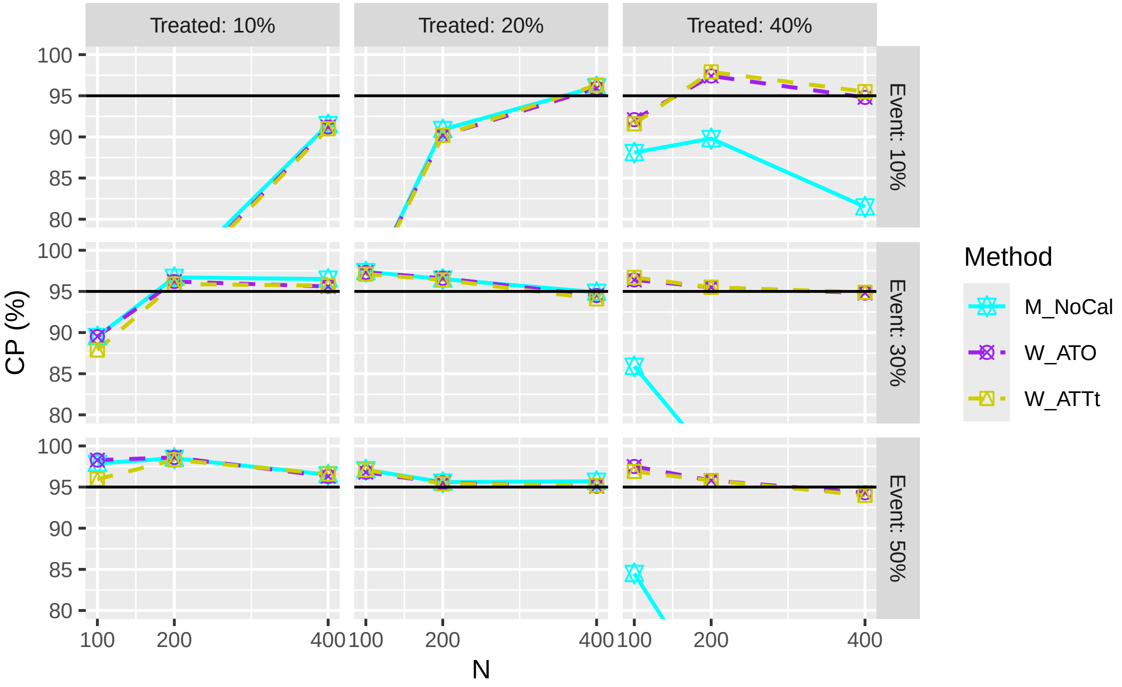

Figure S372. Coverage probability of confidence interval for OR (multimodal continuous covariate, matching ratio 1:2, true OR: 0.5, c statistic: 0.6, robust inference); other methods.

S6. Mean percentage bias of standard error for log odds ratio (caliper: 25%)

Only results with log odds ratios less than 10 were included.

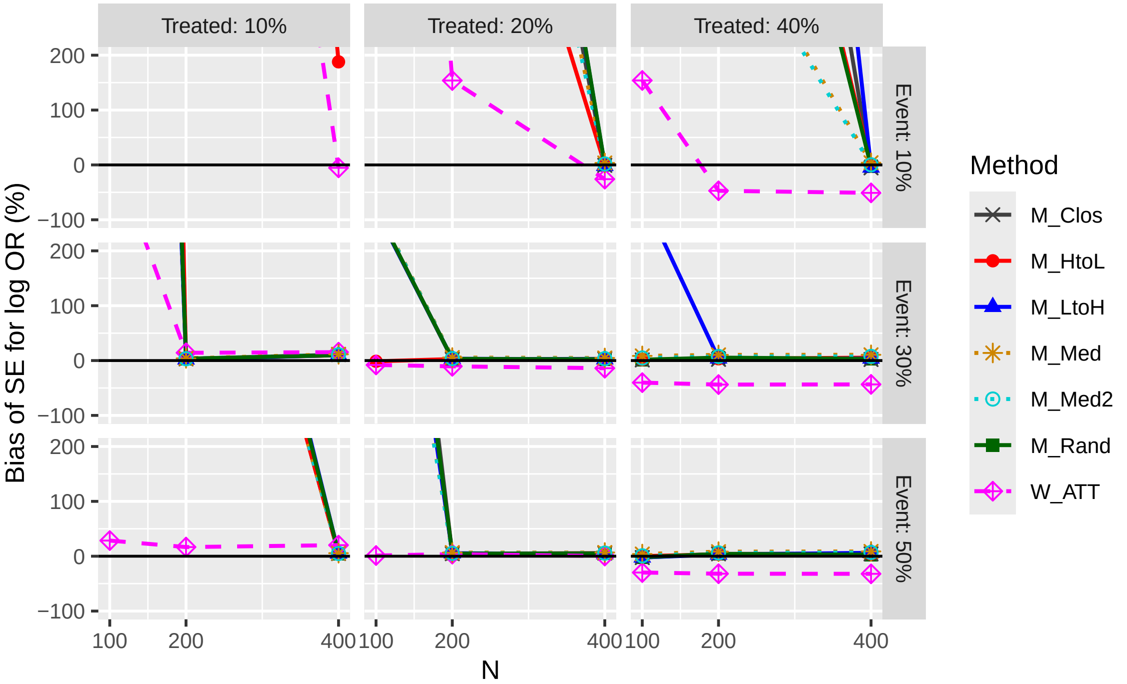

Figure S373. Mean bias of standard error for log odds ratio (unimodal continuous covariate, matching ratio 1:1, true OR: 1, c statistic: 0.85, naive inference).

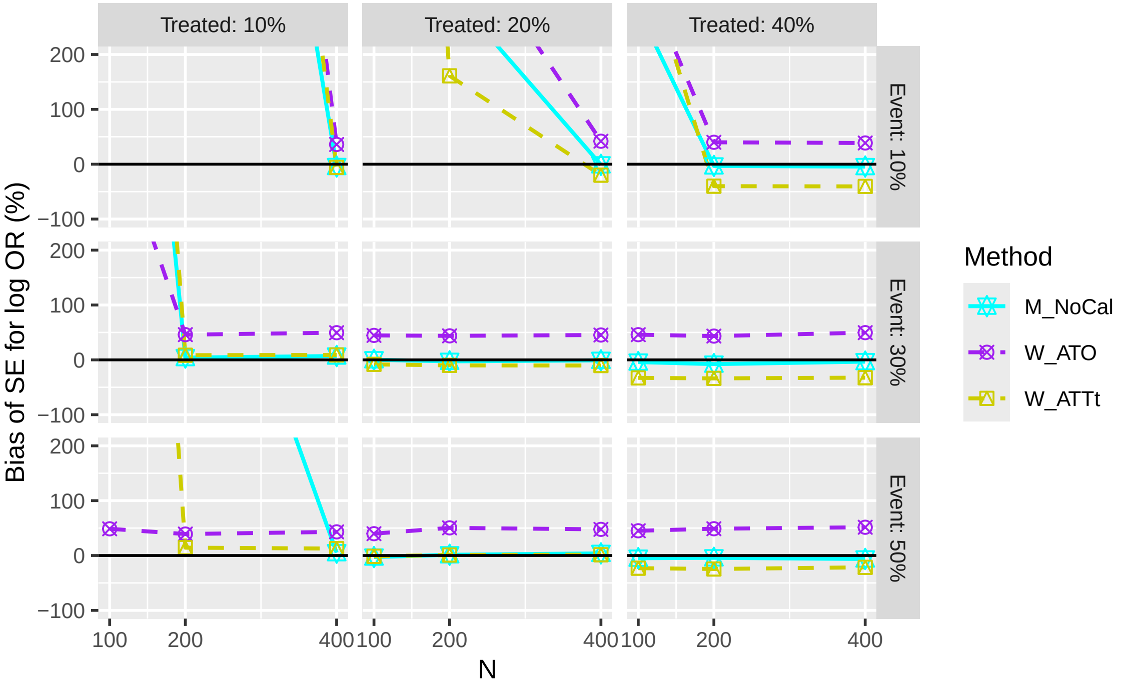

Figure S374. Mean bias of standard error for log odds ratio (unimodal continuous covariate, matching ratio 1:1, true OR: 1, c statistic: 0.85, naive inference); other methods.

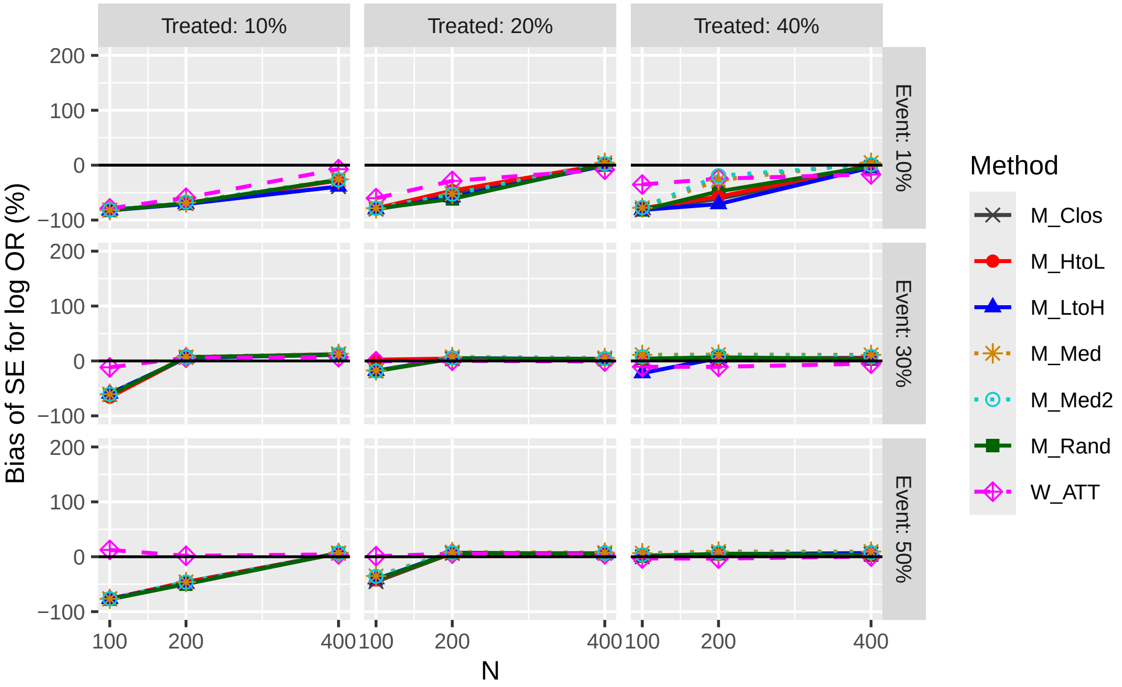

Figure S375. Mean bias of standard error for log odds ratio (unimodal continuous covariate, matching ratio 1:1, true OR: 1, c statistic: 0.85, robust inference).

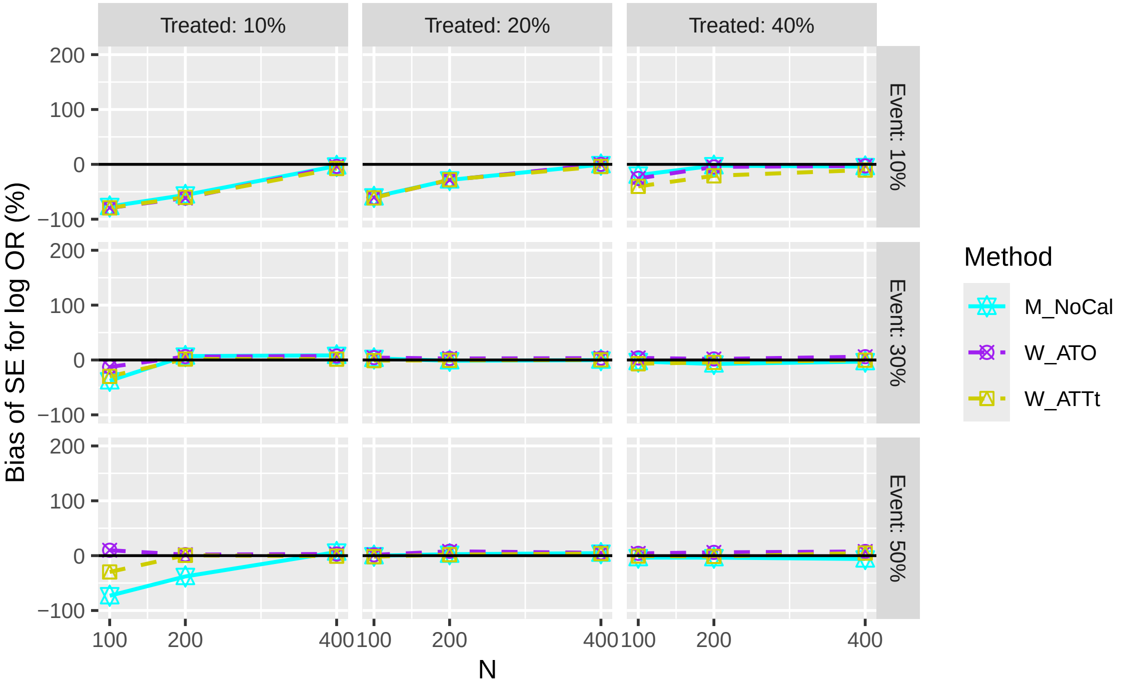

Figure S376. Mean bias of standard error for log odds ratio (unimodal continuous covariate, matching ratio 1:1, true OR: 1, c statistic: 0.85, robust inference); other methods.

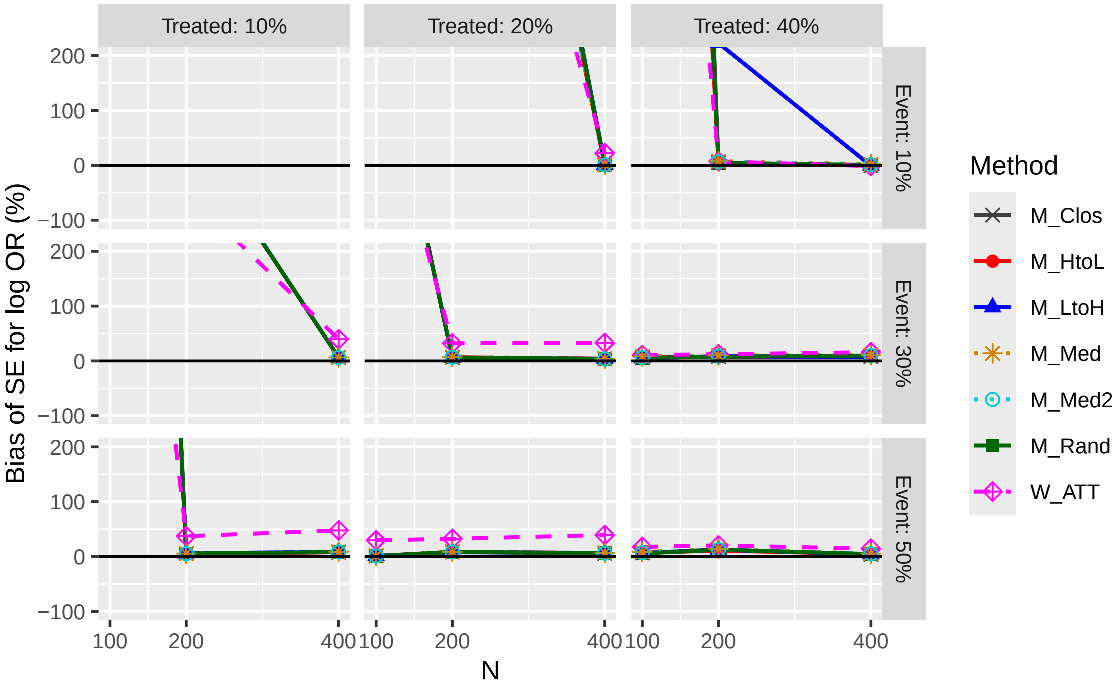

Figure S377. Mean bias of standard error for log odds ratio (unimodal continuous covariate, matching ratio 1:1, true OR: 1, c statistic: 0.6, naive inference).

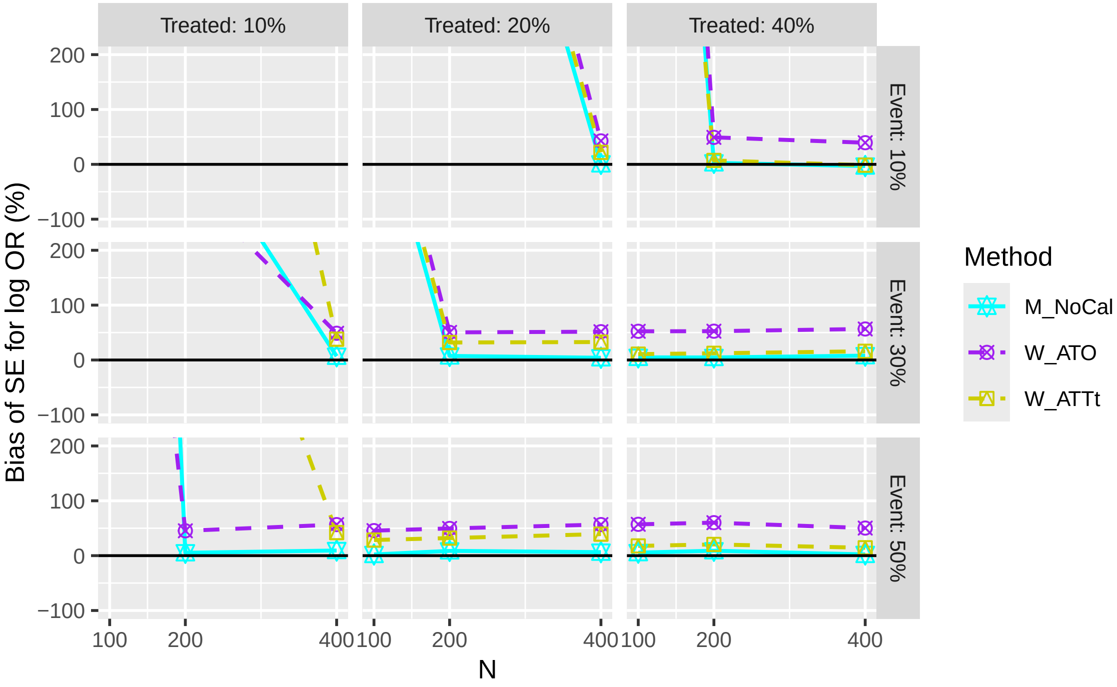

Figure S378. Mean bias of standard error for log odds ratio (unimodal continuous covariate, matching ratio 1:1, true OR: 1, c statistic: 0.6, naive inference); other methods.

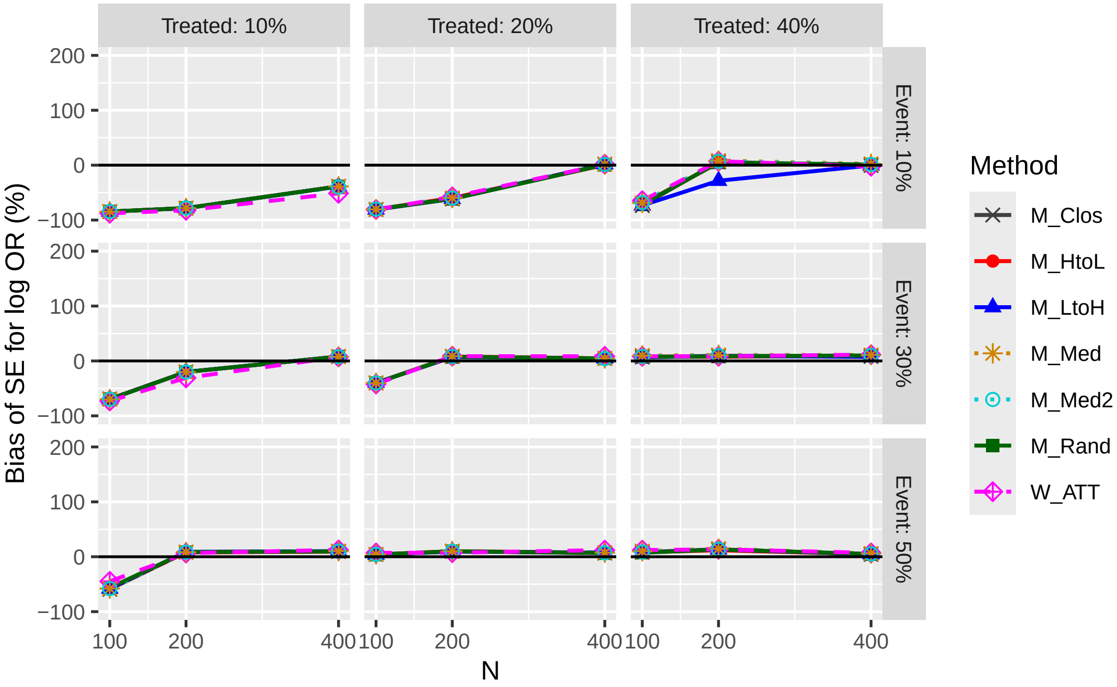

Figure S379. Mean bias of standard error for log odds ratio (unimodal continuous covariate, matching ratio 1:1, true OR: 1, c statistic: 0.6, robust inference).

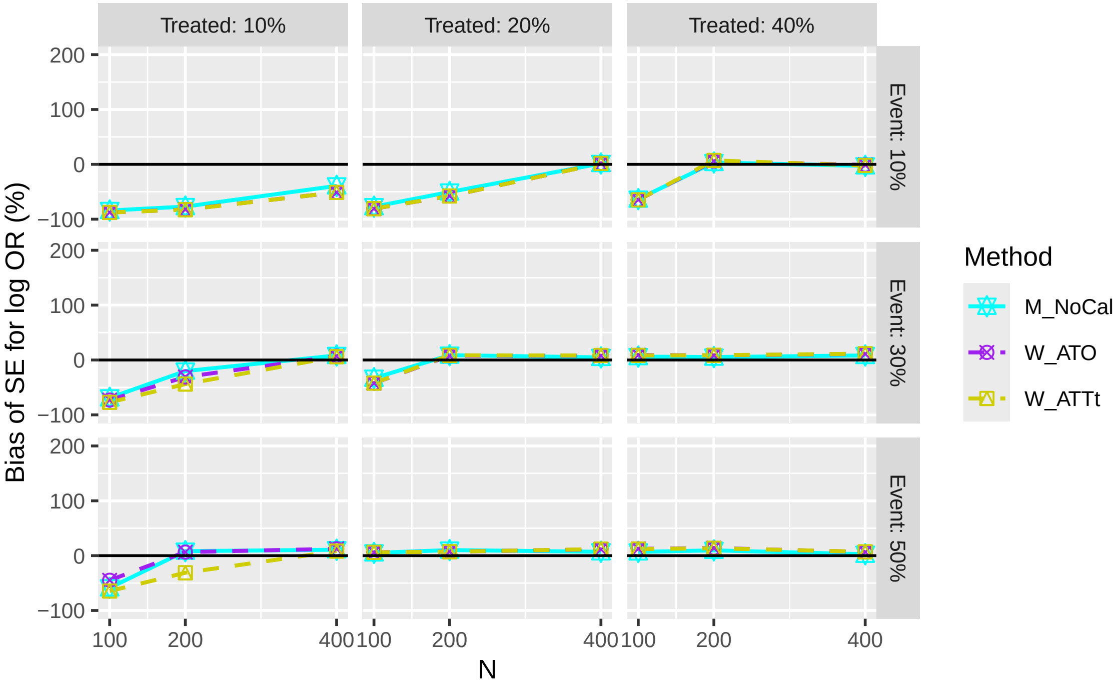

Figure S380. Mean bias of standard error for log odds ratio (unimodal continuous covariate, matching ratio 1:1, true OR: 1, c statistic: 0.6, robust inference); other methods.

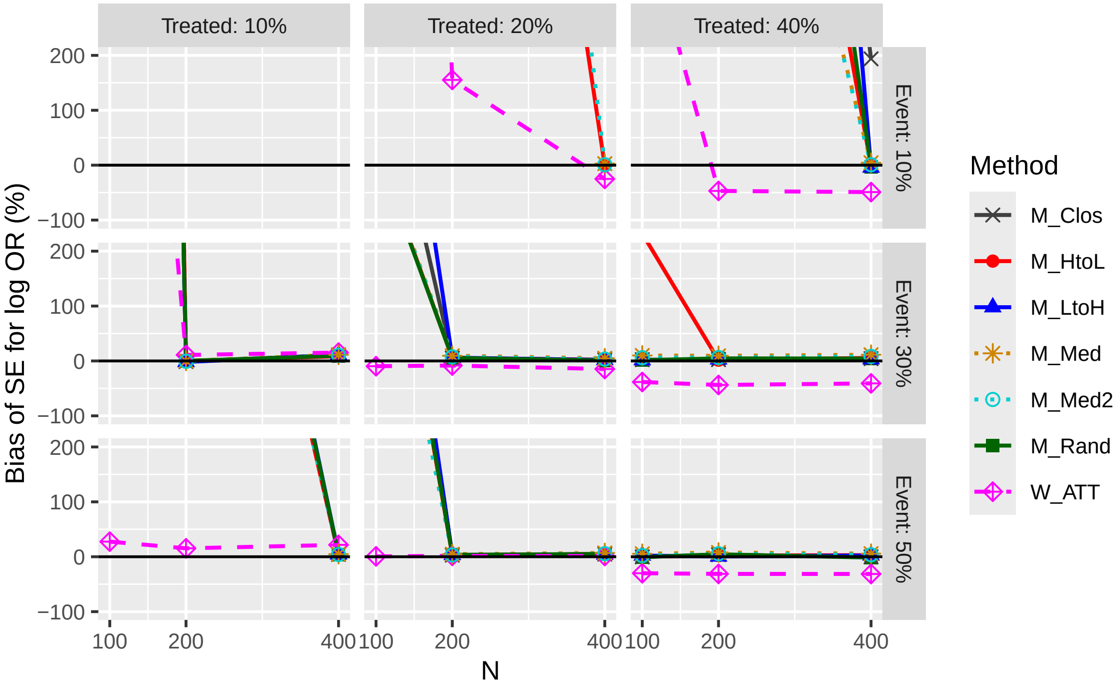

Figure S381. Mean bias of standard error for log odds ratio (unimodal continuous covariate, matching ratio 1:1, true OR: 0.75, c statistic: 0.85, naive inference).

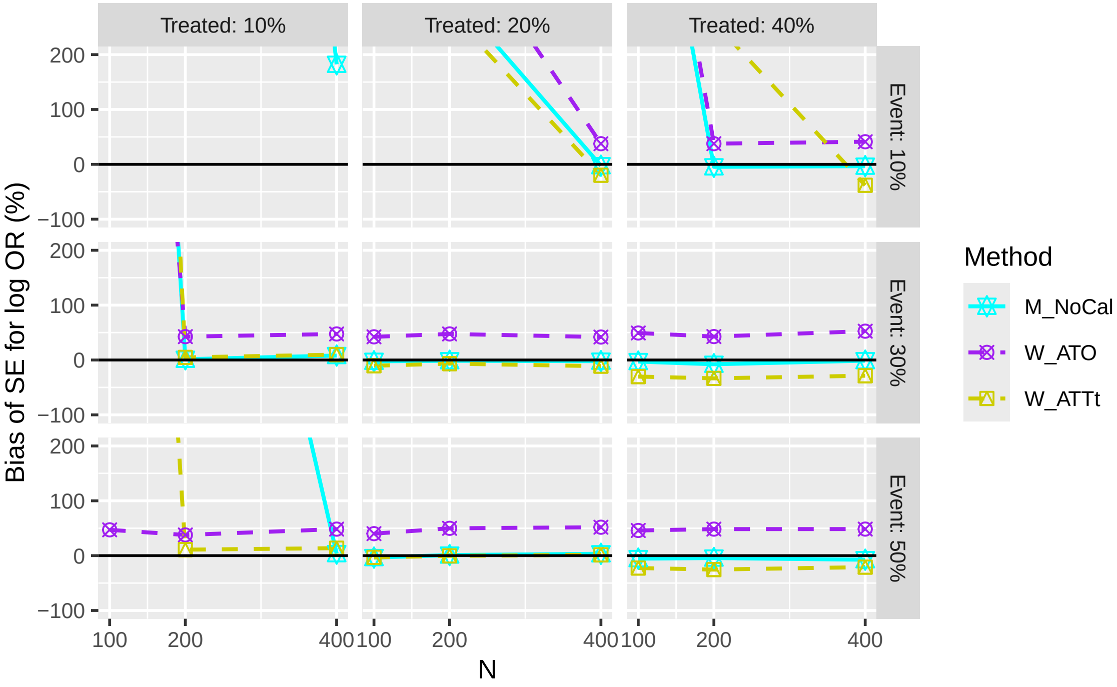

Figure S382. Mean bias of standard error for log odds ratio (unimodal continuous covariate, matching ratio 1:1, true OR: 0.75, c statistic: 0.85, naive inference); other methods.

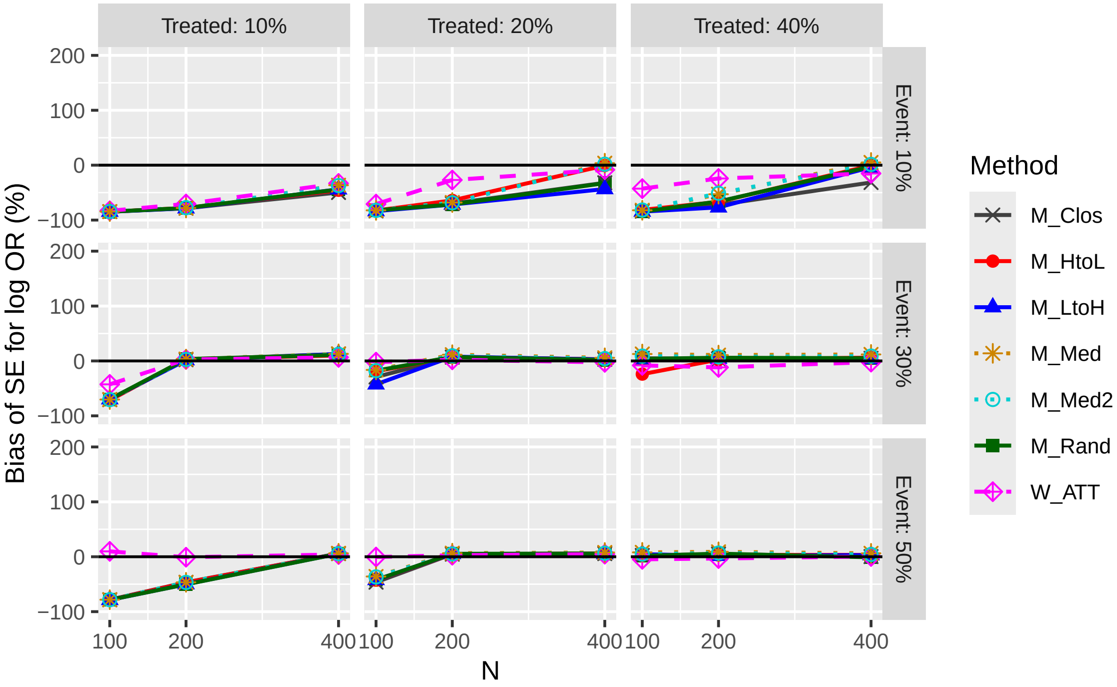

Figure S383. Mean bias of standard error for log odds ratio (unimodal continuous covariate, matching ratio 1:1, true OR: 0.75, c statistic: 0.85, robust inference).

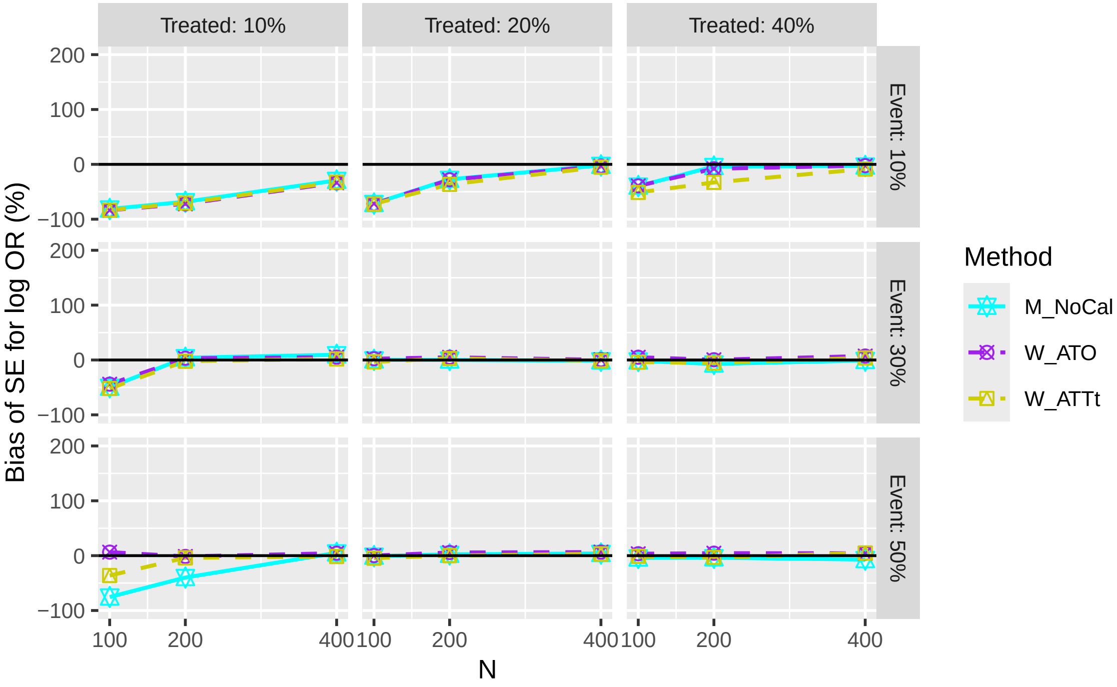

Figure S384. Mean bias of standard error for log odds ratio (unimodal continuous covariate, matching ratio 1:1, true OR: 0.75, c statistic: 0.85, robust inference); other methods.

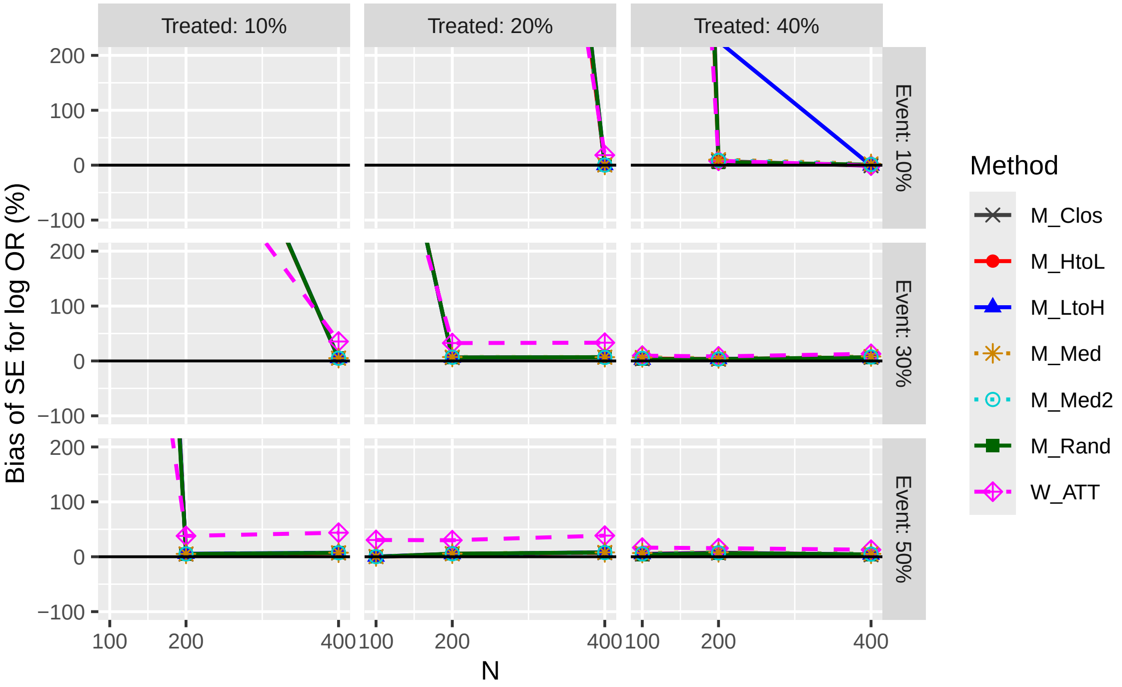

Figure S385. Mean bias of standard error for log odds ratio (unimodal continuous covariate, matching ratio 1:1, true OR: 0.75, c statistic: 0.6, naive inference).

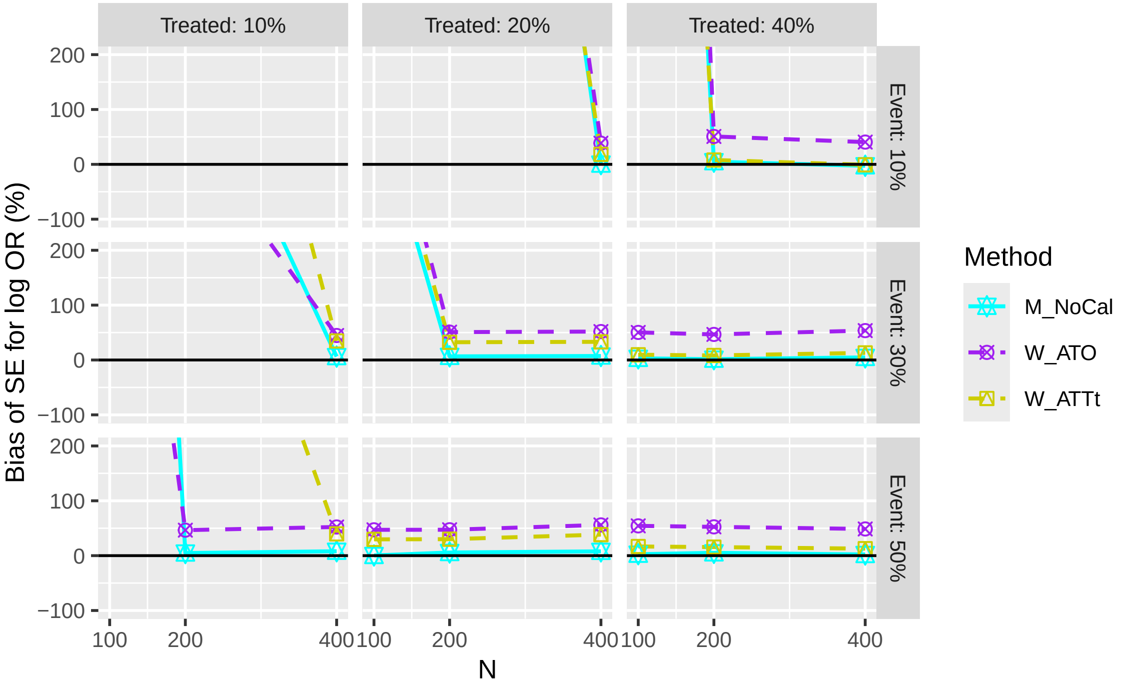

Figure S386. Mean bias of standard error for log odds ratio (unimodal continuous covariate, matching ratio 1:1, true OR: 0.75, c statistic: 0.6, naive inference); other methods.

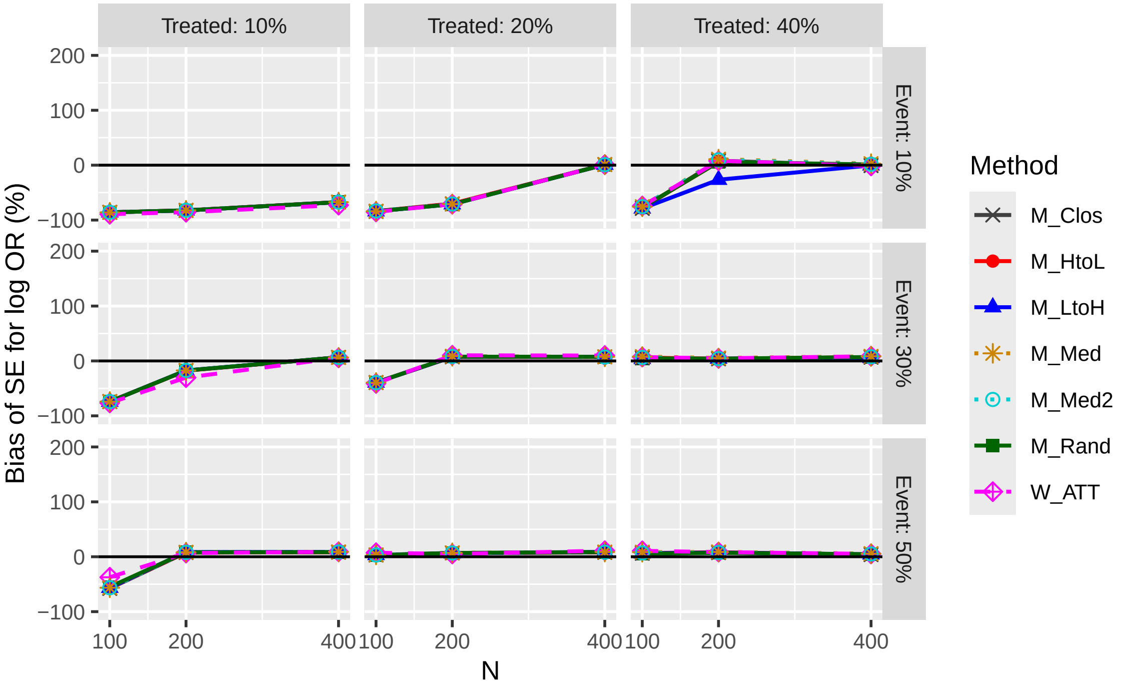

Figure S387. Mean bias of standard error for log odds ratio (unimodal continuous covariate, matching ratio 1:1, true OR: 0.75, c statistic: 0.6, robust inference).

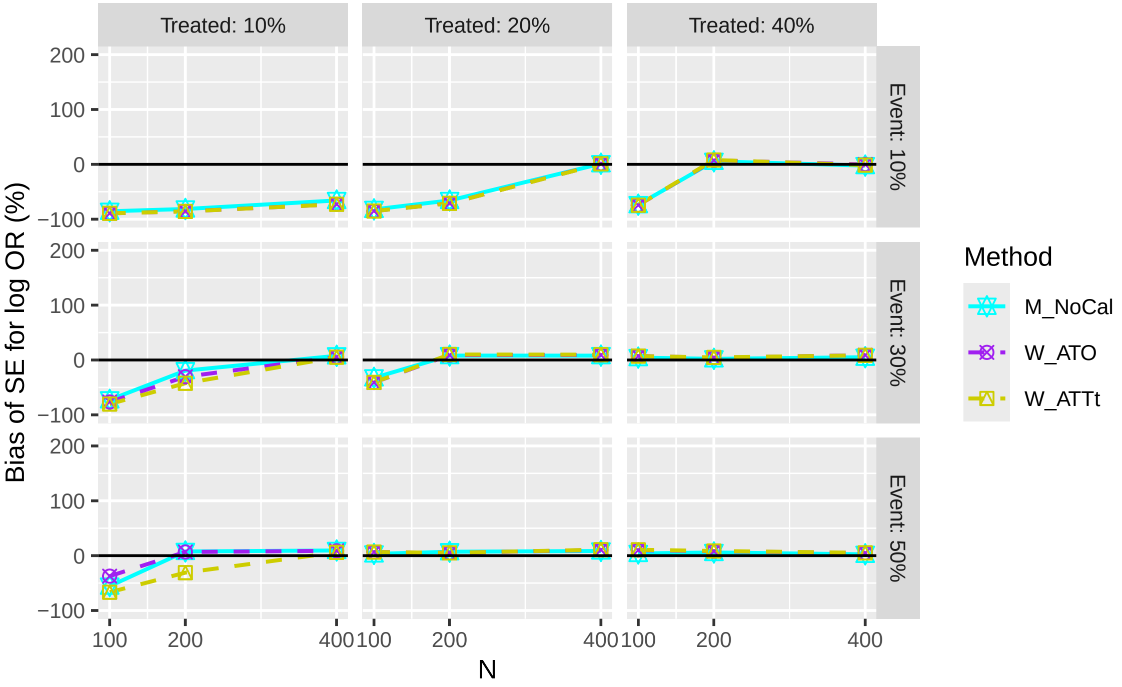

Figure S388. Mean bias of standard error for log odds ratio (unimodal continuous covariate, matching ratio 1:1, true OR: 0.75, c statistic: 0.6, robust inference); other methods.

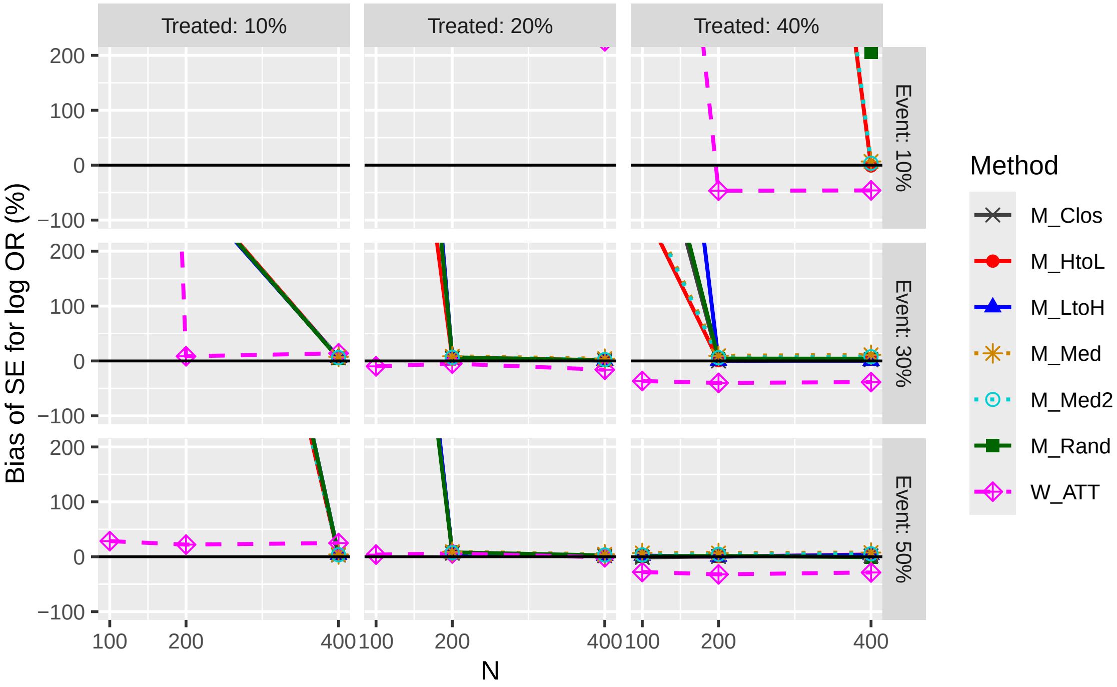

Figure S389. Mean bias of standard error for log odds ratio (unimodal continuous covariate, matching ratio 1:1, true OR: 0.5, c statistic: 0.85, naive inference).

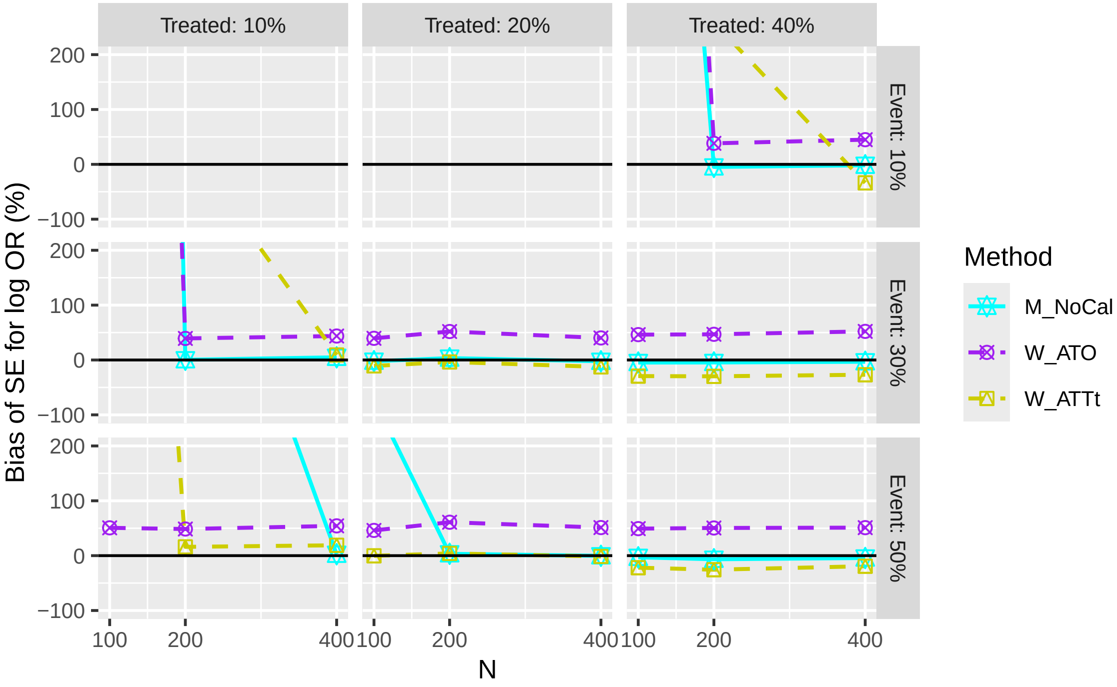

Figure S390. Mean bias of standard error for log odds ratio (unimodal continuous covariate, matching ratio 1:1, true OR: 0.5, c statistic: 0.85, naive inference); other methods.

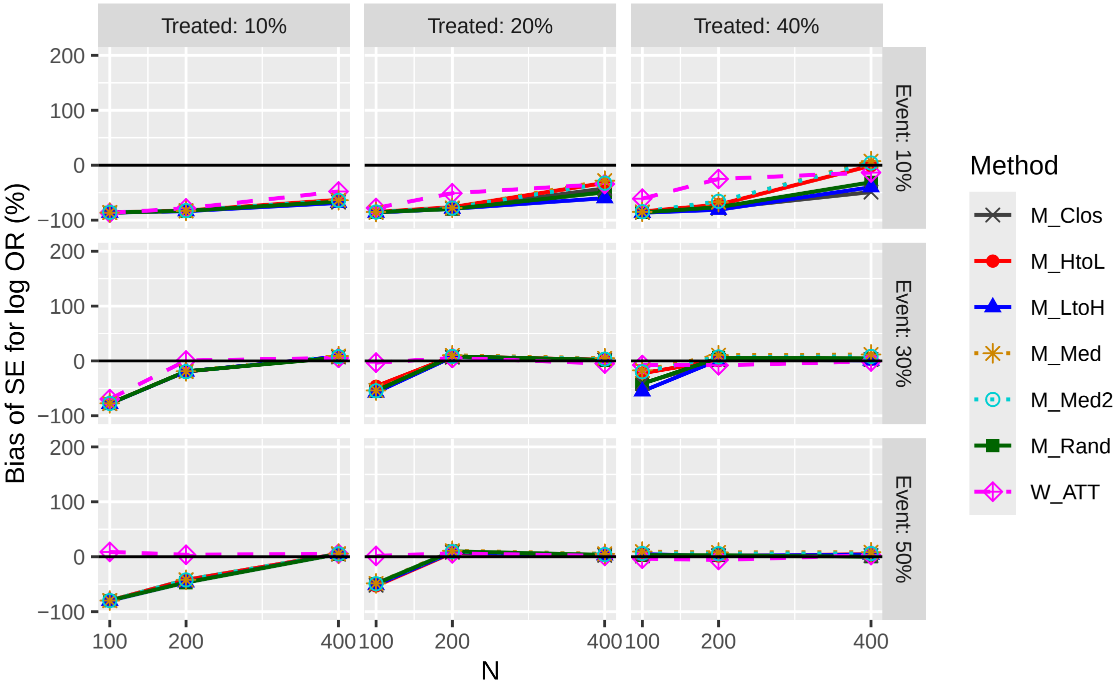

Figure S391. Mean bias of standard error for log odds ratio (unimodal continuous covariate, matching ratio 1:1, true OR: 0.5, c statistic: 0.85, robust inference).

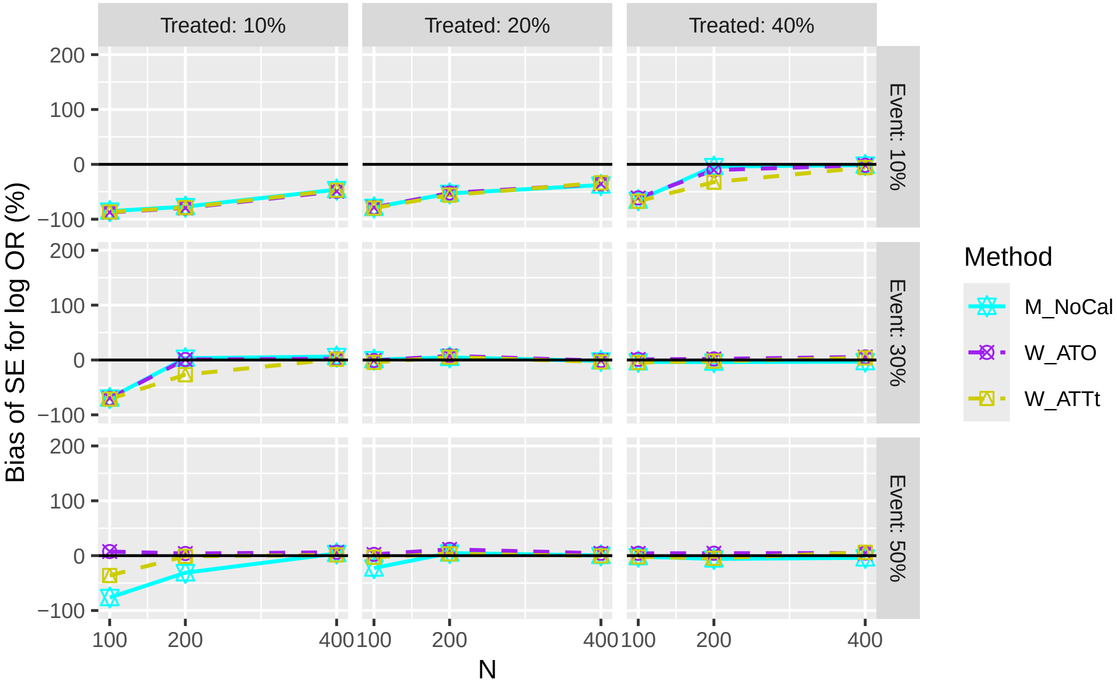

Figure S392. Mean bias of standard error for log odds ratio (unimodal continuous covariate, matching ratio 1:1, true OR: 0.5, c statistic: 0.85, robust inference); other methods.

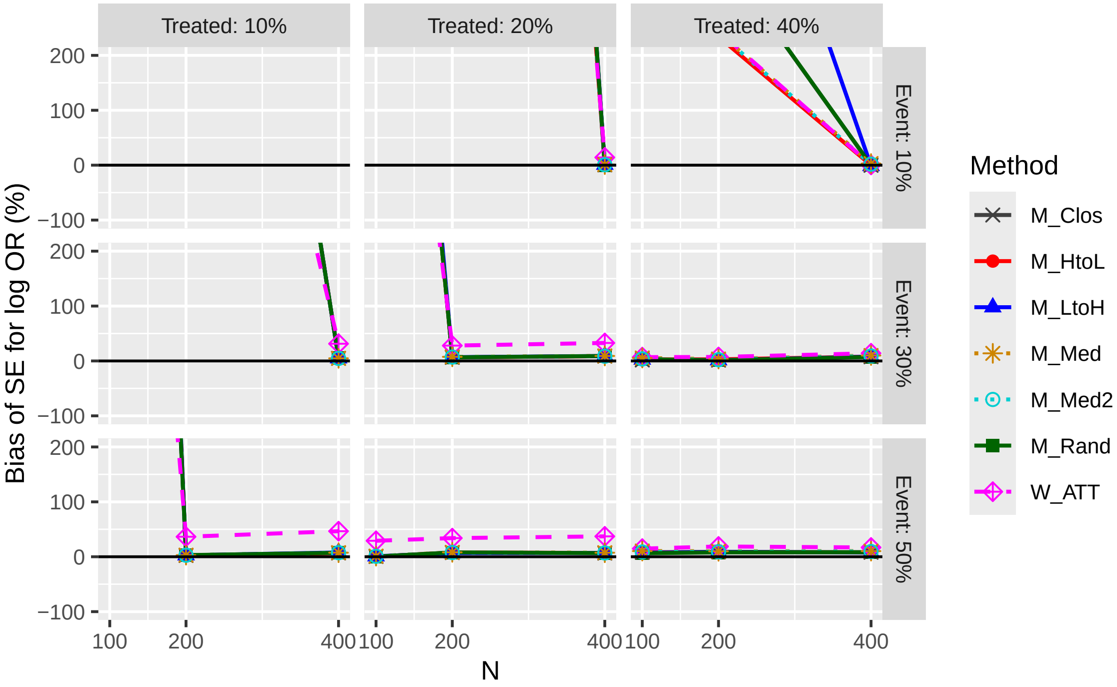

Figure S393. Mean bias of standard error for log odds ratio (unimodal continuous covariate, matching ratio 1:1, true OR: 0.5, c statistic: 0.6, naive inference).

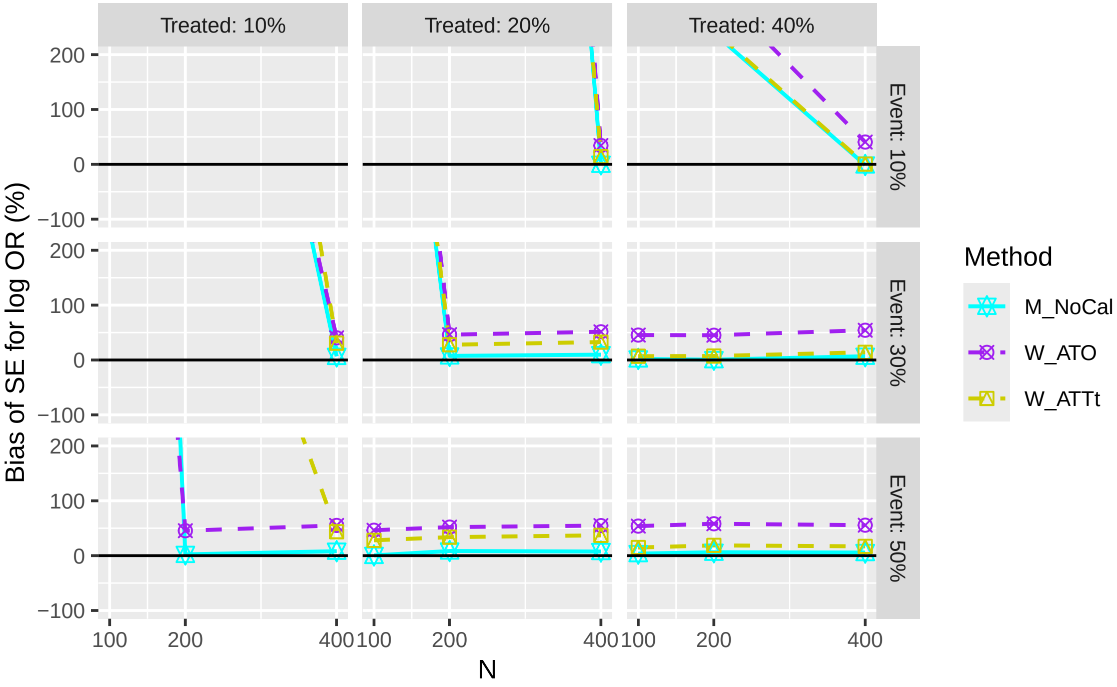

Figure S394. Mean bias of standard error for log odds ratio (unimodal continuous covariate, matching ratio 1:1, true OR: 0.5, c statistic: 0.6, naive inference); other methods.

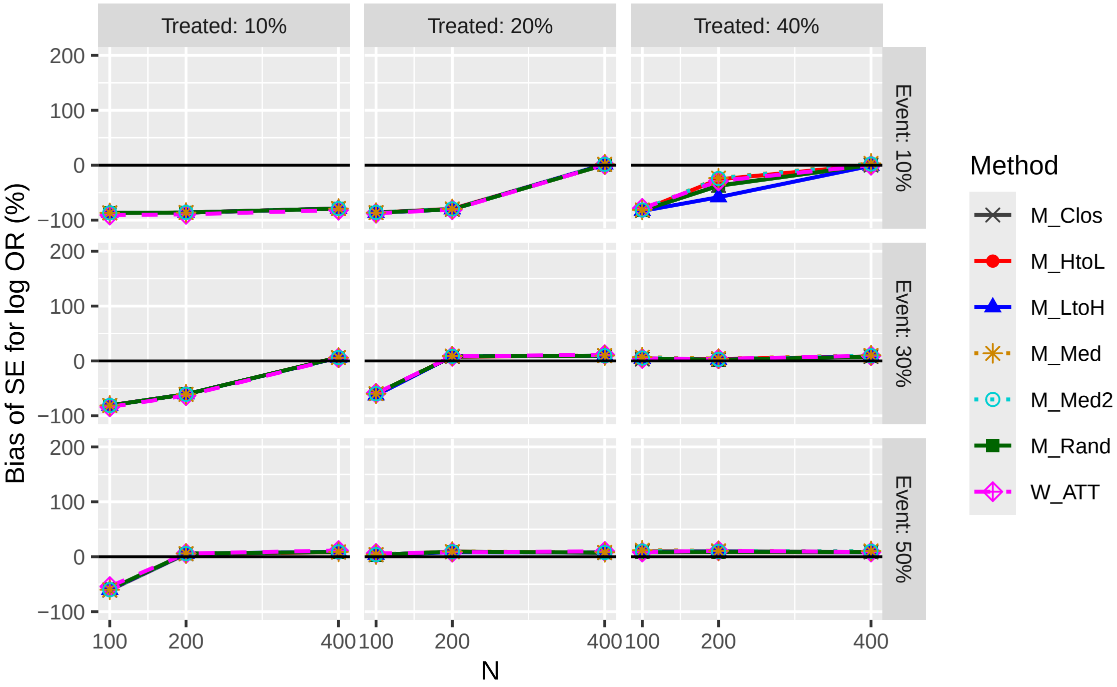

Figure S395. Mean bias of standard error for log odds ratio (unimodal continuous covariate, matching ratio 1:1, true OR: 0.5, c statistic: 0.6, robust inference).

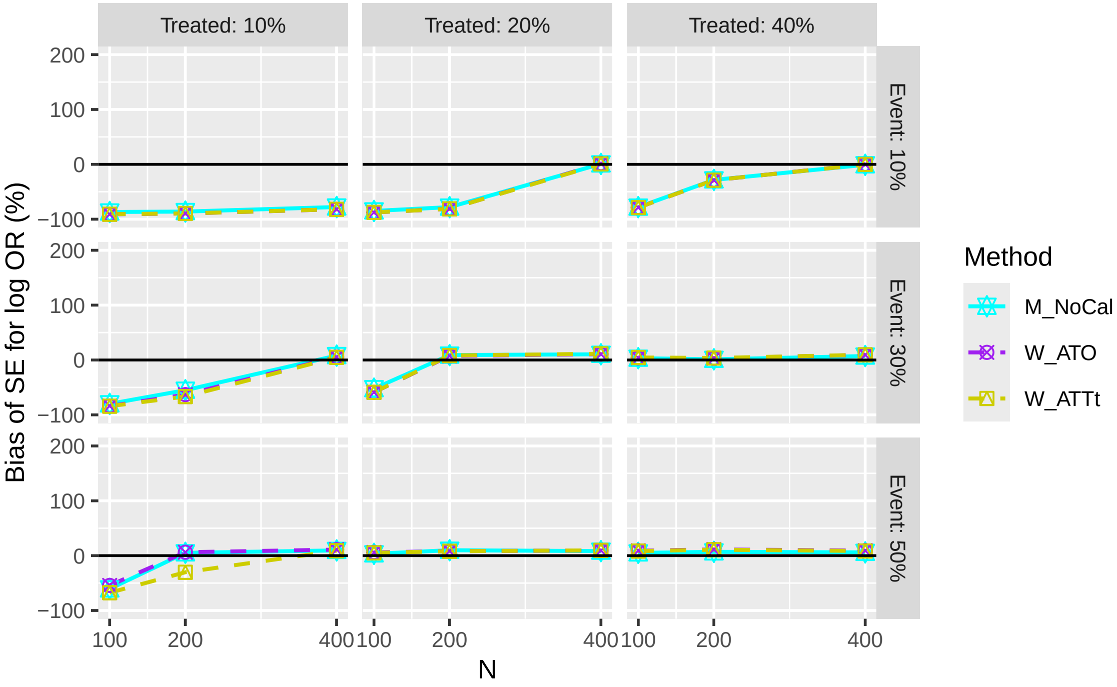

Figure S396. Mean bias of standard error for log odds ratio (unimodal continuous covariate, matching ratio 1:1, true OR: 0.5, c statistic: 0.6, robust inference); other methods.

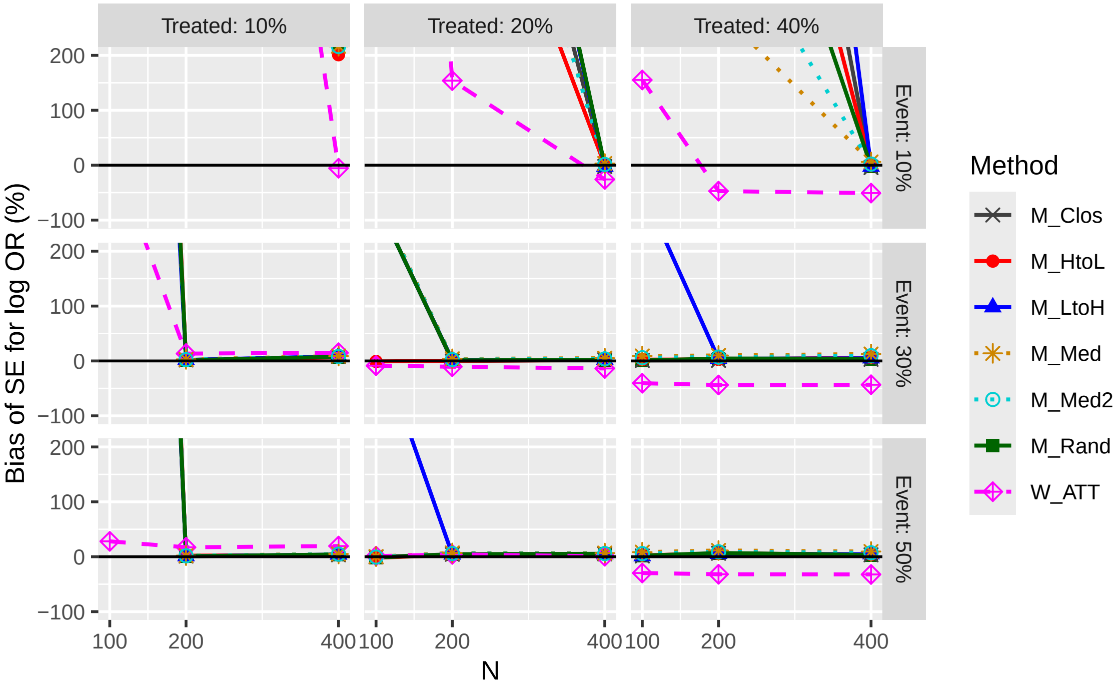

Figure S397. Mean bias of standard error for log odds ratio (unimodal continuous covariate, matching ratio 1:2, true OR: 1, c statistic: 0.85, naive inference).

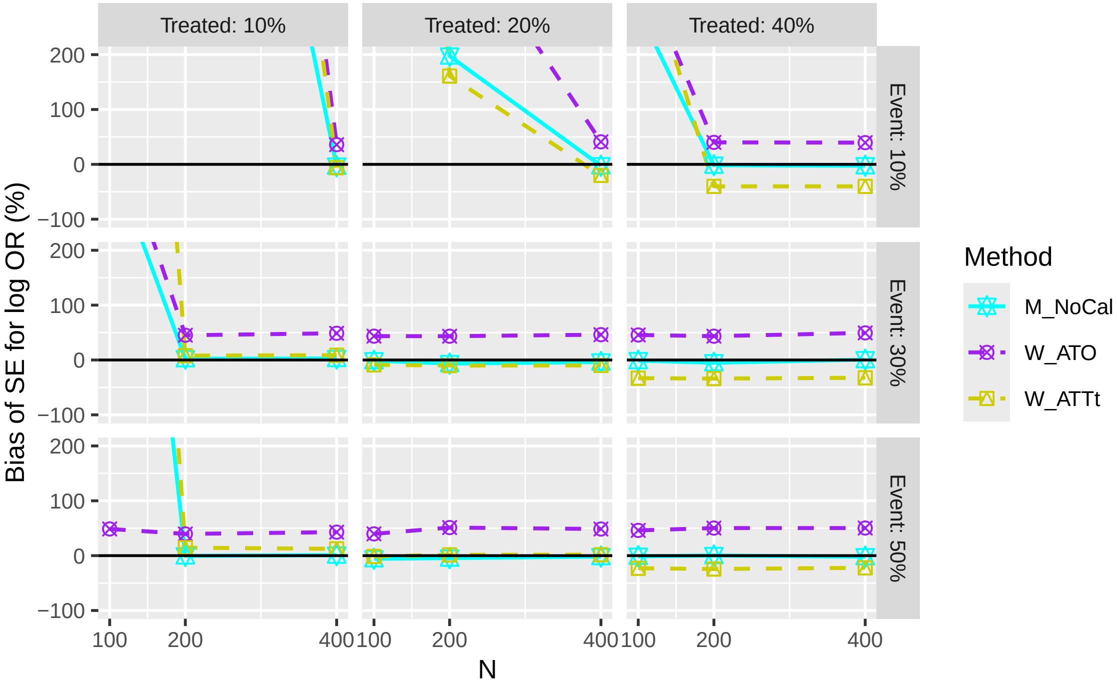

Figure S398. Mean bias of standard error for log odds ratio (unimodal continuous covariate, matching ratio 1:2, true OR: 1, c statistic: 0.85, naive inference); other methods.

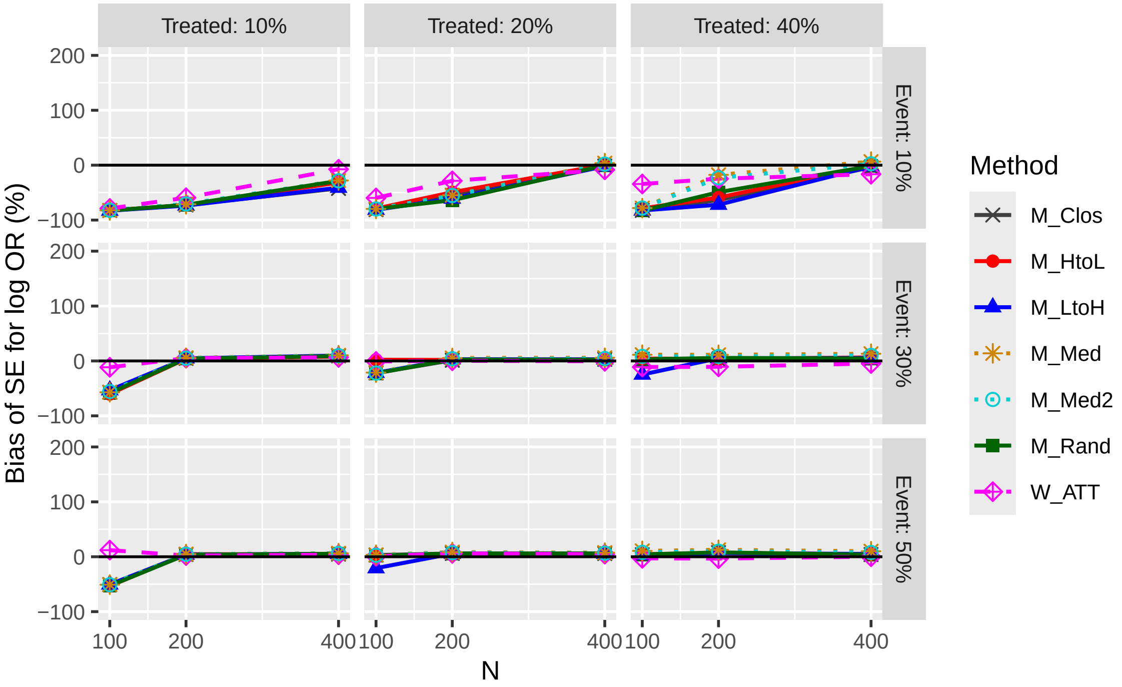

Figure S399. Mean bias of standard error for log odds ratio (unimodal continuous covariate, matching ratio 1:2, true OR: 1, c statistic: 0.85, robust inference).

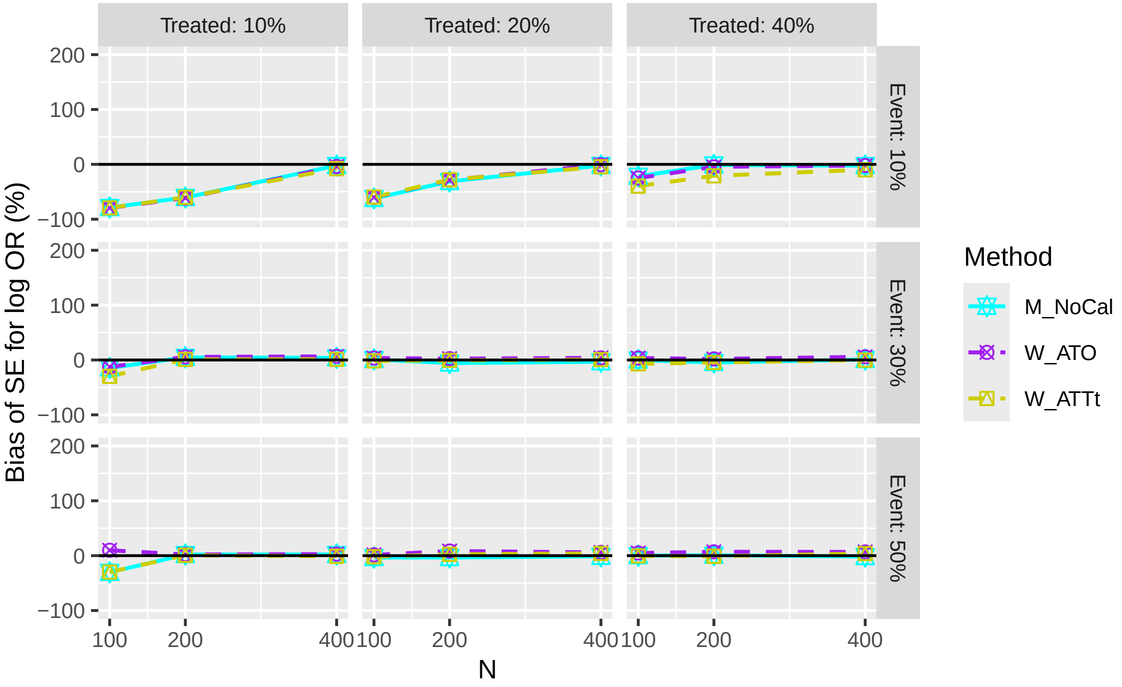

Figure S400. Mean bias of standard error for log odds ratio (unimodal continuous covariate, matching ratio 1:2, true OR: 1, c statistic: 0.85, robust inference); other methods.

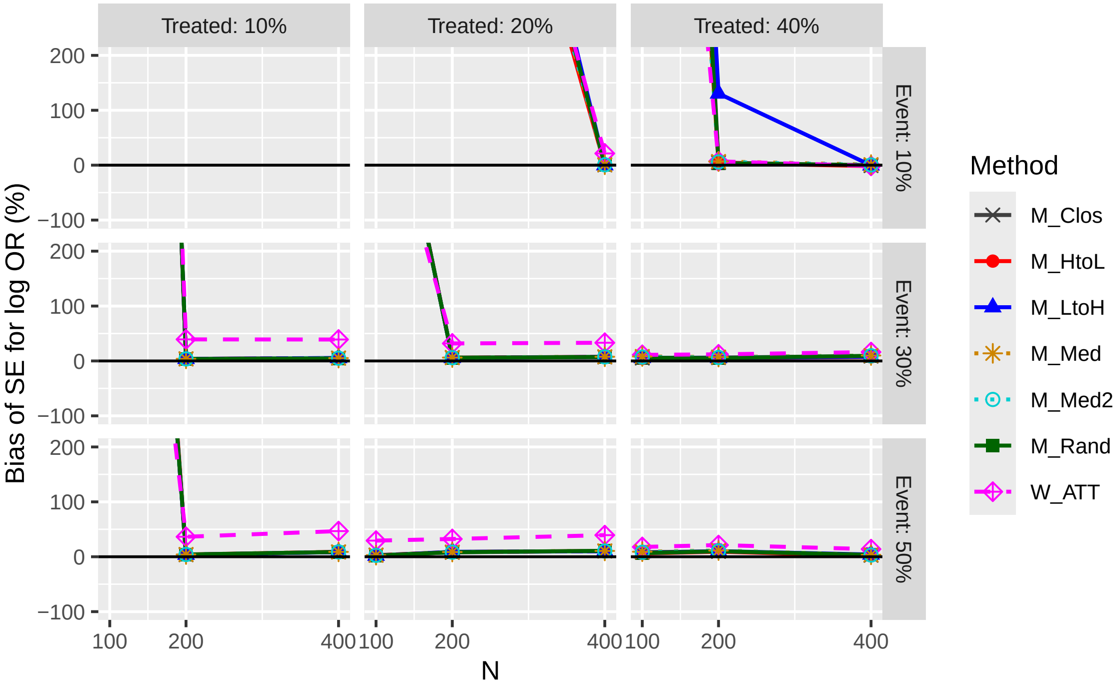

Figure S401. Mean bias of standard error for log odds ratio (unimodal continuous covariate, matching ratio 1:2, true OR: 1, c statistic: 0.6, naive inference).

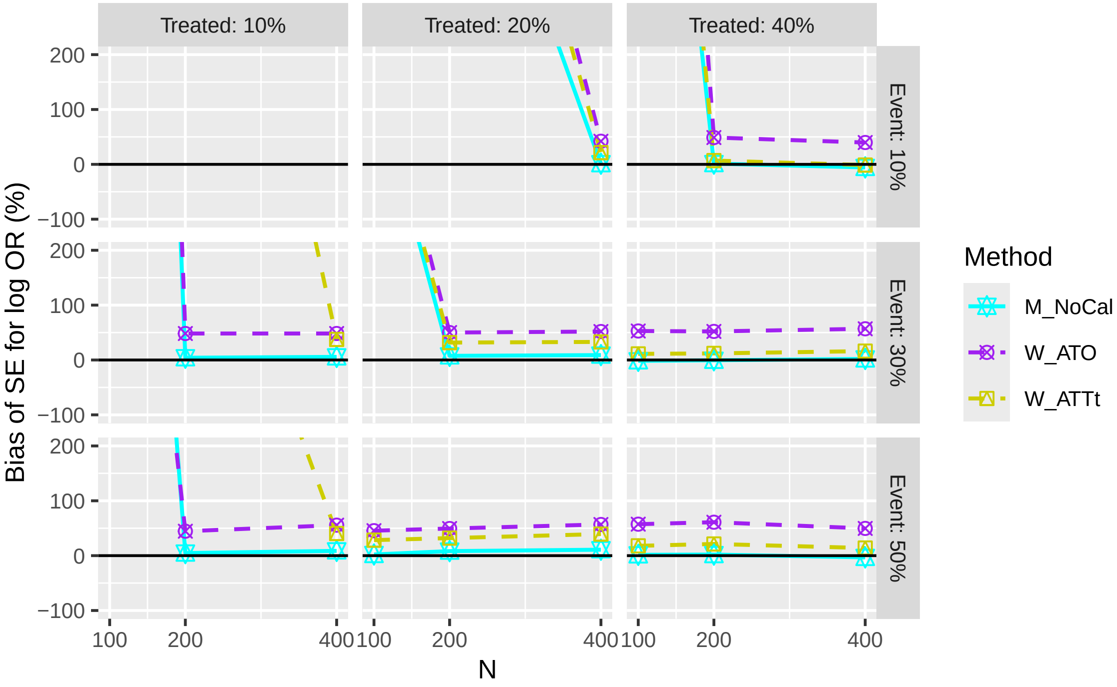

Figure S402. Mean bias of standard error for log odds ratio (unimodal continuous covariate, matching ratio 1:2, true OR: 1, c statistic: 0.6, naive inference); other methods.

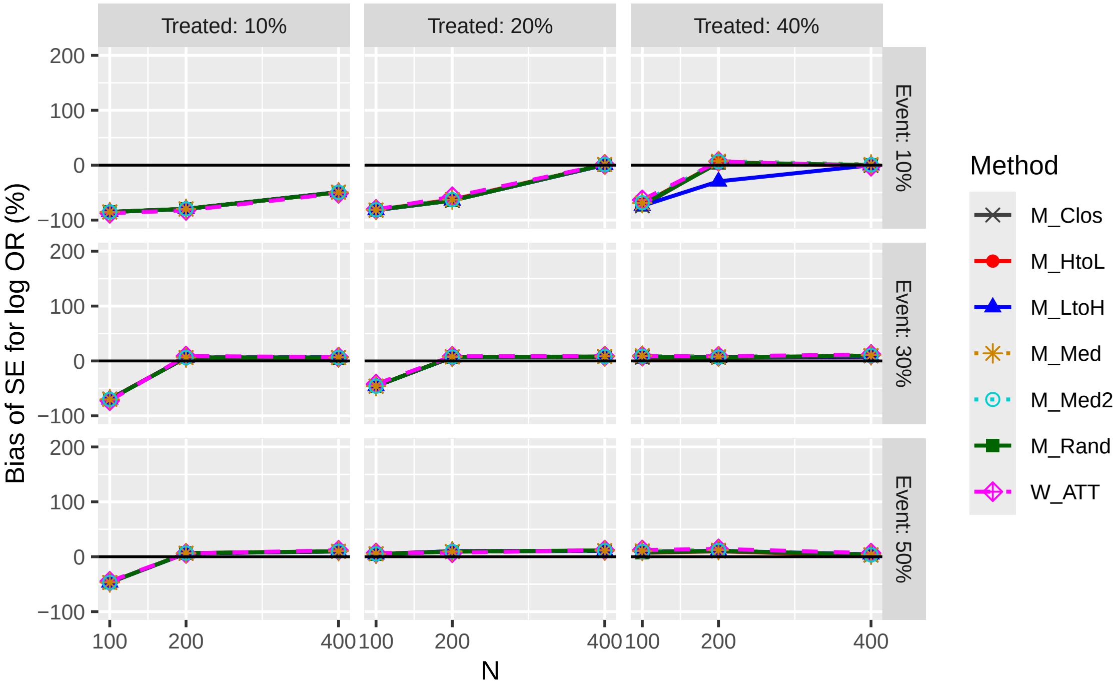

Figure S403. Mean bias of standard error for log odds ratio (unimodal continuous covariate, matching ratio 1:2, true OR: 1, c statistic: 0.6, robust inference).

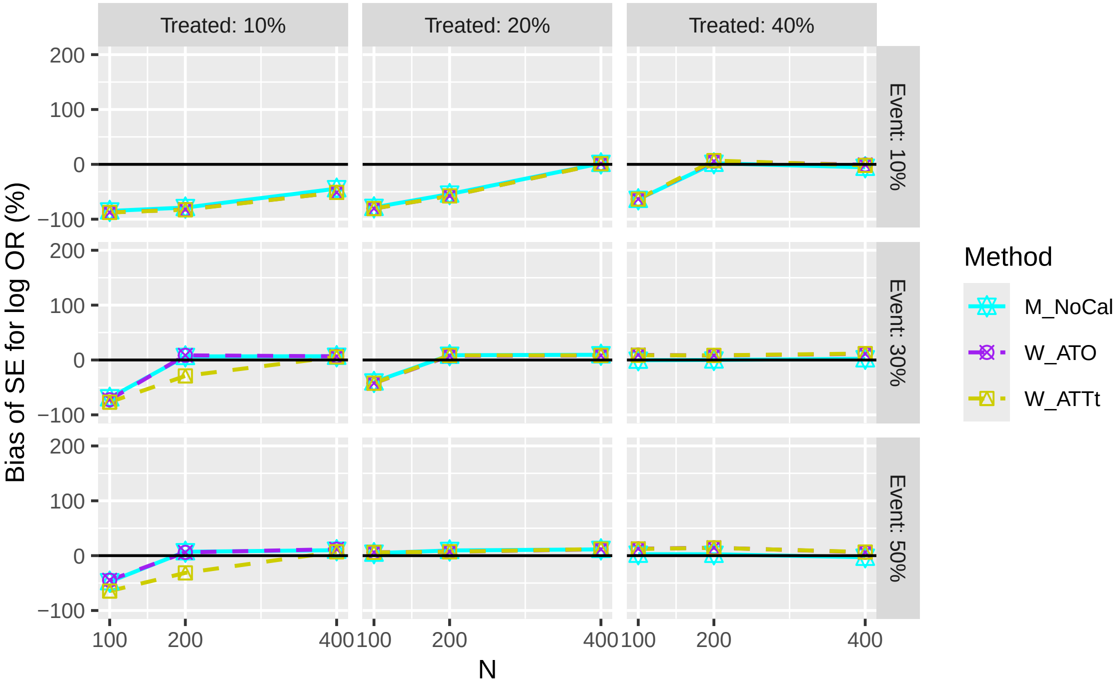

Figure S404. Mean bias of standard error for log odds ratio (unimodal continuous covariate, matching ratio 1:2, true OR: 1, c statistic: 0.6, robust inference); other methods.

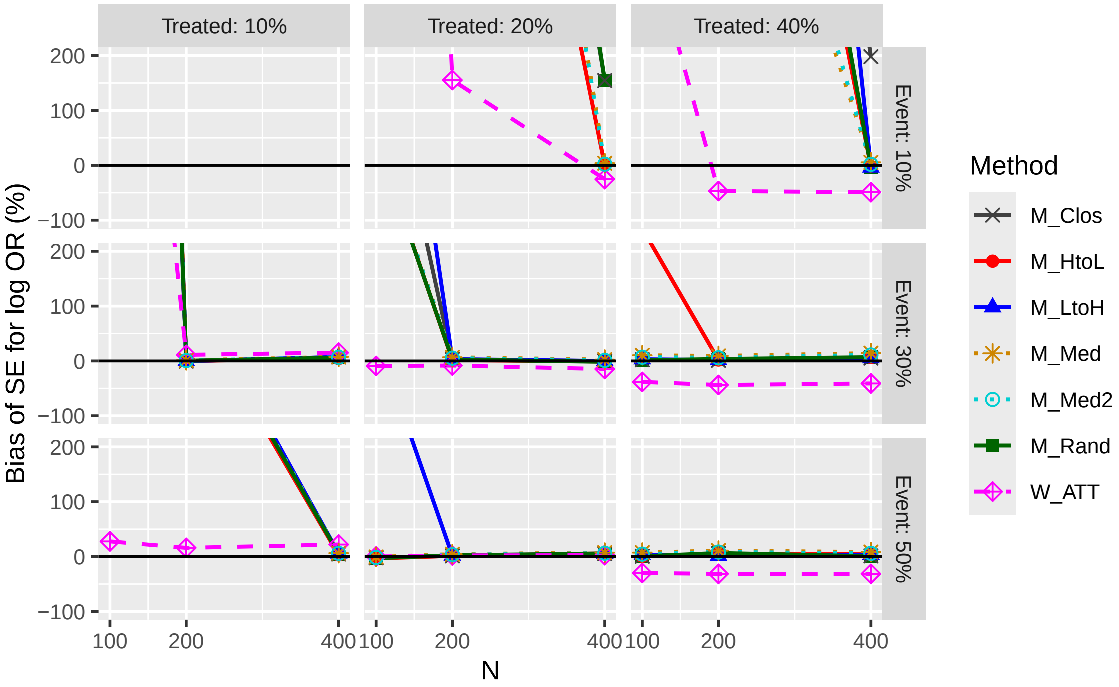

Figure S405. Mean bias of standard error for log odds ratio (unimodal continuous covariate, matching ratio 1:2, true OR: 0.75, c statistic: 0.85, naive inference).

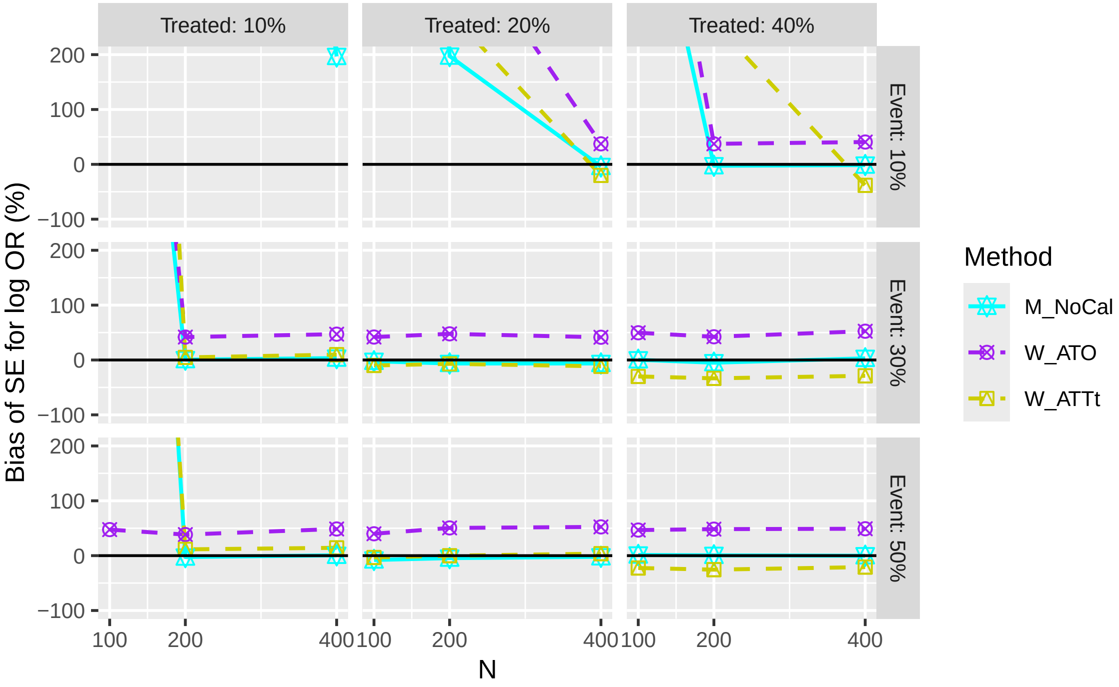

Figure S406. Mean bias of standard error for log odds ratio (unimodal continuous covariate, matching ratio 1:2, true OR: 0.75, c statistic: 0.85, naive inference); other methods.

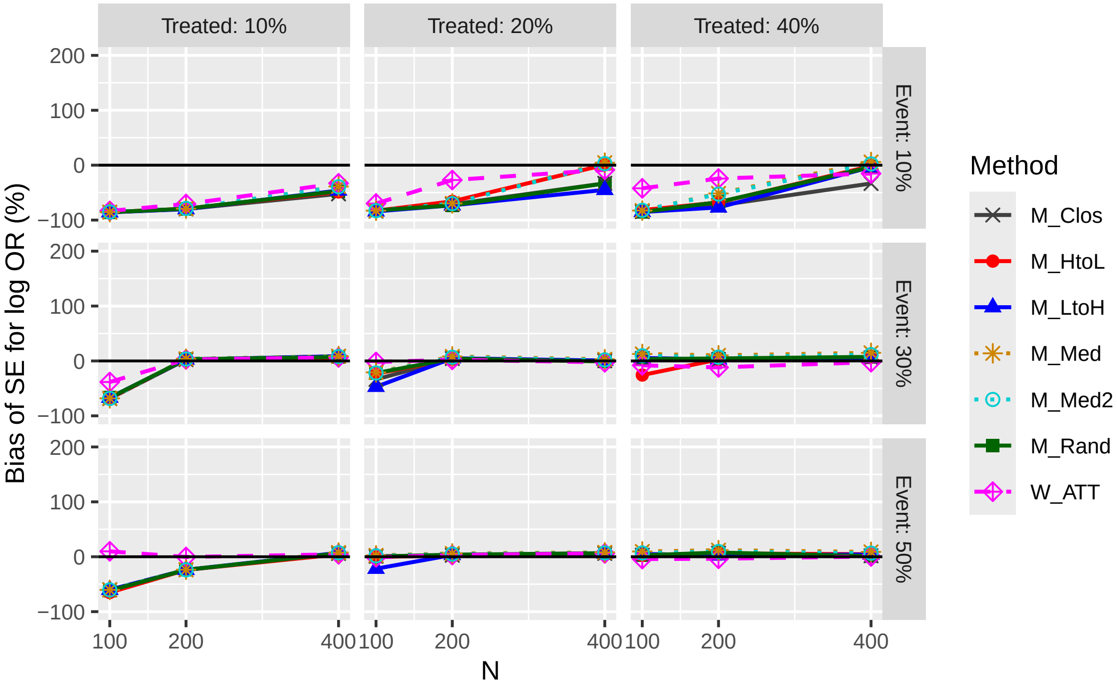

Figure S407. Mean bias of standard error for log odds ratio (unimodal continuous covariate, matching ratio 1:2, true OR: 0.75, c statistic: 0.85, robust inference).

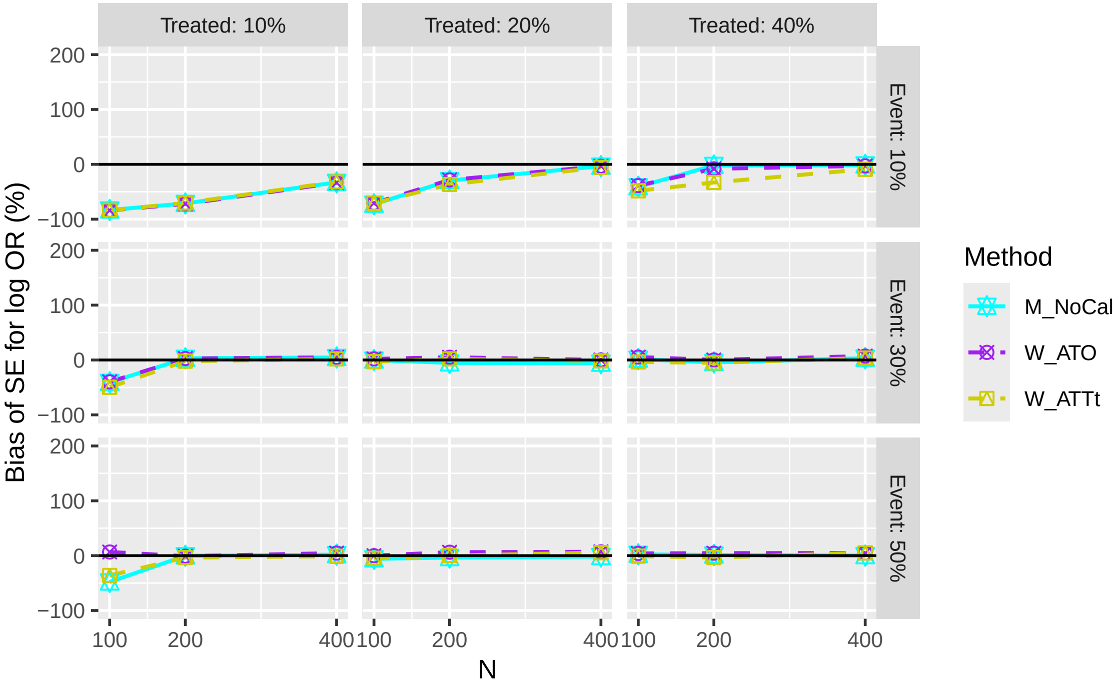

Figure S408. Mean bias of standard error for log odds ratio (unimodal continuous covariate, matching ratio 1:2, true OR: 0.75, c statistic: 0.85, robust inference); other methods.

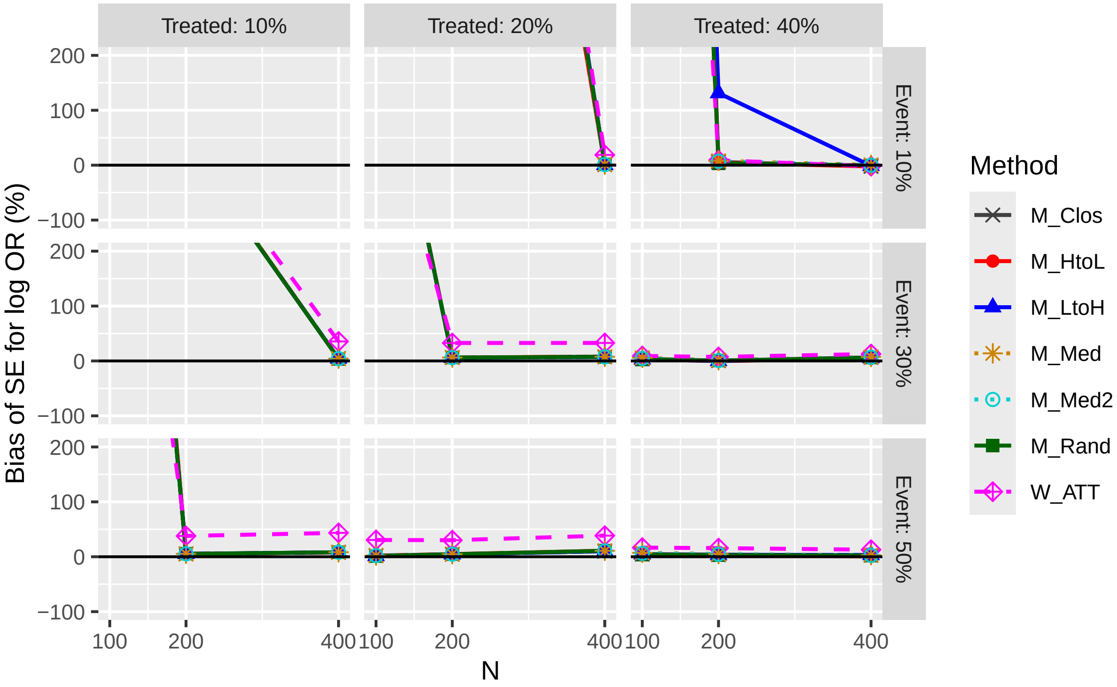

Figure S409. Mean bias of standard error for log odds ratio (unimodal continuous covariate, matching ratio 1:2, true OR: 0.75, c statistic: 0.6, naive inference).

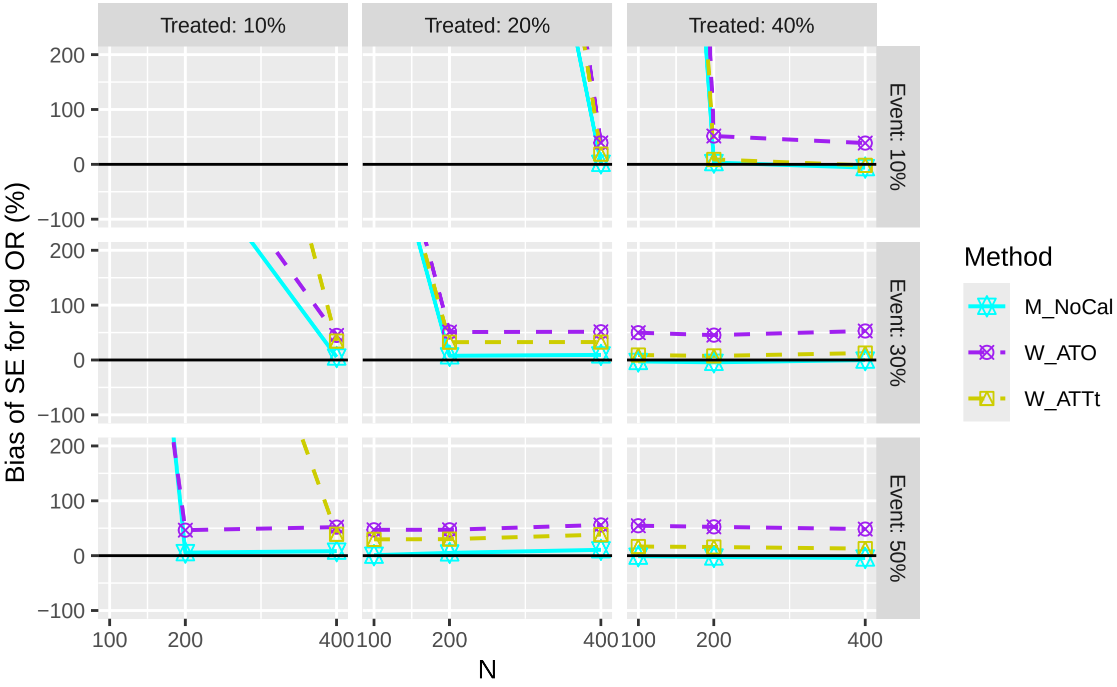

Figure S410. Mean bias of standard error for log odds ratio (unimodal continuous covariate, matching ratio 1:2, true OR: 0.75, c statistic: 0.6, naive inference); other methods.

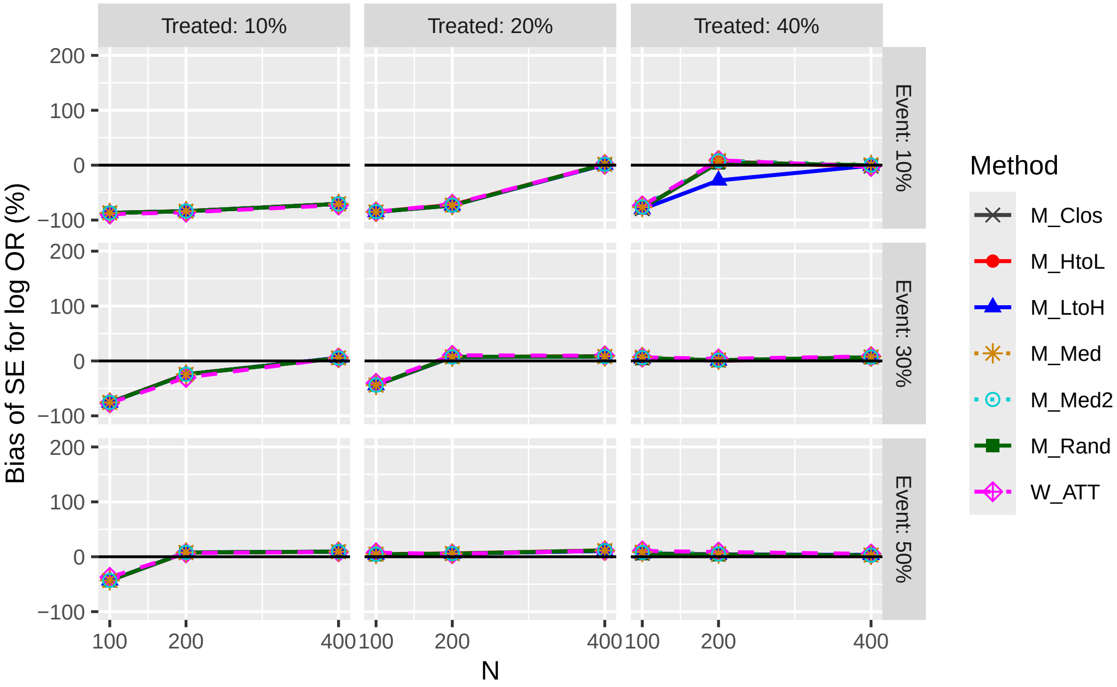

Figure S411. Mean bias of standard error for log odds ratio (unimodal continuous covariate, matching ratio 1:2, true OR: 0.75, c statistic: 0.6, robust inference).

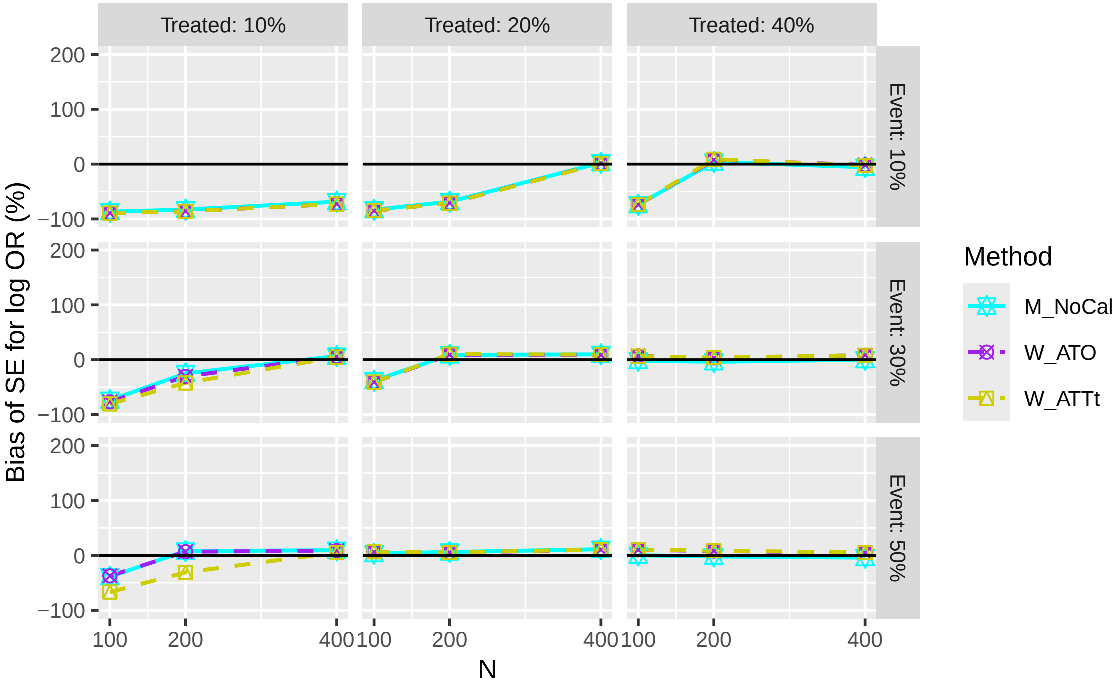

Figure S412. Mean bias of standard error for log odds ratio (unimodal continuous covariate, matching ratio 1:2, true OR: 0.75, c statistic: 0.6, robust inference); other methods.

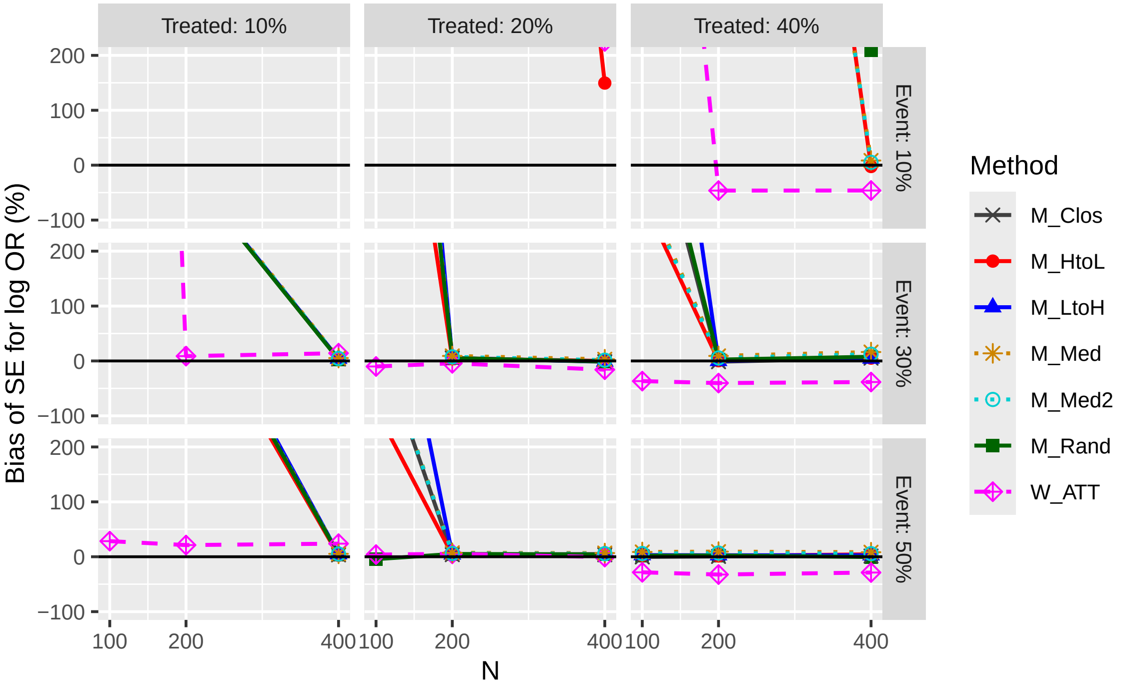

Figure S413. Mean bias of standard error for log odds ratio (unimodal continuous covariate, matching ratio 1:2, true OR: 0.5, c statistic: 0.85, naive inference).

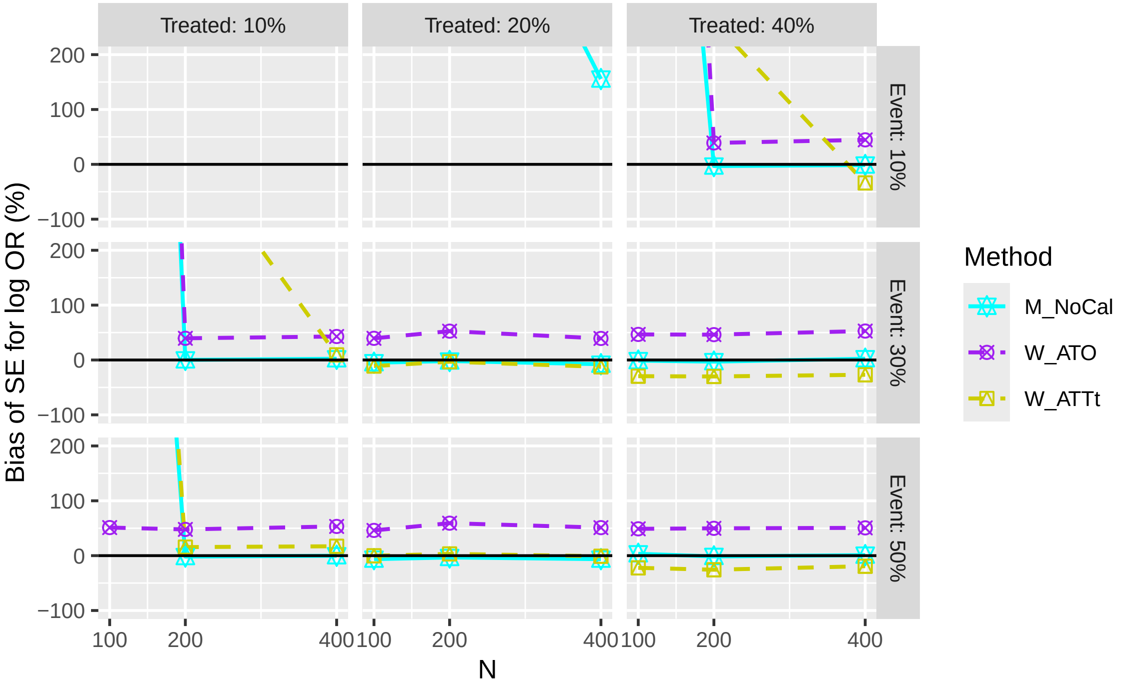

Figure S414. Mean bias of standard error for log odds ratio (unimodal continuous covariate, matching ratio 1:2, true OR: 0.5, c statistic: 0.85, naive inference); other methods.

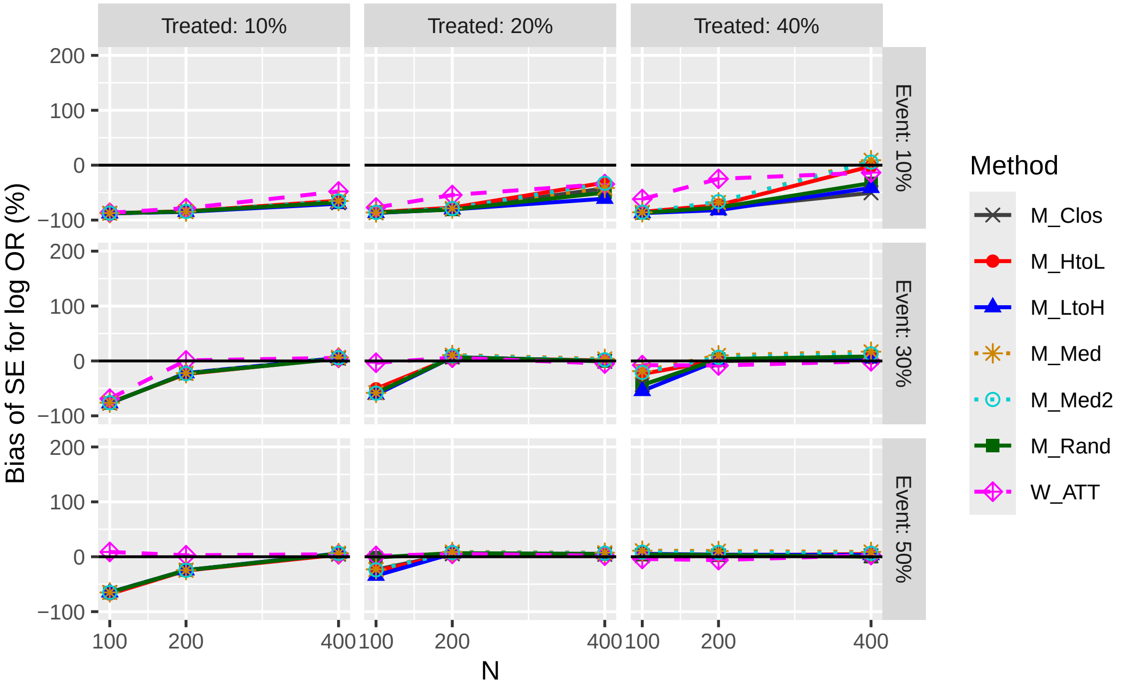

Figure S415. Mean bias of standard error for log odds ratio (unimodal continuous covariate, matching ratio 1:2, true OR: 0.5, c statistic: 0.85, robust inference).

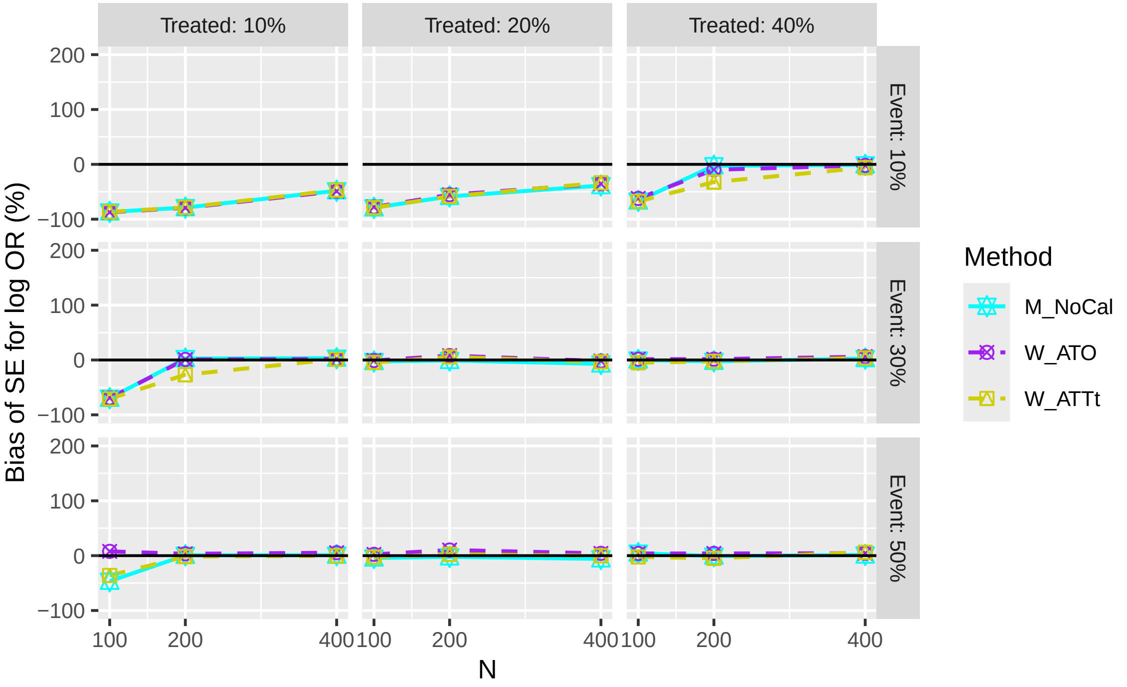

Figure S416. Mean bias of standard error for log odds ratio (unimodal continuous covariate, matching ratio 1:2, true OR: 0.5, c statistic: 0.85, robust inference); other methods.

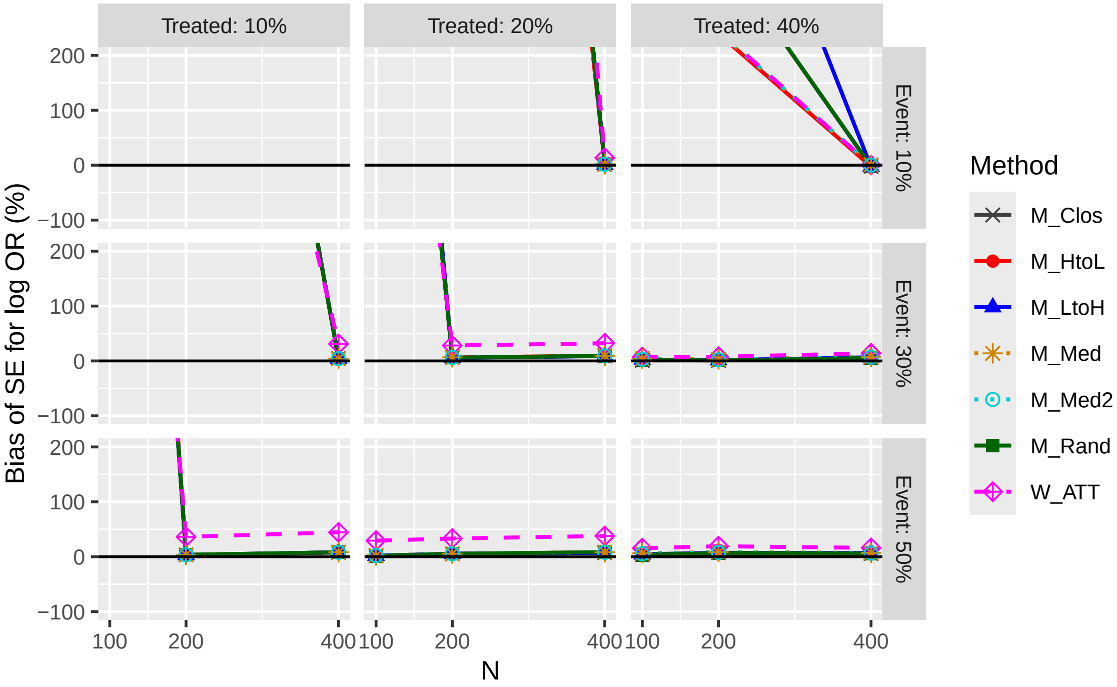

Figure S417. Mean bias of standard error for log odds ratio (unimodal continuous covariate, matching ratio 1:2, true OR: 0.5, c statistic: 0.6, naive inference).

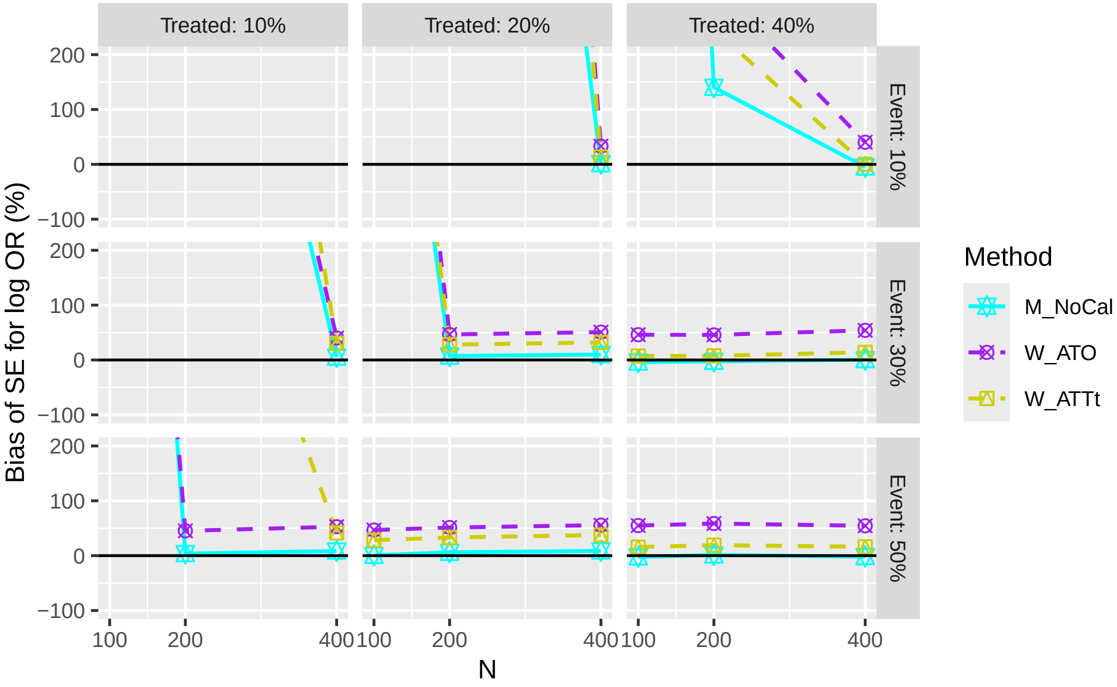

Figure S418. Mean bias of standard error for log odds ratio (unimodal continuous covariate, matching ratio 1:2, true OR: 0.5, c statistic: 0.6, naive inference); other methods.

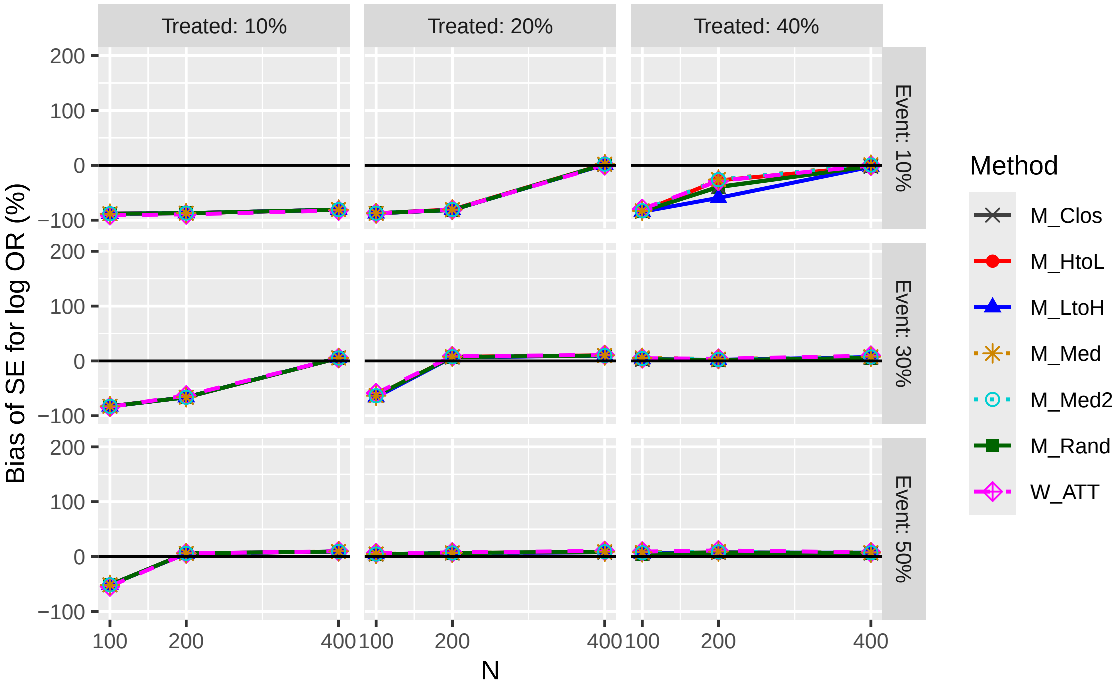

Figure S419. Mean bias of standard error for log odds ratio (unimodal continuous covariate, matching ratio 1:2, true OR: 0.5, c statistic: 0.6, robust inference).

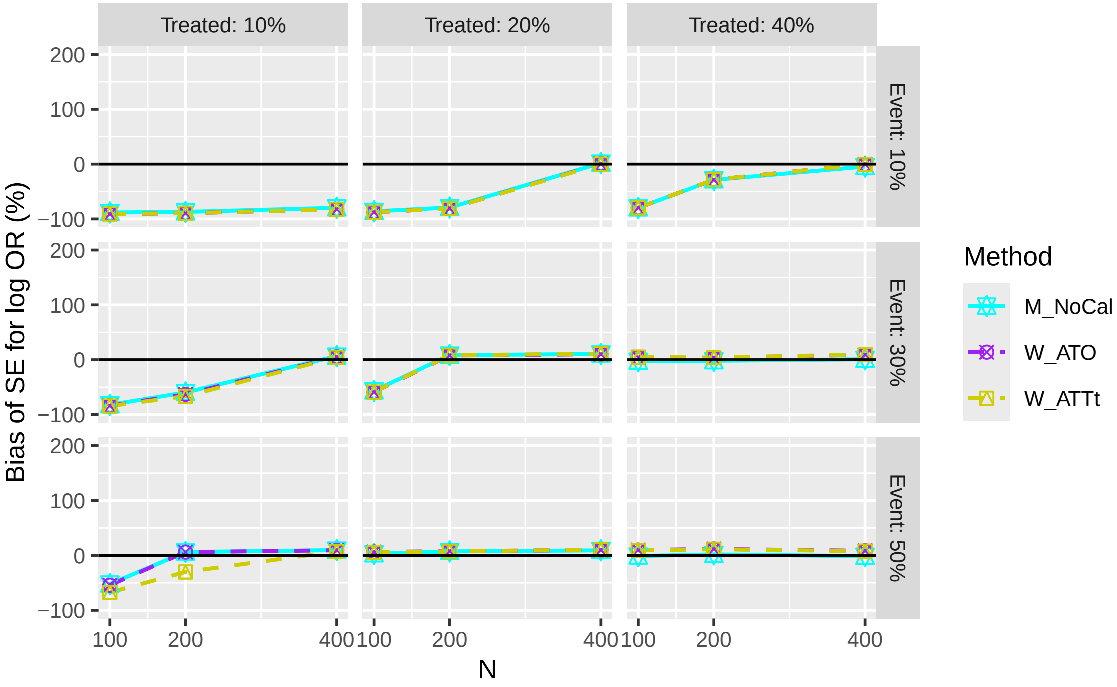

Figure S420. Mean bias of standard error for log odds ratio (unimodal continuous covariate, matching ratio 1:2, true OR: 0.5, c statistic: 0.6, robust inference); other methods.

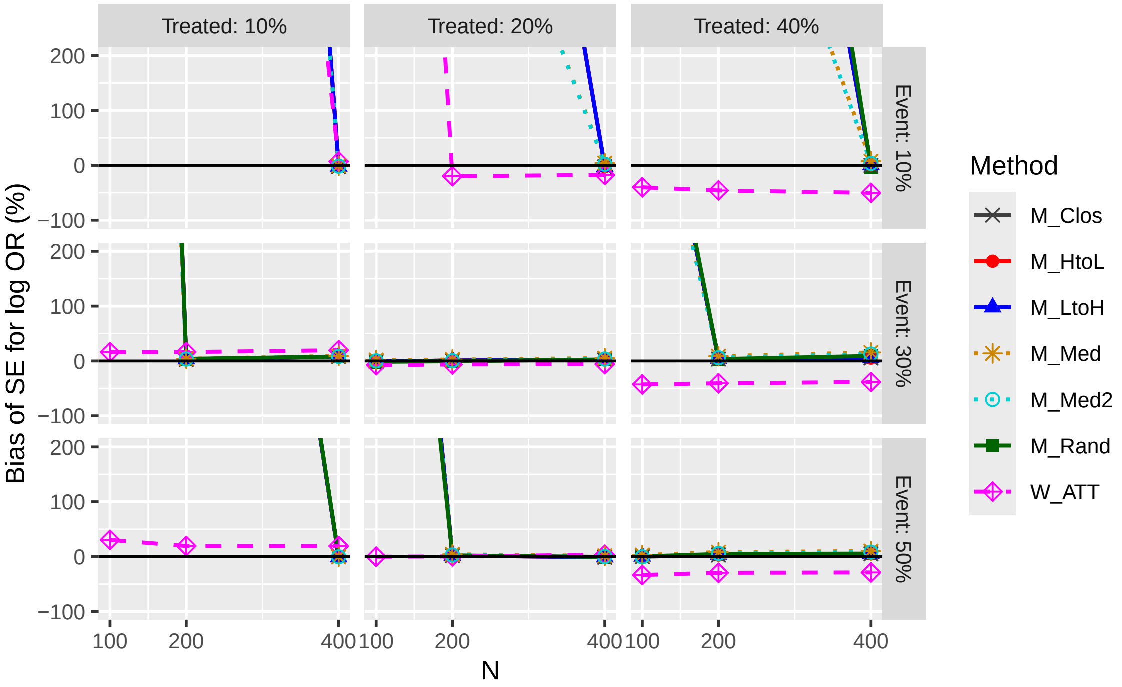

Figure S421. Mean bias of standard error for log odds ratio (categorical covariate, matching ratio 1:1, true OR: 1, c statistic: 0.85, naive inference).

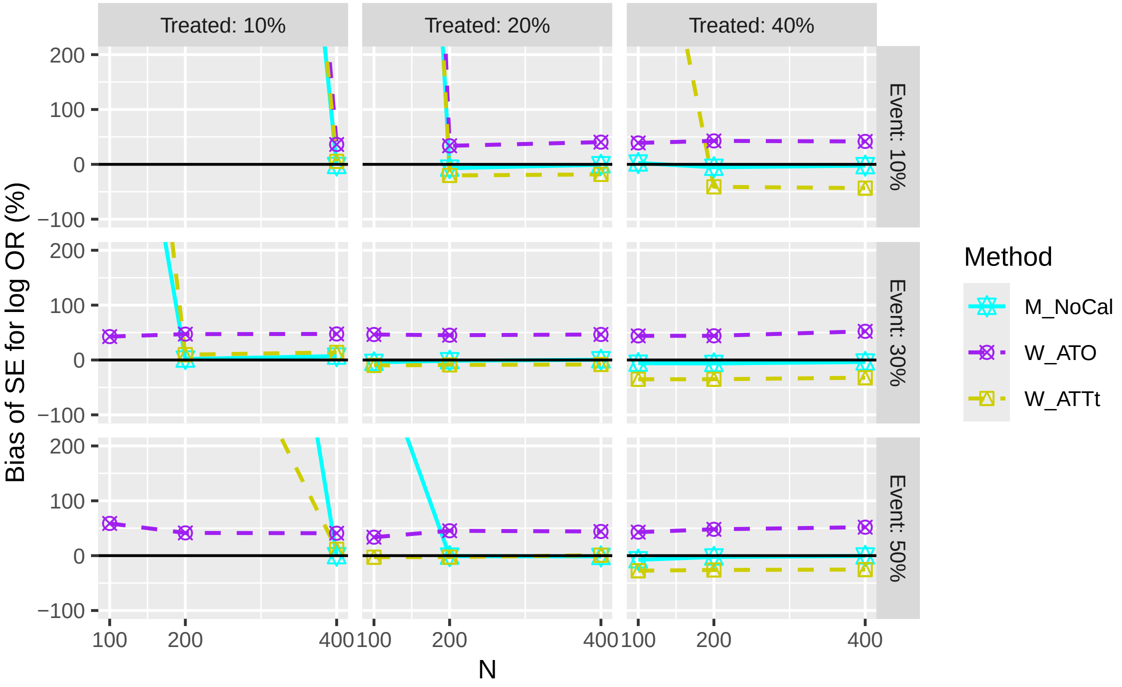

Figure S422. Mean bias of standard error for log odds ratio (categorical covariate, matching ratio 1:1, true OR: 1, c statistic: 0.85, naive inference); other methods.

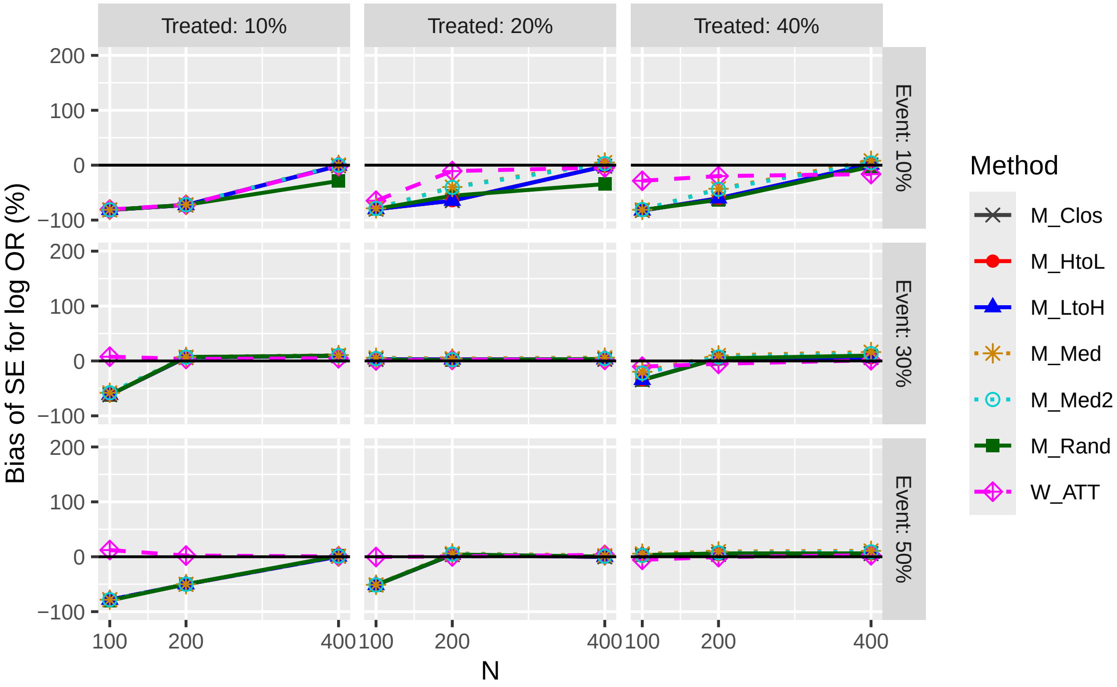

Figure S423. Mean bias of standard error for log odds ratio (categorical covariate, matching ratio 1:1, true OR: 1, c statistic: 0.85, robust inference).

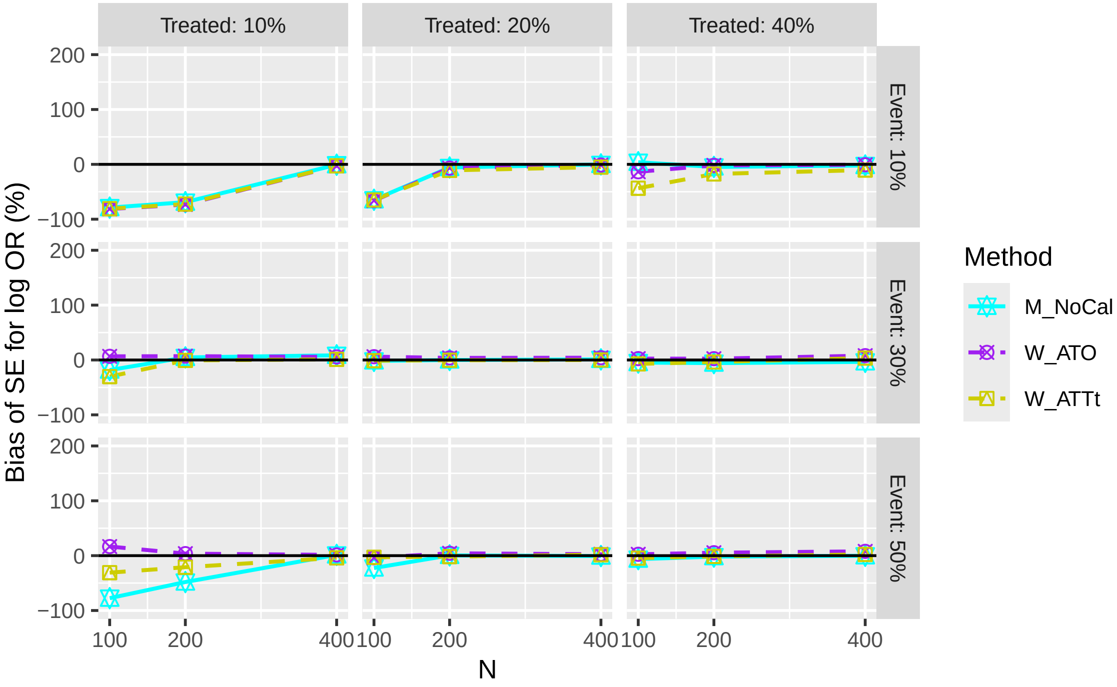

Figure S424. Mean bias of standard error for log odds ratio (categorical covariate, matching ratio 1:1, true OR: 1, c statistic: 0.85, robust inference); other methods.

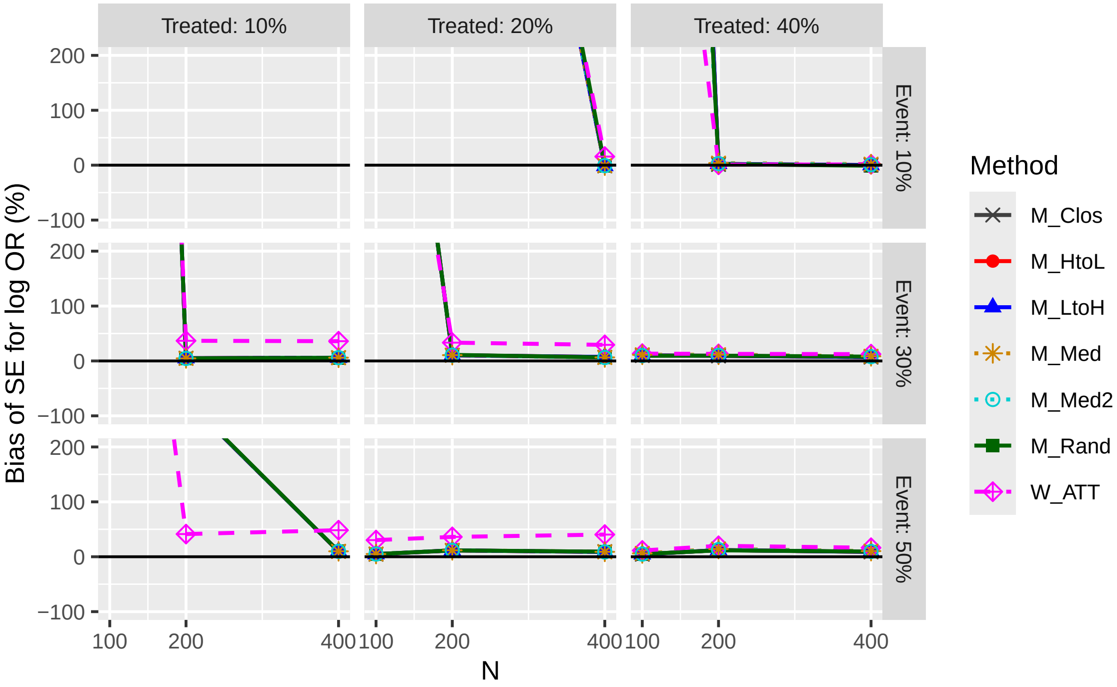

Figure S425. Mean bias of standard error for log odds ratio (categorical covariate, matching ratio 1:1, true OR: 1, c statistic: 0.6, naive inference).

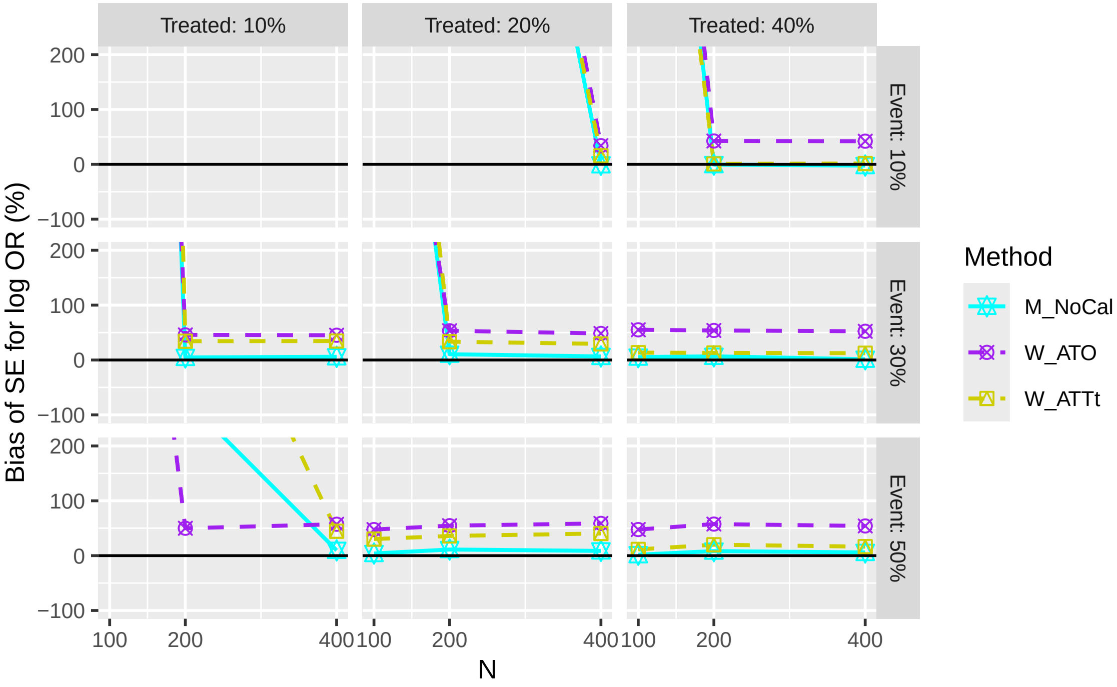

Figure S426. Mean bias of standard error for log odds ratio (categorical covariate, matching ratio 1:1, true OR: 1, c statistic: 0.6, naive inference); other methods.

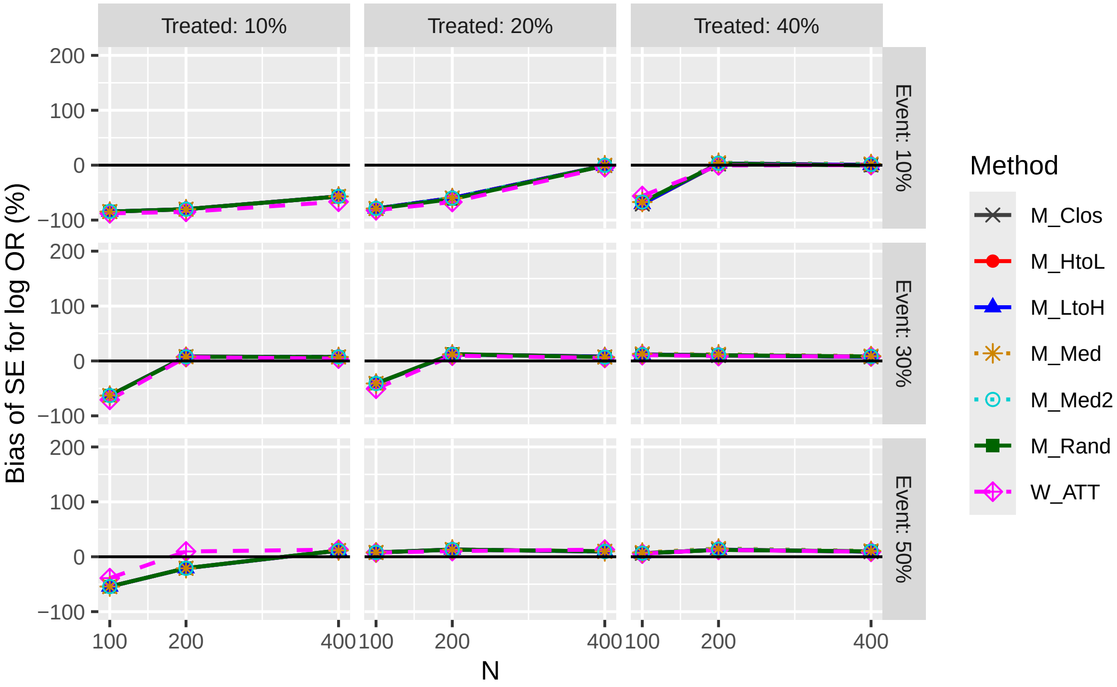

Figure S427. Mean bias of standard error for log odds ratio (categorical covariate, matching ratio 1:1, true OR: 1, c statistic: 0.6, robust inference).

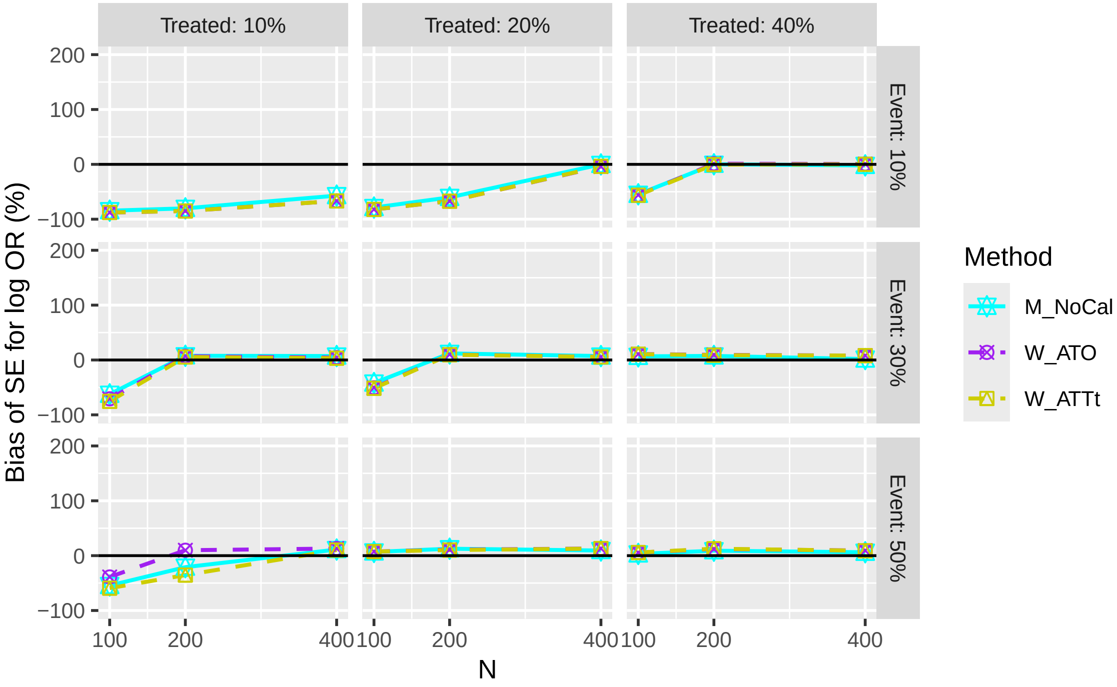

Figure S428. Mean bias of standard error for log odds ratio (categorical covariate, matching ratio 1:1, true OR: 1, c statistic: 0.6, robust inference); other methods.

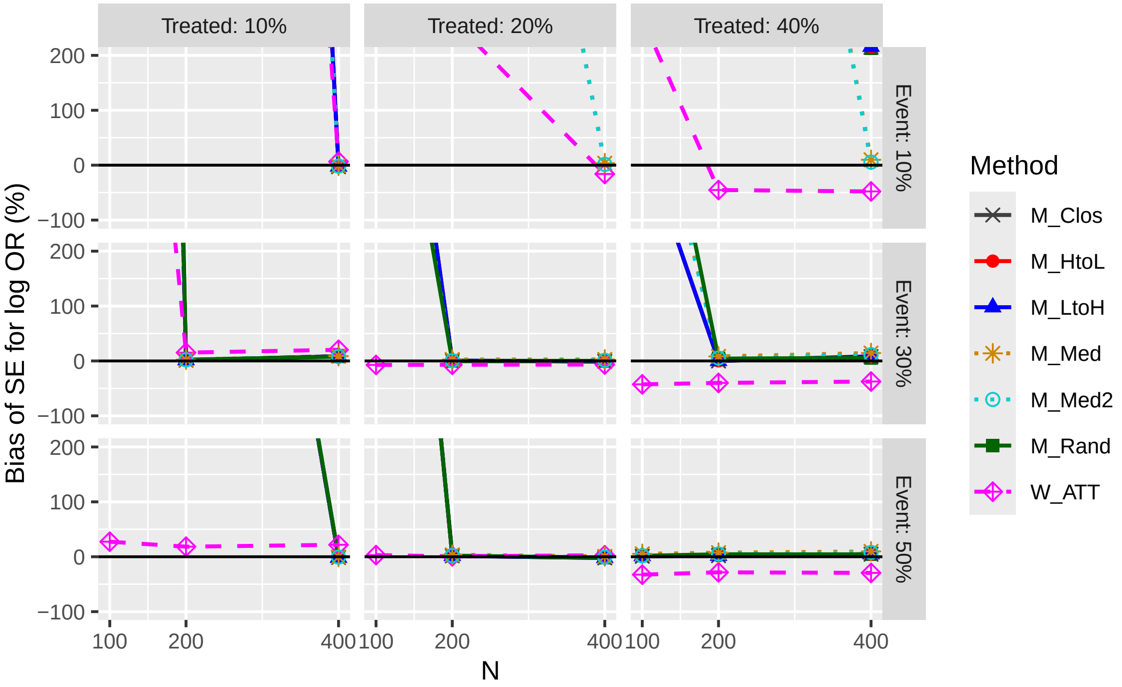

Figure S429. Mean bias of standard error for log odds ratio (categorical covariate, matching ratio 1:1, true OR: 0.75, c statistic: 0.85, naive inference).

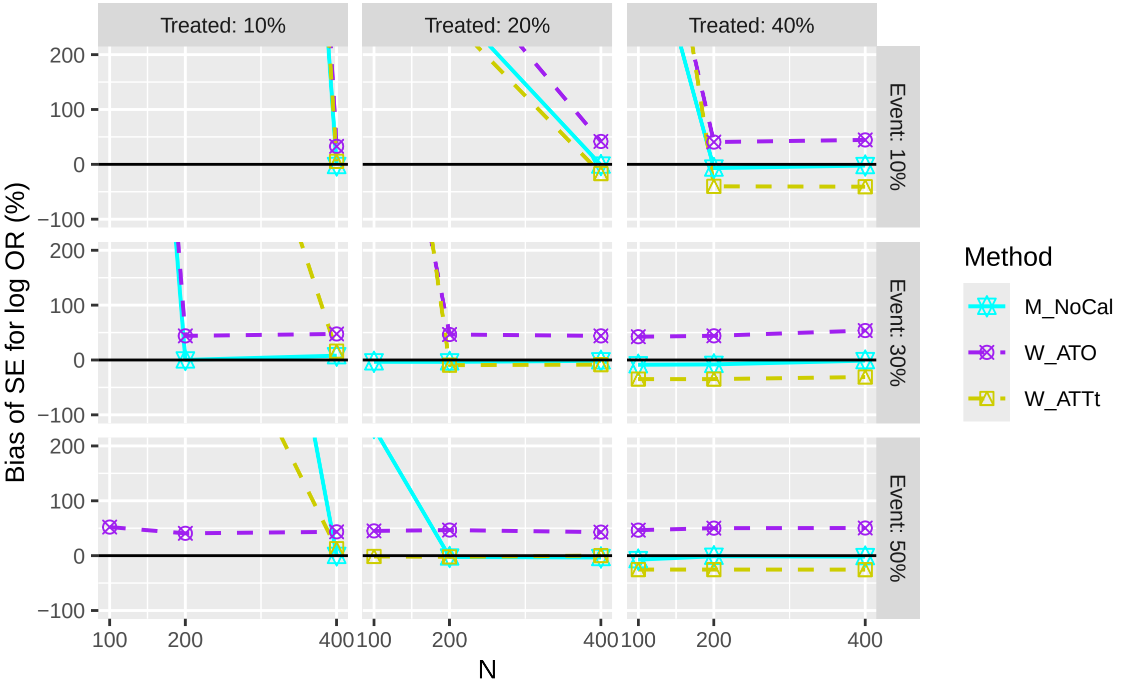

Figure S430. Mean bias of standard error for log odds ratio (categorical covariate, matching ratio 1:1, true OR: 0.75, c statistic: 0.85, naive inference); other methods.

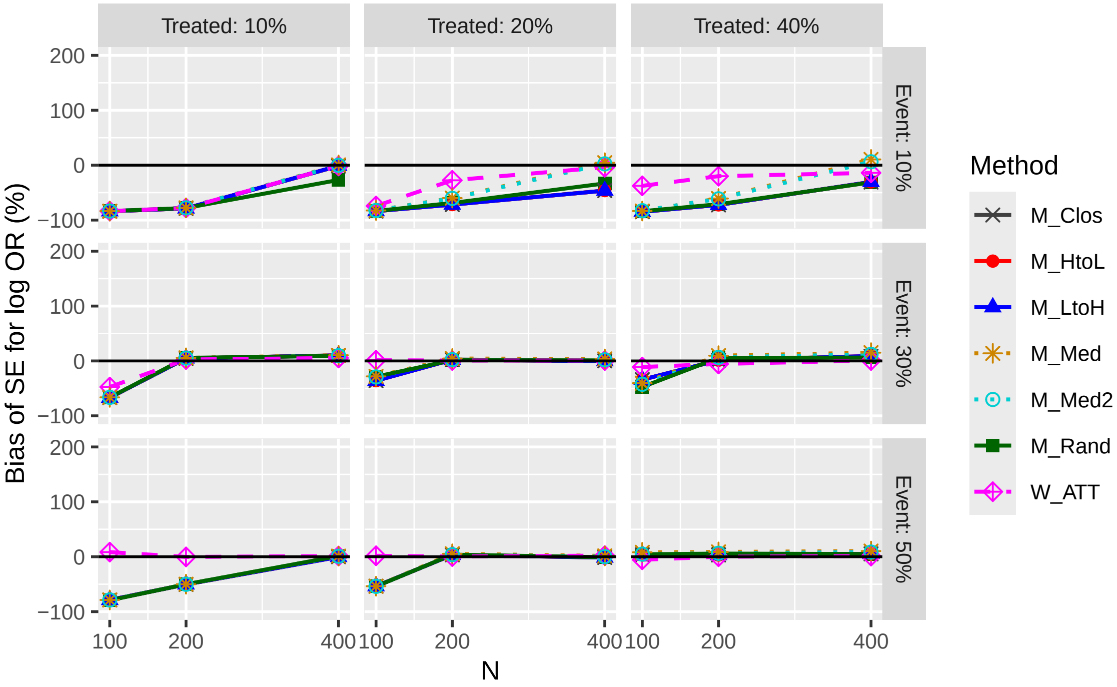

Figure S431. Mean bias of standard error for log odds ratio (categorical covariate, matching ratio 1:1, true OR: 0.75, c statistic: 0.85, robust inference).

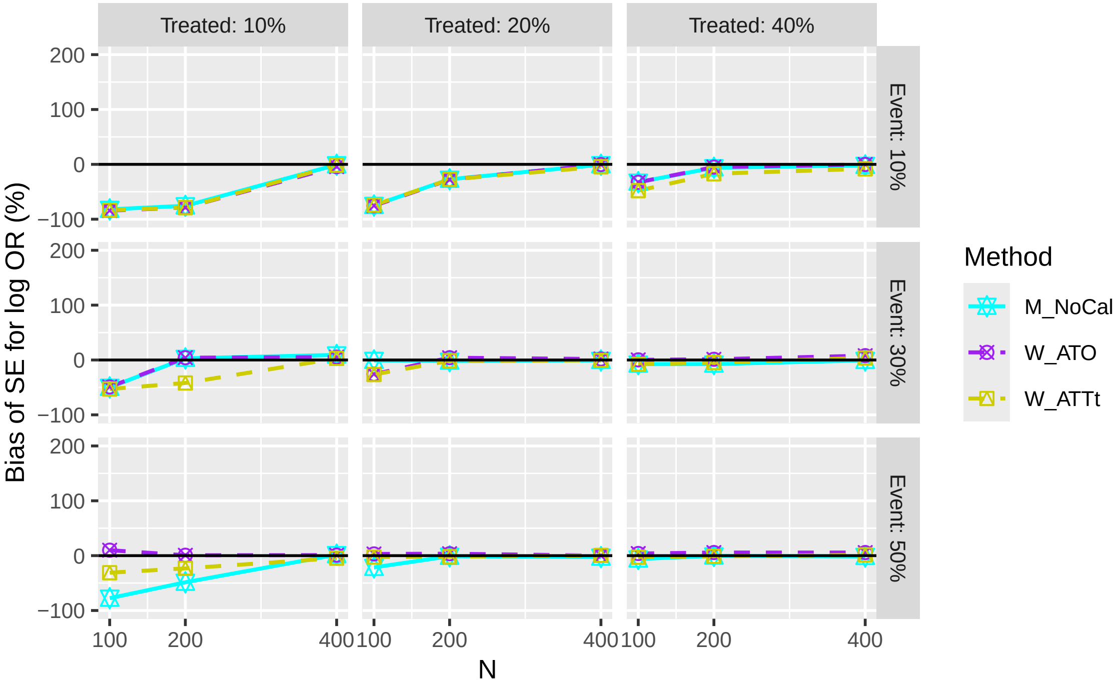

Figure S432. Mean bias of standard error for log odds ratio (categorical covariate, matching ratio 1:1, true OR: 0.75, c statistic: 0.85, robust inference); other methods.

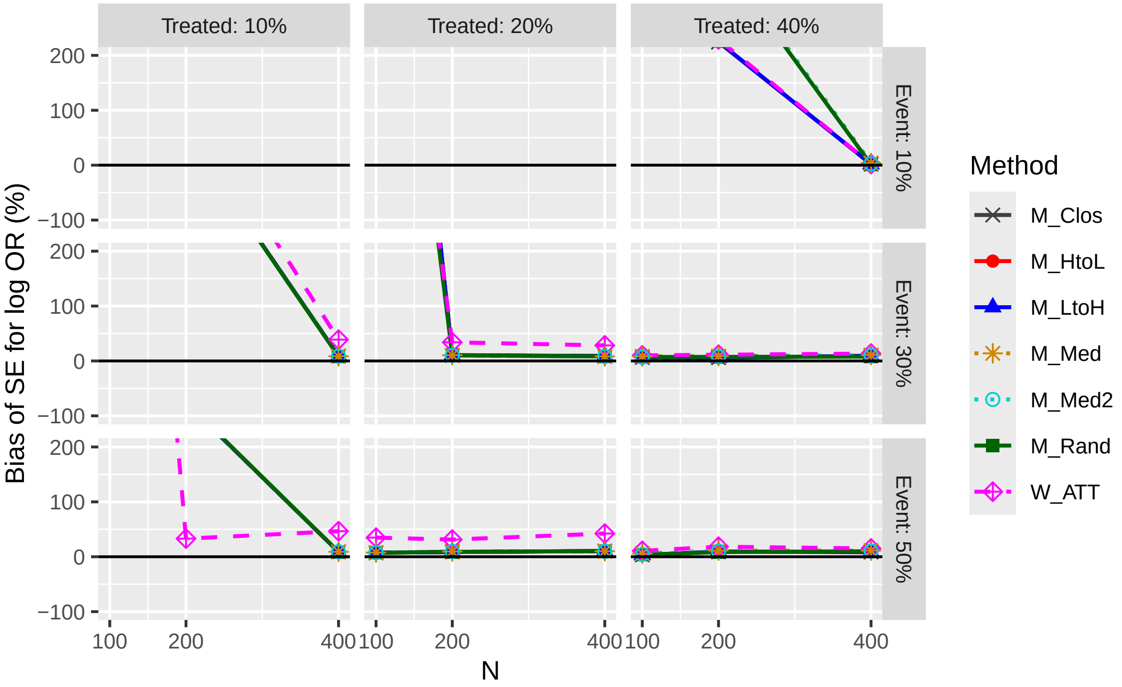

Figure S433. Mean bias of standard error for log odds ratio (categorical covariate, matching ratio 1:1, true OR: 0.75, c statistic: 0.6, naive inference).

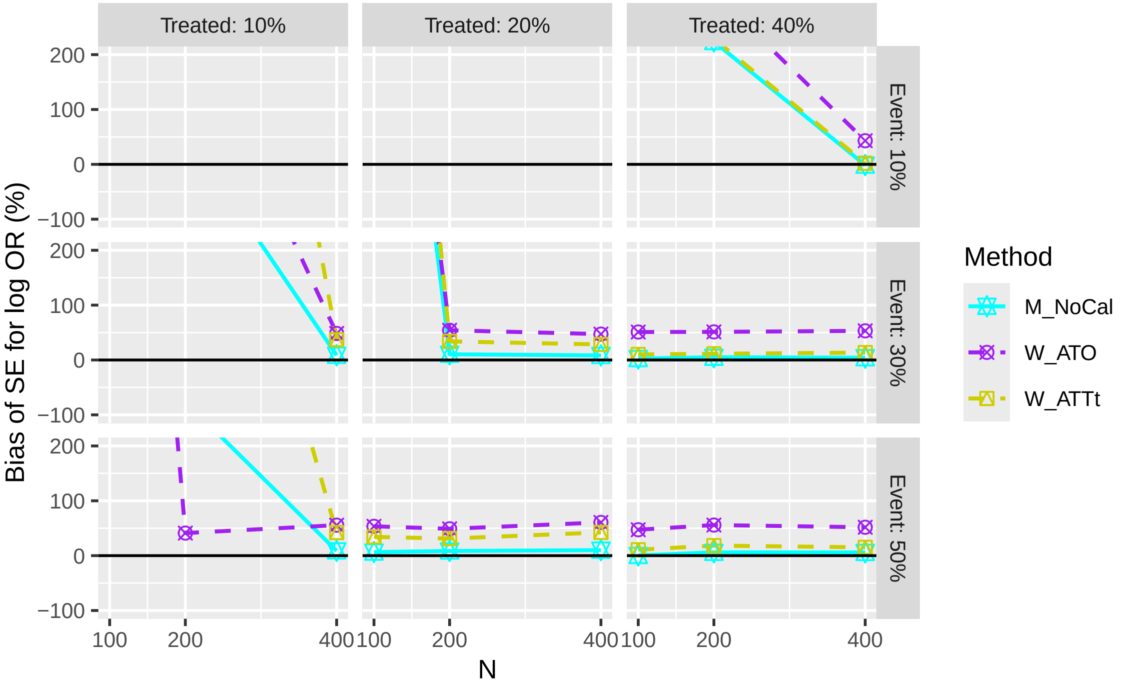

Figure S434. Mean bias of standard error for log odds ratio (categorical covariate, matching ratio 1:1, true OR: 0.75, c statistic: 0.6, naive inference); other methods.

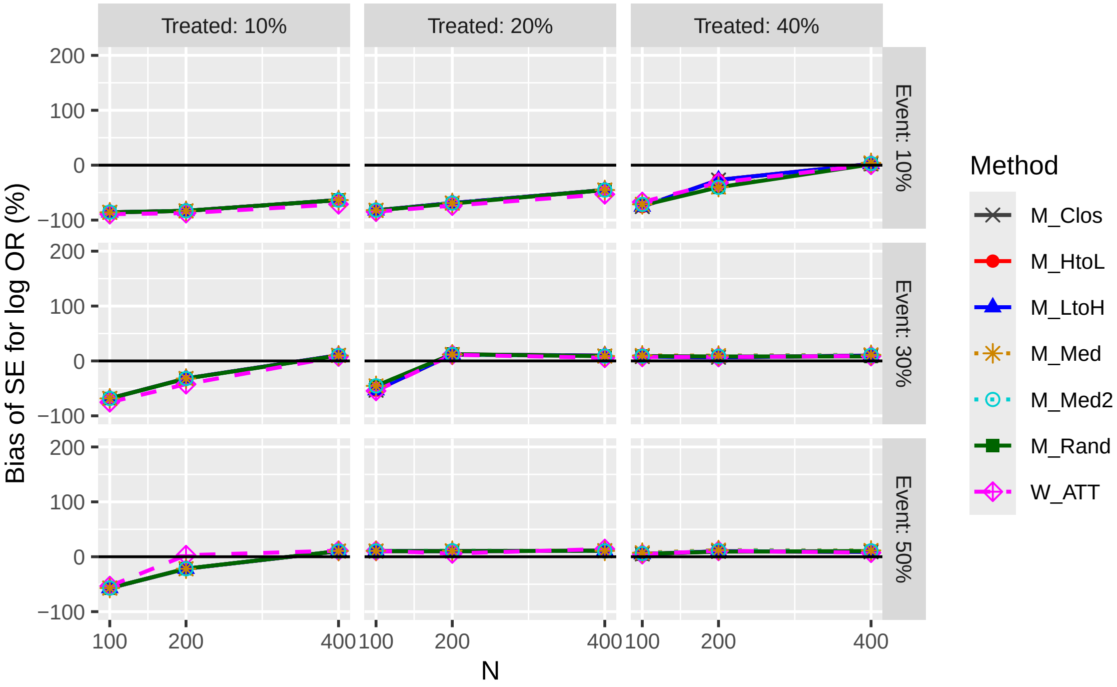

Figure S435. Mean bias of standard error for log odds ratio (categorical covariate, matching ratio 1:1, true OR: 0.75, c statistic: 0.6, robust inference).

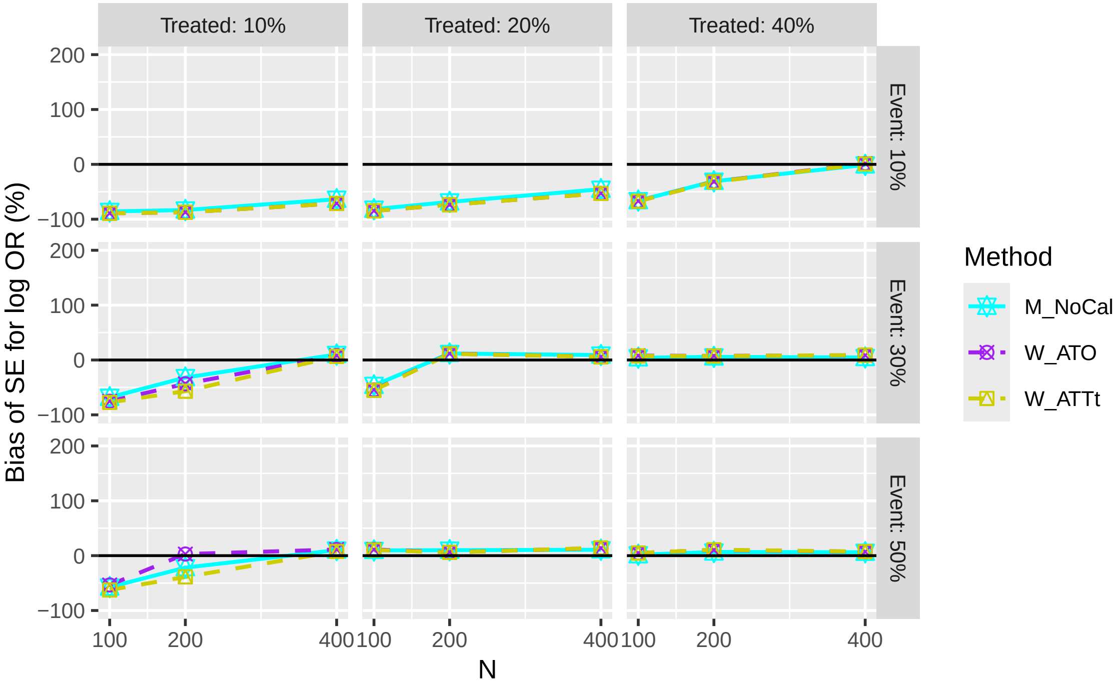

Figure S436. Mean bias of standard error for log odds ratio (categorical covariate, matching ratio 1:1, true OR: 0.75, c statistic: 0.6, robust inference); other methods.

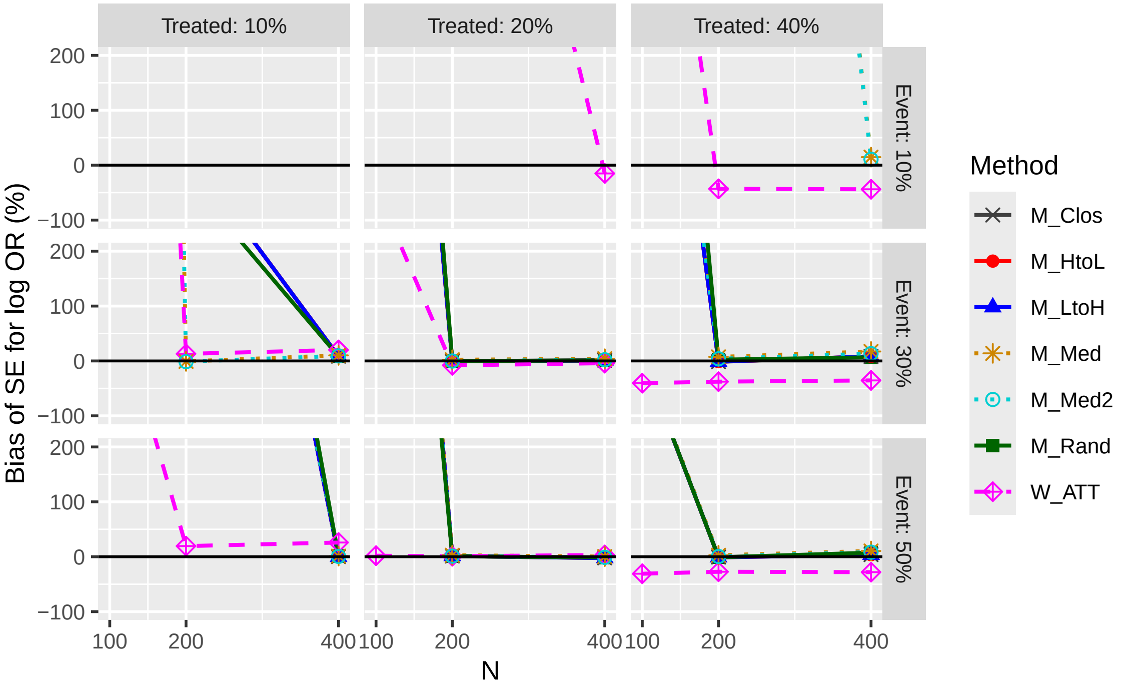

Figure S437. Mean bias of standard error for log odds ratio (categorical covariate, matching ratio 1:1, true OR: 0.5, c statistic: 0.85, naive inference).

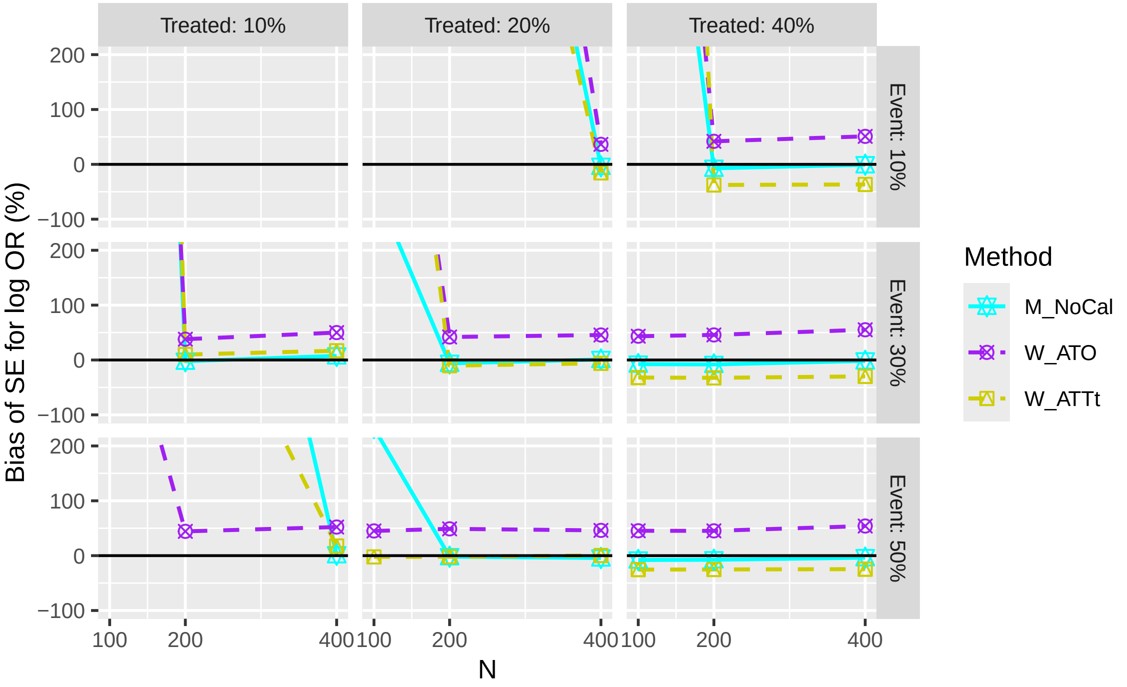

Figure S438. Mean bias of standard error for log odds ratio (categorical covariate, matching ratio 1:1, true OR: 0.5, c statistic: 0.85, naive inference); other methods.

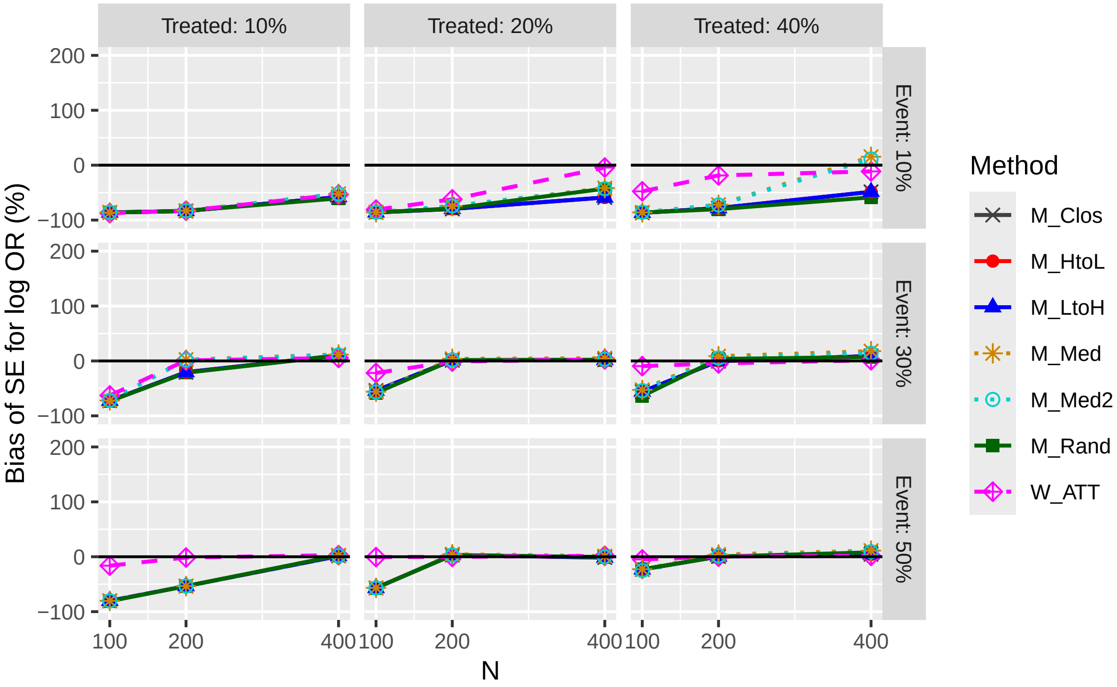

Figure S439. Mean bias of standard error for log odds ratio (categorical covariate, matching ratio 1:1, true OR: 0.5, c statistic: 0.85, robust inference).

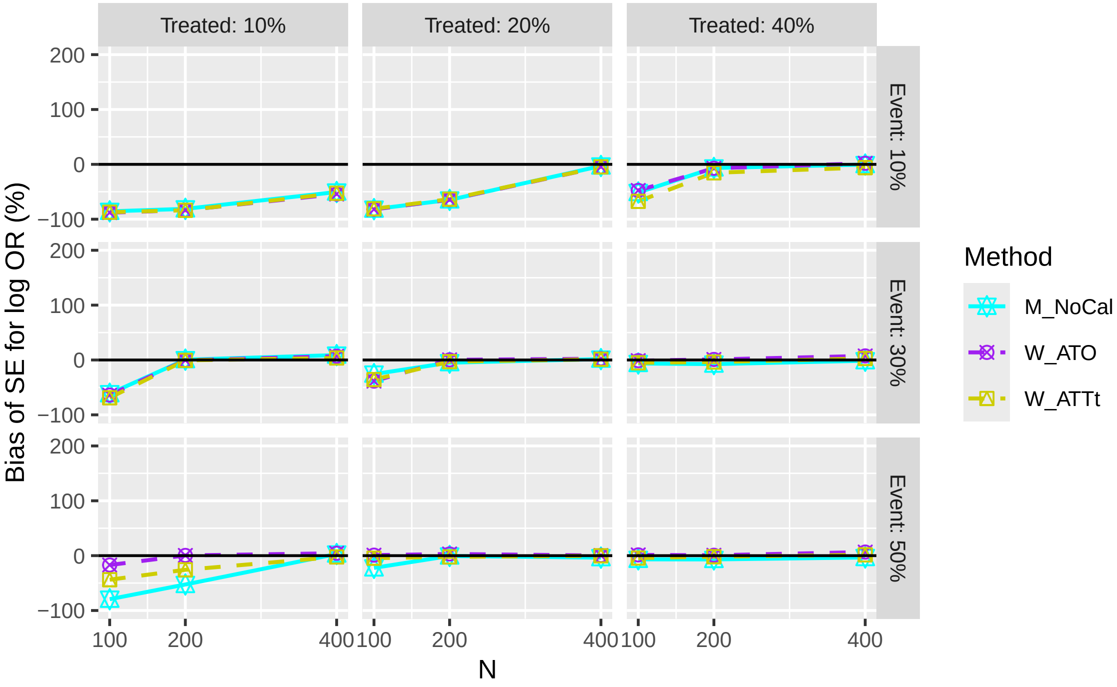

Figure S440. Mean bias of standard error for log odds ratio (categorical covariate, matching ratio 1:1, true OR: 0.5, c statistic: 0.85, robust inference); other methods.

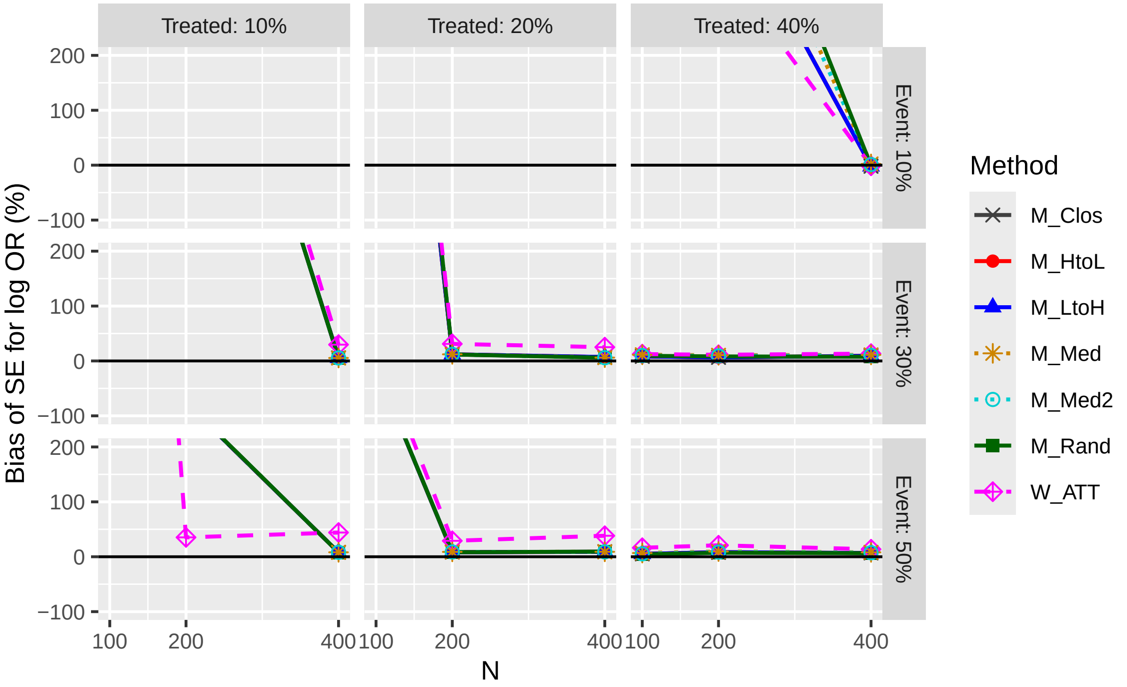

Figure S441. Mean bias of standard error for log odds ratio (categorical covariate, matching ratio 1:1, true OR: 0.5, c statistic: 0.6, naive inference).

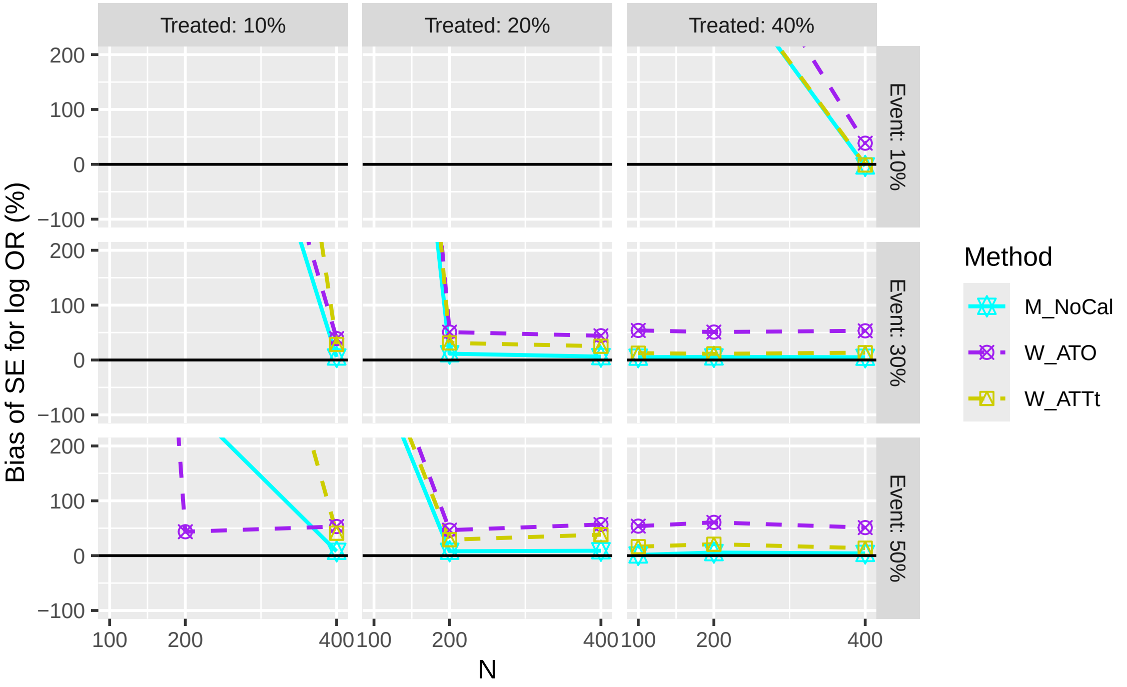

Figure S442. Mean bias of standard error for log odds ratio (categorical covariate, matching ratio 1:1, true OR: 0.5, c statistic: 0.6, naive inference); other methods.

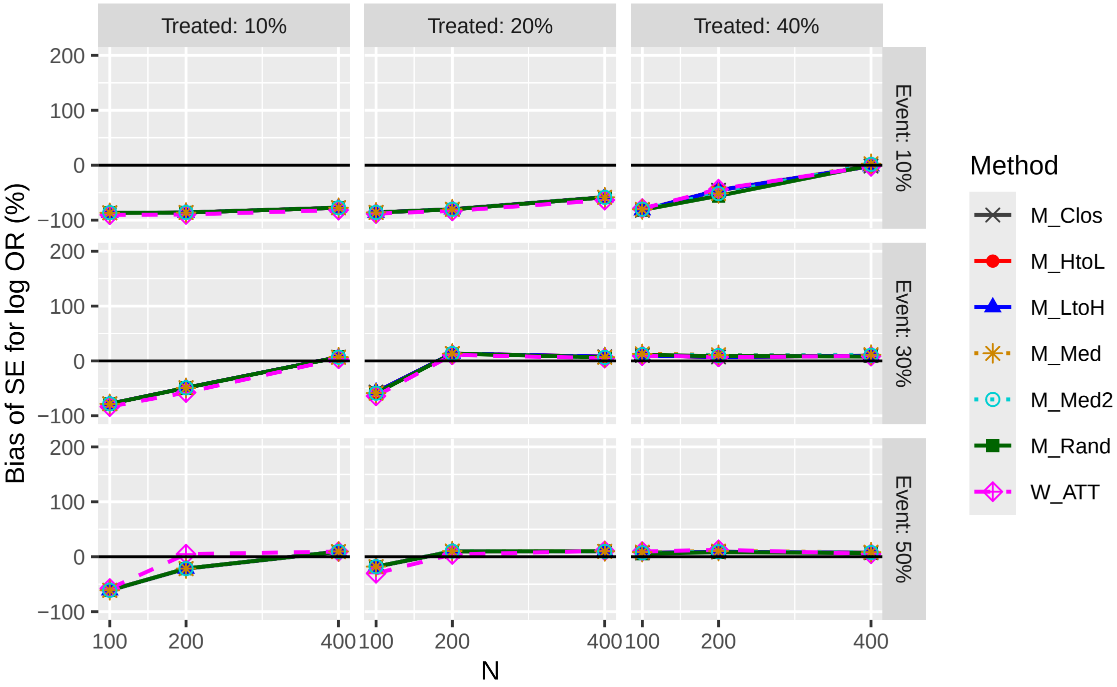

Figure S443. Mean bias of standard error for log odds ratio (categorical covariate, matching ratio 1:1, true OR: 0.5, c statistic: 0.6, robust inference).

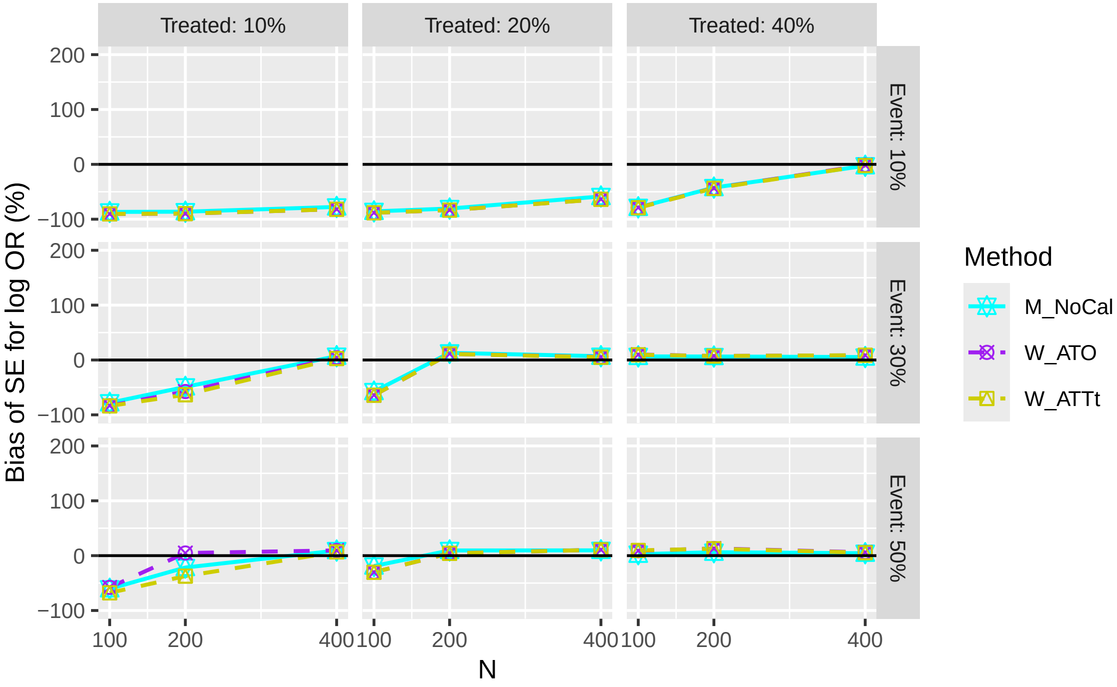

Figure S444. Mean bias of standard error for log odds ratio (categorical covariate, matching ratio 1:1, true OR: 0.5, c statistic: 0.6, robust inference); other methods.

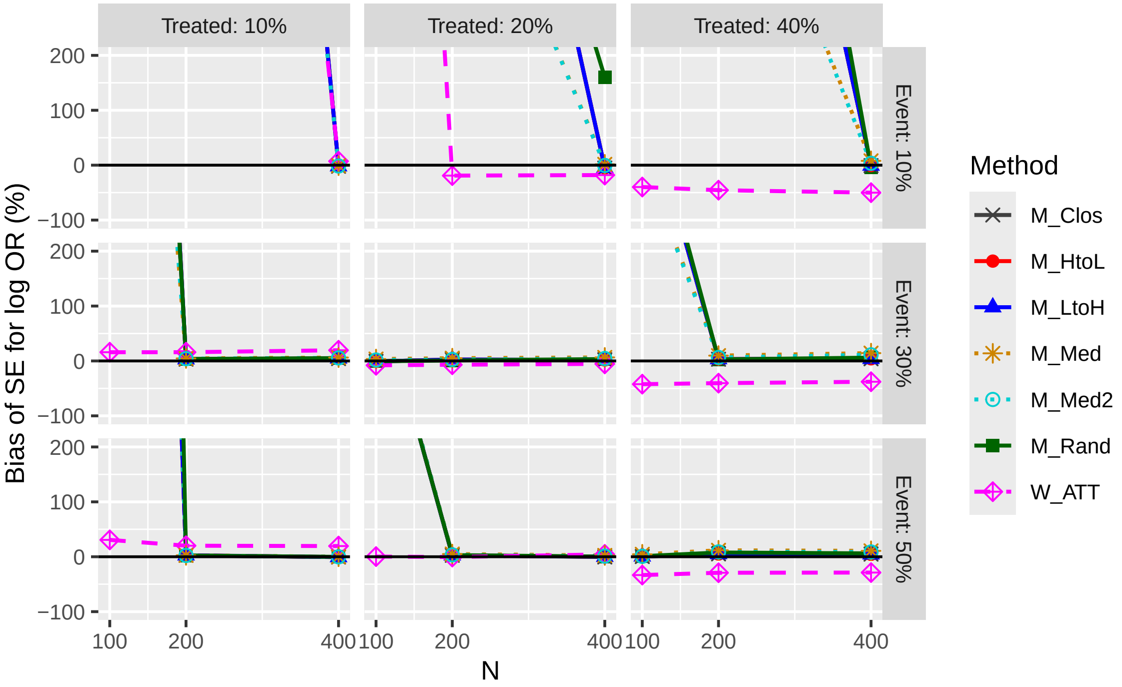

Figure S445. Mean bias of standard error for log odds ratio (categorical covariate, matching ratio 1:2, true OR: 1, c statistic: 0.85, naive inference).

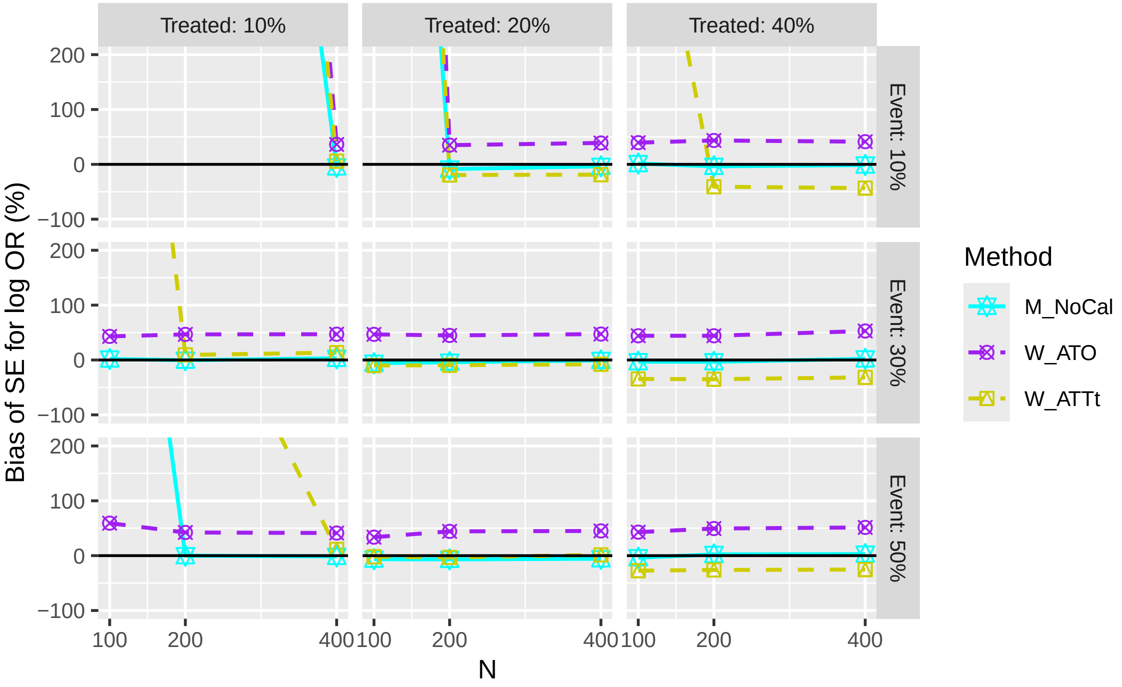

Figure S446. Mean bias of standard error for log odds ratio (categorical covariate, matching ratio 1:2, true OR: 1, c statistic: 0.85, naive inference); other methods.

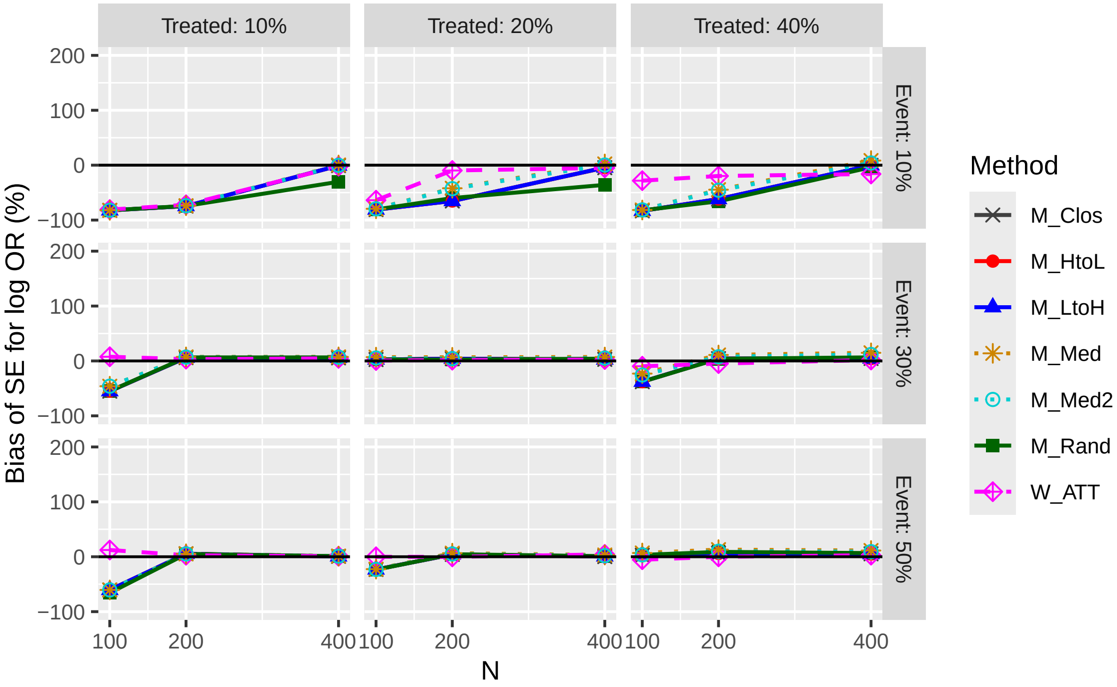

Figure S447. Mean bias of standard error for log odds ratio (categorical covariate, matching ratio 1:2, true OR: 1, c statistic: 0.85, robust inference).

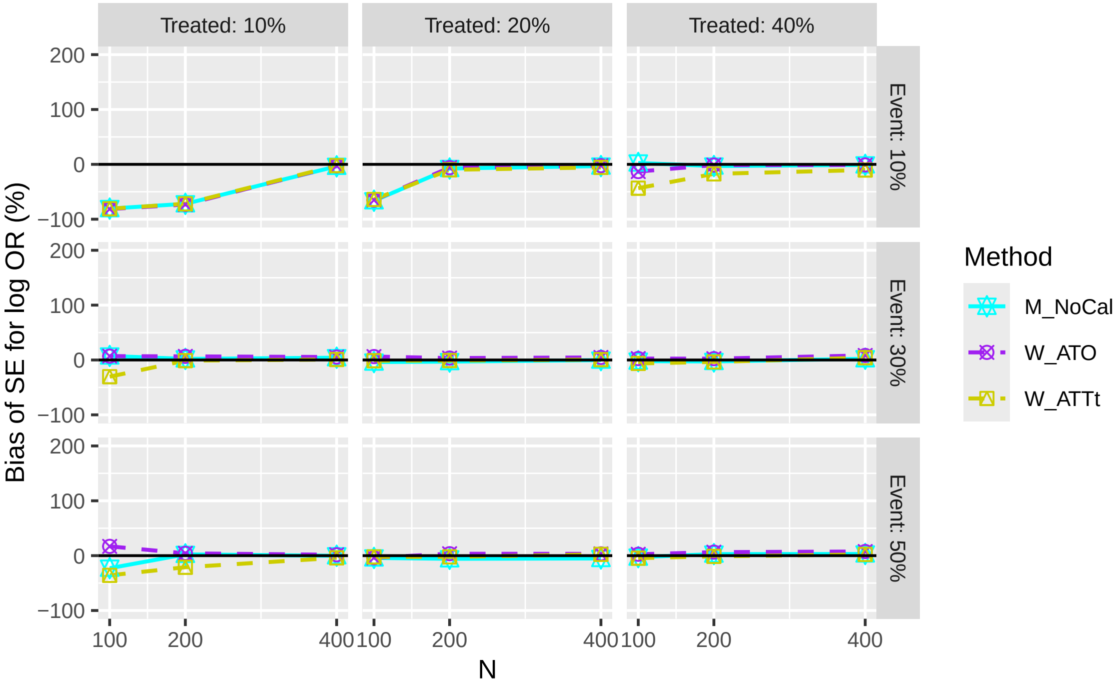

Figure S448. Mean bias of standard error for log odds ratio (categorical covariate, matching ratio 1:2, true OR: 1, c statistic: 0.85, robust inference); other methods.

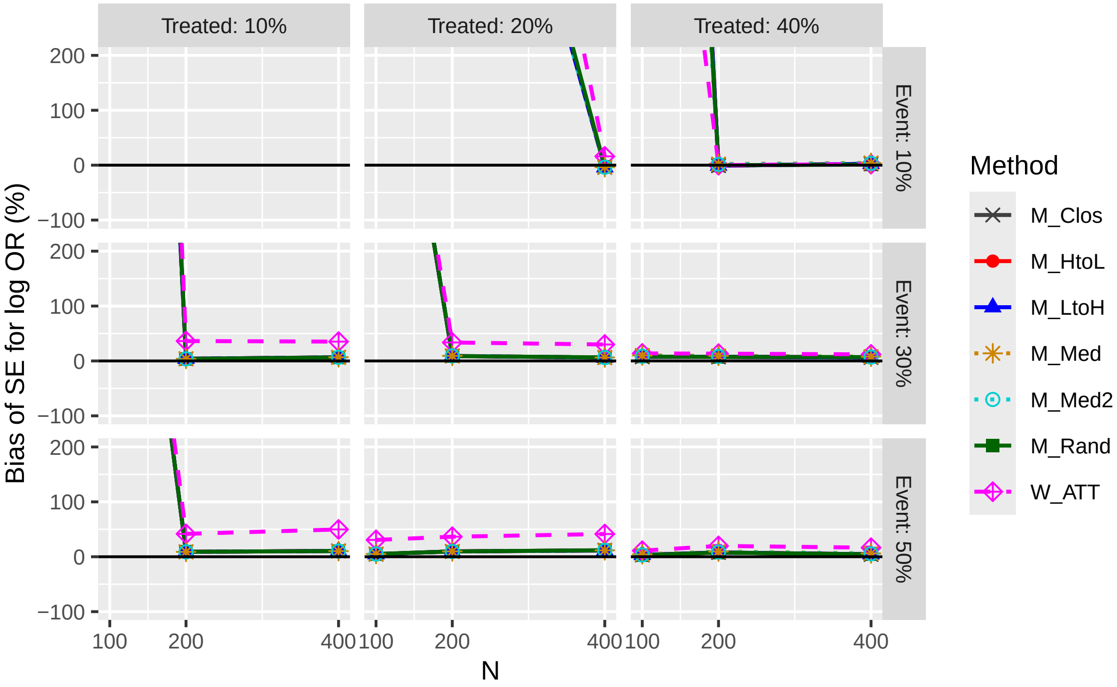

Figure S449. Mean bias of standard error for log odds ratio (categorical covariate, matching ratio 1:2, true OR: 1, c statistic: 0.6, naive inference).

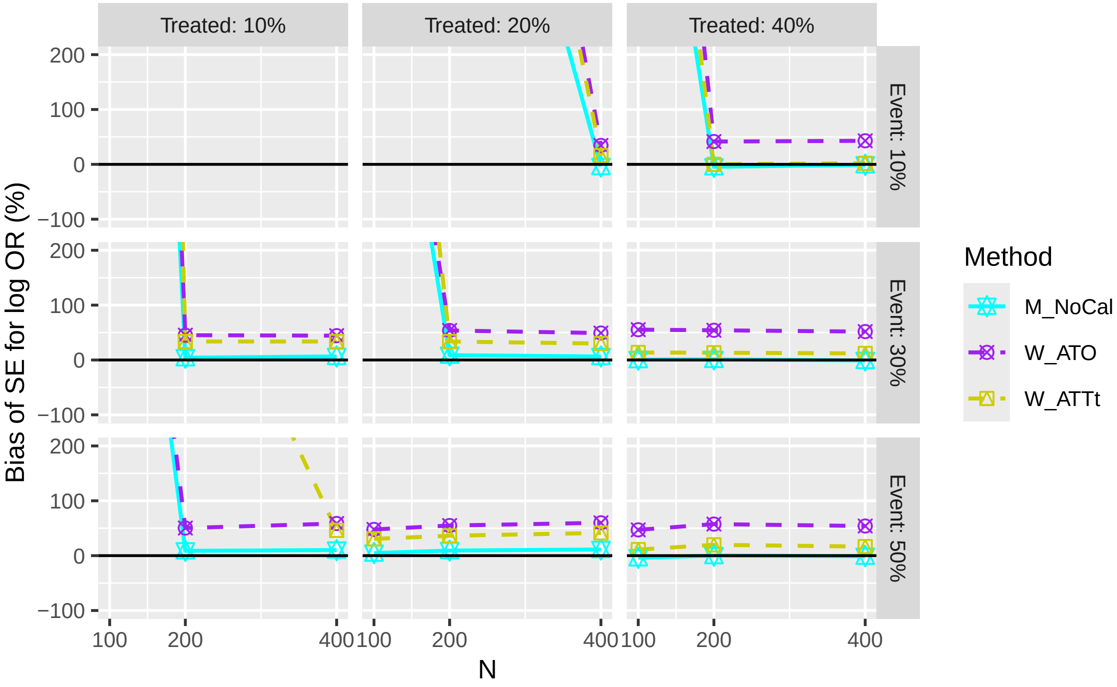

Figure S450. Mean bias of standard error for log odds ratio (categorical covariate, matching ratio 1:2, true OR: 1, c statistic: 0.6, naive inference); other methods.

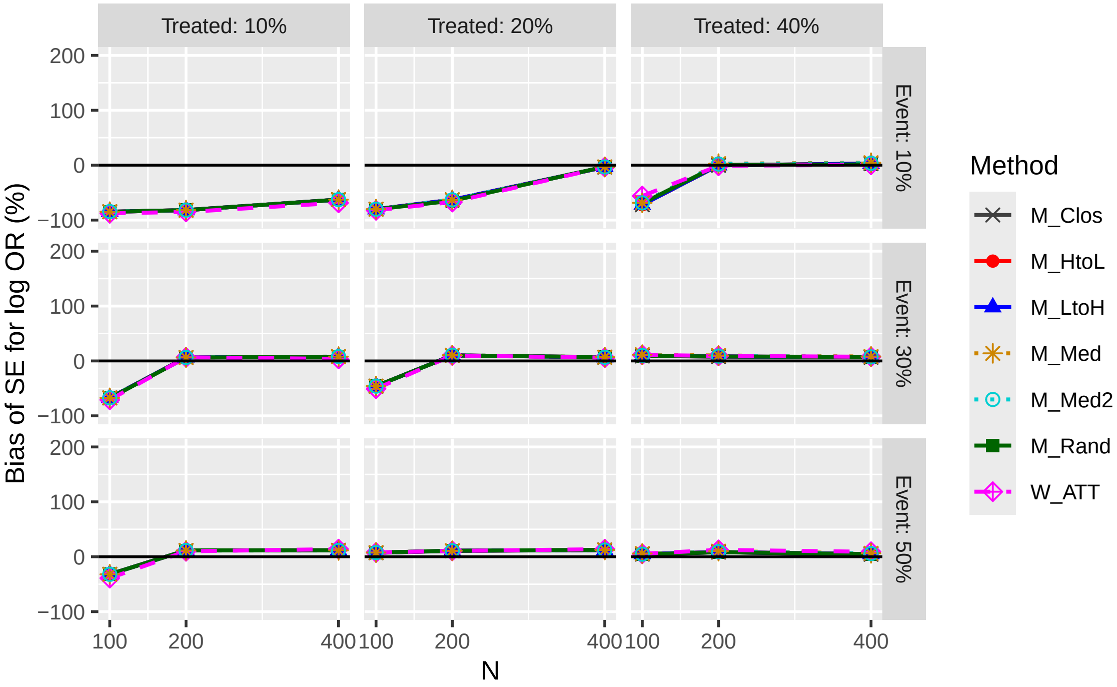

Figure S451. Mean bias of standard error for log odds ratio (categorical covariate, matching ratio 1:2, true OR: 1, c statistic: 0.6, robust inference).

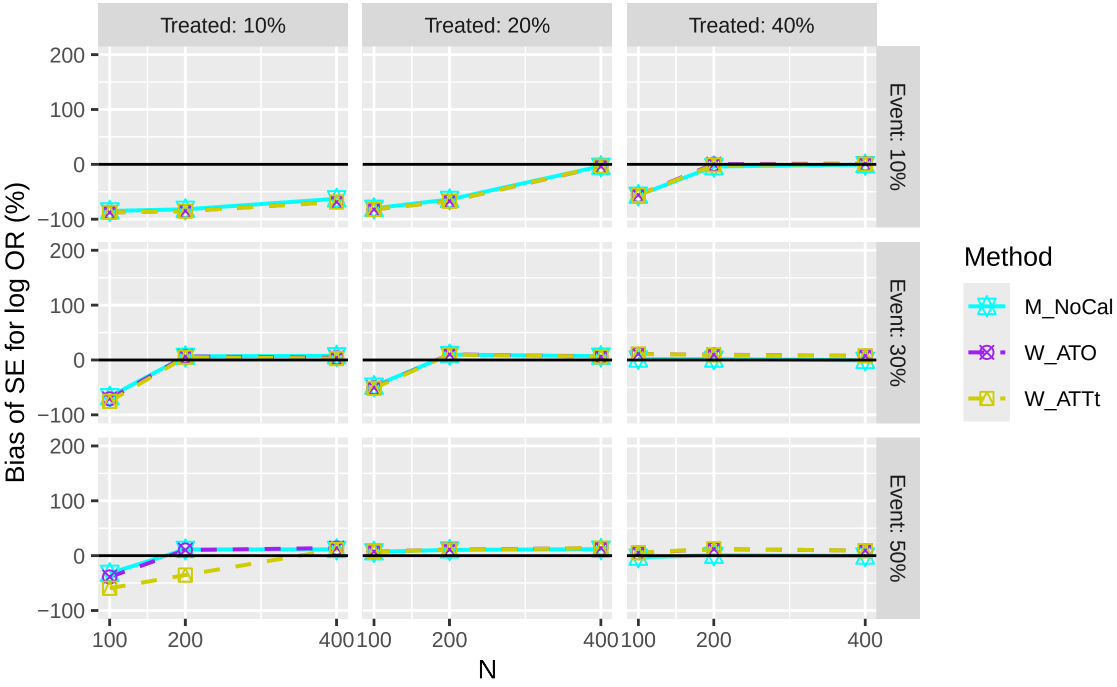

Figure S452. Mean bias of standard error for log odds ratio (categorical covariate, matching ratio 1:2, true OR: 1, c statistic: 0.6, robust inference); other methods.

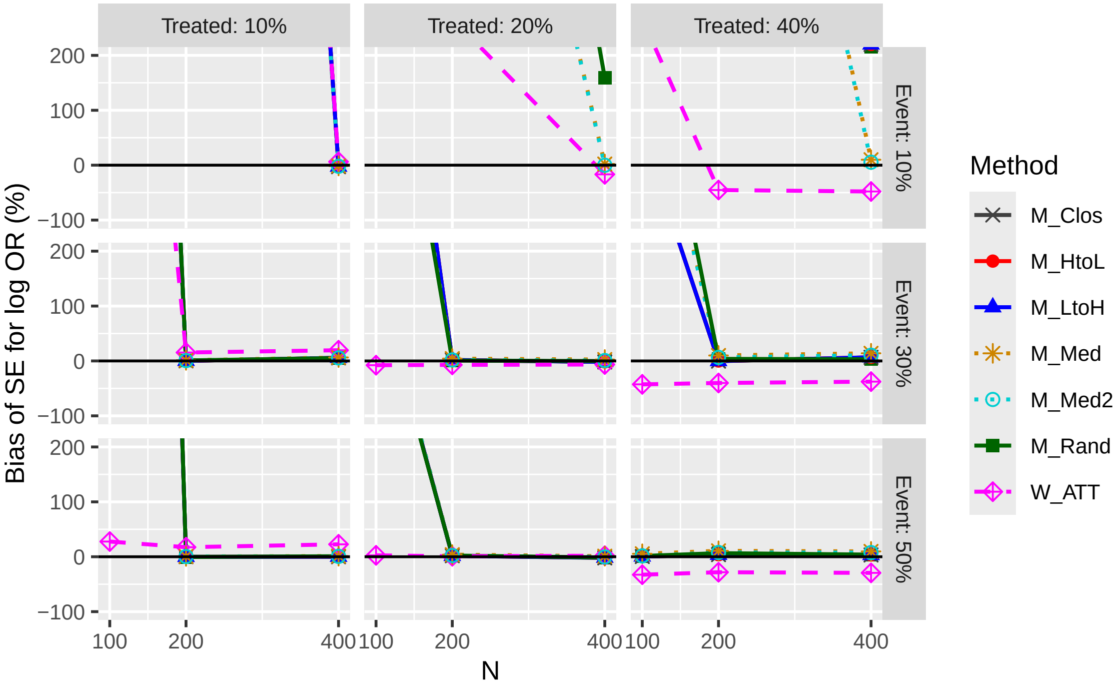

Figure S453. Mean bias of standard error for log odds ratio (categorical covariate, matching ratio 1:2, true OR: 0.75, c statistic: 0.85, naive inference).

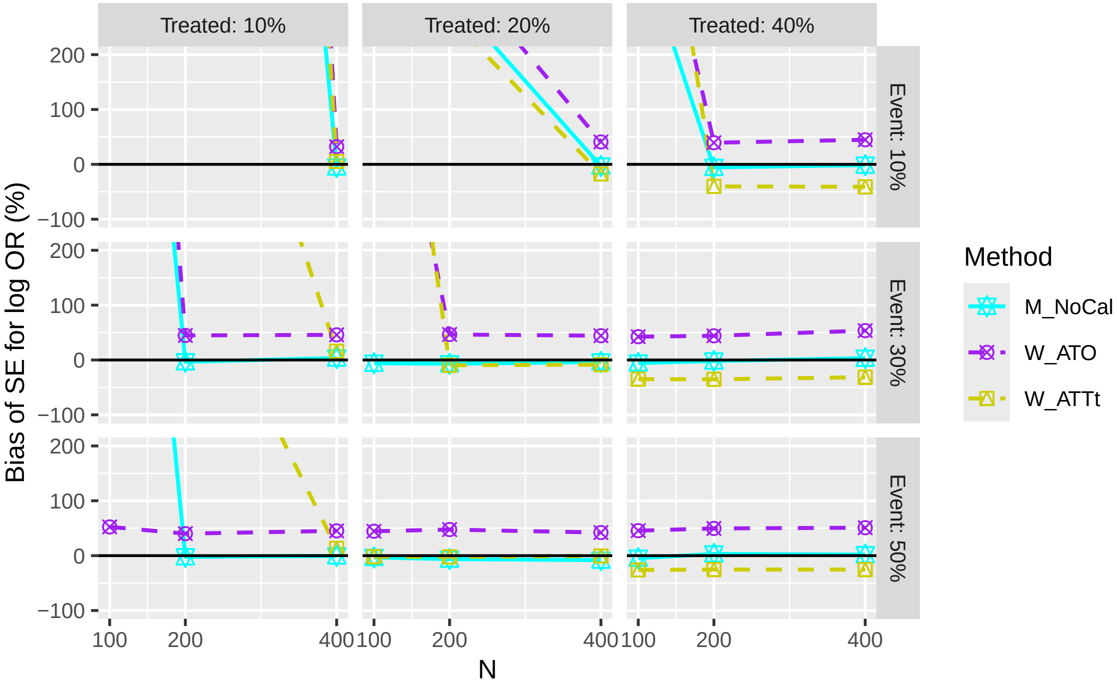

Figure S454. Mean bias of standard error for log odds ratio (categorical covariate, matching ratio 1:2, true OR: 0.75, c statistic: 0.85, naive inference); other methods.

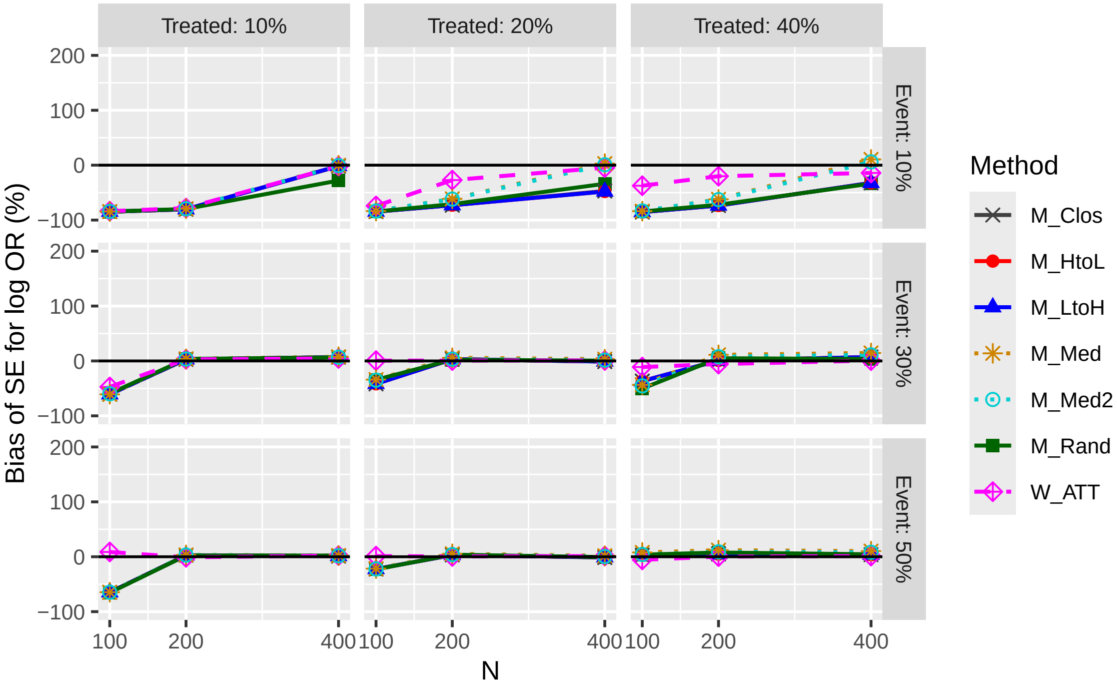

Figure S455. Mean bias of standard error for log odds ratio (categorical covariate, matching ratio 1:2, true OR: 0.75, c statistic: 0.85, robust inference).

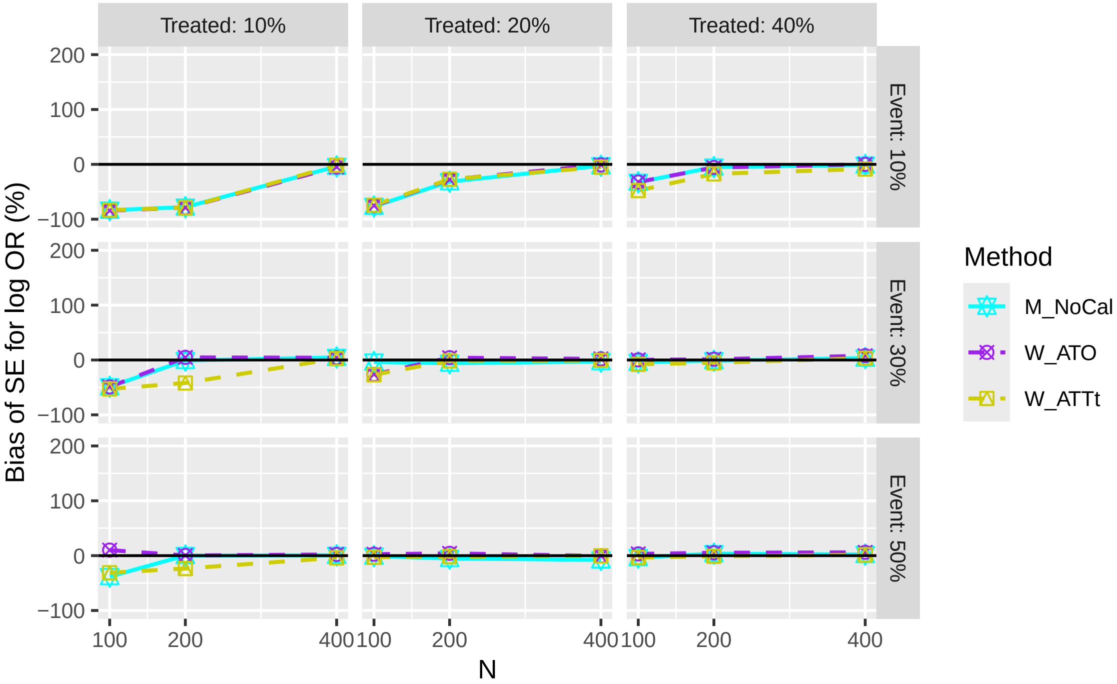

Figure S456. Mean bias of standard error for log odds ratio (categorical covariate, matching ratio 1:2, true OR: 0.75, c statistic: 0.85, robust inference); other methods.

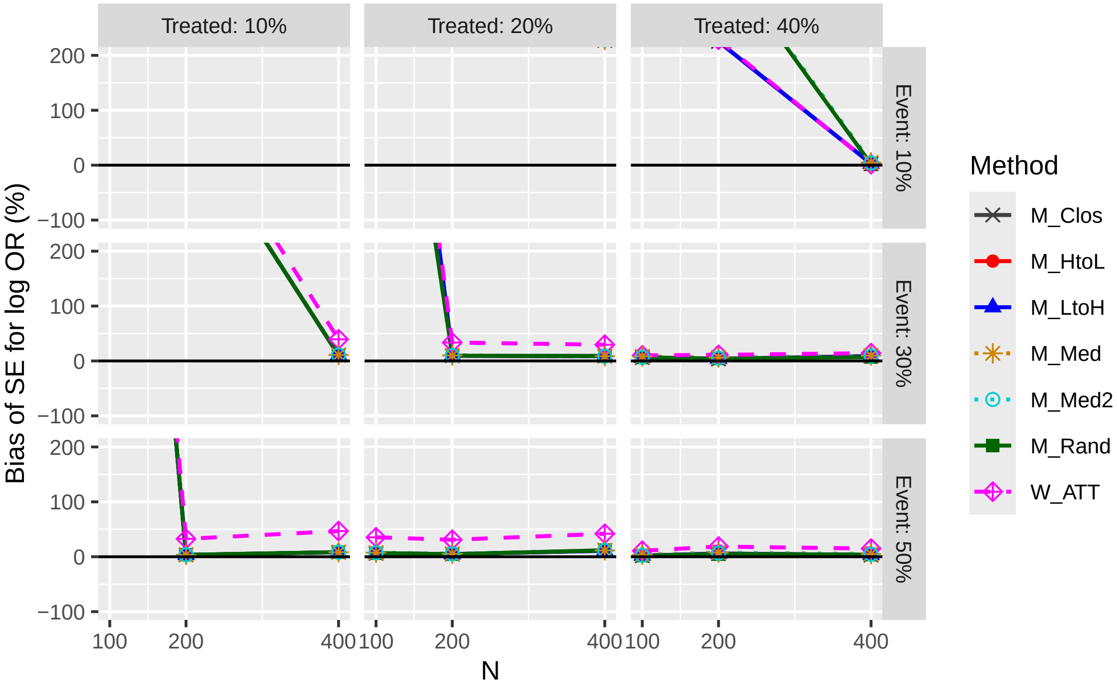

Figure S457. Mean bias of standard error for log odds ratio (categorical covariate, matching ratio 1:2, true OR: 0.75, c statistic: 0.6, naive inference).

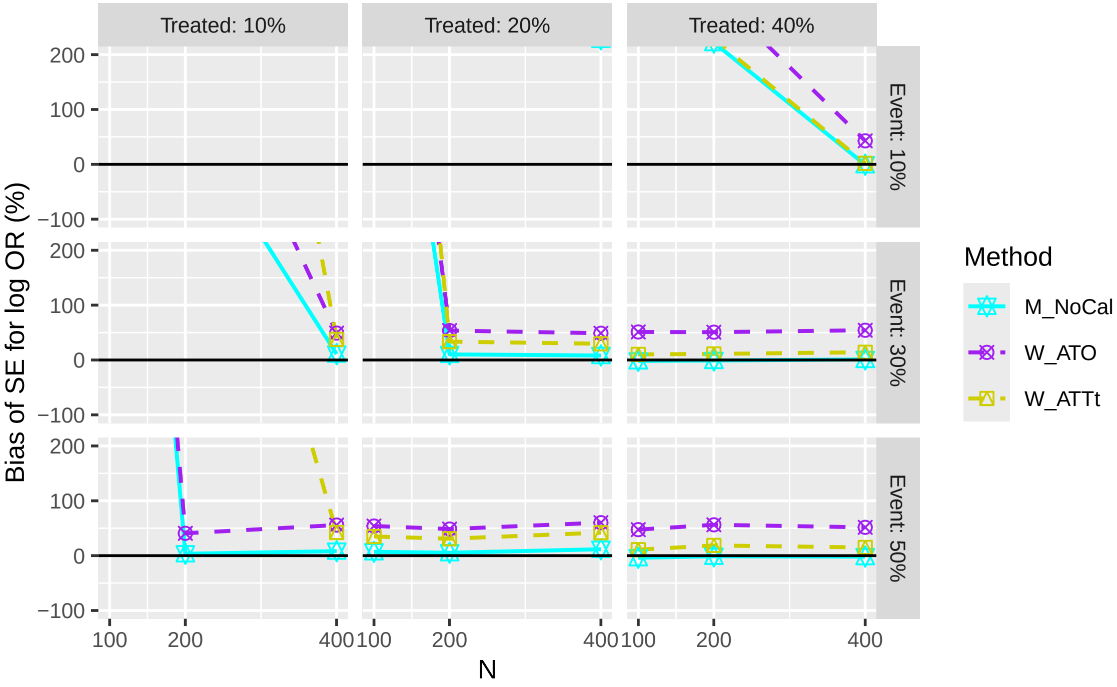

Figure S458. Mean bias of standard error for log odds ratio (categorical covariate, matching ratio 1:2, true OR: 0.75, c statistic: 0.6, naive inference); other methods.

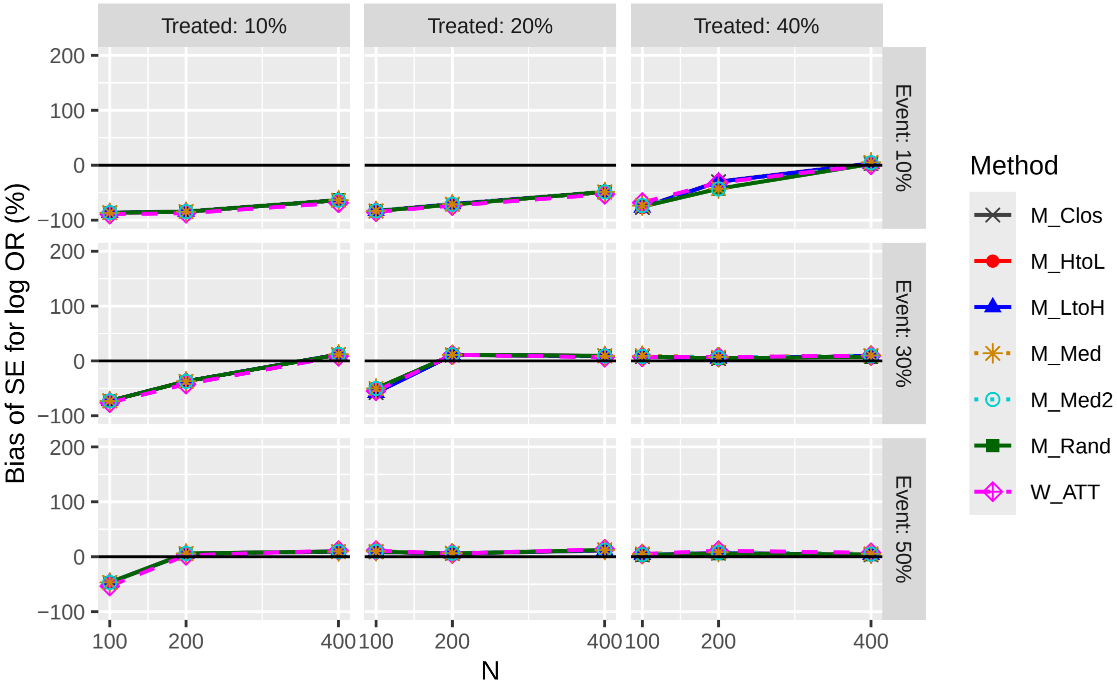

Figure S459. Mean bias of standard error for log odds ratio (categorical covariate, matching ratio 1:2, true OR: 0.75, c statistic: 0.6, robust inference).

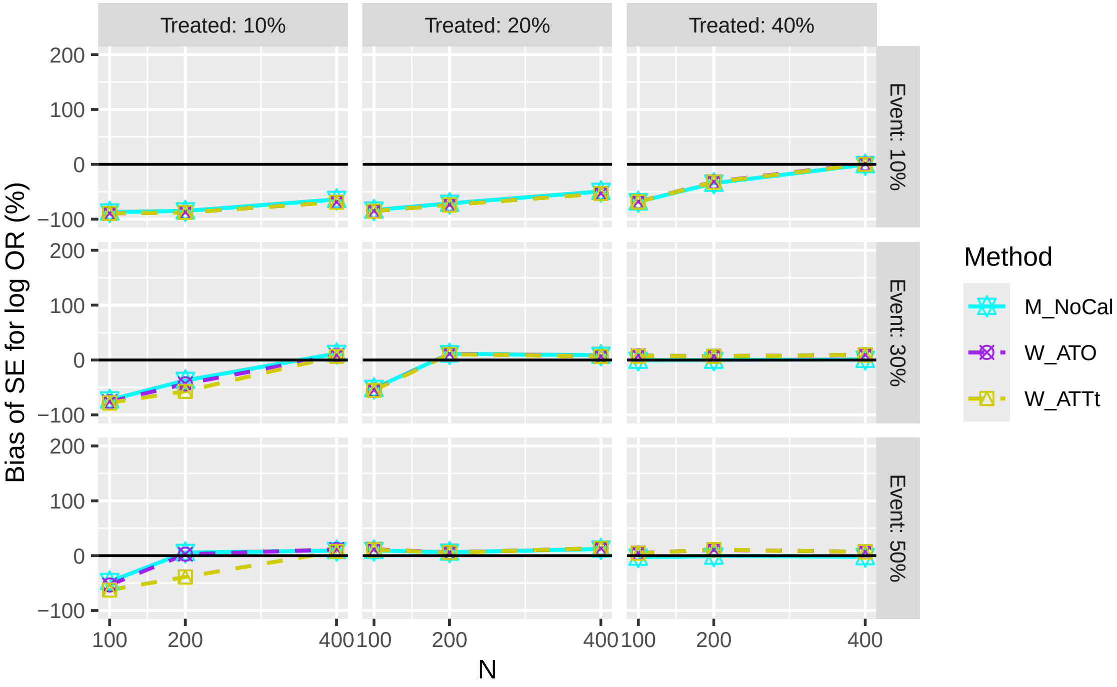

Figure S460. Mean bias of standard error for log odds ratio (categorical covariate, matching ratio 1:2, true OR: 0.75, c statistic: 0.6, robust inference); other methods.

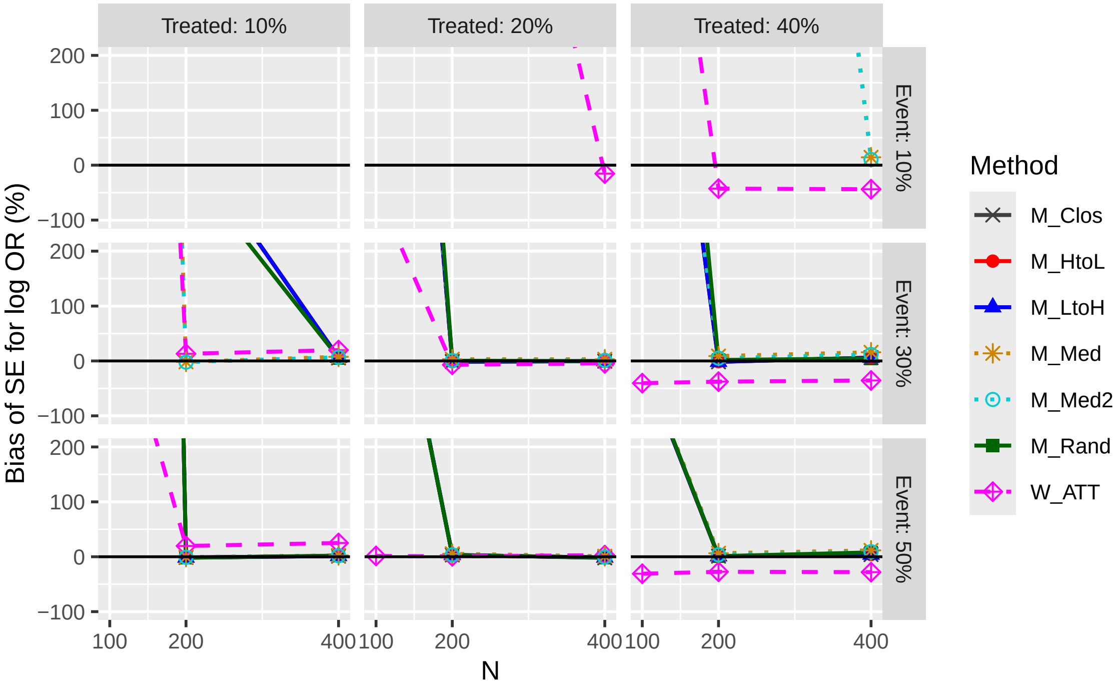

Figure S461. Mean bias of standard error for log odds ratio (categorical covariate, matching ratio 1:2, true OR: 0.5, c statistic: 0.85, naive inference).

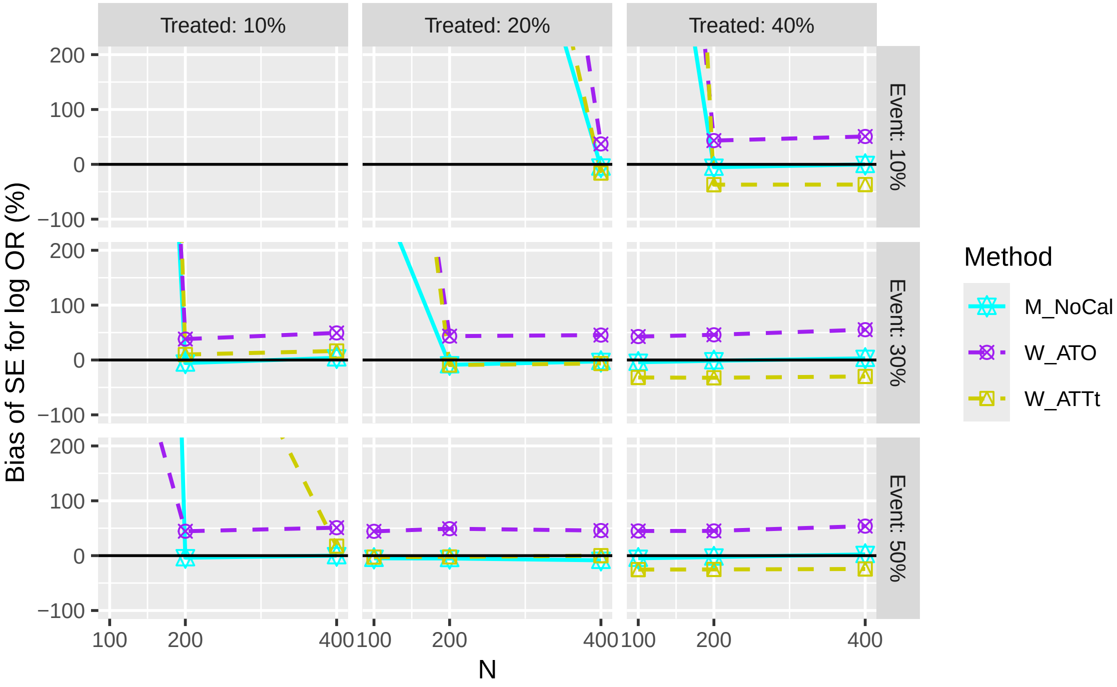

Figure S462. Mean bias of standard error for log odds ratio (categorical covariate, matching ratio 1:2, true OR: 0.5, c statistic: 0.85, naive inference); other methods.

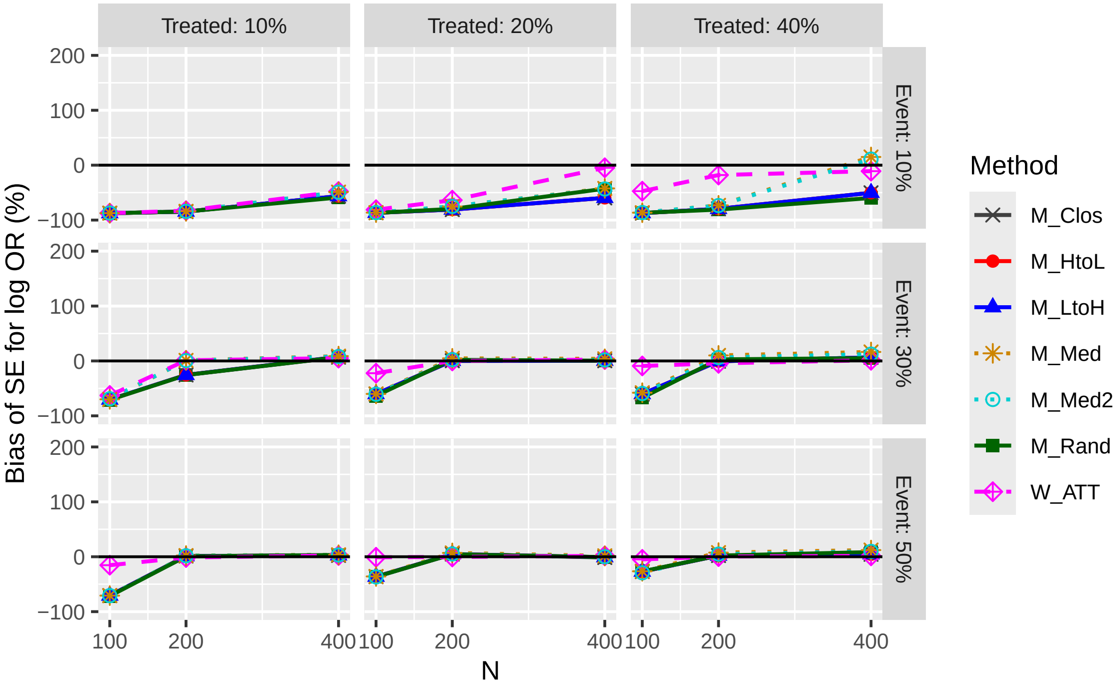

Figure S463. Mean bias of standard error for log odds ratio (categorical covariate, matching ratio 1:2, true OR: 0.5, c statistic: 0.85, robust inference).

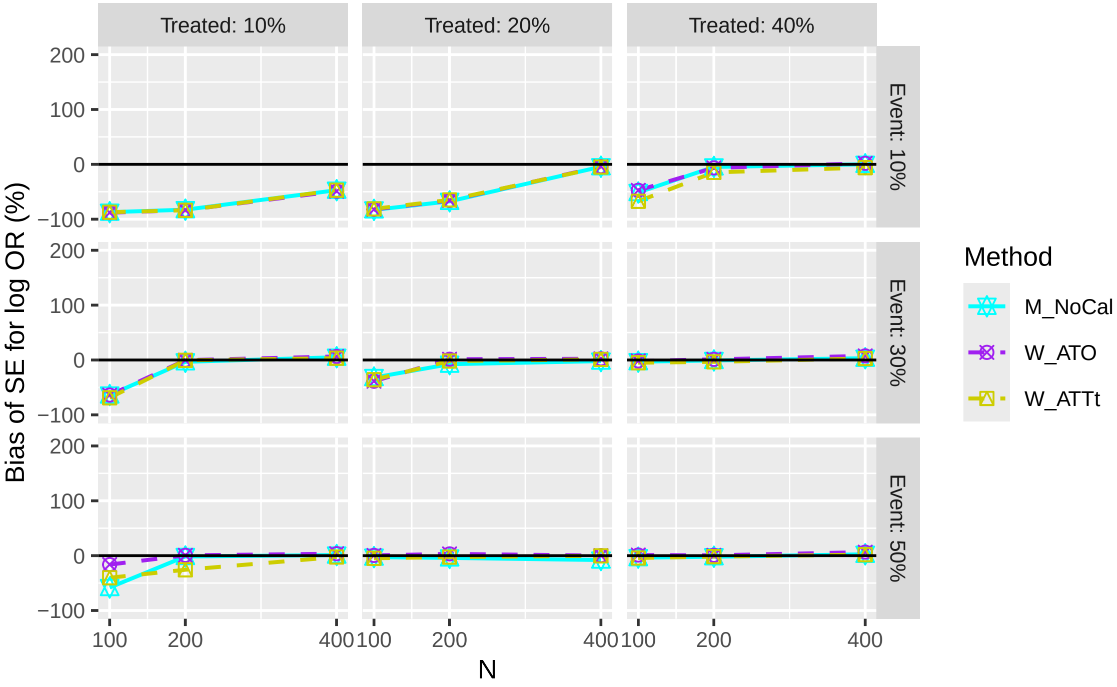

Figure S464. Mean bias of standard error for log odds ratio (categorical covariate, matching ratio 1:2, true OR: 0.5, c statistic: 0.85, robust inference); other methods.

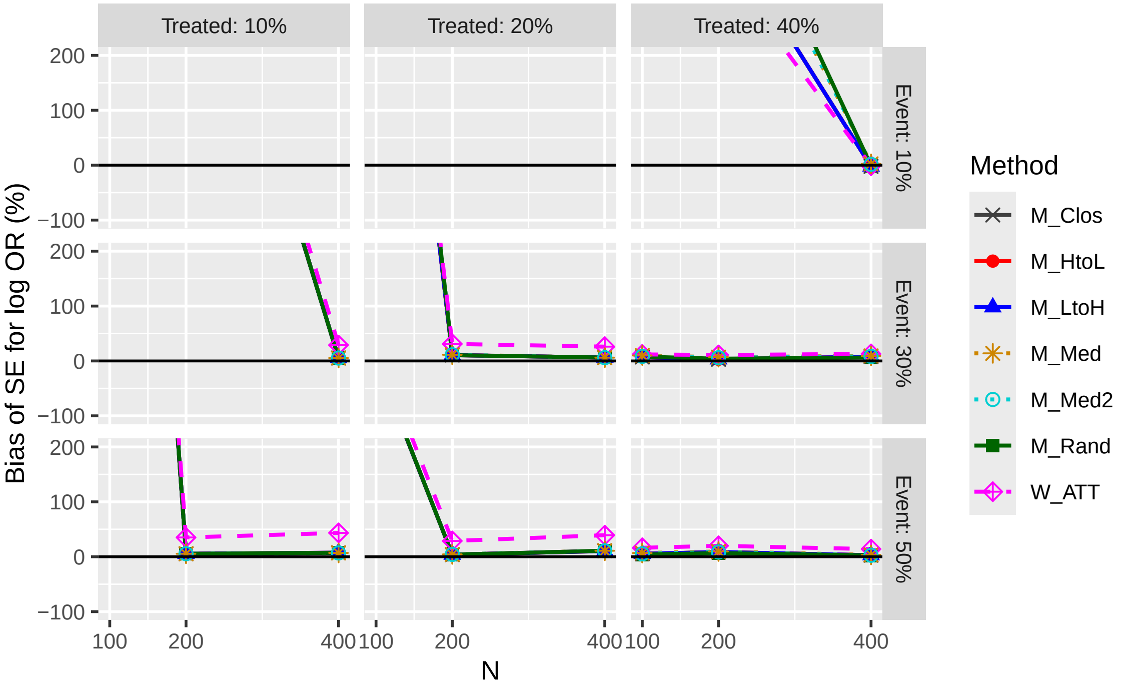

Figure S465. Mean bias of standard error for log odds ratio (categorical covariate, matching ratio 1:2, true OR: 0.5, c statistic: 0.6, naive inference).

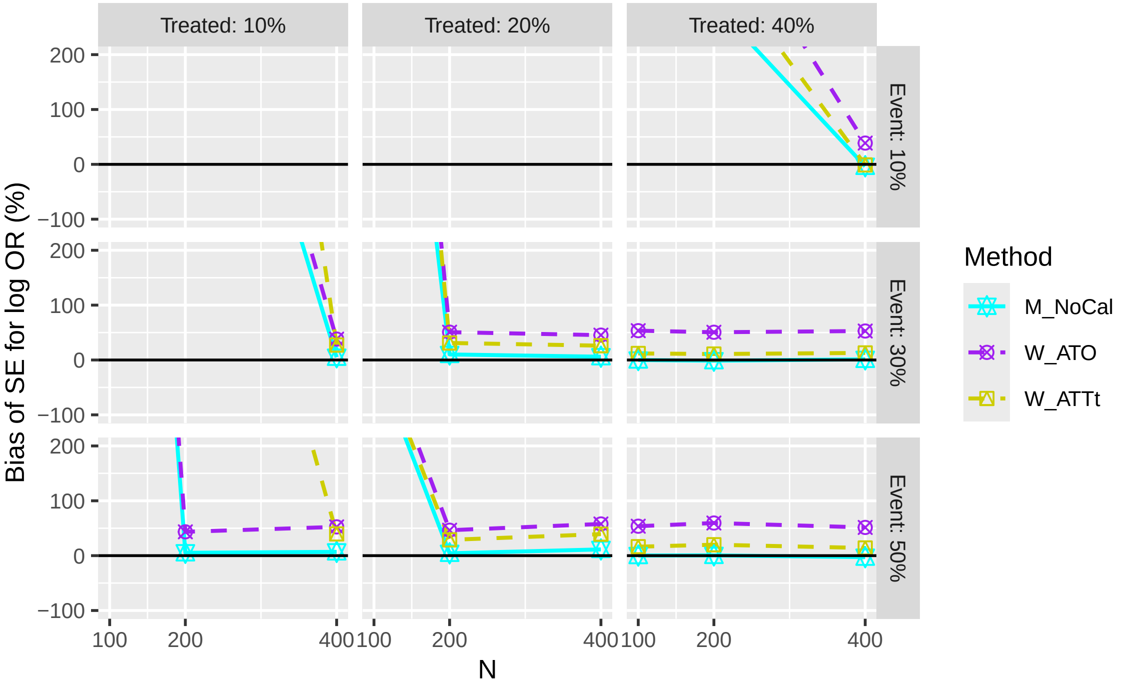

Figure S466. Mean bias of standard error for log odds ratio (categorical covariate, matching ratio 1:2, true OR: 0.5, c statistic: 0.6, naive inference); other methods.

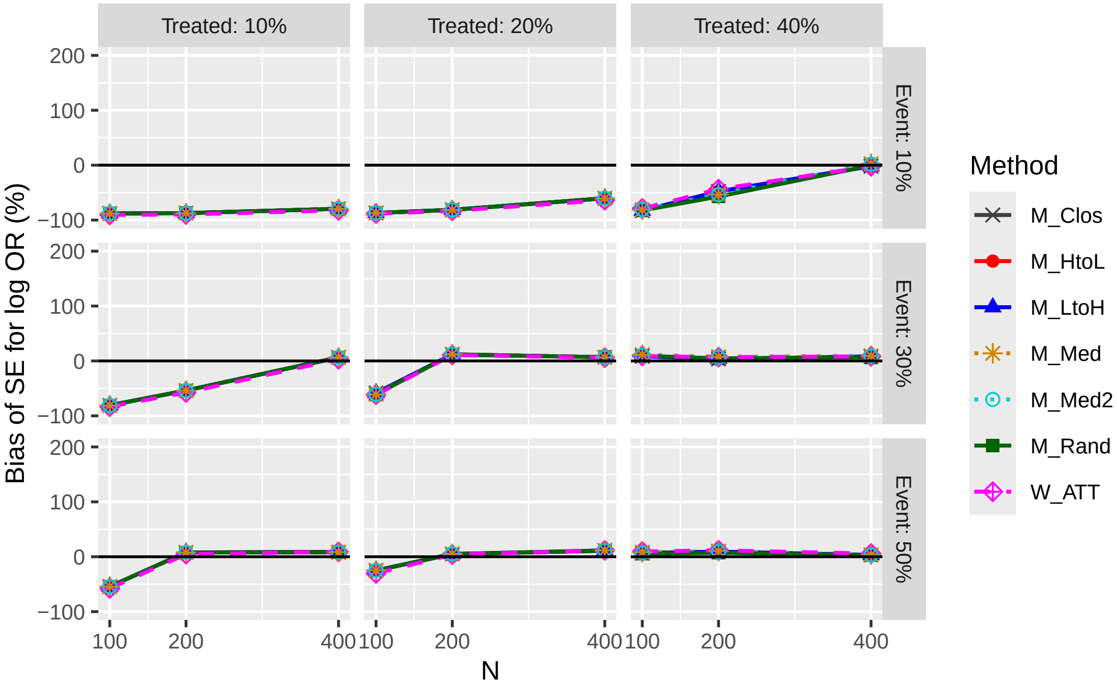

Figure S467. Mean bias of standard error for log odds ratio (categorical covariate, matching ratio 1:2, true OR: 0.5, c statistic: 0.6, robust inference).

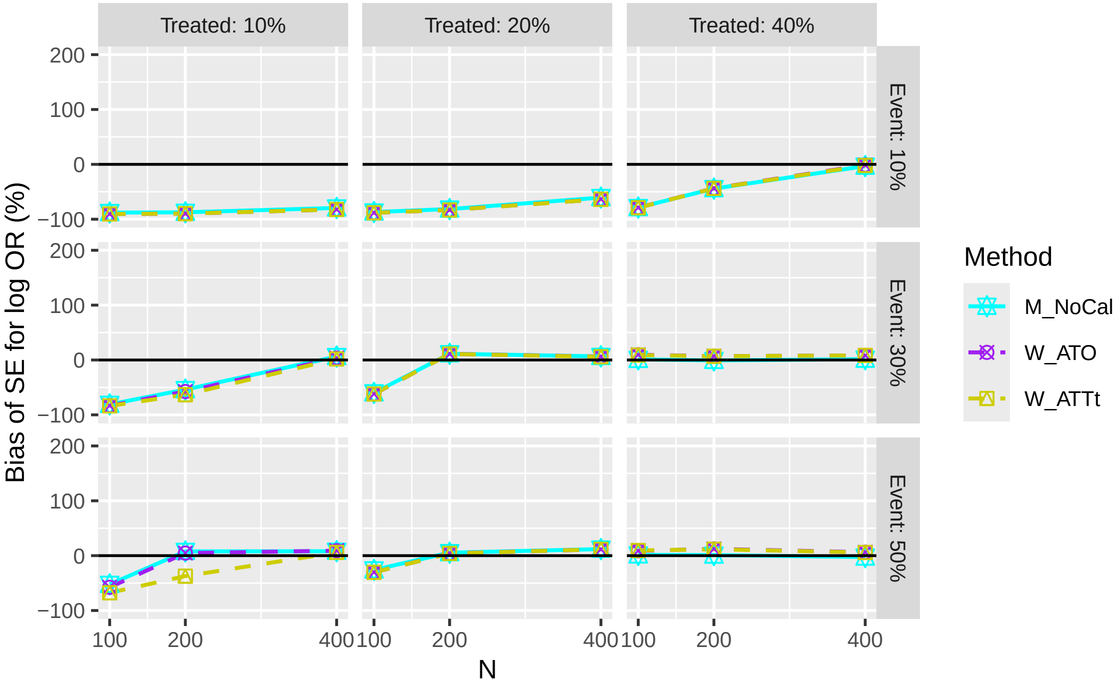

Figure S468. Mean bias of standard error for log odds ratio (categorical covariate, matching ratio 1:2, true OR: 0.5, c statistic: 0.6, robust inference); other methods.

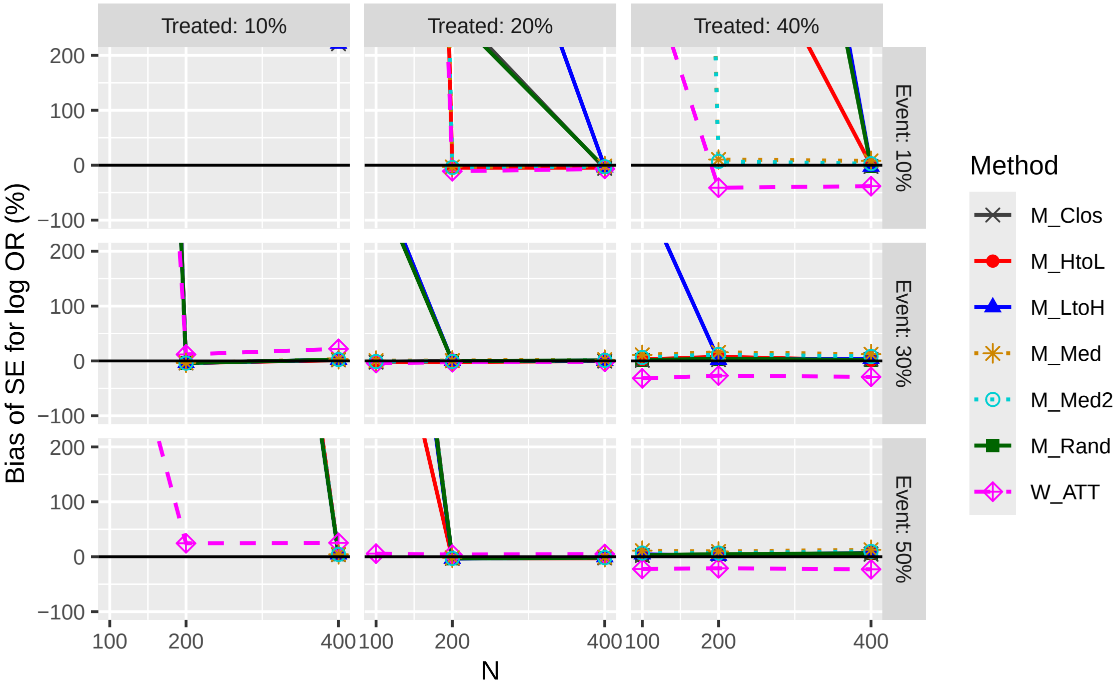

Figure S469. Mean bias of standard error for log odds ratio (multimodal continuous covariate, matching ratio 1:1, true OR: 1, c statistic: 0.85, naive inference).

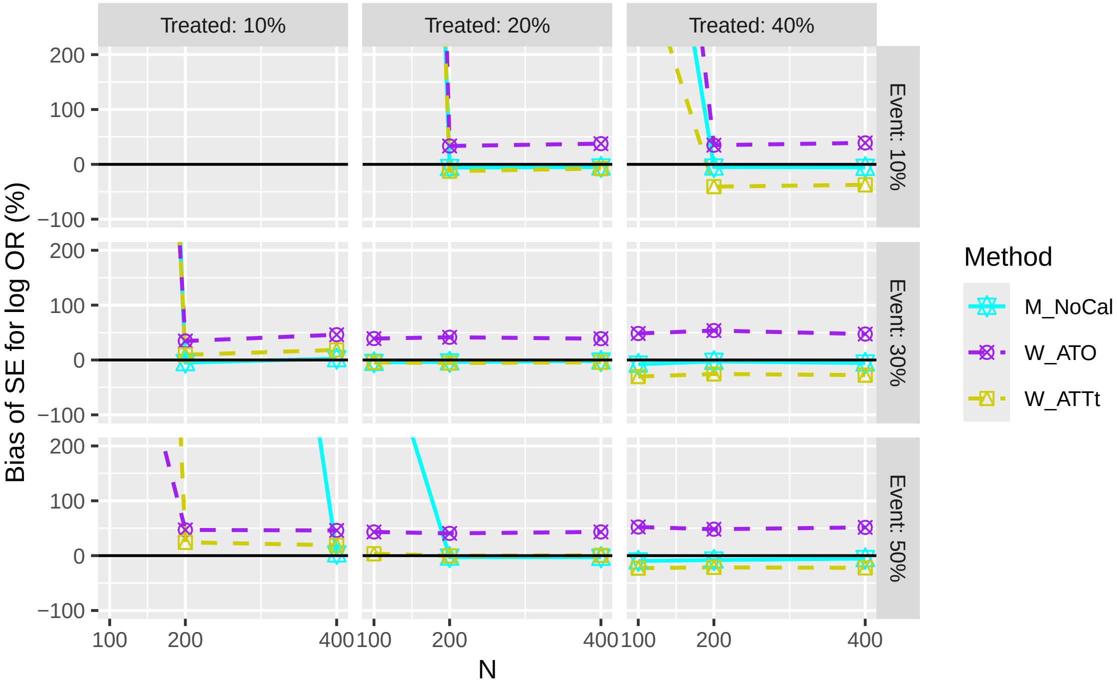

Figure S470. Mean bias of standard error for log odds ratio (multimodal continuous covariate, matching ratio 1:1, true OR: 1, c statistic: 0.85, naive inference); other methods.

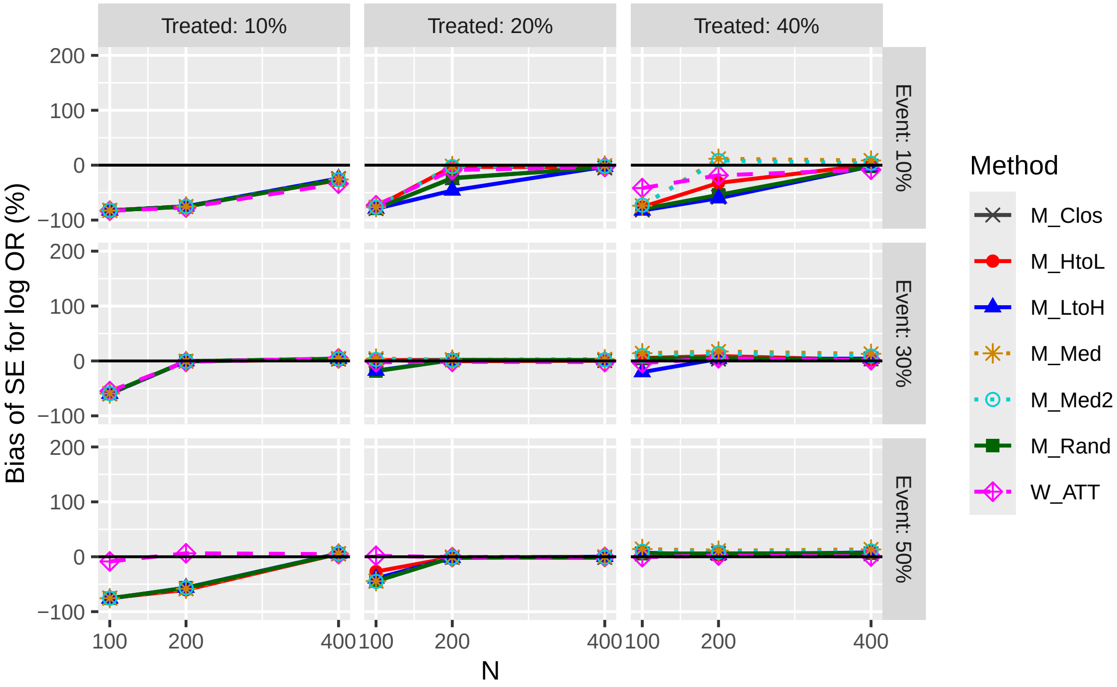

Figure S471. Mean bias of standard error for log odds ratio (multimodal continuous covariate, matching ratio 1:1, true OR: 1, c statistic: 0.85, robust inference).

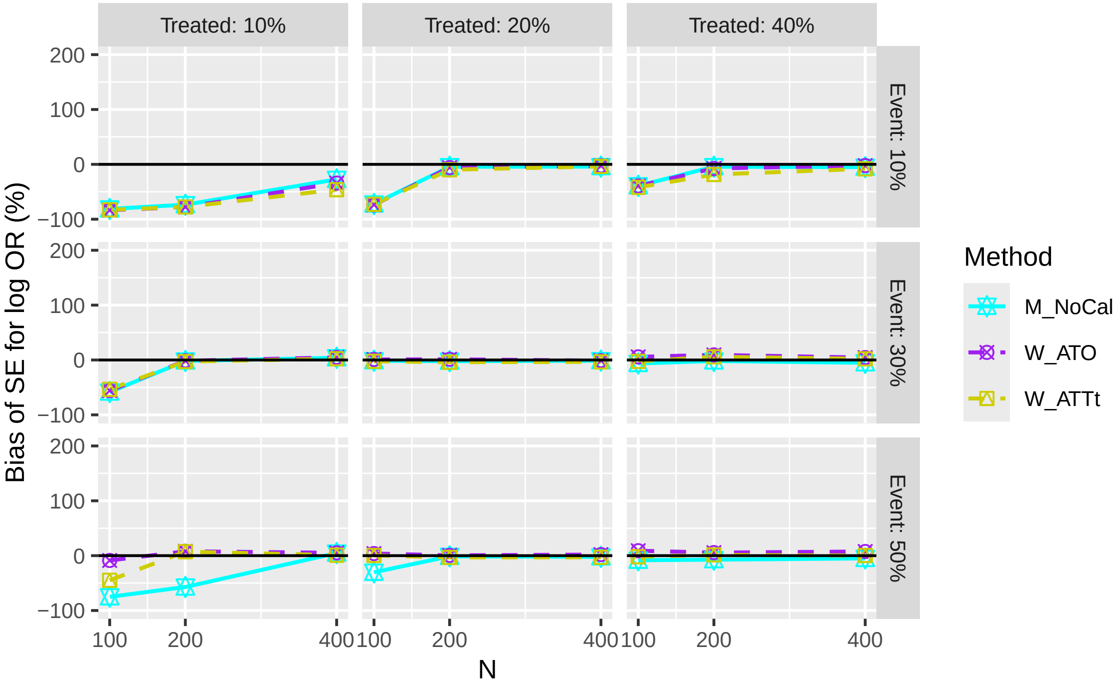

Figure S472. Mean bias of standard error for log odds ratio (multimodal continuous covariate, matching ratio 1:1, true OR: 1, c statistic: 0.85, robust inference); other methods.

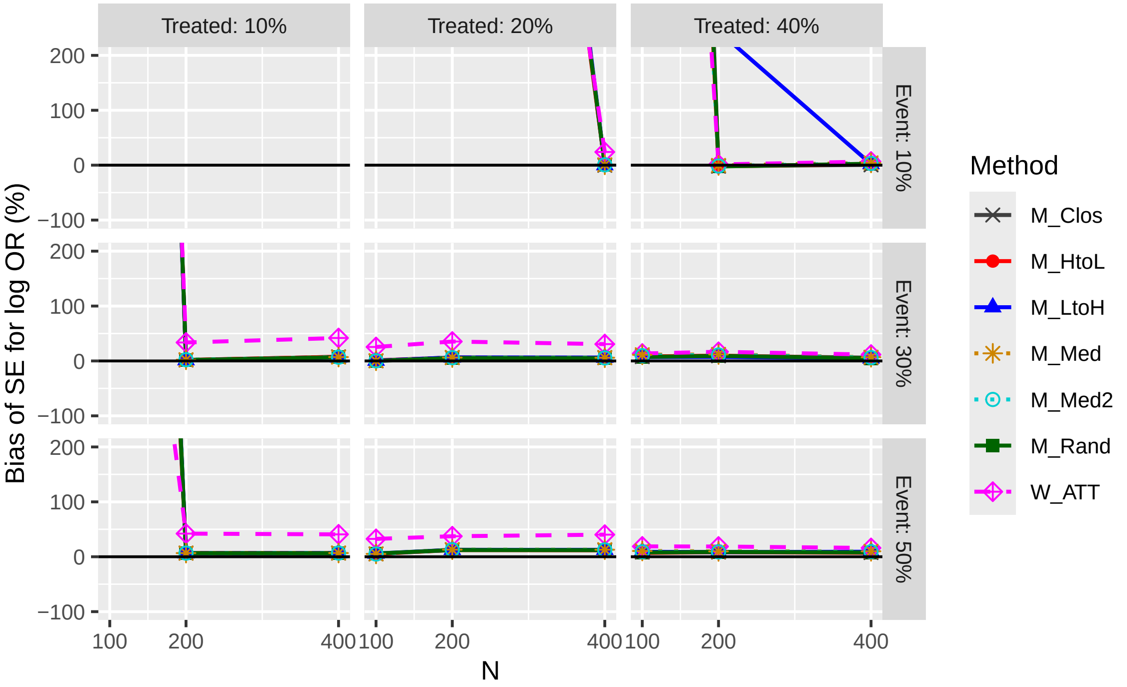

Figure S473. Mean bias of standard error for log odds ratio (multimodal continuous covariate, matching ratio 1:1, true OR: 1, c statistic: 0.6, naive inference).

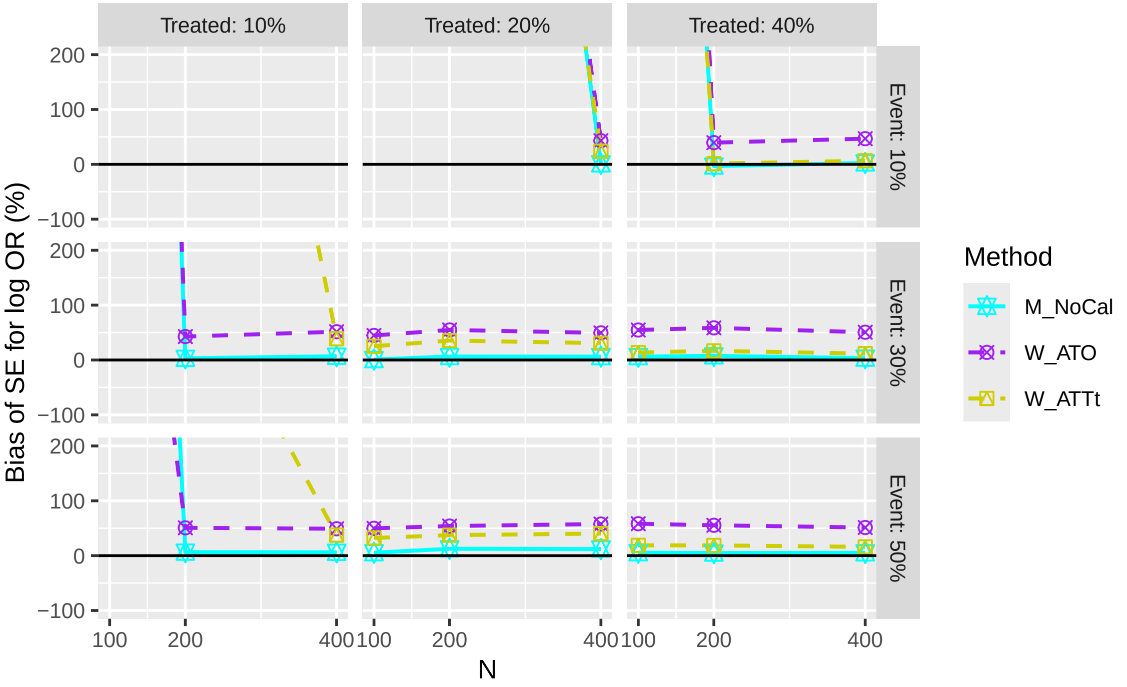

Figure S474. Mean bias of standard error for log odds ratio (multimodal continuous covariate, matching ratio 1:1, true OR: 1, c statistic: 0.6, naive inference); other methods.

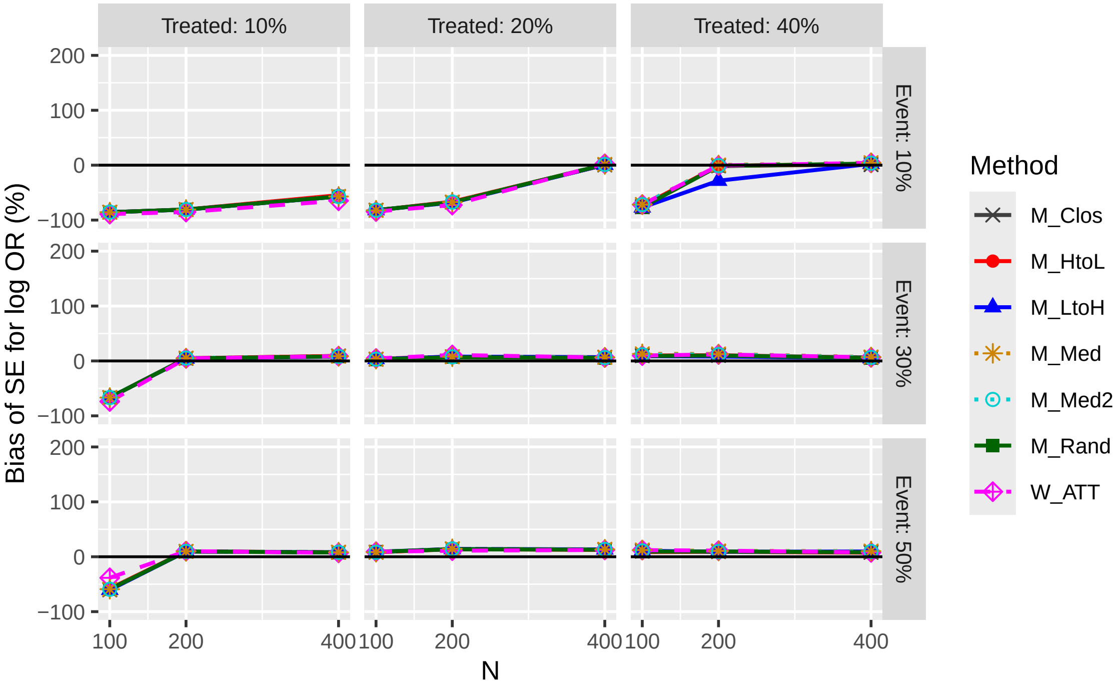

Figure S475. Mean bias of standard error for log odds ratio (multimodal continuous covariate, matching ratio 1:1, true OR: 1, c statistic: 0.6, robust inference).

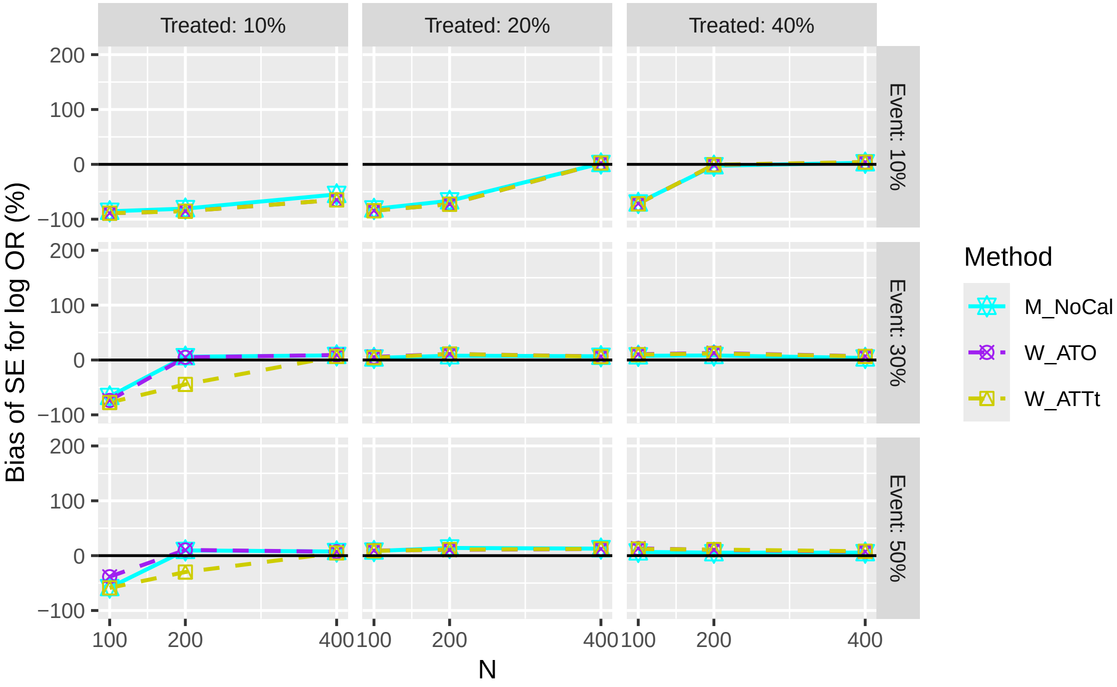

Figure S476. Mean bias of standard error for log odds ratio (multimodal continuous covariate, matching ratio 1:1, true OR: 1, c statistic: 0.6, robust inference); other methods.

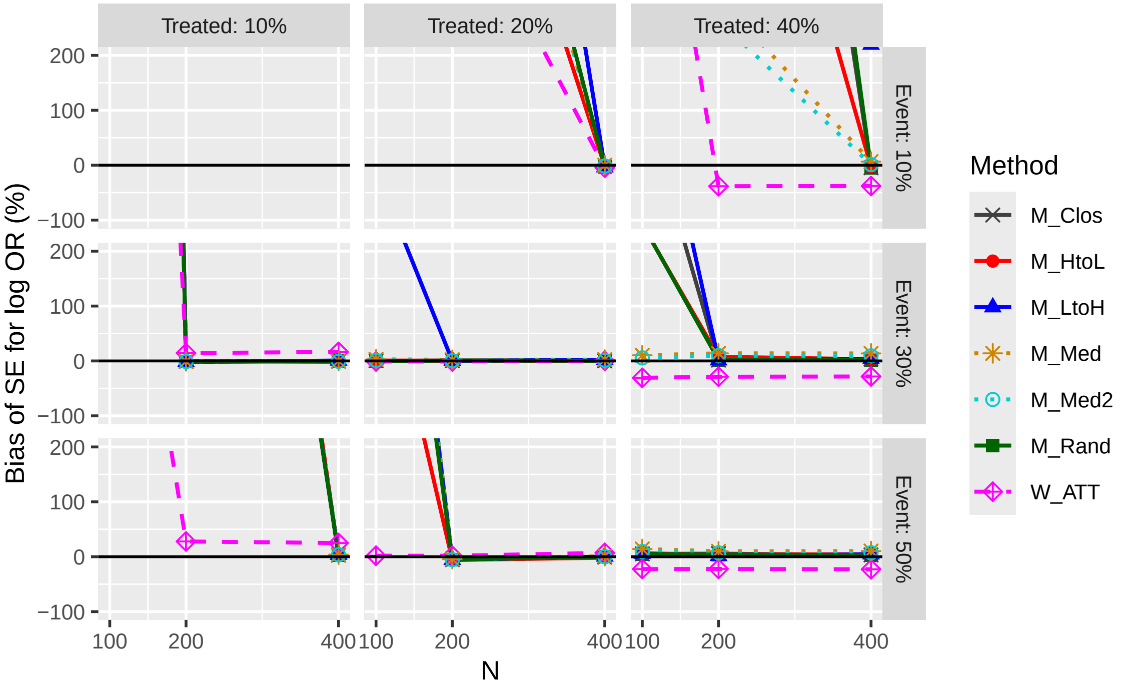

Figure S477. Mean bias of standard error for log odds ratio (multimodal continuous covariate, matching ratio 1:1, true OR: 0.75, c statistic: 0.85, naive inference).

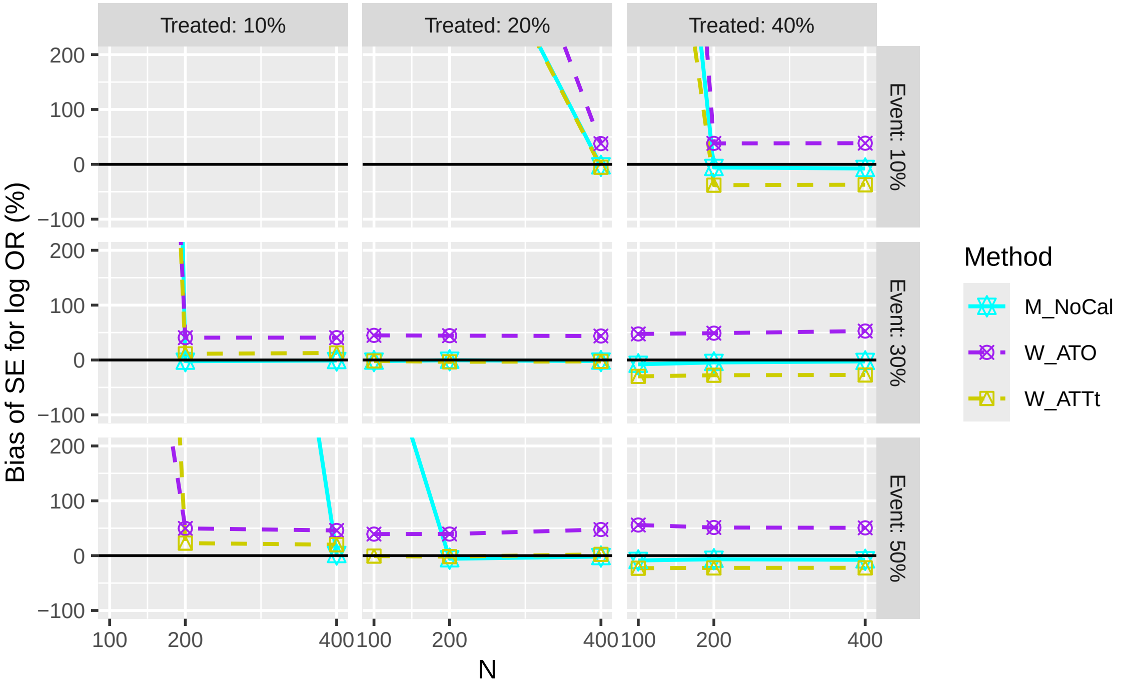

Figure S478. Mean bias of standard error for log odds ratio (multimodal continuous covariate, matching ratio 1:1, true OR: 0.75, c statistic: 0.85, naive inference); other methods.

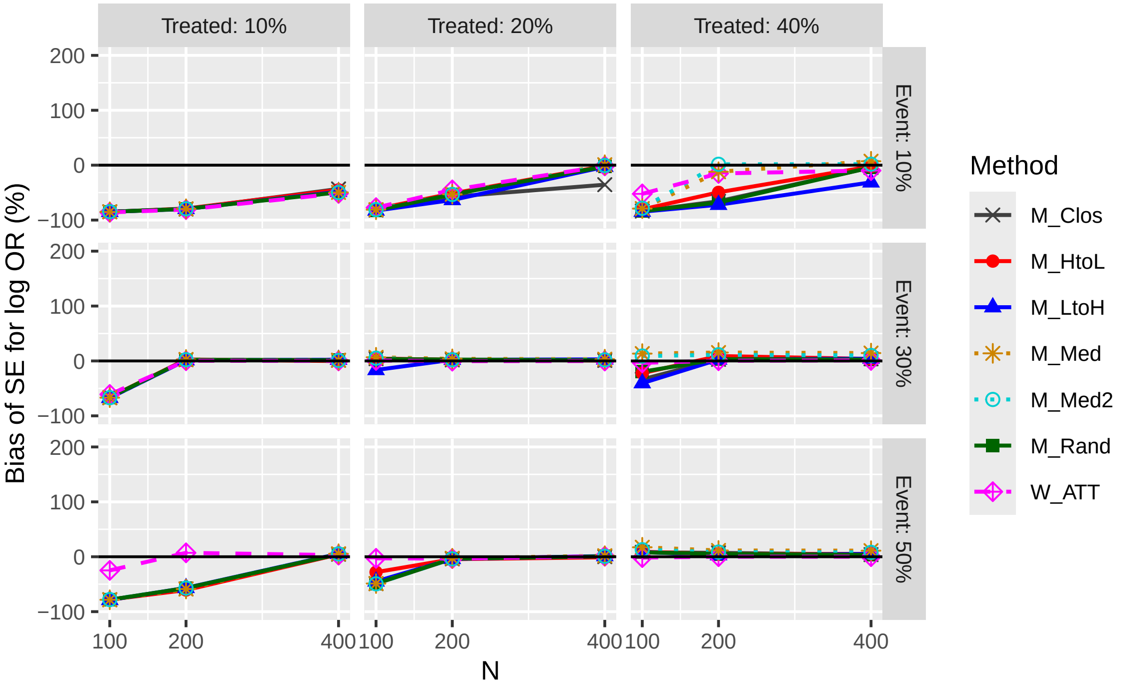

Figure S479. Mean bias of standard error for log odds ratio (multimodal continuous covariate, matching ratio 1:1, true OR: 0.75, c statistic: 0.85, robust inference).

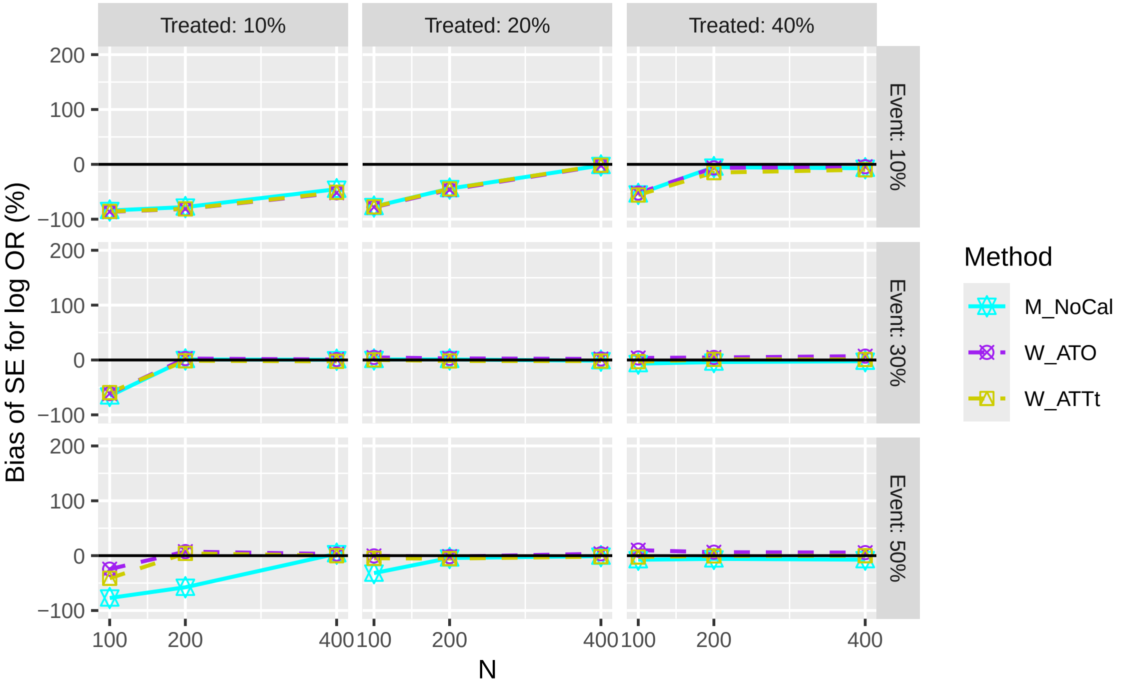

Figure S480. Mean bias of standard error for log odds ratio (multimodal continuous covariate, matching ratio 1:1, true OR: 0.75, c statistic: 0.85, robust inference); other methods.

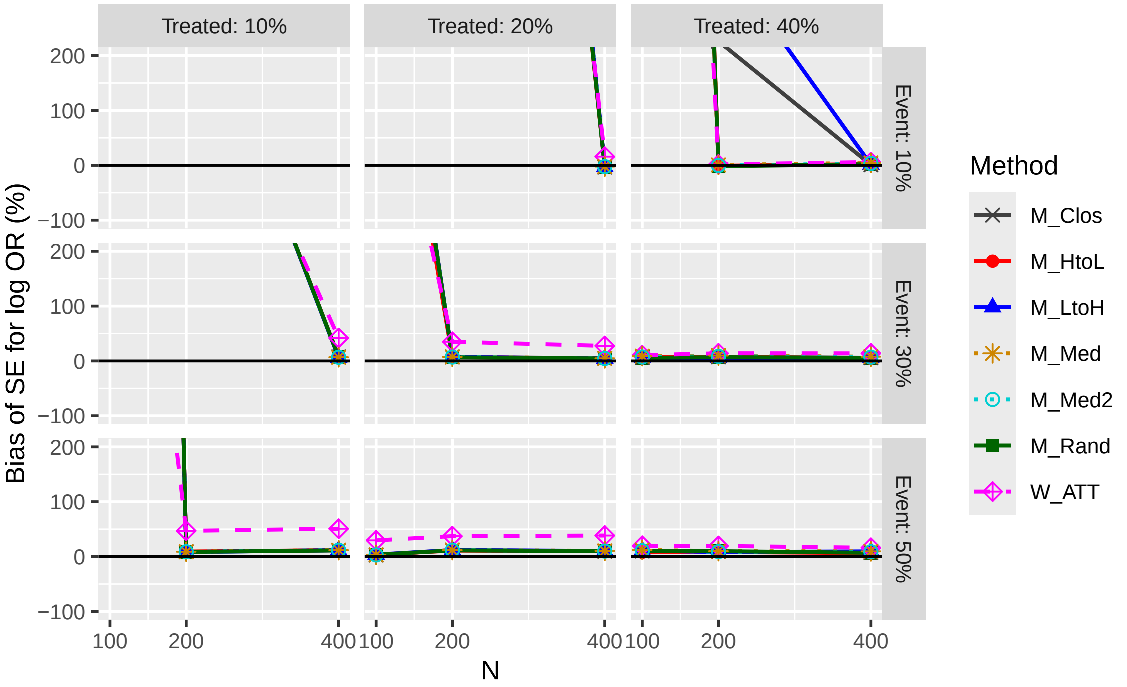

Figure S481. Mean bias of standard error for log odds ratio (multimodal continuous covariate, matching ratio 1:1, true OR: 0.75, c statistic: 0.6, naive inference).

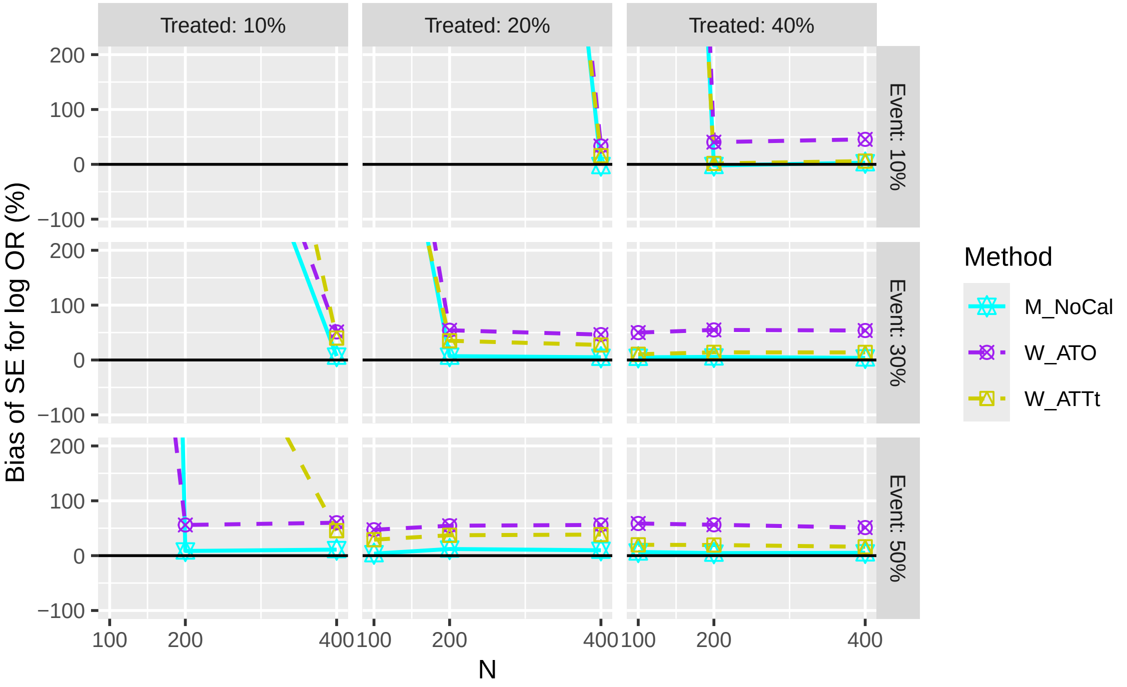

Figure S482. Mean bias of standard error for log odds ratio (multimodal continuous covariate, matching ratio 1:1, true OR: 0.75, c statistic: 0.6, naive inference); other methods.

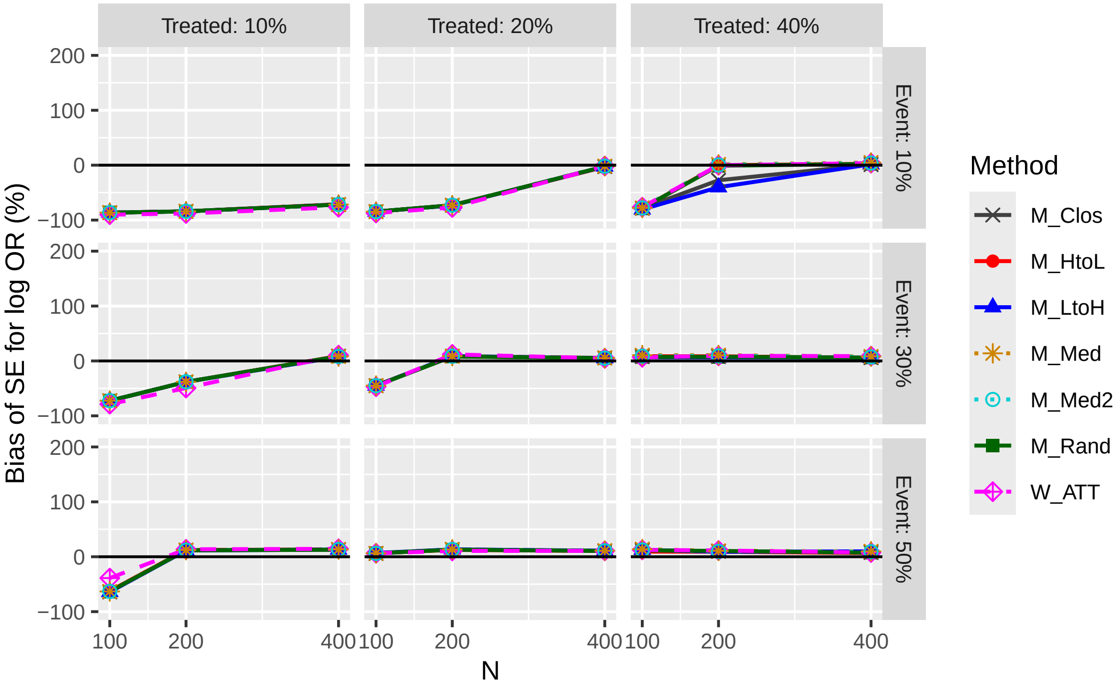

Figure S483. Mean bias of standard error for log odds ratio (multimodal continuous covariate, matching ratio 1:1, true OR: 0.75, c statistic: 0.6, robust inference).

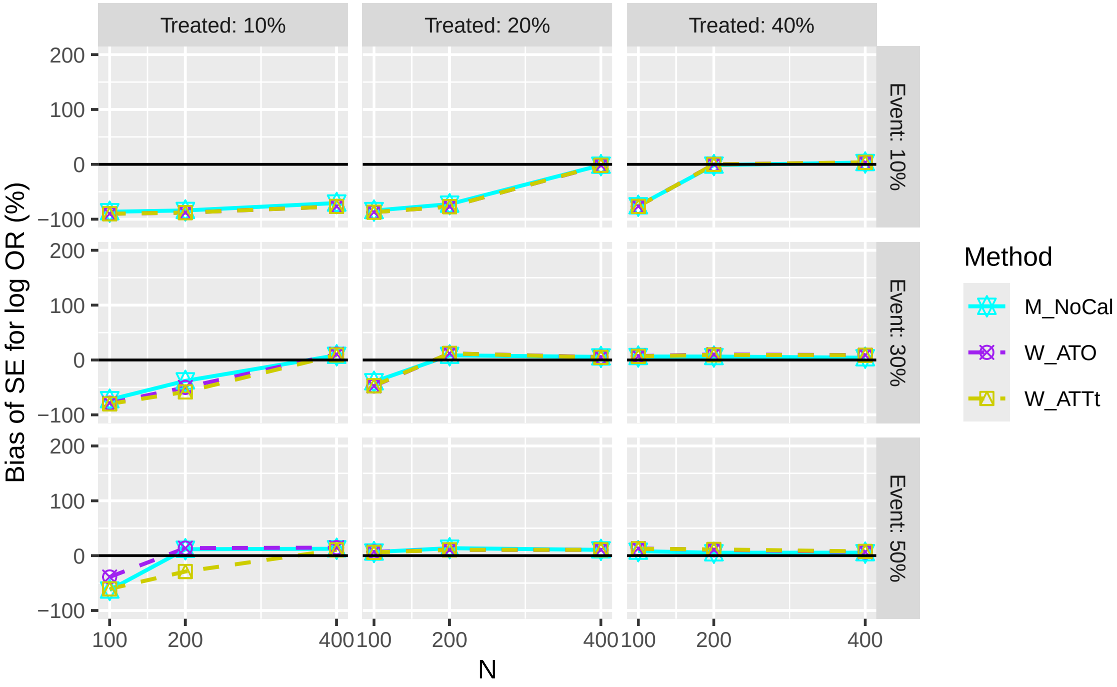

Figure S484. Mean bias of standard error for log odds ratio (multimodal continuous covariate, matching ratio 1:1, true OR: 0.75, c statistic: 0.6, robust inference); other methods.

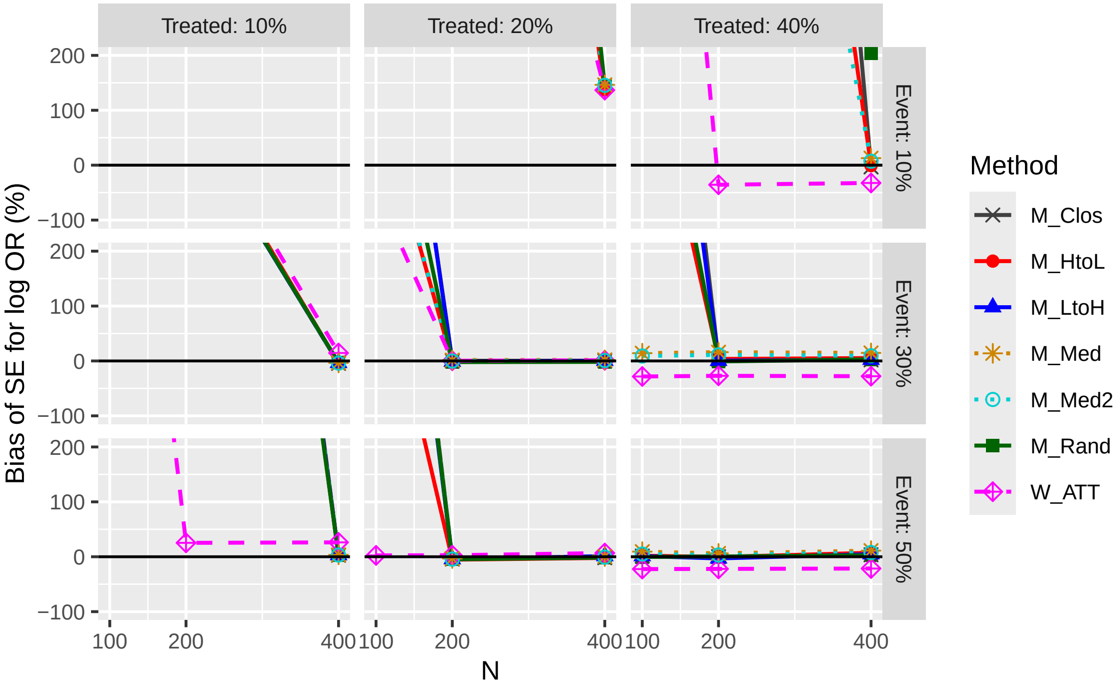

Figure S485. Mean bias of standard error for log odds ratio (multimodal continuous covariate, matching ratio 1:1, true OR: 0.5, c statistic: 0.85, naive inference).

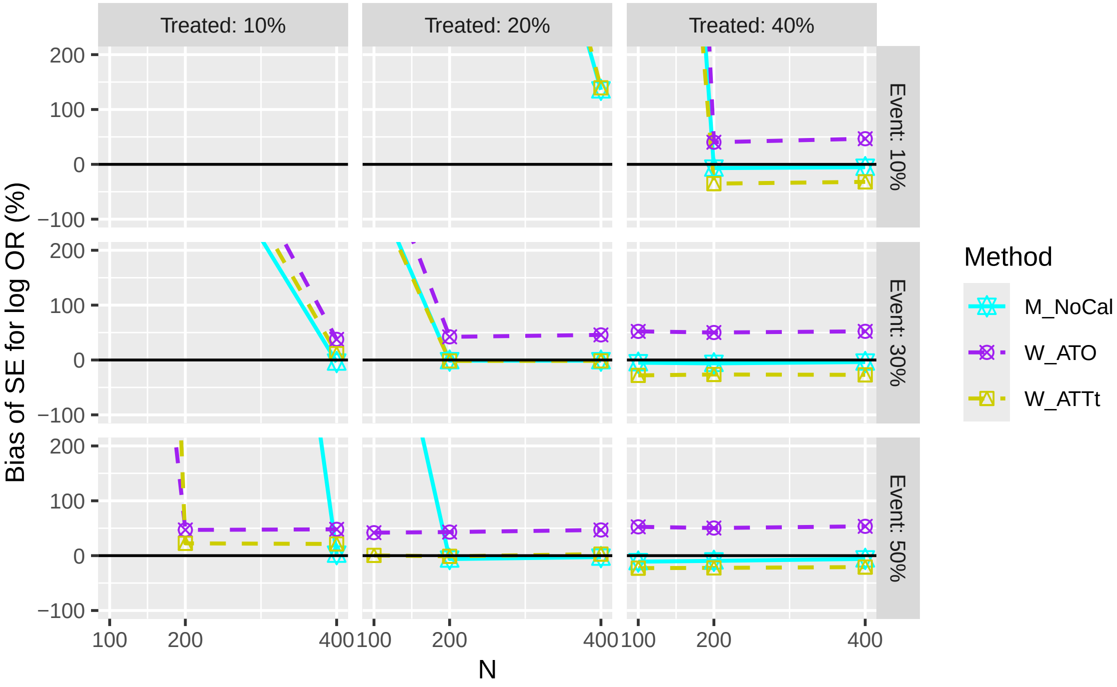

Figure S486. Mean bias of standard error for log odds ratio (multimodal continuous covariate, matching ratio 1:1, true OR: 0.5, c statistic: 0.85, naive inference); other methods.

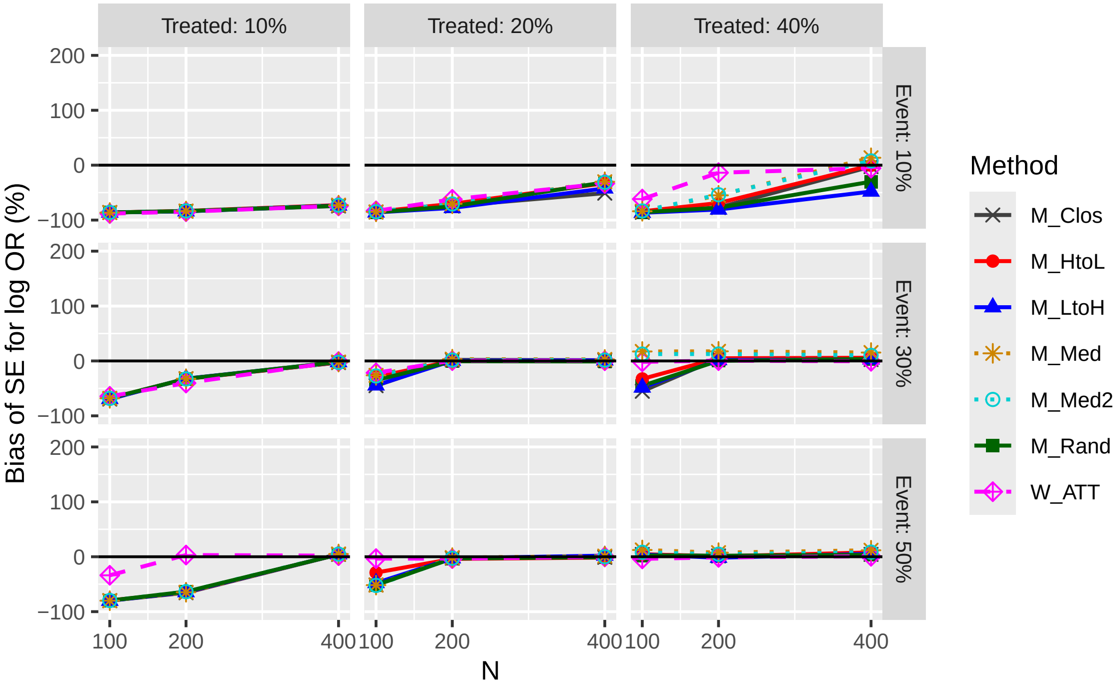

Figure S487. Mean bias of standard error for log odds ratio (multimodal continuous covariate, matching ratio 1:1, true OR: 0.5, c statistic: 0.85, robust inference).

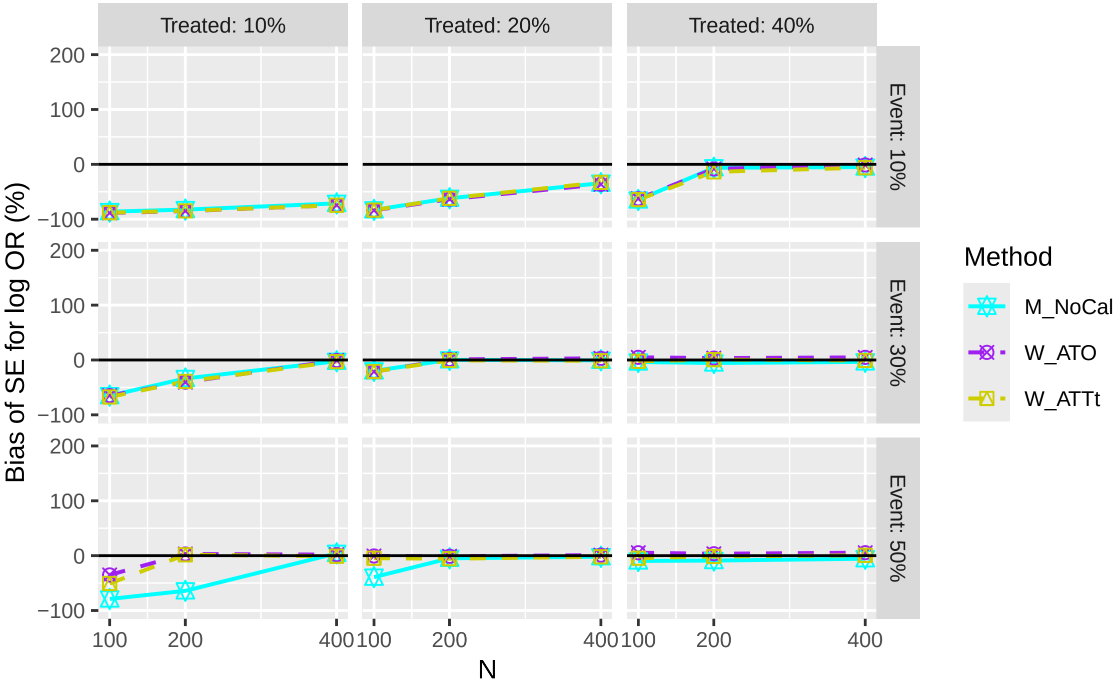

Figure S488. Mean bias of standard error for log odds ratio (multimodal continuous covariate, matching ratio 1:1, true OR: 0.5, c statistic: 0.85, robust inference); other methods.

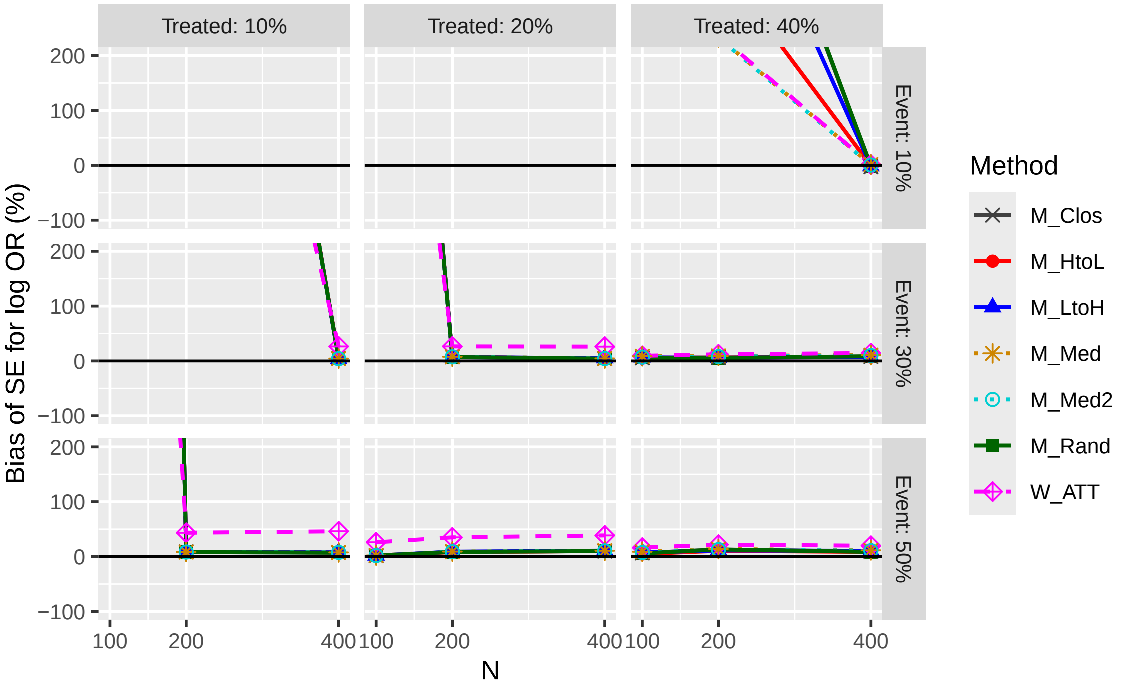

Figure S489. Mean bias of standard error for log odds ratio (multimodal continuous covariate, matching ratio 1:1, true OR: 0.5, c statistic: 0.6, naive inference).

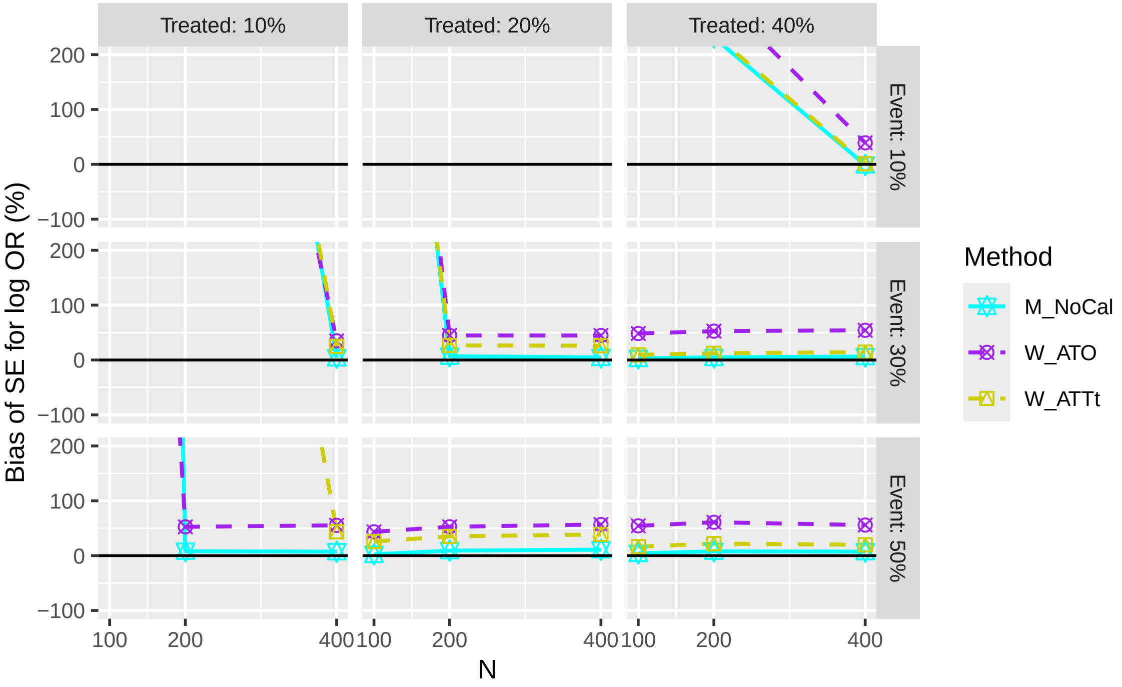

Figure S490. Mean bias of standard error for log odds ratio (multimodal continuous covariate, matching ratio 1:1, true OR: 0.5, c statistic: 0.6, naive inference); other methods.

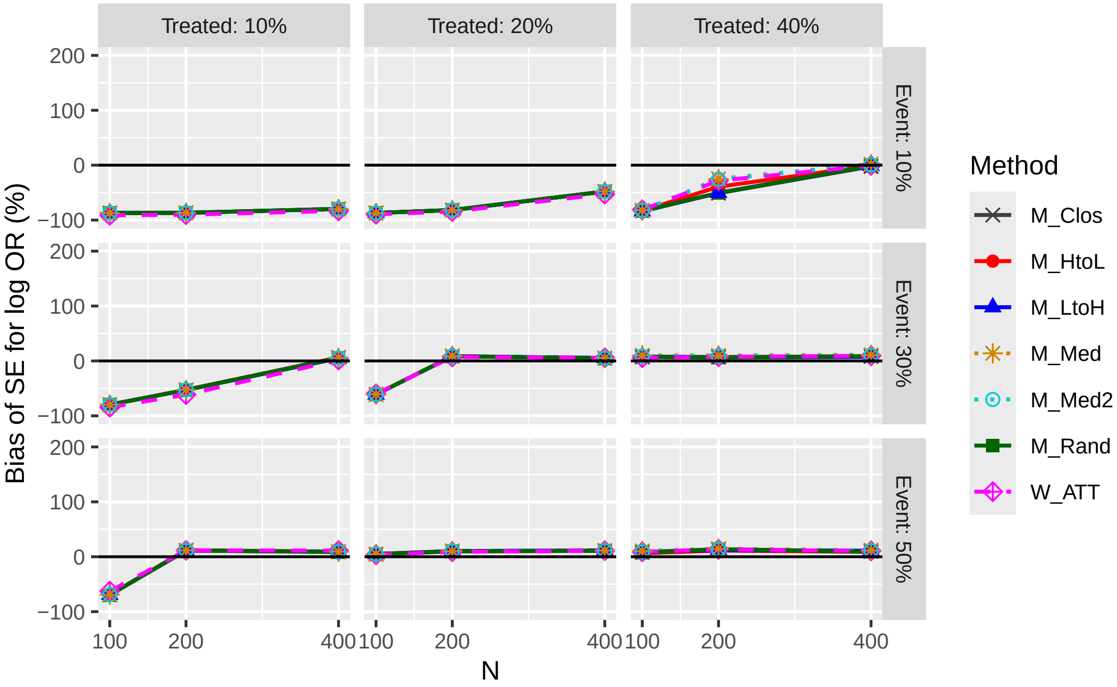

Figure S491. Mean bias of standard error for log odds ratio (multimodal continuous covariate, matching ratio 1:1, true OR: 0.5, c statistic: 0.6, robust inference).

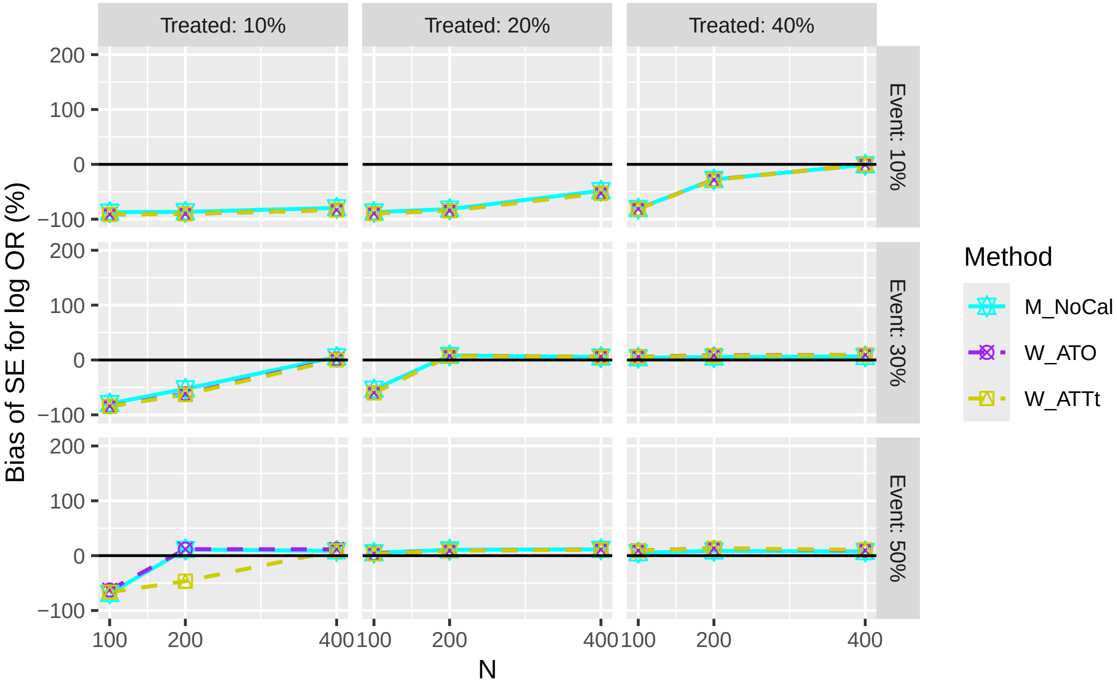

Figure S492. Mean bias of standard error for log odds ratio (multimodal continuous covariate, matching ratio 1:1, true OR: 0.5, c statistic: 0.6, robust inference); other methods.

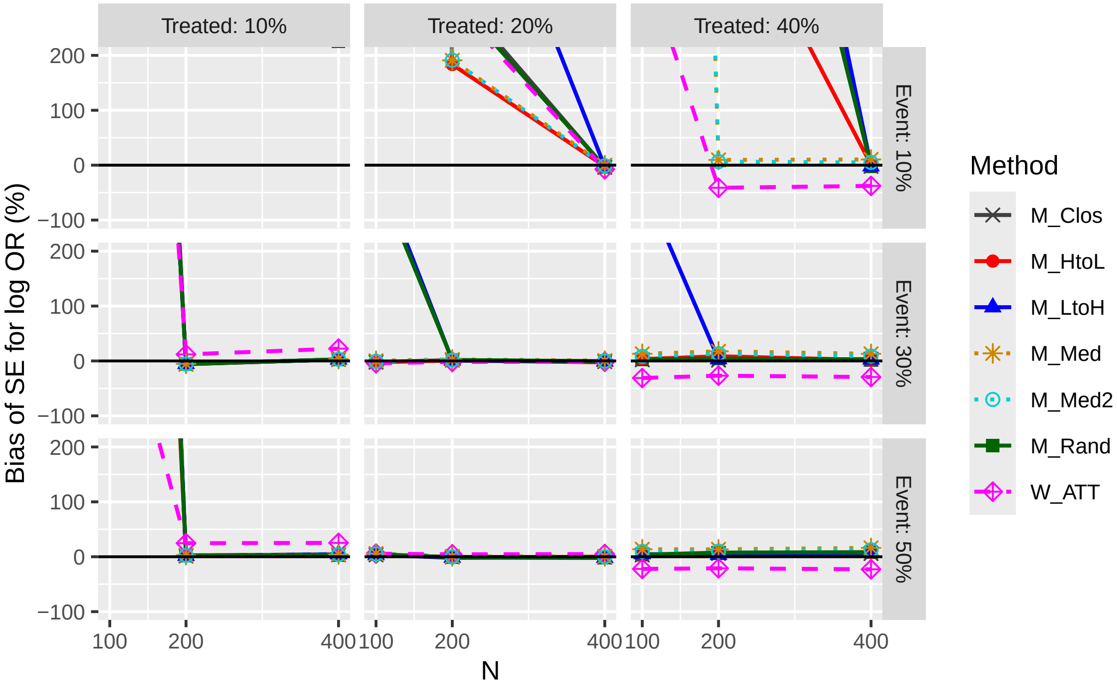

Figure S493. Mean bias of standard error for log odds ratio (multimodal continuous covariate, matching ratio 1:2, true OR: 1, c statistic: 0.85, naive inference).

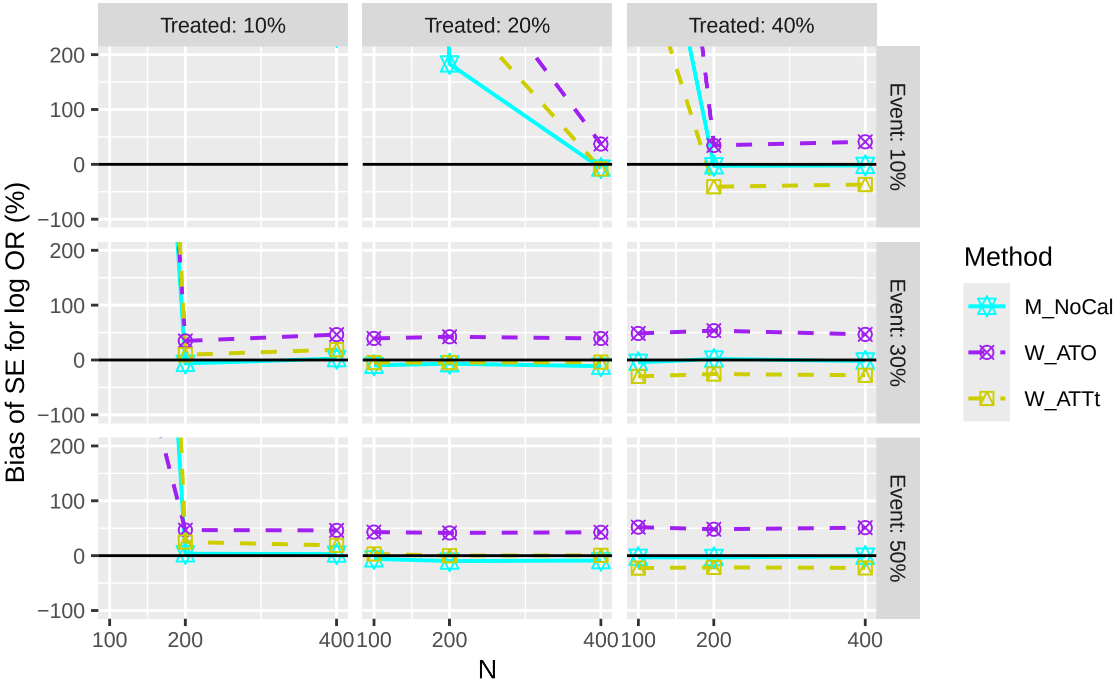

Figure S494. Mean bias of standard error for log odds ratio (multimodal continuous covariate, matching ratio 1:2, true OR: 1, c statistic: 0.85, naive inference); other methods.

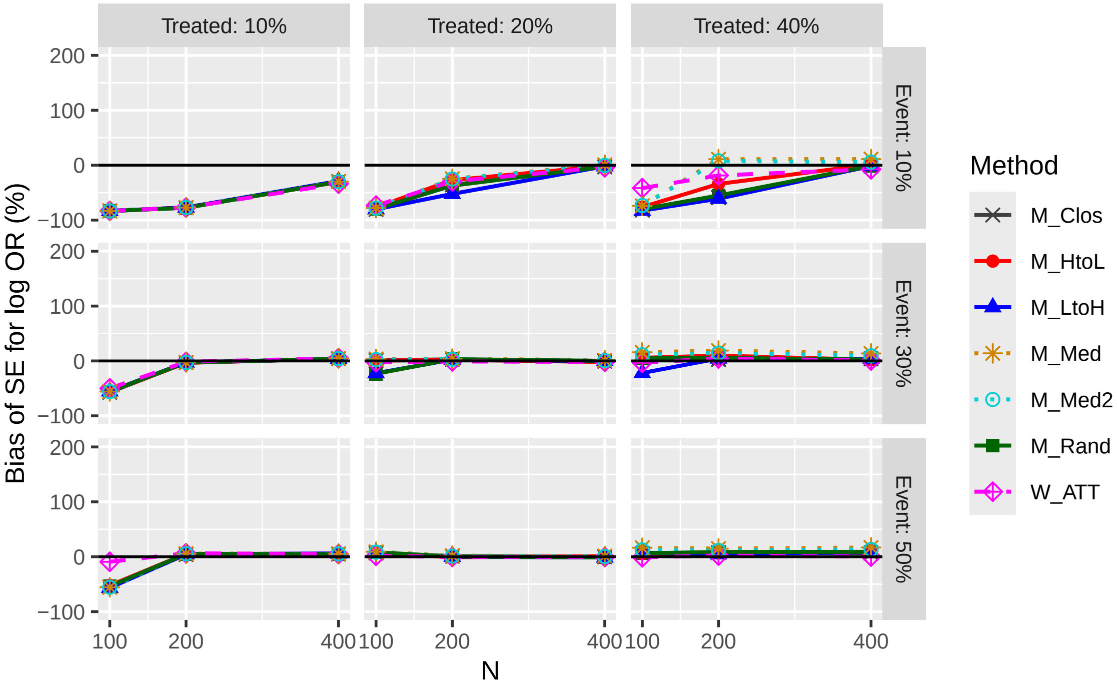

Figure S495. Mean bias of standard error for log odds ratio (multimodal continuous covariate, matching ratio 1:2, true OR: 1, c statistic: 0.85, robust inference).

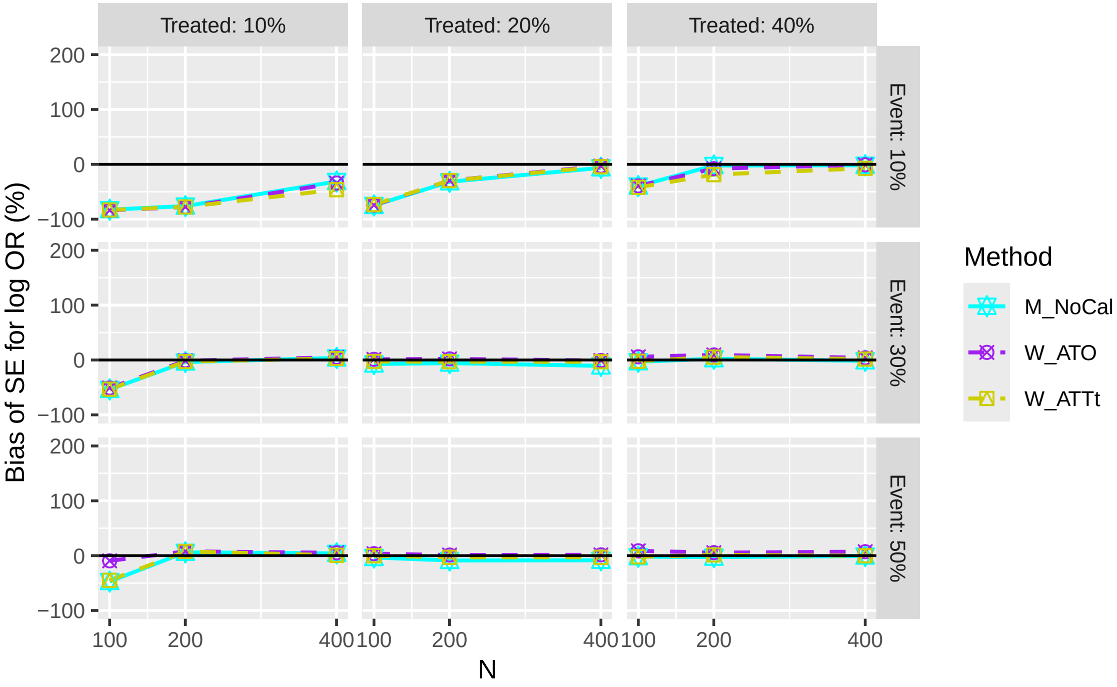

Figure S496. Mean bias of standard error for log odds ratio (multimodal continuous covariate, matching ratio 1:2, true OR: 1, c statistic: 0.85, robust inference); other methods.

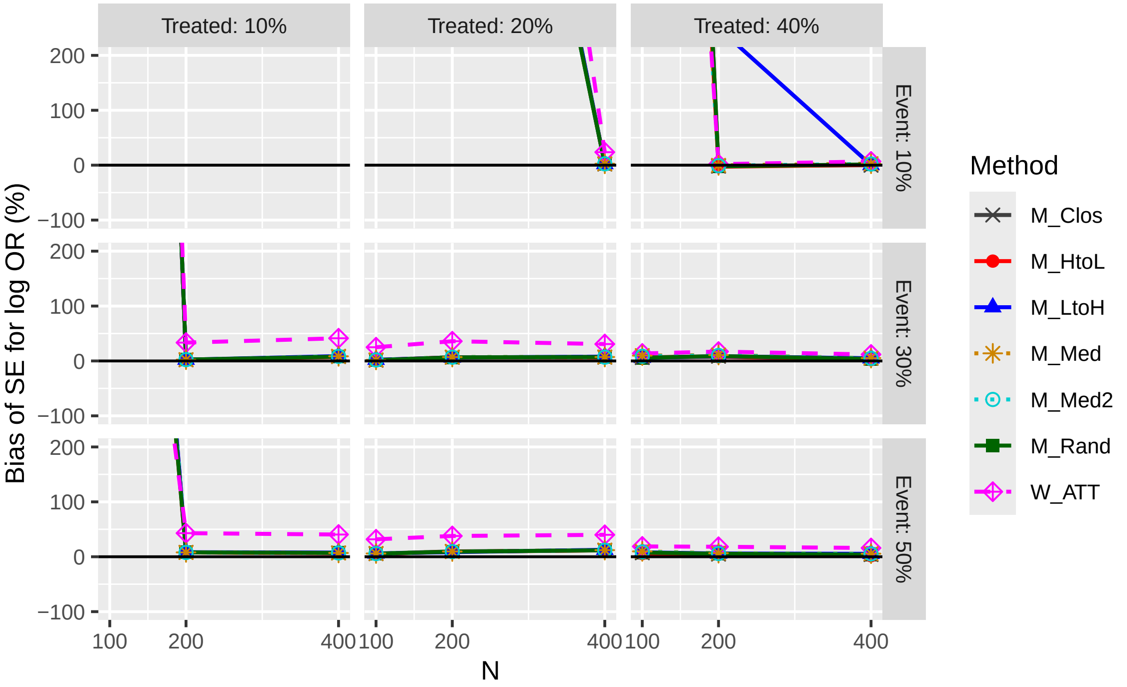

Figure S497. Mean bias of standard error for log odds ratio (multimodal continuous covariate, matching ratio 1:2, true OR: 1, c statistic: 0.6, naive inference).

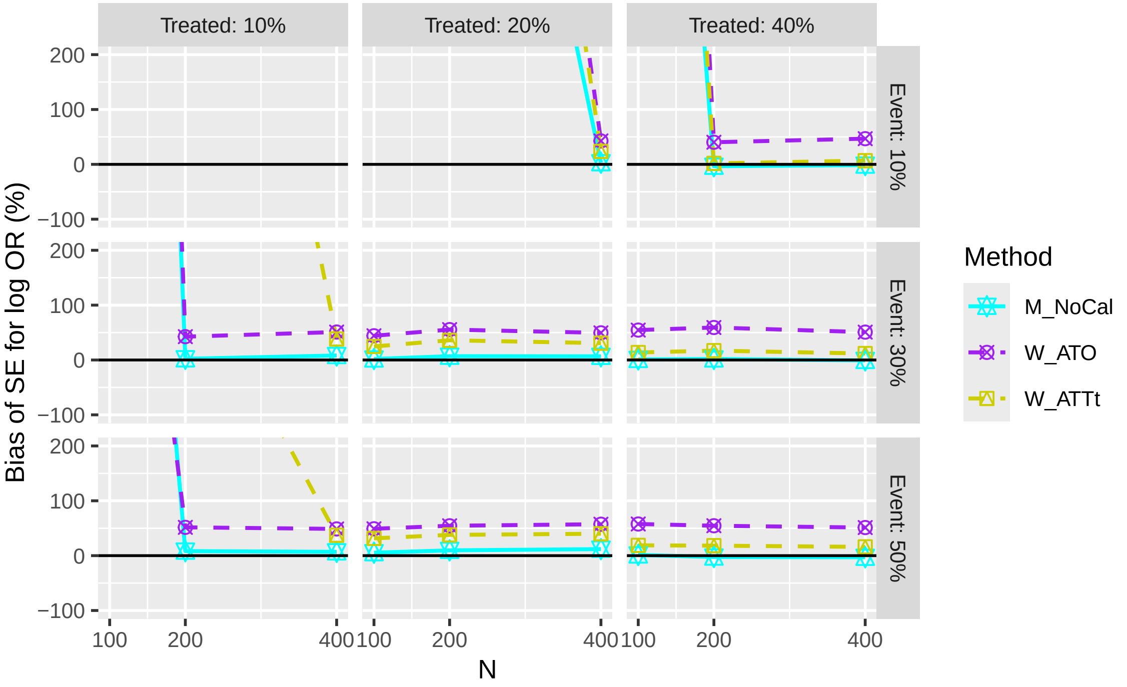

Figure S498. Mean bias of standard error for log odds ratio (multimodal continuous covariate, matching ratio 1:2, true OR: 1, c statistic: 0.6, naive inference); other methods.

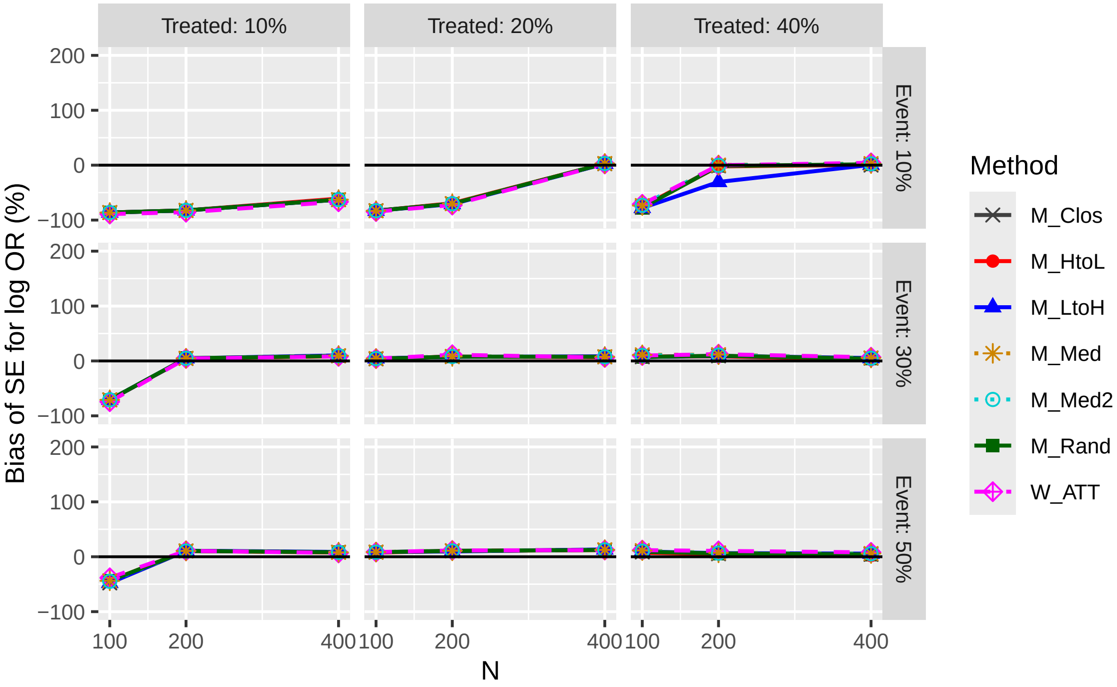

Figure S499. Mean bias of standard error for log odds ratio (multimodal continuous covariate, matching ratio 1:2, true OR: 1, c statistic: 0.6, robust inference).

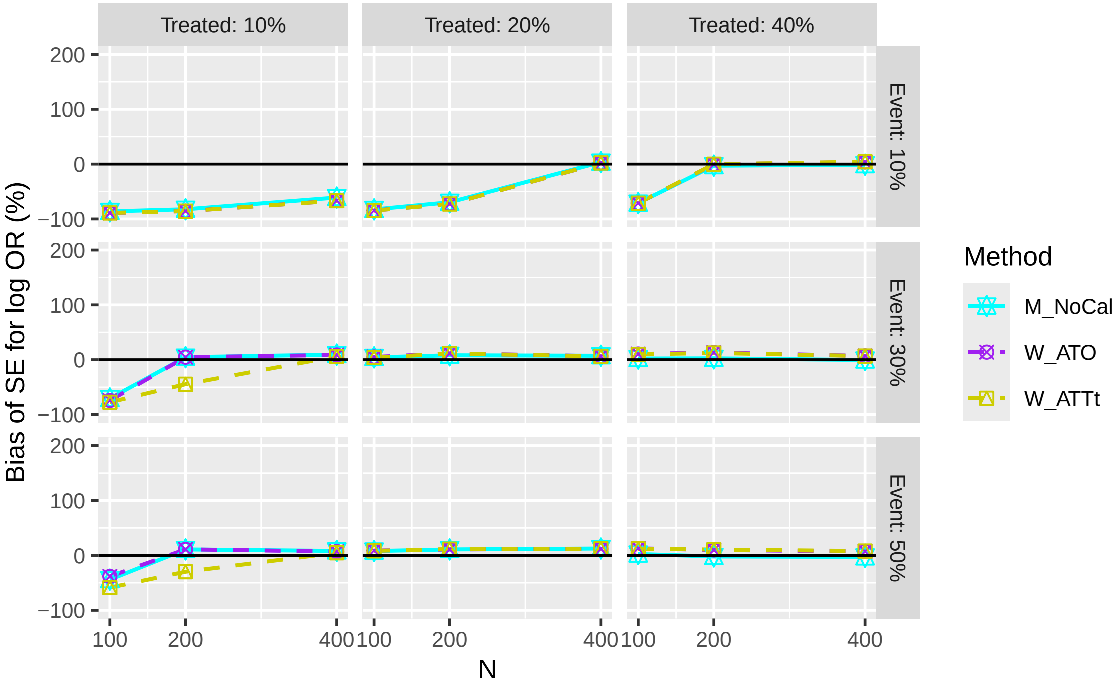

Figure S500. Mean bias of standard error for log odds ratio (multimodal continuous covariate, matching ratio 1:2, true OR: 1, c statistic: 0.6, robust inference); other methods.

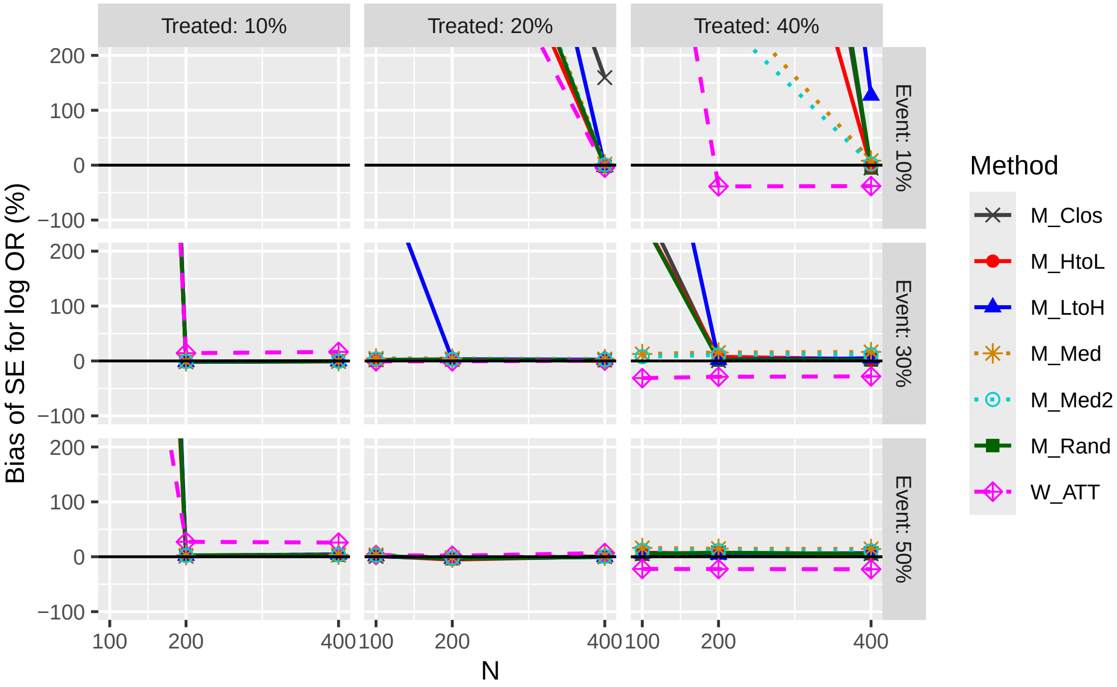

Figure S501. Mean bias of standard error for log odds ratio (multimodal continuous covariate, matching ratio 1:2, true OR: 0.75, c statistic: 0.85, naive inference).

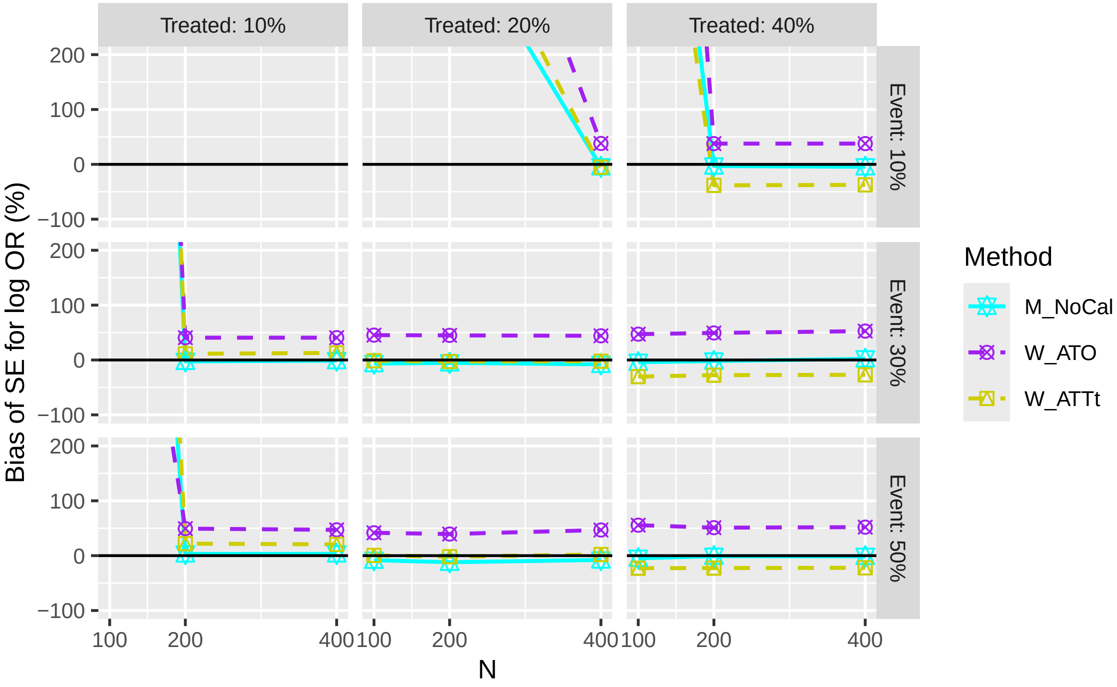

Figure S502. Mean bias of standard error for log odds ratio (multimodal continuous covariate, matching ratio 1:2, true OR: 0.75, c statistic: 0.85, naive inference); other methods.

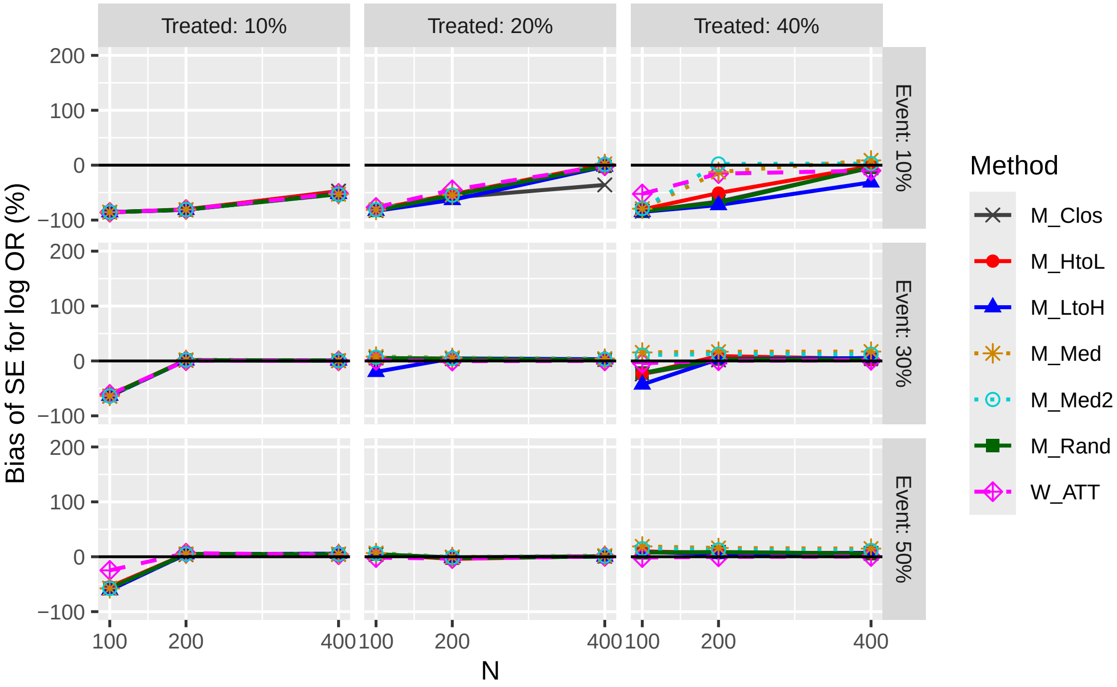

Figure S503. Mean bias of standard error for log odds ratio (multimodal continuous covariate, matching ratio 1:2, true OR: 0.75, c statistic: 0.85, robust inference).

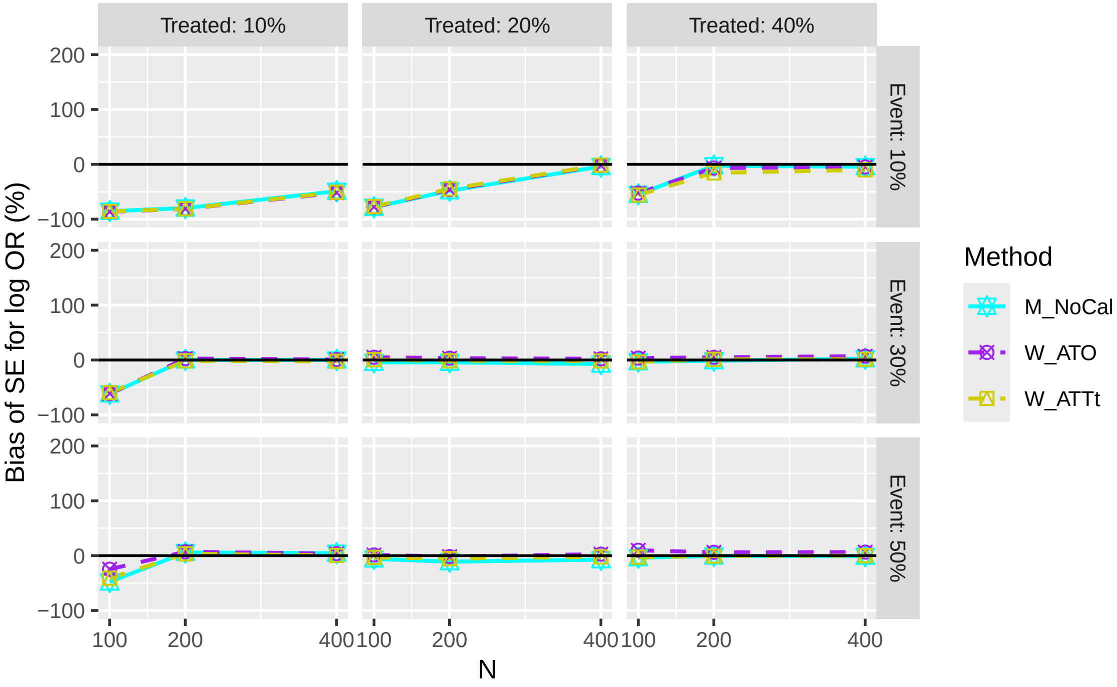

Figure S504. Mean bias of standard error for log odds ratio (multimodal continuous covariate, matching ratio 1:2, true OR: 0.75, c statistic: 0.85, robust inference); other methods.

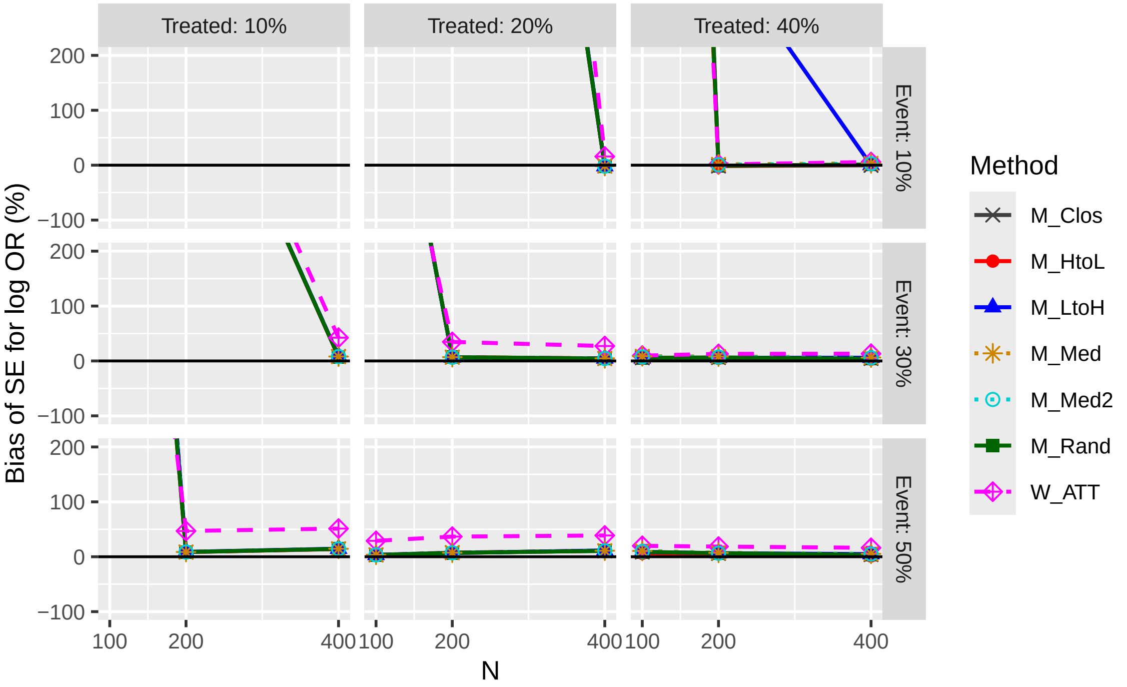

Figure S505. Mean bias of standard error for log odds ratio (multimodal continuous covariate, matching ratio 1:2, true OR: 0.75, c statistic: 0.6, naive inference).

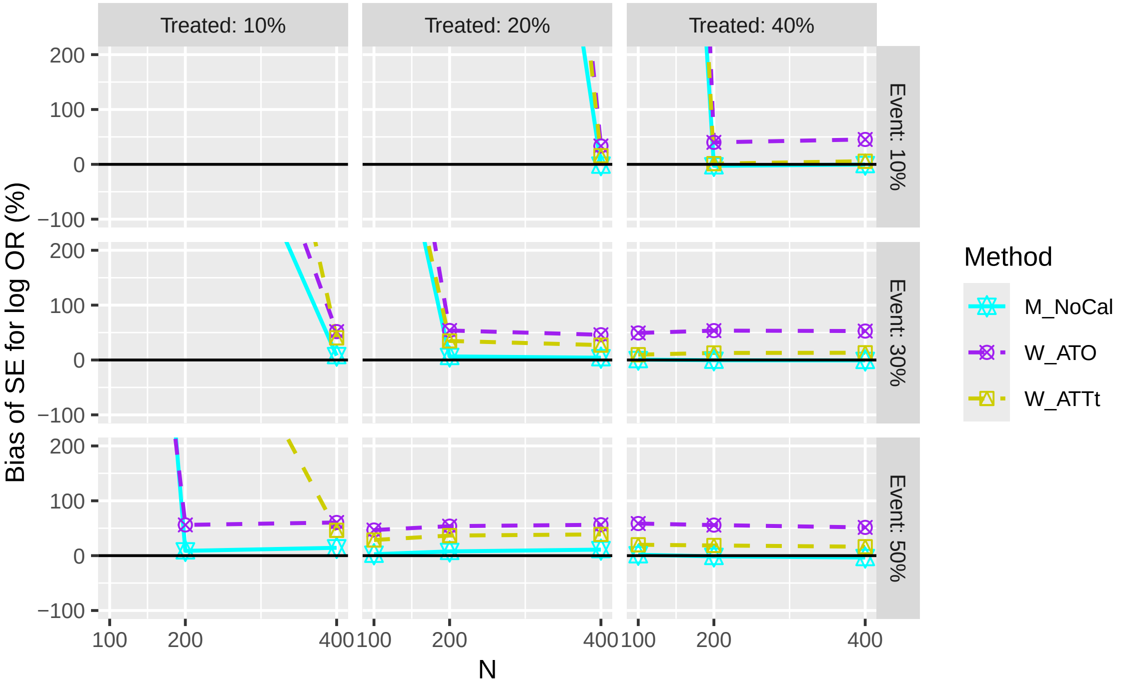

Figure S506. Mean bias of standard error for log odds ratio (multimodal continuous covariate, matching ratio 1:2, true OR: 0.75, c statistic: 0.6, naive inference); other methods.

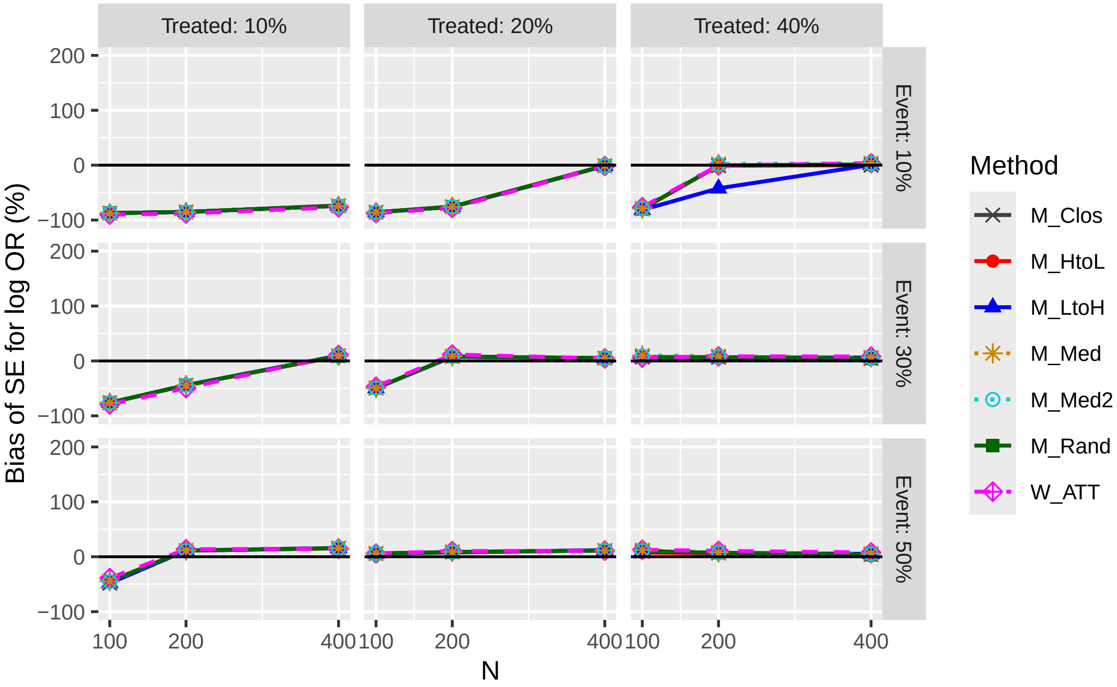

Figure S507. Mean bias of standard error for log odds ratio (multimodal continuous covariate, matching ratio 1:2, true OR: 0.75, c statistic: 0.6, robust inference).

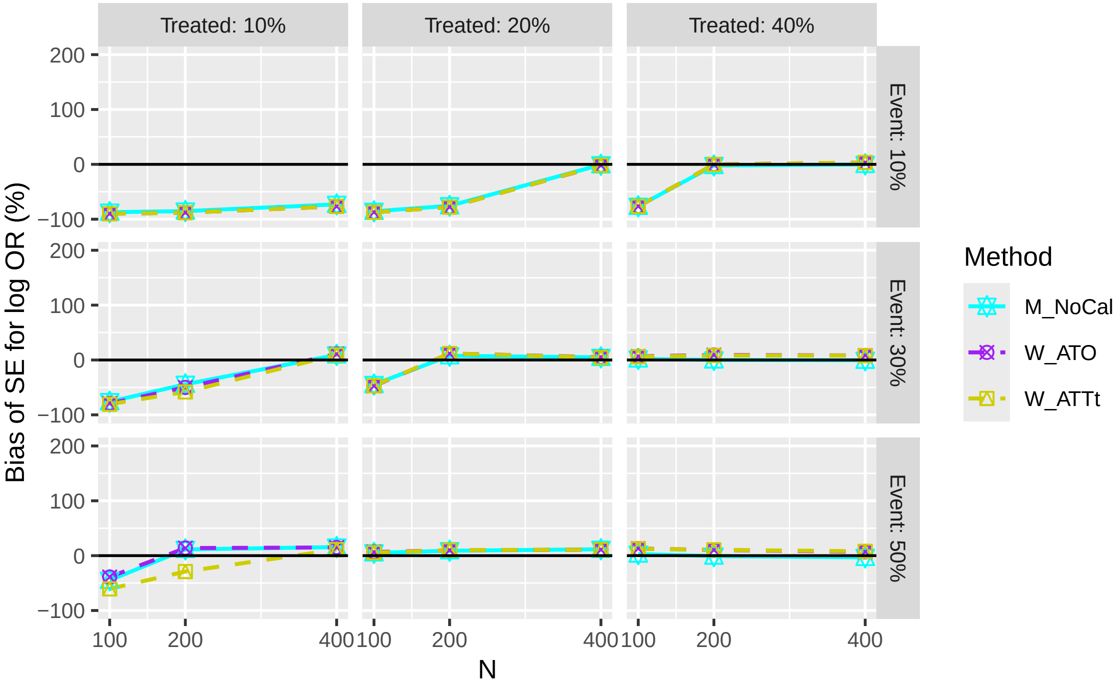

Figure S508. Mean bias of standard error for log odds ratio (multimodal continuous covariate, matching ratio 1:2, true OR: 0.75, c statistic: 0.6, robust inference); other methods.

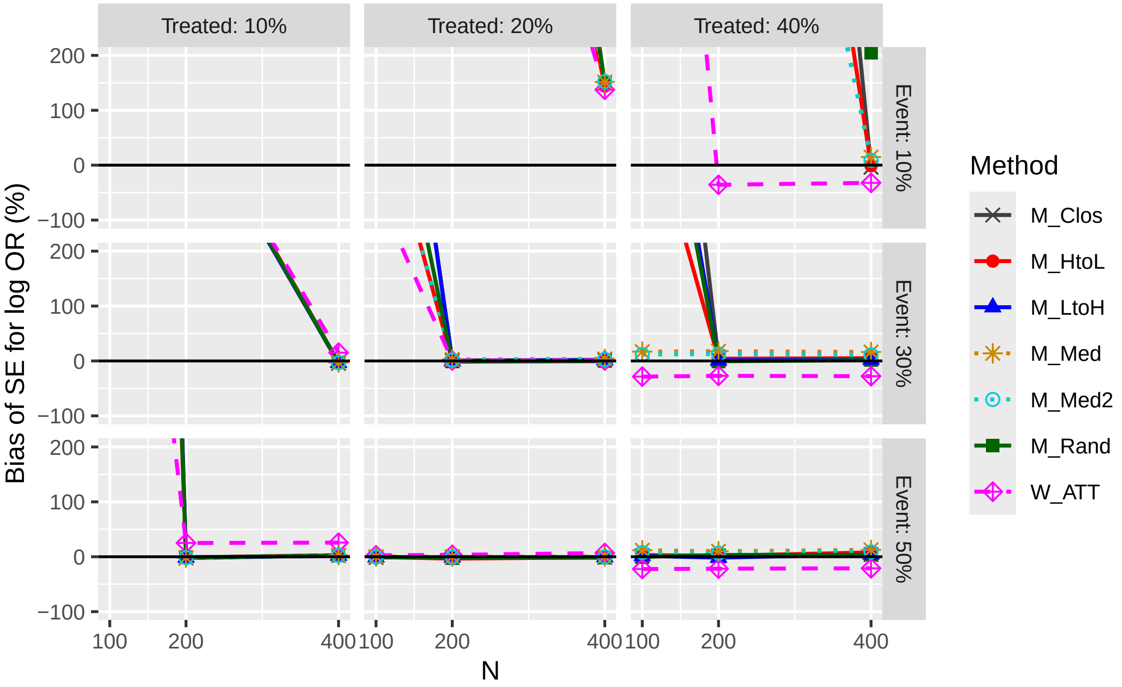

Figure S509. Mean bias of standard error for log odds ratio (multimodal continuous covariate, matching ratio 1:2, true OR: 0.5, c statistic: 0.85, naive inference).

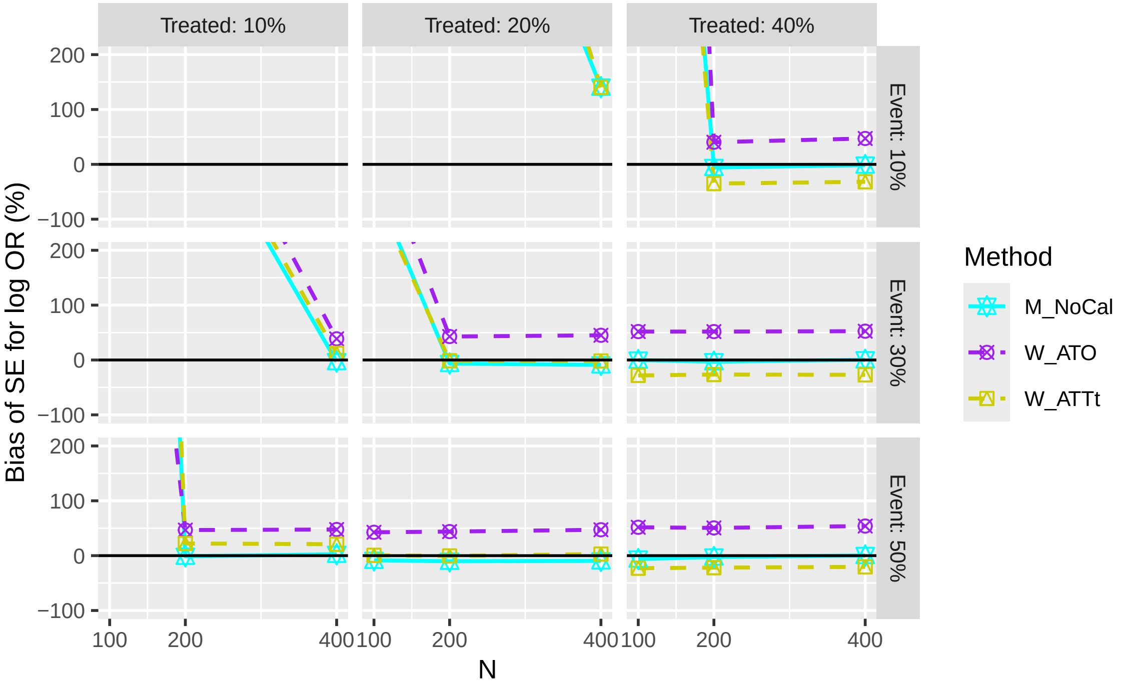

Figure S510. Mean bias of standard error for log odds ratio (multimodal continuous covariate, matching ratio 1:2, true OR: 0.5, c statistic: 0.85, naive inference); other methods.

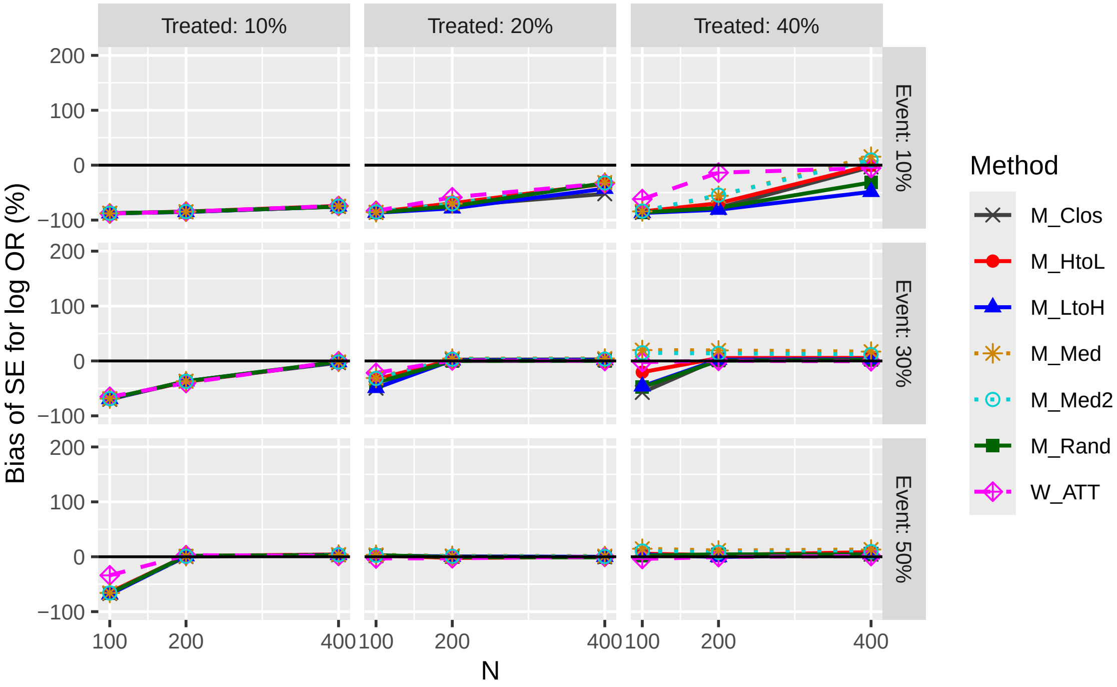

Figure S511. Mean bias of standard error for log odds ratio (multimodal continuous covariate, matching ratio 1:2, true OR: 0.5, c statistic: 0.85, robust inference).

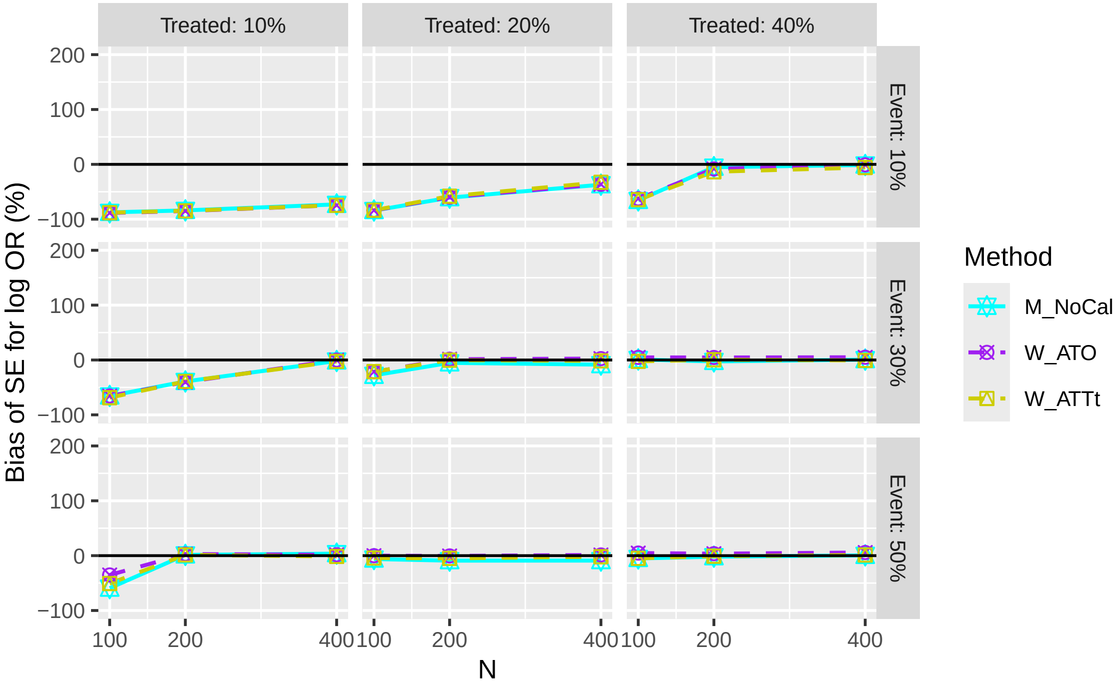

Figure S512. Mean bias of standard error for log odds ratio (multimodal continuous covariate, matching ratio 1:2, true OR: 0.5, c statistic: 0.85, robust inference); other methods.

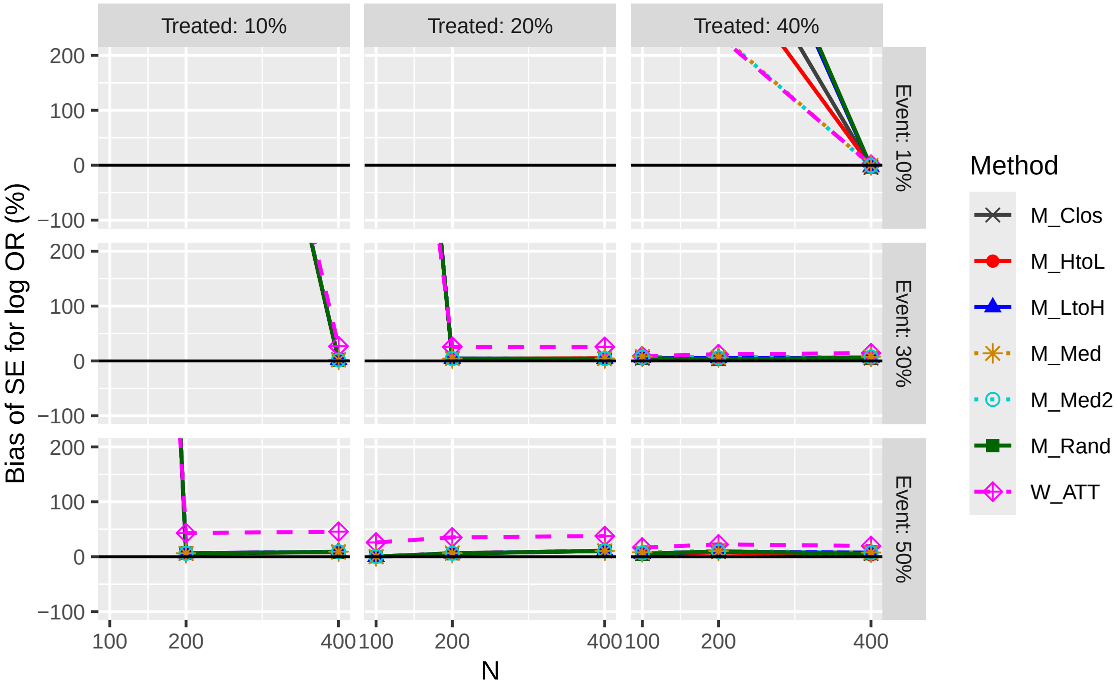

Figure S513. Mean bias of standard error for log odds ratio (multimodal continuous covariate, matching ratio 1:2, true OR: 0.5, c statistic: 0.6, naive inference).

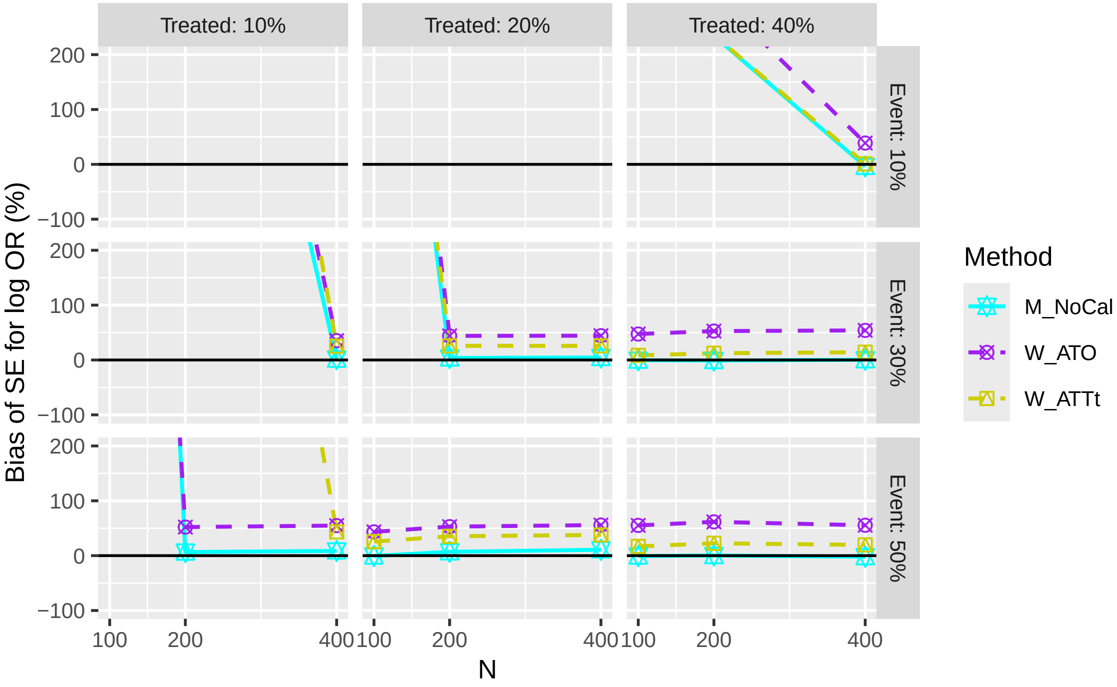

Figure S514. Mean bias of standard error for log odds ratio (multimodal continuous covariate, matching ratio 1:2, true OR: 0.5, c statistic: 0.6, naive inference); other methods.

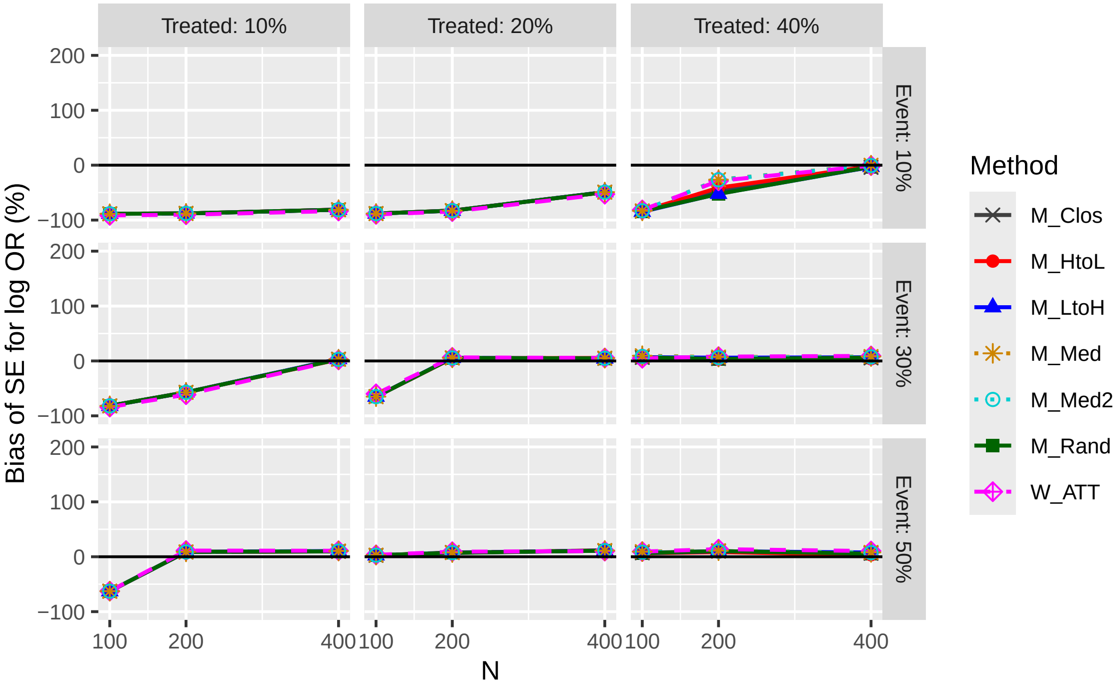

Figure S515. Mean bias of standard error for log odds ratio (multimodal continuous covariate, matching ratio 1:2, true OR: 0.5, c statistic: 0.6, robust inference).

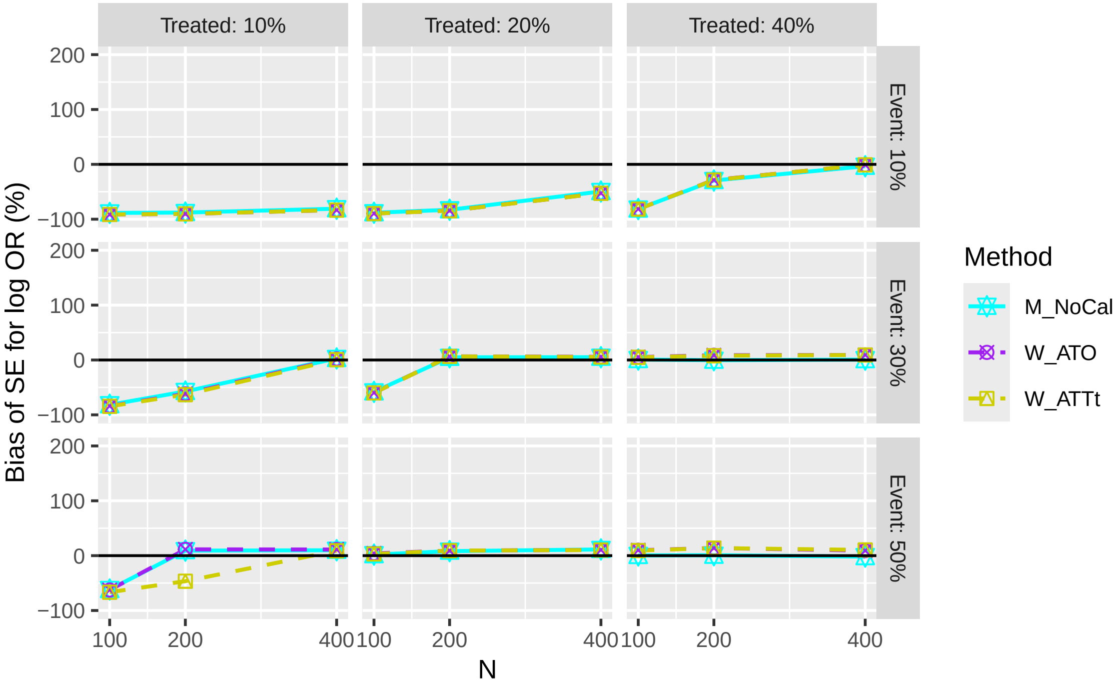

Figure S516. Mean bias of standard error for log odds ratio (multimodal continuous covariate, matching ratio 1:2, true OR: 0.5, c statistic: 0.6, robust inference); other methods.
