# Supplementary material for: Instability of estimation results based on caliper matching with propensity scores
Source: PLoS One. 2025 Jun 6;20(6):e0325317. doi: 10.1371/journal.pone.0325317 (PMC12143538; doi:10.1371/journal.pone.0325317)
Supplement: S2 File — (PDF) [file pone.0325317.s002.pdf]

Supplementary Material for "Instability of Estimation Results Based on Caliper Matching with Propensity Scores"

All simulation results (caliper: 15%)

Kazushi Maruo, Yusuke Yamaguchi, Ryota Ishii, Masahiko Gosho

Contents

S7. Median width of OR for random order matching (caliper: 15%) 1
S8. Median bias for OR (caliper: 15%) 5
S9. IQR for OR (precision, caliper: 15%)) 23
S10. Median absolute difference of OR for 10% data addition (caliper: 15%) 41
S11. Coverage probability of confidence interval for OR (caliper: 15%) 59
S12. Mean percentage bias of standard error for log odds ratio (caliper: 15%) 95

S7. Median width of OR for random order matching (caliper: 15%)

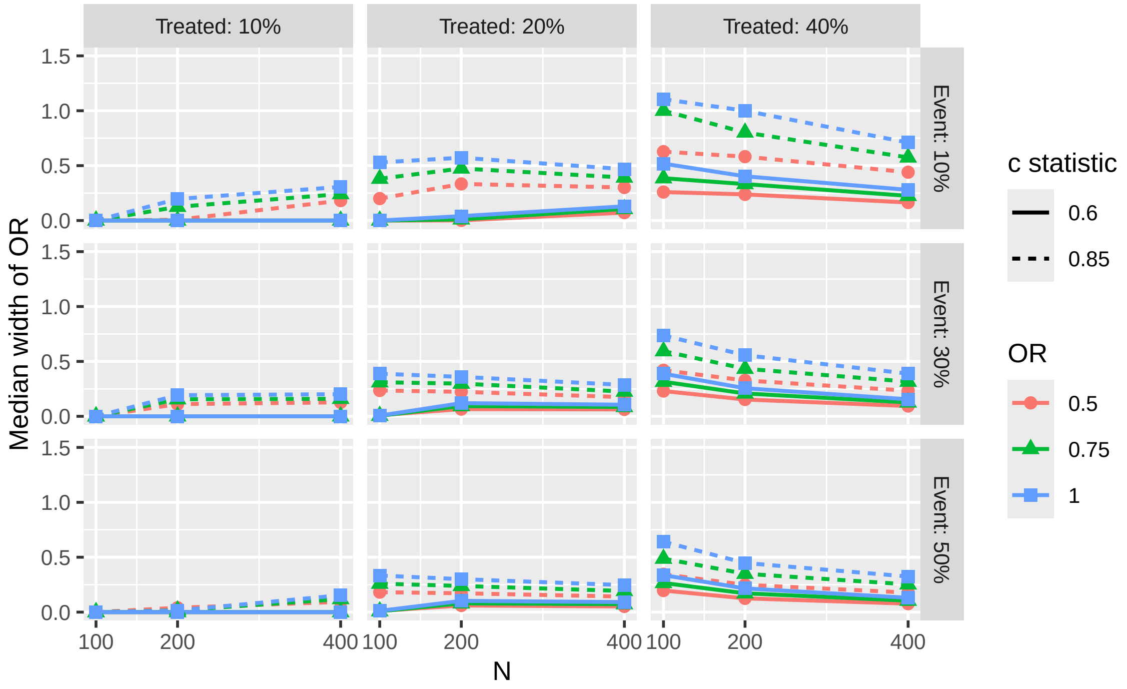

Figure S517. Median width of OR for random order matching (unimodal continuous covariate, with caliper, matching ratio 1:1)

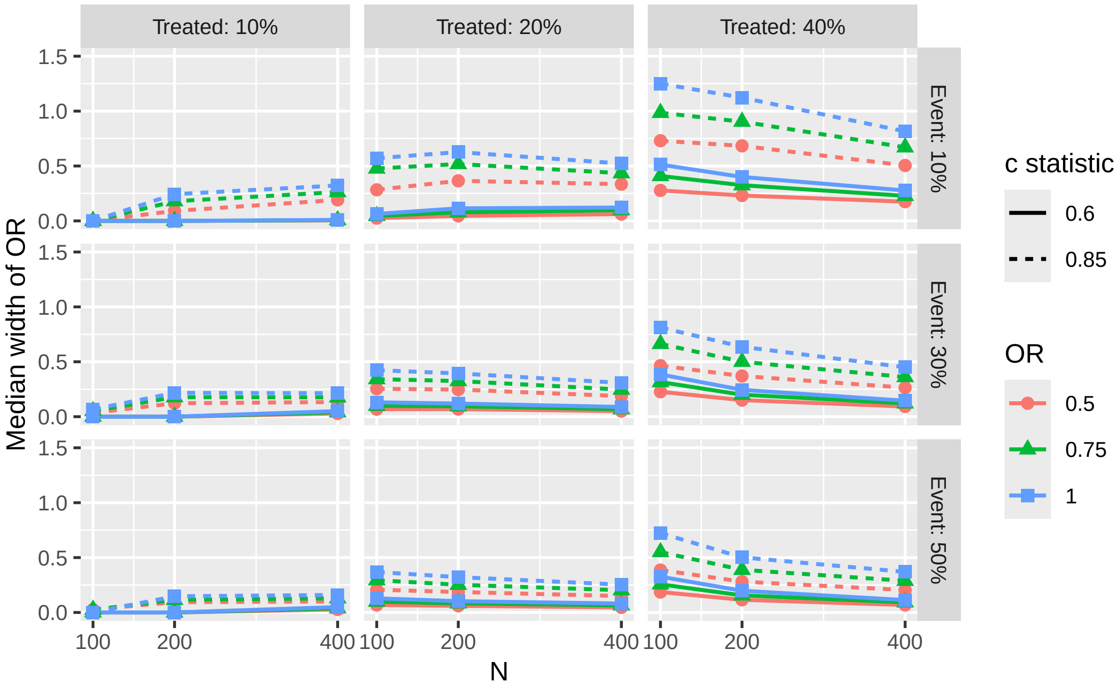

Figure S518. Median width of OR for random order matching (unimodal continuous covariate, with caliper, matching ratio 1:2)

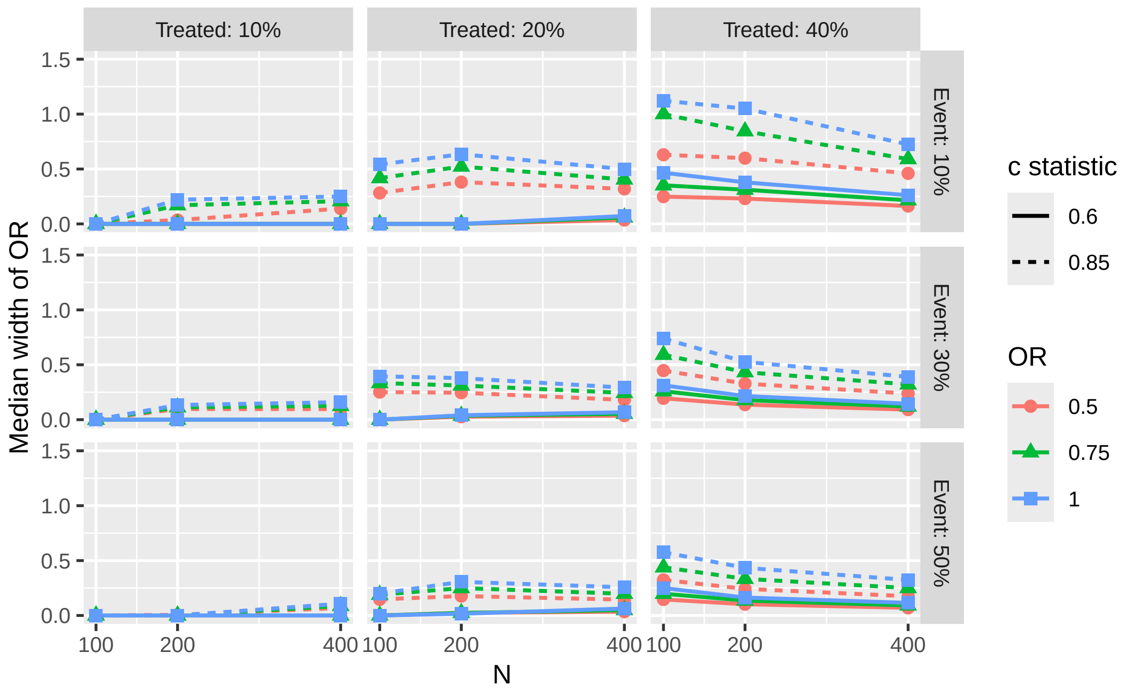

Figure S519. Median width of OR for random order matching (categorical covariate, with caliper, matching ratio 1:1)

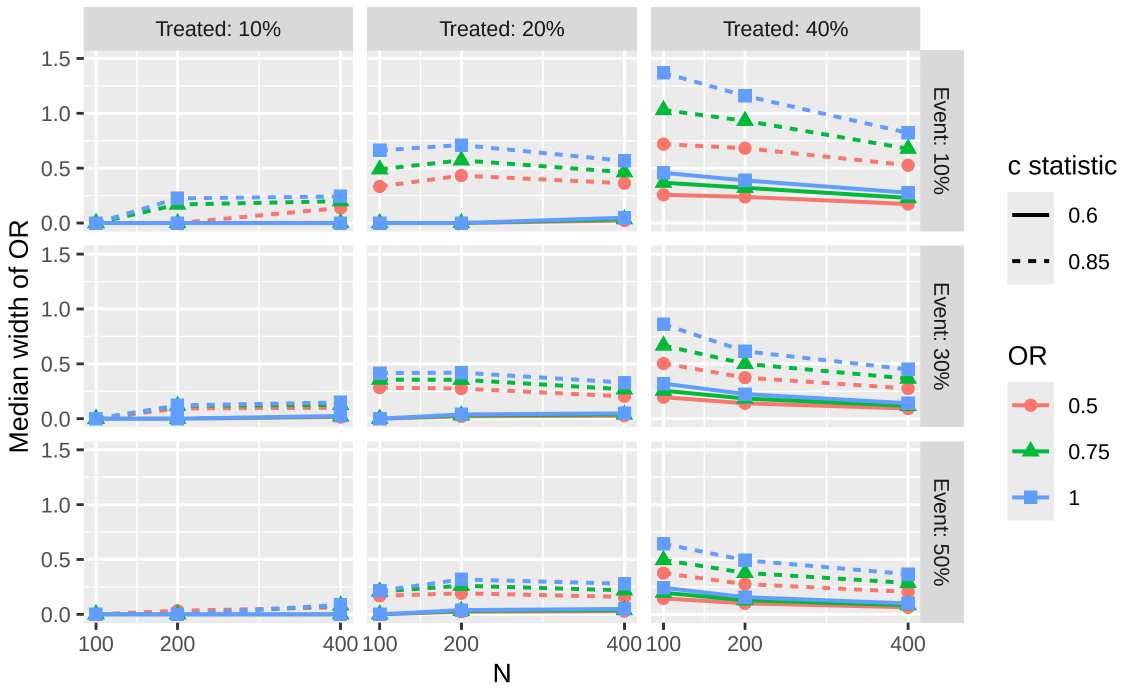

Figure S520. Median width of OR for random order matching (categorical covariate, with caliper, matching ratio 1:2)

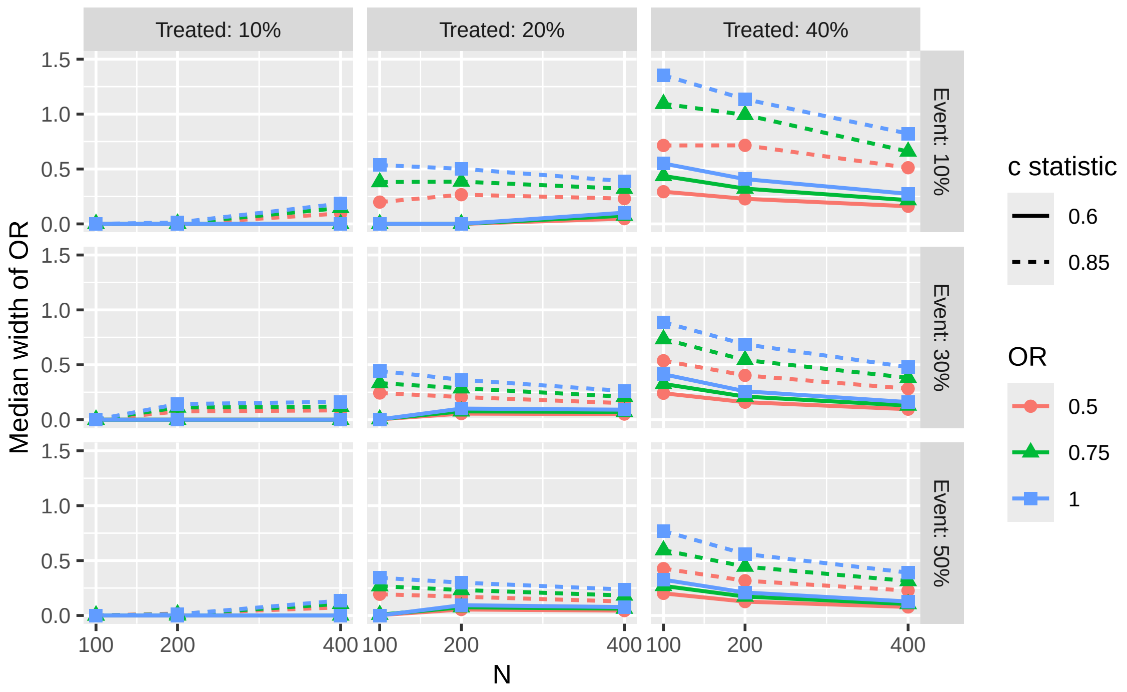

Figure S521. Median width of OR for random order matching (multimodal continuous covariate, with caliper, matching ratio 1:1)

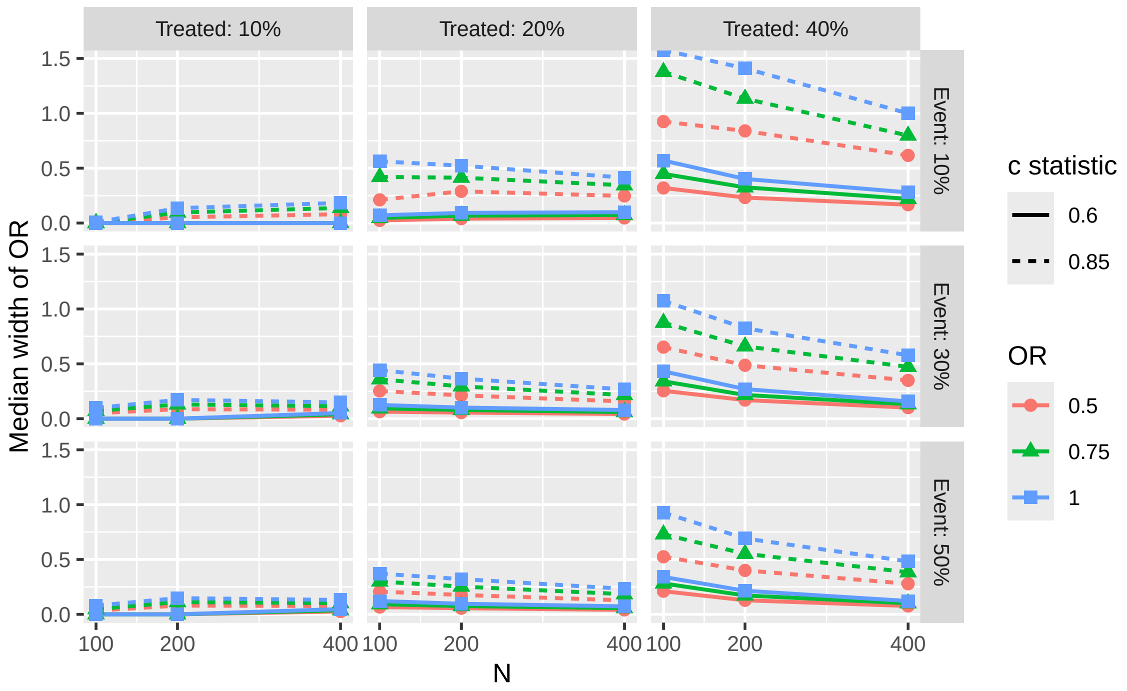

Figure S522. Median width of OR for random order matching (multimodal continuous covariate, with caliper, matching ratio 1:2)

S8. Median bias for OR (caliper: 15%)

For M_NoCal method, the bias may be too large to be within the range displayed in the graph.

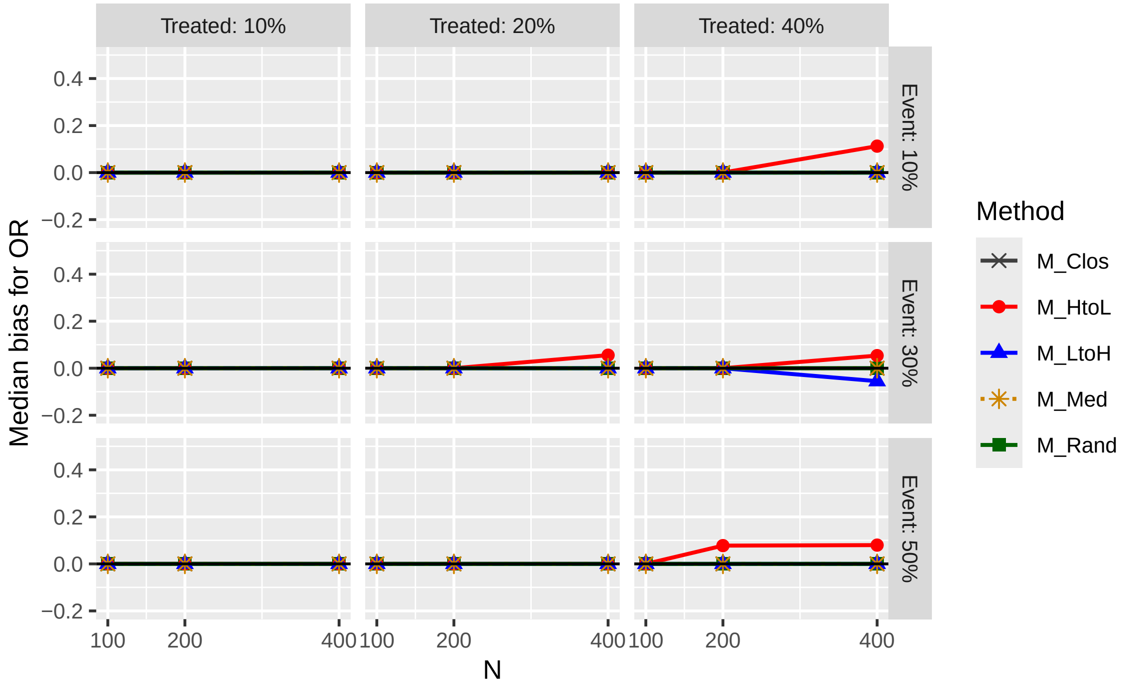

Figure S523. Median bias for OR (unimodal continuous covariate, matching ratio 1:1, true OR: 1, c statistic: 0.85).

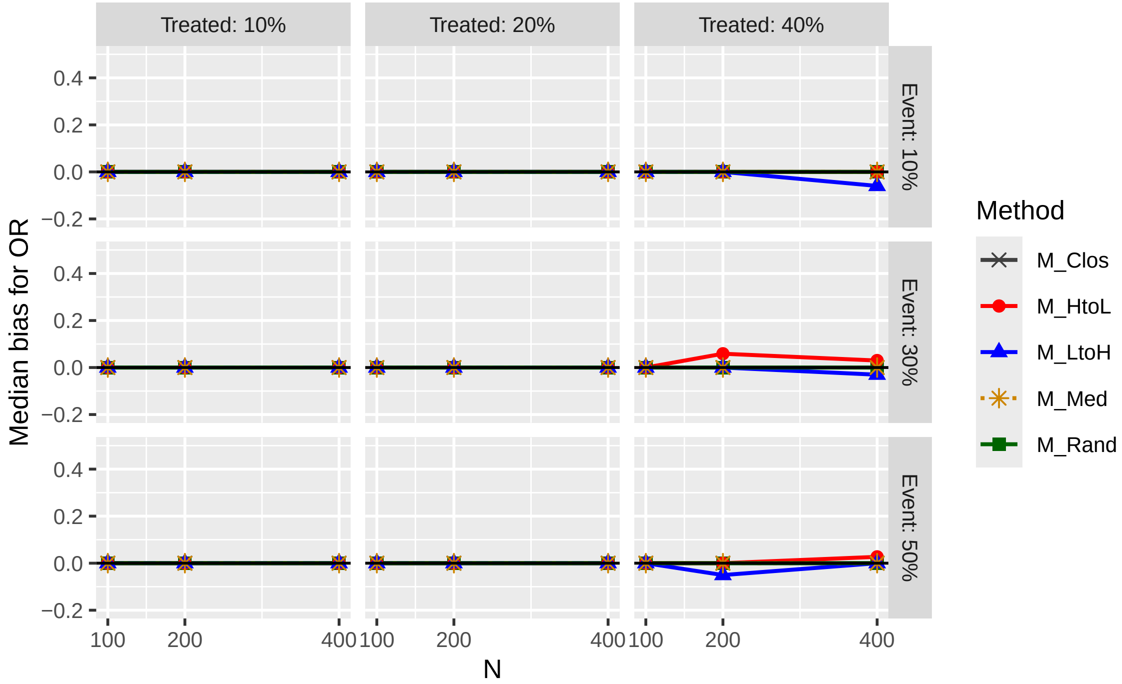

Figure S524. Median bias for OR (unimodal continuous covariate, matching ratio 1:1, true OR: 1, c statistic: 0.6).

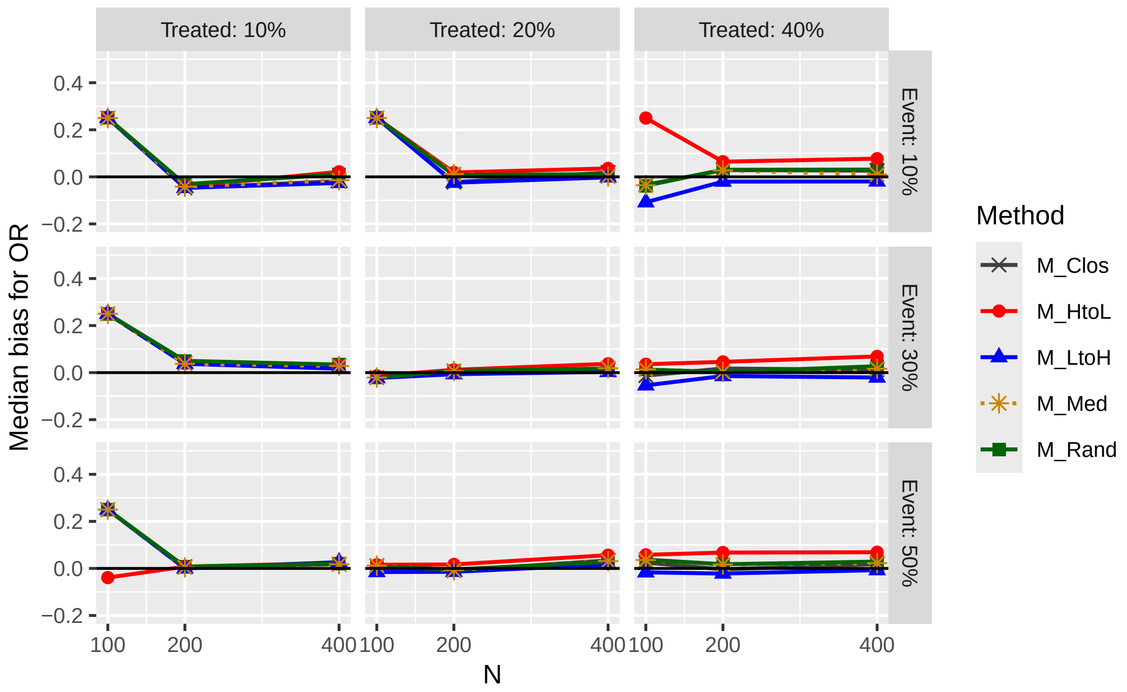

Figure S525. Median bias for OR (unimodal continuous covariate, matching ratio 1:1, true OR: 0.75, c statistic: 0.85).

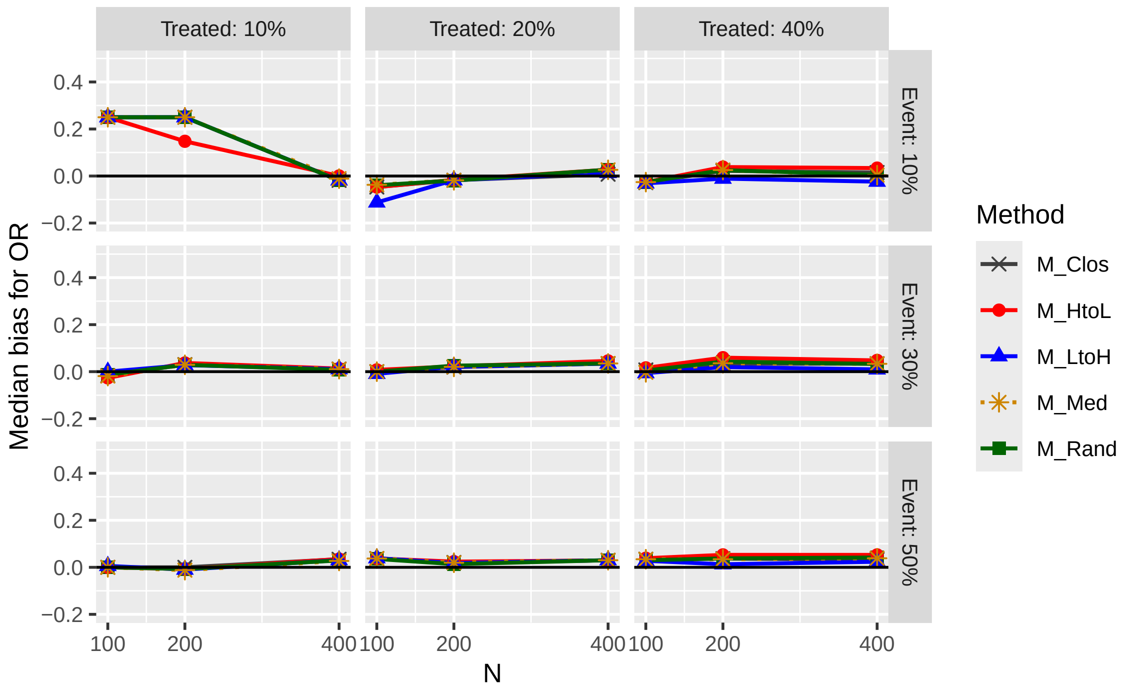

Figure S526. Median bias for OR (unimodal continuous covariate, matching ratio 1:1, true OR: 0.75, c statistic: 0.6).

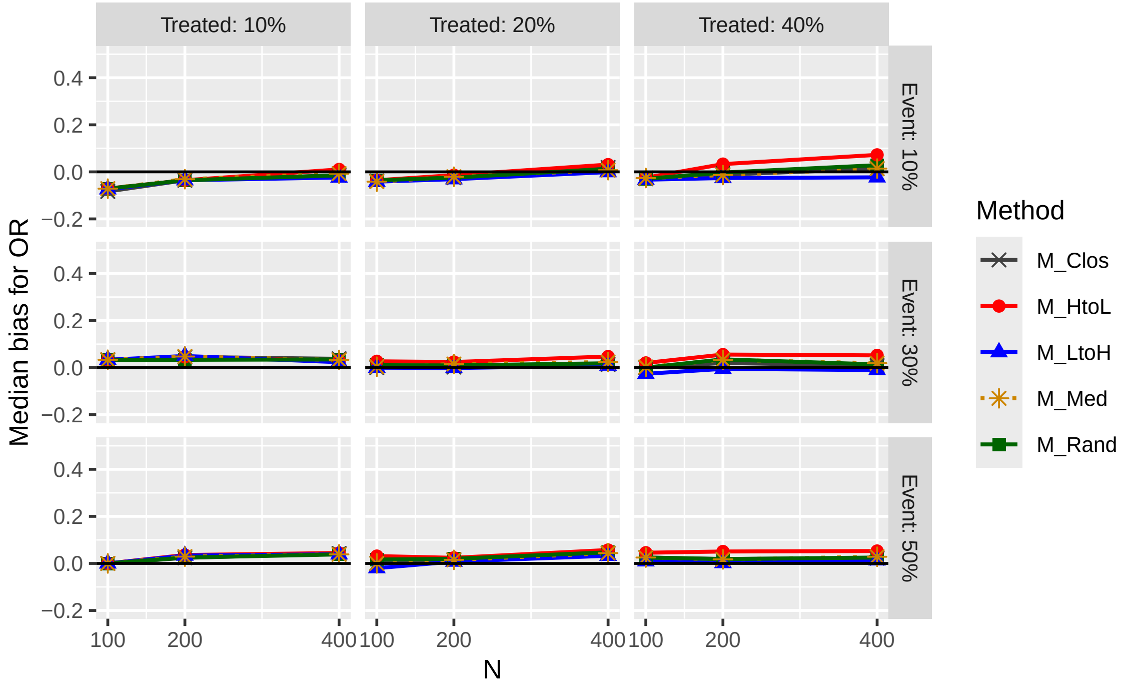

Figure S527. Median bias for OR (unimodal continuous covariate, matching ratio 1:1, true OR: 0.5, c statistic: 0.85).

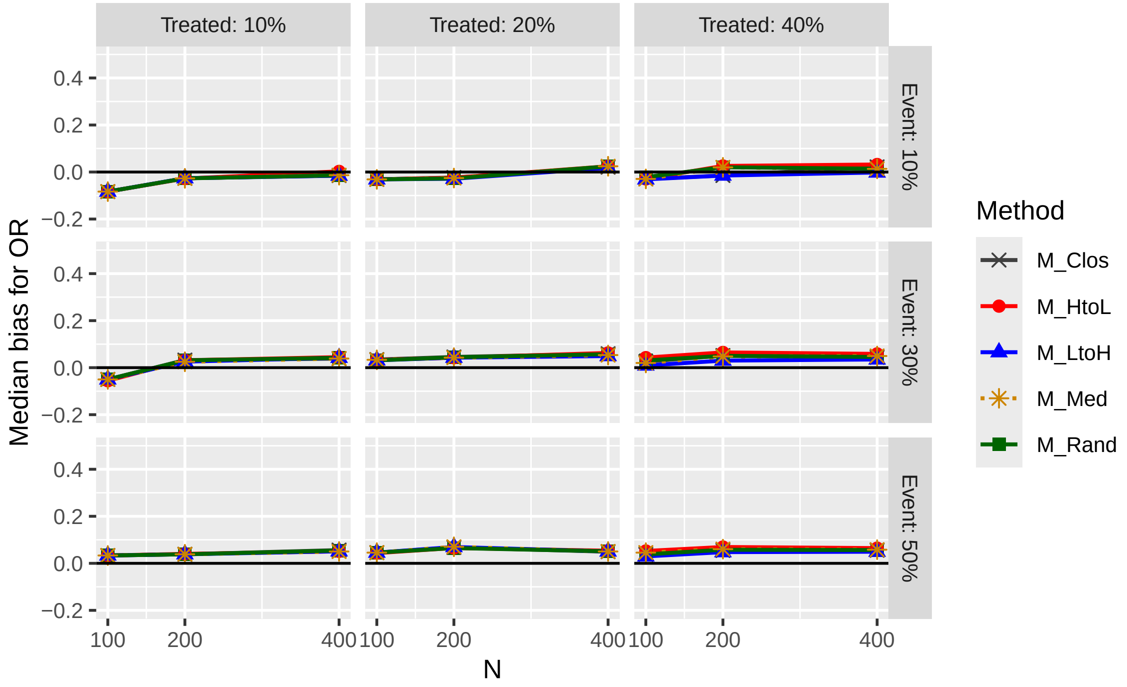

Figure S528. Median bias for OR (unimodal continuous covariate, matching ratio 1:1, true OR: 0.5, c statistic: 0.6).

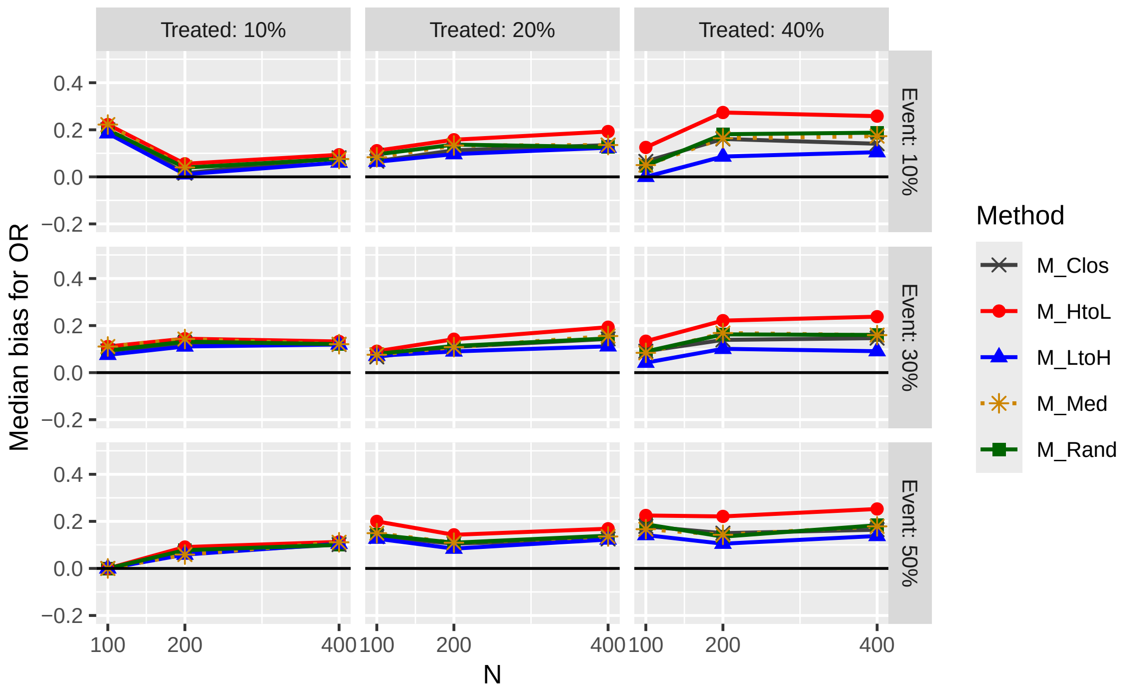

Figure S529. Median bias for OR (unimodal continuous covariate, matching ratio 1:2, true OR: 1, c statistic: 0.85).

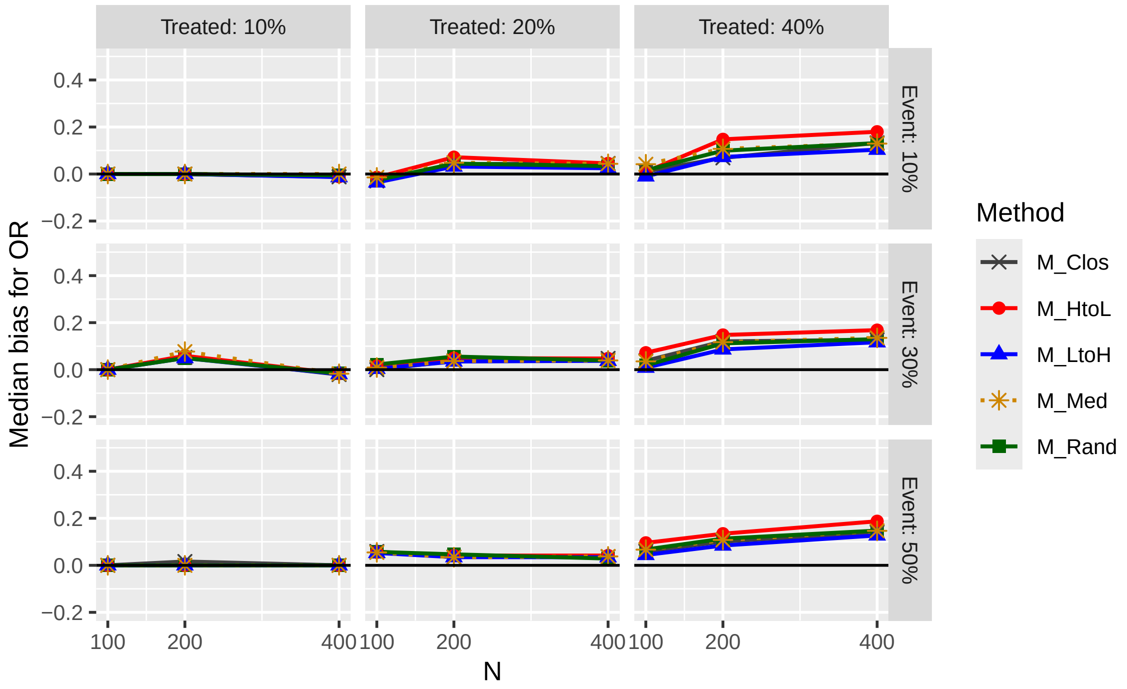

Figure S530. Median bias for OR (unimodal continuous covariate, matching ratio 1:2, true OR: 1, c statistic: 0.6).

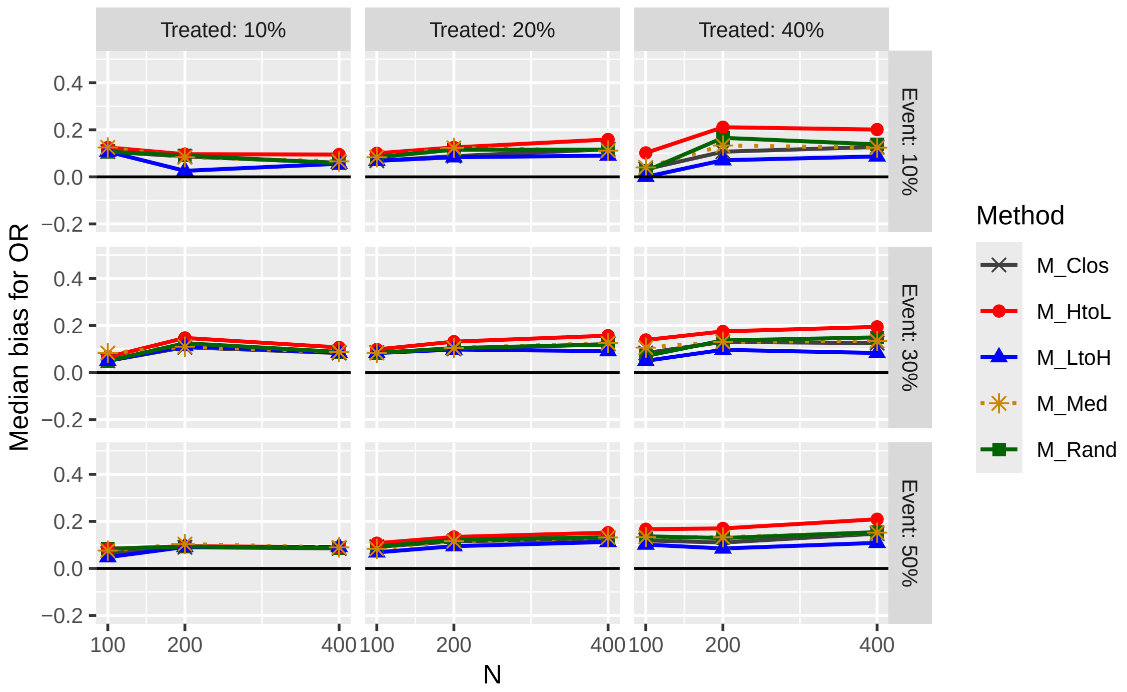

Figure S531. Median bias for OR (unimodal continuous covariate, matching ratio 1:2, true OR: 0.75, c statistic: 0.85).

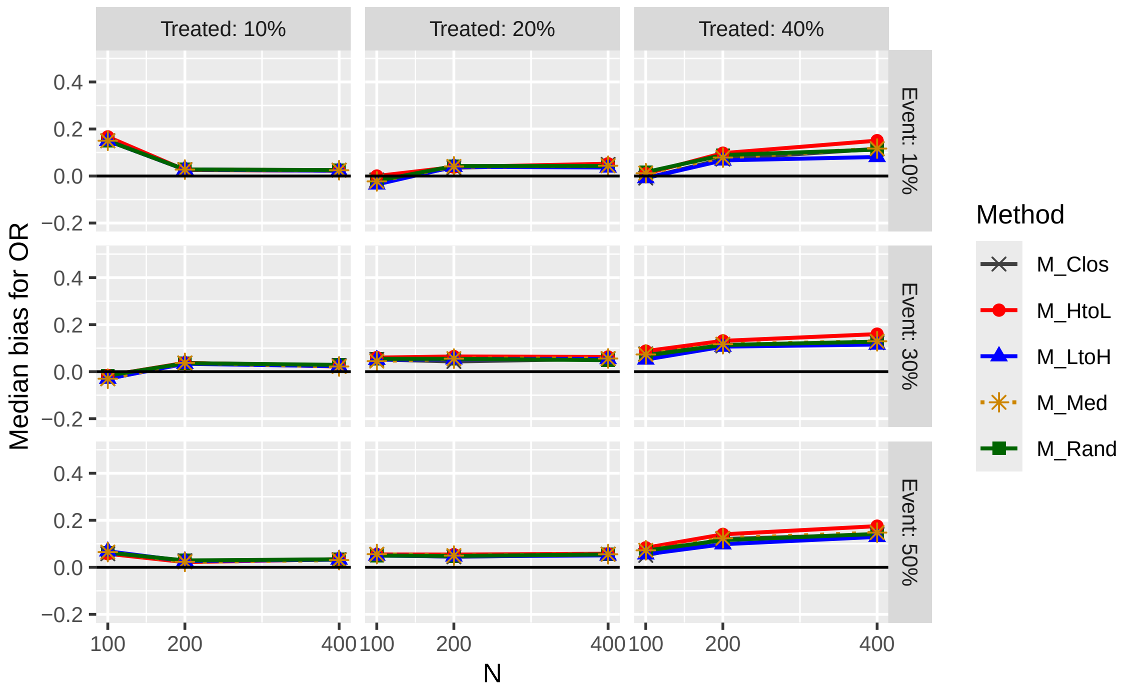

Figure S532. Median bias for OR (unimodal continuous covariate, matching ratio 1:2, true OR: 0.75, c statistic: 0.6).

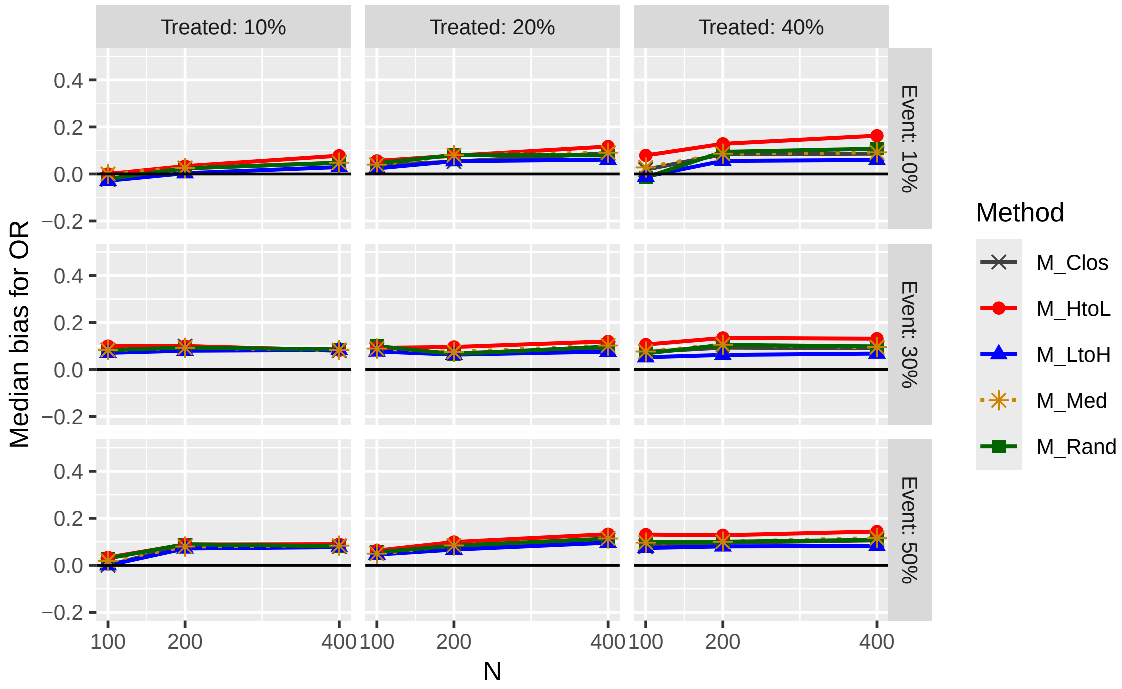

Figure S533. Median bias for OR (unimodal continuous covariate, matching ratio 1:2, true OR: 0.5, c statistic: 0.85).

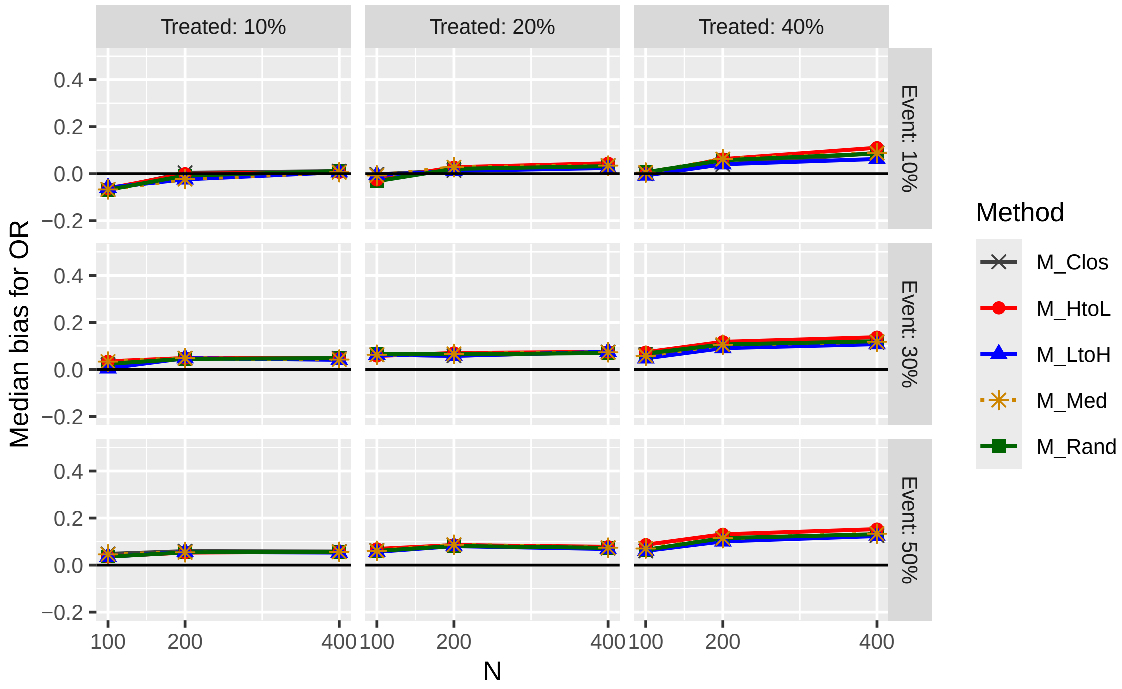

Figure S534. Median bias for OR (unimodal continuous covariate, matching ratio 1:2, true OR: 0.5, c statistic: 0.6).

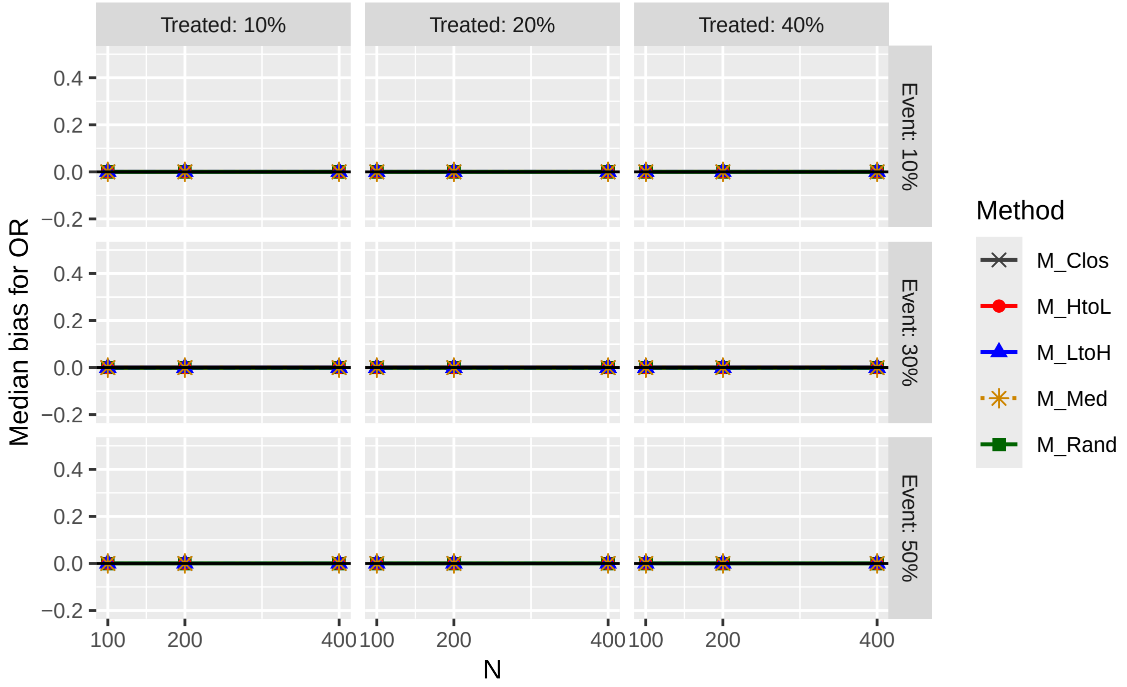

Figure S535. Median bias for OR (categorical covariate, matching ratio 1:1, true OR: 1, c statistic: 0.85).

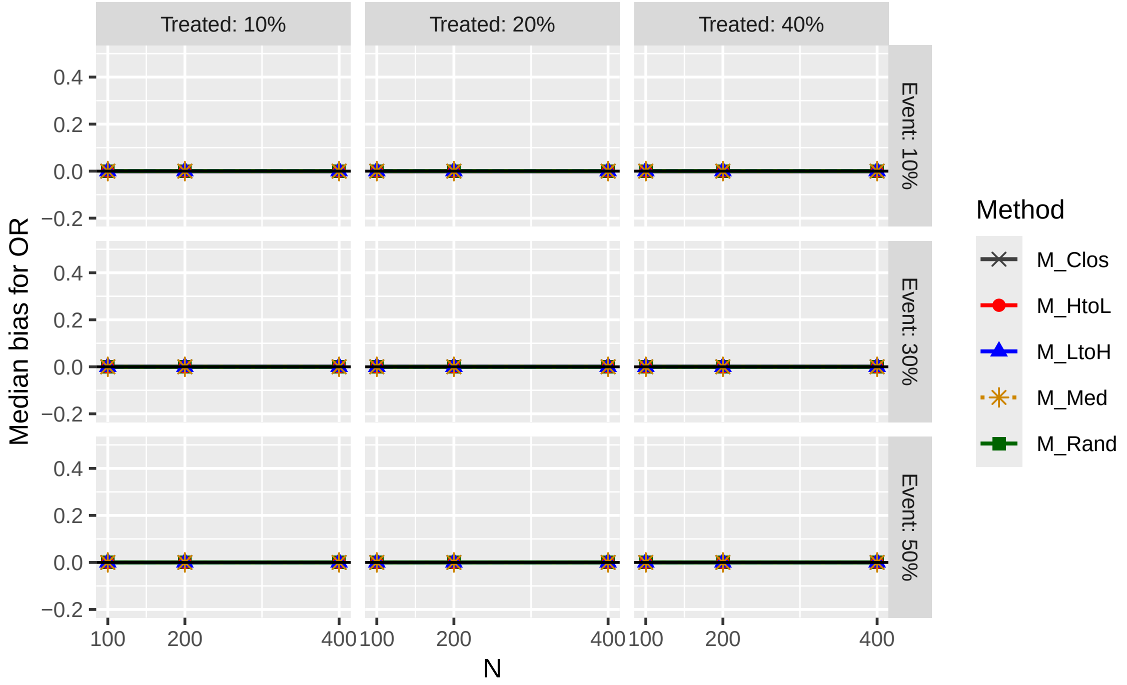

Figure S536. Median bias for OR (categorical covariate, matching ratio 1:1, true OR: 1, c statistic: 0.6).

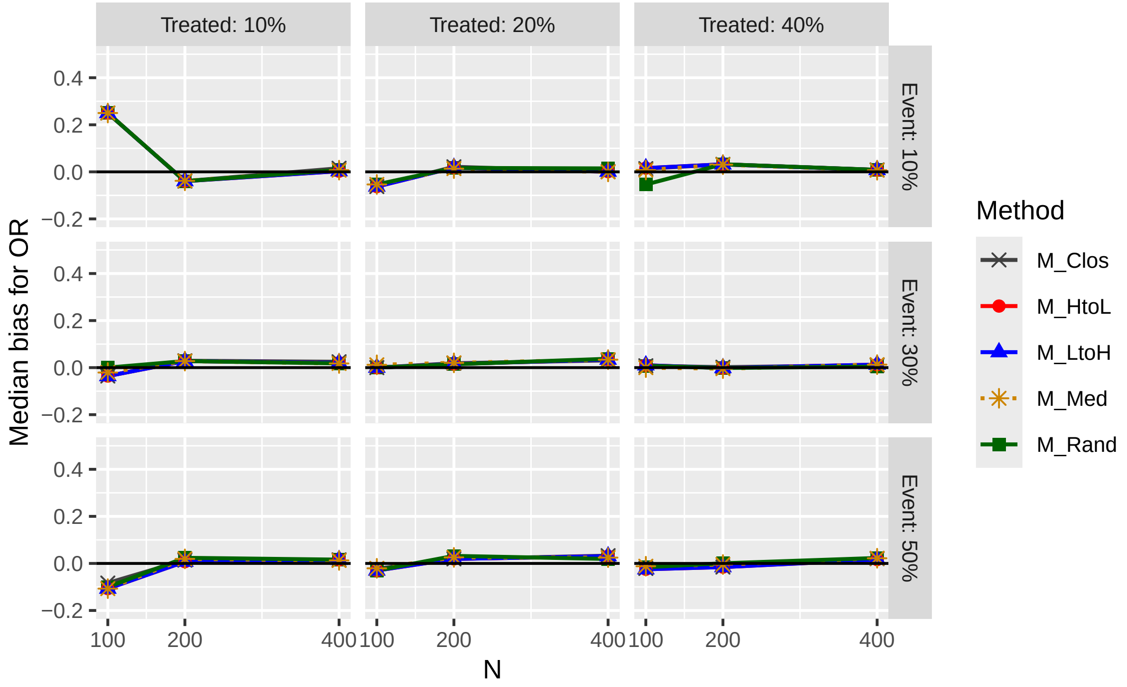

Figure S537. Median bias for OR (categorical covariate, matching ratio 1:1, true OR: 0.75, c statistic: 0.85).

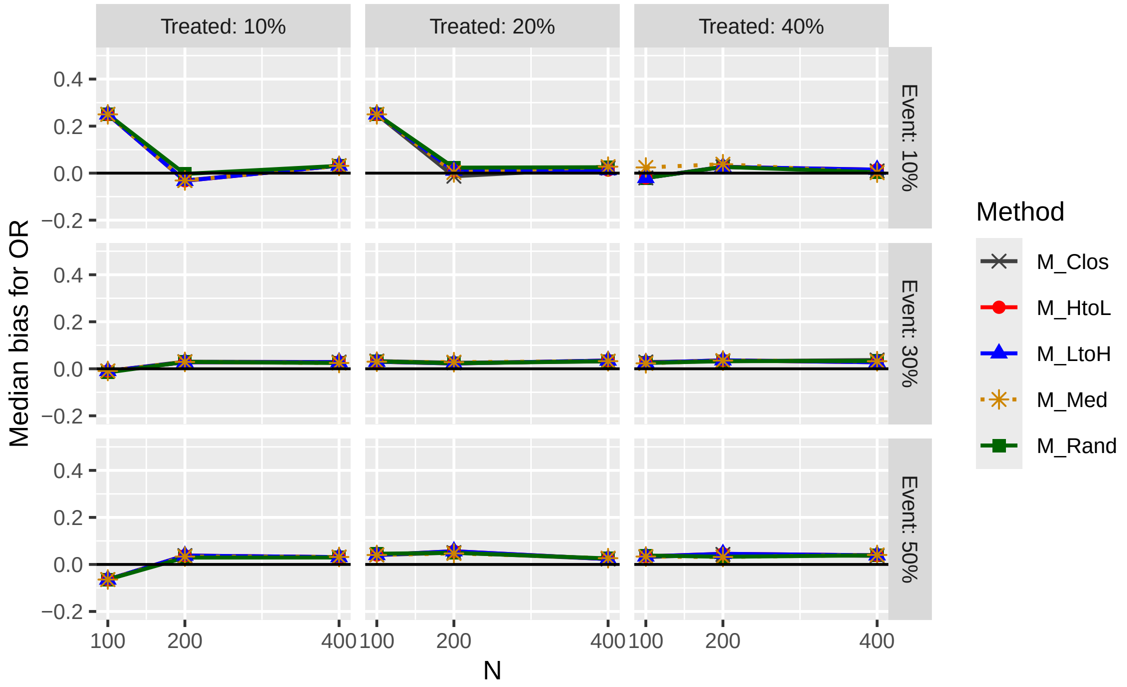

Figure S538. Median bias for OR (categorical covariate, matching ratio 1:1, true OR: 0.75, c statistic: 0.6).

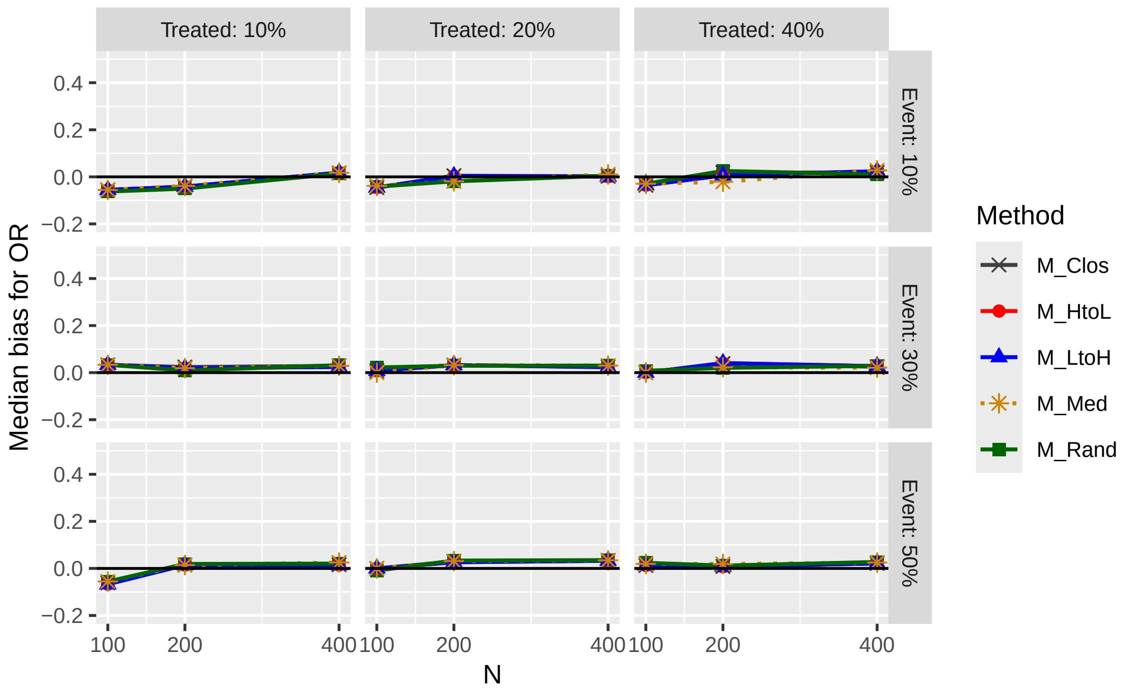

Figure S539. Median bias for OR (categorical covariate, matching ratio 1:1, true OR: 0.5, c statistic: 0.85).

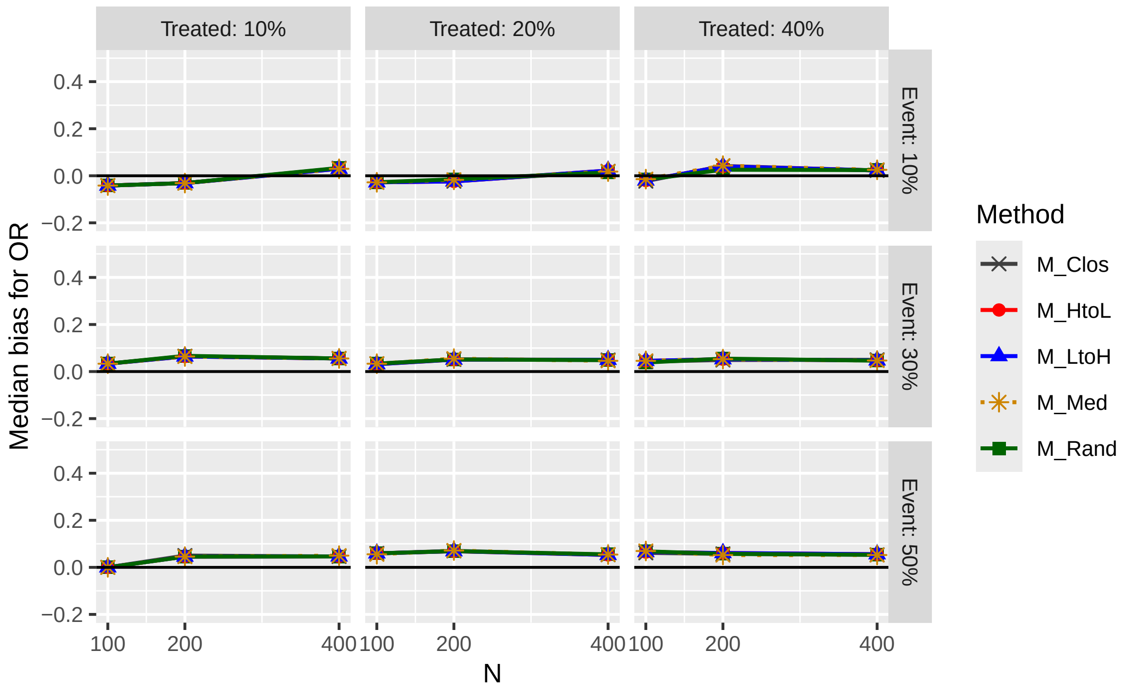

Figure S540. Median bias for OR (categorical covariate, matching ratio 1:1, true OR: 0.5, c statistic: 0.6).

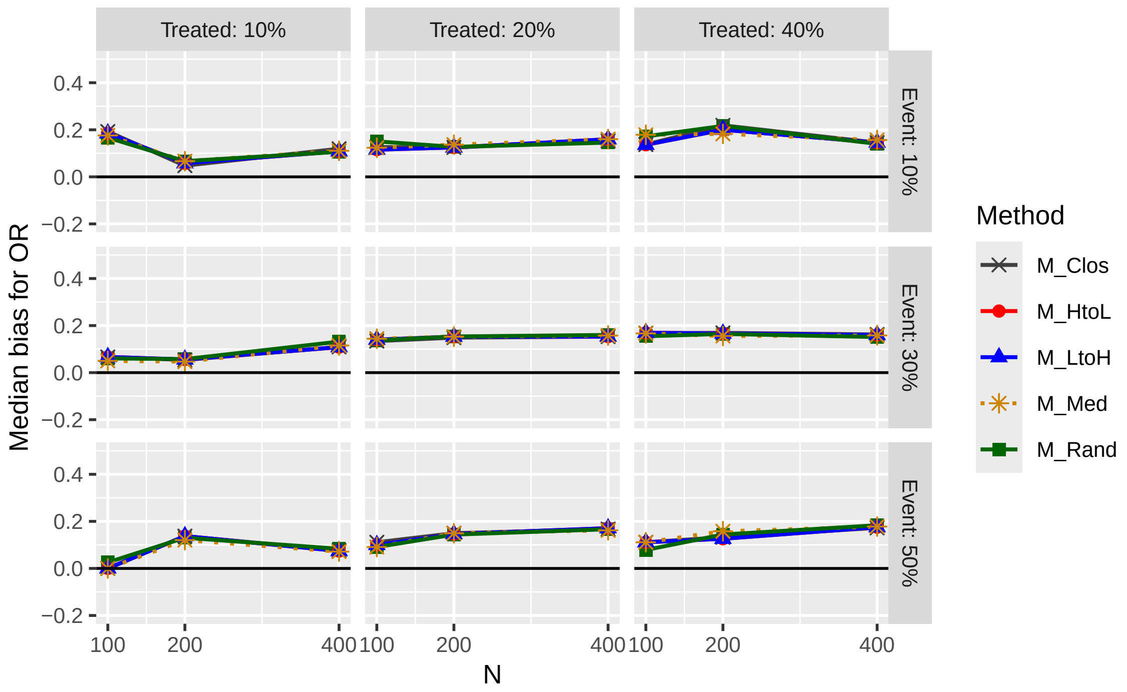

Figure S541. Median bias for OR (categorical covariate, matching ratio 1:2, true OR: 1, c statistic: 0.85).

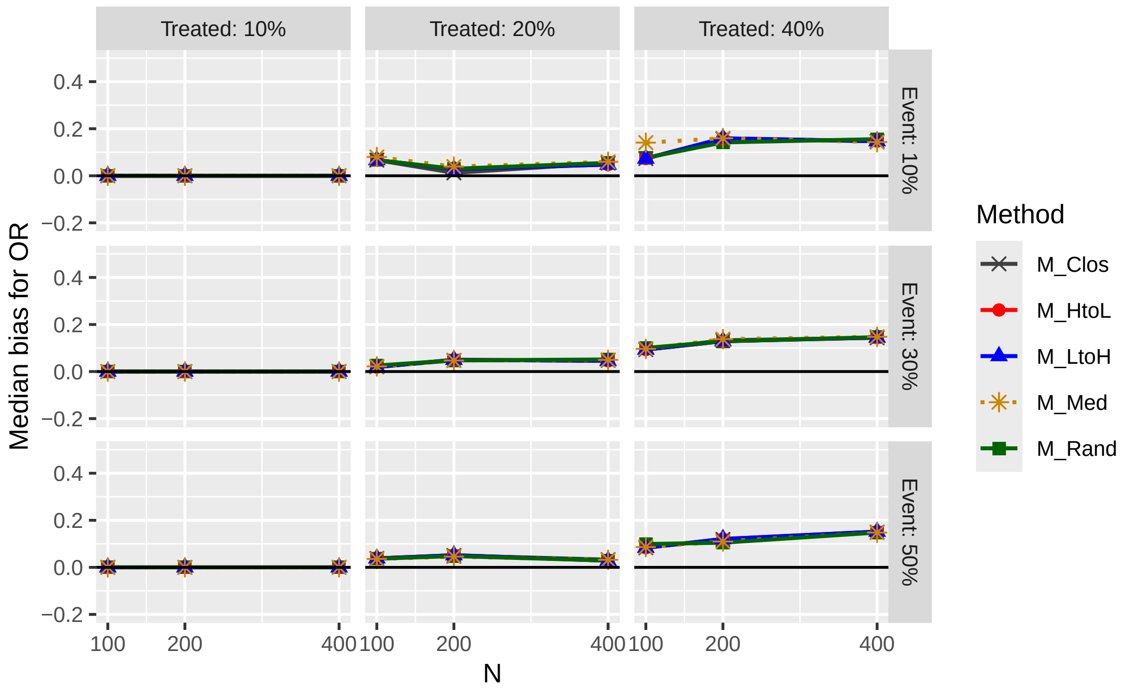

Figure S542. Median bias for OR (categorical covariate, matching ratio 1:2, true OR: 1, c statistic: 0.6).

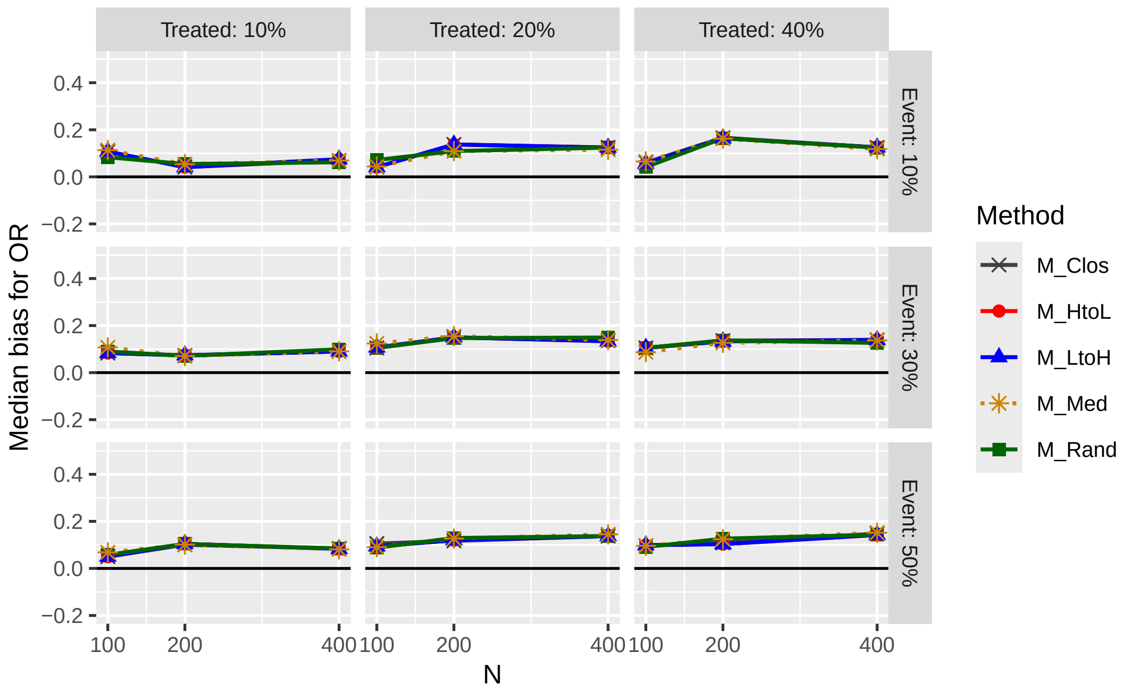

Figure S543. Median bias for OR (categorical covariate, matching ratio 1:2, true OR: 0.75, c statistic: 0.85).

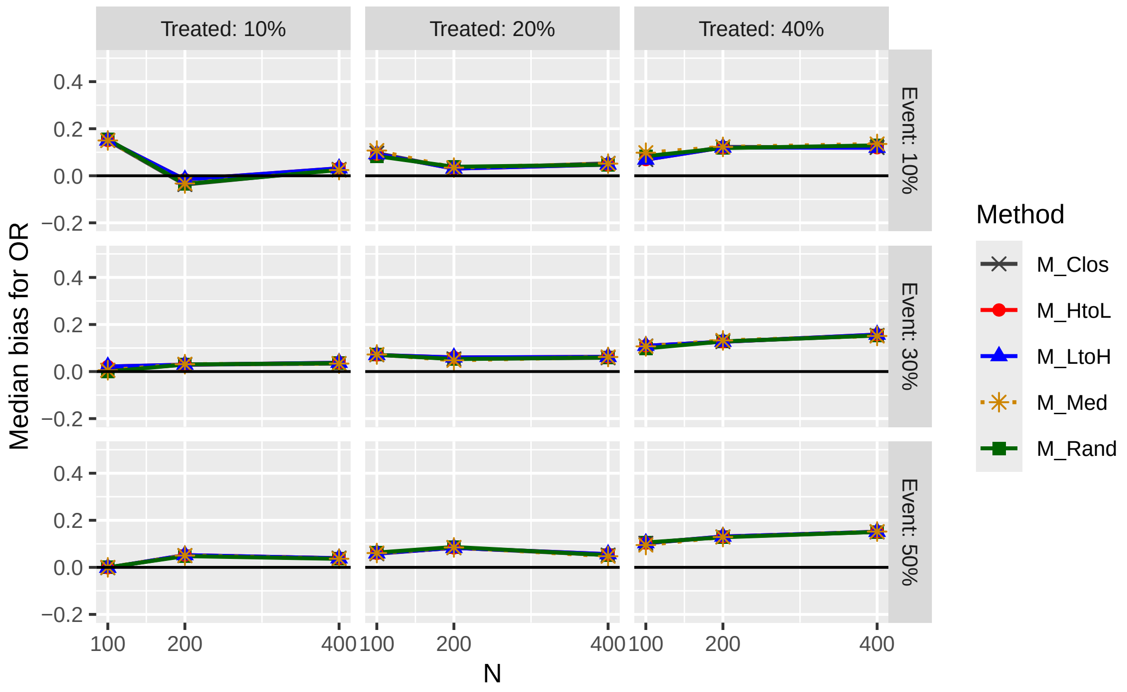

Figure S544. Median bias for OR (categorical covariate, matching ratio 1:2, true OR: 0.75, c statistic: 0.6).

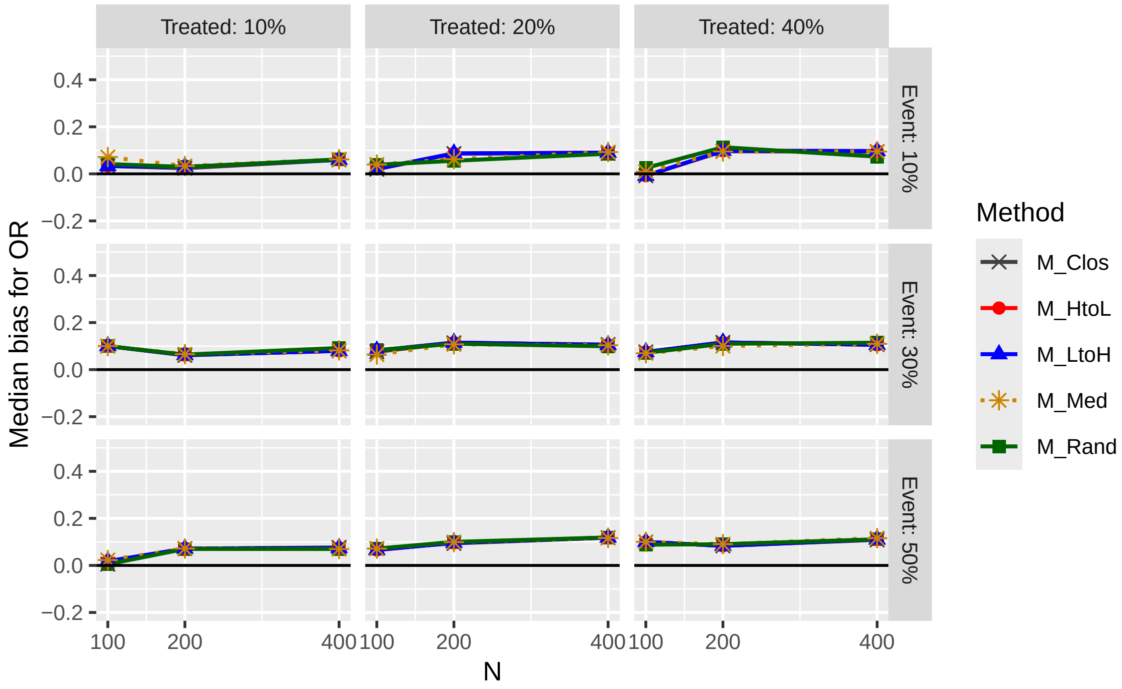

Figure S545. Median bias for OR (categorical covariate, matching ratio 1:2, true OR: 0.5, c statistic: 0.85).

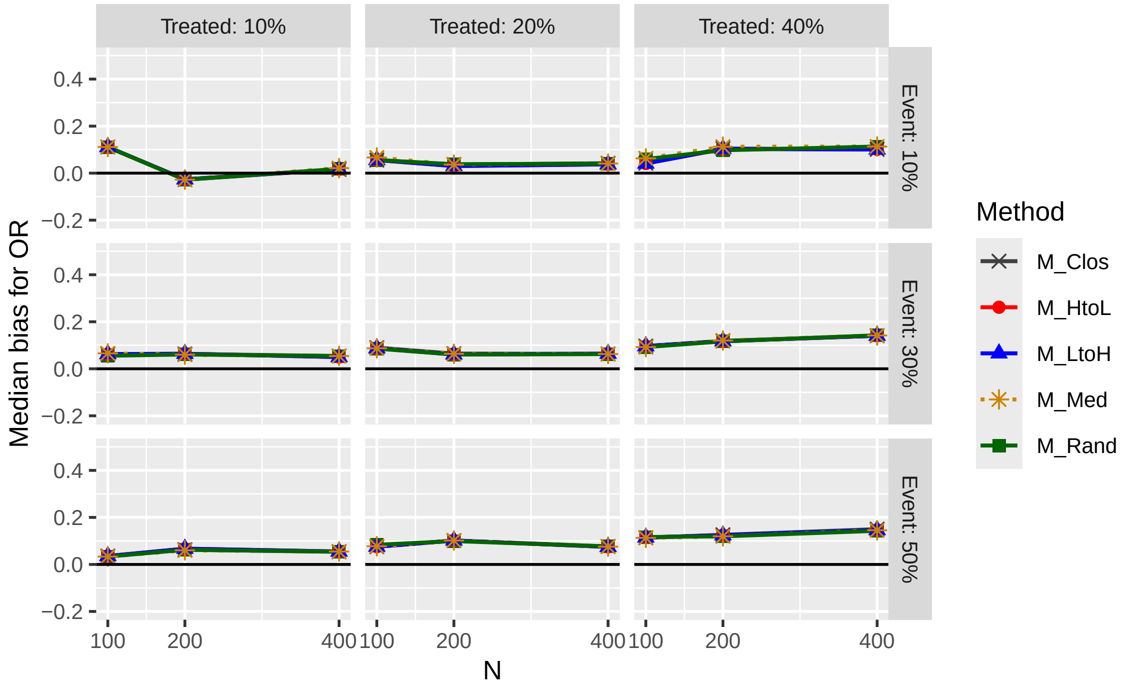

Figure S546. Median bias for OR (categorical covariate, matching ratio 1:2, true OR: 0.5, c statistic: 0.6).

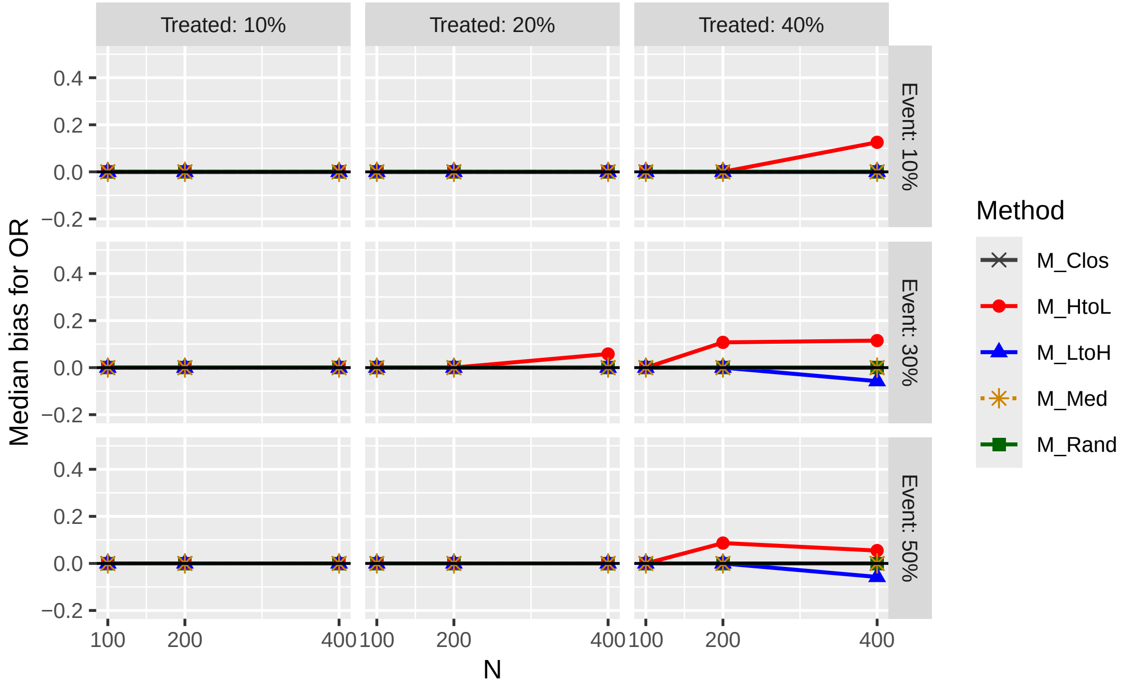

Figure S547. Median bias for OR (multimodal continuous covariate, matching ratio 1:1, true OR: 1, c statistic: 0.85).

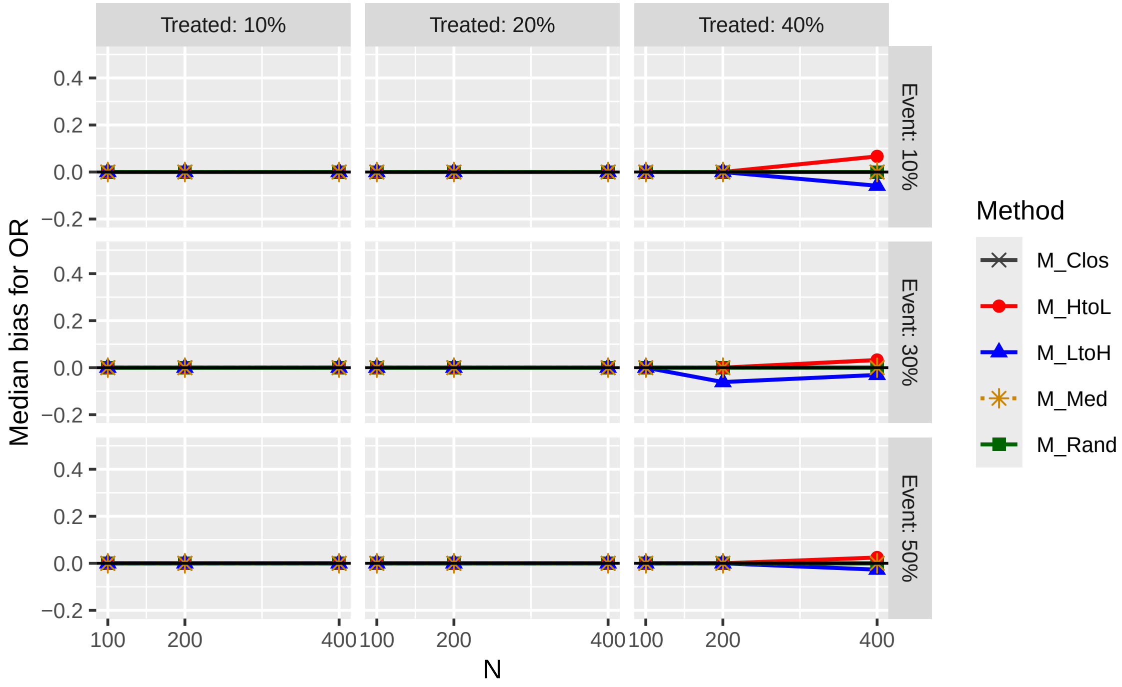

Figure S548. Median bias for OR (multimodal continuous covariate, matching ratio 1:1, true OR: 1, c statistic: 0.6).

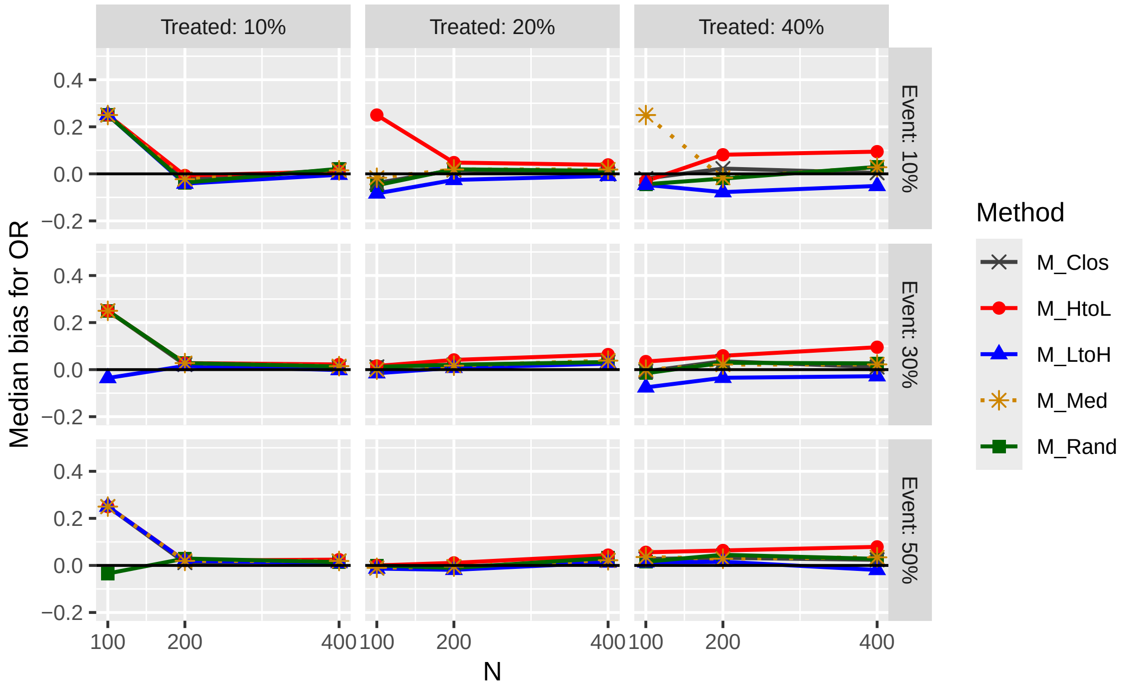

Figure S549. Median bias for OR (multimodal continuous covariate, matching ratio 1:1, true OR: 0.75, c statistic: 0.85).

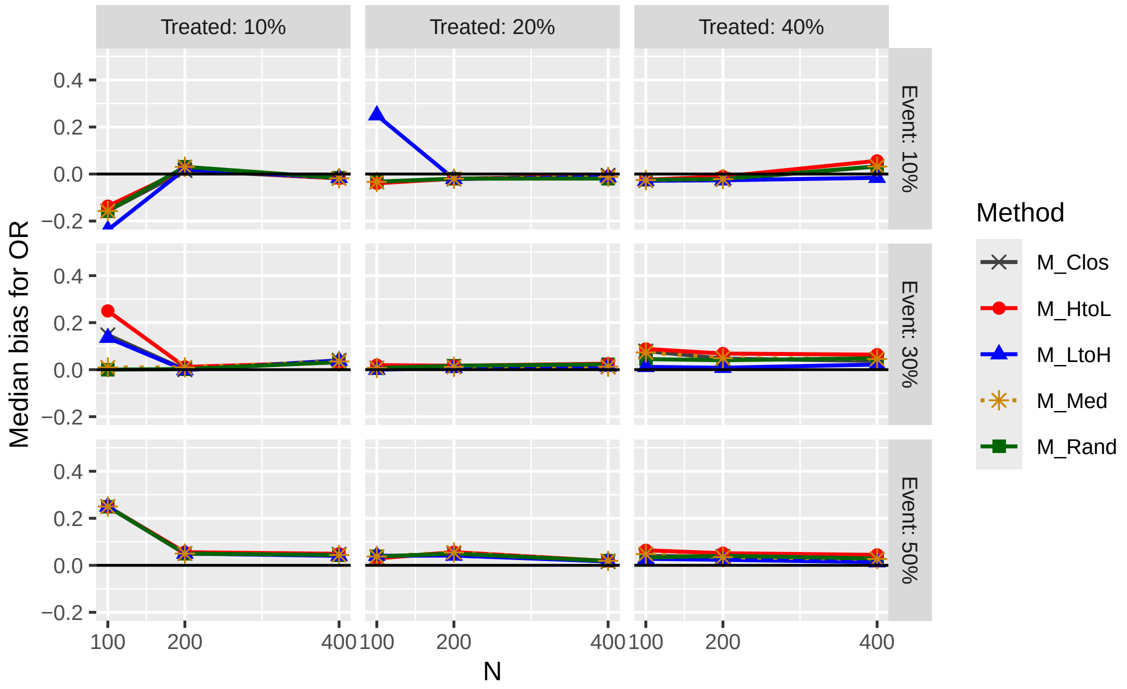

Figure S550. Median bias for OR (multimodal continuous covariate, matching ratio 1:1, true OR: 0.75, c statistic: 0.6).

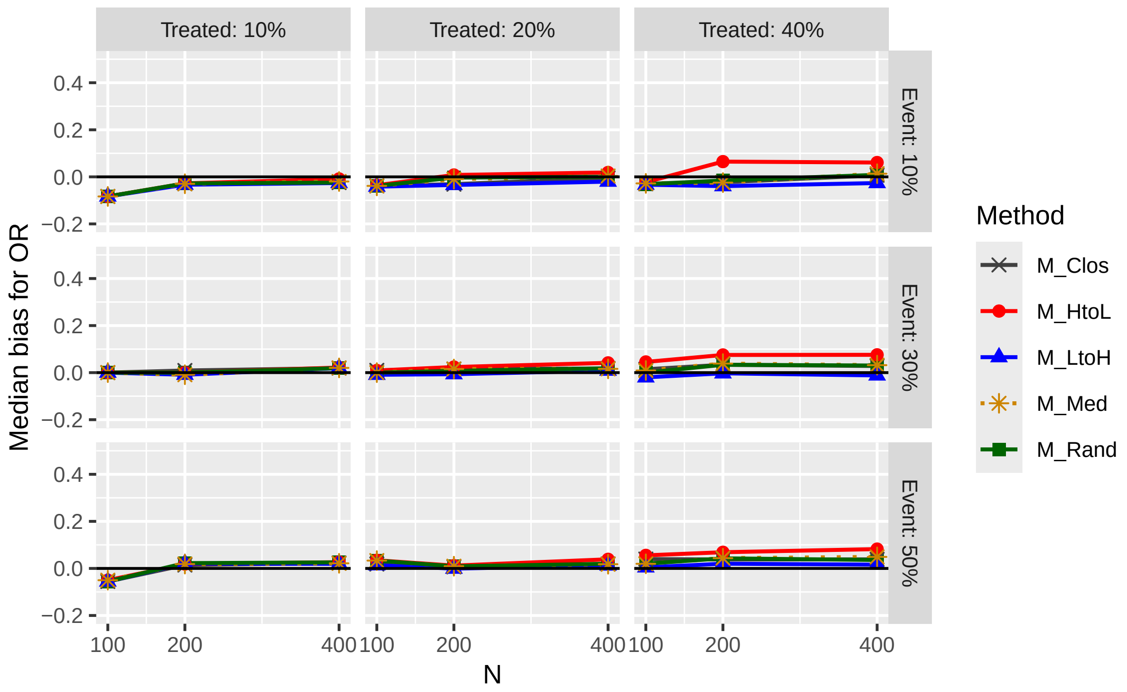

Figure S551. Median bias for OR (multimodal continuous covariate, matching ratio 1:1, true OR: 0.5, c statistic: 0.85).

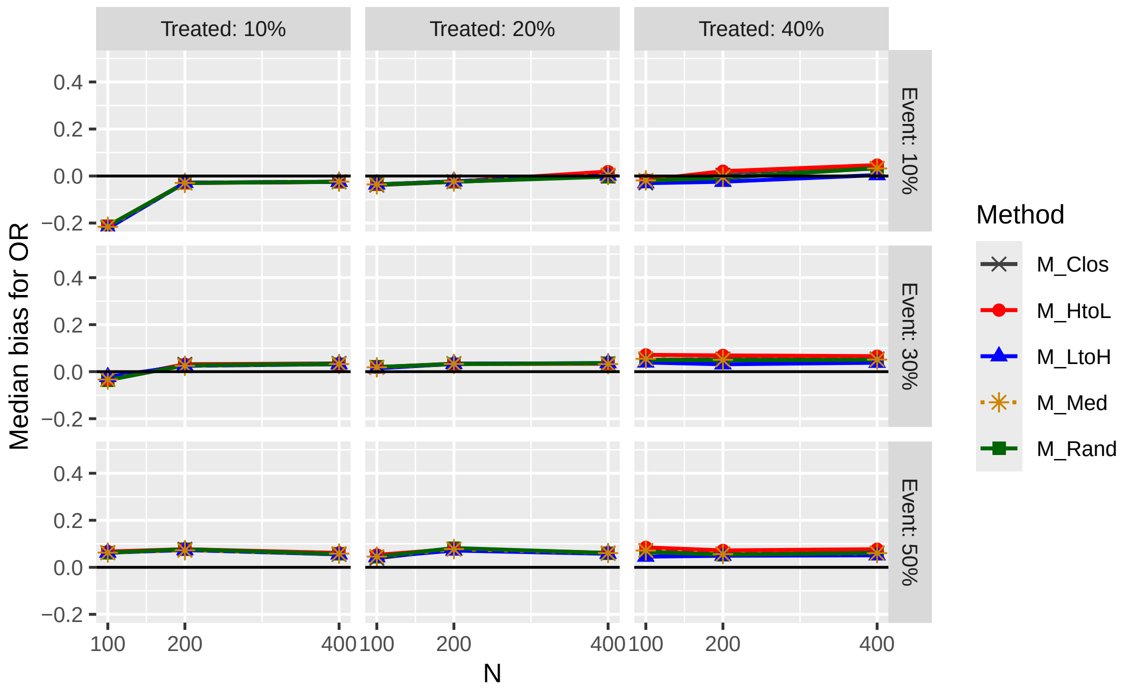

Figure S552. Median bias for OR (multimodal continuous covariate, matching ratio 1:1, true OR: 0.5, c statistic: 0.6).

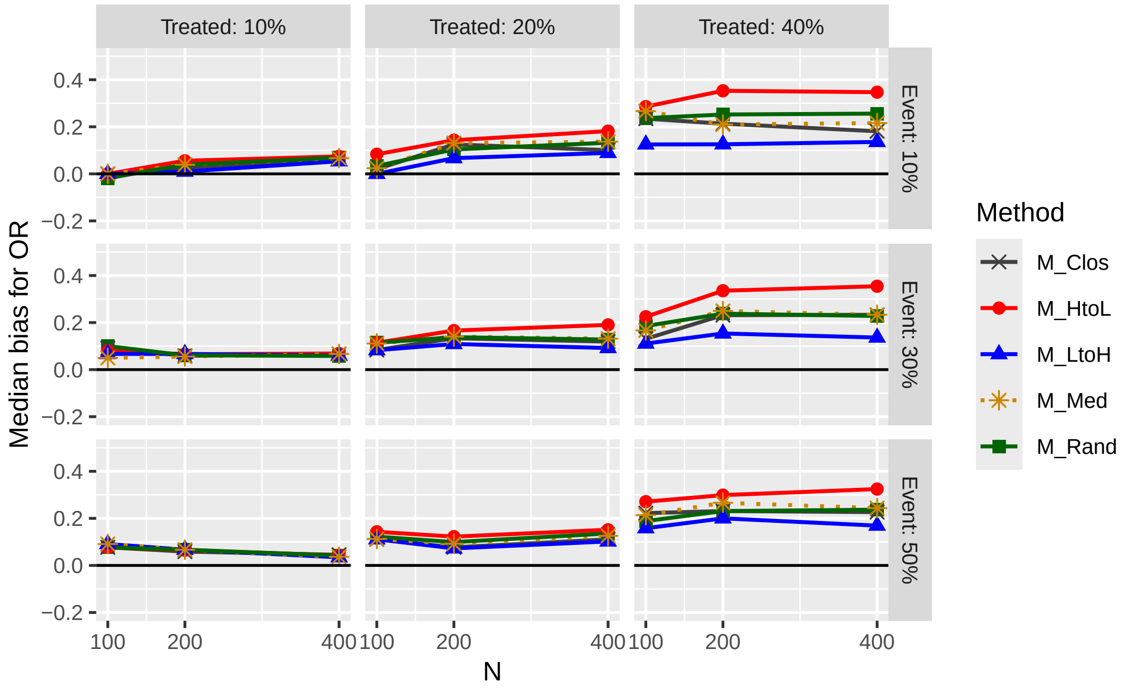

Figure S553. Median bias for OR (multimodal continuous covariate, matching ratio 1:2, true OR: 1, c statistic: 0.85).

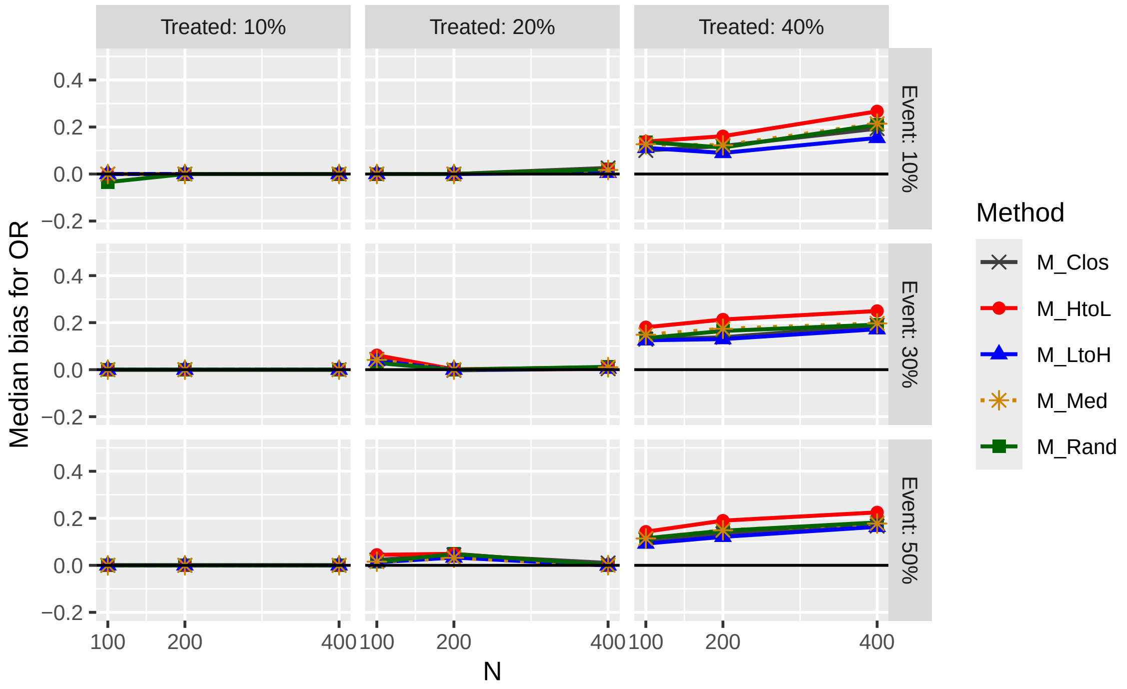

Figure S554. Median bias for OR (multimodal continuous covariate, matching ratio 1:2, true OR: 1, c statistic: 0.6).

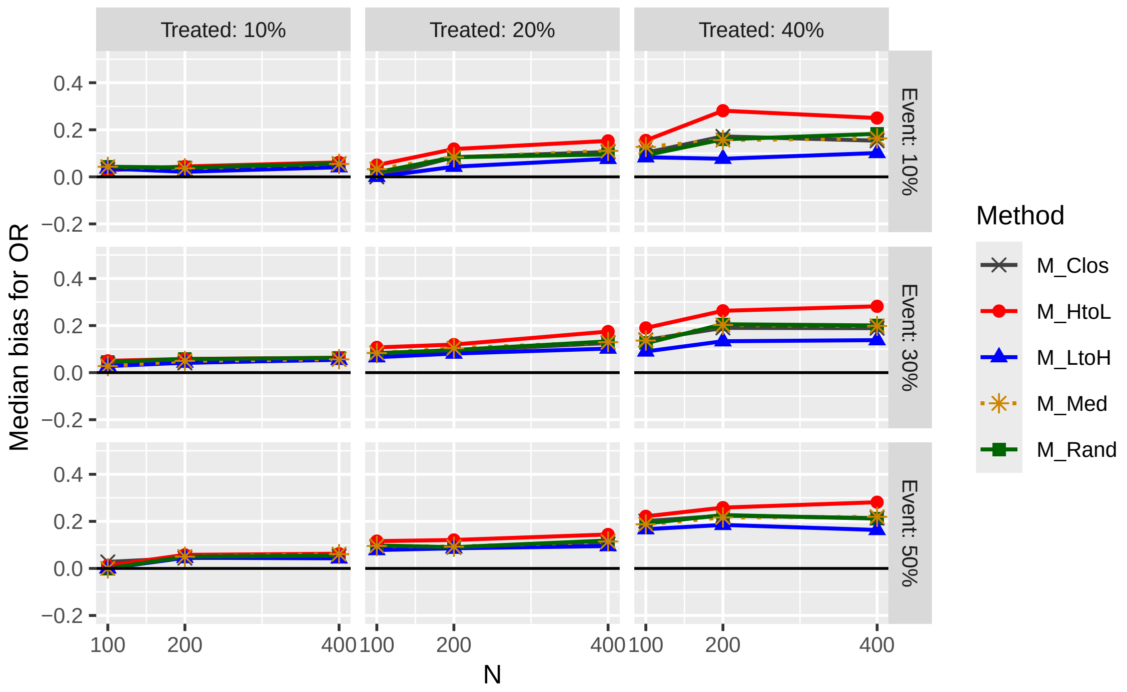

Figure S555. Median bias for OR (multimodal continuous covariate, matching ratio 1:2, true OR: 0.75, c statistic: 0.85).

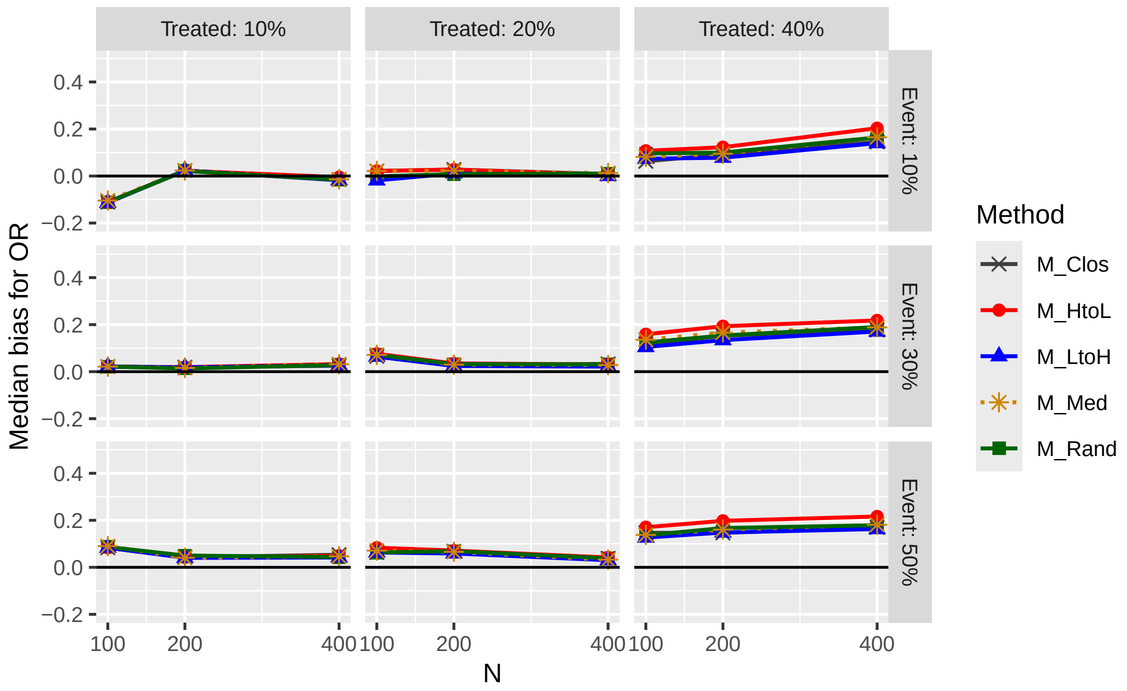

Figure S556. Median bias for OR (multimodal continuous covariate, matching ratio 1:2, true OR: 0.75, c statistic: 0.6).

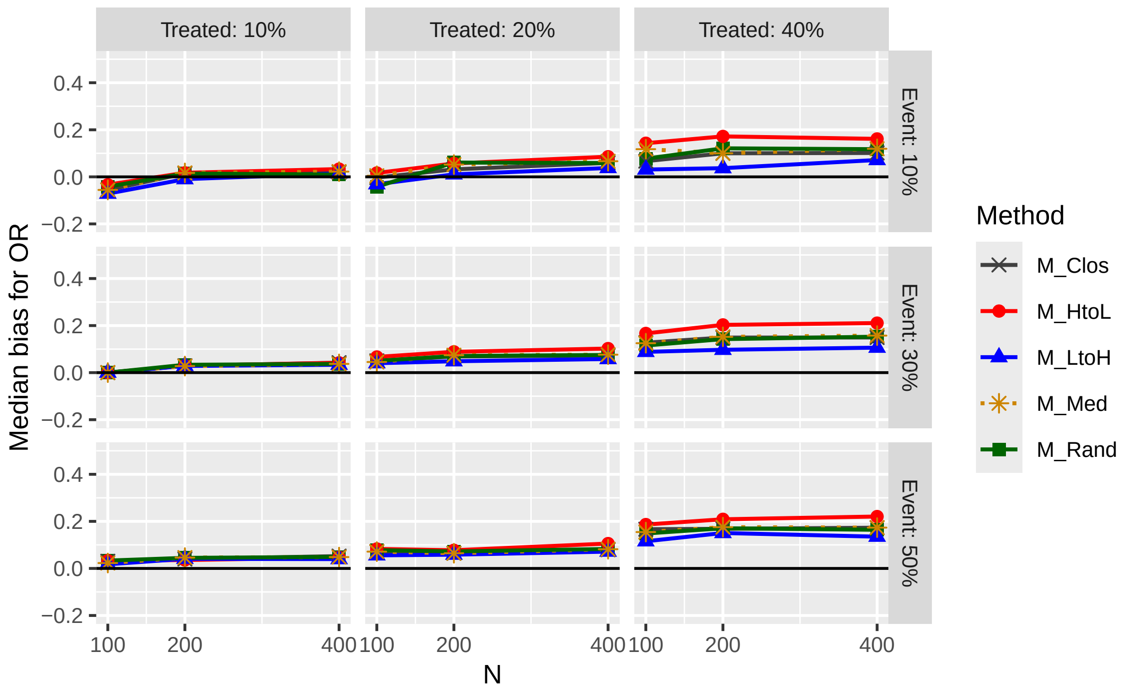

Figure S557. Median bias for OR (multimodal continuous covariate, matching ratio 1:2, true OR: 0.5, c statistic: 0.85).

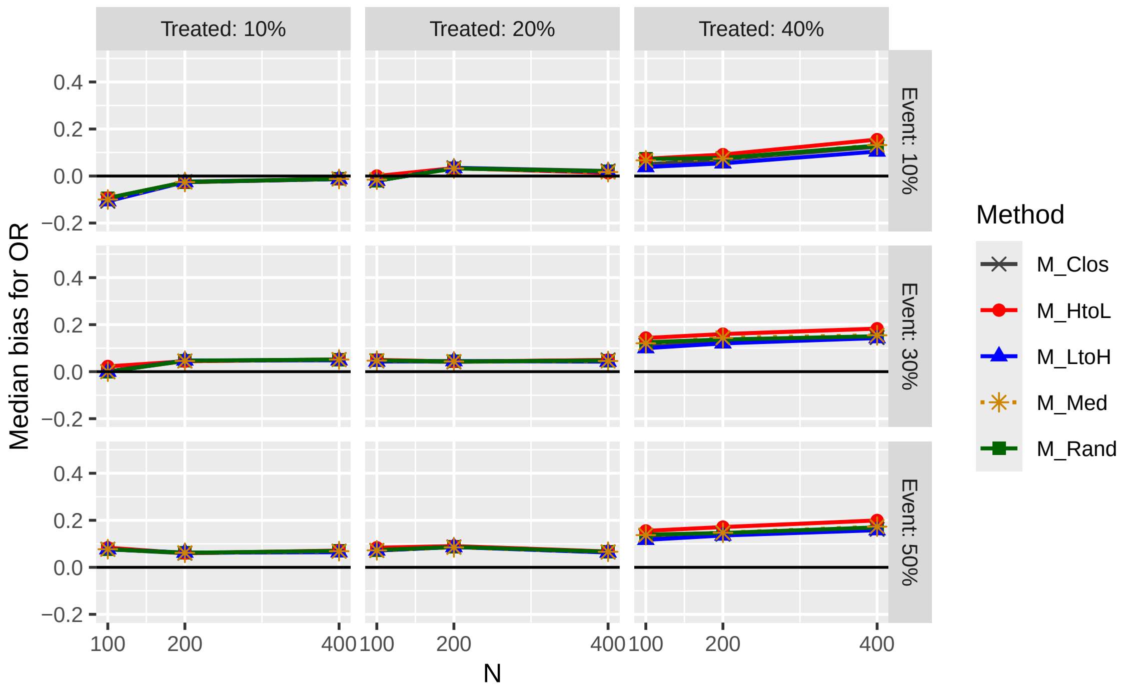

Figure S558. Median bias for OR (multimodal continuous covariate, matching ratio 1:2, true OR: 0.5, c statistic: 0.6).

S9. IQR for OR (precision, caliper: 15%))

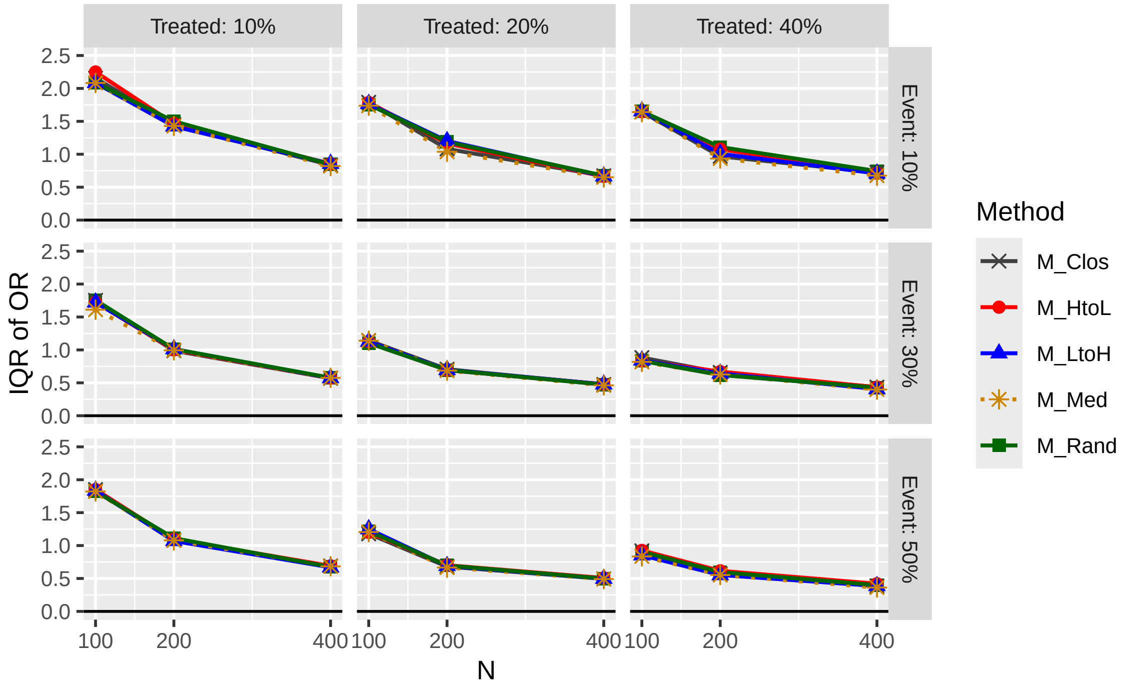

Figure S559. IQR for OR (unimodal continuous covariate, matching ratio 1:1, true OR: 1, c statistic: 0.85).

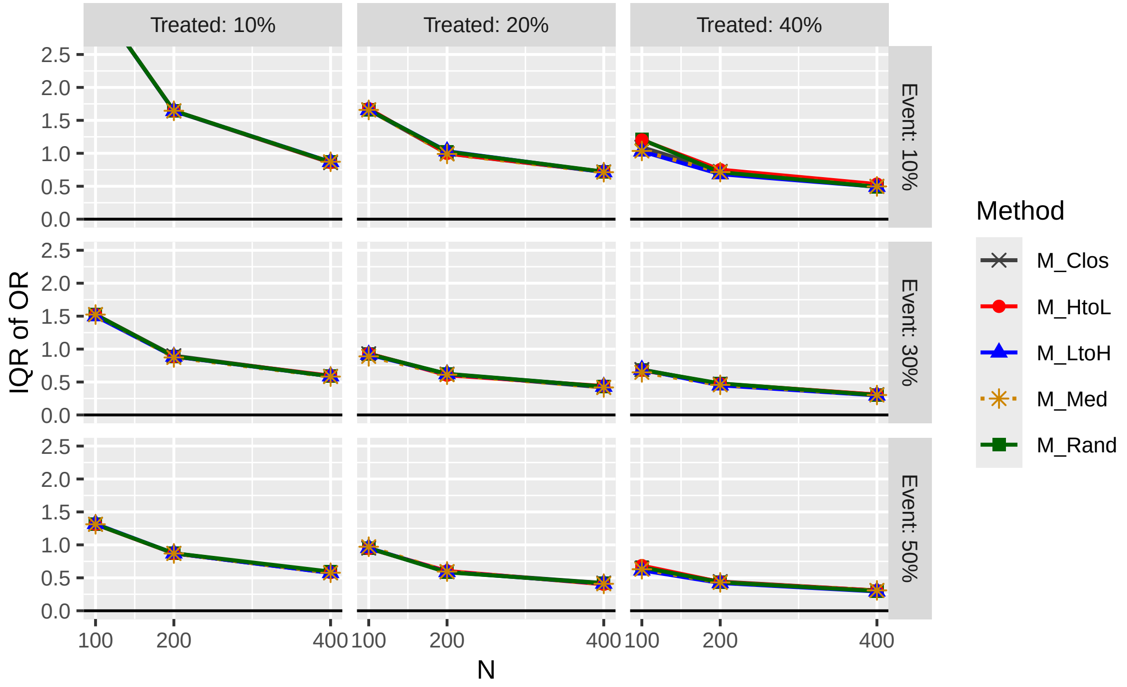

Figure S560. IQR for OR (unimodal continuous covariate, matching ratio 1:1, true OR: 1, c statistic: 0.6).

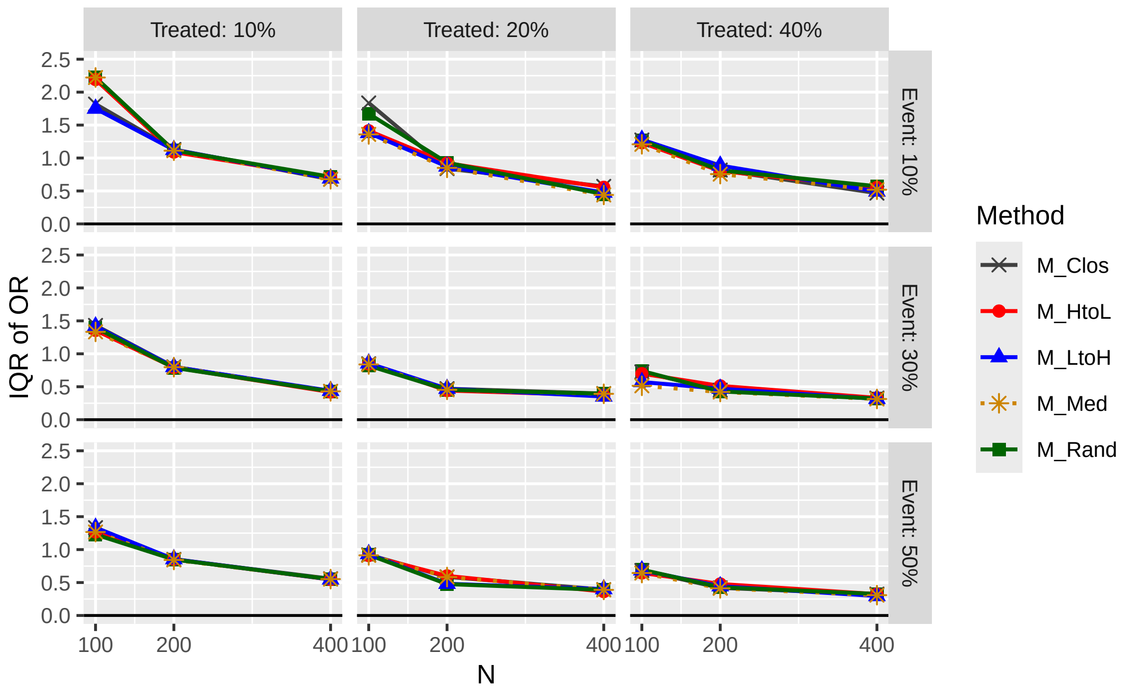

Figure S561. IQR for OR (unimodal continuous covariate, matching ratio 1:1, true OR: 0.75, c statistic: 0.85).

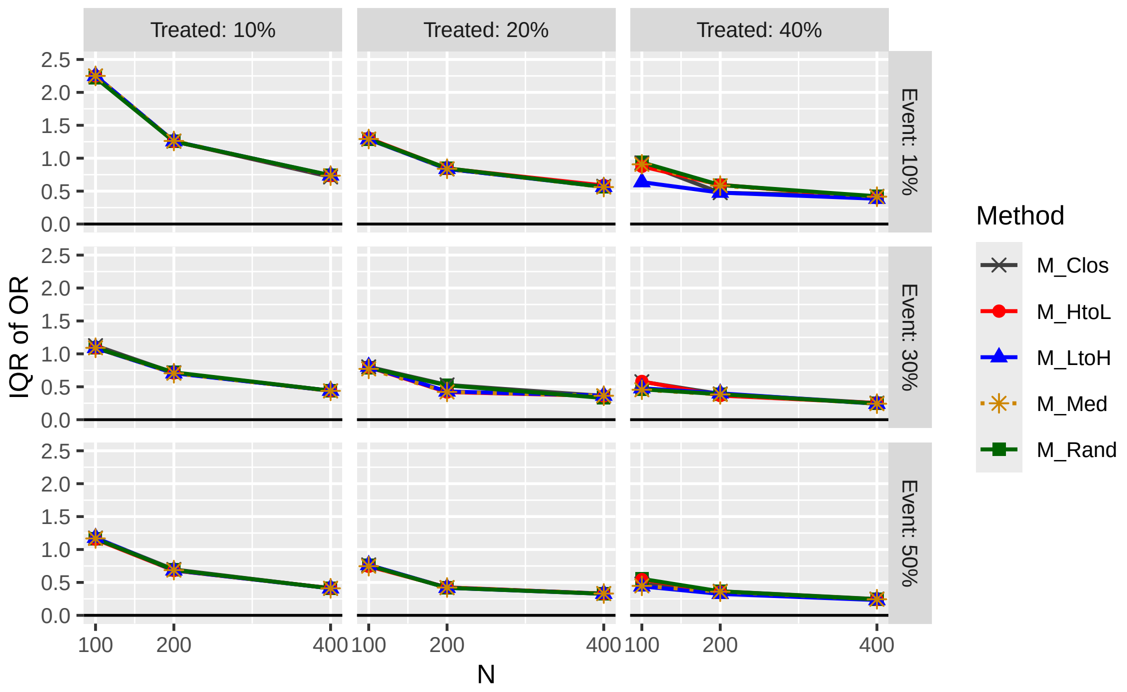

Figure S562. IQR for OR (unimodal continuous covariate, matching ratio 1:1, true OR: 0.75, c statistic: 0.6).

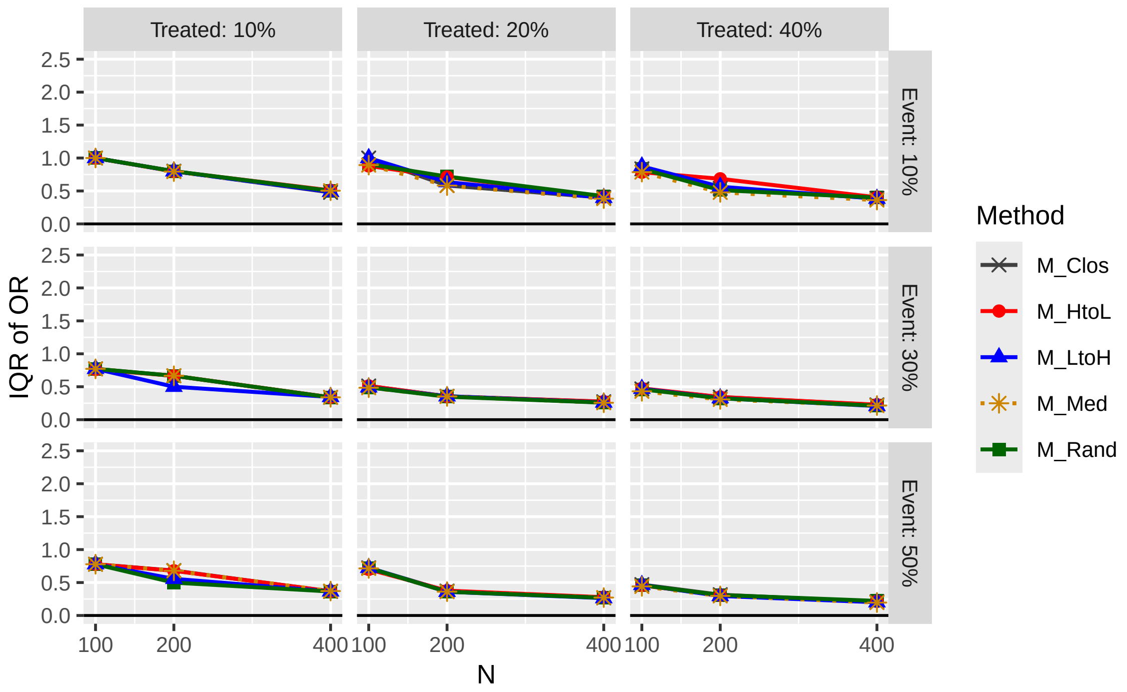

Figure S563. IQR for OR (unimodal continuous covariate, matching ratio 1:1, true OR: 0.5, c statistic: 0.85).

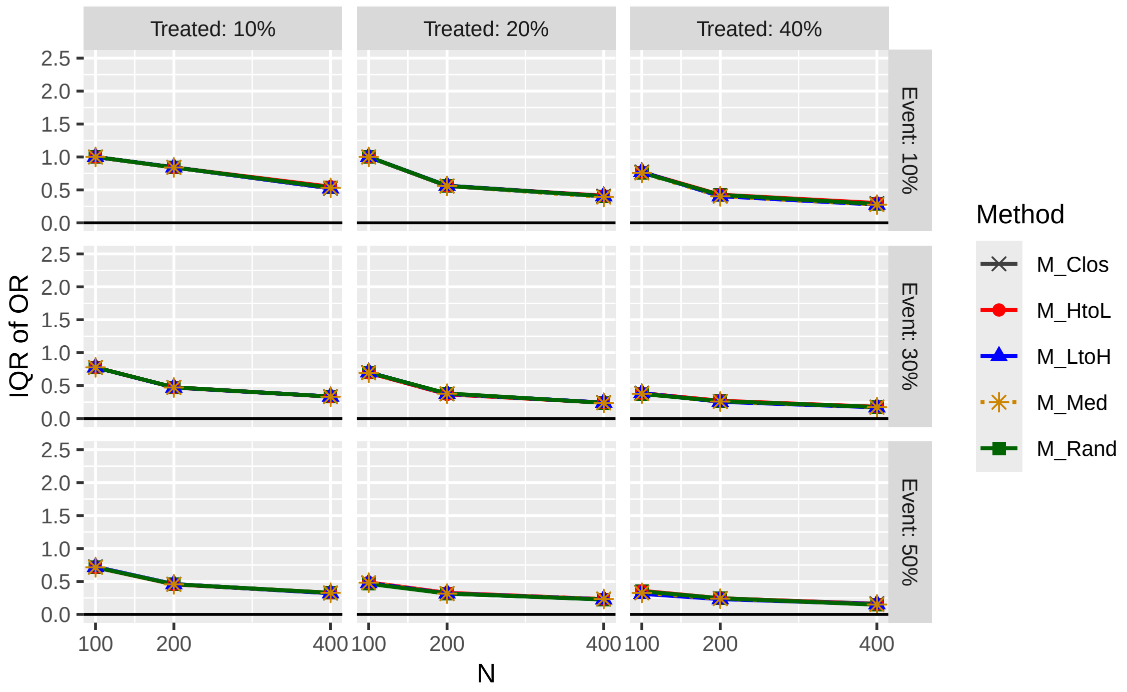

Figure S564. IQR for OR (unimodal continuous covariate, matching ratio 1:1, true OR: 0.5, c statistic: 0.6).

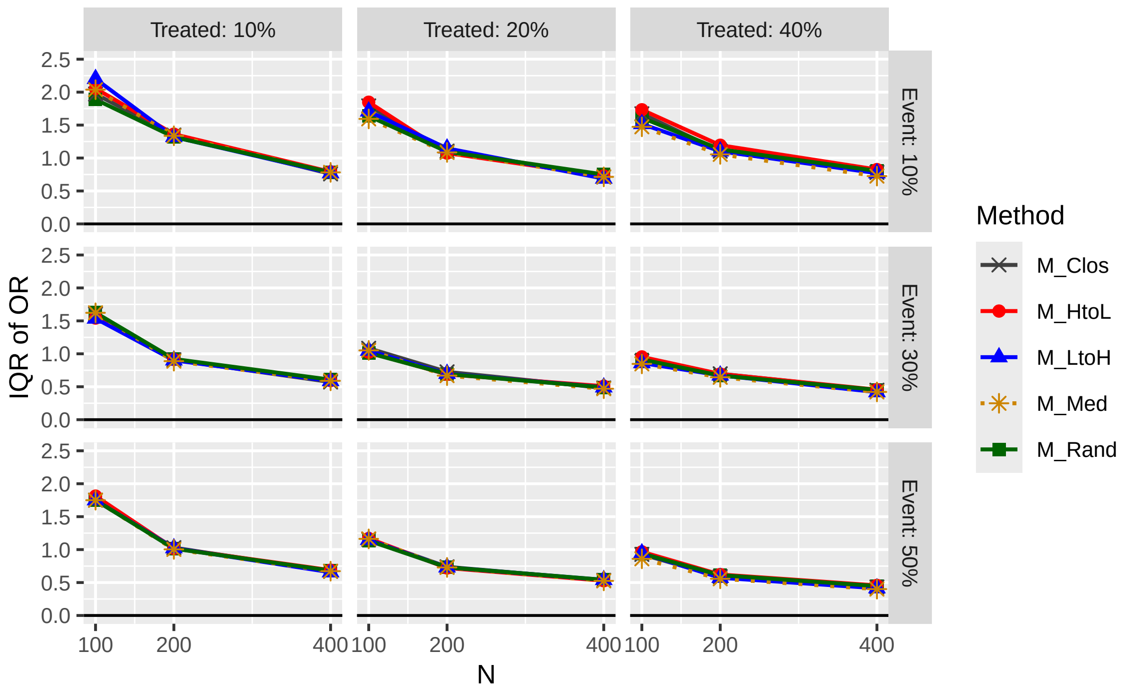

Figure S565. IQR for OR (unimodal continuous covariate, matching ratio 1:2, true OR: 1, c statistic: 0.85).

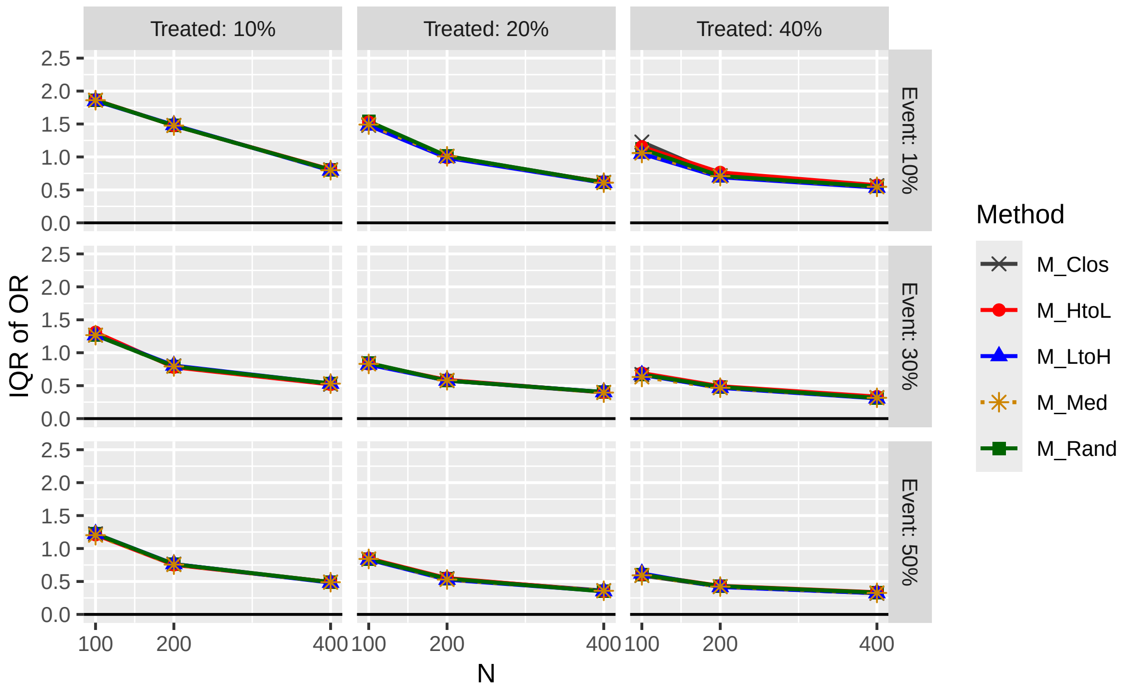

Figure S566. IQR for OR (unimodal continuous covariate, matching ratio 1:2, true OR: 1, c statistic: 0.6).

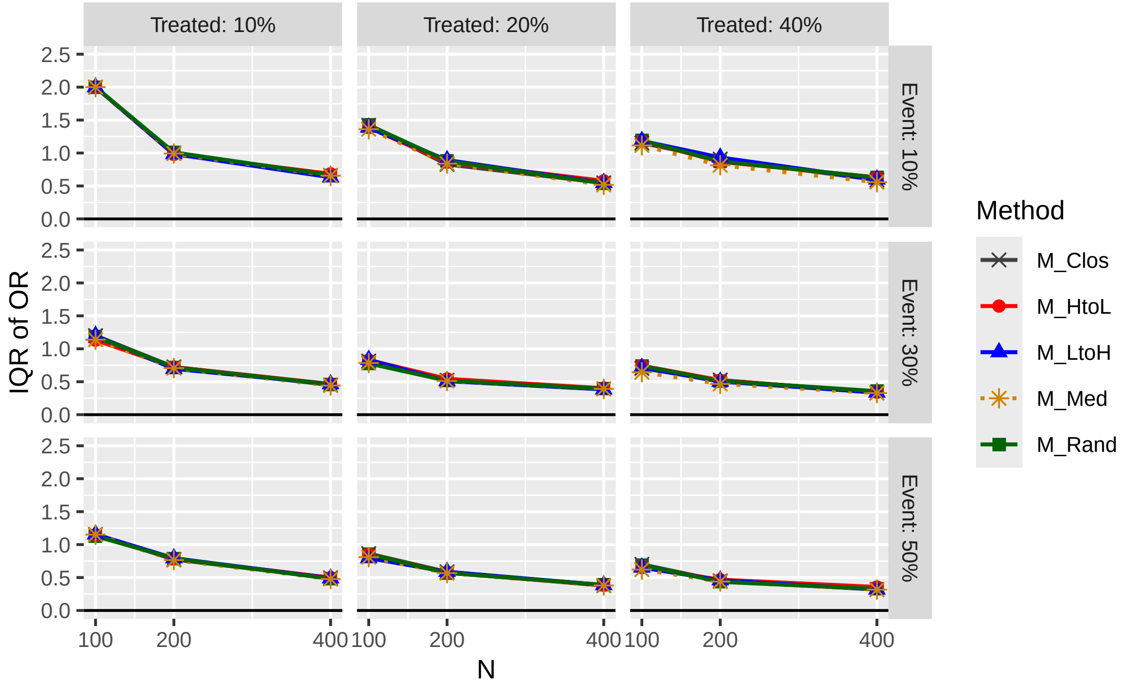

Figure S567. IQR for OR (unimodal continuous covariate, matching ratio 1:2, true OR: 0.75, c statistic: 0.85).

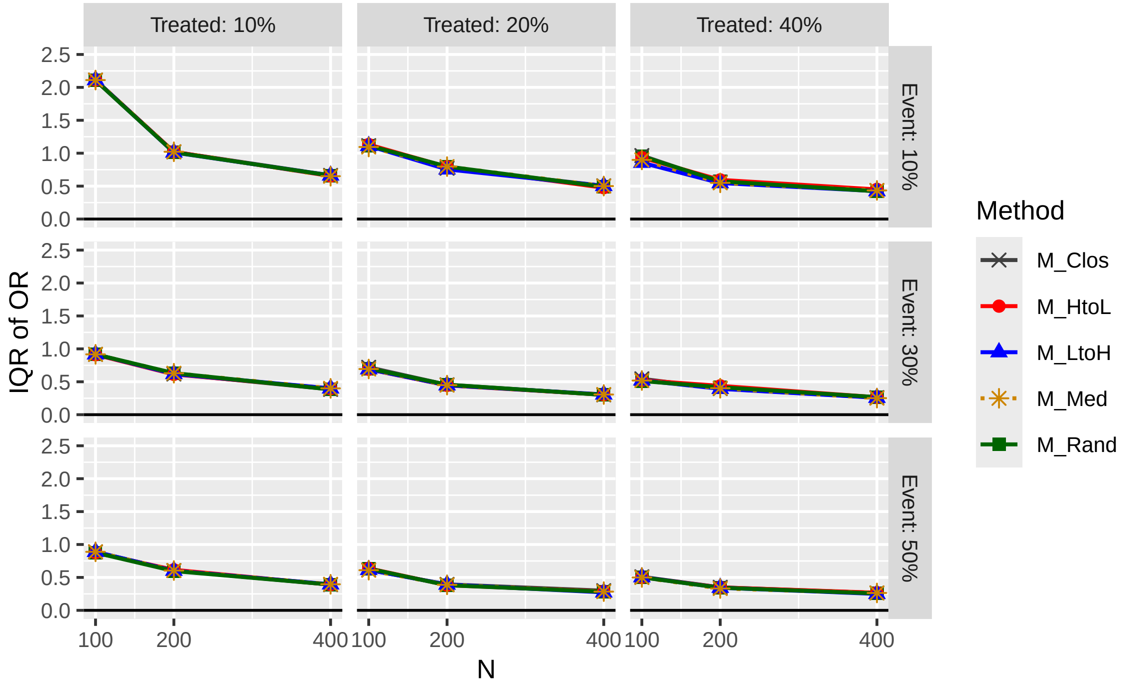

Figure S568. IQR for OR (unimodal continuous covariate, matching ratio 1:2, true OR: 0.75, c statistic: 0.6).

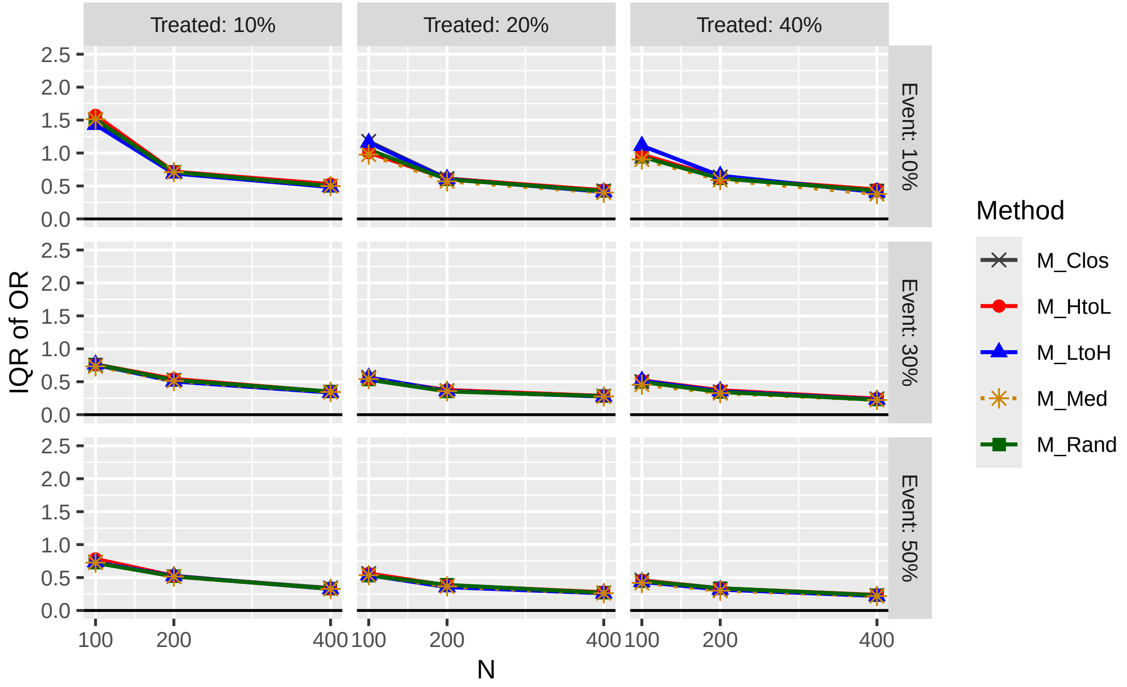

Figure S569. IQR for OR (unimodal continuous covariate, matching ratio 1:2, true OR: 0.5, c statistic: 0.85).

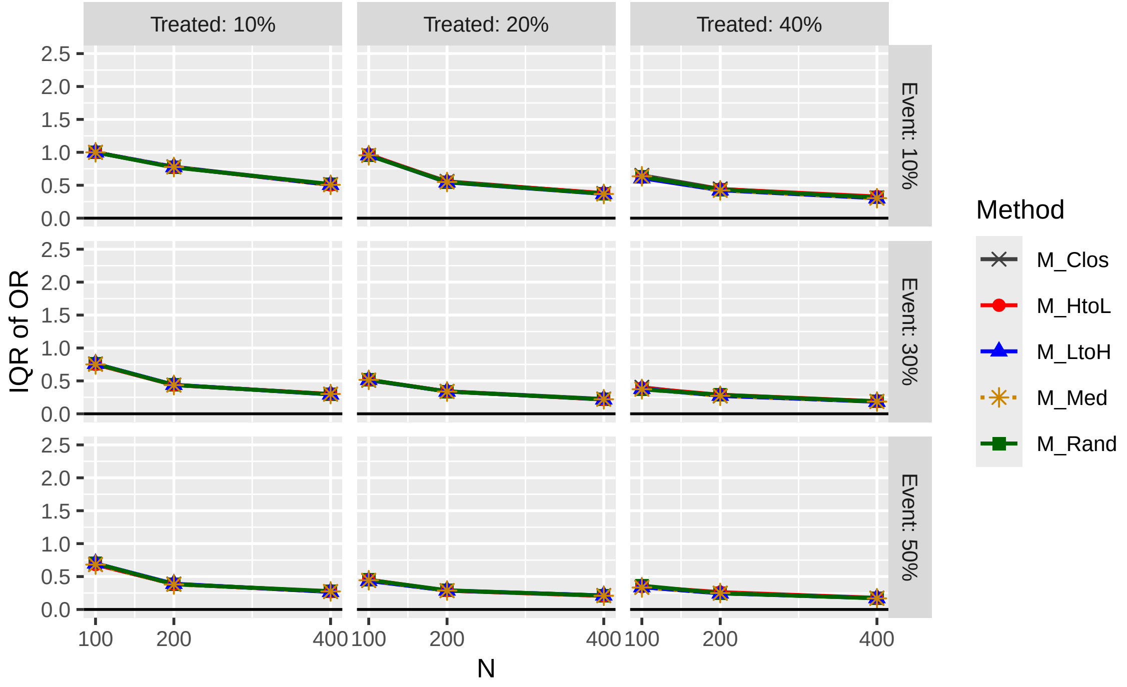

Figure S570. IQR for OR (unimodal continuous covariate, matching ratio 1:2, true OR: 0.5, c statistic: 0.6).

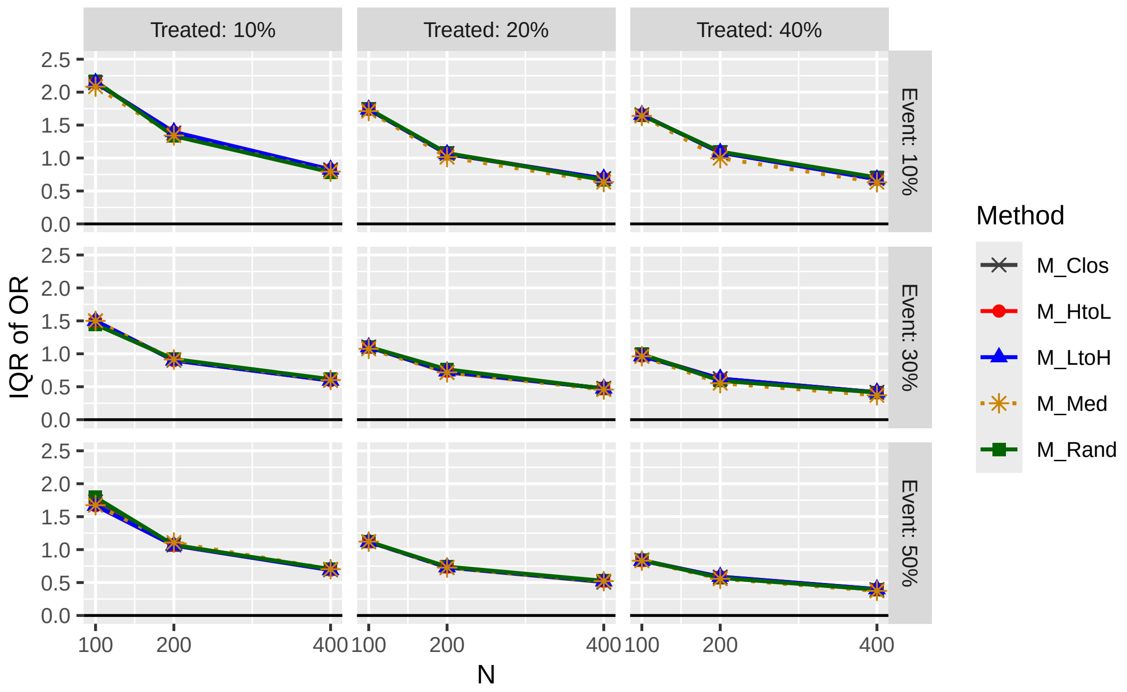

Figure S571. IQR for OR (categorical covariate, matching ratio 1:1, true OR: 1, c statistic: 0.85).

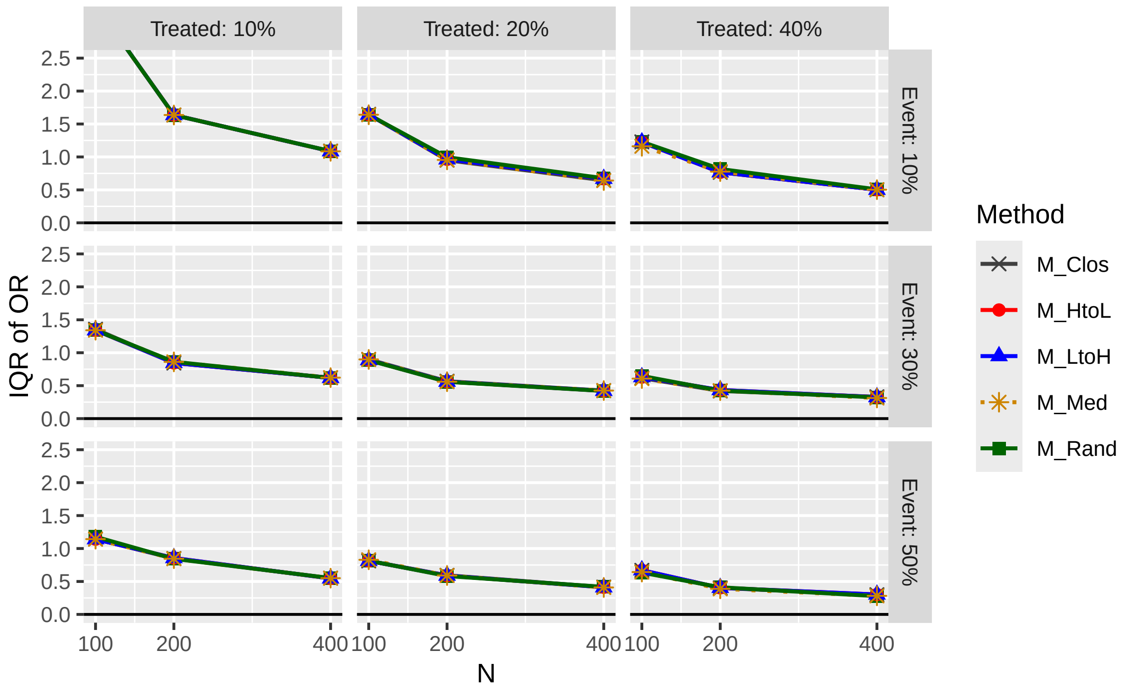

Figure S572. IQR for OR (categorical covariate, matching ratio 1:1, true OR: 1, c statistic: 0.6).

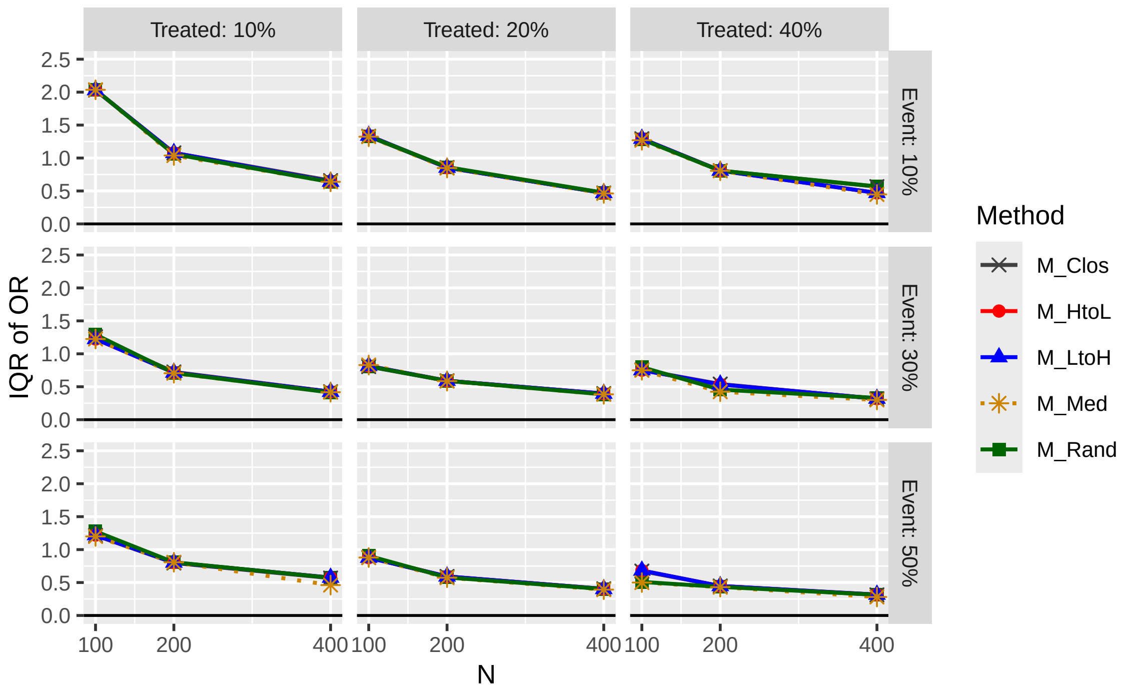

Figure S573. IQR for OR (categorical covariate, matching ratio 1:1, true OR: 0.75, c statistic: 0.85).

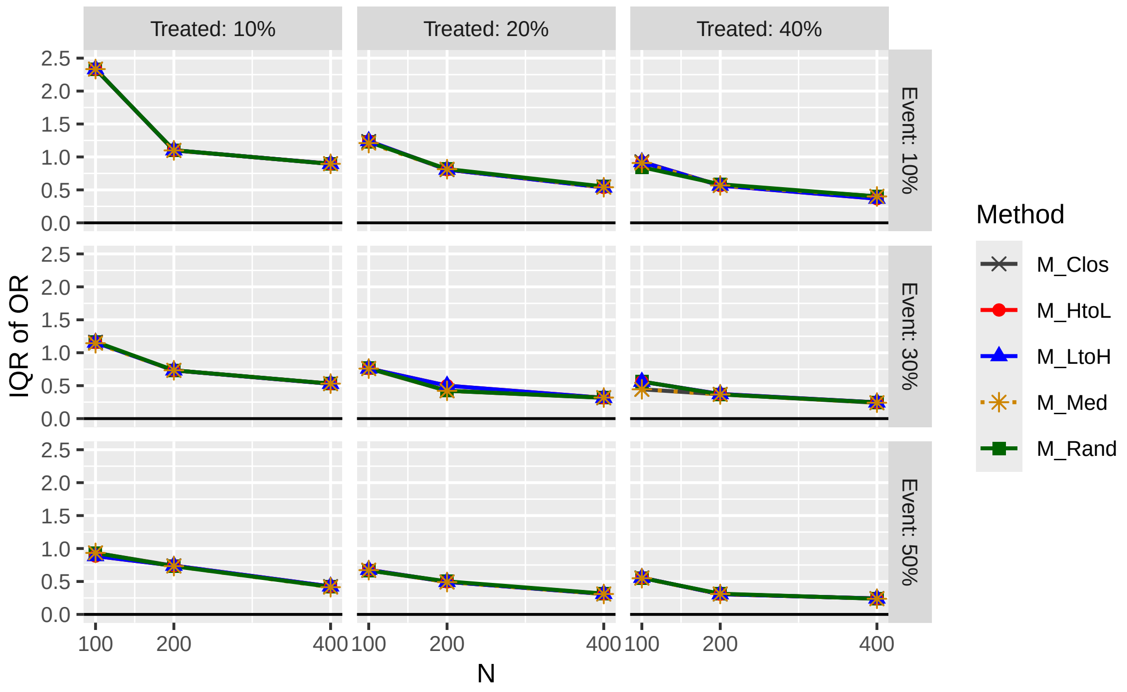

Figure S574. IQR for OR (categorical covariate, matching ratio 1:1, true OR: 0.75, c statistic: 0.6).

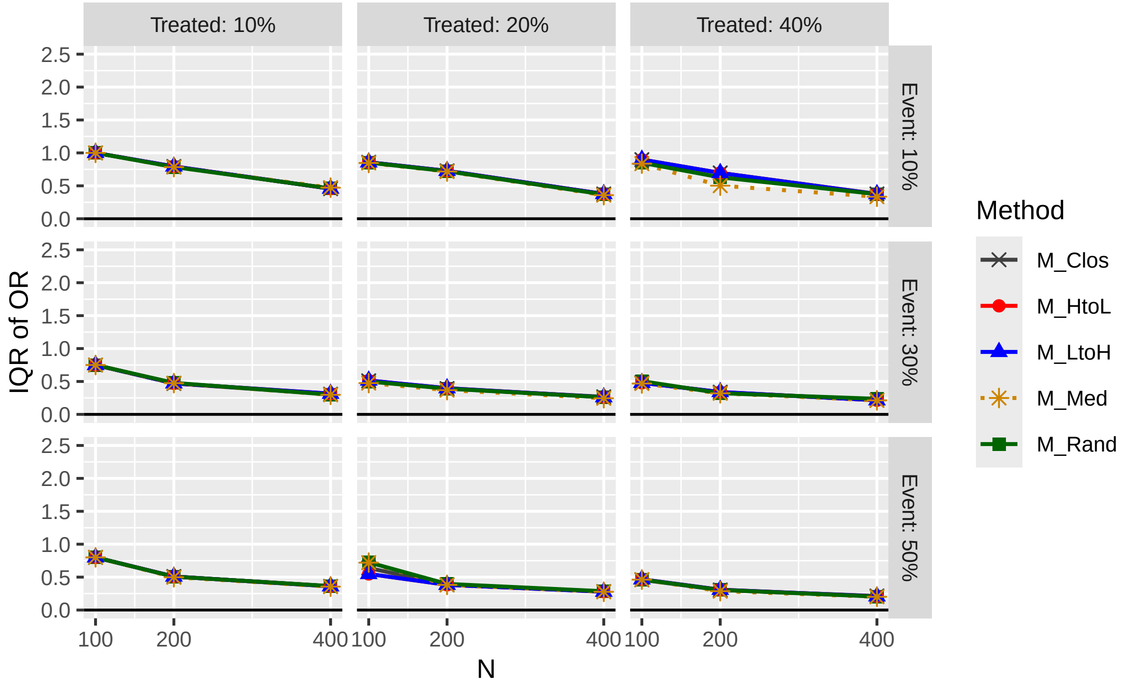

Figure S575. IQR for OR (categorical covariate, matching ratio 1:1, true OR: 0.5, c statistic: 0.85).

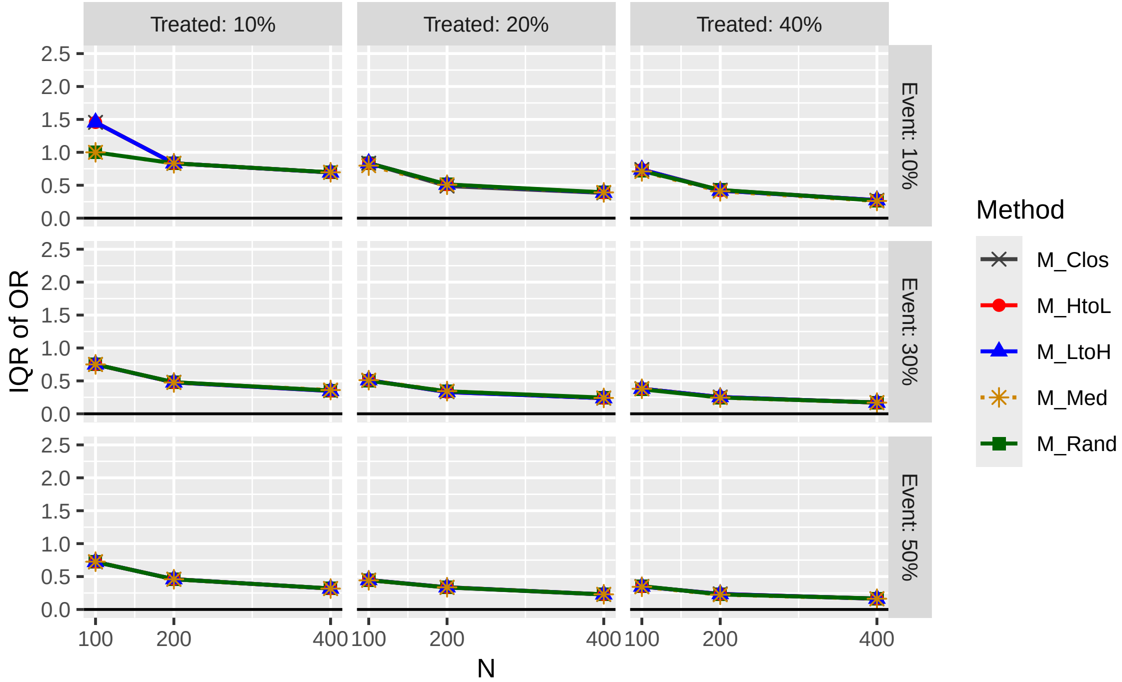

Figure S576. IQR for OR (categorical covariate, matching ratio 1:1, true OR: 0.5, c statistic: 0.6).

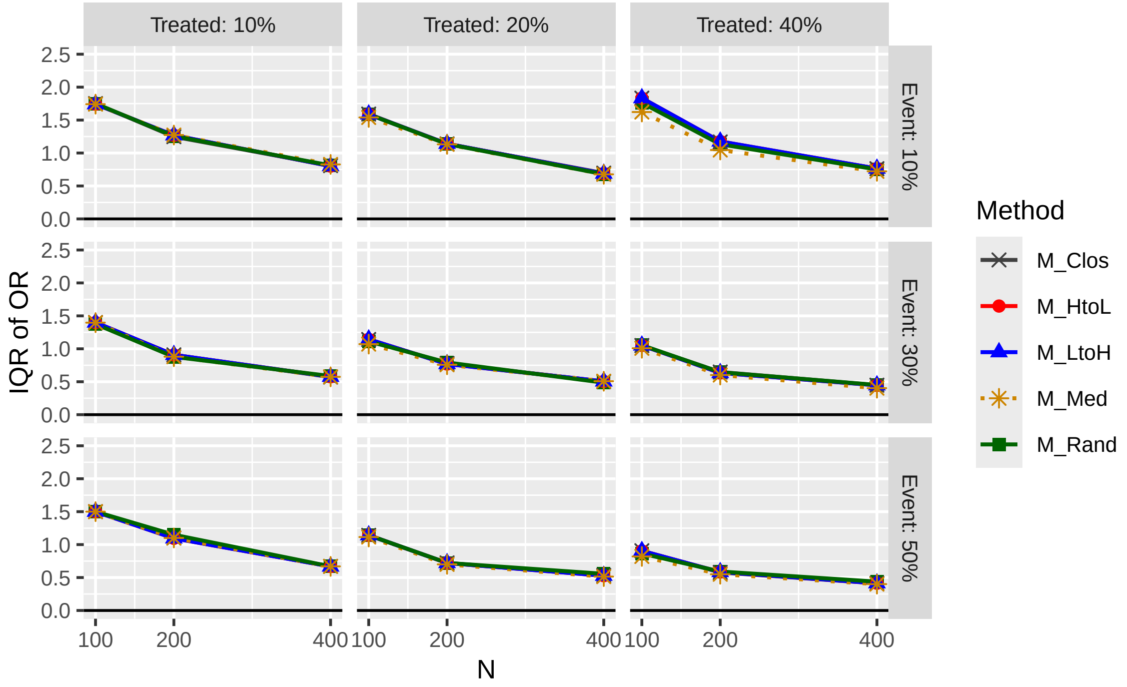

Figure S577. IQR for OR (categorical covariate, matching ratio 1:2, true OR: 1, c statistic: 0.85).

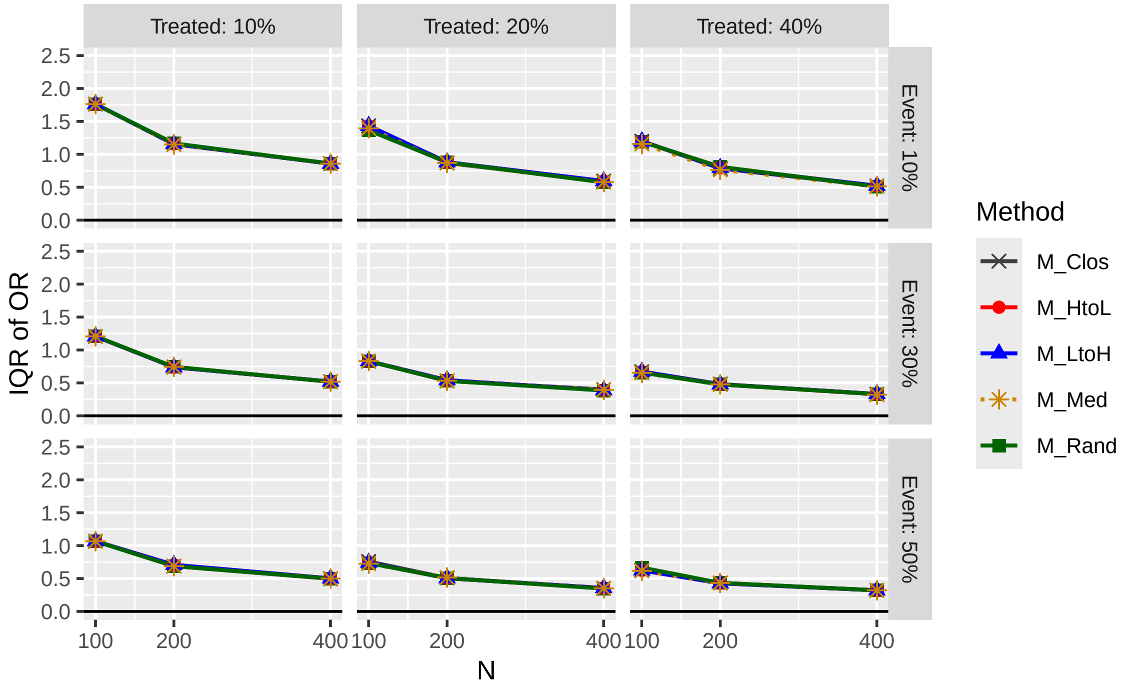

Figure S578. IQR for OR (categorical covariate, matching ratio 1:2, true OR: 1, c statistic: 0.6).

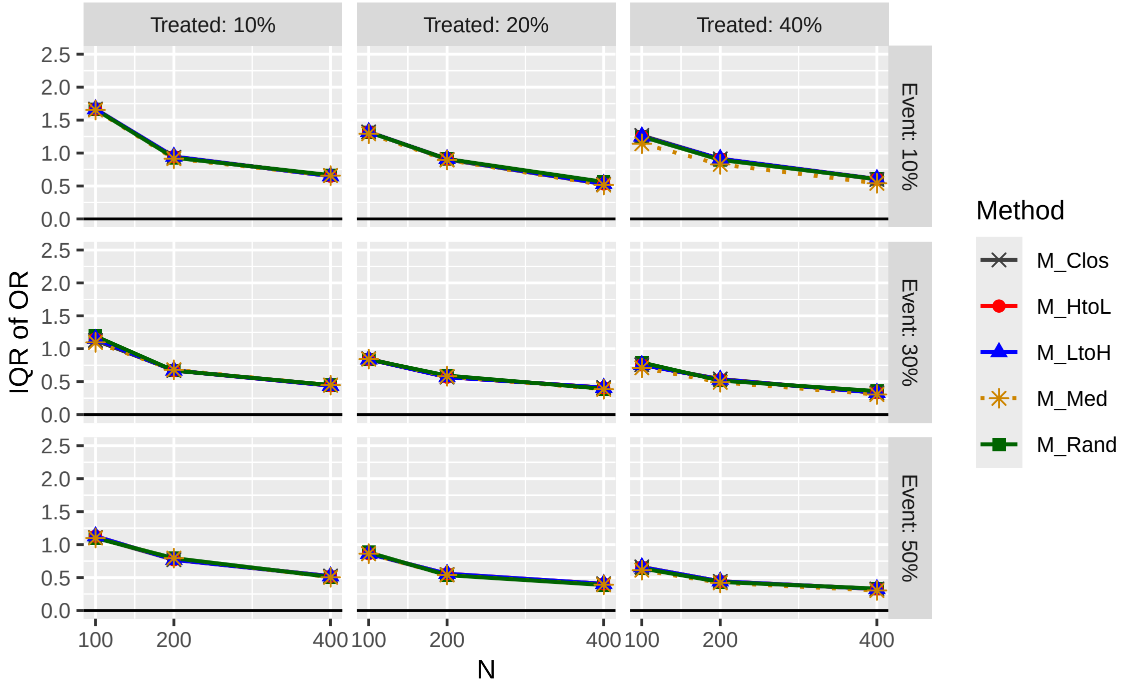

Figure S579. IQR for OR (categorical covariate, matching ratio 1:2, true OR: 0.75, c statistic: 0.85).

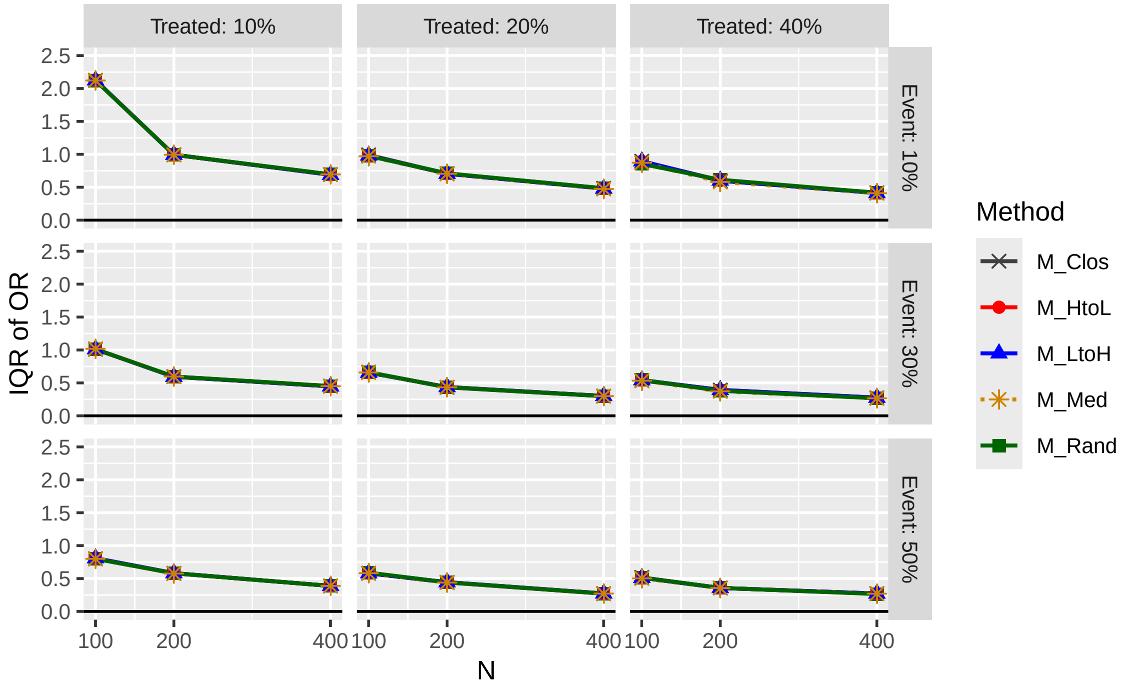

Figure S580. IQR for OR (categorical covariate, matching ratio 1:2, true OR: 0.75, c statistic: 0.6).

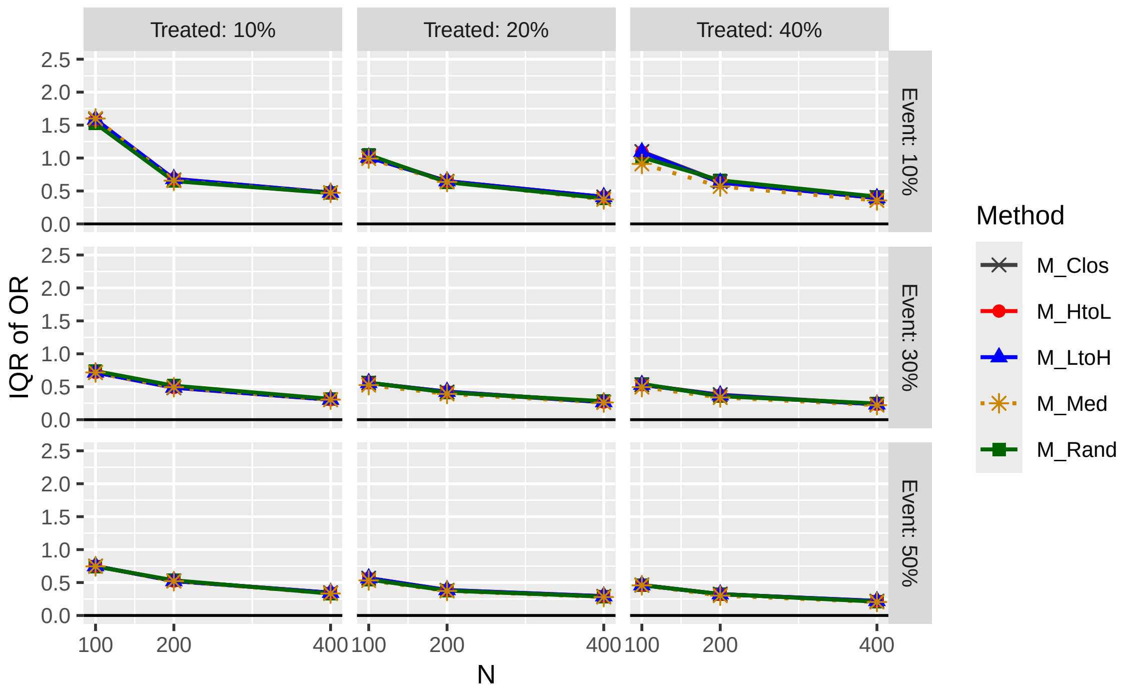

Figure S581. IQR for OR (categorical covariate, matching ratio 1:2, true OR: 0.5, c statistic: 0.85).

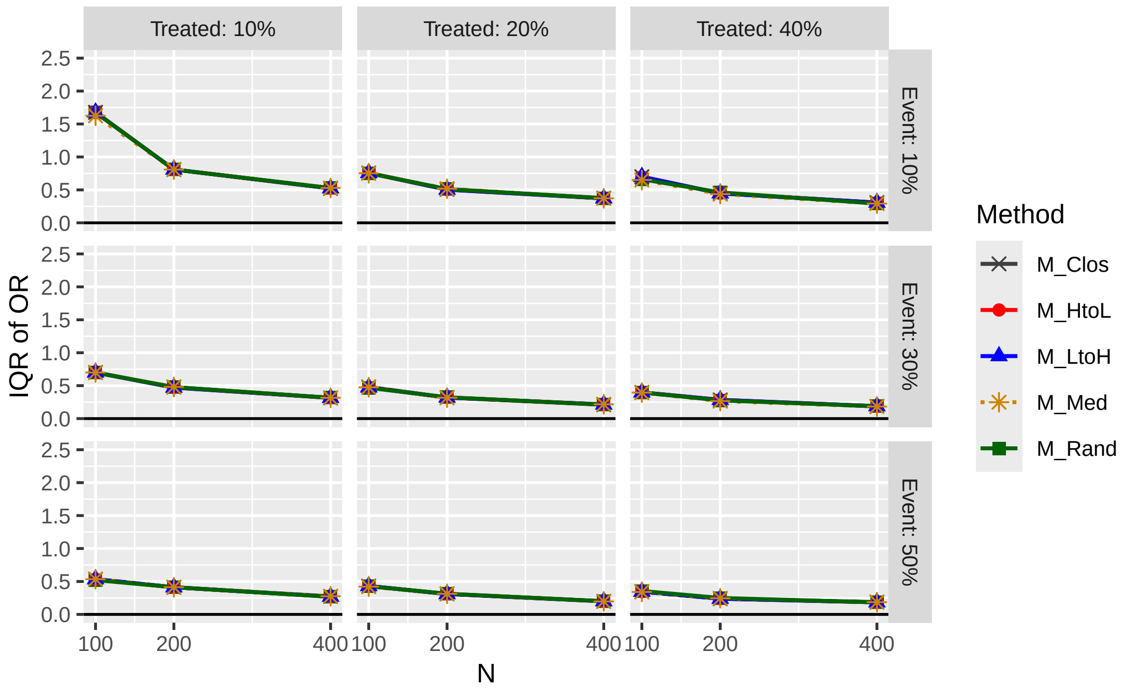

Figure S582. IQR for OR (categorical covariate, matching ratio 1:2, true OR: 0.5, c statistic: 0.6).

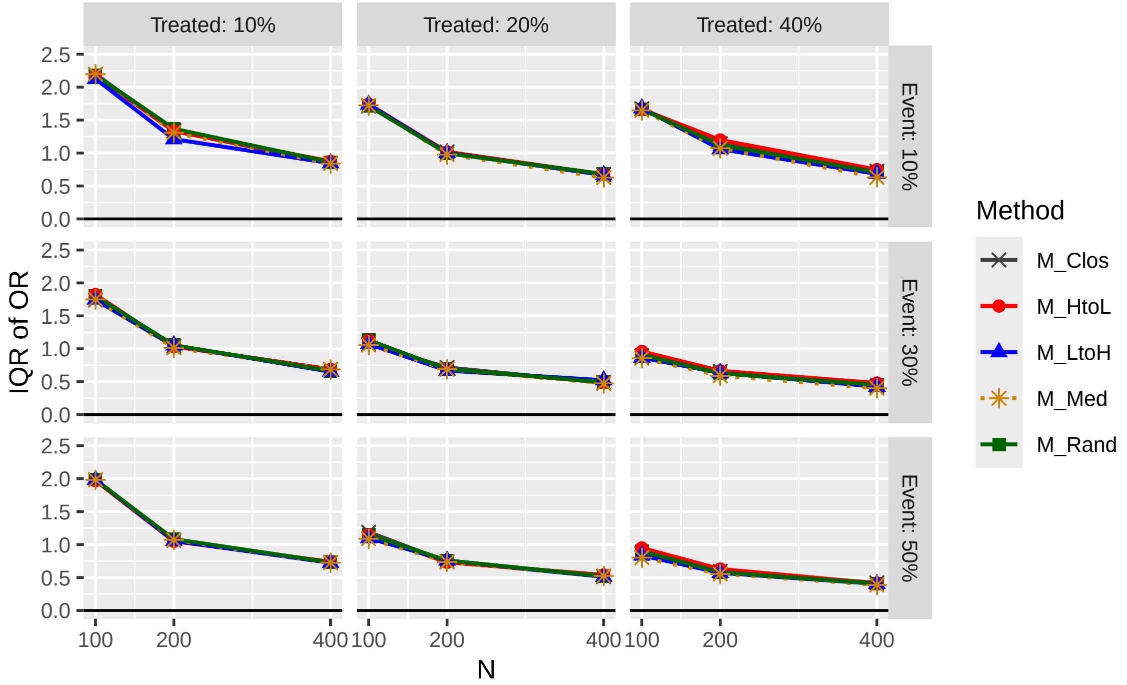

Figure S583. IQR for OR (multimodal continuous covariate, matching ratio 1:1, true OR: 1, c statistic: 0.85).

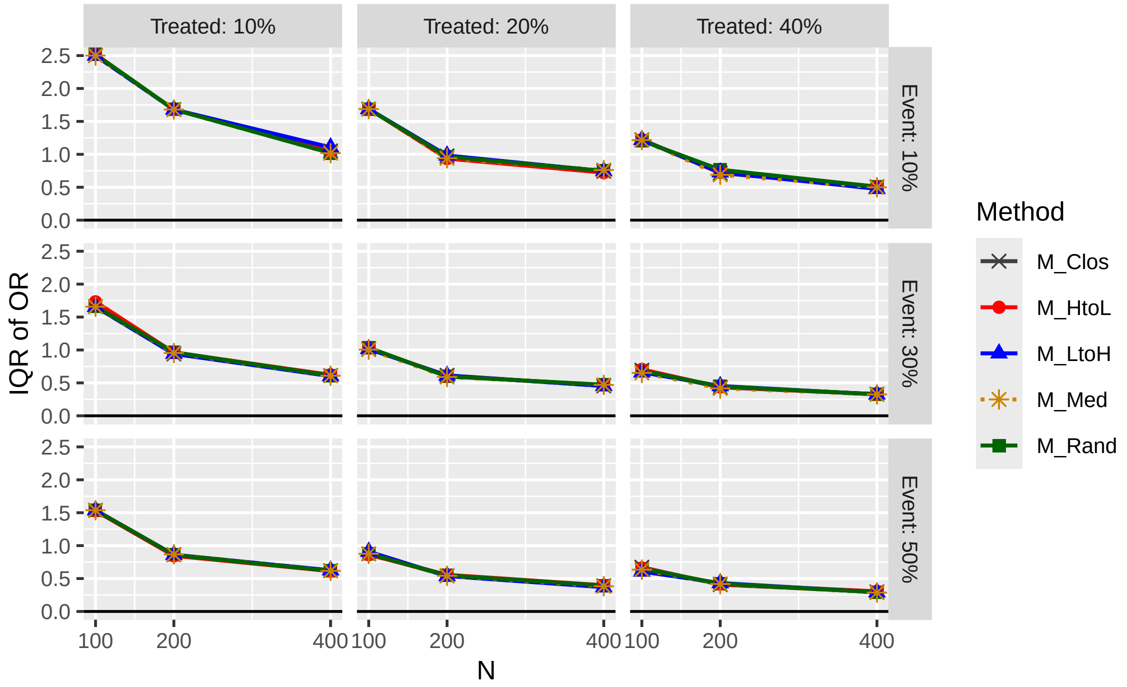

Figure S584. IQR for OR (multimodal continuous covariate, matching ratio 1:1, true OR: 1, c statistic: 0.6).

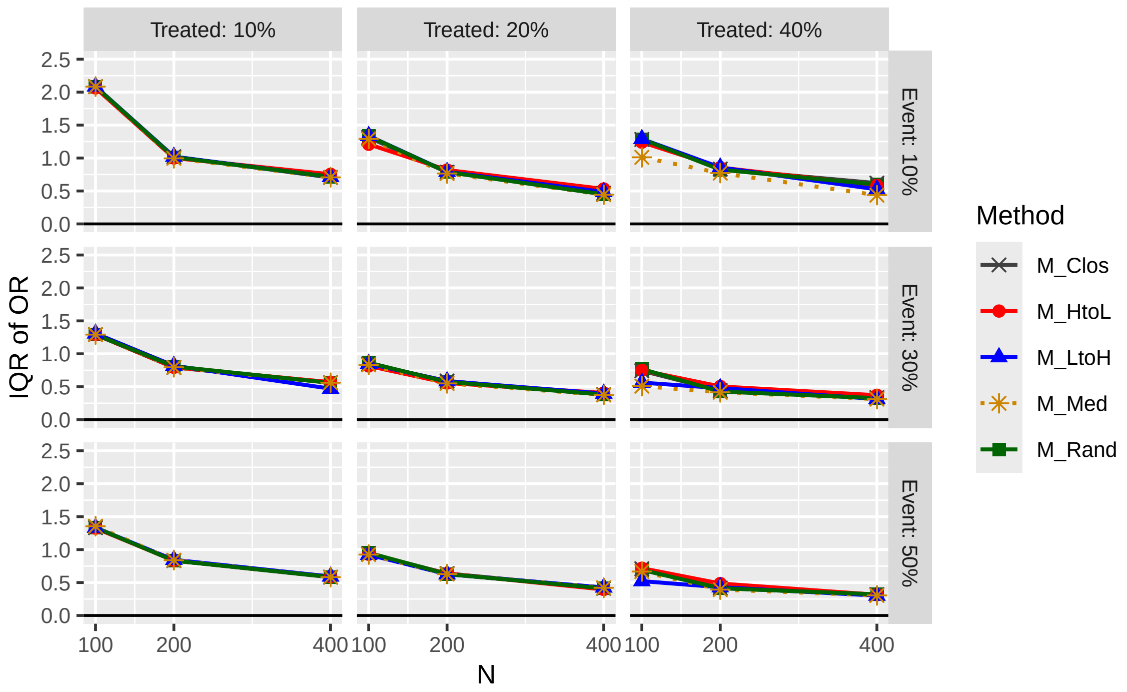

Figure S585. IQR for OR (multimodal continuous covariate, matching ratio 1:1, true OR: 0.75, c statistic: 0.85).

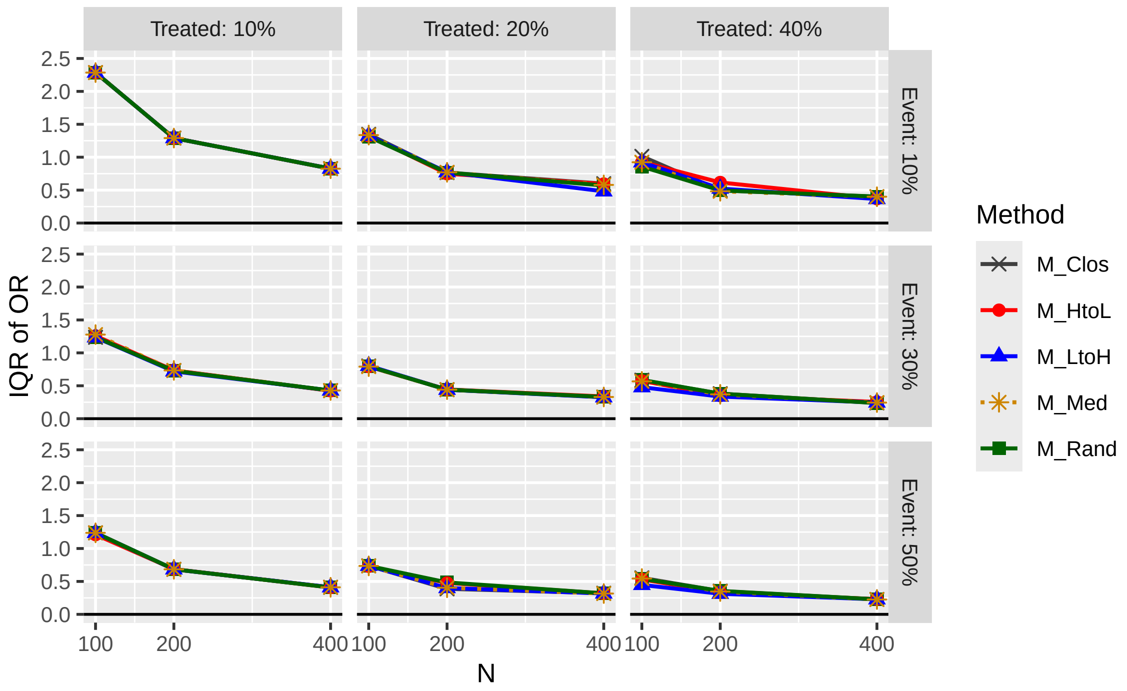

Figure S586. IQR for OR (multimodal continuous covariate, matching ratio 1:1, true OR: 0.75, c statistic: 0.6).

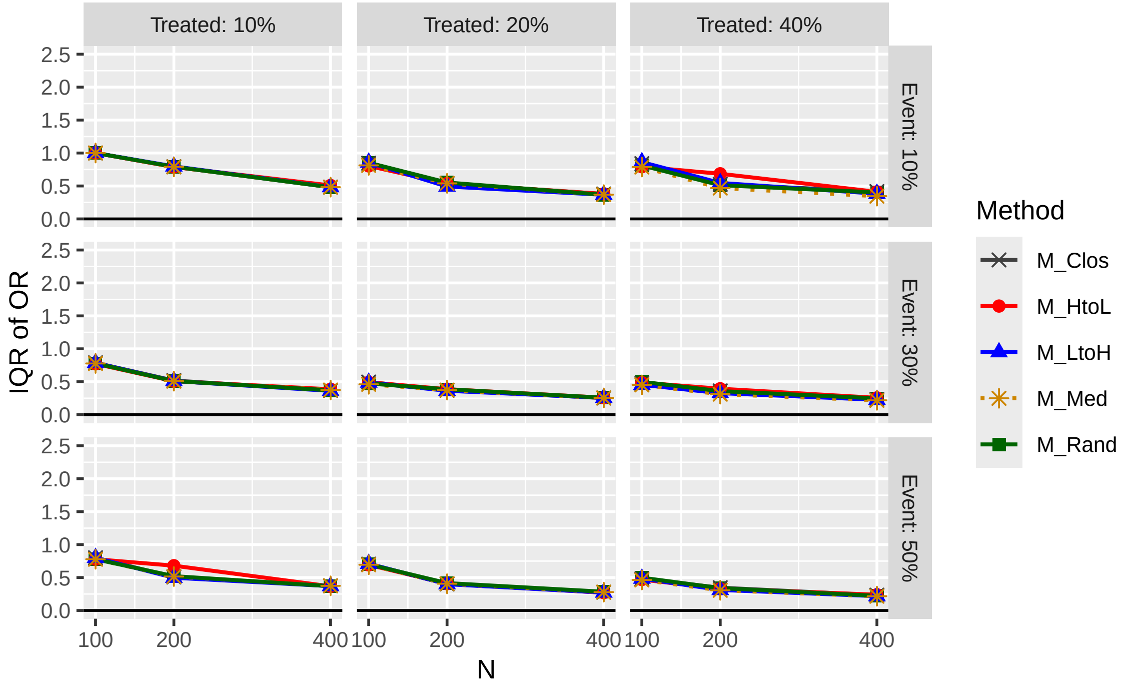

Figure S587. IQR for OR (multimodal continuous covariate, matching ratio 1:1, true OR: 0.5, c statistic: 0.85).

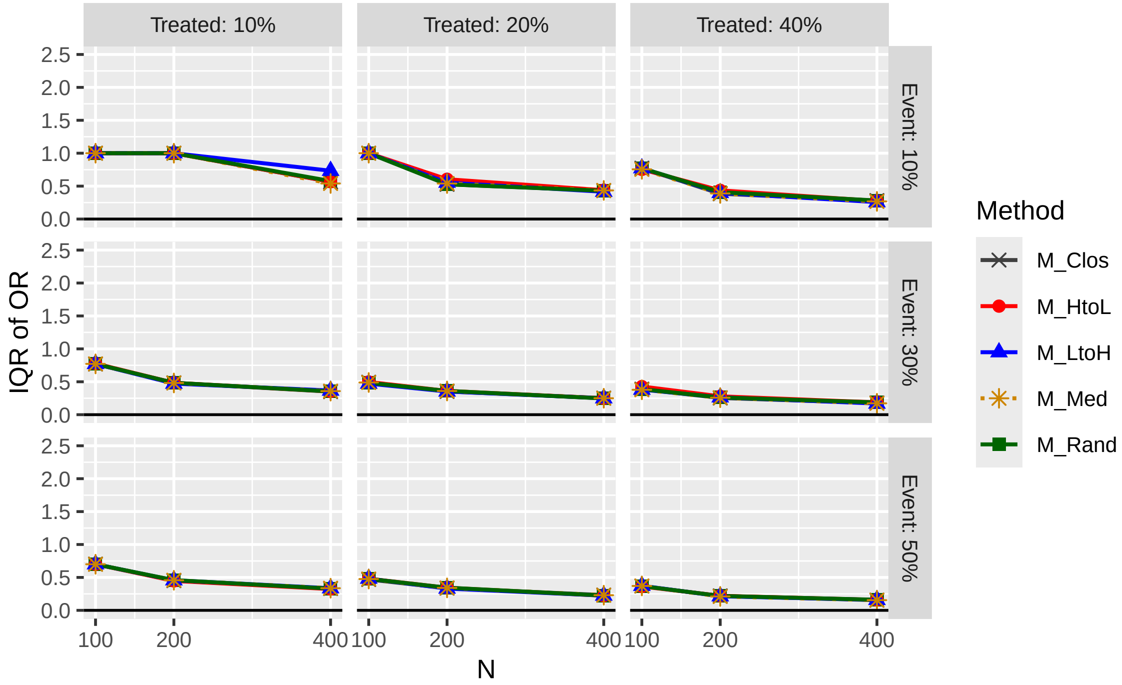

Figure S588. IQR for OR (multimodal continuous covariate, matching ratio 1:1, true OR: 0.5, c statistic: 0.6).

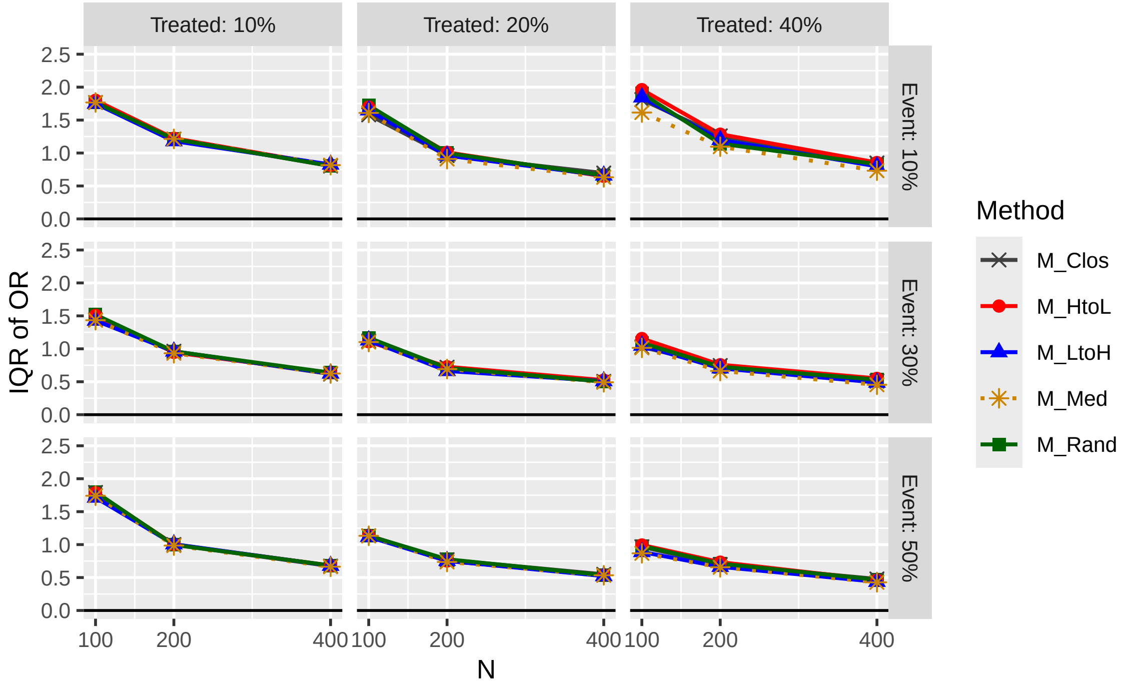

Figure S589. IQR for OR (multimodal continuous covariate, matching ratio 1:2, true OR: 1, c statistic: 0.85).

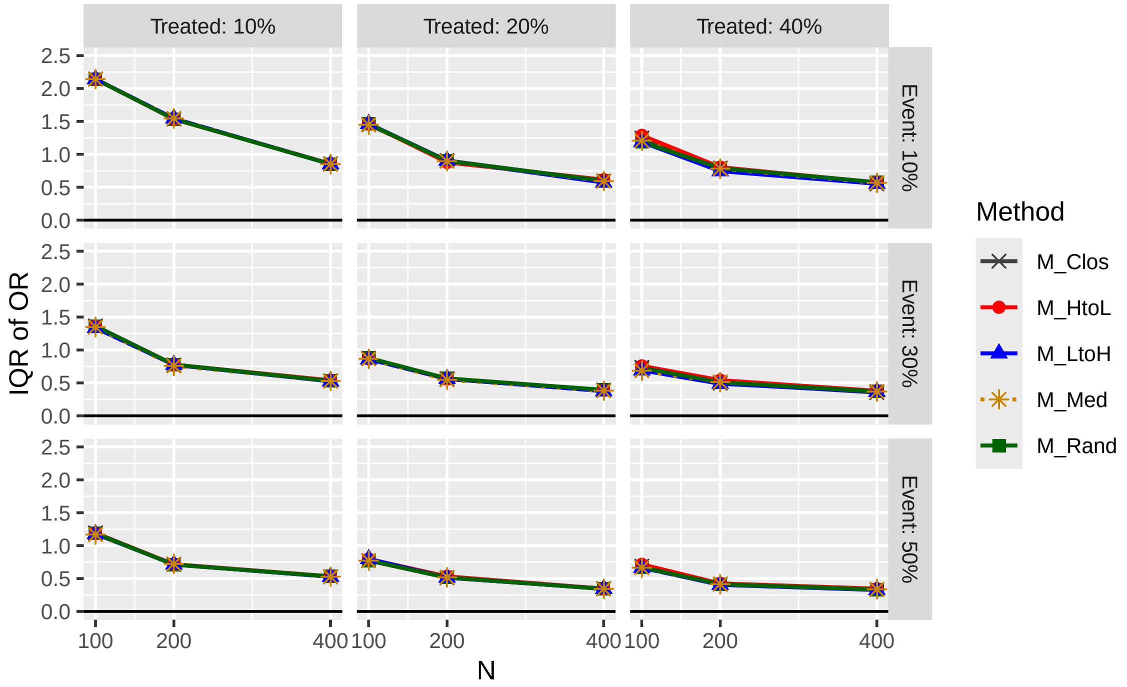

Figure S590. IQR for OR (multimodal continuous covariate, matching ratio 1:2, true OR: 1, c statistic: 0.6).

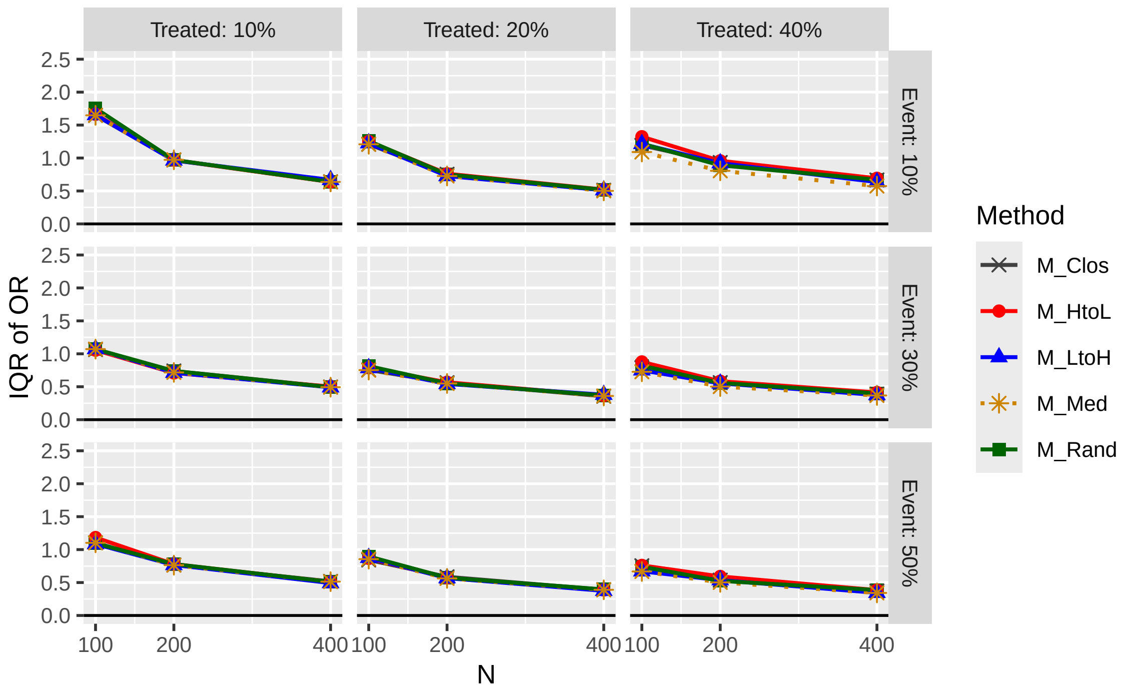

Figure S591. IQR for OR (multimodal continuous covariate, matching ratio 1:2, true OR: 0.75, c statistic: 0.85).

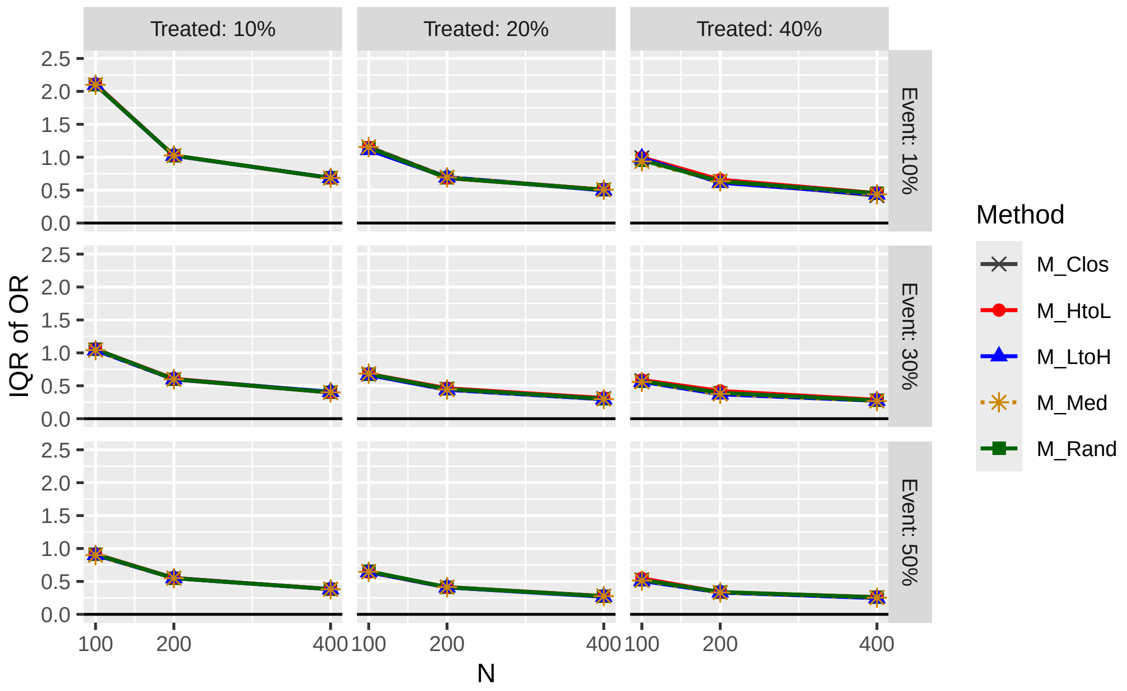

Figure S592. IQR for OR (multimodal continuous covariate, matching ratio 1:2, true OR: 0.75, c statistic: 0.6).

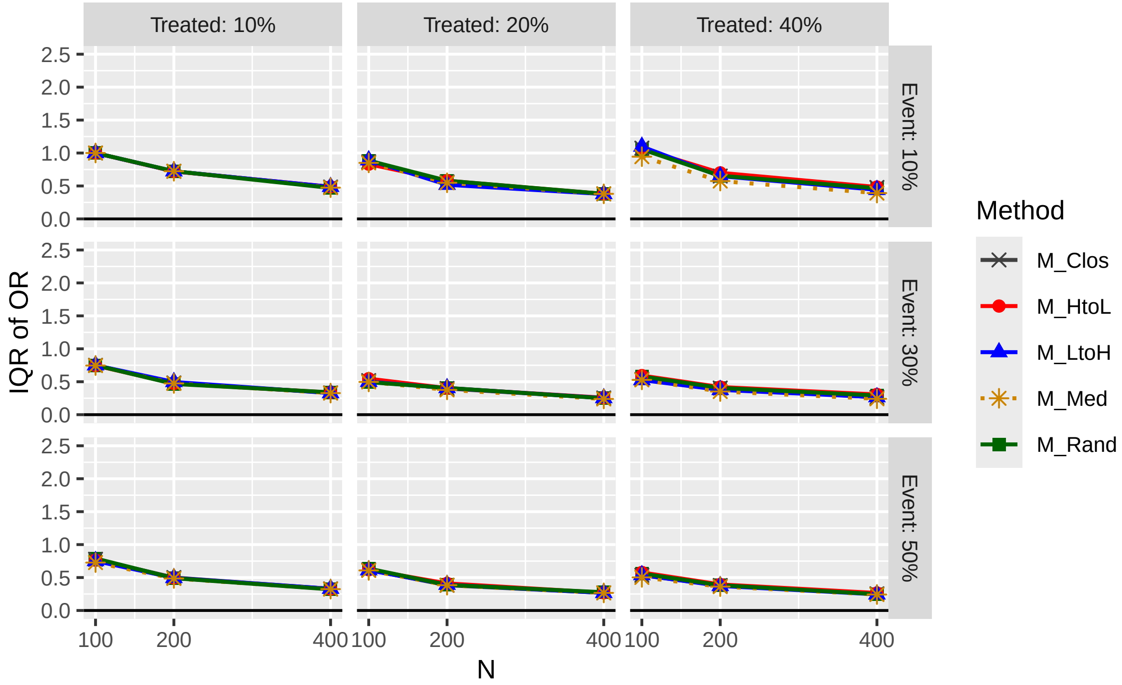

Figure S593. IQR for OR (multimodal continuous covariate, matching ratio 1:2, true OR: 0.5, c statistic: 0.85).

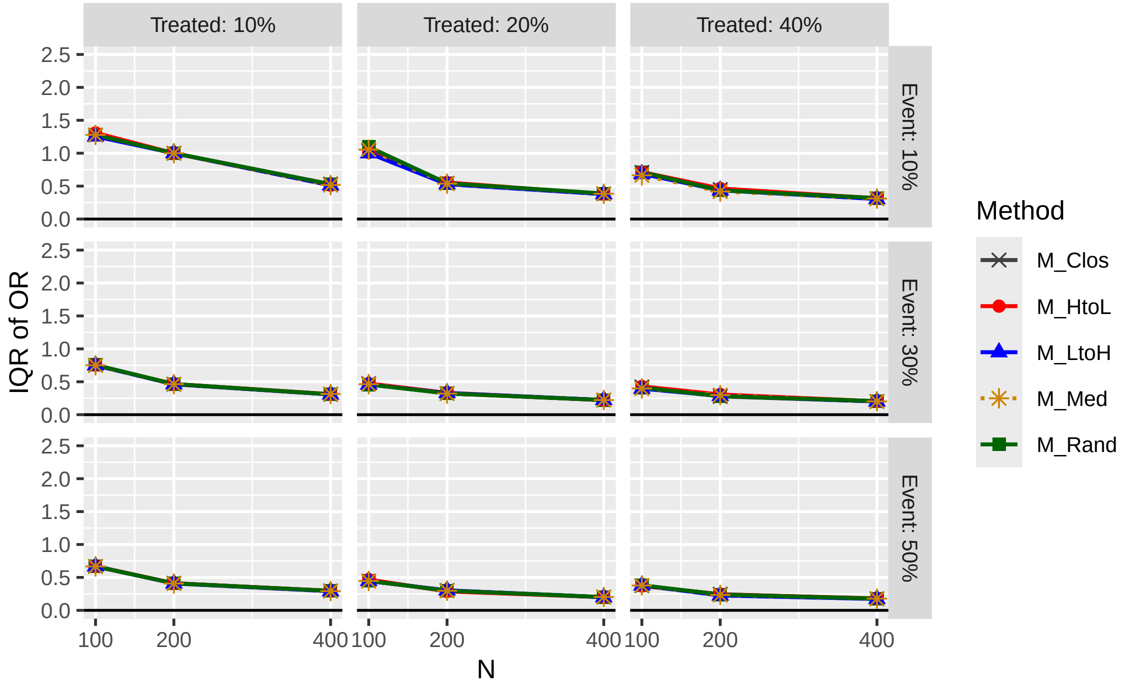

Figure S594. IQR for OR (multimodal continuous covariate, matching ratio 1:2, true OR: 0.5, c statistic: 0.6).

S10. Median absolute difference of OR for 10% data addition (caliper: 15%)

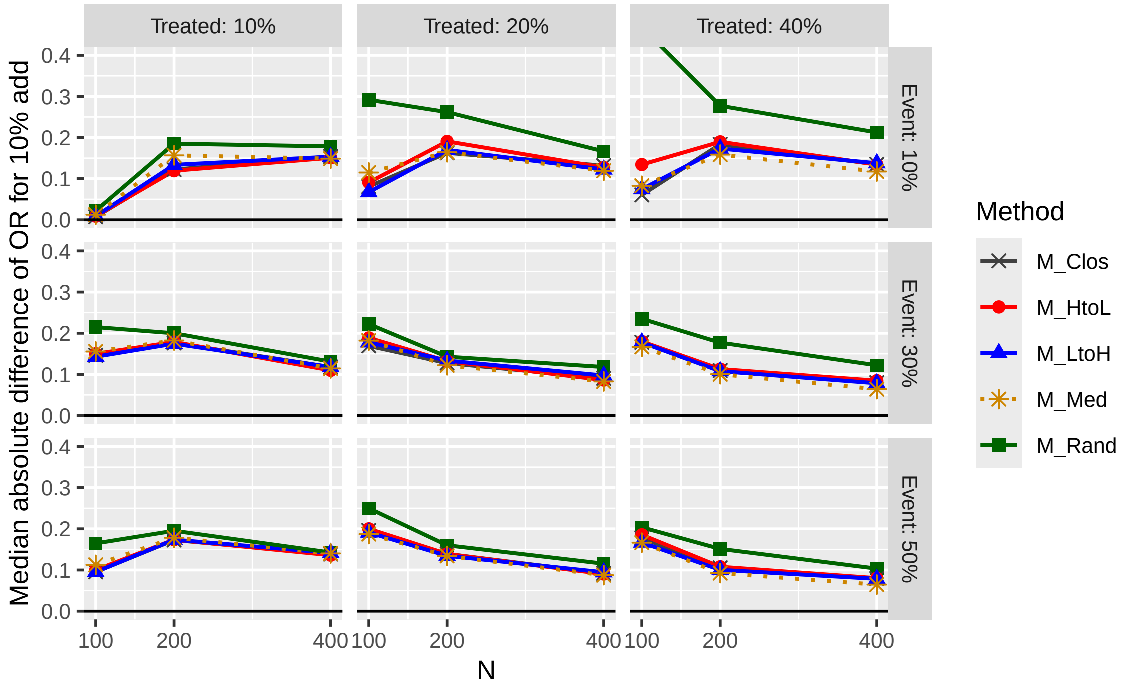

Figure S595. Median absolute difference of OR for 10% data addition (unimodal continuous covariate, matching ratio 1:1, true OR: 1, c statistic: 0.85).

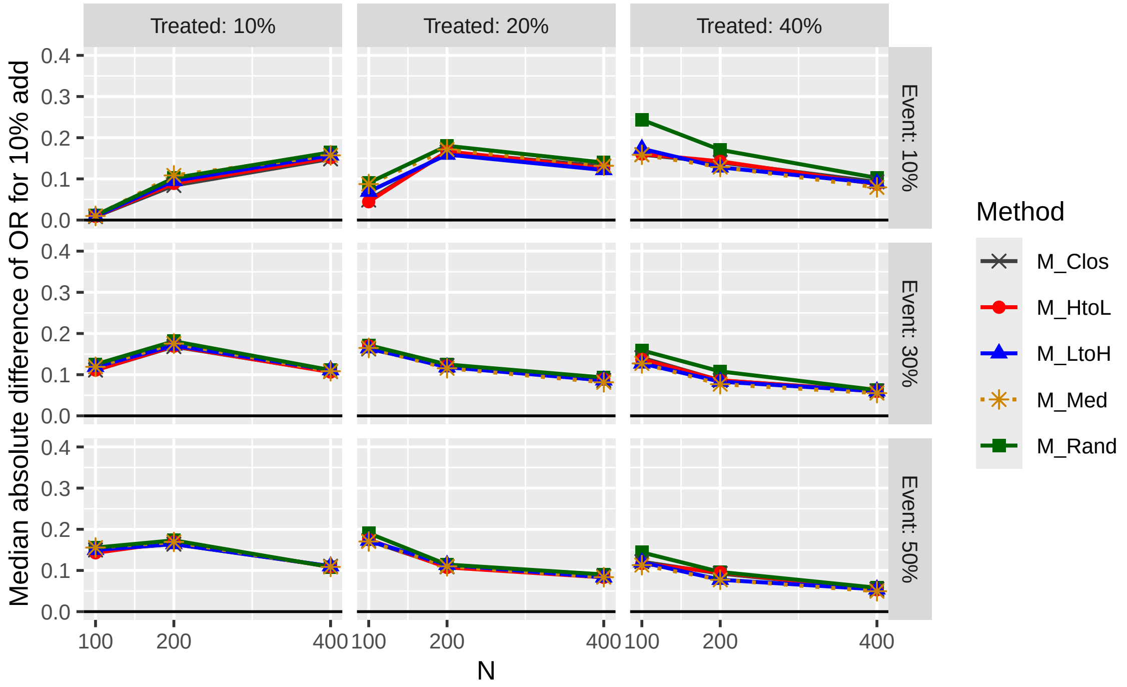

Figure S596. Median absolute difference of OR for 10% data addition (unimodal continuous covariate, matching ratio 1:1, true OR: 1, c statistic: 0.6).

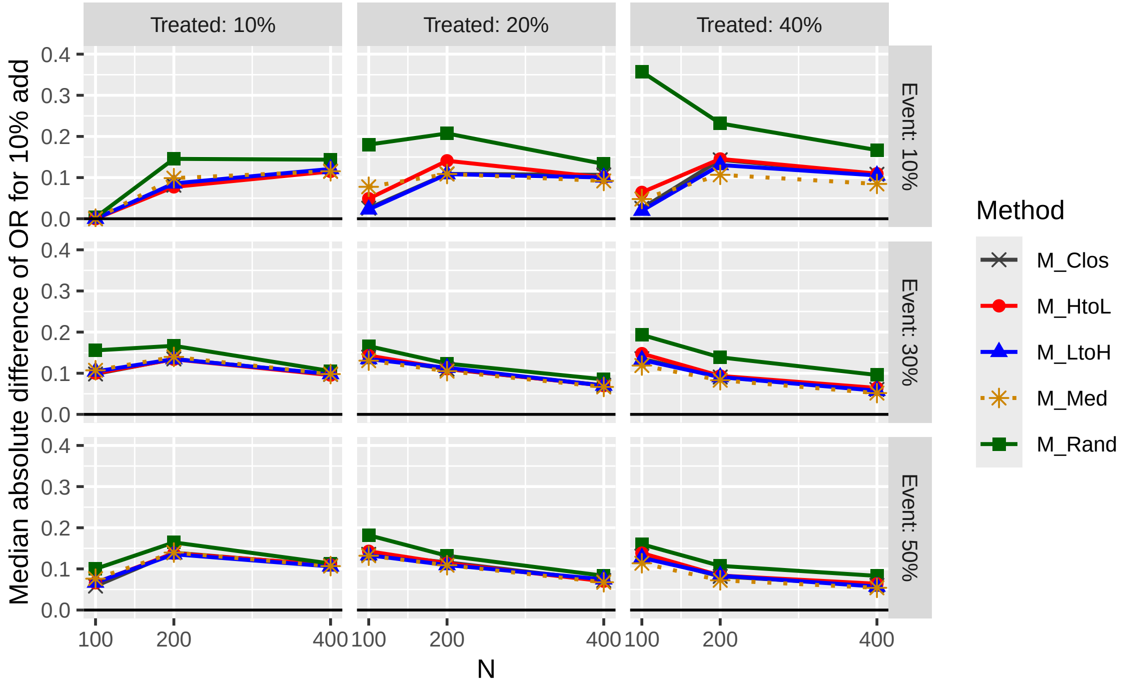

Figure S597. Median absolute difference of OR for 10% data addition (unimodal continuous covariate, matching ratio 1:1, true OR: 0.75, c statistic: 0.85).

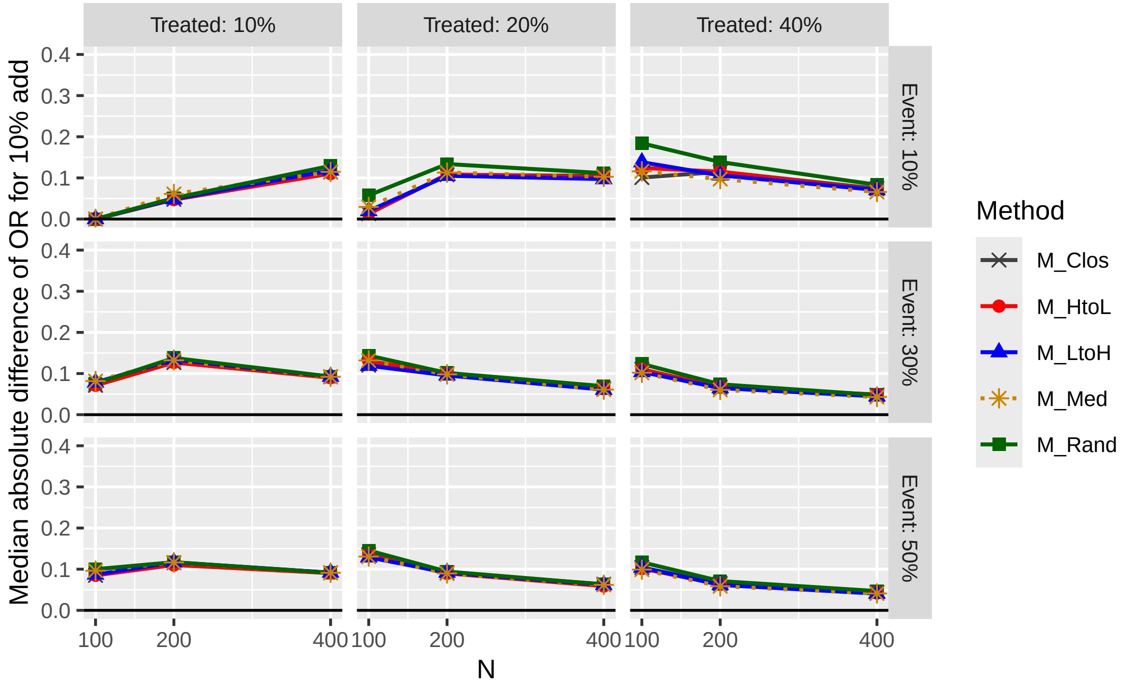

Figure S598. Median absolute difference of OR for 10% data addition (unimodal continuous covariate, matching ratio 1:1, true OR: 0.75, c statistic: 0.6).

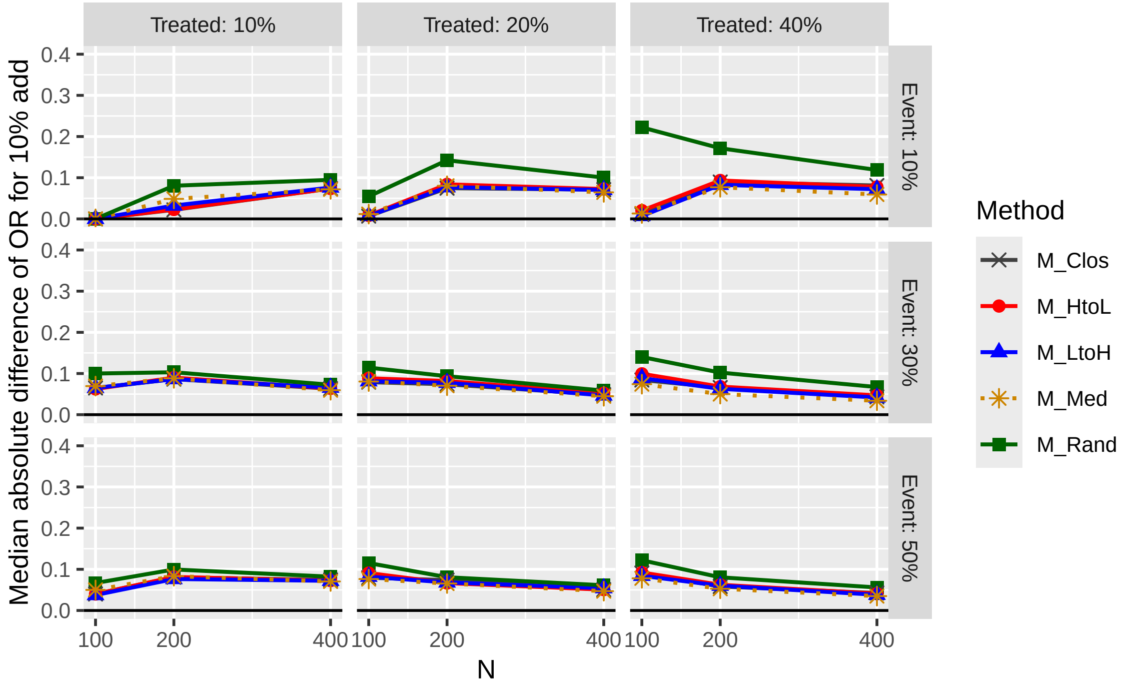

Figure S599. Median absolute difference of OR for 10% data addition (unimodal continuous covariate, matching ratio 1:1, true OR: 0.5, c statistic: 0.85).

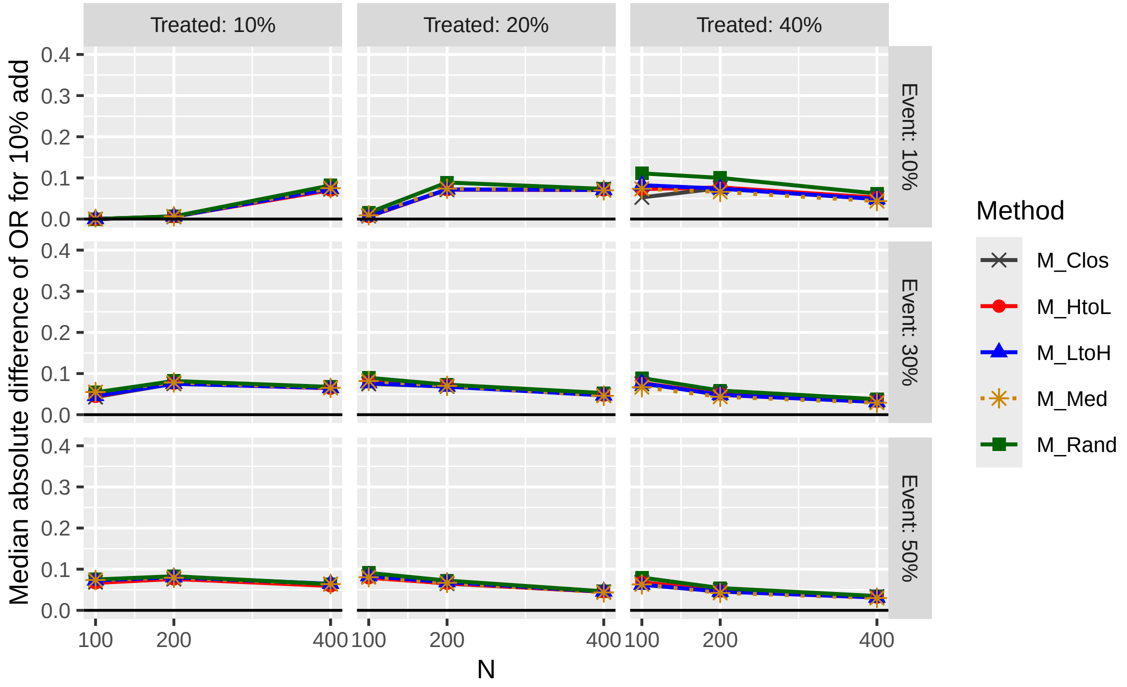

Figure S600. Median absolute difference of OR for 10% data addition (unimodal continuous covariate, matching ratio 1:1, true OR: 0.5, c statistic: 0.6).

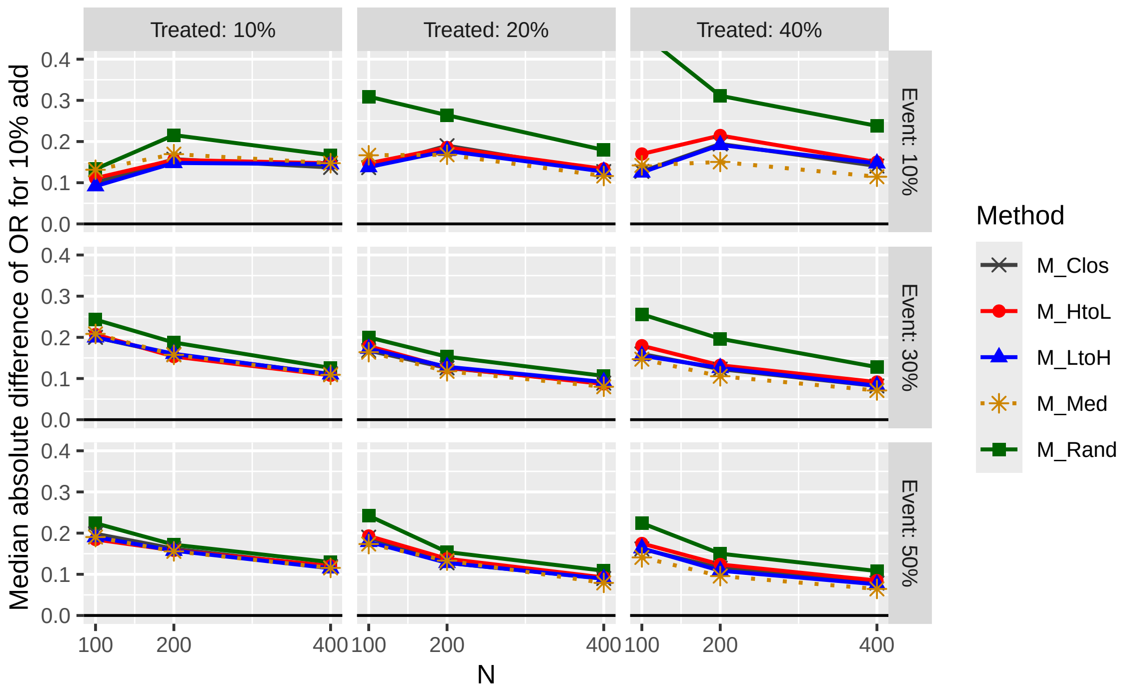

Figure S601. Median absolute difference of OR for 10% data addition (unimodal continuous covariate, matching ratio 1:2, true OR: 1, c statistic: 0.85).

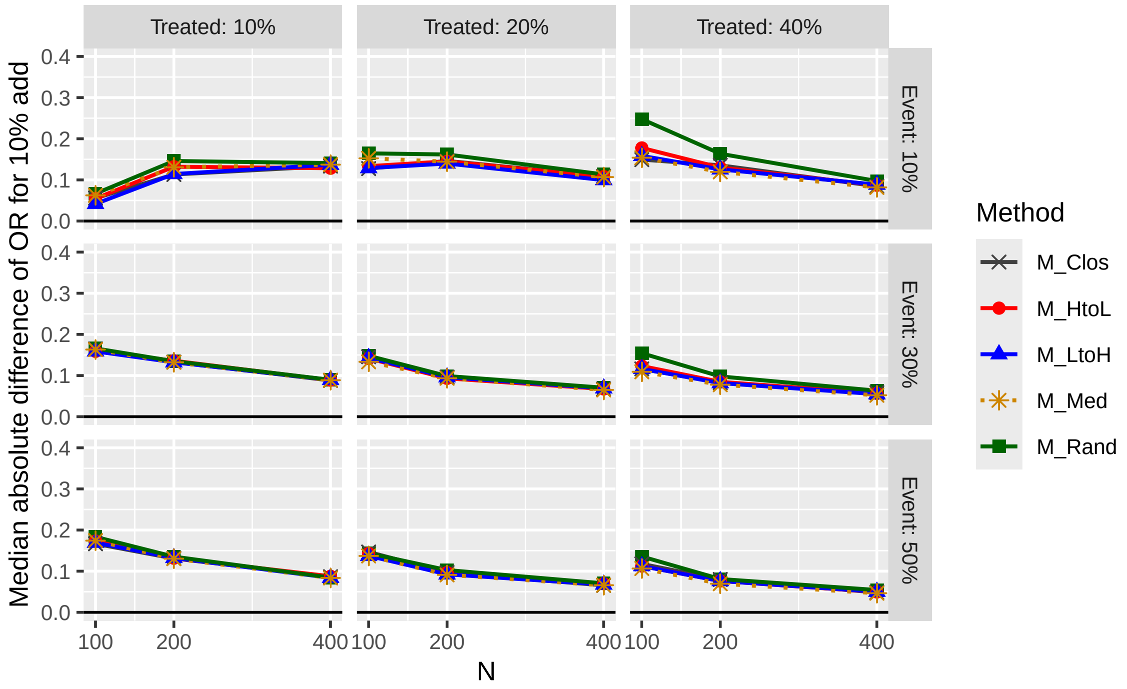

Figure S602. Median absolute difference of OR for 10% data addition (unimodal continuous covariate, matching ratio 1:2, true OR: 1, c statistic: 0.6).

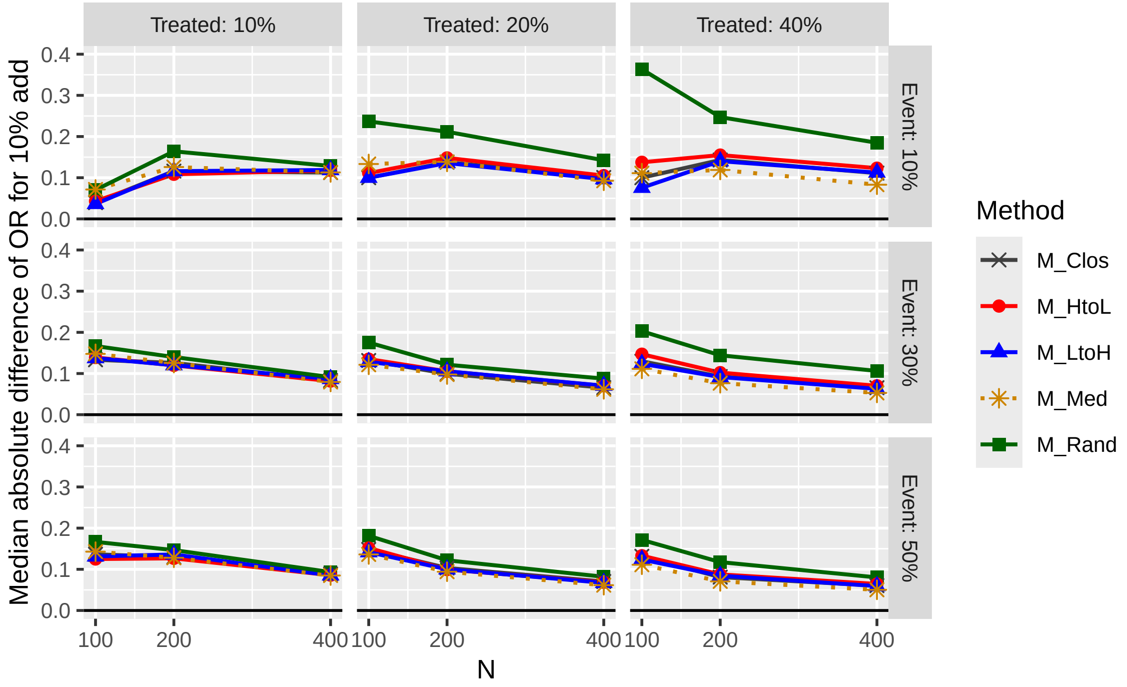

Figure S603. Median absolute difference of OR for 10% data addition (unimodal continuous covariate, matching ratio 1:2, true OR: 0.75, c statistic: 0.85).

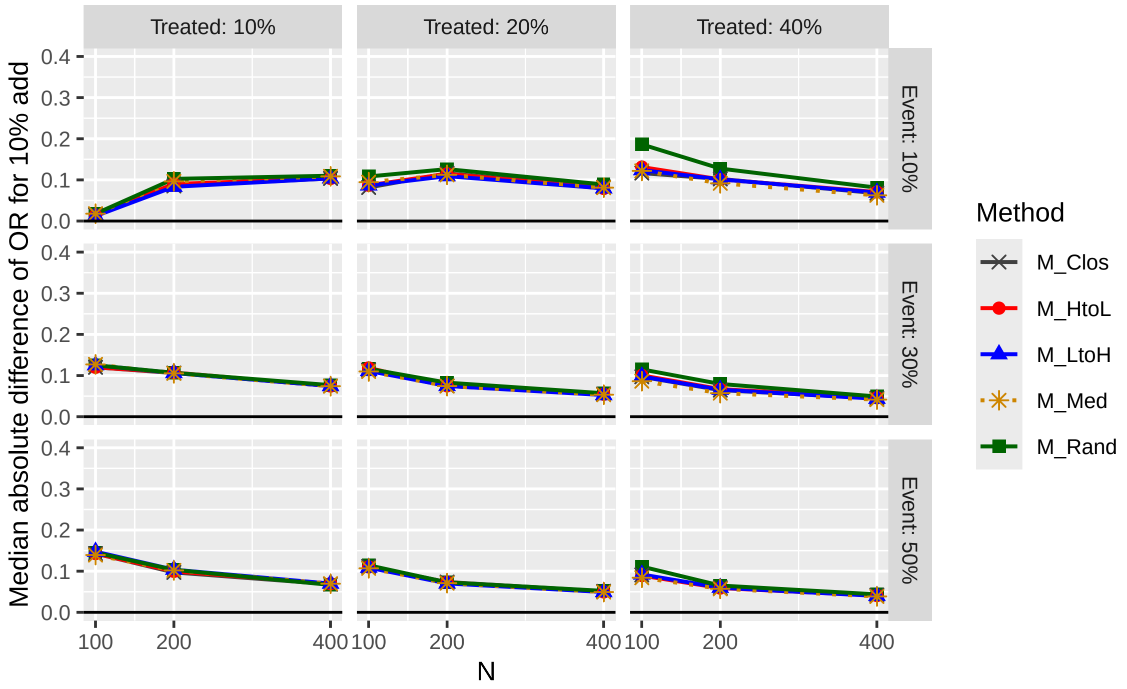

Figure S604. Median absolute difference of OR for 10% data addition (unimodal continuous covariate, matching ratio 1:2, true OR: 0.75, c statistic: 0.6).

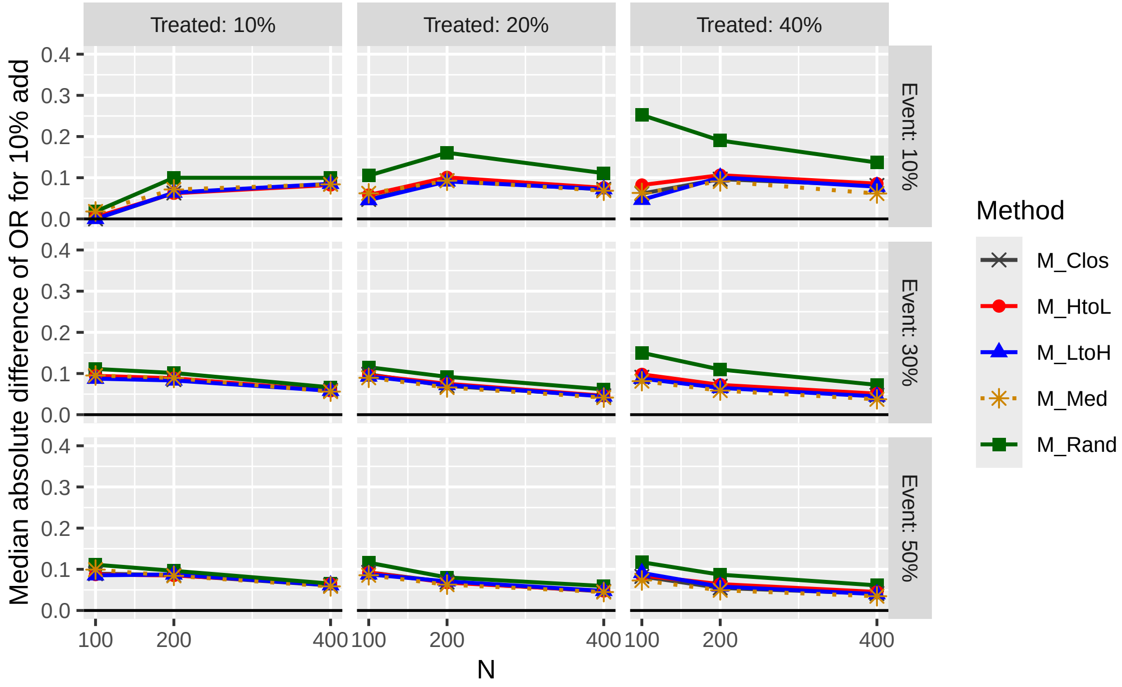

Figure S605. Median absolute difference of OR for 10% data addition (unimodal continuous covariate, matching ratio 1:2, true OR: 0.5, c statistic: 0.85).

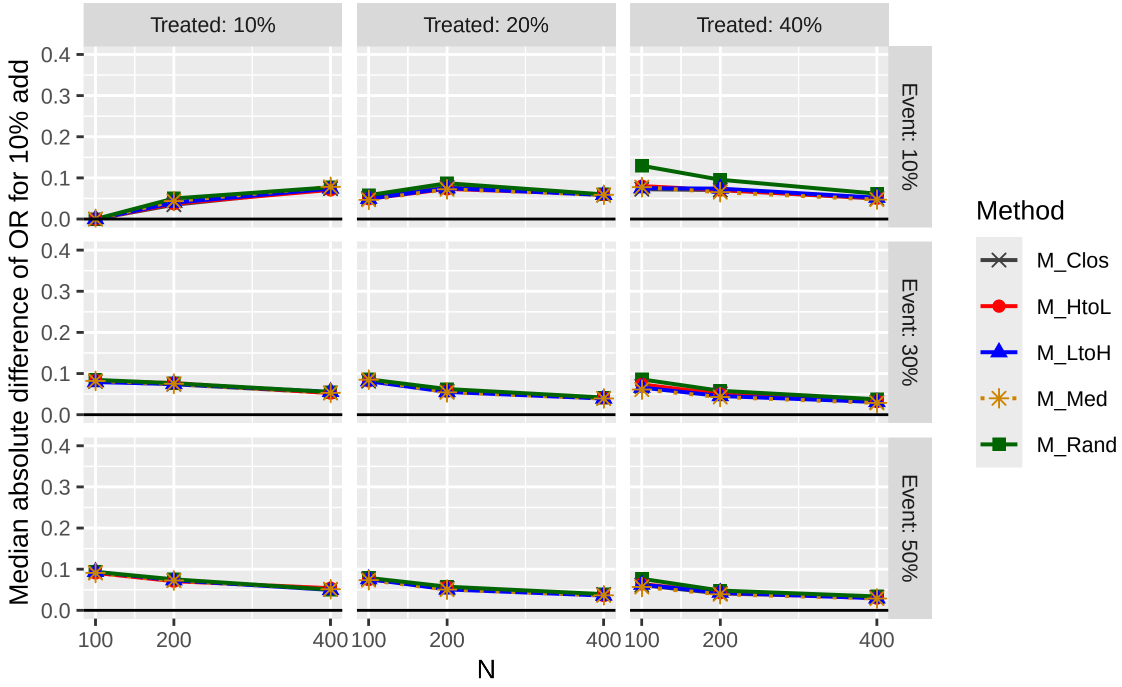

Figure S606. Median absolute difference of OR for 10% data addition (unimodal continuous covariate, matching ratio 1:2, true OR: 0.5, c statistic: 0.6).

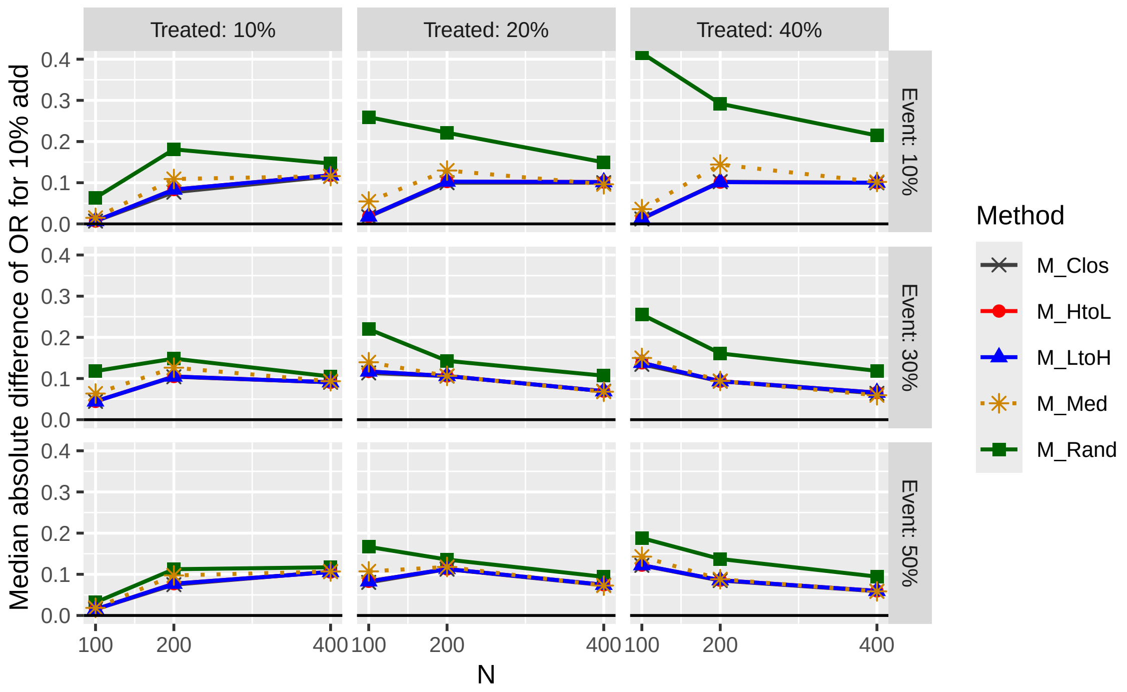

Figure S607. Median absolute difference of OR for 10% data addition (categorical covariate, matching ratio 1:1, true OR: 1, c statistic: 0.85).

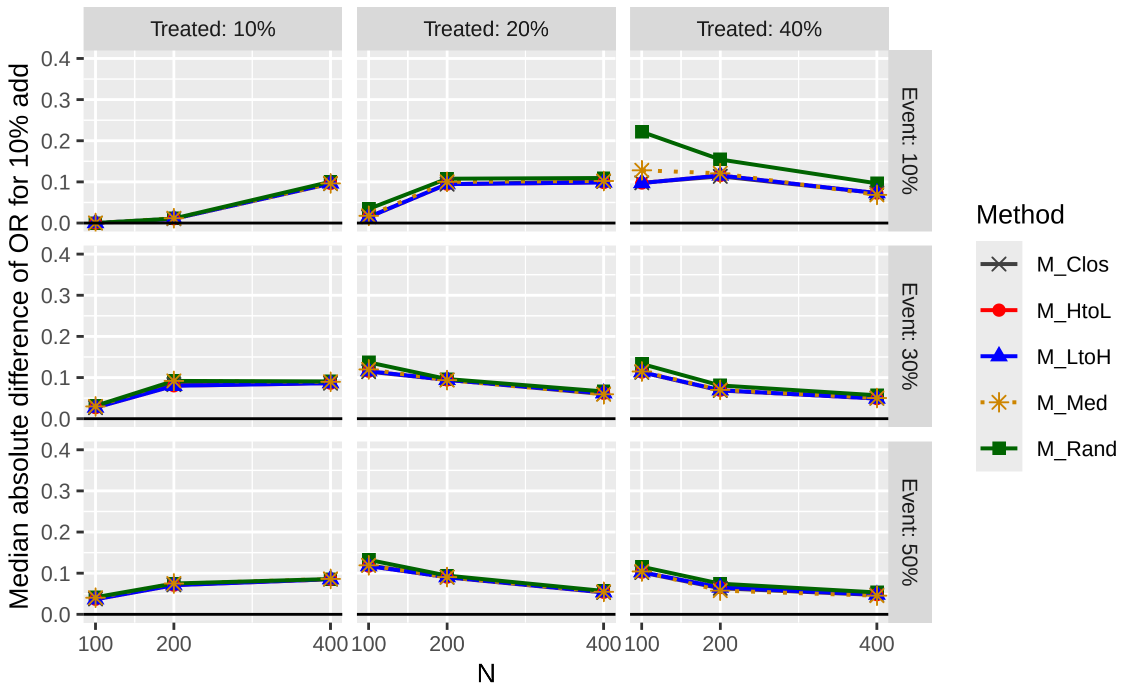

Figure S608. Median absolute difference of OR for 10% data addition (categorical covariate, matching ratio 1:1, true OR: 1, c statistic: 0.6).

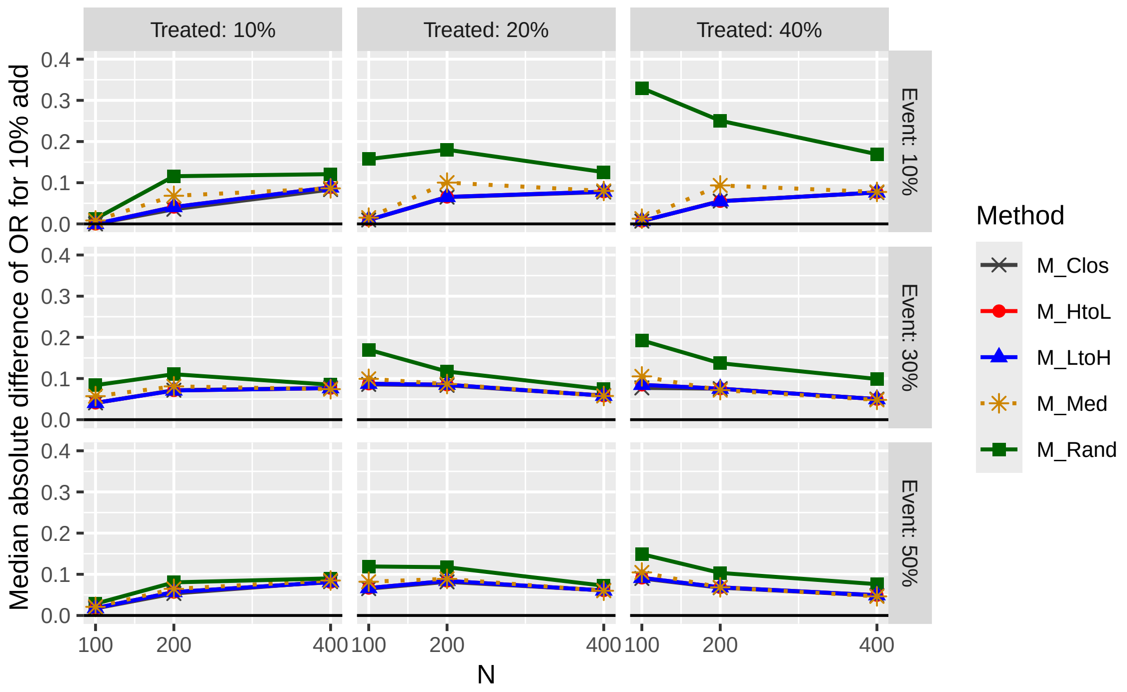

Figure S609. Median absolute difference of OR for 10% data addition (categorical covariate, matching ratio 1:1, true OR: 0.75, c statistic: 0.85).

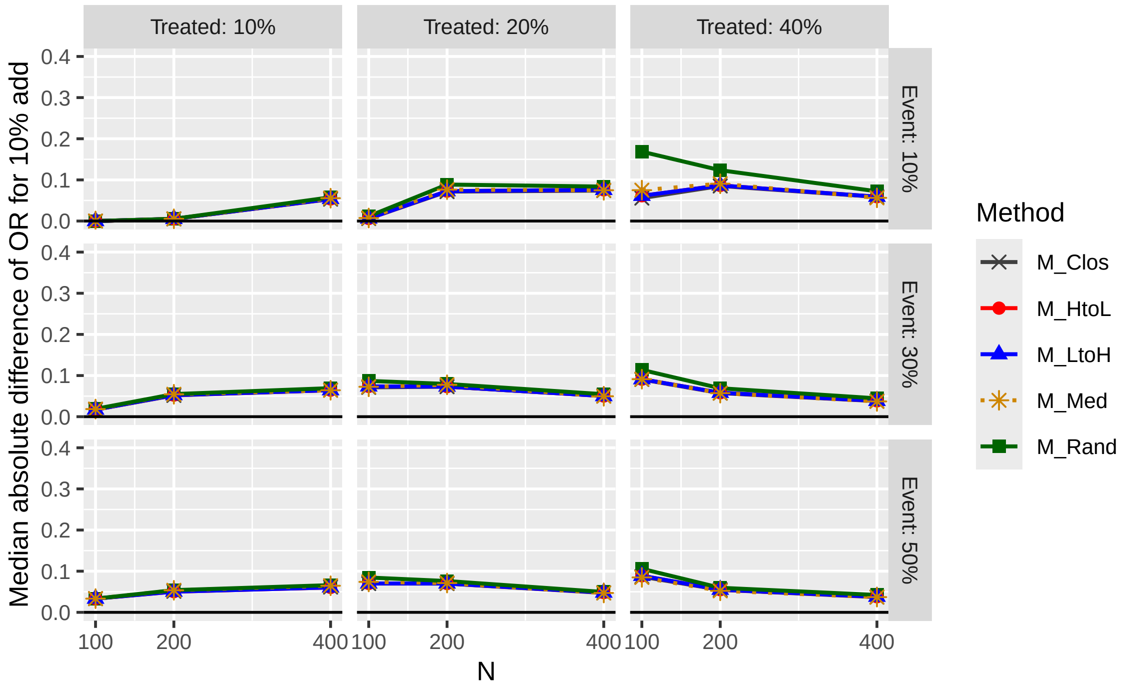

Figure S610. Median absolute difference of OR for 10% data addition (categorical covariate, matching ratio 1:1, true OR: 0.75, c statistic: 0.6).

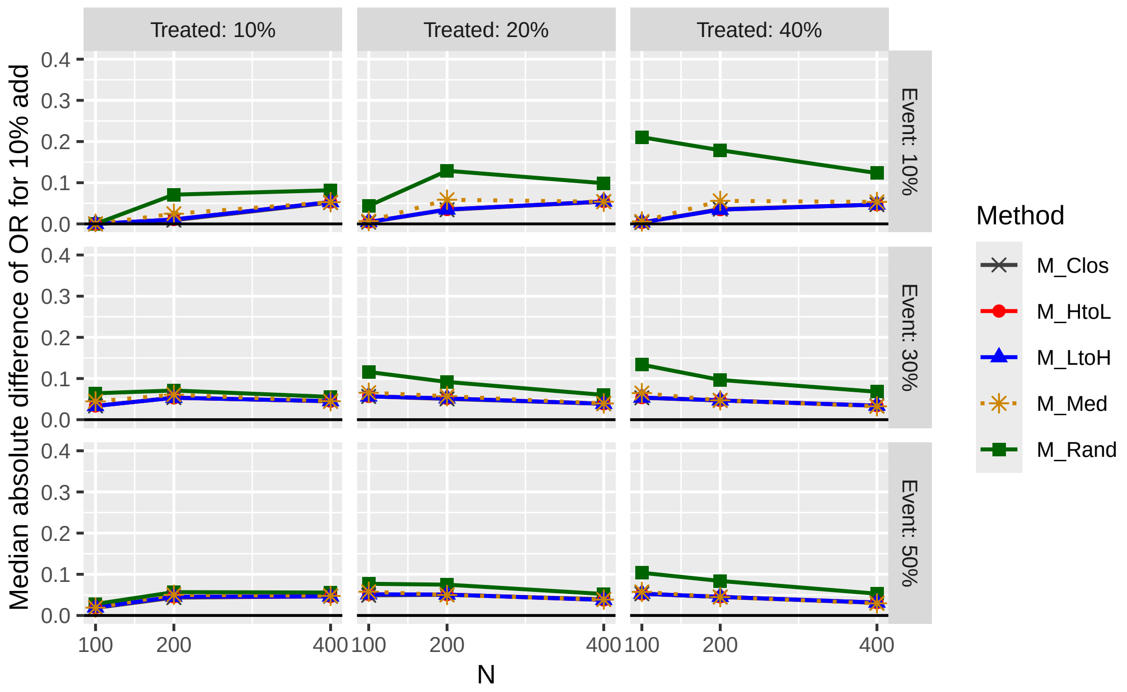

Figure S611. Median absolute difference of OR for 10% data addition (categorical covariate, matching ratio 1:1, true OR: 0.5, c statistic: 0.85).

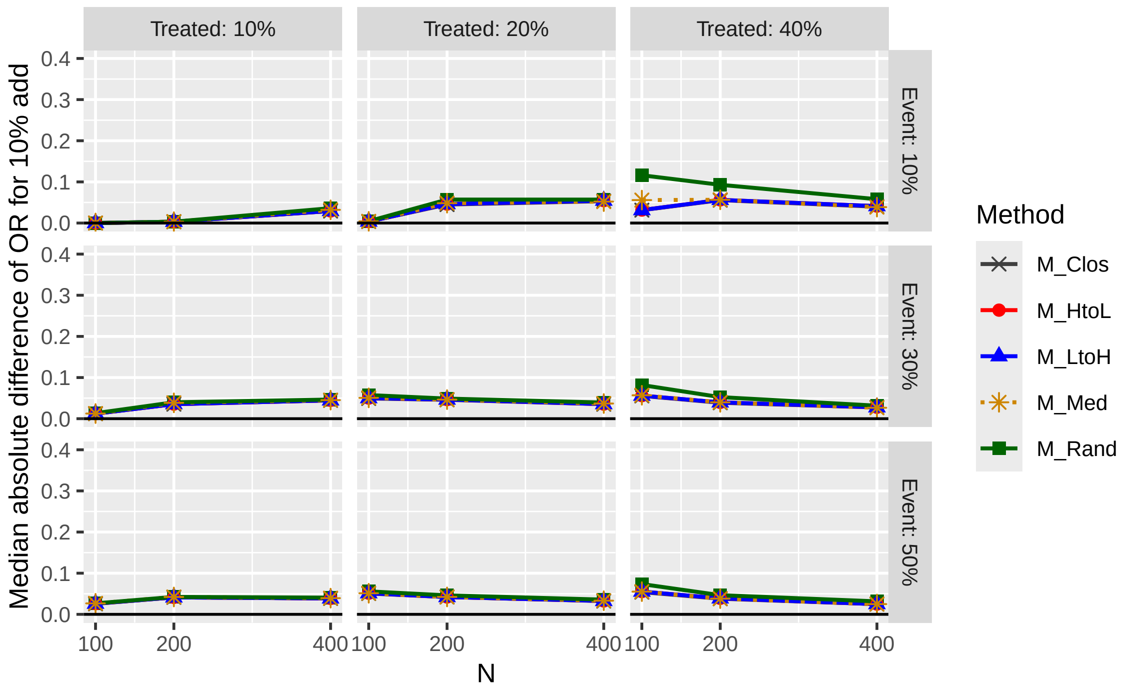

Figure S612. Median absolute difference of OR for 10% data addition (categorical covariate, matching ratio 1:1, true OR: 0.5, c statistic: 0.6).

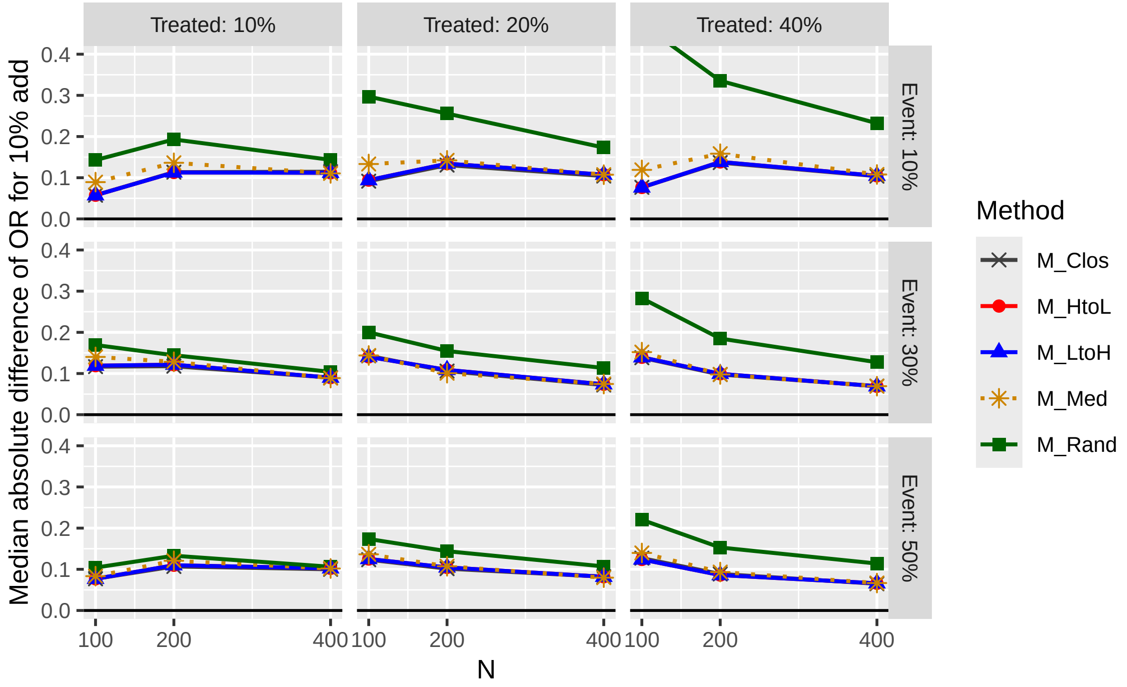

Figure S613. Median absolute difference of OR for 10% data addition (categorical covariate, matching ratio 1:2, true OR: 1, c statistic: 0.85).

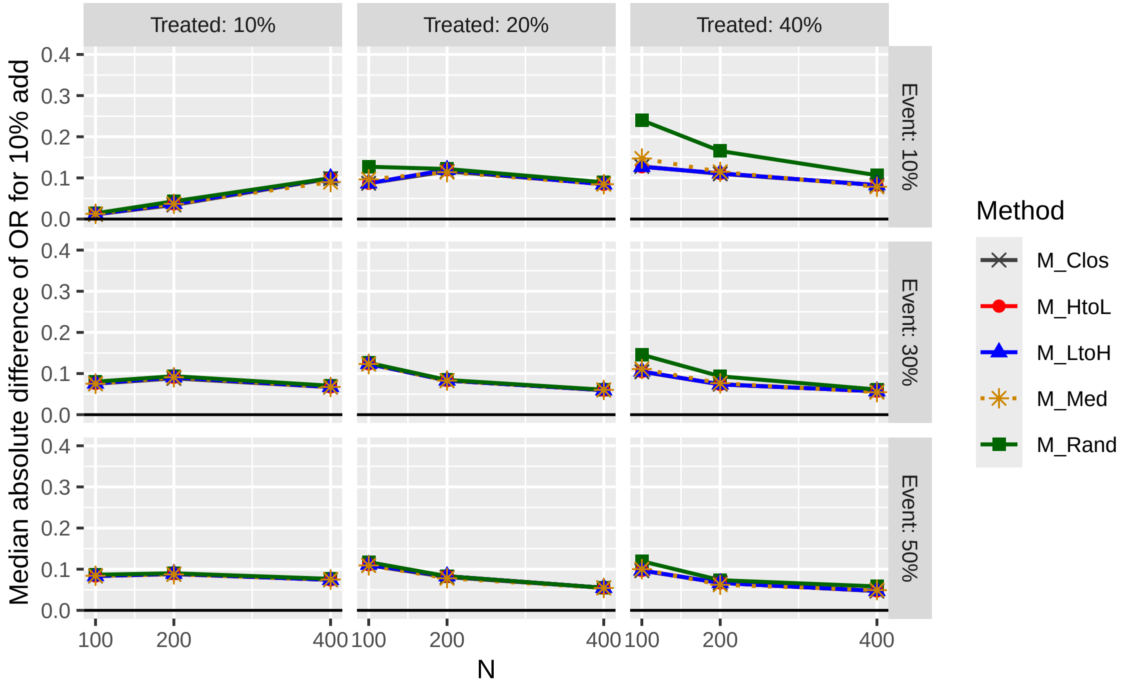

Figure S614. Median absolute difference of OR for 10% data addition (categorical covariate, matching ratio 1:2, true OR: 1, c statistic: 0.6).

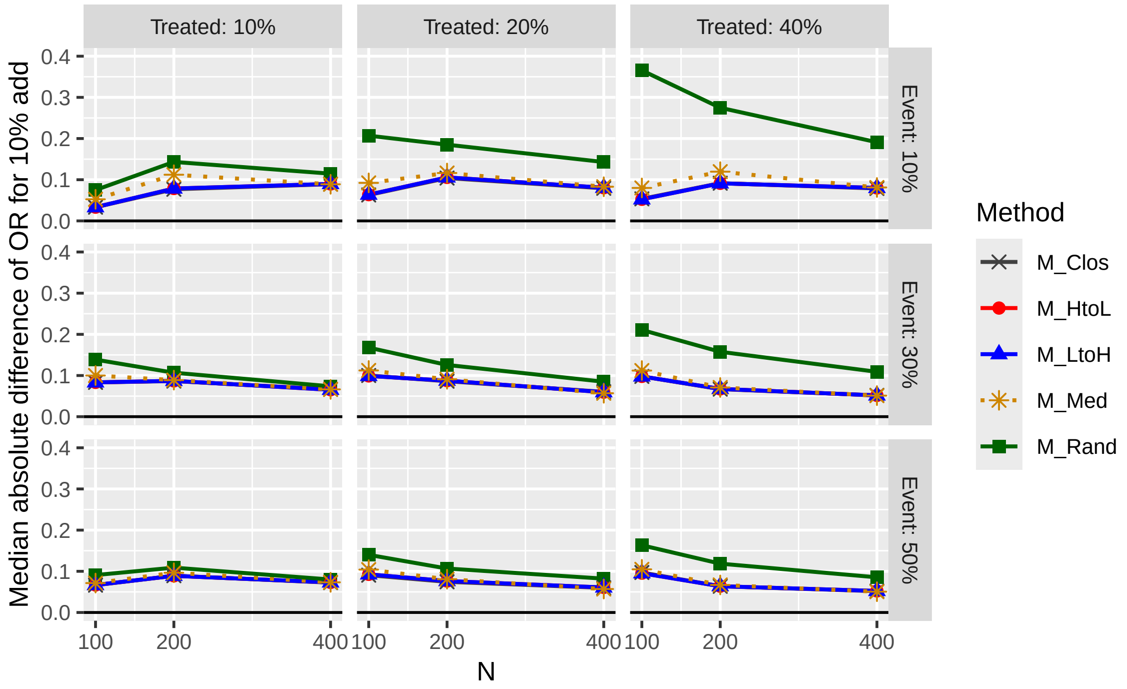

Figure S615. Median absolute difference of OR for 10% data addition (categorical covariate, matching ratio 1:2, true OR: 0.75, c statistic: 0.85).

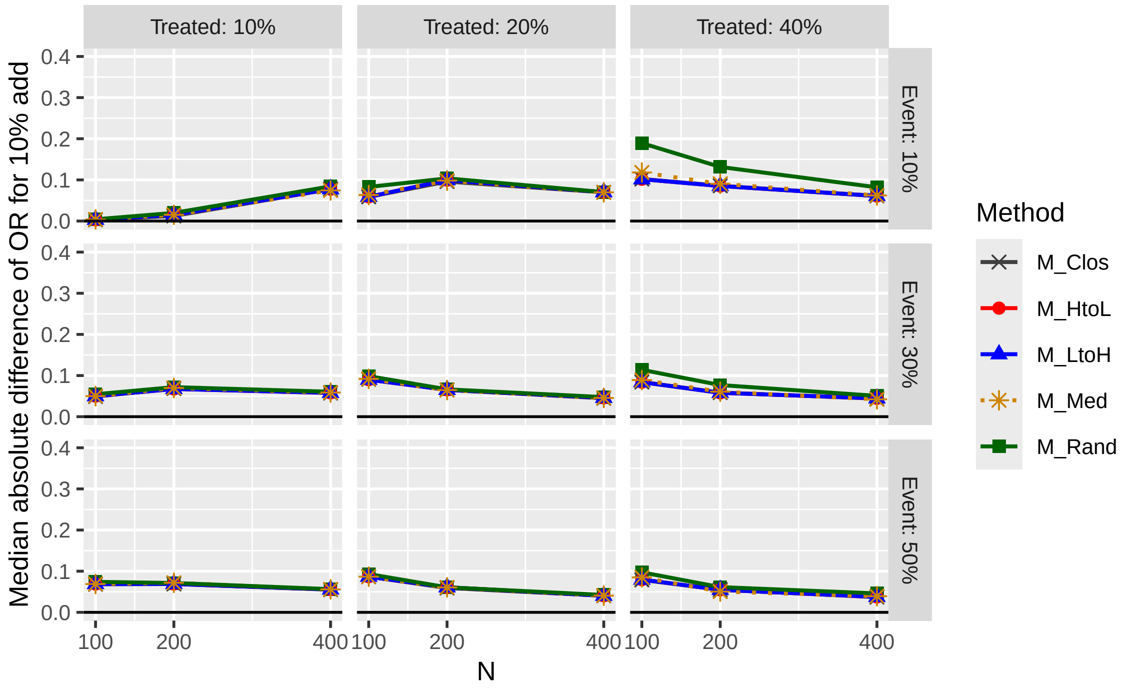

Figure S616. Median absolute difference of OR for 10% data addition (categorical covariate, matching ratio 1:2, true OR: 0.75, c statistic: 0.6).

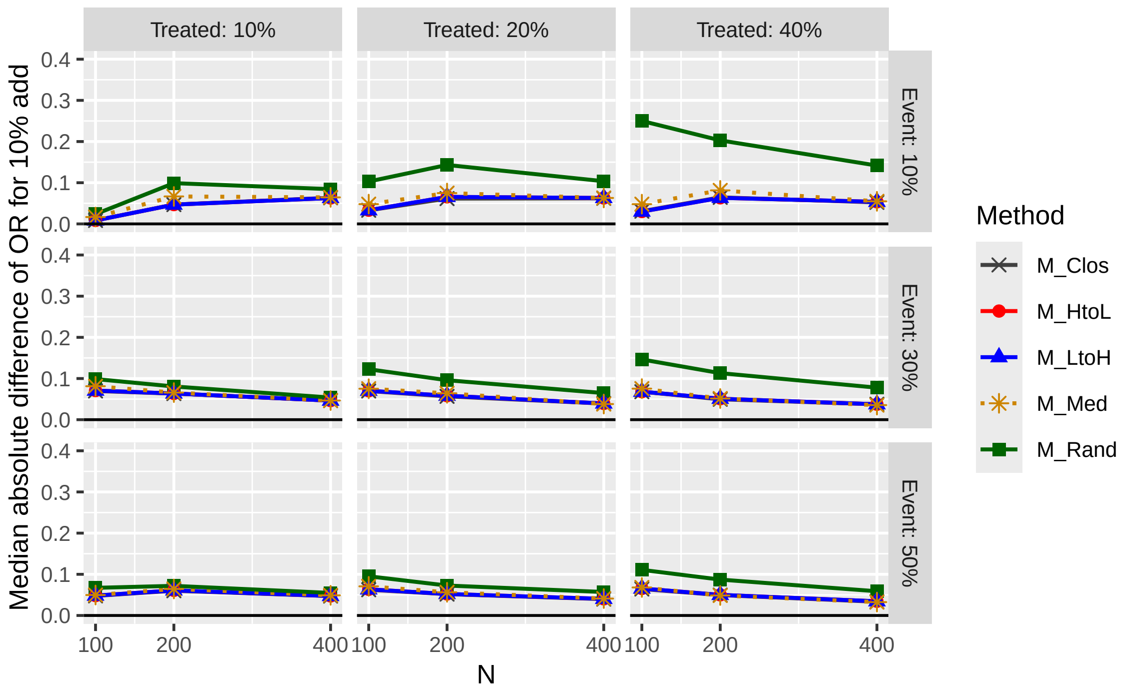

Figure S617. Median absolute difference of OR for 10% data addition (categorical covariate, matching ratio 1:2, true OR: 0.5, c statistic: 0.85).

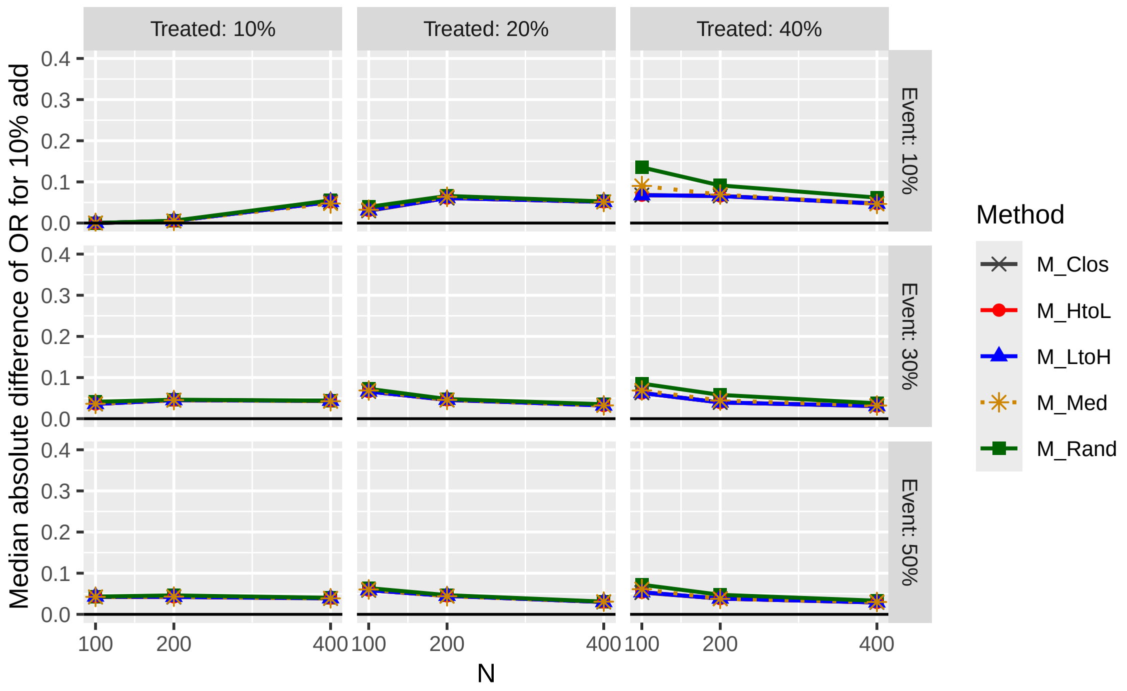

Figure S618. Median absolute difference of OR for 10% data addition (categorical covariate, matching ratio 1:2, true OR: 0.5, c statistic: 0.6).

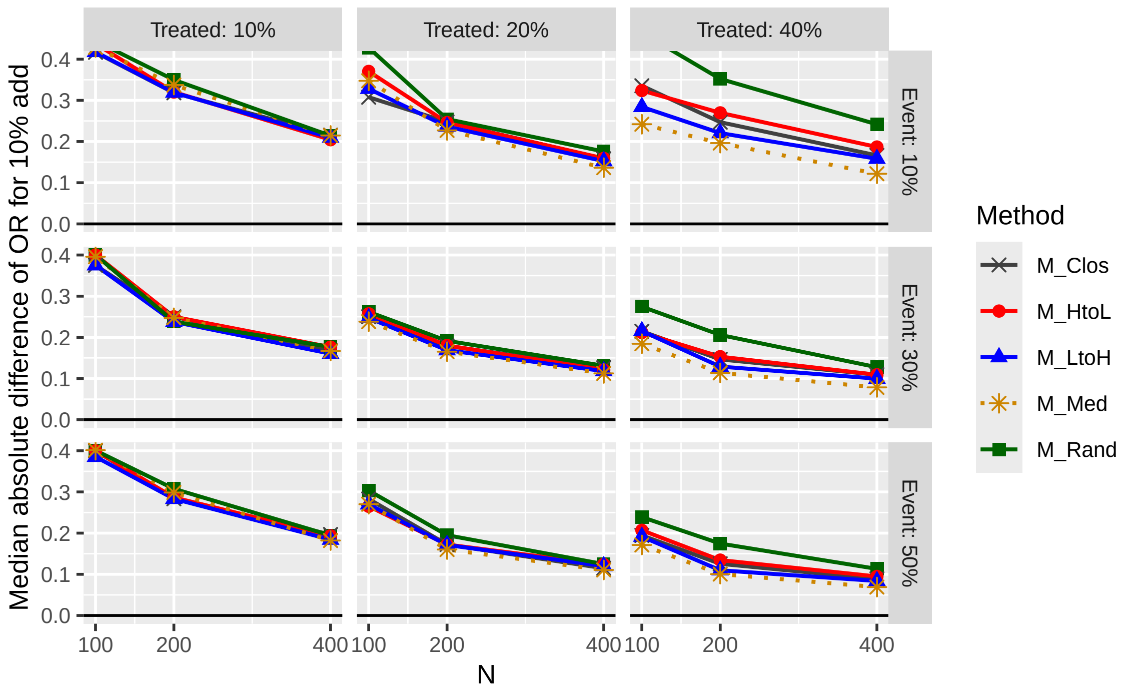

Figure S619. Median absolute difference of OR for 10% data addition (multimodal continuous covariate, matching ratio 1:1, true OR: 1, c statistic: 0.85).

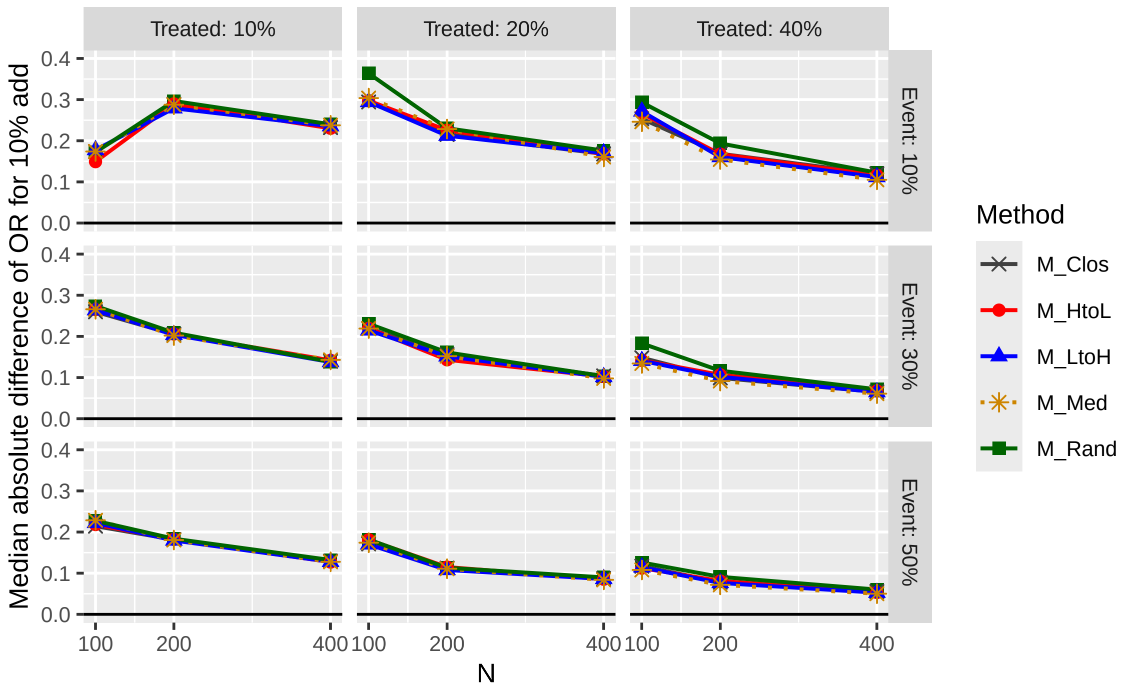

Figure S620. Median absolute difference of OR for 10% data addition (multimodal continuous covariate, matching ratio 1:1, true OR: 1, c statistic: 0.6).

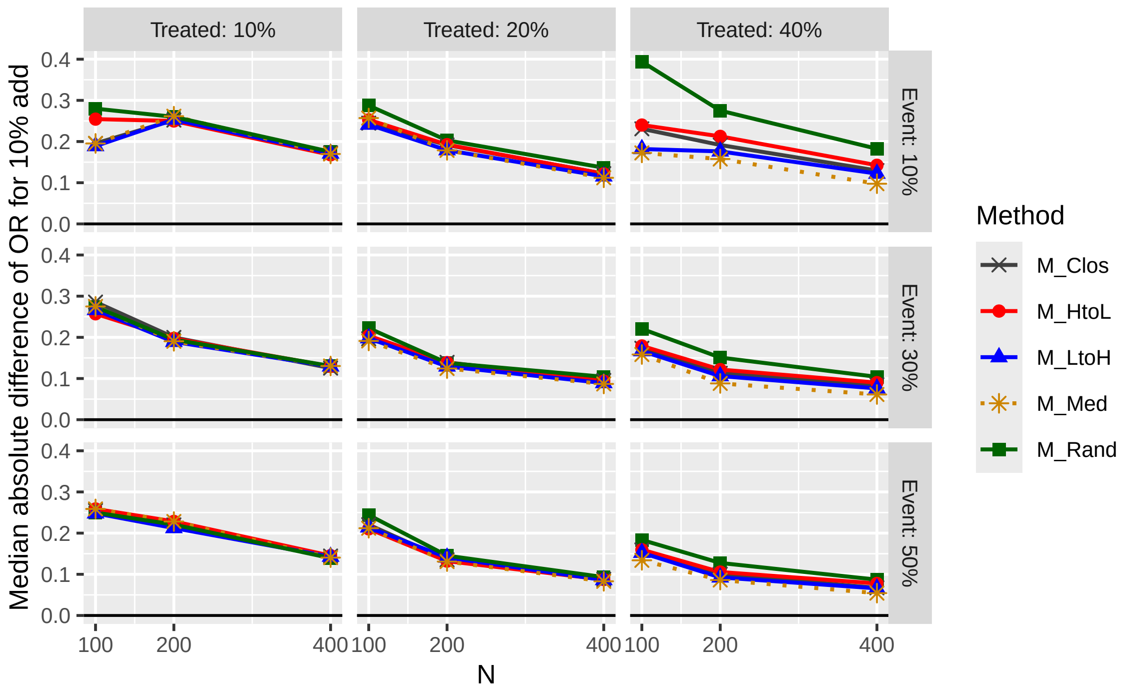

Figure S621. Median absolute difference of OR for 10% data addition (multimodal continuous covariate, matching ratio 1:1, true OR: 0.75, c statistic: 0.85).

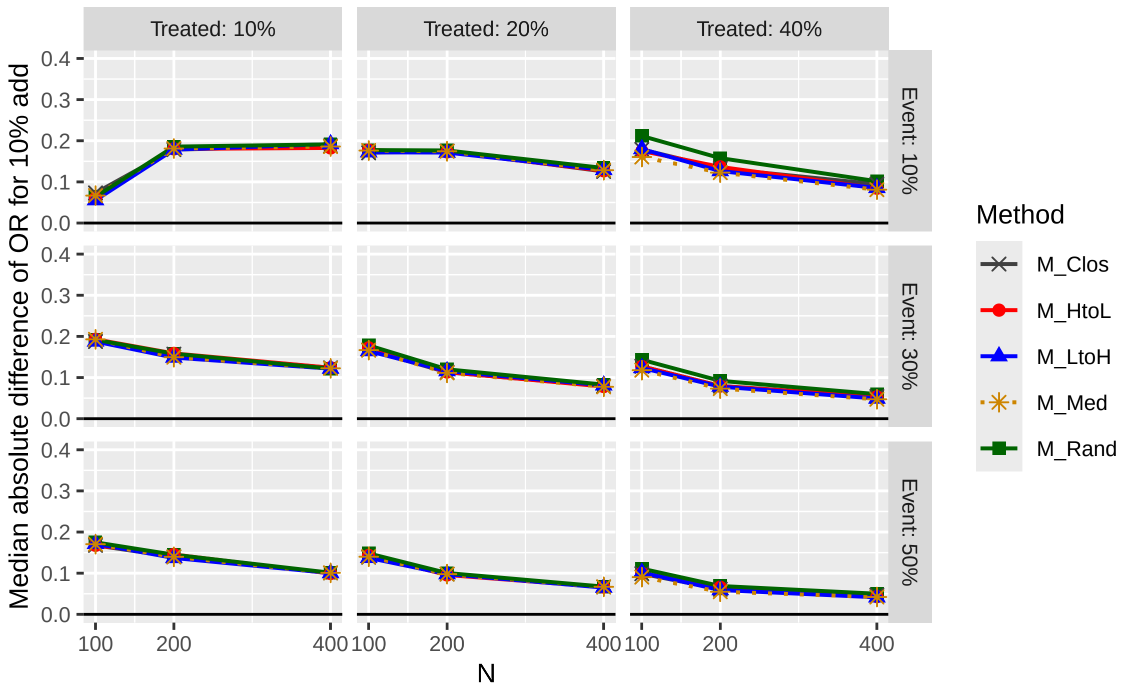

Figure S622. Median absolute difference of OR for 10% data addition (multimodal continuous covariate, matching ratio 1:1, true OR: 0.75, c statistic: 0.6).

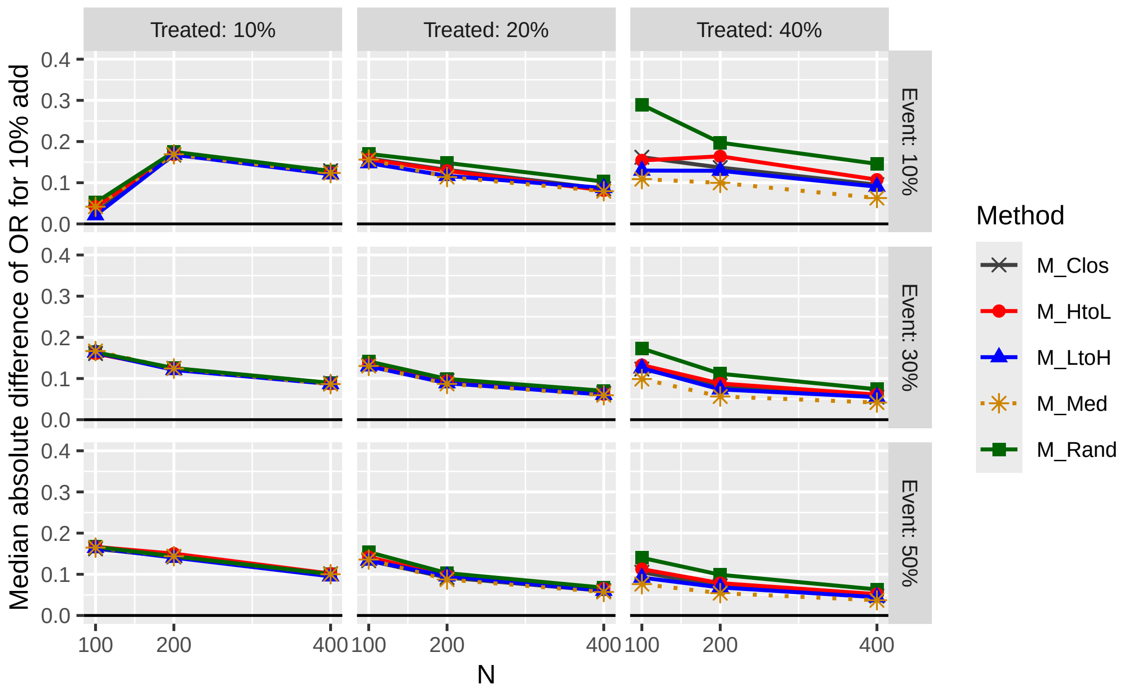

Figure S623. Median absolute difference of OR for 10% data addition (multimodal continuous covariate, matching ratio 1:1, true OR: 0.5, c statistic: 0.85).

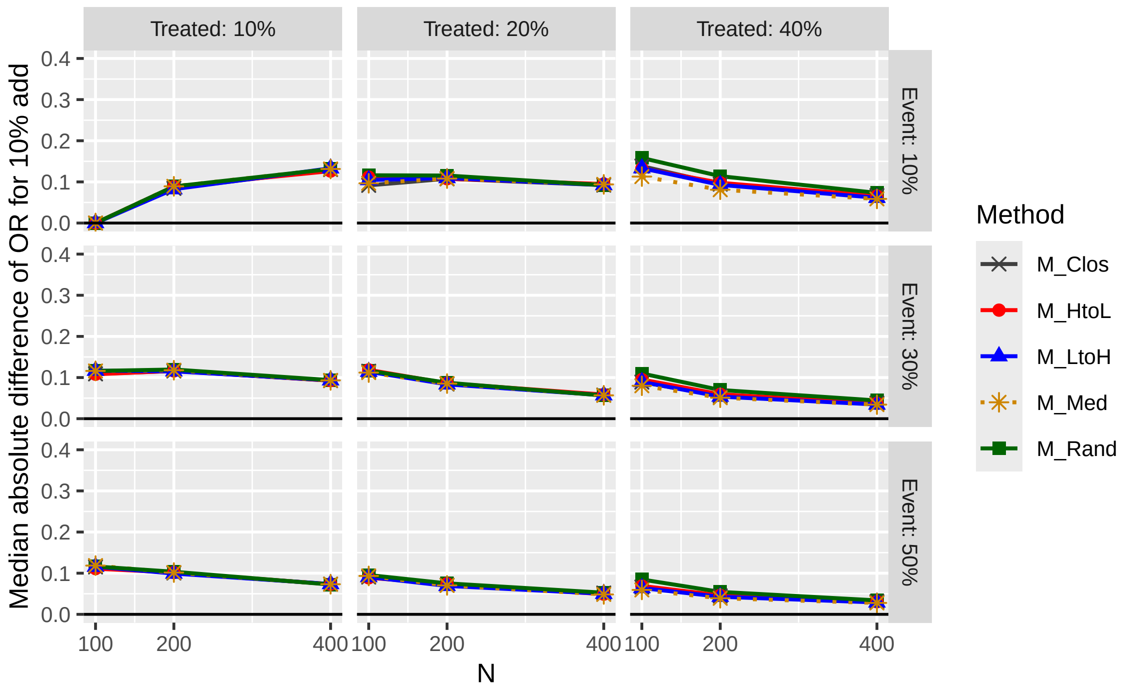

Figure S624. Median absolute difference of OR for 10% data addition (multimodal continuous covariate, matching ratio 1:1, true OR: 0.5, c statistic: 0.6).

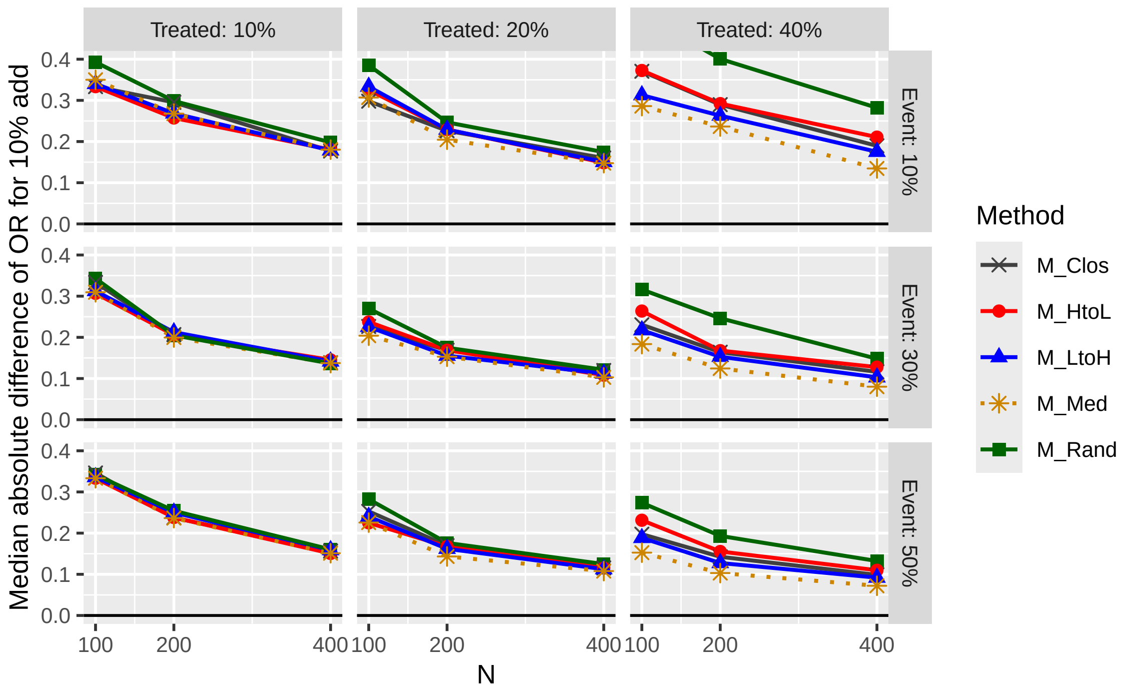

Figure S625. Median absolute difference of OR for 10% data addition (multimodal continuous covariate, matching ratio 1:2, true OR: 1, c statistic: 0.85).

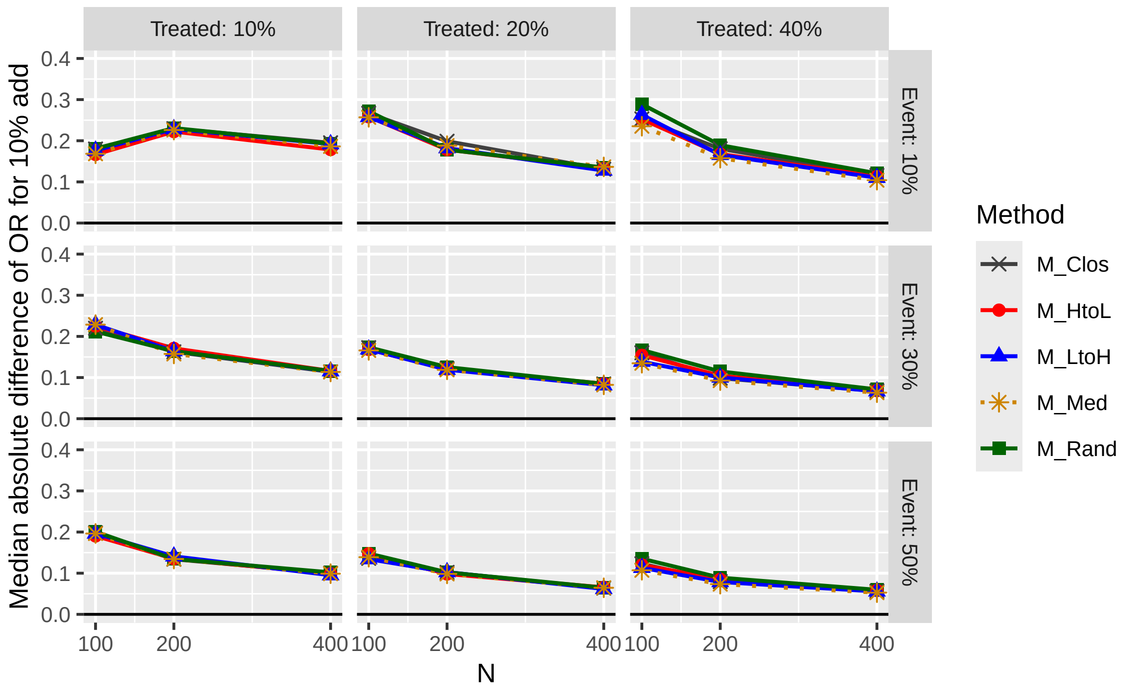

Figure S626. Median absolute difference of OR for 10% data addition (multimodal continuous covariate, matching ratio 1:2, true OR: 1, c statistic: 0.6).

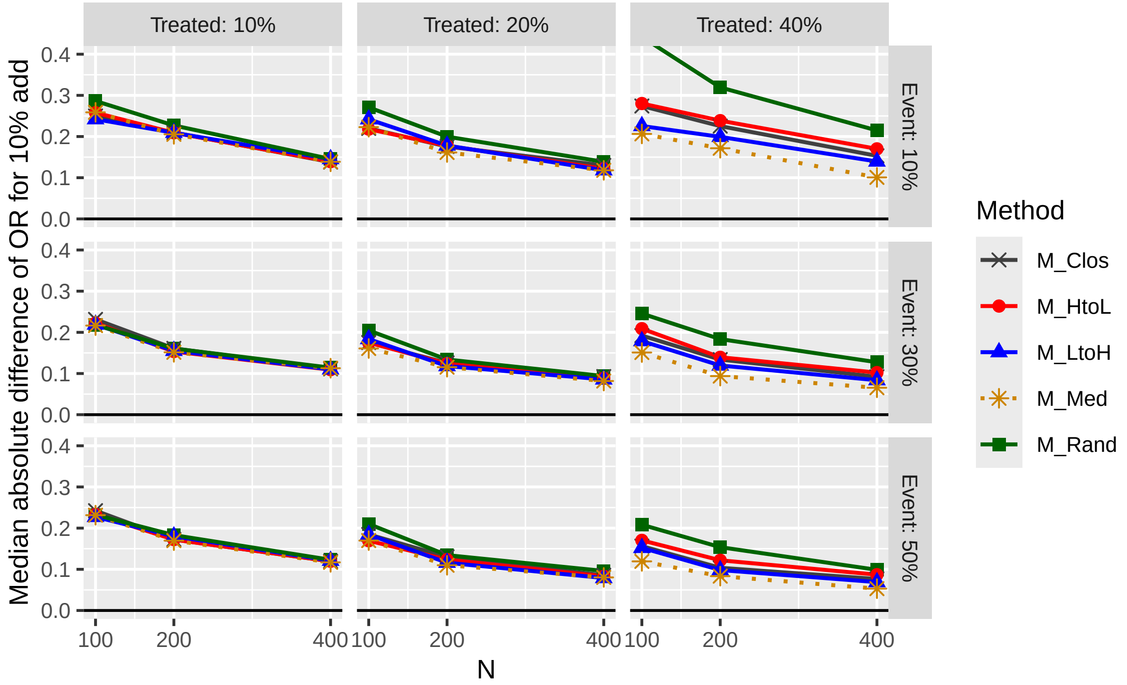

Figure S627. Median absolute difference of OR for 10% data addition (multimodal continuous covariate, matching ratio 1:2, true OR: 0.75, c statistic: 0.85).

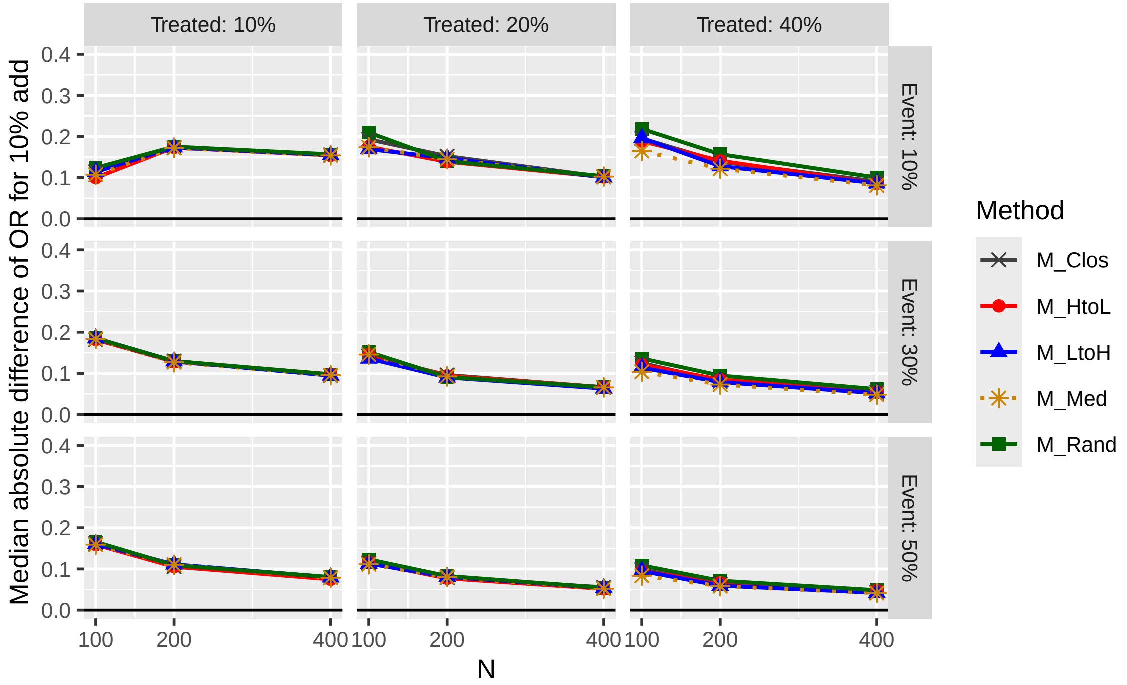

Figure S628. Median absolute difference of OR for 10% data addition (multimodal continuous covariate, matching ratio 1:2, true OR: 0.75, c statistic: 0.6).

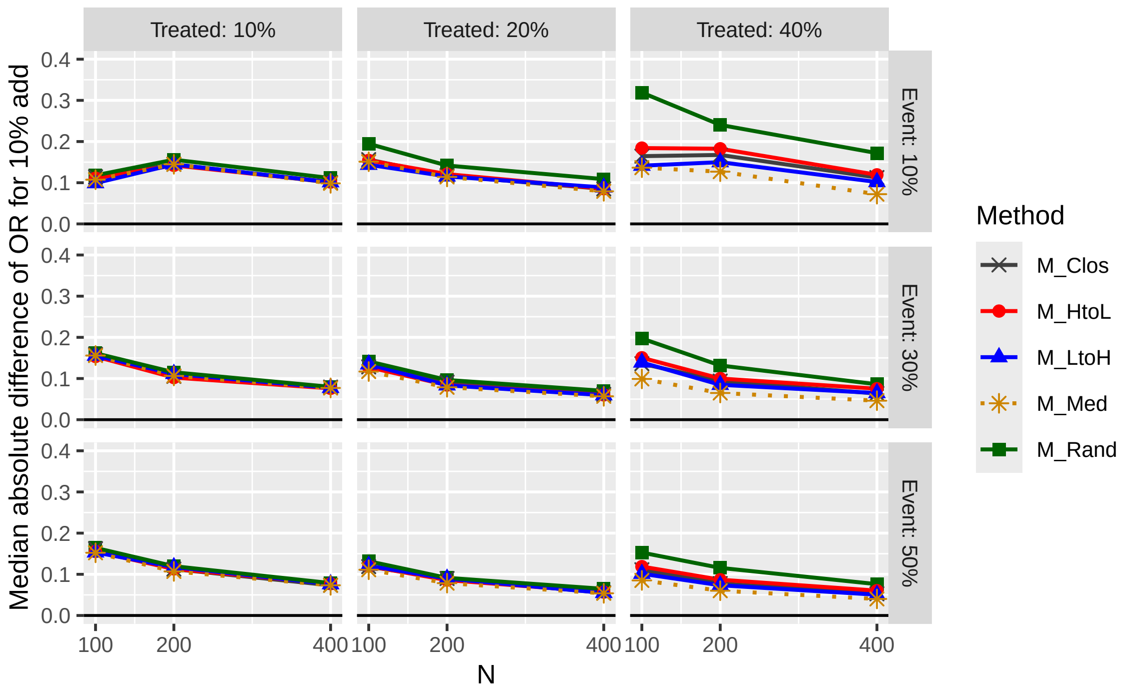

Figure S629. Median absolute difference of OR for 10% data addition (multimodal continuous covariate, matching ratio 1:2, true OR: 0.5, c statistic: 0.85).

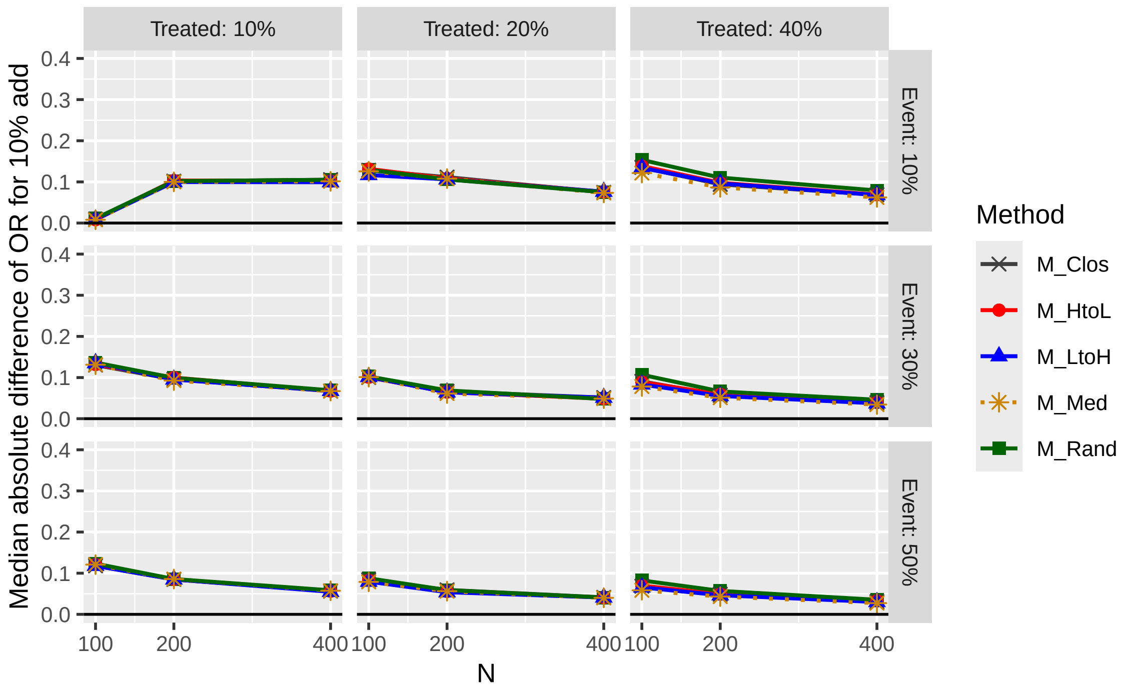

Figure S630. Median absolute difference of OR for 10% data addition (multimodal continuous covariate, matching ratio 1:2, true OR: 0.5, c statistic: 0.6).

S11. Coverage probability of confidence interval for OR (caliper: 15%)

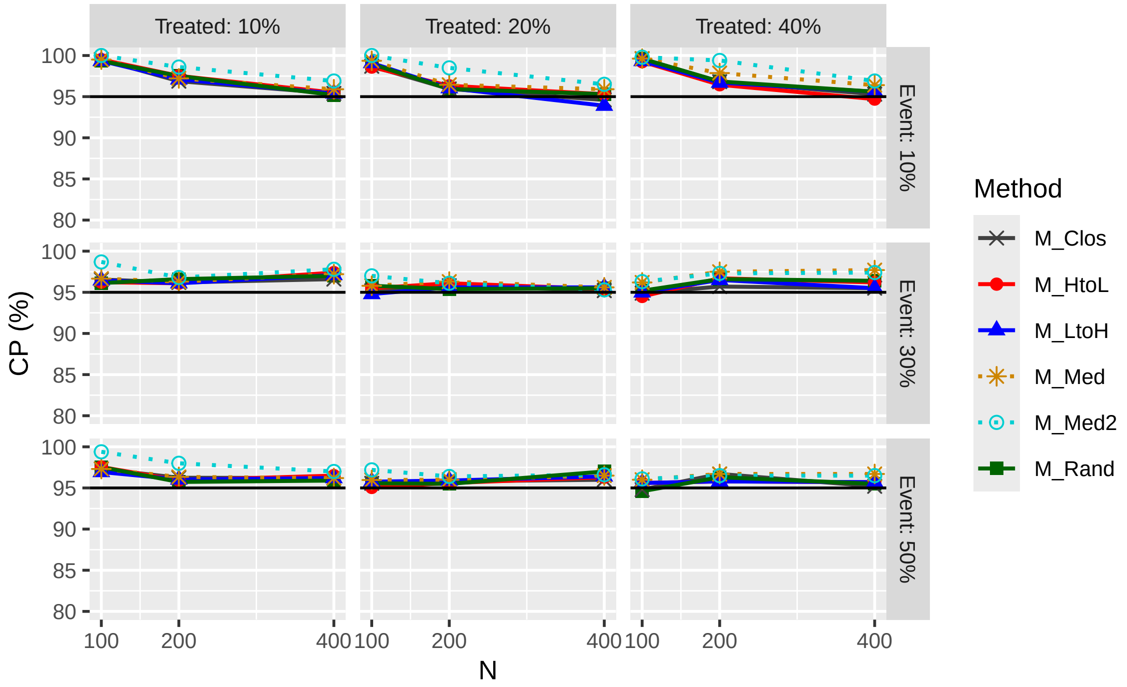

Figure S631. Coverage probability of confidence interval for OR (unimodal continuous covariate, matching ratio 1:1, true OR: 1, c statistic: 0.85, naive inference).

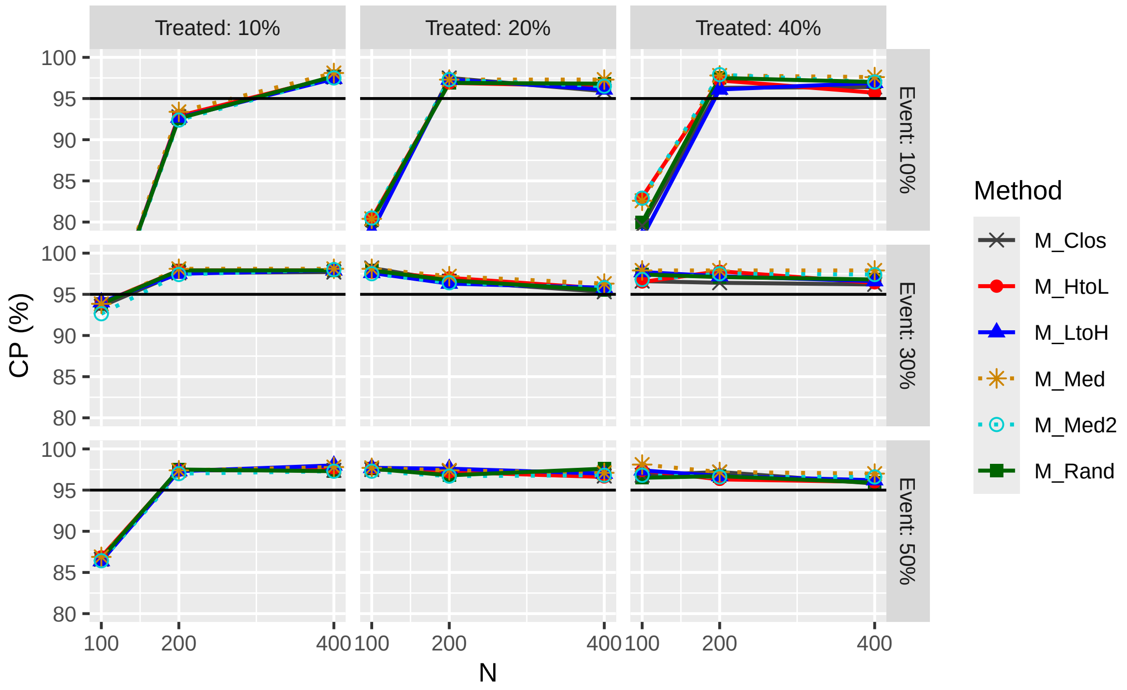

Figure S632. Coverage probability of confidence interval for OR (unimodal continuous covariate, matching ratio 1:1, true OR: 1, c statistic: 0.85, robust inference).

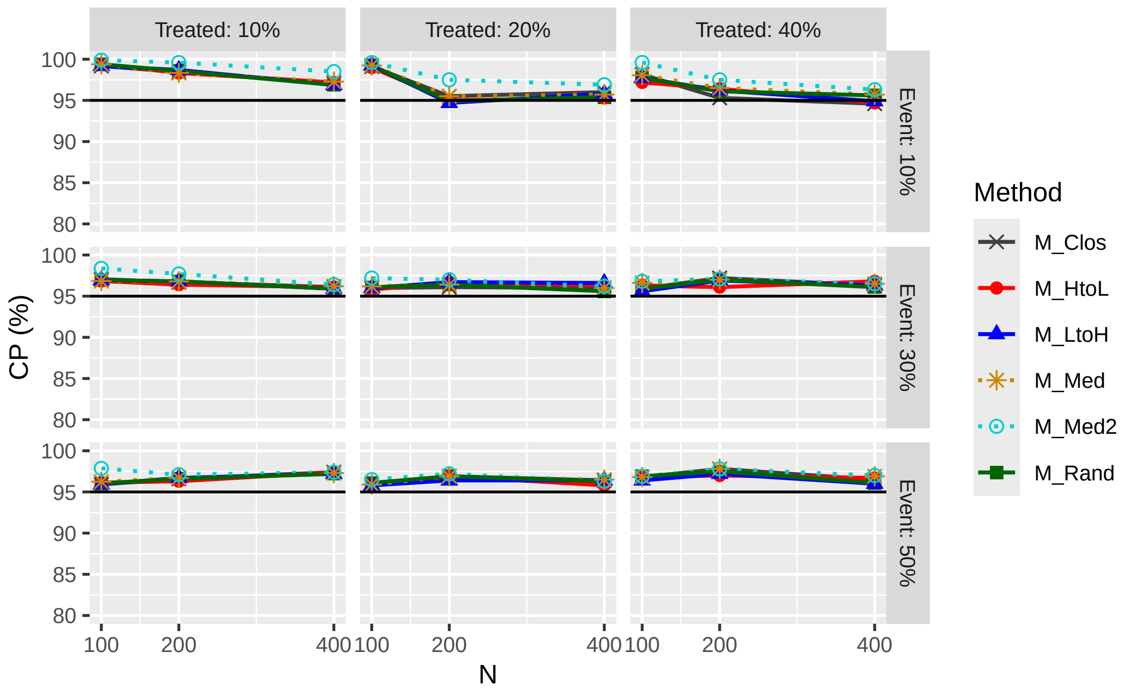

Figure S633. Coverage probability of confidence interval for OR (unimodal continuous covariate, matching ratio 1:1, true OR: 1, c statistic: 0.6, naive inference).

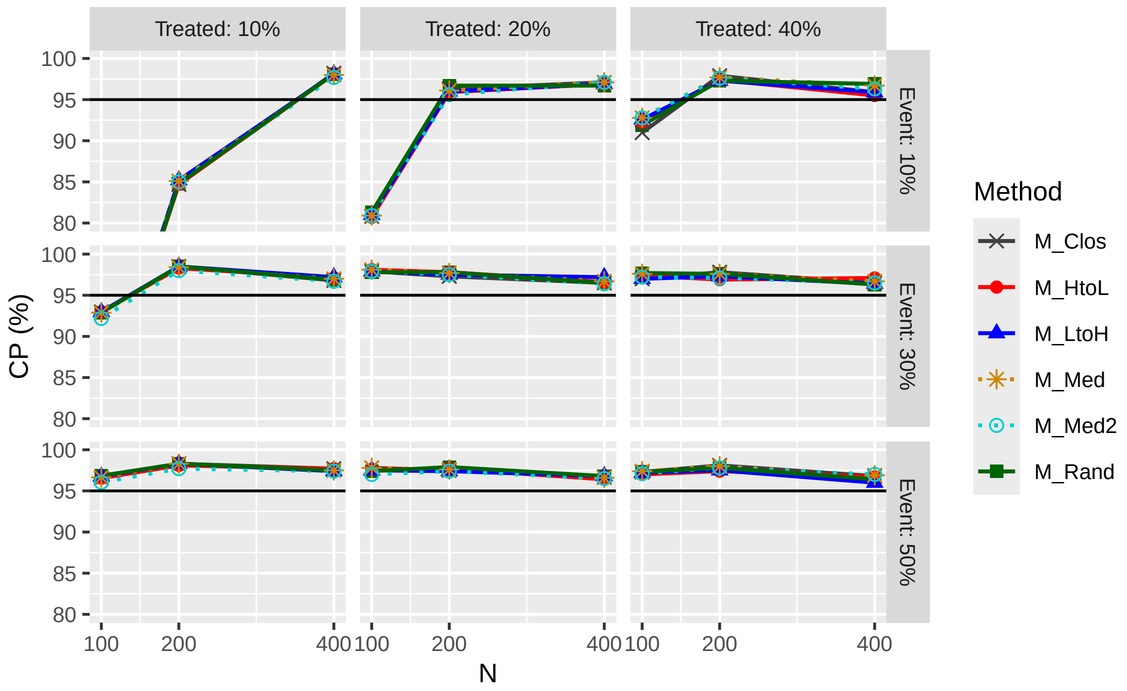

Figure S634. Coverage probability of confidence interval for OR (unimodal continuous covariate, matching ratio 1:1, true OR: 1, c statistic: 0.6, robust inference).

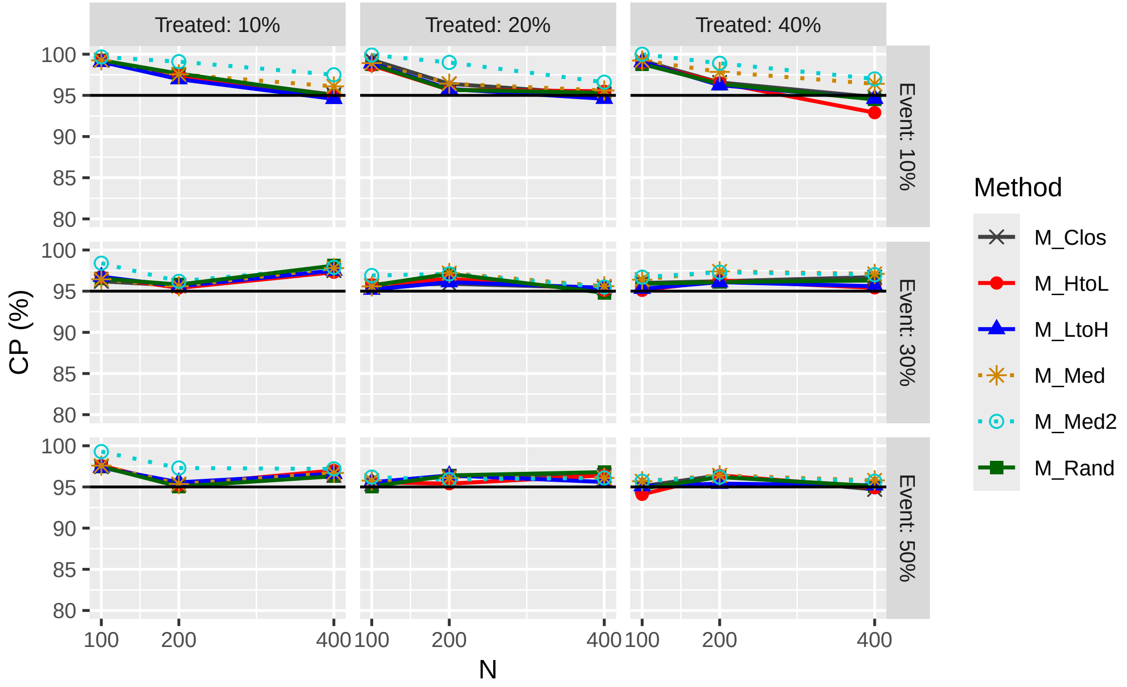

Figure S635. Coverage probability of confidence interval for OR (unimodal continuous covariate, matching ratio 1:1, true OR: 0.75, c statistic: 0.85, naive inference).

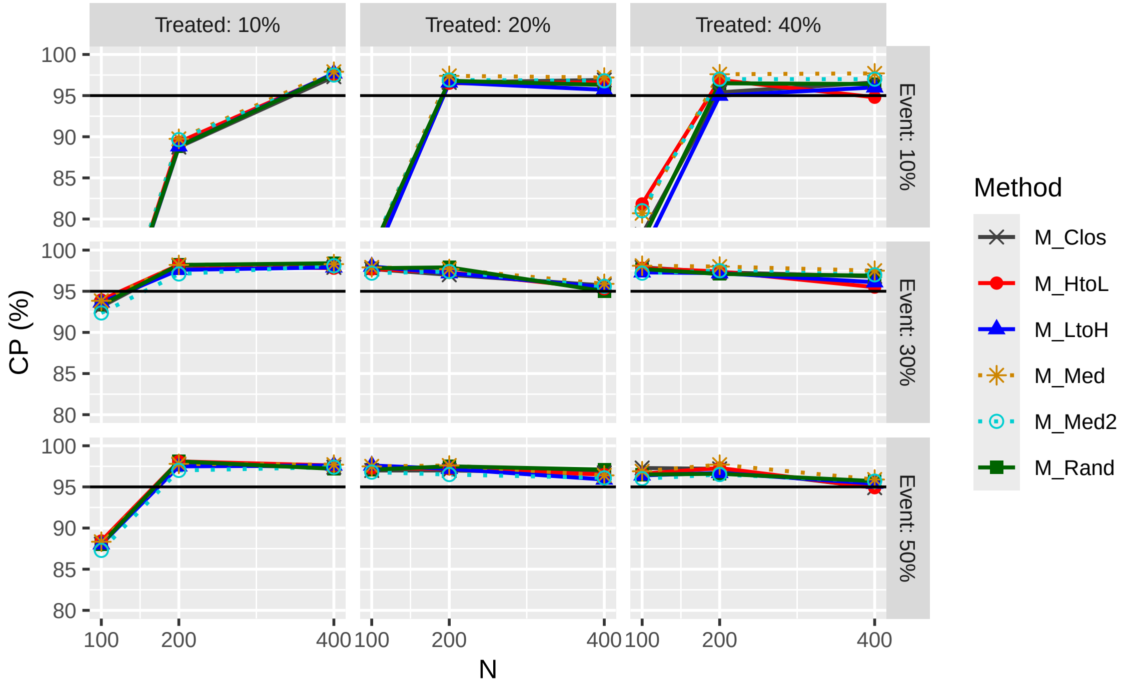

Figure S636. Coverage probability of confidence interval for OR (unimodal continuous covariate, matching ratio 1:1, true OR: 0.75, c statistic: 0.85, robust inference).

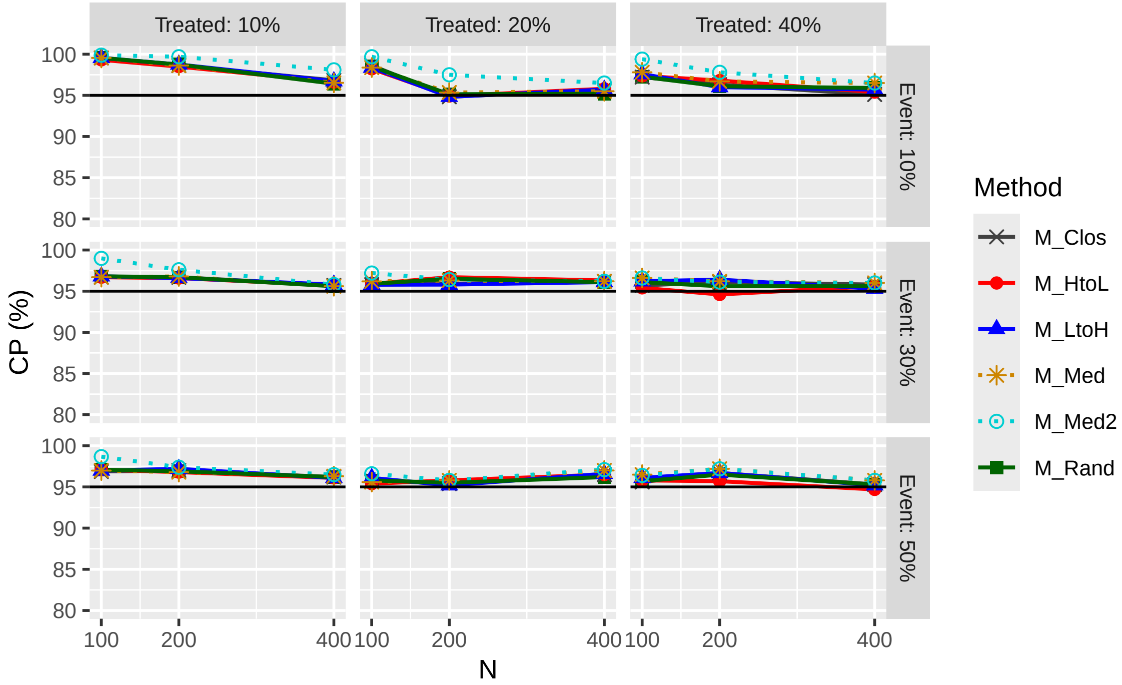

Figure S637. Coverage probability of confidence interval for OR (unimodal continuous covariate, matching ratio 1:1, true OR: 0.75, c statistic: 0.6, naive inference).

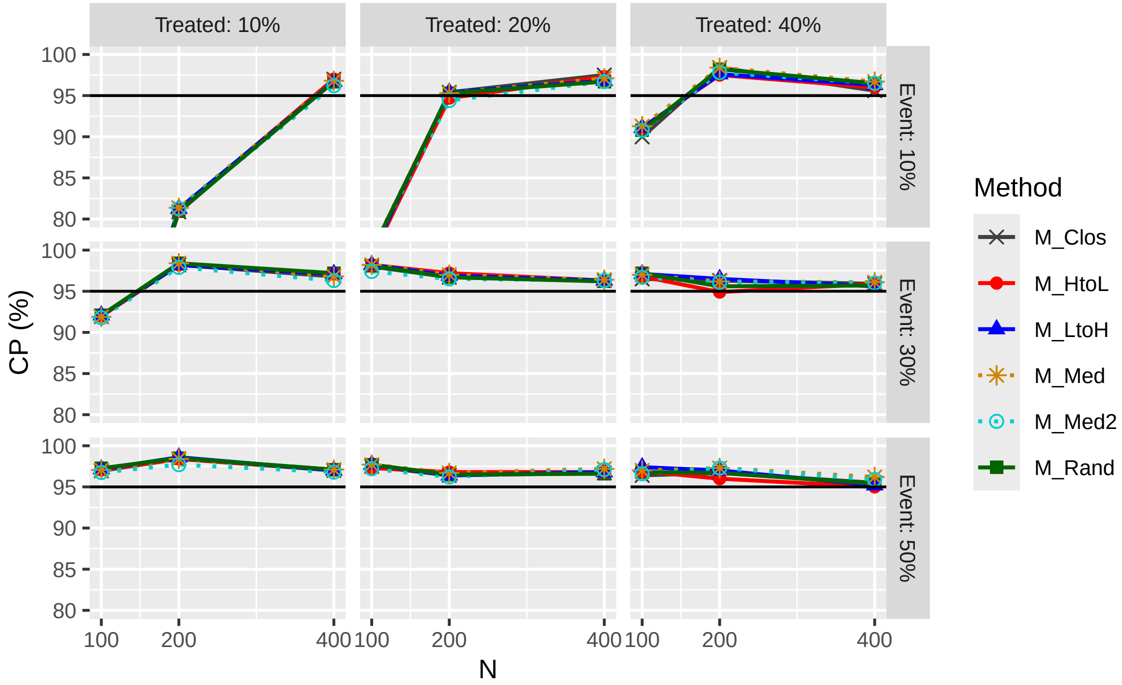

Figure S638. Coverage probability of confidence interval for OR (unimodal continuous covariate, matching ratio 1:1, true OR: 0.75, c statistic: 0.6, robust inference).

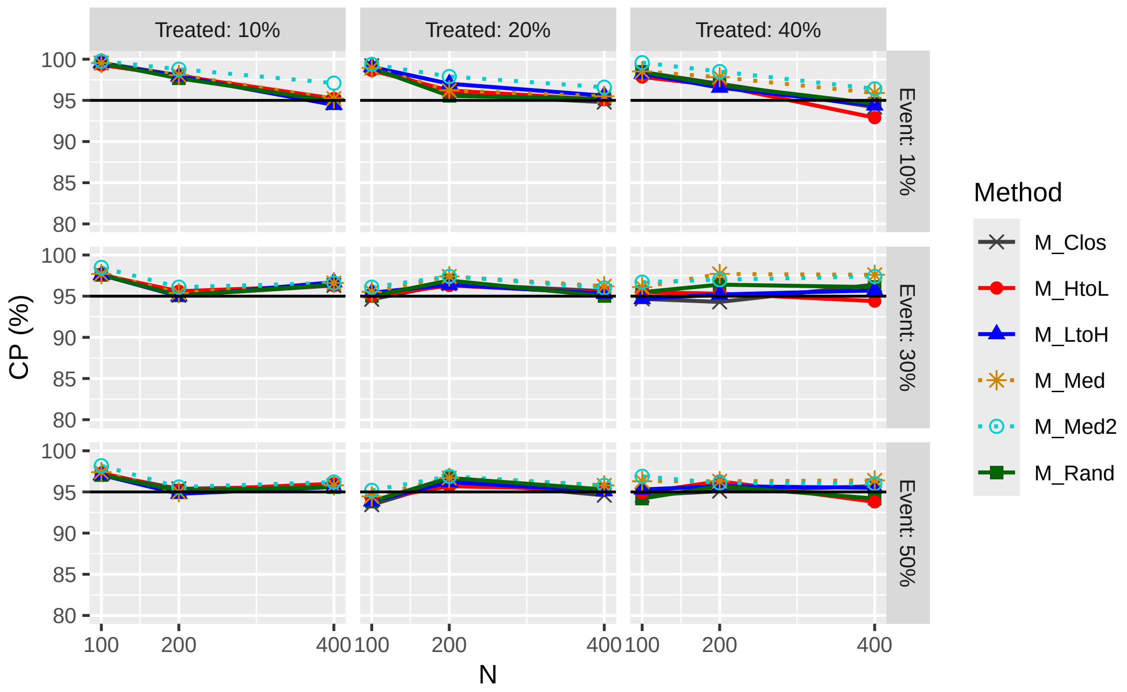

Figure S639. Coverage probability of confidence interval for OR (unimodal continuous covariate, matching ratio 1:1, true OR: 0.5, c statistic: 0.85, naive inference).

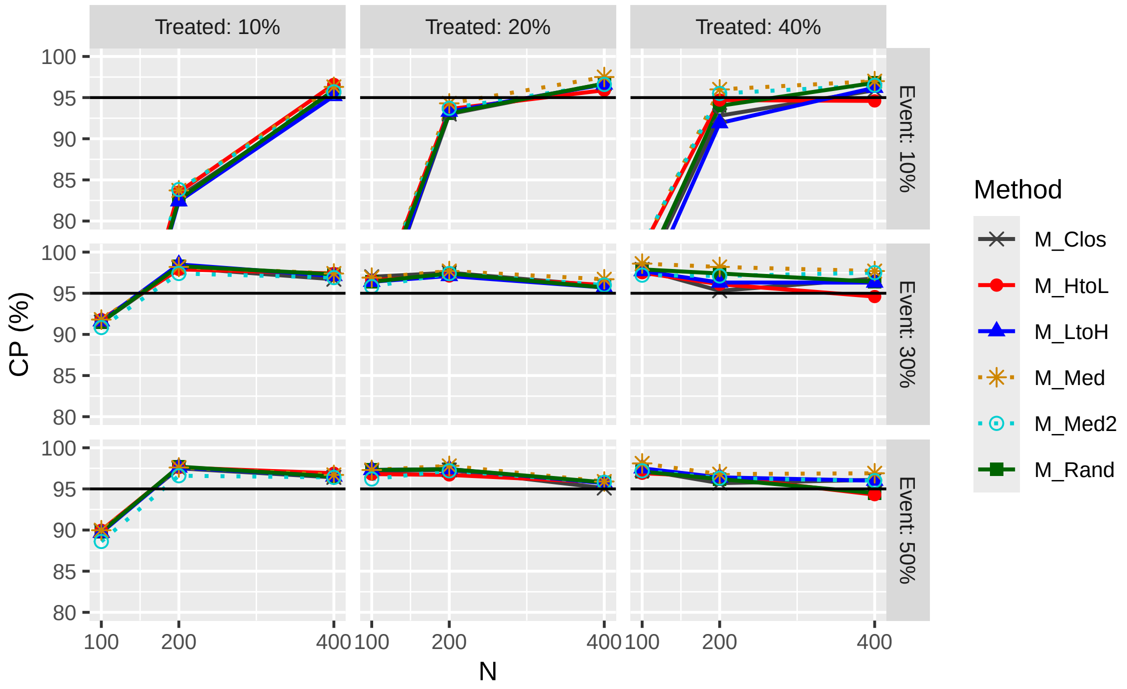

Figure S640. Coverage probability of confidence interval for OR (unimodal continuous covariate, matching ratio 1:1, true OR: 0.5, c statistic: 0.85, robust inference).

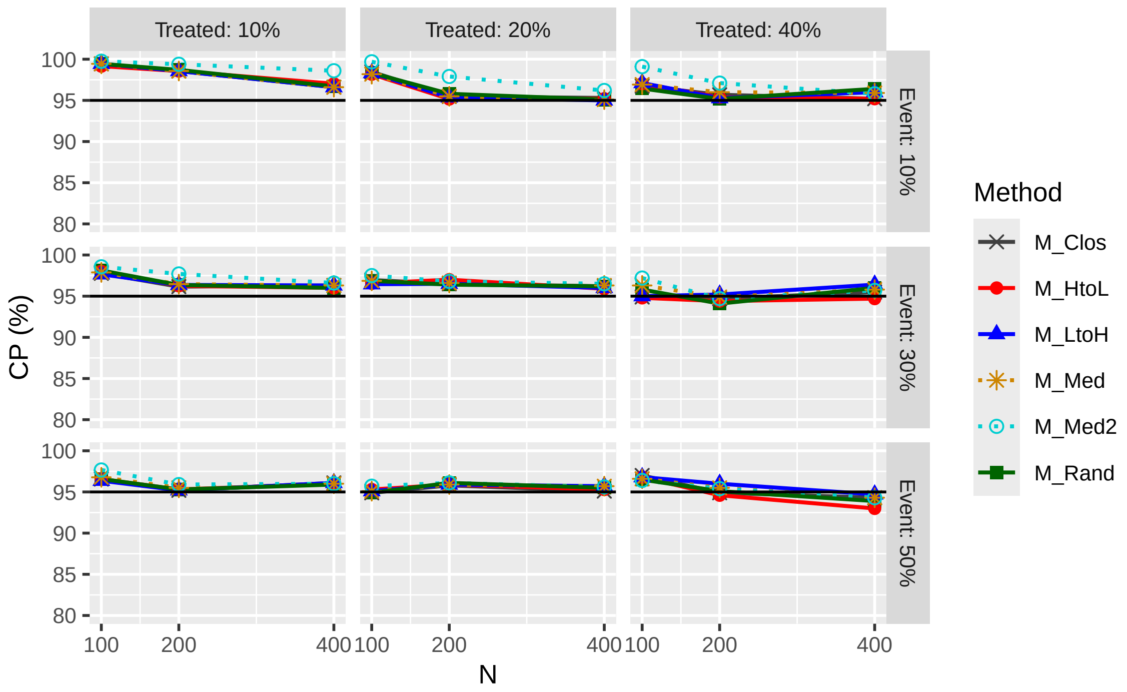

Figure S641. Coverage probability of confidence interval for OR (unimodal continuous covariate, matching ratio 1:1, true OR: 0.5, c statistic: 0.6, naive inference).

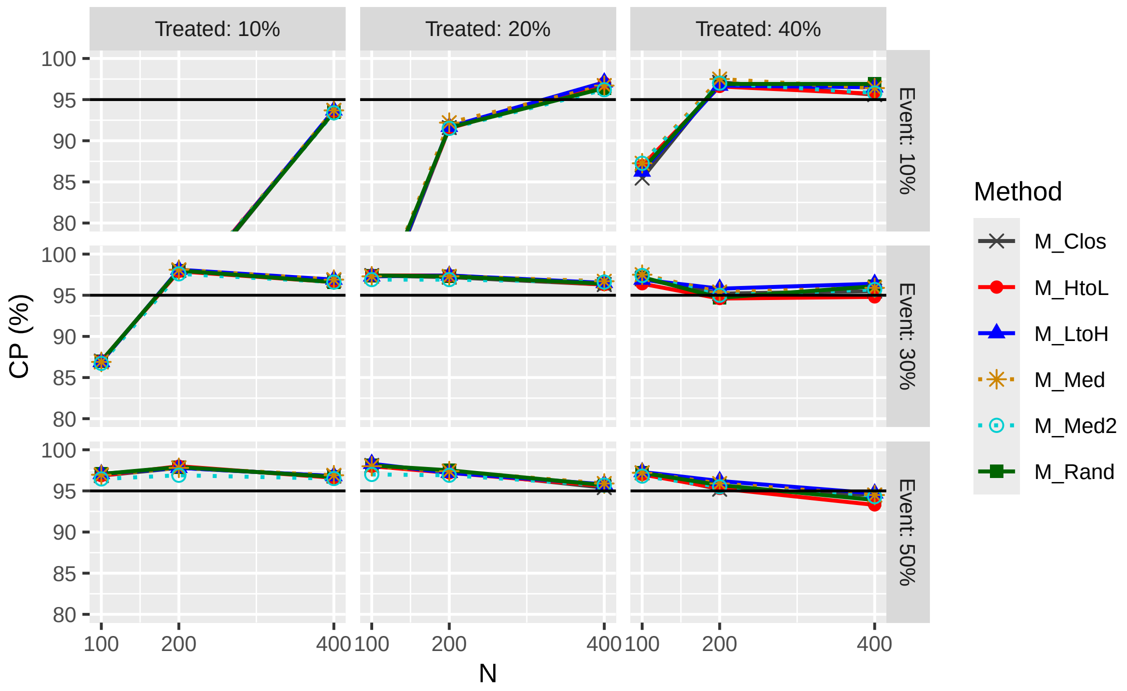

Figure S642. Coverage probability of confidence interval for OR (unimodal continuous covariate, matching ratio 1:1, true OR: 0.5, c statistic: 0.6, robust inference).

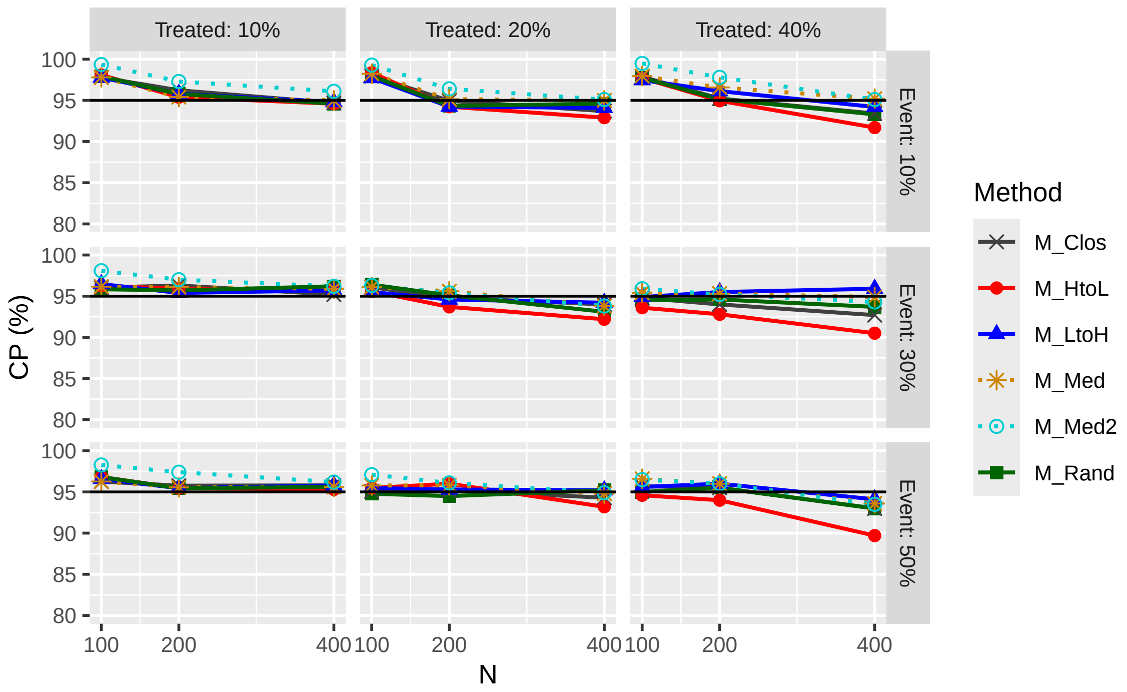

Figure S643. Coverage probability of confidence interval for OR (unimodal continuous covariate, matching ratio 1:2, true OR: 1, c statistic: 0.85, naive inference).

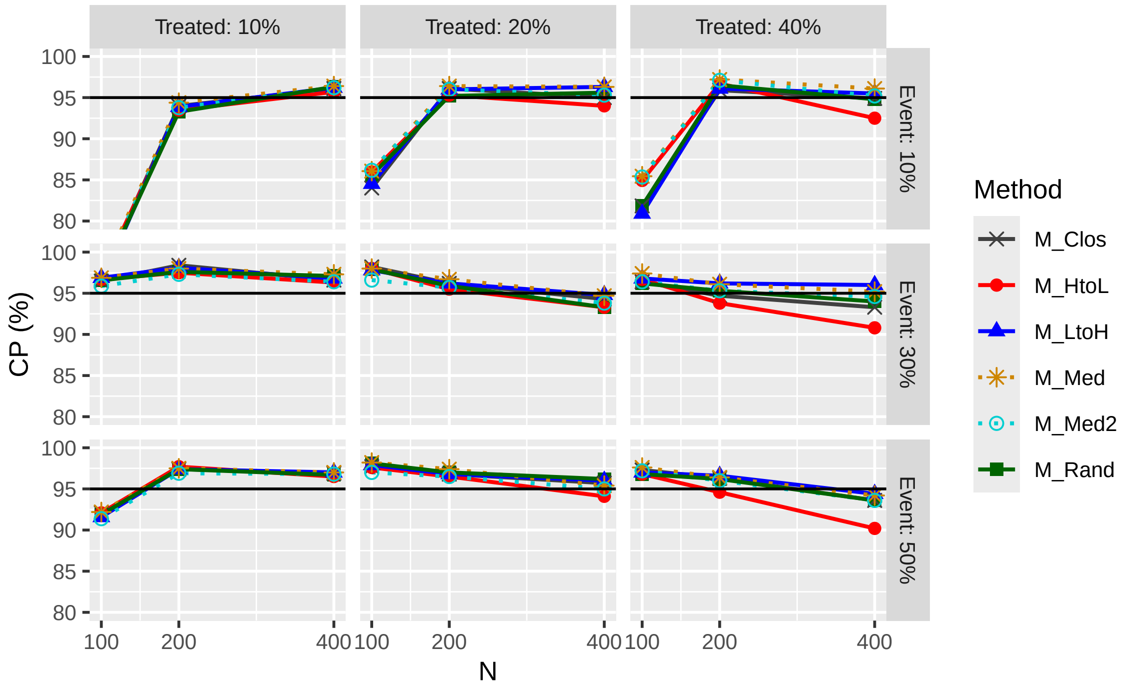

Figure S644. Coverage probability of confidence interval for OR (unimodal continuous covariate, matching ratio 1:2, true OR: 1, c statistic: 0.85, robust inference).

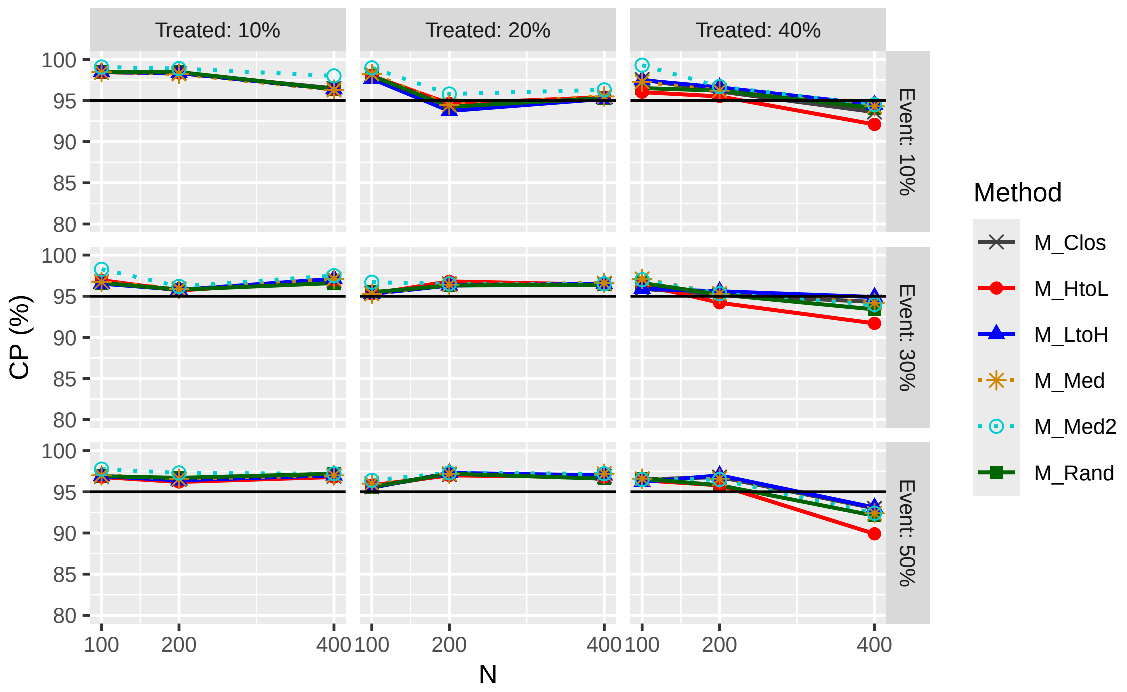

Figure S645. Coverage probability of confidence interval for OR (unimodal continuous covariate, matching ratio 1:2, true OR: 1, c statistic: 0.6, naive inference).

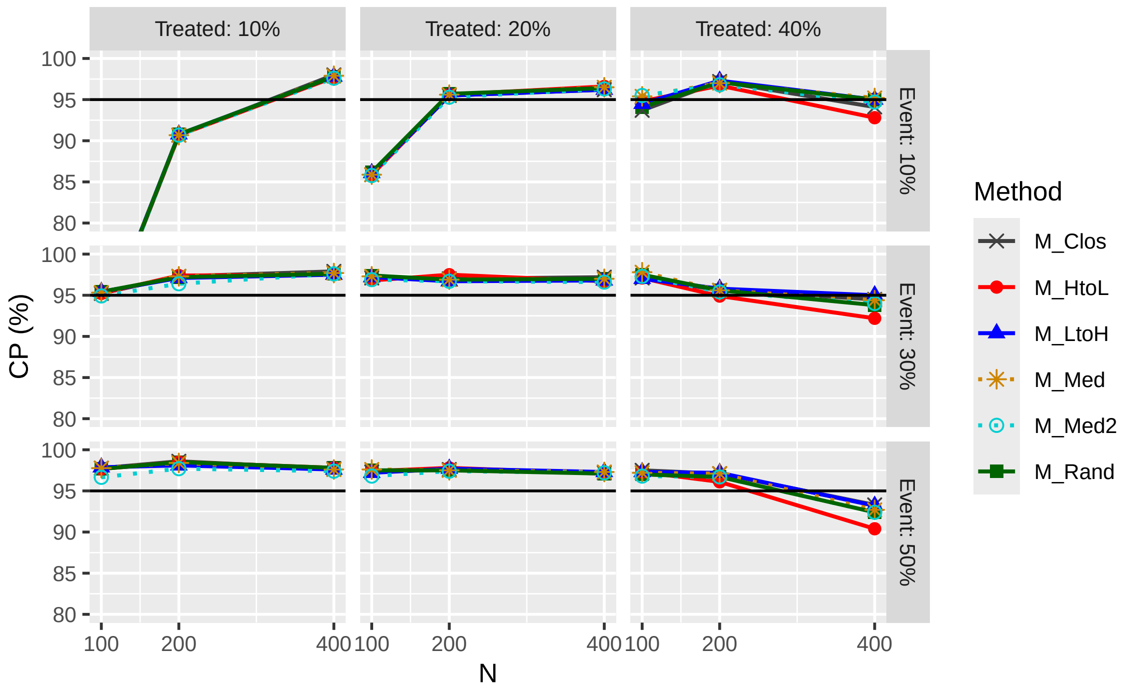

Figure S646. Coverage probability of confidence interval for OR (unimodal continuous covariate, matching ratio 1:2, true OR: 1, c statistic: 0.6, robust inference).

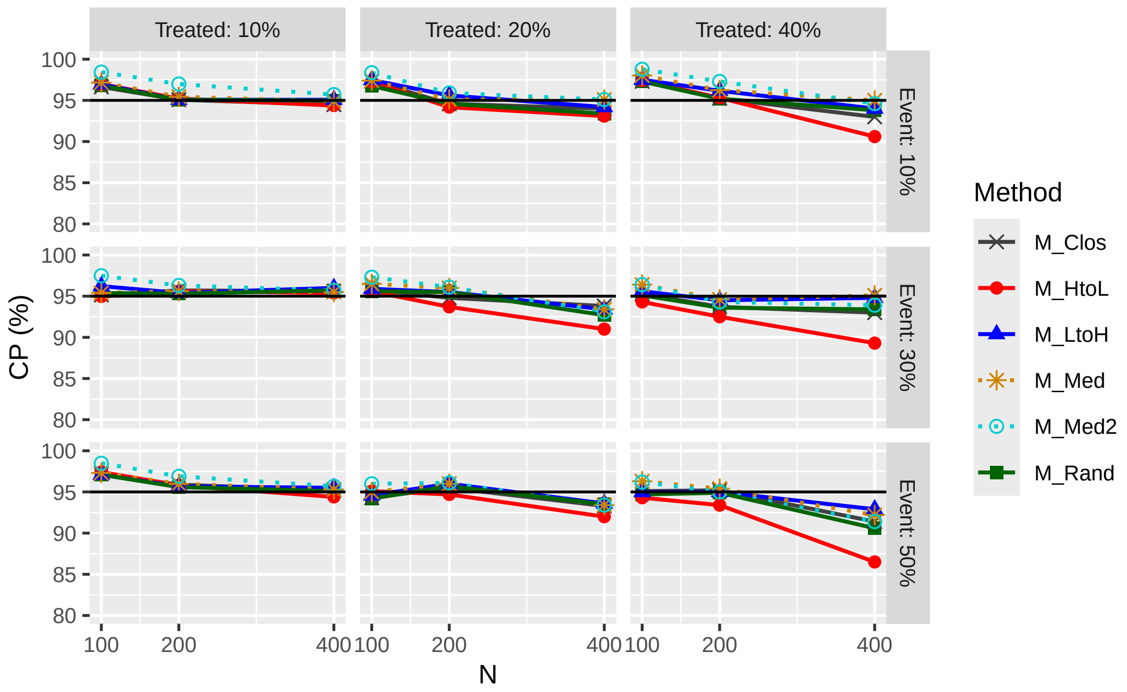

Figure S647. Coverage probability of confidence interval for OR (unimodal continuous covariate, matching ratio 1:2, true OR: 0.75, c statistic: 0.85, naive inference).

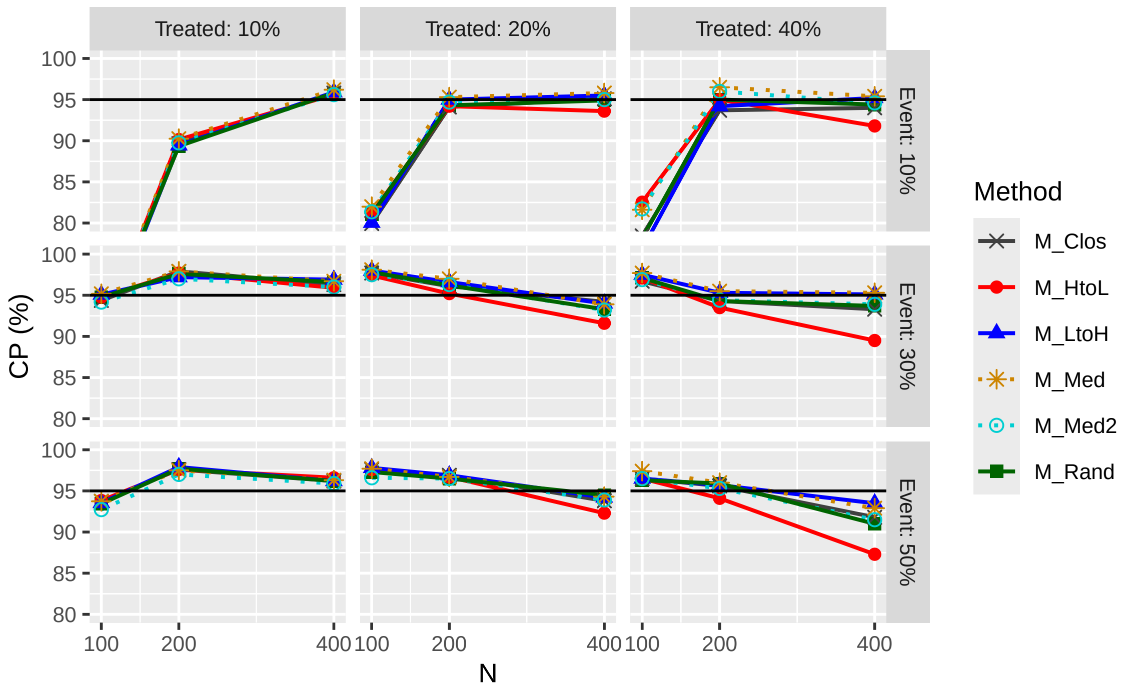

Figure S648. Coverage probability of confidence interval for OR (unimodal continuous covariate, matching ratio 1:2, true OR: 0.75, c statistic: 0.85, robust inference).

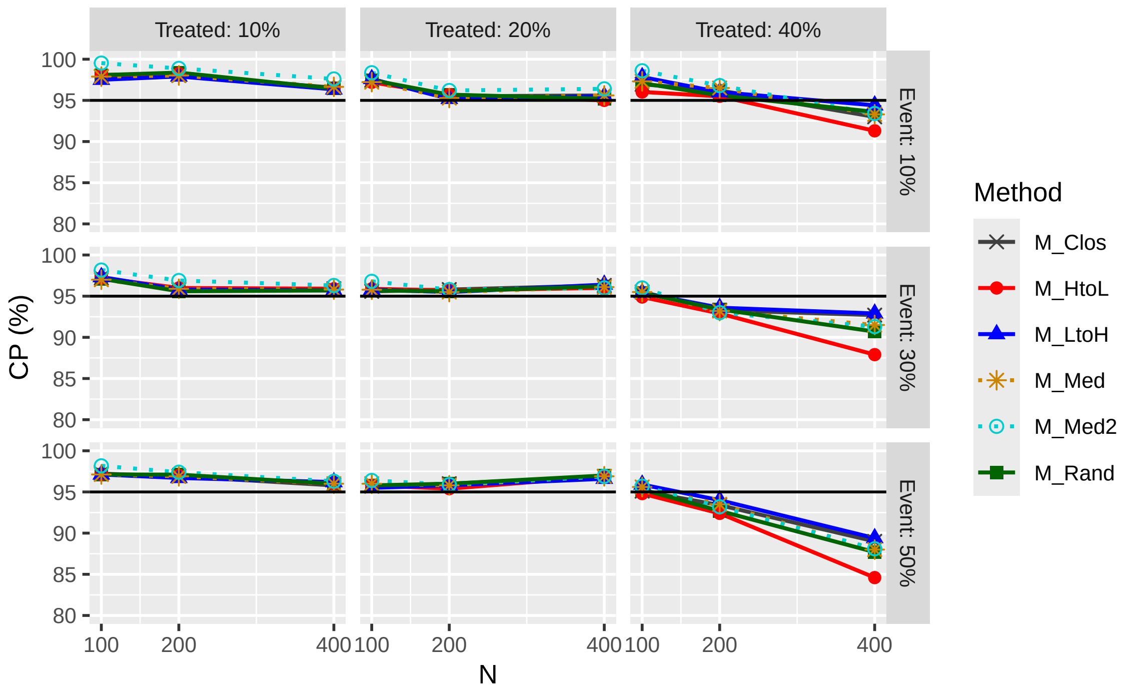

Figure S649. Coverage probability of confidence interval for OR (unimodal continuous covariate, matching ratio 1:2, true OR: 0.75, c statistic: 0.6, naive inference).

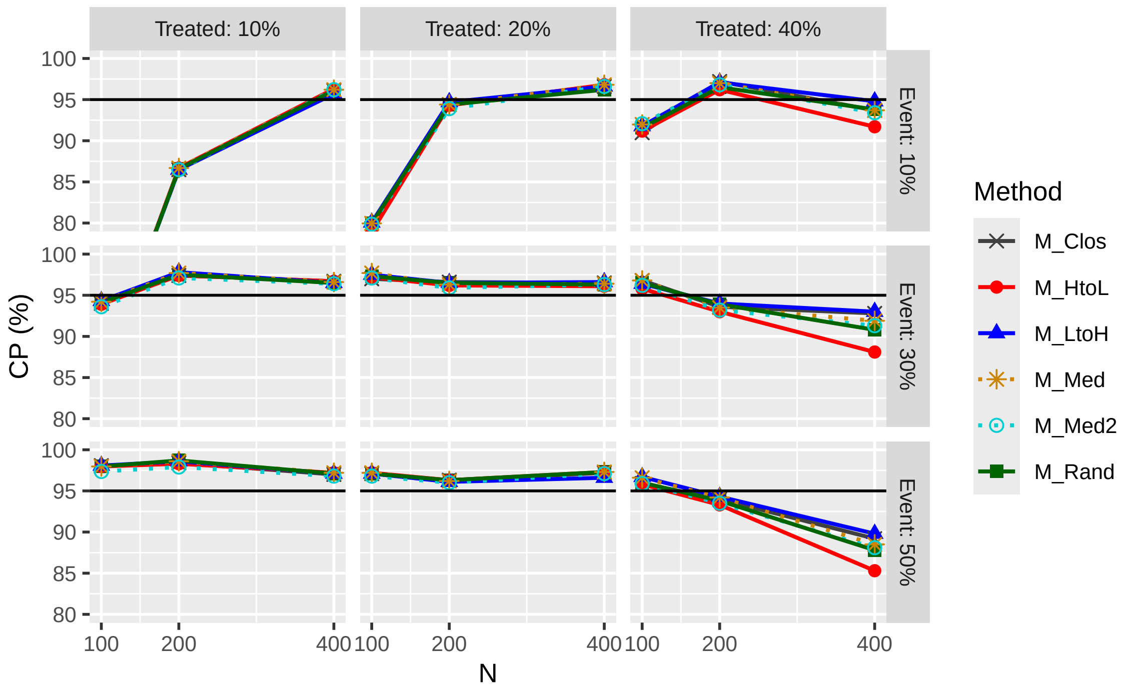

Figure S650. Coverage probability of confidence interval for OR (unimodal continuous covariate, matching ratio 1:2, true OR: 0.75, c statistic: 0.6, robust inference).

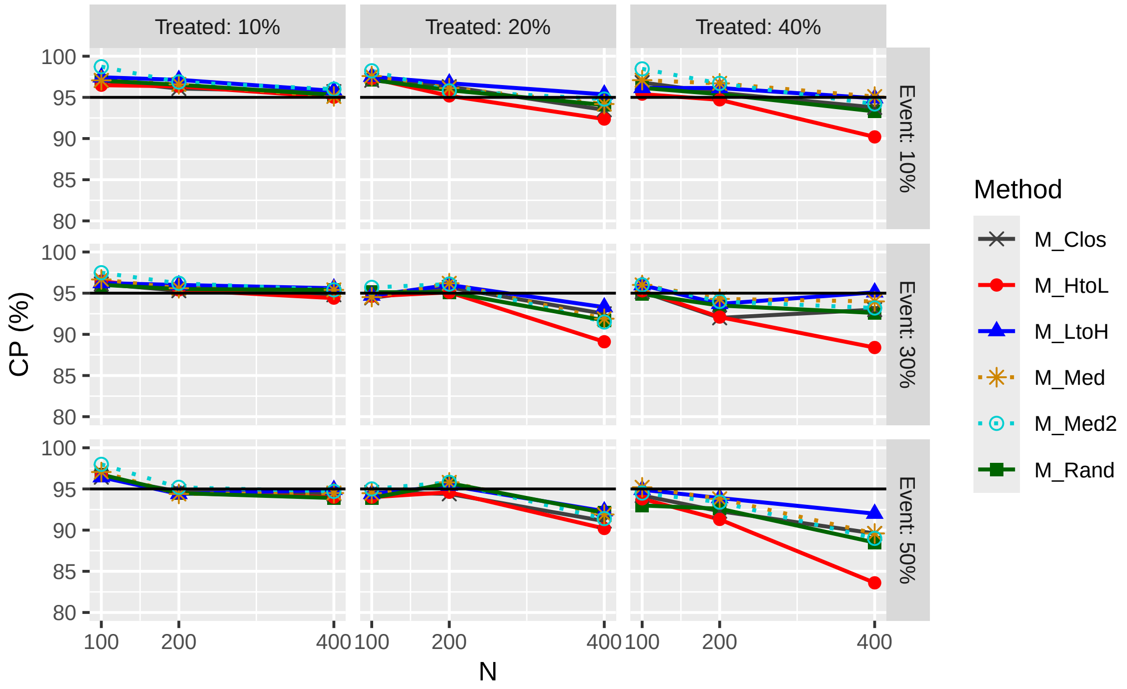

Figure S651. Coverage probability of confidence interval for OR (unimodal continuous covariate, matching ratio 1:2, true OR: 0.5, c statistic: 0.85, naive inference).

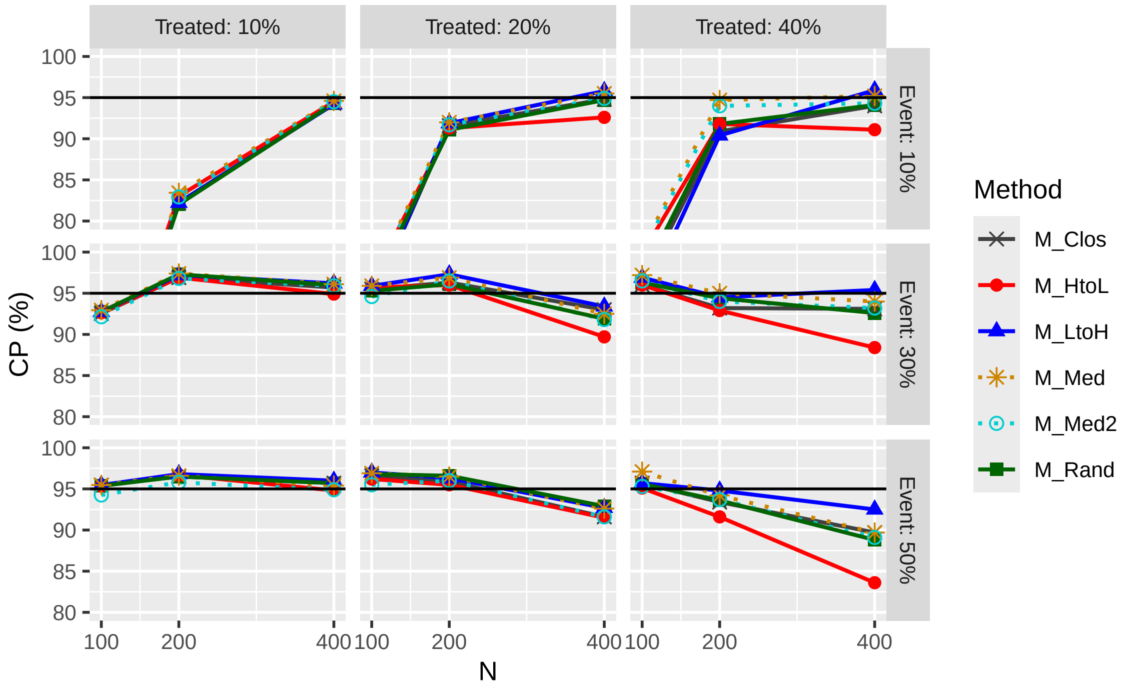

Figure S652. Coverage probability of confidence interval for OR (unimodal continuous covariate, matching ratio 1:2, true OR: 0.5, c statistic: 0.85, robust inference).

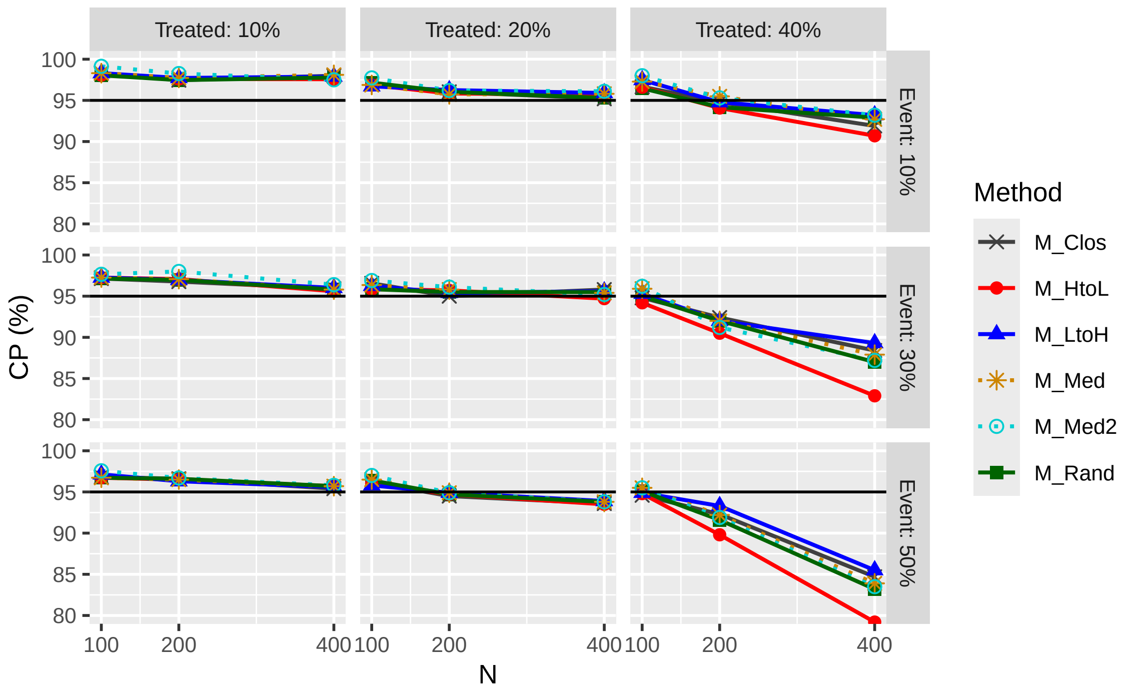

Figure S653. Coverage probability of confidence interval for OR (unimodal continuous covariate, matching ratio 1:2, true OR: 0.5, c statistic: 0.6, naive inference).

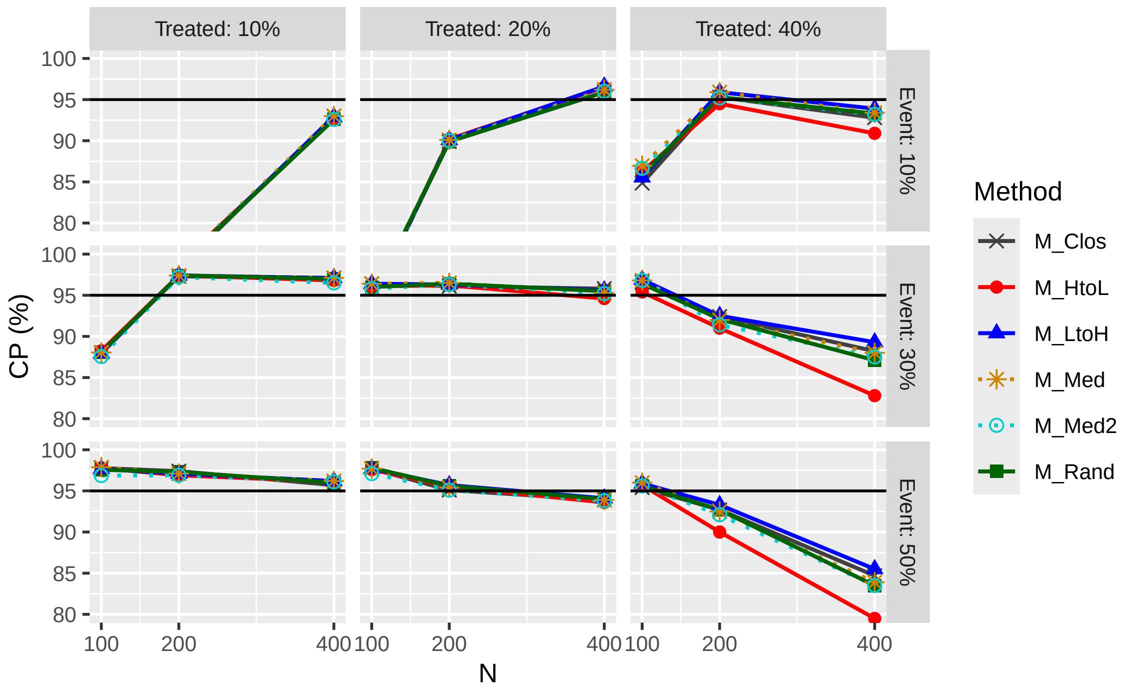

Figure S654. Coverage probability of confidence interval for OR (unimodal continuous covariate, matching ratio 1:2, true OR: 0.5, c statistic: 0.6, robust inference).

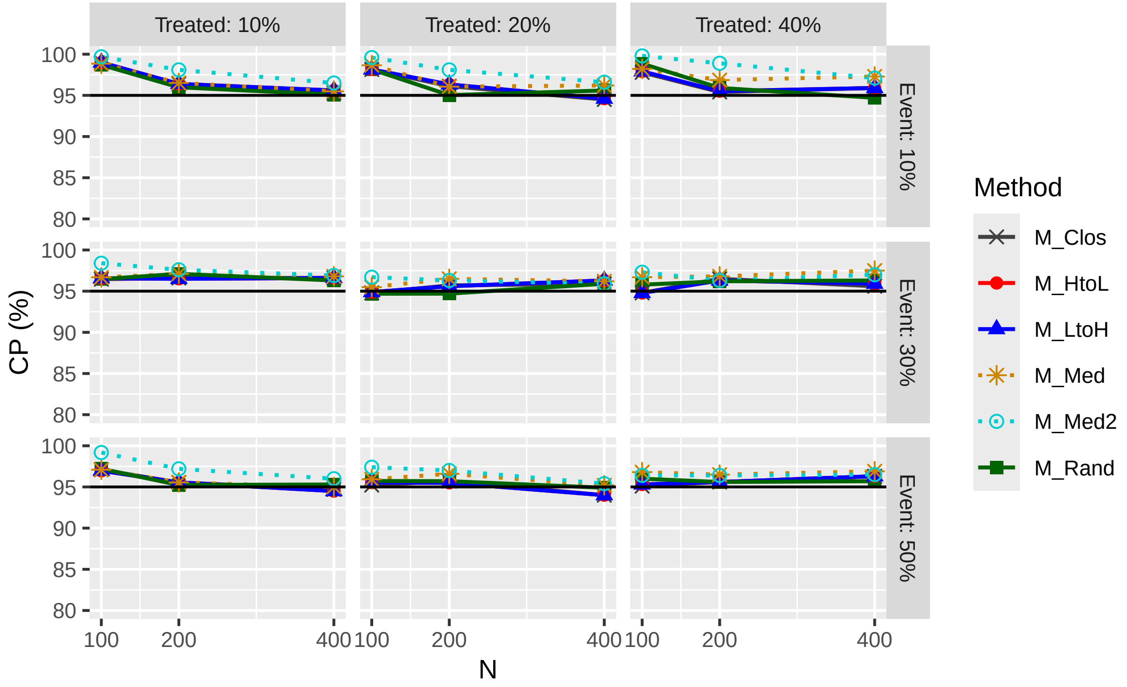

Figure S655. Coverage probability of confidence interval for OR (categorical covariate, matching ratio 1:1, true OR: 1, c statistic: 0.85, naive inference).

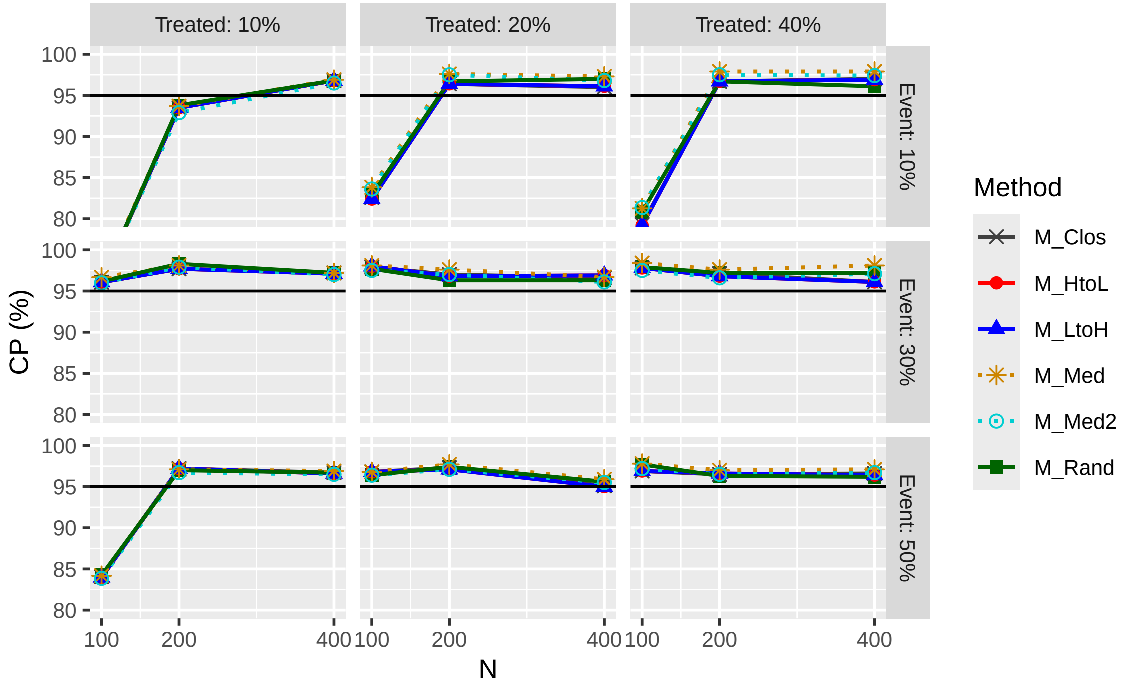

Figure S656. Coverage probability of confidence interval for OR (categorical covariate, matching ratio 1:1, true OR: 1, c statistic: 0.85, robust inference).

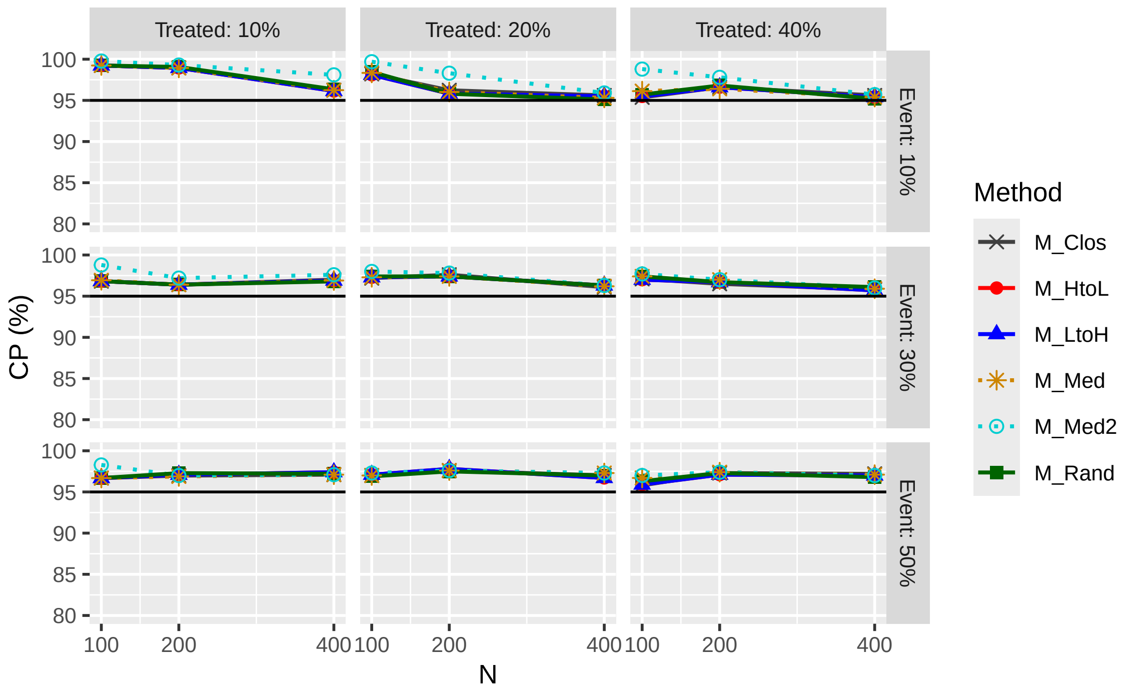

Figure S657. Coverage probability of confidence interval for OR (categorical covariate, matching ratio 1:1, true OR: 1, c statistic: 0.6, naive inference).

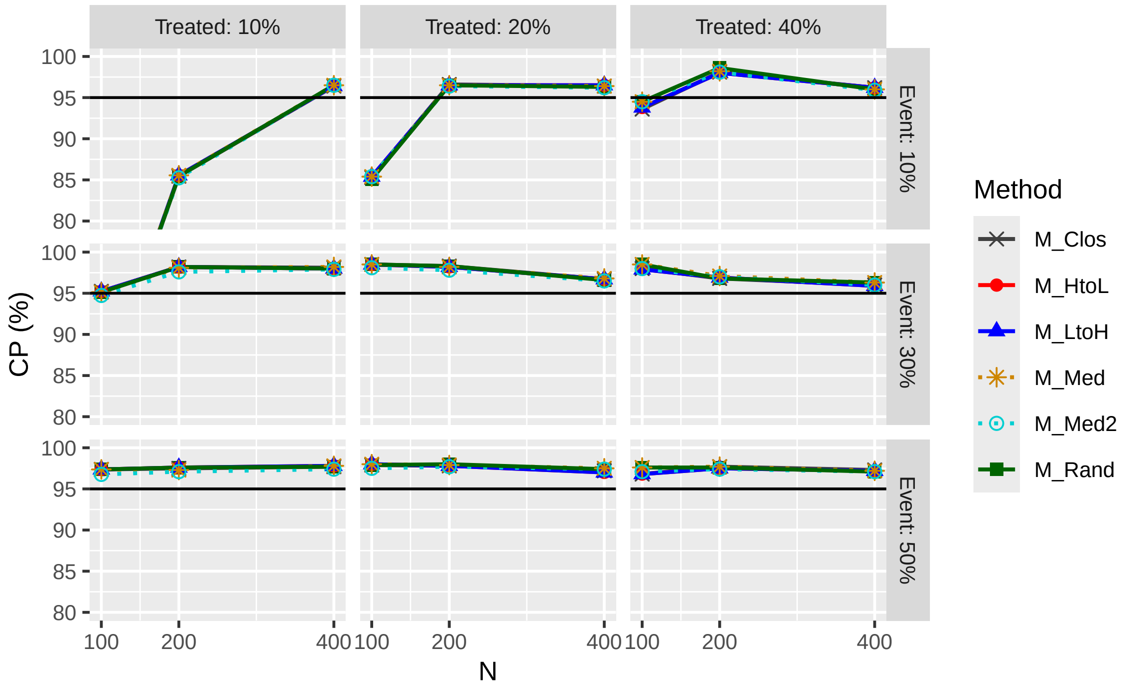

Figure S658. Coverage probability of confidence interval for OR (categorical covariate, matching ratio 1:1, true OR: 1, c statistic: 0.6, robust inference).

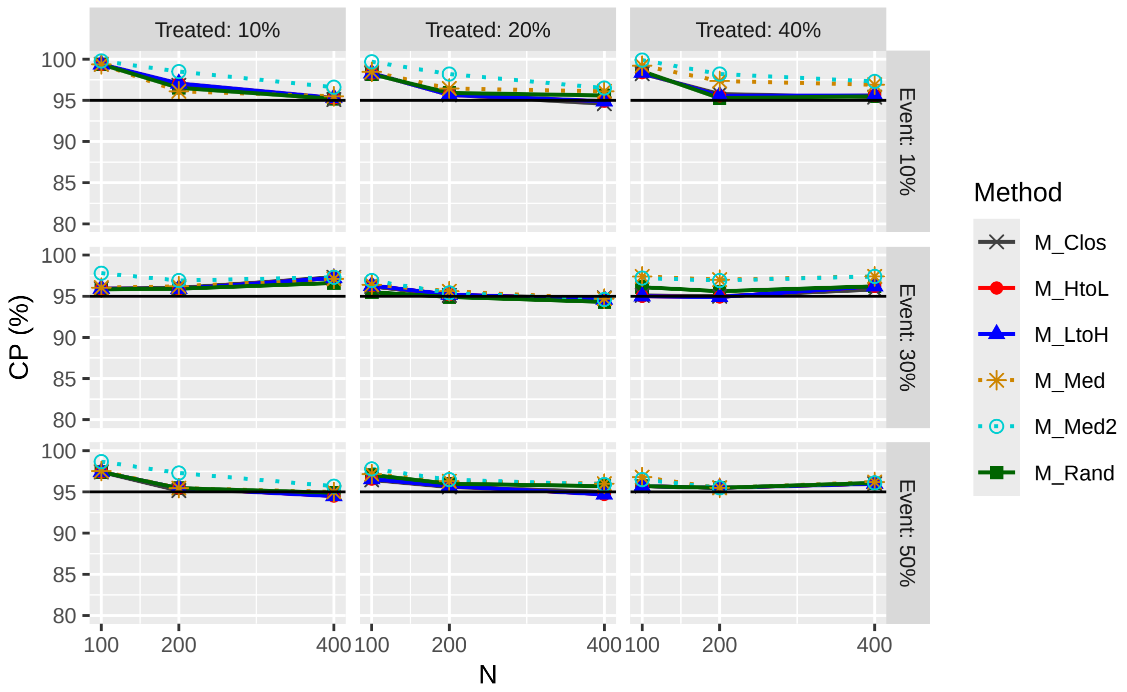

Figure S659. Coverage probability of confidence interval for OR (categorical covariate, matching ratio 1:1, true OR: 0.75, c statistic: 0.85, naive inference).

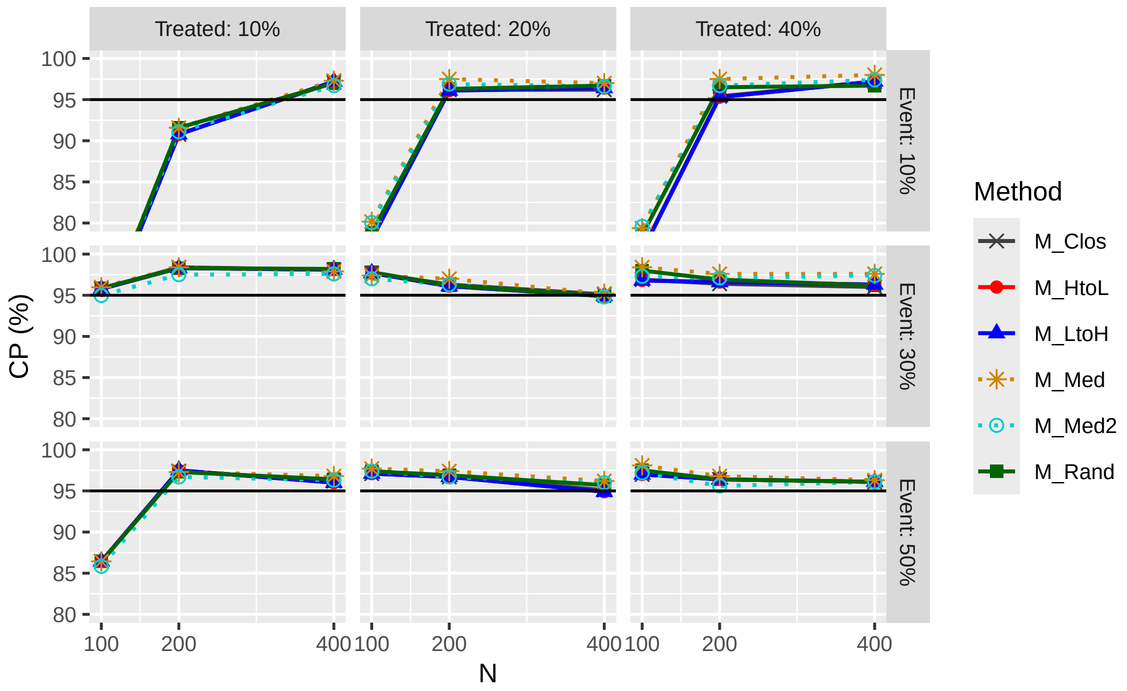

Figure S660. Coverage probability of confidence interval for OR (categorical covariate, matching ratio 1:1, true OR: 0.75, c statistic: 0.85, robust inference).

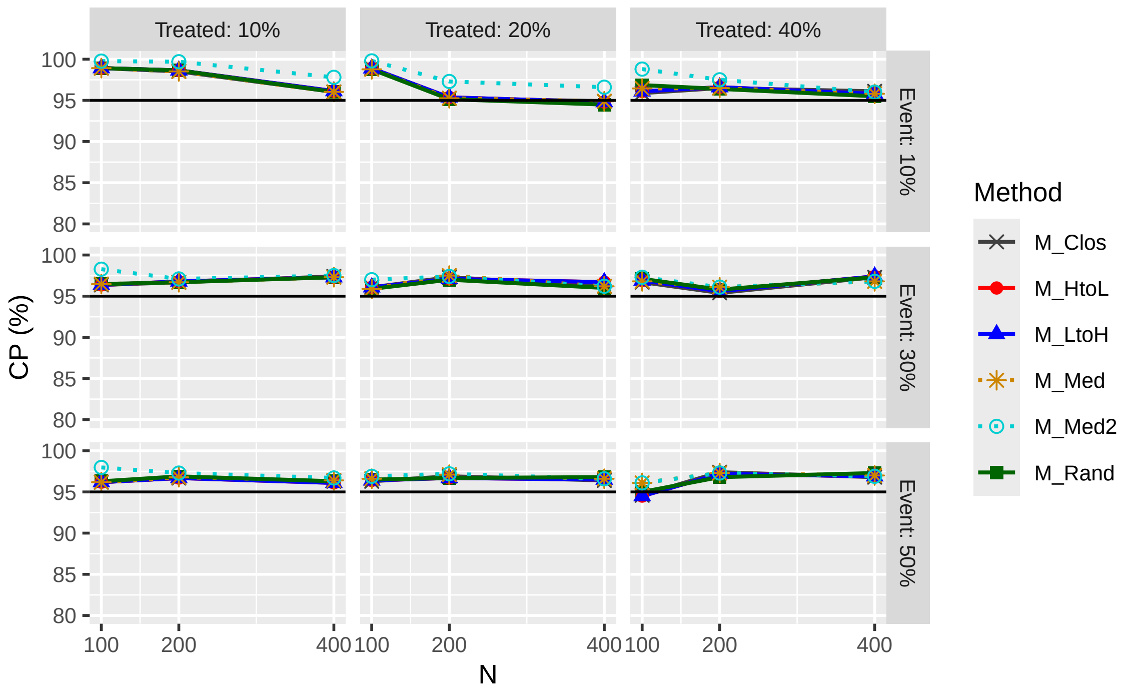

Figure S661. Coverage probability of confidence interval for OR (categorical covariate, matching ratio 1:1, true OR: 0.75, c statistic: 0.6, naive inference).

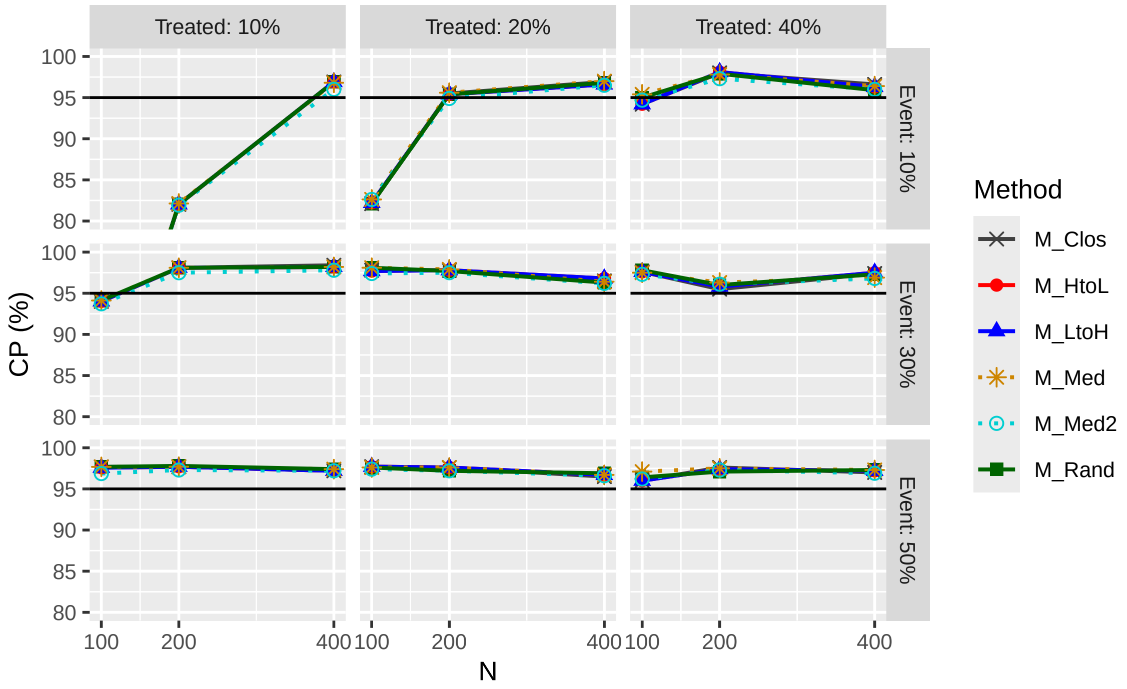

Figure S662. Coverage probability of confidence interval for OR (categorical covariate, matching ratio 1:1, true OR: 0.75, c statistic: 0.6, robust inference).

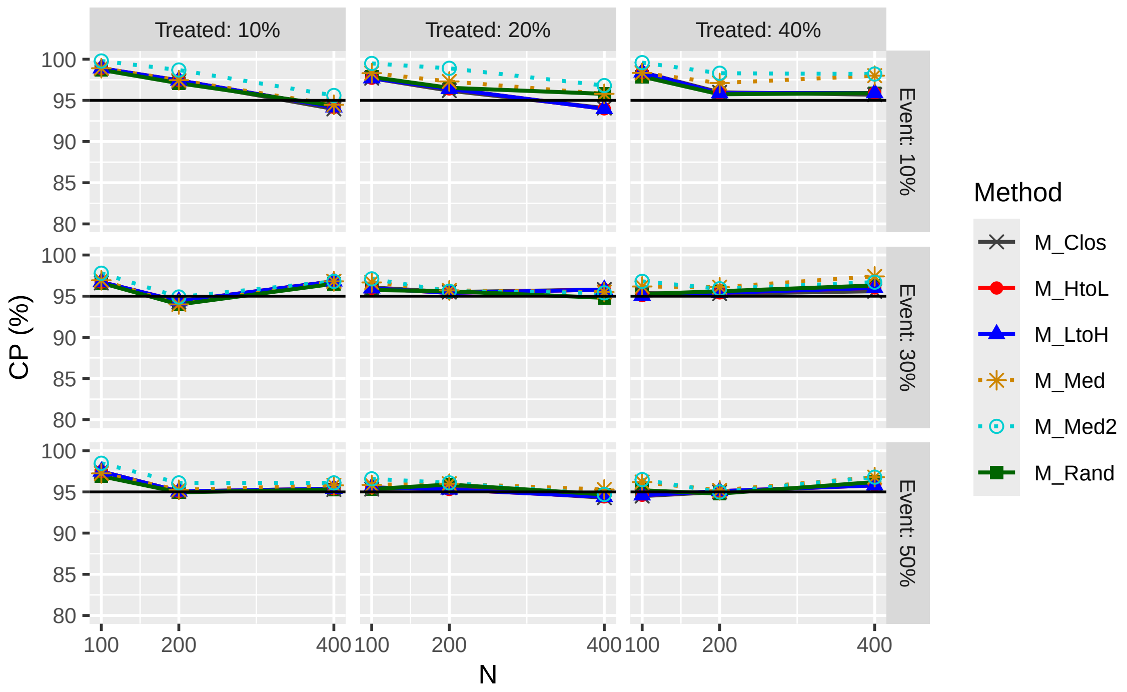

Figure S663. Coverage probability of confidence interval for OR (categorical covariate, matching ratio 1:1, true OR: 0.5, c statistic: 0.85, naive inference).

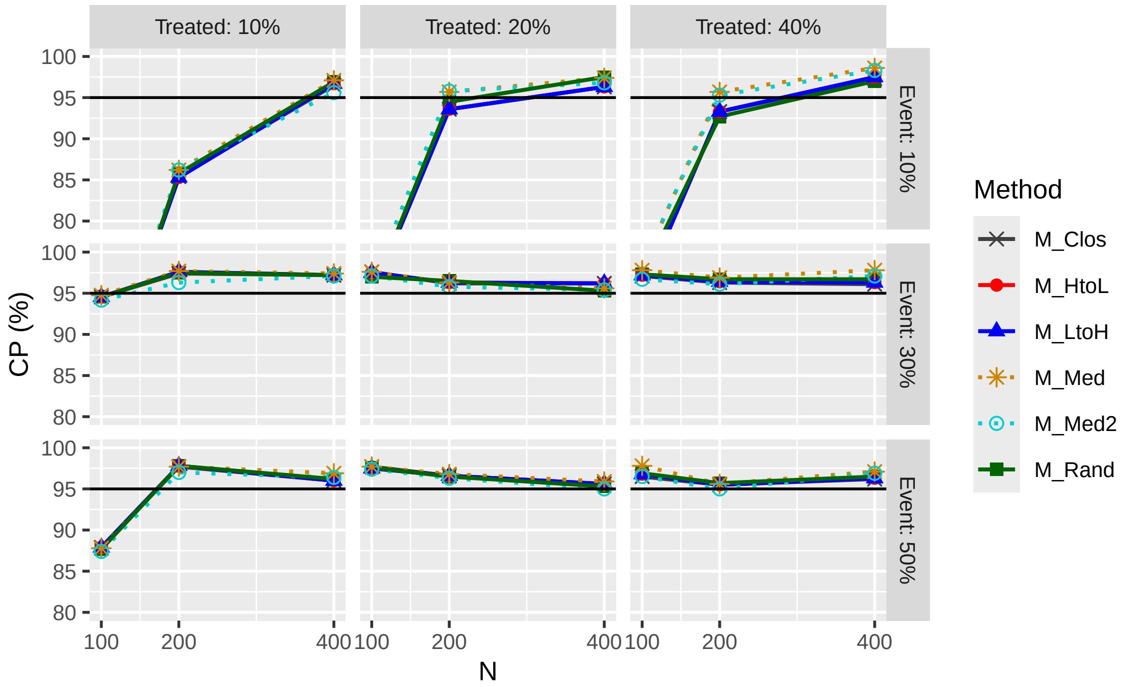

Figure S664. Coverage probability of confidence interval for OR (categorical covariate, matching ratio 1:1, true OR: 0.5, c statistic: 0.85, robust inference).

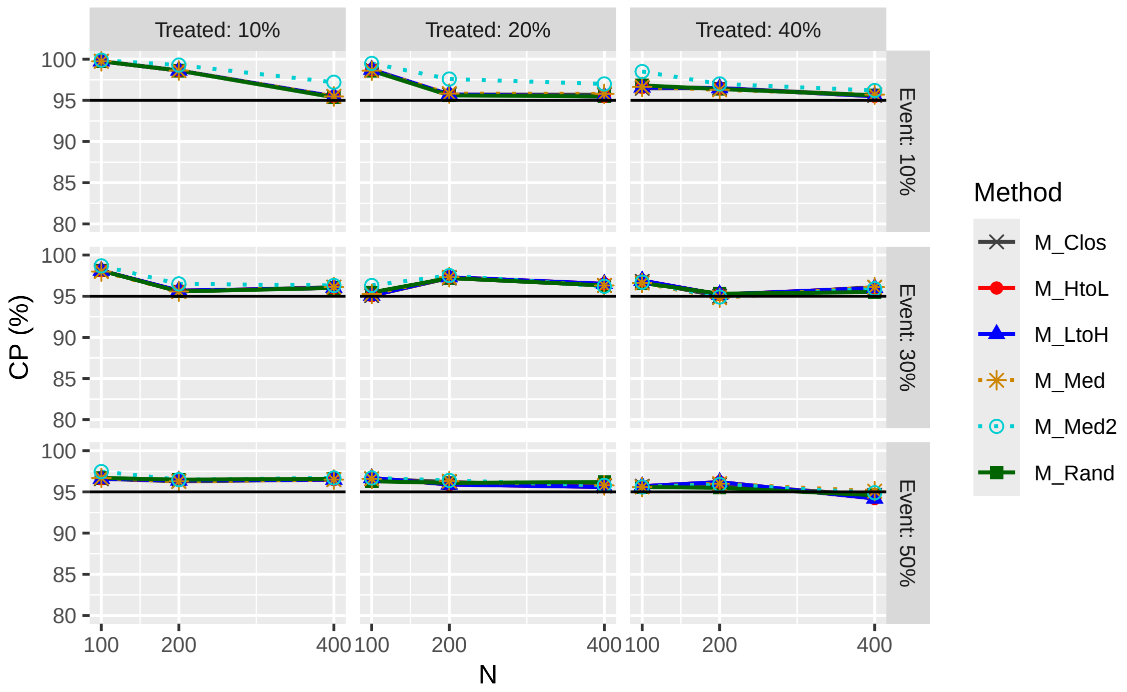

Figure S665. Coverage probability of confidence interval for OR (categorical covariate, matching ratio 1:1, true OR: 0.5, c statistic: 0.6, naive inference).

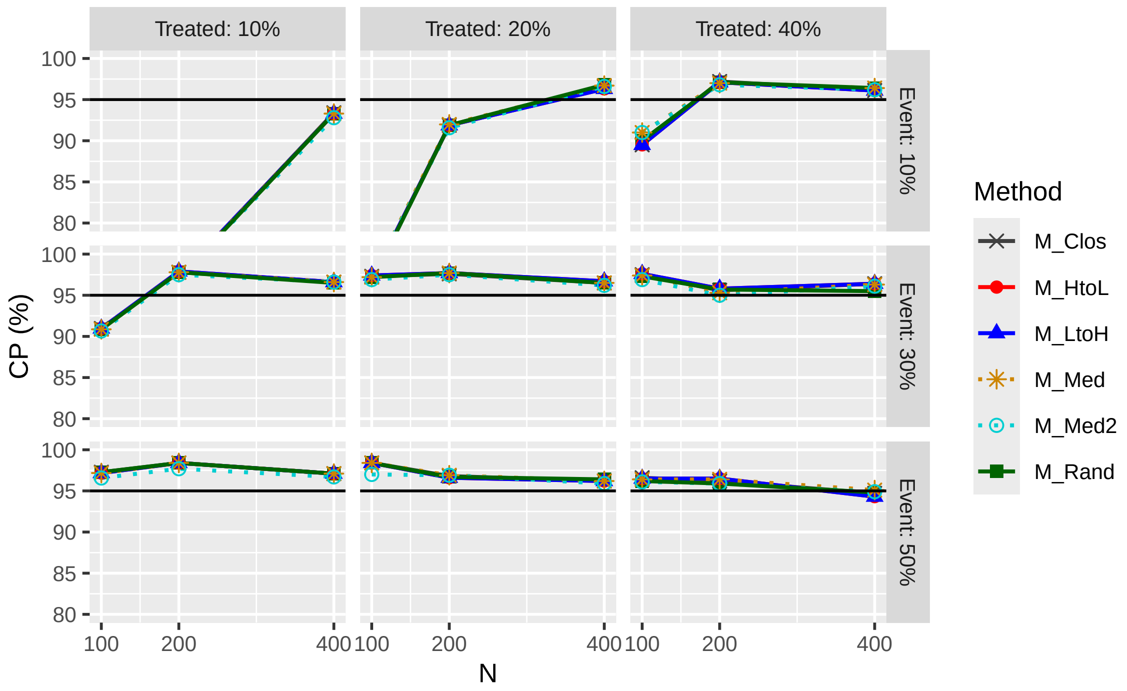

Figure S666. Coverage probability of confidence interval for OR (categorical covariate, matching ratio 1:1, true OR: 0.5, c statistic: 0.6, robust inference).

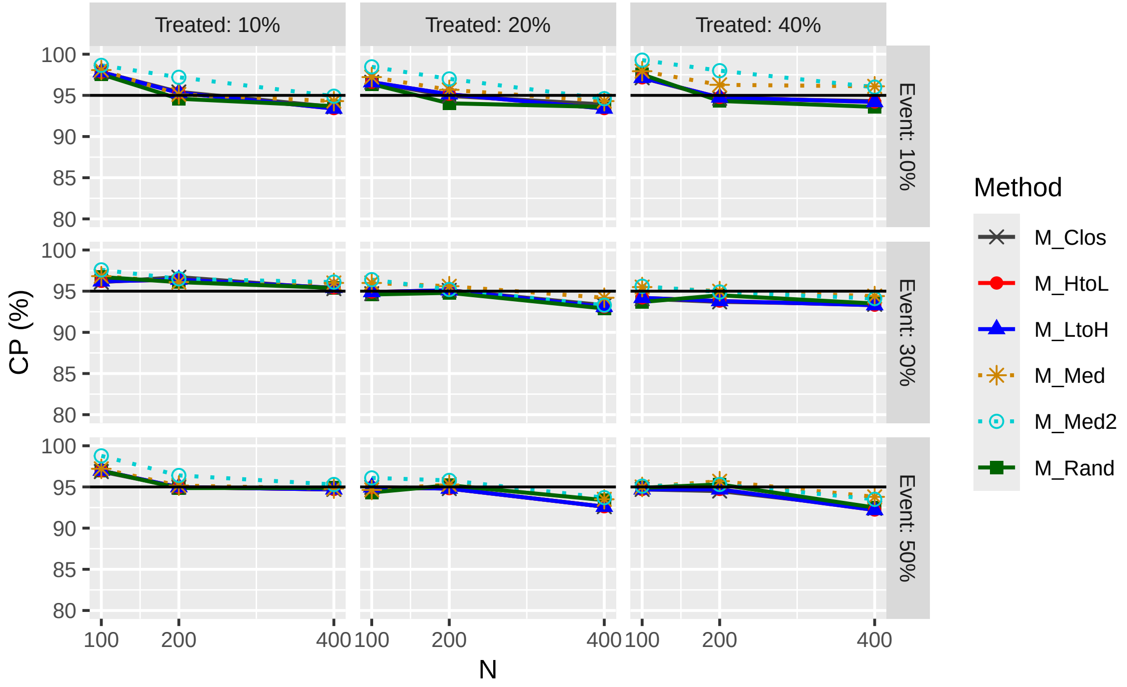

Figure S667. Coverage probability of confidence interval for OR (categorical covariate, matching ratio 1:2, true OR: 1, c statistic: 0.85, naive inference).

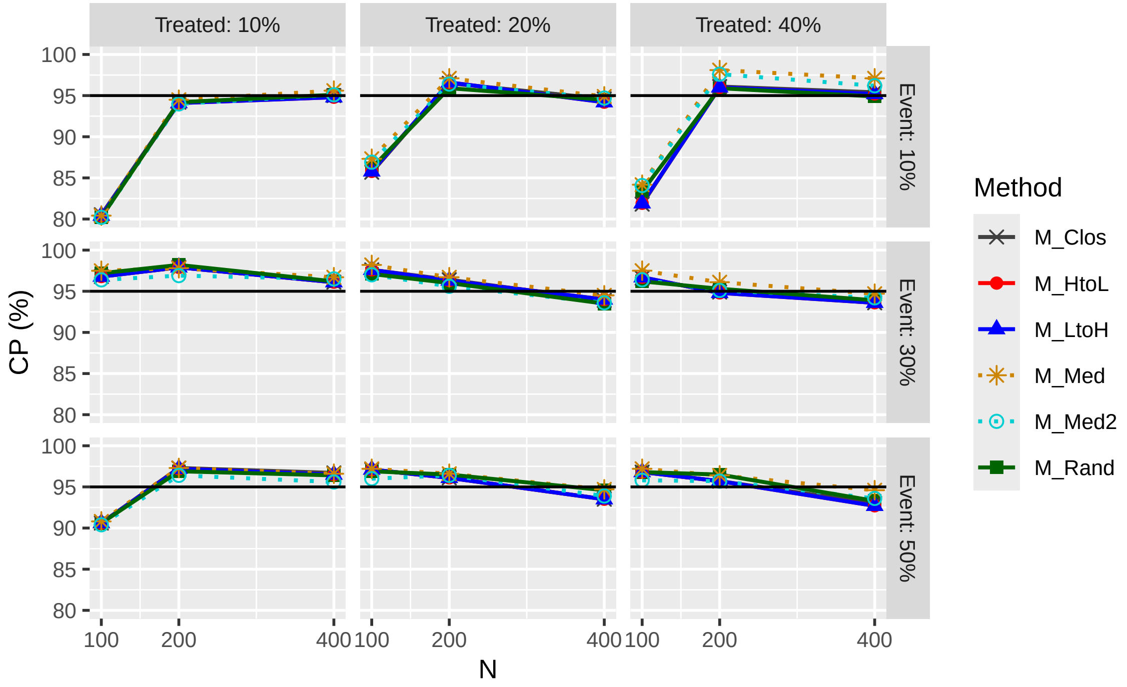

Figure S668. Coverage probability of confidence interval for OR (categorical covariate, matching ratio 1:2, true OR: 1, c statistic: 0.85, robust inference).

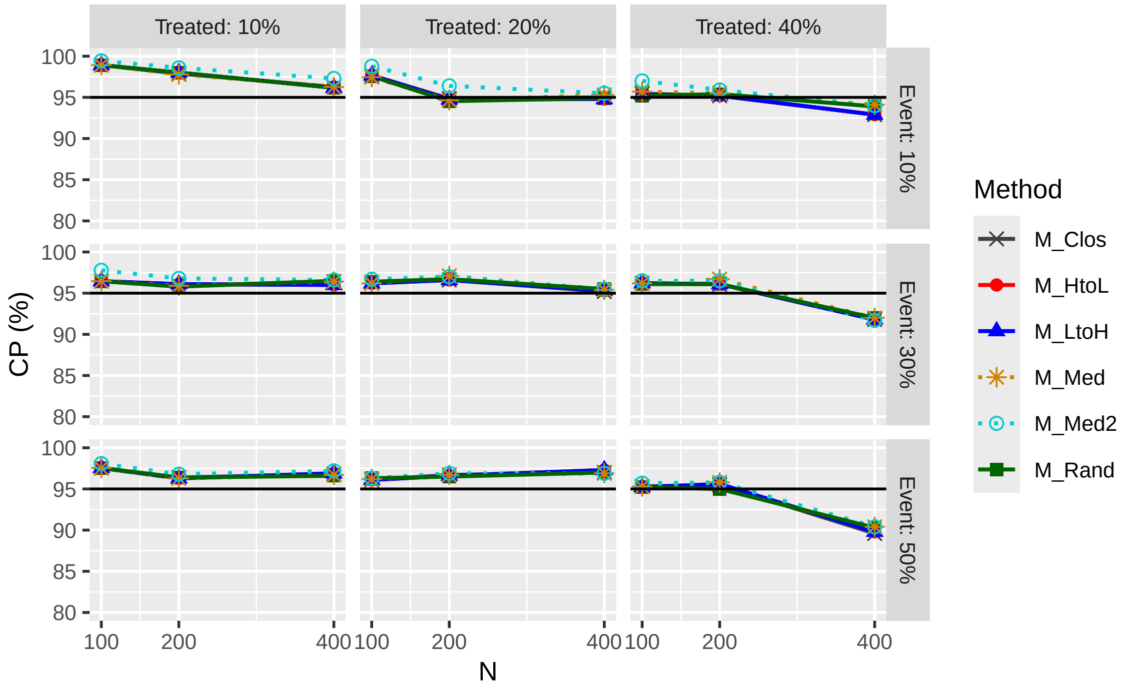

Figure S669. Coverage probability of confidence interval for OR (categorical covariate, matching ratio 1:2, true OR: 1, c statistic: 0.6, naive inference).

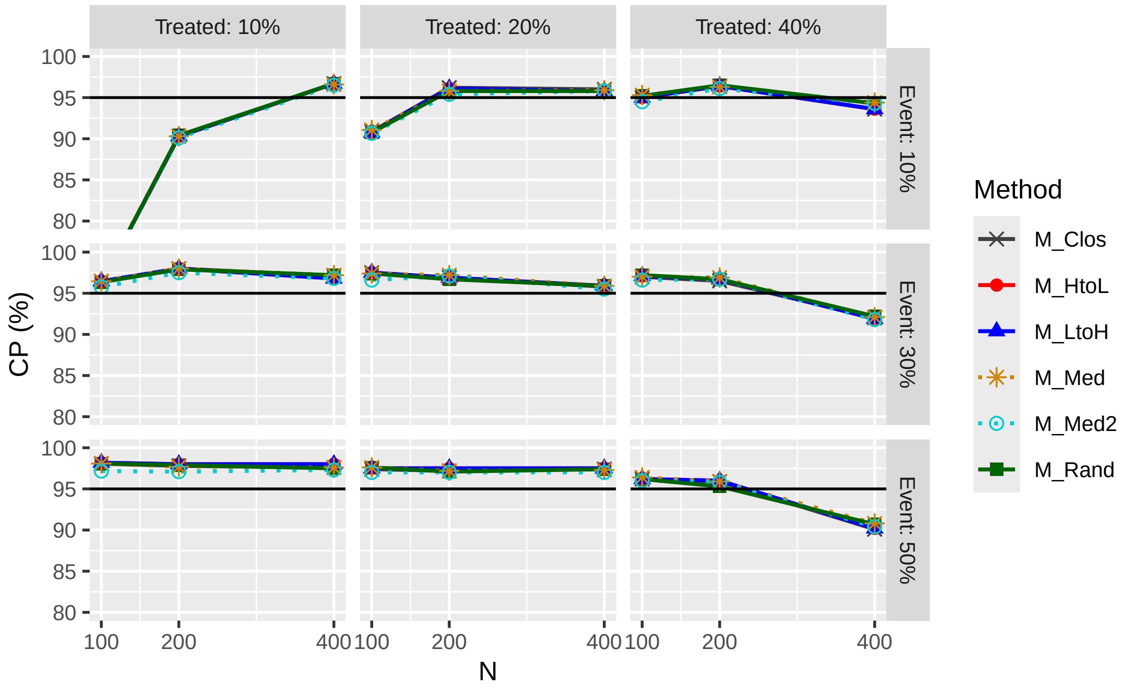

Figure S670. Coverage probability of confidence interval for OR (categorical covariate, matching ratio 1:2, true OR: 1, c statistic: 0.6, robust inference).

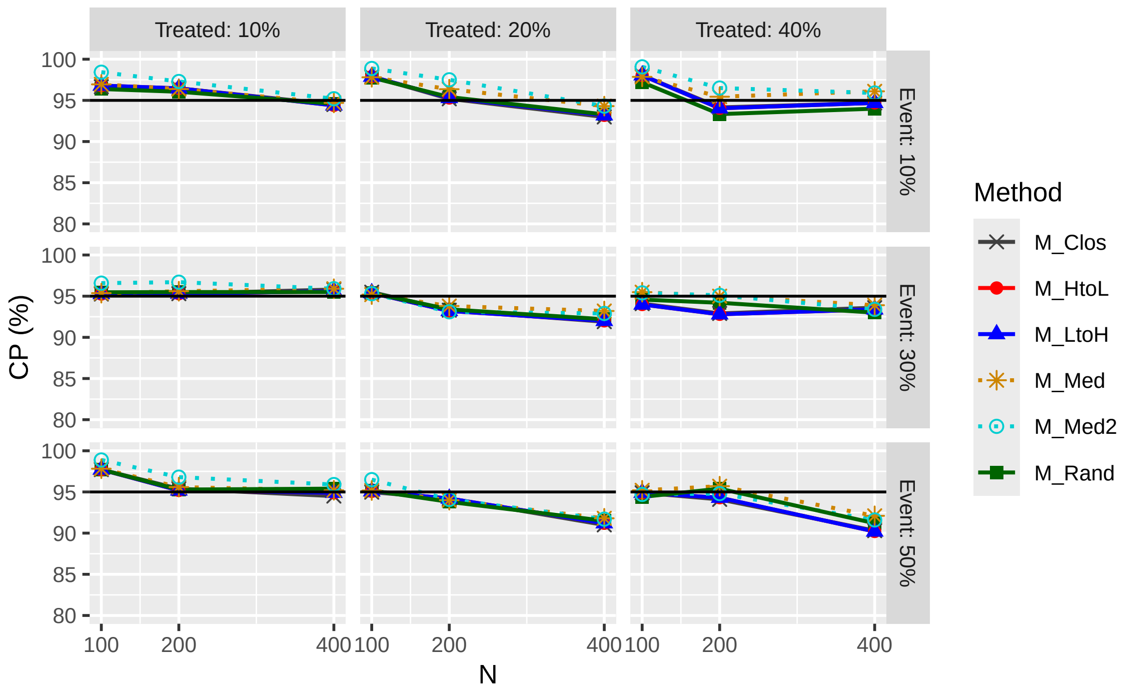

Figure S671. Coverage probability of confidence interval for OR (categorical covariate, matching ratio 1:2, true OR: 0.75, c statistic: 0.85, naive inference).

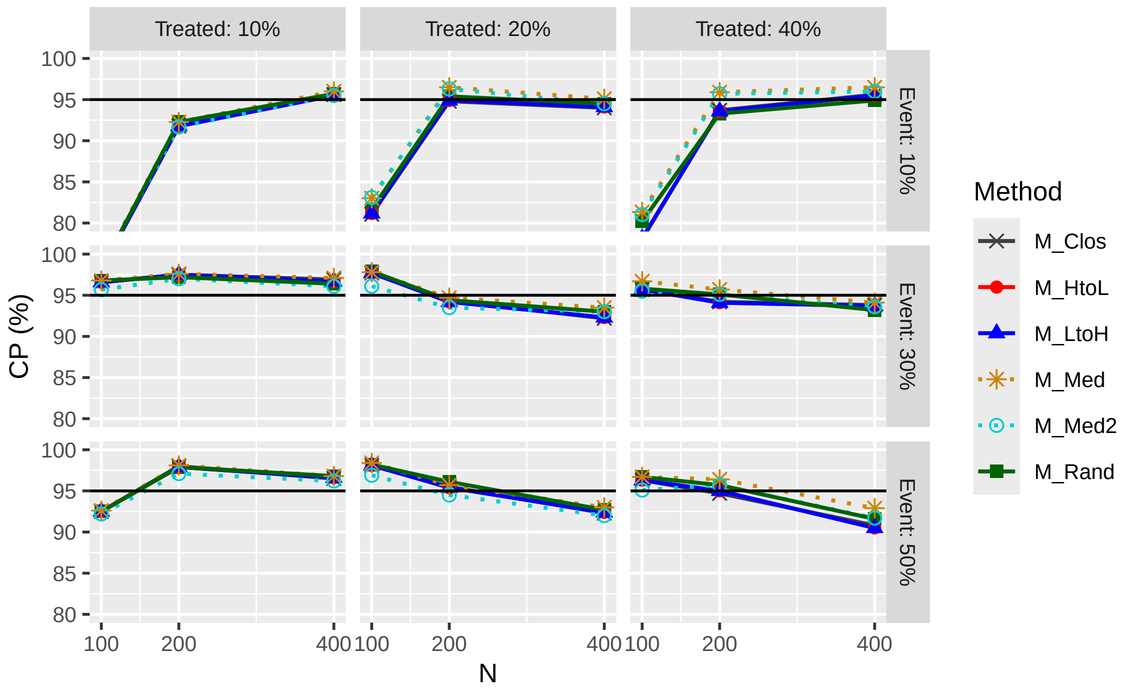

Figure S672. Coverage probability of confidence interval for OR (categorical covariate, matching ratio 1:2, true OR: 0.75, c statistic: 0.85, robust inference).

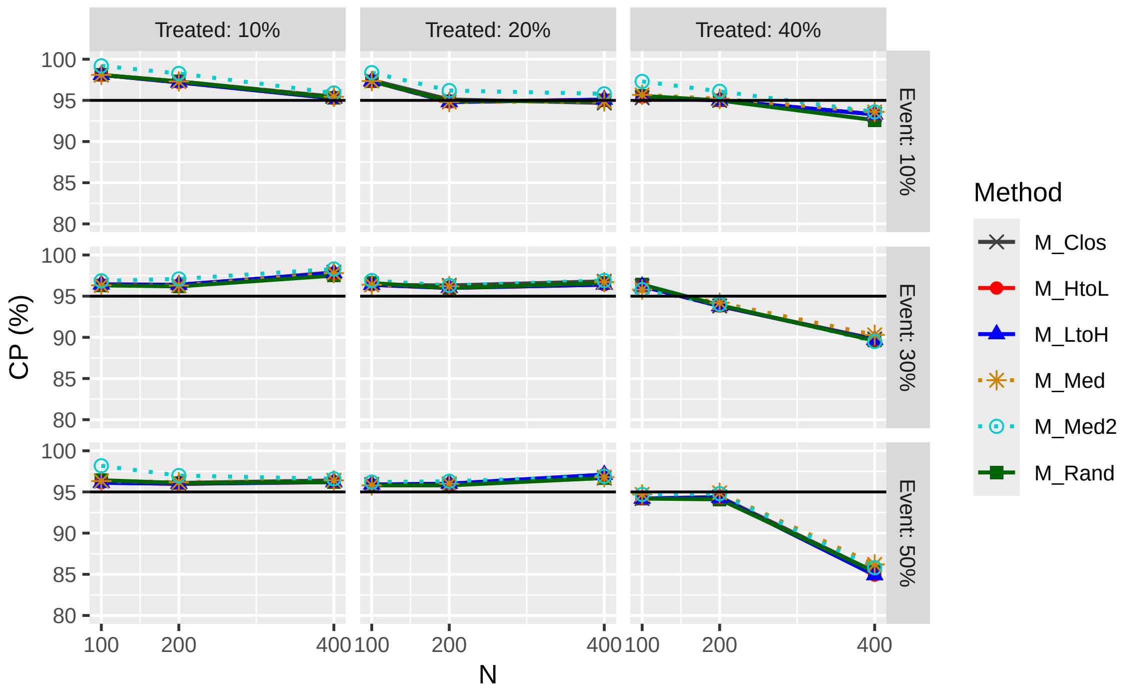

Figure S673. Coverage probability of confidence interval for OR (categorical covariate, matching ratio 1:2, true OR: 0.75, c statistic: 0.6, naive inference).

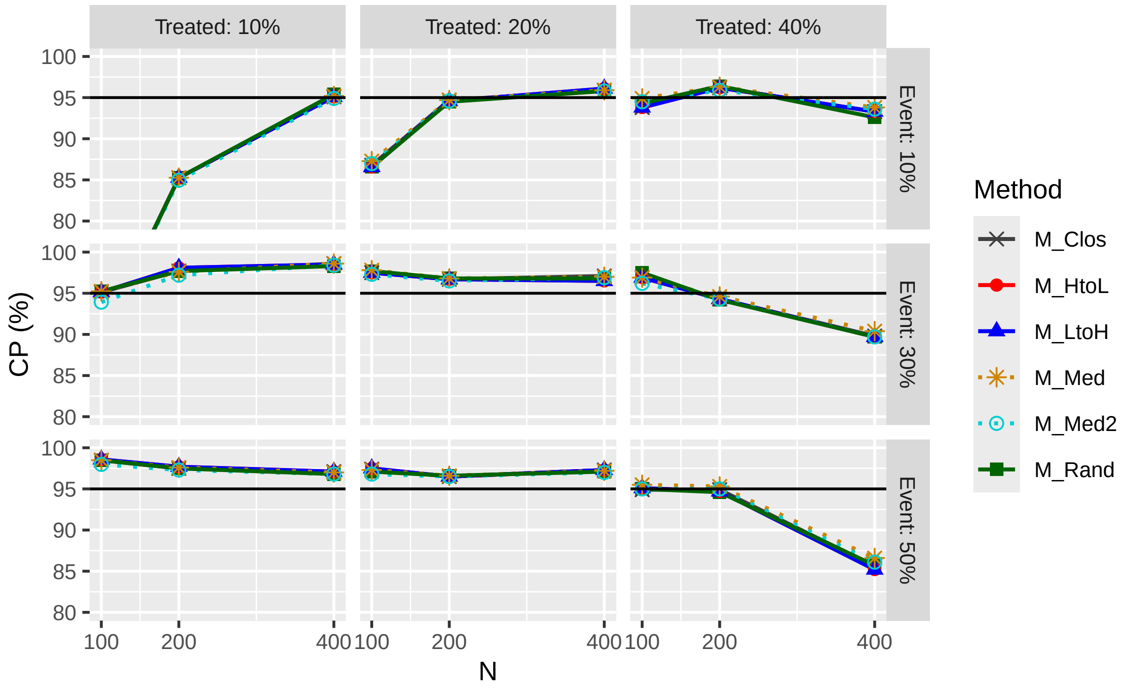

Figure S674. Coverage probability of confidence interval for OR (categorical covariate, matching ratio 1:2, true OR: 0.75, c statistic: 0.6, robust inference).

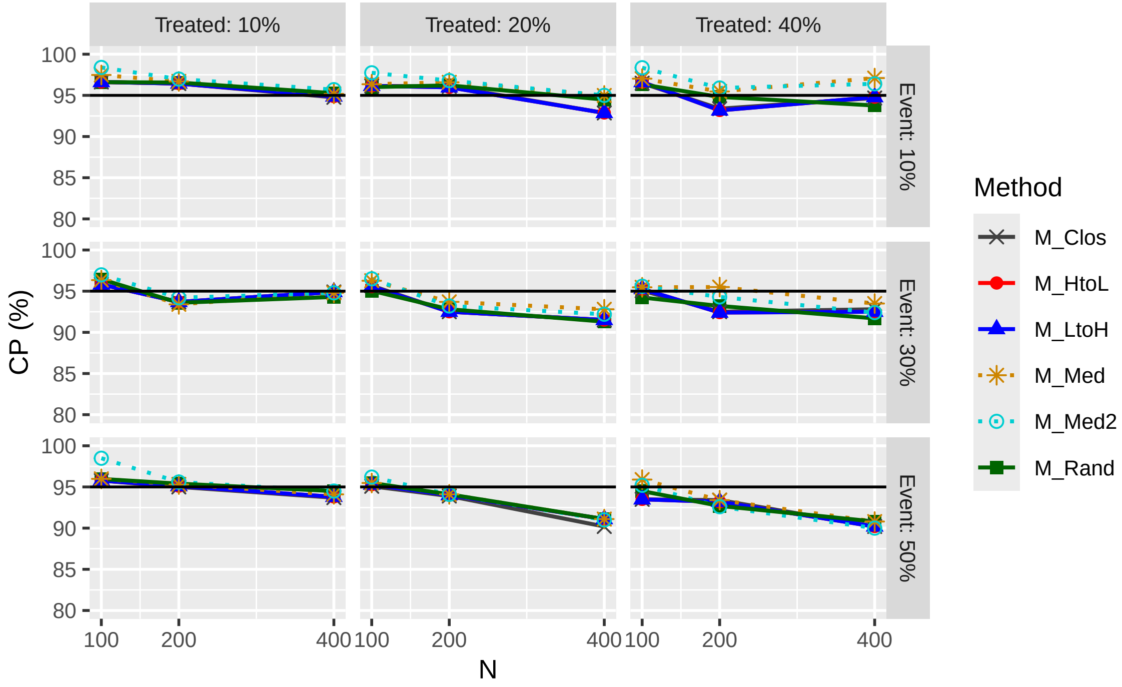

Figure S675. Coverage probability of confidence interval for OR (categorical covariate, matching ratio 1:2, true OR: 0.5, c statistic: 0.85, naive inference).

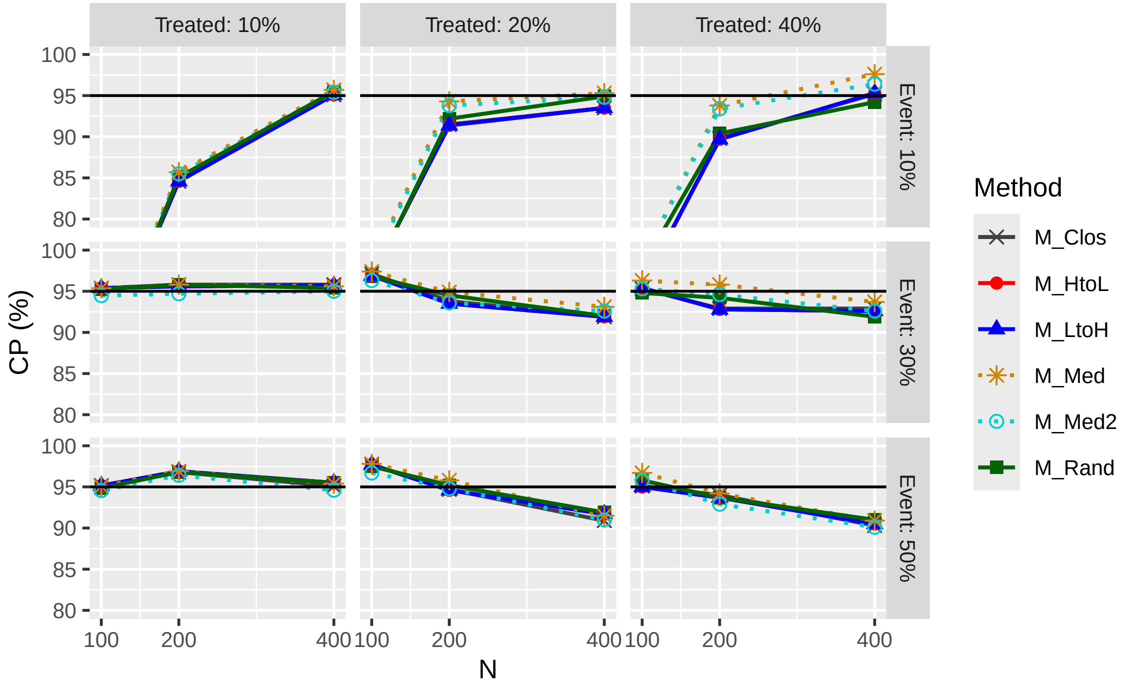

Figure S676. Coverage probability of confidence interval for OR (categorical covariate, matching ratio 1:2, true OR: 0.5, c statistic: 0.85, robust inference).

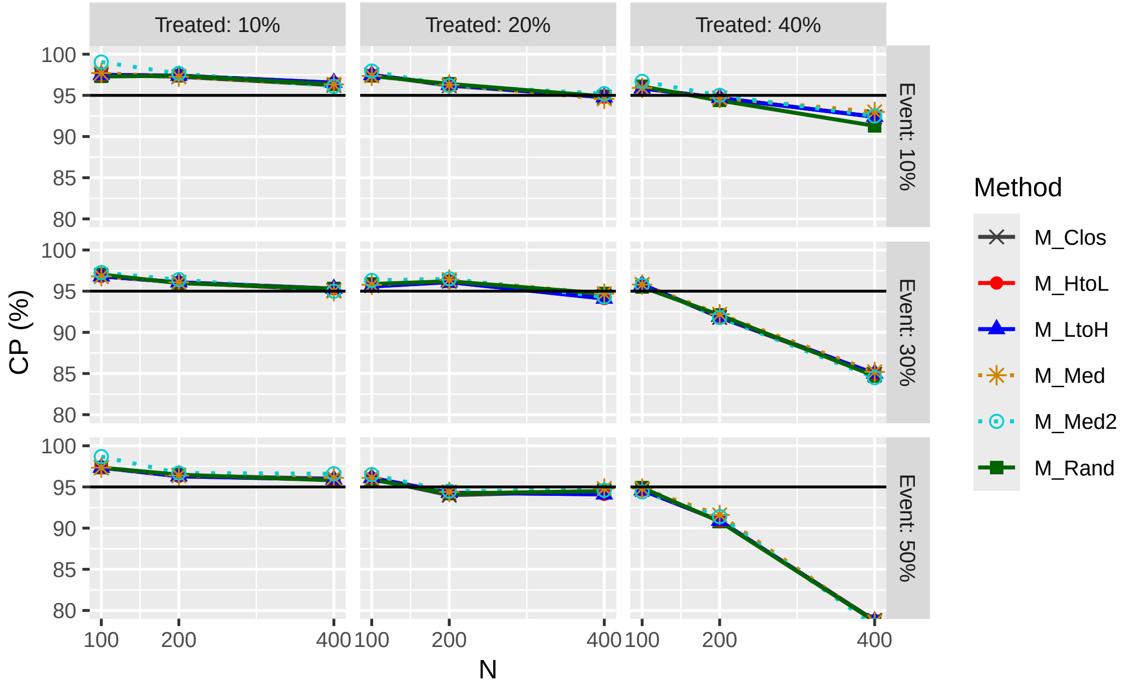

Figure S677. Coverage probability of confidence interval for OR (categorical covariate, matching ratio 1:2, true OR: 0.5, c statistic: 0.6, naive inference).

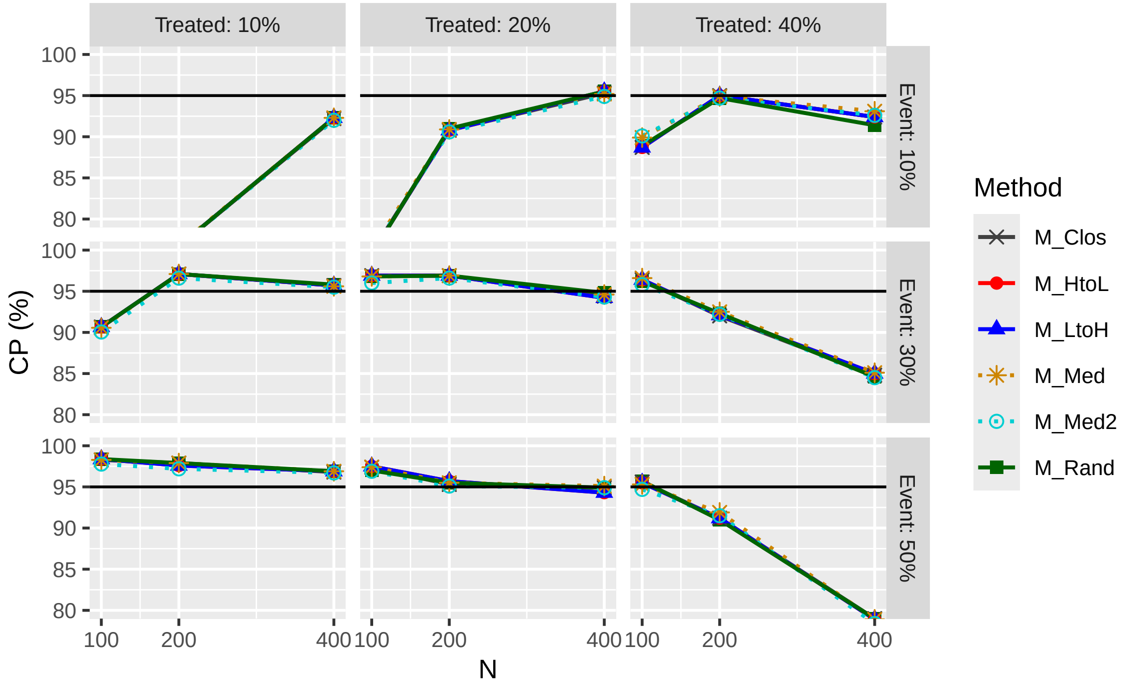

Figure S678. Coverage probability of confidence interval for OR (categorical covariate, matching ratio 1:2, true OR: 0.5, c statistic: 0.6, robust inference).

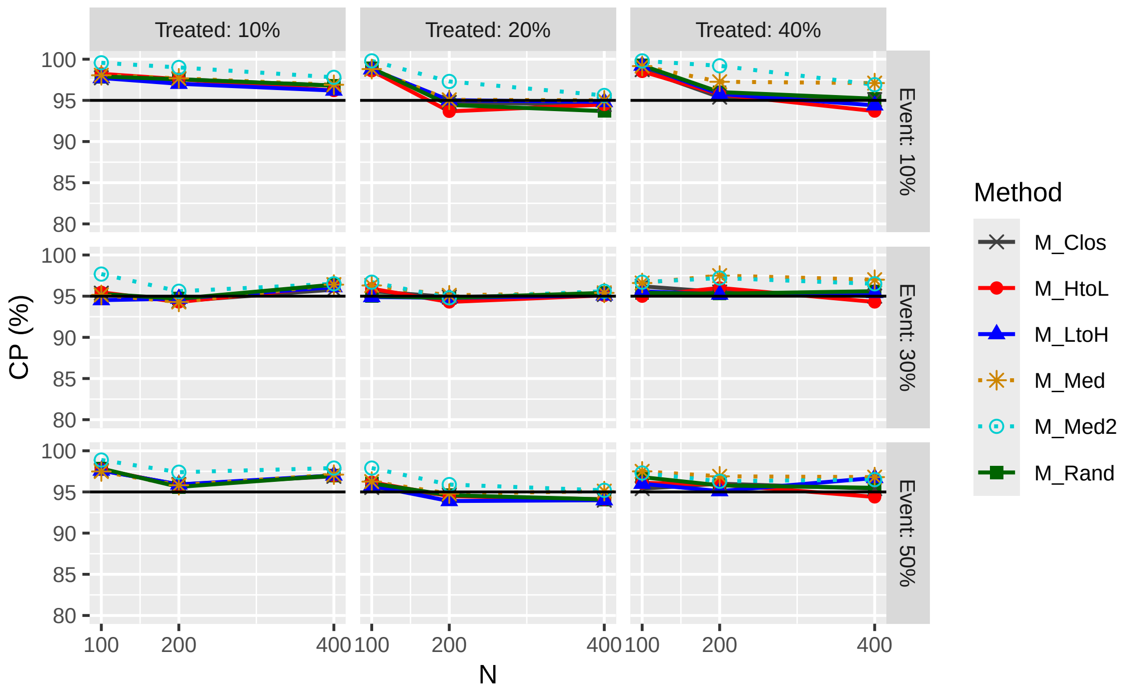

Figure S679. Coverage probability of confidence interval for OR (multimodal continuous covariate, matching ratio 1:1, true OR: 1, c statistic: 0.85, naive inference).

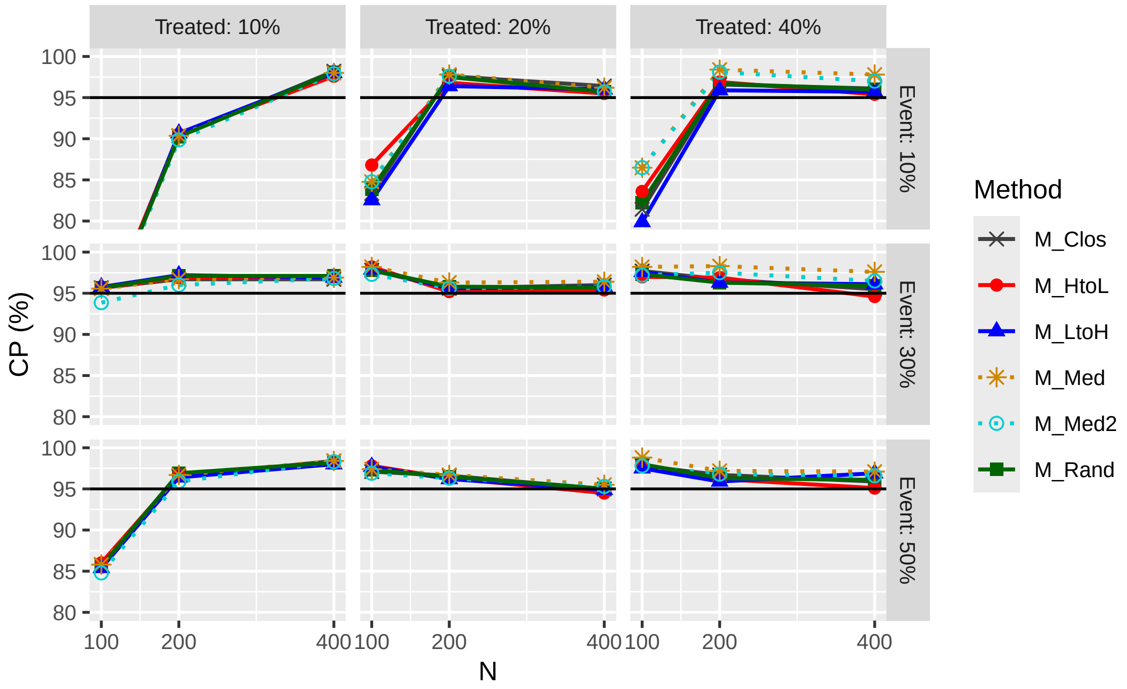

Figure S680. Coverage probability of confidence interval for OR (multimodal continuous covariate, matching ratio 1:1, true OR: 1, c statistic: 0.85, robust inference).

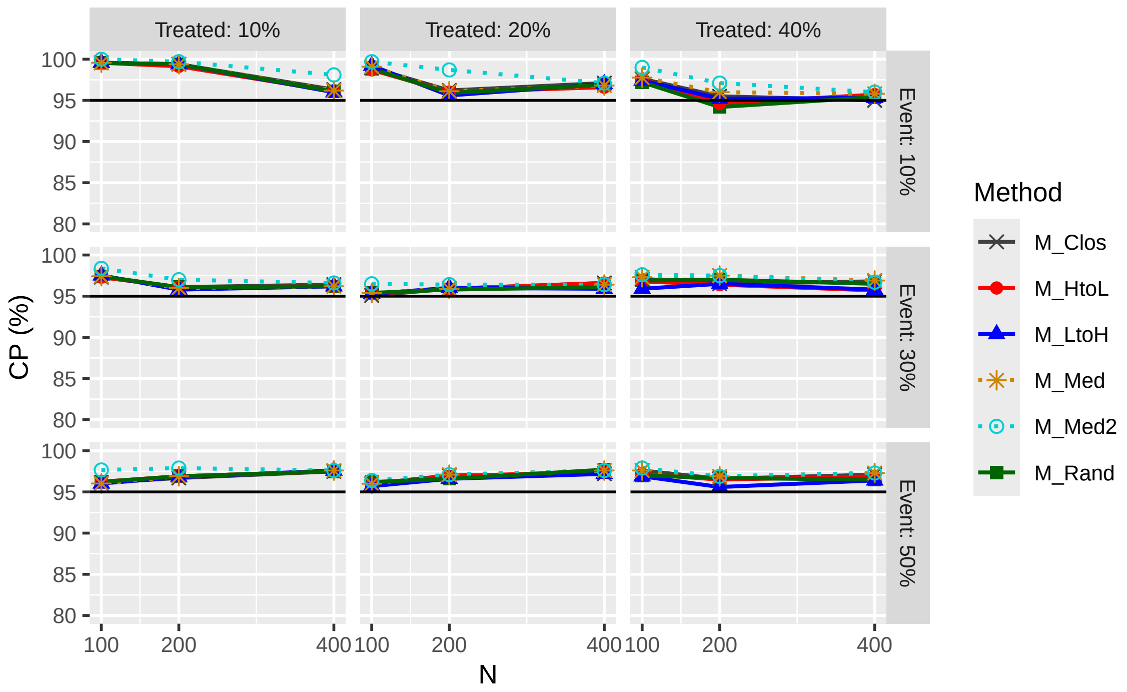

Figure S681. Coverage probability of confidence interval for OR (multimodal continuous covariate, matching ratio 1:1, true OR: 1, c statistic: 0.6, naive inference).

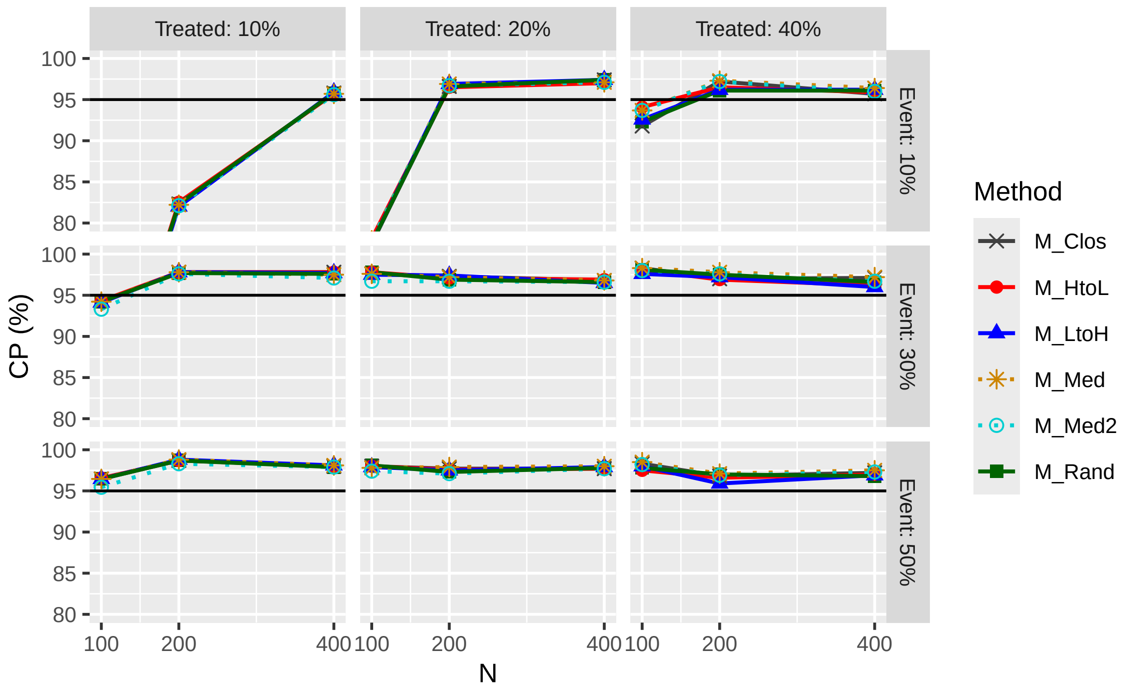

Figure S682. Coverage probability of confidence interval for OR (multimodal continuous covariate, matching ratio 1:1, true OR: 1, c statistic: 0.6, robust inference).

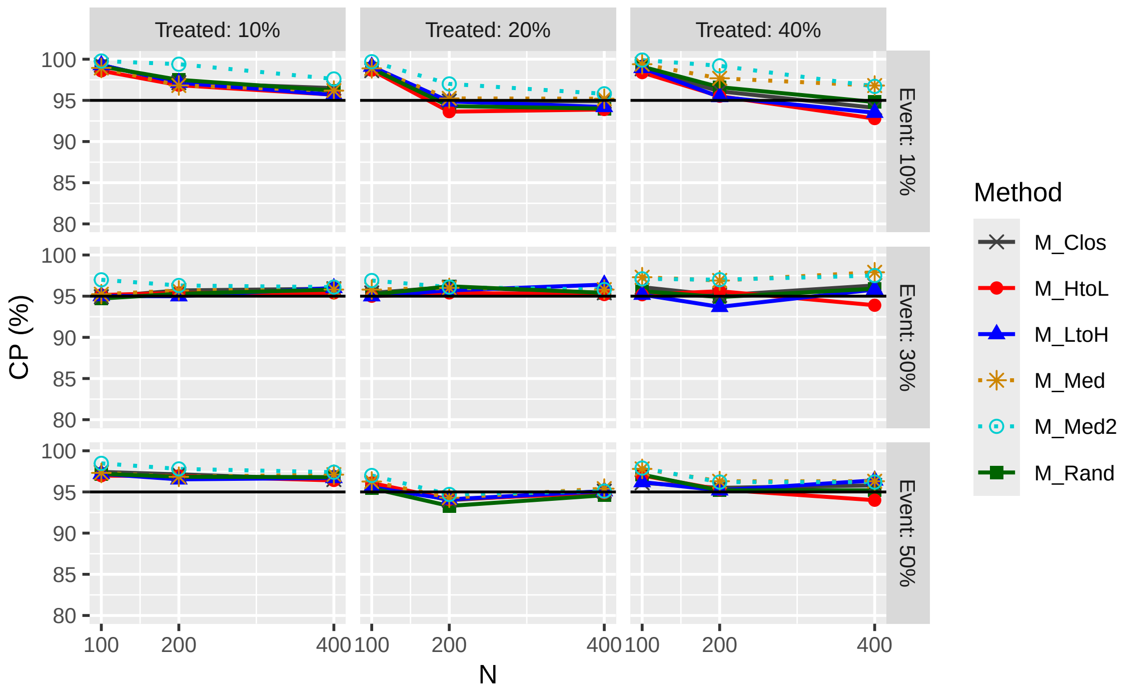

Figure S683. Coverage probability of confidence interval for OR (multimodal continuous covariate, matching ratio 1:1, true OR: 0.75, c statistic: 0.85, naive inference).

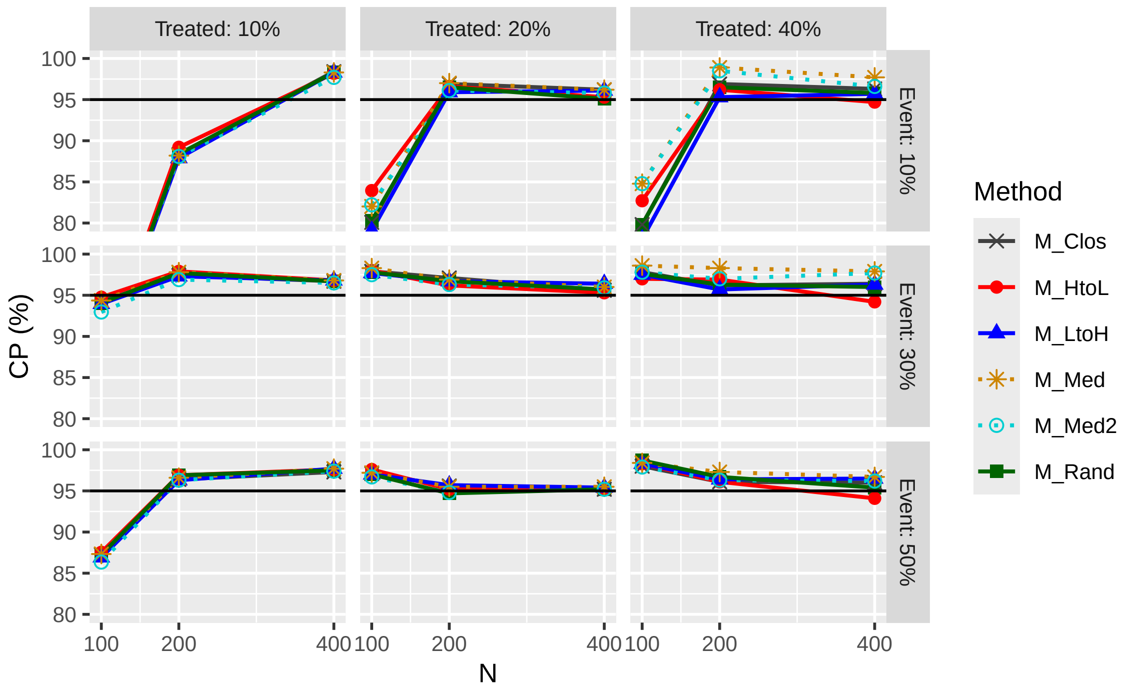

Figure S684. Coverage probability of confidence interval for OR (multimodal continuous covariate, matching ratio 1:1, true OR: 0.75, c statistic: 0.85, robust inference).

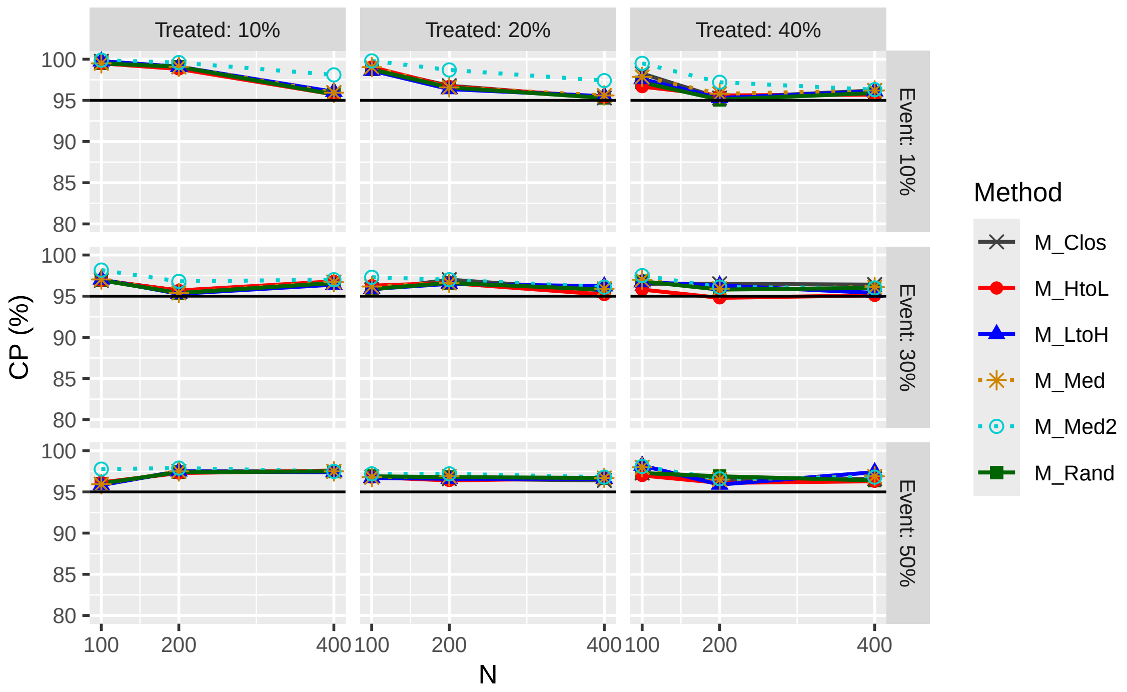

Figure S685. Coverage probability of confidence interval for OR (multimodal continuous covariate, matching ratio 1:1, true OR: 0.75, c statistic: 0.6, naive inference).

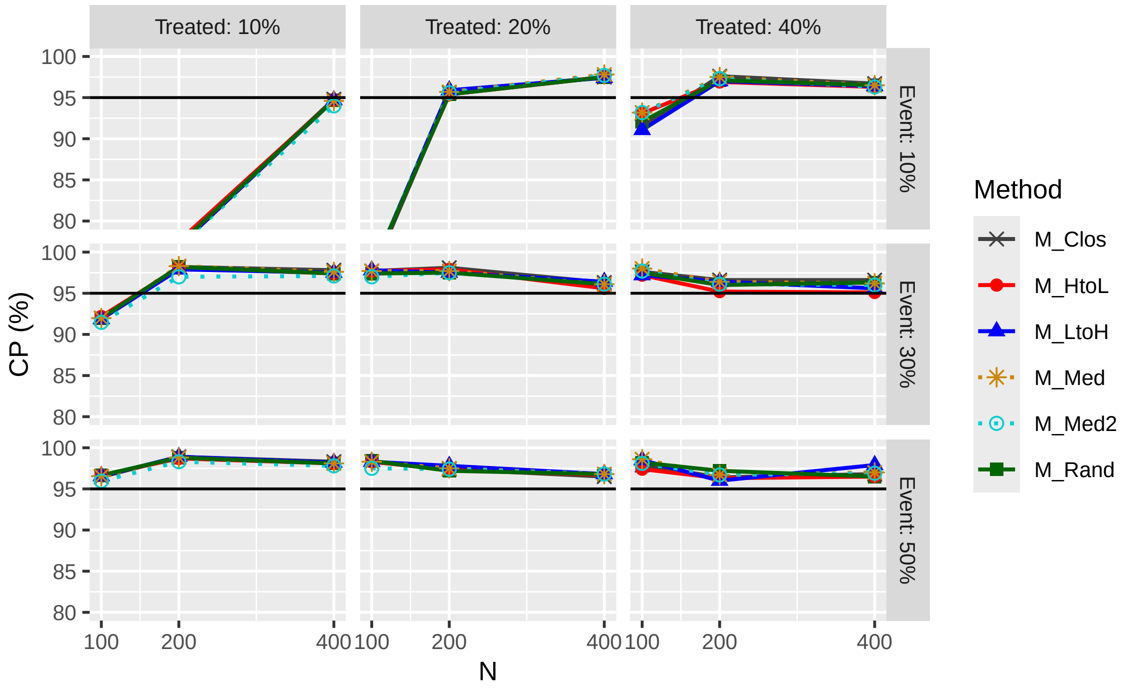

Figure S686. Coverage probability of confidence interval for OR (multimodal continuous covariate, matching ratio 1:1, true OR: 0.75, c statistic: 0.6, robust inference).

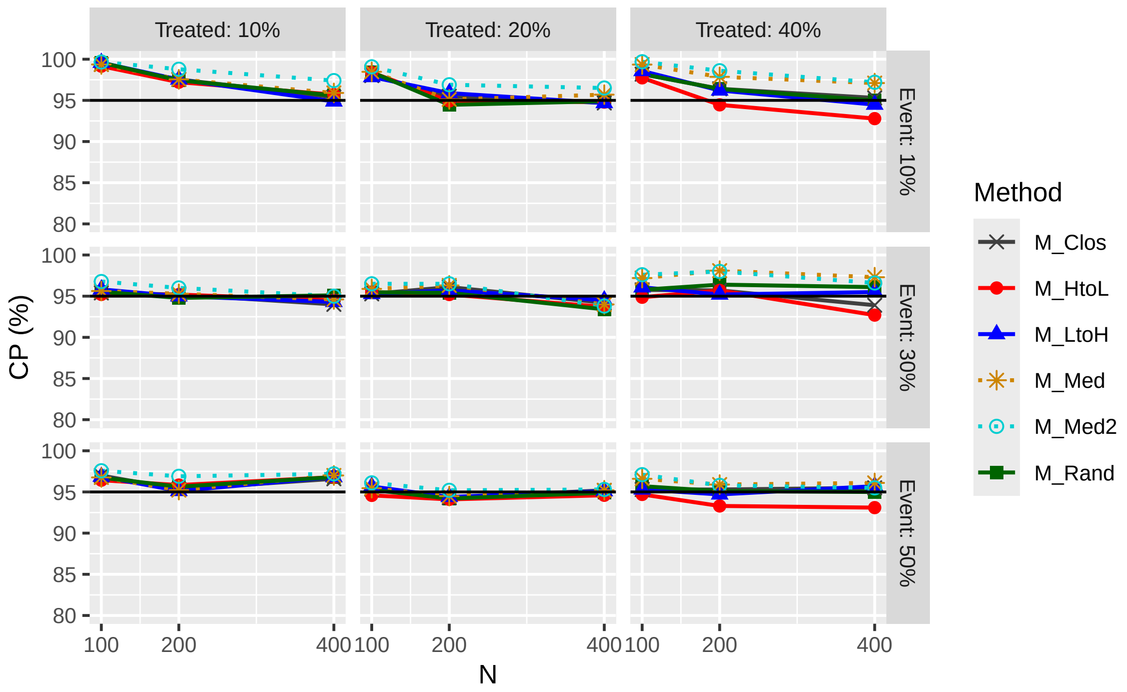

Figure S687. Coverage probability of confidence interval for OR (multimodal continuous covariate, matching ratio 1:1, true OR: 0.5, c statistic: 0.85, naive inference).

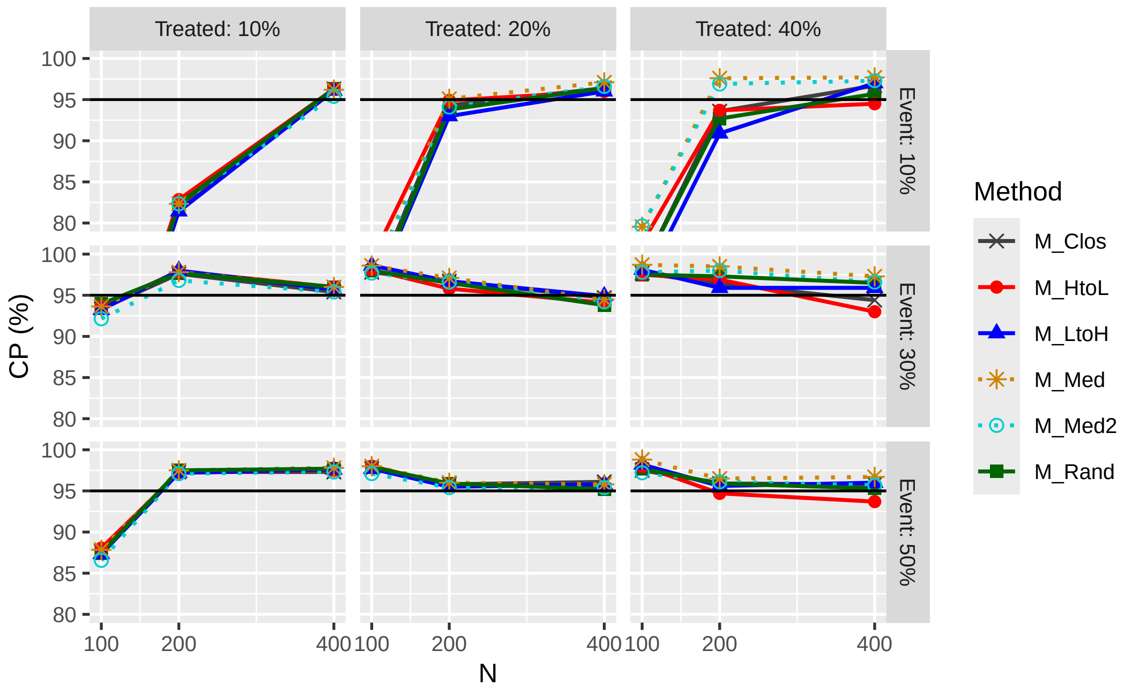

Figure S688. Coverage probability of confidence interval for OR (multimodal continuous covariate, matching ratio 1:1, true OR: 0.5, c statistic: 0.85, robust inference).

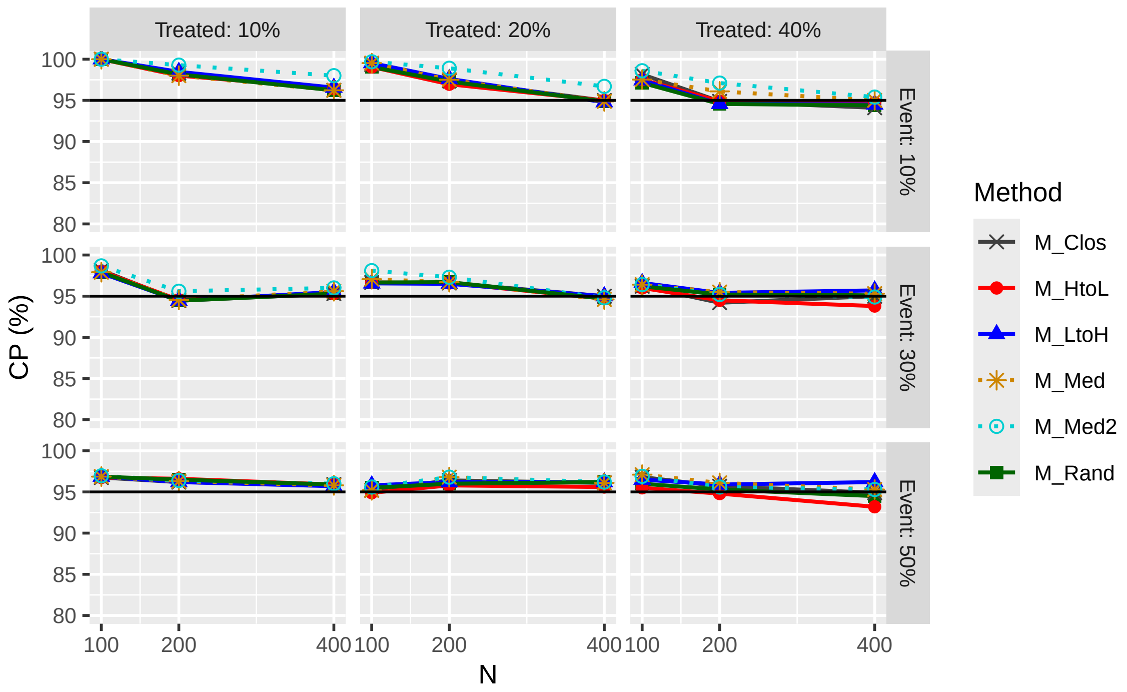

Figure S689. Coverage probability of confidence interval for OR (multimodal continuous covariate, matching ratio 1:1, true OR: 0.5, c statistic: 0.6, naive inference).

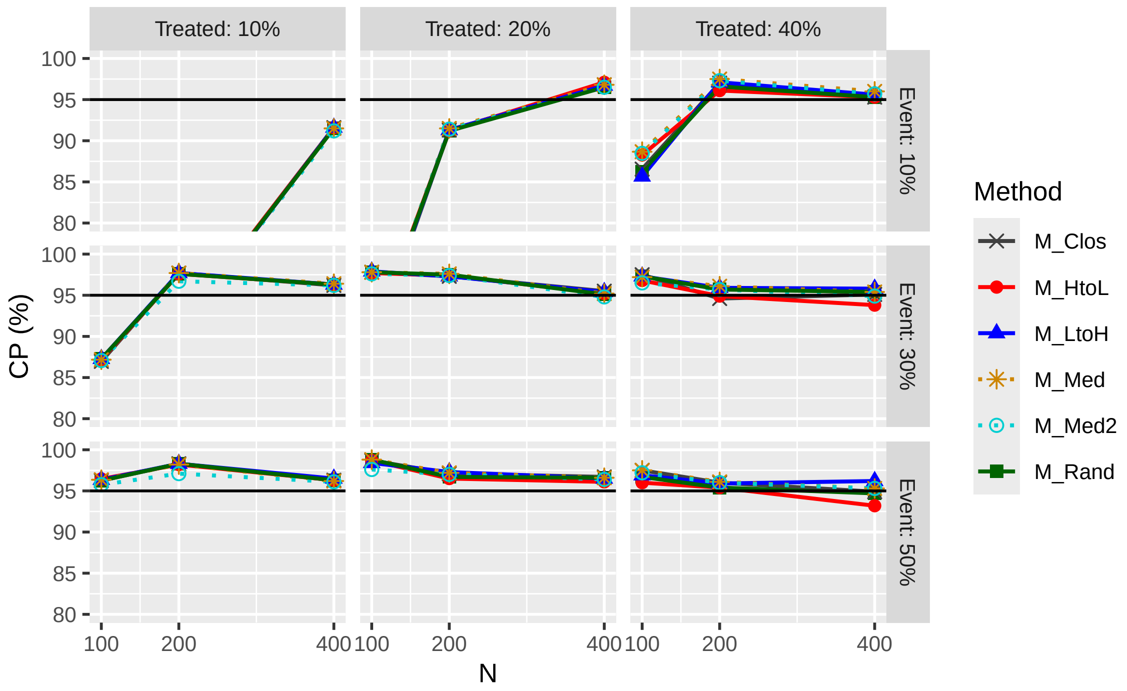

Figure S690. Coverage probability of confidence interval for OR (multimodal continuous covariate, matching ratio 1:1, true OR: 0.5, c statistic: 0.6, robust inference).

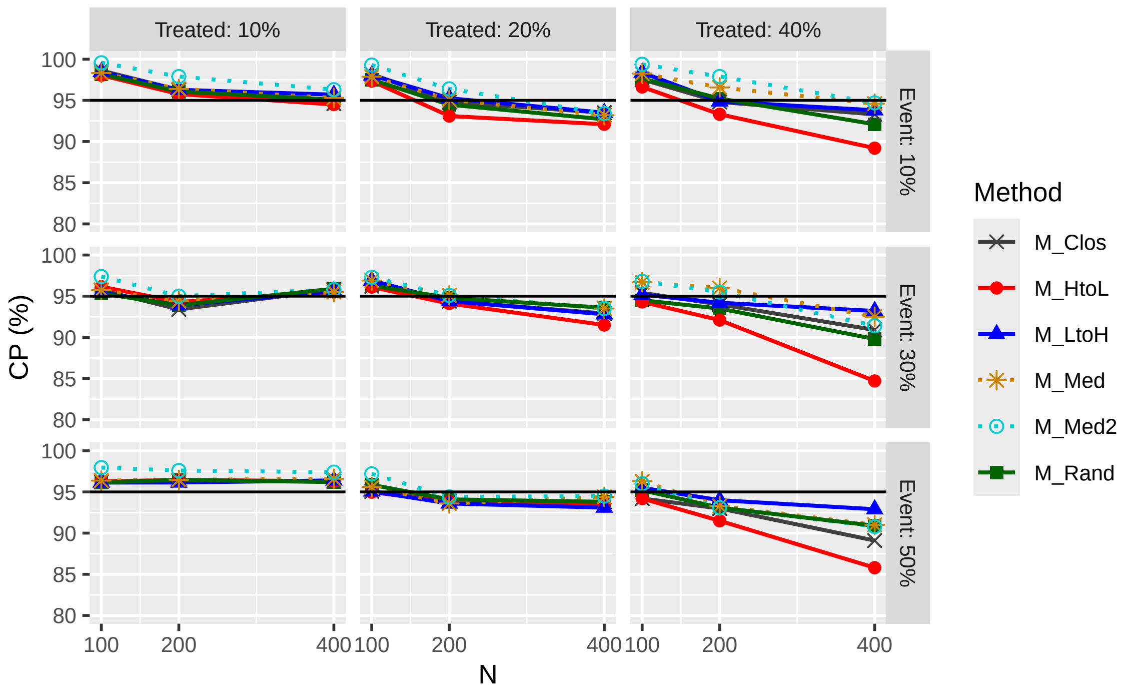

Figure S691. Coverage probability of confidence interval for OR (multimodal continuous covariate, matching ratio 1:2, true OR: 1, c statistic: 0.85, naive inference).

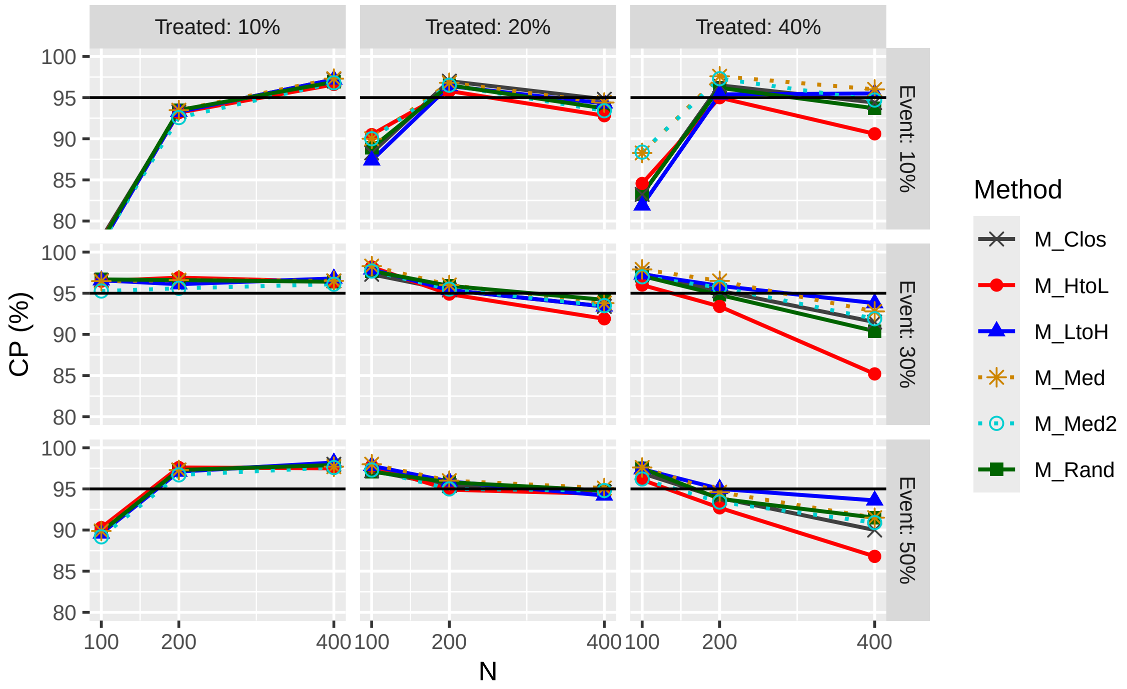

Figure S692. Coverage probability of confidence interval for OR (multimodal continuous covariate, matching ratio 1:2, true OR: 1, c statistic: 0.85, robust inference).

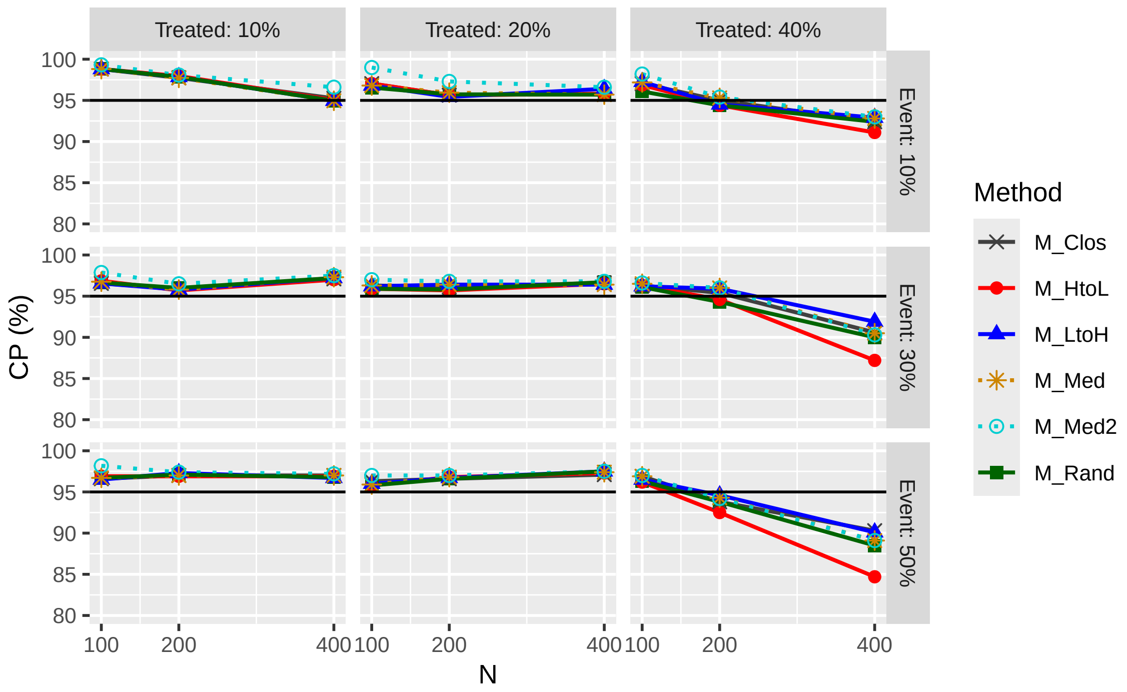

Figure S693. Coverage probability of confidence interval for OR (multimodal continuous covariate, matching ratio 1:2, true OR: 1, c statistic: 0.6, naive inference).

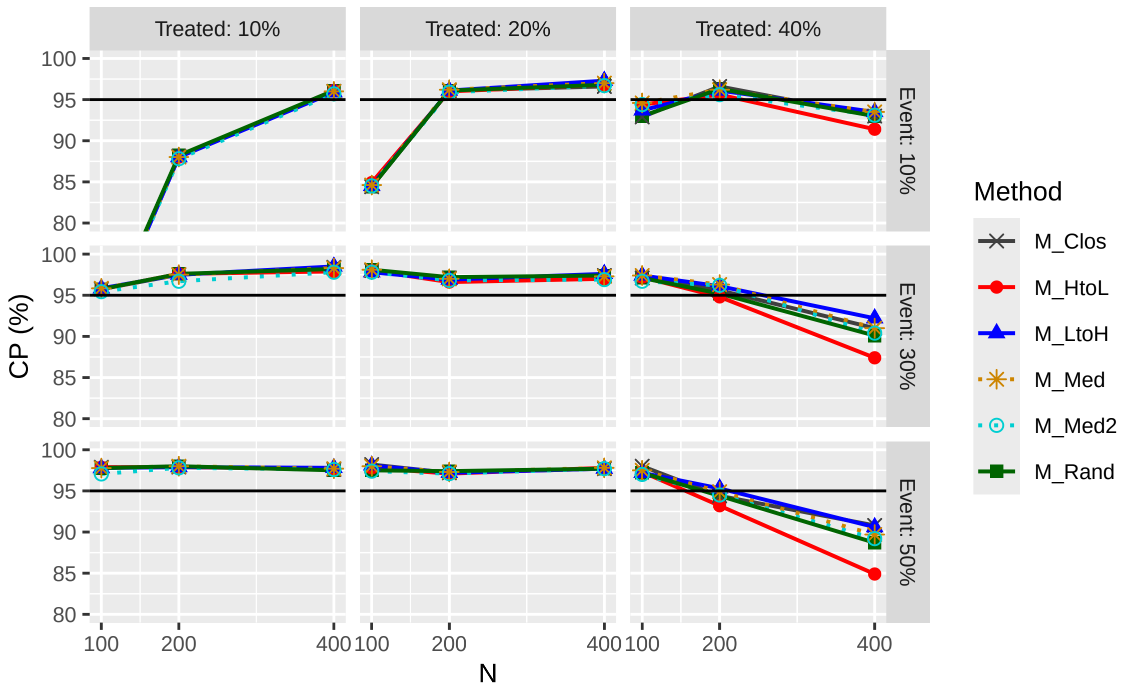

Figure S694. Coverage probability of confidence interval for OR (multimodal continuous covariate, matching ratio 1:2, true OR: 1, c statistic: 0.6, robust inference).

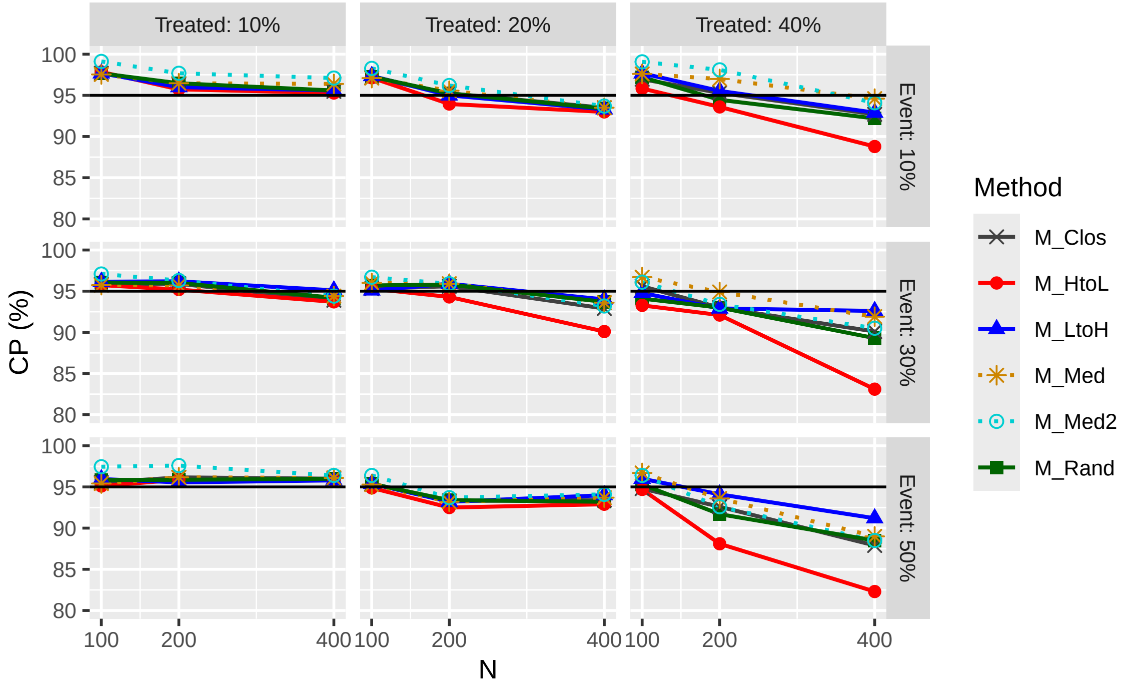

Figure S695. Coverage probability of confidence interval for OR (multimodal continuous covariate, matching ratio 1:2, true OR: 0.75, c statistic: 0.85, naive inference).

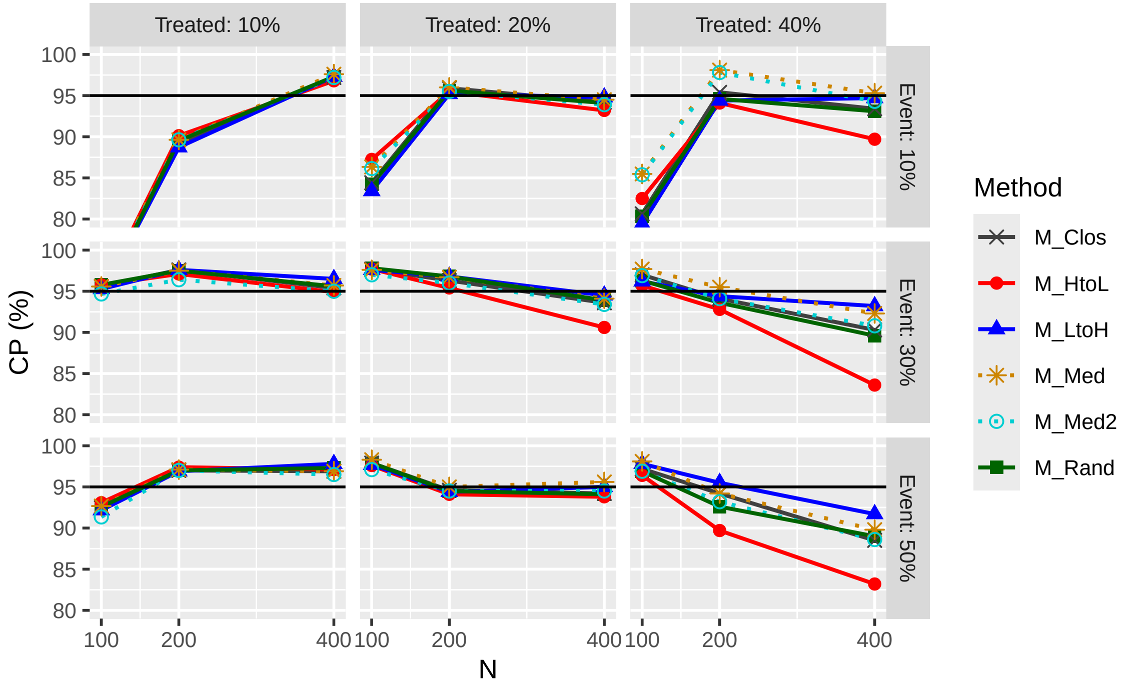

Figure S696. Coverage probability of confidence interval for OR (multimodal continuous covariate, matching ratio 1:2, true OR: 0.75, c statistic: 0.85, robust inference).

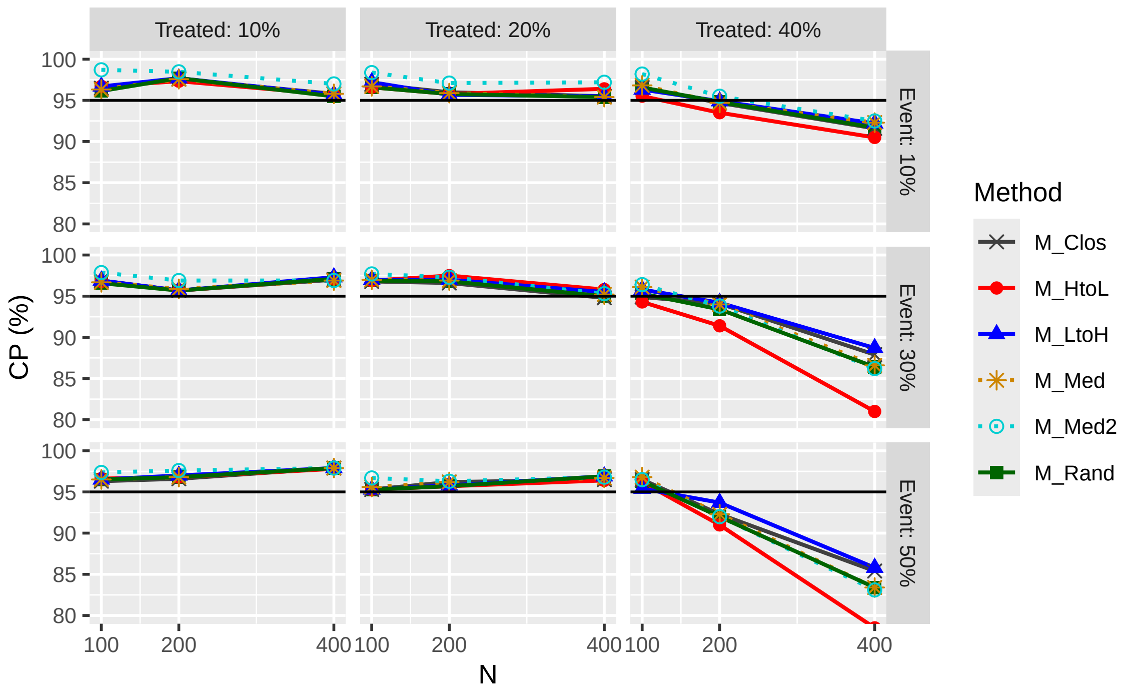

Figure S697. Coverage probability of confidence interval for OR (multimodal continuous covariate, matching ratio 1:2, true OR: 0.75, c statistic: 0.6, naive inference).

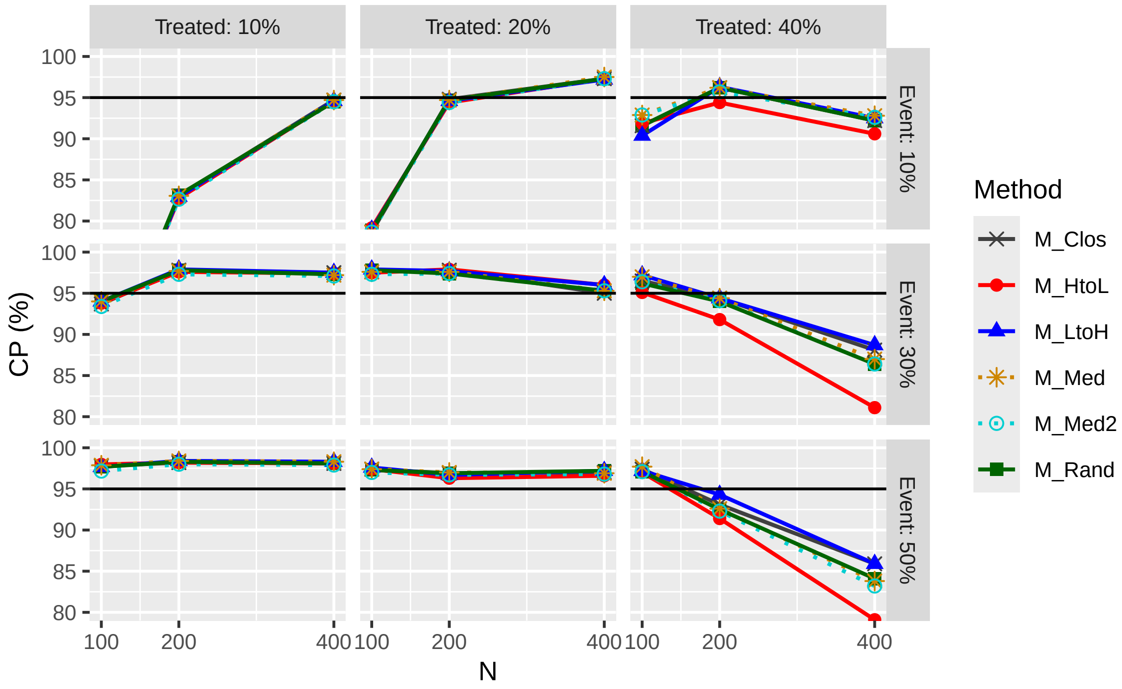

Figure S698. Coverage probability of confidence interval for OR (multimodal continuous covariate, matching ratio 1:2, true OR: 0.75, c statistic: 0.6, robust inference).

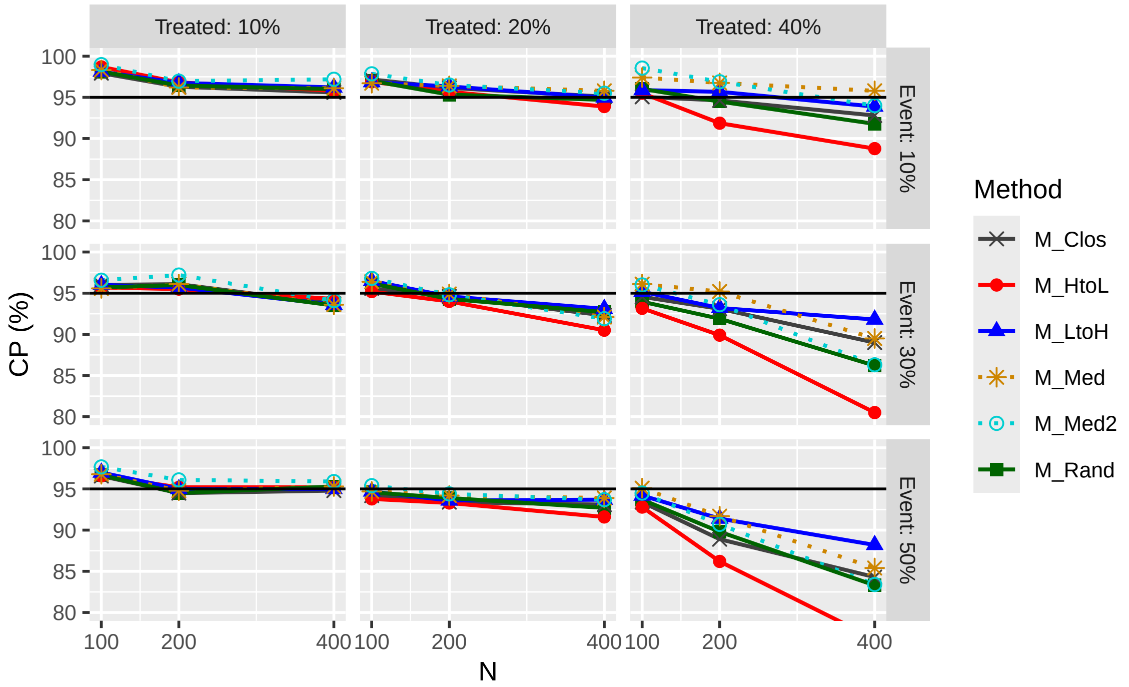

Figure S699. Coverage probability of confidence interval for OR (multimodal continuous covariate, matching ratio 1:2, true OR: 0.5, c statistic: 0.85, naive inference).

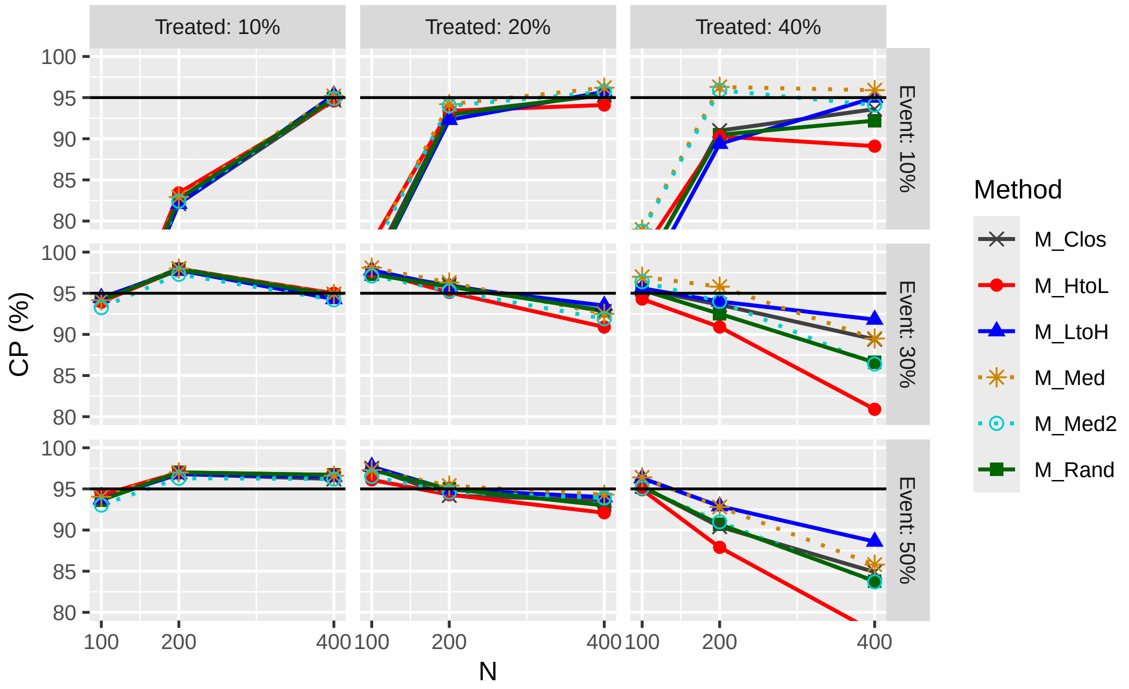

Figure S700. Coverage probability of confidence interval for OR (multimodal continuous covariate, matching ratio 1:2, true OR: 0.5, c statistic: 0.85, robust inference).

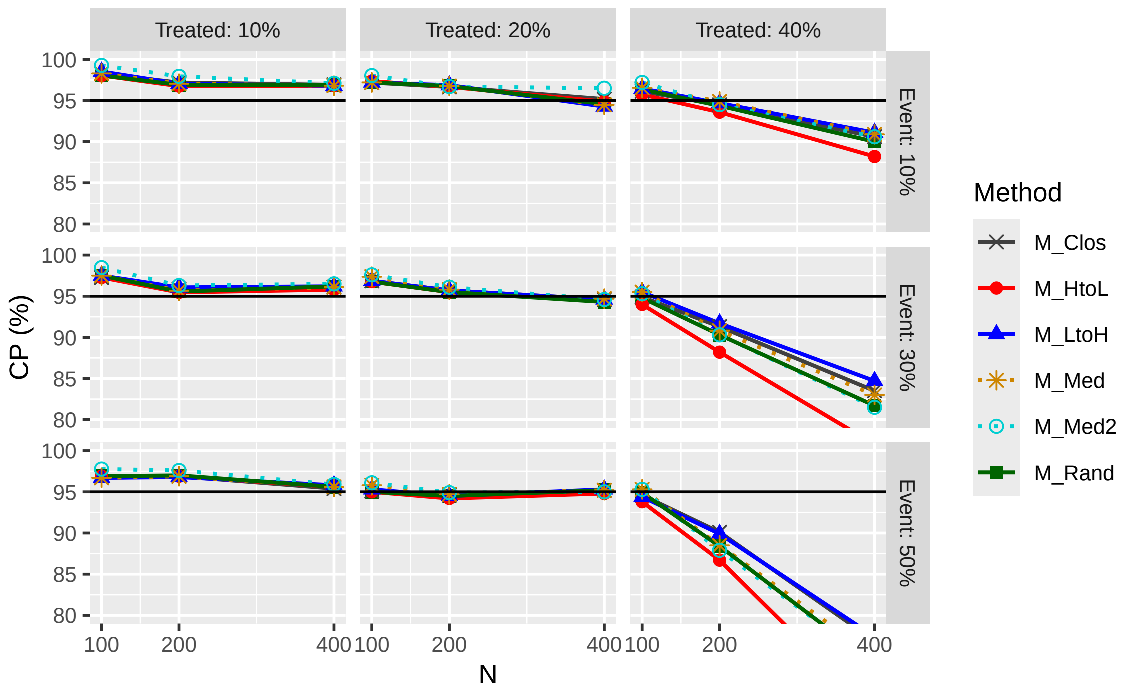

Figure S701. Coverage probability of confidence interval for OR (multimodal continuous covariate, matching ratio 1:2, true OR: 0.5, c statistic: 0.6, naive inference).

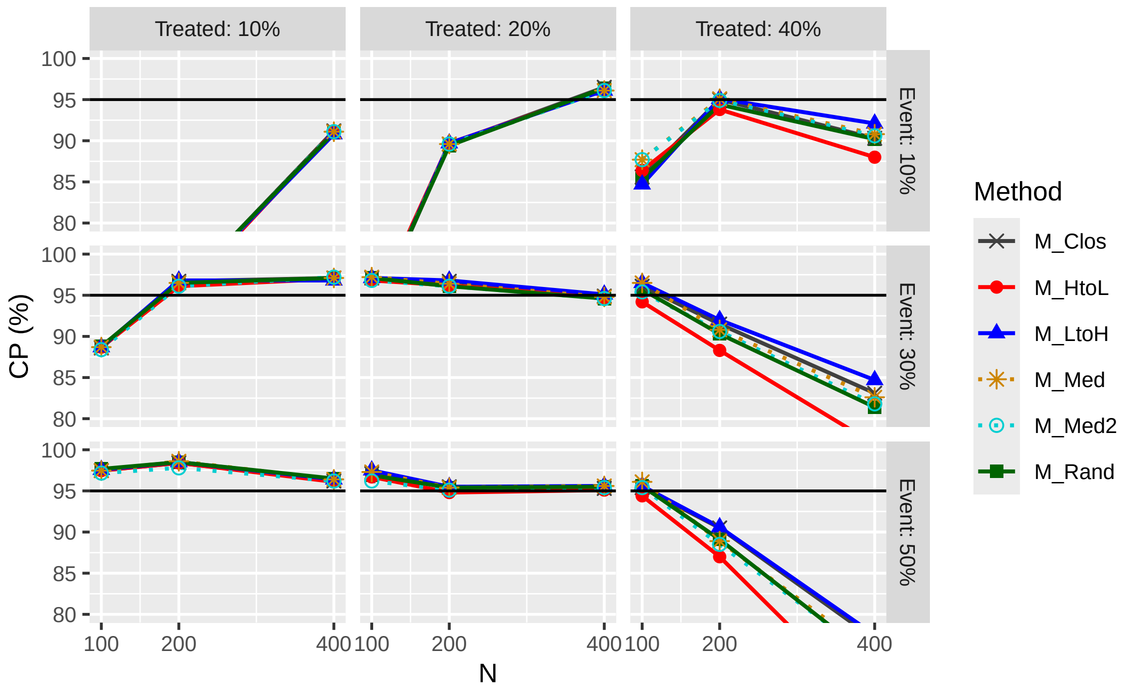

Figure S702. Coverage probability of confidence interval for OR (multimodal continuous covariate, matching ratio 1:2, true OR: 0.5, c statistic: 0.6, robust inference).

S12. Mean percentage bias of standard error for log odds ratio (caliper: 15%)

Only results with log odds ratios less than 10 were included.

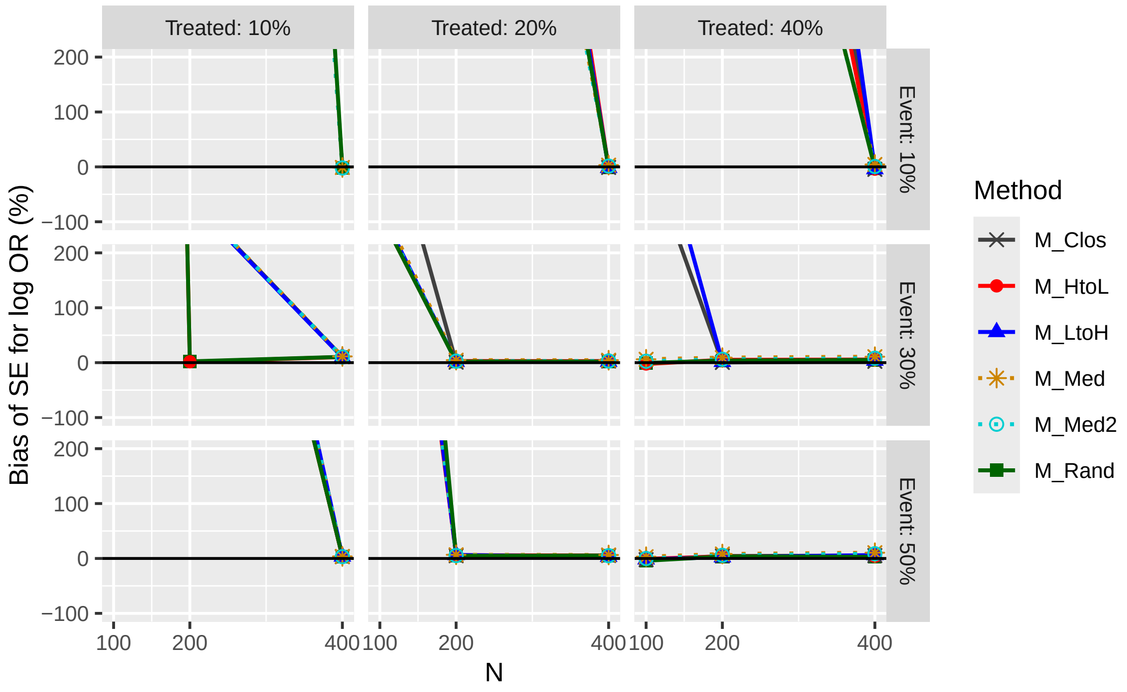

Figure S703. Mean bias of standard error for log odds ratio (unimodal continuous covariate, matching ratio 1:1, true OR: 1, c statistic: 0.85, naive inference).

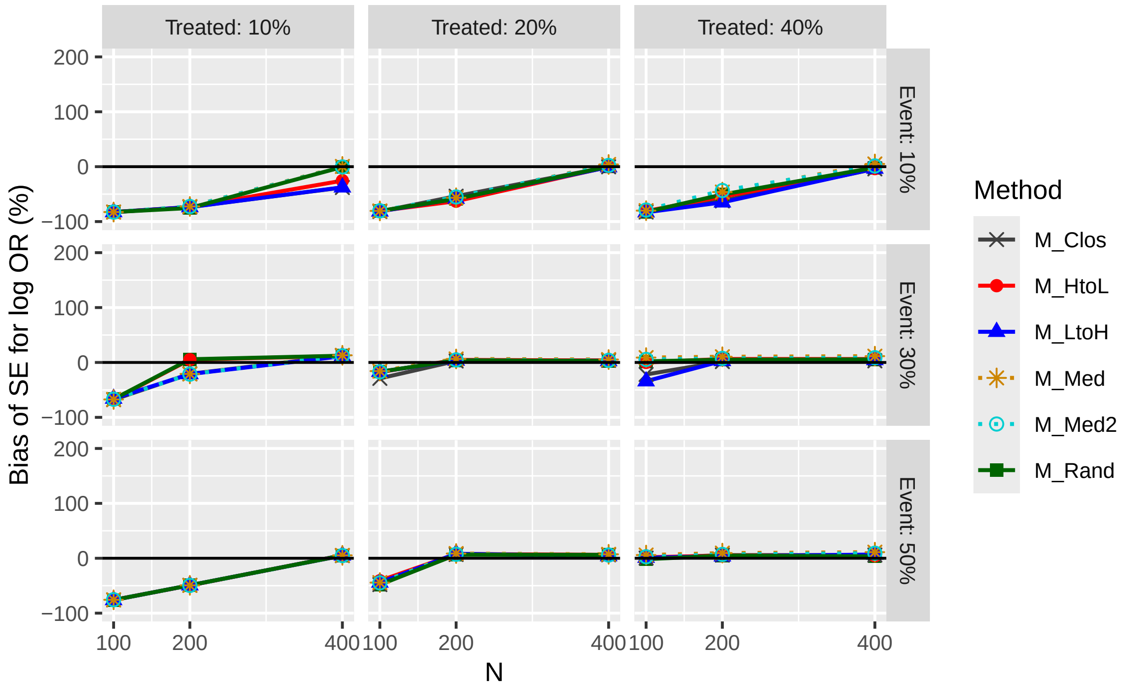

Figure S704. Mean bias of standard error for log odds ratio (unimodal continuous covariate, matching ratio 1:1, true OR: 1, c statistic: 0.85, robust inference).

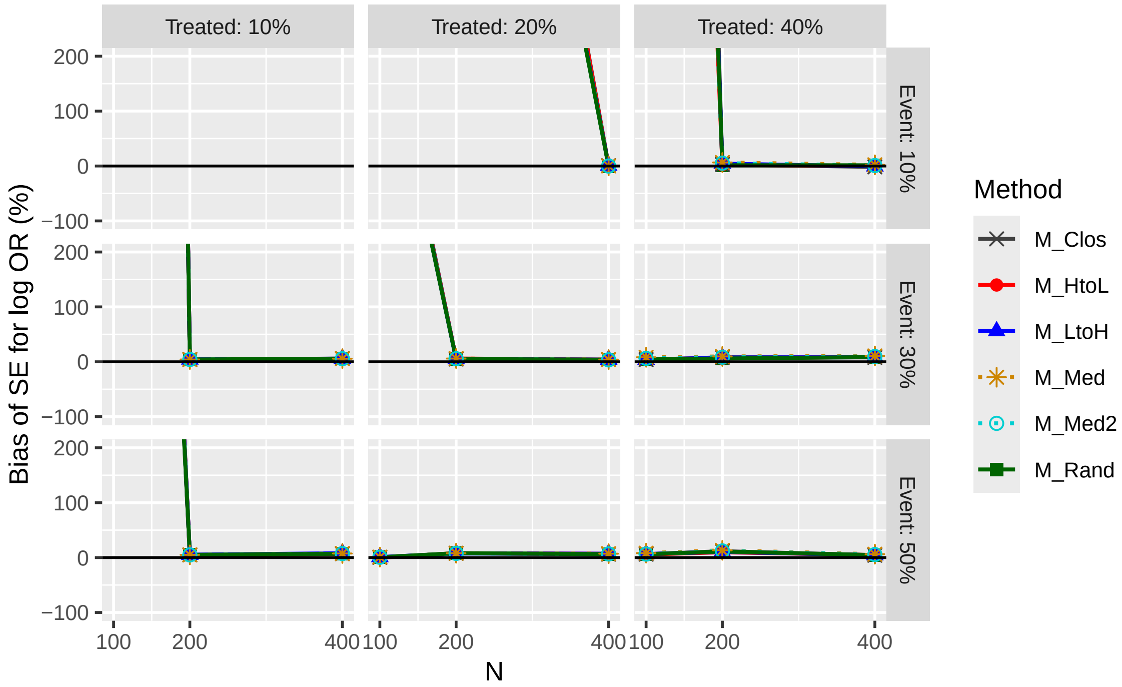

Figure S705. Mean bias of standard error for log odds ratio (unimodal continuous covariate, matching ratio 1:1, true OR: 1, c statistic: 0.6, naive inference).

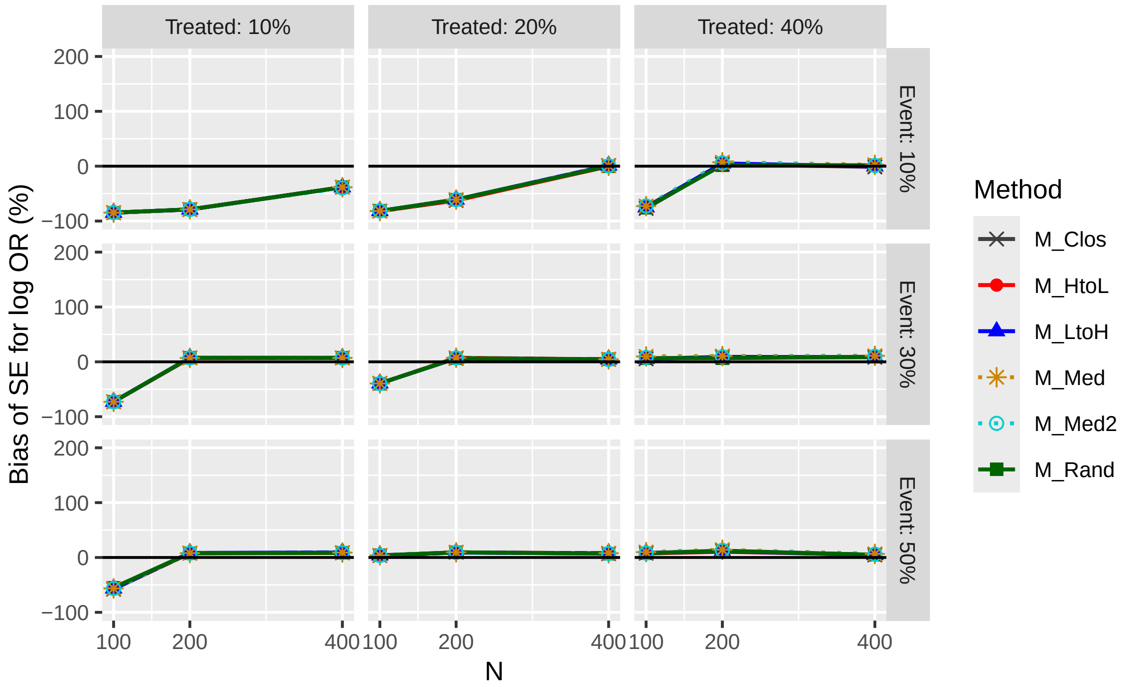

Figure S706. Mean bias of standard error for log odds ratio (unimodal continuous covariate, matching ratio 1:1, true OR: 1, c statistic: 0.6, robust inference).

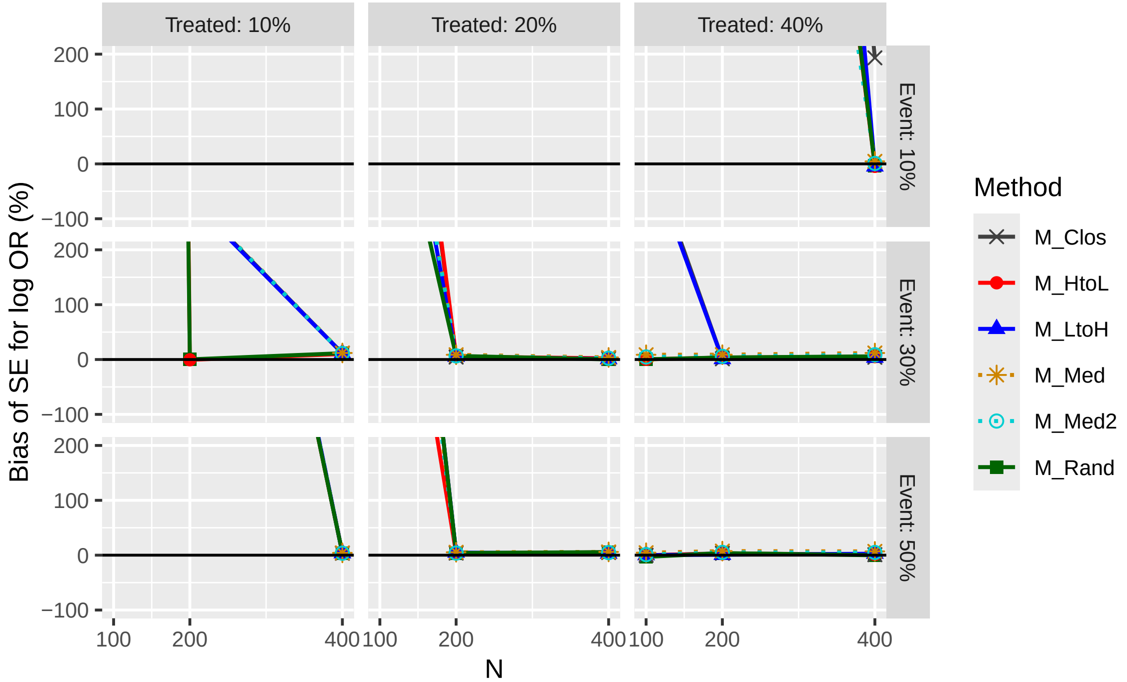

Figure S707. Mean bias of standard error for log odds ratio (unimodal continuous covariate, matching ratio 1:1, true OR: 0.75, c statistic: 0.85, naive inference).

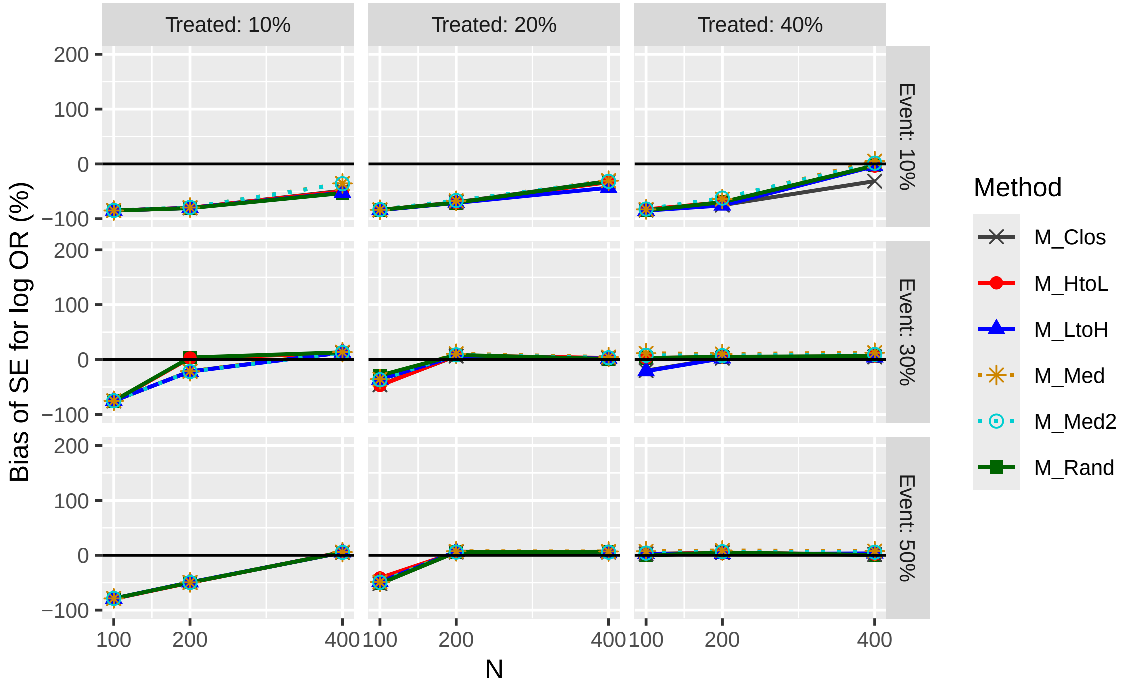

Figure S708. Mean bias of standard error for log odds ratio (unimodal continuous covariate, matching ratio 1:1, true OR: 0.75, c statistic: 0.85, robust inference).

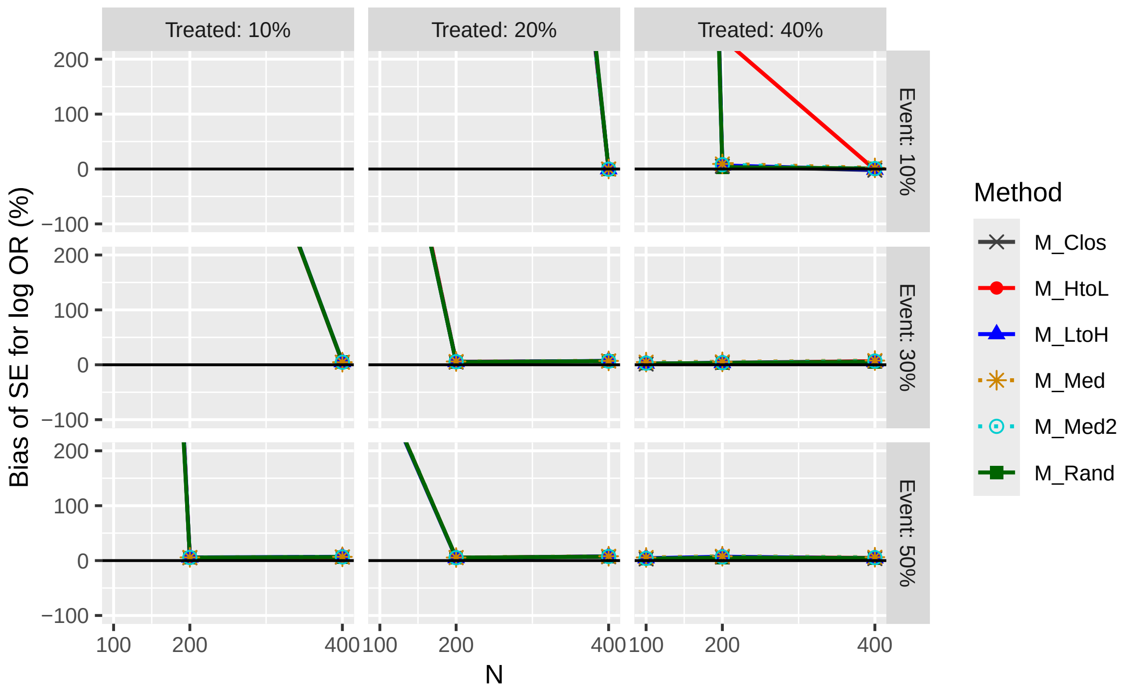

Figure S709. Mean bias of standard error for log odds ratio (unimodal continuous covariate, matching ratio 1:1, true OR: 0.75, c statistic: 0.6, naive inference).

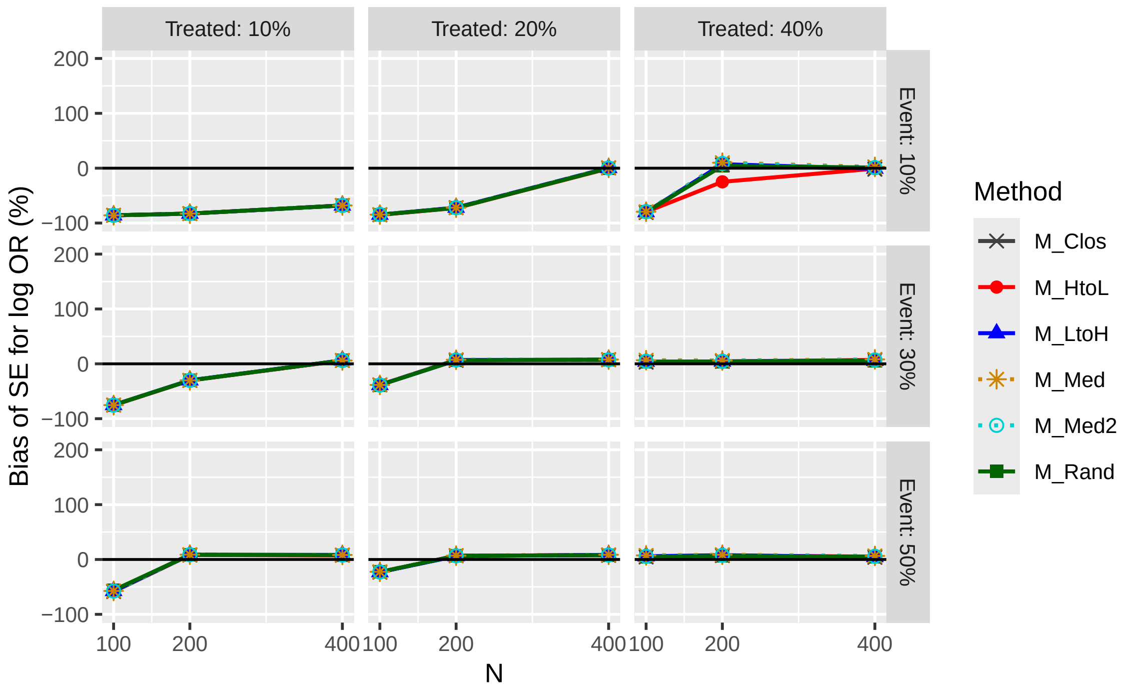

Figure S710. Mean bias of standard error for log odds ratio (unimodal continuous covariate, matching ratio 1:1, true OR: 0.75, c statistic: 0.6, robust inference).

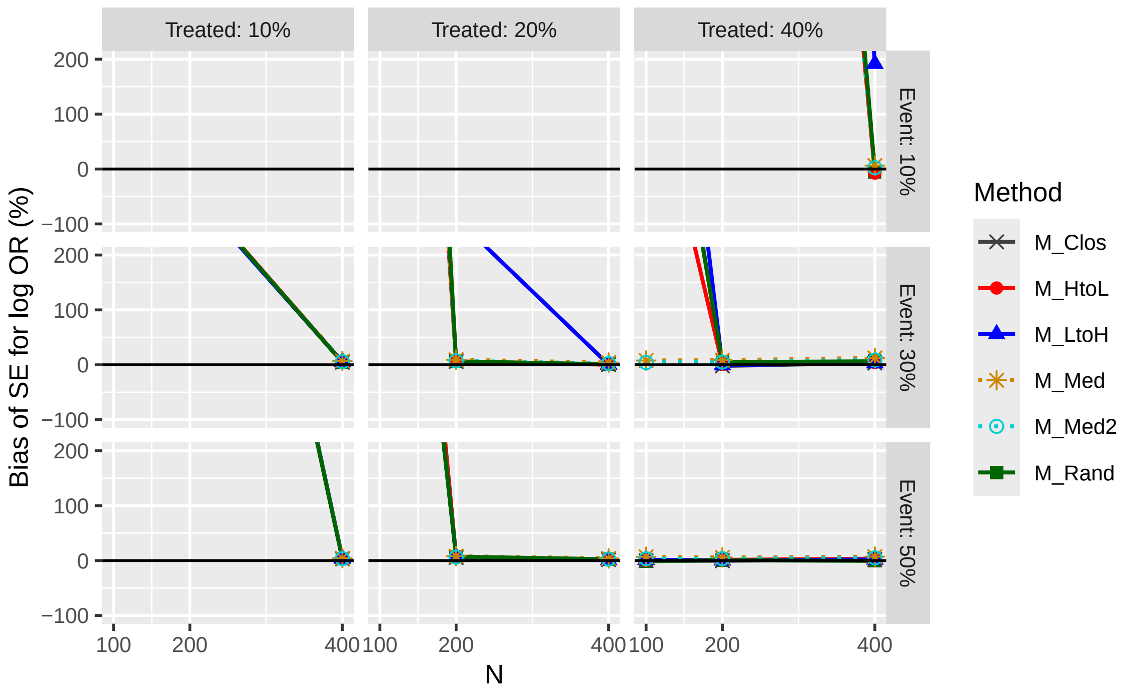

Figure S711. Mean bias of standard error for log odds ratio (unimodal continuous covariate, matching ratio 1:1, true OR: 0.5, c statistic: 0.85, naive inference).

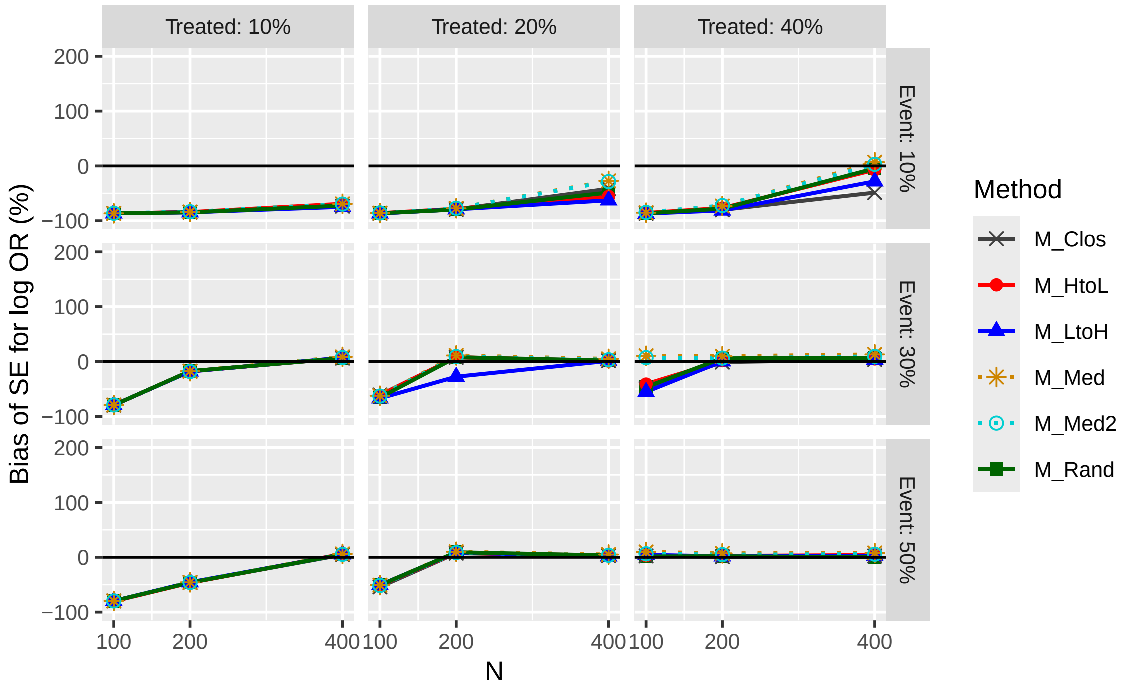

Figure S712. Mean bias of standard error for log odds ratio (unimodal continuous covariate, matching ratio 1:1, true OR: 0.5, c statistic: 0.85, robust inference).

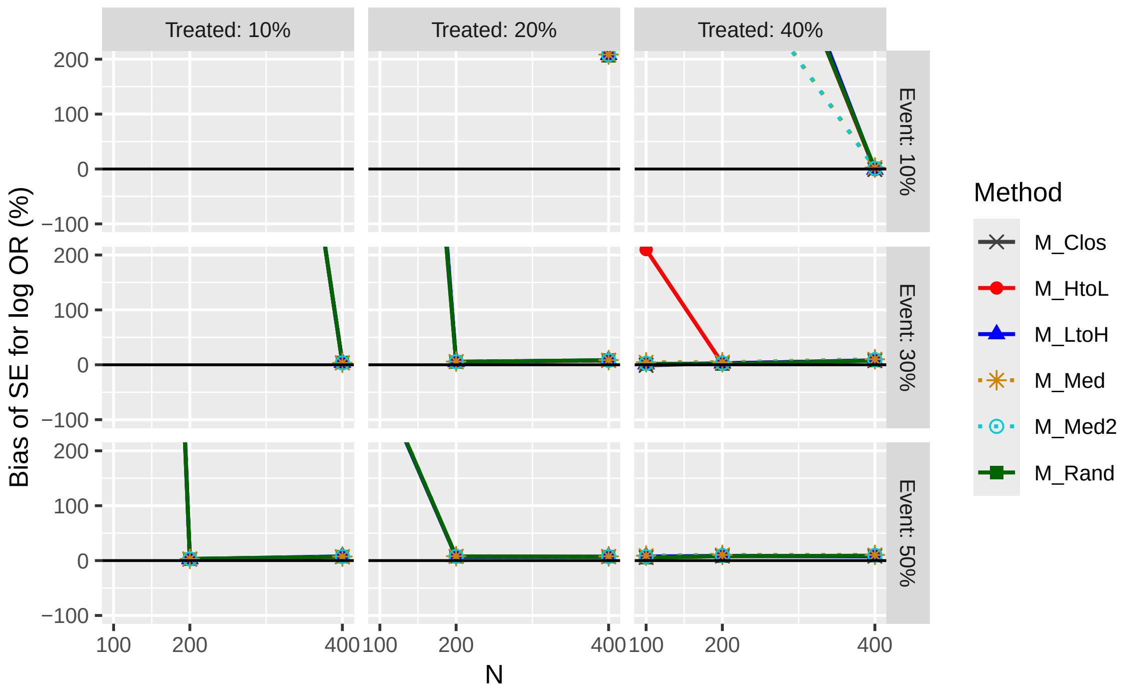

Figure S713. Mean bias of standard error for log odds ratio (unimodal continuous covariate, matching ratio 1:1, true OR: 0.5, c statistic: 0.6, naive inference).

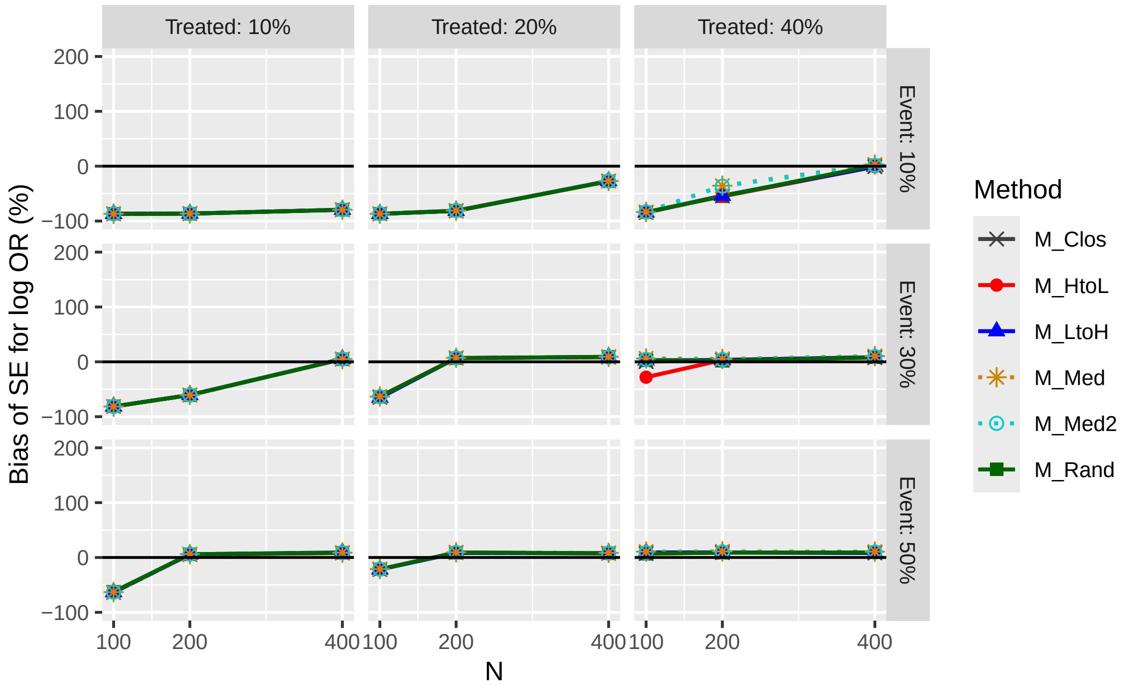

Figure S714. Mean bias of standard error for log odds ratio (unimodal continuous covariate, matching ratio 1:1, true OR: 0.5, c statistic: 0.6, robust inference).

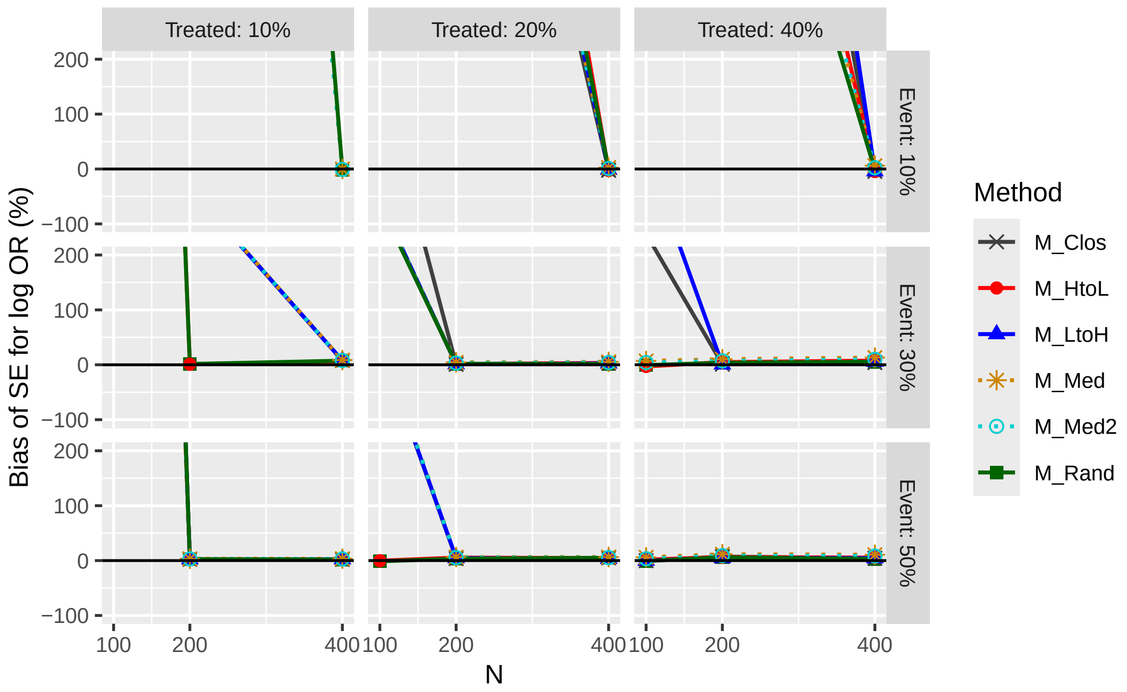

Figure S715. Mean bias of standard error for log odds ratio (unimodal continuous covariate, matching ratio 1:2, true OR: 1, c statistic: 0.85, naive inference).

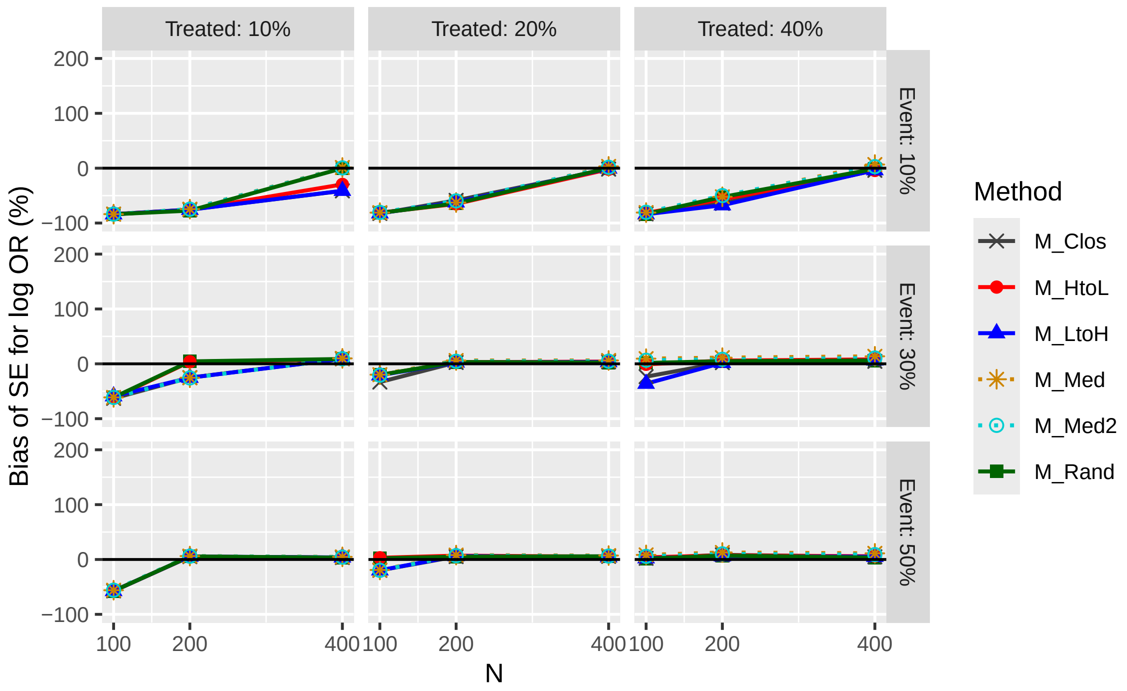

Figure S716. Mean bias of standard error for log odds ratio (unimodal continuous covariate, matching ratio 1:2, true OR: 1, c statistic: 0.85, robust inference).

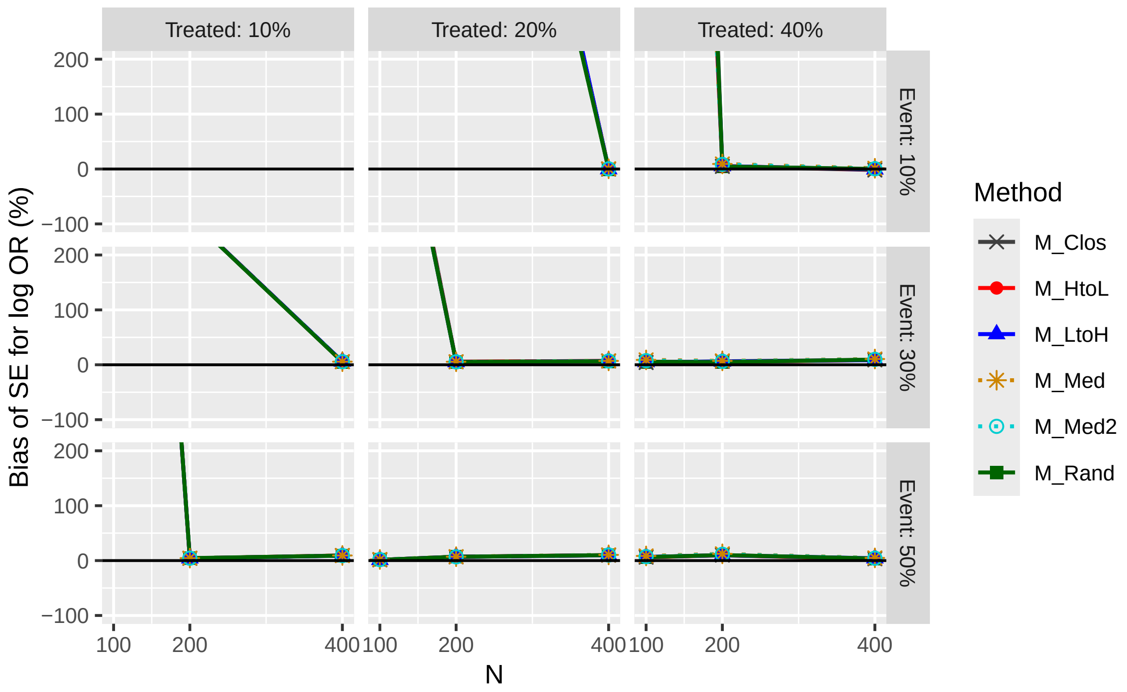

Figure S717. Mean bias of standard error for log odds ratio (unimodal continuous covariate, matching ratio 1:2, true OR: 1, c statistic: 0.6, naive inference).

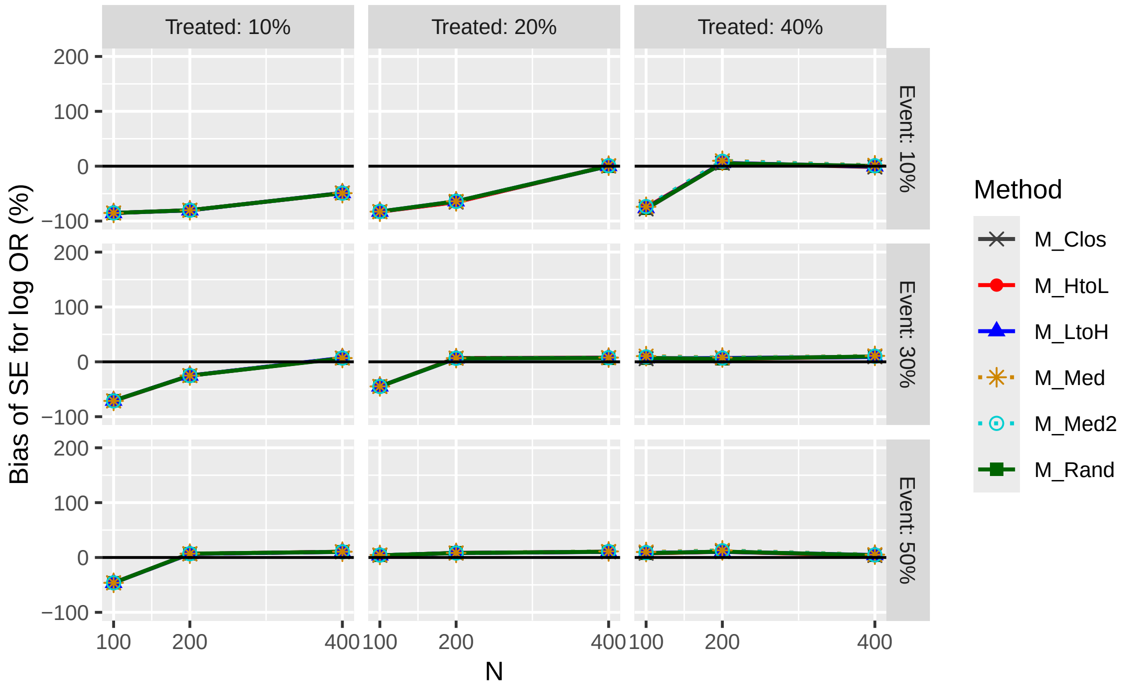

Figure S718. Mean bias of standard error for log odds ratio (unimodal continuous covariate, matching ratio 1:2, true OR: 1, c statistic: 0.6, robust inference).

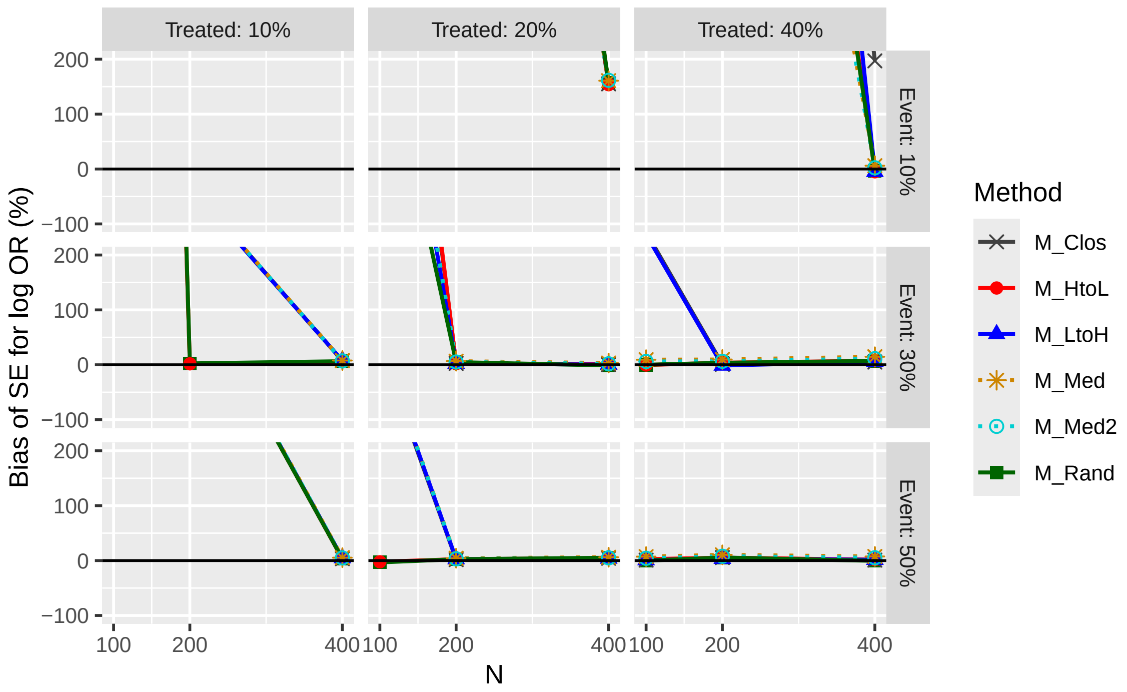

Figure S719. Mean bias of standard error for log odds ratio (unimodal continuous covariate, matching ratio 1:2, true OR: 0.75, c statistic: 0.85, naive inference).

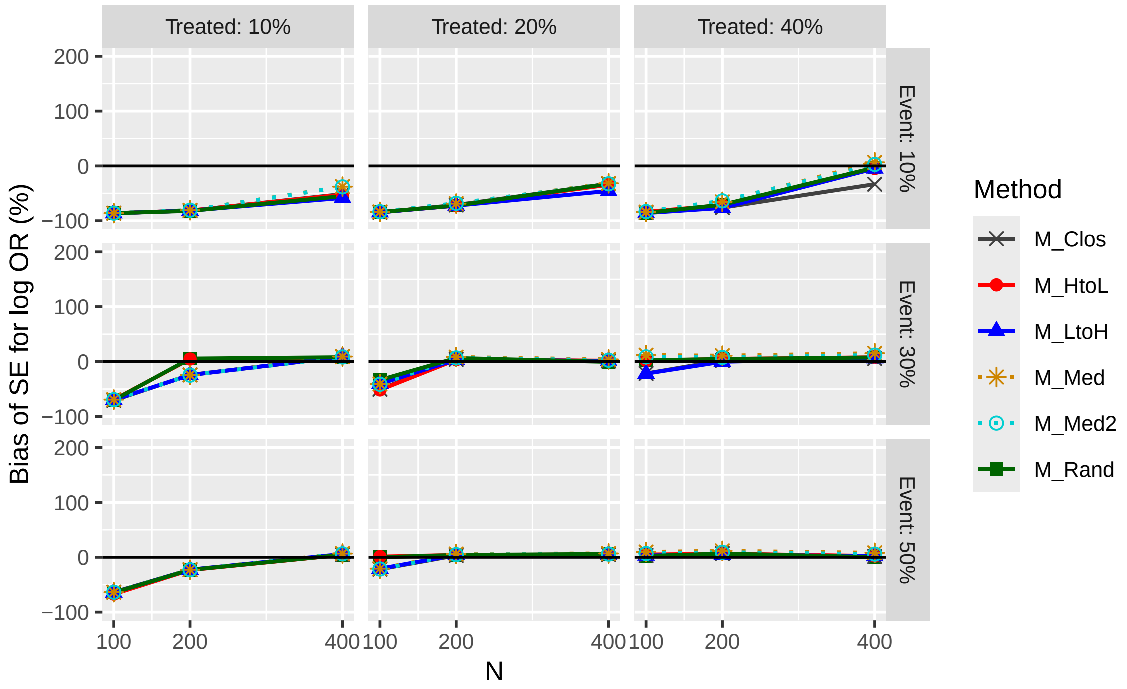

Figure S720. Mean bias of standard error for log odds ratio (unimodal continuous covariate, matching ratio 1:2, true OR: 0.75, c statistic: 0.85, robust inference).

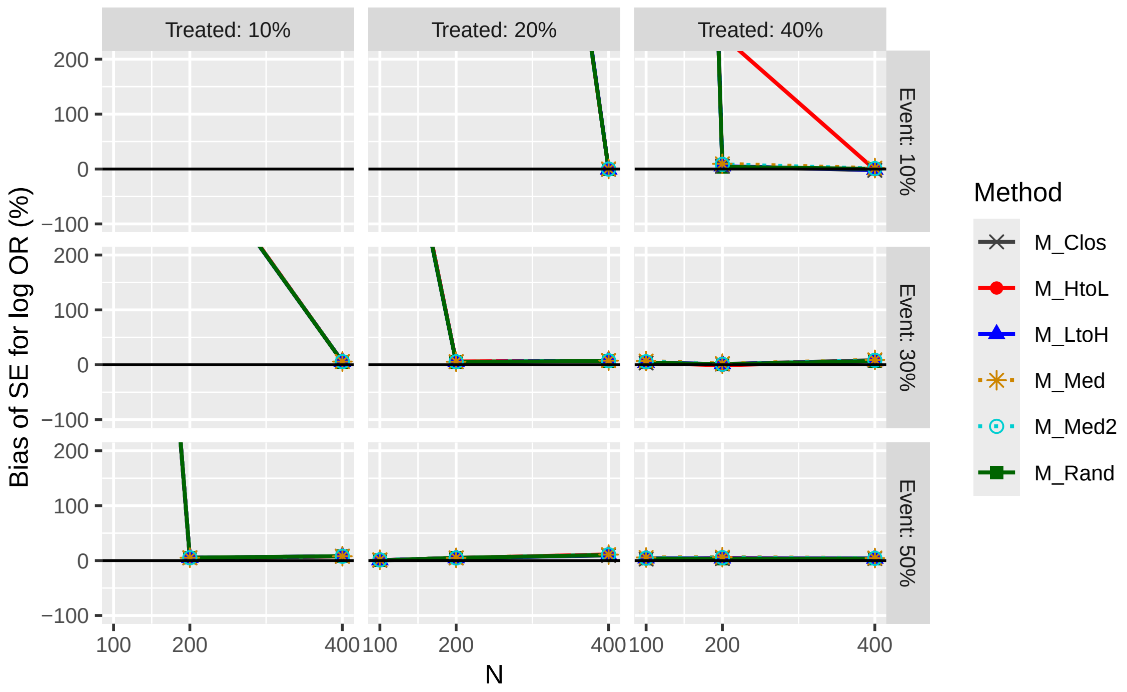

Figure S721. Mean bias of standard error for log odds ratio (unimodal continuous covariate, matching ratio 1:2, true OR: 0.75, c statistic: 0.6, naive inference).

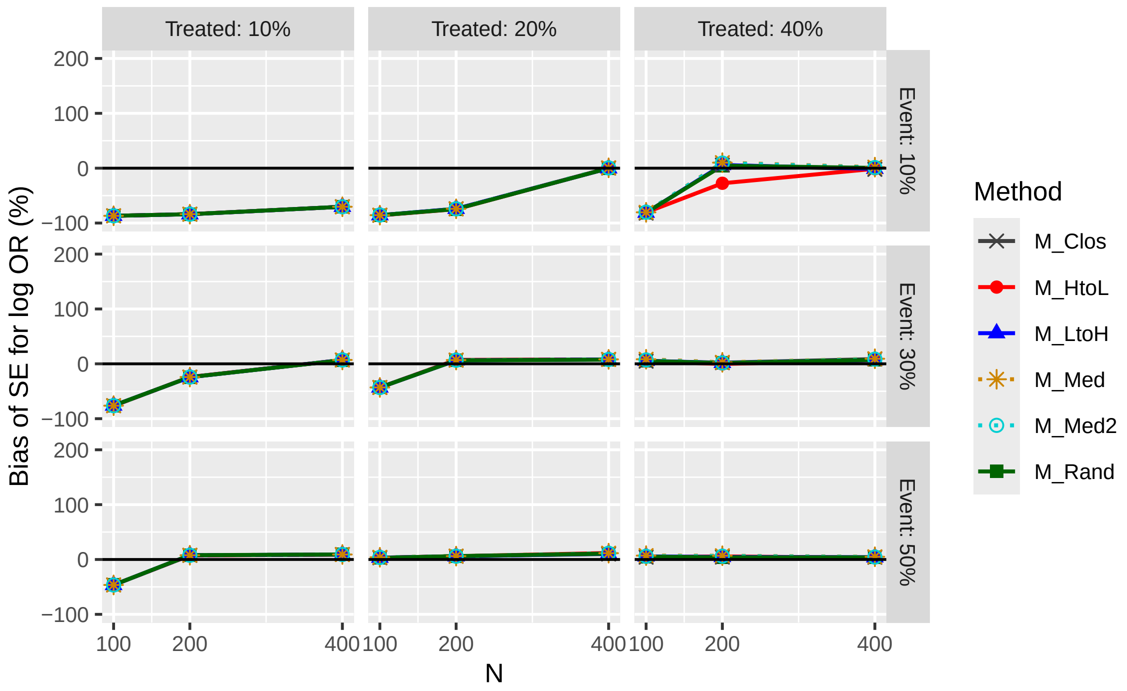

Figure S722. Mean bias of standard error for log odds ratio (unimodal continuous covariate, matching ratio 1:2, true OR: 0.75, c statistic: 0.6, robust inference).

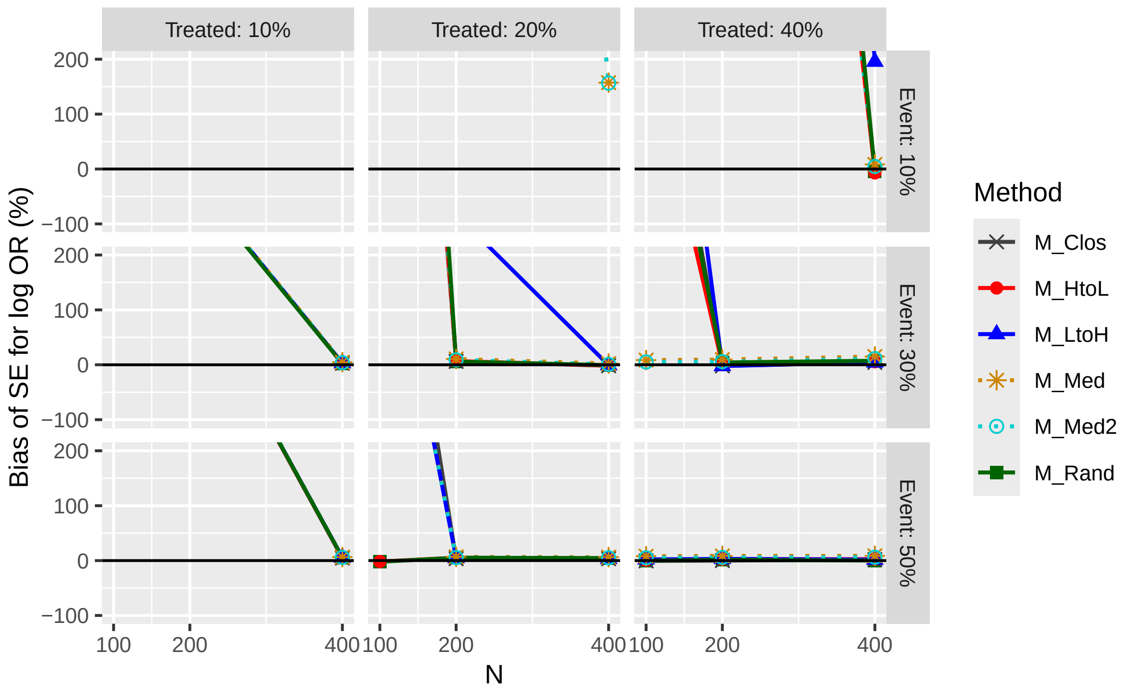

Figure S723. Mean bias of standard error for log odds ratio (unimodal continuous covariate, matching ratio 1:2, true OR: 0.5, c statistic: 0.85, naive inference).

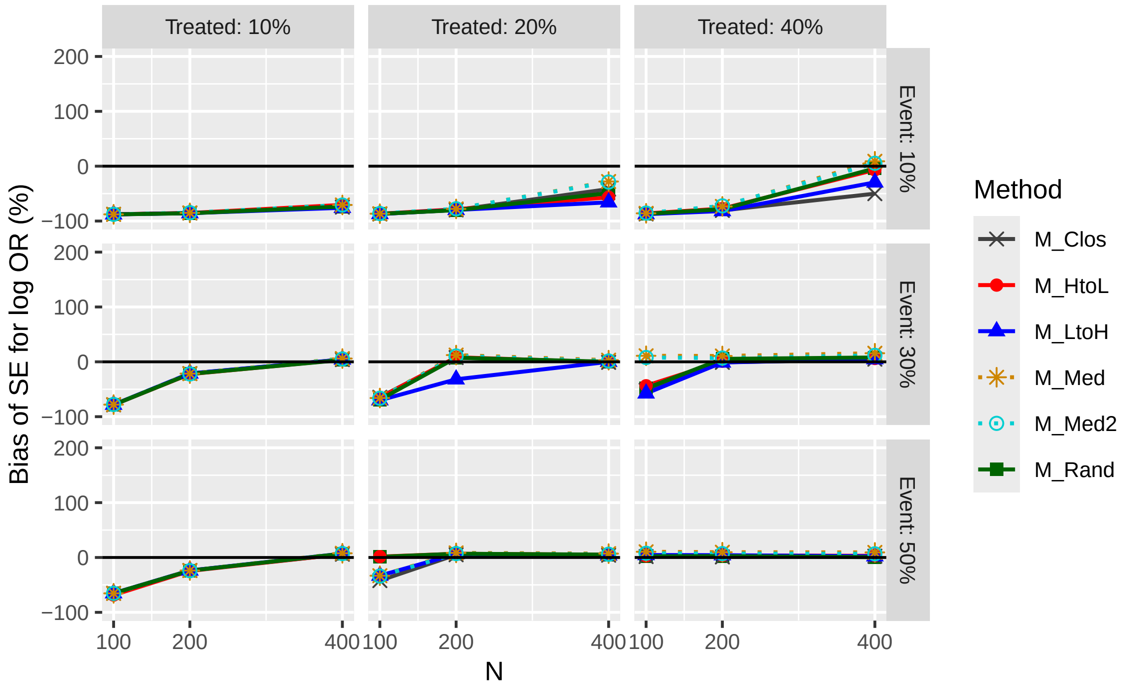

Figure S724. Mean bias of standard error for log odds ratio (unimodal continuous covariate, matching ratio 1:2, true OR: 0.5, c statistic: 0.85, robust inference).

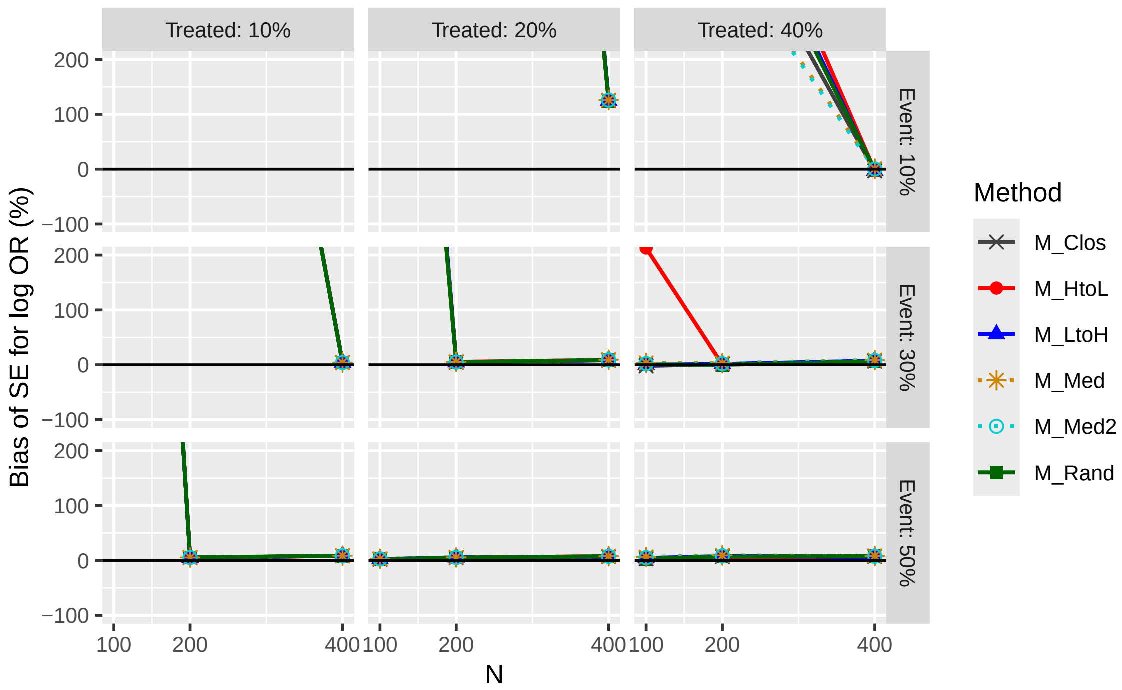

Figure S725. Mean bias of standard error for log odds ratio (unimodal continuous covariate, matching ratio 1:2, true OR: 0.5, c statistic: 0.6, naive inference).

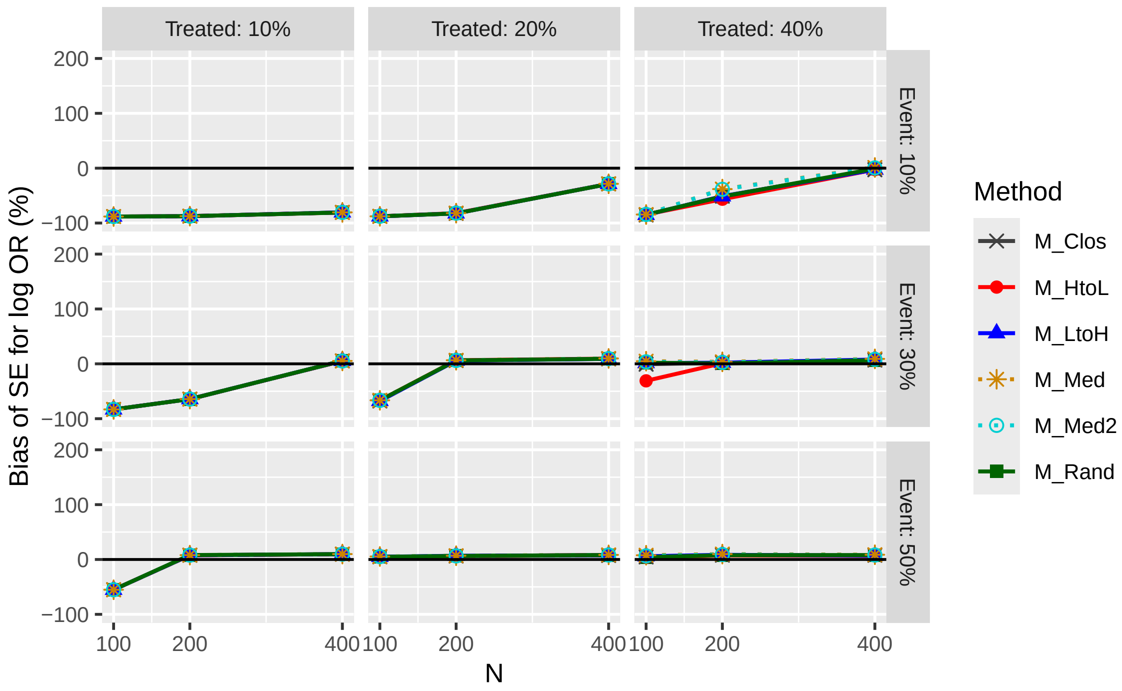

Figure S726. Mean bias of standard error for log odds ratio (unimodal continuous covariate, matching ratio 1:2, true OR: 0.5, c statistic: 0.6, robust inference).

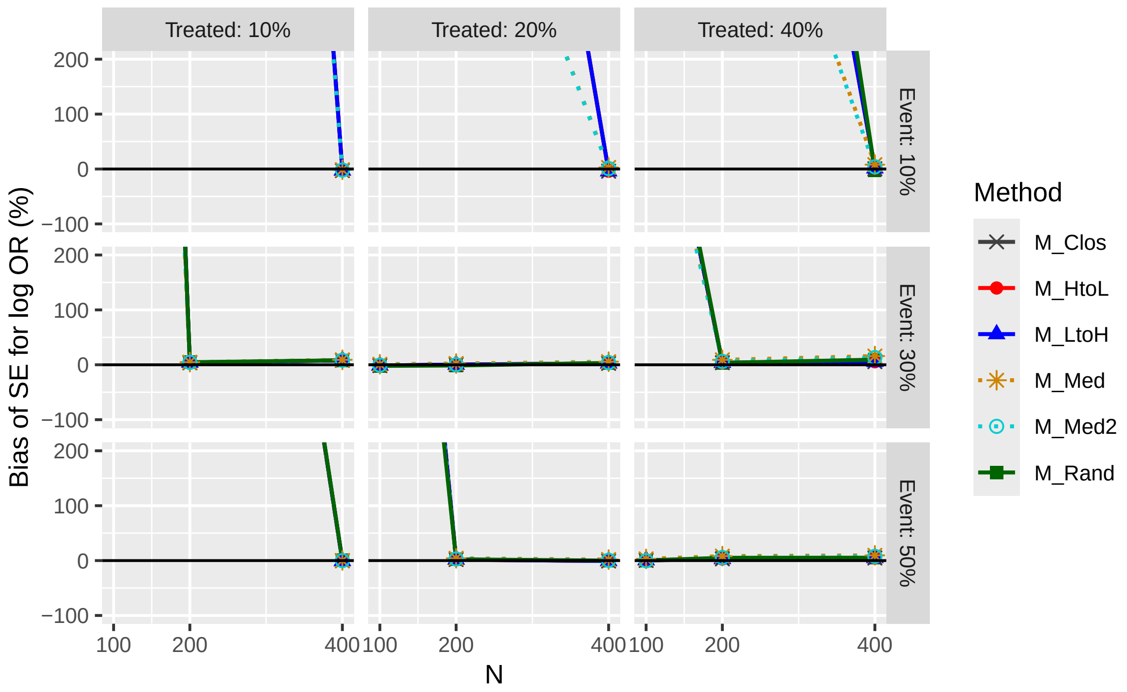

Figure S727. Mean bias of standard error for log odds ratio (categorical covariate, matching ratio 1:1, true OR: 1, c statistic: 0.85, naive inference).

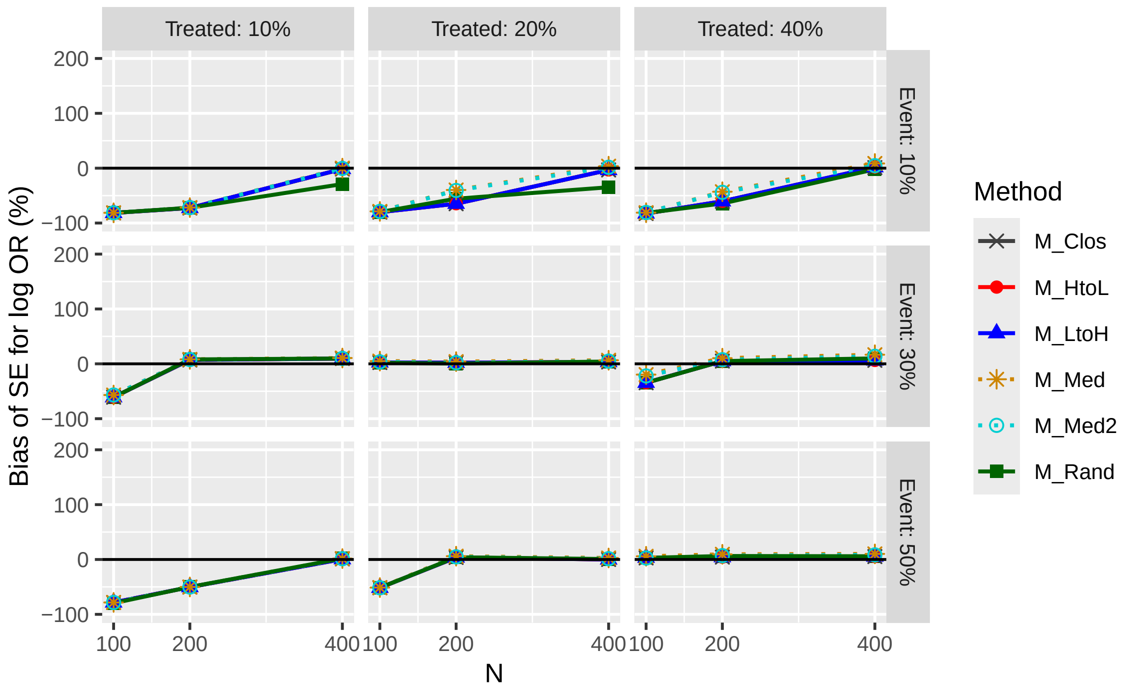

Figure S728. Mean bias of standard error for log odds ratio (categorical covariate, matching ratio 1:1, true OR: 1, c statistic: 0.85, robust inference).

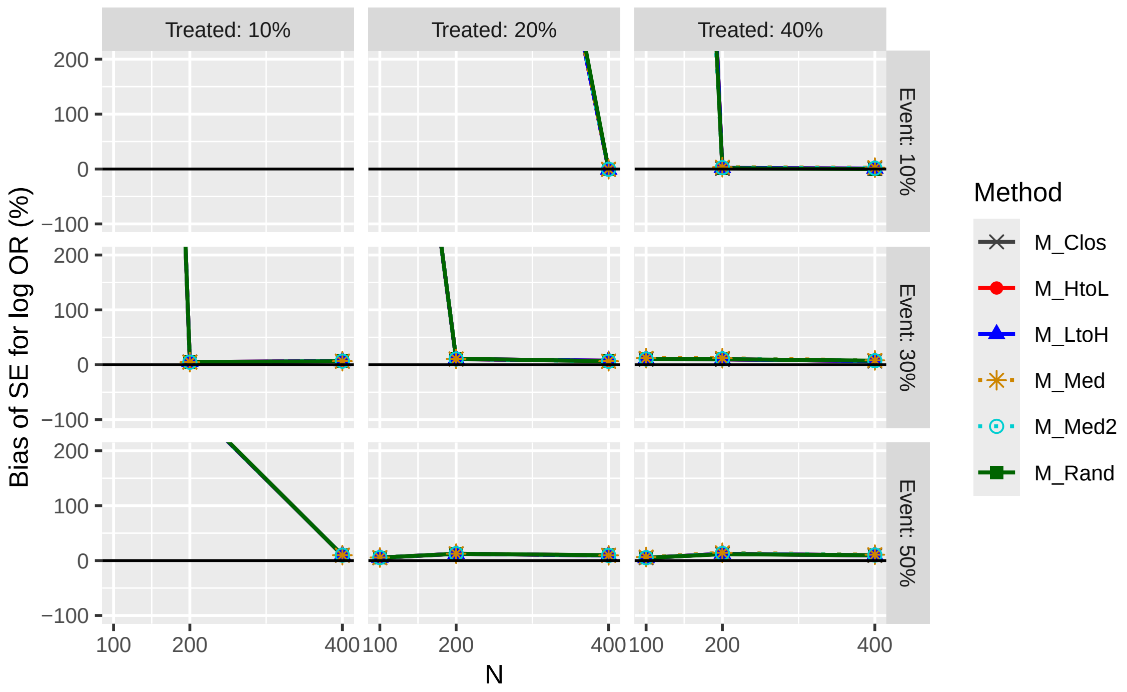

Figure S729. Mean bias of standard error for log odds ratio (categorical covariate, matching ratio 1:1, true OR: 1, c statistic: 0.6, naive inference).

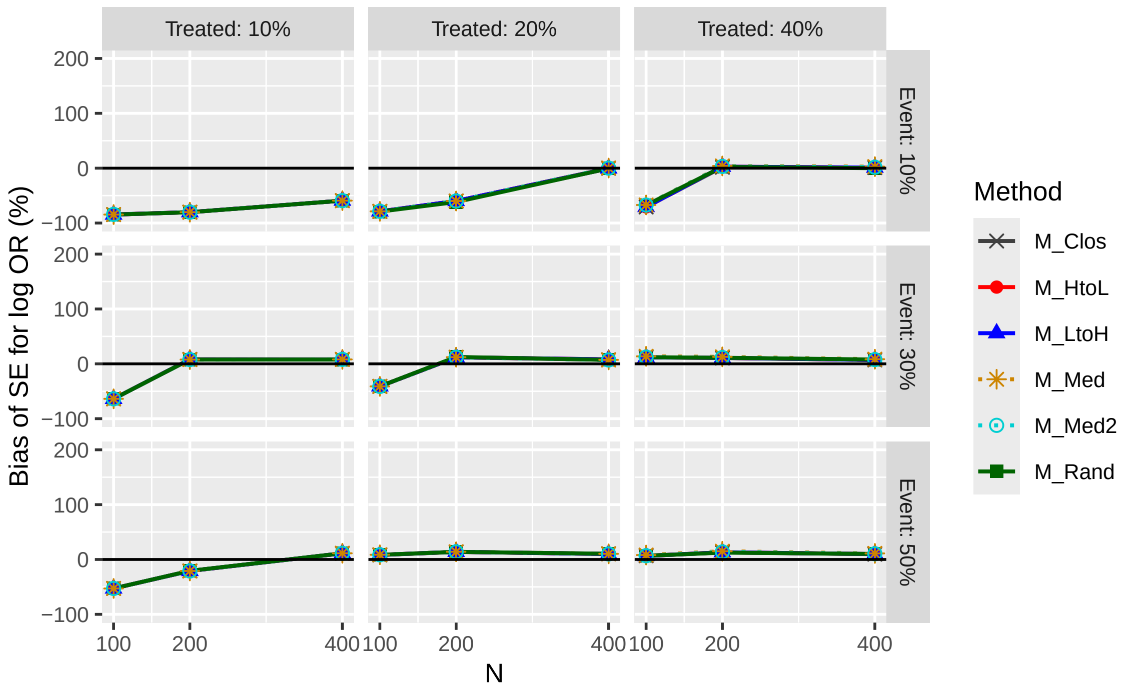

Figure S730. Mean bias of standard error for log odds ratio (categorical covariate, matching ratio 1:1, true OR: 1, c statistic: 0.6, robust inference).

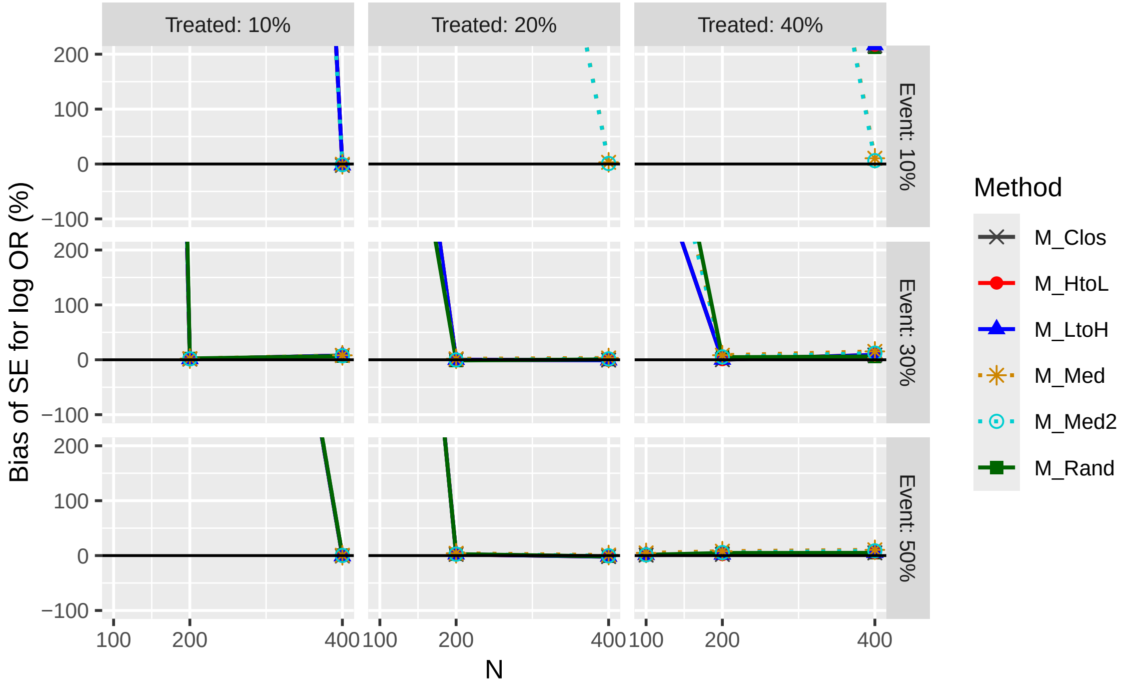

Figure S731. Mean bias of standard error for log odds ratio (categorical covariate, matching ratio 1:1, true OR: 0.75, c statistic: 0.85, naive inference).

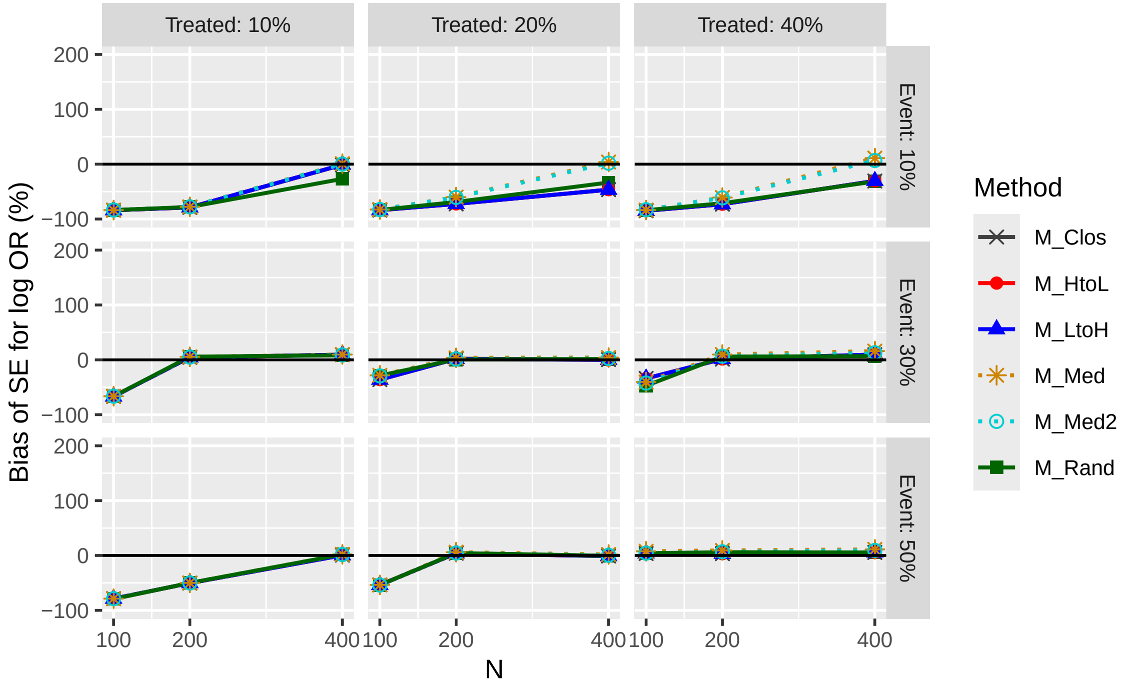

Figure S732. Mean bias of standard error for log odds ratio (categorical covariate, matching ratio 1:1, true OR: 0.75, c statistic: 0.85, robust inference).

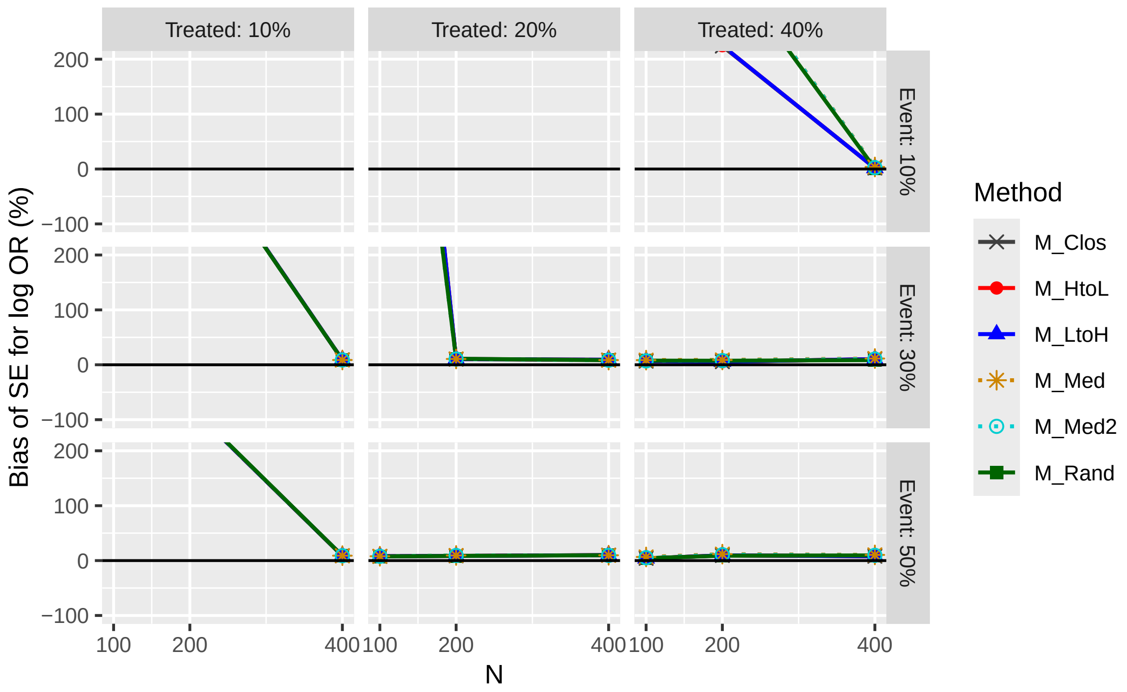

Figure S733. Mean bias of standard error for log odds ratio (categorical covariate, matching ratio 1:1, true OR: 0.75, c statistic: 0.6, naive inference).

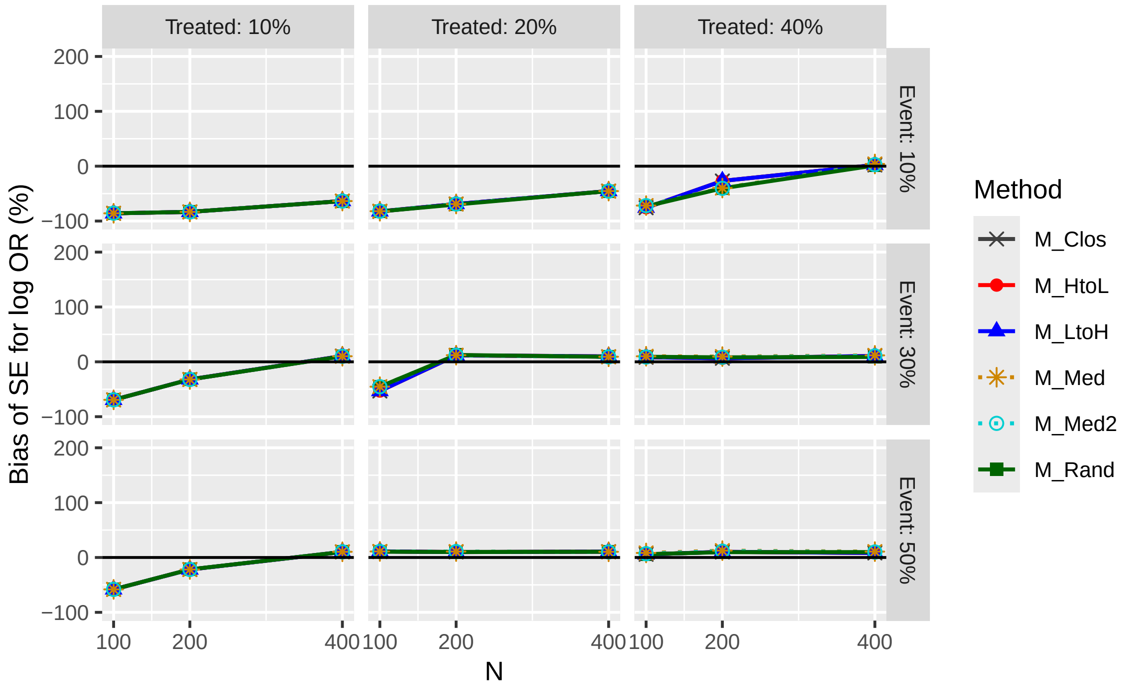

Figure S734. Mean bias of standard error for log odds ratio (categorical covariate, matching ratio 1:1, true OR: 0.75, c statistic: 0.6, robust inference).

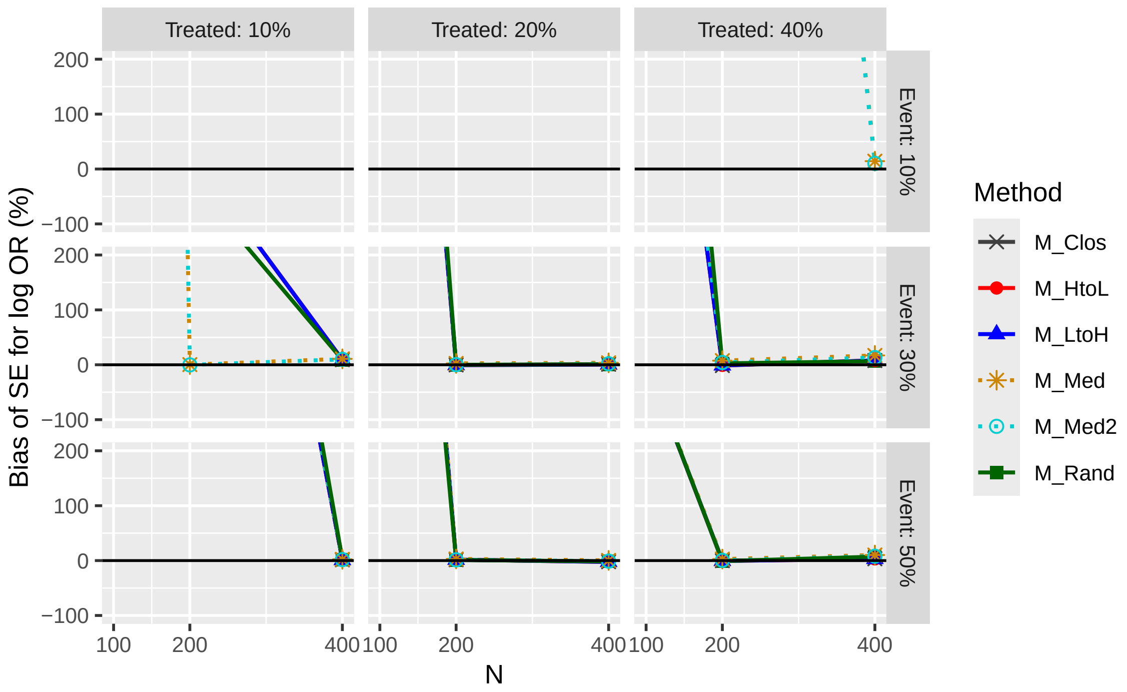

Figure S735. Mean bias of standard error for log odds ratio (categorical covariate, matching ratio 1:1, true OR: 0.5, c statistic: 0.85, naive inference).

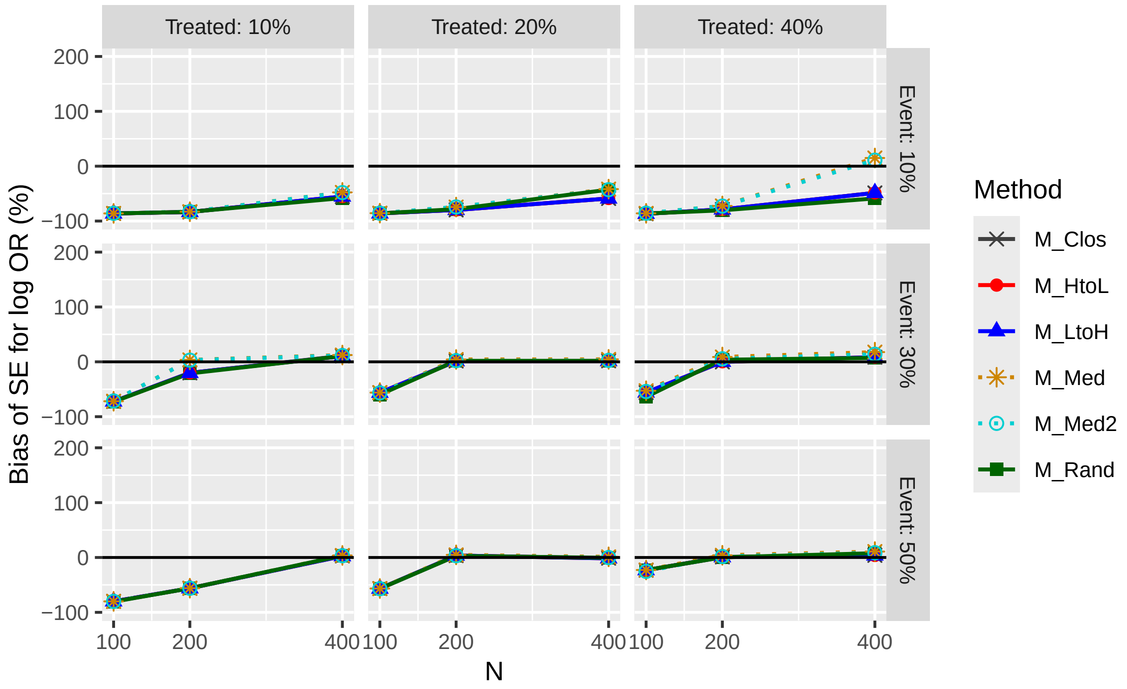

Figure S736. Mean bias of standard error for log odds ratio (categorical covariate, matching ratio 1:1, true OR: 0.5, c statistic: 0.85, robust inference).

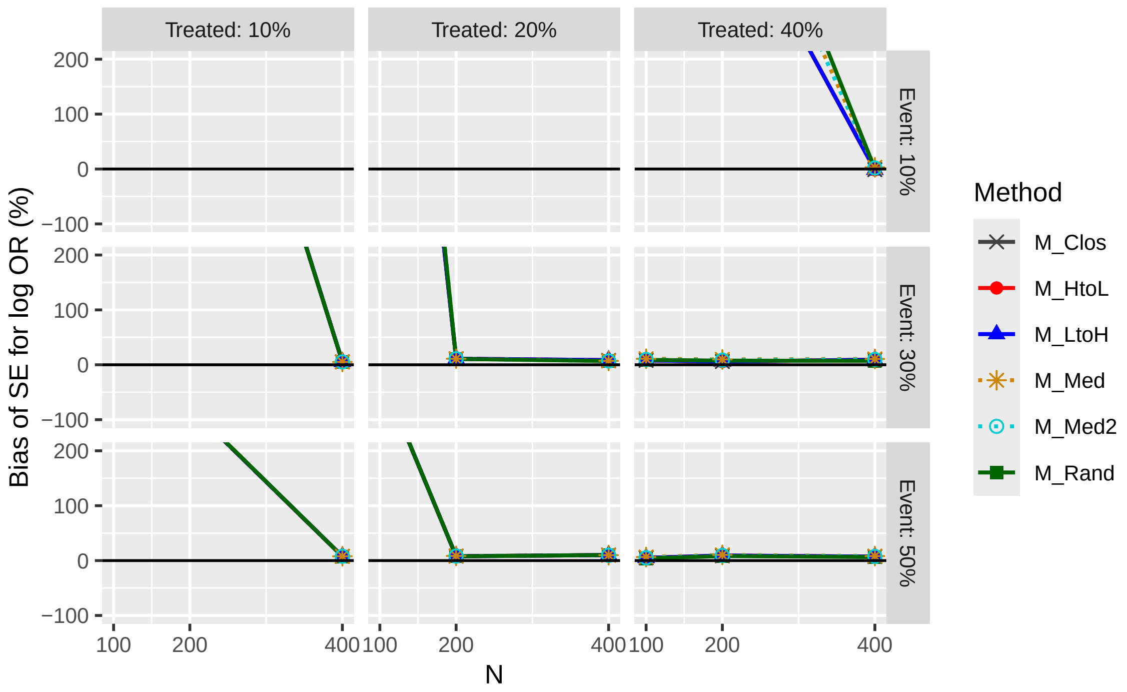

Figure S737. Mean bias of standard error for log odds ratio (categorical covariate, matching ratio 1:1, true OR: 0.5, c statistic: 0.6, naive inference).

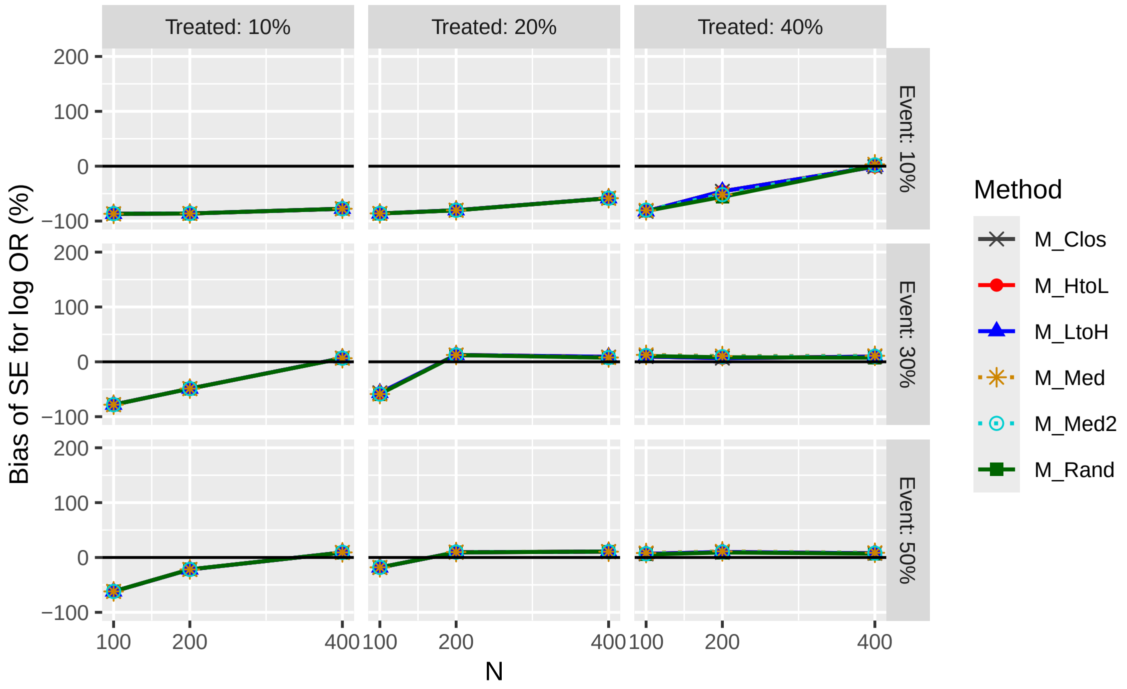

Figure S738. Mean bias of standard error for log odds ratio (categorical covariate, matching ratio 1:1, true OR: 0.5, c statistic: 0.6, robust inference).

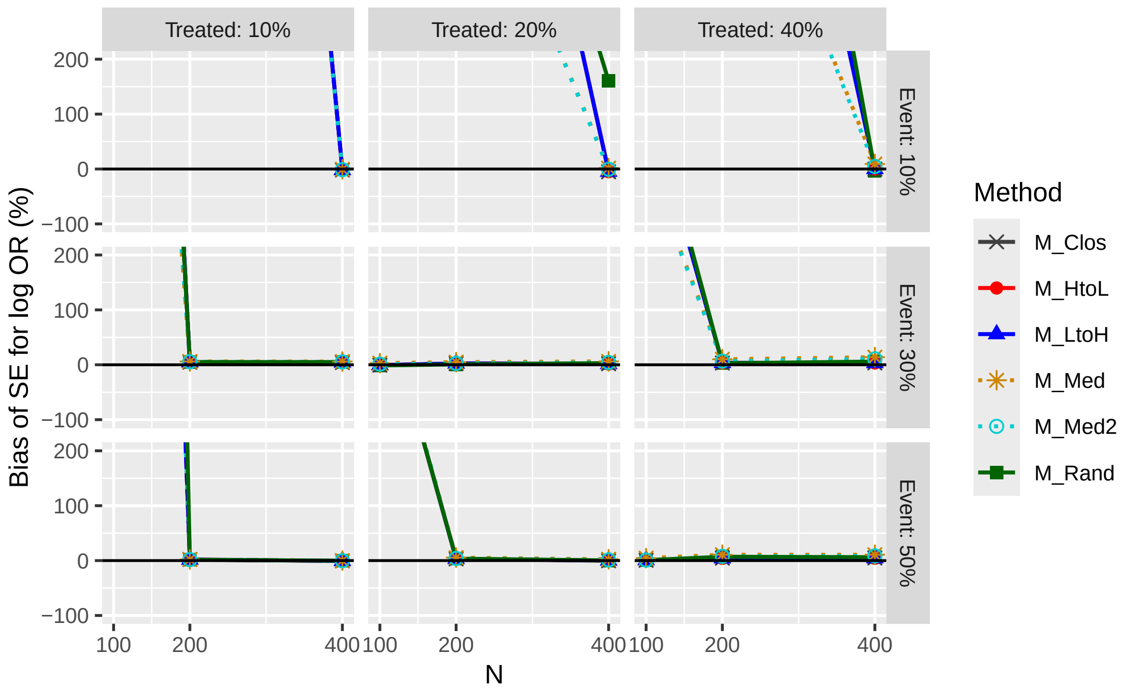

Figure S739. Mean bias of standard error for log odds ratio (categorical covariate, matching ratio 1:2, true OR: 1, c statistic: 0.85, naive inference).

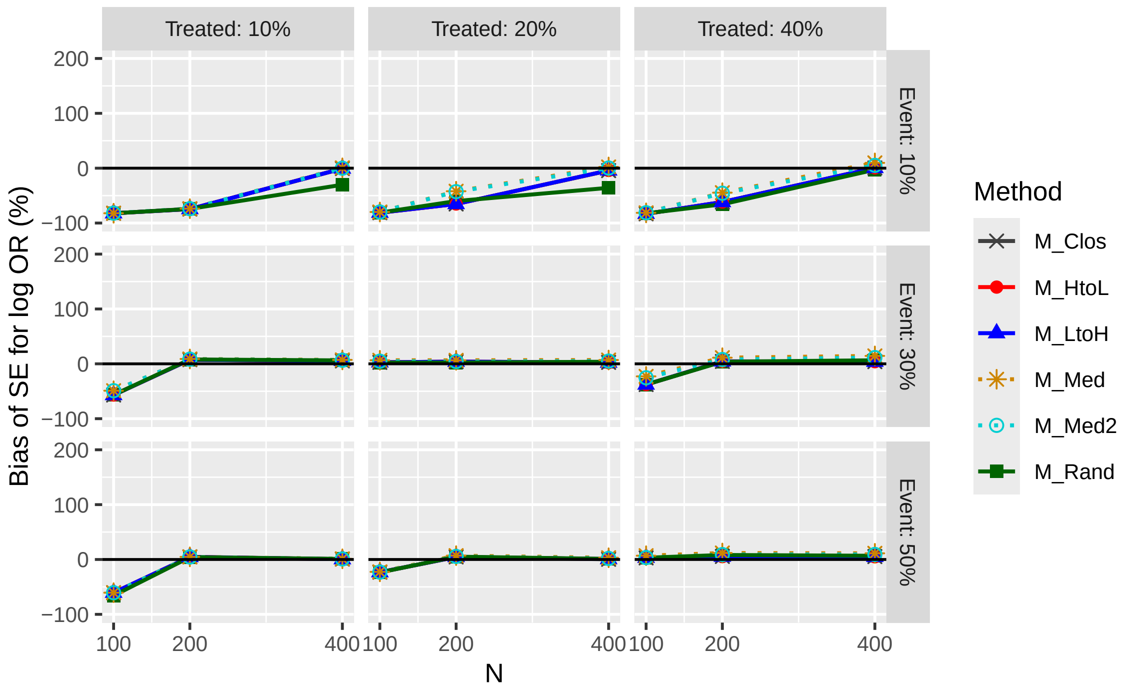

Figure S740. Mean bias of standard error for log odds ratio (categorical covariate, matching ratio 1:2, true OR: 1, c statistic: 0.85, robust inference).

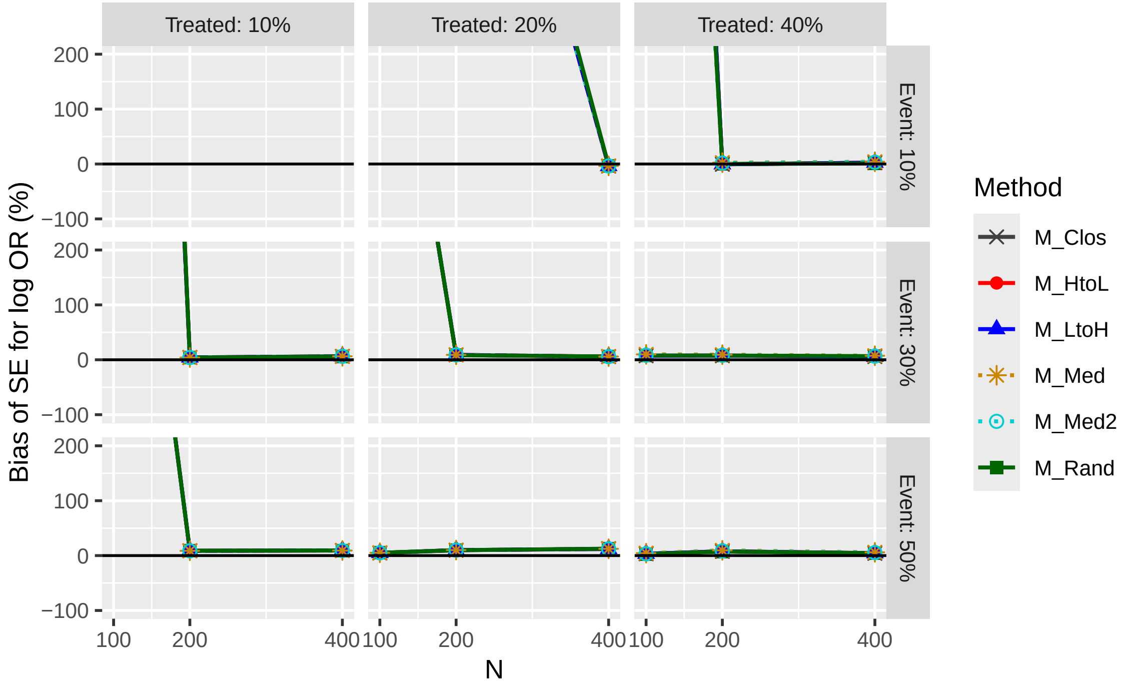

Figure S741. Mean bias of standard error for log odds ratio (categorical covariate, matching ratio 1:2, true OR: 1, c statistic: 0.6, naive inference).

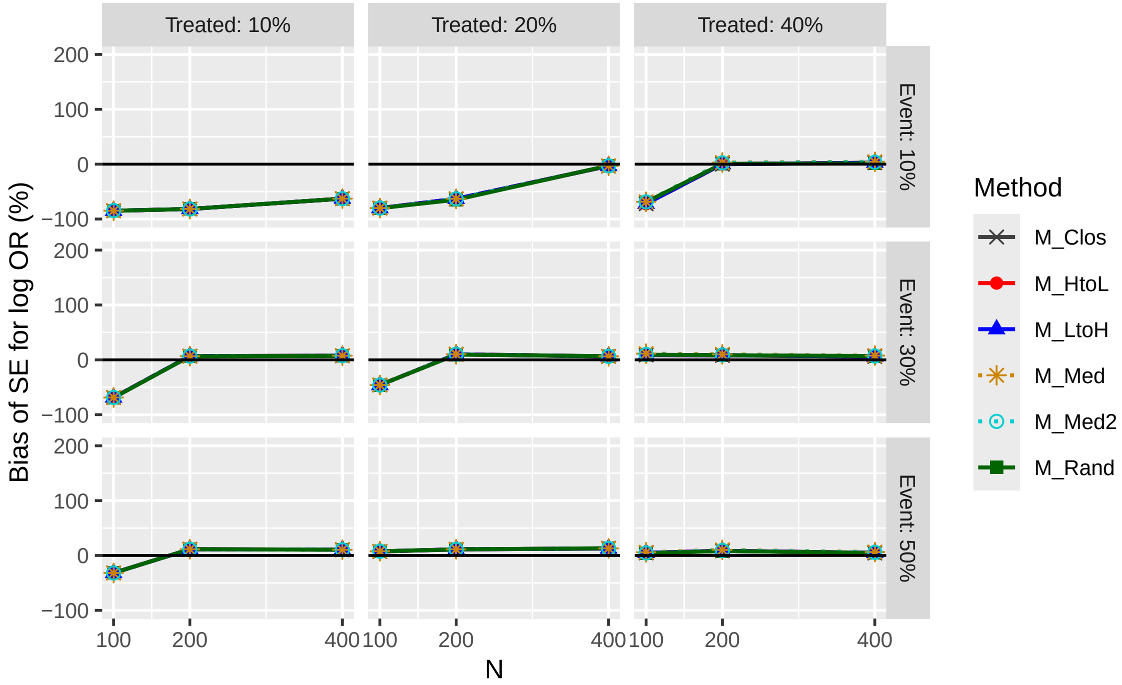

Figure S742. Mean bias of standard error for log odds ratio (categorical covariate, matching ratio 1:2, true OR: 1, c statistic: 0.6, robust inference).

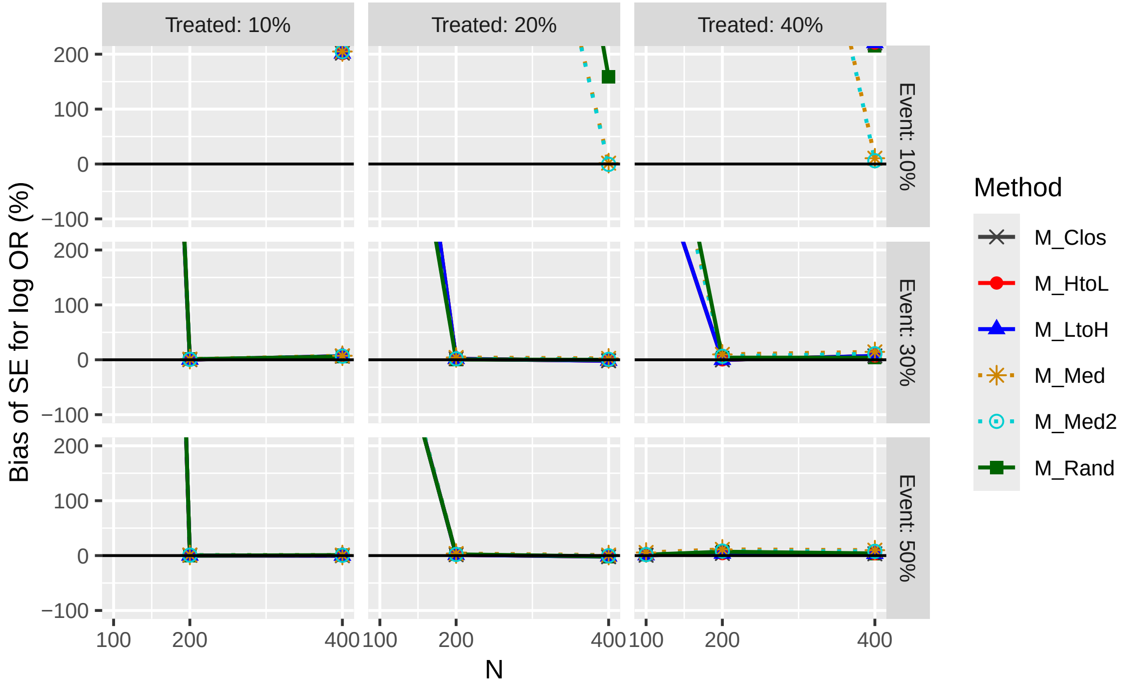

Figure S743. Mean bias of standard error for log odds ratio (categorical covariate, matching ratio 1:2, true OR: 0.75, c statistic: 0.85, naive inference).

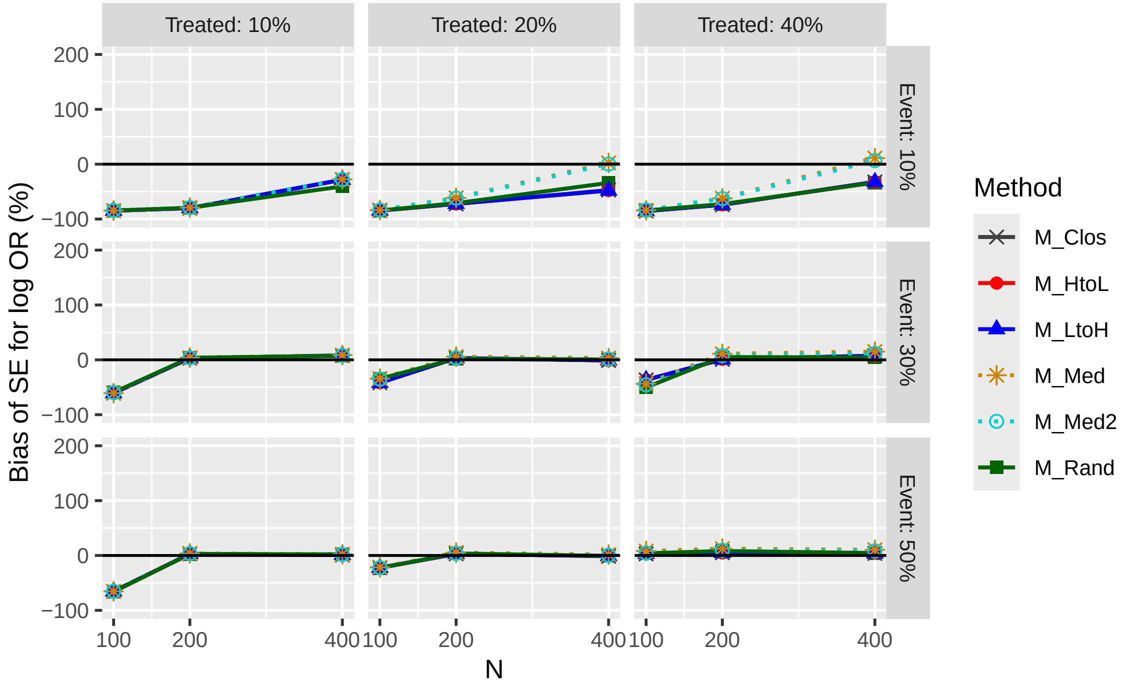

Figure S744. Mean bias of standard error for log odds ratio (categorical covariate, matching ratio 1:2, true OR: 0.75, c statistic: 0.85, robust inference).

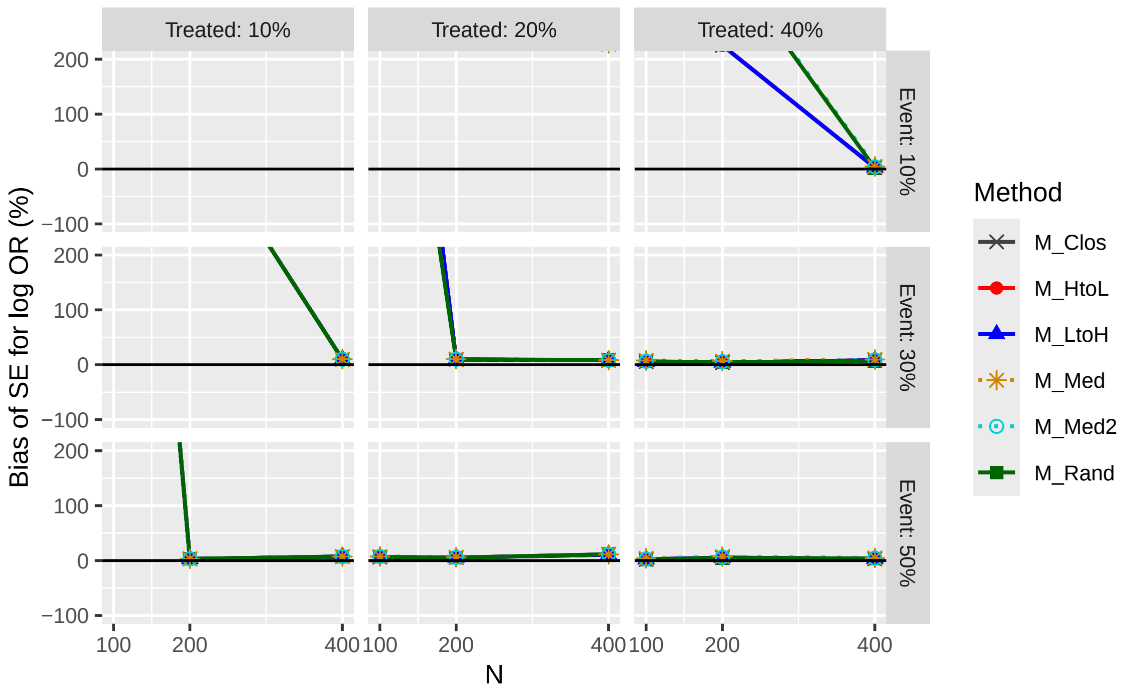

Figure S745. Mean bias of standard error for log odds ratio (categorical covariate, matching ratio 1:2, true OR: 0.75, c statistic: 0.6, naive inference).

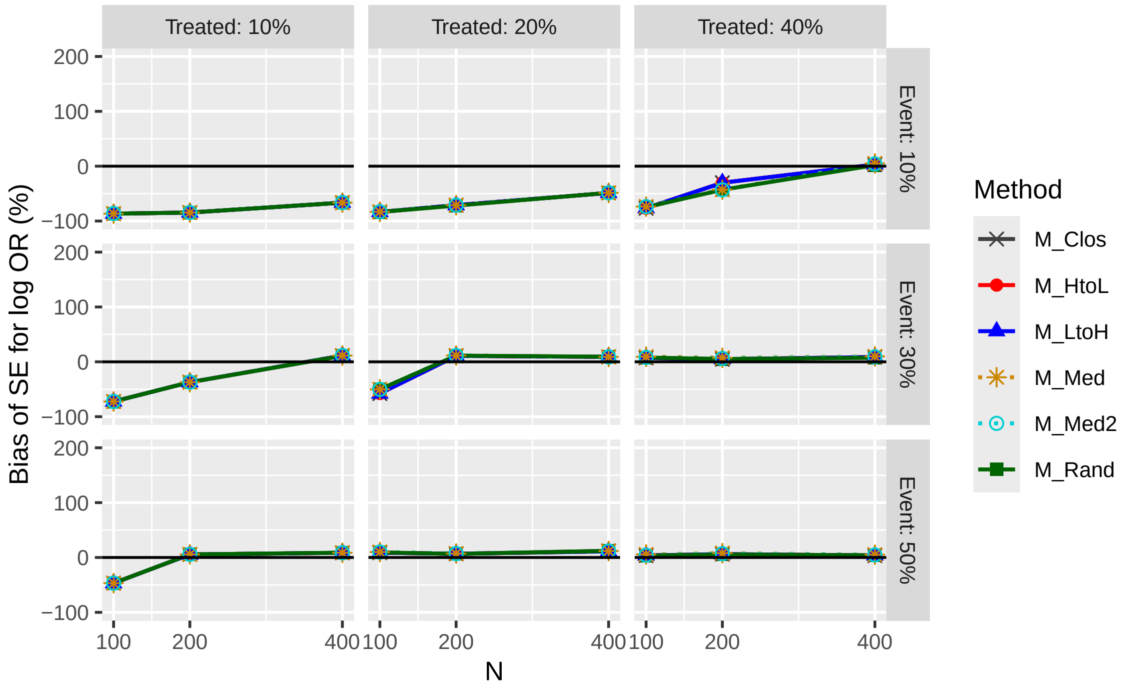

Figure S746. Mean bias of standard error for log odds ratio (categorical covariate, matching ratio 1:2, true OR: 0.75, c statistic: 0.6, robust inference).

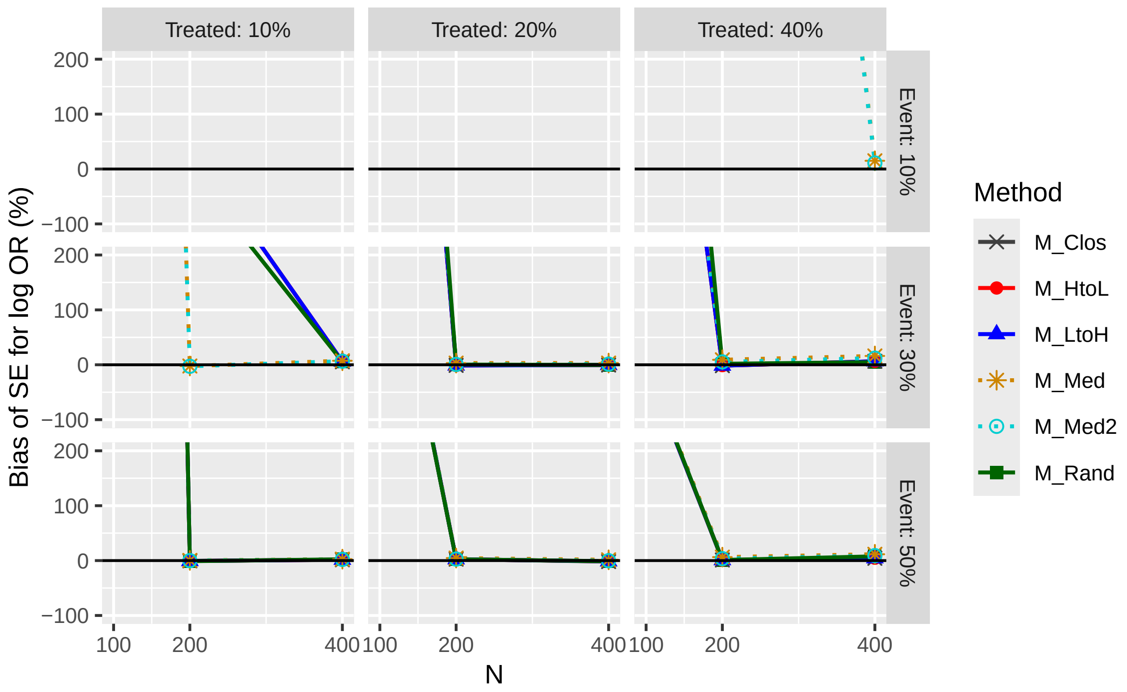

Figure S747. Mean bias of standard error for log odds ratio (categorical covariate, matching ratio 1:2, true OR: 0.5, c statistic: 0.85, naive inference).

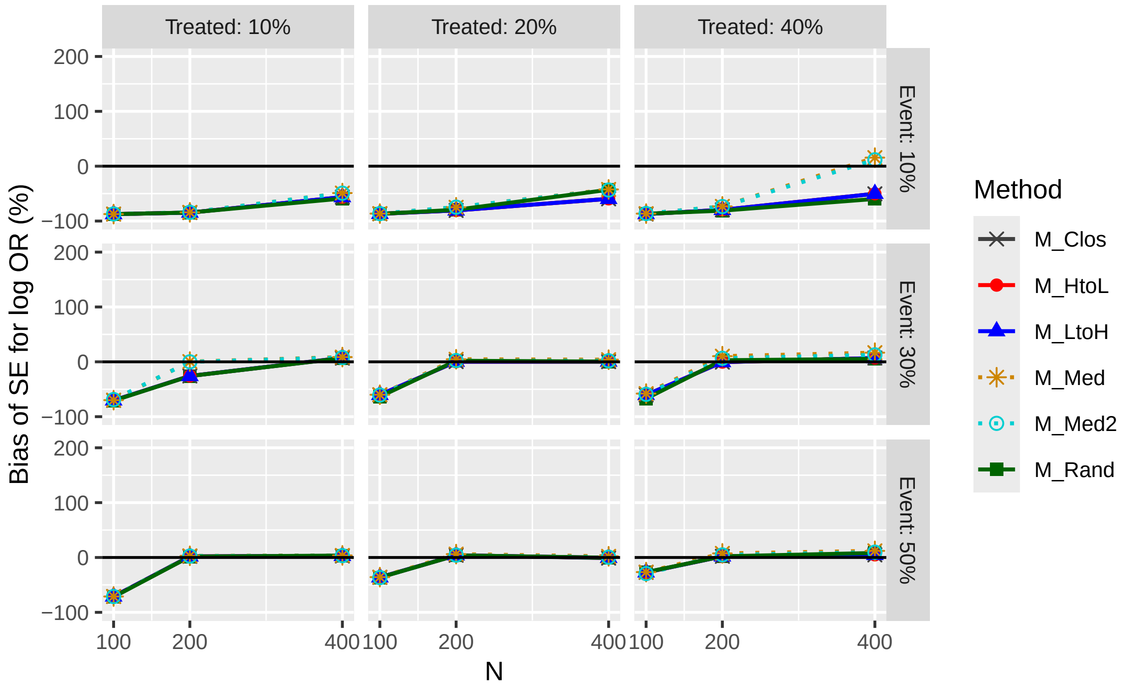

Figure S748. Mean bias of standard error for log odds ratio (categorical covariate, matching ratio 1:2, true OR: 0.5, c statistic: 0.85, robust inference).

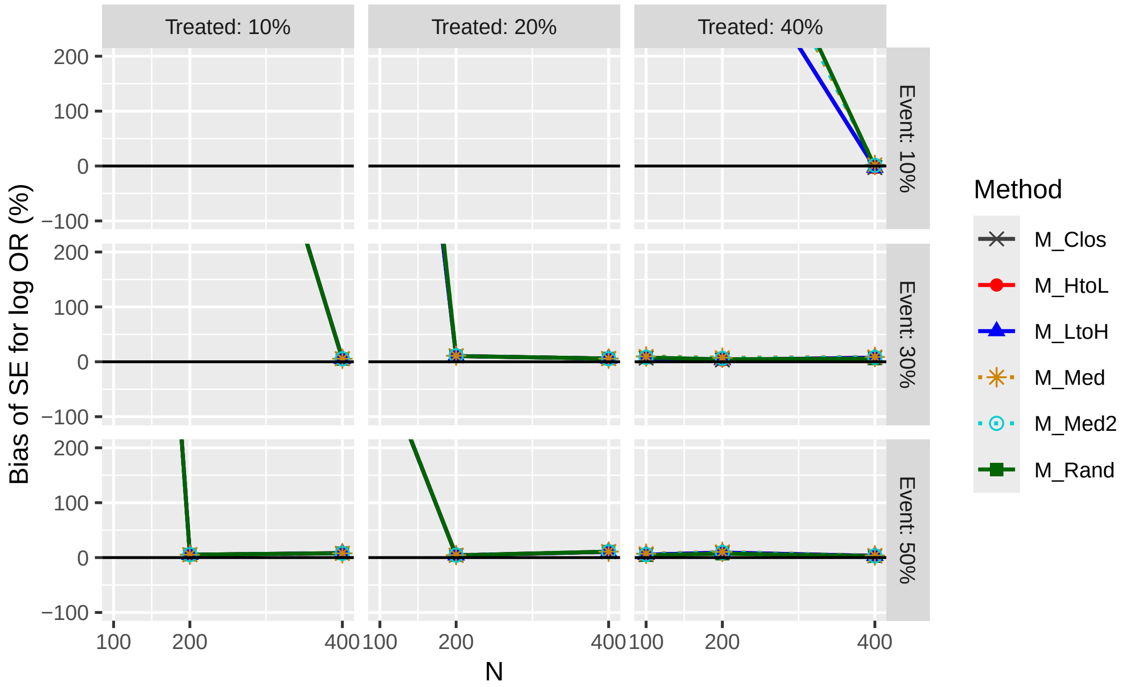

Figure S749. Mean bias of standard error for log odds ratio (categorical covariate, matching ratio 1:2, true OR: 0.5, c statistic: 0.6, naive inference).

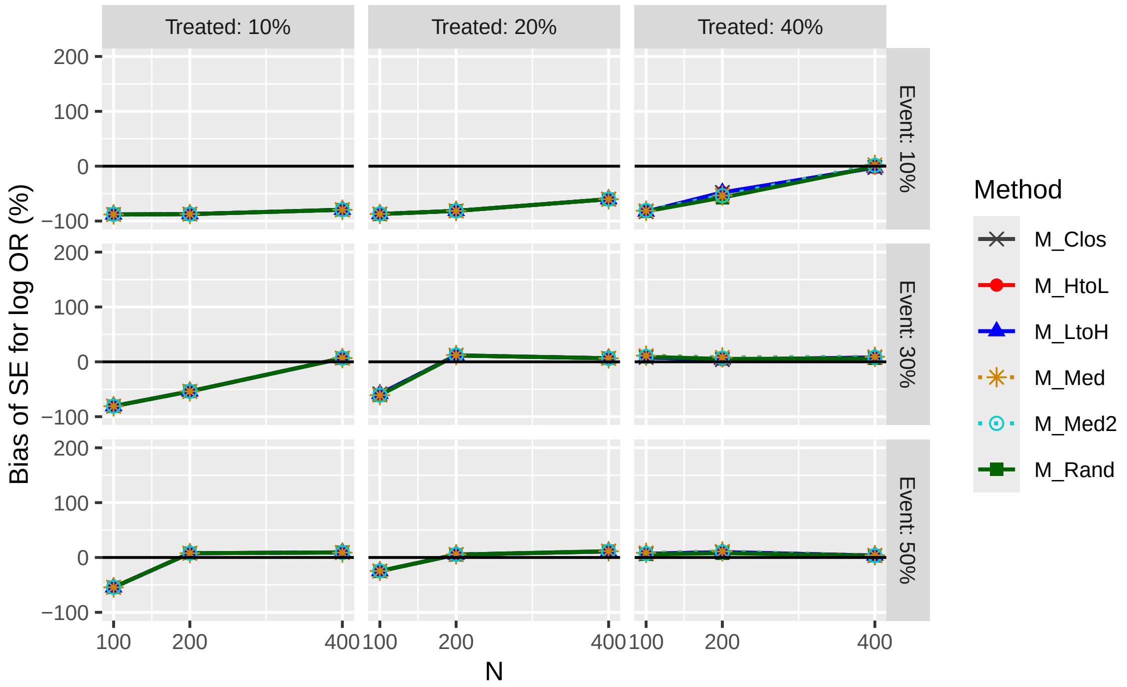

Figure S750. Mean bias of standard error for log odds ratio (categorical covariate, matching ratio 1:2, true OR: 0.5, c statistic: 0.6, robust inference).

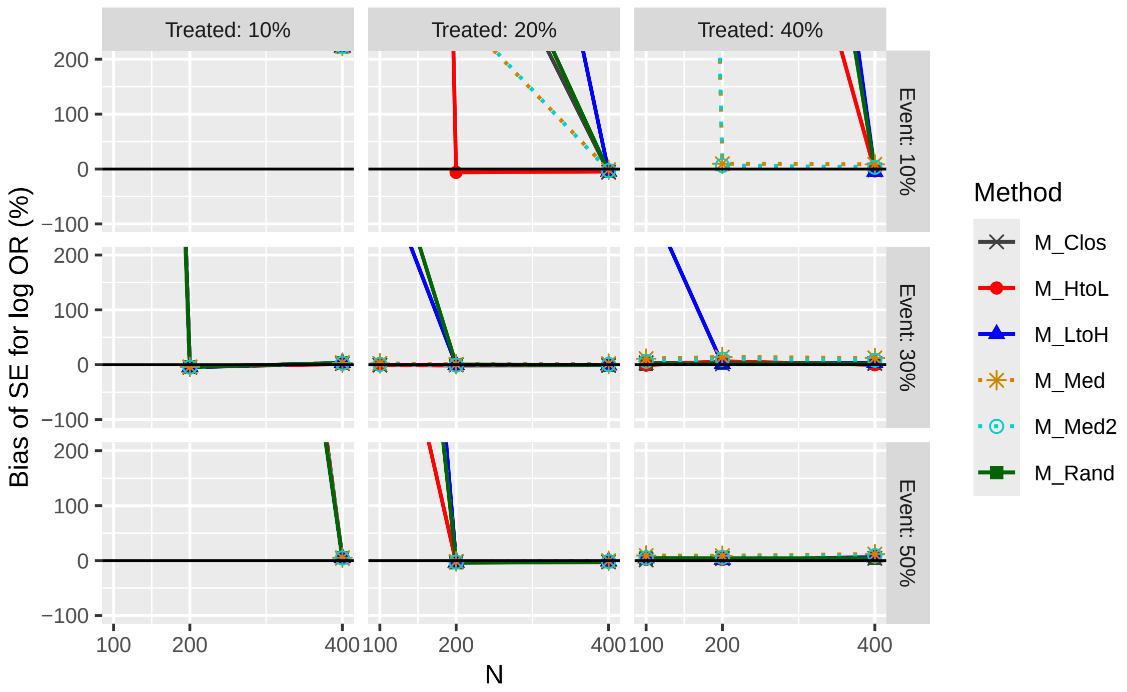

Figure S751. Mean bias of standard error for log odds ratio (multimodal continuous covariate, matching ratio 1:1, true OR: 1, c statistic: 0.85, naive inference).

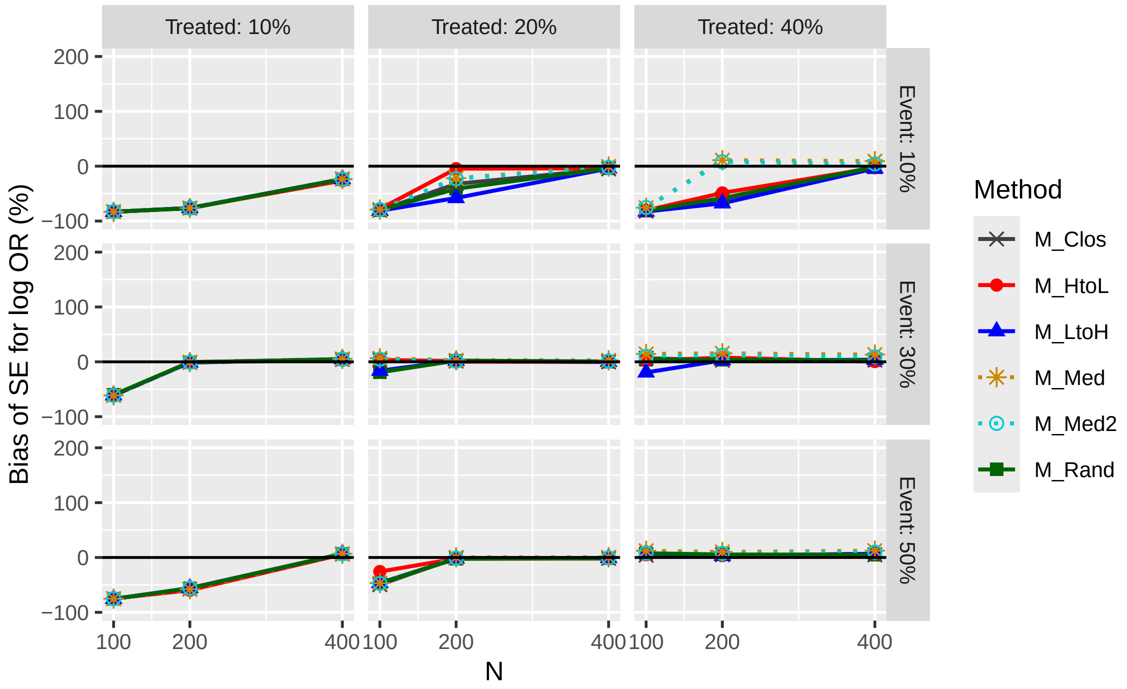

Figure S752. Mean bias of standard error for log odds ratio (multimodal continuous covariate, matching ratio 1:1, true OR: 1, c statistic: 0.85, robust inference).

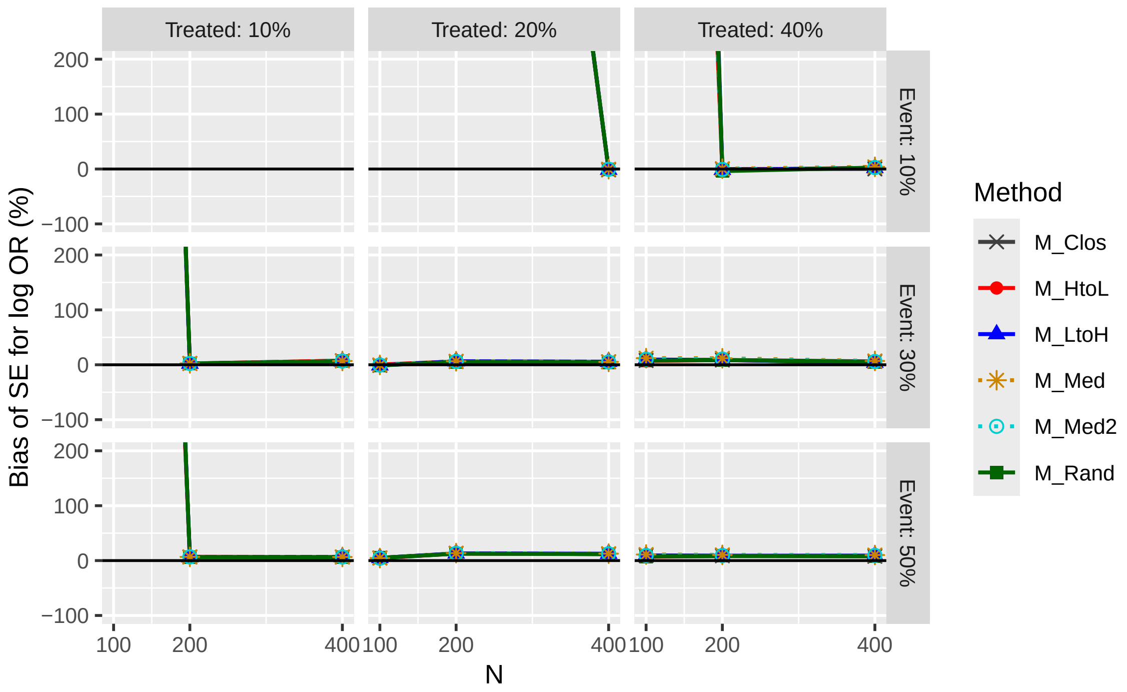

Figure S753. Mean bias of standard error for log odds ratio (multimodal continuous covariate, matching ratio 1:1, true OR: 1, c statistic: 0.6, naive inference).

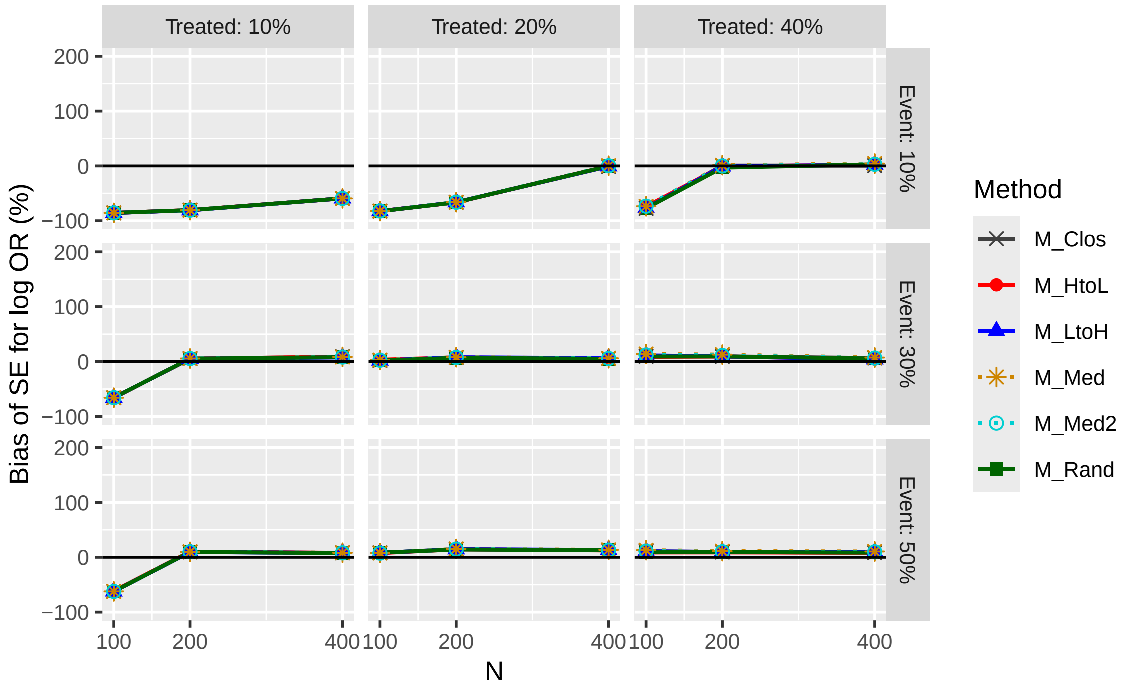

Figure S754. Mean bias of standard error for log odds ratio (multimodal continuous covariate, matching ratio 1:1, true OR: 1, c statistic: 0.6, robust inference).

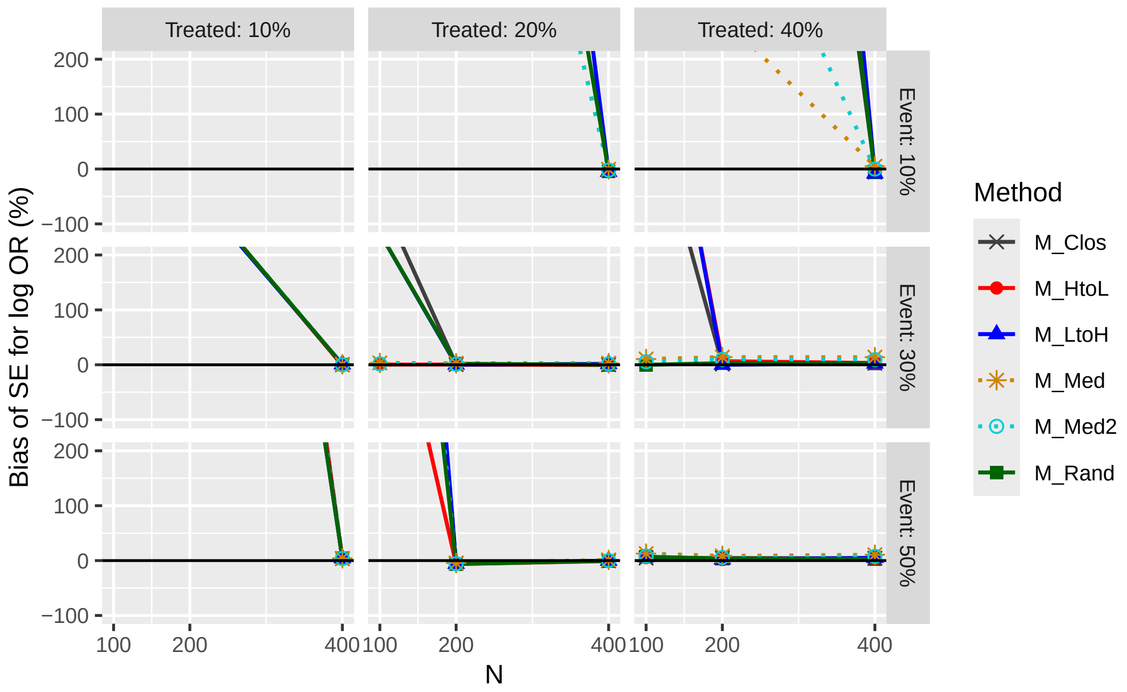

Figure S755. Mean bias of standard error for log odds ratio (multimodal continuous covariate, matching ratio 1:1, true OR: 0.75, c statistic: 0.85, naive inference).

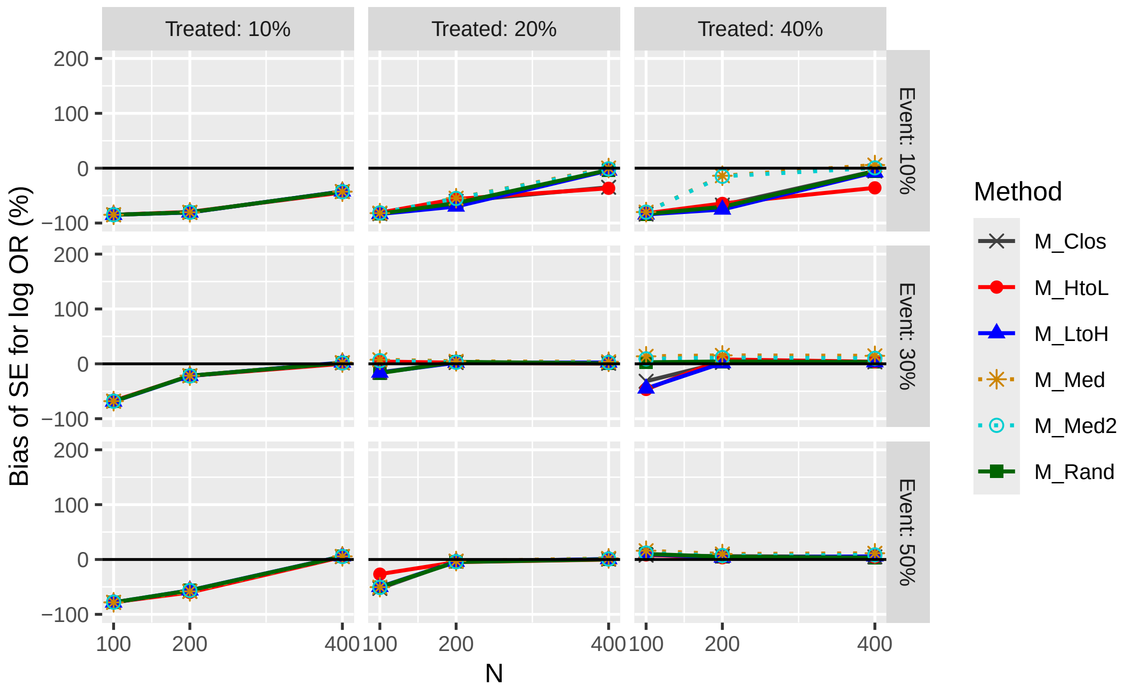

Figure S756. Mean bias of standard error for log odds ratio (multimodal continuous covariate, matching ratio 1:1, true OR: 0.75, c statistic: 0.85, robust inference).

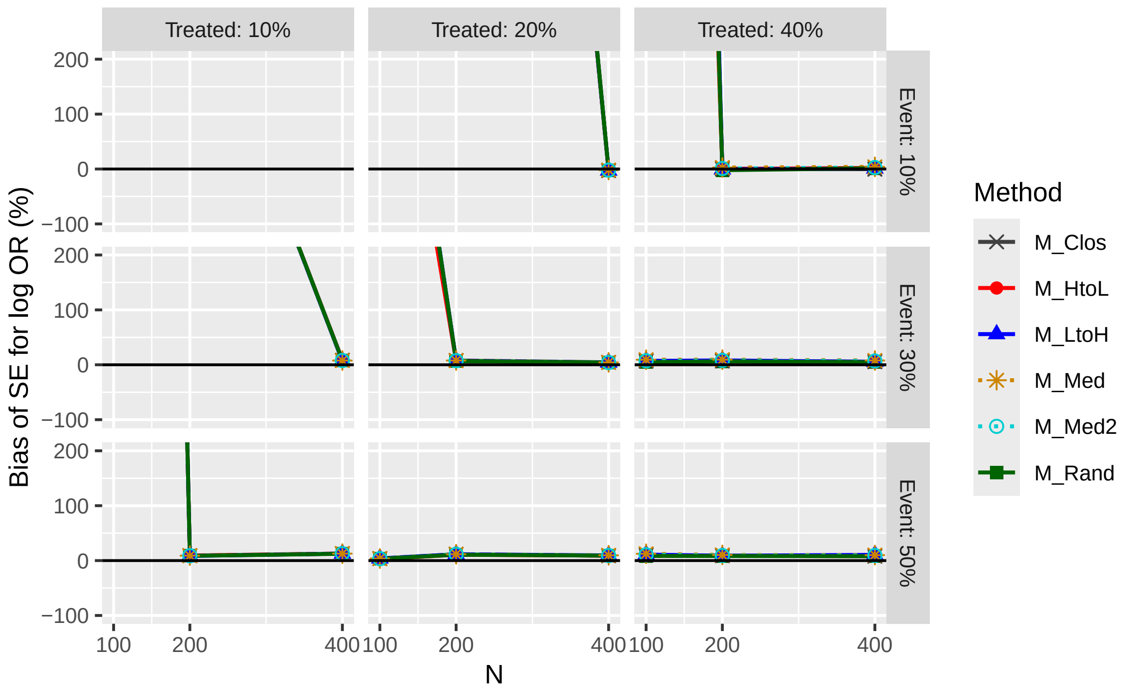

Figure S757. Mean bias of standard error for log odds ratio (multimodal continuous covariate, matching ratio 1:1, true OR: 0.75, c statistic: 0.6, naive inference).

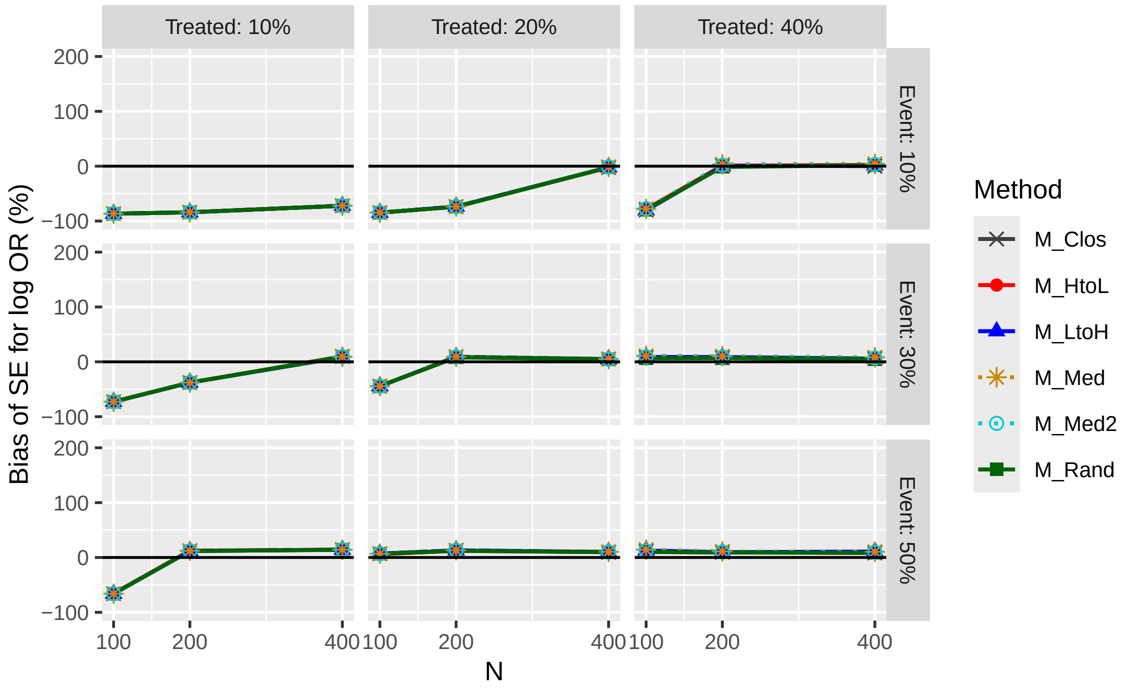

Figure S758. Mean bias of standard error for log odds ratio (multimodal continuous covariate, matching ratio 1:1, true OR: 0.75, c statistic: 0.6, robust inference).

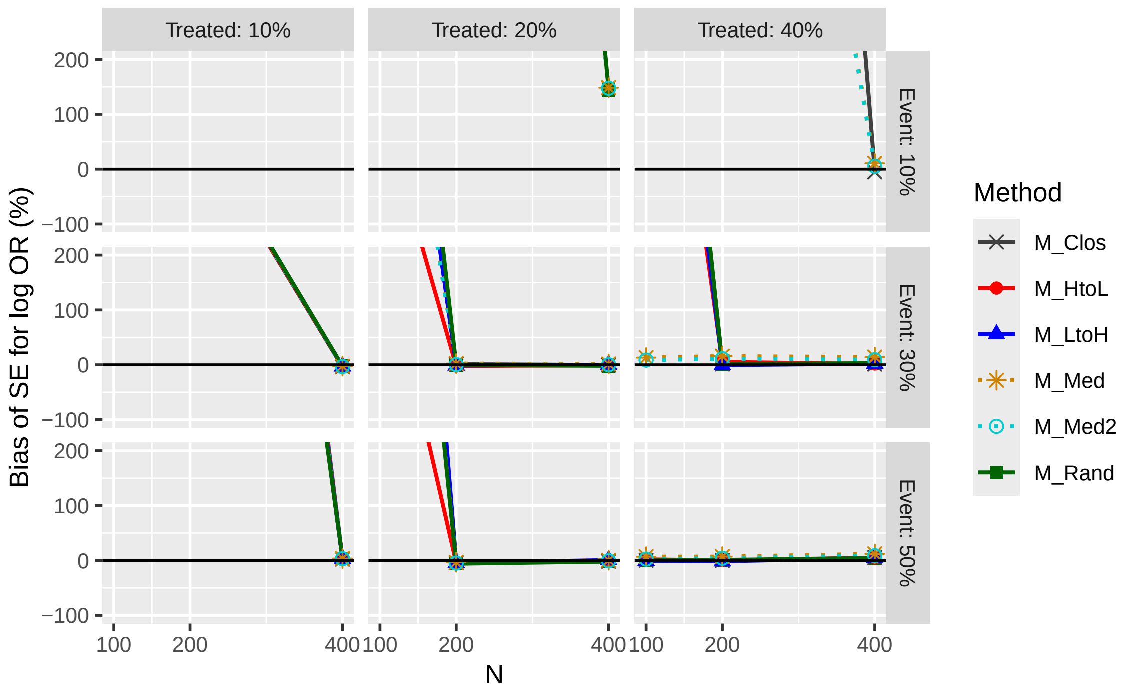

Figure S759. Mean bias of standard error for log odds ratio (multimodal continuous covariate, matching ratio 1:1, true OR: 0.5, c statistic: 0.85, naive inference).

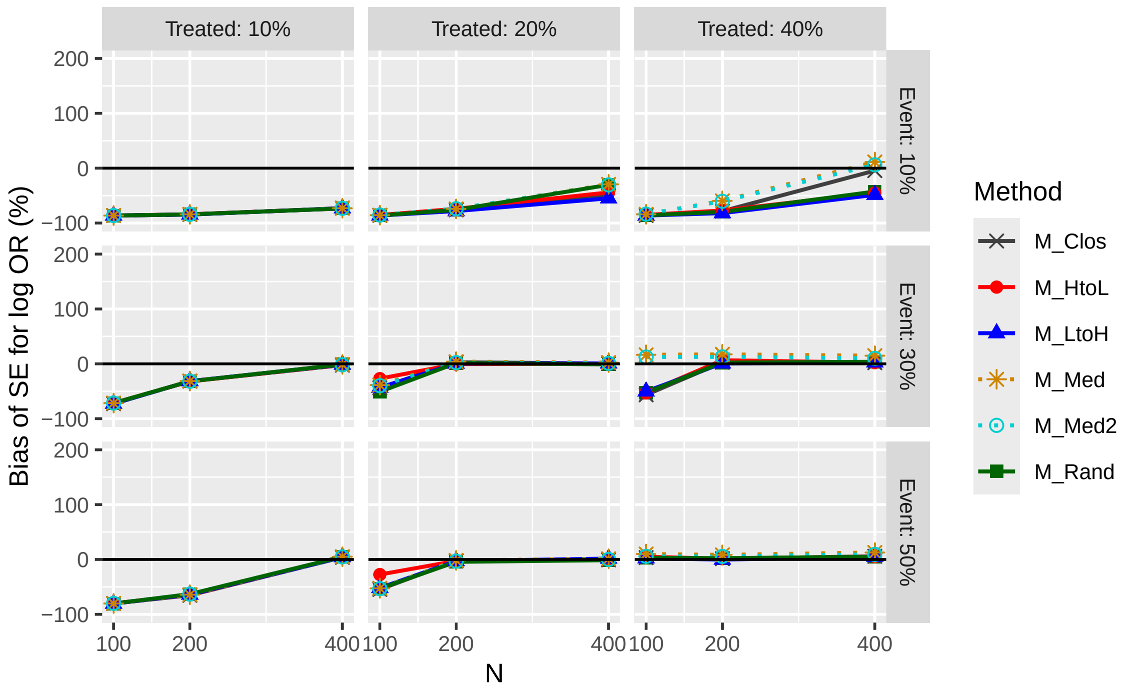

Figure S760. Mean bias of standard error for log odds ratio (multimodal continuous covariate, matching ratio 1:1, true OR: 0.5, c statistic: 0.85, robust inference).

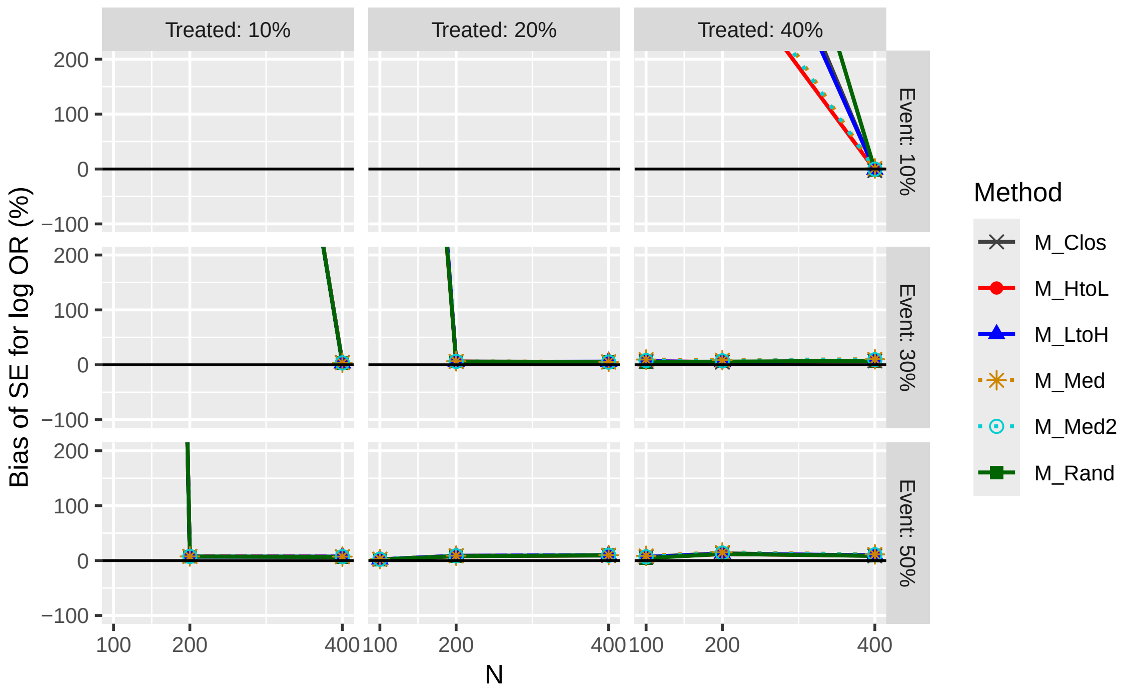

Figure S761. Mean bias of standard error for log odds ratio (multimodal continuous covariate, matching ratio 1:1, true OR: 0.5, c statistic: 0.6, naive inference).

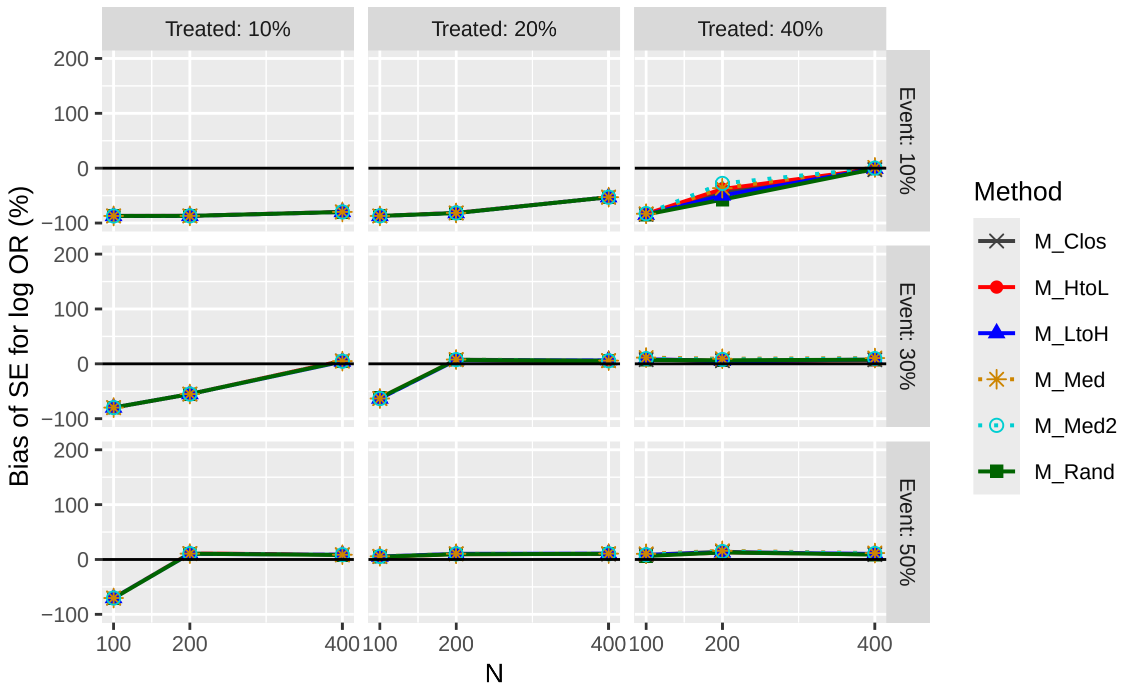

Figure S762. Mean bias of standard error for log odds ratio (multimodal continuous covariate, matching ratio 1:1, true OR: 0.5, c statistic: 0.6, robust inference).

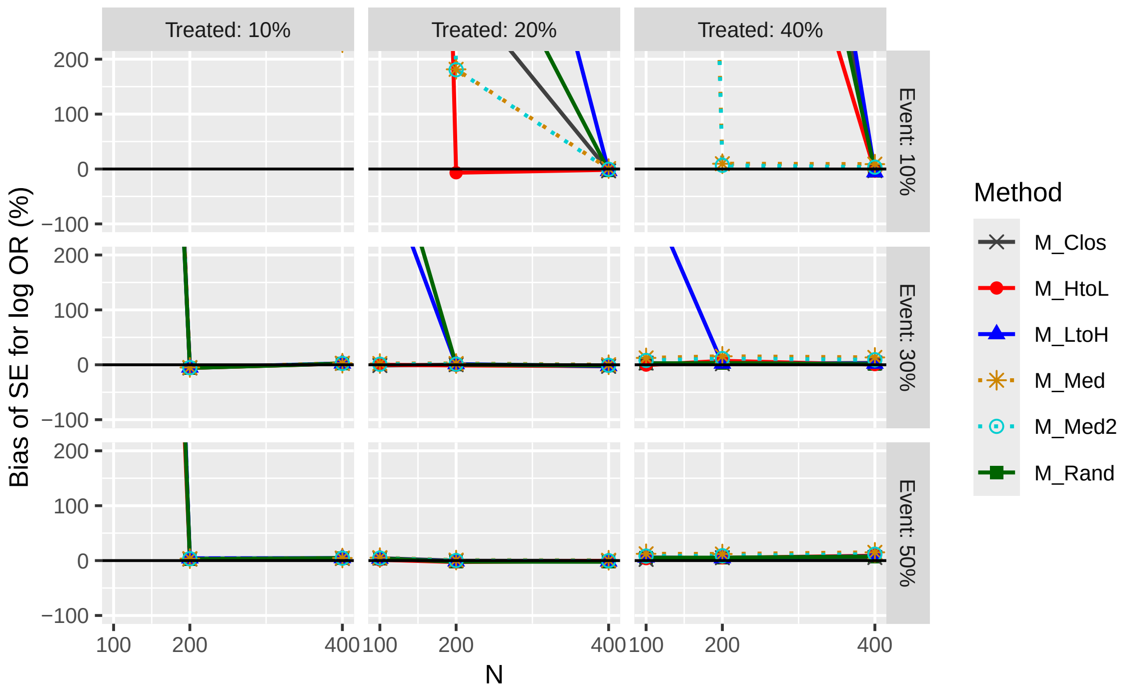

Figure S763. Mean bias of standard error for log odds ratio (multimodal continuous covariate, matching ratio 1:2, true OR: 1, c statistic: 0.85, naive inference).

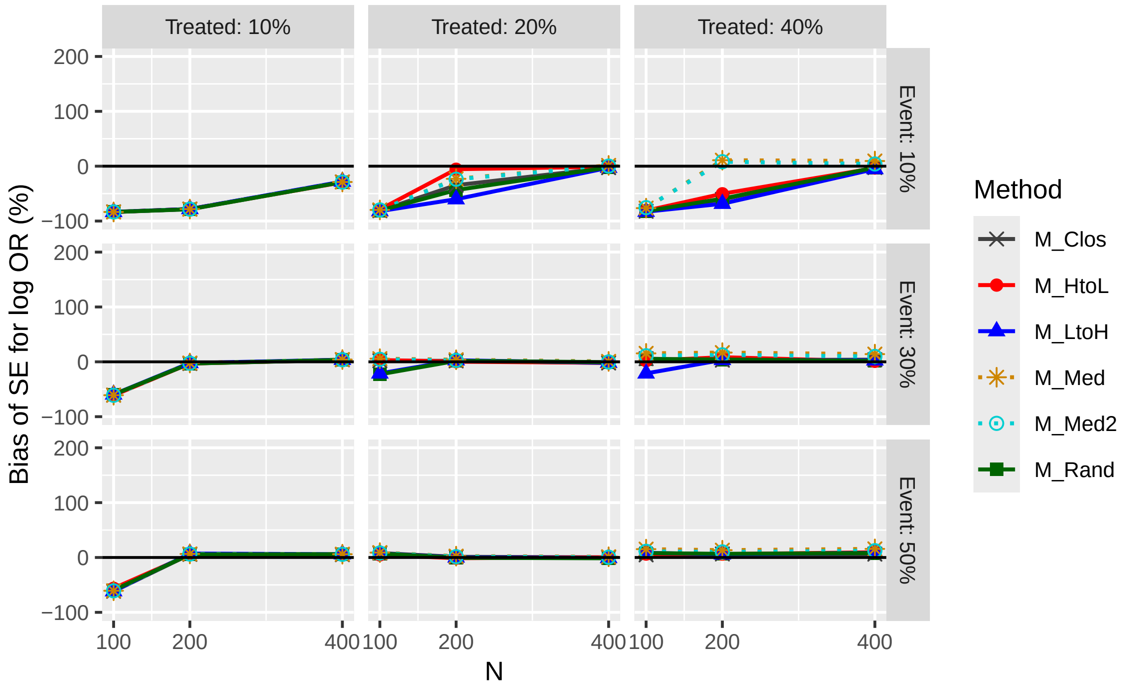

Figure S764. Mean bias of standard error for log odds ratio (multimodal continuous covariate, matching ratio 1:2, true OR: 1, c statistic: 0.85, robust inference).

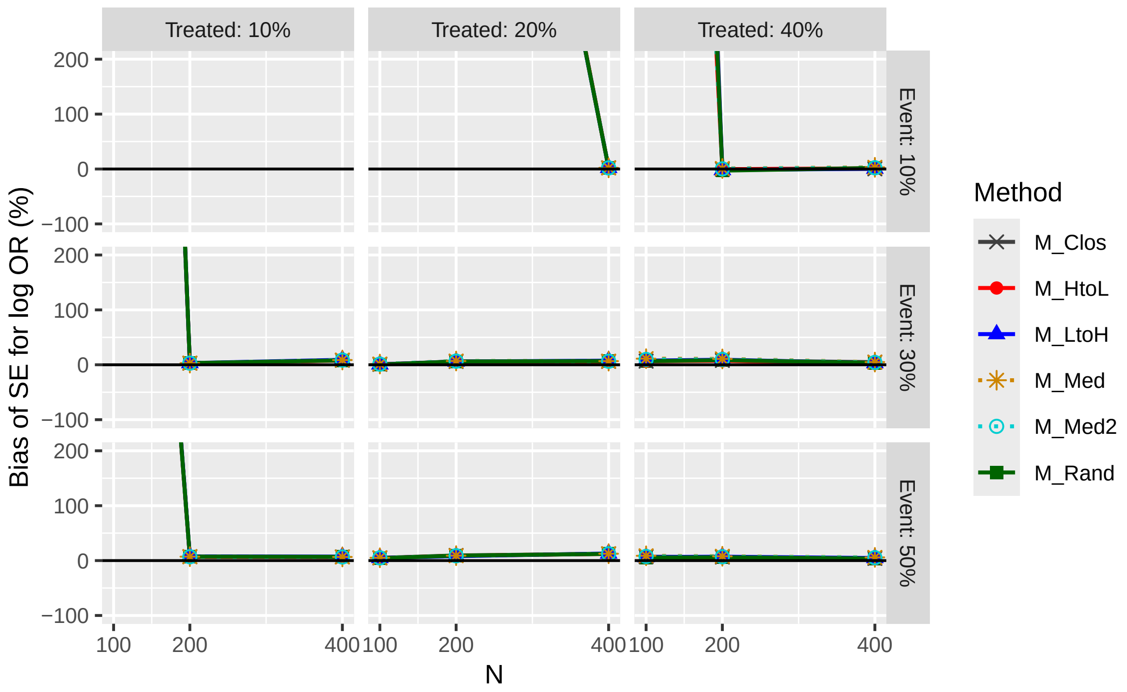

Figure S765. Mean bias of standard error for log odds ratio (multimodal continuous covariate, matching ratio 1:2, true OR: 1, c statistic: 0.6, naive inference).

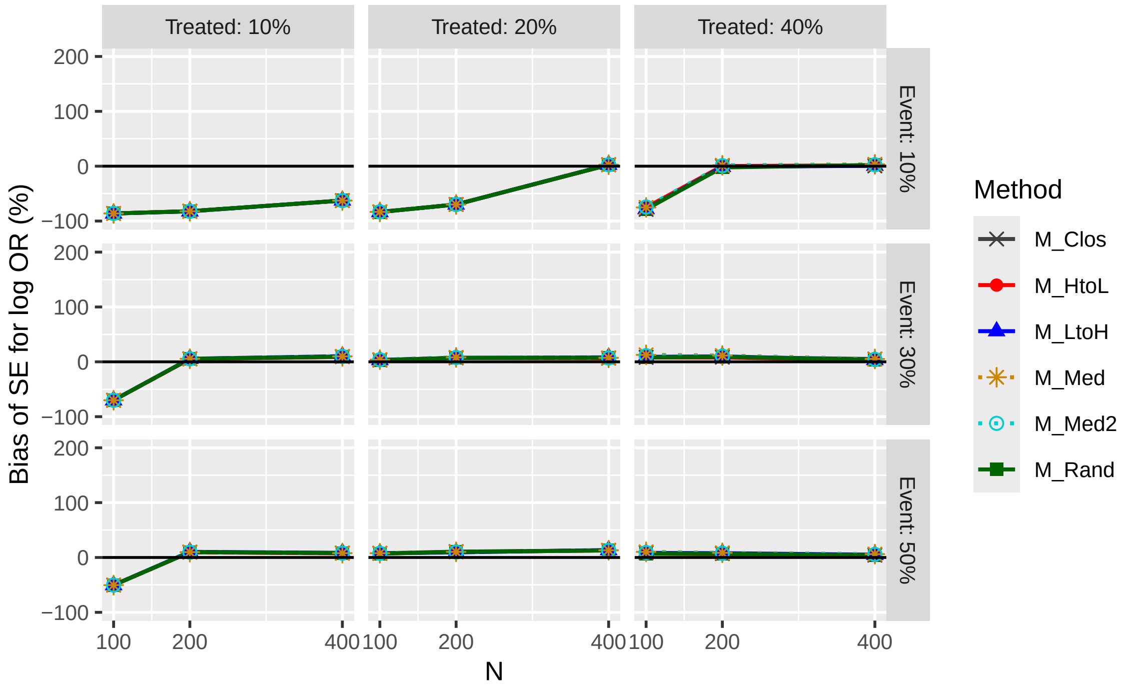

Figure S766. Mean bias of standard error for log odds ratio (multimodal continuous covariate, matching ratio 1:2, true OR: 1, c statistic: 0.6, robust inference).

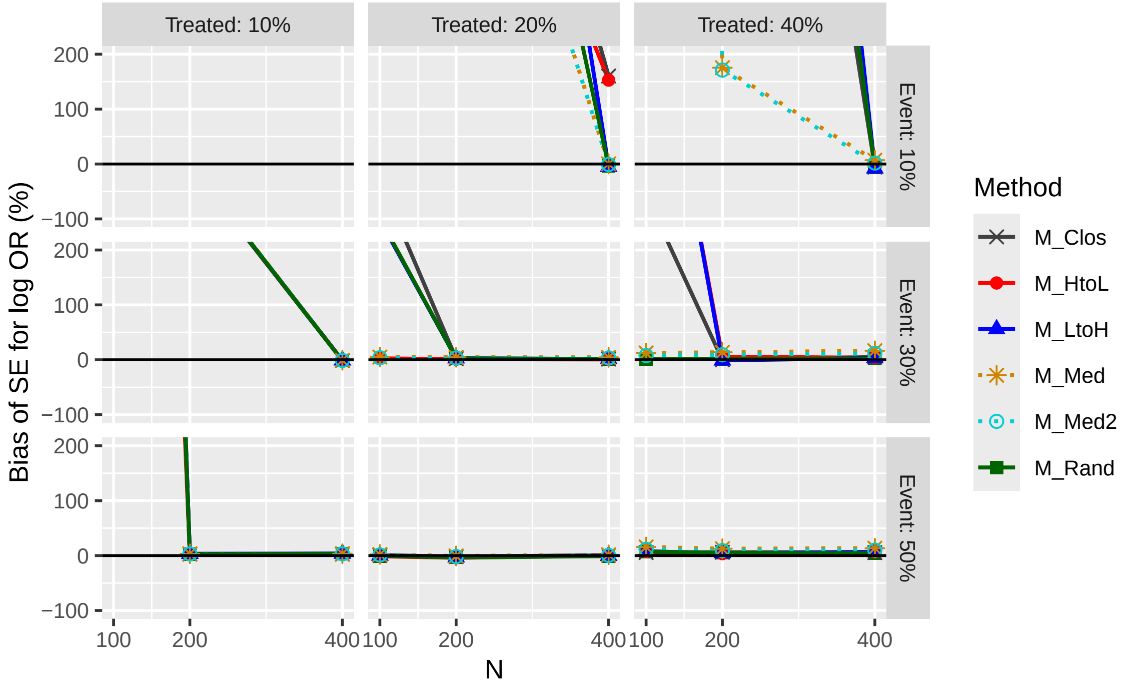

Figure S767. Mean bias of standard error for log odds ratio (multimodal continuous covariate, matching ratio 1:2, true OR: 0.75, c statistic: 0.85, naive inference).

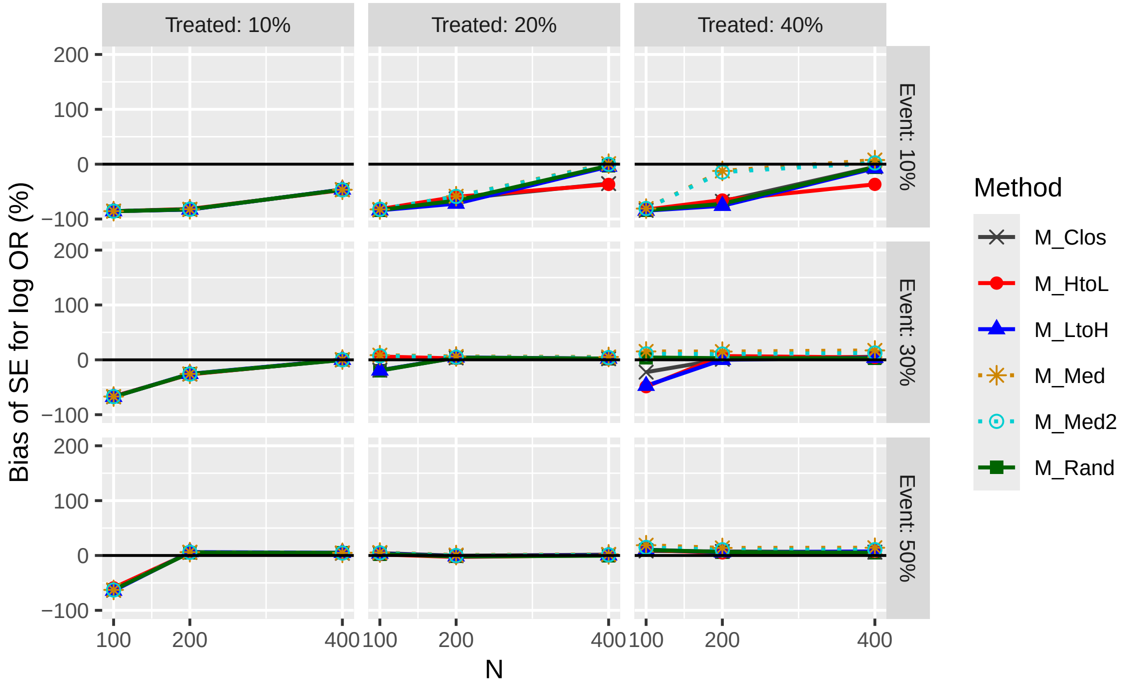

Figure S768. Mean bias of standard error for log odds ratio (multimodal continuous covariate, matching ratio 1:2, true OR: 0.75, c statistic: 0.85, robust inference).

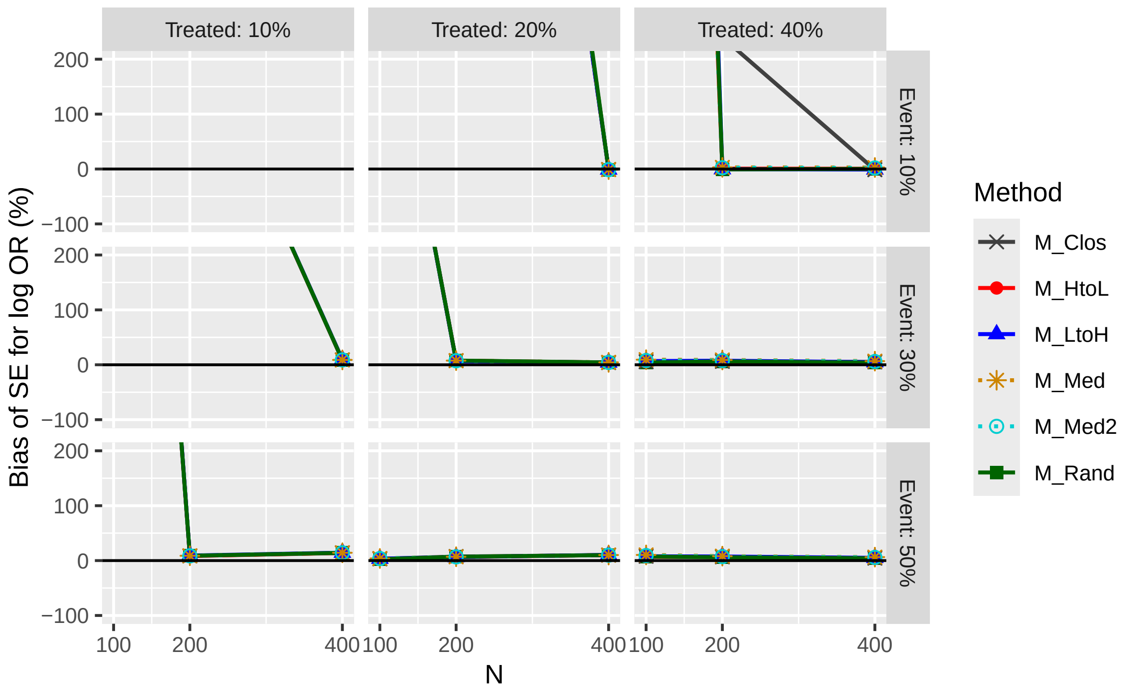

Figure S769. Mean bias of standard error for log odds ratio (multimodal continuous covariate, matching ratio 1:2, true OR: 0.75, c statistic: 0.6, naive inference).

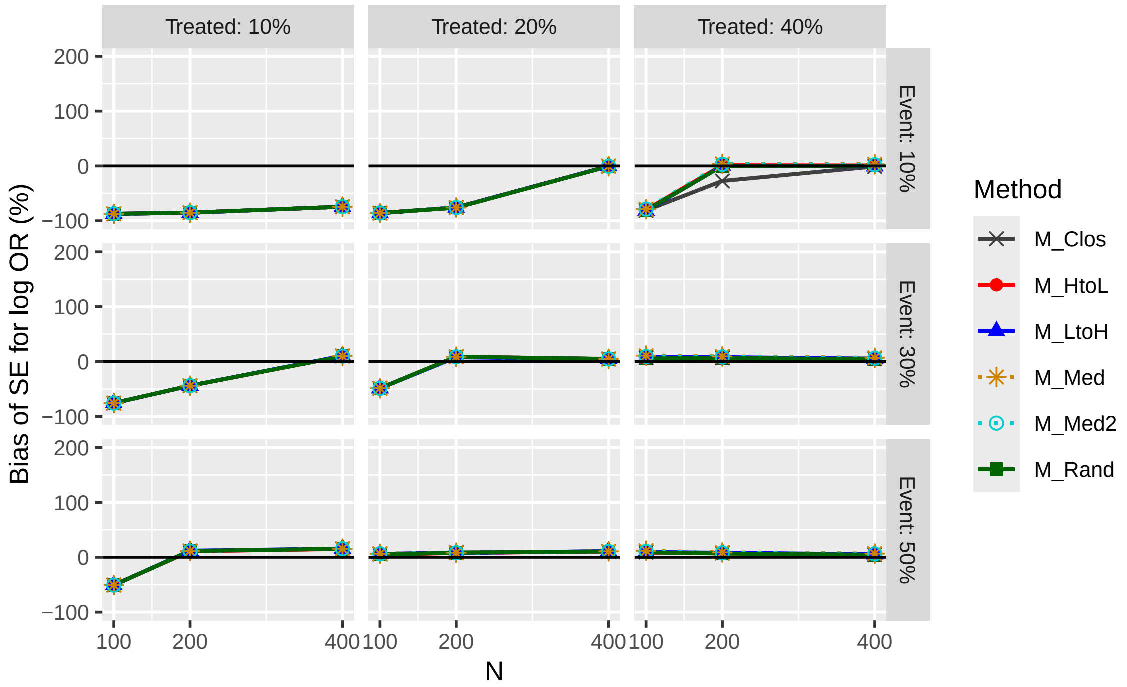

Figure S770. Mean bias of standard error for log odds ratio (multimodal continuous covariate, matching ratio 1:2, true OR: 0.75, c statistic: 0.6, robust inference).

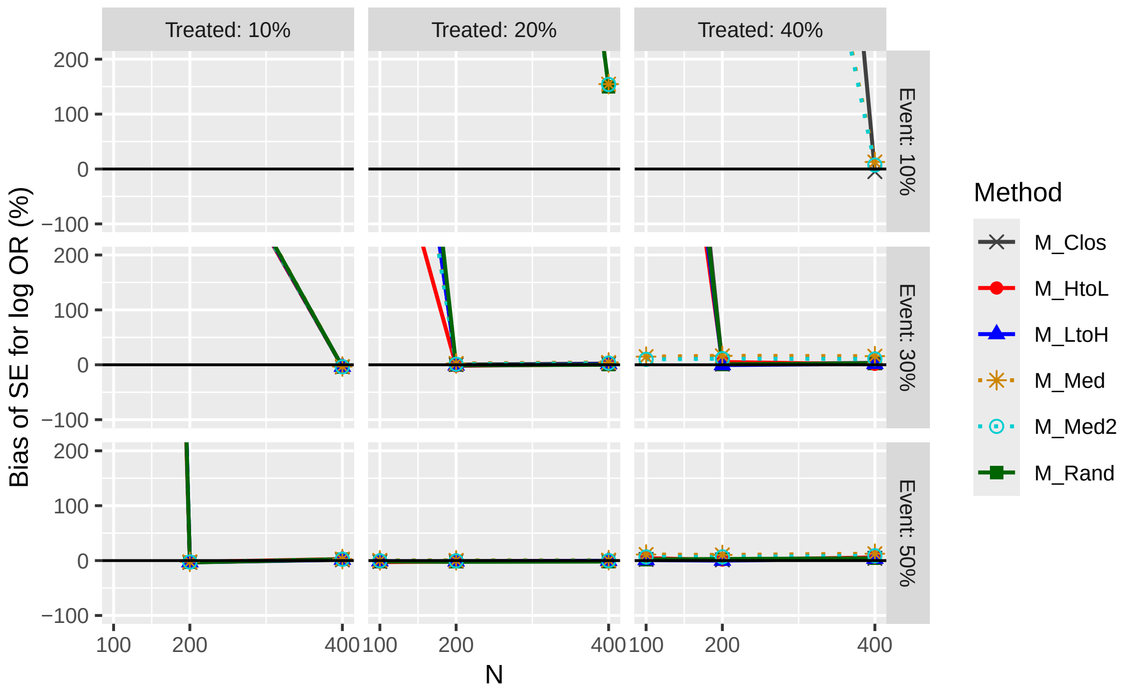

Figure S771. Mean bias of standard error for log odds ratio (multimodal continuous covariate, matching ratio 1:2, true OR: 0.5, c statistic: 0.85, naive inference).

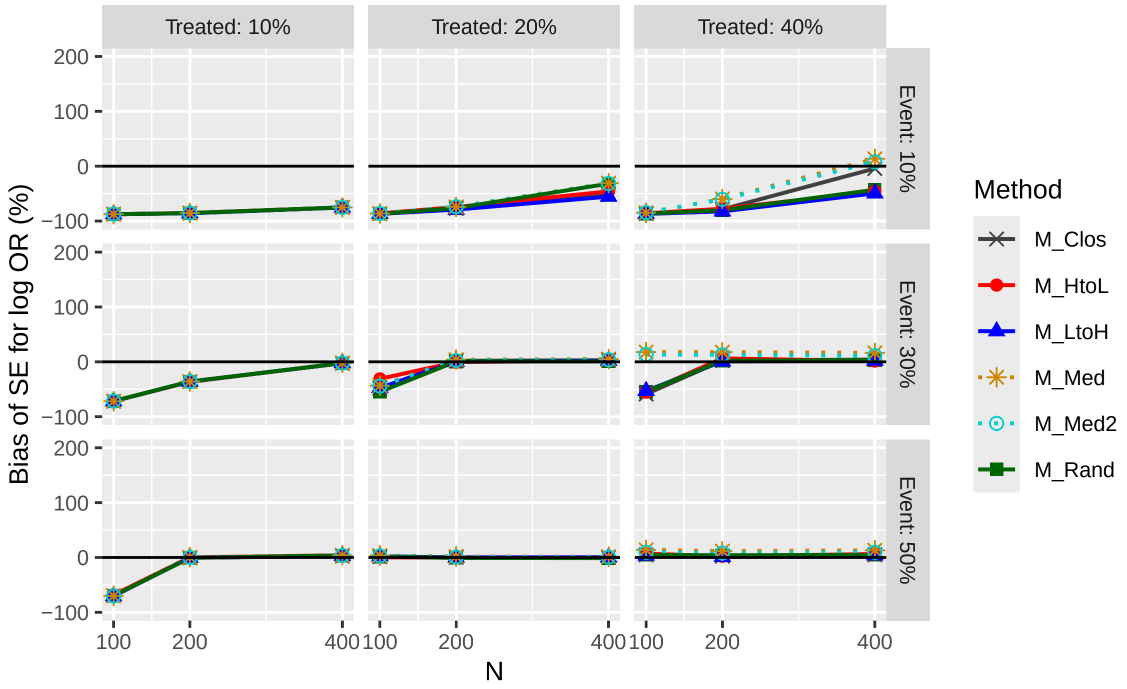

Figure S772. Mean bias of standard error for log odds ratio (multimodal continuous covariate, matching ratio 1:2, true OR: 0.5, c statistic: 0.85, robust inference).

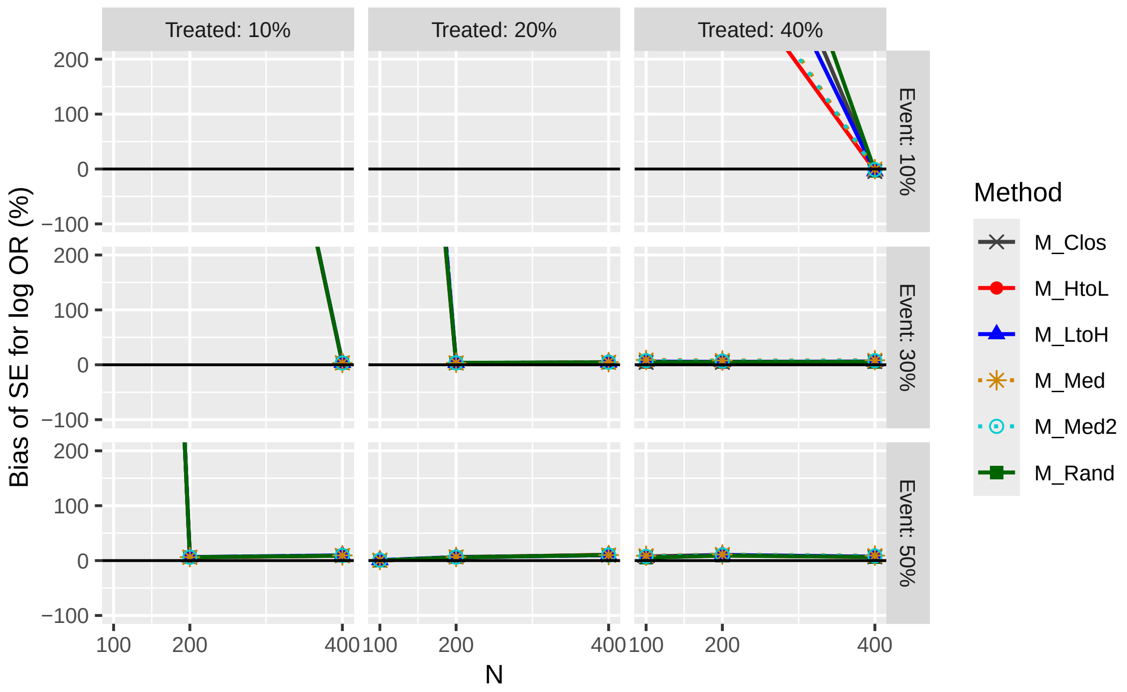

Figure S773. Mean bias of standard error for log odds ratio (multimodal continuous covariate, matching ratio 1:2, true OR: 0.5, c statistic: 0.6, naive inference).

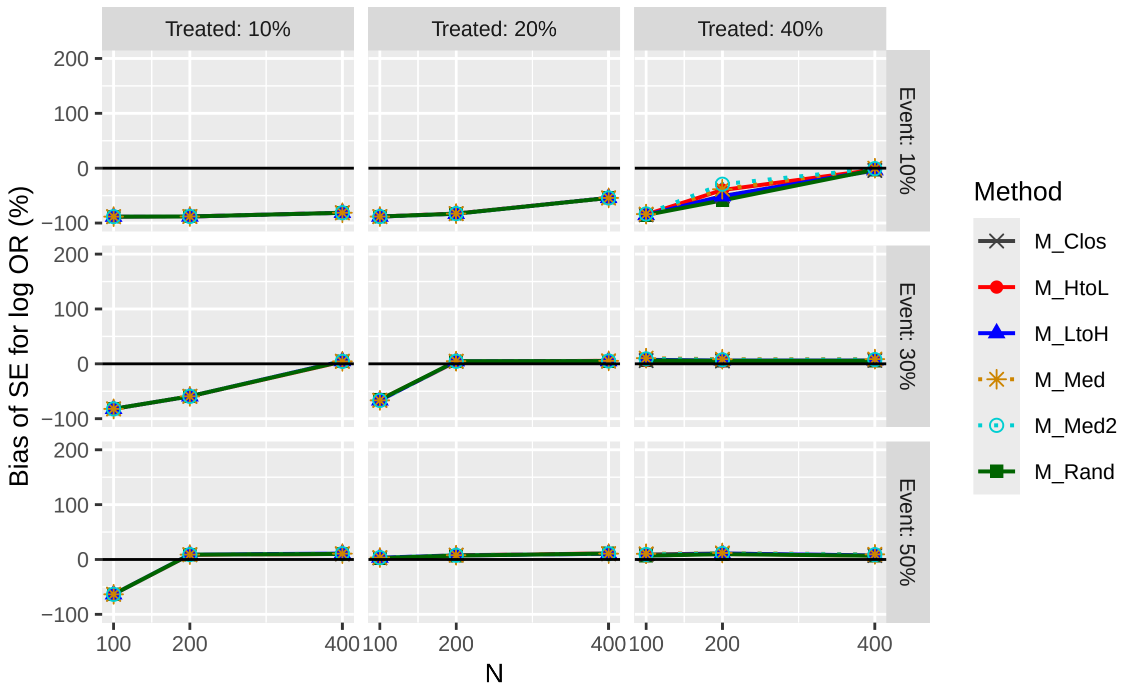

Figure S774. Mean bias of standard error for log odds ratio (multimodal continuous covariate, matching ratio 1:2, true OR: 0.5, c statistic: 0.6, robust inference).
