# Supplementary material for: Instability of estimation results based on caliper matching with propensity scores
Source: PLoS One. 2025 Jun 6;20(6):e0325317. doi: 10.1371/journal.pone.0325317 (PMC12143538; doi:10.1371/journal.pone.0325317)
Supplement: Attachment — Submitted filename: Response_to_reviewers.docx [file pone.0325317.s004.docx]

**Response to Editor**

We deeply appreciate the Editor’s insightful comments. Your comments have helped us to improve the quality of our manuscripts. We have responded to each comment as follows and believe that these responses meet the Editor’s requirements.

1. What if you repeat the analysis multiple times and pool the estimate? This idea is analogous to the analysis technique in multiple imputation literature. Repeating the analysis multiple times and pooling the estimate could solve the instability of the problem.

**Response:** Thank you for the insightful suggestion. We have considered a method similar to multiple imputation (MI) based on the mean but decided that it is not feasible to implement the idea at this time for the following reasons: 1. integrating multiple imputations is challenging because significantly large point estimates and/or SEs are often obtained, particularly when the number of events is small; 2. the variance estimator is typically increased due to the between-imputation variation in the ordinary MI method; however, in this case, it is expected to be decreased, necessitating the construction of a new theory must be constructed.

The median of 100 random matches provided in the manuscript serves as a replacement for your suggestion. Theoretically, as you indicated, we could improve the precision of the estimation. However, because we used a CI based on matched data, where the OR equals the median, the CP became more conservative than intended. We have described this as an issue for future research. This also addresses comment 6.

2. When comparing the methods, the authors should explain why they used a non-collapsible effect measure. Additionally, methods should be compared in terms of bias, model-based standard error (SE), empirical SE, and coverage probability.

**Response:** We acknowledge the importance of interpreting the non-collapsible effect with caution. However, logistic regression remains widely used as a method for analyzing binary data, and it is essential to provide information to its users. We have added these to the introduction as follows. “We primarily focused on the odds ratio based on logistic regression as an effect measure. Although this odds ratio is a non-collapsible effect measure (e.g., see Whitcomb and Naimi [8]) that requires careful interpretation, it remains widely used and is the primary focus of this study.”

We have added model based and robust (empirical) CIs in accordance with your comment. Mean bias is addressed by median bias instead, primarily for the reason stated in response to comment 1 (OR might become significantly large). For the same reason, and furthermore, because SEs are rarely provided in OR inference, only CIs are evaluated for precision.

3. The simulation settings are very unrealistic. Please explain why specific parameter values (e.g., c-statistic, odds ratios) were chosen and how they relate to real-world scenarios. It would be best to use an established simulation setting.

**Response:** We apologize for not fully explaining the intent of our simulation settings. The simulation settings were designed to cover several situations, although they could not cover all situations. We have added extensive explanations of the intent of the simulation settings.

4. In continuation of my previous comment, it seems unrealistic to have sample sizes of 100 and 200. It would be desirable to have at least one simulation scenario with parameters similar to those of an established simulation setting. I am backing this approach because an overfitted PS model could lead to a biased effect estimate and may not be suitable for comparing methods in a simulation study. A better approach could be to consider a well-calibrated model, ensuring that there is at least one scenario where all methods are comparable. On top, given the small sample size with or without a rare event, it is surprising that the authors did not consider 1:m or full matching. 1:1 matching is ideally not recommended with a rare exposure or a rare event.

**Response**: As you indicated, we have added 1:2 matching. While we agree that superior methods exist for small samples, matching is commonly performed in nonrandomized clinical studies with such sample sizes or at the request of clinicians. Thus, we included this setting to illustrate the outcomes of caliper matching on a small sample. In addition, the sparsity problem in small samples was added in the final section as a limitation of this study as follows:

“Although the simulations in this study have been performed in situations where the number of expected events is considerably low, there are other methods specialized for sparse situations (e.g., see Gosho et al. [20]).”

5. The reason for considering 10,000 simulations instead of 1,000 was unclear. Also, how did the authors come up with a repeated sample of 100 to calculate the median?

**Response:** The number of simulations was set based on feasibility in our computing environment to increase the precision of the results. However, the implementation of the confidence intervals suggested in the comments considerably increased the computational load, and consequently, the number of simulations was changed to 1,000 (100 times for the same reason). This was added to the discussion section as follows:

“The median of 100 random matching iterations was provided in our simulations. This number was chosen because of the computational load in the simulations; however, by analogy with the performance of the multiple imputation method, it should provide results with sufficient precision. However, in actual data analysis, a larger number (e.g., 1000 iterations) may be used.”

6. It is not clearly explained why the median was chosen as a summary measure, as calculating standard error for the median is always complex.

**Response:** We would appreciate it if you could refer to our response to comment 1. Additionally, the following was added at the end of Section 2: “Odds ratios may increase significantly when the denominator odds are close to zero. However, even if a small number of extreme estimates are included among multiple random matches, the median remains less sensitive to them."

7. It was not clear how small additions and/or changes in data worked in the case study.

**Response:** Following the suggestions of other reviewers, the data were changed, and additional small data (10% addition) was also considered for further analysis.

8. Also, the explanation for the case study is insufficient. Please provide a brief rationale in the introduction section for selecting the low-birth-weight data, how it exemplifies the instability problem, and the motivation for the proposed comparisons based on the case study dataset.
9. There should be a discussion on the case study results, particularly how they compare to the findings from the simulations.

**Response to comments 8 and 9:** We have enhanced the case study motivation and the contrast with the simulation setting, as you suggested.

10. Limitations should be discussed such as unmeasured confounding, and other issues in observational data analyses

**Response:** In addition to the unmeasured confounding issue you indicated, we have added several limitations in the last paragraph, including the sparsity issue.

**Response to Reviewer #1:**

We deeply appreciate the Editor’s insightful comments. Your comments have helped us to improve the quality of our manuscripts. We have responded to each comment as follows and believe that these responses meet the Editor’s requirements.

- Overall, the paper is crisp and the problem nicely stated and elaborated. I only have severe concerns with the chosen data example, which is exactly the kind of study where propensity methods should not be used.

**Response:** Thank you for your helpful comments. We have changed the dataset from patients with breast cancer in Section 4.

- At some places it seems that the translation into English failed (e.g. Line 263 "paired data that return the median" what does it mean?). See my specific concerns below.

**Response:** We have requested an English proofreading company to proofread our paper.

- Introduction: The authors should give a better motivation for propensity score caliper matching (as opposed to other methods that are less dependent on a stochastic algorithm such as covariate adjustment for propensity score or inverse probability of received treatment weighting). If there are better methods around, why should we take care of another deficiency of PS matching that does not exist with covariate adjustment or IPTW?

**Response:** Despite various problems associated with matching, it is frequently used in observational studies because of its ease of interpretation and implementation for clinicians. This is why we focused on matching. The relevant part of the Introduction has been revised: “Among them, propensity score matching is frequently used in medical and social research articles because of its ease of interpretation and implementation for clinicians [3-6] despite the issue of reduced sample size due to matching.”

- Line 47: the instability problem may not be visible, but different researchers may set different seeds which leads to different results despite full prespecification even if they used the same data set. Maybe this could be mentioned here.
  Line 114: consider rewriting the sentence "As a modified version of W ATT, a method that excludes more than the 95th percentile of the distribution of propensity scores was also applied (W_ATTt) (Lee et al.)" Does it mean that subjects with propensity scores greater than the 95th percentile were excluded?

**Response:** We have revised this in the revised manuscript.

- Line 185: what does it mean '... except when the number of matched pairs was covariates'?

**Response:** This part was an editing error and has been deleted.

- Lines 208ff Case study. While I like the exposition and the simulation study, I don't think it is wise to use propensity score matching for a risk factor. Moreover, in this example the exact causal ordering of the measured variables is not known (it is unclear if the 'pretreatment' condition is fulfilled for all considered confounding variables). The authors should consider showing an example with a proper intervention variable. If the paper is published, other authors will apply PS matching to similar questions and we should generally discourage from using PS methods with non-interventions, in particular if standard assumptions for causal inference are in doubt. A more proper example can be found, e.g., in the paper of Luijken et al (2024, Biometrical Journal) and the accompanying GitHub repository in the folder https://github.com/KLuijken/CI_CovSel/tree/master/rcode/add-ons/simulated_CABG_example. These authors emulated the data of a real observational study with an intervention using R code, which can be found on the GitHub repository.

Line 219: " the odds ratio for smoking variables ..." I assume the authors mean the odds ratio of smoking vs. not smoking. But anyway, I strongly propose to change the example.

**Response:** We have changed the case study to a case study on GBCS data to be consistent with our simulations. We hope that responds to your comment.

- Lines 241ff: I don't quite agree with the conclusion that any of the methods (HtL or LtH) should be recommended. In fact, matching is still arbitrary and not 'wrong' if performed with a different ordering. Hence, as already stated in my comment on line 47, even if the matching algorithm becomes deterministic when using a predefined ordering, it is still one of several possible matchings that are all in line with the caliper requirement. There is no scientific reason to prefer HtL to LtH.

**Response:** The corresponding part has been deleted in accordance with the comment.

- Line 246 & 260: When the authors recommend to use multiple random order matching and report the median, did they investigate the coverage rates of 95% confidence intervals based on the median OR? I would assume that there is some undercoverage if one picks a matching and ignores the variability of matching. Hence I would be more cautious than saying 'there seems to be no major problem' (line 262). The authors could easily add an evaluation of coverage rates at least for the methods which have (almost) no bias. Or they should tone down their conclusion.

**Response:** The simulations for CI were added; M_Med provided a conservative CI, which was added as a future research topic in the final paragraph.

- The major problem with matching is the number of subjects that cannot be matched, which are lost from the analysis. Hence, one should probably better using weighting or covariate adjustment in practice in order not to throw away precious information.

**Response:** Based on your comment, we have added the following to the Discussion: “To avoid the instability problem, weighted methods, particularly the overlap weighting method, can be used from a performance perspective (see Supplemental Material). The weighting methods may also avoid the problem of subject information being lost that may occur during matching.”

- Figure S4: there are no lines for OR=0.5 and OR=0.75. Please check or add explanation on overlaying curves.

**Response:** Based on your suggestion, we have incorporated the following: “In the absence of calipers, the median width was considerably narrower than that of the caliper matching, resulting in overlapping lines for each condition in the graph (S1).”

**Response to Reviewer #2**

We deeply appreciate the Editor’s insightful comments. Your comments have helped us to improve the quality of our manuscripts. We have responded to each comment as follows and believe that these responses meet the Editor’s requirements.

1. In the Introduction section, the authors mention that cherry-picking is easy to conduct in this type of matching. Is this problem very extended in the literature? Do the authors have access to any bibliography regarding the cherry-picking issue in this type of studies, and/or how effective can it be? If so, I would recommend to include it in this section to give a measure of the magnitude of the problem.

**Response:** Currently, the only previous study that discusses the problem of cherry-picking in the matching order is Komen et al. [7], which is our motivation to investigate the instability of random order matching in small-to-medium sample sizes. We have added an additional explanation of how to specifically evaluate instability and how that relates to cherry-picking in the introduction section: “Specifically, we evaluate the degree to which the analysis results may vary using random order matching, that is, the instability of the analysis results, under various situations. This instability reflects the range of analysis results that can be cherry-picked.”

2. The greedy nearest neighbor matching technique should be described in more detail in Section 2, apart from introducing the four types of sorting available in literature.

**Response:** We have added the following part to Section 2: “Greedy nearest neighbor matching first selects a treated subject and identifies the untreated subject with the closest propensity score as a matched control subject. In the caliper matching, treated and untreated subjects are matched only if the absolute difference in their propensity scores is within a prespecified maximal distance (the caliper distance).”

3. Page 6, lines 102-103: “multinomial distribution with 10 categories” What were the probabilities for each of the 10 categories in this distribution? Were they uniform or unequal?

**Response:** We apologize for the insufficient information. We have added the following information for the parameter: $\boldsymbol{p}=\left( 1,2,3,4,5,5,4,3,2,1 \right)^{T}/30$, which was set with the intention of generating a tied propensity score.

4. Page 7, lines 106-107: “represented a case where the propensity in treatment selection was close to random or where there was remarkable propensity” Respectively? 0.65 in the c-statistic is close to random and 0.85 remarkable propensity? Please clarify.

**Response:** We apologize for the insufficient description. We have added the following:

“The c-statistic ranges from 0.5 to 1. If it is considerably close to 0.5, it is equivalent to a randomized controlled trial, and if it is substantially close to 1, the treatment selection is deterministic, which may make any confounding adjustment less meaningful. Therefore, the c-statistics of 0.6 and 0.85 were selected as cases of weak and strong confounding influence, respectively, within realistic and useful ranges.”

5. I understand that the simulation has been done considering that the outcome variable is binary. Would the results be different if the outcome variable was continuous? I think this point should be at least discussed in the manuscript, as continuous outcomes are relatively common in observational studies.

**Response:** We agree that continuous outcomes are equally important. We have described the following in the limitation part: “Although this study focused on binary outcomes, similar problems would likely arise for continuous and survival outcomes. Similar results are expected, particularly for ratio indices such as hazard ratios. Future studies could explore other outcomes using similar investigations.”

6. Page 9, lines 153-155: “We added 0.1N (10%) of the data to each simulation, and the absolute difference between the odds ratio of the added merged data and that of the original data and the median of the absolute difference were calculated.” Could the authors clarify in the text what was the purpose of this procedure?

**Response:** Based on your comment, we have added the following: “This data aggregation process was designed to evaluate the instability of the analysis results when multiple analyses are performed as data accumulate in the same study, as described in the Introduction.”

7. I think the graphics in the Results section (and the Supplementary Material) would benefit from redefining the limits of the Y axis. I find that, in many cases, the span of the axis is very wide and that makes the lines to be too “crushed” and barely distinguishable from each other, and this could be alleviated by readjusting the amplitude of the Y axis. I would recommend trying to redo the graphics if the authors are able to do so.

**Response:** Based on your comment, the axis ranges were narrowed for median bias and IQR. However, the axes of figures for the median width and the 10% data addition have been maintained to show the results of the M_Rand method. We would appreciate your understanding.

8. Page 11, lines 178-180: “For the weighting based analysis method, only the results of the ATT weight-based method are presented in the text because it is one of the most frequently used methods.” I find that W_ATO and W_ATTt showed good performance according to the graphics presented in the supplementary file. Could this statement be revised?

**Response:** As you indicated, it is suggested that W_ATO and W_ATTt perform more efficiently than W_ATT. However, since the main goal is to evaluate performance by matching order, only the most major ATT weights are included in the main graph as references. Nonetheless, the performance evaluation of the weighting methods was also properly conducted and included in the Supplemental Material, with a reference in the main text. We would appreciate your understanding of our approach.

9. I think the authors should include more details about the data used and its origins in the Case Study section, even if it is a dataset taken from an R package. Knowing more details would help the reader to know the quality of the observational study conducted and the expectations they should have regarding propensity adjustments.

**Response:** We have added a reference that provides more details on the data.

10. Is there any possibility to assess the actual bias of the estimations from the case study? Are the authors aware of any data on real randomized experiments on this matter?

**Response:** Unfortunately, discussion for bias is difficult because the true values are unknown. However, following the comments from other reviewers, we have changed the data to those of nonrandomized interventional research and established multiple situations. We have also added a discussion of instability with additional data. We believe this revision significantly increases the value of the Case Study section.

11. Page 6, line 99: “p gj” I think it should read as “p_{gj}”

**Response:** We have corrected it in the revised manuscript.

12. Page 14, lines 233-235: the verb tense should be changed in this sentence (i.e. every “was” should be replaced by “is”).

**Response:** We have corrected it in the revised manuscript.
