# Supplementary material for: Instability of estimation results based on caliper matching with propensity scores
Source: PLoS One. 2025 Jun 6;20(6):e0325317. doi: 10.1371/journal.pone.0325317 (PMC12143538; doi:10.1371/journal.pone.0325317)
Supplement: Attachment — Submitted filename: Response_to_Reviewers_2nd.docx [file pone.0325317.s005.docx]

Response to the second peer review comments on “Instability of Estimation Results Based on Caliper Matching with Propensity Scores”

Kazushi Maruo, Yusuke Yamaguchi, Ryota Ishii, and Masahiko Gosho

***Response to Editor***

We deeply appreciate your comments, which have helped us to improve our manuscript. Our responses to each comment are listed below. We hope that our revisions are sufficient to meet your requirements.

**Comment:** Pooling the estimate: It was proposed to pool the estimate and calculate SE to reflect the improved uncertainty due to polling. There should be at least one comparison of how the proposed method works compared to pooling the estimate. Here is an example where the authors used the modified multiple outputation technique to account for within and between variations in the estimate for improving precision (doi: 10.1007/s12561-024-09461-6).

**Response:** In accordance with your comment, we added the modified multiple outputation technique (referred as M_Med2) proposed by Hossain et al. (2024). We appreciate your guidance, which helped us recommend this technique.

**Comment:** The authors should consider a collapsible effect measure and compare the methods in terms of bias, model-based standard error (SE), empirical SE, and coverage probability. The justification for not doing these was insufficient and unreasonable (doi: 10.1002/sim.8086).

**Response:** In accordance with your comment, we added the percentage bias of model based and empirical SEs. Because extremely large estimates of the log odds ratio can distort the evaluation, we limited the evaluation to cases where the estimated log odds ratio was less than or equal to 10.

***Response to Reviewer 3***

We deeply appreciate your comments, which have helped us to improve our manuscript. Our responses to each comment are listed below. We hope that our revisions are sufficient to meet your requirements.

**Comment 1:** line 160: "the expected number of events was one for each group" is this a realistic scenario for any type of analysis? I am struggling to think of an area of study where we can do any serious sort of analysis with a single event, especially in the context of a frequentist statistical framework. In situations like this I would think that the matching order of the propensity scores is pretty far down on the list of major issues. I would think that this is more than a very small information setting, to the point where the issues this paper are discussing almost become moot due to the severity of the violation of other statistical assumptions. I think maybe a bit more explanation regarding the explicit purpose of modeling such a scenario may help readers better understand the purpose of using such an extreme simulation setting.

**Response:** In accordance with your comment, we described the intent of the setting as follows:

“In such scenarios, the asymptotic assumptions underlying statistical inference are entirely violated. This setting was deliberately designed to highlight the potential pitfalls of applying matching methods under conditions of extreme data sparsity.”

**Comment 2:** At several points in the manuscript the idea of conducting multiple analyses as more data are accumulated is discussed. This is an interesting area that is receiving more attention recently in the clinical trial literature (adaptive clinical trials). My concern with this is the potential for the potential inflation of Type I error and cherry-picking results/p-hacking that can arise from this stream of methodological choices if not conducted carefully and transparently reported (preferably a priori). While this is more of a practical concern not directly relevant to the simulation per se (as the true effects are known) I think it may be worthy of adding some language surrounding this concept cautioning the potential issues surrounding this approach.

**Response:** In accordance with your comment, we added this issue in the Discussion section as follows:

“Although the issue of multiplicity in statistical testing was not addressed in the present study, it is important to recognize the risks associated with conducting multiple statistical tests across repeated analyses with additional data, particularly in the context of confirmatory clinical studies.”

***Response to Reviewer 4***

We deeply appreciate your comments, which have helped us to improve our manuscript. Our responses to each comment are listed below. We hope that our revisions are sufficient to meet your requirements.

**Comment:** The aim could be stated more succinctly at the beginning to ensure the reader understands the problem immediately.

The introduction could benefit from a brief discussion of how the findings will address real-world applications or improve current practices.

The introduction could briefly outline why the chosen design (simulations and case studies) is most suitable for evaluating instability.

Briefly justify the choice of simulation studies as the primary method to explore instability and validate findings through case studies.

**Response:** In accordance with these comments, we added the paragraph (mentioned below) in the Introduction section. As it is difficult to present the objectives without initially defining the instability in matching, a summary of objectives was presented after the definitions. The justification of the simulation conditions are described in detail in Section 3.1.

“In summary, this study has two primary objectives: first, to evaluate the instability of matching procedures in observational studies with small to moderate sample sizes and binary outcomes; and second, to provide practical guidelines for selecting the most appropriate matching method. These objectives were addressed through simulation studies and a case study designed to reflect the characteristics of the problem setting. The simulation settings were carefully constructed to capture a wide spectrum of real-world scenarios. The alerts and recommendations presented in this study aim to assist readers in selecting an appropriate matching procedure with respect to instability.”

**Comment:** Revise for conciseness and grammatical clarity to improve readability. Some sentences are verbose or contain minor grammatical inconsistencies, such as "especially, particularly for a non-large sample" and "no research for variability in propensity score analysis due owing to multiple analyses."

**Response:** We have rewritten each as follows:

“especially for small-to-medium sample sizes”

“no studies have examined the variability in propensity score analyses arising from multiple analyses”

**Comment:** Quantify or provide examples of how variability due to data accumulation affects Methods 1-3 compared to random order matching.

**Response:** In accordance with your comment, we have added the following in Section 2 (Greedy nearest neighbor matching):

“Let us consider the influence of the matching algorithm on repeated analyses conducted through data accumulation. Given that the propensity scores across successive datasets are correlated, the probability of the same pairs being matched in each analysis is relatively high for Methods 1–3 as they use deterministic matching procedures (e.g., Method 1 prioritizes pairing units with the highest propensity scores). In contrast, random matching reduces the probability of such overlap. This raises concerns about potential instability in the results across repeated analyses.”

**Comment:** Including following considerations in discussion section would provide a more balanced perspective:

1. The assumption of a unimodal propensity score distribution might not always hold in real-world data.
2. The exclusion of interaction effects or non-linear covariate relationships in the propensity score model could limit applicability to more complex datasets.
   Simulation design section:

**Response:** For comment 1, we have added a multimodal continuous covariate setting to address the next comment. For comment 2, we have added the following as a limitation of the study in the Discussion section:

“We focused exclusively on simple propensity score models and matching procedures commonly used by general statistical practitioners. The evaluation of more advanced methods—such as propensity score or outcome models incorporating complex structures (e.g., interaction terms), kernel-based matching, and machine learning approaches—remains a subject for future research.”

**Comment:** The unimodal distribution assumption for propensity scores may not always reflect real-world scenarios where multimodal distributions or non-normality are observed. A sensitivity analysis incorporating such variations could strengthen the conclusions.

**Response:** In accordance with your comment, we have added a multimodal continuous covariate setting. The results were not significantly different from the univariate continuous setting and were included in the Supplementary Material.

**Comment:** While different matching orders are evaluated, the robustness of caliper matching to outliers or extreme propensity scores could be explored further. For example, alternative caliper widths, kernel-based matching, or machine learning approaches for propensity score estimation might provide additional insights.

**Response:** First, a 15% caliper setting was incorporated in accordance with your comment. Additionally, the following statement has been added as a limitation in the Discussion section, as described above:

“We focused exclusively on simple propensity score models and matching procedures commonly used by general statistical practitioners. The evaluation of more advanced methods—such as propensity score or outcome models incorporating complex structures (e.g., interactions), kernel-based matching, and machine learning approaches—remains a subject for future research.”

**Comment:** “The risk of a large change due to data addition was higher for the Rand method.” Quantify “large change” to contextualize this risk.

**Response:** We have added as follows:

“Specifically, the impact of data accumulation on the odds ratio was small for all methods except the Rand method, with variations generally remaining close to zero and not exceeding ±0.2. In contrast, the Rand method exhibited a comparatively larger fluctuation, with changes reaching up to approximately 0.4.”

**Comment:** For the low birth weight data, the authors focus on a binary outcome and a single matching ratio. In contrast, the GBCS data are analyzed with different outcomes, sample sizes, and ratios. While this demonstrates versatility, the rationale for the differing approaches between datasets is not discussed. Clarifying this would improve coherence.

The c-statistic for the propensity score model in the low birth weight data is not reported. Given its importance in propensity score analyses, this omission should be rectified for consistency with the GBCS data discussion.

**Response:** The analysis for low birth weight data was removed from the manuscript based on another reviewer's comment.

**Comment:** The impact of adding 10% data on odds ratios is presented but not fully contextualized. For instance: Does the larger change in the Rand method suggest a lack of robustness, or is it expected behavior given its design? How does this finding translate into recommendations for researchers dealing with incomplete or expanding datasets?

**Response:** Our response to this comment is the same as the one provided in the comment above, with the following addition regarding the mechanism in Section 2 (Greedy nearest neighbor matching):

““Let us consider the influence of the matching algorithm on repeated analyses conducted through data accumulation. Given that the propensity scores across successive datasets are correlated, the probability of the same pairs being matched in each analysis is relatively high for Methods 1–3 as they use deterministic matching procedures (e.g., Method 1 prioritizes pairing units with the highest propensity scores). In contrast, random matching reduces the probability of such overlap. This raises concerns about potential instability in the results across repeated analyses.”

In addition, the following was added to the Discussion section:

“The simulation results also suggest that random order matching might produce excessive variability in the results when multiple analyses are performed as the data accumulate. The case study results also supported these findings.”

**Comment:** The recommendation to use 1,000 iterations for random order matching (instead of 100) is presented without sufficient justification. A brief discussion of how this choice balances computational load and result precision would strengthen this recommendation.

**Response:** In accordance with your comment, we have revised as follows:

“The median of 100 random matching iterations was provided in our simulations. This number was chosen because of the computational load in the simulations where many conditions and 1000 number of simulations were set; however, by analogy with the performance of the multiple imputation method, it should provide results with sufficient precision. However, in actual data analysis, the computational load of repetition would be almost negligible. For example, 1,000 or 10,000 iterations will not take too much time if the sample size is not too large.”

**Comment:** The discussion of unmeasured confounders is minimal. It would benefit from a brief exploration of how such confounding might exacerbate instability or bias results.

**Response:** In accordance with your comment, we have added the following sentence:

“While unmeasured confounders are known to introduce bias in the estimation of treatment effects, their impact on the instability remains unclear. As precision can be enhanced by adjusting for confounders correlated with the outcomes, the presence of unmeasured confounders may lead to increased instability.”
